# Dark matter in archaeal genomes: a rich source of novel mobile elements, defense systems and secretory complexes

**DOI:** 10.1007/s00792-014-0672-7

**Published:** 2014-08-12

**Authors:** Kira S. Makarova, Yuri I. Wolf, Patrick Forterre, David Prangishvili, Mart Krupovic, Eugene V. Koonin

**Affiliations:** 1 National Center for Biotechnology Information, National Library of Medicine, Bethesda, MD 20894 USA; 2 Institut Pasteur, Unité Biologie Moléculaire Du Gène Chez Les Extrêmophiles, 25 Rue Du Docteur Roux, 75015 Paris, France

**Keywords:** Archaeal genomes, ORFans, Genomic islands, Integration, Viruses, Defense

## Abstract

Microbial genomes encompass a sizable fraction of poorly characterized, narrowly spread fast-evolving genes. Using sensitive methods for sequences comparison and protein structure prediction, we performed a detailed comparative analysis of clusters of such genes, which we denote “dark matter islands”, in archaeal genomes. The dark matter islands comprise up to 20 % of archaeal genomes and show remarkable heterogeneity and diversity. Nevertheless, three classes of entities are common in these genomic loci: (a) integrated viral genomes and other mobile elements; (b) defense systems, and (c) secretory and other membrane-associated systems. The dark matter islands in the genome of thermophiles and mesophiles show similar general trends of gene content, but thermophiles are substantially enriched in predicted membrane proteins whereas mesophiles have a greater proportion of recognizable mobile elements. Based on this analysis, we predict the existence of several novel groups of viruses and mobile elements, previously unnoticed variants of CRISPR-Cas immune systems, and new secretory systems that might be involved in stress response, intermicrobial conflicts and biogenesis of novel, uncharacterized membrane structures.

**Electronic supplementary material:**

The online version of this article (doi:10.1007/s00792-014-0672-7) contains supplementary material, which is available to authorized users.

## Introduction

Genes of bacteria and archaea widely differ with respect to their phyletic spread and, by inference, the rates of gain and loss (Bellgard et al. 1999; Kolsto 1997; Koonin and Galperin 1997; Koonin and Wolf 2008; Wolf et al. 2012). Although the distribution of gene turnover rates is likely to be contiguous, the apparent qualitative differences between their occurrence patterns gave rise to the “core, shell, cloud” classification (Koonin and Wolf 2008; Makarova et al. 2007). Core genes are nearly ubiquitous and are enriched for components of the information processing machinery, primarily translation. Genes involved in metabolism and basic cellular processes belong to a wide class of shell genes; these genes comprise the majority of the genes in each individual prokaryotic genome. Most of the gene families, including singletons, also known as “ORFans” (Cortez et al. 2009; Siew and Fischer 2003; Yin and Fischer 2006), belong to the cloud of rare and/or narrowly distributed genes. They encompass only 10–20 % of the genes in each individual genome, but collectively these rare genes dominate the prokaryotic genetic space (Koonin and Wolf 2008).

The great majority of the cloud genes, especially ORFans, lack functional assignments because due to their rarity that is often coupled to fast evolution they are rarely characterized experimentally and are not readily amenable to informative computational analysis. However, many of those “cloud” genes that have been experimentally and/or computationally characterized, belong to one of the two functional classes: (a) the widely defined mobilome that is characterized by self-sustained ability to propagate semi-independently from their host genomes (various kinds of transposable elements, plasmids and viruses) and (b) defense systems that are prone to horizontal gene transfer due to the selection pressure that virus predation and other stress factors exert on microbes (Cortez et al. 2009; Daubin and Ochman 2004; Makarova et al. 2013b; Makarova et al. 2011b, c). Recently, the content of integrated viruses and plasmids in bacterial and archaeal genomes has been shown to be substantially higher and more diverse than previously suspected (Cortez et al. 2009; Krupovic and Bamford 2008a; Krupovic and Forterre 2011; Krupovic et al. 2011; Prangishvili 2013). In particular, multiple proviruses have been identified in archaea, and it has been shown that not only tailed bacteriophages, well known to integrate into bacterial chromosomes, but also ssDNA viruses form numerous proviruses (Krupovic and Forterre 2011). In parallel, in recent years, fueled in large by the discovery of the CRISPR-Cas systems of archaeal and bacterial adaptive immunity and their application in genome engineering (Barrangou and Horvath 2012; Kim and Kim 2014; Makarova et al. 2013a, b; Marraffini and Sontheimer 2010), comparative genomic and experimental studies have revealed an unsuspected diversity of prokaryotic defense systems that function on the principles of innate immunity, adaptive immunity or programmed cell death (Makarova et al. 2013a, b; Olovnikov et al. 2013; Swarts et al. 2014). Furthermore, it has been shown that analogous to previously characterized pathogenicity and symbiosis islands, genes encoding components of defense systems tend to form cluster in bacterial and archaeal genomes. Accordingly, such clusters have been dubbed defense islands (Makarova et al. 2011c).

The recent unexpected discoveries on the diversity of the mobilome and the defense repertoire of bacterial and archaeal genomes prompted us to systematically explore the archaeal cloud of rare genes. In particular, we aimed at the characterization of clusters of rare genes, identification of previously unnoticed functional themes, and delineation of possible differences between extremophiles and mesophiles.

## Materials and methods

### Identification of dark matter genes

A 2013 update of the archaeal clusters of orthologous genes (arCOGs) was used to annotate protein-coding genes in 147 archaeal genomes (Wolf et al. 2012); the results of this genome annotation are available at the NCBI FTP site (ftp://ftp.ncbi.nih.gov/pub/wolf/COGs/arCOG/). Genome sequences were downloaded from the NCBI FTP site (ftp://ftp.ncbi.nlm.nih.gov/genomes/Bacteria/). Phyletic patterns (patterns of presence-absence of proteins families) were derived from the respective arCOGs assignments; all genes that were not assigned to arCOGs were included in the analysis as ORFans. Only arCOGs represented in at most 5 archaeal genomes that are either uncharacterized or are annotated as virus-related were chosen for the subsequent in-depth analysis. Thus, the “dark matter” gene set includes this subset of small, uncharacterized arCOGs and all ORFans.

### Sequence analysis

Iterative profile searches with the PSI-BLAST (Altschul et al. 1997), with a cut-off e-value of 0.01, composition-based statistics and low complexity filtering turned off were used to search for distantly similar sequences in NR database. Additionally, another sensitive method for remote homology identification, HHpred, was used with default parameters (Soding et al. 2005).

Similarity based clustering was performed using the BLASTCLUST program (ftp://ftp.ncbi.nih.gov/blast/documents/blastclust.html) to cluster sequences at different thresholds. Multiple sequence alignments were built using PROMALS3D (Pei et al. 2008) and adjusted manually on the basis of the PSI-BLAST and HHpred search results. Protein secondary structure was predicted using Psi-Pred (Jones 1999).

Direct repeats corresponding to the attachment sites of bacteriophages were searched for using UGENE (Okonechnikov et al. 2012). For gene neighborhoods analysis of selected genes, 10 upstream and downstream genes were extracted. For each analyzed gene, the pfam identifiers and COG numbers as well as the corresponding annotations were assigned using RPS-BLAST program and CDD database of profiles (Marchler-Bauer et al. 2009).

Transmembrane segments were predicted using the TMMHMM v. 2.0c program with default parameters (Krogh et al. 2001). Signal peptides were predicted using the SignalP v. 4.1c program; the union of the three predictions (gram-negative, gram-positive and eukaryotic models) was used (Petersen et al. 2011).

Statistical analysis was performed in R environment. The probability density curves were obtained by Gaussian-kernel smoothing of the individual data points (Parzen 1962).

## Results and discussion

### Identification of dark matter islands in archaeal genomes and their major characteristics

**Fig. 1:**
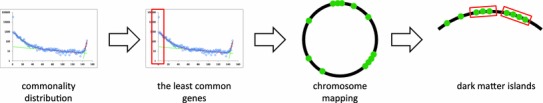
A schematic of the computational procedure for delineation of the dark matter islands in archaeal genomes. The commonality (gene frequency) plot is based on arCOGs for 146 archaeal genomes and built as described previously (Wolf et al. 2012). Green dots are genes on the chromosome

We used the arCOGs database to identify the part of the archaeal gene “cloud” that consists of ORFans and genes with a very narrow spread that, in addition, are not functionally characterized. Specifically, gene families represented in at most 5 archaeal genomes (Fig. 1) that are either uncharacterized or are annotated as virus-related were chosen for further analysis as the genomic “dark matter” (Supplementary Table 1). All “dark matter” genes were mapped to the corresponding genome partitions (chromosomes or plasmids). All contiguous 5-gene regions that contained at least 4 “dark matter” genes were marked as “island seeds”; overlapping seeds were merged into “dark matter islands” (Fig. 1). The rationale behind this approach is twofold. First, both genes comprising the archaeal and bacterial mobilomes and genes involved in antivirus defense that together represent a substantial part of the rare gene cloud, tend to form genomic islands (Makarova et al. 2013a, b; Makarova et al. 2011c). Second, shared genomic context increases the chance to obtain functional prediction and to minimize the impact of stand-alone spurious ORFs that originate from errors of ORF prediction (Aravind 2000; Galperin and Koonin 2000).

**Fig. 2:**
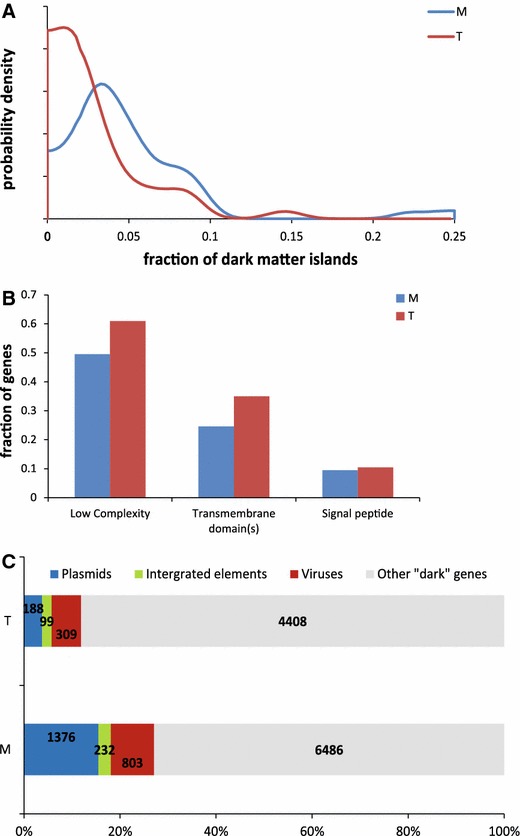
Comparison of the features of dark matter islands in archaeal thermophiles and mesophiles. **a** Distribution of the fraction of genome occupied by dark matter islands in mesophiles (M) and thermophiles (T). **b** Fraction of proteins containing low complexity segments, predicted transmembrane domains and predicted signal peptides in mesophiles (M) and thermophiles (T). **c** Fraction of different integrated elements in mesophiles (M) and thermophiles (T). The figure shows the data obtained with a non-redundant set of thermophile and mesophile genomes that included a single representative genome for each species, in order to eliminate biases potentially caused by the availability of genomes from multiple strains from some but not other species

Altogether we detected 1611 dark matter islands (Supplementary Table 1) that represent from 0 % (*Pyrococcus abyssi* GE5, *Caldisphaera lagunensis* DSM 15908 and *Metallosphaera sedula* DSM 5348) to 22 % (*Cenarchaeum symbiosum* A and Candidatus *Nitrososphaera gargensis* Ga9.2) of the total number of genes in archaeal genomes, with a mean of 3.8 % (Fig. 2a). The two genomes with the highest content of dark matter islands belong to the phylum Thaumarchaea that remains poorly represented among the sequenced genomes and thus possess many lineage-specific uncharacterized genes. Thermophiles generally contain fewer dark matter genes in their genomes compared to mesophiles; in part, this difference can be explained by the fact that several mesophilic archaea possess large genomes that do not have many close relatives among the completely sequenced genomes.

**Fig. 3:**
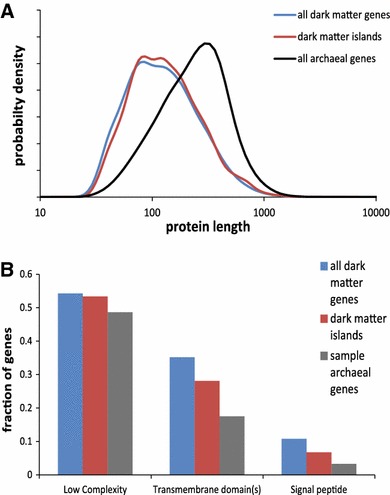
Comparison of the proteins encoded in dark matter islands with random samples of archaeal proteins. **a** Distribution of protein lengths in archaeal genomes, all dark matter genes and genes in dark matter islands. **b** Fraction of proteins containing low complexity segments, predicted transmembrane domains and predicted signal peptides in archaeal genomes (sample of genes with the same distribution of lengths as in dark matter islands), all dark matter genes and genes in dark matter islands

The characteristics of predicted proteins that comprise the dark matter differ from the respective characteristics of randomly sampled archaeal proteins. Thus, the dark matter is strongly enriched in small proteins (Fig. 3a). Approximately 28 % of the dark matter proteins are predicted to contain at least one transmembrane segment, and additional ~3 % are non-membrane proteins with predicted signal peptides, compared to approximately 18 and 2 %, respectively, in a random sample of non-dark matter archaeal genes with the same length distribution (Fig. 3b). Thus, the rare, fast-evolving archaeal genes are enriched in integral membrane proteins and secreted proteins that are likely to be involved in transport, signal transduction, communication and defense functions.

Compared to mesophiles, genes in dark matter islands of thermophilic archaea are somewhat longer (median length of 131 vs. 119 codons, *t* test *p* value of 1 × 10^−6^ for log lengths) and contain substantially more transmembrane proteins (39 vs. 26 %, χ^2^ *p* value ≪ 10^−10^) and slightly more proteins with signal peptides (11 vs. 9 %, χ^2^ *p* value 0.014) (Fig. 2b).

### Most abundant protein families in dark matter islands

**Fig. 4:**
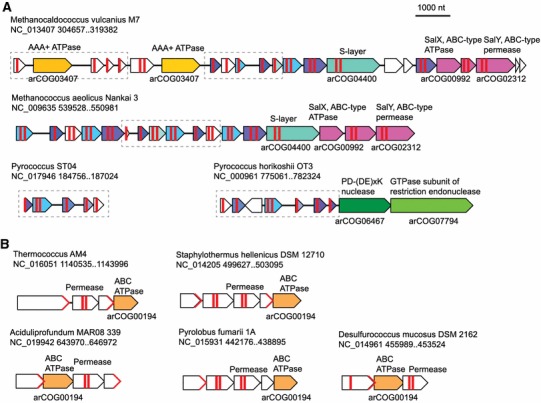
Neighborhood analysis of selected membrane-associated gene systems among the frequent arCOGs in dark matter. **a** A predicted ABC transporter potentially involved in antimicrobial peptide transport. **b** A membrane-associated system with variable gene cassettes. For each gene neighborhood, the organism name, genome partition and coordinates of the locus are indicated. Genes are shown by *block arrows* with the length roughly proportional to the size of the corresponding gene. Homologous genes are shown by the *same color*. Genes that belong to the respective dark matter island are denoted by *dashed boxes*. The positions of the predicted transmembrane segments are shown by *thick vertical* *red lines* inside the *arrows* showing the respective genes. Predicted signal peptides in putative secreted proteins are shown by *red angular brackets* at the ends of the respective *arrows*. The annotated arCOGs are indicated underneath the respective *arrows*; brief annotations of the proteins are shown above the *arrows*

Because our procedure allowed one arCOG from the “negative” (non-dark matter) gene set per a 5-gene island (see above), it is not surprising that many of the functionally characterized genes in the dark matter islands belong to this group (Table 1). Nevertheless, it is notable that most of these arCOGs clearly include bona fide mobilome genes such as integrases, transposases and proteins of apparent viral origin (Table 1). Such high prevalence of mobilome components indicates that many of the dark matter islands consist of or include various classes of mobile elements. Several families that were not associated with mobile elements included various (predicted) membrane proteins. A notable example is arCOG01996 that consists of uncharacterized membrane proteins that are present in *Archaeoglobi*, *Pyrococci* and several methanogens, including *Methanococcales*, where these genes form clusters with genes encoding several other membrane proteins (Fig. 4a). In the vicinity of this locus, *Methanococcales* encode a predicted ABC-type transporter related to the antimicrobial peptide transport system SalXY, whereas some *Pyrococci* encode components of restriction-modification systems (Fig. 4a). These observations suggest that such loci encode novel membrane-associated defense systems containing multiple highly variable membrane-associated components (see below). Another abundant protein family (arCOG00194) in the islands is the ATPase component of an ABC-type transporter. The ATPase gene forms a predicted operon with a 6-transmembrane protein, a predicted permease, and a putative substrate-binding protein. Both the putative permease and the binding protein are highly diverged and show only limited sequence similarity to homologs from other genomes (Fig. 4b). It appears likely that these predicted transport systems are involved in resistance to multiple antibiotics and/or other environmental chemicals, similar to bacterial multidrug resistance transporters. Radical SAM superfamily enzymes of arCOG00938 are encoded outside of any conserved context but along which arCOG00288, nitroreductase, represented the few small molecule-metabolizing enzymes that are over-represented in the islands (Table 1; Supplementary Table 1). It seems likely that these enzymes are involved in stress response and cell-cleaning functions.

**Table 1:** Top 20 most common arCOGs from the “negative set” in the dark matter islands

arCOG	Number in islands	Annotation	Comment
arCOG01241	31	**Integrase**	
arCOG01057	21	**Predicted transcriptional regulator, wHTH**	Virus associated
arCOG00280	21	**HerA-like helicase**	Possible viral DNA pumping ATPase or viral helicase
arCOG01996	17	Uncharacterized membrane protein	DUF95/COG1300, present in many bacteria and archaea
arCOG07960	16	**SSV ‘DnaA-like protein’ ATPase (AAA+superfamily)**	Virus associated
arCOG02134	15	**Transposase**	
arCOG01245	15	**Integrase**	
arCOG01242	14	**Integrase**	
arCOG00194	14	ABC-type multidrug transport system, ATPase component	ABC ATPase associated often associated with membrane protein, likely exporter
arCOG00439	14	**ATPase involved in replication control, Cdc46/Mcm family**	Virus associated
arCOG00467	13	**Orc/Cdc6-related protein, AAA superfamily ATPase**	Virus associated
arCOG03162	12	**Site-specific recombinase, DNA invertase pin homolog**	
arCOG00938	12	Radical SAM superfamily enzyme	
arCOG03032	12	TPR repeats containing protein	
arCOG06160	11	**Transposase**	
arCOG01308	10	**ATPase of the AAA+class, CDC48 family**	Virus/plasmid associated
arCOG03924	10	**Transcriptional regulator, MarR family**	Virus/plasmid associated
arCOG03166	9	**AAA+superfamily ATPase fused to HTH and RecB nuclease domains**	Likely involved in defense (or some plasmid gene)
arCOG01055	9	**Transcriptional regulator, MarR family**	Virus/plasmid associated paralog of arCOG03924
arCOG07102	9	**PD-(D/E)XK superfamily nuclease**	Virus/plasmid associated

Genes that belong to the mobilome genes are highlighted by bold type

In contrast to the over-represented genes from the negative set, among the over-represented arCOGs that were previously annotated as uncharacterized, only a few can be linked to the mobilome (Table 2), e.g., predicted nucleases of the PD-DEXK superfamily (arCOG09441), that appear to be linked to plasmid-like integrated elements (see below). Strikingly, most of these genes, many of which are lineage-specific, encode predicted secreted or membrane proteins (Table 2). For some of these proteins, we were able to identify remote sequence similarity with known protein families such as extracellular proteases (arCOG10864, arCOG06558). Some of these membrane-associated arCOGs form predicted operons although their specific functions remain obscure (see the section on “Predicted new secretion systems” below).

**Table 2:** Top 20 most abundant uncharacterized arCOGs in “dark matter islands”

arCOG	Number in islands	Annotation and comment
arCOG08821	18	Membrane protein, expanded in Thaumarchaeota
arCOG10873	14	Membrane protein
arCOG10027	12	Thermococcus specific secreted protein, paralogs belong to arCOG10066 and arCOG10028, they form genes clusters
arCOG10864	11	Predicted peptidase of C39 family; possibly associated with pseudo-murein binding domains
arCOG10897	10	Small membrane protein
arCOG06558	10	Likely a secreted protease, Propeptide PepSY and peptidase M4
arCOG10066	9	Thermococcus specific secreted protein, same as (see arCOG10027)
arCOG10865	9	Methanobacterium specific
arCOG09441	8	**HJR family endonuclease, PD-DEXK superfamily, associated with arCOG07809, viral Primase fused to AAA DnaA-like ATPAse and Zn finger domain**
arCOG10959	8	**Virus/plasmid associated, often co-occur with primase in particular arCOG06914**
arCOG09176	8	**Often associated with viruses or plasmids**
arCOG09593	8	Secreted protein with immunoglobulin-like domain
arCOG10866	8	Methanosaeta specific
arCOG09761	8	Large secreted protein; pyrobaculum specific expansion
arCOG11121	7	Uncharacterized conserved membrane protein
arCOG03316	7	Secreted enzyme present in bacteria and eukaryotes, duplication in methanosarcina acetivorans DUF3160
arCOG03631	7	Methanosarcina specific, present in bacteria
arCOG07691	7	Secreted protein associated with membrane protein of a number of related arCOGs (e.g., arCOG09771), mostly beta stranded; pyrobaculum specific expansion
arCOG06827	7	Membrane protein expansion in Methanosarcina, MGWCP motif, DUF1673
arCOG10868	7	**PD-(D/E)XK nuclease family transposase**
arCOG10363	7	Associated with Zn-finger containing protein from arCOG08887

Genes that belong to the mobilome genes are highlighted by bold type

### Dark matter and integrated elements

**Fig. 5:**
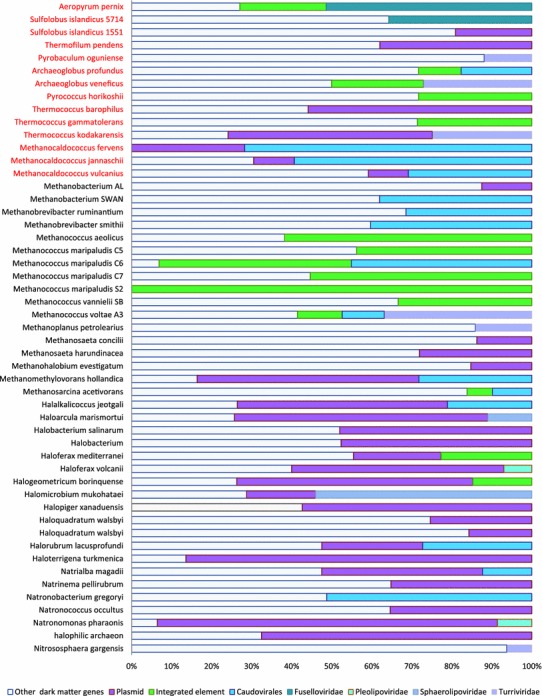
Distribution of the genes associated with different integrated elements in dark matter islands. The number of genes in each category of integrated elements and the rest of the dark matter genes in the respective genome are shown. Thermophiles are highlighted in *red*

Recently, substantial progress has been achieved in the characterization of archaeal viruses including those that are able to integrate in the host genomes (Prangishvili 2013). Among these, the most abundant and well characterized are members of the orders Ligamenvirales and Caudavirales, and the family Fuselloviridae (Krupovic et al. 2014; Prangishvili et al. 2013; Sencilo and Roine 2014).Two proviruses in *Aeropyrum pernix* have been recently identified first by in silico analysis and subsequently experimentally demonstrated to be inducible into lytic viruses (Mochizuki et al. 2011). In the *A. pernix* genome, these two proviruses appear as large genomic loci flanked by direct repeats (attachment sites) and parts of the split integrase gene. Each of the proviruses contained approximately 40 ORFs most of which were uncharacterized. Taking these findings into account, we modified our procedure and identified larger islands that consisted of at least 20 genes, with 5 genes from the negative set allowed. These larger islands are expected to better reflect virus-related loci, whereas the smaller islands could contain other integrated elements such as plasmids and transposons. Comparison of the two island sets showed that nearly all larger islands at least partly overlap the smaller island set (the reverse, obviously, is not the case). We mapped identified (pro)viral and plasmid genes (including genes from plasmids that are genomic partitions; see Supplementary Table 2 for details) on both sets of islands. The results suggest that approximately 20 % of the island content can be attributed to the genes encoded in known viruses and plasmids. All the common groups of viruses mentioned above and many previously described plasmids are present in the islands. The distribution of different classes of integrated elements varies considerably even among closely related archaea (Fig. 5). In many cases, it is not possible to determine whether an integrated element represents a plasmid or a virus because many of these elements encompass both viral and plasmid genes.

Compared to thermophiles, dark matter islands in mesophile genomes contain a significantly larger fraction of identifiable integrative elements (27 vs. 12 %, χ^2^ *p* value ≪ 10^−10^), largely due to the high abundance of plasmids (15 vs. 4 %, χ^2^ p value ≪ 10^−10^) (Fig. 2c). Known viruses comprise a higher fraction of the dark matter islands in thermophiles (9 vs. 6 %, χ^2^ *p* value 3 × 10^−9^) (Fig. 2c).

**Fig. 6:**
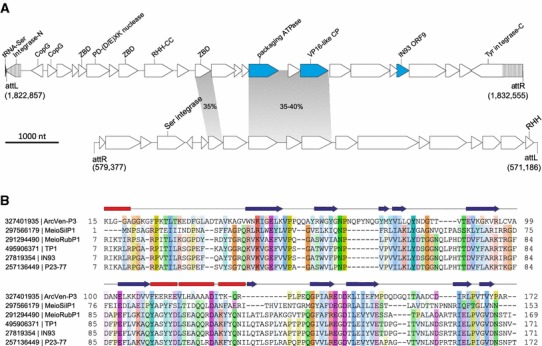
The putative provirus ArcVen-P3 from *Archaeoglobus veneficus* SNP6. **a** Genome maps of putative proviruses residing in the genomes of *A. veneficus* SNP6 (*top*; NC_015320) and *Dehalococcoides sp.* CBDB1 (*bottom*; NC_007356). The exact nucleotide coordinates for the two proviruses are indicated in the figure. Open reading frames are represented with *arrows*, indicating the direction of transcription. ArcVen-P3 genes with homologs in bacterial sphaerolipoviruses are shown in *blue*. The regions of the split ArcVen-P3 integrase gene, which have not been originally annotated, are indicated with *stripes*. The genes shared between the two proviruses are connected with *grey* shading and the pairwise identity between the corresponding proteins is indicated. Abbreviations: *ZBD* Zn-binding domain; *RHH* ribbon-helix-helix motif; *CC* coiled-coil domain; *CP* capsid protein. **b** Multiple sequence alignment of the predicted capsid proteins (VP16) from ArcVen-P3, bacterial sphaerolipoviruses (P23-77 and IN93) and related proviruses (MeioSilP1, MeioRubP1, TP1) (Pawlowski et al. 2014). Sequences are denoted with their GenBank identifiers. The *arrows* and *rectangles* above the alignment denote the experimentally determined secondary structure elements of P23-77 VP16 (PDB ID: 3ZMO) (Rissanen et al. 2013)

It could be expected that, along with already known ones, the dark matter islands would contain unknown or unusual integrated elements. One of such islands identified in the *Archaeoglobus veneficus* SNP6 genome and containing an unusual combination of viral-like genes was selected for more detailed characterization as an example of a virus-derived region. Although some bacterial viruses are known to integrate into the genomes of their hosts using cellular recombination machineries (Huber and Waldor 2002; Krupovic and Forterre 2011), the majority of prokaryotic viruses and plasmids encode dedicated enzymes, known as integrases, which mediate their insertion into specific loci of the cellular genomes (She et al. 2004). The most common integration targets for archaeal mobile elements are tRNA genes (She et al. 2004). Analysis of the selected region from the *A. veneficus* SNP6 genome showed that the potential integrated element is adjacent to a tRNA-Ser gene. Following the analysis of potential recombination sites, we identified a pair of perfect direct repeats of 55 nucleotides flanking a genomic region of 9.5 kb. One of the repeats overlapped the tRNA gene, whereas the other one was adjacent to the integrase gene. Such an arrangement is the most common among archaeal integrated viruses and plasmids (Krupovic et al. 2010; She et al. 2004), and is consistent with the site-specific integration of the element, which we denote ArcVen-P3, into the tRNA-Ser gene (Fig. 6a).

Analysis of the ArcVen-P3 gene content (Supplementary Table 3) showed that this element is not closely related to known viruses and plasmids of archaea. Nevertheless, several virus-like genes could be recognized. BlastP searches against the viral protein database showed that ArcVen-P3 encodes two small CopG-like ribbon–helix–helix proteins similar to the transcriptional regulator F55 of *Sulfolobus* spindle-shaped virus 1 (SSV1) (Fusco et al. 2013). In addition, ArcVen-P3 contains genes for two other proteins with homologues in spindle-shaped viruses, namely an SSV-like integrase and an SSV1 VP2-like protein. The distinguishing feature of SSV-like integrases is that upon recombination the integrase gene is split into two fragments, the N-and C-terminal, that bound the integrated element. Detailed analysis of the termini of ArcVen-P3 indeed revealed the region encoding the originally unannotated N-terminal moiety of the SSV-like integrase and furthermore led to extension of the C-terminal integrase fragment (Fig. 6a). The closest homolog of the ArcVen-P3 integrase is encoded in the *Thermococcus prieurii* virus 1 (Supplementary Table 3) (Gorlas et al. 2012), a spindle-shaped virus recently classified as a member of the proposed *Deltafusellovirus* genus within the family *Fuselloviridae* (Krupovic et al. 2014). VP2 is a virion-associated DNA-binding protein of SSV1 which, unexpectedly, appears to be dispensable for virus viability (Iverson and Stedman 2012; Reiter et al. 1987). A VP2 homolog has been previously detected in the genome of *Thermus* bacteriophage IN93 (ORF9), a member of the proposed genus *Gammasphaerolipovirus* within the family *Sphaerolipoviridae* (Pawlowski et al. 2014). Members of the latter virus family possess icosahedral capsids with an internal membrane and infect either bacterial or archaeal hosts (Pawlowski et al. 2014); these viruses are unrelated to spindle-shaped fuselloviruses. The closest homolog of the VP2-like protein of ArcVen-P3 was found not among archaeal fuselloviruses but rather in the bacteriophage IN9.

Another virus-derived gene of ArcVen-P3 encodes a divergent FtsK-like ATPase related to genome packaging enzymes of viruses with icosahedral capsids and internal membranes (Iyer et al. 2004; Stromsten et al. 2005). The FtsK-like packaging ATPases have so far been exclusively found in viruses with jelly-roll capsid proteins (CP) (Koonin et al. 2006; Krupovic and Bamford 2008b), including members of the *Sphaerolipoviridae*, suggesting that ArcVen-P3 might also encode a jelly-roll CP. Although BlastP analysis did not allow detection of CP candidates, HHpred search seeded with the Arcve_2069 sequence resulted in a partial hit to the capsid protein VP16 of the sphaerolipovirus P23-77 infecting *Thermus thermophilus* (Rissanen et al. 2013). To validate this weak HHpred hit, we generated a multiple sequence alignment of the ArcVen-P3 protein and VP16-like proteins from previously described (Pawlowski et al. 2014) *Thermus* sphaerolipoviruses and related proviruses (Fig. 6b). Not only the sequence similarity was distributed evenly along the protein length, but the secondary structure elements that constitute the jelly-roll fold of the VP16 protein (Rissanen et al. 2013) were conserved in the VenArc-P3 protein. Furthermore, the genomic location of the VP16-like gene in ArcVen-P3 is consistent with its predicted CP function because VP16-like genes in both P23-77-like viruses and ArcVen-P3 are encoded downstream of the genome packaging ATPase, separated by one gene. The virions of sphaerolipoviruses consist of two jelly-roll CPs, VP16 and VP17, that have been suggested to originate by gene duplication (Rissanen et al. 2013). However, we were unable to detect a homolog of the VP17-like CP in the ArcVen-P3 genome. Collectively, these observations suggest that ArcVen-P3 shares at least two of the viral hallmark proteins, FtsK-like ATPase and VP17-like CP, with sphaerolipoviruses (and additionally an ORF9-like gene with sphaerolipovirus IN93).The genomes of bacterial sphaerolipoviruses P23-77 and IN93 (17 and 19.6 kb, respectively) are considerably larger than the genome of ArcVen-P3. Therefore, it appears likely that ArcVen-P3 represents an ancestral, simpler state of P23-77/IN93-like sphaerolipoviruses with only one CP gene. Experimental validation of this hypothesis should provide insights into the origin and evolution of these viruses.

Finally, we noticed that some of the ArcVen-P3 encoded proteins, including those for the putative packaging ATPase and VP16-like CP, produced reciprocal best Blast hits to proteins from *Dehalococcoides sp*. CBDB1 (Supplementary Table 3). The latter bacterium belongs to the phylum *Chloroflexi* and is known for its ability to catabolize many of the most toxic chlorinated aromatics and aliphatics by reductive dechlorination (Kube et al. 2005). Analysis of the *Dehalococcoides sp.* CBDB1 genomic region encompassing the homologs of the ArcVen-P3 genes showed that these genes are also located within an integrated element flanked by perfect direct repeats of 15 nucleotides (Fig. 6a). Notably, instead of the SSV-like tyrosine recombinase, the putative provirus of *Dehalococcoides* encodes an unrelated serine integrase. The similarity between the two putative proviruses, respectively, residing in the archaeal and bacterial genomes highlights them as a potential new group of viruses related to the family *Sphaerolipoviridae* and traversing the boundary between the cellular domains archaea and bacteria. Detailed analysis of the putative (pro)viruses identified in the course of this project will be published elsewhere (MK, KSM, EVK, PF, DP, in preparation).

**Fig. 7:**
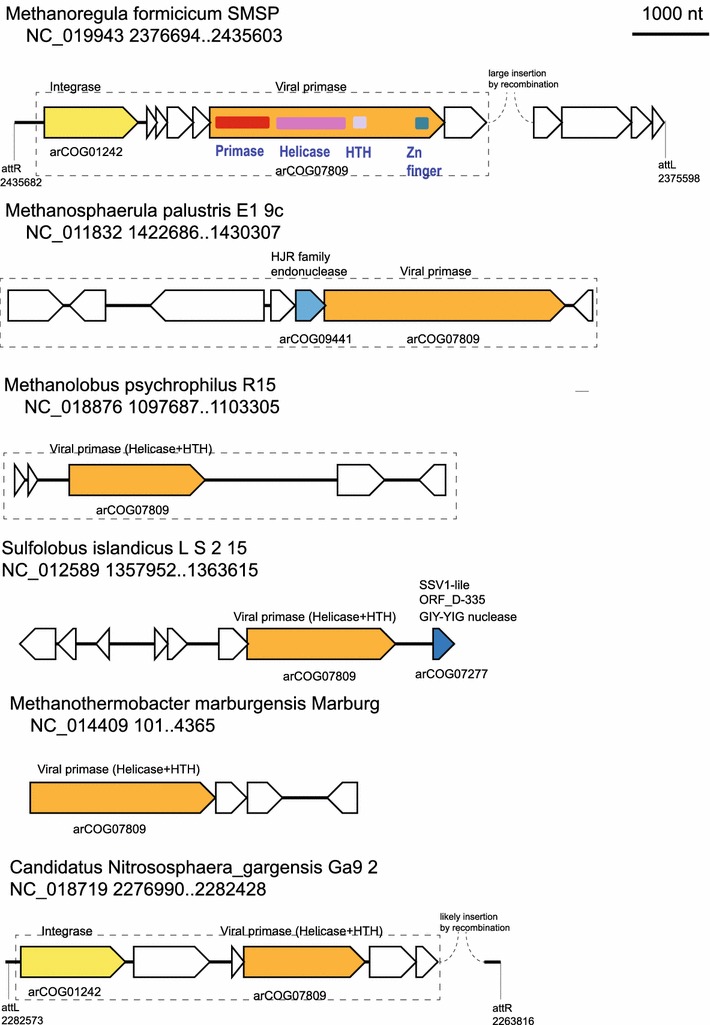
Neighborhood analysis of putative small integrated elements associated with the novel prim-pol protein of arCOG07809. Designations are the same as in Fig. 4. Domains identified in prim-pol-like protein are shown by *colored shapes* within the respective gene *arrow*

Inspection of the smaller islands revealed yet uncharacterized putative elements that encode a protein (arCOG07809, e.g., Mpal_2033 from *Methanosphaerula palustris*) distantly related to the primase-polymerase (prim-pol) domains of replication proteins from plasmids of *Sulfolobus* (Lipps 2011; Prato et al. 2008) and *Thermococcus* (Gill et al. 2014; Krupovic et al. 2013) as well as certain bacterial viruses (Halgasova et al. 2012). All these proteins are members of the large superfamily of archaeao-eukaryotic primases (AEP) which also includes the catalytic subunit of the archaeal and eukaryotic primases, the enzymes responsible for the initiation of DNA replication (Iyer et al. 2005). In different AEP members, the prim-pol domains possess a broad range of enzymatic activities (Gill et al. 2014) and are often fused to a variety of functionally and structurally distinct domains (Iyer et al. 2005). For example, in ORF904 from *S. islandicus* plasmid pRN1, the N-terminal prim-pol domain is followed by superfamily 3 helicase and winged helix–turn–helix (wHTH) domain (Lipps 2011; Lipps et al. 2004). A similar prim-pol protein is encoded by pSSVx which is a fusellovirus satellite virus from *S. islandicus* that combines features of a plasmid and a virus (Arnold et al. 1999; Contursi et al. 2014). However, these proteins are only distantly related to Mpal_2033 and belong to a different arCOG (arCOG06914). Mpal_2033 encompasses four domains: primase, Superfamily I helicase, HTH and Zn finger. We identified several Mpal_2033 homologs that lack the primase domain. Figure 7 shows examples of such elements from different archaeal genomes (including both those identified in the small dark matter islands and those detected on the basis of the presence of an arCOG07809-related gene). In addition, stand-alone proteins closely related to the helicase domain of Mpal_2033 are encoded in the genomes of several fuselloviruses (e.g., C674 protein from *Acidianus* spindle-shaped virus 1) (Redder et al. 2009), suggesting a role in the initiation of viral DNA replication. Several islands that encompass Mpal_2033 homologs also carry genes for integrases, and for some of them, we identified the integration sites, indicating that these are bona fide integrative elements (Fig. 7). The only two other ORFs from these elements with identifiable sequence similarity to characterized proteins are two endonucleases from different families one of which is related to ORF_D-335 of SSV1, the typical representative of fuselloviruses (Fig. 7). Thus, the small size of the element, the presence of an AEP superfamily (albeit through C-terminal domain only) protein and other ORFs shared with fuselloviruses, and the absence of apparent viral proteins involved in capsid formation is compatible with the readily testable hypothesis that this element is a novel integrated satellite virus.

In addition to the identification of these putative novel viruses, we identified two interesting cases of potential IS elements contained in the smaller islands. One is a distant homolog of an experimentally uncharacterized insertion element of the YhgA family (pfam04754) which contains a PD-(DE)xK superfamily domain. Propagation of these elements is especially prominent in *Methanomicrobia* (e.g., Mhun_2695 from *Methanospirillum hungatei*, 11 copies). The second one is a Mutator-like element (MULE) specific for *Methanosarcina acetivorans* (e.g., MA1896, present in 8 copies in the islands and 13 altogether in the genome, not counting fragments) that was previously identified in eukaryotes only (Babu et al. 2006) but, according to the respective pfam set (PF10551), are also present in a few other archaea and bacteria.

### New predicted defense systems identified in dark matter islands

It has been shown that defense genes often form genomic islands and that analysis of such islands allows one to predict new defense systems although the majority of the genes in these islands remain unique or species-specific (Makarova et al. 2013b; Makarova et al. 2011c). Using the procedure described previously (Makarova et al. 2011c), we delineated defense islands in the archaeal genomes analyzed here and then performed cross-mapping of the defense and small dark matter islands. Altogether the overlap constitutes about 15 % of the dark matter. Many of the defense island genes overlap with regions identified as viruses or plasmids (727 of the 3007 detected virus-plasmid-related genes) which is not surprising because many plasmids share defense genes, especially toxin–antitoxin systems, with their hosts (Makarova et al. 2009). Additional 1293 defense genes (approximately 12 % of all genes in the small islands) do not overlap with virus or plasmid regions and might encode novel defense functions.

**Fig. 8:**
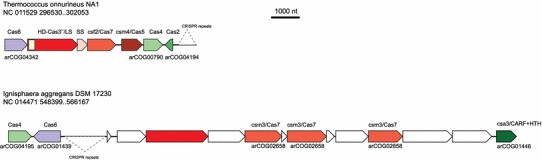
Neighborhood analysis of two highly derived CRISPR-Cas systems. Designations are the same as in Fig. 4. The Cas gene annotation is based on accepted classification (Koonin and Makarova 2013; Makarova et al. 2011a, b)

In particular, we identified two distinct, putative CRISPR-Cas systems that have not been described previously (Fig. 8). Both these CRISPR-Cas variants are so far unique and present in only one genome each, and only a few proteins encoded in these loci show significant sequence similarity with known Cas protein families present in arCOGs (Supplementary Table 1). Despite these unique features, both loci encompass CRISPR-Cas signatures. The putative CRISPR-Cas locus in *Thermococcus onnurineus* includes three clearly identifiable components, namely Cas2 ribonuclease that is implicated in spacer integration along with Cas1, Cas4, a PD-DEXK family nuclease, and Cas6, the ribonuclease involved in the processing of pre-crRNA transcript (Makarova et al. 2011a, c). Two other proteins, TON_0322 and TON_0323, respectively, show limited sequence similarity to the proteins of the Csm4 subfamily, which belongs to the Cas5 family and is associated with type III-A CRISPR-Cas systems, and to the Csf2 subfamily of the Cas7 family that is associated with type IV CRISPR-Cas systems (Koonin and Makarova 2013). In addition, this locus encodes two other proteins that consist of 587 and 120 amino acids, respectively, and are predicted to adopt mostly alpha-helical conformation but do not show significant similarity with other proteins. Nevertheless, the general organizational principles of CRISPR-Cas systems (Makarova et al. 2013a, b) imply that these proteins could be large and small subunits of the CASCADE complex, respectively. Moreover, HHpred analysis detected a diverged HD nuclease domain at the N-terminus of the larger protein (Supplementary Table 4), with the same order of the catalytic motifs as the HD domain present in the Cas3 and Cas10d families (Makarova et al. 2006). The Cas2 protein in this locus is highly similar to the Cas2 proteins from other *Thermococci*, but in those genomes this protein belongs to Type I-A loci. Thus, it seems that this unique system replaced a Type I-A locus specifically in the *Thermococcus onnurineus* lineage. The evidence from protein sequence analysis and the genomic locus organization does not allow us to classify this system as any of the known CRISPR-Cas subtypes. Considering the absence of the Cas1 gene that is essential for spacer integration, this system is not predicted to be capable of inserting new spacers into the CRISPR cassettes. Moreover, in *T. onnurineus*, Cas1 is missing altogether although an otherwise intact type III-A CRISPR-Cas system (TON_0892-TON_0898), and additional CRISPR repeat arrays are present elsewhere in the genome. Whether the CRISPR-Cas systems in *T. onnurineus* are in the beginning of the degradation route, function without spacer integration or employ an alternative integration mechanism, is an intriguing problem for experimental study that could shed light on the general principles of CRISPR-Cas functioning.

Another CRISPR-Cas system was identified in *Ignisphaera aggregans*. The locus also includes clearly identifiable Cas6 and Cas4 family genes and a Csa3-like gene which encodes a predicted transcriptional regulator containing a ligand-binding domain and a HTH domain (Makarova et al. 2011a, b). Three other proteins encoded in this locus, Igag_0617, Igag_0615 and Igag_0612, all show limited similarity to the Csm3 subfamily of the Cas7 family. The HHpred search showed that another protein in this locus, Igag_0619, is a highly diverged version of the Cas10 protein in which all catalytic motifs remain intact (Supplementary Table 4). These observations suggest that the predicted CRISPR-Cas system of *Ignisphaera* is a highly derived variant of Type III.

### New type IV pili-like systems identified in dark matter islands

**Fig. 9:**
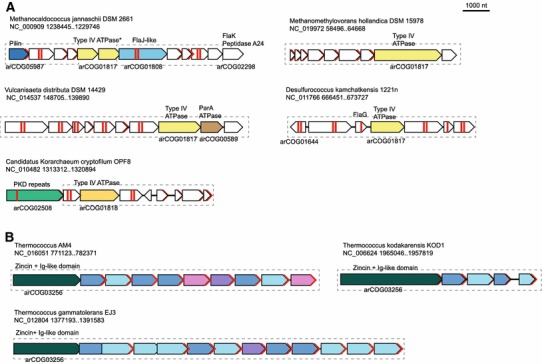
Neighborhood analysis of the predicted new type IV secretion systems. Designations are the same as in Fig. 4

As pointed out above, predicted secreted proteins comprise ~7 % of the genes encoded in the archaeal dark matter islands. This is likely to be a substantial underestimate because many proteins predicted to contain a single transmembrane domain at the N-terminus are likely to be secreted, given that prediction methods optimized for archaeal signal peptides, to our knowledge, are currently unavailable. Several dark matter islands contain genes encoding various predicted ATPases, in particular those of the VirB11 protein family (9 islands) that are involved in a variety of trafficking functions including the biogenesis of archaeal flagella (archaella) and pili, and substrate transport by type IV secretion systems (Alvarez-Martinez and Christie 2009; Guglielmini et al. 2013; Lassak et al. 2012; Ripoll-Rozada et al. 2013). All of these predicted ATPases are encoded within putative operons that also contain genes coding for multiple predicted membrane and/or secreted proteins which (with a few exceptions) do not share any similarity with the components of experimentally characterized archaeal type IV pili-like systems (Lassak et al. 2012). Thus, these operons likely encode novel secretion and membrane-associated systems (Fig. 9a). The motor ATPases in these loci belong to two distinct subfamilies (Fig. 9a), suggesting that some of these putative secretory systems perform specialized functions and/or that the ATPase genes are extensively exchanged.

An unusual putative secretory system was found in the genome of *Methanocaldococcus jannaschii* (Fig. 9a). It shares some proteins, such as flagellins/pilins, FlaJ-like pili assembly protein and FlaK-like peptidase A24A with the archaellum, but does not seem to include other typical proteins associated with this archael motility structure, such as FlaG, FlaF, FlaH (Jarrell and Albers 2012). Nor does this locus encode typical pilins found in pili systems characterized in the genome of another methanococcal species, *Methanococcus maripaludis* (Ng et al. 2011). The majority of the proteins in this operon are shared only by a few other species of methanococci. The loci coding for the typical archaellum and the pili system are conserved between *M. jannaschii* and *M. maripaludis*. Thus, the putative novel Type IV pili-like system that we identified in *M. jannaschii* is likely to perform a distinct function specific to a narrow methanococci group.

Other putative membrane-associated detected in the dark matter islands systems found in the dark islands share even fewer genes with known archaeal motility and surface appendage structures. For example, in a unique system that is encoded on the plasmid pMETHO01 from *Methanomethylovorans hollandica*, even the VirB11 homolog shows low (albeit significant) sequences similarity to the archaellum FlaI subunits (Fig. 9a).

In addition, we examined in greater detail the most common arCOGs that consist of predicted secreted proteins, e.g., arCOG10027 which is specific for *Thermococcus* species (Table 2). We found that the loci encoding proteins of this arCOG also contain genes for paralogous predicted secreted proteins of arCOG10028 and arCOG10066 (Fig. 9b). Furthermore, the predicted operons encoding these proteins also contain a gene for a large protein that contains a zincin metallopeptidase and a distinct immunoglobulin-like fold which is often found in surface proteins (Fig. 9b). This peptidase might cleave some peptides from the multiple proteins encoded in the same locus. The loci show remarkable variation between species suggesting that they encode secreted proteins involved in inter-microbial conflicts, such as bacteriocin-like toxins proteins, or quorum sensing.

Collectively, these observations show that archaeal dark matter islands encode a variety of uncharacterized, lineage-specific, membrane-associated and secreted protein complexes. Further comparative genomic and experimental analysis of these systems can be expected to shed new light on archaeal ecology.

## Conclusions

As expected, detailed analysis of the dark matter islands in archaeal genomes shows that these genomic regions are highly heterogeneous and diverse with respect to gene composition and predicted functions. Nevertheless, three distinct but not necessarily independent themes are clearly discernible:

1. Integrated viral genomes and other mobile elements
2. Defense systems
3. Secretory and other membrane-associated systems.

Jointly, mobilome components account for about 22 %, defense systems for about 15 %, and secreted or membrane proteins for about 31 % of the dark matter islands. The union of all these “known” genes amounts to about 55 % of the gene content of the archaeal dark matter, whereas the rest half of the genes in the islands remain complete unknowns. The most unexpected finding is the striking abundance of predicted secreted and membrane proteins encoded in the dark matter islands. Most of these novel membrane-associated and secretory systems function in inter-microbial conflicts and communication, functions that differ even among closely related microbes.

In evolutionary terms, the key trends are fast evolution and horizontal mobility that result in rapid divergence and rearrangement of the “dark” loci. This mode of evolution could be readily predicted for the mobilome and defense systems, but the similar characteristics of the numerous putative novel membrane-associated and secreted systems are unexpected and point to a poorly understood aspect of microbial biology.

Although the overall characteristics of the dark matter are qualitatively similar in thermophiles and mesophiles, predicted membrane proteins are substantially more abundant and secreted proteins are slightly more abundant in thermophiles. The potential functional link of these systems to the lifestyle of thermophiles remains to be explored. Conversely, mesophiles contain many more recognizable proviruses and mobile elements within the dark matter islands. A possible explanation of this difference could be that the current knowledge of the viruses and mobile elements of thermophiles is substantially incomplete, suggesting that novel classes of such elements remain to be discovered.

The principal conclusion of this work is that the abundant dark matter of archaeal genomes is a rich source of novel functional systems and new types of genetic elements many of which are predicted to be involved in defense and various forms of inter-microbial interaction. Further study of the dark matter is likely to shed new light on archaeal ecology. Some of the novelties in the dark matter islands include new variants of CRISPR-Cas systems that have recently stirred major interest owing to their fundamental role in microbial immunity and applications in genome engineering. Conceivably, completely novel defense systems with potential utility as tools of molecular biology can be identified as well.

## Electronic supplementary material

Below is the link to the electronic supplementary material.

Supplementary material 1 (PDF 6091 kb)

Supplementary material 2 (XLSX 18 kb)

Supplementary material 3 (DOCX 19 kb)

Supplementary material 4 (DOCX 16 kb)

Supplementary Material.

Supplementary table S1.

Annotation of genes in archaeal dark matter islands.

Supplementary table S2.

Integrated mobile elements in archaeal dark matter islands.

Supplementary table S3.

Annotation of the putative provirus ArcVen-P3.

Supplementary table S4.

Sequence similarity search results for selected proteins from archaeal dark matter islands.
