# Supplementary material for: Dark matter in archaeal genomes: a rich source of novel mobile elements, defense systems and secretory complexes
Source: Extremophiles. 2014 Aug 12;18(5):877–93. doi: 10.1007/s00792-014-0672-7 (PMC4158269; doi:10.1007/s00792-014-0672-7)
Supplement: Supplementary file 1 — Supplementary material 1 (PDF 6091 kb) [file 792_2014_672_MOESM1_ESM.pdf]

GI	Refseq protein annotation	Signal peptide present	Number of transmembrane segments	Belong to defense islands	Integrated element	Gene start	Gene end	Strand/Number of genes in an island	Organism	Genome partition	Genome partition type	BLASTclust Cluster	arCOG	arCOG count	(-) - arCOG from negative list	Functional group (as in arCOGs)	Gene name (as in arCOGs)	arCOGs annotation	best BLAST hit in CDD (E-value)	best BLAST hit identificator	best BLAST hit in CDD annotation
===								5	Acidianus_hospitalis_W1_uid66875	NC_015518										
332796667	fuselloviral integrase	No	0			886432	886536	-	Acidianus_hospitalis_W1_uid66875	NC_015518	chromosome										
332796668	hypothetical protein Ahos_0984	No	0			886699	886824	+	Acidianus_hospitalis_W1_uid66875	NC_015518	chromosome										
332796669	hypothetical protein Ahos_0985	No	0			886880	887707	-	Acidianus_hospitalis_W1_uid66875	NC_015518	chromosome	clust_104									
332796670	amino acid transporter	No	14			888325	889992	-	Acidianus_hospitalis_W1_uid66875	NC_015518	chromosome		arCOG00009	2	(-)	E	PotE	Amino acid transporter	5E-30	COG0531	Amino acid transporters
332796671	hypothetical protein Ahos_0987	No	1			890232	890609	+	Acidianus_hospitalis_W1_uid66875	NC_015518	chromosome										
===								7	Acidianus_hospitalis_W1_uid66875	NC_015518	chromosome										
332797229	plasmid protein	No	0			1396913	1397380	-	Acidianus_hospitalis_W1_uid66875	NC_015518	chromosome		arCOG03737	1	(-)	K		Transcriptional regulator of GntR family			
332797230	plasmid protein	No	0			1397502	1397720	-	Acidianus_hospitalis_W1_uid66875	NC_015518	chromosome										
332797231	plasmid protein	No	0			1397717	1398088	-	Acidianus_hospitalis_W1_uid66875	NC_015518	chromosome										
332797232	plasmid protein	No	0			1398030	1398437	-	Acidianus_hospitalis_W1_uid66875	NC_015518	chromosome										
332797233	plasmid protein	No	0			1398427	1399080	-	Acidianus_hospitalis_W1_uid66875	NC_015518	chromosome										
332797234	hypothetical protein Ahos_1553	No	0			1399872	1400807	-	Acidianus_hospitalis_W1_uid66875	NC_015518	chromosome	clust_104									
332797235	ParB domain-containing protein, nuclease	No	0			1400991	1402313	-	Acidianus_hospitalis_W1_uid66875	NC_015518	chromosome		arCOG07935	1	(-)	K	Spo0J	Chromosome partitioning protein ParB	0.000003	COG1196	Chromosome segregation ATPases
===								8	Acidianus_hospitalis_W1_uid66875	NC_015518	chromosome										
332797275	polysaccharide biosynthesis-like protein	No	10			1440266	1441645	-	Acidianus_hospitalis_W1_uid66875	NC_015518	chromosome		arCOG02209	4	(-)	R	RfbX	Polysaccharide biosynthesis protein, Mvin family	2E-25	COG2244	Membrane protein involved in the export
332797276	hypothetical protein Ahos_1597	No	0			1441806	1442552	-	Acidianus_hospitalis_W1_uid66875	NC_015518	chromosome										
332797277	hypothetical protein Ahos_1598	No	0			1442620	1443081	-	Acidianus_hospitalis_W1_uid66875	NC_015518	chromosome										
332797278	hypothetical protein Ahos_1599	No	12			1443617	1446145	-	Acidianus_hospitalis_W1_uid66875	NC_015518	chromosome		arCOG07850	2		S		Uncharacterized conserved protein			
332797279	hypothetical protein Ahos_1601	No	0			1447340	1447696	-	Acidianus_hospitalis_W1_uid66875	NC_015518	chromosome										
332797280	hypothetical protein Ahos_1602	No	0			1448020	1448247	+	Acidianus_hospitalis_W1_uid66875	NC_015518	chromosome										
332797281	methyltransferase, FkbM family	No	0			1448804	1449547	+	Acidianus_hospitalis_W1_uid66875	NC_015518	chromosome		arCOG01401	2	(-)	Q		SAM-dependent methyltransferase	0.000009	pfam02475	Met-10+ like-protein. The methionine-10
332797282	hypothetical protein Ahos_1606	Yes	1			1450523	1451140	+	Acidianus_hospitalis_W1_uid66875	NC_015518	chromosome										
===								8	Acidianus_hospitalis_W1_uid66875	NC_015518	chromosome										
332797435	hypothetical protein Ahos_1760	No	0			1596338	1597540	-	Acidianus_hospitalis_W1_uid66875	NC_015518	chromosome	clust_83									
332797436	hypothetical protein Ahos_1761	No	0			1597595	1597750	-	Acidianus_hospitalis_W1_uid66875	NC_015518	chromosome		arCOG11080	2		S		Uncharacterized conserved protein			
332797437	Cytosine methylase	No	0			1597796	1599007	-	Acidianus_hospitalis_W1_uid66875	NC_015518	chromosome		arCOG00890	3	(-)	L		DNA modification methylase	0.00005	COG0863	DNA modification methylase
332797438	hypothetical protein Ahos_1763	No	0			1598997	1599458	-	Acidianus_hospitalis_W1_uid66875	NC_015518	chromosome		arCOG09819	2		S		Uncharacterized conserved protein			
332797439	hypothetical protein Ahos_1764	No	0			1599682	1600218	-	Acidianus_hospitalis_W1_uid66875	NC_015518	chromosome		arCOG09921	3		S		Uncharacterized conserved protein			
332797440	OrfB transposable element-like protein, IS605	No	0			1600323	1601387	-	Acidianus_hospitalis_W1_uid66875	NC_015518	chromosome		arCOG04197	4	(-)	R		Transposase related protein			
332797441	hypothetical protein Ahos_1766	No	0			1601761	1602189	-	Acidianus_hospitalis_W1_uid66875	NC_015518	chromosome		arCOG09920	3		S		Uncharacterized conserved protein			
332797442	hypothetical protein Ahos_1767	No	0			1602466	1602585	+	Acidianus_hospitalis_W1_uid66875	NC_015518	chromosome		arCOG09919	3		S		Uncharacterized conserved protein			
===								7	Acidianus_hospitalis_W1_uid66875	NC_015518	chromosome										
332797518	aldo/keto reductase	No	0			1667312	1668112	+	Acidianus_hospitalis_W1_uid66875	NC_015518	chromosome		arCOG01619	3	(-)	R	ARA1	Aldo/keto reductase, related to diketogulonate re	8E-52	COG0656	Aldo/keto reductases, related to diketog
332797519	hypothetical protein Ahos_1848	No	0			1668749	1669105	+	Acidianus_hospitalis_W1_uid66875	NC_015518	chromosome										
332797520	hypothetical protein Ahos_1849	No	0			1669207	1669488	-	Acidianus_hospitalis_W1_uid66875	NC_015518	chromosome		arCOG09707	1		S		Uncharacterized conserved protein			
332797521	hypothetical protein Ahos_1850	No	0			1669514	1669711	+	Acidianus_hospitalis_W1_uid66875	NC_015518	chromosome										
332797522	hypothetical protein Ahos_1851	No	0			1670164	1670514	+	Acidianus_hospitalis_W1_uid66875	NC_015518	chromosome										
332797523	hypothetical protein Ahos_1852	No	0			1670794	1671078	-	Acidianus_hospitalis_W1_uid66875	NC_015518	chromosome		arCOG10435	1		S		Uncharacterized conserved protein			
332797524	hypothetical protein Ahos_1853	No	4			1671291	1671878	+	Acidianus_hospitalis_W1_uid66875	NC_015518	chromosome		arCOG05371	4	(-)	S		Uncharacterized conserved protein			
===								5	Acidianus_hospitalis_W1_uid66875	NC_015518	chromosome										
332797580	hypothetical protein Ahos_1909	No	0			1732615	1733031	+	Acidianus_hospitalis_W1_uid66875	NC_015518	chromosome										
332797581	hypothetical protein Ahos_1910	No	0			1733091	1733417	-	Acidianus_hospitalis_W1_uid66875	NC_015518	chromosome										
332797582	hypothetical protein Ahos_1911	No	0			1734727	1735029	+	Acidianus_hospitalis_W1_uid66875	NC_015518	chromosome										
332797583	hypothetical protein Ahos_1912	No	7			1735202	1736011	-	Acidianus_hospitalis_W1_uid66875	NC_015518	chromosome		arCOG01572	1	(-)	S		Uncharacterized conserved membrane protein			
332797584	hypothetical protein Ahos_1913	No	0			1736514	1736714	-	Acidianus_hospitalis_W1_uid66875	NC_015518	chromosome										
===								8	Acidianus_hospitalis_W1_uid66875	NC_015518	chromosome										
332797799	hypothetical protein Ahos_2130	No	0			1939150	1939545	-	Acidianus_hospitalis_W1_uid66875	NC_015518	chromosome										
332797800	hypothetical protein Ahos_2131	No	0			1939594	1939752	-	Acidianus_hospitalis_W1_uid66875	NC_015518	chromosome		arCOG07723	1	(-)	V		RHH/CopG DNA binding protein			
332797801	hypothetical protein Ahos_2132	Yes	1			1939810	1940190	-	Acidianus_hospitalis_W1_uid66875	NC_015518	chromosome										
332797802	hypothetical protein Ahos_2133	Yes	4			1940226	1940588	-	Acidianus_hospitalis_W1_uid66875	NC_015518	chromosome										
332797803	hypothetical protein Ahos_2134	No	0			1940628	1941014	-	Acidianus_hospitalis_W1_uid66875	NC_015518	chromosome										
332797804	hypothetical protein Ahos_2135	No	2			1941703	1942056	-	Acidianus_hospitalis_W1_uid66875	NC_015518	chromosome		arCOG09866	2		S		Uncharacterized conserved protein			
332797805	hypothetical protein Ahos_2136	Yes	1			1942116	1943132	-	Acidianus_hospitalis_W1_uid66875	NC_015518	chromosome										
332797806	fuselloviral-related integrase	No	0			1943845	1944660	+	Acidianus_hospitalis_W1_uid66875	NC_015518	chromosome		arCOG01244	9	(-)	L		Intergrase/Recombinase	1E-32	COG4342	Uncharacterized protein conserved in arc
===								5	Acidilobus_saccharovorans_345_15_uid51395	NC_014374	chromosome										
302348490	hypothetical protein ASAC_0691	No	0			698152	698322	-	Acidilobus_saccharovorans_345_15_uid51395	NC_014374	chromosome										
302348491	hypothetical protein ASAC_0692	No	1			698662	698952	-	Acidilobus_saccharovorans_345_15_uid51395	NC_014374	chromosome										
302348492	Fe-S cluster-containing protein	No	0			699346	699849	-	Acidilobus_saccharovorans_345_15_uid51395	NC_014374	chromosome		arCOG01502	1	(-)	C	HycB	Fe-S-cluster-containing hydrogenase component	6E-26	COG1142	Fe-S-cluster-containing hydrogenase comp
302348493	hypothetical protein ASAC_0694	Yes	3			699917	701338	-	Acidilobus_saccharovorans_345_15_uid51395	NC_014374	chromosome		arCOG07182	3		S		Uncharacterized conserved protein			
302348494	hypothetical protein ASAC_0695	Yes	4			701335	702072	-	Acidilobus_saccharovorans_345_15_uid51395	NC_014374	chromosome										
===								5	Aciduliprofundum_MAR08_339_uid184407	NC_019942	chromosome										
432328092	hypothetical protein AciM339_0173	No	0			158213	158623	-	Aciduliprofundum_MAR08_339_uid184407	NC_019942	chromosome	clust_255									
432328093	hypothetical protein AciM339_0174	No	0			158623	158721	-	Aciduliprofundum_MAR08_339_uid184407	NC_019942	chromosome	clust_516									
432328094	hypothetical protein AciM339_0175	No	0			158718	158903	-	Aciduliprofundum_MAR08_339_uid184407	NC_019942	chromosome	clust_473									
432328095	hypothetical protein AciM339_0176	No	0			158900	159076	-	Aciduliprofundum_MAR08_339_uid184407	NC_019942	chromosome		arCOG04013	1	(-)	S		Uncharacterized conserved protein			
432328096	hypothetical protein AciM339_0177	No	0			159073	159279	-	Aciduliprofundum_MAR08_339_uid184407	NC_019942	chromosome	clust_449									
===								17	Aciduliprofundum_MAR08_339_uid184407	NC_019942	chromosome										
432328100	hypothetical protein AciM339_0181	No	2			161044	161232	+	Aciduliprofundum_MAR08_339_uid184407	NC_019942	chromosome	clust_469									
432328101	site-specific recombinase, DNA invertase Pin	No	0			161407	161997	-	Aciduliprofundum_MAR08_339_uid184407	NC_019942	chromosome		arCOG03162	12	(-)	L	PinR	Site-specific recombinase, DNA invertase Pin ho	6E-34	COG1961	Site-specific recombinases, DNA invertas
432328102	hypothetical protein AciM339_0183	No	2			162102	162338	-	Aciduliprofundum_MAR08_339_uid184407	NC_019942	chromosome	clust_412									
432328103	hypothetical protein AciM339_0184	No	0			162357	163451	-	Aciduliprofundum_MAR08_339_uid184407	NC_019942	chromosome	clust_88									
432328104	hypothetical protein AciM339_0185	No	0			163448	163810	-	Aciduliprofundum_MAR08_339_uid184407	NC_019942	chromosome	clust_297									
432328105	hypothetical protein AciM339_0186	Yes	3			163788	164615	-	Aciduliprofundum_MAR08_339_uid184407	NC_019942	chromosome	clust_118									
432328106	hypothetical protein AciM339_0187	No	2			164608	164982	-	Aciduliprofundum_MAR08_339_uid184407	NC_019942	chromosome	clust_286									
432328107	hypothetical protein AciM339_0188	No	2			164979	165614	-	Aciduliprofundum_MAR08_339_uid184407	NC_019942	chromosome	clust_162									
432328108	hypothetical protein AciM339_0189	No	1			165618	165965	-	Aciduliprofundum_MAR08_339_uid184407	NC_019942	chromosome	clust_307									
432328109	hypothetical protein AciM339_0190	No	0			165972	166133	-	Aciduliprofundum_MAR08_339_uid184407	NC_019942	chromosome	clust_494									
432328110	hypothetical protein AciM339_0191	No	0			166136	166273	-	Aciduliprofundum_MAR08_339_uid184407	NC_019942	chromosome	clust_504									
432328111	hypothetical protein AciM339_0192	No	0			166270	166557	-	Aciduliprofundum_MAR08_339_uid184407	NC_019942	chromosome	clust_364									
432328112	hypothetical protein AciM339_0193	Yes	2			166550	167857	-	Aciduliprofundum_MAR08_339_uid184407	NC_019942	chromosome	clust_79									
432328113	hypothetical protein AciM339_0194	Yes	3			167854	169374	-	Aciduliprofundum_MAR08_339_uid184407	NC_019942	chromosome	clust_73									
432328114	hypothetical protein AciM339_0195	No	2			169359	169655	-	Aciduliprofundum_MAR08_339_uid184407	NC_019942	chromosome	clust_355									
432328115	hypothetical protein AciM339_0196	No	2			169656	169907	-	Aciduliprofundum_MAR08_339_uid184407	NC_019942	chromosome	clust_396									
432328116	hypothetical protein AciM339_0197	Yes	2			169971	171845	-	Aciduliprofundum_MAR08_339_uid184407	NC_019942	chromosome	arCOG07813,a	arCOG07813	7	(-)	G		Glycosyl hydrolases family 43	0.0001	COG3507	Beta-xylosidase
===								12	Aciduliprofundum_MAR08_339_uid184407	NC_019942	chromosome										
432328118	hypothetical protein AciM339_0199	No	0			172685	172939	-	Aciduliprofundum_MAR08_339_uid184407	NC_019942	chromosome	clust_23									
432328119	hypothetical protein AciM339_0200	No	0			172933	173163	-	Aciduliprofundum_MAR08_339_uid184407	NC_019942	chromosome	clust_420									
432328120	Bacterial regulatory protein, arsR family	No	0			173160	174047	-	Aciduliprofundum_MAR08_339_uid184407	NC_019942	chromosome		arCOG01683	3	(-)	K	ArsR	Transcriptional regulator containing HTH domain, ArsR family			
432328121	hypothetical protein AciM339_0202	No	0			174174	174482	-	Aciduliprofundum_MAR08_339_uid184407	NC_019942	chromosome	clust_347									
432328122	hypothetical protein AciM339_0203	No	0			174552	174659	-	Aciduliprofundum_MAR08_339_uid184407	NC_019942	chromosome	clust_514									
432328123	hypothetical protein AciM339_0204	No	0			174656	174865	-	Aciduliprofundum_MAR08_339_uid184407	NC_019942	chromosome										
432328124	site-specific recombinase XerD	No	0			174880	175704	-	Aciduliprofundum_MAR08_339_uid184407	NC_019942	chromosome		arCOG01241	31	(-)	L	XerC	Integrase	7E-11	COG4974	Site-specific recombinase XerD
432328125	hypothetical protein AciM339_0206	No	0			175734	176144	-	Aciduliprofundum_MAR08_339_uid184407	NC_019942	chromosome	clust_255									
432328126	hypothetical protein AciM339_0207	No	0			176144	176242	-	Aciduliprofundum_MAR08_339_uid184407	NC_019942	chromosome	clust_516									
432328127	hypothetical protein AciM339_0208	No	0			176239	176424	-	Aciduliprofundum_MAR08_339_uid184407	NC_019942	chromosome	clust_473									
432328128	hypothetical protein AciM339_0209	No	0			176594	176800	-	Aciduliprofundum_MAR08_339_uid184407	NC_019942	chromosome	clust_449									
432328129	looped-hinge helix DNA binding domain, AbrB	No	0			176790	176942	-	Aciduliprofundum_MAR08_339_uid184407	NC_019942	chromosome		arCOG00818	3	(-)	K	AbrB	Transcriptional regulator AbrB			
===								17	Aciduliprofundum_MAR08_339_uid184407	NC_019942	chromosome										
432328132	hypothetical protein AciM339_0213	No	2			178565	178753	+	Aciduliprofundum_MAR08_339_uid184407	NC_019942	chromosome	clust_469									
432328133	site-specific recombinase, DNA invertase Pin	No	0			178928	179518	-	Aciduliprofundum_MAR08_339_uid184407	NC_019942	chromosome		arCOG03162	12	(-)	L	PinR	Site-specific recombinase, DNA invertase Pin ho	6E-34	COG1961	Site-specific recombinases, DNA invertas
432328134	hypothetical protein AciM339_0215	No	2			179623	179859	-	Aciduliprofundum_MAR08_339_uid184407	NC_019942	chromosome	clust_412									
432328135	hypothetical protein AciM339_0216	No	0			179878	180972	-	Aciduliprofundum_MAR08_339_uid184407	NC_019942	chromosome	clust_88									
432328136	hypothetical protein AciM339_0217	No	0			180969	181331	-	Aciduliprofundum_MAR08_339_uid184407	NC_019942	chromosome	clust_297									
432328137	hypothetical protein AciM339_0218	Yes	3			181309	182136	-	Aciduliprofundum_MAR08_339_uid184407	NC_019942	chromosome	clust_118									
432328138	hypothetical protein AciM339_0219	No	2			182129	182503	-	Aciduliprofundum_MAR08_339_uid184407	NC_019942	chromosome	clust_286									
432328139	hypothetical protein AciM339_0220	No	2			182500	183135	-	Aciduliprofundum_MAR08_339_uid184407	NC_019942	chromosome	clust_162									
432328140	hypothetical protein AciM339_0221	No	1			183139	183486	-	Aciduliprofundum_MAR08_339_uid184407	NC_019942	chromosome	clust_307									
432328141	hypothetical protein AciM339_0222	No	0			183493	183654	-	Aciduliprofundum_MAR08_339_uid184407	NC_019942	chromosome	clust_494									
432328142	hypothetical protein AciM339_0223	No	0			183657	183794	-	Aciduliprofundum_MAR08_339_uid184407	NC_019942	chromosome	clust_504									
432328143	hypothetical protein AciM339_0224	No	0			183791	184078	-	Aciduliprofundum_MAR08_339_uid184407	NC_019942	chromosome	clust_364									
432328144	hypothetical protein AciM339_0225	Yes	2			184071	185378	-	Aciduliprofundum_MAR08_339_uid184407	NC_019942	chromosome	clust_79									
432328145	hypothetical protein AciM339_0226	Yes	3			185375	186895	-	Aciduliprofundum_MAR08_339_uid184407	NC_019942	chromosome	clust_73									
432328146	hypothetical protein AciM339_0227	No	2			186880	187176	-	Aciduliprofundum_MAR08_339_uid184407	NC_019942	chromosome	clust_355									
432328147	hypothetical protein AciM339_0228	No	2			187177	187428	-	Aciduliprofundum_MAR08_339_uid184407	NC_019942	chromosome	clust_396									
432328148	hypothetical protein AciM339_0229	Yes	1			187457	189367	-	Aciduliprofundum_MAR08_339_uid184407	NC_019942	chromosome	arCOG07813,a	arCOG07813	7	(-)	G		Glycosyl hydrolases family 43	0.0001	COG3507	Beta-xylosidase
===								5	Aciduliprofundum_MAR08_339_uid184407	NC_019942	chromosome										
432328150	hypothetical protein AciM339_0231	No	0			190207	190461	-	Aciduliprofundum_MAR08_339_uid184407	NC_019942	chromosome	clust_23									
432328151	hypothetical protein AciM339_0232	No	0			190455	190685	-	Aciduliprofundum_MAR08_339_uid184407	NC_019942	chromosome	clust_420									
432328152	Bacterial regulatory protein, arsR family	No	0			190682	191569	-	Aciduliprofundum_MAR08_339_uid184407	NC_019942	chromosome		arCOG01683	3	(-)	K	ArsR	Transcriptional regulator containing HTH domain, ArsR family			
432328153	hypothetical protein AciM339_0234	No	0			191696	192004	-	Aciduliprofundum_MAR08_339_uid184407	NC_019942	chromosome	clust_347									
432328154	hypothetical protein AciM339_0235	No	0			192074	192181	-	Aciduliprofundum_MAR08_339_uid184407	NC_019942	chromosome	clust_514									
===								6	Aciduliprofundum_MAR08_339_uid184407	NC_019942	chromosome										
432328279	putative RNA-binding protein, snRNP like prote	No	0			319203	320993	-	Aciduliprofundum_MAR08_339_uid184407	NC_019942	chromosome		arCOG01695	1	(-)	K		Predicted RNA-binding protein homologous to eu	3E-76	COG1293	Predicted RNA-binding protein homologous
432328280	hypothetical protein AciM339_0369	No	4			321313	321741	-	Aciduliprofundum_MAR08_339_uid184407	NC_019942	chromosome										
432328281	Caspase domain-containing protein	No	0			321752	322627	-	Aciduliprofundum_MAR08_339_uid184407	NC_019942	chromosome										
432328282	hypothetical protein AciM339_0371	Yes	1			322596	323579	-	Aciduliprofundum_MAR08_339_uid184407	NC_019942	chromosome										
432328283	Peptidase C13 family	No	0			323590	324186	-	Aciduliprofundum_MAR08_339_uid184407	NC_019942	chromosome										
432328284	hypothetical protein AciM339_0373	No	0			324246	325448	-	Aciduliprofundum_MAR08_339_uid184407	NC_019942	chromosome		arCOG07561	7	(-)	K		Predicted regulatory protein containing HTH domain			
===								5	Aciduliprofundum_MAR08_339_uid184407	NC_019942	chromosome										
432328397	PKD domain protein,CARDB domain-containing	Yes	2			423516	426500	-	Aciduliprofundum_MAR08_339_uid184407	NC_019942	chromosome		arCOG07368	2		S		Cell suface protein	0.00009	pfam07705	CARDB. Cell adhesion related domain foun
432328398	putative membrane protein	No	11			426620	427879	+	Aciduliprofundum_MAR08_339_uid184407	NC_019942	chromosome		arCOG04469	2	(-)	S		Predicted membrane protein	1E-65	COG1784	Predicted membrane protein
432328399	hypothetical protein AciM339_0490	No	0			427934	428539	+	Aciduliprofundum_MAR08_339_uid184407	NC_019942	chromosome	clust_169									
432328400	hypothetical protein AciM339_0491	No	0			428536	428814	-	Aciduliprofundum_MAR08_339_uid184407	NC_019942	chromosome	clust_368									
432328401	hypothetical protein AciM339_0492	No	0			428811	429152	-	Aciduliprofundum_MAR08_339_uid184407	NC_019942	chromosome	clust_311									
===								6	Aciduliprofundum_MAR08_339_uid184407	NC_019942	chromosome										
432328597	Zn-dependent protease with chaperone functio	No	4			600496	601209	-	Aciduliprofundum_MAR08_339_uid184407	NC_019942	chromosome		arCOG01331	2	(-)	O	HtpX	Zn-dependent protease with chaperone function	1E-13	COG0501	Zn-dependent protease with chaperone fun
432328598	hypothetical protein AciM339_0695	No	4			601199	601726	-	Aciduliprofundum_MAR08_339_uid184407	NC_019942	chromosome										
432328599	hypothetical protein AciM339_0696	No	4			601719	602231	-	Aciduliprofundum_MAR08_339_uid184407	NC_019942	chromosome										
432328600	hypothetical protein AciM339_0697	No	4			602231	602764	-	Aciduliprofundum_MAR08_339_uid184407	NC_019942	chromosome										
432328601	hypothetical protein AciM339_0698	No	4			602757	603287	-	Aciduliprofundum_MAR08_339_uid184407	NC_019942	chromosome										
432328602	hypothetical protein AciM339_0699	No	0			603371	603739	-	Aciduliprofundum_MAR08_339_uid184407	NC_019942	chromosome		arCOG02734	5	(-)	S		NifX family protein	6E-20	COG1433	Uncharacterized conserved protein
===								9	Aciduliprofundum_MAR08_339_uid184407	NC_019942	chromosome										
432328637	hypothetical protein AciM339_0735	No	2			640384	640596	+	Aciduliprofundum_MAR08_339_uid184407	NC_019942	chromosome										
432328638	hypothetical protein AciM339_0736	No	0			640580	640744	-	Aciduliprofundum_MAR08_339_uid184407	NC_019942	chromosome										
432328639	acyl-CoA synthetase (NDP forming)	No	0			640879	643014	+	Aciduliprofundum_MAR08_339_uid184407	NC_019942	chromosome	arCOG01338,a	arCOG01338	1	(-)	C		Acyl-CoA synthetase, ATP-grasp containing subu	2E-162	COG1042	Acyl-CoA synthetase (NDP forming)
432328640	hypothetical protein AciM339_0738	No	1			643151	643432	+	Aciduliprofundum_MAR08_339_uid184407	NC_019942	chromosome										
432328641	hypothetical protein AciM339_0739	No	0			643590	643901	+	Aciduliprofundum_MAR08_339_uid184407	NC_019942	chromosome										
432328642	hypothetical protein AciM339_0740	Yes	1			643970	644803	+	Aciduliprofundum_MAR08_339_uid184407	NC_019942	chromosome										
432328643	ABC-type multidrug transport system, ATPase	No	0			644751	645620	+	Aciduliprofundum_MAR08_339_uid184407	NC_019942	chromosome		arCOG00194	14	(-)	V	CcmA	ABC-type multidrug transport system, ATPase co	3E-40	COG1131	ABC-type multidrug transport system, ATP
432328644	hypothetical protein AciM339_0742	No	6			645610	646509	+	Aciduliprofundum_MAR08_339_uid184407	NC_019942	chromosome										
432328645	hypothetical protein AciM339_0743	No	1			646502	646972	+	Aciduliprofundum_MAR08_339_uid184407	NC_019942	chromosome										
===								8	Aciduliprofundum_MAR08_339_uid184407	NC_019942	chromosome										
432329107	Protein of unknown function (DUF2791)	No	0			1099273	1100502	-	Aciduliprofundum_MAR08_339_uid184407	NC_019942	chromosome		arCOG06460	1	(-)	R		Predicted ATPase	1E-16	pfam10923	P-loop Domain of unknown function (DUF27
432329108	hypothetical protein AciM339_1224	No	0			1100499	1101344	-	Aciduliprofundum_MAR08_339_uid184407	NC_019942	chromosome								0.00006	COG1293	Predicted RNA-binding protein homologous
432329109	hypothetical protein AciM339_1225	No	1			1101616	1101906	+	Aciduliprofundum_MAR08_339_uid184407	NC_019942	chromosome										
432329110	hypothetical protein AciM339_1226	No	1			1101846	1102220	+	Aciduliprofundum_MAR08_339_uid184407	NC_019942	chromosome										
432329111	hypothetical protein AciM339_1227	No	0			1103109	1103597	+	Aciduliprofundum_MAR08_339_uid184407	NC_019942	chromosome										
432329112	hypothetical protein AciM339_1228	No	0			1103780	1104709	-	Aciduliprofundum_MAR08_339_uid184407	NC_019942	chromosome										
432329113	hypothetical protein AciM339_1229	No	2			1104860	1105081	+	Aciduliprofundum_MAR08_339_uid184407	NC_019942	chromosome										
432329114	hypothetical protein AciM339_1230	No	2			1105095	1105307	-	Aciduliprofundum_MAR08_339_uid184407	NC_019942	chromosome		arCOG06261	1	(-)	S		Uncharacterized conserved protein			
===								7	Aciduliprofundum_MAR08_339_uid184407	NC_019942	chromosome										
432329297	ERCC4-like helicase	No	0			1294441	1296702	+	Aciduliprofundum_MAR08_339_uid184407	NC_019942	chromosome		arCOG00872	1	(-)	L	MPH1	ERCC4-like helicase	6E-162	COG1111	ERCC4-like helicases
432329298	hypothetical protein AciM339_1423	No	1			1297100	1297390	+	Aciduliprofundum_MAR08_339_uid184407	NC_019942	chromosome										
432329299	hypothetical protein AciM339_1424	No	1			1297378	1297737	+	Aciduliprofundum_MAR08_339_uid184407	NC_019942	chromosome										
432329300	hypothetical protein AciM339_1425	No	0			1297776	1298903	-	Aciduliprofundum_MAR08_339_uid184407	NC_019942	chromosome										
432329301	Ribbon-helix-helix protein, copG family	No	0			1298964	1299155	+	Aciduliprofundum_MAR08_339_uid184407	NC_019942	chromosome										
432329302	hypothetical protein AciM339_1427	No	0			1299639	1300448	+	Aciduliprofundum_MAR08_339_uid184407	NC_019942	chromosome										
432329303	hypothetical protein AciM339_1428	No	3			1300696	1302111	-	Aciduliprofundum_MAR08_339_uid184407	NC_019942	chromosome		arCOG05096	3	(-)	S		Uncharacterized conserved membrane protein			
===								6	Aciduliprofundum_boonei_T469_uid43333	NC_013926	chromosome										
289595937	hypothetical protein Aboo_0259	No	0	YES		265096	265503	+	Aciduliprofundum_boonei_T469_uid43333	NC_013926	chromosome										
289595938	hypothetical protein Aboo_0260	No	0	YES		265500	266228	+	Aciduliprofundum_boonei_T469_uid43333	NC_013926	chromosome										
289595939	hypothetical protein Aboo_0261	No	0	YES		266242	266382	+	Aciduliprofundum_boonei_T469_uid43333	NC_013926	chromosome										
289595940	hypothetical protein Aboo_0262	No	0	YES		266472	266681	-	Aciduliprofundum_boonei_T469_uid43333	NC_013926	chromosome		arCOG01665	6	(-)	V	RelE	Cytotoxic translational repressor of toxin-antitoxin	0.00002	COG2026	Cytotoxic translational repressor of tox
289595941	tumor suppressor TSBF1-like protein	No	0	YES		266678	266821	-	Aciduliprofundum_boonei_T469_uid43333	NC_013926	chromosome										
289595942	Viral A-type inclusion protein repeat containing	No	0	YES		267087	267275	+	Aciduliprofundum_boonei_T469_uid43333	NC_013926	chromosome										
===								7	Aciduliprofundum_boonei_T469_uid43333	NC_013926	chromosome										
289596232	circadian clock protein, KaiC	No	0			537054	537743	+	Aciduliprofundum_boonei_T469_uid43333	NC_013926	chromosome		arCOG01171	2	(-)	T	RAD55	RecA-superfamily ATPase implicated in signal tra	4E-38	COG0467	RecA-superfamily ATPases implicated in s
289596233	hypothetical protein Aboo_0556	No	0			537753	538094	+	Aciduliprofundum_boonei_T469_uid43333	NC_013926	chromosome	clust_311									
289596234	hypothetical protein Aboo_0557	No	0			538091	538372	+	Aciduliprofundum_boonei_T469_uid43333	NC_013926	chromosome	clust_368									
289596235	hypothetical protein Aboo_0558	No	0			538369	538980	-	Aciduliprofundum_boonei_T469_uid43333	NC_013926	chromosome	clust_169									
289596236	hypothetical protein Aboo_0559	No	3			539107	539523	+	Aciduliprofundum_boonei_T469_uid43333	NC_013926	chromosome										
289596237	protein of unknown function DUF112 transmem	No	11			539527	540783	-	Aciduliprofundum_boonei_T469_uid43333	NC_013926	chromosome		arCOG04469	2	(-)	S		Predicted membrane protein	1E-64	COG1784	Predicted membrane protein
289596238	Cna B domain protein	Yes	2			540904	543921	+	Aciduliprofundum_boonei_T469_uid43333	NC_013926	chromosome		arCOG07368	2		S		Cell suface protein			
===								11	Aciduliprofundum_boonei_T469_uid43333	NC_013926	chromosome										
289596674	hypothetical protein Aboo_0999	No	0	YES		963072	963452	-	Aciduliprofundum_boonei_T469_uid43333	NC_013926	chromosome										
289596675	hypothetical protein Aboo_1000	No	0	YES		963455	963655	-	Aciduliprofundum_boonei_T469_uid43333	NC_013926	chromosome										
289596676	hypothetical protein Aboo_1001	No	0	YES		963659	964564	-	Aciduliprofundum_boonei_T469_uid43333	NC_013926	chromosome		arCOG00790	6	(-)	V	Cas4	CRISPR-associated protein Cas4, RecB family exonuclease			
289596677	hypothetical protein Aboo_1002	No	0	YES		964530	964790	-	Aciduliprofundum_boonei_T469_uid43333	NC_013926	chromosome										
289596678	zinc finger SWIM domain protein	No	0	YES		964795	965688	-	Aciduliprofundum_boonei_T469_uid43333	NC_013926	chromosome										
289596679	hypothetical protein Aboo_1004	No	0	YES		965698	965997	-	Aciduliprofundum_boonei_T469_uid43333	NC_013926	chromosome										
289596680	hypothetical protein Aboo_1005	No	0	YES		966012	966212	-	Aciduliprofundum_boonei_T469_uid43333	NC_013926	chromosome										
289596681	hypothetical protein Aboo_1006	No	0	YES		966209	966406	-	Aciduliprofundum_boonei_T469_uid43333	NC_013926	chromosome										
289596682	hypothetical protein Aboo_1007	No	0	YES		966403	966594	-	Aciduliprofundum_boonei_T469_uid43333	NC_013926	chromosome										
289596683	K08849 mixed lineage kinase domain-like prot	No	0	YES		966591	966782	-	Aciduliprofundum_boonei_T469_uid43333	NC_013926	chromosome										
289596684	hypothetical protein Aboo_1009	No	0	YES		966779	968089	-	Aciduliprofundum_boonei_T469_uid43333	NC_013926	chromosome		arCOG07546	3	(-)	E		Zincin superfamily protease			
===								5	Aciduliprofundum_boonei_T469_uid43333	NC_013926	chromosome										
289596688	hypothetical protein Aboo_1013	No	0	YES		972531	973193	+	Aciduliprofundum_boonei_T469_uid43333	NC_013926	chromosome										
289596689	hypothetical protein Aboo_1014	No	0	YES		973654	974145	+	Aciduliprofundum_boonei_T469_uid43333	NC_013926	chromosome										
289596690	hypothetical protein Aboo_1015	No	0	YES		974150	974668	+	Aciduliprofundum_boonei_T469_uid43333	NC_013926	chromosome		arCOG10214	7		S		Uncharacterized conserved protein			
289596691	Type IV secretory pathway VirD4 protein-like p	No	3	YES		974668	976476	+	Aciduliprofundum_boonei_T469_uid43333	NC_013926	chromosome		arCOG04816	4	(-)	U		TraG/TraD/VirD4 family enzyme, ATPase	5E-17	pfam10412	Type IV secretion-system coupling protei ; Uncharacterized membrane protein
289596692	hypothetical protein Aboo_1017	Yes	4	YES		976523	977650	+	Aciduliprofundum_boonei_T469_uid43333	NC_013926	chromosome										
===								17	Aciduliprofundum_boonei_T469_uid43333	NC_013926	chromosome										
289596731	Type IV secretory pathway VirB4 protein-like p	No	0			1014731	1016431	-	Aciduliprofundum_boonei_T469_uid43333	NC_013926	chromosome	arCOG04035,a	arCOG04035	5	(-)	U	VirB4	Type IV secretory pathway, VirB4 component	6E-18	pfam12846	AAA-like domain. This family of domains
289596732	hypothetical protein Aboo_1057	No	3			1016425	1016832	-	Aciduliprofundum_boonei_T469_uid43333	NC_013926	chromosome										
289596733	hypothetical protein Aboo_1058	No	1			1016832	1017572	-	Aciduliprofundum_boonei_T469_uid43333	NC_013926	chromosome										
289596734	hypothetical protein Aboo_1059	No	7			1017556	1018824	-	Aciduliprofundum_boonei_T469_uid43333	NC_013926	chromosome										
289596735	hypothetical protein Aboo_1060	Yes	3			1018825	1020042	-	Aciduliprofundum_boonei_T469_uid43333	NC_013926	chromosome										
289596736	hypothetical protein Aboo_1061	Yes	3			1020046	1021008	-	Aciduliprofundum_boonei_T469_uid43333	NC_013926	chromosome								3E-10	pfam07172	Glycine rich protein family. This family
289596737	hypothetical protein Aboo_1062	Yes	1			1021005	1022033	-	Aciduliprofundum_boonei_T469_uid43333	NC_013926	chromosome								0.000001	pfam07172	Glycine rich protein family. This family
289596738	hypothetical protein Aboo_1063	Yes	2			1022030	1022956	-	Aciduliprofundum_boonei_T469_uid43333	NC_013926	chromosome										
289596739	hypothetical protein Aboo_1064	Yes	1			1022953	1023201	-	Aciduliprofundum_boonei_T469_uid43333	NC_013926	chromosome										
289596740	hypothetical protein Aboo_1065	Yes	3			1023202	1023684	-	Aciduliprofundum_boonei_T469_uid43333	NC_013926	chromosome										
289596741	hypothetical protein Aboo_1066	Yes	2			1023692	1025926	-	Aciduliprofundum_boonei_T469_uid43333	NC_013926	chromosome		arCOG03439	2	(-)	S		vWFa domain containing protein			
289596742	hypothetical protein Aboo_1067	No	0			1026104	1026343	-	Aciduliprofundum_boonei_T469_uid43333	NC_013926	chromosome	clust_23									
289596743	hypothetical protein Aboo_1068	No	0			1026336	1027682	-	Aciduliprofundum_boonei_T469_uid43333	NC_013926	chromosome										
289596744	hypothetical protein Aboo_1069	No	0			1027679	1027795	-	Aciduliprofundum_boonei_T469_uid43333	NC_013926	chromosome										
289596745	glycoside hydrolase family 5 domain protein	No	0			1027792	1028037	-	Aciduliprofundum_boonei_T469_uid43333	NC_013926	chromosome	clust_398									
289596746	protein of unknown function DUF46	Yes	5			1028752	1029312	+	Aciduliprofundum_boonei_T469_uid43333	NC_013926	chromosome		arCOG04106	1	(-)	I	CdsA	CDP-diglyceride synthetase	1E-46	pfam01864	Putative integral membrane protein DUF46
289596747	hypothetical protein Aboo_1072	Yes	1			1029345	1030073	+	Aciduliprofundum_boonei_T469_uid43333	NC_013926	chromosome										
===								5	Aciduliprofundum_boonei_T469_uid43333	NC_013926	chromosome										
289596838	hypothetical protein Aboo_1163	No	0			1113084	1113566	+	Aciduliprofundum_boonei_T469_uid43333	NC_013926	chromosome								0.000001	COG1340	Uncharacterized archaeal coiled-coil pro
289596839	transposase IS4 family protein	No	0			1113583	1114413	+	Aciduliprofundum_boonei_T469_uid43333	NC_013926	chromosome		arCOG02751	5	(-)	L		Transposase, IS5 family			
289596840	hypothetical protein Aboo_1165	No	0			1114443	1114691	+	Aciduliprofundum_boonei_T469_uid43333	NC_013926	chromosome								0.0001	COG1340	Uncharacterized archaeal coiled-coil pro
289596841	hypothetical protein Aboo_1166	No	3			1114688	1114999	+	Aciduliprofundum_boonei_T469_uid43333	NC_013926	chromosome										
289596842	hypothetical protein Aboo_1167	No	1			1114996	1115547	+	Aciduliprofundum_boonei_T469_uid43333	NC_013926	chromosome										
===								5	Aciduliprofundum_boonei_T469_uid43333	NC_013926	chromosome										
289596879	hypothetical protein Aboo_1204	No	0			1149071	1149310	+	Aciduliprofundum_boonei_T469_uid43333	NC_013926	chromosome	clust_398									
289596880	hypothetical protein Aboo_1205	No	0			1149363	1150703	+	Aciduliprofundum_boonei_T469_uid43333	NC_013926	chromosome										
289596881	hypothetical protein Aboo_1206	No	0			1150696	1150935	+	Aciduliprofundum_boonei_T469_uid43333	NC_013926	chromosome	clust_23									
289596882	ATPase	No	0			1151081	1152307	-	Aciduliprofundum_boonei_T469_uid43333	NC_013926	chromosome		arCOG03166	9	(-)	R		AAA+ superfamily ATPase fused to HTH and Rec	0.0000004	COG1672	Predicted ATPase (AAA+ superfamily)
289596883	CopG family transcriptional regulator	No	0			1152619	1153380	-	Aciduliprofundum_boonei_T469_uid43333	NC_013926	chromosome										
===								8	Aciduliprofundum_boonei_T469_uid43333	NC_013926	chromosome										
289596885	succinyl-CoA synthetase (alpha subunit)	No	0			1157900	1158106	+	Aciduliprofundum_boonei_T469_uid43333	NC_013926	chromosome										
289596886	hypothetical protein Aboo_1211	No	0			1158166	1158321	+	Aciduliprofundum_boonei_T469_uid43333	NC_013926	chromosome		arCOG09828	3		V		RelB family antitoxin			
289596887	hypothetical protein Aboo_1212	No	0			1158456	1159739	+	Aciduliprofundum_boonei_T469_uid43333	NC_013926	chromosome		arCOG03167	6	(-)	R		Predicted ATPase (AAA+ superfamily)	8E-62	COG1373	Predicted ATPase (AAA+ superfamily)
289596888	hypothetical protein Aboo_1213	No	0			1159935	1160150	+	Aciduliprofundum_boonei_T469_uid43333	NC_013926	chromosome		arCOG09828	3		V		RelB family antitoxin			
289596889	hypothetical protein Aboo_1215	No	0			1160422	1160709	-	Aciduliprofundum_boonei_T469_uid43333	NC_013926	chromosome										
289596890	hypothetical protein Aboo_1216	No	0			1160702	1161007	-	Aciduliprofundum_boonei_T469_uid43333	NC_013926	chromosome										
289596891	integrase family protein	No	0			1161101	1162168	+	Aciduliprofundum_boonei_T469_uid43333	NC_013926	chromosome		arCOG01241	31	(-)	L	XerC	Integrase	5E-18	COG4974	Site-specific recombinase XerD
289596892	hypothetical protein Aboo_1218	No	1			1162293	1163345	+	Aciduliprofundum_boonei_T469_uid43333	NC_013926	chromosome										
===								5	Aeropyrum_pernix_K1_uid57757	NC_000854	chromosome										
14600737	hypothetical protein APE_0471c	Yes	0	YES		328922	329092	-	Aeropyrum_pernix_K1_uid57757	NC_000854	chromosome										
14600738	hypothetical protein APE_0472	No	0	YES		329133	329651	-	Aeropyrum_pernix_K1_uid57757	NC_000854	chromosome		arCOG02121	1	(-)	V		PIN domain containing protein	7E-23	COG5378	Predicted nucleotide-binding protein
118431065	hypothetical protein APE_0472a	No	0	YES		329606	329833	-	Aeropyrum_pernix_K1_uid57757	NC_000854	chromosome		arCOG06086	2		S		Uncharacterized conserved protein			
118431066	hypothetical protein APE_0472b.1	No	0	YES		329873	330058	-	Aeropyrum_pernix_K1_uid57757	NC_000854	chromosome		arCOG10167	2		V		DNA-binding protein, potential antitoxin AbrB/MazE fold			
118431067	hypothetical protein APE_0472c	No	0	YES		330150	330287	+	Aeropyrum_pernix_K1_uid57757	NC_000854	chromosome		arCOG07843	4		S		Uncharacterized conserved protein			
===								16	Aeropyrum_pernix_K1_uid57757	NC_000854	chromosome										
14600918	hypothetical protein APE_0722	Yes	0		integrated_ele	480774	481433	+	Aeropyrum_pernix_K1_uid57757	NC_000854	chromosome		arCOG04268	1	(-)	L	Cdt1	Replication initiator protein WhiP, Cdt1-like protein, contains HTH domain			
118431169	hypothetical protein APE_0722a	No	0		integrated_ele	481345	481620	-	Aeropyrum_pernix_K1_uid57757	NC_000854	chromosome										
118431170	hypothetical protein APE_0722b	No	0		integrated_ele	481978	482151	-	Aeropyrum_pernix_K1_uid57757	NC_000854	chromosome										
118431171	hypothetical protein APE_0722c	No	3		integrated_ele	482257	482580	-	Aeropyrum_pernix_K1_uid57757	NC_000854	chromosome										
118431172	hypothetical protein APE_0725.1	Yes	0		integrated_ele	482633	484669	-	Aeropyrum_pernix_K1_uid57757	NC_000854	chromosome										
14600923	hypothetical protein APE_0727	No	0		integrated_ele	484760	485614	-	Aeropyrum_pernix_K1_uid57757	NC_000854	chromosome		arCOG06087	1		S		Uncharacterized conserved protein	0.0001	pfam03659	Glycosyl hydrolase family 71. Family of
14600924	hypothetical protein APE_0728	Yes	2		integrated_ele	485768	486436	-	Aeropyrum_pernix_K1_uid57757	NC_000854	chromosome										
118431173	hypothetical protein APE_0728a	No	2		integrated_ele	486458	486715	-	Aeropyrum_pernix_K1_uid57757	NC_000854	chromosome										
118431174	hypothetical protein APE_0728b	No	0		integrated_ele	486804	487088	+	Aeropyrum_pernix_K1_uid57757	NC_000854	chromosome										
14600926	hypothetical protein APE_0730	No	0		integrated_ele	487176	487541	-	Aeropyrum_pernix_K1_uid57757	NC_000854	chromosome		arCOG04146	2	(-)	S		Uncharacterized conserved protein			
118431175	hypothetical protein APE_0730a	No	0		integrated_ele	487552	488331	-	Aeropyrum_pernix_K1_uid57757	NC_000854	chromosome										
14600928	hypothetical protein APE_0731	Yes	0		integrated_ele	488396	489571	-	Aeropyrum_pernix_K1_uid57757	NC_000854	chromosome										
14600931	hypothetical protein APE_0734	No	2		integrated_ele	489628	490233	+	Aeropyrum_pernix_K1_uid57757	NC_000854	chromosome										
118431176	hypothetical protein APE_0735.1	No	0		integrated_ele	490226	490624	+	Aeropyrum_pernix_K1_uid57757	NC_000854	chromosome										
14600933	hypothetical protein APE_0736	No	0		integrated_ele	490641	491384	+	Aeropyrum_pernix_K1_uid57757	NC_000854	chromosome		arCOG07960	16	(-)	R		ATPase (AAA+ superfamily)			
14600934	hypothetical protein APE_0737	Yes	3		integrated_ele	491368	491670	+	Aeropyrum_pernix_K1_uid57757	NC_000854	chromosome										
===								38	Aeropyrum_pernix_K1_uid57757	NC_000854	chromosome										
118431211	hypothetical protein APE_0816a.1	No	0		Virus_Fusellov	542538	542666	-	Aeropyrum_pernix_K1_uid57757	NC_000854	chromosome		arCOG07843	4		S		Uncharacterized conserved protein			
118431212	hypothetical protein APE_0818.1	No	0		Virus_Fusellov	542891	544132	+	Aeropyrum_pernix_K1_uid57757	NC_000854	chromosome		arCOG01247	4	(-)	L	XerD	Site-specific recombinase XerD			
118431213	hypothetical protein APE_0818a	No	0		Virus_Fusellov	544129	544380	+	Aeropyrum_pernix_K1_uid57757	NC_000854	chromosome										
118431214	hypothetical protein APE_0820.1	No	0		Virus_Fusellov	544519	544908	+	Aeropyrum_pernix_K1_uid57757	NC_000854	chromosome										
14600993	hypothetical protein APE_0821	No	0		Virus_Fusellov	544909	545889	+	Aeropyrum_pernix_K1_uid57757	NC_000854	chromosome										
14600995	hypothetical protein APE_0824	No	0		Virus_Fusellov	545948	546649	-	Aeropyrum_pernix_K1_uid57757	NC_000854	chromosome		arCOG07513	1	(-)	O	HtpX	Zn-dependent protease with chaperone function	0.00001	COG0501	Zn-dependent protease with chaperone fun
118431215	hypothetical protein APE_0825.1	No	0		Virus_Fusellov	547079	548005	+	Aeropyrum_pernix_K1_uid57757	NC_000854	chromosome										
14600997	hypothetical protein APE_0826	No	2		Virus_Fusellov	548559	549350	-	Aeropyrum_pernix_K1_uid57757	NC_000854	chromosome										
14600998	hypothetical protein APE_0826a	No	0		Virus_Fusellov	549567	549731	+	Aeropyrum_pernix_K1_uid57757	NC_000854	chromosome										
118431216	hypothetical protein APE_0826b	No	2		Virus_Fusellov	549704	549979	+	Aeropyrum_pernix_K1_uid57757	NC_000854	chromosome										
118431217	hypothetical protein APE_0827.1	No	0		Virus_Fusellov	549957	550436	+	Aeropyrum_pernix_K1_uid57757	NC_000854	chromosome										
14601001	hypothetical protein APE_0830	No	3		Virus_Fusellov	550445	551401	+	Aeropyrum_pernix_K1_uid57757	NC_000854	chromosome								0.00003	COG0419	ATPase involved in DNA repair
118431218	hypothetical protein APE_0832.1	No	0		Virus_Fusellov	551405	551809	+	Aeropyrum_pernix_K1_uid57757	NC_000854	chromosome										
118431219	hypothetical protein APE_0833.1	No	0		Virus_Fusellov	551865	552668	+	Aeropyrum_pernix_K1_uid57757	NC_000854	chromosome		arCOG07960	16	(-)	R		ATPase (AAA+ superfamily)			
14601005	hypothetical protein APE_0836	No	1		Virus_Fusellov	552807	553472	+	Aeropyrum_pernix_K1_uid57757	NC_000854	chromosome										
14601006	hypothetical protein APE_0837	No	0		Virus_Fusellov	553499	554215	+	Aeropyrum_pernix_K1_uid57757	NC_000854	chromosome										
14601009	hypothetical protein APE_0840	No	0		Virus_Fusellov	554231	555496	+	Aeropyrum_pernix_K1_uid57757	NC_000854	chromosome										
118431220	hypothetical protein APE_0840a	No	2		Virus_Fusellov	555521	555811	+	Aeropyrum_pernix_K1_uid57757	NC_000854	chromosome										
118431221	hypothetical protein APE_0843.1	Yes	3		Virus_Fusellov	555824	558604	+	Aeropyrum_pernix_K1_uid57757	NC_000854	chromosome										
118431222	hypothetical protein APE_0847.1	Yes	2		Virus_Fusellov	558639	559010	+	Aeropyrum_pernix_K1_uid57757	NC_000854	chromosome										
118431223	hypothetical protein APE_0848.1	No	2		Virus_Fusellov	559007	559300	+	Aeropyrum_pernix_K1_uid57757	NC_000854	chromosome										
14601017	hypothetical protein APE_0850	No	2		Virus_Fusellov	559331	559648	+	Aeropyrum_pernix_K1_uid57757	NC_000854	chromosome	clust_331									
118431224	hypothetical protein APE_0850a	No	2		Virus_Fusellov	559676	559942	+	Aeropyrum_pernix_K1_uid57757	NC_000854	chromosome	clust_331									
118431225	hypothetical protein APE_0852.1	Yes	4		Virus_Fusellov	559993	561267	+	Aeropyrum_pernix_K1_uid57757	NC_000854	chromosome										
118431226	hypothetical protein APE_0855.1	No	2		Virus_Fusellov	561264	561488	+	Aeropyrum_pernix_K1_uid57757	NC_000854	chromosome										
14601023	hypothetical protein APE_0856	Yes	1		Virus_Fusellov	561510	562358	+	Aeropyrum_pernix_K1_uid57757	NC_000854	chromosome										
14601024	hypothetical protein APE_0858	Yes	1		Virus_Fusellov	562446	564050	+	Aeropyrum_pernix_K1_uid57757	NC_000854	chromosome										
118431227	hypothetical protein APE_0859	No	0		Virus_Fusellov	564324	564830	+	Aeropyrum_pernix_K1_uid57757	NC_000854	chromosome		arCOG03377	4	(-)	E		Serine protease			
14601025	hypothetical protein APE_0860	Yes	3		Virus_Fusellov	564811	565827	+	Aeropyrum_pernix_K1_uid57757	NC_000854	chromosome										
118431228	hypothetical protein APE_0862.1	No	3		Virus_Fusellov	565904	566224	+	Aeropyrum_pernix_K1_uid57757	NC_000854	chromosome										
118431229	hypothetical protein APE_0864.1	No	0		Virus_Fusellov	566269	566676	+	Aeropyrum_pernix_K1_uid57757	NC_000854	chromosome										
118431230	hypothetical protein APE_0865.1	No	0		Virus_Fusellov	566712	567041	+	Aeropyrum_pernix_K1_uid57757	NC_000854	chromosome		arCOG03881	5	(-)	S		Uncharacterized conserved protein			
118431231	hypothetical protein APE_0867.1	No	0		Virus_Fusellov	567135	568112	+	Aeropyrum_pernix_K1_uid57757	NC_000854	chromosome										
118431232	hypothetical protein APE_0867a	No	0		Virus_Fusellov	568103	568360	+	Aeropyrum_pernix_K1_uid57757	NC_000854	chromosome										
14601034	hypothetical protein APE_0867b	No	1		Virus_Fusellov	568627	568878	+	Aeropyrum_pernix_K1_uid57757	NC_000854	chromosome										
118431233	hypothetical protein APE_0870.1	No	4		Virus_Fusellov	568890	569357	+	Aeropyrum_pernix_K1_uid57757	NC_000854	chromosome										
118431234	hypothetical protein APE_0871.1	No	0		Virus_Fusellov	569321	570364	-	Aeropyrum_pernix_K1_uid57757	NC_000854	chromosome										
118431235	hypothetical protein APE_0871a.1	No	0		Virus_Fusellov	570844	571044	+	Aeropyrum_pernix_K1_uid57757	NC_000854	chromosome		arCOG07514	1	(-)	V		RHH/CopG family DNA-binding protein			
===								8	Aeropyrum_pernix_K1_uid57757	NC_000854	chromosome										
118431241	hypothetical protein APE_0880c	No	0			578583	578834	+	Aeropyrum_pernix_K1_uid57757	NC_000854	chromosome										
14601046	hypothetical protein APE_0883	No	0			578834	579262	+	Aeropyrum_pernix_K1_uid57757	NC_000854	chromosome										
118431242	hypothetical protein APE_0883a	No	0			579279	579509	+	Aeropyrum_pernix_K1_uid57757	NC_000854	chromosome		arCOG03845	3	(-)	V		Predicted antitoxin, copG family			
118431243	hypothetical protein APE_0883b	No	0			579522	579803	+	Aeropyrum_pernix_K1_uid57757	NC_000854	chromosome										
118431244	hypothetical protein APE_0883c	No	0			579791	580021	+	Aeropyrum_pernix_K1_uid57757	NC_000854	chromosome										
118431245	hypothetical protein APE_0885.1	No	0			580035	580361	+	Aeropyrum_pernix_K1_uid57757	NC_000854	chromosome										
118431246	hypothetical protein APE_0885a	No	0			580367	580612	+	Aeropyrum_pernix_K1_uid57757	NC_000854	chromosome										
118431247	hypothetical protein APE_0885b	Yes	0			581194	581448	-	Aeropyrum_pernix_K1_uid57757	NC_000854	chromosome		arCOG04197	4	(-)	R		Transposase related protein			
===								7	Aeropyrum_pernix_K1_uid57757	NC_000854	chromosome										
118431441	hypothetical protein APE_1405.1	No	0			893970	894296	+	Aeropyrum_pernix_K1_uid57757	NC_000854	chromosome								0.00003	COG0711	F0F1-type ATP synthase, subunit b
118431442	hypothetical protein APE_1406.1	No	0			894303	895343	+	Aeropyrum_pernix_K1_uid57757	NC_000854	chromosome										
118431443	hypothetical protein APE_1406a	No	0			895512	895781	+	Aeropyrum_pernix_K1_uid57757	NC_000854	chromosome										
118431444	hypothetical protein APE_1407.1	No	0			895833	896633	+	Aeropyrum_pernix_K1_uid57757	NC_000854	chromosome		arCOG05634	1	(-)	O	FdhE	Uncharacterized protein involved in formate dehydrogenase formation			
14601389	hypothetical protein APE_1408	No	1			896868	897626	-	Aeropyrum_pernix_K1_uid57757	NC_000854	chromosome										
118431445	hypothetical protein APE_1409.1	No	6			897633	898628	-	Aeropyrum_pernix_K1_uid57757	NC_000854	chromosome										
118431446	hypothetical protein APE_1409a	No	0			898978	899073	-	Aeropyrum_pernix_K1_uid57757	NC_000854	chromosome										
===								6	Archaeoglobus_fulgidus_DSM_4304_uid57717	NC_000917	chromosome										
11497714	molybdate ABC transporter periplasmic substra	Yes	1			91373	92401	-	Archaeoglobus_fulgidus_DSM_4304_uid57717	NC_000917	chromosome		arCOG00219	1	(-)	P	ModA	ABC-type molybdate transport system, periplasm	2E-27	COG0725	ABC-type molybdate transport system, per
11497715	hypothetical protein AF0095	No	1	YES		93028	93354	-	Archaeoglobus_fulgidus_DSM_4304_uid57717	NC_000917	chromosome										
11497716	hypothetical protein AF0096	Yes	0	YES		93365	93679	-	Archaeoglobus_fulgidus_DSM_4304_uid57717	NC_000917	chromosome		arCOG07521	6		S		Uncharacterized conserved protein			
11497717	hypothetical protein AF0097	No	2	YES		93981	94277	-	Archaeoglobus_fulgidus_DSM_4304_uid57717	NC_000917	chromosome										
11497718	hypothetical protein AF0098	No	4	YES		94327	94650	-	Archaeoglobus_fulgidus_DSM_4304_uid57717	NC_000917	chromosome										
11497719	hypothetical protein AF0099	No	0	YES		94932	95396	-	Archaeoglobus_fulgidus_DSM_4304_uid57717	NC_000917	chromosome		arCOG00719	1	(-)	V		PIN domain containing protein	3E-36	COG2405	Predicted nucleic acid-binding protein,

===										8	Archaeoglobus_fulgidus_DSM_4304_uid57717	NC_000917	chromosome										
	11497762	hypothetical protein AF0145	Yes	2			129386	129724	-		Archaeoglobus_fulgidus_DSM_4304_uid57717	NC_000917	chromosome										
	11497763	hypothetical protein AF0146	Yes	1			129935	130150	-		Archaeoglobus_fulgidus_DSM_4304_uid57717	NC_000917	chromosome										
	11497764	hypothetical protein AF0147	No	4			130213	130737	-		Archaeoglobus_fulgidus_DSM_4304_uid57717	NC_000917	chromosome		arCOG02054	1	(-)	S		Uncharacterized conserved protein	0.00001	COG2881	Uncharacterized protein conserved in arc
	11497765	hypothetical protein AF0148	No	2			130973	131230	+		Archaeoglobus_fulgidus_DSM_4304_uid57717	NC_000917	chromosome										
	11497766	hypothetical protein AF0149	No	3			131375	131629	+		Archaeoglobus_fulgidus_DSM_4304_uid57717	NC_000917	chromosome										
	11497767	hypothetical protein AF0150	No	0			132113	132382	-		Archaeoglobus_fulgidus_DSM_4304_uid57717	NC_000917	chromosome										
	11497768	hypothetical protein AF0151	No	0			132594	132896	-		Archaeoglobus_fulgidus_DSM_4304_uid57717	NC_000917	chromosome										
	11497769	copper-transporting ATPase P-type	No	8			132861	134933	+		Archaeoglobus_fulgidus_DSM_4304_uid57717	NC_000917	chromosome		arCOG01576	3	(-)	P	ZntA	Cation transport ATPase		COG2217	Cation transport ATPase
===										6	Archaeoglobus_fulgidus_DSM_4304_uid57717	NC_000917	chromosome										
	11497918	hypothetical protein AF0303	No	4	YES		276358	277551	+		Archaeoglobus_fulgidus_DSM_4304_uid57717	NC_000917	chromosome		arCOG07506	3		S		Uncharacterized conserved protein			
	11497919	hypothetical protein AF0304	No	0	YES		277582	278661	-		Archaeoglobus_fulgidus_DSM_4304_uid57717	NC_000917	chromosome										
	11497920	ISA1214-2 transposase	No	0	YES		278708	278851	-		Archaeoglobus_fulgidus_DSM_4304_uid57717	NC_000917	chromosome		arCOG03883	2		S	ISA1214	Putative transposon-encoded protein	7E-24	COG3466	Putative transposon-encoded protein
	11497921	ISA1214-2 transposase	No	0	YES		278918	279886	+		Archaeoglobus_fulgidus_DSM_4304_uid57717	NC_000917	chromosome		arCOG07762	1	(-)	L		Transposase	8E-179	COG5558	Transposase
	11497922	hypothetical protein AF0307	No	0	YES		280145	280906	-		Archaeoglobus_fulgidus_DSM_4304_uid57717	NC_000917	chromosome										
	11497923	hypothetical protein AF0308	No	0	YES		281004	281312	-		Archaeoglobus_fulgidus_DSM_4304_uid57717	NC_000917	chromosome										
===										6	Archaeoglobus_fulgidus_DSM_4304_uid57717	NC_000917	chromosome										
	11498025	hypothetical protein AF0413	No	0			376244	377077	+		Archaeoglobus_fulgidus_DSM_4304_uid57717	NC_000917	chromosome		arCOG04365	1	(-)	R		Actin superfamily ATPase	1E-61	COG4012	Uncharacterized protein conserved in arc
	11498026	hypothetical protein AF0414	No	0			377070	377504	-		Archaeoglobus_fulgidus_DSM_4304_uid57717	NC_000917	chromosome										
	11498027	hypothetical protein AF0415	No	0			377587	377793	-		Archaeoglobus_fulgidus_DSM_4304_uid57717	NC_000917	chromosome								0.00002	pfam11655	Protein of unknown function (DUF2589). T
	11498028	hypothetical protein AF0416	No	0			377790	377978	-		Archaeoglobus_fulgidus_DSM_4304_uid57717	NC_000917	chromosome										
	11498029	hypothetical protein AF0417	No	0			377992	378564	-		Archaeoglobus_fulgidus_DSM_4304_uid57717	NC_000917	chromosome								1E-22	pfam11655	Protein of unknown function (DUF2589). T
	11498030	hypothetical protein AF0418	No	0			378623	379162	-		Archaeoglobus_fulgidus_DSM_4304_uid57717	NC_000917	chromosome		arCOG01182	1	(-)	T		Predicted Ser/Thr protein kinase	9E-26	COG2112	Predicted Ser/Thr protein kinase
===										6	Archaeoglobus_fulgidus_DSM_4304_uid57717	NC_000917	chromosome										
	11498864	metal-dependent hydrolase	No	0			1128580	1129275	-		Archaeoglobus_fulgidus_DSM_4304_uid57717	NC_000917	chromosome		arCOG00497	1	(-)	R		Zn-dependent hydrolase of the beta-lactamase fo	2E-44	COG2220	Predicted Zn-dependent hydrolases of the
	11498865	hypothetical protein AF1266	No	2	YES		1129378	1129887	+		Archaeoglobus_fulgidus_DSM_4304_uid57717	NC_000917	chromosome										
	11498866	hypothetical protein AF1267	No	0	YES		1130368	1130490	+		Archaeoglobus_fulgidus_DSM_4304_uid57717	NC_000917	chromosome										
	11498867	hypothetical protein AF1268	No	5	YES		1130820	1131389	+		Archaeoglobus_fulgidus_DSM_4304_uid57717	NC_000917	chromosome										
	11498868	hypothetical protein AF1269	No	0	YES		1131912	1132019	+		Archaeoglobus_fulgidus_DSM_4304_uid57717	NC_000917	chromosome										
	11498869	ArsR family transcriptional regulator	No	0	YES		1132053	1132331	+		Archaeoglobus_fulgidus_DSM_4304_uid57717	NC_000917	chromosome		arCOG01680	1	(-)	K	ArsR	Transcriptional regulator containing HTH domain,	8E-11	COG0640	Predicted transcriptional regulators
===										5	Archaeoglobus_fulgidus_DSM_4304_uid57717	NC_000917	chromosome										
	11499057	hypothetical protein AF1462	No	4			1321849	1322400	+		Archaeoglobus_fulgidus_DSM_4304_uid57717	NC_000917	chromosome										
	11499058	fumarate reductase flavoprotein subunit	No	1			1322352	1324079	+		Archaeoglobus_fulgidus_DSM_4304_uid57717	NC_000917	chromosome		arCOG00571	1	(-)	C	SdhA	Succinate dehydrogenase/fumarate reductase, fla	3E-39	COG1053	Succinate dehydrogenase/fumarate reducta
	11499059	hypothetical protein AF1464	Yes	1			1324209	1324727	+		Archaeoglobus_fulgidus_DSM_4304_uid57717	NC_000917	chromosome		arCOG03973	2		S		Uncharacterized conserved protein	0.00001	COG1196	Chromosome segregation ATPases
	11499060	hypothetical protein AF1465	Yes	0			1324761	1325210	+		Archaeoglobus_fulgidus_DSM_4304_uid57717	NC_000917	chromosome		arCOG03973	2		S		Uncharacterized conserved protein			
	11499061	hypothetical protein AF1466	No	0			1325937	1326704	+		Archaeoglobus_fulgidus_DSM_4304_uid57717	NC_000917	chromosome										
===										14	Archaeoglobus_fulgidus_DSM_4304_uid57717	NC_000917	chromosome										
	11499164	hypothetical protein AF1569	No	6			1412510	1413289	+		Archaeoglobus_fulgidus_DSM_4304_uid57717	NC_000917	chromosome										
	11499165	hypothetical protein AF1570	Yes	0			1413286	1413909	+		Archaeoglobus_fulgidus_DSM_4304_uid57717	NC_000917	chromosome		arCOG07527	5	(-)	S		Predicted metal-binding protein			
	11499166	hypothetical protein AF1571	Yes	1			1414105	1415127	+		Archaeoglobus_fulgidus_DSM_4304_uid57717	NC_000917	chromosome										
	11499167	hypothetical protein AF1572	No	0			1415348	1415755	+		Archaeoglobus_fulgidus_DSM_4304_uid57717	NC_000917	chromosome										
	11499168	hypothetical protein AF1573	No	1			1415956	1416351	+		Archaeoglobus_fulgidus_DSM_4304_uid57717	NC_000917	chromosome		arCOG07521	6		S		Uncharacterized conserved protein			
	11499169	hypothetical protein AF1574	Yes	1			1416429	1417631	+		Archaeoglobus_fulgidus_DSM_4304_uid57717	NC_000917	chromosome		arCOG06135	1		S		Uncharacterized conserved protein			
	11499170	hypothetical protein AF1575	Yes	1			1417671	1418303	+		Archaeoglobus_fulgidus_DSM_4304_uid57717	NC_000917	chromosome		arCOG06136	2		S		Uncharacterized conserved protein			
	11499171	hypothetical protein AF1576	No	0			1418387	1418635	+		Archaeoglobus_fulgidus_DSM_4304_uid57717	NC_000917	chromosome										
	11499172	hypothetical protein AF1577	No	2			1418723	1419163	+		Archaeoglobus_fulgidus_DSM_4304_uid57717	NC_000917	chromosome										
	11499173	hypothetical protein AF1578	No	3			1419168	1419401	+		Archaeoglobus_fulgidus_DSM_4304_uid57717	NC_000917	chromosome										
	11499174	hypothetical protein AF1579	No	6			1419561	1420154	+		Archaeoglobus_fulgidus_DSM_4304_uid57717	NC_000917	chromosome										
	11499175	hypothetical protein AF1580	No	0			1420445	1420945	-		Archaeoglobus_fulgidus_DSM_4304_uid57717	NC_000917	chromosome		arCOG02613	1	(-)	K		Chromosome segregation and condensation pro	4E-31	pfam04079	Putative transcriptional regulators (Ypu
	11499176	hypothetical protein AF1581	No	0			1421046	1421366	+		Archaeoglobus_fulgidus_DSM_4304_uid57717	NC_000917	chromosome										
	11499177	hypothetical protein AF1582	No	2			1421806	1422009	-		Archaeoglobus_fulgidus_DSM_4304_uid57717	NC_000917	chromosome										
===										6	Archaeoglobus_fulgidus_DSM_4304_uid57717	NC_000917	chromosome										
	11499268	hypothetical protein AF1678	No	0			1507155	1507778	-		Archaeoglobus_fulgidus_DSM_4304_uid57717	NC_000917	chromosome	arCOG01842,a	arCOG01842	1	(-)	I		Putative sterol carrier protein	4E-13	COG3255	Putative sterol carrier protein
	11499269	vtpJ-therm	Yes	0			1507887	1509062	+		Archaeoglobus_fulgidus_DSM_4304_uid57717	NC_000917	chromosome								0.000009	pfam03283	Pectinacetylesterase.
	11499270	hypothetical protein AF1680	No	0	YES		1509070	1509663	-		Archaeoglobus_fulgidus_DSM_4304_uid57717	NC_000917	chromosome		arCOG09517	1		S		Uncharacterized conserved protein			
	11499271	hypothetical protein AF1681	No	0	YES		1509605	1510243	-		Archaeoglobus_fulgidus_DSM_4304_uid57717	NC_000917	chromosome		arCOG08622	1		S		Uncharacterized conserved protein			
	11499272	hypothetical protein AF1682	No	0	YES		1510405	1510644	+		Archaeoglobus_fulgidus_DSM_4304_uid57717	NC_000917	chromosome										
	11499273	hypothetical protein AF1683	No	0	YES		1510628	1511098	+		Archaeoglobus_fulgidus_DSM_4304_uid57717	NC_000917	chromosome		arCOG04502	2	(-)	V		PIN domain containing protein	2E-19	COG2402	Predicted nucleic acid-binding protein,
===										5	Archaeoglobus_fulgidus_DSM_4304_uid57717	NC_000917	chromosome										
	11499429	hypothetical protein AF1844	No	0			1655739	1656011	-		Archaeoglobus_fulgidus_DSM_4304_uid57717	NC_000917	chromosome		arCOG10356	2		S		Uncharacterized conserved protein			
	11499430	hypothetical protein AF1845	Yes	0			1655972	1656838	-		Archaeoglobus_fulgidus_DSM_4304_uid57717	NC_000917	chromosome		arCOG10356	2		S		Uncharacterized conserved protein			
	11499431	hypothetical protein AF1846	No	0			1656933	1657343	-		Archaeoglobus_fulgidus_DSM_4304_uid57717	NC_000917	chromosome		arCOG02242	2	(-)	K		Transcriptional regulator, contains HTH domain	4E-23	COG3355	Predicted transcriptional regulator
	11499432	hypothetical protein AF1847	No	0			1657537	1657788	-		Archaeoglobus_fulgidus_DSM_4304_uid57717	NC_000917	chromosome										
	11499433	hypothetical protein AF1848	No	0			1657839	1658030	-		Archaeoglobus_fulgidus_DSM_4304_uid57717	NC_000917	chromosome										
===										5	Archaeoglobus_fulgidus_DSM_4304_uid57717	NC_000917	chromosome										
	11499526	hypothetical protein AF1944	No	0			1743944	1744981	+		Archaeoglobus_fulgidus_DSM_4304_uid57717	NC_000917	chromosome										
	11499527	hypothetical protein AF1945	Yes	0			1744968	1745678	+		Archaeoglobus_fulgidus_DSM_4304_uid57717	NC_000917	chromosome										
	11499528	cysteine proteinase	Yes	0			1745815	1749081	+		Archaeoglobus_fulgidus_DSM_4304_uid57717	NC_000917	chromosome	arCOG02532,a	arCOG02532	2	(-)	S		beta-propeller repeat protein fused to CARDB-like	2E-57	COG1572	Uncharacterized conserved protein
	11499529	hypothetical protein AF1947	Yes	0			1749078	1749716	+		Archaeoglobus_fulgidus_DSM_4304_uid57717	NC_000917	chromosome								0.000001	COG3042	Putative hemolysin
	11499530	hypothetical protein AF1948	Yes	0			1749869	1752319	+		Archaeoglobus_fulgidus_DSM_4304_uid57717	NC_000917	chromosome										
===										5	Archaeoglobus_fulgidus_DSM_4304_uid57717	NC_000917	chromosome										
	11499572	hypothetical protein AF1990	Yes	0			1792318	1794426	+		Archaeoglobus_fulgidus_DSM_4304_uid57717	NC_000917	chromosome		arCOG07530	3		S		Uncharacterized conserved protein			
	11499573	hypothetical protein AF1991	No	3			1794419	1794775	+		Archaeoglobus_fulgidus_DSM_4304_uid57717	NC_000917	chromosome										
	11499574	calcium-binding protein	Yes	1			1794786	1795727	+		Archaeoglobus_fulgidus_DSM_4304_uid57717	NC_000917	chromosome		arCOG07765	3		S		Uncharacterized conserved protein			
	11499575	hypothetical protein AF1993	No	1			1795732	1796112	+		Archaeoglobus_fulgidus_DSM_4304_uid57717	NC_000917	chromosome		arCOG06145	2	(-)	S		Uncharacterized conserved protein			
	11499576	hypothetical protein AF1994	Yes	0			1796109	1796819	+		Archaeoglobus_fulgidus_DSM_4304_uid57717	NC_000917	chromosome		arCOG06146	3		S		Uncharacterized conserved protein			
===										8	Archaeoglobus_fulgidus_DSM_4304_uid57717	NC_000917	chromosome										
	11499701	inosine monophosphate dehydrogenase	No	0			1907488	1908039	+		Archaeoglobus_fulgidus_DSM_4304_uid57717	NC_000917	chromosome		arCOG00608	3	(-)	K		Predicted transcriptional regulator with C-terminal	3E-72	COG3620	Predicted transcriptional regulator with
	11499702	hypothetical protein AF2119	Yes	0			1908351	1910576	-		Archaeoglobus_fulgidus_DSM_4304_uid57717	NC_000917	chromosome										
	11499703	hypothetical protein AF2120	No	1			1910695	1911147	-		Archaeoglobus_fulgidus_DSM_4304_uid57717	NC_000917	chromosome										
	11499704	hypothetical protein AF2121	No	0			1911154	1911624	-		Archaeoglobus_fulgidus_DSM_4304_uid57717	NC_000917	chromosome										
	11499705	hypothetical protein AF2122	No	2			1911634	1912062	-		Archaeoglobus_fulgidus_DSM_4304_uid57717	NC_000917	chromosome										
	11499706	hypothetical protein AF2123	No	0			1912067	1912498	-		Archaeoglobus_fulgidus_DSM_4304_uid57717	NC_000917	chromosome										
	11499707	hypothetical protein AF2124	Yes	2			1912608	1913036	-		Archaeoglobus_fulgidus_DSM_4304_uid57717	NC_000917	chromosome										
	11499708	hypothetical protein AF2125	No	7			1913033	1914010	-		Archaeoglobus_fulgidus_DSM_4304_uid57717	NC_000917	chromosome		arCOG02050	1	(-)	R		Predicted permease	4E-14	COG0730	Predicted permeases
===										6	Archaeoglobus_fulgidus_DSM_4304_uid57717	NC_000917	chromosome										
	11499713	hypothetical protein AF2130	No	3	YES		1918026	1918325	-		Archaeoglobus_fulgidus_DSM_4304_uid57717	NC_000917	chromosome										
	11499714	hypothetical protein AF2131	No	9	YES		1918699	1919688	-		Archaeoglobus_fulgidus_DSM_4304_uid57717	NC_000917	chromosome										
	11499715	hypothetical protein AF2132	No	6	YES		1919960	1920694	-		Archaeoglobus_fulgidus_DSM_4304_uid57717	NC_000917	chromosome		arCOG07090	2	(-)	S		Uncharacterized conserved protein			
	11499716	hypothetical protein AF2133	No	6	YES		1920752	1921612	-		Archaeoglobus_fulgidus_DSM_4304_uid57717	NC_000917	chromosome										
	11499717	hypothetical protein AF2134	Yes	0	YES		1921687	1921977	+		Archaeoglobus_fulgidus_DSM_4304_uid57717	NC_000917	chromosome										
	11499718	hypothetical protein AF2135	No	0	YES		1922033	1922416	+		Archaeoglobus_fulgidus_DSM_4304_uid57717	NC_000917	chromosome										
===										6	Archaeoglobus_fulgidus_DSM_4304_uid57717	NC_000917	chromosome										
	11499746	hypothetical protein AF2163	No	1	YES		1939259	1939837	-		Archaeoglobus_fulgidus_DSM_4304_uid57717	NC_000917	chromosome		arCOG05285	2	(-)	S		Uncharacterized conserved protein	2E-48	COG5402	Uncharacterized conserved protein
	11499747	hypothetical protein AF2164	No	4	YES		1939992	1940390	-		Archaeoglobus_fulgidus_DSM_4304_uid57717	NC_000917	chromosome										
	11499748	hypothetical protein AF2165	No	0	YES		1940397	1941140	-		Archaeoglobus_fulgidus_DSM_4304_uid57717	NC_000917	chromosome										
	11499749	hypothetical protein AF2166	No	4	YES		1941053	1941517	-		Archaeoglobus_fulgidus_DSM_4304_uid57717	NC_000917	chromosome										
	11499750	hypothetical protein AF2167	No	0	YES		1941522	1942463	-		Archaeoglobus_fulgidus_DSM_4304_uid57717	NC_000917	chromosome										
	11499751	hypothetical protein AF2168	No	0	YES		1942700	1943074	-		Archaeoglobus_fulgidus_DSM_4304_uid57717	NC_000917	chromosome		arCOG02107	1	(-)	V		Minimal nucleotidyltransferase			
===										6	Archaeoglobus_fulgidus_DSM_4304_uid57717	NC_000917	chromosome										
	11499928	hypothetical protein AF2351	No	0	YES		2099926	2100345	+		Archaeoglobus_fulgidus_DSM_4304_uid57717	NC_000917	chromosome		arCOG04492	1	(-)	S		Uncharacterized conserved protein	5E-62	COG2028	Uncharacterized conserved protein
	11499929	NADH dehydrogenase, subunit 1	No	2	YES		2100342	2100617	+		Archaeoglobus_fulgidus_DSM_4304_uid57717	NC_000917	chromosome										
	11499930	hypothetical protein AF2353	No	0	YES		2101053	2101358	+		Archaeoglobus_fulgidus_DSM_4304_uid57717	NC_000917	chromosome										
	11499931	hypothetical protein AF2354	Yes	0	YES		2101462	2101749	+		Archaeoglobus_fulgidus_DSM_4304_uid57717	NC_000917	chromosome										
	11499932	hypothetical protein AF2355	No	0	YES		2102052	2102273	+		Archaeoglobus_fulgidus_DSM_4304_uid57717	NC_000917	chromosome										
	11499933	hypothetical protein AF2356	No	0	YES		2102386	2102829	+		Archaeoglobus_fulgidus_DSM_4304_uid57717	NC_000917	chromosome		arCOG02727	1	(-)	R		Predicted RNA-binding protein containing PUA-d	9E-59	COG1673	Uncharacterized protein conserved in arc
===										13	Archaeoglobus_profundus_DSM_5631_uid43493	NC_013741	chromosome										
	284161235	hypothetical protein Arcpr_0113	No	0			98551	98805	-		Archaeoglobus_profundus_DSM_5631_uid43493	NC_013741	chromosome		arCOG11129	1		S		Uncharacterized conserved protein			
	284161236	hypothetical protein Arcpr_0114	No	0			99411	99977	-		Archaeoglobus_profundus_DSM_5631_uid43493	NC_013741	chromosome										
	284161237	hypothetical protein Arcpr_0115	No	0			99941	100669	-		Archaeoglobus_profundus_DSM_5631_uid43493	NC_013741	chromosome										
	284161238	radical SAM protein	No	0			100670	101986	-		Archaeoglobus_profundus_DSM_5631_uid43493	NC_013741	chromosome		arCOG00938	12	(-)	R		Radical SAM superfamily enzyme			
	284161239	hypothetical protein Arcpr_0117	No	0	YES		101983	102204	-		Archaeoglobus_profundus_DSM_5631_uid43493	NC_013741	chromosome										
	284161240	hypothetical protein Arcpr_0118	No	0	YES		102209	102400	-		Archaeoglobus_profundus_DSM_5631_uid43493	NC_013741	chromosome	clust_464									
	284161241	hypothetical protein Arcpr_0119	No	0	YES		102410	102769	-		Archaeoglobus_profundus_DSM_5631_uid43493	NC_013741	chromosome	clust_298									
	284161242	hypothetical protein Arcpr_0120	No	0	YES		102759	103199	-		Archaeoglobus_profundus_DSM_5631_uid43493	NC_013741	chromosome		arCOG10366	7		L		PD-(D/E)XK superfamily nuclease			
	284161243	hypothetical protein Arcpr_0121	No	0	YES		103196	104293	-		Archaeoglobus_profundus_DSM_5631_uid43493	NC_013741	chromosome										
	284161244	UvrD/REP helicase	No	0	YES		104268	105893	-		Archaeoglobus_profundus_DSM_5631_uid43493	NC_013741	chromosome	arCOG00797	arCOG00797	3	(-)	L	UvrD	Superfamily I DNA and RNA helicase	3E-12	COG0210	Superfamily I DNA and RNA helicases
	284161245	hypothetical protein Arcpr_0123	No	0	YES		105844	106881	-		Archaeoglobus_profundus_DSM_5631_uid43493	NC_013741	chromosome										
	284161246	hypothetical protein Arcpr_0124	No	0	YES		106883	107089	-		Archaeoglobus_profundus_DSM_5631_uid43493	NC_013741	chromosome										
	284161247	hypothetical protein Arcpr_0125	No	0	YES		107092	107298	-		Archaeoglobus_profundus_DSM_5631_uid43493	NC_013741	chromosome										
===										8	Archaeoglobus_profundus_DSM_5631_uid43493	NC_013741	chromosome										
	284161567	GntR family transcriptional regulator	No	0			384124	384798	+		Archaeoglobus_profundus_DSM_5631_uid43493	NC_013741	chromosome										
	284161568	hypothetical protein Arcpr_0452	No	0			384855	385826	+		Archaeoglobus_profundus_DSM_5631_uid43493	NC_013741	chromosome		arCOG01585	1	(-)	P	Lrp	Transcriptional regulator (Lrp/AsnC family)			
	284161569	hypothetical protein Arcpr_0453	No	0			386191	386622	-		Archaeoglobus_profundus_DSM_5631_uid43493	NC_013741	chromosome								0.000002	COG0419	ATPase involved in DNA repair
	284161570	hypothetical protein Arcpr_0454	No	0			386763	387287	-		Archaeoglobus_profundus_DSM_5631_uid43493	NC_013741	chromosome										
	284161571	hypothetical protein Arcpr_0455	No	0			387456	387848	+		Archaeoglobus_profundus_DSM_5631_uid43493	NC_013741	chromosome										
	284161572	hypothetical protein Arcpr_0456	No	0			387875	388384	+		Archaeoglobus_profundus_DSM_5631_uid43493	NC_013741	chromosome										
	284161573	hypothetical protein Arcpr_0457	No	0			388369	388704	+		Archaeoglobus_profundus_DSM_5631_uid43493	NC_013741	chromosome										
	284161574	hypothetical protein Arcpr_0458	No	0			388729	388986	-		Archaeoglobus_profundus_DSM_5631_uid43493	NC_013741	chromosome		arCOG04362	5	(-)	K		Predicted transcriptional regulator			
===										12	Archaeoglobus_profundus_DSM_5631_uid43493	NC_013741	chromosome										
	284161724	hypothetical protein Arcpr_0609	No	0			516940	517233	-		Archaeoglobus_profundus_DSM_5631_uid43493	NC_013741	chromosome										
	284161725	hypothetical protein Arcpr_0610	No	0			517220	517768	-		Archaeoglobus_profundus_DSM_5631_uid43493	NC_013741	chromosome		arCOG01139	1	(-)	R		Predicted metal-dependent protease of the PAD1/JAB1 superfamily			
	284161726	hypothetical protein Arcpr_0611	No	0			517769	518533	-		Archaeoglobus_profundus_DSM_5631_uid43493	NC_013741	chromosome										
	284161727	hypothetical protein Arcpr_0612	No	0			518546	518728	-		Archaeoglobus_profundus_DSM_5631_uid43493	NC_013741	chromosome										
	284161728	hypothetical protein Arcpr_0613	No	1			518746	518886	-		Archaeoglobus_profundus_DSM_5631_uid43493	NC_013741	chromosome										
	284161729	hypothetical protein Arcpr_0614	No	0			518892	520079	-		Archaeoglobus_profundus_DSM_5631_uid43493	NC_013741	chromosome										
	284161730	hypothetical protein Arcpr_0615	Yes	2			520085	520867	-		Archaeoglobus_profundus_DSM_5631_uid43493	NC_013741	chromosome										
	284161731	hypothetical protein Arcpr_0616	No	0			521014	521319	-		Archaeoglobus_profundus_DSM_5631_uid43493	NC_013741	chromosome										
	284161732	hypothetical protein Arcpr_0617	No	0			521359	521553	-		Archaeoglobus_profundus_DSM_5631_uid43493	NC_013741	chromosome										
	284161733	hypothetical protein Arcpr_0618	No	0			521807	522223	+		Archaeoglobus_profundus_DSM_5631_uid43493	NC_013741	chromosome										
	284161734	hypothetical protein Arcpr_0619	No	0			522227	523279	+		Archaeoglobus_profundus_DSM_5631_uid43493	NC_013741	chromosome										
	284161735	hypothetical protein Arcpr_0620	No	0			523401	523601	-		Archaeoglobus_profundus_DSM_5631_uid43493	NC_013741	chromosome		arCOG03748	2	(-)	K	MarR	Transcriptional regulator, MarR family			
===										32	Archaeoglobus_profundus_DSM_5631_uid43493	NC_013741	chromosome										
	284161942	hypothetical protein Arcpr_0829	No	0			714541	714744	+		Archaeoglobus_profundus_DSM_5631_uid43493	NC_013741	chromosome										
	284161943	hypothetical protein Arcpr_0830	No	0			715078	715272	+		Archaeoglobus_profundus_DSM_5631_uid43493	NC_013741	chromosome										
	284161944	hypothetical protein Arcpr_0831	No	0			715259	715777	+		Archaeoglobus_profundus_DSM_5631_uid43493	NC_013741	chromosome								0.000005	COG1196	Chromosome segregation ATPases
	284161945	MCM family protein	No	0			715764	717746	+		Archaeoglobus_profundus_DSM_5631_uid43493	NC_013741	chromosome		arCOG00439	14	(-)	L	MCM2	Predicted ATPase involved in replication control,	2E-163	COG1241	Predicted ATPase involved in replication
	284161946	hypothetical protein Arcpr_0833	No	0	YES		717747	718151	+		Archaeoglobus_profundus_DSM_5631_uid43493	NC_013741	chromosome										
	284161947	hypothetical protein Arcpr_0834	No	0	YES		718165	718716	+		Archaeoglobus_profundus_DSM_5631_uid43493	NC_013741	chromosome	clust_190									
	284161948	hypothetical protein Arcpr_0835	No	0	YES		718717	718902	+		Archaeoglobus_profundus_DSM_5631_uid43493	NC_013741	chromosome										
	284161949	hypothetical protein Arcpr_0836	No	0	YES		718902	718997	+		Archaeoglobus_profundus_DSM_5631_uid43493	NC_013741	chromosome										
	284161950	hypothetical protein Arcpr_0837	No	0	YES		718984	719373	+		Archaeoglobus_profundus_DSM_5631_uid43493	NC_013741	chromosome										
	284161951	hypothetical protein Arcpr_0838	No	0	YES		719527	719721	+		Archaeoglobus_profundus_DSM_5631_uid43493	NC_013741	chromosome										
	284161952	hypothetical protein Arcpr_0839	No	0	YES		719718	720038	+		Archaeoglobus_profundus_DSM_5631_uid43493	NC_013741	chromosome		arCOG10970	3		S		Uncharacterized conserved protein			
	284161953	hypothetical protein Arcpr_0840	No	0	YES		720123	720482	+		Archaeoglobus_profundus_DSM_5631_uid43493	NC_013741	chromosome										
	284161954	hypothetical protein Arcpr_0841	No	0	YES		720668	720973	+		Archaeoglobus_profundus_DSM_5631_uid43493	NC_013741	chromosome										
	284161955	hypothetical protein Arcpr_0842	No	0	YES		720957	721256	+		Archaeoglobus_profundus_DSM_5631_uid43493	NC_013741	chromosome										
	284161956	resolvase	No	0	YES		721268	721885	+		Archaeoglobus_profundus_DSM_5631_uid43493	NC_013741	chromosome		arCOG03162	12	(-)	L	PinR	Site-specific recombinase, DNA invertase Pin ho	1E-23	COG1961	Site-specific recombinases, DNA invertas
	284161957	hypothetical protein Arcpr_0844	No	0	YES		721882	722145	+		Archaeoglobus_profundus_DSM_5631_uid43493	NC_013741	chromosome										
	284161958	hypothetical protein Arcpr_0845	No	0	YES		722148	722300	+		Archaeoglobus_profundus_DSM_5631_uid43493	NC_013741	chromosome										
	284161959	hypothetical protein Arcpr_0846	Yes	1	YES		722747	723415	+		Archaeoglobus_profundus_DSM_5631_uid43493	NC_013741	chromosome										
	284161960	LamG domain protein jellyroll fold domain prot	Yes	1	YES		723412	725160	+		Archaeoglobus_profundus_DSM_5631_uid43493	NC_013741	chromosome		arCOG07813	7	(-)	G		Glycosyl hydrolases family 43	3E-12	pfam13385	Concanavalin A-like lectin/glucanases su
	284161961	hypothetical protein Arcpr_0848	Yes	1	YES		725168	726439	+		Archaeoglobus_profundus_DSM_5631_uid43493	NC_013741	chromosome										
	284161962	peptidase S8/S53 subtilisin kexin sedolisin	Yes	0			726453	728087	+		Archaeoglobus_profundus_DSM_5631_uid43493	NC_013741	chromosome		arCOG00702	6	(-)	O	AprE	Subtilisin-like serine protease	5E-32	COG1404	Subtilisin-like serine proteases
	284161963	hypothetical protein Arcpr_0850	No	3	YES		728088	728396	+		Archaeoglobus_profundus_DSM_5631_uid43493	NC_013741	chromosome										
	284161964	hypothetical protein Arcpr_0851	No	0	YES		728393	728653	+		Archaeoglobus_profundus_DSM_5631_uid43493	NC_013741	chromosome	clust_386									
	284161965	hypothetical protein Arcpr_0852	No	1	YES		728650	728799	+		Archaeoglobus_profundus_DSM_5631_uid43493	NC_013741	chromosome										
	284161966	hypothetical protein Arcpr_0853	No	4	YES		728789	729142	+		Archaeoglobus_profundus_DSM_5631_uid43493	NC_013741	chromosome										
	284161967	hypothetical protein Arcpr_0854	No	8	YES		729142	729876	+		Archaeoglobus_profundus_DSM_5631_uid43493	NC_013741	chromosome										
	284161968	hypothetical protein Arcpr_0855	Yes	0	YES		729863	731254	+		Archaeoglobus_profundus_DSM_5631_uid43493	NC_013741	chromosome										
	284161969	hypothetical protein Arcpr_0856	No	3	YES		731265	731573	+		Archaeoglobus_profundus_DSM_5631_uid43493	NC_013741	chromosome										
	284161970	hypothetical protein Arcpr_0857	No	2	YES		731575	732540	+		Archaeoglobus_profundus_DSM_5631_uid43493	NC_013741	chromosome										
	284161971	hypothetical protein Arcpr_0858	No	0	YES		732537	732662	+		Archaeoglobus_profundus_DSM_5631_uid43493	NC_013741	chromosome										
	284161972	hypothetical protein Arcpr_0859	No	0	YES		732797	733348	+		Archaeoglobus_profundus_DSM_5631_uid43493	NC_013741	chromosome										
	284161973	hypothetical protein Arcpr_0860	No	0	YES		733354	734595	+		Archaeoglobus_profundus_DSM_5631_uid43493	NC_013741	chromosome		arCOG00280	21	(-)	L		HerA helicase			
===										24	Archaeoglobus_profundus_DSM_5631_uid43493	NC_013741	chromosome										
	284162001	hypothetical protein Arcpr_0889	No	0			753828	753983	-		Archaeoglobus_profundus_DSM_5631_uid43493	NC_013741	chromosome										
	284162002	hypothetical protein Arcpr_0891	No	3			754335	754706	-		Archaeoglobus_profundus_DSM_5631_uid43493	NC_013741	chromosome										
	284162003	LamG domain protein jellyroll fold domain prot	Yes	3			754703	756856	-		Archaeoglobus_profundus_DSM_5631_uid43493	NC_013741	chromosome		arCOG07813	7	(-)	G		Glycosyl hydrolases family 43	8E-14	pfam13385	Concanavalin A-like lectin/glucanases su
	284162004	hypothetical protein Arcpr_0893	No	2			756853	757149	-		Archaeoglobus_profundus_DSM_5631_uid43493	NC_013741	chromosome										
	284162005	hypothetical protein Arcpr_0894	No	0			757103	759196	-		Archaeoglobus_profundus_DSM_5631_uid43493	NC_013741	chromosome								0.0000001	pfam06682	Protein of unknown function (DUF1183). T
	284162006	hypothetical protein Arcpr_0895	No	0			759542	760483	-		Archaeoglobus_profundus_DSM_5631_uid43493	NC_013741	chromosome		arCOG00188	1	(-)	P	CbiO	ABC-type cobalt transport system, ATPase component			
	284162007	hypothetical protein Arcpr_0896	No	0			760473	760826	-		Archaeoglobus_profundus_DSM_5631_uid43493	NC_013741	chromosome										
	284162008	hypothetical protein Arcpr_0897	No	3			760837	761655	-		Archaeoglobus_profundus_DSM_5631_uid43493	NC_013741	chromosome										
	284162009	hypothetical protein Arcpr_0898	No	0			761780	762259	-		Archaeoglobus_profundus_DSM_5631_uid43493	NC_013741	chromosome										
	284162010	hypothetical protein Arcpr_0899	No	3			762240	762578	-		Archaeoglobus_profundus_DSM_5631_uid43493	NC_013741	chromosome										
	284162011	hypothetical protein Arcpr_0900	No	3			762649	763080	-		Archaeoglobus_profundus_DSM_5631_uid43493	NC_013741	chromosome										
	284162012	hypothetical protein Arcpr_0901	No	0			763386	763673	-		Archaeoglobus_profundus_DSM_5631_uid43493	NC_013741	chromosome										
	284162013	integrase family protein	No	0			763627	764649	-		Archaeoglobus_profundus_DSM_5631_uid43493	NC_013741	chromosome		arCOG08624	3	(-)	L		Phage integrase family protein			
	284162014	hypothetical protein Arcpr_0903	No	0			764655	765110	-		Archaeoglobus_profundus_DSM_5631_uid43493	NC_013741	chromosome										
	284162015	hypothetical protein Arcpr_0904	No	0			765168	765479	-		Archaeoglobus_profundus_DSM_5631_uid43493	NC_013741	chromosome										
	284162016	hypothetical protein Arcpr_0905	No	0			765479	765838	-		Archaeoglobus_profundus_DSM_5631_uid43493	NC_013741	chromosome										
	284162017	hypothetical protein Arcpr_0906	No	0			765828	766013	-		Archaeoglobus_profundus_DSM_5631_uid43493	NC_013741	chromosome										
	284162018	hypothetical protein Arcpr_0907	No	0			766277	766498	-		Archaeoglobus_profundus_DSM_5631_uid43493	NC_013741	chromosome										
	284162019	hypothetical protein Arcpr_0908	No	0			766505	767065	-		Archaeoglobus_profundus_DSM_5631_uid43493	NC_013741	chromosome		arCOG07102	9	(-)	L		PD-(D/E)XK superfamily nuclease			
	284162020	hypothetical protein Arcpr_0909	No	0			767062	767250	-		Archaeoglobus_profundus_DSM_5631_uid43493	NC_013741	chromosome	clust_467									
	284162021	hypothetical protein Arcpr_0910	No	0			767264	767500	-		Archaeoglobus_profundus_DSM_5631_uid43493	NC_013741	chromosome										
	284162022	hypothetical protein Arcpr_0911	No	0			767493	767798	-		Archaeoglobus_profundus_DSM_5631_uid43493	NC_013741	chromosome										
	284162023	hypothetical protein Arcpr_0912	No	0			767795	768580	-		Archaeoglobus_profundus_DSM_5631_uid43493	NC_013741	chromosome		arCOG01290	5	(-)	L	SplB	DNA repair photolyase			
	284162024	hypothetical protein Arcpr_0913	No	0			768586	768792	-		Archaeoglobus_profundus_DSM_5631_uid43493	NC_013741	chromosome										
===										7	Archaeoglobus_profundus_DSM_5631_uid43493	NC_013741	chromosome										
	284162054	hypothetical protein Arcpr_0943	No	0			793937	794293	+		Archaeoglobus_profundus_DSM_5631_uid43493	NC_013741	chromosome		arCOG08624	3	(-)	L		Phage integrase family protein			
	284162055	hypothetical protein Arcpr_0944	No	0			794288	794548	-		Archaeoglobus_profundus_DSM_5631_uid43493	NC_013741	chromosome								0.000002	COG1196	Chromosome segregation ATPases
	284162056	hypothetical protein Arcpr_0945	No	0			794793	795110	-		Archaeoglobus_profundus_DSM_5631_uid43493	NC_013741	chromosome								0.00009	COG0419	ATPase involved in DNA repair
	284162057	hypothetical protein Arcpr_0946	No	2			795246	795512	+		Archaeoglobus_profundus_DSM_5631_uid43493	NC_013741	chromosome		arCOG11131	4		S		Uncharacterized conserved protein			
	284162058	Bifunctional DNA primase/polymerase	No	0			795629	796558	-		Archaeoglobus_profundus_DSM_5631_uid43493	NC_013741	chromosome								0.00002	pfam09250	Bifunctional DNA primase/polymerase, N-t
	284162059	hypothetical protein Arcpr_0948	No	0			796583	796777	-		Archaeoglobus_profundus_DSM_5631_uid43493	NC_013741	chromosome	clust_442									
	284162060	hypothetical protein Arcpr_0949	No	0			796860	797441	+		Archaeoglobus_profundus_DSM_5631_uid43493	NC_013741	chromosome		arCOG00394	7	(-)	K	ArsR	Transcriptional regulator containing HTH domain, ArsR family			
===										9	Archaeoglobus_profundus_DSM_5631_uid43493	NC_013741	chromosome										
	284162233	hypothetical protein Arcpr_1125	No	2			959141	959425	+		Archaeoglobus_profundus_DSM_5631_uid43493	NC_013741	chromosome										
	284162234	hypothetical protein Arcpr_1126	No	0			959454	959699	-		Archaeoglobus_profundus_DSM_5631_uid43493	NC_013741	chromosome		arCOG10220	1		S		Uncharacterized conserved protein			
	284162235	hypothetical protein Arcpr_1127	No	0			959710	961713	-		Archaeoglobus_profundus_DSM_5631_uid43493	NC_013741	chromosome		arCOG02163	1	(-)	O		Predicted ATP-dependent Lon-type protease	0.00002	pfam13337	Putative ATP-dependent Lon protease. Thi
	284162236	hypothetical protein Arcpr_1128	No	0			961719	962129	-		Archaeoglobus_profundus_DSM_5631_uid43493	NC_013741	chromosome										
	284162237	hypothetical protein Arcpr_1129	No	0			962117	962641	-		Archaeoglobus_profundus_DSM_5631_uid43493	NC_013741	chromosome										
	284162238	CopG domain protein DNA-binding domain pro	No	0			962669	962884	-		Archaeoglobus_profundus_DSM_5631_uid43493	NC_013741	chromosome										
	284162239	hypothetical protein Arcpr_1131	No	0			962972	963643	+		Archaeoglobus_profundus_DSM_5631_uid43493	NC_013741	chromosome										
	284162240	hypothetical protein Arcpr_1132	No	0			964083	964217	-		Archaeoglobus_profundus_DSM_5631_uid43493	NC_013741	chromosome										
	284162241	signal transduction protein	No	12			965133	967202	-		Archaeoglobus_profundus_DSM_5631_uid43493	NC_013741	chromosome		arCOG00600	2	(-)	R		CBS domain	5E-16	pfam07690	Major Facilitator Superfamily. ; FOG: CBS domain
===										6	Archaeoglobus_profundus_DSM_5631_uid43493	NC_013741	chromosome										
	284162248	transposase, IS605 OrfB family	No	0	YES		973180	974226	-		Archaeoglobus_profundus_DSM_5631_uid43493	NC_013741	chromosome		arCOG04197	4	(-)	R		Transposase related protein			
	284162249	hypothetical protein Arcpr_1143	No	0	YES		974193	974345	-		Archaeoglobus_profundus_DSM_5631_uid43493	NC_013741	chromosome										
	284162250	hypothetical protein Arcpr_1144	No	0	YES		975239	975364	-		Archaeoglobus_profundus_DSM_5631_uid43493	NC_013741	chromosome										
	284162251	hypothetical protein Arcpr_1145	No	0	YES		975361	975642	-		Archaeoglobus_profundus_DSM_5631_uid43493	NC_013741	chromosome										
	284162252	hypothetical protein Arcpr_1146	No	0	YES		975817	976140	+		Archaeoglobus_profundus_DSM_5631_uid43493	NC_013741	chromosome										
	284162253	hypothetical protein Arcpr_1147	No	0	YES		976455	976784	-		Archaeoglobus_profundus_DSM_5631_uid43493	NC_013741	chromosome		arCOG02219	4	(-)	V	VapC	PIN domain containing protein			
===										7	Archaeoglobus_profundus_DSM_5631_uid43493	NC_013741	chromosome										
	284162262	hypothetical protein Arcpr_1157	No	0			982491	982793	+		Archaeoglobus_profundus_DSM_5631_uid43493	NC_013741	chromosome										
	284162263	UBA/THIF-type NAD/FAD binding protein	No	1			982771	983397	+		Archaeoglobus_profundus_DSM_5631_uid43493	NC_013741	chromosome		arCOG01676	1	(-)	H	ThiF	Dinucleotide-utilizing enzyme involved in molybdopterin and thiamine biosynthesis			
	284162264	hypothetical protein Arcpr_1159	No	0	YES		983394	984047	+		Archaeoglobus_profundus_DSM_5631_uid43493	NC_013741	chromosome										
	284162265	hypothetical protein Arcpr_1160	No	0	YES		984187	984657	+		Archaeoglobus_profundus_DSM_5631_uid43493	NC_013741	chromosome										
	284162266	hypothetical protein Arcpr_1161	No	0	YES		984983	985693	+		Archaeoglobus_profundus_DSM_5631_uid43493	NC_013741	chromosome		arCOG10386	1		S		Uncharacterized conserved protein			
	284162267	hypothetical protein Arcpr_1162	No	0	YES		985823	987046	-		Archaeoglobus_profundus_DSM_5631_uid43493	NC_013741	chromosome		arCOG03166	9	(-)	R		AAA+ superfamily ATPase fused to HTH and RecB nuclease domains			
	284162268	hypothetical protein Arcpr_1163	No	0	YES		987258	987467	+		Archaeoglobus_profundus_DSM_5631_uid43493	NC_013741	chromosome										
===										6	Archaeoglobus_profundus_DSM_5631_uid43493	NC_013741	chromosome										
	284162272	hypothetical protein Arcpr_1168	No	0	YES		993881	994498	+		Archaeoglobus_profundus_DSM_5631_uid43493	NC_013741	chromosome								0.00003	COG0419	ATPase involved in DNA repair
	284162273	hypothetical protein Arcpr_1169	Yes	0	YES		994541	995074	-		Archaeoglobus_profundus_DSM_5631_uid43493	NC_013741	chromosome										
	284162274	hypothetical protein Arcpr_1170	No	0	YES		995214	995510	+		Archaeoglobus_profundus_DSM_5631_uid43493	NC_013741	chromosome		arCOG04629	1	(-)	V		Predicted antitoxins containing the HTH domain	0.000000004	pfam03683	Uncharacterized protein family (UPF0175)
	284162275	hypothetical protein Arcpr_1171	No	0	YES		995585	995800	+		Archaeoglobus_profundus_DSM_5631_uid43493	NC_013741	chromosome										
	284162276	hypothetical protein Arcpr_1173	No	0	YES		996237	996530	+		Archaeoglobus_profundus_DSM_5631_uid43493	NC_013741	chromosome								0.00000004	pfam03683	Uncharacterized protein family (UPF0175)
	284162277	hypothetical protein Arcpr_1174	No	0	YES		996527	996865	+		Archaeoglobus_profundus_DSM_5631_uid43493	NC_013741	chromosome										
===										6	Archaeoglobus_profundus_DSM_5631_uid43493	NC_013741	chromosome										
	284162309	glycosyl transferase family 2	No	0			1023588	1027082	+		Archaeoglobus_profundus_DSM_5631_uid43493	NC_013741	chromosome	arCOG01384,a	arCOG01384	1	(-)	M	WcaA	Glycosyltransferase	4E-24	pfam00535	Glycosyl transferase family 2. Diverse f ; Glycosyl transferase family 2. Diverse f ; Chromosome segregation ATPases
	284162310	hypothetical protein Arcpr_1208	No	10			1027092	1029647	+		Archaeoglobus_profundus_DSM_5631_uid43493	NC_013741	chromosome										
	284162311	hypothetical protein Arcpr_1209	No	0			1029631	1029771	+		Archaeoglobus_profundus_DSM_5631_uid43493	NC_013741	chromosome										
	284162312	hypothetical protein Arcpr_1210	No	3			1030066	1030506	-		Archaeoglobus_profundus_DSM_5631_uid43493	NC_013741	chromosome										
	284162313	hypothetical protein Arcpr_1211	No	1			1030905	1031135	-		Archaeoglobus_profundus_DSM_5631_uid43493	NC_013741	chromosome										
	284162314	methyltransferase type 11	No	0			1031163	1031771	-		Archaeoglobus_profundus_DSM_5631_uid43493	NC_013741	chromosome		arCOG04347	4	(-)	Q		SAM-dependent methyltransferase	2E-10	pfam13847	Methyltransferase domain. This family ap
===										48	Archaeoglobus_profundus_DSM_5631_uid43493	NC_013741	chromosome										
	284162578	hypothetical protein Arcpr_1481	No	0		Virus_Caudovi	1267585	1267938	-		Archaeoglobus_profundus_DSM_5631_uid43493	NC_013741	chromosome										
	284162579	hypothetical protein Arcpr_1482	No	0		Virus_Caudovi	1267975	1268505	-		Archaeoglobus_profundus_DSM_5631_uid43493	NC_013741	chromosome		arCOG11245	4		S		Uncharacterized conserved protein			
	284162580	hypothetical protein Arcpr_1483	No	0		Virus_Caudovi	1268502	1269371	-		Archaeoglobus_profundus_DSM_5631_uid43493	NC_013741	chromosome		arCOG10308	5	(-)	S		Uncharacterized conserved protein			
	284162581	hypothetical protein Arcpr_1484	No	0		Virus_Caudovi	1269364	1269651	-		Archaeoglobus_profundus_DSM_5631_uid43493	NC_013741	chromosome	clust_361									
	284162582	hypothetical protein Arcpr_1485	No	0		Virus_Caudovi	1269652	1270197	-		Archaeoglobus_profundus_DSM_5631_uid43493	NC_013741	chromosome										
	284162583	phage tail tape measure protein, TP901 family	No	2		Virus_Caudovi	1270227	1272416	-		Archaeoglobus_profundus_DSM_5631_uid43493	NC_013741	chromosome		arCOG11109	8		Y		Phage tail tape measure protein, TP901 family	2E-16	pfam10145	Phage-related minor tail protein. Member
	284162584	hypothetical protein Arcpr_1487	Yes	1		Virus_Caudovi	1272453	1272893	+		Archaeoglobus_profundus_DSM_5631_uid43493	NC_013741	chromosome										
	284162585	hypothetical protein Arcpr_1488	No	0		Virus_Caudovi	1273107	1273406	-		Archaeoglobus_profundus_DSM_5631_uid43493	NC_013741	chromosome										
	284162586	hypothetical protein Arcpr_1489	No	0		Virus_Caudovi	1273631	1273933	-		Archaeoglobus_profundus_DSM_5631_uid43493	NC_013741	chromosome		arCOG04911	1	(-)	S		Uncharacterized conserved protein			
	284162587	hypothetical protein Arcpr_1490	No	0		Virus_Caudovi	1273920	1274381	-		Archaeoglobus_profundus_DSM_5631_uid43493	NC_013741	chromosome		arCOG01057	21	(-)	K		Predicted transcriptional regulator			
	284162588	hypothetical protein Arcpr_1491	No	0		Virus_Caudovi	1274521	1275114	+		Archaeoglobus_profundus_DSM_5631_uid43493	NC_013741	chromosome		arCOG09442	1		S		Uncharacterized conserved protein			
	284162589	hypothetical protein Arcpr_1492	No	0		Virus_Caudovi	1275137	1275544	-		Archaeoglobus_profundus_DSM_5631_uid43493	NC_013741	chromosome										
	284162590	hypothetical protein Arcpr_1493	No	0		Virus_Caudovi	1275555	1276631	-		Archaeoglobus_profundus_DSM_5631_uid43493	NC_013741	chromosome								0.00000006	pfam11863	Protein of unknown function (DUF3383). T
	284162591	hypothetical protein Arcpr_1494	No	0		Virus_Caudovi	1276698	1277216	-		Archaeoglobus_profundus_DSM_5631_uid43493	NC_013741	chromosome										
	284162592	phage virion morphogenesis protein	No	0		Virus_Caudovi	1277226	1277729	-		Archaeoglobus_profundus_DSM_5631_uid43493	NC_013741	chromosome										
	284162593	hypothetical protein Arcpr_1496	No	0		Virus_Caudovi	1277719	1278060	-		Archaeoglobus_profundus_DSM_5631_uid43493	NC_013741	chromosome										
	284162594	hypothetical protein Arcpr_1497	No	2		Virus_Caudovi	1278057	1278278	-		Archaeoglobus_profundus_DSM_5631_uid43493	NC_013741	chromosome	clust_415									
	284162595	hypothetical protein Arcpr_1498	No	0		Virus_Caudovi	1278275	1278421	-		Archaeoglobus_profundus_DSM_5631_uid43493	NC_013741	chromosome										
	284162596	hypothetical protein Arcpr_1500	No	0		Virus_Caudovi	1278987	1279253	-		Archaeoglobus_profundus_DSM_5631_uid43493	NC_013741	chromosome										
	284162597	hypothetical protein Arcpr_1501	No	1		Virus_Caudovi	1279284	1279523	-		Archaeoglobus_profundus_DSM_5631_uid43493	NC_013741	chromosome										
	284162598	hypothetical protein Arcpr_1502	No	0		Virus_Caudovi	1279533	1279922	-		Archaeoglobus_profundus_DSM_5631_uid43493	NC_013741	chromosome										
	284162599	hypothetical protein Arcpr_1503	No	0		Virus_Caudovi	1279947	1280753	-		Archaeoglobus_profundus_DSM_5631_uid43493	NC_013741	chromosome		arCOG05909	1	(-)	S		Uncharacterized protein, linocin/CFP29 homolog			
	284162600	hypothetical protein Arcpr_1504	No	0		Virus_Caudovi	1280767	1281168	-		Archaeoglobus_profundus_DSM_5631_uid43493	NC_013741	chromosome								0.00007	pfam09956	Uncharacterized conserved protein (DUF21
	284162601	phage prohead protease HK97 family	No	0		Virus_Caudovi	1281179	1282501	-		Archaeoglobus_profundus_DSM_5631_uid43493	NC_013741	chromosome								0.000005	pfam02463	RecF/RecN/SMC N terminal domain. This do
	284162602	hypothetical protein Arcpr_1506	No	0		Virus_Caudovi	1282533	1282799	-		Archaeoglobus_profundus_DSM_5631_uid43493	NC_013741	chromosome										
	284162603	phage head morphogenesis protein, SPP1 gp	No	0		Virus_Caudovi	1282796	1283992	-		Archaeoglobus_profundus_DSM_5631_uid43493	NC_013741	chromosome		arCOG01356	2	(-)	C		Radical SAM superfamily enzyme	1E-11	pfam04233	Phage Mu protein F like protein. Members
	284162604	hypothetical protein Arcpr_1508	No	0		Virus_Caudovi	1283998	1285149	-		Archaeoglobus_profundus_DSM_5631_uid43493	NC_013741	chromosome										
	284162605	hypothetical protein Arcpr_1509	No	3		Virus_Caudovi	1285199	1285549	+		Archaeoglobus_profundus_DSM_5631_uid43493	NC_013741	chromosome										
	284162606	hypothetical protein Arcpr_1510	No	0		Virus_Caudovi	1285609	1285902	-		Archaeoglobus_profundus_DSM_5631_uid43493	NC_013741	chromosome										
	284162607	hypothetical protein Arcpr_1511	No	0		Virus_Caudovi	1285975	1287282	-		Archaeoglobus_profundus_DSM_5631_uid43493	NC_013741	chromosome	clust_78	arCOG09550	17		Y	TerL	Phage terminase large subunit	0.000000002	pfam03237	Terminase-like family. This family repre
	284162608	hypothetical protein Arcpr_1512	No	0		Virus_Caudovi	1287272	1287778	-		Archaeoglobus_profundus_DSM_5631_uid43493	NC_013741	chromosome										
	284162609	hypothetical protein Arcpr_1513	No	0		Virus_Caudovi	1288148	1288399	-		Archaeoglobus_profundus_DSM_5631_uid43493	NC_013741	chromosome										
	284162610	hypothetical protein Arcpr_1514	No	0		Virus_Caudovi	1288393	1288986	-		Archaeoglobus_profundus_DSM_5631_uid43493	NC_013741	chromosome								0.0001	COG5493	Uncharacterized conserved protein contai
	284162611	hypothetical protein Arcpr_1515	No	0		Virus_Caudovi	1288998	1289159	-		Archaeoglobus_profundus_DSM_5631_uid43493	NC_013741	chromosome										
	284162612	zinc finger C2H2-type domain protein	No	0		Virus_Caudovi	1289152	1289460	-		Archaeoglobus_profundus_DSM_5631_uid43493	NC_013741	chromosome		arCOG07930	3	(-)	S		Zinc finger C2H2-type			
	284162613	hypothetical protein Arcpr_1517	No	0		Virus_Caudovi	1289484	1290392	-		Archaeoglobus_profundus_DSM_5631_uid43493	NC_013741	chromosome		arCOG09665	3		S		Uncharacterized conserved protein			
	284162614	hypothetical protein Arcpr_1518	No	0		Virus_Caudovi	1290403	1290636	-		Archaeoglobus_profundus_DSM_5631_uid43493	NC_013741	chromosome										
	284162615	hypothetical protein Arcpr_1519	No	0		Virus_Caudovi	1290656	1291033	-		Archaeoglobus_profundus_DSM_5631_uid43493	NC_013741	chromosome										
	284162616	hypothetical protein Arcpr_1520	No	0		Virus_Caudovi	1291030	1291587	-		Archaeoglobus_profundus_DSM_5631_uid43493	NC_013741	chromosome										
	284162617	hypothetical protein Arcpr_1521	No	0		Virus_Caudovi	1291584	1292285	-		Archaeoglobus_profundus_DSM_5631_uid43493	NC_013741	chromosome		arCOG03130	2	(-)	L		Predicted exonuclease	0.000003	pfam13482	RNase_H superfamily.
	284162618	hypothetical protein Arcpr_1522	No	0		Virus_Caudovi	1292291	1292455	-		Archaeoglobus_profundus_DSM_5631_uid43493	NC_013741	chromosome		arCOG10177	2	(-)	S		Uncharacterized conserved protein			
	284162619	hypothetical protein Arcpr_1523	No	0		Virus_Caudovi	1292452	1293084	-		Archaeoglobus_profundus_DSM_5631_uid43493	NC_013741	chromosome										
	284162620	hypothetical protein Arcpr_1524	No	0		Virus_Caudovi	1293108	1293395	-		Archaeoglobus_profundus_DSM_5631_uid43493	NC_013741	chromosome										
	284162621	hypothetical protein Arcpr_1525	No	0		Virus_Caudovi	1293397	1293948	-		Archaeoglobus_profundus_DSM_5631_uid43493	NC_013741	chromosome	clust_190									
	284162622	hypothetical protein Arcpr_1526	No	0		Virus_Caudovi	1293936	1294184	-		Archaeoglobus_profundus_DSM_5631_uid43493	NC_013741	chromosome										
	284162623	hypothetical protein Arcpr_1527	No	0		Virus_Caudovi	1294236	1294553	+		Archaeoglobus_profundus_DSM_5631_uid43493	NC_013741	chromosome										
	284162624	hypothetical protein Arcpr_1528	No	0		Virus_Caudovi	1294658	1294846	+		Archaeoglobus_profundus_DSM_5631_uid43493	NC_013741	chromosome										
	284162625	hypothetical protein Arcpr_1529	No	0		Virus_Caudovi	1294950	1295720	+		Archaeoglobus_profundus_DSM_5631_uid43493	NC_013741	chromosome		arCOG01911	2	(-)	O	SppA	Periplasmic serine protease (ClpP class)	2E-14	pfam01972	Serine dehydrogenase proteinase. This fa
===										14	Archaeoglobus_profundus_DSM_5631_uid43493	NC_013741	chromosome										
	284162697	hypothetical protein Arcpr_1601	No	0	YES		1365984	1366811	+		Archaeoglobus_profundus_DSM_5631_uid43493	NC_013741	chromosome		arCOG01244	9	(-)	L		Intergrase/Recombinase	1E-32	COG4342	Uncharacterized protein conserved in arc
	284162698	hypothetical protein Arcpr_1602	No	0	YES		1366914	1367375	+		Archaeoglobus_profundus_DSM_5631_uid43493	NC_013741	chromosome										
	284162699	hypothetical protein Arcpr_1603	No	0	YES		1367435	1367662	-		Archaeoglobus_profundus_DSM_5631_uid43493	NC_013741	chromosome										
	284162700	hypothetical protein Arcpr_1604	No	1	YES		1367664	1367945	-		Archaeoglobus_profundus_DSM_5631_uid43493	NC_013741	chromosome										
	284162701	hypothetical protein Arcpr_1605	No	2	YES		1368316	1368576	+		Archaeoglobus_profundus_DSM_5631_uid43493	NC_013741	chromosome		arCOG11131	4		S		Uncharacterized conserved protein			
	284162702	hypothetical protein Arcpr_1606	No	2	YES		1368604	1368846	+		Archaeoglobus_profundus_DSM_5631_uid43493	NC_013741	chromosome	clust_401									
	284162703	hypothetical protein Arcpr_1607	No	0	YES		1368849	1369022	+		Archaeoglobus_profundus_DSM_5631_uid43493	NC_013741	chromosome	clust_459									
	284162704	hypothetical protein Arcpr_1608	No	0	YES		1369019	1369213	+		Archaeoglobus_profundus_DSM_5631_uid43493	NC_013741	chromosome										
	284162705	hypothetical protein Arcpr_1609	No	0	YES		1369383	1369925	-		Archaeoglobus_profundus_DSM_5631_uid43493	NC_013741	chromosome		arCOG02611	4	(-)	K		Predicted transcriptional regulator, containd two HTH domains			
	284162706	hypothetical protein Arcpr_1610	No	0	YES		1369950	1370141	-		Archaeoglobus_profundus_DSM_5631_uid43493	NC_013741	chromosome	clust_464									
	284162707	hypothetical protein Arcpr_1611	No	0	YES		1370153	1370512	-		Archaeoglobus_profundus_DSM_5631_uid43493	NC_013741	chromosome	clust_298									
	284162708	hypothetical protein Arcpr_1612	No	0	YES		1370514	1370942	-		Archaeoglobus_profundus_DSM_5631_uid43493	NC_013741	chromosome		arCOG10366	7		L		PD-(D/E)XK superfamily nuclease			
	284162709	Bifunctional DNA primase/polymerase	No	0	YES		1370939	1374262	-		Archaeoglobus_profundus_DSM_5631_uid43493	NC_013741	chromosome		arCOG08578	4	(-)	Y		RepA plasmid replication protein	0.000001	pfam09250	Bifunctional DNA primase/polymerase, N-t ; RecF/RecN/SMC N terminal domain. This do
	284162710	hypothetical protein Arcpr_1614	No	0	YES		1374246	1374404	-		Archaeoglobus_profundus_DSM_5631_uid43493	NC_013741	chromosome										
===										21	Archaeoglobus_profundus_DSM_5631_uid43493	NC_013741	chromosome										
	284162759	hypothetical protein Arcpr_1663	No	0		integrated_ele	1417066	1417956	+		Archaeoglobus_profundus_DSM_5631_uid43493	NC_013741	chromosome		arCOG01244	9	(-)	L		Intergrase/Recombinase	4E-19	COG4342	Uncharacterized protein conserved in arc
	284162760	hypothetical protein Arcpr_1664	No	0		integrated_ele	1417962	1418168	+		Archaeoglobus_profundus_DSM_5631_uid43493	NC_013741	chromosome										
	284162761	hypothetical protein Arcpr_1665	No	1		integrated_ele	1418165	1418446	+		Archaeoglobus_profundus_DSM_5631_uid43493	NC_013741	chromosome										
	284162762	hypothetical protein Arcpr_1666	No	0		integrated_ele	1418448	1418675	+		Archaeoglobus_profundus_DSM_5631_uid43493	NC_013741	chromosome										
	284162763	hypothetical protein Arcpr_1667	No	0		integrated_ele	1418662	1418859	-		Archaeoglobus_profundus_DSM_5631_uid43493	NC_013741	chromosome	clust_459									
	284162764	hypothetical protein Arcpr_1668	No	2		integrated_ele	1419012	1419407	+		Archaeoglobus_profundus_DSM_5631_uid43493	NC_013741	chromosome		arCOG11131	4		S		Uncharacterized conserved protein			
	284162765	hypothetical protein Arcpr_1669	No	3		integrated_ele	1419415	1419735	+		Archaeoglobus_profundus_DSM_5631_uid43493	NC_013741	chromosome										
	284162766	hypothetical protein Arcpr_1670	No	1		integrated_ele	1419713	1420105	+		Archaeoglobus_profundus_DSM_5631_uid43493	NC_013741	chromosome										
	284162767	hypothetical protein Arcpr_1671	No	1		integrated_ele	1420108	1420896	+		Archaeoglobus_profundus_DSM_5631_uid43493	NC_013741	chromosome										
	284162768	hypothetical protein Arcpr_1672	No	0		integrated_ele	1420893	1421279	+		Archaeoglobus_profundus_DSM_5631_uid43493	NC_013741	chromosome										
	284162769	hypothetical protein Arcpr_1673	No	0		integrated_ele	1421269	1422243	+		Archaeoglobus_profundus_DSM_5631_uid43493	NC_013741	chromosome		arCOG07960	16	(-)	R		ATPase (AAA+ superfamily)			
	284162770	hypothetical protein Arcpr_1674	No	1		integrated_ele	1422212	1422487	+		Archaeoglobus_profundus_DSM_5631_uid43493	NC_013741	chromosome										
	284162771	hypothetical protein Arcpr_1675	No	0		integrated_ele	1422489	1422821	+		Archaeoglobus_profundus_DSM_5631_uid43493	NC_013741	chromosome										
	284162772	hypothetical protein Arcpr_1676	No	0		integrated_ele	1422824	1422976	+		Archaeoglobus_profundus_DSM_5631_uid43493	NC_013741	chromosome										
	284162773	hypothetical protein Arcpr_1677	No	0		integrated_ele	1422982	1423338	+		Archaeoglobus_profundus_DSM_5631_uid43493	NC_013741	chromosome		arCOG03881	5	(-)	S		Uncharacterized conserved protein			
	284162774	hypothetical protein Arcpr_1678	No	0		integrated_ele	1423349	1425913	+		Archaeoglobus_profundus_DSM_5631_uid43493	NC_013741	chromosome								0.0000008	pfam06682	Protein of unknown function (DUF1183). T ; Chromosome segregation ATPases
	284162775	hypothetical protein Arcpr_1679	No	2		integrated_ele	1425910	1426152	+		Archaeoglobus_profundus_DSM_5631_uid43493	NC_013741	chromosome	clust_401									
	284162776	LamG domain protein jellyroll fold domain prot	Yes	2		integrated_ele	1426154	1428346	+		Archaeoglobus_profundus_DSM_5631_uid43493	NC_013741	chromosome		arCOG07813	7	(-)	G		Glycosyl hydrolases family 43	0.000000009	pfam13385	Concanavalin A-like lectin/glucanases su
	284162777	hypothetical protein Arcpr_1681	Yes	2		integrated_ele	1428346	1428672	+		Archaeoglobus_profundus_DSM_5631_uid43493	NC_013741	chromosome										
	284162778	hypothetical protein Arcpr_1682	No	3		integrated_ele	1428714	1428950	+		Archaeoglobus_profundus_DSM_5631_uid43493	NC_013741	chromosome										
	284162779	hypothetical protein Arcpr_1683	No	0		integrated_ele	1428955	1429119	+		Archaeoglobus_profundus_DSM_5631_uid43493	NC_013741	chromosome										
===										8	Archaeoglobus_profundus_DSM_5631_uid43493	NC_013741	chromosome										
	284162782	hypothetical protein Arcpr_1686	No	0	YES	integrated_ele	1431901	1432059	-		Archaeoglobus_profundus_DSM_5631_uid43493	NC_013741	chromosome										

284162783	hypothetical protein Arcpr_1687	No	0	YES	integrated_ele	1432066	1432884	-		Archaeoglobus_profundus_DSM_5631_uid43493	NC_013741	chromosome									
284162784	MazF family transcriptional regulator	No	0	YES	integrated_ele	1432976	1433335	-		Archaeoglobus_profundus_DSM_5631_uid43493	NC_013741	chromosome		arCOG03403	5 (-)	V		PemK/MazF family toxin	0.00000002	pfam02452	PemK-like protein. PemK is a growth inhi
284162785	hypothetical protein Arcpr_1689	No	0	YES	integrated_ele	1433310	1433495	-		Archaeoglobus_profundus_DSM_5631_uid43493	NC_013741	chromosome		arCOG10167	2	V		DNA-binding protein, potential antitoxin AbrB/Maz	0.00001	pfam01954	Protein of unknown function DUF104. This
284162786	hypothetical protein Arcpr_1690	No	0	YES	integrated_ele	1433578	1434264	-		Archaeoglobus_profundus_DSM_5631_uid43493	NC_013741	chromosome									
284162787	hypothetical protein Arcpr_1691	No	0	YES	integrated_ele	1434278	1435234	-		Archaeoglobus_profundus_DSM_5631_uid43493	NC_013741	chromosome		arCOG09152	2	S		Uncharacterized conserved protein			
284162788	AN1-type Zinc finger protein	Yes	5	YES	integrated_ele	1435590	1436645	+		Archaeoglobus_profundus_DSM_5631_uid43493	NC_013741	chromosome		arCOG00607	2 (-)	R		Zn-dependent protease fused to CBS domain	0.00000002	pfam01428	AN1-like Zinc finger. Zinc finger at the
284162789	hypothetical protein Arcpr_1693	No	0	YES	integrated_ele	1436882	1437028	-		Archaeoglobus_profundus_DSM_5631_uid43493	NC_013741	chromosome									
===									13	Archaeoglobus_profundus_DSM_5631_uid43493	NC_013741	chromosome									
284162793	hypothetical protein Arcpr_1697	No	0			1439593	1439772	+		Archaeoglobus_profundus_DSM_5631_uid43493	NC_013741	chromosome									
284162794	hypothetical protein Arcpr_1698	No	0			1439765	1440385	+		Archaeoglobus_profundus_DSM_5631_uid43493	NC_013741	chromosome							9E-11	COG1196	Chromosome segregation ATPases
284162795	MCM family protein	No	0			1440382	1442349	+		Archaeoglobus_profundus_DSM_5631_uid43493	NC_013741	chromosome		arCOG00439	14 (-)	L	MCM2	Predicted ATPase involved in replication control,	1E-154	COG1241	Predicted ATPase involved in replication
284162796	hypothetical protein Arcpr_1700	No	0			1442356	1443012	+		Archaeoglobus_profundus_DSM_5631_uid43493	NC_013741	chromosome									
284162797	hypothetical protein Arcpr_1701	No	0			1443009	1443335	+		Archaeoglobus_profundus_DSM_5631_uid43493	NC_013741	chromosome									
284162798	hypothetical protein Arcpr_1702	No	0			1443354	1443638	+		Archaeoglobus_profundus_DSM_5631_uid43493	NC_013741	chromosome									
284162799	hypothetical protein Arcpr_1703	No	1			1443661	1443915	+		Archaeoglobus_profundus_DSM_5631_uid43493	NC_013741	chromosome									
284162800	hypothetical protein Arcpr_1704	No	0			1443908	1444090	+		Archaeoglobus_profundus_DSM_5631_uid43493	NC_013741	chromosome									
284162801	hypothetical protein Arcpr_1705	No	0			1444367	1444564	+		Archaeoglobus_profundus_DSM_5631_uid43493	NC_013741	chromosome									
284162802	hypothetical protein Arcpr_1706	Yes	1			1444557	1444901	+		Archaeoglobus_profundus_DSM_5631_uid43493	NC_013741	chromosome									
284162803	hypothetical protein Arcpr_1707	No	0			1444984	1445412	+		Archaeoglobus_profundus_DSM_5631_uid43493	NC_013741	chromosome		arCOG10366	7	L		PD-(D/E)XK superfamily nuclease			
284162804	Aminoacyl-tRNA hydrolase	No	0			1445775	1446125	-		Archaeoglobus_profundus_DSM_5631_uid43493	NC_013741	chromosome		arCOG04228	1 (-)	J	pth2	Peptidyl-tRNA hydrolase	5E-38	pfam01981	Peptidyl-tRNA hydrolase PTH2. Peptidyl-t
284162805	hypothetical protein Arcpr_1710	No	0			1446122	1446379	-		Archaeoglobus_profundus_DSM_5631_uid43493	NC_013741	chromosome		arCOG10234	1	S		Uncharacterized conserved protein			
===									29	Archaeoglobus_profundus_DSM_5631_uid43493	NC_013741	chromosome									
284162836	thiamine biosynthesis protein ThiC	No	0			1468501	1469808	-		Archaeoglobus_profundus_DSM_5631_uid43493	NC_013741	chromosome		arCOG02741	4 (-)	H	ThiC	Thiamine biosynthesis protein ThiC	9E-169	pfam01964	ThiC family. ThiC is found within the th
284162837	hypothetical protein Arcpr_1743	No	0	YES		1470412	1470636	-		Archaeoglobus_profundus_DSM_5631_uid43493	NC_013741	chromosome									
284162838	hypothetical protein Arcpr_1744	No	0	YES		1470633	1471058	-		Archaeoglobus_profundus_DSM_5631_uid43493	NC_013741	chromosome									
284162839	hypothetical protein Arcpr_1745	No	0	YES		1471048	1471242	-		Archaeoglobus_profundus_DSM_5631_uid43493	NC_013741	chromosome									
284162840	hypothetical protein Arcpr_1746	No	0	YES		1471293	1471454	-		Archaeoglobus_profundus_DSM_5631_uid43493	NC_013741	chromosome									
284162841	hypothetical protein Arcpr_1747	No	0	YES		1471475	1472035	-		Archaeoglobus_profundus_DSM_5631_uid43493	NC_013741	chromosome		arCOG07102	9 (-)	L		PD-(D/E)XK superfamily nuclease			
284162842	hypothetical protein Arcpr_1748	No	0	YES		1472032	1472220	-		Archaeoglobus_profundus_DSM_5631_uid43493	NC_013741	chromosome	clust_467								
284162843	hypothetical protein Arcpr_1749	No	0	YES		1472217	1472624	-		Archaeoglobus_profundus_DSM_5631_uid43493	NC_013741	chromosome									
284162844	hypothetical protein Arcpr_1750	No	0	YES		1472621	1472854	-		Archaeoglobus_profundus_DSM_5631_uid43493	NC_013741	chromosome									
284162845	hypothetical protein Arcpr_1751	No	0	YES		1472858	1473772	-		Archaeoglobus_profundus_DSM_5631_uid43493	NC_013741	chromosome									
284162846	transcriptional regulators, CopG/Arc/MetJ famil	No	0	YES		1473766	1474008	-		Archaeoglobus_profundus_DSM_5631_uid43493	NC_013741	chromosome									
284162847	hypothetical protein Arcpr_1753	No	0	YES		1474130	1474393	+		Archaeoglobus_profundus_DSM_5631_uid43493	NC_013741	chromosome		arCOG01009	8 (-)	V		Ribbon-helix-helix protein, copG family			
284162848	hypothetical protein Arcpr_1754	No	0	YES		1474410	1474736	+		Archaeoglobus_profundus_DSM_5631_uid43493	NC_013741	chromosome		arCOG06736	1 (-)	S		Uncharacterized conserved protein			
284162849	hypothetical protein Arcpr_1755	No	0	YES		1474748	1475449	+		Archaeoglobus_profundus_DSM_5631_uid43493	NC_013741	chromosome									
284162850	hypothetical protein Arcpr_1756	No	0	YES		1475451	1476512	+		Archaeoglobus_profundus_DSM_5631_uid43493	NC_013741	chromosome									
284162851	hypothetical protein Arcpr_1757	No	1	YES		1476490	1477038	+		Archaeoglobus_profundus_DSM_5631_uid43493	NC_013741	chromosome									
284162852	hypothetical protein Arcpr_1758	No	5	YES		1477242	1477781	+		Archaeoglobus_profundus_DSM_5631_uid43493	NC_013741	chromosome									
284162853	hypothetical protein Arcpr_1759	No	0	YES		1477827	1478552	+		Archaeoglobus_profundus_DSM_5631_uid43493	NC_013741	chromosome									
284162854	hypothetical protein Arcpr_1760	No	0	YES		1478726	1479019	+		Archaeoglobus_profundus_DSM_5631_uid43493	NC_013741	chromosome									
284162855	hypothetical protein Arcpr_1761	No	0	YES		1479046	1479261	+		Archaeoglobus_profundus_DSM_5631_uid43493	NC_013741	chromosome									
284162856	hypothetical protein Arcpr_1762	No	0	YES		1479419	1479772	+		Archaeoglobus_profundus_DSM_5631_uid43493	NC_013741	chromosome									
284162857	hypothetical protein Arcpr_1763	No	0	YES		1479744	1480208	+		Archaeoglobus_profundus_DSM_5631_uid43493	NC_013741	chromosome									
284162858	hypothetical protein Arcpr_1764	No	0	YES		1480357	1480968	+		Archaeoglobus_profundus_DSM_5631_uid43493	NC_013741	chromosome									
284162859	hypothetical protein Arcpr_1765	No	0	YES		1480971	1481972	+		Archaeoglobus_profundus_DSM_5631_uid43493	NC_013741	chromosome		arCOG10924	1	S		Uncharacterized conserved protein			
284162860	hypothetical protein Arcpr_1766	No	0	YES		1482069	1482497	-		Archaeoglobus_profundus_DSM_5631_uid43493	NC_013741	chromosome									
284162861	hypothetical protein Arcpr_1767	No	0	YES		1482469	1483197	-		Archaeoglobus_profundus_DSM_5631_uid43493	NC_013741	chromosome									
284162862	hypothetical protein Arcpr_1768	No	0	YES		1483275	1483934	-		Archaeoglobus_profundus_DSM_5631_uid43493	NC_013741	chromosome									
284162863	hypothetical protein Arcpr_1769	No	3	YES		1484187	1484660	-		Archaeoglobus_profundus_DSM_5631_uid43493	NC_013741	chromosome									
284162864	hypothetical protein Arcpr_1770	No	0	YES		1484662	1485084	-		Archaeoglobus_profundus_DSM_5631_uid43493	NC_013741	chromosome		arCOG06471	1 (-)	S		TIR domain containing protein			
===									9	Archaeoglobus_profundus_DSM_5631_uid43493	NC_013741	chromosome									
284162881	hypothetical protein Arcpr_1787	No	2	YES		1501521	1501784	-		Archaeoglobus_profundus_DSM_5631_uid43493	NC_013741	chromosome									
284162882	hypothetical protein Arcpr_1788	No	0	YES		1501784	1502692	-		Archaeoglobus_profundus_DSM_5631_uid43493	NC_013741	chromosome		arCOG07960	16 (-)	R		ATPase (AAA+ superfamily)			
284162883	hypothetical protein Arcpr_1789	No	0	YES		1502704	1503171	-		Archaeoglobus_profundus_DSM_5631_uid43493	NC_013741	chromosome									
284162884	hypothetical protein Arcpr_1790	Yes	5	YES		1503152	1504573	-		Archaeoglobus_profundus_DSM_5631_uid43493	NC_013741	chromosome									
284162885	hypothetical protein Arcpr_1791	No	0	YES		1504582	1504986	-		Archaeoglobus_profundus_DSM_5631_uid43493	NC_013741	chromosome									
284162886	hypothetical protein Arcpr_1792	Yes	1	YES		1504983	1506509	-		Archaeoglobus_profundus_DSM_5631_uid43493	NC_013741	chromosome		arCOG07782	7 (-)	S		Uncharacterized conserved protein			
284162887	hypothetical protein Arcpr_1793	No	4	YES		1506524	1506931	-		Archaeoglobus_profundus_DSM_5631_uid43493	NC_013741	chromosome									
284162888	hypothetical protein Arcpr_1794	No	2	YES		1506897	1507187	-		Archaeoglobus_profundus_DSM_5631_uid43493	NC_013741	chromosome									
284162889	hypothetical protein Arcpr_1795	No	0	YES		1507267	1507467	-		Archaeoglobus_profundus_DSM_5631_uid43493	NC_013741	chromosome									
===									19	Archaeoglobus_veneficus_SNP6_uid65269	NC_015320	chromosome									
327399923	hypothetical protein Arcve_0003	No	0		integrated_ele	6432	6629	+		Archaeoglobus_veneficus_SNP6_uid65269	NC_015320	chromosome		arCOG10696	1	S		Uncharacterized conserved protein			
327399924	beta-lactamase domain-containing protein	No	0		integrated_ele	6935	7858	+		Archaeoglobus_veneficus_SNP6_uid65269	NC_015320	chromosome		arCOG00545	2 (-)	J	YSH1	Predicted exonuclease of the beta-lactamase fol	4E-11	COG1236	Predicted exonuclease of the beta-lactam
327399925	hypothetical protein Arcve_0005	No	0		integrated_ele	8357	8704	-		Archaeoglobus_veneficus_SNP6_uid65269	NC_015320	chromosome									
327399926	hypothetical protein Arcve_0006	No	0		integrated_ele	8701	8979	-		Archaeoglobus_veneficus_SNP6_uid65269	NC_015320	chromosome									
327399927	hypothetical protein Arcve_0007	No	0		integrated_ele	8980	9381	-		Archaeoglobus_veneficus_SNP6_uid65269	NC_015320	chromosome									
327399928	hypothetical protein Arcve_0008	No	0		integrated_ele	9831	10286	-		Archaeoglobus_veneficus_SNP6_uid65269	NC_015320	chromosome		arCOG10366	7	L		PD-(D/E)XK superfamily nuclease			
327399929	hypothetical protein Arcve_0009	No	0		integrated_ele	10292	10792	-		Archaeoglobus_veneficus_SNP6_uid65269	NC_015320	chromosome							0.000001	pfam13476	AAA domain.
327399930	hypothetical protein Arcve_0010	No	0		integrated_ele	11095	11775	+		Archaeoglobus_veneficus_SNP6_uid65269	NC_015320	chromosome		arCOG01057	21 (-)	K		Predicted transcriptional regulator			
327399931	hypothetical protein Arcve_0011	No	0		integrated_ele	11918	12337	-		Archaeoglobus_veneficus_SNP6_uid65269	NC_015320	chromosome									
327399932	hypothetical protein Arcve_0012	No	0		integrated_ele	12363	13331	-		Archaeoglobus_veneficus_SNP6_uid65269	NC_015320	chromosome							0.00004	COG0419	ATPase involved in DNA repair
327399933	hypothetical protein Arcve_0013	No	0		integrated_ele	13868	13981	-		Archaeoglobus_veneficus_SNP6_uid65269	NC_015320	chromosome									
327399934	hypothetical protein Arcve_0014	No	0		integrated_ele	14472	14603	-		Archaeoglobus_veneficus_SNP6_uid65269	NC_015320	chromosome									
327399935	hypothetical protein Arcve_0015	No	0		integrated_ele	14665	14904	-		Archaeoglobus_veneficus_SNP6_uid65269	NC_015320	chromosome									
327399936	hypothetical protein Arcve_0016	No	0		integrated_ele	15009	15653	+		Archaeoglobus_veneficus_SNP6_uid65269	NC_015320	chromosome		arCOG04793	2 (-)	V		Transcriptional regulator, predicted component of viral defense system			
327399937	hypothetical protein Arcve_0017	No	0		integrated_ele	15754	16113	-		Archaeoglobus_veneficus_SNP6_uid65269	NC_015320	chromosome									
327399938	hypothetical protein Arcve_0018	No	2		integrated_ele	16183	16440	-		Archaeoglobus_veneficus_SNP6_uid65269	NC_015320	chromosome									
327399939	hypothetical protein Arcve_0019	No	1		integrated_ele	16445	16558	-		Archaeoglobus_veneficus_SNP6_uid65269	NC_015320	chromosome									
327399940	hypothetical protein Arcve_0020	No	0		integrated_ele	16910	18010	-		Archaeoglobus_veneficus_SNP6_uid65269	NC_015320	chromosome		arCOG07960	16 (-)	R		ATPase (AAA+ superfamily)			
327399941	hypothetical protein Arcve_0021	No	0		integrated_ele	17994	18368	-		Archaeoglobus_veneficus_SNP6_uid65269	NC_015320	chromosome									
===									8	Archaeoglobus_veneficus_SNP6_uid65269	NC_015320	chromosome									
327399943	hypothetical protein Arcve_0023	No	0		integrated_ele	20055	20435	-		Archaeoglobus_veneficus_SNP6_uid65269	NC_015320	chromosome									
327399944	hypothetical protein Arcve_0024	No	2		integrated_ele	20476	21936	-		Archaeoglobus_veneficus_SNP6_uid65269	NC_015320	chromosome		arCOG07782	7 (-)	S		Uncharacterized conserved protein			
327399945	hypothetical protein Arcve_0025	Yes	2		integrated_ele	21974	23821	-		Archaeoglobus_veneficus_SNP6_uid65269	NC_015320	chromosome									
327399946	hypothetical protein Arcve_0026	No	4		integrated_ele	23847	24254	-		Archaeoglobus_veneficus_SNP6_uid65269	NC_015320	chromosome									
327399947	hypothetical protein Arcve_0027	No	2		integrated_ele	24261	24587	-		Archaeoglobus_veneficus_SNP6_uid65269	NC_015320	chromosome									
327399948	hypothetical protein Arcve_0028	No	0		integrated_ele	24910	25167	-		Archaeoglobus_veneficus_SNP6_uid65269	NC_015320	chromosome									
327399949	von Willebrand factor type A	Yes	1		integrated_ele	26548	28131	+		Archaeoglobus_veneficus_SNP6_uid65269	NC_015320	chromosome	arCOG02902,a	arCOG02902	1 (-)	R		Uncharacterized protein containing a von Willebra	3E-10	pfam13519	von Willebrand factor type A domain.
327399950	hypothetical protein Arcve_0031	No	0		integrated_ele	28332	28688	+		Archaeoglobus_veneficus_SNP6_uid65269	NC_015320	chromosome									
===									32	Archaeoglobus_veneficus_SNP6_uid65269	NC_015320	chromosome									
327400548	Viral A-type inclusion protein	No	0		Virus_Turrivirid	562634	562882	-		Archaeoglobus_veneficus_SNP6_uid65269	NC_015320	chromosome									
327400549	hypothetical protein Arcve_0653	No	1		Virus_Turrivirid	562882	562992	-		Archaeoglobus_veneficus_SNP6_uid65269	NC_015320	chromosome									
327400550	ATPase	No	0		Virus_Turrivirid	563073	563756	-		Archaeoglobus_veneficus_SNP6_uid65269	NC_015320	chromosome		arCOG00280	21 (-)	L		HerA helicase	0.0000002	COG0433	HerA helicase
327400551	hypothetical protein Arcve_0655	No	0		Virus_Turrivirid	563728	564183	-		Archaeoglobus_veneficus_SNP6_uid65269	NC_015320	chromosome									
327400552	hypothetical protein Arcve_0656	No	0		Virus_Turrivirid	564176	564316	-		Archaeoglobus_veneficus_SNP6_uid65269	NC_015320	chromosome									
327400553	hypothetical protein Arcve_0657	No	1		Virus_Turrivirid	564313	564720	-		Archaeoglobus_veneficus_SNP6_uid65269	NC_015320	chromosome		arCOG04838	1 (-)	S		Zn-ribbon containing protein			
327400554	other/FunK1 protein kinase	No	1		Virus_Turrivirid	564787	564996	-		Archaeoglobus_veneficus_SNP6_uid65269	NC_015320	chromosome									
327400555	hypothetical protein Arcve_0659	No	0		Virus_Turrivirid	564978	565661	-		Archaeoglobus_veneficus_SNP6_uid65269	NC_015320	chromosome									
327400556	hypothetical protein Arcve_0660	No	0		Virus_Turrivirid	565665	566168	-		Archaeoglobus_veneficus_SNP6_uid65269	NC_015320	chromosome									
327400557	SpoVT/AbrB domain-containing protein	No	0		Virus_Turrivirid	566374	566568	-		Archaeoglobus_veneficus_SNP6_uid65269	NC_015320	chromosome		arCOG00318	1 (-)	P	PhoU	Phosphate uptake regulator			
327400558	hypothetical protein Arcve_0662	No	0		Virus_Turrivirid	566565	566678	-		Archaeoglobus_veneficus_SNP6_uid65269	NC_015320	chromosome									
327400559	hypothetical protein Arcve_0663	No	0		Virus_Turrivirid	566675	567241	-		Archaeoglobus_veneficus_SNP6_uid65269	NC_015320	chromosome		arCOG07102	9 (-)	L		PD-(D/E)XK superfamily nuclease			
327400560	hypothetical protein Arcve_0664	No	0		Virus_Turrivirid	567219	567518	-		Archaeoglobus_veneficus_SNP6_uid65269	NC_015320	chromosome									
327400561	hypothetical protein Arcve_0665	No	0		Virus_Turrivirid	567515	567736	-		Archaeoglobus_veneficus_SNP6_uid65269	NC_015320	chromosome									
327400562	putative transcriptional regulator protein	No	0		Virus_Turrivirid	567740	568000	-		Archaeoglobus_veneficus_SNP6_uid65269	NC_015320	chromosome									
327400563	hypothetical protein Arcve_0667	No	0		Virus_Turrivirid	568000	568197	-		Archaeoglobus_veneficus_SNP6_uid65269	NC_015320	chromosome									
327400564	hypothetical protein Arcve_0668	No	0		Virus_Turrivirid	568201	568485	-		Archaeoglobus_veneficus_SNP6_uid65269	NC_015320	chromosome									
327400565	hypothetical protein Arcve_0669	No	0		Virus_Turrivirid	568485	568664	-		Archaeoglobus_veneficus_SNP6_uid65269	NC_015320	chromosome									
327400566	hypothetical protein Arcve_0670	No	1		Virus_Turrivirid	569087	569341	+		Archaeoglobus_veneficus_SNP6_uid65269	NC_015320	chromosome									
327400567	hypothetical protein Arcve_0671	No	1		Virus_Turrivirid	569409	569654	+		Archaeoglobus_veneficus_SNP6_uid65269	NC_015320	chromosome									
327400568	hypothetical protein Arcve_0672	No	0		Virus_Turrivirid	569665	570627	+		Archaeoglobus_veneficus_SNP6_uid65269	NC_015320	chromosome		arCOG10335	5 (-)	Y		Major capsid protein, double jelly roll fold protein			
327400569	hypothetical protein Arcve_0673	No	1		Virus_Turrivirid	570631	570732	+		Archaeoglobus_veneficus_SNP6_uid65269	NC_015320	chromosome									
327400570	hypothetical protein Arcve_0674	No	0		Virus_Turrivirid	570777	570971	+		Archaeoglobus_veneficus_SNP6_uid65269	NC_015320	chromosome									
327400571	hypothetical protein Arcve_0675	No	0		Virus_Turrivirid	570977	571231	+		Archaeoglobus_veneficus_SNP6_uid65269	NC_015320	chromosome	clust_393								
327400572	hypothetical protein Arcve_0676	No	1		Virus_Turrivirid	571228	571326	+		Archaeoglobus_veneficus_SNP6_uid65269	NC_015320	chromosome									
327400573	hypothetical protein Arcve_0677	No	2		Virus_Turrivirid	571329	571838	+		Archaeoglobus_veneficus_SNP6_uid65269	NC_015320	chromosome									
327400574	hypothetical protein Arcve_0678	No	1		Virus_Turrivirid	571835	572812	+		Archaeoglobus_veneficus_SNP6_uid65269	NC_015320	chromosome									
327400575	hypothetical protein Arcve_0679	No	0		Virus_Turrivirid	572814	573809	+		Archaeoglobus_veneficus_SNP6_uid65269	NC_015320	chromosome									
327400576	hypothetical protein Arcve_0680	No	0		Virus_Turrivirid	573820	573918	+		Archaeoglobus_veneficus_SNP6_uid65269	NC_015320	chromosome									
327400577	hypothetical protein Arcve_0681	No	0		Virus_Turrivirid	573919	574218	+		Archaeoglobus_veneficus_SNP6_uid65269	NC_015320	chromosome									
327400578	hypothetical protein Arcve_0682	No	0		Virus_Turrivirid	574215	574730	+		Archaeoglobus_veneficus_SNP6_uid65269	NC_015320	chromosome									
327400579	hypothetical protein Arcve_0683	No	0		Virus_Turrivirid	574747	575622	-		Archaeoglobus_veneficus_SNP6_uid65269	NC_015320	chromosome		arCOG01244	9 (-)	L		Intergrase/Recombinase	3E-20	COG4342	Uncharacterized protein conserved in arc
===									12	Archaeoglobus_veneficus_SNP6_uid65269	NC_015320	chromosome									
327400617	regulatory protein ArsR	No	0			609828	610646	-		Archaeoglobus_veneficus_SNP6_uid65269	NC_015320	chromosome									
327400618	CopG-like DNA-binding domain-containing prot	No	0			610798	610962	+		Archaeoglobus_veneficus_SNP6_uid65269	NC_015320	chromosome		arCOG01057	21 (-)	K		Predicted transcriptional regulator			
327400619	hypothetical protein Arcve_0724	No	0			610977	611135	+		Archaeoglobus_veneficus_SNP6_uid65269	NC_015320	chromosome									
327400620	DNA primase catalytic core domain-containing	No	0			611128	614481	+		Archaeoglobus_veneficus_SNP6_uid65269	NC_015320	chromosome							1E-10	COG0358	DNA primase (bacterial type)
327400621	hypothetical protein Arcve_0726	No	0			614478	614906	+		Archaeoglobus_veneficus_SNP6_uid65269	NC_015320	chromosome		arCOG10366	7	L		PD-(D/E)XK superfamily nuclease			
327400622	hypothetical protein Arcve_0727	No	0			614903	615088	+		Archaeoglobus_veneficus_SNP6_uid65269	NC_015320	chromosome		arCOG11281	2	S		Uncharacterized conserved protein			
327400623	hypothetical protein Arcve_0728	No	0			615108	615344	+		Archaeoglobus_veneficus_SNP6_uid65269	NC_015320	chromosome	clust_410								
327400624	hypothetical protein Arcve_0730	No	0			615748	616158	+		Archaeoglobus_veneficus_SNP6_uid65269	NC_015320	chromosome									
327400625	hypothetical protein Arcve_0731	No	0			616192	616395	+		Archaeoglobus_veneficus_SNP6_uid65269	NC_015320	chromosome									
327400626	hypothetical protein Arcve_0732	No	0			616418	616879	+		Archaeoglobus_veneficus_SNP6_uid65269	NC_015320	chromosome									
327400627	hypothetical protein Arcve_0733	No	2			617107	617385	-		Archaeoglobus_veneficus_SNP6_uid65269	NC_015320	chromosome									
327400628	hypothetical protein Arcve_0734	No	0			618073	619164	-		Archaeoglobus_veneficus_SNP6_uid65269	NC_015320	chromosome		arCOG08624	3 (-)	L		Phage integrase family protein			
===									6	Archaeoglobus_veneficus_SNP6_uid65269	NC_015320	chromosome									
327400730	hypothetical protein Arcve_0839	Yes	1			716297	718306	+		Archaeoglobus_veneficus_SNP6_uid65269	NC_015320	chromosome		arCOG05791	1 (-)	S		Uncharacterized cell suface protein, containing DUF11 domain			
327400731	hypothetical protein Arcve_0840	No	0			718522	718758	-		Archaeoglobus_veneficus_SNP6_uid65269	NC_015320	chromosome									
327400732	hypothetical protein Arcve_0841	No	0			719222	719536	-		Archaeoglobus_veneficus_SNP6_uid65269	NC_015320	chromosome									
327400733	hypothetical protein Arcve_0843	No	0			720224	720493	-		Archaeoglobus_veneficus_SNP6_uid65269	NC_015320	chromosome									
327400734	hypothetical protein Arcve_0844	No	0			720784	721680	-		Archaeoglobus_veneficus_SNP6_uid65269	NC_015320	chromosome									
327400735	3-dehydroquinate synthase	No	0			721920	722990	+		Archaeoglobus_veneficus_SNP6_uid65269	NC_015320	chromosome		arCOG00982	1 (-)	C	GldA	Glycerol dehydrogenase or related enzyme	4E-66	COG0371	Glycerol dehydrogenase and related enzym
===									7	Archaeoglobus_veneficus_SNP6_uid65269	NC_015320	chromosome									
327401391	hypothetical protein Arcve_1513	No	0			1346870	1347073	-		Archaeoglobus_veneficus_SNP6_uid65269	NC_015320	chromosome									
327401392	hypothetical protein Arcve_1514	No	0			1347161	1347538	+		Archaeoglobus_veneficus_SNP6_uid65269	NC_015320	chromosome									
327401393	hypothetical protein Arcve_1515	No	0			1347564	1347716	+		Archaeoglobus_veneficus_SNP6_uid65269	NC_015320	chromosome							0.00004	COG3677	Transposase and inactivated derivatives
327401394	peptidase M10A and M12B matrixin and adama	No	3			1347854	1349083	+		Archaeoglobus_veneficus_SNP6_uid65269	NC_015320	chromosome		arCOG04994	2 (-)	O		Predicted Zn-dependent protease			
327401395	CopG-like DNA-binding domain-containing prot	No	0			1349381	1349548	+		Archaeoglobus_veneficus_SNP6_uid65269	NC_015320	chromosome									
327401396	hypothetical protein Arcve_1518	No	0			1349579	1351831	+		Archaeoglobus_veneficus_SNP6_uid65269	NC_015320	chromosome									
327401397	hypothetical protein Arcve_1519	No	0			1351951	1352166	+		Archaeoglobus_veneficus_SNP6_uid65269	NC_015320	chromosome									
===									7	Archaeoglobus_veneficus_SNP6_uid65269	NC_015320	chromosome									
327401756	hypothetical protein Arcve_1887	No	0			1685895	1686860	+		Archaeoglobus_veneficus_SNP6_uid65269	NC_015320	chromosome									
327401757	hypothetical protein Arcve_1888	No	0			1686844	1687836	+		Archaeoglobus_veneficus_SNP6_uid65269	NC_015320	chromosome									
327401758	hypothetical protein Arcve_1889	No	0			1687829	1688581	-		Archaeoglobus_veneficus_SNP6_uid65269	NC_015320	chromosome									
327401759	hypothetical protein Arcve_1890	No	0			1688610	1688963	-		Archaeoglobus_veneficus_SNP6_uid65269	NC_015320	chromosome		arCOG01057	21 (-)	K		Predicted transcriptional regulator			
327401760	hypothetical protein Arcve_1891	No	0			1689092	1689280	+		Archaeoglobus_veneficus_SNP6_uid65269	NC_015320	chromosome									
327401761	hypothetical protein Arcve_1892	No	0			1689284	1689472	+		Archaeoglobus_veneficus_SNP6_uid65269	NC_015320	chromosome									
327401762	hypothetical protein Arcve_1893	No	3			1689718	1690026	+		Archaeoglobus_veneficus_SNP6_uid65269	NC_015320	chromosome		arCOG10228	2	S		Uncharacterized conserved protein			
===									27	Archaeoglobus_veneficus_SNP6_uid65269	NC_015320	chromosome									
327401917	hypothetical protein Arcve_2051	No	2			1822215	1822451	+		Archaeoglobus_veneficus_SNP6_uid65269	NC_015320	chromosome									
327401918	hypothetical protein Arcve_2052	No	2			1822479	1822712	-		Archaeoglobus_veneficus_SNP6_uid65269	NC_015320	chromosome									
327401919	CopG-like DNA-binding domain-containing prot	No	0			1823332	1823547	-		Archaeoglobus_veneficus_SNP6_uid65269	NC_015320	chromosome		arCOG01008	7 (-)	V	NikR	Transcriptional regulator, CopG/Arc/MetJ family (I	0.000005	COG0864	Predicted transcriptional regulators con
327401920	hypothetical protein Arcve_2054	No	0			1823642	1823809	+		Archaeoglobus_veneficus_SNP6_uid65269	NC_015320	chromosome									
327401921	hypothetical protein Arcve_2055	No	0			1823813	1824103	+		Archaeoglobus_veneficus_SNP6_uid65269	NC_015320	chromosome									
327401922	hypothetical protein Arcve_2056	No	0			1824100	1824216	+		Archaeoglobus_veneficus_SNP6_uid65269	NC_015320	chromosome									
327401923	hypothetical protein Arcve_2057	No	0			1824216	1824374	+		Archaeoglobus_veneficus_SNP6_uid65269	NC_015320	chromosome									
327401924	hypothetical protein Arcve_2058	No	0			1824371	1824805	+		Archaeoglobus_veneficus_SNP6_uid65269	NC_015320	chromosome		arCOG10366	7	L		PD-(D/E)XK superfamily nuclease			
327401925	hypothetical protein Arcve_2059	No	0			1824795	1824962	+		Archaeoglobus_veneficus_SNP6_uid65269	NC_015320	chromosome									
327401926	hypothetical protein Arcve_2060	No	0			1824973	1825332	+		Archaeoglobus_veneficus_SNP6_uid65269	NC_015320	chromosome									
327401927	DNA-binding domain-containing protein	No	0			1825457	1825981	+		Archaeoglobus_veneficus_SNP6_uid65269	NC_015320	chromosome							0.000004	COG0419	ATPase involved in DNA repair
327401928	hypothetical protein Arcve_2062	No	0			1826070	1826285	+		Archaeoglobus_veneficus_SNP6_uid65269	NC_015320	chromosome									
327401929	heterogeneous nuclear ribonucleoprotein A3	No	0			1826407	1826700	+		Archaeoglobus_veneficus_SNP6_uid65269	NC_015320	chromosome									
327401930	hypothetical protein Arcve_2064	No	0			1826709	1827173	+		Archaeoglobus_veneficus_SNP6_uid65269	NC_015320	chromosome									
327401931	hypothetical protein Arcve_2065	No	0			1827139	1827285	+		Archaeoglobus_veneficus_SNP6_uid65269	NC_015320	chromosome									
327401932	hypothetical protein Arcve_2066	No	0			1827282	1827416	+		Archaeoglobus_veneficus_SNP6_uid65269	NC_015320	chromosome									
327401933	AAA ATPase	No	0			1827413	1827964	+		Archaeoglobus_veneficus_SNP6_uid65269	NC_015320	chromosome		arCOG00280	21 (-)	L		HerA helicase			
327401934	hypothetical protein Arcve_2068	No	1			1828148	1828381	+		Archaeoglobus_veneficus_SNP6_uid65269	NC_015320	chromosome									
327401935	hypothetical protein Arcve_2069	No	0			1828381	1828899	+		Archaeoglobus_veneficus_SNP6_uid65269	NC_015320	chromosome									
327401936	hypothetical protein Arcve_2070	No	0			1828930	1829328	+		Archaeoglobus_veneficus_SNP6_uid65269	NC_015320	chromosome									
327401937	hypothetical protein Arcve_2071	Yes	0			1829325	1830062	+		Archaeoglobus_veneficus_SNP6_uid65269	NC_015320	chromosome									
327401938	hypothetical protein Arcve_2072	No	0			1830059	1830214	+		Archaeoglobus_veneficus_SNP6_uid65269	NC_015320	chromosome									
327401939	hypothetical protein Arcve_2073	No	0			1830186	1830419	+		Archaeoglobus_veneficus_SNP6_uid65269	NC_015320	chromosome	clust_393								
327401940	hypothetical protein Arcve_2074	No	0			1830416	1831111	+		Archaeoglobus_veneficus_SNP6_uid65269	NC_015320	chromosome									
327401941	hypothetical protein Arcve_2075	No	0			1831102	1831368	+		Archaeoglobus_veneficus_SNP6_uid65269	NC_015320	chromosome									
327401942	hypothetical protein Arcve_2076	No	2			1831365	1831598	+		Archaeoglobus_veneficus_SNP6_uid65269	NC_015320	chromosome	clust_415								
327401943	hypothetical protein Arcve_2077	No	0			1831573	1832178	-		Archaeoglobus_veneficus_SNP6_uid65269	NC_015320	chromosome		arCOG01244	9 (-)	L		Intergrase/Recombinase	8E-16	COG4342	Uncharacterized protein conserved in arc
===									8	Caldivirga_maquilingensis_IC_167_uid58711	NC_009954	chromosome									
159040818	extracellular solute-binding protein	Yes	2			267533	269896	-		Caldivirga_maquilingensis_IC_167_uid58711	NC_009954	chromosome		arCOG01534	2 (-)	E	DdpA	ABC-type transport system, periplasmic compone	0.000000004	COG0747	ABC-type dipeptide transport system, per ; Mucin-like glycoprotein. This family of
159040819	hypothetical protein Cmaq_0233	No	0			270030	271133	-		Caldivirga_maquilingensis_IC_167_uid58711	NC_009954	chromosome									
159040820	hypothetical protein Cmaq_0234	No	0			271385	272365	-		Caldivirga_maquilingensis_IC_167_uid58711	NC_009954	chromosome							7E-63	pfam11175	Protein of unknown function (DUF2961). T
159040821	hypothetical protein Cmaq_0235	No	0			272583	272789	+		Caldivirga_maquilingensis_IC_167_uid58711	NC_009954	chromosome									
159040822	hypothetical protein Cmaq_0236	No	0			272786	272887	+		Caldivirga_maquilingensis_IC_167_uid58711	NC_009954	chromosome									
159040823	sulfatase	No	0			273001	274344	-		Caldivirga_maquilingensis_IC_167_uid58711	NC_009954	chromosome		arCOG02785	3 (-)	P	AslA	Arylsulfatase A or related enzyme	8E-26	COG3119	Arylsulfatase A and related enzymes
159040824	hypothetical protein Cmaq_0238	No	0			274354	274704	-		Caldivirga_maquilingensis_IC_167_uid58711	NC_009954	chromosome									
159040825	hypothetical protein Cmaq_0239	Yes	1			274818	275483	+		Caldivirga_maquilingensis_IC_167_uid58711	NC_009954	chromosome									
===									5	Caldivirga_maquilingensis_IC_167_uid58711	NC_009954	chromosome									
159041483	hypothetical protein Cmaq_0912	No	6			956167	956751	+		Caldivirga_maquilingensis_IC_167_uid58711	NC_009954	chromosome		arCOG08084	1	S		Membrane protein			
159041484	hypothetical protein Cmaq_0913	No	6			956788	957465	+		Caldivirga_maquilingensis_IC_167_uid58711	NC_009954	chromosome									
159041485	alcohol dehydrogenase	No	0			957462	958511	-		Caldivirga_maquilingensis_IC_167_uid58711	NC_009954	chromosome		arCOG01455	1 (-)	R	AdhP	Zn-dependent alcohol dehydrogenase	7E-77	COG1064	Zn-dependent alcohol dehydrogenases
159041486	hypothetical protein Cmaq_0915	No	0			958675	961659	-		Caldivirga_maquilingensis_IC_167_uid58711	NC_009954	chromosome									
159041487	hypothetical protein Cmaq_0916	No	1			961779	962117	+		Caldivirga_maquilingensis_IC_167_uid58711	NC_009954	chromosome									
===									6	Caldivirga_maquilingensis_IC_167_uid58711	NC_009954	chromosome									
159041828	hypothetical protein Cmaq_1263	No	0			1335039	1335521	-		Caldivirga_maquilingensis_IC_167_uid58711	NC_009954	chromosome									
159041829	hypothetical protein Cmaq_1264	No	0			1335637	1335945	-		Caldivirga_maquilingensis_IC_167_uid58711	NC_009954	chromosome									
159041830	hypothetical protein Cmaq_1265	Yes	0			1336152	1336913	+		Caldivirga_maquilingensis_IC_167_uid58711	NC_009954	chromosome		arCOG06964	1 (-)	E		Peptidase A4 family	5E-18	pfam01828	Peptidase A4 family.
159041831	hypothetical protein Cmaq_1266	No	2			1337058	1337306	+		Caldivirga_maquilingensis_IC_167_uid58711	NC_009954	chromosome									
159041832	hypothetical protein Cmaq_1267	No	2			1337296	1337502	+		Caldivirga_maquilingensis_IC_167_uid58711	NC_009954	chromosome									
159041833	hypothetical protein Cmaq_1268	No	0			1337602	1337943	-		Caldivirga_maquilingensis_IC_167_uid58711	NC_009954	chromosome									
===									6	Caldivirga_maquilingensis_IC_167_uid58711	NC_009954	chromosome									
159042293	hypothetical protein Cmaq_1732	No	5			1807933	1809138	-		Caldivirga_maquilingensis_IC_167_uid58711	NC_009954	chromosome									
159042294	hypothetical protein Cmaq_1733	No	2			1809216	1809731	-		Caldivirga_maquilingensis_IC_167_uid58711	NC_009954	chromosome		arCOG10499	1	S		Uncharacterized conserved protein			
159042295	molybdenum cofactor synthesis domain-contai	No	0			1809786	1810340	-		Caldivirga_maquilingensis_IC_167_uid58711	NC_009954	chromosome		arCOG00214	1 (-)	H	MoaB	Molybdopterin biosynthesis enzyme	2E-33	COG0521	Molybdopterin biosynthesis enzymes
159042296	hypothetical protein Cmaq_1735	No	5			1810513	1813803	-		Caldivirga_maquilingensis_IC_167_uid58711	NC_009954	chromosome									
159042297	hypothetical protein Cmaq_1736	No	2			1813800	1814204	-		Caldivirga_maquilingensis_IC_167_uid58711	NC_009954	chromosome		arCOG10500	4	S		Uncharacterized conserved protein			
159042298	hypothetical protein Cmaq_1737	No	0			1814294	1815328	+		Caldivirga_maquilingensis_IC_167_uid58711	NC_009954	chromosome									
===									6	Caldivirga_maquilingensis_IC_167_uid58711	NC_009954	chromosome									
159042429	hypothetical protein Cmaq_1874	No	3			1947594	1948982	+		Caldivirga_maquilingensis_IC_167_uid58711	NC_009954	chromosome		arCOG05475	4 (-)	S		Uncharacterized conserved protein			
159042430	hypothetical protein Cmaq_1875	No	3			1948984	1951080	+		Caldivirga_maquilingensis_IC_167_uid58711	NC_009954	chromosome		arCOG10501	3	S		Uncharacterized conserved protein			
159042431	hypothetical protein Cmaq_1876	No	1			1951074	1952672	+		Caldivirga_maquilingensis_IC_167_uid58711	NC_009954	chromosome		arCOG10502	4	S		Uncharacterized conserved protein			
159042432	hypothetical protein Cmaq_1877	No	4			1952669	1953439	+		Caldivirga_maquilingensis_IC_167_uid58711	NC_009954	chromosome		arCOG10503	5	S		Uncharacterized conserved protein			
159042433	hypothetical protein Cmaq_1878	No	1			1953444	1954001	+		Caldivirga_maquilingensis_IC_167_uid58711	NC_009954	chromosome		arCOG10504	3	S		Uncharacterized conserved protein			
159042434	DEAD/DEAH box helicase domain-containing p	No	0			1953998	1956910	-		Caldivirga_maquilingensis_IC_167_uid58711	NC_009954	chromosome		arCOG00557	3 (-)	R	Lhr	Lhr-like helicase	7E-176	COG1201	Lhr-like helicases
===									5	Caldivirga_maquilingensis_IC_167_uid58711	NC_009954	chromosome									
159042474	hypothetical protein Cmaq_1919	No	1			1997053	1997553	-		Caldivirga_maquilingensis_IC_167_uid58711	NC_009954	chromosome		arCOG11037	1	S		Uncharacterized conserved protein	0.000002	COG3430	Uncharacterized protein conserved in arc
159042475	hypothetical protein Cmaq_1920	No	3			1997726	1998382	-		Caldivirga_maquilingensis_IC_167_uid58711	NC_009954	chromosome									
159042476	hypothetical protein Cmaq_1921	No	0			1998407	1999042	-		Caldivirga_maquilingensis_IC_167_uid58711	NC_009954	chromosome		arCOG04306	1 (-)	L		Predicted endonuclease (RecB family)	6E-32	COG4998	Predicted endonuclease (RecB family)
159042477	hypothetical protein Cmaq_1922	No	0			1999679	2000005	-		Caldivirga_maquilingensis_IC_167_uid58711	NC_009954	chromosome		arCOG10506	1	S		Uncharacterized conserved protein			
159042478	hypothetical protein Cmaq_1923	No	0			2000154	2000819	+		Caldivirga_maquilingensis_IC_167_uid58711	NC_009954	chromosome		arCOG10349	1	S		Uncharacterized conserved protein			
===									8	Candidatus_Korarchaeum_cryptofilum_OPF8_uid	NC_010482	chromosome									
170289725	XRE family transcriptional regulator	No	0			98455	99234	+		Candidatus_Korarchaeum_cryptofilum_OPF8_uid	NC_010482	chromosome		arCOG04060	1 (-)	K		Predicted transcriptional regulator	3E-85	COG1709	Predicted transcriptional regulator
170289726	hypothetical protein Kcr_0099	Yes	1			99507	100376	-		Candidatus_Korarchaeum_cryptofilum_OPF8_uid	NC_010482	chromosome	clust_32								
170289727	hypothetical protein Kcr_0100	Yes	1			100481	101773	+		Candidatus_Korarchaeum_cryptofilum_OPF8_uid	NC_010482	chromosome	clust_27								
170289728	hypothetical protein Kcr_0101	Yes	0			101912	102787	-		Candidatus_Korarchaeum_cryptofilum_OPF8_uid	NC_010482	chromosome	clust_32								
170289729	hypothetical protein Kcr_0102	Yes	1			102876	104072	+		Candidatus_Korarchaeum_cryptofilum_OPF8_uid	NC_010482	chromosome	clust_27								
170289730	hypothetical protein Kcr_0103	Yes	1			104162	105040	-		Candidatus_Korarchaeum_cryptofilum_OPF8_uid	NC_010482	chromosome	clust_32								
170289731	hypothetical protein Kcr_0104	No	1			105109	106353	+		Candidatus_Korarchaeum_cryptofilum_OPF8_uid	NC_010482	chromosome	clust_27								
170289732	peptidase M28	Yes	1			106593	108752	+		Candidatus_Korarchaeum_cryptofilum_OPF8_uid	NC_010482	chromosome		arCOG02960	1 (-)	R		Predicted aminopeptidase, lap family	0.0000003	pfam04389	Peptidase family M28.
===									5	Candidatus_Korarchaeum_cryptofilum_OPF8_uid	NC_010482	chromosome									
170289799	alkyl hydroperoxide reductase/ Thiol specific a	No	1			172077	173348	+		Candidatus_Korarchaeum_cryptofilum_OPF8_uid	NC_010482	chromosome		arCOG07842	2	S		Uncharacterized conserved protein			
170289800	hypothetical protein Kcr_0173	No	0			173345	174343	-		Candidatus_Korarchaeum_cryptofilum_OPF8_uid	NC_010482	chromosome									
170289801	hypothetical protein Kcr_0174	No	0			174698	176251	+		Candidatus_Korarchaeum_cryptofilum_OPF8_uid	NC_010482	chromosome									
170289802	D-isomer specific 2-hydroxyacid dehydrogena	No	0			176228	177133	-		Candidatus_Korarchaeum_cryptofilum_OPF8_uid	NC_010482	chromosome		arCOG01754	1 (-)	H	SerA	Phosphoglycerate dehydrogenase or related del	1E-58	COG1052	Lactate dehydrogenase and related dehydr
170289803	hypothetical protein Kcr_0176	No	5			177901	178434	-		Candidatus_Korarchaeum_cryptofilum_OPF8_uid	NC_010482	chromosome									
===									7	Candidatus_Korarchaeum_cryptofilum_OPF8_uid	NC_010482	chromosome									
170290400	hypothetical protein Kcr_0786	No	1			797798	798151	+		Candidatus_Korarchaeum_cryptofilum_OPF8_uid	NC_010482	chromosome		arCOG05419	1 (-)	S		Uncharacterized conserved protein			
170290401	hypothetical protein Kcr_0787	No	0			798172	798399	-		Candidatus_Korarchaeum_cryptofilum_OPF8_uid	NC_010482	chromosome									
170290402	hypothetical protein Kcr_0788	No	3			798539	798790	-		Candidatus_Korarchaeum_cryptofilum_OPF8_uid	NC_010482	chromosome							0.00004	COG2034	Predicted membrane protein
170290403	hypothetical protein Kcr_0789	Yes	3			798787	798999	-		Candidatus_Korarchaeum_cryptofilum_OPF8_uid	NC_010482	chromosome							2E-11	COG2034	Predicted membrane protein
170290404	hypothetical protein Kcr_0790	No	0			799274	799927	+		Candidatus_Korarchaeum_cryptofilum_OPF8_uid	NC_010482	chromosome		arCOG09429	1	S		Uncharacterized conserved protein			
170290405	ABC transporter-like protein	No	0			799958	800695	+		Candidatus_Korarchaeum_cryptofilum_OPF8_uid	NC_010482	chromosome		arCOG00194	14 (-)	V	CcmA	ABC-type multidrug transport system, ATPase co	2E-58	COG1131	ABC-type multidrug transport system, ATP
170290406	permease	No	12			800704	802164	+		Candidatus_Korarchaeum_cryptofilum_OPF8_uid	NC_010482	chromosome							3E-16	COG3368	Predicted permease
===									6	Candidatus_Korarchaeum_cryptofilum_OPF8_uid	NC_010482	chromosome									
170290511	tubulin/FtsZ GTPase	No	0			905186	906157	+		Candidatus_Korarchaeum_cryptofilum_OPF8_uid	NC_010482	chromosome		arCOG02201	4 (-)	D	FtsZ	Cell division GTPase	1E-48	COG0206	Cell division GTPase
170290512	hypothetical protein Kcr_0899	No	0			906264	907172	+		Candidatus_Korarchaeum_cryptofilum_OPF8_uid	NC_010482	chromosome									
170290513	hypothetical protein Kcr_0900	No	0			907173	907451	-		Candidatus_Korarchaeum_cryptofilum_OPF8_uid	NC_010482	chromosome									
170290514	hypothetical protein Kcr_0901	No	1			907456	908886	-		Candidatus_Korarchaeum_cryptofilum_OPF8_uid	NC_010482	chromosome							0.00005	COG0419	ATPase involved in DNA repair
170290515	hypothetical protein Kcr_0902	No	0			909227	909457	+		Candidatus_Korarchaeum_cryptofilum_OPF8_uid	NC_010482	chromosome									
170290516	small GTP-binding protein	No	0			909462	910067	-		Candidatus_Korarchaeum_cryptofilum_OPF8_uid	NC_010482	chromosome		arCOG00362	2 (-)	R		Predicted GTPase	2E-15	COG2229	Predicted GTPase
===									6	Candidatus_Korarchaeum_cryptofilum_OPF8_uid	NC_010482	chromosome									
170290518	hypothetical protein Kcr_0905	No	0			911697	912452	+		Candidatus_Korarchaeum_cryptofilum_OPF8_uid	NC_010482	chromosome									
170290519	hypothetical protein Kcr_0906	No	0			912481	913014	-		Candidatus_Korarchaeum_cryptofilum_OPF8_uid	NC_010482	chromosome									
170290520	hypothetical protein Kcr_0907	No	0			913238	913600	+		Candidatus_Korarchaeum_cryptofilum_OPF8_uid	NC_010482	chromosome									
170290521	small GTP-binding protein	No	0			913644	914546	+		Candidatus_Korarchaeum_cryptofilum_OPF8_uid	NC_010482	chromosome		arCOG00362	2 (-)	R		Predicted GTPase	5E-41	pfam00025	ADP-ribosylation factor family. Pfam com
170290522	hypothetical protein Kcr_0909	No	0			914559	915014	+		Candidatus_Korarchaeum_cryptofilum_OPF8_uid	NC_010482	chromosome									
170290523	hypothetical protein Kcr_0910	No	6			915083	915856	+		Candidatus_Korarchaeum_cryptofilum_OPF8_uid	NC_010482	chromosome									
===									9	Candidatus_Korarchaeum_cryptofilum_OPF8_uid	NC_010482	chromosome									
170290926	hypothetical protein Kcr_1313	No	0			1312846	1313322	+		Candidatus_Korarchaeum_cryptofilum_OPF8_uid	NC_010482	chromosome		arCOG05463	1 (-)	S		Uncharacterized conserved protein			
170290927	hypothetical protein Kcr_1314	No	2			1313312	1313578	-		Candidatus_Korarchaeum_cryptofilum_OPF8_uid	NC_010482	chromosome									
170290928	hypothetical protein Kcr_1315	Yes	1			1313568	1314371	-		Candidatus_Korarchaeum_cryptofilum_OPF8_uid	NC_010482	chromosome									
170290929	hypothetical protein Kcr_1316	No	1			1314368	1314637	-		Candidatus_Korarchaeum_cryptofilum_OPF8_uid	NC_010482	chromosome									
170290930	hypothetical protein Kcr_1317	Yes	0			1314897	1315298	-		Candidatus_Korarchaeum_cryptofilum_OPF8_uid	NC_010482	chromosome									
170290931	hypothetical protein Kcr_1318	No	1			1315525	1315788	+		Candidatus_Korarchaeum_cryptofilum_OPF8_uid	NC_010482	chromosome									
170290932	hypothetical protein Kcr_1319	No	4			1315848	1316690	-		Candidatus_Korarchaeum_cryptofilum_OPF8_uid	NC_010482	chromosome									
170290933	type II secretion system protein E	No	0			1316725	1318164	-		Candidatus_Korarchaeum_cryptofilum_OPF8_uid	NC_010482	chromosome		arCOG01818	1 (-)	N	VirB11	Type IV secretory pathway, VirB11 component, o	1E-12	COG0630	Type IV secretory pathway, VirB11 compon
170290934	hypothetical protein Kcr_1321	No	2			1318151	1318786	-		Candidatus_Korarchaeum_cryptofilum_OPF8_uid	NC_010482	chromosome									
===									6	Candidatus_Nitrosopumilus_AR2_uid176130	NC_018656	chromosome									
407463979	hypothetical protein NSED_00535	Yes	0			94883	96631	+		Candidatus_Nitrosopumilus_AR2_uid176130	NC_018656	chromosome		arCOG08777	3 (-)	S		Uncharacterized conserved protein			
407463980	hypothetical protein NSED_00540	No	1			96656	98128	+		Candidatus_Nitrosopumilus_AR2_uid176130	NC_018656	chromosome									
407463981	hypothetical protein NSED_00545	No	0			98338	98421	+		Candidatus_Nitrosopumilus_AR2_uid176130	NC_018656	chromosome									
407463982	hypothetical protein NSED_00550	Yes	0			98445	98924	-		Candidatus_Nitrosopumilus_AR2_uid176130	NC_018656	chromosome		arCOG10592	2	S		Uncharacterized conserved protein			
407463983	hypothetical protein NSED_00555	No	0			99240	99449	+		Candidatus_Nitrosopumilus_AR2_uid176130	NC_018656	chromosome		arCOG10591	2	S		Uncharacterized conserved protein			
407463984	protoheme IX farnesyltransferase	No	9			99444	100373	-		Candidatus_Nitrosopumilus_AR2_uid176130	NC_018656	chromosome		arCOG00479	2 (-)	O	CyoE	Polyprenyltransferase (cytochrome oxidase asse	2E-50	COG0109	Polyprenyltransferase (cytochrome oxidas
===									8	Candidatus_Nitrosopumilus_AR2_uid176130	NC_018656	chromosome									
407464142	hypothetical protein NSED_01350	No	0			229925	230146	+		Candidatus_Nitrosopumilus_AR2_uid176130	NC_018656	chromosome									
407464143	hypothetical protein NSED_01355	Yes	0			230272	230613	+		Candidatus_Nitrosopumilus_AR2_uid176130	NC_018656	chromosome							0.0000004	pfam07382	Histone H1-like nucleoprotein HC2. This
407464144	hypothetical protein NSED_01360	No	0			230769	230945	-		Candidatus_Nitrosopumilus_AR2_uid176130	NC_018656	chromosome		arCOG01667	2 (-)	S		Zinc finger domain containing protein (CDGSH-typ	0.00000002	pfam09360	Iron-binding zinc finger CDGSH type. The
407464145	hypothetical protein NSED_01365	No	0			231052	231267	+		Candidatus_Nitrosopumilus_AR2_uid176130	NC_018656	chromosome									
407464146	hypothetical protein NSED_01370	No	0			231368	231838	-		Candidatus_Nitrosopumilus_AR2_uid176130	NC_018656	chromosome									
407464147	hypothetical protein NSED_01375	No	0			231868	232134	-		Candidatus_Nitrosopumilus_AR2_uid176130	NC_018656	chromosome									
407464148	hypothetical protein NSED_01380	No	0			232358	232609	+		Candidatus_Nitrosopumilus_AR2_uid176130	NC_018656	chromosome		arCOG01117	5 (-)	K	Lrp	Lrp/AsnC family C-terminal domain			
407464149	hypothetical protein NSED_01385	No	0			232683	232904	+		Candidatus_Nitrosopumilus_AR2_uid176130	NC_018656	chromosome									
===									8	Candidatus_Nitrosopumilus_AR2_uid176130	NC_018656	chromosome									
407464219	nitroreductase	No	0			301498	302037	+		Candidatus_Nitrosopumilus_AR2_uid176130	NC_018656	chromosome		arCOG00288	8 (-)	C	NfnB	Nitroreductase	4E-12	COG0778	Nitroreductase
407464220	hypothetical protein NSED_01740	No	0			302081	302293	+		Candidatus_Nitrosopumilus_AR2_uid176130	NC_018656	chromosome									
407464221	hypothetical protein NSED_01745	Yes	0			302467	302586	-		Candidatus_Nitrosopumilus_AR2_uid176130	NC_018656	chromosome									
407464222	hypothetical protein NSED_01750	No	0			302695	302895	+		Candidatus_Nitrosopumilus_AR2_uid176130	NC_018656	chromosome	clust_456								
407464223	hypothetical protein NSED_01755	No	0			302885	303124	+		Candidatus_Nitrosopumilus_AR2_uid176130	NC_018656	chromosome									
407464224	hypothetical protein NSED_01760	No	0			303150	303821	+		Candidatus_Nitrosopumilus_AR2_uid176130	NC_018656	chromosome		arCOG01907	1 (-)	S		Uncharacterized conserved protein	1E-40	pfam01987	Mitochondrial biogenesis AIM24. In eukar
407464225	hypothetical protein NSED_01765	No	0			303858	304040	+		Candidatus_Nitrosopumilus_AR2_uid176130	NC_018656	chromosome									
407464226	hypothetical protein NSED_01770	No	1			304060	304374	+		Candidatus_Nitrosopumilus_AR2_uid176130	NC_018656	chromosome									
===									7	Candidatus_Nitrosopumilus_AR2_uid176130	NC_018656	chromosome									
407464438	transcriptional regulator protein-like protein	No	0			477012	477824	+		Candidatus_Nitrosopumilus_AR2_uid176130	NC_018656	chromosome		arCOG04362	5 (-)	K		Predicted transcriptional regulator	0.000003	COG4742	Predicted transcriptional regulator
407464439	hypothetical protein NSED_02865	Yes	1			478331	478564	+		Candidatus_Nitrosopumilus_AR2_uid176130	NC_018656	chromosome									
407464440	hypothetical protein NSED_02870	No	0			478557	478760	+		Candidatus_Nitrosopumilus_AR2_uid176130	NC_018656	chromosome									
407464441	hypothetical protein NSED_02875	No	0			479230	479418	-		Candidatus_Nitrosopumilus_AR2_uid176130	NC_018656	chromosome	clust_465						0.00008	pfam10368	Putative cell-wall binding lipoprotein.
407464442	hypothetical protein NSED_02880	No	0			479856	480719	+		Candidatus_Nitrosopumilus_AR2_uid176130	NC_018656	chromosome	clust_115								
407464443	transcription factor TFIIB cyclin-related protein	No	0			480741	481637	+		Candidatus_Nitrosopumilus_AR2_uid176130	NC_018656	chromosome		arCOG01981	8 (-)	K	SUA7	Transcription initiation factor TFIIB, Brf1 subunit/Tra	5E-78	COG1405	Transcription initiation factor TFIIIB,
407464444	hypothetical protein NSED_02890	No	0			481646	481858	-		Candidatus_Nitrosopumilus_AR2_uid176130	NC_018656	chromosome									
===									5	Candidatus_Nitrosopumilus_AR2_uid176130	NC_018656	chromosome									
407464576	hypothetical protein NSED_03550	No	0			591303	591680	-		Candidatus_Nitrosopumilus_AR2_uid176130	NC_018656	chromosome									
407464577	hypothetical protein NSED_03555	No	0			591673	592185	-		Candidatus_Nitrosopumilus_AR2_uid176130	NC_018656	chromosome									
407464578	hypothetical protein NSED_03560	No	0			592442	592762	-		Candidatus_Nitrosopumilus_AR2_uid176130	NC_018656	chromosome		arCOG08023	1 (-)	R		Protein implicated in RNA metabolism, contains two Zn-ribbon domain and PRC barrel domain			
407464579	hypothetical protein NSED_03565	No	0			593183	593611	-		Candidatus_Nitrosopumilus_AR2_uid176130	NC_018656	chromosome									
407464580	hypothetical protein NSED_03570	No	0			593838	594026	-		Candidatus_Nitrosopumilus_AR2_uid176130	NC_018656	chromosome	clust_468								
===									15	Candidatus_Nitrosopumilus_AR2_uid176130	NC_018656	chromosome									
407464627	hypothetical protein NSED_03805	No	0			632463	633065	-		Candidatus_Nitrosopumilus_AR2_uid176130	NC_018656	chromosome									
407464628	hypothetical protein NSED_03810	No	0			633098	633805	-		Candidatus_Nitrosopumilus_AR2_uid176130	NC_018656	chromosome									
407464629	hypothetical protein NSED_03815	No	0			633919	634830	+		Candidatus_Nitrosopumilus_AR2_uid176130	NC_018656	chromosome		arCOG01875	2 (-)	K		ParB-like nuclease domain			
407464630	hypothetical protein NSED_03820	No	0			635380	635634	+		Candidatus_Nitrosopumilus_AR2_uid176130	NC_018656	chromosome		arCOG11049	2	S		Uncharacterized conserved protein			
407464631	hypothetical protein NSED_03825	No	1			635631	636311	+		Candidatus_Nitrosopumilus_AR2_uid176130	NC_018656	chromosome	clust_150								
407464632	hypothetical protein NSED_03830	Yes	2			636315	636581	+		Candidatus_Nitrosopumilus_AR2_uid176130	NC_018656	chromosome	clust_379								
407464633	hypothetical protein NSED_03835	No	1			636578	637366	+		Candidatus_Nitrosopumilus_AR2_uid176130	NC_018656	chromosome									
407464634	hypothetical protein NSED_03840	No	1			637354	638442	+		Candidatus_Nitrosopumilus_AR2_uid176130	NC_018656	chromosome									
407464635	hypothetical protein NSED_03845	Yes	1			638538	639650	+		Candidatus_Nitrosopumilus_AR2_uid176130	NC_018656	chromosome		arCOG02470	4 (-)	M	LolA	Outer membrane lipoprotein-sorting protein			
407464636	hypothetical protein NSED_03850	No	3			639647	640000	+		Candidatus_Nitrosopumilus_AR2_uid176130	NC_018656	chromosome									
407464637	hypothetical protein NSED_03855	No	1			639982	640947	+		Candidatus_Nitrosopumilus_AR2_uid176130	NC_018656	chromosome	clust_101								
407464638	hypothetical protein NSED_03860	No	1			640951	641406	+		Candidatus_Nitrosopumilus_AR2_uid176130	NC_018656	chromosome									
407464639	hypothetical protein NSED_03865	Yes	1			641762	643189	+		Candidatus_Nitrosopumilus_AR2_uid176130	NC_018656	chromosome		arCOG08796	2 (-)	S		Uncharacterized conserved protein			
407464640	hypothetical protein NSED_03870	Yes	2			643215	643781	+		Candidatus_Nitrosopumilus_AR2_uid176130	NC_018656	chromosome									
407464641	hypothetical protein NSED_03875	Yes	1			643778	644005	+		Candidatus_Nitrosopumilus_AR2_uid176130	NC_018656	chromosome									
===									6	Candidatus_Nitrosopumilus_AR2_uid176130	NC_018656	chromosome									
407464643	hypothetical protein NSED_03885	Yes	2			645184	645498	+		Candidatus_Nitrosopumilus_AR2_uid176130	NC_018656	chromosome		arCOG10587	1 (-)	S		Uncharacterized conserved protein			
407464644	hypothetical protein NSED_03890	No	1			645590	646234	+		Candidatus_Nitrosopumilus_AR2_uid176130	NC_018656	chromosome									
407464645	hypothetical protein NSED_03895	No	2			646259	646468	+		Candidatus_Nitrosopumilus_AR2_uid176130	NC_018656	chromosome	clust_55								

407464646	hypothetical protein NSED_03900	No	2	646465	646725	+		Candidatus_Nitrosopumilus_AR2_uid176130	NC_018656	chromosome									
407464647	hypothetical protein NSED_03905	No	0	646873	647091	-		Candidatus_Nitrosopumilus_AR2_uid176130	NC_018656	chromosome									
407464648	hypothetical protein NSED_03910	No	0	647376	647993	-		Candidatus_Nitrosopumilus_AR2_uid176130	NC_018656	chromosome		arCOG01686	3 (-)	K	ArsR	Transcriptional regulator containing HTH domain, ArsR family			
===							20	Candidatus_Nitrosopumilus_AR2_uid176130	NC_018656	chromosome									
407464683	hypothetical protein NSED_04085	No	1	678533	679183	+		Candidatus_Nitrosopumilus_AR2_uid176130	NC_018656	chromosome									
407464684	type B DNA polymerase	No	0	679214	680968	-		Candidatus_Nitrosopumilus_AR2_uid176130	NC_018656	chromosome		arCOG00328	8 (-)	L	PolB	DNA polymerase elongation subunit (family B)	5E-16	pfam03175	DNA polymerase type B, organellar and vi
407464685	hypothetical protein NSED_04095	No	0	680977	681294	-		Candidatus_Nitrosopumilus_AR2_uid176130	NC_018656	chromosome									
407464686	hypothetical protein NSED_04100	No	0	681327	681668	-		Candidatus_Nitrosopumilus_AR2_uid176130	NC_018656	chromosome									
407464687	hypothetical protein NSED_04105	No	0	681652	681882	-		Candidatus_Nitrosopumilus_AR2_uid176130	NC_018656	chromosome	clust_416								
407464688	hypothetical protein NSED_04110	No	0	681936	682472	+		Candidatus_Nitrosopumilus_AR2_uid176130	NC_018656	chromosome									
407464689	hypothetical protein NSED_04115	No	0	682515	682781	+		Candidatus_Nitrosopumilus_AR2_uid176130	NC_018656	chromosome	clust_378								
407464690	hypothetical protein NSED_04120	Yes	2	682778	683371	-		Candidatus_Nitrosopumilus_AR2_uid176130	NC_018656	chromosome									
407464691	hypothetical protein NSED_04125	No	2	683439	683822	+		Candidatus_Nitrosopumilus_AR2_uid176130	NC_018656	chromosome									
407464692	hypothetical protein NSED_04130	No	0	683876	684460	+		Candidatus_Nitrosopumilus_AR2_uid176130	NC_018656	chromosome									
407464693	CRISPR-associated Cas1 family protein	No	0	684468	685481	-		Candidatus_Nitrosopumilus_AR2_uid176130	NC_018656	chromosome		arCOG01452	5 (-)	V	Cas1	CRISPR-associated protein Cas1	2E-25	pfam01867	CRISPR associated protein Cas1. Clustere
407464694	hypothetical protein NSED_04140	No	5	685482	685976	-		Candidatus_Nitrosopumilus_AR2_uid176130	NC_018656	chromosome									
407464695	hypothetical protein NSED_04145	No	0	686563	686976	+		Candidatus_Nitrosopumilus_AR2_uid176130	NC_018656	chromosome		arCOG08754	3 (-)	S		Uncharacterized conserved protein			
407464696	hypothetical protein NSED_04150	No	0	687514	688095	+		Candidatus_Nitrosopumilus_AR2_uid176130	NC_018656	chromosome		arCOG10540	4	S		Uncharacterized conserved protein			
407464697	hypothetical protein NSED_04155	No	0	688167	688367	+		Candidatus_Nitrosopumilus_AR2_uid176130	NC_018656	chromosome		arCOG10541	3	S		Uncharacterized conserved protein			
407464698	hypothetical protein NSED_04160	No	0	688405	688635	+		Candidatus_Nitrosopumilus_AR2_uid176130	NC_018656	chromosome		arCOG10542	3	S		Uncharacterized conserved protein			
407464699	hypothetical protein NSED_04165	No	0	688638	689063	-		Candidatus_Nitrosopumilus_AR2_uid176130	NC_018656	chromosome		arCOG10543	3	S		Uncharacterized conserved protein	0.00005	COG4477	Negative regulator of septation ring for
407464700	hypothetical protein NSED_04170	No	0	689164	689355	+		Candidatus_Nitrosopumilus_AR2_uid176130	NC_018656	chromosome									
407464701	hypothetical protein NSED_04175	No	2	689352	689576	-		Candidatus_Nitrosopumilus_AR2_uid176130	NC_018656	chromosome		arCOG10544	3	S		Uncharacterized conserved protein			
407464702	hypothetical protein NSED_04180	Yes	1	689698	690507	+		Candidatus_Nitrosopumilus_AR2_uid176130	NC_018656	chromosome		arCOG02926	8 (-)	C	PetE	Plastocyanin	0.000000005	COG3794	Plastocyanin
===							6	Candidatus_Nitrosopumilus_AR2_uid176130	NC_018656	chromosome									
407464705	hypothetical protein NSED_04195	No	0	692811	693188	+		Candidatus_Nitrosopumilus_AR2_uid176130	NC_018656	chromosome		arCOG05565	3 (-)	R		Roadblock/LC7 domain superfamily protein			
407464706	hypothetical protein NSED_04200	No	0	693217	693606	+		Candidatus_Nitrosopumilus_AR2_uid176130	NC_018656	chromosome	clust_42								
407464707	hypothetical protein NSED_04205	No	0	693569	693775	-		Candidatus_Nitrosopumilus_AR2_uid176130	NC_018656	chromosome	clust_446								
407464708	hypothetical protein NSED_04210	No	0	693759	693995	-		Candidatus_Nitrosopumilus_AR2_uid176130	NC_018656	chromosome	clust_59								
407464709	hypothetical protein NSED_04215	No	2	694130	694369	-		Candidatus_Nitrosopumilus_AR2_uid176130	NC_018656	chromosome	clust_388								
407464710	hypothetical protein NSED_04220	No	0	694474	694869	+		Candidatus_Nitrosopumilus_AR2_uid176130	NC_018656	chromosome		arCOG08684	7 (-)	R		Roadblock/LC7 domain superfamily protein			
===							7	Candidatus_Nitrosopumilus_AR2_uid176130	NC_018656	chromosome									
407464739	pyridoxamine 5'-phosphate oxidase-like FMN-	No	0	715659	716063	+		Candidatus_Nitrosopumilus_AR2_uid176130	NC_018656	chromosome		arCOG00516	6 (-)	R		Predicted flavin-nucleotide-binding protein			
407464740	hypothetical protein NSED_04370	No	0	716145	716543	-		Candidatus_Nitrosopumilus_AR2_uid176130	NC_018656	chromosome	clust_41						2E-16	COG3064	Membrane protein involved in colicin upt
407464741	hypothetical protein NSED_04375	No	1	716646	717131	-		Candidatus_Nitrosopumilus_AR2_uid176130	NC_018656	chromosome		arCOG10550	3	S		Uncharacterized conserved protein	0.000004	pfam11611	Domain of unknown function (DUF4352). Me
407464742	hypothetical protein NSED_04380	No	2	717234	717986	+		Candidatus_Nitrosopumilus_AR2_uid176130	NC_018656	chromosome									
407464743	hypothetical protein NSED_04385	No	1	718834	719085	+		Candidatus_Nitrosopumilus_AR2_uid176130	NC_018656	chromosome									
407464744	hypothetical protein NSED_04390	No	0	719071	719520	-		Candidatus_Nitrosopumilus_AR2_uid176130	NC_018656	chromosome		arCOG01713	2 (-)	S		Uncharacterized conserved protein			
407464745	hypothetical protein NSED_04395	No	2	719648	719875	-		Candidatus_Nitrosopumilus_AR2_uid176130	NC_018656	chromosome	clust_426								
===							10	Candidatus_Nitrosopumilus_AR2_uid176130	NC_018656	chromosome									
407464864	hypothetical protein NSED_05000	No	1	819330	819569	+		Candidatus_Nitrosopumilus_AR2_uid176130	NC_018656	chromosome	clust_58								
407464865	hypothetical protein NSED_05005	No	0	819908	820174	+		Candidatus_Nitrosopumilus_AR2_uid176130	NC_018656	chromosome		arCOG10560	3	S		Uncharacterized conserved protein			
407464866	hypothetical protein NSED_05010	No	0	820367	820903	-		Candidatus_Nitrosopumilus_AR2_uid176130	NC_018656	chromosome		arCOG10561	3	S		Uncharacterized conserved protein			
407464867	hypothetical protein NSED_05015	No	1	821327	822229	-		Candidatus_Nitrosopumilus_AR2_uid176130	NC_018656	chromosome		arCOG04994	2 (-)	O		Predicted Zn-dependent protease			
407464868	hypothetical protein NSED_05020	No	0	822443	822709	-		Candidatus_Nitrosopumilus_AR2_uid176130	NC_018656	chromosome		arCOG10562	3	S		Uncharacterized conserved protein			
407464869	hypothetical protein NSED_05025	No	0	822763	822969	+		Candidatus_Nitrosopumilus_AR2_uid176130	NC_018656	chromosome	clust_447								
407464870	hypothetical protein NSED_05030	No	0	823011	823598	+		Candidatus_Nitrosopumilus_AR2_uid176130	NC_018656	chromosome		arCOG10563	3	S		Uncharacterized conserved protein			
407464871	hypothetical protein NSED_05035	No	0	823585	823977	+		Candidatus_Nitrosopumilus_AR2_uid176130	NC_018656	chromosome		arCOG10564	3	S		Uncharacterized conserved protein			
407464872	hypothetical protein NSED_05040	No	0	824668	824916	+		Candidatus_Nitrosopumilus_AR2_uid176130	NC_018656	chromosome		arCOG07442	3 (-)	S		Uncharacterized conserved protein			
407464873	hypothetical protein NSED_05045	No	0	825089	825556	+		Candidatus_Nitrosopumilus_AR2_uid176130	NC_018656	chromosome		arCOG10565	3	S		Uncharacterized conserved protein			
===							5	Candidatus_Nitrosopumilus_AR2_uid176130	NC_018656	chromosome									
407465033	hypothetical protein NSED_05855	No	2	972052	972249	+		Candidatus_Nitrosopumilus_AR2_uid176130	NC_018656	chromosome									
407465034	hypothetical protein NSED_05860	No	0	972656	972955	+		Candidatus_Nitrosopumilus_AR2_uid176130	NC_018656	chromosome		arCOG10582	2	S		Uncharacterized conserved protein			
407465035	iron permease FTR1	Yes	1	973035	973517	-		Candidatus_Nitrosopumilus_AR2_uid176130	NC_018656	chromosome		arCOG08716	1 (-)	S		Uncharacterized conserved protein			
407465036	hypothetical protein NSED_05870	No	0	973645	974103	-		Candidatus_Nitrosopumilus_AR2_uid176130	NC_018656	chromosome							0.00003	pfam02463	RecF/RecN/SMC N terminal domain. This do
407465037	hypothetical protein NSED_05875	Yes	1	974165	976432	-		Candidatus_Nitrosopumilus_AR2_uid176130	NC_018656	chromosome									
===							15	Candidatus_Nitrosopumilus_AR2_uid176130	NC_018656	chromosome									
407465048	transcriptional regulator	No	0	986189	986518	+		Candidatus_Nitrosopumilus_AR2_uid176130	NC_018656	chromosome		arCOG03067	3 (-)	K	ArsR	Transcriptional regulator containing HTH domain, ArsR family			
407465049	hypothetical protein NSED_05935	Yes	2	986764	987030	-		Candidatus_Nitrosopumilus_AR2_uid176130	NC_018656	chromosome	clust_379								
407465050	hypothetical protein NSED_05940	No	1	987034	987714	-		Candidatus_Nitrosopumilus_AR2_uid176130	NC_018656	chromosome	clust_150								
407465051	hypothetical protein NSED_05945	No	2	987711	987953	-		Candidatus_Nitrosopumilus_AR2_uid176130	NC_018656	chromosome	clust_55								
407465052	hypothetical protein NSED_05950	Yes	1	987935	988411	-		Candidatus_Nitrosopumilus_AR2_uid176130	NC_018656	chromosome									
407465053	hypothetical protein NSED_05955	Yes	1	988448	989770	-		Candidatus_Nitrosopumilus_AR2_uid176130	NC_018656	chromosome	clust_75								
407465054	hypothetical protein NSED_05960	No	0	989845	990465	-		Candidatus_Nitrosopumilus_AR2_uid176130	NC_018656	chromosome									
407465055	hypothetical protein NSED_05965	No	0	990459	991100	-		Candidatus_Nitrosopumilus_AR2_uid176130	NC_018656	chromosome									
407465056	hypothetical protein NSED_05970	No	0	991280	993088	-		Candidatus_Nitrosopumilus_AR2_uid176130	NC_018656	chromosome									
407465057	hypothetical protein NSED_05975	No	0	993092	993880	-		Candidatus_Nitrosopumilus_AR2_uid176130	NC_018656	chromosome									
407465058	hypothetical protein NSED_05980	No	0	993868	994083	-		Candidatus_Nitrosopumilus_AR2_uid176130	NC_018656	chromosome									
407465059	RelA/SpoT domain-containing protein	No	0	994121	995107	-		Candidatus_Nitrosopumilus_AR2_uid176130	NC_018656	chromosome		arCOG10830	3	S		Uncharacterized conserved protein	8E-11	COG2357	PpGpp synthetase catalytic domain
407465060	hypothetical protein NSED_05990	No	0	995590	996441	-		Candidatus_Nitrosopumilus_AR2_uid176130	NC_018656	chromosome									
407465061	hypothetical protein NSED_05995	No	0	996814	997083	+		Candidatus_Nitrosopumilus_AR2_uid176130	NC_018656	chromosome									
407465062	integrase family protein	No	0	997929	999116	-		Candidatus_Nitrosopumilus_AR2_uid176130	NC_018656	chromosome		arCOG01245	15 (-)	L	XerC	Integrase			
===							15	Candidatus_Nitrosopumilus_AR2_uid176130	NC_018656	chromosome									
407465194	multicopper oxidase type 3	Yes	1	1118843	1120192	-		Candidatus_Nitrosopumilus_AR2_uid176130	NC_018656	chromosome		arCOG02926	8 (-)	C	PetE	Plastocyanin	5E-14	pfam07732	Multicopper oxidase. This entry contains ; Plastocyanin
407465195	hypothetical protein NSED_06685	No	0	1120863	1122566	-		Candidatus_Nitrosopumilus_AR2_uid176130	NC_018656	chromosome									
407465196	hypothetical protein NSED_06690	No	0	1122553	1122891	-		Candidatus_Nitrosopumilus_AR2_uid176130	NC_018656	chromosome									
407465197	hypothetical protein NSED_06695	No	1	1122936	1123340	-		Candidatus_Nitrosopumilus_AR2_uid176130	NC_018656	chromosome									
407465198	hypothetical protein NSED_06700	No	0	1123381	1125270	-		Candidatus_Nitrosopumilus_AR2_uid176130	NC_018656	chromosome									
407465199	hypothetical protein NSED_06705	No	0	1125403	1125603	-		Candidatus_Nitrosopumilus_AR2_uid176130	NC_018656	chromosome									
407465200	hypothetical protein NSED_06710	No	0	1125909	1126052	-		Candidatus_Nitrosopumilus_AR2_uid176130	NC_018656	chromosome									
407465201	hypothetical protein NSED_06715	No	0	1126132	1126494	+		Candidatus_Nitrosopumilus_AR2_uid176130	NC_018656	chromosome									
407465202	hypothetical protein NSED_06720	No	0	1126608	1128407	+		Candidatus_Nitrosopumilus_AR2_uid176130	NC_018656	chromosome									
407465203	hypothetical protein NSED_06725	No	0	1128474	1128710	+		Candidatus_Nitrosopumilus_AR2_uid176130	NC_018656	chromosome									
407465204	hypothetical protein NSED_06730	No	2	1128750	1129259	+		Candidatus_Nitrosopumilus_AR2_uid176130	NC_018656	chromosome									
407465205	hypothetical protein NSED_06735	No	0	1129256	1129612	-		Candidatus_Nitrosopumilus_AR2_uid176130	NC_018656	chromosome									
407465206	hypothetical protein NSED_06740	No	0	1129614	1130384	-		Candidatus_Nitrosopumilus_AR2_uid176130	NC_018656	chromosome									
407465207	hypothetical protein NSED_06745	No	0	1130381	1131262	-		Candidatus_Nitrosopumilus_AR2_uid176130	NC_018656	chromosome									
407465208	hypothetical protein NSED_06750	No	0	1131456	1132973	-		Candidatus_Nitrosopumilus_AR2_uid176130	NC_018656	chromosome		arCOG01245	15 (-)	L	XerC	Integrase			
===							6	Candidatus_Nitrosopumilus_AR2_uid176130	NC_018656	chromosome									
407465435	hypothetical protein NSED_07915	No	0	1327404	1327937	+		Candidatus_Nitrosopumilus_AR2_uid176130	NC_018656	chromosome		arCOG05252	3 (-)	F		Predicted secreted endonuclease distantly related to archaeal Holliday junction resolvase			
407465436	hypothetical protein NSED_07920	No	0	1328082	1328348	+		Candidatus_Nitrosopumilus_AR2_uid176130	NC_018656	chromosome									
407465437	hypothetical protein NSED_07925	No	0	1328434	1330464	+		Candidatus_Nitrosopumilus_AR2_uid176130	NC_018656	chromosome									
407465438	hypothetical protein NSED_07930	No	0	1330464	1331135	+		Candidatus_Nitrosopumilus_AR2_uid176130	NC_018656	chromosome									
407465439	hypothetical protein NSED_07935	No	0	1331140	1331433	-		Candidatus_Nitrosopumilus_AR2_uid176130	NC_018656	chromosome									
407465440	modification methylase	No	0	1331433	1332422	-		Candidatus_Nitrosopumilus_AR2_uid176130	NC_018656	chromosome		arCOG04157	7 (-)	L	Dcm	Site-specific DNA methylase	2E-74	pfam00145	C-5 cytosine-specific DNA methylase.
===							5	Candidatus_Nitrosopumilus_AR2_uid176130	NC_018656	chromosome									
407465523	hypothetical protein NSED_08355	No	0	1398277	1398498	+		Candidatus_Nitrosopumilus_AR2_uid176130	NC_018656	chromosome		arCOG10588	1	S		Uncharacterized conserved protein			
407465524	hypothetical protein NSED_08360	No	0	1398570	1399157	+		Candidatus_Nitrosopumilus_AR2_uid176130	NC_018656	chromosome		arCOG10589	1	S		Uncharacterized conserved protein			
407465525	hypothetical protein NSED_08365	Yes	0	1399147	1399506	-		Candidatus_Nitrosopumilus_AR2_uid176130	NC_018656	chromosome		arCOG08643	2 (-)	S		Uncharacterized conserved protein			
407465526	hypothetical protein NSED_08370	No	0	1399463	1399843	-		Candidatus_Nitrosopumilus_AR2_uid176130	NC_018656	chromosome									
407465527	hypothetical protein NSED_08375	No	0	1399890	1400174	-		Candidatus_Nitrosopumilus_AR2_uid176130	NC_018656	chromosome									
===							5	Candidatus_Nitrosopumilus_koreensis_AR1_uid1	NC_018655	chromosome									
407461658	hypothetical protein NKOR_00575	No	0	110978	112753	-		Candidatus_Nitrosopumilus_koreensis_AR1_uid1	NC_018655	chromosome		arCOG09550	17	Y	TerL	Phage terminase large subunit			
407461659	hypothetical protein NKOR_00580	No	0	112740	113210	-		Candidatus_Nitrosopumilus_koreensis_AR1_uid1	NC_018655	chromosome									
407461660	hypothetical protein NKOR_00585	No	1	113760	114824	+		Candidatus_Nitrosopumilus_koreensis_AR1_uid1	NC_018655	chromosome		arCOG03788	1 (-)	V		LEA14-like dessication related protein			
407461661	hypothetical protein NKOR_00590	No	2	114805	115134	+		Candidatus_Nitrosopumilus_koreensis_AR1_uid1	NC_018655	chromosome									
407461662	hypothetical protein NKOR_00595	No	0	115187	116041	-		Candidatus_Nitrosopumilus_koreensis_AR1_uid1	NC_018655	chromosome									
===							11	Candidatus_Nitrosopumilus_koreensis_AR1_uid1	NC_018655	chromosome									
407461666	hypothetical protein NKOR_00615	No	0	117474	118001	+		Candidatus_Nitrosopumilus_koreensis_AR1_uid1	NC_018655	chromosome		arCOG05252	3 (-)	F		Predicted secreted endonuclease distantly related to archaeal Holliday junction resolvase			
407461667	hypothetical protein NKOR_00620	No	0	118086	118415	+		Candidatus_Nitrosopumilus_koreensis_AR1_uid1	NC_018655	chromosome									
407461668	hypothetical protein NKOR_00625	No	0	118497	119156	+		Candidatus_Nitrosopumilus_koreensis_AR1_uid1	NC_018655	chromosome									
407461669	hypothetical protein NKOR_00630	No	0	119149	121383	+		Candidatus_Nitrosopumilus_koreensis_AR1_uid1	NC_018655	chromosome									
407461670	hypothetical protein NKOR_00635	No	0	121401	121802	+		Candidatus_Nitrosopumilus_koreensis_AR1_uid1	NC_018655	chromosome									
407461671	hypothetical protein NKOR_00640	No	4	121910	122386	-		Candidatus_Nitrosopumilus_koreensis_AR1_uid1	NC_018655	chromosome									
407461672	hypothetical protein NKOR_00645	No	2	122468	122896	-		Candidatus_Nitrosopumilus_koreensis_AR1_uid1	NC_018655	chromosome									
407461673	hypothetical protein NKOR_00650	No	0	122993	123781	-		Candidatus_Nitrosopumilus_koreensis_AR1_uid1	NC_018655	chromosome									
407461674	hypothetical protein NKOR_00655	No	0	123872	124468	-		Candidatus_Nitrosopumilus_koreensis_AR1_uid1	NC_018655	chromosome									
407461675	hypothetical protein NKOR_00660	No	0	124584	125801	-		Candidatus_Nitrosopumilus_koreensis_AR1_uid1	NC_018655	chromosome		arCOG01245	15 (-)	L	XerC	Integrase			
407461676	hypothetical protein NKOR_00665	No	0	126234	126473	-		Candidatus_Nitrosopumilus_koreensis_AR1_uid1	NC_018655	chromosome		arCOG10516	1	S		Uncharacterized conserved protein			
===							11	Candidatus_Nitrosopumilus_koreensis_AR1_uid1	NC_018655	chromosome									
407461861	nitroreductase	No	0	289374	289976	+		Candidatus_Nitrosopumilus_koreensis_AR1_uid1	NC_018655	chromosome		arCOG00288	8 (-)	C	NfnB	Nitroreductase	2E-23	COG0778	Nitroreductase
407461862	hypothetical protein NKOR_01655	No	1	290136	290381	+		Candidatus_Nitrosopumilus_koreensis_AR1_uid1	NC_018655	chromosome									
407461863	hypothetical protein NKOR_01660	No	0	290417	290614	+		Candidatus_Nitrosopumilus_koreensis_AR1_uid1	NC_018655	chromosome									
407461864	hypothetical protein NKOR_01665	No	0	290654	290887	+		Candidatus_Nitrosopumilus_koreensis_AR1_uid1	NC_018655	chromosome									
407461865	hypothetical protein NKOR_01670	No	0	291275	291676	+		Candidatus_Nitrosopumilus_koreensis_AR1_uid1	NC_018655	chromosome									
407461866	DEAD/DEAH box helicase	No	0	291673	293916	+		Candidatus_Nitrosopumilus_koreensis_AR1_uid1	NC_018655	chromosome		arCOG00555	5 (-)	R		Distinct helicase family with a unique C-terminal d	9E-52	COG1205	Distinct helicase family with a unique C
407461867	hypothetical protein NKOR_01680	No	0	294547	294756	+		Candidatus_Nitrosopumilus_koreensis_AR1_uid1	NC_018655	chromosome									
407461868	hypothetical protein NKOR_01685	No	0	294824	295015	+		Candidatus_Nitrosopumilus_koreensis_AR1_uid1	NC_018655	chromosome									
407461869	hypothetical protein NKOR_01690	No	0	295058	295255	+		Candidatus_Nitrosopumilus_koreensis_AR1_uid1	NC_018655	chromosome	clust_456								
407461870	hypothetical protein NKOR_01695	No	0	295245	295433	-		Candidatus_Nitrosopumilus_koreensis_AR1_uid1	NC_018655	chromosome	clust_468								
407461871	FAD-dependent pyridine nucleotide-disulfide c	No	0	295543	295836	+		Candidatus_Nitrosopumilus_koreensis_AR1_uid1	NC_018655	chromosome		arCOG02021	2 (-)	P	PspE	Rhodanese-related sulfurtransferase	0.000000002	pfam00581	Rhodanese-like domain. Rhodanese has an
===							8	Candidatus_Nitrosopumilus_koreensis_AR1_uid1	NC_018655	chromosome									
407461989	hypothetical protein NKOR_02320	No	0	403317	403472	-		Candidatus_Nitrosopumilus_koreensis_AR1_uid1	NC_018655	chromosome									
407461990	hypothetical protein NKOR_02335	No	0	404271	405203	-		Candidatus_Nitrosopumilus_koreensis_AR1_uid1	NC_018655	chromosome		arCOG08787	3 (-)	S		Uncharacterized conserved protein			
407461991	hypothetical protein NKOR_02340	No	2	405250	406368	+		Candidatus_Nitrosopumilus_koreensis_AR1_uid1	NC_018655	chromosome									
407461992	hypothetical protein NKOR_02345	No	0	406949	407146	-		Candidatus_Nitrosopumilus_koreensis_AR1_uid1	NC_018655	chromosome									
407461993	hypothetical protein NKOR_02350	No	0	407187	409646	-		Candidatus_Nitrosopumilus_koreensis_AR1_uid1	NC_018655	chromosome							9E-35	pfam12770	CHAT domain. These proteins appear to be
407461994	hypothetical protein NKOR_02355	No	0	409643	410530	-		Candidatus_Nitrosopumilus_koreensis_AR1_uid1	NC_018655	chromosome									
407461995	hypothetical protein NKOR_02360	No	0	410927	411208	-		Candidatus_Nitrosopumilus_koreensis_AR1_uid1	NC_018655	chromosome									
407461996	arsR family transcriptional regulator	No	0	411280	412071	+		Candidatus_Nitrosopumilus_koreensis_AR1_uid1	NC_018655	chromosome		arCOG04362	5 (-)	K		Predicted transcriptional regulator	0.000000001	COG4742	Predicted transcriptional regulator
===							6	Candidatus_Nitrosopumilus_koreensis_AR1_uid1	NC_018655	chromosome									
407461999	hypothetical protein NKOR_02380	Yes	1	416556	417980	+		Candidatus_Nitrosopumilus_koreensis_AR1_uid1	NC_018655	chromosome		arCOG07561	7 (-)	K		Predicted regulatory protein containing HTH domain			
407462000	hypothetical protein NKOR_02385	No	0	418271	418597	-		Candidatus_Nitrosopumilus_koreensis_AR1_uid1	NC_018655	chromosome									
407462001	hypothetical protein NKOR_02390	No	0	418917	419519	+		Candidatus_Nitrosopumilus_koreensis_AR1_uid1	NC_018655	chromosome									
407462002	hypothetical protein NKOR_02395	No	0	419866	420459	+		Candidatus_Nitrosopumilus_koreensis_AR1_uid1	NC_018655	chromosome									
407462003	hypothetical protein NKOR_02400	No	0	420462	420965	+		Candidatus_Nitrosopumilus_koreensis_AR1_uid1	NC_018655	chromosome									
407462004	integrase family protein	No	0	421888	423228	-		Candidatus_Nitrosopumilus_koreensis_AR1_uid1	NC_018655	chromosome		arCOG01242	14 (-)	L	XerC	Integrase			
===							5	Candidatus_Nitrosopumilus_koreensis_AR1_uid1	NC_018655	chromosome									
407462100	hypothetical protein NKOR_02905	No	0	499189	499611	+		Candidatus_Nitrosopumilus_koreensis_AR1_uid1	NC_018655	chromosome									
407462101	hypothetical protein NKOR_02910	No	0	499608	500426	+		Candidatus_Nitrosopumilus_koreensis_AR1_uid1	NC_018655	chromosome									
407462102	NADPH-dependent FMN reductase	No	0	501120	501695	+		Candidatus_Nitrosopumilus_koreensis_AR1_uid1	NC_018655	chromosome		arCOG04624	1 (-)	R		Multimeric flavodoxin WrbA	9E-30	pfam03358	NADPH-dependent FMN reductase.
407462103	hypothetical protein NKOR_02920	No	1	502552	502761	-		Candidatus_Nitrosopumilus_koreensis_AR1_uid1	NC_018655	chromosome									
407462104	hypothetical protein NKOR_02925	No	0	502762	502962	+		Candidatus_Nitrosopumilus_koreensis_AR1_uid1	NC_018655	chromosome									
===							6	Candidatus_Nitrosopumilus_koreensis_AR1_uid1	NC_018655	chromosome									
407462256	binding-protein-dependent transport system in	No	6	630137	631186	-		Candidatus_Nitrosopumilus_koreensis_AR1_uid1	NC_018655	chromosome		arCOG00751	1 (-)	E	DppB	ABC-type dipeptide/oligopeptide/nickel transpor	5E-51	COG0601	ABC-type dipeptide/oligopeptide/nickel t
407462257	hypothetical protein NKOR_03690	No	0	631387	631686	+		Candidatus_Nitrosopumilus_koreensis_AR1_uid1	NC_018655	chromosome		arCOG08789	1	S		Uncharacterized conserved protein			
407462258	hypothetical protein NKOR_03695	No	0	631706	631948	+		Candidatus_Nitrosopumilus_koreensis_AR1_uid1	NC_018655	chromosome		arCOG10537	1	S		Uncharacterized conserved protein			
407462259	arsenate reductase-like protein	No	0	631951	632286	-		Candidatus_Nitrosopumilus_koreensis_AR1_uid1	NC_018655	chromosome							1E-16	COG1393	Arsenate reductase and related proteins,
407462260	hypothetical protein NKOR_03705	No	0	632311	632508	-		Candidatus_Nitrosopumilus_koreensis_AR1_uid1	NC_018655	chromosome									
407462261	hypothetical protein NKOR_03710	Yes	0	632554	633504	-		Candidatus_Nitrosopumilus_koreensis_AR1_uid1	NC_018655	chromosome		arCOG08796	2 (-)	S		Uncharacterized conserved protein			
===							12	Candidatus_Nitrosopumilus_koreensis_AR1_uid1	NC_018655	chromosome									
407462287	hypothetical protein NKOR_03860	No	5	655435	655935	+		Candidatus_Nitrosopumilus_koreensis_AR1_uid1	NC_018655	chromosome									
407462288	CRISPR-associated Cas1 family protein	No	0	655943	656986	+		Candidatus_Nitrosopumilus_koreensis_AR1_uid1	NC_018655	chromosome		arCOG01452	5 (-)	V	Cas1	CRISPR-associated protein Cas1	1E-23	pfam01867	CRISPR associated protein Cas1. Clustere
407462289	hypothetical protein NKOR_03870	No	4	656958	657359	-		Candidatus_Nitrosopumilus_koreensis_AR1_uid1	NC_018655	chromosome									
407462290	hypothetical protein NKOR_03875	No	0	657403	657597	-		Candidatus_Nitrosopumilus_koreensis_AR1_uid1	NC_018655	chromosome									
407462291	hypothetical protein NKOR_03880	No	1	657644	657940	-		Candidatus_Nitrosopumilus_koreensis_AR1_uid1	NC_018655	chromosome									
407462292	hypothetical protein NKOR_03885	Yes	2	658011	658808	+		Candidatus_Nitrosopumilus_koreensis_AR1_uid1	NC_018655	chromosome									
407462293	hypothetical protein NKOR_03890	No	0	658805	659071	-		Candidatus_Nitrosopumilus_koreensis_AR1_uid1	NC_018655	chromosome	clust_378								
407462294	hypothetical protein NKOR_03895	No	0	659116	659730	-		Candidatus_Nitrosopumilus_koreensis_AR1_uid1	NC_018655	chromosome		arCOG10540	4	S		Uncharacterized conserved protein			
407462295	hypothetical protein NKOR_03900	No	0	659786	660019	+		Candidatus_Nitrosopumilus_koreensis_AR1_uid1	NC_018655	chromosome	clust_416								
407462296	hypothetical protein NKOR_03905	No	0	660003	660194	+		Candidatus_Nitrosopumilus_koreensis_AR1_uid1	NC_018655	chromosome									
407462297	hypothetical protein NKOR_03910	No	0	660232	660492	+		Candidatus_Nitrosopumilus_koreensis_AR1_uid1	NC_018655	chromosome									
407462298	type B DNA polymerase	No	0	660674	662458	+		Candidatus_Nitrosopumilus_koreensis_AR1_uid1	NC_018655	chromosome		arCOG00328	8 (-)	L	PolB	DNA polymerase elongation subunit (family B)	1E-12	pfam03175	DNA polymerase type B, organellar and vi
===							6	Candidatus_Nitrosopumilus_koreensis_AR1_uid1	NC_018655	chromosome									
407462304	UbiE/COQ5 methyltransferase	No	0	666326	667003	-		Candidatus_Nitrosopumilus_koreensis_AR1_uid1	NC_018655	chromosome		arCOG04347	4 (-)	Q		SAM-dependent methyltransferase	2E-20	COG2226	Methylase involved in ubiquinone/menaqui
407462305	hypothetical protein NKOR_03950	No	2	667038	667247	-		Candidatus_Nitrosopumilus_koreensis_AR1_uid1	NC_018655	chromosome									
407462306	hypothetical protein NKOR_03955	Yes	0	667548	668624	-		Candidatus_Nitrosopumilus_koreensis_AR1_uid1	NC_018655	chromosome							0.000001	pfam09731	Mitochondrial inner membrane protein. Mi
407462307	hypothetical protein NKOR_03960	Yes	0	668699	668920	-		Candidatus_Nitrosopumilus_koreensis_AR1_uid1	NC_018655	chromosome									
407462308	hypothetical protein NKOR_03965	No	1	669213	669524	+		Candidatus_Nitrosopumilus_koreensis_AR1_uid1	NC_018655	chromosome									
407462309	hypothetical protein NKOR_03970	No	3	669560	669970	-		Candidatus_Nitrosopumilus_koreensis_AR1_uid1	NC_018655	chromosome		arCOG09752	1 (-)	S		Predicted membrane protein, DUF2068			
===							9	Candidatus_Nitrosopumilus_koreensis_AR1_uid1	NC_018655	chromosome									
407462314	hypothetical protein NKOR_03995	No	0	673624	674016	-		Candidatus_Nitrosopumilus_koreensis_AR1_uid1	NC_018655	chromosome		arCOG05565	3 (-)	R		Roadblock/LC7 domain superfamily protein			
407462315	opacity protein	No	0	674356	674574	+		Candidatus_Nitrosopumilus_koreensis_AR1_uid1	NC_018655	chromosome									
407462316	hypothetical protein NKOR_04005	No	0	675436	675618	+		Candidatus_Nitrosopumilus_koreensis_AR1_uid1	NC_018655	chromosome									
407462317	hypothetical protein NKOR_04010	No	0	675708	676121	-		Candidatus_Nitrosopumilus_koreensis_AR1_uid1	NC_018655	chromosome		arCOG10594	1	S		Uncharacterized conserved protein			
407462318	hypothetical protein NKOR_04015	No	0	676228	676515	+		Candidatus_Nitrosopumilus_koreensis_AR1_uid1	NC_018655	chromosome									
407462319	hypothetical protein NKOR_04020	No	0	676522	676734	+		Candidatus_Nitrosopumilus_koreensis_AR1_uid1	NC_018655	chromosome									
407462320	ATPase AAA	No	0	677124	679259	+		Candidatus_Nitrosopumilus_koreensis_AR1_uid1	NC_018655	chromosome		arCOG01308	10 (-)	O	Cdc48	ATPase of the AAA+ class , CDC48 family	5E-173	COG0464	ATPases of the AAA+ class
407462321	hypothetical protein NKOR_04030	No	0	679406	679570	+		Candidatus_Nitrosopumilus_koreensis_AR1_uid1	NC_018655	chromosome									
407462322	hypothetical protein NKOR_04035	No	2	680015	680275	-		Candidatus_Nitrosopumilus_koreensis_AR1_uid1	NC_018655	chromosome	clust_388								
===							7	Candidatus_Nitrosopumilus_koreensis_AR1_uid1	NC_018655	chromosome									
407462334	hypothetical protein NKOR_04095	Yes	1	690935	691906	+		Candidatus_Nitrosopumilus_koreensis_AR1_uid1	NC_018655	chromosome		arCOG02562	2 (-)	S		Beta-propeller repeat containing protein	1E-31	COG3391	Uncharacterized conserved protein
407462335	hypothetical protein NKOR_04100	No	0	692230	692811	+		Candidatus_Nitrosopumilus_koreensis_AR1_uid1	NC_018655	chromosome		arCOG10540	4	S		Uncharacterized conserved protein			
407462336	hypothetical protein NKOR_04105	No	0	692843	693073	+		Candidatus_Nitrosopumilus_koreensis_AR1_uid1	NC_018655	chromosome		arCOG10541	3	S		Uncharacterized conserved protein			
407462337	hypothetical protein NKOR_04110	No	0	693106	693336	+		Candidatus_Nitrosopumilus_koreensis_AR1_uid1	NC_018655	chromosome		arCOG10542	3	S		Uncharacterized conserved protein			
407462338	hypothetical protein NKOR_04115	No	0	693339	693764	-		Candidatus_Nitrosopumilus_koreensis_AR1_uid1	NC_018655	chromosome		arCOG10543	3	S		Uncharacterized conserved protein			
407462339	hypothetical protein NKOR_04120	No	2	693838	694062	-		Candidatus_Nitrosopumilus_koreensis_AR1_uid1	NC_018655	chromosome		arCOG10544	3	S		Uncharacterized conserved protein			
407462340	hypothetical protein NKOR_04125	No	0	694270	695622	+		Candidatus_Nitrosopumilus_koreensis_AR1_uid1	NC_018655	chromosome		arCOG00371	3 (-)	D	Smc	Chromosome segregation ATPase	3E-21	COG1196	Chromosome segregation ATPases
===							7	Candidatus_Nitrosopumilus_koreensis_AR1_uid1	NC_018655	chromosome									
407462343	hypothetical protein NKOR_04140	No	0	696732	697079	+		Candidatus_Nitrosopumilus_koreensis_AR1_uid1	NC_018655	chromosome	clust_42								
407462344	hypothetical protein NKOR_04145	No	0	697076	697282	-		Candidatus_Nitrosopumilus_koreensis_AR1_uid1	NC_018655	chromosome	clust_446								
407462345	hypothetical protein NKOR_04150	No	0	697284	697493	-		Candidatus_Nitrosopumilus_koreensis_AR1_uid1	NC_018655	chromosome	clust_59								
407462346	hypothetical protein NKOR_04155	No	0	697629	697904	+		Candidatus_Nitrosopumilus_koreensis_AR1_uid1	NC_018655	chromosome		arCOG04290	2 (-)	R		PIN-domain and Zn ribbon			
407462347	hypothetical protein NKOR_04160	No	0	697897	698463	-		Candidatus_Nitrosopumilus_koreensis_AR1_uid1	NC_018655	chromosome	clust_186								
407462348	hypothetical protein NKOR_04165	No	0	698540	698701	-		Candidatus_Nitrosopumilus_koreensis_AR1_uid1	NC_018655	chromosome		arCOG10545	2	S		Uncharacterized conserved protein			
407462349	hypothetical protein NKOR_04170	No	0	698780	699043	+		Candidatus_Nitrosopumilus_koreensis_AR1_uid1	NC_018655	chromosome		arCOG10546	2	S		Uncharacterized conserved protein			
===							7	Candidatus_Nitrosopumilus_koreensis_AR1_uid1	NC_018655	chromosome									
407462379	hypothetical protein NKOR_04330	No	0	722442	722762	+		Candidatus_Nitrosopumilus_koreensis_AR1_uid1	NC_018655	chromosome	clust_327								
407462380	pyridoxamine 5'-phosphate oxidase-like FMN-	No	0	722755	723159	+		Candidatus_Nitrosopumilus_koreensis_AR1_uid1	NC_018655	chromosome		arCOG00516	6 (-)	R		Predicted flavin-nucleotide-binding protein	0.00008	pfam01243	Pyridoxamine 5'-phosphate oxidase.
407462381	hypothetical protein NKOR_04340	No	0	723241	723612	-		Candidatus_Nitrosopumilus_koreensis_AR1_uid1	NC_018655	chromosome	clust_41						1E-15	COG3064	Membrane protein involved in colicin upt
407462382	hypothetical protein NKOR_04345	No	1	723715	724200	-		Candidatus_Nitrosopumilus_koreensis_AR1_uid1	NC_018655	chromosome		arCOG10550	3	S		Uncharacterized conserved protein			
407462383	hypothetical protein NKOR_04350	No	1	724455	725090	+		Candidatus_Nitrosopumilus_koreensis_AR1_uid1	NC_018655	chromosome									
407462384	hypothetical protein NKOR_04355	No	0	726251	726700	-		Candidatus_Nitrosopumilus_koreensis_AR1_uid1	NC_018655	chromosome		arCOG01713	2 (-)	S		Uncharacterized conserved protein			
407462385	hypothetical protein NKOR_04360	No	2	726816	727043	-		Candidatus_Nitrosopumilus_koreensis_AR1_uid1	NC_018655	chromosome	clust_426								
===							8	Candidatus_Nitrosopumilus_koreensis_AR1_uid1	NC_018655	chromosome									
407462433	hypothetical protein NKOR_04600	No	0	765782	766135	+		Candidatus_Nitrosopumilus_koreensis_AR1_uid1	NC_018655	chromosome		arCOG10552	1	S		Uncharacterized conserved protein			
407462434	hypothetical protein NKOR_04605	No	0	766438	766755	-		Candidatus_Nitrosopumilus_koreensis_AR1_uid1	NC_018655	chromosome		arCOG08697	1 (-)	S		Uncharacterized conserved protein			
407462435	hypothetical protein NKOR_04610	No	0	766921	767763	-		Candidatus_Nitrosopumilus_koreensis_AR1_uid1	NC_018655	chromosome									
407462436	hypothetical protein NKOR_04615	No	0	767687	767929	-		Candidatus_Nitrosopumilus_koreensis_AR1_uid1	NC_018655	chromosome									
407462437	hypothetical protein NKOR_04620	No	0	768125	768427	+		Candidatus_Nitrosopumilus_koreensis_AR1_uid1	NC_018655	chromosome									
407462438	hypothetical protein NKOR_04625	No	0	768829	769413	-		Candidatus_Nitrosopumilus_koreensis_AR1_uid1	NC_018655	chromosome									
407462439	resolvase, Holliday junction-type	No	0	769468	770022	-		Candidatus_Nitrosopumilus_koreensis_AR1_uid1	NC_018655	chromosome		arCOG00919	2 (-)	L		Holliday junction resolvase			
407462440	hypothetical protein NKOR_04635	No	0	770205	770405	+		Candidatus_Nitrosopumilus_koreensis_AR1_uid1	NC_018655	chromosome									
===							11	Candidatus_Nitrosopumilus_koreensis_AR1_uid1	NC_018655	chromosome									
407462513	hypothetical protein NKOR_05010	No	0	834663	834980	+		Candidatus_Nitrosopumilus_koreensis_AR1_uid1	NC_018655	chromosome		arCOG10559	2 (-)	L		GIY-YIG domain containing nuclease			
407462514	hypothetical protein NKOR_05015	No	1	835043	835288	+		Candidatus_Nitrosopumilus_koreensis_AR1_uid1	NC_018655	chromosome	clust_58								
407462515	hypothetical protein NKOR_05020	No	0	835599	835865	+		Candidatus_Nitrosopumilus_koreensis_AR1_uid1	NC_018655	chromosome		arCOG10560	3	S		Uncharacterized conserved protein			
407462516	hypothetical protein NKOR_05025	No	0	835893	836432	-		Candidatus_Nitrosopumilus_koreensis_AR1_uid1	NC_018655	chromosome		arCOG10561	3	S		Uncharacterized conserved protein			
407462517	hypothetical protein NKOR_05030	No	0	836474	836791	-		Candidatus_Nitrosopumilus_koreensis_AR1_uid1	NC_018655	chromosome									
407462518	hypothetical protein NKOR_05035	No	0	837323	837574	-		Candidatus_Nitrosopumilus_koreensis_AR1_uid1	NC_018655	chromosome		arCOG10562	3	S		Uncharacterized conserved protein			
407462519	hypothetical protein NKOR_05040	No	0	837632	837835	+		Candidatus_Nitrosopumilus_koreensis_AR1_uid1	NC_018655	chromosome	clust_447								
407462520	hypothetical protein NKOR_05045	No	0	837876	838466	+		Candidatus_Nitrosopumilus_koreensis_AR1_uid1	NC_018655	chromosome		arCOG10563	3	S		Uncharacterized conserved protein			
407462521	hypothetical protein NKOR_05050	No	0	838503	838838	+		Candidatus_Nitrosopumilus_koreensis_AR1_uid1	NC_018655	chromosome		arCOG10564	3	S		Uncharacterized conserved protein			
407462522	hypothetical protein NKOR_05055	No	0	839531	839764	+		Candidatus_Nitrosopumilus_koreensis_AR1_uid1	NC_018655	chromosome		arCOG07442	3 (-)	S		Uncharacterized conserved protein			
407462523	hypothetical protein NKOR_05060	No	0	839836	840303	+		Candidatus_Nitrosopumilus_koreensis_AR1_uid1	NC_018655	chromosome		arCOG10565	3	S		Uncharacterized conserved protein			
===							11	Candidatus_Nitrosopumilus_koreensis_AR1_uid1	NC_018655	chromosome									
407462527	hypothetical protein NKOR_05080	No	0	842348	842557	+		Candidatus_Nitrosopumilus_koreensis_AR1_uid1	NC_018655	chromosome	clust_441								
407462528	hypothetical protein NKOR_05085	No	0	842600	843073	+		Candidatus_Nitrosopumilus_koreensis_AR1_uid1	NC_018655	chromosome	clust_196								
407462529	hypothetical protein NKOR_05090	No	0	843163	843576	-		Candidatus_Nitrosopumilus_koreensis_AR1_uid1	NC_018655	chromosome		arCOG08754	3 (-)	S		Uncharacterized conserved protein			
407462530	hypothetical protein NKOR_05095	No	0	843683	843982	+		Candidatus_Nitrosopumilus_koreensis_AR1_uid1	NC_018655	chromosome	clust_352								
407462531	hypothetical protein NKOR_05100	No	0	844152	844346	+		Candidatus_Nitrosopumilus_koreensis_AR1_uid1	NC_018655	chromosome									
407462532	hypothetical protein NKOR_05105	No	0	844421	845074	-		Candidatus_Nitrosopumilus_koreensis_AR1_uid1	NC_018655	chromosome		arCOG00786	4 (-)	V	Cas4	CRISPR-associated protein Cas4, RecB family exonuclease			
407462533	hypothetical protein NKOR_05110	No	0	845128	845529	-		Candidatus_Nitrosopumilus_koreensis_AR1_uid1	NC_018655	chromosome	clust_265								
407462534	hypothetical protein NKOR_05115	No	0	845583	845780	+		Candidatus_Nitrosopumilus_koreensis_AR1_uid1	NC_018655	chromosome	clust_458								
407462535	hypothetical protein NKOR_05120	No	0	845985	846218	+		Candidatus_Nitrosopumilus_koreensis_AR1_uid1	NC_018655	chromosome									
407462536	hypothetical protein NKOR_05125	No	0	846232	846564	+		Candidatus_Nitrosopumilus_koreensis_AR1_uid1	NC_018655	chromosome	clust_314								
407462537	anthranilate synthase component I	No	0	846925	848274	+		Candidatus_Nitrosopumilus_koreensis_AR1_uid1	NC_018655	chromosome		arCOG02014	1 (-)	E	TrpE	Anthranilate/para-aminobenzoate synthase comp	2E-116	COG0147	Anthranilate/para-aminobenzoate synthase
===							6	Candidatus_Nitrosopumilus_koreensis_AR1_uid1	NC_018655	chromosome									
407462717	transcription factor TFIIB cyclin-related protein	No	0	1008456	1009346	-		Candidatus_Nitrosopumilus_koreensis_AR1_uid1	NC_018655	chromosome		arCOG01981	8 (-)	K	SUA7	Transcription initiation factor TFIIB, Brf1 subunit/Tr	1E-77	COG1405	Transcription initiation factor TFIIIB,
407462718	hypothetical protein NKOR_06050	No	0	1009370	1010233	-		Candidatus_Nitrosopumilus_koreensis_AR1_uid1	NC_018655	chromosome	clust_115								
407462719	hypothetical protein NKOR_06055	No	0	1010664	1010855	+		Candidatus_Nitrosopumilus_koreensis_AR1_uid1	NC_018655	chromosome	clust_465						0.00001	COG0419	ATPase involved in DNA repair
407462720	hypothetical protein NKOR_06060	No	0	1010977	1011099	-		Candidatus_Nitrosopumilus_koreensis_AR1_uid1	NC_018655	chromosome									
407462721	hypothetical protein NKOR_06065	No	0	1011172	1011384	-		Candidatus_Nitrosopumilus_koreensis_AR1_uid1	NC_018655	chromosome									
407462722	parB-like partition protein	No	0	1011526	1012335	+		Candidatus_Nitrosopumilus_koreensis_AR1_uid1	NC_018655	chromosome		arCOG01875	2 (-)	K		ParB-like nuclease domain	2E-11	COG1475	Stage 0 sporulation protein J (antagonis
===							5	Candidatus_Nitrosopumilus_koreensis_AR1_uid1	NC_018655	chromosome									
407462724	hypothetical protein NKOR_06080	No	0	1013748	1013936	+		Candidatus_Nitrosopumilus_koreensis_AR1_uid1	NC_018655	chromosome									
407462725	hypothetical protein NKOR_06085	No	0	1013959	1014387	-		Candidatus_Nitrosopumilus_koreensis_AR1_uid1	NC_018655	chromosome									
407462726	hypothetical protein NKOR_06090	Yes	1	1014513	1015076	+		Candidatus_Nitrosopumilus_koreensis_AR1_uid1	NC_018655	chromosome	arCOG08643,a	arCOG08643	2 (-)	S		Uncharacterized conserved protein			
407462727	hypothetical protein NKOR_06095	No	0	1015359	1015772	+		Candidatus_Nitrosopumilus_koreensis_AR1_uid1	NC_018655	chromosome									
407462728	hypothetical protein NKOR_06100	Yes	1	1015921	1016148	+		Candidatus_Nitrosopumilus_koreensis_AR1_uid1	NC_018655	chromosome									
===							11	Candidatus_Nitrosopumilus_koreensis_AR1_uid1	NC_018655	chromosome									
407462739	hypothetical protein NKOR_06165	No	2	1027852	1028109	-		Candidatus_Nitrosopumilus_koreensis_AR1_uid1	NC_018655	chromosome	clust_55								
407462740	hypothetical protein NKOR_06170	No	1	1028195	1028650	+		Candidatus_Nitrosopumilus_koreensis_AR1_uid1	NC_018655	chromosome									
407462741	hypothetical protein NKOR_06175	No	2	1028686	1028940	-		Candidatus_Nitrosopumilus_koreensis_AR1_uid1	NC_018655	chromosome									
407462742	hypothetical protein NKOR_06180	Yes	0	1028944	1029462	-		Candidatus_Nitrosopumilus_koreensis_AR1_uid1	NC_018655	chromosome		arCOG08800	2 (-)	S		PKD domain containing protein			
407462743	hypothetical protein NKOR_06185	No	1	1029825	1030070	-		Candidatus_Nitrosopumilus_koreensis_AR1_uid1	NC_018655	chromosome									
407462744	hypothetical protein NKOR_06190	Yes	0	1030060	1030725	-		Candidatus_Nitrosopumilus_koreensis_AR1_uid1	NC_018655	chromosome									
407462745	hypothetical protein NKOR_06195	No	1	1030779	1031330	-		Candidatus_Nitrosopumilus_koreensis_AR1_uid1	NC_018655	chromosome		arCOG10557	1	S		Uncharacterized conserved protein			
407462746	hypothetical protein NKOR_06200	No	1	1031337	1031798	-		Candidatus_Nitrosopumilus_koreensis_AR1_uid1	NC_018655	chromosome									
407462747	hypothetical protein NKOR_06205	No	2	1031773	1032222	-		Candidatus_Nitrosopumilus_koreensis_AR1_uid1	NC_018655	chromosome		arCOG00579	4 (-)	K	RPB9	DNA-directed RNA polymerase, subunit M/Transcription elongation factor TFIIS			
407462748	hypothetical protein NKOR_06210	Yes	2	1032219	1032953	-		Candidatus_Nitrosopumilus_koreensis_AR1_uid1	NC_018655	chromosome									
407462749	hypothetical protein NKOR_06215	Yes	2	1032950	1033489	-		Candidatus_Nitrosopumilus_koreensis_AR1_uid1	NC_018655	chromosome									
===							5	Candidatus_Nitrosopumilus_koreensis_AR1_uid1	NC_018655	chromosome									
407462761	hypothetical protein NKOR_06275	No	0	1045797	1046039	+		Candidatus_Nitrosopumilus_koreensis_AR1_uid1	NC_018655	chromosome									
407462762	hypothetical protein NKOR_06280	No	0	1046143	1048074	-		Candidatus_Nitrosopumilus_koreensis_AR1_uid1	NC_018655	chromosome		arCOG00369	1 (-)	S		Uncharacterized conserved protein	4E-40	pfam12532	Protein of unknown function (DUF3732). T ; Chromosome segregation ATPases
407462763	hypothetical protein NKOR_06285	No	0	1048074	1048562	-		Candidatus_Nitrosopumilus_koreensis_AR1_uid1	NC_018655	chromosome									
407462764	hypothetical protein NKOR_06290	No	0	1048546	1049181	-		Candidatus_Nitrosopumilus_koreensis_AR1_uid1	NC_018655	chromosome									
407462765	hypothetical protein NKOR_06295	No	0	1049327	1049734	-		Candidatus_Nitrosopumilus_koreensis_AR1_uid1	NC_018655	chromosome									
===							8	Candidatus_Nitrosopumilus_koreensis_AR1_uid1	NC_018655	chromosome									
407462888	hypothetical protein NKOR_06960	No	0	1155032	1155418	-		Candidatus_Nitrosopumilus_koreensis_AR1_uid1	NC_018655	chromosome		arCOG08718	1 (-)	S		Uncharacterized conserved protein			
407462889	hypothetical protein NKOR_06965	No	0	1155543	1156754	-		Candidatus_Nitrosopumilus_koreensis_AR1_uid1	NC_018655	chromosome									
407462890	hypothetical protein NKOR_06970	No	0	1156907	1158010	+		Candidatus_Nitrosopumilus_koreensis_AR1_uid1	NC_018655	chromosome		arCOG10679	2	S		Uncharacterized conserved protein			
407462891	hypothetical protein NKOR_06975	No	2	1158128	1158442	-		Candidatus_Nitrosopumilus_koreensis_AR1_uid1	NC_018655	chromosome									
407462892	hypothetical protein NKOR_06980	No	0	1158411	1158671	+		Candidatus_Nitrosopumilus_koreensis_AR1_uid1	NC_018655	chromosome									
407462893	hypothetical protein NKOR_06985	No	0	1158971	1160887	-		Candidatus_Nitrosopumilus_koreensis_AR1_uid1	NC_018655	chromosome									
407462894	hypothetical protein NKOR_06990	No	0	1160951	1161190	-		Candidatus_Nitrosopumilus_koreensis_AR1_uid1	NC_018655	chromosome									
407462895	hypothetical protein NKOR_06995	No	0	1161369	1161863	-		Candidatus_Nitrosopumilus_koreensis_AR1_uid1	NC_018655	chromosome		arCOG04076	1 (-)	S		Uncharacterized conserved protein	2E-31	COG1909	Uncharacterized protein conserved in arc
===							5	Candidatus_Nitrosopumilus_koreensis_AR1_uid1	NC_018655	chromosome									
407462992	hypothetical protein NKOR_07530	No	0	1253977	1254189	-		Candidatus_Nitrosopumilus_koreensis_AR1_uid1	NC_018655	chromosome									
407462993	hypothetical protein NKOR_07535	No	1	1254341	1254865	-		Candidatus_Nitrosopumilus_koreensis_AR1_uid1	NC_018655	chromosome									
407462994	transcription factor TFIIB cyclin-related protein	No	0	1255647	1256576	-		Candidatus_Nitrosopumilus_koreensis_AR1_uid1	NC_018655	chromosome		arCOG01981	8 (-)	K	SUA7	Transcription initiation factor TFIIB, Brf1 subunit/Tr	8E-64	COG1405	Transcription initiation factor TFIIIB,
407462995	hypothetical protein NKOR_07545	No	0	1256830	1257012	-		Candidatus_Nitrosopumilus_koreensis_AR1_uid1	NC_018655	chromosome									
407462996	hypothetical protein NKOR_07550	No	0	1257405	1257632	+		Candidatus_Nitrosopumilus_koreensis_AR1_uid1	NC_018655	chromosome									
===							7	Candidatus_Nitrosopumilus_koreensis_AR1_uid1	NC_018655	chromosome									
407463206	hypothetical protein NKOR_08630	No	0	1443475	1443669	-		Candidatus_Nitrosopumilus_koreensis_AR1_uid1	NC_018655	chromosome									
407463207	protoheme IX farnesyltransferase	No	9	1443918	1444847	+		Candidatus_Nitrosopumilus_koreensis_AR1_uid1	NC_018655	chromosome		arCOG00479	2 (-)	O	CyoE	Polyprenyltransferase (cytochrome oxidase asse	1E-49	COG0109	Polyprenyltransferase (cytochrome oxidas
407463208	hypothetical protein NKOR_08650	No	0	1444842	1445051	-		Candidatus_Nitrosopumilus_koreensis_AR1_uid1	NC_018655	chromosome		arCOG10591	2	S		Uncharacterized conserved protein			
407463209	hypothetical protein NKOR_08655	Yes	0	1445374	1445874	+		Candidatus_Nitrosopumilus_koreensis_AR1_uid1	NC_018655	chromosome		arCOG10592	2	S		Uncharacterized conserved protein			
407463210	hypothetical protein NKOR_08660	No	0	1446504	1447001	-		Candidatus_Nitrosopumilus_koreensis_AR1_uid1	NC_018655	chromosome									
407463211	hypothetical protein NKOR_08665	No	0	1447087	1447494	+		Candidatus_Nitrosopumilus_koreensis_AR1_uid1	NC_018655	chromosome		arCOG07348	2 (-)	S		Uncharacterized conserved protein			
407463212	hypothetical protein NKOR_08670	No	0	1447491	1448174	+		Candidatus_Nitrosopumilus_koreensis_AR1_uid1	NC_018655	chromosome		arCOG10593	1	S		Uncharacterized conserved protein			
===							10	Candidatus_Nitrososphaera_gargensis_Ga9_2_ui	NC_018719	chromosome									
408402656	Kelch repeat-containing protein	Yes	1	27397	28419	+		Candidatus_Nitrososphaera_gargensis_Ga9_2_ui	NC_018719	chromosome		arCOG07536	1 (-)	S		Uncharacterized conserved protein	0.000000001	pfam13964	Kelch motif. ; Kelch motif.
408402657	hypothetical protein Ngar_c00330	No	0	28430	28606	-		Candidatus_Nitrososphaera_gargensis_Ga9_2_ui	NC_018719	chromosome									
408402658	hypothetical protein Ngar_c00340	No	0	28609	28809	-		Candidatus_Nitrososphaera_gargensis_Ga9_2_ui	NC_018719	chromosome									
408402659	hypothetical protein Ngar_c00350	No	0	29003	29212	-		Candidatus_Nitrososphaera_gargensis_Ga9_2_ui	NC_018719	chromosome									
408402660	hypothetical protein Ngar_c00360	No	0	29286	29639	-		Candidatus_Nitrososphaera_gargensis_Ga9_2_ui	NC_018719	chromosome									
408402661	hypothetical protein Ngar_c00370	No	0	29662	29844	-		Candidatus_Nitrososphaera_gargensis_Ga9_2_ui	NC_018719	chromosome	clust_476								
408402662	GTP-binding protein	No	0	30200	31330	+		Candidatus_Nitrososphaera_gargensis_Ga9_2_ui	NC_018719	chromosome		arCOG00353	1 (-)	R	HflX	GTP-binding protein protease modulator	5E-60	COG2262	GTPases
408402663	hypothetical protein Ngar_c00390	No	0	31459	31764	+		Candidatus_Nitrososphaera_gargensis_Ga9_2_ui	NC_018719	chromosome	clust_305								
408402664	hypothetical protein Ngar_c00400	No	0	31827	32021	+		Candidatus_Nitrososphaera_gargensis_Ga9_2_ui	NC_018719	chromosome									
408402665	hypothetical protein Ngar_c00410	No	0	32110	32481	+		Candidatus_Nitrososphaera_gargensis_Ga9_2_ui	NC_018719	chromosome									
===							8	Candidatus_Nitrososphaera_gargensis_Ga9_2_ui	NC_018719	chromosome									
408402676	PIN domain-containing protein	No	0	39102	39494	+		Candidatus_Nitrososphaera_gargensis_Ga9_2_ui	NC_018719	chromosome		arCOG02219	4 (-)	V	VapC	PIN domain containing protein	2E-16	COG1487	Predicted nucleic acid-binding protein,
408402677	hypothetical protein Ngar_c00530	No	1	39751	39867	+		Candidatus_Nitrososphaera_gargensis_Ga9_2_ui	NC_018719	chromosome									
408402678	hypothetical protein Ngar_c00540	Yes	1	40179	41138	+		Candidatus_Nitrososphaera_gargensis_Ga9_2_ui	NC_018719	chromosome									
408402679	hypothetical protein Ngar_c00550	No	0	41257	41502	-		Candidatus_Nitrososphaera_gargensis_Ga9_2_ui	NC_018719	chromosome									
408402680	hypothetical protein Ngar_c00560	No	0	41481	41621	-		Candidatus_Nitrososphaera_gargensis_Ga9_2_ui	NC_018719	chromosome									
408402681	hypothetical protein Ngar_c00570	No	0	41625	41822	-		Candidatus_Nitrososphaera_gargensis_Ga9_2_ui	NC_018719	chromosome									
408402682	transposase	No	0	42034	42243	-		Candidatus_Nitrososphaera_gargensis_Ga9_2_ui	NC_018719	chromosome							0.0000001	pfam13518	Helix-turn-helix domain. This helix-turn
408402683	hypothetical protein Ngar_c00590	No	0	42351	42590	+		Candidatus_Nitrososphaera_gargensis_Ga9_2_ui	NC_018719	chromosome		arCOG08146	1 (-)	V		Predicted antitoxin, copG family	0.000007	pfam02697	Uncharacterized ACR, COG1753. Structural
===							5	Candidatus_Nitrososphaera_gargensis_Ga9_2_ui	NC_018719	chromosome									
408402691	hypothetical protein Ngar_c00670	No	0	46016	46168	+		Candidatus_Nitrososphaera_gargensis_Ga9_2_ui	NC_018719	chromosome									
408402692	hypothetical protein Ngar_c00680	No	0	46493	46669	+		Candidatus_Nitrososphaera_gargensis_Ga9_2_ui	NC_018719	chromosome	clust_481								
408402693	hypothetical protein Ngar_c00690	No	0	46975	47169	+		Candidatus_Nitrososphaera_gargensis_Ga9_2_ui	NC_018719	chromosome	clust_440								
408402694	transposase, IS116/IS110/IS902 family protein	No	0	47488	47841	+		Candidatus_Nitrososphaera_gargensis_Ga9_2_ui	NC_018719	chromosome		arCOG03585	6 (-)	L		Transposase	0.00001	pfam02371	Transposase IS116/IS110/IS902 family. Tr
408402695	hypothetical protein Ngar_c00710	No	0	47801	47983	+		Candidatus_Nitrososphaera_gargensis_Ga9_2_ui	NC_018719	chromosome	clust_477								
===							10	Candidatus_Nitrososphaera_gargensis_Ga9_2_ui	NC_018719	chromosome									
408402786	hypothetical protein Ngar_c01630	No	0	130956	131357	+		Candidatus_Nitrososphaera_gargensis_Ga9_2_ui	NC_018719	chromosome		arCOG08784	1 (-)	C		4Fe-4S ferredoxin, iron-sulfur binding domain protein			
408402787	glycoside hydrolase family 37	No	0	131368	131631	-		Candidatus_Nitrososphaera_gargensis_Ga9_2_ui	NC_018719	chromosome									
408402788	glycoside hydrolase family 37	No	0	131982	132239	-		Candidatus_Nitrososphaera_gargensis_Ga9_2_ui	NC_018719	chromosome									
408402789	hypothetical protein Ngar_c01660	No	0	132346	132741	-		Candidatus_Nitrososphaera_gargensis_Ga9_2_ui	NC_018719	chromosome									
408402790	hypothetical protein Ngar_c01670	No	4	132952	133398	-		Candidatus_Nitrososphaera_gargensis_Ga9_2_ui	NC_018719	chromosome									
408402791	C-type lectin domain-containing protein	No	0	133429	133662	-		Candidatus_Nitrososphaera_gargensis_Ga9_2_ui	NC_018719	chromosome									
408402792	hypothetical protein Ngar_c01690	No	0	133804	134286	+		Candidatus_Nitrososphaera_gargensis_Ga9_2_ui	NC_018719	chromosome									

408402793	inosine-5'-monophosphate dehydrogenase	No	0		134314	134772	-		Candidatus_Nitrososphaera_gargensis_Ga9_2_ui	NC_018719	chromosome			arCOG00606	4 (-)	R		CBS domain	3E-14	COG0517	FOG: CBS domain
408402794	zinc finger C2H2 domain-containing protein	No	0		134870	135061	+		Candidatus_Nitrososphaera_gargensis_Ga9_2_ui	NC_018719	chromosome										
408402795	glucose starvation-inducible protein B	No	0		135111	135434	-		Candidatus_Nitrososphaera_gargensis_Ga9_2_ui	NC_018719	chromosome			arCOG10928	3	S		Uncharacterized conserved protein	0.0000007	pfam00477	Small hydrophilic plant seed protein.
===								6	Candidatus_Nitrososphaera_gargensis_Ga9_2_ui	NC_018719	chromosome										
408402799	small-conductance mechanosensitive ion chan	No	5		138177	139208	-		Candidatus_Nitrososphaera_gargensis_Ga9_2_ui	NC_018719	chromosome			arCOG01568	4 (-)	M	MscS	Small-conductance mechanosensitive channel	8E-13	COG0668	Small-conductance mechanosensitive chann
408402800	hypothetical protein Ngar_c01770	No	0		139363	139539	-		Candidatus_Nitrososphaera_gargensis_Ga9_2_ui	NC_018719	chromosome										
408402801	hypothetical protein Ngar_c01780	No	0		139665	139886	+		Candidatus_Nitrososphaera_gargensis_Ga9_2_ui	NC_018719	chromosome										
408402802	zinc finger C2H2 domain-containing protein, pa	No	0		140372	140701	-		Candidatus_Nitrososphaera_gargensis_Ga9_2_ui	NC_018719	chromosome										
408402803	hypothetical protein Ngar_c01800	No	0		140910	141650	-		Candidatus_Nitrososphaera_gargensis_Ga9_2_ui	NC_018719	chromosome	clust_135									
408402804	hypothetical protein Ngar_c01810	No	0		141657	142223	-		Candidatus_Nitrososphaera_gargensis_Ga9_2_ui	NC_018719	chromosome		arCOG03593,a	arCOG03593	2 (-)	S		Uncharacterized conserved protein	0.0001	pfam11387	Protein of unknown function (DUF2795). T ; Protein of unknown function (DUF2795). T
===								18	Candidatus_Nitrososphaera_gargensis_Ga9_2_ui	NC_018719	chromosome										
408402848	hypothetical protein Ngar_c02260	No	0		181422	181679	+		Candidatus_Nitrososphaera_gargensis_Ga9_2_ui	NC_018719	chromosome										
408402849	UspA domain-containing protein	No	0		182146	182625	+		Candidatus_Nitrososphaera_gargensis_Ga9_2_ui	NC_018719	chromosome			arCOG02053	7 (-)	T	UspA	Nucleotide-binding protein, UspA family	2E-16	pfam00582	Universal stress protein family. The uni
408402850	hypothetical protein Ngar_c02280	No	0		182790	183029	-		Candidatus_Nitrososphaera_gargensis_Ga9_2_ui	NC_018719	chromosome										
408402851	hypothetical protein Ngar_c02290	No	0		183126	183311	-		Candidatus_Nitrososphaera_gargensis_Ga9_2_ui	NC_018719	chromosome										
408402852	hypothetical protein Ngar_c02300	No	0		183422	183628	-		Candidatus_Nitrososphaera_gargensis_Ga9_2_ui	NC_018719	chromosome										
408402853	transcription initiation factor IIB	No	0		183943	184821	+		Candidatus_Nitrososphaera_gargensis_Ga9_2_ui	NC_018719	chromosome			arCOG01981	8 (-)	K	SUA7	Transcription initiation factor TFIIB, Brf1 subunit/Tr	2E-72	COG1405	Transcription initiation factor TFIIB,
408402854	hypothetical protein Ngar_c02320	No	0		184838	185095	+		Candidatus_Nitrososphaera_gargensis_Ga9_2_ui	NC_018719	chromosome										
408402855	hypothetical protein Ngar_c02330	No	0		185130	185378	-		Candidatus_Nitrososphaera_gargensis_Ga9_2_ui	NC_018719	chromosome										
408402856	hypothetical protein Ngar_c02340	No	0		185393	185569	-		Candidatus_Nitrososphaera_gargensis_Ga9_2_ui	NC_018719	chromosome	clust_476									
408402857	hypothetical protein Ngar_c02350	No	0		185722	186003	+		Candidatus_Nitrososphaera_gargensis_Ga9_2_ui	NC_018719	chromosome			arCOG01117	5 (-)	K	Lrp	Lrp/AsnC family C-terminal domain			
408402858	hypothetical protein Ngar_c02360	No	0		186256	186462	-		Candidatus_Nitrososphaera_gargensis_Ga9_2_ui	NC_018719	chromosome										
408402859	hypothetical protein Ngar_c02370	No	0		186709	186963	-		Candidatus_Nitrososphaera_gargensis_Ga9_2_ui	NC_018719	chromosome										
408402860	hypothetical protein Ngar_c02380	No	0		187018	187401	-		Candidatus_Nitrososphaera_gargensis_Ga9_2_ui	NC_018719	chromosome										
408402861	hypothetical protein Ngar_c02390	No	0		187683	187862	-		Candidatus_Nitrososphaera_gargensis_Ga9_2_ui	NC_018719	chromosome										
408402862	hypothetical protein Ngar_c02400	No	0		188136	188564	+		Candidatus_Nitrososphaera_gargensis_Ga9_2_ui	NC_018719	chromosome										
408402863	hypothetical protein Ngar_c02410	No	0		188587	188757	-		Candidatus_Nitrososphaera_gargensis_Ga9_2_ui	NC_018719	chromosome										
408402864	hypothetical protein Ngar_c02420	No	0		188839	189024	-		Candidatus_Nitrososphaera_gargensis_Ga9_2_ui	NC_018719	chromosome										
408402865	AsnC family transcriptional regulator	No	0		189027	189296	-		Candidatus_Nitrososphaera_gargensis_Ga9_2_ui	NC_018719	chromosome			arCOG01117	5 (-)	K	Lrp	Lrp/AsnC family C-terminal domain			
===								11	Candidatus_Nitrososphaera_gargensis_Ga9_2_ui	NC_018719	chromosome										
408402916	triosephosphate isomerase	No	0		226895	227554	+		Candidatus_Nitrososphaera_gargensis_Ga9_2_ui	NC_018719	chromosome			arCOG01087	1 (-)	G	TpiA	Triosephosphate isomerase	1E-15	COG0149	Triosephosphate isomerase
408402917	hypothetical protein Ngar_c02950	No	0		227789	228481	-		Candidatus_Nitrososphaera_gargensis_Ga9_2_ui	NC_018719	chromosome										
408402918	hypothetical protein Ngar_c02960	No	0		228475	229206	-		Candidatus_Nitrososphaera_gargensis_Ga9_2_ui	NC_018719	chromosome										
408402919	hypothetical protein Ngar_c02970	No	0		229331	230131	-		Candidatus_Nitrososphaera_gargensis_Ga9_2_ui	NC_018719	chromosome										
408402920	hypothetical protein Ngar_c02980	No	0		230103	230294	-		Candidatus_Nitrososphaera_gargensis_Ga9_2_ui	NC_018719	chromosome										
408402921	hypothetical protein Ngar_c02990	No	0		230472	231305	+		Candidatus_Nitrososphaera_gargensis_Ga9_2_ui	NC_018719	chromosome										
408402922	hypothetical protein Ngar_c03000	No	0		231315	232160	+		Candidatus_Nitrososphaera_gargensis_Ga9_2_ui	NC_018719	chromosome										
408402923	hypothetical protein Ngar_c03010	No	0		232157	232441	-		Candidatus_Nitrososphaera_gargensis_Ga9_2_ui	NC_018719	chromosome										
408402924	hypothetical protein Ngar_c03020	No	0		232690	233313	-		Candidatus_Nitrososphaera_gargensis_Ga9_2_ui	NC_018719	chromosome										
408402925	hypothetical protein Ngar_c03030	No	0		233289	233924	-		Candidatus_Nitrososphaera_gargensis_Ga9_2_ui	NC_018719	chromosome			arCOG01446	5 (-)	VK	Csa3	CRISPR-Cas assicated transcriptional regulator, contains CARF and HTH domain			
408402926	hypothetical protein Ngar_c03040	No	0		234067	234219	+		Candidatus_Nitrososphaera_gargensis_Ga9_2_ui	NC_018719	chromosome										
===								16	Candidatus_Nitrososphaera_gargensis_Ga9_2_ui	NC_018719	chromosome										
408402935	hypothetical protein Ngar_c03130	No	0	Virus_Turrivirid	239151	239330	+		Candidatus_Nitrososphaera_gargensis_Ga9_2_ui	NC_018719	chromosome	clust_480									
408402936	hypothetical protein Ngar_c03140	No	0	Virus_Turrivirid	239486	241045	+		Candidatus_Nitrososphaera_gargensis_Ga9_2_ui	NC_018719	chromosome			arCOG01245	15 (-)	L	XerC	Integrase			
408402937	hypothetical protein Ngar_c03150	No	0	Virus_Turrivirid	241053	241850	+		Candidatus_Nitrososphaera_gargensis_Ga9_2_ui	NC_018719	chromosome	clust_124									
408402938	hypothetical protein Ngar_c03160	No	0	Virus_Turrivirid	241847	242269	+		Candidatus_Nitrososphaera_gargensis_Ga9_2_ui	NC_018719	chromosome	clust_249									
408402939	hypothetical protein Ngar_c03170	No	0	Virus_Turrivirid	242350	242826	-		Candidatus_Nitrososphaera_gargensis_Ga9_2_ui	NC_018719	chromosome	clust_220									
408402940	hypothetical protein Ngar_c03180	No	0	Virus_Turrivirid	242852	243028	+		Candidatus_Nitrososphaera_gargensis_Ga9_2_ui	NC_018719	chromosome	clust_482									
408402941	hypothetical protein Ngar_c03190	No	0	Virus_Turrivirid	243584	243817	+		Candidatus_Nitrososphaera_gargensis_Ga9_2_ui	NC_018719	chromosome	clust_417									
408402942	hypothetical protein Ngar_c03200	No	0	Virus_Turrivirid	243820	244539	+		Candidatus_Nitrososphaera_gargensis_Ga9_2_ui	NC_018719	chromosome	clust_138									
408402943	IS-4 family transposase	No	0	Virus_Turrivirid	244654	245871	-		Candidatus_Nitrososphaera_gargensis_Ga9_2_ui	NC_018719	chromosome			arCOG03902	8 (-)	L		Transposase			
408402944	hypothetical protein Ngar_c03220	No	1	Virus_Turrivirid	246099	246719	+		Candidatus_Nitrososphaera_gargensis_Ga9_2_ui	NC_018719	chromosome	clust_164									
408402945	hypothetical protein Ngar_c03230	No	0	Virus_Turrivirid	246860	247015	+		Candidatus_Nitrososphaera_gargensis_Ga9_2_ui	NC_018719	chromosome										
408402946	hypothetical protein Ngar_c03240	No	0	Virus_Turrivirid	247031	247207	+		Candidatus_Nitrososphaera_gargensis_Ga9_2_ui	NC_018719	chromosome	clust_483									
408402947	hypothetical protein Ngar_c03250	No	0	Virus_Turrivirid	247213	248097	+		Candidatus_Nitrososphaera_gargensis_Ga9_2_ui	NC_018719	chromosome	clust_110							0.000001	pfam12406	Surface protein. This family of proteins
408402948	hypothetical protein Ngar_c03260	No	0	Virus_Turrivirid	248132	248368	+		Candidatus_Nitrososphaera_gargensis_Ga9_2_ui	NC_018719	chromosome	clust_411									
408402949	hypothetical protein Ngar_c03270	No	0	Virus_Turrivirid	248365	249069	+		Candidatus_Nitrososphaera_gargensis_Ga9_2_ui	NC_018719	chromosome			arCOG03166	9 (-)	R		AAA+ superfamily ATPase fused to HTH and RecB nuclease domains			
408402950	hypothetical protein Ngar_c03280	No	0	Virus_Turrivirid	249066	249248	+		Candidatus_Nitrososphaera_gargensis_Ga9_2_ui	NC_018719	chromosome	clust_478									
===								11	Candidatus_Nitrososphaera_gargensis_Ga9_2_ui	NC_018719	chromosome										
408402952	hypothetical protein Ngar_c03300	No	1	Virus_Turrivirid	250651	250845	+		Candidatus_Nitrososphaera_gargensis_Ga9_2_ui	NC_018719	chromosome	clust_462									
408402953	hypothetical protein Ngar_c03310	No	1	Virus_Turrivirid	251377	251532	+		Candidatus_Nitrososphaera_gargensis_Ga9_2_ui	NC_018719	chromosome	clust_496									
408402954	hypothetical protein Ngar_c03320	No	1	Virus_Turrivirid	251550	252344	+		Candidatus_Nitrososphaera_gargensis_Ga9_2_ui	NC_018719	chromosome			arCOG02338	2 (-)	T	AtoS	Sensory protein, contains PAS and GAF domains			
408402955	hypothetical protein Ngar_c03330	No	0	Virus_Turrivirid	252699	253406	+		Candidatus_Nitrososphaera_gargensis_Ga9_2_ui	NC_018719	chromosome	clust_142									
408402956	hypothetical protein Ngar_c03340	No	1	Virus_Turrivirid	253403	253609	+		Candidatus_Nitrososphaera_gargensis_Ga9_2_ui	NC_018719	chromosome	clust_448									
408402957	hypothetical protein Ngar_c03350	No	0	Virus_Turrivirid	253610	254497	+		Candidatus_Nitrososphaera_gargensis_Ga9_2_ui	NC_018719	chromosome	clust_109									
408402958	hypothetical protein Ngar_c03360	No	1	Virus_Turrivirid	254549	255064	+		Candidatus_Nitrososphaera_gargensis_Ga9_2_ui	NC_018719	chromosome	clust_189									
408402959	hypothetical protein Ngar_c03370	No	0	Virus_Turrivirid	255052	255348	+		Candidatus_Nitrososphaera_gargensis_Ga9_2_ui	NC_018719	chromosome										
408402960	hypothetical protein Ngar_c03380	No	1	Virus_Turrivirid	255348	255983	+		Candidatus_Nitrososphaera_gargensis_Ga9_2_ui	NC_018719	chromosome								0.00004	pfam01456	Mucin-like glycoprotein. This family of
408402961	hypothetical protein Ngar_c03390	No	0	Virus_Turrivirid	256747	256872	+		Candidatus_Nitrososphaera_gargensis_Ga9_2_ui	NC_018719	chromosome										
408402962	hypothetical protein Ngar_c03400	No	0	Virus_Turrivirid	256874	257248	+		Candidatus_Nitrososphaera_gargensis_Ga9_2_ui	NC_018719	chromosome			arCOG08550	2 (-)	V		Predicted antitoxin, copG family			
===								15	Candidatus_Nitrososphaera_gargensis_Ga9_2_ui	NC_018719	chromosome										
408402965	hypothetical protein Ngar_c03430	No	0	Virus_Turrivirid	258214	258393	+		Candidatus_Nitrososphaera_gargensis_Ga9_2_ui	NC_018719	chromosome	clust_480									
408402966	hypothetical protein Ngar_c03440	No	0	Virus_Turrivirid	258549	260108	+		Candidatus_Nitrososphaera_gargensis_Ga9_2_ui	NC_018719	chromosome			arCOG01245	15 (-)	L	XerC	Integrase			
408402967	hypothetical protein Ngar_c03450	No	0	Virus_Turrivirid	260116	260913	+		Candidatus_Nitrososphaera_gargensis_Ga9_2_ui	NC_018719	chromosome	clust_124									
408402968	hypothetical protein Ngar_c03460	No	0	Virus_Turrivirid	260910	261332	+		Candidatus_Nitrososphaera_gargensis_Ga9_2_ui	NC_018719	chromosome	clust_249									
408402969	hypothetical protein Ngar_c03470	No	0	Virus_Turrivirid	261413	261889	-		Candidatus_Nitrososphaera_gargensis_Ga9_2_ui	NC_018719	chromosome	clust_220									
408402970	hypothetical protein Ngar_c03480	No	0	Virus_Turrivirid	261915	262091	+		Candidatus_Nitrososphaera_gargensis_Ga9_2_ui	NC_018719	chromosome	clust_482									
408402971	hypothetical protein Ngar_c03490	No	0	Virus_Turrivirid	262647	262880	+		Candidatus_Nitrososphaera_gargensis_Ga9_2_ui	NC_018719	chromosome	clust_417									
408402972	hypothetical protein Ngar_c03500	No	0	Virus_Turrivirid	262883	263602	+		Candidatus_Nitrososphaera_gargensis_Ga9_2_ui	NC_018719	chromosome	clust_138									
408402973	hypothetical protein Ngar_c03510	No	0	Virus_Turrivirid	263599	263805	+		Candidatus_Nitrososphaera_gargensis_Ga9_2_ui	NC_018719	chromosome										
408402974	hypothetical protein Ngar_c03520	No	1	Virus_Turrivirid	263828	264448	+		Candidatus_Nitrososphaera_gargensis_Ga9_2_ui	NC_018719	chromosome	clust_164									
408402975	hypothetical protein Ngar_c03530	No	0	Virus_Turrivirid	264760	264936	+		Candidatus_Nitrososphaera_gargensis_Ga9_2_ui	NC_018719	chromosome	clust_483									
408402976	hypothetical protein Ngar_c03540	No	0	Virus_Turrivirid	264942	265826	+		Candidatus_Nitrososphaera_gargensis_Ga9_2_ui	NC_018719	chromosome	clust_110							0.000001	pfam12406	Surface protein. This family of proteins
408402977	hypothetical protein Ngar_c03550	No	0	Virus_Turrivirid	265861	266097	+		Candidatus_Nitrososphaera_gargensis_Ga9_2_ui	NC_018719	chromosome	clust_411									
408402978	hypothetical protein Ngar_c03560	No	0	Virus_Turrivirid	266094	266798	+		Candidatus_Nitrososphaera_gargensis_Ga9_2_ui	NC_018719	chromosome			arCOG03166	9 (-)	R		AAA+ superfamily ATPase fused to HTH and RecB nuclease domains			
408402979	hypothetical protein Ngar_c03570	No	0	Virus_Turrivirid	266795	266977	+		Candidatus_Nitrososphaera_gargensis_Ga9_2_ui	NC_018719	chromosome	clust_478									
===								12	Candidatus_Nitrososphaera_gargensis_Ga9_2_ui	NC_018719	chromosome										
408402981	hypothetical protein Ngar_c03590	No	1	Virus_Turrivirid	268380	268574	+		Candidatus_Nitrososphaera_gargensis_Ga9_2_ui	NC_018719	chromosome	clust_462									
408402982	hypothetical protein Ngar_c03600	No	1	Virus_Turrivirid	269106	269261	+		Candidatus_Nitrososphaera_gargensis_Ga9_2_ui	NC_018719	chromosome	clust_496									
408402983	hypothetical protein Ngar_c03610	No	1	Virus_Turrivirid	269279	270073	+		Candidatus_Nitrososphaera_gargensis_Ga9_2_ui	NC_018719	chromosome			arCOG02338	2 (-)	T	AtoS	Sensory protein, contains PAS and GAF domains			
408402984	hypothetical protein Ngar_c03620	No	2	Virus_Turrivirid	270076	270258	+		Candidatus_Nitrososphaera_gargensis_Ga9_2_ui	NC_018719	chromosome										
408402985	hypothetical protein Ngar_c03630	No	0	Virus_Turrivirid	270255	270428	+		Candidatus_Nitrososphaera_gargensis_Ga9_2_ui	NC_018719	chromosome										
408402986	hypothetical protein Ngar_c03640	No	0	Virus_Turrivirid	270428	271135	+		Candidatus_Nitrososphaera_gargensis_Ga9_2_ui	NC_018719	chromosome	clust_142									
408402987	hypothetical protein Ngar_c03650	No	1	Virus_Turrivirid	271132	271338	+		Candidatus_Nitrososphaera_gargensis_Ga9_2_ui	NC_018719	chromosome	clust_448									
408402988	hypothetical protein Ngar_c03660	No	0	Virus_Turrivirid	271339	272226	+		Candidatus_Nitrososphaera_gargensis_Ga9_2_ui	NC_018719	chromosome	clust_109									
408402989	hypothetical protein Ngar_c03670	No	1	Virus_Turrivirid	272236	272793	+		Candidatus_Nitrososphaera_gargensis_Ga9_2_ui	NC_018719	chromosome	clust_189									
408402990	hypothetical protein Ngar_c03680	No	0	Virus_Turrivirid	272889	273077	+		Candidatus_Nitrososphaera_gargensis_Ga9_2_ui	NC_018719	chromosome										
408402991	hypothetical protein Ngar_c03690	No	1	Virus_Turrivirid	273077	273844	+		Candidatus_Nitrososphaera_gargensis_Ga9_2_ui	NC_018719	chromosome								0.000000003	pfam01456	Mucin-like glycoprotein. This family of
408402992	hypothetical protein Ngar_c03700	No	0	Virus_Turrivirid	274004	274228	+		Candidatus_Nitrososphaera_gargensis_Ga9_2_ui	NC_018719	chromosome			arCOG08706	1 (-)	S		Uncharacterized conserved protein			
===								8	Candidatus_Nitrososphaera_gargensis_Ga9_2_ui	NC_018719	chromosome										
408403086	hypothetical protein Ngar_c04680	No	0		348612	348863	+		Candidatus_Nitrososphaera_gargensis_Ga9_2_ui	NC_018719	chromosome										
408403087	acetyltransferase (GNAT) family protein	No	0		348863	349384	+		Candidatus_Nitrososphaera_gargensis_Ga9_2_ui	NC_018719	chromosome			arCOG04971	3 (-)	K	WecD	Acetyltransferase (GNAT) family			
408403088	hypothetical protein Ngar_c04700	No	0		349493	350164	+		Candidatus_Nitrososphaera_gargensis_Ga9_2_ui	NC_018719	chromosome										
408403089	hypothetical protein Ngar_c04710	No	0		350178	350417	+		Candidatus_Nitrososphaera_gargensis_Ga9_2_ui	NC_018719	chromosome										
408403090	hypothetical protein Ngar_c04720	No	0		350381	350944	+		Candidatus_Nitrososphaera_gargensis_Ga9_2_ui	NC_018719	chromosome										
408403091	hypothetical protein Ngar_c04730	Yes	2		351319	352218	-		Candidatus_Nitrososphaera_gargensis_Ga9_2_ui	NC_018719	chromosome										
408403092	hypothetical protein Ngar_c04740	No	0		352251	352487	-		Candidatus_Nitrososphaera_gargensis_Ga9_2_ui	NC_018719	chromosome			arCOG01247	4 (-)	L	XerD	Site-specific recombinase XerD			
408403093	hypothetical protein Ngar_c04750	No	0		352521	352847	-		Candidatus_Nitrososphaera_gargensis_Ga9_2_ui	NC_018719	chromosome										
===								5	Candidatus_Nitrososphaera_gargensis_Ga9_2_ui	NC_018719	chromosome										
408403096	hypothetical protein Ngar_c04780	No	0		354513	354983	+		Candidatus_Nitrososphaera_gargensis_Ga9_2_ui	NC_018719	chromosome										
408403097	hypothetical protein Ngar_c04790	No	0		355083	355265	+		Candidatus_Nitrososphaera_gargensis_Ga9_2_ui	NC_018719	chromosome										
408403098	hypothetical protein Ngar_c04800	Yes	1		355289	355999	-		Candidatus_Nitrososphaera_gargensis_Ga9_2_ui	NC_018719	chromosome										
408403099	hypothetical protein Ngar_c04810	No	3		356070	356417	-		Candidatus_Nitrososphaera_gargensis_Ga9_2_ui	NC_018719	chromosome			arCOG03295	1 (-)	S		Membrane protein containing Zn-ribbon domain			
408403100	hypothetical protein Ngar_c04820	No	0		356475	356726	-		Candidatus_Nitrososphaera_gargensis_Ga9_2_ui	NC_018719	chromosome										
===								10	Candidatus_Nitrososphaera_gargensis_Ga9_2_ui	NC_018719	chromosome										
408403113	methionine synthase	No	0		366570	369059	+		Candidatus_Nitrososphaera_gargensis_Ga9_2_ui	NC_018719	chromosome		arCOG01978,a	arCOG01978	1 (-)	H	FolP	Dihydropteroate synthase or related enzyme	1E-134	COG1410	Methionine synthase I, cobalamin-binding
408403114	hypothetical protein Ngar_c04960	No	0		369135	369509	+		Candidatus_Nitrososphaera_gargensis_Ga9_2_ui	NC_018719	chromosome										
408403115	hypothetical protein Ngar_c04970	Yes	1		369478	369921	-		Candidatus_Nitrososphaera_gargensis_Ga9_2_ui	NC_018719	chromosome	clust_184									
408403116	hypothetical protein Ngar_c04980	No	1		369976	370338	-		Candidatus_Nitrososphaera_gargensis_Ga9_2_ui	NC_018719	chromosome										
408403117	hypothetical protein Ngar_c04990	No	0		370572	370724	+		Candidatus_Nitrososphaera_gargensis_Ga9_2_ui	NC_018719	chromosome										
408403118	hypothetical protein Ngar_c05000	Yes	1		370883	371251	-		Candidatus_Nitrososphaera_gargensis_Ga9_2_ui	NC_018719	chromosome										
408403119	hypothetical protein Ngar_c05010	No	1		371534	371953	-		Candidatus_Nitrososphaera_gargensis_Ga9_2_ui	NC_018719	chromosome										
408403120	hypothetical protein Ngar_c05020	No	0		371950	372138	-		Candidatus_Nitrososphaera_gargensis_Ga9_2_ui	NC_018719	chromosome										
408403121	ISCpe7, transposase	No	0		372222	373358	+		Candidatus_Nitrososphaera_gargensis_Ga9_2_ui	NC_018719	chromosome			arCOG02134	15 (-)	L		Transposase	1E-10	pfam13610	DDE domain. This DDE domain is found in ; Transposase and inactivated derivatives
408403122	hypothetical protein Ngar_c05040	No	0		373344	373766	-		Candidatus_Nitrososphaera_gargensis_Ga9_2_ui	NC_018719	chromosome										
===								5	Candidatus_Nitrososphaera_gargensis_Ga9_2_ui	NC_018719	chromosome										
408403132	hypothetical protein Ngar_c05150	No	0		383444	384385	+		Candidatus_Nitrososphaera_gargensis_Ga9_2_ui	NC_018719	chromosome			arCOG06120	2	S		Uncharacterized conserved protein			
408403133	glycosyl transferase, group 1	No	0		384588	385913	+		Candidatus_Nitrososphaera_gargensis_Ga9_2_ui	NC_018719	chromosome			arCOG04796	1 (-)	M	RfaG	Glycosyltransferase	0.000000008	COG0438	Glycosyltransferase
408403134	hypothetical protein Ngar_c05170	No	0		385974	386231	-		Candidatus_Nitrososphaera_gargensis_Ga9_2_ui	NC_018719	chromosome										
408403135	hypothetical protein Ngar_c05180	No	2		386291	386533	+		Candidatus_Nitrososphaera_gargensis_Ga9_2_ui	NC_018719	chromosome										
408403136	hypothetical protein Ngar_c05190	No	1		387250	387441	-		Candidatus_Nitrososphaera_gargensis_Ga9_2_ui	NC_018719	chromosome										
===								5	Candidatus_Nitrososphaera_gargensis_Ga9_2_ui	NC_018719	chromosome										
408403139	hypothetical protein Ngar_c05220	No	0		389185	389343	-		Candidatus_Nitrososphaera_gargensis_Ga9_2_ui	NC_018719	chromosome										
408403140	hypothetical protein Ngar_c05230	No	0		389575	389715	-		Candidatus_Nitrososphaera_gargensis_Ga9_2_ui	NC_018719	chromosome										
408403141	signal transduction protein	No	0		390353	390841	+		Candidatus_Nitrososphaera_gargensis_Ga9_2_ui	NC_018719	chromosome			arCOG00606	4 (-)	R		CBS domain	4E-17	COG0517	FOG: CBS domain
408403142	hypothetical protein Ngar_c05250	No	0		390922	391071	-		Candidatus_Nitrososphaera_gargensis_Ga9_2_ui	NC_018719	chromosome										
408403143	hypothetical protein Ngar_c05260	No	0		391252	391644	-		Candidatus_Nitrososphaera_gargensis_Ga9_2_ui	NC_018719	chromosome										
===								9	Candidatus_Nitrososphaera_gargensis_Ga9_2_ui	NC_018719	chromosome										
408403153	archaeal serine protease	Yes	1		399629	400591	+		Candidatus_Nitrososphaera_gargensis_Ga9_2_ui	NC_018719	chromosome			arCOG01937	2 (-)	R		Archaeal serine protease	4E-26	COG1750	Archaeal serine proteases
408403154	hypothetical protein Ngar_c05370	No	1		400792	401274	-		Candidatus_Nitrososphaera_gargensis_Ga9_2_ui	NC_018719	chromosome										
408403155	hypothetical protein Ngar_c05380	No	4		401325	401735	-		Candidatus_Nitrososphaera_gargensis_Ga9_2_ui	NC_018719	chromosome										
408403156	hypothetical protein Ngar_c05390	No	1		401778	402296	-		Candidatus_Nitrososphaera_gargensis_Ga9_2_ui	NC_018719	chromosome										
408403157	hypothetical protein Ngar_c05400	No	0		402394	402852	+		Candidatus_Nitrososphaera_gargensis_Ga9_2_ui	NC_018719	chromosome										
408403158	hypothetical protein Ngar_c05410	No	0		402965	403939	+		Candidatus_Nitrososphaera_gargensis_Ga9_2_ui	NC_018719	chromosome			arCOG07813	7 (-)	G		Glycosyl hydrolases family 43			
408403159	small-conductance mechanosensitive ion chan	No	0		403936	404397	+		Candidatus_Nitrososphaera_gargensis_Ga9_2_ui	NC_018719	chromosome										
408403160	coagulation factor 5/8 type domain-containing p	No	0		404397	405098	+		Candidatus_Nitrososphaera_gargensis_Ga9_2_ui	NC_018719	chromosome										
408403161	coagulation factor 5/8 type domain-containing p	No	0		405098	406276	+		Candidatus_Nitrososphaera_gargensis_Ga9_2_ui	NC_018719	chromosome										
===								9	Candidatus_Nitrososphaera_gargensis_Ga9_2_ui	NC_018719	chromosome										
408403164	AAA ATPase	No	0		407225	407737	+		Candidatus_Nitrososphaera_gargensis_Ga9_2_ui	NC_018719	chromosome			arCOG01308	10 (-)	O	Cdc48	ATPase of the AAA+ class , CDC48 family			
408403165	hypothetical protein Ngar_c05480	No	0		407768	408745	-		Candidatus_Nitrososphaera_gargensis_Ga9_2_ui	NC_018719	chromosome										
408403166	hypothetical protein Ngar_c05490	No	2		409180	409821	+		Candidatus_Nitrososphaera_gargensis_Ga9_2_ui	NC_018719	chromosome										
408403167	zinc finger C2H2 domain-containing protein	No	0		409827	410081	+		Candidatus_Nitrososphaera_gargensis_Ga9_2_ui	NC_018719	chromosome										
408403168	hypothetical protein Ngar_c05510	No	0		410352	410666	+		Candidatus_Nitrososphaera_gargensis_Ga9_2_ui	NC_018719	chromosome										
408403169	hypothetical protein Ngar_c05520	No	1		410818	411267	+		Candidatus_Nitrososphaera_gargensis_Ga9_2_ui	NC_018719	chromosome										
408403170	hypothetical protein Ngar_c05530	No	0		411445	411624	+		Candidatus_Nitrososphaera_gargensis_Ga9_2_ui	NC_018719	chromosome										
408403171	hypothetical protein Ngar_c05540	No	0		411785	412264	+		Candidatus_Nitrososphaera_gargensis_Ga9_2_ui	NC_018719	chromosome										
408403172	phosphoglycolate phosphatase	No	0		412685	413395	+		Candidatus_Nitrososphaera_gargensis_Ga9_2_ui	NC_018719	chromosome			arCOG01213	2 (-)	R	Cof	HAD superfamily hydrolase	1E-15	COG0561	Predicted hydrolases of the HAD superfam
===								6	Candidatus_Nitrososphaera_gargensis_Ga9_2_ui	NC_018719	chromosome										
408403209	glycosyltransferase	No	0		445418	446599	+		Candidatus_Nitrososphaera_gargensis_Ga9_2_ui	NC_018719	chromosome			arCOG06759	1 (-)	M	RfaG	Glycosyltransferase	2E-11	COG0438	Glycosyltransferase
408403210	hypothetical protein Ngar_c05930	No	0		447440	447658	+		Candidatus_Nitrososphaera_gargensis_Ga9_2_ui	NC_018719	chromosome										
408403211	hypothetical protein Ngar_c05940	No	0		447736	447927	+		Candidatus_Nitrososphaera_gargensis_Ga9_2_ui	NC_018719	chromosome										
408403212	hypothetical protein Ngar_c05950	No	0		448167	448406	+		Candidatus_Nitrososphaera_gargensis_Ga9_2_ui	NC_018719	chromosome										
408403213	hypothetical protein Ngar_c05960	No	0		448645	448839	+		Candidatus_Nitrososphaera_gargensis_Ga9_2_ui	NC_018719	chromosome										
408403214	methyltransferase	No	0		448887	449618	+		Candidatus_Nitrososphaera_gargensis_Ga9_2_ui	NC_018719	chromosome			arCOG01773	2 (-)	Q		SAM-dependent methyltransferase	7E-11	pfam08241	Methyltransferase domain. Members of thi
===								5	Candidatus_Nitrososphaera_gargensis_Ga9_2_ui	NC_018719	chromosome										
408403223	hypothetical protein Ngar_c06060	No	0		461488	461673	-		Candidatus_Nitrososphaera_gargensis_Ga9_2_ui	NC_018719	chromosome										
408403224	transposase, IS605 OrfB family	No	0		462033	462356	-		Candidatus_Nitrososphaera_gargensis_Ga9_2_ui	NC_018719	chromosome			arCOG00684	5 (-)	L		Transposase	0.0000004	COG0675	Transposase and inactivated derivatives
408403225	hypothetical protein Ngar_c06080	No	0		462845	463006	+		Candidatus_Nitrososphaera_gargensis_Ga9_2_ui	NC_018719	chromosome										
408403226	urea transporter	No	9		463062	464180	+		Candidatus_Nitrososphaera_gargensis_Ga9_2_ui	NC_018719	chromosome								2E-31	pfam03253	Urea transporter. Members of this family
408403227	hypothetical protein Ngar_c06100	No	0		464280	464885	+		Candidatus_Nitrososphaera_gargensis_Ga9_2_ui	NC_018719	chromosome										
===								11	Candidatus_Nitrososphaera_gargensis_Ga9_2_ui	NC_018719	chromosome										
408403267	ABC efflux transporter permease	No	6		495194	495973	+		Candidatus_Nitrososphaera_gargensis_Ga9_2_ui	NC_018719	chromosome			arCOG01467	2 (-)	V		ABC-type multidrug transport system, permease	3E-32	COG0842	ABC-type multidrug transport system, per
408403268	hypothetical protein Ngar_c06510	No	0		496011	496217	+		Candidatus_Nitrososphaera_gargensis_Ga9_2_ui	NC_018719	chromosome										
408403269	hypothetical protein Ngar_c06520	No	0		496514	497164	+		Candidatus_Nitrososphaera_gargensis_Ga9_2_ui	NC_018719	chromosome										
408403270	hypothetical protein Ngar_c06530	No	0		497196	497534	+		Candidatus_Nitrososphaera_gargensis_Ga9_2_ui	NC_018719	chromosome										
408403271	hypothetical protein Ngar_c06540	No	0		498043	498315	-		Candidatus_Nitrososphaera_gargensis_Ga9_2_ui	NC_018719	chromosome										
408403272	hypothetical protein Ngar_c06550	No	0		498457	498792	+		Candidatus_Nitrososphaera_gargensis_Ga9_2_ui	NC_018719	chromosome			arCOG07904	1 (-)	V		PIN domain			
408403273	hypothetical protein Ngar_c06560	No	0		499153	499446	+		Candidatus_Nitrososphaera_gargensis_Ga9_2_ui	NC_018719	chromosome										
408403274	hypothetical protein Ngar_c06570	No	1		500104	500322	-		Candidatus_Nitrososphaera_gargensis_Ga9_2_ui	NC_018719	chromosome										
408403275	hypothetical protein Ngar_c06580	No	0		500510	500788	-		Candidatus_Nitrososphaera_gargensis_Ga9_2_ui	NC_018719	chromosome										
408403276	hypothetical protein Ngar_c06590	No	0		500792	501025	-		Candidatus_Nitrososphaera_gargensis_Ga9_2_ui	NC_018719	chromosome										
408403277	hypothetical protein Ngar_c06600	No	0		501399	501692	-		Candidatus_Nitrososphaera_gargensis_Ga9_2_ui	NC_018719	chromosome			arCOG00818	3 (-)	K	AbrB	Transcriptional regulator AbrB			
===								7	Candidatus_Nitrososphaera_gargensis_Ga9_2_ui	NC_018719	chromosome										
408403295	hypothetical protein Ngar_c06780	No	0		516218	516580	-		Candidatus_Nitrososphaera_gargensis_Ga9_2_ui	NC_018719	chromosome	clust_295									
408403296	archaeal preflagellin peptidase FlaK	No	6		517102	517851	+		Candidatus_Nitrososphaera_gargensis_Ga9_2_ui	NC_018719	chromosome			arCOG02298	6 (-)	N	FlaK/PulC	Peptidase A24A, prepilin type IV			
408403297	hypothetical protein Ngar_c06800	No	0		517917	518129	-		Candidatus_Nitrososphaera_gargensis_Ga9_2_ui	NC_018719	chromosome	clust_439									
408403298	hypothetical protein Ngar_c06810	No	0		518176	518343	+		Candidatus_Nitrososphaera_gargensis_Ga9_2_ui	NC_018719	chromosome										
408403299	zinc finger C2H2 domain-containing protein	No	0		518566	519153	+		Candidatus_Nitrososphaera_gargensis_Ga9_2_ui	NC_018719	chromosome										
408403300	hypothetical protein Ngar_c06830	No	0		519146	519550	+		Candidatus_Nitrososphaera_gargensis_Ga9_2_ui	NC_018719	chromosome			arCOG08684	7 (-)	R		Roadblock/LC7 domain superfamily protein			
408403301	hypothetical protein Ngar_c06840	No	0		519685	519882	-		Candidatus_Nitrososphaera_gargensis_Ga9_2_ui	NC_018719	chromosome										
===								7	Candidatus_Nitrososphaera_gargensis_Ga9_2_ui	NC_018719	chromosome										
408403308	hypothetical protein Ngar_c06910	No	0		525363	526784	-		Candidatus_Nitrososphaera_gargensis_Ga9_2_ui	NC_018719	chromosome			arCOG03287	4 (-)	G	GDB1	Glycogen debranching enzyme			
408403309	zinc finger C2H2 domain-containing protein	No	0		526833	527030	+		Candidatus_Nitrososphaera_gargensis_Ga9_2_ui	NC_018719	chromosome										
408403310	acetylglutamate kinase	No	0		527137	527544	-		Candidatus_Nitrososphaera_gargensis_Ga9_2_ui	NC_018719	chromosome										
408403311	hypothetical protein Ngar_c06940	No	1		527693	527851	-		Candidatus_Nitrososphaera_gargensis_Ga9_2_ui	NC_018719	chromosome										
408403312	hypothetical protein Ngar_c06950	No	0		527907	528167	+		Candidatus_Nitrososphaera_gargensis_Ga9_2_ui	NC_018719	chromosome										
408403313	hypothetical protein Ngar_c06960	No	0		528761	529330	+		Candidatus_Nitrososphaera_gargensis_Ga9_2_ui	NC_018719	chromosome			arCOG03287	4 (-)	G	GDB1	Glycogen debranching enzyme			
408403314	hypothetical protein Ngar_c06970	No	0		529602	529856	+		Candidatus_Nitrososphaera_gargensis_Ga9_2_ui	NC_018719	chromosome										
===								7	Candidatus_Nitrososphaera_gargensis_Ga9_2_ui	NC_018719	chromosome										
408403357	NurA domain-containing protein	No	0		568898	569926	-		Candidatus_Nitrososphaera_gargensis_Ga9_2_ui	NC_018719	chromosome			arCOG00367	3 (-)	L		NurA 5'-3' nuclease			
408403358	hypothetical protein Ngar_c07410	No	0		570026	570520	+		Candidatus_Nitrososphaera_gargensis_Ga9_2_ui	NC_018719	chromosome										
408403359	hypothetical protein Ngar_c07420	No	0		570507	571016	-		Candidatus_Nitrososphaera_gargensis_Ga9_2_ui	NC_018719	chromosome										
408403360	hypothetical protein Ngar_c07430	No	2		571447	571836	-		Candidatus_Nitrososphaera_gargensis_Ga9_2_ui	NC_018719	chromosome										
408403361	abortive infection protein	No	4		572138	572770	-		Candidatus_Nitrososphaera_gargensis_Ga9_2_ui	NC_018719	chromosome										
408403362	hypothetical protein Ngar_c07450	No	0		572822	573148	-		Candidatus_Nitrososphaera_gargensis_Ga9_2_ui	NC_018719	chromosome										
408403363	glutamyl-tRNA(Gln) amidotransferase subunit E	No	0		573154	575052	-		Candidatus_Nitrososphaera_gargensis_Ga9_2_ui	NC_018719	chromosome			arCOG01719	3 (-)	J	GatE	Archaeal Glu-tRNAGln amidotransferase subunit E (contains GAI		COG2511	Archaeal Glu-tRNAGln amidotransferase su
===								17	Candidatus_Nitrososphaera_gargensis_Ga9_2_ui	NC_018719	chromosome										
408403386	hypothetical protein Ngar_c07700	No	0		591174	591539	-		Candidatus_Nitrososphaera_gargensis_Ga9_2_ui	NC_018719	chromosome			arCOG08684	7 (-)	R		Roadblock/LC7 domain superfamily protein			
408403387	hypothetical protein Ngar_c07710	No	2		592159	592503	+		Candidatus_Nitrososphaera_gargensis_Ga9_2_ui	NC_018719	chromosome										
408403388	hypothetical protein Ngar_c07720	No	0		592795	592914	-		Candidatus_Nitrososphaera_gargensis_Ga9_2_ui	NC_018719	chromosome	clust_510									
408403389	hypothetical protein Ngar_c07730	No	0		593680	594000	-		Candidatus_Nitrososphaera_gargensis_Ga9_2_ui	NC_018719	chromosome										
408403390	hypothetical protein Ngar_c07740	No	1		594430	594543	+		Candidatus_Nitrososphaera_gargensis_Ga9_2_ui	NC_018719	chromosome										
408403391	CDGSH-type zinc finger protein	No	0		595143	595286	+		Candidatus_Nitrososphaera_gargensis_Ga9_2_ui	NC_018719	chromosome										
408403392	hypothetical protein Ngar_c07760	No	0		595376	595645	+		Candidatus_Nitrososphaera_gargensis_Ga9_2_ui	NC_018719	chromosome			arCOG01055	9 (-)	K		Predicted transcriptional regulator	0.000009	COG3432	Predicted transcriptional regulator
408403393	CDGSH-type zinc finger protein	No	0		595778	596479	+		Candidatus_Nitrososphaera_gargensis_Ga9_2_ui	NC_018719	chromosome	clust_141							0.0000006	COG1378	Predicted transcriptional regulators
408403394	hypothetical protein Ngar_c07780	No	0		596537	596761	-		Candidatus_Nitrososphaera_gargensis_Ga9_2_ui	NC_018719	chromosome										
408403395	hypothetical protein Ngar_c07790	No	0		597185	597613	-		Candidatus_Nitrososphaera_gargensis_Ga9_2_ui	NC_018719	chromosome										
408403396	hypothetical protein Ngar_c07800	No	1		597659	597910	+		Candidatus_Nitrososphaera_gargensis_Ga9_2_ui	NC_018719	chromosome										
408403397	hypothetical protein Ngar_c07810	No	0		598356	598520	+		Candidatus_Nitrososphaera_gargensis_Ga9_2_ui	NC_018719	chromosome										
408403398	hypothetical protein Ngar_c07820	No	0		598787	599041	-		Candidatus_Nitrososphaera_gargensis_Ga9_2_ui	NC_018719	chromosome										
408403399	hypothetical protein Ngar_c07830	No	0		599139	599498	+		Candidatus_Nitrososphaera_gargensis_Ga9_2_ui	NC_018719	chromosome			arCOG08684	7 (-)	R		Roadblock/LC7 domain superfamily protein			
408403400	hypothetical protein Ngar_c07840	No	0		599488	599874	-		Candidatus_Nitrososphaera_gargensis_Ga9_2_ui	NC_018719	chromosome										
408403401	hypothetical protein Ngar_c07850	No	0		599916	600665	-		Candidatus_Nitrososphaera_gargensis_Ga9_2_ui	NC_018719	chromosome										
408403402	hypothetical protein Ngar_c07860	No	1		601163	601321	-		Candidatus_Nitrososphaera_gargensis_Ga9_2_ui	NC_018719	chromosome										
===								12	Candidatus_Nitrososphaera_gargensis_Ga9_2_ui	NC_018719	chromosome										
408403409	hypothetical protein Ngar_c07930	No	0		606266	606430	+		Candidatus_Nitrososphaera_gargensis_Ga9_2_ui	NC_018719	chromosome			arCOG04291	2 (-)	R		Sugar isomerase related protein	0.0000001	pfam01904	Protein of unknown function DUF72. The f
408403410	hypothetical protein Ngar_c07940	No	0		606746	606970	-		Candidatus_Nitrososphaera_gargensis_Ga9_2_ui	NC_018719	chromosome										
408403411	hypothetical protein Ngar_c07950	No	0		607357	607572	-		Candidatus_Nitrososphaera_gargensis_Ga9_2_ui	NC_018719	chromosome										
408403412	hypothetical protein Ngar_c07960	No	0		607716	607907	-		Candidatus_Nitrososphaera_gargensis_Ga9_2_ui	NC_018719	chromosome										
408403413	hypothetical protein Ngar_c07970	No	0		608094	608561	-		Candidatus_Nitrososphaera_gargensis_Ga9_2_ui	NC_018719	chromosome										
408403414	hypothetical protein Ngar_c07980	No	0		608672	609022	-		Candidatus_Nitrososphaera_gargensis_Ga9_2_ui	NC_018719	chromosome	clust_305									
408403415	hypothetical protein Ngar_c07990	No	0		609423	609554	-		Candidatus_Nitrososphaera_gargensis_Ga9_2_ui	NC_018719	chromosome										
408403416	hypothetical protein Ngar_c08000	No	0		609582	609716	-		Candidatus_Nitrososphaera_gargensis_Ga9_2_ui	NC_018719	chromosome										
408403417	transcription regulator, containing CBS domain	No	0		610124	610738	-		Candidatus_Nitrososphaera_gargensis_Ga9_2_ui	NC_018719	chromosome			arCOG00608	3 (-)	K		Predicted transcriptional regulator with C-terminal	7E-33	COG3620	Predicted transcriptional regulator with
408403418	hypothetical protein Ngar_c08020	No	0		610829	611146	+		Candidatus_Nitrososphaera_gargensis_Ga9_2_ui	NC_018719	chromosome										
408403419	hypothetical protein Ngar_c08030	No	0		611235	611402	+		Candidatus_Nitrososphaera_gargensis_Ga9_2_ui	NC_018719	chromosome										
408403420	hypothetical protein Ngar_c08040	No	0		611429	611998	+		Candidatus_Nitrososphaera_gargensis_Ga9_2_ui	NC_018719	chromosome										
===								8	Candidatus_Nitrososphaera_gargensis_Ga9_2_ui	NC_018719	chromosome										
408403438	ATPase, P-loop superfamily	No	0		628032	628979	+		Candidatus_Nitrososphaera_gargensis_Ga9_2_ui	NC_018719	chromosome			arCOG00042	4 (-)	J	MesJ	tRNA(Ile)-lysidine synthase MesJ	2E-34	COG0037	tRNA(Ile)-lysidine synthase MesJ
408403439	hemolysin-type calcium-binding repeat protein	Yes	1		629611	630354	-		Candidatus_Nitrososphaera_gargensis_Ga9_2_ui	NC_018719	chromosome								2E-32	COG2931	RTX toxins and related Ca2+-binding prot
408403440	antibiotic biosynthesis monooxygenase	No	0		630515	630778	+		Candidatus_Nitrososphaera_gargensis_Ga9_2_ui	NC_018719	chromosome										
408403441	hypothetical protein Ngar_c08250	No	1		631011	631304	+		Candidatus_Nitrososphaera_gargensis_Ga9_2_ui	NC_018719	chromosome										
408403442	hypothetical protein Ngar_c08260	No	0		631784	631963	+		Candidatus_Nitrososphaera_gargensis_Ga9_2_ui	NC_018719	chromosome										
408403443	hypothetical protein Ngar_c08270	No	0		632665	632868	-		Candidatus_Nitrososphaera_gargensis_Ga9_2_ui	NC_018719	chromosome										
408403444	hypothetical protein Ngar_c08280	No	0		633178	633399	+		Candidatus_Nitrososphaera_gargensis_Ga9_2_ui	NC_018719	chromosome										
408403445	hypothetical protein Ngar_c08290	No	0		633563	633856	+		Candidatus_Nitrososphaera_gargensis_Ga9_2_ui	NC_018719	chromosome			arCOG01055	9 (-)	K		Predicted transcriptional regulator	0.00000004	COG3432	Predicted transcriptional regulator
===								12	Candidatus_Nitrososphaera_gargensis_Ga9_2_ui	NC_018719	chromosome										
408403458	diacylglycerol kinase catalytic region	No	0		644109	644291	+		Candidatus_Nitrososphaera_gargensis_Ga9_2_ui	NC_018719	chromosome										
408403459	diacylglycerol kinase catalytic region	No	0		644444	644647	+		Candidatus_Nitrososphaera_gargensis_Ga9_2_ui	NC_018719	chromosome										
408403460	diacylglycerol kinase catalytic region	No	0		644653	645087	+		Candidatus_Nitrososphaera_gargensis_Ga9_2_ui	NC_018719	chromosome			arCOG08932	1 (-)	I	LCB5	Diacylglycerol kinase family enzyme	1E-15	COG1597	Sphingosine kinase and enzymes related t
408403461	hypothetical protein Ngar_c08450	No	0		645099	645590	-		Candidatus_Nitrososphaera_gargensis_Ga9_2_ui	NC_018719	chromosome										
408403462	hypothetical protein Ngar_c08460	No	2		645631	646203	+		Candidatus_Nitrososphaera_gargensis_Ga9_2_ui	NC_018719	chromosome	clust_184									
408403463	hypothetical protein Ngar_c08470	No	0		646204	646398	-		Candidatus_Nitrososphaera_gargensis_Ga9_2_ui	NC_018719	chromosome										
408403464	hypothetical protein Ngar_c08480	No	0		646637	647092	-		Candidatus_Nitrososphaera_gargensis_Ga9_2_ui	NC_018719	chromosome	clust_226							0.000002	COG0840	Methyl-accepting chemotaxis protein
408403465	hypothetical protein Ngar_c08490	No	0		647143	647679	-		Candidatus_Nitrososphaera_gargensis_Ga9_2_ui	NC_018719	chromosome	clust_197									
408403466	hypothetical protein Ngar_c08500	No	2		648027	648368	-		Candidatus_Nitrososphaera_gargensis_Ga9_2_ui	NC_018719	chromosome										
408403467	hypothetical protein Ngar_c08510	No	0		648481	649032	+		Candidatus_Nitrososphaera_gargensis_Ga9_2_ui	NC_018719	chromosome								0.00004	COG3064	Membrane protein involved in colicin upt
408403468	signal transduction response regulator, receive	No	0		649311	649721	-		Candidatus_Nitrososphaera_gargensis_Ga9_2_ui	NC_018719	chromosome			arCOG02391	3 (-)	T	CheY	Rec domain	9E-15	COG2204	Response regulator containing CheY-like
408403469	hypothetical protein Ngar_c08530	No	1		649767	649985	+		Candidatus_Nitrososphaera_gargensis_Ga9_2_ui	NC_018719	chromosome										
===								8	Candidatus_Nitrososphaera_gargensis_Ga9_2_ui	NC_018719	chromosome										
408403476	arsR family transcriptional regulator	No	0		657099	657521	-		Candidatus_Nitrososphaera_gargensis_Ga9_2_ui	NC_018719	chromosome			arCOG00731	3 (-)	K	ArsR	Transcriptional regulator containing HTH domain, ArsR family			
408403477	hypothetical protein Ngar_c08610	No	1		657633	658667	+		Candidatus_Nitrososphaera_gargensis_Ga9_2_ui	NC_018719	chromosome										
408403478	hypothetical protein Ngar_c08620	No	0		658836	658982	-		Candidatus_Nitrososphaera_gargensis_Ga9_2_ui	NC_018719	chromosome										
408403479	hypothetical protein Ngar_c08630	Yes	0		659494	659646	-		Candidatus_Nitrososphaera_gargensis_Ga9_2_ui	NC_018719	chromosome										
408403480	hypothetical protein Ngar_c08640	No	0		659989	660276	-		Candidatus_Nitrososphaera_gargensis_Ga9_2_ui	NC_018719	chromosome										
408403481	hypothetical protein Ngar_c08650	No	0		660331	660621	-		Candidatus_Nitrososphaera_gargensis_Ga9_2_ui	NC_018719	chromosome										
408403482	3-isopropylmalate dehydratase	No	1		660939	661853	-		Candidatus_Nitrososphaera_gargensis_Ga9_2_ui	NC_018719	chromosome			arCOG00347	2 (-)	R		Archaeal enzyme of ATP-grasp superfamily	8E-36	COG1938	Archaeal enzymes of ATP-grasp superfamil
408403483	hypothetical protein Ngar_c08670	Yes	0		661860	662060	-		Candidatus_Nitrososphaera_gargensis_Ga9_2_ui	NC_018719	chromosome										
===								5	Candidatus_Nitrososphaera_gargensis_Ga9_2_ui	NC_018719	chromosome										
408403494	hypothetical protein Ngar_c08780	No	0		674218	674448	+		Candidatus_Nitrososphaera_gargensis_Ga9_2_ui	NC_018719	chromosome										
408403495	hypothetical protein Ngar_c08790	No	0		674454	674687	+		Candidatus_Nitrososphaera_gargensis_Ga9_2_ui	NC_018719	chromosome										
408403496	hypothetical protein Ngar_c08800	No	1		675316	675885	-		Candidatus_Nitrososphaera_gargensis_Ga9_2_ui	NC_018719	chromosome										
408403497	transposase, IS605 OrfB	No	0		675967	677016	+		Candidatus_Nitrososphaera_gargensis_Ga9_2_ui	NC_018719	chromosome			arCOG00684	5 (-)	L		Transposase	1E-28	COG0675	Transposase and inactivated derivatives
408403498	hypothetical protein Ngar_c08820	Yes	1		677093	677683	+		Candidatus_Nitrososphaera_gargensis_Ga9_2_ui	NC_018719	chromosome			arCOG08821	18	S		Uncharacterized conserved protein (expansion in Thaumarchaeota)			
===								5	Candidatus_Nitrososphaera_gargensis_Ga9_2_ui	NC_018719	chromosome										
408403548	hypothetical protein Ngar_c09320	No	0		723145	723282	-		Candidatus_Nitrososphaera_gargensis_Ga9_2_ui	NC_018719	chromosome										
408403549	hypothetical protein Ngar_c09330	No	0		723336	723512	+		Candidatus_Nitrososphaera_gargensis_Ga9_2_ui	NC_018719	chromosome	clust_481									
408403550	hypothetical protein Ngar_c09340	No	0		723800	724012	+		Candidatus_Nitrososphaera_gargensis_Ga9_2_ui	NC_018719	chromosome	clust_440									
408403551	transposase IS116/IS110/IS902 family	No	0		724263	724688	+		Candidatus_Nitrososphaera_gargensis_Ga9_2_ui	NC_018719	chromosome			arCOG03585	6 (-)	L		Transposase	0.00002	pfam02371	Transposase IS116/IS110/IS902 family. Tr
408403552	hypothetical protein Ngar_c09360	No	0		724648	724830	+		Candidatus_Nitrososphaera_gargensis_Ga9_2_ui	NC_018719	chromosome	clust_477									
===								5	Candidatus_Nitrososphaera_gargensis_Ga9_2_ui	NC_018719	chromosome										
408403555	hypothetical protein Ngar_c09390	No	0		726740	727216	+		Candidatus_Nitrososphaera_gargensis_Ga9_2_ui	NC_018719	chromosome										
408403556	hypothetical protein Ngar_c09400	No	17		727504	729414	-		Candidatus_Nitrososphaera_gargensis_Ga9_2_ui	NC_018719	chromosome								0.000001	pfam02516	Oligosaccharyl transferase STT3 subunit.
408403557	NAD-dependent epimerase/dehydratase family	No	0		729592	730533	-		Candidatus_Nitrososphaera_gargensis_Ga9_2_ui	NC_018719	chromosome			arCOG01369	5 (-)	M	WcaG	Nucleoside-diphosphate-sugar epimerase	2E-52	COG0451	Nucleoside-diphosphate-sugar epimerases
408403558	transposase	No	0		730663	730815	+		Candidatus_Nitrososphaera_gargensis_Ga9_2_ui	NC_018719	chromosome			arCOG10890	2	S		Uncharacterized conserved protein			
408403559	hypothetical protein Ngar_c09430	No	0		730821	731048	+		Candidatus_Nitrososphaera_gargensis_Ga9_2_ui	NC_018719	chromosome										
===								5	Candidatus_Nitrososphaera_gargensis_Ga9_2_ui	NC_018719	chromosome										
408403574	hypothetical protein Ngar_c09580	No	0		742347	742634	+		Candidatus_Nitrososphaera_gargensis_Ga9_2_ui	NC_018719	chromosome										
408403575	hypothetical protein Ngar_c09590	No	0		742779	743015	+		Candidatus_Nitrososphaera_gargensis_Ga9_2_ui	NC_018719	chromosome										
408403576	hypothetical protein Ngar_c09600	Yes	2		743040	744776	+		Candidatus_Nitrososphaera_gargensis_Ga9_2_ui	NC_018719	chromosome			arCOG08033	4 (-)	S		Uncharacterized protein			
408403577	hypothetical protein Ngar_c09610	No	0		744888	745031	-		Candidatus_Nitrososphaera_gargensis_Ga9_2_ui	NC_018719	chromosome										
408403578	transposase, IS66 family	No	0		745383	745577	-		Candidatus_Nitrososphaera_gargensis_Ga9_2_ui	NC_018719	chromosome										
===								8	Candidatus_Nitrososphaera_gargensis_Ga9_2_ui	NC_018719	chromosome										
408403588	hypothetical protein Ngar_c09720	No	2		753559	753882	+		Candidatus_Nitrososphaera_gargensis_Ga9_2_ui	NC_018719	chromosome			arCOG08750	1 (-)	S		Zn finger protein			
408403589	hypothetical protein Ngar_c09730	No	0		753964	754113	+		Candidatus_Nitrososphaera_gargensis_Ga9_2_ui	NC_018719	chromosome										
408403590	hypothetical protein Ngar_c09740	No	0		754127	754243	+		Candidatus_Nitrososphaera_gargensis_Ga9_2_ui	NC_018719	chromosome	clust_508									
408403591	hypothetical protein Ngar_c09750	No	0		754308	754844	-		Candidatus_Nitrososphaera_gargensis_Ga9_2_ui	NC_018719	chromosome	clust_192							0.00008	pfam01576	Myosin tail. The myosin molecule is a mu
408403592	hypothetical protein Ngar_c09760	No	0		755572	755688	-		Candidatus_Nitrososphaera_gargensis_Ga9_2_ui	NC_018719	chromosome										
408403593	DNA protection protein DPS	No	0		756776	757384	+		Candidatus_Nitrososphaera_gargensis_Ga9_2_ui	NC_018719	chromosome			arCOG01093	1 (-)	R		Protein distantly related to bacterial ferritins	9E-74	COG2406	Protein distantly related to bacterial f
408403594	hypothetical protein Ngar_c09780	No	0		757659	758117	-		Candidatus_Nitrososphaera_gargensis_Ga9_2_ui	NC_018719	chromosome										
408403595	hypothetical protein Ngar_c09790	No	0		758201	758479	-		Candidatus_Nitrososphaera_gargensis_Ga9_2_ui	NC_018719	chromosome										
===								19	Candidatus_Nitrososphaera_gargensis_Ga9_2_ui	NC_018719	chromosome										
408403600	phosphate uptake regulator, PhoU	No	0		763936	764598	+		Candidatus_Nitrososphaera_gargensis_Ga9_2_ui	NC_018719	chromosome			arCOG00232	2 (-)	P	PhoU	Phosphate uptake regulator	6E-20	COG0704	Phosphate uptake regulator
408403601	hypothetical protein Ngar_c09850	No	0		764669	764860	-		Candidatus_Nitrososphaera_gargensis_Ga9_2_ui	NC_018719	chromosome										
408403602	hypothetical protein Ngar_c09860	No	0		765213	765554	+		Candidatus_Nitrososphaera_gargensis_Ga9_2_ui	NC_018719	chromosome										
408403603	hypothetical protein Ngar_c09870	No	0		765585	765737	-		Candidatus_Nitrososphaera_gargensis_Ga9_2_ui	NC_018719	chromosome										
408403604	hypothetical protein Ngar_c09880	No	0		765885	766094	-		Candidatus_Nitrososphaera_gargensis_Ga9_2_ui	NC_018719	chromosome										
408403605	hypothetical protein Ngar_c09890	No	0		766140	766268	-		Candidatus_Nitrososphaera_gargensis_Ga9_2_ui	NC_018719	chromosome										
408403606	hypothetical protein Ngar_c09900	No	0		766574	766975	+		Candidatus_Nitrososphaera_gargensis_Ga9_2_ui	NC_018719	chromosome										
408403607	IS-4 family transposase	No	0		766952	768169	-		Candidatus_Nitrososphaera_gargensis_Ga9_2_ui	NC_018719	chromosome			arCOG03902	8 (-)	L		Transposase			
408403608	hypothetical protein Ngar_c09920	No	0		768280	768597	+		Candidatus_Nitrososphaera_gargensis_Ga9_2_ui	NC_018719	chromosome										
408403609	hypothetical protein Ngar_c09930	Yes	1		769076	769309	+		Candidatus_Nitrososphaera_gargensis_Ga9_2_ui	NC_018719	chromosome										
408403610	hypothetical protein Ngar_c09940	No	0		769655	770362	+		Candidatus_Nitrososphaera_gargensis_Ga9_2_ui	NC_018719	chromosome										
408403611	hypothetical protein Ngar_c09950	No	0		770443	770724	+		Candidatus_Nitrososphaera_gargensis_Ga9_2_ui	NC_018719	chromosome										
408403612	hypothetical protein Ngar_c09960	No	0		770721	770897	+		Candidatus_Nitrososphaera_gargensis_Ga9_2_ui	NC_018719	chromosome										
408403613	signal transduction histidine kinase, with phosp	No	0		771239	773437	-		Candidatus_Nitrososphaera_gargensis_Ga9_2_ui	NC_018719	chromosome			arCOG02358	4 (-)	T		Signal transduction histidine kinase and HAMP d	3E-34	COG0642	Signal transduction histidine kinase
408403614	zinc finger C2H2 domain-containing protein	No	0		773934	774647	-		Candidatus_Nitrososphaera_gargensis_Ga9_2_ui	NC_018719	chromosome	clust_141									
408403615	zinc finger C2H2 domain-containing protein	No	0		774879	775130	-		Candidatus_Nitrososphaera_gargensis_Ga9_2_ui	NC_018719	chromosome			arCOG08662	3 (-)	S		Uncharacterized conserved metal-binding protein			
408403616	hypothetical protein Ngar_c10000	No	0		775309	775443	+		Candidatus_Nitrososphaera_gargensis_Ga9_2_ui	NC_018719	chromosome										
408403617	hypothetical protein Ngar_c10010	No	0		775724	775846	+		Candidatus_Nitrososphaera_gargensis_Ga9_2_ui	NC_018719	chromosome	clust_508									
408403618	hypothetical protein Ngar_c10020	No	0		775914	776462	-		Candidatus_Nitrososphaera_gargensis_Ga9_2_ui	NC_018719	chromosome	clust_192									
===								5	Candidatus_Nitrososphaera_gargensis_Ga9_2_ui	NC_018719	chromosome										
408403640	hypothetical protein Ngar_c10240	No	1		797581	797991	+		Candidatus_Nitrososphaera_gargensis_Ga9_2_ui	NC_018719	chromosome										
408403641	hypothetical protein Ngar_c10250	No	0		798094	798345	+		Candidatus_Nitrososphaera_gargensis_Ga9_2_ui	NC_018719	chromosome										
408403642	hypothetical protein Ngar_c10260	No	0		798376	798534	-		Candidatus_Nitrososphaera_gargensis_Ga9_2_ui	NC_018719	chromosome										
408403643	transposase	No	0		799302	800246	+		Candidatus_Nitrososphaera_gargensis_Ga9_2_ui	NC_018719	chromosome			arCOG00683	3 (-)	L		Transposase	4E-21	pfam01385	Probable transposase. This family includ
408403644	hypothetical protein Ngar_c10280	Yes	1		800457	800990	+		Candidatus_Nitrososphaera_gargensis_Ga9_2_ui	NC_018719	chromosome										
===								11	Candidatus_Nitrososphaera_gargensis_Ga9_2_ui	NC_018719	chromosome										
408403682	methionine--tRNA ligase	No	0		835049	835396	+		Candidatus_Nitrososphaera_gargensis_Ga9_2_ui	NC_018719	chromosome			arCOG01136	1 (-)	R	ARC1	EMAP domain RNA-binding protein	3E-15	pfam01588	Putative tRNA binding domain. This domai
408403683	hypothetical protein Ngar_c10670	No	0		835611	835787	+		Candidatus_Nitrososphaera_gargensis_Ga9_2_ui	NC_018719	chromosome										
408403684	hypothetical protein Ngar_c10680	No	4		835890	836399	+		Candidatus_Nitrososphaera_gargensis_Ga9_2_ui	NC_018719	chromosome										
408403685	hypothetical protein Ngar_c10690	No	0		836599	837099	-		Candidatus_Nitrososphaera_gargensis_Ga9_2_ui	NC_018719	chromosome			arCOG11117	1	S		Uncharacterized conserved protein	0.00001	pfam07485	Domain of Unknown Function (DUF1259). Th
408403686	hypothetical protein Ngar_c10700	Yes	1		837173	837403	+		Candidatus_Nitrososphaera_gargensis_Ga9_2_ui	NC_018719	chromosome										
408403687	hypothetical protein Ngar_c10710	No	0		837557	837739	-		Candidatus_Nitrososphaera_gargensis_Ga9_2_ui	NC_018719	chromosome			arCOG02053	7 (-)	T	UspA	Nucleotide-binding protein, UspA family	0.00007	pfam00582	Universal stress protein family. The uni
408403688	hypothetical protein Ngar_c10720	No	0		837788	837943	-		Candidatus_Nitrososphaera_gargensis_Ga9_2_ui	NC_018719	chromosome										

	408403689	hypothetical protein Ngar_c10730	No	0	838309	838488	+		Candidatus_Nitrososphaera_gargensis_Ga9_2_ui	NC_018719	chromosome									
	408403690	Cdc48 ATPase, partial	No	0	838481	838675	+		Candidatus_Nitrososphaera_gargensis_Ga9_2_ui	NC_018719	chromosome		arCOG01308	10 (-)	O	Cdc48	ATPase of the AAA+ class , CDC48 family			
	408403691	hypothetical protein Ngar_c10750	No	0	838723	839100	+		Candidatus_Nitrososphaera_gargensis_Ga9_2_ui	NC_018719	chromosome									
	408403692	hypothetical protein Ngar_c10760	No	4	839117	839593	-		Candidatus_Nitrososphaera_gargensis_Ga9_2_ui	NC_018719	chromosome									
===								6	Candidatus_Nitrososphaera_gargensis_Ga9_2_ui	NC_018719	chromosome									
	408403706	phosphoesterase PA-phosphatase	No	6	850232	850966	+		Candidatus_Nitrososphaera_gargensis_Ga9_2_ui	NC_018719	chromosome		arCOG03056	1 (-)	I	PgpB	Membrane-associated phospholipid phosphatas	1E-12	pfam01569	PAP2 superfamily. This family includes t
	408403707	hypothetical protein Ngar_c10910	No	1	851046	851258	+		Candidatus_Nitrososphaera_gargensis_Ga9_2_ui	NC_018719	chromosome									
	408403708	hypothetical protein Ngar_c10920	No	0	851310	851639	+		Candidatus_Nitrososphaera_gargensis_Ga9_2_ui	NC_018719	chromosome									
	408403709	hypothetical protein Ngar_c10930	No	0	851758	851943	+		Candidatus_Nitrososphaera_gargensis_Ga9_2_ui	NC_018719	chromosome									
	408403710	hypothetical protein Ngar_c10940	No	0	852159	852341	+		Candidatus_Nitrososphaera_gargensis_Ga9_2_ui	NC_018719	chromosome									
	408403711	TatA subunit of twin-arginine targeting system	No	1	852466	852816	+		Candidatus_Nitrososphaera_gargensis_Ga9_2_ui	NC_018719	chromosome		arCOG02694	2 (-)	U	TatA	Sec-independent protein secretion pathway com	0.000000005	COG1826	Sec-independent protein secretion pathwa
===								9	Candidatus_Nitrososphaera_gargensis_Ga9_2_ui	NC_018719	chromosome									
	408403741	hypothetical protein Ngar_c11250	No	1	878200	878322	+		Candidatus_Nitrososphaera_gargensis_Ga9_2_ui	NC_018719	chromosome									
	408403742	blue (type1) copper domain-containing protein	No	1	878331	878804	-		Candidatus_Nitrososphaera_gargensis_Ga9_2_ui	NC_018719	chromosome		arCOG02926	8 (-)	C	PetE	Plastocyanin	0.000002	COG3794	Plastocyanin
	408403743	hypothetical protein Ngar_c11270	Yes	0	878897	879391	-		Candidatus_Nitrososphaera_gargensis_Ga9_2_ui	NC_018719	chromosome									
	408403744	hypothetical protein Ngar_c11280	Yes	2	879448	879945	-		Candidatus_Nitrososphaera_gargensis_Ga9_2_ui	NC_018719	chromosome									
	408403745	hypothetical protein Ngar_c11290	No	0	880063	880692	-		Candidatus_Nitrososphaera_gargensis_Ga9_2_ui	NC_018719	chromosome									
	408403746	hypothetical protein Ngar_c11300	No	0	880739	881407	-		Candidatus_Nitrososphaera_gargensis_Ga9_2_ui	NC_018719	chromosome									
	408403747	hypothetical protein Ngar_c11310	No	0	881509	881679	+		Candidatus_Nitrososphaera_gargensis_Ga9_2_ui	NC_018719	chromosome									
	408403748	hypothetical protein Ngar_c11320	No	0	881772	882431	+		Candidatus_Nitrososphaera_gargensis_Ga9_2_ui	NC_018719	chromosome									
	408403749	hypothetical protein Ngar_c11330	No	0	882436	882966	+		Candidatus_Nitrososphaera_gargensis_Ga9_2_ui	NC_018719	chromosome		arCOG04119	1 (-)	R		Predicted nucleotidyltransferase			
===								8	Candidatus_Nitrososphaera_gargensis_Ga9_2_ui	NC_018719	chromosome									
	408403782	hypothetical protein Ngar_c11680	No	0	911696	912064	+		Candidatus_Nitrososphaera_gargensis_Ga9_2_ui	NC_018719	chromosome		arCOG02808	1 (-)	K		Predicted transcriptional regulator			
	408403783	hypothetical protein Ngar_c11690	Yes	0	912268	912624	+		Candidatus_Nitrososphaera_gargensis_Ga9_2_ui	NC_018719	chromosome									
	408403784	hypothetical protein Ngar_c11700	No	0	912669	914726	+		Candidatus_Nitrososphaera_gargensis_Ga9_2_ui	NC_018719	chromosome									
	408403785	hypothetical protein Ngar_c11710	No	0	914766	916070	+		Candidatus_Nitrososphaera_gargensis_Ga9_2_ui	NC_018719	chromosome									
	408403786	hypothetical protein Ngar_c11720	No	0	916099	916641	-		Candidatus_Nitrososphaera_gargensis_Ga9_2_ui	NC_018719	chromosome									
	408403787	short signal transduction response regulator, re	No	0	917595	917780	+		Candidatus_Nitrososphaera_gargensis_Ga9_2_ui	NC_018719	chromosome		arCOG02391	3 (-)	T	CheY	Rec domain	0.00003	COG2204	Response regulator containing CheY-like
	408403788	hypothetical protein Ngar_c11740	Yes	1	918127	919170	-		Candidatus_Nitrososphaera_gargensis_Ga9_2_ui	NC_018719	chromosome									
	408403789	hypothetical protein Ngar_c11750	No	0	919575	919760	+		Candidatus_Nitrososphaera_gargensis_Ga9_2_ui	NC_018719	chromosome									
===								11	Candidatus_Nitrososphaera_gargensis_Ga9_2_ui	NC_018719	chromosome									
	408403806	hypothetical protein Ngar_c11920	No	0	933201	933386	+		Candidatus_Nitrososphaera_gargensis_Ga9_2_ui	NC_018719	chromosome									
	408403807	hypothetical protein Ngar_c11930	No	0	933624	933788	-		Candidatus_Nitrososphaera_gargensis_Ga9_2_ui	NC_018719	chromosome									
	408403808	hypothetical protein Ngar_c11940	No	0	934157	934507	-		Candidatus_Nitrososphaera_gargensis_Ga9_2_ui	NC_018719	chromosome									
	408403809	hypothetical protein Ngar_c11950	No	1	934584	935381	-		Candidatus_Nitrososphaera_gargensis_Ga9_2_ui	NC_018719	chromosome		arCOG00347	2 (-)	R		Archaeal enzyme of ATP-grasp superfamily	8E-37	COG1938	Archaeal enzymes of ATP-grasp superfamil
	408403810	hypothetical protein Ngar_c11960	No	0	935381	935719	-		Candidatus_Nitrososphaera_gargensis_Ga9_2_ui	NC_018719	chromosome									
	408403811	hypothetical protein Ngar_c11970	No	2	935926	936210	-		Candidatus_Nitrososphaera_gargensis_Ga9_2_ui	NC_018719	chromosome									
	408403812	hypothetical protein Ngar_c11980	No	0	936242	936478	-		Candidatus_Nitrososphaera_gargensis_Ga9_2_ui	NC_018719	chromosome									
	408403813	NADH dehydrogenase/NAD(P)H nitroreductase	No	0	936722	937243	-		Candidatus_Nitrososphaera_gargensis_Ga9_2_ui	NC_018719	chromosome		arCOG00288	8 (-)	C	NfnB	Nitroreductase	2E-20	COG0778	Nitroreductase
	408403814	hypothetical protein Ngar_c12000	No	0	937263	937418	-		Candidatus_Nitrososphaera_gargensis_Ga9_2_ui	NC_018719	chromosome									
	408403815	hypothetical protein Ngar_c12010	No	0	937490	937792	-		Candidatus_Nitrososphaera_gargensis_Ga9_2_ui	NC_018719	chromosome									
	408403816	hypothetical protein Ngar_c12020	No	0	938000	938161	+		Candidatus_Nitrososphaera_gargensis_Ga9_2_ui	NC_018719	chromosome									
===								17	Candidatus_Nitrososphaera_gargensis_Ga9_2_ui	NC_018719	chromosome									
	408403822	luciferase-like/flavin-/F420 oxidoreductase	No	2	942800	943285	+		Candidatus_Nitrososphaera_gargensis_Ga9_2_ui	NC_018719	chromosome		arCOG02410	2 (-)	C		Coenzyme F420-dependent N5,N10-methylene te	1E-11	pfam00296	Luciferase-like monooxygenase.
	408403823	hypothetical protein Ngar_c12090	Yes	2	943729	944097	-		Candidatus_Nitrososphaera_gargensis_Ga9_2_ui	NC_018719	chromosome									
	408403824	hypothetical protein Ngar_c12100	No	4	944124	944624	-		Candidatus_Nitrososphaera_gargensis_Ga9_2_ui	NC_018719	chromosome									
	408403825	hypothetical protein Ngar_c12110	No	0	944727	945302	+		Candidatus_Nitrososphaera_gargensis_Ga9_2_ui	NC_018719	chromosome									
	408403826	hypothetical protein Ngar_c12120	No	0	945299	945688	-		Candidatus_Nitrososphaera_gargensis_Ga9_2_ui	NC_018719	chromosome									
	408403827	peptidase, M23 precursor	Yes	1	945720	946418	-		Candidatus_Nitrososphaera_gargensis_Ga9_2_ui	NC_018719	chromosome							2E-15	pfam01551	Peptidase family M23. Members of this fa
	408403828	hypothetical protein Ngar_c12140	No	0	946535	946816	+		Candidatus_Nitrososphaera_gargensis_Ga9_2_ui	NC_018719	chromosome		arCOG04557	1 (-)	S		Uncharacterized conserved protein			
	408403829	hypothetical protein Ngar_c12150	No	0	946820	947239	+		Candidatus_Nitrososphaera_gargensis_Ga9_2_ui	NC_018719	chromosome									
	408403830	hypothetical protein Ngar_c12160	No	0	947263	947496	-		Candidatus_Nitrososphaera_gargensis_Ga9_2_ui	NC_018719	chromosome									
	408403831	hypothetical protein Ngar_c12170	No	1	947624	948034	+		Candidatus_Nitrososphaera_gargensis_Ga9_2_ui	NC_018719	chromosome									
	408403832	hypothetical protein Ngar_c12180	No	0	948043	948534	-		Candidatus_Nitrososphaera_gargensis_Ga9_2_ui	NC_018719	chromosome									
	408403833	zinc finger C2H2 domain-containing protein	No	0	948887	949225	-		Candidatus_Nitrososphaera_gargensis_Ga9_2_ui	NC_018719	chromosome									
	408403834	hypothetical protein Ngar_c12200	No	0	949464	950060	-		Candidatus_Nitrososphaera_gargensis_Ga9_2_ui	NC_018719	chromosome	clust_135								
	408403835	hypothetical protein Ngar_c12210	No	0	950065	950538	-		Candidatus_Nitrososphaera_gargensis_Ga9_2_ui	NC_018719	chromosome									
	408403836	hypothetical protein Ngar_c12220	No	0	950647	950820	+		Candidatus_Nitrososphaera_gargensis_Ga9_2_ui	NC_018719	chromosome									
	408403837	universal stress protein	No	0	950921	951376	-		Candidatus_Nitrososphaera_gargensis_Ga9_2_ui	NC_018719	chromosome		arCOG02053	7 (-)	T	UspA	Nucleotide-binding protein, UspA family	1E-23	pfam00582	Universal stress protein family. The uni
	408403838	zinc finger C2H2 domain-containing protein	No	0	951461	951616	+		Candidatus_Nitrososphaera_gargensis_Ga9_2_ui	NC_018719	chromosome									
===								5	Candidatus_Nitrososphaera_gargensis_Ga9_2_ui	NC_018719	chromosome									
	408403893	hypothetical protein Ngar_c12790	No	0	999260	999475	+		Candidatus_Nitrososphaera_gargensis_Ga9_2_ui	NC_018719	chromosome									
	408403894	hypothetical protein Ngar_c12800	No	0	999634	999903	-		Candidatus_Nitrososphaera_gargensis_Ga9_2_ui	NC_018719	chromosome									
	408403895	hypothetical protein Ngar_c12810	No	0	1000267	1000485	+		Candidatus_Nitrososphaera_gargensis_Ga9_2_ui	NC_018719	chromosome									
	408403896	hypothetical protein Ngar_c12820	Yes	0	1000577	1001497	+		Candidatus_Nitrososphaera_gargensis_Ga9_2_ui	NC_018719	chromosome	arCOG00379,a	arCOG00379	1 (-)	S		Uncharacterized conserved protein	5E-15	COG1750	Archaeal serine proteases
	408403897	hypothetical protein Ngar_c12830	No	0	1001544	1001897	+		Candidatus_Nitrososphaera_gargensis_Ga9_2_ui	NC_018719	chromosome									
===								7	Candidatus_Nitrososphaera_gargensis_Ga9_2_ui	NC_018719	chromosome									
	408403918	cation diffusion facilitator family transporter	No	6	1020965	1021828	+		Candidatus_Nitrososphaera_gargensis_Ga9_2_ui	NC_018719	chromosome		arCOG01474	2 (-)	P	MMT1	Predicted Co/Zn/Cd cation transporter	1E-30	COG0053	Predicted Co/Zn/Cd cation transporters [
	408403919	hypothetical protein Ngar_c13070	No	0	1021851	1022009	+		Candidatus_Nitrososphaera_gargensis_Ga9_2_ui	NC_018719	chromosome									
	408403920	hypothetical protein Ngar_c13080	No	6	1022006	1022824	-		Candidatus_Nitrososphaera_gargensis_Ga9_2_ui	NC_018719	chromosome									
	408403921	hypothetical protein Ngar_c13090	No	4	1023162	1023734	+		Candidatus_Nitrososphaera_gargensis_Ga9_2_ui	NC_018719	chromosome									
	408403922	hypothetical protein Ngar_c13100	No	3	1023731	1024084	+		Candidatus_Nitrososphaera_gargensis_Ga9_2_ui	NC_018719	chromosome									
	408403923	hypothetical protein Ngar_c13110	No	1	1024118	1024336	+		Candidatus_Nitrososphaera_gargensis_Ga9_2_ui	NC_018719	chromosome									
	408403924	hypothetical protein Ngar_c13120	Yes	1	1024540	1025259	+		Candidatus_Nitrososphaera_gargensis_Ga9_2_ui	NC_018719	chromosome		arCOG02868	2 (-)	O	DsbG	Protein-disulfide isomerase	7E-24	pfam13462	Thioredoxin.
===								19	Candidatus_Nitrososphaera_gargensis_Ga9_2_ui	NC_018719	chromosome									
	408403958	hypothetical protein Ngar_c13460	No	0	1049257	1049598	-		Candidatus_Nitrososphaera_gargensis_Ga9_2_ui	NC_018719	chromosome		arCOG01055	9 (-)	K		Predicted transcriptional regulator	0.00000008	COG3432	Predicted transcriptional regulator
	408403959	hypothetical protein Ngar_c13470	No	0	1049803	1050330	+		Candidatus_Nitrososphaera_gargensis_Ga9_2_ui	NC_018719	chromosome									
	408403960	hypothetical protein Ngar_c13480	No	0	1050373	1050756	-		Candidatus_Nitrososphaera_gargensis_Ga9_2_ui	NC_018719	chromosome									
	408403961	hypothetical protein Ngar_c13490	No	0	1050756	1050971	-		Candidatus_Nitrososphaera_gargensis_Ga9_2_ui	NC_018719	chromosome									
	408403962	hypothetical protein Ngar_c13500	No	1	1050984	1051307	-		Candidatus_Nitrososphaera_gargensis_Ga9_2_ui	NC_018719	chromosome									
	408403963	hypothetical protein Ngar_c13510	No	0	1051314	1051496	+		Candidatus_Nitrososphaera_gargensis_Ga9_2_ui	NC_018719	chromosome									
	408403964	hypothetical protein Ngar_c13520	No	1	1051596	1051856	+		Candidatus_Nitrososphaera_gargensis_Ga9_2_ui	NC_018719	chromosome									
	408403965	hypothetical protein Ngar_c13530	No	0	1051888	1052259	-		Candidatus_Nitrososphaera_gargensis_Ga9_2_ui	NC_018719	chromosome									
	408403966	hypothetical protein Ngar_c13540	No	5	1052266	1053789	-		Candidatus_Nitrososphaera_gargensis_Ga9_2_ui	NC_018719	chromosome		arCOG09857	2	S		Uncharacterized conserved protein			
	408403967	hypothetical protein Ngar_c13550	No	0	1053790	1054206	-		Candidatus_Nitrososphaera_gargensis_Ga9_2_ui	NC_018719	chromosome									
	408403968	hypothetical protein Ngar_c13560	No	0	1054653	1054907	-		Candidatus_Nitrososphaera_gargensis_Ga9_2_ui	NC_018719	chromosome									
	408403969	hypothetical protein Ngar_c13570	No	0	1055068	1056147	-		Candidatus_Nitrososphaera_gargensis_Ga9_2_ui	NC_018719	chromosome									
	408403970	hypothetical protein Ngar_c13580	No	0	1056302	1056847	-		Candidatus_Nitrososphaera_gargensis_Ga9_2_ui	NC_018719	chromosome									
	408403971	hypothetical protein Ngar_c13590	No	0	1056952	1057383	+		Candidatus_Nitrososphaera_gargensis_Ga9_2_ui	NC_018719	chromosome									
	408403972	hypothetical protein Ngar_c13600	No	0	1057723	1057980	-		Candidatus_Nitrososphaera_gargensis_Ga9_2_ui	NC_018719	chromosome									
	408403973	hypothetical protein Ngar_c13610	No	0	1057993	1058709	-		Candidatus_Nitrososphaera_gargensis_Ga9_2_ui	NC_018719	chromosome		arCOG00801	1 (-)	L		RecB family exonuclease			
	408403974	hypothetical protein Ngar_c13620	No	0	1058709	1059698	-		Candidatus_Nitrososphaera_gargensis_Ga9_2_ui	NC_018719	chromosome									
	408403975	hypothetical protein Ngar_c13630	No	0	1059915	1060406	-		Candidatus_Nitrososphaera_gargensis_Ga9_2_ui	NC_018719	chromosome									
	408403976	hypothetical protein Ngar_c13640	No	0	1060763	1061098	-		Candidatus_Nitrososphaera_gargensis_Ga9_2_ui	NC_018719	chromosome									
===								5	Candidatus_Nitrososphaera_gargensis_Ga9_2_ui	NC_018719	chromosome									
	408403986	hypothetical protein Ngar_c13750	No	0	1071298	1071624	-		Candidatus_Nitrososphaera_gargensis_Ga9_2_ui	NC_018719	chromosome									
	408403987	hypothetical protein Ngar_c13760	No	0	1071730	1072419	+		Candidatus_Nitrososphaera_gargensis_Ga9_2_ui	NC_018719	chromosome									
	408403988	hypothetical protein Ngar_c13770	No	2	1073246	1073500	+		Candidatus_Nitrososphaera_gargensis_Ga9_2_ui	NC_018719	chromosome									
	408403989	transcription initiation factor IIB	No	0	1073695	1074552	+		Candidatus_Nitrososphaera_gargensis_Ga9_2_ui	NC_018719	chromosome		arCOG01981	8 (-)	K	SUA7	Transcription initiation factor TFIIB, Brf1 subunit/Tr	8E-86	COG1405	Transcription initiation factor TFIIIB,
	408403990	hypothetical protein Ngar_c13790	No	0	1074542	1074976	-		Candidatus_Nitrososphaera_gargensis_Ga9_2_ui	NC_018719	chromosome									
===								6	Candidatus_Nitrososphaera_gargensis_Ga9_2_ui	NC_018719	chromosome									
	408404043	sodium/hydrogen exchange	No	13	1115566	1116732	-		Candidatus_Nitrososphaera_gargensis_Ga9_2_ui	NC_018719	chromosome		arCOG01955	3 (-)	P	KefB	Kef-type K+ transport system, membrane compo	4E-61	COG0475	Kef-type K+ transport systems, membrane
	408404044	hypothetical protein Ngar_c14340	No	1	1116883	1117365	+		Candidatus_Nitrososphaera_gargensis_Ga9_2_ui	NC_018719	chromosome									
	408404045	hypothetical protein Ngar_c14350	No	0	1117433	1117657	+		Candidatus_Nitrososphaera_gargensis_Ga9_2_ui	NC_018719	chromosome									
	408404046	hypothetical protein Ngar_c14360, partial	No	0	1117913	1118107	+		Candidatus_Nitrososphaera_gargensis_Ga9_2_ui	NC_018719	chromosome									
	408404047	hypothetical protein Ngar_c14370	Yes	1	1118223	1119191	+		Candidatus_Nitrososphaera_gargensis_Ga9_2_ui	NC_018719	chromosome									
	408404048	8-oxoguanine DNA glycosylase	No	0	1119287	1120195	-		Candidatus_Nitrososphaera_gargensis_Ga9_2_ui	NC_018719	chromosome		arCOG00464	2 (-)	L	AlkA	3-methyladenine DNA glycosylase/8-oxoguanine	2E-17	COG0122	3-methyladenine DNA glycosylase/8-oxogua
===								5	Candidatus_Nitrososphaera_gargensis_Ga9_2_ui	NC_018719	chromosome									
	408404091	hypothetical protein Ngar_c14810	No	0	1153246	1153431	-		Candidatus_Nitrososphaera_gargensis_Ga9_2_ui	NC_018719	chromosome									
	408404092	hypothetical protein Ngar_c14820	No	2	1153794	1154003	-		Candidatus_Nitrososphaera_gargensis_Ga9_2_ui	NC_018719	chromosome									
	408404093	hypothetical protein Ngar_c14840	No	12	1154738	1155976	-		Candidatus_Nitrososphaera_gargensis_Ga9_2_ui	NC_018719	chromosome		arCOG10675	1 (-)	S		Uncharacterized conserved protein			
	408404094	hypothetical protein Ngar_c14850	Yes	1	1156060	1156266	+		Candidatus_Nitrososphaera_gargensis_Ga9_2_ui	NC_018719	chromosome									
	408404095	zinc finger C2H2 domain-containing protein	No	0	1156576	1156707	+		Candidatus_Nitrososphaera_gargensis_Ga9_2_ui	NC_018719	chromosome									
===								7	Candidatus_Nitrososphaera_gargensis_Ga9_2_ui	NC_018719	chromosome									
	408404115	zinc finger C2H2 domain-containing protein	No	0	1175453	1175926	+		Candidatus_Nitrososphaera_gargensis_Ga9_2_ui	NC_018719	chromosome	clust_221								
	408404116	isochorismatase hydrolase	No	0	1176037	1176462	+		Candidatus_Nitrososphaera_gargensis_Ga9_2_ui	NC_018719	chromosome		arCOG01943	2 (-)	Q	PncA	Amidase related to nicotinamidase	0.0000006	pfam00857	Isochorismatase family. This family are
	408404117	hypothetical protein Ngar_c15080	No	0	1176549	1176770	+		Candidatus_Nitrososphaera_gargensis_Ga9_2_ui	NC_018719	chromosome									
	408404118	hypothetical protein Ngar_c15090, partial	No	0	1176937	1177119	+		Candidatus_Nitrososphaera_gargensis_Ga9_2_ui	NC_018719	chromosome									
	408404119	hypothetical protein Ngar_c15100	No	0	1178085	1178390	+		Candidatus_Nitrososphaera_gargensis_Ga9_2_ui	NC_018719	chromosome									
	408404120	hypothetical protein Ngar_c15110	No	2	1178604	1178978	-		Candidatus_Nitrososphaera_gargensis_Ga9_2_ui	NC_018719	chromosome									
	408404121	hypothetical protein Ngar_c15120	No	0	1179018	1179278	-		Candidatus_Nitrososphaera_gargensis_Ga9_2_ui	NC_018719	chromosome		arCOG01057	21 (-)	K		Predicted transcriptional regulator			
===								8	Candidatus_Nitrososphaera_gargensis_Ga9_2_ui	NC_018719	chromosome									
	408404124	hypothetical protein Ngar_c15150	No	4	1180328	1180693	+		Candidatus_Nitrososphaera_gargensis_Ga9_2_ui	NC_018719	chromosome									
	408404125	hypothetical protein Ngar_c15160	No	4	1180733	1181104	+		Candidatus_Nitrososphaera_gargensis_Ga9_2_ui	NC_018719	chromosome									
	408404126	hypothetical protein Ngar_c15180	Yes	1	1181463	1181945	-		Candidatus_Nitrososphaera_gargensis_Ga9_2_ui	NC_018719	chromosome		arCOG08689	1 (-)	S		Uncharacterized conserved protein			
	408404127	hypothetical protein Ngar_c15190	No	0	1181967	1182275	-		Candidatus_Nitrososphaera_gargensis_Ga9_2_ui	NC_018719	chromosome									
	408404128	hypothetical protein Ngar_c15200	Yes	1	1183569	1183925	+		Candidatus_Nitrososphaera_gargensis_Ga9_2_ui	NC_018719	chromosome									
	408404129	hypothetical protein Ngar_c15210	No	0	1184210	1184377	-		Candidatus_Nitrososphaera_gargensis_Ga9_2_ui	NC_018719	chromosome									
	408404130	hypothetical protein Ngar_c15220	Yes	1	1184597	1184929	-		Candidatus_Nitrososphaera_gargensis_Ga9_2_ui	NC_018719	chromosome									
	408404131	hypothetical protein Ngar_c15230	No	0	1185304	1186023	+		Candidatus_Nitrososphaera_gargensis_Ga9_2_ui	NC_018719	chromosome		arCOG02610	2 (-)	L		Rec8/ScpA/Scc1-like protein (kleisin family)	0.0000003	COG1354	Rec8/ScpA/Scc1-like protein (kleisin fam
===								19	Candidatus_Nitrososphaera_gargensis_Ga9_2_ui	NC_018719	chromosome									
	408404171	zinc finger C2H2 domain-containing protein	No	0	1219690	1220430	+		Candidatus_Nitrososphaera_gargensis_Ga9_2_ui	NC_018719	chromosome									
	408404172	hypothetical protein Ngar_c15650	No	0	1220874	1221107	+		Candidatus_Nitrososphaera_gargensis_Ga9_2_ui	NC_018719	chromosome									
	408404173	hypothetical protein Ngar_c15660	No	0	1221375	1221557	-		Candidatus_Nitrososphaera_gargensis_Ga9_2_ui	NC_018719	chromosome									
	408404174	hypothetical protein Ngar_c15670	No	0	1221642	1222214	-		Candidatus_Nitrososphaera_gargensis_Ga9_2_ui	NC_018719	chromosome		arCOG00280	21 (-)	L		HerA helicase			
	408404175	hypothetical protein Ngar_c15680	No	0	1222198	1222377	-		Candidatus_Nitrososphaera_gargensis_Ga9_2_ui	NC_018719	chromosome									
	408404176	hypothetical protein Ngar_c15690	No	0	1222462	1223229	-		Candidatus_Nitrososphaera_gargensis_Ga9_2_ui	NC_018719	chromosome									
	408404177	hypothetical protein Ngar_c15700	No	3	1223336	1223638	+		Candidatus_Nitrososphaera_gargensis_Ga9_2_ui	NC_018719	chromosome									
	408404178	hypothetical protein Ngar_c15710	No	0	1223638	1224531	+		Candidatus_Nitrososphaera_gargensis_Ga9_2_ui	NC_018719	chromosome									
	408404179	hypothetical protein Ngar_c15720	No	2	1224549	1224821	+		Candidatus_Nitrososphaera_gargensis_Ga9_2_ui	NC_018719	chromosome									
	408404180	hypothetical protein Ngar_c15730	Yes	0	1224826	1225296	+		Candidatus_Nitrososphaera_gargensis_Ga9_2_ui	NC_018719	chromosome									
	408404181	hypothetical protein Ngar_c15740	No	0	1225293	1225871	+		Candidatus_Nitrososphaera_gargensis_Ga9_2_ui	NC_018719	chromosome									
	408404182	hypothetical protein Ngar_c15750	No	0	1225871	1226515	+		Candidatus_Nitrososphaera_gargensis_Ga9_2_ui	NC_018719	chromosome									
	408404183	hypothetical protein Ngar_c15760	No	0	1226806	1227192	-		Candidatus_Nitrososphaera_gargensis_Ga9_2_ui	NC_018719	chromosome									
	408404184	hypothetical protein Ngar_c15770	No	0	1227352	1228683	+		Candidatus_Nitrososphaera_gargensis_Ga9_2_ui	NC_018719	chromosome		arCOG06451	4 (-)	L	XerC	Integrase			
	408404185	hypothetical protein Ngar_c15780	No	0	1229087	1229320	+		Candidatus_Nitrososphaera_gargensis_Ga9_2_ui	NC_018719	chromosome									
	408404186	hypothetical protein Ngar_c15790	No	0	1229549	1229761	+		Candidatus_Nitrososphaera_gargensis_Ga9_2_ui	NC_018719	chromosome									
	408404187	hypothetical protein Ngar_c15800	No	0	1229758	1229961	+		Candidatus_Nitrososphaera_gargensis_Ga9_2_ui	NC_018719	chromosome									
	408404188	hypothetical protein Ngar_c15810	No	2	1230436	1230669	+		Candidatus_Nitrososphaera_gargensis_Ga9_2_ui	NC_018719	chromosome									
	408404189	phosphoribosylformylglycinamidine synthase F	No	0	1230688	1230969	+		Candidatus_Nitrososphaera_gargensis_Ga9_2_ui	NC_018719	chromosome		arCOG04462	1 (-)	F	PurS	Phosphoribosylformylglycinamidine (FGAM) synt	2E-11	COG1828	Phosphoribosylformylglycinamidine (FGAM)
===								5	Candidatus_Nitrososphaera_gargensis_Ga9_2_ui	NC_018719	chromosome									
	408404290	hypothetical protein Ngar_c16830	No	0	1313227	1313385	+		Candidatus_Nitrososphaera_gargensis_Ga9_2_ui	NC_018719	chromosome									
	408404291	hypothetical protein Ngar_c16840	No	0	1313581	1314153	-		Candidatus_Nitrososphaera_gargensis_Ga9_2_ui	NC_018719	chromosome									
	408404292	hypothetical protein Ngar_c16850	No	0	1314420	1314584	+		Candidatus_Nitrososphaera_gargensis_Ga9_2_ui	NC_018719	chromosome									
	408404293	XRE family transcriptional regulator	No	0	1314857	1315411	-		Candidatus_Nitrososphaera_gargensis_Ga9_2_ui	NC_018719	chromosome		arCOG00608	3 (-)	K		Predicted transcriptional regulator with C-terminal	1E-29	COG3620	Predicted transcriptional regulator with
	408404294	hypothetical protein Ngar_c16870	No	0	1315718	1315891	+		Candidatus_Nitrososphaera_gargensis_Ga9_2_ui	NC_018719	chromosome									
===								20	Candidatus_Nitrososphaera_gargensis_Ga9_2_ui	NC_018719	chromosome									
	408404304	integrase family protein	No	0	1325015	1326226	+		Candidatus_Nitrososphaera_gargensis_Ga9_2_ui	NC_018719	chromosome		arCOG01245	15 (-)	L	XerC	Integrase			
	408404305	hypothetical protein Ngar_c16980	No	0	1326188	1327117	-		Candidatus_Nitrososphaera_gargensis_Ga9_2_ui	NC_018719	chromosome									
	408404306	hypothetical protein Ngar_c16990	No	0	1327168	1327386	+		Candidatus_Nitrososphaera_gargensis_Ga9_2_ui	NC_018719	chromosome									
	408404307	hypothetical protein Ngar_c17000	No	0	1327571	1327813	+		Candidatus_Nitrososphaera_gargensis_Ga9_2_ui	NC_018719	chromosome									
	408404308	hypothetical protein Ngar_c17010	No	0	1327810	1328043	+		Candidatus_Nitrososphaera_gargensis_Ga9_2_ui	NC_018719	chromosome									
	408404309	MCM family domain-containing protein	No	0	1328040	1329989	+		Candidatus_Nitrososphaera_gargensis_Ga9_2_ui	NC_018719	chromosome		arCOG00439	14 (-)	L	MCM2	Predicted ATPase involved in replication control,	8E-16	COG1241	Predicted ATPase involved in replication
	408404310	hypothetical protein Ngar_c17030	No	0	1330206	1330814	+		Candidatus_Nitrososphaera_gargensis_Ga9_2_ui	NC_018719	chromosome									
	408404311	hypothetical protein Ngar_c17040	No	0	1331030	1331245	-		Candidatus_Nitrososphaera_gargensis_Ga9_2_ui	NC_018719	chromosome		arCOG10363	7	V		AbrB-like transcriptional regulator			
	408404312	hypothetical protein Ngar_c17050	No	0	1331315	1331527	+		Candidatus_Nitrososphaera_gargensis_Ga9_2_ui	NC_018719	chromosome									
	408404313	hypothetical protein Ngar_c17060	No	0	1331524	1331706	-		Candidatus_Nitrososphaera_gargensis_Ga9_2_ui	NC_018719	chromosome									
	408404314	hypothetical protein Ngar_c17070	No	0	1331813	1332331	+		Candidatus_Nitrososphaera_gargensis_Ga9_2_ui	NC_018719	chromosome									
	408404315	hypothetical protein Ngar_c17080	No	0	1332945	1333340	-		Candidatus_Nitrososphaera_gargensis_Ga9_2_ui	NC_018719	chromosome									
	408404316	hypothetical protein Ngar_c17090	No	2	1333510	1333866	+		Candidatus_Nitrososphaera_gargensis_Ga9_2_ui	NC_018719	chromosome									
	408404317	hypothetical protein Ngar_c17100	No	0	1334038	1334631	+		Candidatus_Nitrososphaera_gargensis_Ga9_2_ui	NC_018719	chromosome									
	408404318	hypothetical protein Ngar_c17110	No	0	1334664	1335377	+		Candidatus_Nitrososphaera_gargensis_Ga9_2_ui	NC_018719	chromosome									
	408404319	hypothetical protein Ngar_c17120	No	0	1335395	1335901	+		Candidatus_Nitrososphaera_gargensis_Ga9_2_ui	NC_018719	chromosome									
	408404320	hypothetical protein Ngar_c17130	No	0	1336374	1336547	+		Candidatus_Nitrososphaera_gargensis_Ga9_2_ui	NC_018719	chromosome									
	408404321	SMF family protein	No	0	1336730	1337425	+		Candidatus_Nitrososphaera_gargensis_Ga9_2_ui	NC_018719	chromosome		arCOG02431	4 (-)	R		Predicted Rossmann fold nucleotide-binding prot	9E-40	COG0758	Predicted Rossmann fold nucleotide-bindi
	408404322	purine/pyrimidine phosphoribosyl transferases	No	0	1337518	1338075	+		Candidatus_Nitrososphaera_gargensis_Ga9_2_ui	NC_018719	chromosome							0.00006	COG1040	Predicted amidophosphoribosyltransferase
	408404323	hypothetical protein Ngar_c17160	No	0	1338072	1338266	-		Candidatus_Nitrososphaera_gargensis_Ga9_2_ui	NC_018719	chromosome									
===								5	Candidatus_Nitrososphaera_gargensis_Ga9_2_ui	NC_018719	chromosome									
	408404372	hypothetical protein Ngar_c17650	No	0	1374253	1374447	-		Candidatus_Nitrososphaera_gargensis_Ga9_2_ui	NC_018719	chromosome									
	408404373	histidine triad protein	No	0	1374539	1374973	+		Candidatus_Nitrososphaera_gargensis_Ga9_2_ui	NC_018719	chromosome		arCOG00419	4 (-)	F	Hit	HIT family hydrolase	5E-36	COG0537	Diadenosine tetraphosphate (Ap4A) hydrol
	408404374	hypothetical protein Ngar_c17670	No	0	1375038	1375412	+		Candidatus_Nitrososphaera_gargensis_Ga9_2_ui	NC_018719	chromosome									
	408404375	hypothetical protein Ngar_c17680	No	0	1375753	1376013	-		Candidatus_Nitrososphaera_gargensis_Ga9_2_ui	NC_018719	chromosome									
	408404376	hypothetical protein Ngar_c17690	No	0	1376130	1376792	+		Candidatus_Nitrososphaera_gargensis_Ga9_2_ui	NC_018719	chromosome									
===								12	Candidatus_Nitrososphaera_gargensis_Ga9_2_ui	NC_018719	chromosome									
	408404430	hypothetical protein Ngar_c18230	No	1	1417549	1417746	+		Candidatus_Nitrososphaera_gargensis_Ga9_2_ui	NC_018719	chromosome									
	408404431	heat shock protein Hsp20	No	0	1418637	1419041	+		Candidatus_Nitrososphaera_gargensis_Ga9_2_ui	NC_018719	chromosome		arCOG01832	1 (-)	O	IbpA	Molecular chaperone (HSP20 family)	0.000002	COG0071	Molecular chaperone (small heat shock pr
	408404432	hypothetical protein Ngar_c18250	No	0	1419379	1419657	+		Candidatus_Nitrososphaera_gargensis_Ga9_2_ui	NC_018719	chromosome									
	408404433	hypothetical protein Ngar_c18260	No	0	1419861	1420016	-		Candidatus_Nitrososphaera_gargensis_Ga9_2_ui	NC_018719	chromosome									
	408404434	hypothetical protein Ngar_c18270	No	0	1420772	1420909	-		Candidatus_Nitrososphaera_gargensis_Ga9_2_ui	NC_018719	chromosome									
	408404435	hypothetical protein Ngar_c18280	No	0	1420839	1421084	-		Candidatus_Nitrososphaera_gargensis_Ga9_2_ui	NC_018719	chromosome		arCOG01055	9 (-)	K		Predicted transcriptional regulator			
	408404436	hypothetical protein Ngar_c18290	No	0	1421897	1422058	+		Candidatus_Nitrososphaera_gargensis_Ga9_2_ui	NC_018719	chromosome									
	408404437	hypothetical protein Ngar_c18300	No	1	1422555	1422827	+		Candidatus_Nitrososphaera_gargensis_Ga9_2_ui	NC_018719	chromosome									
	408404438	hypothetical protein Ngar_c18310	No	0	1422869	1423078	+		Candidatus_Nitrososphaera_gargensis_Ga9_2_ui	NC_018719	chromosome									
	408404439	hypothetical protein Ngar_c18320	Yes	0	1423059	1423355	-		Candidatus_Nitrososphaera_gargensis_Ga9_2_ui	NC_018719	chromosome									
	408404440	hypothetical protein Ngar_c18330	No	0	1423598	1423915	-		Candidatus_Nitrososphaera_gargensis_Ga9_2_ui	NC_018719	chromosome	clust_335								
	408404441	glyoxalase/bleomycin resistance protein/dioxy	No	0	1424201	1424557	+		Candidatus_Nitrososphaera_gargensis_Ga9_2_ui	NC_018719	chromosome		arCOG02708	2 (-)	E	GloA	Lactoylglutathione lyase or related enzyme	0.00000007	pfam12681	Glyoxalase-like domain. This domain is r
===								6	Candidatus_Nitrososphaera_gargensis_Ga9_2_ui	NC_018719	chromosome									
	408404458	hypothetical protein Ngar_c18510	No	0	1437576	1437932	+		Candidatus_Nitrososphaera_gargensis_Ga9_2_ui	NC_018719	chromosome							0.00004	pfam12762	ISXO2-like transposase domain. This doma
	408404459	hypothetical protein Ngar_c18520	No	0	1438043	1438141	+		Candidatus_Nitrososphaera_gargensis_Ga9_2_ui	NC_018719	chromosome									
	408404460	drug/metabolite transporter superfamily protein	No	9	1439302	1440123	-		Candidatus_Nitrososphaera_gargensis_Ga9_2_ui	NC_018719	chromosome		arCOG00271	3 (-)	G	RhaT	Permease of the drug/metabolite transporter (DM	0.000000001	COG0697	Permeases of the drug/metabolite transpo
	408404461	hypothetical protein Ngar_c18550	No	1	1440154	1440369	+		Candidatus_Nitrososphaera_gargensis_Ga9_2_ui	NC_018719	chromosome									
	408404462	hypothetical protein Ngar_c18560	No	1	1440474	1440734	+		Candidatus_Nitrososphaera_gargensis_Ga9_2_ui	NC_018719	chromosome									
	408404463	hypothetical protein Ngar_c18570	No	0	1440810	1441088	+		Candidatus_Nitrososphaera_gargensis_Ga9_2_ui	NC_018719	chromosome							0.000004	pfam11373	Protein of unknown function (DUF3175). T
===								7	Candidatus_Nitrososphaera_gargensis_Ga9_2_ui	NC_018719	chromosome									
	408404468	hypothetical protein Ngar_c18620	No	0	1443285	1444019	-		Candidatus_Nitrososphaera_gargensis_Ga9_2_ui	NC_018719	chromosome		arCOG03842	1 (-)	R		TlpA/TrxA-like family protein	0.0000001	pfam06053	Domain of unknown function (DUF929). Fam
	408404469	hypothetical protein Ngar_c18630	No	0	1444195	1444395	+		Candidatus_Nitrososphaera_gargensis_Ga9_2_ui	NC_018719	chromosome									
	408404470	hypothetical protein Ngar_c18640	No	0	1444417	1444638	+		Candidatus_Nitrososphaera_gargensis_Ga9_2_ui	NC_018719	chromosome									
	408404471	hypothetical protein Ngar_c18650, partial	No	0	1445104	1445295	+		Candidatus_Nitrososphaera_gargensis_Ga9_2_ui	NC_018719	chromosome									
	408404472	hypothetical protein Ngar_c18660	No	0	1445467	1445544	+		Candidatus_Nitrososphaera_gargensis_Ga9_2_ui	NC_018719	chromosome									
	408404473	arginase/agmatinase/formiminoglutamase	No	0	1445675	1445920	+		Candidatus_Nitrososphaera_gargensis_Ga9_2_ui	NC_018719	chromosome		arCOG01700	2 (-)	E	SpeB	Arginase family enzyme	0.0000001	COG0010	Arginase/agmatinase/formimionoglutamate
	408404474	hypothetical protein Ngar_c18680	No	2	1445946	1446215	-		Candidatus_Nitrososphaera_gargensis_Ga9_2_ui	NC_018719	chromosome									
===								19	Candidatus_Nitrososphaera_gargensis_Ga9_2_ui	NC_018719	chromosome									
	408404544	integrase family protein	No	0	1508325	1509740	+		Candidatus_Nitrososphaera_gargensis_Ga9_2_ui	NC_018719	chromosome		arCOG01245	15 (-)	L	XerC	Integrase			
	408404545	hypothetical protein Ngar_c19390	No	2	1509932	1510366	+		Candidatus_Nitrososphaera_gargensis_Ga9_2_ui	NC_018719	chromosome									
	408404546	hypothetical protein Ngar_c19400	No	0	1510433	1511020	+		Candidatus_Nitrososphaera_gargensis_Ga9_2_ui	NC_018719	chromosome									
	408404547	hypothetical protein Ngar_c19410	No	0	1511467	1511784	-		Candidatus_Nitrososphaera_gargensis_Ga9_2_ui	NC_018719	chromosome									
	408404548	hypothetical protein Ngar_c19420	No	0	1511870	1513204	-		Candidatus_Nitrososphaera_gargensis_Ga9_2_ui	NC_018719	chromosome									
	408404549	piwi domain-containing protein	No	0	1513201	1514520	-		Candidatus_Nitrososphaera_gargensis_Ga9_2_ui	NC_018719	chromosome		arCOG06164	4 (-)	V		Inactivated PIWI domain containing phage-defense protein			
	408404550	hypothetical protein Ngar_c19440	No	0	1514520	1515326	-		Candidatus_Nitrososphaera_gargensis_Ga9_2_ui	NC_018719	chromosome									
	408404551	archaeal preflagellin peptidase FlaK	No	5	1515407	1516138	-		Candidatus_Nitrososphaera_gargensis_Ga9_2_ui	NC_018719	chromosome		arCOG02298	6 (-)	N	FlaK/PulC	Peptidase A24A, prepilin type IV			
	408404552	hypothetical protein Ngar_c19460	No	0	1516640	1517005	+		Candidatus_Nitrososphaera_gargensis_Ga9_2_ui	NC_018719	chromosome	clust_295								
	408404553	hypothetical protein Ngar_c19470	No	0	1517935	1518153	-		Candidatus_Nitrososphaera_gargensis_Ga9_2_ui	NC_018719	chromosome									
	408404554	hypothetical protein Ngar_c19480	No	0	1518140	1518502	-		Candidatus_Nitrososphaera_gargensis_Ga9_2_ui	NC_018719	chromosome									
	408404555	Hsp90 domain-containing protein	No	0	1518519	1519256	-		Candidatus_Nitrososphaera_gargensis_Ga9_2_ui	NC_018719	chromosome									
	408404556	hypothetical protein with ATP-binding domain	No	0	1519253	1521577	-		Candidatus_Nitrososphaera_gargensis_Ga9_2_ui	NC_018719	chromosome									
	408404557	hypothetical protein Ngar_c19510	No	0	1521747	1521995	-		Candidatus_Nitrososphaera_gargensis_Ga9_2_ui	NC_018719	chromosome									
	408404558	hypothetical protein Ngar_c19520	No	0	1521995	1522513	-		Candidatus_Nitrososphaera_gargensis_Ga9_2_ui	NC_018719	chromosome									
	408404559	hypothetical protein Ngar_c19530	No	0	1522674	1523078	-		Candidatus_Nitrososphaera_gargensis_Ga9_2_ui	NC_018719	chromosome									
	408404560	hypothetical protein Ngar_c19540	No	0	1523164	1523613	-		Candidatus_Nitrososphaera_gargensis_Ga9_2_ui	NC_018719	chromosome									
	408404561	hypothetical protein Ngar_c19550	No	0	1523610	1523924	-		Candidatus_Nitrososphaera_gargensis_Ga9_2_ui	NC_018719	chromosome									
	408404562	hypothetical protein Ngar_c19560	No	0	1524717	1525427	+		Candidatus_Nitrososphaera_gargensis_Ga9_2_ui	NC_018719	chromosome		arCOG03560	2 (-)	S		Uncharacterized conserved protein			
===								5	Candidatus_Nitrososphaera_gargensis_Ga9_2_ui	NC_018719	chromosome									
	408404593	hypothetical protein Ngar_c19870	No	0	1552107	1552340	+		Candidatus_Nitrososphaera_gargensis_Ga9_2_ui	NC_018719	chromosome									
	408404594	GCN5-like N-acetyltransferase	No	0	1552485	1553009	-		Candidatus_Nitrososphaera_gargensis_Ga9_2_ui	NC_018719	chromosome		arCOG04971	3 (-)	K	WecD	Acetyltransferase (GNAT) family			
	408404595	hypothetical protein Ngar_c19890	No	0	1553388	1553645	+		Candidatus_Nitrososphaera_gargensis_Ga9_2_ui	NC_018719	chromosome									
	408404596	hypothetical protein Ngar_c19900	No	0	1553656	1554390	+		Candidatus_Nitrososphaera_gargensis_Ga9_2_ui	NC_018719	chromosome									
	408404597	hypothetical protein Ngar_c19910	Yes	8	1554408	1556216	-		Candidatus_Nitrososphaera_gargensis_Ga9_2_ui	NC_018719	chromosome									
===								7	Candidatus_Nitrososphaera_gargensis_Ga9_2_ui	NC_018719	chromosome									
	408404609	hypothetical protein Ngar_c20030	Yes	2	1572724	1572981	+		Candidatus_Nitrososphaera_gargensis_Ga9_2_ui	NC_018719	chromosome									
	408404610	hypothetical protein Ngar_c20040	Yes	1	1573173	1574540	-		Candidatus_Nitrososphaera_gargensis_Ga9_2_ui	NC_018719	chromosome		arCOG02562	2 (-)	S		Beta-propeller repeat containing protein	0.000000003	COG3391	Uncharacterized conserved protein
	408404611	hypothetical protein Ngar_c20050	No	2	1574798	1575076	-		Candidatus_Nitrososphaera_gargensis_Ga9_2_ui	NC_018719	chromosome									
	408404612	hypothetical protein Ngar_c20060	No	1	1575206	1575664	+		Candidatus_Nitrososphaera_gargensis_Ga9_2_ui	NC_018719	chromosome									
	408404613	hypothetical protein Ngar_c20070	Yes	2	1575661	1575930	-		Candidatus_Nitrososphaera_gargensis_Ga9_2_ui	NC_018719	chromosome									
	408404614	hypothetical protein Ngar_c20080	No	1	1576014	1576451	+		Candidatus_Nitrososphaera_gargensis_Ga9_2_ui	NC_018719	chromosome									
	408404615	hypothetical protein Ngar_c20090	No	0	1576610	1576906	-		Candidatus_Nitrososphaera_gargensis_Ga9_2_ui	NC_018719	chromosome		arCOG01207	2 (-)	K		Predicted transcriptional regulator, MarR family			
===								7	Candidatus_Nitrososphaera_gargensis_Ga9_2_ui	NC_018719	chromosome									
	408404620	hypothetical protein Ngar_c20140	No	3	1580472	1580813	-		Candidatus_Nitrososphaera_gargensis_Ga9_2_ui	NC_018719	chromosome									
	408404621	inosine-5'-monophosphate dehydrogenase pr	No	0	1580973	1581827	+		Candidatus_Nitrososphaera_gargensis_Ga9_2_ui	NC_018719	chromosome		arCOG00600	2 (-)	R		CBS domain	7E-16	COG0517	FOG: CBS domain
	408404622	hypothetical protein Ngar_c20160	No	4	1581875	1582342	-		Candidatus_Nitrososphaera_gargensis_Ga9_2_ui	NC_018719	chromosome									
	408404623	hypothetical protein Ngar_c20170	No	0	1582422	1582658	-		Candidatus_Nitrososphaera_gargensis_Ga9_2_ui	NC_018719	chromosome									
	408404624	hypothetical protein Ngar_c20180	No	2	1582886	1583248	-		Candidatus_Nitrososphaera_gargensis_Ga9_2_ui	NC_018719	chromosome									
	408404625	RDD domain containing protein	No	4	1583339	1584037	-		Candidatus_Nitrososphaera_gargensis_Ga9_2_ui	NC_018719	chromosome		arCOG03633	2 (-)	S		Predicted membrane protein/domain			
	408404626	hypothetical protein Ngar_c20200	No	4	1584187	1584795	+		Candidatus_Nitrososphaera_gargensis_Ga9_2_ui	NC_018719	chromosome		arCOG10856	3	S		Uncharacterized conserved membrane protein			
===								5	Candidatus_Nitrososphaera_gargensis_Ga9_2_ui	NC_018719	chromosome									
	408404655	hypothetical protein Ngar_c20490	No	0	1603798	1603956	+		Candidatus_Nitrososphaera_gargensis_Ga9_2_ui	NC_018719	chromosome									
	408404656	hypothetical protein Ngar_c20500	Yes	0	1604041	1604349	+		Candidatus_Nitrososphaera_gargensis_Ga9_2_ui	NC_018719	chromosome									
	408404657	CBS domain-containing protein	No	0	1604409	1604852	-		Candidatus_Nitrososphaera_gargensis_Ga9_2_ui	NC_018719	chromosome		arCOG00606	4 (-)	R		CBS domain	4E-15	COG0517	FOG: CBS domain
	408404658	hypothetical protein Ngar_c20520	No	1	1604849	1605049	-		Candidatus_Nitrososphaera_gargensis_Ga9_2_ui	NC_018719	chromosome									
	408404659	hypothetical protein Ngar_c20530	No	4	1605202	1605705	+		Candidatus_Nitrososphaera_gargensis_Ga9_2_ui	NC_018719	chromosome		arCOG10856	3	S		Uncharacterized conserved membrane protein			
===								5	Candidatus_Nitrososphaera_gargensis_Ga9_2_ui	NC_018719	chromosome									
	408404667	hypothetical protein Ngar_c20610	No	0	1611345	1611560	+		Candidatus_Nitrososphaera_gargensis_Ga9_2_ui	NC_018719	chromosome									
	408404668	hypothetical protein Ngar_c20620	No	0	1611928	1612458	-		Candidatus_Nitrososphaera_gargensis_Ga9_2_ui	NC_018719	chromosome		arCOG02626	3 (-)	V	HsdS	Restriction endonuclease S subunit			
	408404669	hypothetical protein Ngar_c20630	No	0	1612445	1612834	-		Candidatus_Nitrososphaera_gargensis_Ga9_2_ui	NC_018719	chromosome									
	408404670	hypothetical protein Ngar_c20640	No	0	1612846	1613019	+		Candidatus_Nitrososphaera_gargensis_Ga9_2_ui	NC_018719	chromosome									
	408404671	hypothetical protein Ngar_c20650	No	0	1613020	1613199	+		Candidatus_Nitrososphaera_gargensis_Ga9_2_ui	NC_018719	chromosome									
===								12	Candidatus_Nitrososphaera_gargensis_Ga9_2_ui	NC_018719	chromosome									
	408404744	hypothetical protein Ngar_c21400	No	0	1665287	1666399	+		Candidatus_Nitrososphaera_gargensis_Ga9_2_ui	NC_018719	chromosome		arCOG01245	15 (-)	L	XerC	Integrase			
	408404745	hypothetical protein Ngar_c21410	No	1	1666686	1666901	-		Candidatus_Nitrososphaera_gargensis_Ga9_2_ui	NC_018719	chromosome									
	408404746	hypothetical protein Ngar_c21420	No	0	1667007	1667411	+		Candidatus_Nitrososphaera_gargensis_Ga9_2_ui	NC_018719	chromosome									
	408404747	hypothetical protein Ngar_c21430	No	0	1667413	1667583	+		Candidatus_Nitrososphaera_gargensis_Ga9_2_ui	NC_018719	chromosome									
	408404748	hypothetical protein Ngar_c21440	No	0	1667643	1667840	-		Candidatus_Nitrososphaera_gargensis_Ga9_2_ui	NC_018719	chromosome									
	408404749	hypothetical protein Ngar_c21450	No	1	1667972	1668166	+		Candidatus_Nitrososphaera_gargensis_Ga9_2_ui	NC_018719	chromosome									
	408404750	hypothetical protein Ngar_c21460	No	0	1668416	1669039	-		Candidatus_Nitrososphaera_gargensis_Ga9_2_ui	NC_018719	chromosome									
	408404751	hypothetical protein Ngar_c21470	No	2	1669928	1670311	-		Candidatus_Nitrososphaera_gargensis_Ga9_2_ui	NC_018719	chromosome									
	408404752	hypothetical protein Ngar_c21480	No	1	1670732	1671775	-		Candidatus_Nitrososphaera_gargensis_Ga9_2_ui	NC_018719	chromosome		arCOG07088	1 (-)	S		Uncharacterized conserved protein			
	408404753	hypothetical protein Ngar_c21490	Yes	0	1671927	1672190	-		Candidatus_Nitrososphaera_gargensis_Ga9_2_ui	NC_018719	chromosome									
	408404754	hypothetical protein Ngar_c21500	No	0	1672893	1673354	+		Candidatus_Nitrososphaera_gargensis_Ga9_2_ui	NC_018719	chromosome									
	408404755	hypothetical protein Ngar_c21510	No	0	1673405	1673629	+		Candidatus_Nitrososphaera_gargensis_Ga9_2_ui	NC_018719	chromosome									
===								5	Candidatus_Nitrososphaera_gargensis_Ga9_2_ui	NC_018719	chromosome									
	408404876	hypothetical protein Ngar_c22720	No	0	1771547	1772701	-		Candidatus_Nitrososphaera_gargensis_Ga9_2_ui	NC_018719	chromosome									
	408404877	hypothetical protein Ngar_c22730	No	0	1773437	1773817	+		Candidatus_Nitrososphaera_gargensis_Ga9_2_ui	NC_018719	chromosome									
	408404878	hypothetical protein Ngar_c22740	Yes	0	1773919	1774392	+		Candidatus_Nitrososphaera_gargensis_Ga9_2_ui	NC_018719	chromosome									
	408404879	transposase, IS4 family protein	No	0	1774769	1775131	-		Candidatus_Nitrososphaera_gargensis_Ga9_2_ui	NC_018719	chromosome		arCOG02751	5 (-)	L		Transposase, IS5 family			
	408404880	hypothetical protein Ngar_c22760	No	0	1775461	1775652	+		Candidatus_Nitrososphaera_gargensis_Ga9_2_ui	NC_018719	chromosome									
===								8	Candidatus_Nitrososphaera_gargensis_Ga9_2_ui	NC_018719	chromosome									
	408404959	4Fe-4S ferredoxin	No	0	1842296	1843318	-		Candidatus_Nitrososphaera_gargensis_Ga9_2_ui	NC_018719	chromosome		arCOG04548	2 (-)	C		Ferredoxin	0.00000008	COG1146	Ferredoxin
	408404960	zinc finger C2H2 domain-containing protein	No	0	1843466	1843606	-		Candidatus_Nitrososphaera_gargensis_Ga9_2_ui	NC_018719	chromosome									
	408404961	hypothetical protein Ngar_c23620	No	0	1844745	1845011	+		Candidatus_Nitrososphaera_gargensis_Ga9_2_ui	NC_018719	chromosome									
	408404962	hypothetical protein Ngar_c23630	No	0	1845069	1845377	+		Candidatus_Nitrososphaera_gargensis_Ga9_2_ui	NC_018719	chromosome									
	408404963	zinc finger C2H2 domain-containing protein	No	0	1845486	1845683	-		Candidatus_Nitrososphaera_gargensis_Ga9_2_ui	NC_018719	chromosome									
	408404964	hypothetical protein Ngar_c23650	No	0	1845760	1846008	+		Candidatus_Nitrososphaera_gargensis_Ga9_2_ui	NC_018719	chromosome									
	408404965	hypothetical protein Ngar_c23660	No	1	1845995	1846132	+		Candidatus_Nitrososphaera_gargensis_Ga9_2_ui	NC_018719	chromosome									
	408404966	hypothetical protein Ngar_c23670	No	0	1846545	1846832	-		Candidatus_Nitrososphaera_gargensis_Ga9_2_ui	NC_018719	chromosome		arCOG08760	1 (-)	S		Uncharacterized conserved protein			
===								5	Candidatus_Nitrososphaera_gargensis_Ga9_2_ui	NC_018719	chromosome									
	408404968	hypothetical protein Ngar_c23690	No	0	1847871	1848170	-		Candidatus_Nitrososphaera_gargensis_Ga9_2_ui	NC_018719	chromosome									
	408404969	hypothetical protein Ngar_c23700	No	0	1848259	1848462	-		Candidatus_Nitrososphaera_gargensis_Ga9_2_ui	NC_018719	chromosome									
	408404970	hypothetical protein Ngar_c23710	No	0	1848463	1848738	-		Candidatus_Nitrososphaera_gargensis_Ga9_2_ui	NC_018719	chromosome									
	408404971	hypothetical protein Ngar_c23720	No	0	1849012	1849302	+		Candidatus_Nitrososphaera_gargensis_Ga9_2_ui	NC_018719	chromosome		arCOG08684	7 (-)	R		Roadblock/LC7 domain superfamily protein			
	408404972	hypothetical protein Ngar_c23730	No	0	1849374	1849595	+		Candidatus_Nitrososphaera_gargensis_Ga9_2_ui	NC_018719	chromosome									
===								7	Candidatus_Nitrososphaera_gargensis_Ga9_2_ui	NC_018719	chromosome									
	408405015	hypothetical protein Ngar_c24170	No	0	1879714	1881405	+		Candidatus_Nitrososphaera_gargensis_Ga9_2_ui	NC_018719	chromosome		arCOG02749	3 (-)	K		Predicted transcriptional regulator			
	408405016	hypothetical protein Ngar_c24180	No	0	1881368	1881601	-		Candidatus_Nitrososphaera_gargensis_Ga9_2_ui	NC_018719	chromosome									
	408405017	hypothetical protein Ngar_c24190	No	0	1881928	1882089	-		Candidatus_Nitrososphaera_gargensis_Ga9_2_ui	NC_018719	chromosome									
	408405018	hypothetical protein Ngar_c24200	No	0	1882180	1882344	-		Candidatus_Nitrososphaera_gargensis_Ga9_2_ui	NC_018719	chromosome									
	408405019	hypothetical protein Ngar_c24210	Yes	1	1882737	1882928	+		Candidatus_Nitrososphaera_gargensis_Ga9_2_ui	NC_018719	chromosome									
	408405020	hypothetical protein Ngar_c24220	No	0	1883208	1883885	+		Candidatus_Nitrososphaera_gargensis_Ga9_2_ui	NC_018719	chromosome									
	408405021	hypothetical protein Ngar_c24230	No	0	1884537	1884884	+		Candidatus_Nitrososphaera_gargensis_Ga9_2_ui	NC_018719	chromosome		arCOG08800	2 (-)	S		PKD domain containing protein			
===								6	Candidatus_Nitrososphaera_gargensis_Ga9_2_ui	NC_018719	chromosome									
	408405178	zinc finger C2H2 domain-containing protein	No	4	2008918	2009622	+		Candidatus_Nitrososphaera_gargensis_Ga9_2_ui	NC_018719	chromosome		arCOG06357	1 (-)	S		Uncharacterized conserved protein			
	408405179	hypothetical protein Ngar_c25830	No	0	2009785	2009979	-		Candidatus_Nitrososphaera_gargensis_Ga9_2_ui	NC_018719	chromosome									
	408405180	hypothetical protein Ngar_c25840	No	0	2010339	2010488	+		Candidatus_Nitrososphaera_gargensis_Ga9_2_ui	NC_018719	chromosome									
	408405181	hypothetical protein Ngar_c25850	No	0	2010566	2010703	-		Candidatus_Nitrososphaera_gargensis_Ga9_2_ui	NC_018719	chromosome									
	408405182	hypothetical protein Ngar_c25860	No	0	2010748	2010903	-		Candidatus_Nitrososphaera_gargensis_Ga9_2_ui	NC_018719	chromosome	clust_488								
	408405183	poly-beta-hydroxybutyrate polymerase	No	0	2011054	2012529	-		Candidatus_Nitrososphaera_gargensis_Ga9_2_ui	NC_018719	chromosome	arCOG06343,a	arCOG06343	1 (-)	S		Uncharacterized conserved protein	3E-28	COG3243	Poly(3-hydroxyalkanoate) synthetase
===								8	Candidatus_Nitrososphaera_gargensis_Ga9_2_ui	NC_018719	chromosome									
	408405196	hypothetical protein Ngar_c26000	No	0	2023062	2023481	-		Candidatus_Nitrososphaera_gargensis_Ga9_2_ui	NC_018719	chromosome		arCOG00516	6 (-)	R		Predicted flavin-nucleotide-binding protein	0.00001	pfam01243	Pyridoxamine 5'-phosphate oxidase.
	408405197	zinc finger C2H2 domain-containing protein	No	0	2023824	2024300	+		Candidatus_Nitrososphaera_gargensis_Ga9_2_ui	NC_018719	chromosome	clust_221								
	408405198	hypothetical protein Ngar_c26020	No	0	2024981	2025193	+		Candidatus_Nitrososphaera_gargensis_Ga9_2_ui	NC_018719	chromosome									
	408405199	hypothetical protein Ngar_c26030	No	0	2025378	2025584	-		Candidatus_Nitrososphaera_gargensis_Ga9_2_ui	NC_018719	chromosome									
	408405200	hypothetical protein Ngar_c26040	No	1	2025569	2025757	+		Candidatus_Nitrososphaera_gargensis_Ga9_2_ui	NC_018719	chromosome									
	408405201	hypothetical protein Ngar_c26050	No	0	2026094	2026270	+		Candidatus_Nitrososphaera_gargensis_Ga9_2_ui	NC_018719	chromosome									
	408405202	hypothetical protein Ngar_c26060	No	1	2026356	2026550	+		Candidatus_Nitrososphaera_gargensis_Ga9_2_ui	NC_018719	chromosome		arCOG08033	4 (-)	S		Uncharacterized protein			
	408405203	hypothetical protein Ngar_c26070	No	0	2026691	2027245	+		Candidatus_Nitrososphaera_gargensis_Ga9_2_ui	NC_018719	chromosome									
===								5	Candidatus_Nitrososphaera_gargensis_Ga9_2_ui	NC_018719	chromosome									
	408405213	hypothetical protein Ngar_c26170	No	0	2033619	2033948	-		Candidatus_Nitrososphaera_gargensis_Ga9_2_ui	NC_018719	chromosome									
	408405214	hypothetical protein Ngar_c26180	No	0	2034048	2034539	-		Candidatus_Nitrososphaera_gargensis_Ga9_2_ui	NC_018719	chromosome	clust_197						0.00009	COG0840	Methyl-accepting chemotaxis protein
	408405215	hypothetical protein Ngar_c26190	No	0	2034612	2035013	-		Candidatus_Nitrososphaera_gargensis_Ga9_2_ui	NC_018719	chromosome		arCOG08640	1 (-)	R		Predicted RNA-binding protein containing PUA-d	3E-27	pfam01878	EVE domain. This domain was formerly kno
	408405216	hypothetical protein Ngar_c26200	No	0	2035044	2035415	-		Candidatus_Nitrososphaera_gargensis_Ga9_2_ui	NC_018719	chromosome									
	408405217	hypothetical protein Ngar_c26210	No	0	2035420	2035680	-		Candidatus_Nitrososphaera_gargensis_Ga9_2_ui	NC_018719	chromosome									
===								7	Candidatus_Nitrososphaera_gargensis_Ga9_2_ui	NC_018719	chromosome									
	408405264	ATP-dependent helicase	No	0	2073634	2076447	-		Candidatus_Nitrososphaera_gargensis_Ga9_2_ui	NC_018719	chromosome		arCOG00557	3 (-)	R	Lhr	Lhr-like helicase	2E-133	COG1201	Lhr-like helicases
	408405265	hypothetical protein Ngar_c26700	No	0	2077215	2077343	+		Candidatus_Nitrososphaera_gargensis_Ga9_2_ui	NC_018719	chromosome									
	408405266	hypothetical protein Ngar_c26710	No	1	2077428	2077697	-		Candidatus_Nitrososphaera_gargensis_Ga9_2_ui	NC_018719	chromosome									
	408405267	hypothetical protein Ngar_c26720	Yes	1	2078211	2078681	+		Candidatus_Nitrososphaera_gargensis_Ga9_2_ui	NC_018719	chromosome		arCOG09427	1	S		Uncharacterized conserved protein			
	408405268	hypothetical protein Ngar_c26730	No	0	2078837	2079151	+		Candidatus_Nitrososphaera_gargensis_Ga9_2_ui	NC_018719	chromosome									

408405269	hypothetical protein Ngar_c26740	No	0	2079176	2079334	+		Candidatus_Nitrososphaera_gargensis_Ga9_2_ui	NC_018719	chromosome										
408405270	50S ribosomal protein L15e	No	0	2079636	2080061	-		Candidatus_Nitrososphaera_gargensis_Ga9_2_ui	NC_018719	chromosome		arCOG04209	3	(-)	J	RPL15A	Ribosomal protein L15E	2E-42	COG1632	Ribosomal protein L15E
===							6	Candidatus_Nitrososphaera_gargensis_Ga9_2_ui	NC_018719	chromosome										
408405308	hypothetical protein Ngar_c27130	No	0	2115442	2115708	+		Candidatus_Nitrososphaera_gargensis_Ga9_2_ui	NC_018719	chromosome										
408405309	hypothetical protein Ngar_c27140	Yes	0	2115780	2115962	-		Candidatus_Nitrososphaera_gargensis_Ga9_2_ui	NC_018719	chromosome										
408405310	hypothetical protein Ngar_c27150	No	0	2116215	2116454	-		Candidatus_Nitrososphaera_gargensis_Ga9_2_ui	NC_018719	chromosome										
408405311	polyketide cyclase/dehydrase	No	0	2117031	2117465	+		Candidatus_Nitrososphaera_gargensis_Ga9_2_ui	NC_018719	chromosome		arCOG08713	1	(-)	I		Oligoketide cyclase/lipid transport protein	0.00000001	pfam03364	Polyketide cyclase / dehydrase and lipid
408405312	hypothetical protein Ngar_c27170	No	0	2117566	2117760	-		Candidatus_Nitrososphaera_gargensis_Ga9_2_ui	NC_018719	chromosome										
408405313	hypothetical protein Ngar_c27180	Yes	1	2117822	2118190	+		Candidatus_Nitrososphaera_gargensis_Ga9_2_ui	NC_018719	chromosome										
===							5	Candidatus_Nitrososphaera_gargensis_Ga9_2_ui	NC_018719	chromosome										
408405344	hypothetical protein Ngar_c27490	No	0	2140652	2140879	+		Candidatus_Nitrososphaera_gargensis_Ga9_2_ui	NC_018719	chromosome										
408405345	hypothetical protein Ngar_c27500	No	0	2141070	2141522	+		Candidatus_Nitrososphaera_gargensis_Ga9_2_ui	NC_018719	chromosome										
408405346	hypothetical protein Ngar_c27510	No	0	2141515	2141736	+		Candidatus_Nitrososphaera_gargensis_Ga9_2_ui	NC_018719	chromosome										
408405347	HEAT repeat-containing PBS lyase	No	0	2142145	2142591	+		Candidatus_Nitrososphaera_gargensis_Ga9_2_ui	NC_018719	chromosome		arCOG04006	3	(-)	S		HEAT repeats containing protein	3E-12	COG1413	FOG: HEAT repeat
408405348	hypothetical protein Ngar_c27530	No	0	2142617	2142934	+		Candidatus_Nitrososphaera_gargensis_Ga9_2_ui	NC_018719	chromosome										
===							9	Candidatus_Nitrososphaera_gargensis_Ga9_2_ui	NC_018719	chromosome										
408405364	hypothetical protein Ngar_c27690	No	0	2155818	2156024	-		Candidatus_Nitrososphaera_gargensis_Ga9_2_ui	NC_018719	chromosome										
408405365	hypothetical protein Ngar_c27700	No	0	2156081	2156341	-		Candidatus_Nitrososphaera_gargensis_Ga9_2_ui	NC_018719	chromosome										
408405366	hypothetical protein Ngar_c27710	No	0	2156338	2156682	-		Candidatus_Nitrososphaera_gargensis_Ga9_2_ui	NC_018719	chromosome		arCOG01242	14	(-)	L	XerC	Integrase			
408405367	hypothetical protein Ngar_c27720	No	0	2156797	2157255	-		Candidatus_Nitrososphaera_gargensis_Ga9_2_ui	NC_018719	chromosome										
408405368	hypothetical protein Ngar_c27730	No	0	2157509	2158294	+		Candidatus_Nitrososphaera_gargensis_Ga9_2_ui	NC_018719	chromosome										
408405369	hypothetical protein Ngar_c27740	No	5	2158406	2158936	+		Candidatus_Nitrososphaera_gargensis_Ga9_2_ui	NC_018719	chromosome										
408405370	hypothetical protein Ngar_c27750	No	0	2159009	2159218	+		Candidatus_Nitrososphaera_gargensis_Ga9_2_ui	NC_018719	chromosome										
408405371	hypothetical protein Ngar_c27760	No	0	2159381	2159629	+		Candidatus_Nitrososphaera_gargensis_Ga9_2_ui	NC_018719	chromosome										
408405372	hypothetical protein Ngar_c27770	No	0	2159875	2160429	-		Candidatus_Nitrososphaera_gargensis_Ga9_2_ui	NC_018719	chromosome		arCOG04291	2	(-)	R		Sugar isomerase related protein	2E-37	COG1801	Uncharacterized conserved protein
===							23	Candidatus_Nitrososphaera_gargensis_Ga9_2_ui	NC_018719	chromosome										
408405381	hypothetical protein Ngar_c27860	No	0	2168593	2169033	+		Candidatus_Nitrososphaera_gargensis_Ga9_2_ui	NC_018719	chromosome										
408405382	hypothetical protein Ngar_c27870	No	0	2169525	2170766	-		Candidatus_Nitrososphaera_gargensis_Ga9_2_ui	NC_018719	chromosome		arCOG01245	15	(-)	L	XerC	Integrase			
408405383	hypothetical protein Ngar_c27880	No	0	2171789	2172577	+		Candidatus_Nitrososphaera_gargensis_Ga9_2_ui	NC_018719	chromosome										
408405384	hypothetical protein Ngar_c27890	No	0	2172598	2172963	+		Candidatus_Nitrososphaera_gargensis_Ga9_2_ui	NC_018719	chromosome										
408405385	hypothetical protein Ngar_c27900	No	0	2172969	2173277	+		Candidatus_Nitrososphaera_gargensis_Ga9_2_ui	NC_018719	chromosome										
408405386	hypothetical protein Ngar_c27910	No	0	2173575	2174132	+		Candidatus_Nitrososphaera_gargensis_Ga9_2_ui	NC_018719	chromosome										
408405387	hypothetical protein Ngar_c27920	No	1	2174249	2174686	+		Candidatus_Nitrososphaera_gargensis_Ga9_2_ui	NC_018719	chromosome										
408405388	IS-4 family transposase	No	0	2174816	2176033	+		Candidatus_Nitrososphaera_gargensis_Ga9_2_ui	NC_018719	chromosome		arCOG03902	8	(-)	L		Transposase			
408405389	hypothetical protein Ngar_c27940	No	0	2176023	2176226	+		Candidatus_Nitrososphaera_gargensis_Ga9_2_ui	NC_018719	chromosome										
408405390	hypothetical protein Ngar_c27950	Yes	1	2176883	2177383	-		Candidatus_Nitrososphaera_gargensis_Ga9_2_ui	NC_018719	chromosome										
408405391	hypothetical protein Ngar_c27960	No	2	2177453	2177689	-		Candidatus_Nitrososphaera_gargensis_Ga9_2_ui	NC_018719	chromosome										
408405392	hypothetical protein Ngar_c27970	No	0	2178077	2178535	+		Candidatus_Nitrososphaera_gargensis_Ga9_2_ui	NC_018719	chromosome										
408405393	hypothetical protein Ngar_c27980	No	0	2179106	2179729	-		Candidatus_Nitrososphaera_gargensis_Ga9_2_ui	NC_018719	chromosome								0.00007	COG5185	Protein involved in chromosome segregati
408405394	hypothetical protein Ngar_c27990	No	1	2179763	2179927	+		Candidatus_Nitrososphaera_gargensis_Ga9_2_ui	NC_018719	chromosome										
408405395	hypothetical protein Ngar_c28000	No	0	2179983	2180159	-		Candidatus_Nitrososphaera_gargensis_Ga9_2_ui	NC_018719	chromosome										
408405396	hypothetical protein Ngar_c28010	No	0	2180639	2180872	+		Candidatus_Nitrososphaera_gargensis_Ga9_2_ui	NC_018719	chromosome										
408405397	hypothetical protein with integrase-like domain	No	0	2180955	2182445	-		Candidatus_Nitrososphaera_gargensis_Ga9_2_ui	NC_018719	chromosome		arCOG01242	14	(-)	L	XerC	Integrase			
408405398	integrase family protein	No	0	2182862	2183575	+		Candidatus_Nitrososphaera_gargensis_Ga9_2_ui	NC_018719	chromosome								2E-11	pfam12138	Spherulation-specific family 4. This pro
408405399	hypothetical protein Ngar_c28050	No	0	2183559	2183771	-		Candidatus_Nitrososphaera_gargensis_Ga9_2_ui	NC_018719	chromosome										
408405400	hypothetical protein Ngar_c28060	No	0	2184194	2184307	-		Candidatus_Nitrososphaera_gargensis_Ga9_2_ui	NC_018719	chromosome										
408405401	hypothetical protein Ngar_c28070	No	0	2184466	2184579	-		Candidatus_Nitrososphaera_gargensis_Ga9_2_ui	NC_018719	chromosome	clust_510									
408405402	hypothetical protein Ngar_c28080	No	0	2184876	2185025	+		Candidatus_Nitrososphaera_gargensis_Ga9_2_ui	NC_018719	chromosome										
408405403	cyclase	No	0	2185085	2185717	-		Candidatus_Nitrososphaera_gargensis_Ga9_2_ui	NC_018719	chromosome		arCOG02462	1	(-)	E		Kynurenine formamidase	4E-39	COG1878	Kynurenine formamidase
===							11	Candidatus_Nitrososphaera_gargensis_Ga9_2_ui	NC_018719	chromosome										
408405427	hypothetical protein Ngar_c28330	No	0	2206562	2206816	+		Candidatus_Nitrososphaera_gargensis_Ga9_2_ui	NC_018719	chromosome										
408405428	integrase family protein	No	0	2206813	2207844	+		Candidatus_Nitrososphaera_gargensis_Ga9_2_ui	NC_018719	chromosome		arCOG01242	14	(-)	L	XerC	Integrase	0.0000009	COG4974	Site-specific recombinase XerD
408405429	zinc finger C2H2 domain-containing protein	No	0	2208300	2208752	-		Candidatus_Nitrososphaera_gargensis_Ga9_2_ui	NC_018719	chromosome										
408405430	hypothetical protein Ngar_c28360	No	0	2209007	2209186	-		Candidatus_Nitrososphaera_gargensis_Ga9_2_ui	NC_018719	chromosome										
408405431	hypothetical protein Ngar_c28370	No	0	2209251	2209442	+		Candidatus_Nitrososphaera_gargensis_Ga9_2_ui	NC_018719	chromosome	clust_439									
408405432	iron sulfur domain-containing protein, CDGSH-t	No	0	2209698	2209898	+		Candidatus_Nitrososphaera_gargensis_Ga9_2_ui	NC_018719	chromosome		arCOG01667	2	(-)	S		Zinc finger domain containing protein (CDGSH-typ	0.00000002	pfam09360	Iron-binding zinc finger CDGSH type. The
408405433	hypothetical protein Ngar_c28390	No	4	2210105	2210647	-		Candidatus_Nitrososphaera_gargensis_Ga9_2_ui	NC_018719	chromosome		arCOG10856	3		S		Uncharacterized conserved membrane protein			
408405434	hypothetical protein Ngar_c28400	Yes	1	2211506	2211835	+		Candidatus_Nitrososphaera_gargensis_Ga9_2_ui	NC_018719	chromosome										
408405435	hypothetical protein Ngar_c28410	No	1	2212682	2212909	-		Candidatus_Nitrososphaera_gargensis_Ga9_2_ui	NC_018719	chromosome		arCOG02298	6	(-)	N	FlaK/PulC	Peptidase A24A, prepilin type IV			
408405436	hypothetical protein Ngar_c28420	No	0	2213190	2213513	-		Candidatus_Nitrososphaera_gargensis_Ga9_2_ui	NC_018719	chromosome										
408405437	hypothetical protein Ngar_c28430	No	0	2213841	2214062	+		Candidatus_Nitrososphaera_gargensis_Ga9_2_ui	NC_018719	chromosome										
===							7	Candidatus_Nitrososphaera_gargensis_Ga9_2_ui	NC_018719	chromosome										
408405463	blue (Type 1) copper domain-containing protei	No	1	2235991	2236995	+		Candidatus_Nitrososphaera_gargensis_Ga9_2_ui	NC_018719	chromosome		arCOG02926	8	(-)	C	PetE	Plastocyanin			
408405464	hypothetical protein Ngar_c28710	No	0	2237305	2237508	-		Candidatus_Nitrososphaera_gargensis_Ga9_2_ui	NC_018719	chromosome										
408405465	hypothetical protein Ngar_c28720	Yes	1	2238142	2238819	+		Candidatus_Nitrososphaera_gargensis_Ga9_2_ui	NC_018719	chromosome										
408405466	hypothetical protein Ngar_c28730	Yes	1	2238824	2239594	+		Candidatus_Nitrososphaera_gargensis_Ga9_2_ui	NC_018719	chromosome										
408405467	hypothetical protein Ngar_c28740	No	0	2239893	2240165	-		Candidatus_Nitrososphaera_gargensis_Ga9_2_ui	NC_018719	chromosome										
408405468	signal transduction response regulator, receive	No	0	2240522	2240908	+		Candidatus_Nitrososphaera_gargensis_Ga9_2_ui	NC_018719	chromosome		arCOG02385	2	(-)	T	CheY	Rec domain	7E-11	COG2204	Response regulator containing CheY-like
408405469	hypothetical protein Ngar_c28760	No	5	2241137	2241967	+		Candidatus_Nitrososphaera_gargensis_Ga9_2_ui	NC_018719	chromosome										
===							8	Candidatus_Nitrososphaera_gargensis_Ga9_2_ui	NC_018719	chromosome										
408405494	hypothetical protein Ngar_c29010	Yes	1	2260119	2260835	+		Candidatus_Nitrososphaera_gargensis_Ga9_2_ui	NC_018719	chromosome										
408405495	marR family transcriptional regulator	No	0	2260848	2261333	-		Candidatus_Nitrososphaera_gargensis_Ga9_2_ui	NC_018719	chromosome		arCOG03182	3	(-)	K	MarR	Transcriptional regulator, MarR family	0.00000005	COG1846	Transcriptional regulators
408405496	hypothetical protein Ngar_c29030	Yes	4	2261400	2261996	+		Candidatus_Nitrososphaera_gargensis_Ga9_2_ui	NC_018719	chromosome										
408405497	hypothetical protein Ngar_c29040	No	0	2262099	2262563	+		Candidatus_Nitrososphaera_gargensis_Ga9_2_ui	NC_018719	chromosome	clust_226							0.00003	COG0840	Methyl-accepting chemotaxis protein
408405498	hypothetical protein Ngar_c29050	No	1	2262645	2262854	+		Candidatus_Nitrososphaera_gargensis_Ga9_2_ui	NC_018719	chromosome										
408405499	hypothetical protein Ngar_c29060	No	0	2262893	2263702	+		Candidatus_Nitrososphaera_gargensis_Ga9_2_ui	NC_018719	chromosome										
408405500	hypothetical protein Ngar_c29070	No	0	2263988	2264641	-		Candidatus_Nitrososphaera_gargensis_Ga9_2_ui	NC_018719	chromosome										
408405501	hypothetical protein Ngar_c29080	No	0	2264984	2265718	-		Candidatus_Nitrososphaera_gargensis_Ga9_2_ui	NC_018719	chromosome		arCOG04347	4	(-)	Q		SAM-dependent methyltransferase			
===							11	Candidatus_Nitrososphaera_gargensis_Ga9_2_ui	NC_018719	chromosome										
408405509	hypothetical protein Ngar_c29160	No	0	2276990	2277271	-		Candidatus_Nitrososphaera_gargensis_Ga9_2_ui	NC_018719	chromosome										
408405510	hypothetical protein Ngar_c29170	No	0	2277271	2277879	-		Candidatus_Nitrososphaera_gargensis_Ga9_2_ui	NC_018719	chromosome										
408405511	hypothetical protein Ngar_c29180	No	0	2277936	2279519	-		Candidatus_Nitrososphaera_gargensis_Ga9_2_ui	NC_018719	chromosome	arCOG03177,a	arCOG03177	3	(-)	K	MarR	Transcriptional regulator, MarR family			
408405512	hypothetical protein Ngar_c29190	No	0	2279516	2279674	-		Candidatus_Nitrososphaera_gargensis_Ga9_2_ui	NC_018719	chromosome										
408405513	hypothetical protein Ngar_c29200	No	0	2279962	2280954	-		Candidatus_Nitrososphaera_gargensis_Ga9_2_ui	NC_018719	chromosome										
408405514	integrase family protein	No	0	2281064	2282428	-		Candidatus_Nitrososphaera_gargensis_Ga9_2_ui	NC_018719	chromosome		arCOG01242	14	(-)	L	XerC	Integrase			
408405515	hypothetical protein Ngar_c29230	No	0	2282971	2283126	+		Candidatus_Nitrososphaera_gargensis_Ga9_2_ui	NC_018719	chromosome										
408405516	hypothetical protein Ngar_c29240	No	0	2283156	2283533	-		Candidatus_Nitrososphaera_gargensis_Ga9_2_ui	NC_018719	chromosome										
408405517	hypothetical protein Ngar_c29250	No	0	2283543	2283926	-		Candidatus_Nitrososphaera_gargensis_Ga9_2_ui	NC_018719	chromosome										
408405518	hypothetical protein Ngar_c29260	No	0	2284315	2285058	-		Candidatus_Nitrososphaera_gargensis_Ga9_2_ui	NC_018719	chromosome										
408405519	transcriptional regulator, TrmB	No	0	2285107	2285928	-		Candidatus_Nitrososphaera_gargensis_Ga9_2_ui	NC_018719	chromosome		arCOG02037	2	(-)	K		Sugar-specific transcriptional regulator TrmB	2E-15	COG1378	Predicted transcriptional regulators
===							6	Candidatus_Nitrososphaera_gargensis_Ga9_2_ui	NC_018719	chromosome										
408405521	ATPase, AAA-5	No	0	2287895	2288962	-		Candidatus_Nitrososphaera_gargensis_Ga9_2_ui	NC_018719	chromosome		arCOG00441	3	(-)	R		MoxR-like ATPase	2E-15	COG5271	AAA ATPase containing von Willebrand fac
408405522	hypothetical protein Ngar_c29300	No	0	2289066	2289713	+		Candidatus_Nitrososphaera_gargensis_Ga9_2_ui	NC_018719	chromosome										
408405523	hypothetical protein Ngar_c29310	No	2	2289876	2290082	+		Candidatus_Nitrososphaera_gargensis_Ga9_2_ui	NC_018719	chromosome										
408405524	hypothetical protein Ngar_c29320	No	0	2290355	2291026	-		Candidatus_Nitrososphaera_gargensis_Ga9_2_ui	NC_018719	chromosome										
408405525	hypothetical protein Ngar_c29330	No	0	2291029	2291538	-		Candidatus_Nitrososphaera_gargensis_Ga9_2_ui	NC_018719	chromosome										
408405526	hypothetical protein possibly linked to secE2	No	1	2291677	2292216	+		Candidatus_Nitrososphaera_gargensis_Ga9_2_ui	NC_018719	chromosome		arCOG03871	6	(-)	N		Flagellar protein G			
===							8	Candidatus_Nitrososphaera_gargensis_Ga9_2_ui	NC_018719	chromosome										
408405546	hypothetical protein Ngar_c29540	No	0	2311602	2311979	+		Candidatus_Nitrososphaera_gargensis_Ga9_2_ui	NC_018719	chromosome										
408405547	hypothetical protein Ngar_c29550	No	0	2311966	2312199	+		Candidatus_Nitrososphaera_gargensis_Ga9_2_ui	NC_018719	chromosome										
408405548	hypothetical protein Ngar_c29560	No	0	2312210	2312575	-		Candidatus_Nitrososphaera_gargensis_Ga9_2_ui	NC_018719	chromosome		arCOG08677	2	(-)	S		Zn-ribbon domain containing protein			
408405549	hypothetical protein Ngar_c29570	No	0	2312684	2313076	+		Candidatus_Nitrososphaera_gargensis_Ga9_2_ui	NC_018719	chromosome										
408405550	hypothetical protein Ngar_c29580	No	0	2313616	2313816	-		Candidatus_Nitrososphaera_gargensis_Ga9_2_ui	NC_018719	chromosome										
408405551	hypothetical protein Ngar_c29590	No	0	2314188	2314328	-		Candidatus_Nitrososphaera_gargensis_Ga9_2_ui	NC_018719	chromosome										
408405552	hypothetical protein Ngar_c29600	No	0	2314463	2314582	-		Candidatus_Nitrososphaera_gargensis_Ga9_2_ui	NC_018719	chromosome										
408405553	metal dependent phosphohydrolase	No	0	2315002	2315625	+		Candidatus_Nitrososphaera_gargensis_Ga9_2_ui	NC_018719	chromosome		arCOG01860	1	(-)	R		Predicted HD superfamily hydrolase	3E-14	COG1418	Predicted HD superfamily hydrolase
===							5	Candidatus_Nitrososphaera_gargensis_Ga9_2_ui	NC_018719	chromosome										
408405616	hypothetical protein Ngar_c30260	Yes	1	2361212	2361568	-		Candidatus_Nitrososphaera_gargensis_Ga9_2_ui	NC_018719	chromosome										
408405617	hypothetical protein Ngar_c30270	No	0	2362092	2362430	+		Candidatus_Nitrososphaera_gargensis_Ga9_2_ui	NC_018719	chromosome										
408405618	hypothetical protein Ngar_c30280, partial	No	0	2362624	2362824	-		Candidatus_Nitrososphaera_gargensis_Ga9_2_ui	NC_018719	chromosome		arCOG01055	9	(-)	K		Predicted transcriptional regulator	0.000002	COG3432	Predicted transcriptional regulator
408405619	hypothetical protein Ngar_c30290	No	0	2362984	2363220	-		Candidatus_Nitrososphaera_gargensis_Ga9_2_ui	NC_018719	chromosome										
408405620	hypothetical protein Ngar_c30300	Yes	1	2363520	2366378	+		Candidatus_Nitrososphaera_gargensis_Ga9_2_ui	NC_018719	chromosome										
===							6	Candidatus_Nitrososphaera_gargensis_Ga9_2_ui	NC_018719	chromosome										
408405627	thioredoxin	No	0	2374085	2374525	+		Candidatus_Nitrososphaera_gargensis_Ga9_2_ui	NC_018719	chromosome		arCOG01972	5	(-)	O	TrxA	Thiol-disulfide isomerase or thioredoxin	3E-28	COG3118	Thioredoxin domain-containing protein
408405628	hypothetical protein Ngar_c30380	No	0	2374555	2374761	-		Candidatus_Nitrososphaera_gargensis_Ga9_2_ui	NC_018719	chromosome										
408405629	hypothetical protein Ngar_c30390	No	0	2374976	2375476	+		Candidatus_Nitrososphaera_gargensis_Ga9_2_ui	NC_018719	chromosome										
408405630	hypothetical protein Ngar_c30400	No	0	2375548	2375763	+		Candidatus_Nitrososphaera_gargensis_Ga9_2_ui	NC_018719	chromosome										
408405631	hypothetical protein Ngar_c30410	No	1	2375813	2376106	+		Candidatus_Nitrososphaera_gargensis_Ga9_2_ui	NC_018719	chromosome										
408405632	translation initiation factor 2, subunit beta	No	0	2376340	2376774	-		Candidatus_Nitrososphaera_gargensis_Ga9_2_ui	NC_018719	chromosome		arCOG01640	1	(-)	J	GCD7	Translation initiation factor 2, beta subunit (eIF-2be	7E-29	COG1601	Translation initiation factor 2, beta su
===							8	Candidatus_Nitrososphaera_gargensis_Ga9_2_ui	NC_018719	chromosome										
408405643	4Fe-4S ferredoxin iron-sulfur binding domain-c	No	0	2387771	2388307	+		Candidatus_Nitrososphaera_gargensis_Ga9_2_ui	NC_018719	chromosome		arCOG04548	2	(-)	C		Ferredoxin	0.00006	COG1146	Ferredoxin
408405644	hypothetical protein Ngar_c30540	No	0	2388333	2388950	+		Candidatus_Nitrososphaera_gargensis_Ga9_2_ui	NC_018719	chromosome										
408405645	hypothetical protein Ngar_c30550	No	0	2388939	2389493	-		Candidatus_Nitrososphaera_gargensis_Ga9_2_ui	NC_018719	chromosome										
408405646	hypothetical protein Ngar_c30560	No	0	2389501	2389770	-		Candidatus_Nitrososphaera_gargensis_Ga9_2_ui	NC_018719	chromosome										
408405647	hypothetical protein Ngar_c30570	No	0	2389956	2390126	-		Candidatus_Nitrososphaera_gargensis_Ga9_2_ui	NC_018719	chromosome										
408405648	AAA ATPase	No	0	2390405	2392600	+		Candidatus_Nitrososphaera_gargensis_Ga9_2_ui	NC_018719	chromosome		arCOG01308	10	(-)	O	Cdc48	ATPase of the AAA+ class , CDC48 family		COG0464	ATPases of the AAA+ class
408405649	hypothetical protein Ngar_c30590	No	0	2393277	2393555	-		Candidatus_Nitrososphaera_gargensis_Ga9_2_ui	NC_018719	chromosome										
408405650	zinc finger C2H2 domain-containing protein	No	0	2393996	2394202	-		Candidatus_Nitrososphaera_gargensis_Ga9_2_ui	NC_018719	chromosome										
===							12	Candidatus_Nitrososphaera_gargensis_Ga9_2_ui	NC_018719	chromosome										
408405691	hypothetical protein Ngar_c31010	No	0	2429616	2429891	-		Candidatus_Nitrososphaera_gargensis_Ga9_2_ui	NC_018719	chromosome										
408405692	hypothetical protein Ngar_c31020	No	0	2430024	2430344	-		Candidatus_Nitrososphaera_gargensis_Ga9_2_ui	NC_018719	chromosome										
408405693	hypothetical protein Ngar_c31030	No	0	2430740	2430925	-		Candidatus_Nitrososphaera_gargensis_Ga9_2_ui	NC_018719	chromosome										
408405694	hypothetical protein Ngar_c31040	No	0	2430991	2431371	-		Candidatus_Nitrososphaera_gargensis_Ga9_2_ui	NC_018719	chromosome		arCOG00509	3	(-)	C	FpaA	Uncharacterized flavoprotein			
408405695	hypothetical protein Ngar_c31050	No	0	2431847	2432239	+		Candidatus_Nitrososphaera_gargensis_Ga9_2_ui	NC_018719	chromosome								2E-22	COG5588	Uncharacterized conserved protein
408405696	hypothetical protein Ngar_c31060	No	0	2432746	2433072	-		Candidatus_Nitrososphaera_gargensis_Ga9_2_ui	NC_018719	chromosome	clust_316									
408405697	zinc finger C2H2 domain-containing protein	No	0	2433575	2433733	-		Candidatus_Nitrososphaera_gargensis_Ga9_2_ui	NC_018719	chromosome										
408405698	hypothetical protein Ngar_c31080	No	0	2433726	2433893	-		Candidatus_Nitrososphaera_gargensis_Ga9_2_ui	NC_018719	chromosome										
408405699	hypothetical protein Ngar_c31090	No	0	2434164	2434526	+		Candidatus_Nitrososphaera_gargensis_Ga9_2_ui	NC_018719	chromosome		arCOG08684	7	(-)	R		Roadblock/LC7 domain superfamily protein			
408405700	hypothetical protein Ngar_c31100	No	0	2434534	2434698	-		Candidatus_Nitrososphaera_gargensis_Ga9_2_ui	NC_018719	chromosome										
408405701	hypothetical protein Ngar_c31110	No	0	2434929	2435111	+		Candidatus_Nitrososphaera_gargensis_Ga9_2_ui	NC_018719	chromosome										
408405702	hypothetical protein Ngar_c31120	No	0	2435205	2435387	-		Candidatus_Nitrososphaera_gargensis_Ga9_2_ui	NC_018719	chromosome										
===							8	Candidatus_Nitrososphaera_gargensis_Ga9_2_ui	NC_018719	chromosome										
408405715	hypothetical protein Ngar_c31250	No	0	2448520	2448660	+		Candidatus_Nitrososphaera_gargensis_Ga9_2_ui	NC_018719	chromosome										
408405716	signal transduction histidine kinase, with phosp	No	0	2449869	2451221	+		Candidatus_Nitrososphaera_gargensis_Ga9_2_ui	NC_018719	chromosome	arCOG02039,a	arCOG02039	1	(-)	I	Cls	Phosphatidylserine/phosphatidylglycerophospha	5E-29	COG2205	Osmosensitive K+ channel histidine kinas
408405717	hypothetical protein Ngar_c31270	No	0	2452082	2452465	+		Candidatus_Nitrososphaera_gargensis_Ga9_2_ui	NC_018719	chromosome										
408405718	hypothetical protein Ngar_c31280	No	0	2452462	2452659	+		Candidatus_Nitrososphaera_gargensis_Ga9_2_ui	NC_018719	chromosome										
408405719	hypothetical protein Ngar_c31290	No	1	2453066	2453251	+		Candidatus_Nitrososphaera_gargensis_Ga9_2_ui	NC_018719	chromosome										
408405720	hypothetical protein Ngar_c31300	No	1	2453362	2453556	-		Candidatus_Nitrososphaera_gargensis_Ga9_2_ui	NC_018719	chromosome										
408405721	hypothetical protein Ngar_c31310	No	0	2453854	2454192	+		Candidatus_Nitrososphaera_gargensis_Ga9_2_ui	NC_018719	chromosome										
408405722	phospholipase/carboxylesterase family proteir	No	0	2454238	2454903	-		Candidatus_Nitrososphaera_gargensis_Ga9_2_ui	NC_018719	chromosome		arCOG06256	1	(-)	R		Predicted esterase	7E-50	COG0400	Predicted esterase
===							8	Candidatus_Nitrososphaera_gargensis_Ga9_2_ui	NC_018719	chromosome										
408405745	hypothetical protein Ngar_c31550	No	0	2474216	2474500	+		Candidatus_Nitrososphaera_gargensis_Ga9_2_ui	NC_018719	chromosome										
408405746	hypothetical protein Ngar_c31560	No	0	2474810	2474995	+		Candidatus_Nitrososphaera_gargensis_Ga9_2_ui	NC_018719	chromosome										
408405747	phosphomethylpyrimidine synthase	No	0	2475131	2476468	+		Candidatus_Nitrososphaera_gargensis_Ga9_2_ui	NC_018719	chromosome		arCOG02741	4	(-)	H	ThiC	Thiamine biosynthesis protein ThiC	1E-152	pfam01964	ThiC family. ThiC is found within the th
408405748	hypothetical protein Ngar_c31580	No	0	2477328	2477606	-		Candidatus_Nitrososphaera_gargensis_Ga9_2_ui	NC_018719	chromosome										
408405749	hypothetical protein Ngar_c31590	No	0	2477596	2477766	-		Candidatus_Nitrososphaera_gargensis_Ga9_2_ui	NC_018719	chromosome	clust_488									
408405750	hypothetical protein Ngar_c31600	No	0	2479369	2479635	+		Candidatus_Nitrososphaera_gargensis_Ga9_2_ui	NC_018719	chromosome										
408405751	hypothetical protein Ngar_c31610	No	0	2479946	2480254	+		Candidatus_Nitrososphaera_gargensis_Ga9_2_ui	NC_018719	chromosome		arCOG08677	2	(-)	S		Zn-ribbon domain containing protein			
408405752	hypothetical protein Ngar_c31620	No	0	2480365	2480958	-		Candidatus_Nitrososphaera_gargensis_Ga9_2_ui	NC_018719	chromosome										
===							9	Candidatus_Nitrososphaera_gargensis_Ga9_2_ui	NC_018719	chromosome										
408405762	RNA methyltransferase	No	0	243	2489680	-		Candidatus_Nitrososphaera_gargensis_Ga9_2_ui	NC_018719	chromosome		arCOG01018	1	(-)	J	LasT	rRNA methylase	2E-31	COG0565	rRNA methylase
408405763	hypothetical protein Ngar_c31730	No	0	2489717	2490052	-		Candidatus_Nitrososphaera_gargensis_Ga9_2_ui	NC_018719	chromosome										
408405764	hypothetical protein Ngar_c31740	No	0	2490134	2490529	+		Candidatus_Nitrososphaera_gargensis_Ga9_2_ui	NC_018719	chromosome								1E-38	COG5588	Uncharacterized conserved protein
408405765	hypothetical protein Ngar_c31750	No	0	2490819	2491061	-		Candidatus_Nitrososphaera_gargensis_Ga9_2_ui	NC_018719	chromosome										
408405766	hypothetical protein Ngar_c31760	Yes	0	2491434	2492015	+		Candidatus_Nitrososphaera_gargensis_Ga9_2_ui	NC_018719	chromosome										
408405767	hypothetical protein Ngar_c31770	Yes	1	2492024	2492689	-		Candidatus_Nitrososphaera_gargensis_Ga9_2_ui	NC_018719	chromosome										
408405768	hypothetical protein Ngar_c31780	Yes	0	2492765	2493325	+		Candidatus_Nitrososphaera_gargensis_Ga9_2_ui	NC_018719	chromosome										
408405769	hypothetical protein Ngar_c31790	No	10	2493357	2494433	+		Candidatus_Nitrososphaera_gargensis_Ga9_2_ui	NC_018719	chromosome		arCOG10517	1	(-)	S		Uncharacterized conserved protein			
408405770	hypothetical protein Ngar_c31800	No	0	2494501	2494740	+		Candidatus_Nitrososphaera_gargensis_Ga9_2_ui	NC_018719	chromosome										
===							7	Candidatus_Nitrososphaera_gargensis_Ga9_2_ui	NC_018719	chromosome										
408405828	hypothetical protein Ngar_c32380	No	0	2542059	2542808	+		Candidatus_Nitrososphaera_gargensis_Ga9_2_ui	NC_018719	chromosome		arCOG01057	21	(-)	K		Predicted transcriptional regulator			
408405829	hypothetical protein Ngar_c32390	No	4	2542818	2543333	-		Candidatus_Nitrososphaera_gargensis_Ga9_2_ui	NC_018719	chromosome								2E-19	pfam14342	Domain of unknown function (DUF4396). Th
408405830	hypothetical protein Ngar_c32400	No	0	2543404	2543517	-		Candidatus_Nitrososphaera_gargensis_Ga9_2_ui	NC_018719	chromosome										
408405831	hypothetical protein Ngar_c32410	No	1	2543588	2543731	-		Candidatus_Nitrososphaera_gargensis_Ga9_2_ui	NC_018719	chromosome										
408405832	hypothetical protein Ngar_c32420	Yes	0	2544126	2544251	-		Candidatus_Nitrososphaera_gargensis_Ga9_2_ui	NC_018719	chromosome										
408405833	hypothetical protein Ngar_c32430	No	0	2544514	2544699	-		Candidatus_Nitrososphaera_gargensis_Ga9_2_ui	NC_018719	chromosome										
408405834	hypothetical protein Ngar_c32440, partial	No	0	2544581	2544742	-		Candidatus_Nitrososphaera_gargensis_Ga9_2_ui	NC_018719	chromosome		arCOG10152	1	(-)	S		Uncharacterized conserved protein			
===							16	Candidatus_Nitrososphaera_gargensis_Ga9_2_ui	NC_018719	chromosome										
408405890	hypothetical protein Ngar_c33010	No	0	2595677	2595898	+		Candidatus_Nitrososphaera_gargensis_Ga9_2_ui	NC_018719	chromosome										
408405891	hypothetical protein Ngar_c33020	No	0	2595895	2596065	+		Candidatus_Nitrososphaera_gargensis_Ga9_2_ui	NC_018719	chromosome										
408405892	aldo/keto reductase	No	0	2596171	2597055	+		Candidatus_Nitrososphaera_gargensis_Ga9_2_ui	NC_018719	chromosome		arCOG01619	3	(-)	R	ARA1	Aldo/keto reductase, related to diketogulonate re	3E-56	COG0656	Aldo/keto reductases, related to diketog
408405893	hypothetical protein Ngar_c33040	No	2	2597651	2597923	+		Candidatus_Nitrososphaera_gargensis_Ga9_2_ui	NC_018719	chromosome										
408405894	hypothetical protein Ngar_c33050	No	0	2598069	2598305	-		Candidatus_Nitrososphaera_gargensis_Ga9_2_ui	NC_018719	chromosome										
408405895	hypothetical protein Ngar_c33060	No	0	2598524	2598808	-		Candidatus_Nitrososphaera_gargensis_Ga9_2_ui	NC_018719	chromosome										
408405896	hypothetical protein Ngar_c33070	No	0	2598783	2599151	-		Candidatus_Nitrososphaera_gargensis_Ga9_2_ui	NC_018719	chromosome										
408405897	radical SAM domain-containing protein	No	0	2599664	2600962	-		Candidatus_Nitrososphaera_gargensis_Ga9_2_ui	NC_018719	chromosome		arCOG00934	1	(-)	C	PflX	Radical SAM superfamily enzyme	3E-71	COG1313	Uncharacterized Fe-S protein PflX, homol
408405898	hypothetical protein Ngar_c33090	No	0	2601218	2601985	+		Candidatus_Nitrososphaera_gargensis_Ga9_2_ui	NC_018719	chromosome										
408405899	hypothetical protein Ngar_c33100	No	0	2602046	2602210	+		Candidatus_Nitrososphaera_gargensis_Ga9_2_ui	NC_018719	chromosome										
408405900	hypothetical protein Ngar_c33110	No	0	2602481	2602819	+		Candidatus_Nitrososphaera_gargensis_Ga9_2_ui	NC_018719	chromosome		arCOG08684	7	(-)	R		Roadblock/LC7 domain superfamily protein			
408405901	hypothetical protein Ngar_c33120	No	0	2602841	2603470	-		Candidatus_Nitrososphaera_gargensis_Ga9_2_ui	NC_018719	chromosome										
408405902	hypothetical protein Ngar_c33130	No	0	2603816	2604322	+		Candidatus_Nitrososphaera_gargensis_Ga9_2_ui	NC_018719	chromosome										
408405903	hypothetical protein Ngar_c33140	No	0	2604450	2604701	+		Candidatus_Nitrososphaera_gargensis_Ga9_2_ui	NC_018719	chromosome										
408405904	signal transduction response regulator, receive	No	0	2604789	2605328	+		Candidatus_Nitrososphaera_gargensis_Ga9_2_ui	NC_018719	chromosome		arCOG02391	3	(-)	T	CheY	Rec domain	1E-10	COG3947	Response regulator containing CheY-like
408405905	hypothetical protein Ngar_c33160	Yes	1	2605422	2605649	+		Candidatus_Nitrososphaera_gargensis_Ga9_2_ui	NC_018719	chromosome										
===							8	Candidatus_Nitrososphaera_gargensis_Ga9_2_ui	NC_018719	chromosome										
408405910	sensor histidine kinase	No	0	2608117	2608623	+		Candidatus_Nitrososphaera_gargensis_Ga9_2_ui	NC_018719	chromosome		arCOG02358	4	(-)	T		Signal transduction histidine kinase and HAMP do	1E-17	COG0642	Signal transduction histidine kinase
408405911	hypothetical protein Ngar_c33220	Yes	0	2608704	2609495	-		Candidatus_Nitrososphaera_gargensis_Ga9_2_ui	NC_018719	chromosome										
408405912	hypothetical protein Ngar_c33230	No	0	2609492	2609755	-		Candidatus_Nitrososphaera_gargensis_Ga9_2_ui	NC_018719	chromosome										
408405913	hypothetical protein Ngar_c33240	No	4	2610074	2610679	+		Candidatus_Nitrososphaera_gargensis_Ga9_2_ui	NC_018719	chromosome										
408405914	hypothetical protein Ngar_c33250	No	4	2610718	2611326	+		Candidatus_Nitrososphaera_gargensis_Ga9_2_ui	NC_018719	chromosome										
408405915	hypothetical protein Ngar_c33260	No	0	2611394	2612134	-		Candidatus_Nitrososphaera_gargensis_Ga9_2_ui	NC_018719	chromosome										
408405916	hypothetical protein Ngar_c33270	No	0	2612317	2612511	-		Candidatus_Nitrososphaera_gargensis_Ga9_2_ui	NC_018719	chromosome										
408405917	hypothetical protein Ngar_c33280	No	0	2613058	2613246	+		Candidatus_Nitrososphaera_gargensis_Ga9_2_ui	NC_018719	chromosome		arCOG08662	3	(-)	S		Uncharacterized conserved metal-binding protein			
===							12	Candidatus_Nitrososphaera_gargensis_Ga9_2_ui	NC_018719	chromosome										
408405922	signal transduction response regulator, receive	No	0	2616902	2617300	-		Candidatus_Nitrososphaera_gargensis_Ga9_2_ui	NC_018719	chromosome		arCOG02595	1	(-)	T	OmpR	REC domain	0.00000001	COG2204	Response regulator containing CheY-like
408405923	hypothetical protein Ngar_c33340	No	0	2617447	2617758	+		Candidatus_Nitrososphaera_gargensis_Ga9_2_ui	NC_018719	chromosome										
408405924	hypothetical protein Ngar_c33350	No	0	2617900	2618112	-		Candidatus_Nitrososphaera_gargensis_Ga9_2_ui	NC_018719	chromosome										
408405925	hypothetical protein Ngar_c33360	No	0	2618353	2618790	-		Candidatus_Nitrososphaera_gargensis_Ga9_2_ui	NC_018719	chromosome										
408405926	hypothetical protein Ngar_c33370	No	0	2618898	2619113	-		Candidatus_Nitrososphaera_gargensis_Ga9_2_ui	NC_018719	chromosome										
408405927	hypothetical protein Ngar_c33380	No	0	2619115	2619441	-		Candidatus_Nitrososphaera_gargensis_Ga9_2_ui	NC_018719	chromosome										
408405928	HxlR family transcriptional regulator	No	0	2619820	2619999	+		Candidatus_Nitrososphaera_gargensis_Ga9_2_ui	NC_018719	chromosome		arCOG01057	21	(-)	K		Predicted transcriptional regulator	0.0001	pfam01638	HxlR-like helix-turn-helix. HxlR, a memb
408405929	hypothetical protein Ngar_c33400, partial	No	0	2619939	2620070	+		Candidatus_Nitrososphaera_gargensis_Ga9_2_ui	NC_018719	chromosome										
408405930	hypothetical protein Ngar_c33410	No	0	2620100	2620213	-		Candidatus_Nitrososphaera_gargensis_Ga9_2_ui	NC_018719	chromosome										
408405931	hypothetical protein Ngar_c33420	No	1	2620812	2621024	+		Candidatus_Nitrososphaera_gargensis_Ga9_2_ui	NC_018719	chromosome										
408405932	hypothetical protein Ngar_c33430	Yes	0	2621115	2621360	-		Candidatus_Nitrososphaera_gargensis_Ga9_2_ui	NC_018719	chromosome		arCOG02926	8	(-)	C	PetE	Plastocyanin			
408405933	hypothetical protein Ngar_c33440	No	0	2621534	2621815	-		Candidatus_Nitrososphaera_gargensis_Ga9_2_ui	NC_018719	chromosome										
===							5	Candidatus_Nitrososphaera_gargensis_Ga9_2_ui	NC_018719	chromosome										
408405970	hypothetical protein Ngar_c33810	No	3	2646949	2647197	+		Candidatus_Nitrososphaera_gargensis_Ga9_2_ui	NC_018719	chromosome										
408405971	hemolysin-type calcium-binding region	No	0	2647212	2647808	-		Candidatus_Nitrososphaera_gargensis_Ga9_2_ui	NC_018719	chromosome								1E-32	COG2931	RTX toxins and related Ca2+-binding prot
408405972	cell division protein FtsZ	No	0	2648107	2649150	-		Candidatus_Nitrososphaera_gargensis_Ga9_2_ui	NC_018719	chromosome		arCOG02201	4	(-)	D	FtsZ	Cell division GTPase	8E-85	COG0206	Cell division GTPase
408405973	hypothetical protein Ngar_c33840	No	0	2649428	2649586	+		Candidatus_Nitrososphaera_gargensis_Ga9_2_ui	NC_018719	chromosome										
408405974	hypothetical protein Ngar_c33850	No	1	2649600	2650106	-		Candidatus_Nitrososphaera_gargensis_Ga9_2_ui	NC_018719	chromosome										
===							5	Candidatus_Nitrososphaera_gargensis_Ga9_2_ui	NC_018719	chromosome										
408405993	hypothetical protein Ngar_c34040	No	0	2668777	2668902	+		Candidatus_Nitrososphaera_gargensis_Ga9_2_ui	NC_018719	chromosome										
408405994	hypothetical protein Ngar_c34050	No	0	2669143	2669286	+		Candidatus_Nitrososphaera_gargensis_Ga9_2_ui	NC_018719	chromosome										
408405995	hypothetical protein Ngar_c34060	No	0	2669758	2670426	+		Candidatus_Nitrososphaera_gargensis_Ga9_2_ui	NC_018719	chromosome										
408405996	hypothetical protein Ngar_c34070	Yes	2	2670878	2671735	+		Candidatus_Nitrososphaera_gargensis_Ga9_2_ui	NC_018719	chromosome	arCOG08033,a	arCOG08033	4	(-)	S		Uncharacterized protein			
408405997	hypothetical protein Ngar_c34080	No	0	2671883	2672149	+		Candidatus_Nitrososphaera_gargensis_Ga9_2_ui	NC_018719	chromosome										
===							24	Candidatus_Nitrososphaera_gargensis_Ga9_2_ui	NC_018719	chromosome										
408406127	hypothetical protein Ngar_c35400	No	0	2782767	2783216	+		Candidatus_Nitrososphaera_gargensis_Ga9_2_ui	NC_018719	chromosome		arCOG08730	1	(-)	S		Uncharacterized conserved Zn-binding protein			
408406128	hypothetical protein Ngar_c35410	No	0	2783502	2783657	+		Candidatus_Nitrososphaera_gargensis_Ga9_2_ui	NC_018719	chromosome										
408406129	hypothetical protein Ngar_c35420	Yes	0	2783884	2784468	+		Candidatus_Nitrososphaera_gargensis_Ga9_2_ui	NC_018719	chromosome								0.000000003	pfam06884	Protein of unknown function (DUF1264). T
408406130	hypothetical protein Ngar_c35430	No	1	2784547	2784771	-		Candidatus_Nitrososphaera_gargensis_Ga9_2_ui	NC_018719	chromosome										
408406131	hypothetical protein Ngar_c35440	No	0	2784797	2785003	+		Candidatus_Nitrososphaera_gargensis_Ga9_2_ui	NC_018719	chromosome										
408406132	hypothetical protein Ngar_c35450	No	0	2785085	2785249	+		Candidatus_Nitrososphaera_gargensis_Ga9_2_ui	NC_018719	chromosome										
408406133	hypothetical protein Ngar_c35460	No	2	2785685	2785891	-		Candidatus_Nitrososphaera_gargensis_Ga9_2_ui	NC_018719	chromosome										
408406134	hypothetical protein Ngar_c35470	Yes	1	2786165	2786557	-		Candidatus_Nitrososphaera_gargensis_Ga9_2_ui	NC_018719	chromosome										
408406135	magnesium transporter-C family protein	No	4	2786916	2787359	-		Candidatus_Nitrososphaera_gargensis_Ga9_2_ui	NC_018719	chromosome								7E-25	COG1285	Uncharacterized membrane protein
408406136	AsnC family transcriptional regulator	No	0	2787764	2788066	+		Candidatus_Nitrososphaera_gargensis_Ga9_2_ui	NC_018719	chromosome		arCOG01117	5	(-)	K	Lrp	Lrp/AsnC family C-terminal domain			
408406137	glutamate racemase	No	0	2788377	2789177	+		Candidatus_Nitrososphaera_gargensis_Ga9_2_ui	NC_018719	chromosome								1E-33	COG0796	Glutamate racemase
408406138	undecaprenyl pyrophosphate synthase	No	0	2789254	2790075	+		Candidatus_Nitrososphaera_gargensis_Ga9_2_ui	NC_018719	chromosome		arCOG01532	2	(-)	I	UppS	Undecaprenyl pyrophosphate synthase	2E-73	COG0020	Undecaprenyl pyrophosphate synthase
408406139	hypothetical protein Ngar_c35520	No	1	2790179	2791147	+		Candidatus_Nitrososphaera_gargensis_Ga9_2_ui	NC_018719	chromosome										
408406140	hypothetical protein Ngar_c35530	No	0	2791525	2792829	-		Candidatus_Nitrososphaera_gargensis_Ga9_2_ui	NC_018719	chromosome										
408406141	hypothetical protein Ngar_c35540	No	0	2792911	2793135	+		Candidatus_Nitrososphaera_gargensis_Ga9_2_ui	NC_018719	chromosome										
408406142	quinoprotein glucose/ sorbosone dehydrogen	Yes	1	2793179	2794459	+		Candidatus_Nitrososphaera_gargensis_Ga9_2_ui	NC_018719	chromosome		arCOG02796	1	(-)	G		Glucose/sorbosone dehydrogenase	2E-27	COG2133	Glucose/sorbosone dehydrogenases
408406143	hypothetical protein Ngar_c35560	No	4	2794504	2795016	+		Candidatus_Nitrososphaera_gargensis_Ga9_2_ui	NC_018719	chromosome										
408406144	phosphate ABC transporter substrate-binding p	Yes	1	2795078	2796187	+		Candidatus_Nitrososphaera_gargensis_Ga9_2_ui	NC_018719	chromosome		arCOG00213	1	(-)	P	PstS	ABC-type phosphate transport system, periplasm	7E-13	COG0226	ABC-type phosphate transport system, per
408406145	hypothetical protein Ngar_c35580	No	0	2796194	2796484	-		Candidatus_Nitrososphaera_gargensis_Ga9_2_ui	NC_018719	chromosome										
408406146	hypothetical protein Ngar_c35590	No	0	2796560	2796781	-		Candidatus_Nitrososphaera_gargensis_Ga9_2_ui	NC_018719	chromosome										
408406147	hypothetical protein Ngar_c35600	No	0	2796829	2797398	+		Candidatus_Nitrososphaera_gargensis_Ga9_2_ui	NC_018719	chromosome										
408406148	hypothetical protein Ngar_c35610	No	0	2797879	2798082	+		Candidatus_Nitrososphaera_gargensis_Ga9_2_ui	NC_018719	chromosome										
408406149	NH(3)-dependent NAD(+) synthase	No	0	2798157	2798960	-		Candidatus_Nitrososphaera_gargensis_Ga9_2_ui	NC_018719	chromosome		arCOG00069	1	(-)	H	NadE	NH3-dependent NAD+ synthetase	3E-63	pfam02540	NAD synthase. NAD synthase (EC:6.3.5.1)
408406150	hypothetical protein Ngar_c35630	No	0	2799386	2799637	-		Candidatus_Nitrososphaera_gargensis_Ga9_2_ui	NC_018719	chromosome								0.00003	pfam14026	Protein of unknown function (DUF4242). T
===							5	Cenarchaeum_symbiosum_A_uid61411	NC_014820	chromosome										
118575224	hypothetical protein CENSYa_0012	Yes	1	9395	10531	+		Cenarchaeum_symbiosum_A_uid61411	NC_014820	chromosome								1E-41	pfam14247	Domain of unknown function (DUF4344). Th
118575225	hypothetical protein CENSYa_0013	No	0	10539	10727	+		Cenarchaeum_symbiosum_A_uid61411	NC_014820	chromosome										
118575226	hypothetical protein CENSYa_0014	No	0	10855	11502	+		Cenarchaeum_symbiosum_A_uid61411	NC_014820	chromosome		arCOG08728	1	(-)	S		Uncharacterized conserved protein	1E-18	pfam14247	Domain of unknown function (DUF4344). Th
118575227	hypothetical protein CENSYa_0015	No	0	11515	11823	+		Cenarchaeum_symbiosum_A_uid61411	NC_014820	chromosome										
118575228	hypothetical protein CENSYa_0016	No	1	11923	12090	+		Cenarchaeum_symbiosum_A_uid61411	NC_014820	chromosome										
===							6	Cenarchaeum_symbiosum_A_uid61411	NC_014820	chromosome										
118575266	ATPase of the PP-loop superfamily	No	0	42784	43593	+		Cenarchaeum_symbiosum_A_uid61411	NC_014820	chromosome		arCOG00042	4	(-)	J	MesJ	tRNA(Ile)-lysidine synthase MesJ	1E-29	COG0037	tRNA(Ile)-lysidine synthase MesJ
118575267	hypothetical protein CENSYa_0058	No	0	43651	44130	+		Cenarchaeum_symbiosum_A_uid61411	NC_014820	chromosome										
118575268	hypothetical protein CENSYa_0061	No	0	44727	45002	-		Cenarchaeum_symbiosum_A_uid61411	NC_014820	chromosome										
118575269	hypothetical protein CENSYa_0062	No	0	45013	45435	+		Cenarchaeum_symbiosum_A_uid61411	NC_014820	chromosome										
118575270	hypothetical membrane protein	No	1	45468	45692	+		Cenarchaeum_symbiosum_A_uid61411	NC_014820	chromosome										
118575271	hypothetical membrane protein	No	0	45734	46117	+		Cenarchaeum_symbiosum_A_uid61411	NC_014820	chromosome		arCOG05565	3	(-)	R		Roadblock/LC7 domain superfamily protein			
===							8	Cenarchaeum_symbiosum_A_uid61411	NC_014820	chromosome										
118575340	hypothetical protein CENSYa_0134	No	0	107260	107460	-		Cenarchaeum_symbiosum_A_uid61411	NC_014820	chromosome		arCOG04792	9	(-)	L		Transposase			
118575341	hypothetical protein CENSYa_0135	No	0	107643	108083	+		Cenarchaeum_symbiosum_A_uid61411	NC_014820	chromosome										
118575342	hypothetical protein CENSYa_0136	No	0	108136	110394	+		Cenarchaeum_symbiosum_A_uid61411	NC_014820	chromosome										
118575343	hypothetical protein CENSYa_0137	No	0	110465	110722	+		Cenarchaeum_symbiosum_A_uid61411	NC_014820	chromosome										
118575344	hypothetical protein CENSYa_0138	No	0	110771	110971	+		Cenarchaeum_symbiosum_A_uid61411	NC_014820	chromosome										
118575345	hypothetical protein CENSYa_0139	No	0	110968	111141	+		Cenarchaeum_symbiosum_A_uid61411	NC_014820	chromosome										
118575346	hypothetical protein CENSYa_0140	No	0	111138	111797	+		Cenarchaeum_symbiosum_A_uid61411	NC_014820	chromosome										
118575347	hypothetical protein CENSYa_0141	No	5	112016	114631	-		Cenarchaeum_symbiosum_A_uid61411	NC_014820	chromosome		arCOG05096	3	(-)	S		Uncharacterized conserved membrane protein			
===							6	Cenarchaeum_symbiosum_A_uid61411	NC_014820	chromosome										
118575354	bacterial-type DNA primase	No	0	123279	123863	+		Cenarchaeum_symbiosum_A_uid61411	NC_014820	chromosome		arCOG04281	1	(-)	L	DnaG	DNA primase (bacterial type)			
118575355	hypothetical protein CENSYa_0149	No	0	123925	124125	+		Cenarchaeum_symbiosum_A_uid61411	NC_014820	chromosome										
118575356	hypothetical protein CENSYa_0150	No	0	124198	124401	+		Cenarchaeum_symbiosum_A_uid61411	NC_014820	chromosome										
118575357	hypothetical protein CENSYa_0151	No	0	124434	124922	-		Cenarchaeum_symbiosum_A_uid61411	NC_014820	chromosome										
118575358	hypothetical protein CENSYa_0152	No	0	124943	125257	-		Cenarchaeum_symbiosum_A_uid61411	NC_014820	chromosome										
118575359	superfamily II DNA/RNA helicase, SNF2 family	No	0	125479	127086	+		Cenarchaeum_symbiosum_A_uid61411	NC_014820	chromosome		arCOG00871	1	(-)	K	HepA	Superfamily II DNA/RNA helicase, SNF2 family	7E-22	COG0553	Superfamily II DNA/RNA helicases, SNF2 f ; Chromosome segregation ATPases
===							5	Cenarchaeum_symbiosum_A_uid61411	NC_014820	chromosome										
118575362	hypothetical protein CENSYa_0156	No	0	131570	131785	-		Cenarchaeum_symbiosum_A_uid61411	NC_014820	chromosome										
118575363	hypothetical protein CENSYa_0157	No	0	132079	132345	-		Cenarchaeum_symbiosum_A_uid61411	NC_014820	chromosome										
118575364	hypothetical protein CENSYa_0158	No	0	132414	132836	+		Cenarchaeum_symbiosum_A_uid61411	NC_014820	chromosome										
118575365	hypothetical protein CENSYa_0159	Yes	0	133102	138144	+		Cenarchaeum_symbiosum_A_uid61411	NC_014820	chromosome		arCOG08647	2	(-)	S		Uncharacterized conserved protein			
118575366	hypothetical protein CENSYa_0160	No	2	138428	138742	+		Cenarchaeum_symbiosum_A_uid61411	NC_014820	chromosome										
===							7	Cenarchaeum_symbiosum_A_uid61411	NC_014820	chromosome										
118575374	GMP synthase, glutamine amidotransferase do	No	0	152821	153453	-		Cenarchaeum_symbiosum_A_uid61411	NC_014820	chromosome		arCOG00090	1	(-)	F	GuaA	GMP synthase - Glutamine amidotransferase don	1E-27	COG0518	GMP synthase - Glutamine amidotransferas
118575375	hypothetical protein CENSYa_0169	No	0	153540	154094	+		Cenarchaeum_symbiosum_A_uid61411	NC_014820	chromosome										
118575376	hypothetical protein CENSYa_0170	No	0	154275	155450	+		Cenarchaeum_symbiosum_A_uid61411	NC_014820	chromosome										
118575377	hypothetical protein CENSYa_0171	No	0	155540	155965	+		Cenarchaeum_symbiosum_A_uid61411	NC_014820	chromosome										
118575378	hypothetical protein CENSYa_0172	No	1	155920	156207	-		Cenarchaeum_symbiosum_A_uid61411	NC_014820	chromosome										
118575379	glutamate dehydrogenase/leucine dehydroger	No	0	156263	157543	-		Cenarchaeum_symbiosum_A_uid61411	NC_014820	chromosome		arCOG01352	1	(-)	E	GdhA	Glutamate dehydrogenase/leucine dehydrogena	3E-126	COG0334	Glutamate dehydrogenase/leucine dehydrog
118575380	hypothetical protein CENSYa_0174	No	0	157564	157812	-		Cenarchaeum_symbiosum_A_uid61411	NC_014820	chromosome										
===							6	Cenarchaeum_symbiosum_A_uid61411	NC_014820	chromosome										
118575420	thiamine biosynthesis protein	No	0	195975	197330	-		Cenarchaeum_symbiosum_A_uid61411	NC_014820	chromosome		arCOG02741	4	(-)	H	ThiC	Thiamine biosynthesis protein ThiC	1E-151	pfam01964	ThiC family. ThiC is found within the th
118575421	hypothetical protein CENSYa_0217	No	0	197332	197676	-		Cenarchaeum_symbiosum_A_uid61411	NC_014820	chromosome										
118575422	hypothetical protein CENSYa_0218	No	0	197779	198351	+		Cenarchaeum_symbiosum_A_uid61411	NC_014820	chromosome										
118575423	hypothetical protein CENSYa_0219	No	0	198437	198685	-		Cenarchaeum_symbiosum_A_uid61411	NC_014820	chromosome										
118575424	hypothetical protein CENSYa_0220	No	0	198939	199538	+		Cenarchaeum_symbiosum_A_uid61411	NC_014820	chromosome										
118575425	hydroxymethylpyrimidine phosphate kinase	No	0	199804	201120	+		Cenarchaeum_symbiosum_A_uid61411	NC_014820	chromosome		arCOG00020	2	(-)	H	ThiD	Hydroxymethylpyrimidine/phosphomethylpyrimid	3E-50	pfam08543	Phosphomethylpyrimidine kinase. This enz ; Putative aldolase. Members of this famil
===							7	Cenarchaeum_symbiosum_A_uid61411	NC_014820	chromosome										
118575427	protein-disulfide isomerase	Yes	1	201595	202233	-		Cenarchaeum_symbiosum_A_uid61411	NC_014820	chromosome		arCOG02868	2	(-)	O	DsbG	Protein-disulfide isomerase	6E-26	pfam13462	Thioredoxin.
118575428	hypothetical protein CENSYa_0224	No	1	202420	203106	+		Cenarchaeum_symbiosum_A_uid61411	NC_014820	chromosome										
118575429	hypothetical protein CENSYa_0225	No	0	203225	204172	+		Cenarchaeum_symbiosum_A_uid61411	NC_014820	chromosome										
118575430	hypothetical protein CENSYa_0226	No	0	204290	204472	+		Cenarchaeum_symbiosum_A_uid61411	NC_014820	chromosome										
118575431	hypothetical protein CENSYa_0227	No	0	204508	205557	+		Cenarchaeum_symbiosum_A_uid61411	NC_014820	chromosome										
118575432	hypothetical protein CENSYa_0228	No	0	205590	205976	+		Cenarchaeum_symbiosum_A_uid61411	NC_014820	chromosome		arCOG08654	1	(-)	S		Uncharacterized conserved protein			
118575433	hypothetical protein CENSYa_0229	No	0	205985	206575	-		Cenarchaeum_symbiosum_A_uid61411	NC_014820	chromosome										
===							8	Cenarchaeum_symbiosum_A_uid61411	NC_014820	chromosome										
118575445	ATP-utilizing enzyme of ATP-grasp superfamily	No	0	215698	216723	+		Cenarchaeum_symbiosum_A_uid61411	NC_014820	chromosome		arCOG04346	1	(-)	F		5-formaminoimidazole-4-carboxamide-1-beta-D-ri	4E-114	COG1759	5-formaminoimidazole-4-carboxamide-1-bet
118575446	hypothetical protein CENSYa_0243	No	0	216769	217278	+		Cenarchaeum_symbiosum_A_uid61411	NC_014820	chromosome										
118575447	hypothetical protein CENSYa_0244	No	0	217358	217678	-		Cenarchaeum_symbiosum_A_uid61411	NC_014820	chromosome										
118575448	hypothetical protein CENSYa_0245	No	0	217727	218170	+		Cenarchaeum_symbiosum_A_uid61411	NC_014820	chromosome										
118575449	hypothetical protein CENSYa_0246	No	0	218413	218742	+		Cenarchaeum_symbiosum_A_uid61411	NC_014820	chromosome										
118575450	hypothetical protein CENSYa_0247	No	0	218903	219304	+		Cenarchaeum_symbiosum_A_uid61411	NC_014820	chromosome										
118575451	L-asparaginase	No	0	219643	220482	+		Cenarchaeum_symbiosum_A_uid61411	NC_014820	chromosome		arCOG04779	1	(-)	E		Asparaginase	2E-51	COG1446	Asparaginase
118575452	hypothetical protein CENSYa_0249	No	0	220479	220763	+		Cenarchaeum_symbiosum_A_uid61411	NC_014820	chromosome										
===							5	Cenarchaeum_symbiosum_A_uid61411	NC_014820	chromosome										
118575478	hypothetical protein CENSYa_0277	No	2	241100	241705	-		Cenarchaeum_symbiosum_A_uid61411	NC_014820	chromosome										
118575479	hypothetical protein CENSYa_0278	No	0	241761	241916	-		Cenarchaeum_symbiosum_A_uid61411	NC_014820	chromosome		arCOG08661	1	(-)	S		Uncharacterized conserved protein			
118575480	hypothetical protein CENSYa_0279	No	0	242033	242293	-		Cenarchaeum_symbiosum_A_uid61411	NC_014820	chromosome										
118575481	phage related minor tail protein	Yes	1	242395	244872	-		Cenarchaeum_symbiosum_A_uid61411	NC_014820	chromosome										
118575482	hypothetical protein CENSYa_0281	No	0	245178	245420	-		Cenarchaeum_symbiosum_A_uid61411	NC_014820	chromosome										
===							5	Cenarchaeum_symbiosum_A_uid61411	NC_014820	chromosome										
118575567	hypothetical protein CENSYa_0370	Yes	1	314843	315478	-		Cenarchaeum_symbiosum_A_uid61411	NC_014820	chromosome		arCOG08821	18		S		Uncharacterized conserved protein (expansion in Thaumarchaeota)			
118575568	hypothetical protein CENSYa_0371	No	0	315657	316961	+		Cenarchaeum_symbiosum_A_uid61411	NC_014820	chromosome		arCOG03517	1	(-)	T		Formylglycine-generating sulfatase enzyme	7E-29	COG1262	Uncharacterized conserved protein
118575569	hypothetical protein CENSYa_0372	No	0	316933	317973	-		Cenarchaeum_symbiosum_A_uid61411	NC_014820	chromosome								4E-67	COG4301	Uncharacterized conserved protein
118575570	hypothetical protein CENSYa_0373	No	0	318085	318300	-		Cenarchaeum_symbiosum_A_uid61411	NC_014820	chromosome										
118575571	hypothetical protein CENSYa_0374	No	0	318299	318613	+		Cenarchaeum_symbiosum_A_uid61411	NC_014820	chromosome										
===							7	Cenarchaeum_symbiosum_A_uid61411	NC_014820	chromosome										
118575591	ammonia monooxygenase subunit B	No	0	340470	341045	-		Cenarchaeum_symbiosum_A_uid61411	NC_014820	chromosome		arCOG08675	1	(-)	C		Methane monooxygenase/Ammonia monooxygenase, subunit B			
118575592	hypothetical protein CENSYa_0395	No	0	341154	341408	-		Cenarchaeum_symbiosum_A_uid61411	NC_014820	chromosome										

118575593	hypothetical protein CENSYa_0396	No	0	341513	341983	+		Cenarchaeum_symbiosum_A_uid61411	NC_014820	chromosome									
118575594	hypothetical protein CENSYa_0397	No	0	342054	342290	+		Cenarchaeum_symbiosum_A_uid61411	NC_014820	chromosome									
118575595	hypothetical protein CENSYa_0398	No	0	342280	343080	+		Cenarchaeum_symbiosum_A_uid61411	NC_014820	chromosome									
118575596	ammonia monooxygenase subunit C	No	4	343290	343859	+		Cenarchaeum_symbiosum_A_uid61411	NC_014820	chromosome		arCOG08699	1 (-)	C		Methane monooxygenase/Ammonia monooxyge	3E-11	pfam04896	Ammonia monooxygenase/methane monooxygen
118575597	hypothetical protein CENSYa_0400	No	0	343919	344320	+		Cenarchaeum_symbiosum_A_uid61411	NC_014820	chromosome									
===							10	Cenarchaeum_symbiosum_A_uid61411	NC_014820	chromosome									
118575728	hypothetical protein CENSYa_0534	No	0	466229	466567	+		Cenarchaeum_symbiosum_A_uid61411	NC_014820	chromosome									
118575729	hypothetical protein CENSYa_0535	No	0	466724	466900	-		Cenarchaeum_symbiosum_A_uid61411	NC_014820	chromosome									
118575730	hypothetical protein CENSYa_0536	Yes	1	466936	467574	+		Cenarchaeum_symbiosum_A_uid61411	NC_014820	chromosome									
118575731	serine protease inhibitor	Yes	1	467571	468815	+		Cenarchaeum_symbiosum_A_uid61411	NC_014820	chromosome		arCOG04933	4 (-)	O		Serine protease inhibitor	1E-75	pfam00079	Serpin (serine protease inhibitor). Stru
118575732	hypothetical protein CENSYa_0538	No	0	468876	469115	+		Cenarchaeum_symbiosum_A_uid61411	NC_014820	chromosome									
118575733	hypothetical protein CENSYa_0539	No	0	469129	469431	+		Cenarchaeum_symbiosum_A_uid61411	NC_014820	chromosome									
118575734	hypothetical protein CENSYa_0540	No	0	469752	470066	+		Cenarchaeum_symbiosum_A_uid61411	NC_014820	chromosome									
118575735	hypothetical protein CENSYa_0541	No	0	470361	470834	-		Cenarchaeum_symbiosum_A_uid61411	NC_014820	chromosome									
118575736	hypothetical protein CENSYa_0542	No	0	470896	471060	-		Cenarchaeum_symbiosum_A_uid61411	NC_014820	chromosome									
118575737	excinuclease nuclease subunit	No	0	471136	472731	-		Cenarchaeum_symbiosum_A_uid61411	NC_014820	chromosome		arCOG04753	1 (-)	L	UvrC	Excinuclease ABC subunit C	8E-115	COG0322	Nuclease subunit of the excinuclease com
===							6	Cenarchaeum_symbiosum_A_uid61411	NC_014820	chromosome									
118575786	hypothetical protein CENSYa_0592	Yes	1	542525	543196	+		Cenarchaeum_symbiosum_A_uid61411	NC_014820	chromosome		arCOG08821	18	S		Uncharacterized conserved protein (expansion in Thaumarchaeota)			
118575787	hypothetical protein CENSYa_0593	Yes	1	543293	543952	+		Cenarchaeum_symbiosum_A_uid61411	NC_014820	chromosome		arCOG08821	18	S		Uncharacterized conserved protein (expansion in Thaumarchaeota)			
118575788	hypothetical protein CENSYa_0594	No	0	544021	544236	-		Cenarchaeum_symbiosum_A_uid61411	NC_014820	chromosome									
118575789	transcriptional activator, adenine-specific DNA	No	0	544554	545204	+		Cenarchaeum_symbiosum_A_uid61411	NC_014820	chromosome		arCOG08695	1 (-)	K	IME4	Transcriptional activator, adenine-specific DNA m	1E-18	pfam05063	MT-A70. MT-A70 is the S-adenosylmethioni
118575790	MunI restriction endonuclease	No	0	545196	545744	-		Cenarchaeum_symbiosum_A_uid61411	NC_014820	chromosome							2E-50	pfam11407	Type II restriction enzyme MunI. Type II
118575791	hypothetical protein CENSYa_0597	No	0	545878	546261	+		Cenarchaeum_symbiosum_A_uid61411	NC_014820	chromosome									
===							8	Cenarchaeum_symbiosum_A_uid61411	NC_014820	chromosome									
118575797	hypothetical protein CENSYa_0603	No	4	549997	550815	+		Cenarchaeum_symbiosum_A_uid61411	NC_014820	chromosome									
118575798	adenine specific DNA methylase	No	0	550812	552569	-		Cenarchaeum_symbiosum_A_uid61411	NC_014820	chromosome		arCOG00890	3 (-)	L		DNA modification methylase	0.000000009	COG0863	DNA modification methylase
118575799	hypothetical protein CENSYa_0605	No	0	552689	552883	-		Cenarchaeum_symbiosum_A_uid61411	NC_014820	chromosome		arCOG04485	4	S		Uncharacterized conserved protein			
118575800	hypothetical protein CENSYa_0606	No	0	552922	553812	-		Cenarchaeum_symbiosum_A_uid61411	NC_014820	chromosome		arCOG04485	4	S		Uncharacterized conserved protein			
118575801	hypothetical protein CENSYa_0607	No	0	553931	554269	+		Cenarchaeum_symbiosum_A_uid61411	NC_014820	chromosome									
118575802	flavin-nucleotide-binding protein	No	0	554585	555085	-		Cenarchaeum_symbiosum_A_uid61411	NC_014820	chromosome		arCOG00516	6 (-)	R		Predicted flavin-nucleotide-binding protein	0.00002	pfam01243	Pyridoxamine 5'-phosphate oxidase.
118575803	hypothetical protein CENSYa_0609	No	0	555350	555580	-		Cenarchaeum_symbiosum_A_uid61411	NC_014820	chromosome									
118575804	hypothetical protein CENSYa_0610	No	0	555625	556125	+		Cenarchaeum_symbiosum_A_uid61411	NC_014820	chromosome									
===							6	Cenarchaeum_symbiosum_A_uid61411	NC_014820	chromosome									
118575811	N-6-adenine-methyltransferase	No	0	563637	564236	-		Cenarchaeum_symbiosum_A_uid61411	NC_014820	chromosome									
118575812	hypothetical protein CENSYa_0618	No	0	564426	564725	+		Cenarchaeum_symbiosum_A_uid61411	NC_014820	chromosome							0.00001	pfam05887	Procyclic acidic repetitive protein (PAR
118575813	hypothetical protein CENSYa_0619	No	0	565055	565405	-		Cenarchaeum_symbiosum_A_uid61411	NC_014820	chromosome									
118575814	hypothetical protein CENSYa_0620	No	0	565622	566005	-		Cenarchaeum_symbiosum_A_uid61411	NC_014820	chromosome		arCOG04061	1 (-)	K	EGD2	Transcription factor homologous to NACalpha-BTI	0.00002	COG1308	Transcription factor homologous to NACal
118575815	hypothetical protein CENSYa_0621	No	0	566008	566853	-		Cenarchaeum_symbiosum_A_uid61411	NC_014820	chromosome									
118575816	hypothetical protein CENSYa_0622	No	0	566850	567062	-		Cenarchaeum_symbiosum_A_uid61411	NC_014820	chromosome									
===							10	Cenarchaeum_symbiosum_A_uid61411	NC_014820	chromosome									
118575824	hypothetical protein CENSYa_0630	No	0	578670	578960	-		Cenarchaeum_symbiosum_A_uid61411	NC_014820	chromosome		arCOG04102	1 (-)	C	NtpG	Archaeal/vacuolar-type H+-ATPase subunit F	0.000000002	COG1436	Archaeal/vacuolar-type H+-ATPase subunit
118575825	hypothetical protein CENSYa_0631	No	0	579089	579982	-		Cenarchaeum_symbiosum_A_uid61411	NC_014820	chromosome									
118575826	hypothetical protein CENSYa_0632	No	0	580119	580415	-		Cenarchaeum_symbiosum_A_uid61411	NC_014820	chromosome									
118575827	hypothetical protein CENSYa_0633	No	0	580430	581989	-		Cenarchaeum_symbiosum_A_uid61411	NC_014820	chromosome									
118575828	hypothetical protein CENSYa_0634	No	0	581998	582336	-		Cenarchaeum_symbiosum_A_uid61411	NC_014820	chromosome									
118575829	hypothetical protein CENSYa_0635	No	0	582443	584491	-		Cenarchaeum_symbiosum_A_uid61411	NC_014820	chromosome									
118575830	hypothetical protein CENSYa_0636	Yes	1	584600	586270	-		Cenarchaeum_symbiosum_A_uid61411	NC_014820	chromosome		arCOG02507	9	S		beta-propeller repeat protein			
118575831	hypothetical protein CENSYa_0637	Yes	1	586359	591230	-		Cenarchaeum_symbiosum_A_uid61411	NC_014820	chromosome		arCOG02507	9	S		beta-propeller repeat protein	0.00000006	COG3794	Plastocyanin
118575832	hypothetical protein CENSYa_0638	No	0	591337	592347	+		Cenarchaeum_symbiosum_A_uid61411	NC_014820	chromosome									
118575833	DNA mismatch repair enzyme	No	0	592378	594096	-		Cenarchaeum_symbiosum_A_uid61411	NC_014820	chromosome		arCOG07860	1 (-)	R		Histidine kinase-like ATPase domain containing p	2E-13	pfam13589	Histidine kinase-, DNA gyrase B-, and HS
===							13	Cenarchaeum_symbiosum_A_uid61411	NC_014820	chromosome									
118575847	hypothetical protein CENSYa_0653	Yes	0	618896	619126	+		Cenarchaeum_symbiosum_A_uid61411	NC_014820	chromosome									
118575848	hypothetical protein CENSYa_0654	Yes	1	619255	623712	-		Cenarchaeum_symbiosum_A_uid61411	NC_014820	chromosome		arCOG02507	9	S		beta-propeller repeat protein	0.0000004	COG3794	Plastocyanin
118575849	hypothetical protein CENSYa_0655	No	0	623829	624035	+		Cenarchaeum_symbiosum_A_uid61411	NC_014820	chromosome									
118575850	3'-phosphoadenosine 5'-phosphosulfate sulfot	No	0	624061	624900	-		Cenarchaeum_symbiosum_A_uid61411	NC_014820	chromosome		arCOG00074	1 (-)	E	CysH	3'-phosphoadenosine 5'-phosphosulfate sulfotran	0.00001	COG0175	3'-phosphoadenosine 5'-phosphosulfate su
118575851	hypothetical protein CENSYa_0657	No	0	624965	625906	-		Cenarchaeum_symbiosum_A_uid61411	NC_014820	chromosome							2E-60	pfam13182	Protein of unknown function (DUF4007). T
118575852	cysteine sulfinate desulfinase/cysteine desulfu	No	0	625906	626985	-		Cenarchaeum_symbiosum_A_uid61411	NC_014820	chromosome		arCOG00066	2 (-)	E	NifS	Cysteine sulfinate desulfinase/cysteine desulfura	8E-95	COG1104	Cysteine sulfinate desulfinase/cysteine
118575853	hypothetical protein CENSYa_0659	No	0	627033	627236	-		Cenarchaeum_symbiosum_A_uid61411	NC_014820	chromosome									
118575854	hypothetical protein CENSYa_0660	No	0	627281	628387	+		Cenarchaeum_symbiosum_A_uid61411	NC_014820	chromosome	clust_87								
118575855	hypothetical protein CENSYa_0661	No	0	628424	628822	+		Cenarchaeum_symbiosum_A_uid61411	NC_014820	chromosome									
118575856	transposase	No	0	628943	630601	+		Cenarchaeum_symbiosum_A_uid61411	NC_014820	chromosome	arCOG04792,a	arCOG04792	9 (-)	L		Transposase	0.00001	pfam03050	Transposase IS66 family. Transposase pro
118575857	hypothetical protein CENSYa_0663	No	0	630668	631774	+		Cenarchaeum_symbiosum_A_uid61411	NC_014820	chromosome	clust_87								
118575858	hypothetical protein CENSYa_0664	No	7	631842	633821	-		Cenarchaeum_symbiosum_A_uid61411	NC_014820	chromosome									
118575859	hypothetical protein CENSYa_0665	No	0	633881	634186	-		Cenarchaeum_symbiosum_A_uid61411	NC_014820	chromosome									
===							19	Cenarchaeum_symbiosum_A_uid61411	NC_014820	chromosome									
118575865	hypothetical protein CENSYa_0671	No	0	639924	640541	-		Cenarchaeum_symbiosum_A_uid61411	NC_014820	chromosome		arCOG00787	3 (-)	L		UvrD/Rep family helicase fused to exonuclease family domain			
118575866	hypothetical protein CENSYa_0672	No	0	640555	641343	+		Cenarchaeum_symbiosum_A_uid61411	NC_014820	chromosome									
118575867	hypothetical protein CENSYa_0673	No	4	641312	641713	-		Cenarchaeum_symbiosum_A_uid61411	NC_014820	chromosome									
118575868	hypothetical protein CENSYa_0674	No	2	641707	642006	-		Cenarchaeum_symbiosum_A_uid61411	NC_014820	chromosome									
118575869	hypothetical protein CENSYa_0675	No	0	642126	642605	+		Cenarchaeum_symbiosum_A_uid61411	NC_014820	chromosome									
118575870	hypothetical protein CENSYa_0676	No	1	642630	643250	+		Cenarchaeum_symbiosum_A_uid61411	NC_014820	chromosome									
118575871	hypothetical protein CENSYa_0677	No	0	643338	643733	+		Cenarchaeum_symbiosum_A_uid61411	NC_014820	chromosome									
118575872	hypothetical protein CENSYa_0678	No	0	643751	644053	+		Cenarchaeum_symbiosum_A_uid61411	NC_014820	chromosome									
118575873	hypothetical protein CENSYa_0679	No	0	644138	644437	+		Cenarchaeum_symbiosum_A_uid61411	NC_014820	chromosome	clust_351								
118575874	hypothetical protein CENSYa_0680	No	0	644479	644751	+		Cenarchaeum_symbiosum_A_uid61411	NC_014820	chromosome									
118575875	hypothetical protein CENSYa_0681	No	0	644839	645138	+		Cenarchaeum_symbiosum_A_uid61411	NC_014820	chromosome	clust_351								
118575876	hypothetical protein CENSYa_0682	No	3	645322	645801	-		Cenarchaeum_symbiosum_A_uid61411	NC_014820	chromosome									
118575877	DNA modification methylase	No	0	645833	647356	-		Cenarchaeum_symbiosum_A_uid61411	NC_014820	chromosome									
118575878	hypothetical protein CENSYa_0684	No	0	647405	647755	+		Cenarchaeum_symbiosum_A_uid61411	NC_014820	chromosome									
118575879	hypothetical protein CENSYa_0685	No	0	647769	648032	+		Cenarchaeum_symbiosum_A_uid61411	NC_014820	chromosome									
118575880	hypothetical protein CENSYa_0686	No	4	648137	648607	+		Cenarchaeum_symbiosum_A_uid61411	NC_014820	chromosome									
118575881	hypothetical protein CENSYa_0687	No	2	648715	649356	+		Cenarchaeum_symbiosum_A_uid61411	NC_014820	chromosome									
118575882	micrococcal nuclease-like protein	Yes	0	649353	651266	+		Cenarchaeum_symbiosum_A_uid61411	NC_014820	chromosome	arCOG03192,a	arCOG03192	6 (-)	L		Micrococcal nuclease (thermonuclease)	0.000001	COG1525	Micrococcal nuclease (thermonuclease) ho
118575883	hypothetical protein CENSYa_0689	No	0	651263	652105	+		Cenarchaeum_symbiosum_A_uid61411	NC_014820	chromosome									
===							70	Cenarchaeum_symbiosum_A_uid61411	NC_014820	chromosome									
118575890	hypothetical protein CENSYa_0696	No	1	663005	663709	+		Cenarchaeum_symbiosum_A_uid61411	NC_014820	chromosome									
118575891	hypothetical protein CENSYa_0697	No	0	663975	664328	-		Cenarchaeum_symbiosum_A_uid61411	NC_014820	chromosome		arCOG03889	1 (-)	S		Uncharacterized conserved protein			
118575892	hypothetical protein CENSYa_0698	No	1	664401	664877	+		Cenarchaeum_symbiosum_A_uid61411	NC_014820	chromosome									
118575893	hypothetical protein CENSYa_0699	Yes	1	664874	667306	+		Cenarchaeum_symbiosum_A_uid61411	NC_014820	chromosome									
118575894	hypothetical protein CENSYa_0700	No	0	667266	668096	+		Cenarchaeum_symbiosum_A_uid61411	NC_014820	chromosome									
118575895	hypothetical protein CENSYa_0701	No	0	668224	668982	+		Cenarchaeum_symbiosum_A_uid61411	NC_014820	chromosome									
118575896	hypothetical protein CENSYa_0702	No	0	668984	669118	+		Cenarchaeum_symbiosum_A_uid61411	NC_014820	chromosome									
118575897	hypothetical protein CENSYa_0703	No	2	669154	669420	+		Cenarchaeum_symbiosum_A_uid61411	NC_014820	chromosome									
118575898	hypothetical protein CENSYa_0704	No	4	669823	670566	+		Cenarchaeum_symbiosum_A_uid61411	NC_014820	chromosome									
118575899	hypothetical protein CENSYa_0705	No	6	670572	671795	-		Cenarchaeum_symbiosum_A_uid61411	NC_014820	chromosome									
118575900	hypothetical protein CENSYa_0706	No	0	671827	672099	-		Cenarchaeum_symbiosum_A_uid61411	NC_014820	chromosome									
118575901	hypothetical protein CENSYa_0707	No	0	672154	676266	+		Cenarchaeum_symbiosum_A_uid61411	NC_014820	chromosome									
118575902	hypothetical protein CENSYa_0708	No	0	676331	683608	+		Cenarchaeum_symbiosum_A_uid61411	NC_014820	chromosome		arCOG02507	9	S		beta-propeller repeat protein	0.00000002	COG3391	Uncharacterized conserved protein
118575903	hypothetical protein CENSYa_0709	No	0	683605	685434	+		Cenarchaeum_symbiosum_A_uid61411	NC_014820	chromosome		arCOG02507	9	S		beta-propeller repeat protein	1E-12	COG3391	Uncharacterized conserved protein
118575904	hypothetical protein CENSYa_0710	No	0	685510	685905	-		Cenarchaeum_symbiosum_A_uid61411	NC_014820	chromosome									
118575905	hypothetical protein CENSYa_0711	No	0	686124	686438	-		Cenarchaeum_symbiosum_A_uid61411	NC_014820	chromosome									
118575906	hypothetical protein CENSYa_0712	No	0	686626	687312	-		Cenarchaeum_symbiosum_A_uid61411	NC_014820	chromosome									
118575907	hypothetical protein CENSYa_0713	No	0	687311	687529	+		Cenarchaeum_symbiosum_A_uid61411	NC_014820	chromosome									
118575908	hypothetical protein CENSYa_0714	No	0	687536	688489	+		Cenarchaeum_symbiosum_A_uid61411	NC_014820	chromosome									
118575909	hypothetical protein CENSYa_0715	No	0	688646	689044	+		Cenarchaeum_symbiosum_A_uid61411	NC_014820	chromosome									
118575910	hypothetical protein CENSYa_0716	No	2	689041	689976	+		Cenarchaeum_symbiosum_A_uid61411	NC_014820	chromosome		arCOG03724	3 (-)	S		KID-like repeats containing protein, paREP15 family			
118575911	hypothetical protein CENSYa_0717	No	1	689973	690398	+		Cenarchaeum_symbiosum_A_uid61411	NC_014820	chromosome									
118575912	hypothetical protein CENSYa_0718	No	0	690518	690847	+		Cenarchaeum_symbiosum_A_uid61411	NC_014820	chromosome									
118575913	hypothetical protein CENSYa_0719	No	0	690849	691406	+		Cenarchaeum_symbiosum_A_uid61411	NC_014820	chromosome									
118575914	hypothetical protein CENSYa_0720	No	0	691629	691964	+		Cenarchaeum_symbiosum_A_uid61411	NC_014820	chromosome	clust_313								
118575915	hypothetical protein CENSYa_0721	No	0	691967	692083	+		Cenarchaeum_symbiosum_A_uid61411	NC_014820	chromosome	clust_511								
118575916	hypothetical protein CENSYa_0722	No	2	692135	693109	+		Cenarchaeum_symbiosum_A_uid61411	NC_014820	chromosome		arCOG01159	1 (-)	S		Uncharacterized archaeal coiled-coil protein	0.00005	pfam04156	IncA protein. Chlamydia trachomatis is a
118575917	hypothetical protein CENSYa_0723	No	0	693189	694070	-		Cenarchaeum_symbiosum_A_uid61411	NC_014820	chromosome									
118575918	hypothetical protein CENSYa_0724	No	0	694121	696304	+		Cenarchaeum_symbiosum_A_uid61411	NC_014820	chromosome									
118575919	hypothetical protein CENSYa_0725	No	0	696330	698342	+		Cenarchaeum_symbiosum_A_uid61411	NC_014820	chromosome									
118575920	hypothetical protein CENSYa_0726	No	0	698349	698837	+		Cenarchaeum_symbiosum_A_uid61411	NC_014820	chromosome									
118575921	hypothetical protein CENSYa_0727	No	2	698952	699938	+		Cenarchaeum_symbiosum_A_uid61411	NC_014820	chromosome	clust_13								
118575922	hypothetical protein CENSYa_0728	No	0	699983	700252	+		Cenarchaeum_symbiosum_A_uid61411	NC_014820	chromosome	clust_3								
118575923	hypothetical protein CENSYa_0729	No	0	700297	700599	+		Cenarchaeum_symbiosum_A_uid61411	NC_014820	chromosome	clust_3								
118575924	hypothetical protein CENSYa_0730	No	0	700644	700922	+		Cenarchaeum_symbiosum_A_uid61411	NC_014820	chromosome	clust_3								
118575925	hypothetical protein CENSYa_0731	No	2	700973	701707	+		Cenarchaeum_symbiosum_A_uid61411	NC_014820	chromosome	clust_13								
118575926	hypothetical protein CENSYa_0732	No	0	701707	702048	+		Cenarchaeum_symbiosum_A_uid61411	NC_014820	chromosome									
118575927	hypothetical protein CENSYa_0733	No	0	702045	704153	+		Cenarchaeum_symbiosum_A_uid61411	NC_014820	chromosome									
118575928	hypothetical protein CENSYa_0734	No	0	704150	704869	+		Cenarchaeum_symbiosum_A_uid61411	NC_014820	chromosome									
118575929	hypothetical protein CENSYa_0735	No	0	704921	705256	+		Cenarchaeum_symbiosum_A_uid61411	NC_014820	chromosome	clust_313								
118575930	hypothetical protein CENSYa_0736	No	0	705259	705375	+		Cenarchaeum_symbiosum_A_uid61411	NC_014820	chromosome	clust_511								
118575931	hypothetical protein CENSYa_0737	No	2	705427	706338	+		Cenarchaeum_symbiosum_A_uid61411	NC_014820	chromosome	clust_13								
118575932	hypothetical protein CENSYa_0738	No	0	706383	706682	+		Cenarchaeum_symbiosum_A_uid61411	NC_014820	chromosome	clust_3								
118575933	hypothetical protein CENSYa_0739	No	0	706727	707080	+		Cenarchaeum_symbiosum_A_uid61411	NC_014820	chromosome	clust_3								
118575934	hypothetical protein CENSYa_0740	Yes	0	707080	707382	+		Cenarchaeum_symbiosum_A_uid61411	NC_014820	chromosome	clust_3								
118575935	hypothetical protein CENSYa_0741	No	0	707427	707756	+		Cenarchaeum_symbiosum_A_uid61411	NC_014820	chromosome	clust_3								
118575936	hypothetical protein CENSYa_0742	No	0	707809	708084	-		Cenarchaeum_symbiosum_A_uid61411	NC_014820	chromosome									
118575937	hypothetical protein CENSYa_0743	No	0	708083	709060	+		Cenarchaeum_symbiosum_A_uid61411	NC_014820	chromosome		arCOG07546	3 (-)	E		Zincin superfamily protease			
118575938	hypothetical protein CENSYa_0744	No	2	709057	709671	+		Cenarchaeum_symbiosum_A_uid61411	NC_014820	chromosome	clust_13								
118575939	hypothetical protein CENSYa_0745	No	0	709766	710083	+		Cenarchaeum_symbiosum_A_uid61411	NC_014820	chromosome	clust_3								
118575940	hypothetical protein CENSYa_0746	No	2	710132	710752	+		Cenarchaeum_symbiosum_A_uid61411	NC_014820	chromosome									
118575941	hypothetical protein CENSYa_0747	No	0	710762	710971	+		Cenarchaeum_symbiosum_A_uid61411	NC_014820	chromosome									
118575942	hypothetical protein CENSYa_0748	No	0	710973	711320	+		Cenarchaeum_symbiosum_A_uid61411	NC_014820	chromosome									
118575943	hypothetical protein CENSYa_0749	No	0	711320	711577	+		Cenarchaeum_symbiosum_A_uid61411	NC_014820	chromosome									
118575944	hypothetical protein CENSYa_0750	No	0	711818	712090	+		Cenarchaeum_symbiosum_A_uid61411	NC_014820	chromosome									
118575945	hypothetical protein CENSYa_0751	No	0	712179	712397	-		Cenarchaeum_symbiosum_A_uid61411	NC_014820	chromosome									
118575946	hypothetical protein CENSYa_0752	No	3	712407	712754	+		Cenarchaeum_symbiosum_A_uid61411	NC_014820	chromosome									
118575947	hypothetical protein CENSYa_0753	No	1	712782	712997	+		Cenarchaeum_symbiosum_A_uid61411	NC_014820	chromosome									
118575948	hypothetical protein CENSYa_0754	No	0	713061	713339	-		Cenarchaeum_symbiosum_A_uid61411	NC_014820	chromosome									
118575949	hypothetical protein CENSYa_0755	No	0	713395	714444	-		Cenarchaeum_symbiosum_A_uid61411	NC_014820	chromosome									
118575950	hypothetical protein CENSYa_0756	No	0	714529	715134	-		Cenarchaeum_symbiosum_A_uid61411	NC_014820	chromosome		arCOG08662	3 (-)	S		Uncharacterized conserved metal-binding protein			
118575951	hypothetical protein CENSYa_0757	No	0	715148	715567	-		Cenarchaeum_symbiosum_A_uid61411	NC_014820	chromosome									
118575952	hypothetical protein CENSYa_0758	No	0	716153	716491	-		Cenarchaeum_symbiosum_A_uid61411	NC_014820	chromosome									
118575953	hypothetical protein CENSYa_0759	No	0	716536	716796	+		Cenarchaeum_symbiosum_A_uid61411	NC_014820	chromosome									
118575954	hypothetical protein CENSYa_0760	No	0	716793	717062	+		Cenarchaeum_symbiosum_A_uid61411	NC_014820	chromosome									
118575955	hypothetical protein CENSYa_0761	No	0	717117	717404	+		Cenarchaeum_symbiosum_A_uid61411	NC_014820	chromosome									
118575956	hypothetical protein CENSYa_0762	Yes	1	717440	718072	+		Cenarchaeum_symbiosum_A_uid61411	NC_014820	chromosome		arCOG08819	6	S		Uncharacterized conserved protein (expansion in Thaumarchaeota)			
118575957	hypothetical protein CENSYa_0763	No	0	718069	718572	-		Cenarchaeum_symbiosum_A_uid61411	NC_014820	chromosome									
118575958	hypothetical protein CENSYa_0764	No	0	718571	718801	+		Cenarchaeum_symbiosum_A_uid61411	NC_014820	chromosome									
118575959	hypothetical protein CENSYa_0765	No	0	718810	719109	+		Cenarchaeum_symbiosum_A_uid61411	NC_014820	chromosome		arCOG08700	1 (-)	S		Uncharacterized conserved protein			
===							8	Cenarchaeum_symbiosum_A_uid61411	NC_014820	chromosome									
118575966	hypothetical protein CENSYa_0772	No	0	725022	725306	-		Cenarchaeum_symbiosum_A_uid61411	NC_014820	chromosome									
118575967	hypothetical protein CENSYa_0773	No	0	725321	725944	-		Cenarchaeum_symbiosum_A_uid61411	NC_014820	chromosome		arCOG03706	3 (-)	V		PIN domain containing protein			
118575968	hypothetical protein CENSYa_0774	No	0	726023	726655	-		Cenarchaeum_symbiosum_A_uid61411	NC_014820	chromosome									
118575969	hypothetical protein CENSYa_0775	No	0	726774	728144	-		Cenarchaeum_symbiosum_A_uid61411	NC_014820	chromosome									
118575970	hypothetical protein CENSYa_0776	No	0	728297	729067	-		Cenarchaeum_symbiosum_A_uid61411	NC_014820	chromosome	clust_129								
118575971	hypothetical protein CENSYa_0777	No	0	729273	730034	-		Cenarchaeum_symbiosum_A_uid61411	NC_014820	chromosome	clust_129								
118575972	hypothetical protein CENSYa_0778	No	0	730449	730718	-		Cenarchaeum_symbiosum_A_uid61411	NC_014820	chromosome									
118575973	translation initiation factor 1	No	0	731412	731969	+		Cenarchaeum_symbiosum_A_uid61411	NC_014820	chromosome		arCOG01179	2 (-)	J	InfA	Translation initiation factor 1 (IF-1)	0.000000006	pfam01176	Translation initiation factor 1A / IF-1.
===							7	Cenarchaeum_symbiosum_A_uid61411	NC_014820	chromosome									
118575988	ATP-dependent endonuclease	No	0	742613	744697	-		Cenarchaeum_symbiosum_A_uid61411	NC_014820	chromosome	arCOG03235,a	arCOG03235	4 (-)	L		Predicted ATP-dependent endonuclease of the (	0.0000003	pfam13175	AAA ATPase domain. This family of domain
118575989	hypothetical protein CENSYa_0795	No	0	744752	744946	-		Cenarchaeum_symbiosum_A_uid61411	NC_014820	chromosome									
118575990	hypothetical protein CENSYa_0796	No	0	745116	745358	+		Cenarchaeum_symbiosum_A_uid61411	NC_014820	chromosome									
118575991	hypothetical protein CENSYa_0797	No	0	745382	745600	+		Cenarchaeum_symbiosum_A_uid61411	NC_014820	chromosome									
118575992	hypothetical protein CENSYa_0798	No	0	745636	746031	+		Cenarchaeum_symbiosum_A_uid61411	NC_014820	chromosome									
118575993	hydroxypyruvate reductase	No	0	746118	747377	+		Cenarchaeum_symbiosum_A_uid61411	NC_014820	chromosome		arCOG04170	1 (-)	G	GckA	Putative glycerate kinase	5E-61	COG2379	Putative glycerate kinase
118575994	hypothetical protein CENSYa_0800	No	0	747374	747733	+		Cenarchaeum_symbiosum_A_uid61411	NC_014820	chromosome									
===							14	Cenarchaeum_symbiosum_A_uid61411	NC_014820	chromosome									
118575996	citrate synthase	No	0	747976	749373	-		Cenarchaeum_symbiosum_A_uid61411	NC_014820	chromosome		arCOG04237	1 (-)	C	GltA	Citrate synthase	4E-114	pfam00285	Citrate synthase.
118575997	hypothetical protein CENSYa_0803	No	0	749466	749858	+		Cenarchaeum_symbiosum_A_uid61411	NC_014820	chromosome									
118575998	hypothetical protein CENSYa_0804	No	0	750061	750639	+		Cenarchaeum_symbiosum_A_uid61411	NC_014820	chromosome	clust_175								
118575999	hypothetical protein CENSYa_0805	No	0	750639	750935	+		Cenarchaeum_symbiosum_A_uid61411	NC_014820	chromosome									
118576000	hypothetical protein CENSYa_0806	No	0	751203	751442	+		Cenarchaeum_symbiosum_A_uid61411	NC_014820	chromosome									
118576001	hypothetical protein CENSYa_0807	No	0	751656	751829	+		Cenarchaeum_symbiosum_A_uid61411	NC_014820	chromosome									
118576002	hypothetical protein CENSYa_0808	No	0	752168	752770	+		Cenarchaeum_symbiosum_A_uid61411	NC_014820	chromosome									
118576003	hypothetical protein CENSYa_0809	No	0	752963	753553	+		Cenarchaeum_symbiosum_A_uid61411	NC_014820	chromosome	clust_175								
118576004	hypothetical protein CENSYa_0810	No	0	753666	754043	+		Cenarchaeum_symbiosum_A_uid61411	NC_014820	chromosome									
118576005	hypothetical protein CENSYa_0811	No	0	754069	754296	+		Cenarchaeum_symbiosum_A_uid61411	NC_014820	chromosome	clust_380								
118576006	hypothetical protein CENSYa_0812	No	0	755487	755963	+		Cenarchaeum_symbiosum_A_uid61411	NC_014820	chromosome									
118576007	hypothetical protein CENSYa_0813	No	0	756036	756242	+		Cenarchaeum_symbiosum_A_uid61411	NC_014820	chromosome									
118576008	hypothetical protein CENSYa_0814	No	0	756799	756996	+		Cenarchaeum_symbiosum_A_uid61411	NC_014820	chromosome		arCOG10409	1	S		Uncharacterized conserved protein			
118576009	single-stranded DNA-specific exonuclease	No	0	756993	757994	+		Cenarchaeum_symbiosum_A_uid61411	NC_014820	chromosome		arCOG00424	1 (-)	L		DHH superfamily phosphohydrolase/exonuclease			
===							5	Cenarchaeum_symbiosum_A_uid61411	NC_014820	chromosome									
118576030	hypothetical protein CENSYa_0836	No	0	815152	815367	-		Cenarchaeum_symbiosum_A_uid61411	NC_014820	chromosome									
118576031	hypothetical protein CENSYa_0837	No	0	815364	815846	-		Cenarchaeum_symbiosum_A_uid61411	NC_014820	chromosome									
118576032	adenylate cyclase	No	0	815903	816799	-		Cenarchaeum_symbiosum_A_uid61411	NC_014820	chromosome		arCOG07810	2 (-)	T	CyaA	Adenylate cyclase, family 3	4E-17	COG2114	Adenylate cyclase, family 3 (some protei
118576033	hypothetical protein CENSYa_0839	Yes	0	816881	817426	-		Cenarchaeum_symbiosum_A_uid61411	NC_014820	chromosome									
118576034	hypothetical protein CENSYa_0840	No	0	817535	817798	+		Cenarchaeum_symbiosum_A_uid61411	NC_014820	chromosome	clust_380								
===							7	Cenarchaeum_symbiosum_A_uid61411	NC_014820	chromosome									
118576038	hypothetical protein CENSYa_0844	Yes	0	832133	839686	+		Cenarchaeum_symbiosum_A_uid61411	NC_014820	chromosome		arCOG02507	9	S		beta-propeller repeat protein	5E-12	COG3391	Uncharacterized conserved protein
118576039	hypothetical protein CENSYa_0845	No	0	839751	839936	+		Cenarchaeum_symbiosum_A_uid61411	NC_014820	chromosome									
118576040	hypothetical protein CENSYa_0846	Yes	1	839974	847263	+		Cenarchaeum_symbiosum_A_uid61411	NC_014820	chromosome	arCOG02503,a	arCOG02503	2	S		beta-propeller repeat protein	0.000000003	COG2319	FOG: WD40 repeat
118576041	phosphoribosylaminoimidazole carboxylase (N	No	0	847322	848458	+		Cenarchaeum_symbiosum_A_uid61411	NC_014820	chromosome		arCOG01597	1 (-)	F	PurK	Phosphoribosylaminoimidazole carboxylase (NC	2E-99	COG0026	Phosphoribosylaminoimidazole carboxylase
118576042	hypothetical protein CENSYa_0848	Yes	1	848541	859541	+		Cenarchaeum_symbiosum_A_uid61411	NC_014820	chromosome		arCOG02507	9	S		beta-propeller repeat protein	0.000000001	COG3391	Uncharacterized conserved protein
118576043	hypothetical protein CENSYa_0849	Yes	1	859605	875345	+		Cenarchaeum_symbiosum_A_uid61411	NC_014820	chromosome	arCOG02503,a	arCOG02503	2	S		beta-propeller repeat protein	1E-11	COG2319	FOG: WD40 repeat
118576044	hypothetical protein CENSYa_0850	No	0	875348	877651	+		Cenarchaeum_symbiosum_A_uid61411	NC_014820	chromosome		arCOG02507	9	S		beta-propeller repeat protein	0.0001	pfam12406	Surface protein. This family of proteins
===							8	Cenarchaeum_symbiosum_A_uid61411	NC_014820	chromosome									
118576082	DNA modification methylase	No	0	908279	909037	+		Cenarchaeum_symbiosum_A_uid61411	NC_014820	chromosome		arCOG00115	10 (-)	L		DNA modification methylase	7E-35	pfam01555	DNA methylase. Members of this family ar
118576083	hypothetical protein CENSYa_0890	No	0	909034	909183	+		Cenarchaeum_symbiosum_A_uid61411	NC_014820	chromosome									
118576084	hypothetical protein CENSYa_0891	No	0	909332	909931	-		Cenarchaeum_symbiosum_A_uid61411	NC_014820	chromosome	clust_173								
118576085	hypothetical protein CENSYa_0892	No	0	909957	910895	-		Cenarchaeum_symbiosum_A_uid61411	NC_014820	chromosome	clust_103								
118576086	hypothetical protein CENSYa_0893	No	0	911046	911345	-		Cenarchaeum_symbiosum_A_uid61411	NC_014820	chromosome									
118576087	hypothetical protein CENSYa_0894	No	1	911380	911700	-		Cenarchaeum_symbiosum_A_uid61411	NC_014820	chromosome									
118576088	hypothetical protein CENSYa_0895	No	0	911771	912091	-		Cenarchaeum_symbiosum_A_uid61411	NC_014820	chromosome		arCOG06569	1 (-)	S		Uncharacterized conserved protein	1E-20	COG2343	Uncharacterized protein conserved in bac
118576089	hypothetical protein CENSYa_0896	Yes	1	912145	912786	-		Cenarchaeum_symbiosum_A_uid61411	NC_014820	chromosome		arCOG08821	18	S		Uncharacterized conserved protein (expansion in Thaumarchaeota)			
===							6	Cenarchaeum_symbiosum_A_uid61411	NC_014820	chromosome									
118576113	universal stress protein	No	0	975284	975703	+		Cenarchaeum_symbiosum_A_uid61411	NC_014820	chromosome		arCOG02053	7 (-)	T	UspA	Nucleotide-binding protein, UspA family	1E-15	pfam00582	Universal stress protein family. The uni
118576114	hypothetical protein CENSYa_0921	No	0	975720	976049	+		Cenarchaeum_symbiosum_A_uid61411	NC_014820	chromosome									
118576115	hypothetical protein CENSYa_0922	No	0	976094	976318	+		Cenarchaeum_symbiosum_A_uid61411	NC_014820	chromosome									
118576116	hypothetical protein CENSYa_0923	No	0	976432	976617	+		Cenarchaeum_symbiosum_A_uid61411	NC_014820	chromosome									
118576117	hypothetical protein CENSYa_0924	No	0	976614	976907	-		Cenarchaeum_symbiosum_A_uid61411	NC_014820	chromosome									
118576118	molecular chaperone DnaJ	No	0	977223	978425	+		Cenarchaeum_symbiosum_A_uid61411	NC_014820	chromosome		arCOG02846	3 (-)	O	DnaJ	DnaJ-class molecular chaperone	0.000001	COG2214	DnaJ-class molecular chaperone
===							6	Cenarchaeum_symbiosum_A_uid61411	NC_014820	chromosome									
118576137	hypothetical protein CENSYa_0944	Yes	1	997413	998363	+		Cenarchaeum_symbiosum_A_uid61411	NC_014820	chromosome		arCOG08033	4 (-)	S		Uncharacterized protein			
118576138	hypothetical protein CENSYa_0945	No	0	998394	998633	+		Cenarchaeum_symbiosum_A_uid61411	NC_014820	chromosome									
118576139	hypothetical protein CENSYa_0946	No	0	998695	998916	+		Cenarchaeum_symbiosum_A_uid61411	NC_014820	chromosome									
118576140	hypothetical protein CENSYa_0947	No	0	998969	999205	+		Cenarchaeum_symbiosum_A_uid61411	NC_014820	chromosome									
118576141	hypothetical protein CENSYa_0948	Yes	0	999211	999483	+		Cenarchaeum_symbiosum_A_uid61411	NC_014820	chromosome									
118576142	ribosomal protein L15E	No	0	999655	1000113	+		Cenarchaeum_symbiosum_A_uid61411	NC_014820	chromosome		arCOG04209	3 (-)	J	RPL15A	Ribosomal protein L15E	3E-44	COG1632	Ribosomal protein L15E
===							9	Cenarchaeum_symbiosum_A_uid61411	NC_014820	chromosome									
118576216	hypothetical protein CENSYa_1023	No	0	1079854	1080078	-		Cenarchaeum_symbiosum_A_uid61411	NC_014820	chromosome									
118576217	hypothetical protein CENSYa_1024	No	0	1079998	1080228	+		Cenarchaeum_symbiosum_A_uid61411	NC_014820	chromosome									
118576218	hypothetical protein CENSYa_1025	No	0	1080253	1080756	+		Cenarchaeum_symbiosum_A_uid61411	NC_014820	chromosome		arCOG00725	2 (-)	S		Uncharacterized conserved protein			
118576219	hypothetical protein CENSYa_1026	No	0	1080813	1080983	+		Cenarchaeum_symbiosum_A_uid61411	NC_014820	chromosome									
118576220	hypothetical protein CENSYa_1027	No	0	1081006	1081380	+		Cenarchaeum_symbiosum_A_uid61411	NC_014820	chromosome									
118576221	hypothetical protein CENSYa_1029	No	0	1081529	1082125	-		Cenarchaeum_symbiosum_A_uid61411	NC_014820	chromosome									
118576222	hypothetical protein CENSYa_1030	Yes	0	1082296	1082940	-		Cenarchaeum_symbiosum_A_uid61411	NC_014820	chromosome		arCOG08819	6	S		Uncharacterized conserved protein (expansion in Thaumarchaeota)			
118576223	copper-binding protein	No	0	1083059	1083400	-		Cenarchaeum_symbiosum_A_uid61411	NC_014820	chromosome		arCOG02926	8 (-)	C	PetE	Plastocyanin			
118576224	hypothetical protein CENSYa_1032	No	2	1083471	1084883	-		Cenarchaeum_symbiosum_A_uid61411	NC_014820	chromosome									
===							6	Cenarchaeum_symbiosum_A_uid61411	NC_014820	chromosome									
118576231	hypothetical protein CENSYa_1039	No	0	1089807	1090043	-		Cenarchaeum_symbiosum_A_uid61411	NC_014820	chromosome		arCOG08727	1 (-)	S		Uncharacterized conserved protein			
118576232	hypothetical protein CENSYa_1040	No	0	1090074	1091054	-		Cenarchaeum_symbiosum_A_uid61411	NC_014820	chromosome									
118576233	hypothetical protein CENSYa_1041	No	0	1091250	1091633	+		Cenarchaeum_symbiosum_A_uid61411	NC_014820	chromosome									
118576234	hypothetical protein CENSYa_1042	Yes	0	1091781	1092107	+		Cenarchaeum_symbiosum_A_uid61411	NC_014820	chromosome		arCOG10890	2	S		Uncharacterized conserved protein			
118576235	hypothetical protein CENSYa_1043	No	0	1092207	1092476	+		Cenarchaeum_symbiosum_A_uid61411	NC_014820	chromosome									
118576236	transcriptional regulator	No	0	1092881	1093765	+		Cenarchaeum_symbiosum_A_uid61411	NC_014820	chromosome		arCOG04793	2 (-)	V		Transcriptional regulator, predicted component of	2E-21	COG5340	Predicted transcriptional regulator
===							5	Cenarchaeum_symbiosum_A_uid61411	NC_014820	chromosome									
118576270	hypothetical protein CENSYa_1080	No	2	1121092	1121277	-		Cenarchaeum_symbiosum_A_uid61411	NC_014820	chromosome		arCOG10410	1	S		Uncharacterized conserved protein			
118576271	hypothetical protein CENSYa_1081	No	1	1121274	1121459	-		Cenarchaeum_symbiosum_A_uid61411	NC_014820	chromosome									
118576272	transcriptional regulator	No	0	1121523	1121801	-		Cenarchaeum_symbiosum_A_uid61411	NC_014820	chromosome		arCOG01055	9 (-)	K		Predicted transcriptional regulator	0.0000006	COG3432	Predicted transcriptional regulator
118576273	hypothetical protein CENSYa_1083	No	0	1121951	1122241	-		Cenarchaeum_symbiosum_A_uid61411	NC_014820	chromosome									
118576274	hypothetical protein CENSYa_1084	No	0	1122295	1122855	-		Cenarchaeum_symbiosum_A_uid61411	NC_014820	chromosome									
===							18	Cenarchaeum_symbiosum_A_uid61411	NC_014820	chromosome									
118576279	transposase	No	0	1125185	1125712	-		Cenarchaeum_symbiosum_A_uid61411	NC_014820	chromosome									
118576280	helicase	No	0	1126021	1126515	+		Cenarchaeum_symbiosum_A_uid61411	NC_014820	chromosome									
118576281	hypothetical protein CENSYa_1091	No	0	1126946	1127254	-		Cenarchaeum_symbiosum_A_uid61411	NC_014820	chromosome		arCOG04792	9 (-)	L		Transposase			
118576282	hypothetical protein CENSYa_1092	No	0	1127267	1127449	-		Cenarchaeum_symbiosum_A_uid61411	NC_014820	chromosome									
118576283	hypothetical protein CENSYa_1093	No	1	1127877	1128470	+		Cenarchaeum_symbiosum_A_uid61411	NC_014820	chromosome									
118576284	hypothetical protein CENSYa_1094	No	1	1128866	1129081	+		Cenarchaeum_symbiosum_A_uid61411	NC_014820	chromosome		arCOG08038	1 (-)	O		Putative membrane-associated protease inhibitor, contains alpha-2-macroglobulin-like domain			
118576285	hypothetical protein CENSYa_1095	No	0	1129308	1129655	-		Cenarchaeum_symbiosum_A_uid61411	NC_014820	chromosome									
118576286	hypothetical protein CENSYa_1096	No	0	1129791	1129970	-		Cenarchaeum_symbiosum_A_uid61411	NC_014820	chromosome									
118576287	hypothetical protein CENSYa_1097	No	0	1130317	1130535	-		Cenarchaeum_symbiosum_A_uid61411	NC_014820	chromosome									
118576288	hypothetical protein CENSYa_1098	No	0	1130586	1130807	-		Cenarchaeum_symbiosum_A_uid61411	NC_014820	chromosome									
118576289	hypothetical protein CENSYa_1099	No	0	1130977	1131225	-		Cenarchaeum_symbiosum_A_uid61411	NC_014820	chromosome									
118576290	hypothetical protein CENSYa_1100	Yes	1	1131255	1131935	-		Cenarchaeum_symbiosum_A_uid61411	NC_014820	chromosome		arCOG08821	18	S		Uncharacterized conserved protein (expansion in Thaumarchaeota)			
118576291	hypothetical protein CENSYa_1101	No	1	1132295	1133257	+		Cenarchaeum_symbiosum_A_uid61411	NC_014820	chromosome		arCOG08821	18	S		Uncharacterized conserved protein (expansion in Thaumarchaeota)			
118576292	hypothetical protein CENSYa_1102	No	0	1133262	1133816	-		Cenarchaeum_symbiosum_A_uid61411	NC_014820	chromosome									
118576293	integrase	No	0	1134113	1135633	-		Cenarchaeum_symbiosum_A_uid61411	NC_014820	chromosome		arCOG01241	31 (-)	L	XerC	Integrase			
118576294	hypothetical protein CENSYa_1104	No	0	1135648	1135932	-		Cenarchaeum_symbiosum_A_uid61411	NC_014820	chromosome									
118576295	hypothetical protein CENSYa_1105	No	1	1135978	1136346	-		Cenarchaeum_symbiosum_A_uid61411	NC_014820	chromosome									
118576296	hypothetical protein CENSYa_1106	No	0	1136414	1137073	-		Cenarchaeum_symbiosum_A_uid61411	NC_014820	chromosome									
===							6	Cenarchaeum_symbiosum_A_uid61411	NC_014820	chromosome									
118576300	hypothetical protein CENSYa_1110	No	0	1142673	1142894	-		Cenarchaeum_symbiosum_A_uid61411	NC_014820	chromosome									
118576301	hypothetical protein CENSYa_1111	No	0	1142897	1143076	-		Cenarchaeum_symbiosum_A_uid61411	NC_014820	chromosome									
118576302	hypothetical protein CENSYa_1112	No	0	1143199	1143930	-		Cenarchaeum_symbiosum_A_uid61411	NC_014820	chromosome		arCOG08678	1 (-)	S		Zn finger containing protein			
118576303	hypothetical protein CENSYa_1113	No	1	1144034	1144522	+		Cenarchaeum_symbiosum_A_uid61411	NC_014820	chromosome									
118576304	hypothetical protein CENSYa_1114	No	0	1144768	1145238	-		Cenarchaeum_symbiosum_A_uid61411	NC_014820	chromosome									
118576305	hypothetical protein CENSYa_1115	No	0	1145295	1145516	-		Cenarchaeum_symbiosum_A_uid61411	NC_014820	chromosome									
===							10	Cenarchaeum_symbiosum_A_uid61411	NC_014820	chromosome									
118576370	1,4-dihydroxy-2-naphthoate octaprenyltransfera	No	6	1216088	1216966	+		Cenarchaeum_symbiosum_A_uid61411	NC_014820	chromosome		arCOG00480	2 (-)	H	MenA	1,4-dihydroxy-2-naphthoate octaprenyltransferase	1E-36	COG1575	1,4-dihydroxy-2-naphthoate octaprenyltra
118576371	hypothetical protein CENSYa_1186	No	0	1217275	1217412	-		Cenarchaeum_symbiosum_A_uid61411	NC_014820	chromosome									
118576372	hypothetical protein CENSYa_1187	No	0	1217532	1217717	-		Cenarchaeum_symbiosum_A_uid61411	NC_014820	chromosome									
118576373	hypothetical protein CENSYa_1188	No	0	1217788	1217967	+		Cenarchaeum_symbiosum_A_uid61411	NC_014820	chromosome	clust_479								
118576374	hypothetical protein CENSYa_1189	No	0	1218118	1218297	+		Cenarchaeum_symbiosum_A_uid61411	NC_014820	chromosome									
118576375	hypothetical protein CENSYa_1190	No	0	1218364	1218543	+		Cenarchaeum_symbiosum_A_uid61411	NC_014820	chromosome	clust_479								
118576376	hypothetical protein CENSYa_1191	No	0	1218688	1219095	+		Cenarchaeum_symbiosum_A_uid61411	NC_014820	chromosome									
118576377	hypothetical protein CENSYa_1192	No	0	1219561	1219926	-		Cenarchaeum_symbiosum_A_uid61411	NC_014820	chromosome									
118576378	hypothetical protein CENSYa_1193	No	0	1220046	1220363	-		Cenarchaeum_symbiosum_A_uid61411	NC_014820	chromosome									
118576379	oxidoreductase	No	0	1220391	1221509	+		Cenarchaeum_symbiosum_A_uid61411	NC_014820	chromosome		arCOG01623	1 (-)	C	Tas	Predicted oxidoreductase (related to aryl-alcohol	2E-13	COG0667	Predicted oxidoreductases (related to ar
===							8	Cenarchaeum_symbiosum_A_uid61411	NC_014820	chromosome									
118576426	ribosomal protein L12E/L44/L45/RPP1/RPP2	No	0	1268526	1268822	-		Cenarchaeum_symbiosum_A_uid61411	NC_014820	chromosome		arCOG04287	1 (-)	J	RPP1A	Ribosomal protein L12E/L44/L45/RPP1/RPP2	2E-24	COG2058	Ribosomal protein L12E/L44/L45/RPP1/RPP2
118576427	hypothetical protein CENSYa_1243	No	0	1268948	1269274	-		Cenarchaeum_symbiosum_A_uid61411	NC_014820	chromosome									
118576428	hypothetical protein CENSYa_1244	No	0	1269327	1269599	+		Cenarchaeum_symbiosum_A_uid61411	NC_014820	chromosome									
118576429	hypothetical protein CENSYa_1245	No	1	1269744	1270154	-		Cenarchaeum_symbiosum_A_uid61411	NC_014820	chromosome									
118576430	hypothetical protein CENSYa_1246	No	0	1270291	1270572	-		Cenarchaeum_symbiosum_A_uid61411	NC_014820	chromosome									
118576431	hypothetical protein CENSYa_1247	No	1	1270607	1270765	-		Cenarchaeum_symbiosum_A_uid61411	NC_014820	chromosome									
118576432	hypothetical protein CENSYa_1248	No	0	1271069	1271470	-		Cenarchaeum_symbiosum_A_uid61411	NC_014820	chromosome									
118576433	hypothetical protein CENSYa_1249	No	0	1271524	1271829	-		Cenarchaeum_symbiosum_A_uid61411	NC_014820	chromosome		arCOG03377	4 (-)	E		Serine protease			
===							6	Cenarchaeum_symbiosum_A_uid61411	NC_014820	chromosome									
118576476	Fe-S oxidoreductase	No	0	1306840	1307787	-		Cenarchaeum_symbiosum_A_uid61411	NC_014820	chromosome		arCOG00938	12 (-)	R		Radical SAM superfamily enzyme	7E-15	COG0535	Predicted Fe-S oxidoreductases
118576477	hypothetical protein CENSYa_1293	No	0	1307786	1308460	+		Cenarchaeum_symbiosum_A_uid61411	NC_014820	chromosome									
118576478	hypothetical protein CENSYa_1294	No	0	1308622	1308768	+		Cenarchaeum_symbiosum_A_uid61411	NC_014820	chromosome									
118576479	hypothetical protein CENSYa_1295	No	0	1308891	1309226	+		Cenarchaeum_symbiosum_A_uid61411	NC_014820	chromosome									
118576480	hypothetical protein CENSYa_1296	No	0	1309226	1309435	+		Cenarchaeum_symbiosum_A_uid61411	NC_014820	chromosome									
118576481	protein-tyrosine phosphatase	No	0	1309462	1309962	+		Cenarchaeum_symbiosum_A_uid61411	NC_014820	chromosome		arCOG03413	1 (-)	T	CDC14	Protein-tyrosine phosphatase	2E-14	COG2453	Predicted protein-tyrosine phosphatase [
===							7	Cenarchaeum_symbiosum_A_uid61411	NC_014820	chromosome									
118576500	hypothetical protein CENSYa_1316	No	0	1325819	1328764	+		Cenarchaeum_symbiosum_A_uid61411	NC_014820	chromosome		arCOG08752	1	S		Uncharacterized conserved protein			
118576501	dehydrogenase	No	0	1328925	1329713	-		Cenarchaeum_symbiosum_A_uid61411	NC_014820	chromosome		arCOG00570	2 (-)	C	FixC	Dehydrogenase (flavoprotein)	1E-35	COG0644	Dehydrogenases (flavoproteins)
118576502	hypothetical protein CENSYa_1318	No	0	1330108	1331244	+		Cenarchaeum_symbiosum_A_uid61411	NC_014820	chromosome	arCOG08819,a	arCOG08819	6	S		Uncharacterized conserved protein (expansion in Thaumarchaeota)			
118576503	hypothetical protein CENSYa_1319	No	0	1331508	1331702	+		Cenarchaeum_symbiosum_A_uid61411	NC_014820	chromosome									
118576504	hypothetical protein CENSYa_1320	No	1	1331815	1333269	+		Cenarchaeum_symbiosum_A_uid61411	NC_014820	chromosome	arCOG08819,a	arCOG08819	6	S		Uncharacterized conserved protein (expansion in Thaumarchaeota)			
118576505	hypothetical protein CENSYa_1321	No	1	1333353	1334525	+		Cenarchaeum_symbiosum_A_uid61411	NC_014820	chromosome	arCOG08819,a	arCOG08819	6	S		Uncharacterized conserved protein (expansion in Thaumarchaeota)			
118576506	hypothetical protein CENSYa_1322	No	0	1334601	1334810	-		Cenarchaeum_symbiosum_A_uid61411	NC_014820	chromosome		arCOG08753	1 (-)	S		Zn finger containing protein			
===							8	Cenarchaeum_symbiosum_A_uid61411	NC_014820	chromosome									
118576544	short-chain alcohol dehydrogenase	No	0	1386379	1387209	-		Cenarchaeum_symbiosum_A_uid61411	NC_014820	chromosome		arCOG01259	2 (-)	I	FabG	Short-chain alcohol dehydrogenase	1E-31	COG1028	Dehydrogenases with different specificit
118576545	hypothetical protein CENSYa_1361	Yes	0	1387550	1394299	+		Cenarchaeum_symbiosum_A_uid61411	NC_014820	chromosome		arCOG02507	9	S		beta-propeller repeat protein	0.000000001	COG3391	Uncharacterized conserved protein
118576546	hypothetical protein CENSYa_1362	No	1	1395236	1395682	+		Cenarchaeum_symbiosum_A_uid61411	NC_014820	chromosome									
118576547	hypothetical protein CENSYa_1363	No	2	1395780	1396010	-		Cenarchaeum_symbiosum_A_uid61411	NC_014820	chromosome									
118576548	hypothetical protein CENSYa_1364	No	2	1396308	1396718	+		Cenarchaeum_symbiosum_A_uid61411	NC_014820	chromosome									
118576549	hypothetical protein CENSYa_1365	Yes	0	1396842	1397219	-		Cenarchaeum_symbiosum_A_uid61411	NC_014820	chromosome									
118576550	hypothetical protein CENSYa_1366	Yes	0	1397216	1397383	-		Cenarchaeum_symbiosum_A_uid61411	NC_014820	chromosome									
118576551	phosphate uptake regulator	No	0	1397483	1398139	-		Cenarchaeum_symbiosum_A_uid61411	NC_014820	chromosome		arCOG00232	2 (-)	P	PhoU	Phosphate uptake regulator	2E-11	COG0704	Phosphate uptake regulator
===							5	Cenarchaeum_symbiosum_A_uid61411	NC_014820	chromosome									
118576556	hypothetical protein CENSYa_1372	No	0	1402739	1403380	+		Cenarchaeum_symbiosum_A_uid61411	NC_014820	chromosome									
118576557	hypothetical protein CENSYa_1373	No	0	1403454	1403711	+		Cenarchaeum_symbiosum_A_uid61411	NC_014820	chromosome									
118576558	hypothetical protein CENSYa_1374	No	0	1403714	1404076	+		Cenarchaeum_symbiosum_A_uid61411	NC_014820	chromosome									
118576559	branched-chain amino acid aminotransferase/4	No	0	1404088	1405023	+		Cenarchaeum_symbiosum_A_uid61411	NC_014820	chromosome		arCOG02297	2 (-)	E	IlvE	Branched-chain amino acid aminotransferase/4-ar	9E-51	COG0115	Branched-chain amino acid aminotransfera
118576560	hypothetical protein CENSYa_1376	No	0	1405056	1405232	+		Cenarchaeum_symbiosum_A_uid61411	NC_014820	chromosome		arCOG08759	1	S		Zn finger containing protein			
===							16	Cenarchaeum_symbiosum_A_uid61411	NC_014820	chromosome									
118576697	type II restriction enzyme, methylase subunit	No	0	1527616	1531587	-		Cenarchaeum_symbiosum_A_uid61411	NC_014820	chromosome	arCOG04814,a	arCOG04814	1 (-)	V		Type II restriction enzyme, methylase subunit			
118576698	hypothetical protein CENSYa_1515	No	0	1531832	1532428	-		Cenarchaeum_symbiosum_A_uid61411	NC_014820	chromosome		arCOG08821	18	S		Uncharacterized conserved protein (expansion in Thaumarchaeota)			
118576699	hypothetical protein CENSYa_1516	No	0	1532514	1532735	+		Cenarchaeum_symbiosum_A_uid61411	NC_014820	chromosome									
118576700	hypothetical protein CENSYa_1517	Yes	1	1532794	1534713	-		Cenarchaeum_symbiosum_A_uid61411	NC_014820	chromosome		arCOG09437	1	S		Uncharacterized conserved protein	0.00001	pfam05887	Procyclic acidic repetitive protein (PAR
118576701	hypothetical protein CENSYa_1518	Yes	1	1535025	1535654	-		Cenarchaeum_symbiosum_A_uid61411	NC_014820	chromosome		arCOG08821	18	S		Uncharacterized conserved protein (expansion in Thaumarchaeota)			
118576702	hypothetical protein CENSYa_1519	Yes	1	1535754	1536410	-		Cenarchaeum_symbiosum_A_uid61411	NC_014820	chromosome		arCOG08821	18	S		Uncharacterized conserved protein (expansion in Thaumarchaeota)			
118576703	hypothetical protein CENSYa_1520	Yes	1	1536671	1537327	-		Cenarchaeum_symbiosum_A_uid61411	NC_014820	chromosome		arCOG08821	18	S		Uncharacterized conserved protein (expansion in Thaumarchaeota)			
118576704	hypothetical protein CENSYa_1521	Yes	1	1537396	1538055	-		Cenarchaeum_symbiosum_A_uid61411	NC_014820	chromosome		arCOG08821	18	S		Uncharacterized conserved protein (expansion in Thaumarchaeota)			
118576705	hypothetical protein CENSYa_1522	No	0	1538339	1538566	-		Cenarchaeum_symbiosum_A_uid61411	NC_014820	chromosome									
118576706	transposase	No	0	1538566	1539876	-		Cenarchaeum_symbiosum_A_uid61411	NC_014820	chromosome		arCOG02134	15 (-)	L		Transposase	0.0000004	COG3316	Transposase and inactivated derivatives
118576707	hypothetical protein CENSYa_1524	No	0	1540020	1540457	+		Cenarchaeum_symbiosum_A_uid61411	NC_014820	chromosome									

118576708	hypothetical protein CENSYa_1525	No	0		1540539	1540721	+		Cenarchaeum_symbiosum_A_uid61411	NC_014820	chromosome										
118576709	hypothetical protein CENSYa_1526	No	0		1540751	1541194	+		Cenarchaeum_symbiosum_A_uid61411	NC_014820	chromosome	clust_236									
118576710	hypothetical protein CENSYa_1527	No	0		1541858	1542301	+		Cenarchaeum_symbiosum_A_uid61411	NC_014820	chromosome	clust_236									
118576711	hypothetical protein CENSYa_1528	No	0		1542381	1542848	+		Cenarchaeum_symbiosum_A_uid61411	NC_014820	chromosome										
118576712	thymidylate kinase	No	0		1543420	1543980	+		Cenarchaeum_symbiosum_A_uid61411	NC_014820	chromosome		arCOG01891	2	(-)	F	Tmk	Thymidylate kinase	2E-11	COG0125	Thymidylate kinase
===								7	Cenarchaeum_symbiosum_A_uid61411	NC_014820	chromosome										
118576721	exosome complex subunit	No	0		1549535	1550221	-		Cenarchaeum_symbiosum_A_uid61411	NC_014820	chromosome		arCOG04187	2	(-)	J		Predicted exosome subunit	6E-67	COG1500	Predicted exosome subunit
118576722	hypothetical protein CENSYa_1539	No	0		1550220	1550750	+		Cenarchaeum_symbiosum_A_uid61411	NC_014820	chromosome										
118576723	hypothetical protein CENSYa_1540	Yes	1		1550947	1551618	-		Cenarchaeum_symbiosum_A_uid61411	NC_014820	chromosome		arCOG08821	18		S		Uncharacterized conserved protein (expansion in Thaumarchaeota)			
118576724	hypothetical protein CENSYa_1541	No	0		1551615	1551815	-		Cenarchaeum_symbiosum_A_uid61411	NC_014820	chromosome										
118576725	hypothetical protein CENSYa_1542	No	0		1551812	1552168	-		Cenarchaeum_symbiosum_A_uid61411	NC_014820	chromosome										
118576726	hypothetical protein CENSYa_1543	Yes	1		1552229	1552930	-		Cenarchaeum_symbiosum_A_uid61411	NC_014820	chromosome		arCOG08821	18		S		Uncharacterized conserved protein (expansion in Thaumarchaeota)			
118576727	DNA-directed RNA polymerase, alpha subunit/	No	0		1553362	1554000	+		Cenarchaeum_symbiosum_A_uid61411	NC_014820	chromosome		arCOG04241	1	(-)	K	RpoA/Rp(	DNA-directed RNA polymerase subunit D	0.00002	pfam01000	RNA polymerase Rpb3/RpoA insert domain.
===								6	Cenarchaeum_symbiosum_A_uid61411	NC_014820	chromosome										
118576741	hypothetical protein CENSYa_1560	No	0		1564847	1565596	-		Cenarchaeum_symbiosum_A_uid61411	NC_014820	chromosome		arCOG07100	1	(-)	R		RNAse H superfamily ribonuclease	8E-11	pfam12686	Protein of unknown function (DUF3800). T
118576742	hypothetical protein CENSYa_1561	No	0		1565713	1565934	-		Cenarchaeum_symbiosum_A_uid61411	NC_014820	chromosome										
118576743	hypothetical protein CENSYa_1562	No	1		1565969	1567027	-		Cenarchaeum_symbiosum_A_uid61411	NC_014820	chromosome	clust_90									
118576744	hypothetical protein CENSYa_1563	No	0		1567050	1568027	-		Cenarchaeum_symbiosum_A_uid61411	NC_014820	chromosome										
118576745	hypothetical protein CENSYa_1564	No	0		1568418	1568831	+		Cenarchaeum_symbiosum_A_uid61411	NC_014820	chromosome										
118576746	hypothetical protein CENSYa_1565	Yes	0		1569188	1570354	-		Cenarchaeum_symbiosum_A_uid61411	NC_014820	chromosome		arCOG08777	3	(-)	S		Uncharacterized conserved protein			
===								14	Cenarchaeum_symbiosum_A_uid61411	NC_014820	chromosome										
118576766	uncharacterized protein with a von Willebrand f	No	0		1595530	1596843	-		Cenarchaeum_symbiosum_A_uid61411	NC_014820	chromosome								0.00000001	COG4867	Uncharacterized protein with a von Wille
118576767	Mg-chelatase subunit	No	0		1596935	1598692	-		Cenarchaeum_symbiosum_A_uid61411	NC_014820	chromosome		arCOG00438	2	(-)	R		AAA family ATPase	5E-12	COG1239	Mg-chelatase subunit ChlI
118576768	hypothetical protein CENSYa_1587	No	0		1598693	1598932	+		Cenarchaeum_symbiosum_A_uid61411	NC_014820	chromosome	clust_9									
118576769	hypothetical protein CENSYa_1588	No	0		1599003	1599281	+		Cenarchaeum_symbiosum_A_uid61411	NC_014820	chromosome										
118576770	hypothetical protein CENSYa_1589	No	0		1599320	1599487	+		Cenarchaeum_symbiosum_A_uid61411	NC_014820	chromosome	clust_9									
118576771	hypothetical protein CENSYa_1590	No	0		1599493	1599702	+		Cenarchaeum_symbiosum_A_uid61411	NC_014820	chromosome										
118576772	hypothetical protein CENSYa_1591	No	0		1599728	1599967	+		Cenarchaeum_symbiosum_A_uid61411	NC_014820	chromosome	clust_9									
118576773	hypothetical protein CENSYa_1592	No	0		1600016	1600222	+		Cenarchaeum_symbiosum_A_uid61411	NC_014820	chromosome										
118576774	hypothetical protein CENSYa_1593	No	0		1600249	1600452	+		Cenarchaeum_symbiosum_A_uid61411	NC_014820	chromosome	clust_9									
118576775	hypothetical protein CENSYa_1594	No	0		1600458	1600727	+		Cenarchaeum_symbiosum_A_uid61411	NC_014820	chromosome										
118576776	hypothetical protein CENSYa_1595	No	0		1600730	1600933	+		Cenarchaeum_symbiosum_A_uid61411	NC_014820	chromosome	clust_9									
118576777	hypothetical protein CENSYa_1596	No	0		1600930	1601115	+		Cenarchaeum_symbiosum_A_uid61411	NC_014820	chromosome										
118576778	hypothetical protein CENSYa_1597	No	0		1601372	1601953	-		Cenarchaeum_symbiosum_A_uid61411	NC_014820	chromosome										
118576779	ribosomal protein S13	No	0		1602364	1603047	+		Cenarchaeum_symbiosum_A_uid61411	NC_014820	chromosome		arCOG01722	1	(-)	J	RpsM	Ribosomal protein S13	3E-15	COG0099	Ribosomal protein S13
===								5	Cenarchaeum_symbiosum_A_uid61411	NC_014820	chromosome										
118576855	hypothetical protein CENSYa_1677	No	2		1679844	1680215	-		Cenarchaeum_symbiosum_A_uid61411	NC_014820	chromosome										
118576856	hypothetical protein CENSYa_1678	No	0		1680231	1680683	-		Cenarchaeum_symbiosum_A_uid61411	NC_014820	chromosome		arCOG08787	3	(-)	S		Uncharacterized conserved protein			
118576857	hypothetical protein CENSYa_1679	Yes	1		1680716	1680859	-		Cenarchaeum_symbiosum_A_uid61411	NC_014820	chromosome										
118576858	hypothetical protein CENSYa_1680	Yes	0		1680928	1681122	-		Cenarchaeum_symbiosum_A_uid61411	NC_014820	chromosome										
118576859	hypothetical protein CENSYa_1681	Yes	1		1681320	1681997	-		Cenarchaeum_symbiosum_A_uid61411	NC_014820	chromosome		arCOG08821	18		S		Uncharacterized conserved protein (expansion in Thaumarchaeota)			
===								7	Cenarchaeum_symbiosum_A_uid61411	NC_014820	chromosome										
118576917	hypothetical protein CENSYa_1745	No	0		1732262	1732492	-		Cenarchaeum_symbiosum_A_uid61411	NC_014820	chromosome										
118576918	hypothetical protein CENSYa_1746	No	0		1732509	1733099	-		Cenarchaeum_symbiosum_A_uid61411	NC_014820	chromosome		arCOG03706	3	(-)	V		PIN domain containing protein			
118576919	hypothetical protein CENSYa_1747	No	0		1733099	1733863	-		Cenarchaeum_symbiosum_A_uid61411	NC_014820	chromosome										
118576920	hypothetical protein CENSYa_1748	No	0		1734041	1737916	+		Cenarchaeum_symbiosum_A_uid61411	NC_014820	chromosome										
118576921	hypothetical protein CENSYa_1749	No	1		1737921	1738751	+		Cenarchaeum_symbiosum_A_uid61411	NC_014820	chromosome		arCOG08821	18		S		Uncharacterized conserved protein (expansion in Thaumarchaeota)			
118576922	hypothetical protein CENSYa_1750	Yes	0		1739041	1739682	-		Cenarchaeum_symbiosum_A_uid61411	NC_014820	chromosome		arCOG08821	18		S		Uncharacterized conserved protein (expansion in Thaumarchaeota)			
118576923	Zn-dependent oxidoreductase	No	0		1739791	1740870	+		Cenarchaeum_symbiosum_A_uid61411	NC_014820	chromosome		arCOG01458	1	(-)	C	Qor	NADPH:quinone reductase or related Zn-depend	5E-50	COG0604	NADPH:quinone reductase and related Zn-d
===								6	Cenarchaeum_symbiosum_A_uid61411	NC_014820	chromosome										
118576943	oxygen-sensitive ribonucleoside-triphosphate	No	0		1756597	1758057	-		Cenarchaeum_symbiosum_A_uid61411	NC_014820	chromosome	arCOG04889,a	arCOG04889	1	(-)	F	NrdD	Oxygen-sensitive ribonucleoside-triphosphate re	0.000000004	COG1328	Oxygen-sensitive ribonucleoside-triphosp
118576944	hypothetical protein CENSYa_1773	No	1		1758066	1758299	-		Cenarchaeum_symbiosum_A_uid61411	NC_014820	chromosome										
118576945	hypothetical protein CENSYa_1774	No	0		1758304	1758450	-		Cenarchaeum_symbiosum_A_uid61411	NC_014820	chromosome										
118576946	hypothetical protein CENSYa_1775	No	0		1758519	1759070	-		Cenarchaeum_symbiosum_A_uid61411	NC_014820	chromosome	clust_173									
118576947	hypothetical protein CENSYa_1776	No	0		1759162	1760109	-		Cenarchaeum_symbiosum_A_uid61411	NC_014820	chromosome	clust_103									
118576948	autotransporter adhesin	No	1		1760307	1763201	-		Cenarchaeum_symbiosum_A_uid61411	NC_014820	chromosome	arCOG07561,a	arCOG07561	7	(-)	K		Predicted regulatory protein containing HTH domain			
===								5	Cenarchaeum_symbiosum_A_uid61411	NC_014820	chromosome										
118577000	hypothetical protein CENSYa_1829	No	0		1809408	1809617	+		Cenarchaeum_symbiosum_A_uid61411	NC_014820	chromosome										
118577001	hypothetical protein CENSYa_1830	No	0		1809627	1809950	+		Cenarchaeum_symbiosum_A_uid61411	NC_014820	chromosome										
118577002	hypothetical protein CENSYa_1831	Yes	1		1810033	1811232	-		Cenarchaeum_symbiosum_A_uid61411	NC_014820	chromosome		arCOG02926	8	(-)	C	PetE	Plastocyanin	0.000007	COG3794	Plastocyanin
118577003	hypothetical protein CENSYa_1832	Yes	1		1811229	1811453	-		Cenarchaeum_symbiosum_A_uid61411	NC_014820	chromosome										
118577004	hypothetical protein CENSYa_1833	No	0		1811487	1811813	+		Cenarchaeum_symbiosum_A_uid61411	NC_014820	chromosome										
===								14	Cenarchaeum_symbiosum_A_uid61411	NC_014820	chromosome										
118577210	4-methyl-5-(beta-hydroxyethyl)thiazole monoph	No	0		2007223	2008599	+		Cenarchaeum_symbiosum_A_uid61411	NC_014820	chromosome	arCOG00536,a	arCOG00536	1	(-)	H	MoaD	Molybdopterin converting factor, small subunit	5E-56	COG0476	Dinucleotide-utilizing enzymes involved
118577211	hypothetical protein CENSYa_2047	No	0		2008883	2011555	-		Cenarchaeum_symbiosum_A_uid61411	NC_014820	chromosome										
118577212	hypothetical protein CENSYa_2048	No	4		2011552	2012247	-		Cenarchaeum_symbiosum_A_uid61411	NC_014820	chromosome										
118577213	hypothetical protein CENSYa_2049	No	0		2012343	2012567	-		Cenarchaeum_symbiosum_A_uid61411	NC_014820	chromosome										
118577214	hypothetical protein CENSYa_2050	No	0		2012607	2012888	+		Cenarchaeum_symbiosum_A_uid61411	NC_014820	chromosome										
118577215	hypothetical protein CENSYa_2051	No	0		2013120	2013734	+		Cenarchaeum_symbiosum_A_uid61411	NC_014820	chromosome		arCOG00725	2	(-)	S		Uncharacterized conserved protein			
118577216	hypothetical protein CENSYa_2052	No	0		2013953	2014357	+		Cenarchaeum_symbiosum_A_uid61411	NC_014820	chromosome										
118577217	hypothetical protein CENSYa_2053	No	0		2014409	2016274	+		Cenarchaeum_symbiosum_A_uid61411	NC_014820	chromosome										
118577218	hypothetical protein CENSYa_2054	No	0		2016351	2017487	+		Cenarchaeum_symbiosum_A_uid61411	NC_014820	chromosome										
118577219	hypothetical protein CENSYa_2055	No	0		2017581	2018819	+		Cenarchaeum_symbiosum_A_uid61411	NC_014820	chromosome										
118577220	hypothetical protein CENSYa_2056	Yes	0		2018918	2021014	+		Cenarchaeum_symbiosum_A_uid61411	NC_014820	chromosome										
118577221	hypothetical protein CENSYa_2057	No	1		2021083	2022129	+		Cenarchaeum_symbiosum_A_uid61411	NC_014820	chromosome	clust_90									
118577222	hypothetical protein CENSYa_2058	Yes	2		2022318	2023262	-		Cenarchaeum_symbiosum_A_uid61411	NC_014820	chromosome										
118577223	hypothetical protein CENSYa_2059	No	1		2023388	2028928	+		Cenarchaeum_symbiosum_A_uid61411	NC_014820	chromosome		arCOG08647	2	(-)	S		Uncharacterized conserved protein			
===								5	Desulfurococcus_fermentans_DSM_16532_uid75	NC_018001	chromosome										
390938556	hypothetical protein Desfe_0828	No	0		807052	807315	-		Desulfurococcus_fermentans_DSM_16532_uid75	NC_018001	chromosome										
390938557	hypothetical protein Desfe_0829	No	0		807485	807772	+		Desulfurococcus_fermentans_DSM_16532_uid75	NC_018001	chromosome										
390938558	HAD-superfamily hydrolase	No	0		808221	808931	+		Desulfurococcus_fermentans_DSM_16532_uid75	NC_018001	chromosome		arCOG02291	4	(-)	R		HAD superfamily hydrolase	2E-19	COG1011	Predicted hydrolase (HAD superfamily)
390938559	hypothetical protein Desfe_0831	No	0		809174	809341	-		Desulfurococcus_fermentans_DSM_16532_uid75	NC_018001	chromosome										
390938560	hypothetical protein Desfe_0832	No	0		809537	809836	+		Desulfurococcus_fermentans_DSM_16532_uid75	NC_018001	chromosome										
===								5	Desulfurococcus_fermentans_DSM_16532_uid75	NC_018001	chromosome										
390938641	Exosortase EpsH-like protein	No	13		887208	888806	-		Desulfurococcus_fermentans_DSM_16532_uid75	NC_018001	chromosome								2E-10	pfam09721	Transmembrane exosortase (Exosortase_Eps
390938642	ATPase	No	0		889040	890002	+		Desulfurococcus_fermentans_DSM_16532_uid75	NC_018001	chromosome		arCOG00434	3	(-)	R		MoxR-like ATPase	1E-62	COG0714	MoxR-like ATPases
390938643	hypothetical protein Desfe_0918	No	6		890008	890622	+		Desulfurococcus_fermentans_DSM_16532_uid75	NC_018001	chromosome										
390938644	hypothetical protein Desfe_0919	No	2		890615	891283	+		Desulfurococcus_fermentans_DSM_16532_uid75	NC_018001	chromosome										
390938645	hypothetical protein Desfe_0920	No	3		891264	891536	+		Desulfurococcus_fermentans_DSM_16532_uid75	NC_018001	chromosome	clust_52									
===								5	Desulfurococcus_fermentans_DSM_16532_uid75	NC_018001	chromosome										
390939010	hypothetical protein Desfe_1305	Yes	4		1228907	1230376	+		Desulfurococcus_fermentans_DSM_16532_uid75	NC_018001	chromosome		arCOG10888	7		S		Uncharacterized conserved protein	0.000001	pfam01456	Mucin-like glycoprotein. This family of
390939011	ATPase	No	0		1231700	1232299	+		Desulfurococcus_fermentans_DSM_16532_uid75	NC_018001	chromosome		arCOG03408	3	(-)	R		Predicted ATPase (AAA+ superfamily)	5E-12	pfam01637	Archaeal ATPase. This family contain a c
390939012	hypothetical protein Desfe_1307	No	3		1232641	1233126	+		Desulfurococcus_fermentans_DSM_16532_uid75	NC_018001	chromosome										
390939013	hypothetical protein Desfe_1308	No	12		1233193	1234548	+		Desulfurococcus_fermentans_DSM_16532_uid75	NC_018001	chromosome										
390939014	hypothetical protein Desfe_1309	No	3		1234542	1235012	+		Desulfurococcus_fermentans_DSM_16532_uid75	NC_018001	chromosome										
===								8	Desulfurococcus_kamchatkensis_1221n_uid5913	NC_011766	chromosome										
218883391	Biotin synthase-like protein	No	0		69072	69869	+		Desulfurococcus_kamchatkensis_1221n_uid5913	NC_011766	chromosome		arCOG05825	1	(-)	R		Radical SAM superfamily enzyme	4E-28	COG1856	Uncharacterized homolog of biotin synthe
218883392	hypothetical protein DKAM_0078	No	0		70364	70504	+		Desulfurococcus_kamchatkensis_1221n_uid5913	NC_011766	chromosome										
218883393	hypothetical protein DKAM_0079	Yes	1		70588	71253	+		Desulfurococcus_kamchatkensis_1221n_uid5913	NC_011766	chromosome										
218883394	hypothetical protein DKAM_0080	No	3		71271	72440	+		Desulfurococcus_kamchatkensis_1221n_uid5913	NC_011766	chromosome										
218883395	hypothetical protein DKAM_0081	No	4		72421	73113	+		Desulfurococcus_kamchatkensis_1221n_uid5913	NC_011766	chromosome										
218883396	hypothetical protein DKAM_0082	Yes	0		73131	75182	+		Desulfurococcus_kamchatkensis_1221n_uid5913	NC_011766	chromosome										
218883397	hypothetical protein DKAM_0083	No	0		75305	75430	+		Desulfurococcus_kamchatkensis_1221n_uid5913	NC_011766	chromosome										
218883398	hypothetical protein DKAM_0084	No	0		77043	77534	+		Desulfurococcus_kamchatkensis_1221n_uid5913	NC_011766	chromosome		arCOG06133	1	(-)	S		Uncharacterized conserved protein			
===								9	Desulfurococcus_kamchatkensis_1221n_uid5913	NC_011766	chromosome										
218883616	hypothetical protein DKAM_0305	No	1	YES	284255	284785	-		Desulfurococcus_kamchatkensis_1221n_uid5913	NC_011766	chromosome										
218883617	putative DNA methylase	No	0	YES	284979	285740	-		Desulfurococcus_kamchatkensis_1221n_uid5913	NC_011766	chromosome		arCOG00889	6	(-)	L		Adenine-specific DNA methylase containing a Zn	2E-71	COG1743	Adenine-specific DNA methylase containin
218883618	hypothetical protein DKAM_0307	No	0	YES	285740	285976	+		Desulfurococcus_kamchatkensis_1221n_uid5913	NC_011766	chromosome										
218883619	hypothetical protein DKAM_0308	No	0	YES	286622	286795	+		Desulfurococcus_kamchatkensis_1221n_uid5913	NC_011766	chromosome										
218883620	hypothetical protein DKAM_0309	No	0	YES	286818	287270	+		Desulfurococcus_kamchatkensis_1221n_uid5913	NC_011766	chromosome										
218883621	hypothetical protein DKAM_0310	No	0	YES	287228	287353	-		Desulfurococcus_kamchatkensis_1221n_uid5913	NC_011766	chromosome										
218883622	hypothetical protein DKAM_0311	No	0		287304	287876	-		Desulfurococcus_kamchatkensis_1221n_uid5913	NC_011766	chromosome										
218883623	glycosyl transferase, family 2	No	0		287866	288855	-		Desulfurococcus_kamchatkensis_1221n_uid5913	NC_011766	chromosome		arCOG01381	4	(-)	M	WcaA	Glycosyltransferase			
218883624	hypothetical protein DKAM_0313	No	0		288907	289572	-		Desulfurococcus_kamchatkensis_1221n_uid5913	NC_011766	chromosome										
===								6	Desulfurococcus_kamchatkensis_1221n_uid5913	NC_011766	chromosome										
218883708	putative solute binding protein	Yes	2		368530	371478	-		Desulfurococcus_kamchatkensis_1221n_uid5913	NC_011766	chromosome		arCOG01672	1	(-)	R		Predicted solute binding protein	6E-60	COG3889	Predicted solute binding protein
218883709	hypothetical protein DKAM_0398	No	2		371548	372093	+		Desulfurococcus_kamchatkensis_1221n_uid5913	NC_011766	chromosome										
218883710	hypothetical protein DKAM_0399	No	2		372155	372385	-		Desulfurococcus_kamchatkensis_1221n_uid5913	NC_011766	chromosome										
218883711	hypothetical protein DKAM_0400	No	0		372934	373170	-		Desulfurococcus_kamchatkensis_1221n_uid5913	NC_011766	chromosome										
218883712	hypothetical protein DKAM_0401	Yes	0		373154	373267	+		Desulfurococcus_kamchatkensis_1221n_uid5913	NC_011766	chromosome										
218883713	beta-galactosidase	No	0		373592	376519	+		Desulfurococcus_kamchatkensis_1221n_uid5913	NC_011766	chromosome		arCOG05856	1	(-)	G	LacA	Beta-galactosidase	2E-34	pfam01301	Glycosyl hydrolases family 35.
===								5	Desulfurococcus_kamchatkensis_1221n_uid5913	NC_011766	chromosome										
218883815	carbon starvation protein	No	2		474971	475192	-		Desulfurococcus_kamchatkensis_1221n_uid5913	NC_011766	chromosome										
218883816	hypothetical protein DKAM_0505	No	0		475398	475586	+		Desulfurococcus_kamchatkensis_1221n_uid5913	NC_011766	chromosome										
218883817	putative polysaccharide biosynthesis protein	No	13		475598	477163	-		Desulfurococcus_kamchatkensis_1221n_uid5913	NC_011766	chromosome		arCOG02209	4	(-)	R	RfbX	Polysaccharide biosynthesis protein, Mvin family	1E-20	COG2244	Membrane protein involved in the export
218883818	hypothetical protein DKAM_0507	No	0		477291	477506	+		Desulfurococcus_kamchatkensis_1221n_uid5913	NC_011766	chromosome										
218883819	hypothetical protein DKAM_0508	No	0		477775	478512	+		Desulfurococcus_kamchatkensis_1221n_uid5913	NC_011766	chromosome										
===								7	Desulfurococcus_kamchatkensis_1221n_uid5913	NC_011766	chromosome										
218883873	putative DNA methylase containing a Zn-ribbon	No	0	YES	526680	529643	-		Desulfurococcus_kamchatkensis_1221n_uid5913	NC_011766	chromosome		arCOG00889	6	(-)	L		Adenine-specific DNA methylase containing a Zn	1E-57	COG1743	Adenine-specific DNA methylase containin
218883874	hypothetical protein DKAM_0563	No	0	YES	530022	530168	-		Desulfurococcus_kamchatkensis_1221n_uid5913	NC_011766	chromosome										
218883875	hypothetical protein DKAM_0564	No	0	YES	530177	530341	-		Desulfurococcus_kamchatkensis_1221n_uid5913	NC_011766	chromosome										
218883876	hypothetical protein DKAM_0565	No	3	YES	531010	531294	+		Desulfurococcus_kamchatkensis_1221n_uid5913	NC_011766	chromosome										
218883877	hypothetical protein DKAM_0566	No	5	YES	531291	531869	+		Desulfurococcus_kamchatkensis_1221n_uid5913	NC_011766	chromosome										
218883878	GIYc domain containing protein	No	0		532149	532595	-		Desulfurococcus_kamchatkensis_1221n_uid5913	NC_011766	chromosome										
218883879	hypothetical protein DKAM_0568	Yes	4		532604	533023	-		Desulfurococcus_kamchatkensis_1221n_uid5913	NC_011766	chromosome		arCOG04811	3	(-)	S		Uncharacterized conserved membrane protein, Fun14 family			
===								11	Desulfurococcus_kamchatkensis_1221n_uid5913	NC_011766	chromosome										
218884004	mechanosensitive ion channel MscS	No	3		664325	665155	+		Desulfurococcus_kamchatkensis_1221n_uid5913	NC_011766	chromosome		arCOG01568	4	(-)	M	MscS	Small-conductance mechanosensitive channel	5E-47	COG0668	Small-conductance mechanosensitive chann
218884005	hypothetical protein DKAM_0694	No	0		665254	665907	+		Desulfurococcus_kamchatkensis_1221n_uid5913	NC_011766	chromosome										
218884006	hypothetical protein DKAM_0695	No	2		666451	667218	-		Desulfurococcus_kamchatkensis_1221n_uid5913	NC_011766	chromosome	clust_131									
218884007	hypothetical protein DKAM_0696	No	1		667215	667589	-		Desulfurococcus_kamchatkensis_1221n_uid5913	NC_011766	chromosome	clust_284									
218884008	hypothetical protein DKAM_0697	No	9		667628	669052	-		Desulfurococcus_kamchatkensis_1221n_uid5913	NC_011766	chromosome										
218884009	Flagella-like protein I	No	0		669049	670296	-		Desulfurococcus_kamchatkensis_1221n_uid5913	NC_011766	chromosome		arCOG01817	8	(-)	N	VirB11	Type IV secretory pathway, VirB11 component, o	2E-36	COG0630	Type IV secretory pathway, VirB11 compon
218884010	flagellar protein G	No	2		670447	670869	-		Desulfurococcus_kamchatkensis_1221n_uid5913	NC_011766	chromosome	clust_248									
218884011	hypothetical protein DKAM_0700	No	9		671019	672611	-		Desulfurococcus_kamchatkensis_1221n_uid5913	NC_011766	chromosome										
218884012	hypothetical protein DKAM_0701	No	4		672725	673288	+		Desulfurococcus_kamchatkensis_1221n_uid5913	NC_011766	chromosome		arCOG01644	2	(-)	S		Predicted membrane protein	2E-35	COG2245	Predicted membrane protein
218884013	hypothetical protein DKAM_0702	No	0		673487	673612	+		Desulfurococcus_kamchatkensis_1221n_uid5913	NC_011766	chromosome										
218884014	hypothetical protein DKAM_0703	No	0		673575	673727	+		Desulfurococcus_kamchatkensis_1221n_uid5913	NC_011766	chromosome										
===								5	Desulfurococcus_kamchatkensis_1221n_uid5913	NC_011766	chromosome										
218884206	hypothetical protein DKAM_0895	No	1		854579	854713	-		Desulfurococcus_kamchatkensis_1221n_uid5913	NC_011766	chromosome										
218884207	hypothetical protein DKAM_0896	No	0		855314	855571	-		Desulfurococcus_kamchatkensis_1221n_uid5913	NC_011766	chromosome										
218884208	hypothetical protein DKAM_0897	No	1		855575	855769	-		Desulfurococcus_kamchatkensis_1221n_uid5913	NC_011766	chromosome										
218884209	DNA primase, large subunit	No	0		855787	856932	-		Desulfurococcus_kamchatkensis_1221n_uid5913	NC_011766	chromosome		arCOG03013	2	(-)	L	PRI2	Eukaryotic-type DNA primase, large subunit	5E-33	COG2219	Eukaryotic-type DNA primase, large subun
218884210	hypothetical protein DKAM_0899	No	0		857193	857339	-		Desulfurococcus_kamchatkensis_1221n_uid5913	NC_011766	chromosome										
===								7	Desulfurococcus_kamchatkensis_1221n_uid5913	NC_011766	chromosome										
218884249	hypothetical protein DKAM_0938	Yes	2		891747	893330	-		Desulfurococcus_kamchatkensis_1221n_uid5913	NC_011766	chromosome		arCOG10888	7		S		Uncharacterized conserved protein	0.000004	pfam01456	Mucin-like glycoprotein. This family of
218884250	hypothetical protein DKAM_0939	No	0		893447	893695	-		Desulfurococcus_kamchatkensis_1221n_uid5913	NC_011766	chromosome		arCOG02238	1	(-)	R		Predicted DNA-binding protein	3E-13	COG1342	Predicted DNA-binding proteins
218884251	hypothetical protein DKAM_0940	No	0		893898	894032	+		Desulfurococcus_kamchatkensis_1221n_uid5913	NC_011766	chromosome										
218884252	hypothetical protein DKAM_0941	No	0		894046	894180	+		Desulfurococcus_kamchatkensis_1221n_uid5913	NC_011766	chromosome		arCOG11066	1		S		Uncharacterized conserved protein			
218884253	hypothetical protein DKAM_0942	No	0		894473	894628	+		Desulfurococcus_kamchatkensis_1221n_uid5913	NC_011766	chromosome										
218884254	hypothetical protein DKAM_0943	Yes	0		894668	894841	-		Desulfurococcus_kamchatkensis_1221n_uid5913	NC_011766	chromosome										
218884255	putative deoxyhypusine synthase	No	0		895518	896531	+		Desulfurococcus_kamchatkensis_1221n_uid5913	NC_011766	chromosome		arCOG04142	1	(-)	O	DYS1	Deoxyhypusine synthase	5E-52	COG1899	Deoxyhypusine synthase
===								5	Desulfurococcus_kamchatkensis_1221n_uid5913	NC_011766	chromosome										
218884739	hypothetical protein DKAM_1428	No	0		1324998	1325111	+		Desulfurococcus_kamchatkensis_1221n_uid5913	NC_011766	chromosome										
218884740	hypothetical protein DKAM_1429	No	0		1325111	1325230	+		Desulfurococcus_kamchatkensis_1221n_uid5913	NC_011766	chromosome										
218884741	hypothetical protein DKAM_1430	No	0		1325239	1325499	+		Desulfurococcus_kamchatkensis_1221n_uid5913	NC_011766	chromosome		arCOG00398	2	(-)	J		ASCH domain, predicted RNA-binding domain	1E-10	COG2411	Uncharacterized conserved protein
218884742	hypothetical protein DKAM_1431	No	0		1326198	1326338	-		Desulfurococcus_kamchatkensis_1221n_uid5913	NC_011766	chromosome										
218884743	hypothetical protein DKAM_1432	No	0		1326335	1326469	-		Desulfurococcus_kamchatkensis_1221n_uid5913	NC_011766	chromosome										
===								6	Desulfurococcus_mucosus_DSM_2162_uid62227	NC_014961	chromosome										
320100665	hypothetical protein Desmu_0461	No	0		453162	453527	+		Desulfurococcus_mucosus_DSM_2162_uid62227	NC_014961	chromosome										
320100666	hypothetical protein Desmu_0462	No	6		453524	454243	-		Desulfurococcus_mucosus_DSM_2162_uid62227	NC_014961	chromosome		arCOG11078	2		S		Uncharacterized conserved protein			
320100667	ABC transporter-like protein	No	0		454209	455066	-		Desulfurococcus_mucosus_DSM_2162_uid62227	NC_014961	chromosome		arCOG00194	14	(-)	V	CcmA	ABC-type multidrug transport system, ATPase co	4E-27	COG1118	ABC-type sulfate/molybdate transport sys
320100668	hypothetical protein Desmu_0464	No	2		455063	455989	-		Desulfurococcus_mucosus_DSM_2162_uid62227	NC_014961	chromosome		arCOG10923	1		S		Uncharacterized conserved protein			
320100669	hypothetical protein Desmu_0465	No	1		456508	456636	-		Desulfurococcus_mucosus_DSM_2162_uid62227	NC_014961	chromosome										
320100670	hypothetical protein Desmu_0466	No	1		457007	459052	-		Desulfurococcus_mucosus_DSM_2162_uid62227	NC_014961	chromosome										
===								7	Desulfurococcus_mucosus_DSM_2162_uid62227	NC_014961	chromosome										
320100675	putative TrbE protein	No	0		464205	465989	+		Desulfurococcus_mucosus_DSM_2162_uid62227	NC_014961	chromosome	arCOG04035,a	arCOG04035	5	(-)	U	VirB4	Type IV secretory pathway, VirB4 component	3E-18	pfam12846	AAA-like domain. This family of domains
320100676	hypothetical protein Desmu_0473	Yes	0		465972	466226	-		Desulfurococcus_mucosus_DSM_2162_uid62227	NC_014961	chromosome										
320100677	hypothetical protein Desmu_0474	Yes	4		466319	467224	+		Desulfurococcus_mucosus_DSM_2162_uid62227	NC_014961	chromosome										
320100678	hypothetical protein Desmu_0475	No	4		467221	468282	+		Desulfurococcus_mucosus_DSM_2162_uid62227	NC_014961	chromosome										
320100679	hypothetical protein Desmu_0476	No	9		468279	469781	+		Desulfurococcus_mucosus_DSM_2162_uid62227	NC_014961	chromosome										
320100680	ABC transporter-like protein	No	0		469778	470440	+		Desulfurococcus_mucosus_DSM_2162_uid62227	NC_014961	chromosome		arCOG00922	4	(-)	V	SalX	ABC-type antimicrobial peptide transport system,	1E-41	COG1136	ABC-type antimicrobial peptide transport
320100681	flagellar protein G	No	2		470495	470917	+		Desulfurococcus_mucosus_DSM_2162_uid62227	NC_014961	chromosome	clust_248									
===								6	Desulfurococcus_mucosus_DSM_2162_uid62227	NC_014961	chromosome										
320100683	hypothetical protein Desmu_0480	No	8		472210	473631	+		Desulfurococcus_mucosus_DSM_2162_uid62227	NC_014961	chromosome		arCOG01815	1	(-)	N	TadC	Flp pilus assembly protein TadC			
320100684	hypothetical protein Desmu_0481	Yes	1		473677	474042	+		Desulfurococcus_mucosus_DSM_2162_uid62227	NC_014961	chromosome	clust_284									
320100685	hypothetical protein Desmu_0482	No	1		474039	474791	+		Desulfurococcus_mucosus_DSM_2162_uid62227	NC_014961	chromosome	clust_131									
320100686	hypothetical protein Desmu_0483	No	0		474788	476434	-		Desulfurococcus_mucosus_DSM_2162_uid62227	NC_014961	chromosome										
320100687	hypothetical protein Desmu_0484	No	0		476594	476863	+		Desulfurococcus_mucosus_DSM_2162_uid62227	NC_014961	chromosome		arCOG11222	1		S		Uncharacterized conserved protein			
320100688	arginine decarboxylase	No	0		476891	478696	+		Desulfurococcus_mucosus_DSM_2162_uid62227	NC_014961	chromosome		arCOG02269	1	(-)	E	SpeA	Arginine decarboxylase (spermidine biosynthesi	3E-49	COG1166	Arginine decarboxylase (spermidine biosy
===								5	Ferroglobus_placidus_DSM_10642_uid40863	NC_013849	chromosome										
288930425	hypothetical protein Ferp_0018	No	0	YES	15530	15988	+		Ferroglobus_placidus_DSM_10642_uid40863	NC_013849	chromosome										
288930426	hypothetical protein Ferp_0019	No	0	YES	16075	16290	+		Ferroglobus_placidus_DSM_10642_uid40863	NC_013849	chromosome		arCOG09828	3		V		RelB family antitoxin	0.0000002	COG1196	Chromosome segregation ATPases
288930427	plasmid stabilization system	No	0	YES	16280	16540	+		Ferroglobus_placidus_DSM_10642_uid40863	NC_013849	chromosome		arCOG01665	6	(-)	V	RelE	Cytotoxic translational repressor of toxin-antitoxin	0.000000007	COG2026	Cytotoxic translational repressor of tox
288930428	hypothetical protein Ferp_0021	No	0	YES	16806	16925	+		Ferroglobus_placidus_DSM_10642_uid40863	NC_013849	chromosome										
288930429	hypothetical protein Ferp_0022	No	4	YES	17186	17896	+		Ferroglobus_placidus_DSM_10642_uid40863	NC_013849	chromosome										
===								8	Ferroglobus_placidus_DSM_10642_uid40863	NC_013849	chromosome										
288930727	hypothetical protein Ferp_0333	No	2		291747	292277	+		Ferroglobus_placidus_DSM_10642_uid40863	NC_013849	chromosome										
288930728	hypothetical protein Ferp_0334	No	1		292353	292586	-		Ferroglobus_placidus_DSM_10642_uid40863	NC_013849	chromosome										
288930729	transposase IS240-A	No	0		293028	293240	-		Ferroglobus_placidus_DSM_10642_uid40863	NC_013849	chromosome		arCOG02134	15	(-)	L		Transposase			
288930730	hypothetical protein Ferp_0336	Yes	1		293455	293955	+		Ferroglobus_placidus_DSM_10642_uid40863	NC_013849	chromosome										
288930731	hypothetical protein Ferp_0337	No	5		294001	294459	-		Ferroglobus_placidus_DSM_10642_uid40863	NC_013849	chromosome		arCOG10980	1		S		Uncharacterized conserved protein	0.0001	COG4585	Signal transduction histidine kinase
288930732	hypothetical protein Ferp_0338	No	0		294494	294652	-		Ferroglobus_placidus_DSM_10642_uid40863	NC_013849	chromosome										
288930733	radical SAM protein	No	0		294908	295915	-		Ferroglobus_placidus_DSM_10642_uid40863	NC_013849	chromosome		arCOG00938	12	(-)	R		Radical SAM superfamily enzyme	9E-25	COG2896	Molybdenum cofactor biosynthesis enzyme
288930734	hypothetical protein Ferp_0340	Yes	0		295922	296503	-		Ferroglobus_placidus_DSM_10642_uid40863	NC_013849	chromosome		arCOG10329	1		S		Uncharacterized conserved protein			
===								9	Ferroglobus_placidus_DSM_10642_uid40863	NC_013849	chromosome										
288930797	hypothetical protein Ferp_0405	No	0		352279	352440	+		Ferroglobus_placidus_DSM_10642_uid40863	NC_013849	chromosome										
288930798	hypothetical protein Ferp_0406	No	0		352461	353351	-		Ferroglobus_placidus_DSM_10642_uid40863	NC_013849	chromosome										
288930799	SMC domain protein	No	0		353332	355245	-		Ferroglobus_placidus_DSM_10642_uid40863	NC_013849	chromosome		arCOG03235	4	(-)	L		Predicted ATP-dependent endonuclease of the (	4E-26	COG3593	Predicted ATP-dependent endonuclease of
288930800	hypothetical protein Ferp_0408	No	0		355594	355944	+		Ferroglobus_placidus_DSM_10642_uid40863	NC_013849	chromosome		arCOG10907	1		S		Uncharacterized conserved protein			
288930801	hypothetical protein Ferp_0409	No	6		356164	356691	+		Ferroglobus_placidus_DSM_10642_uid40863	NC_013849	chromosome		arCOG11170	1		S		Uncharacterized conserved protein			
288930802	hypothetical protein Ferp_0410	No	0		356714	357658	-		Ferroglobus_placidus_DSM_10642_uid40863	NC_013849	chromosome										
288930803	extracellular solute-binding protein family 1	No	0		357826	357975	+		Ferroglobus_placidus_DSM_10642_uid40863	NC_013849	chromosome										
288930804	hypothetical protein Ferp_0412	No	0		357972	358196	+		Ferroglobus_placidus_DSM_10642_uid40863	NC_013849	chromosome										
288930805	hypothetical protein Ferp_0413	No	0		358196	359149	+		Ferroglobus_placidus_DSM_10642_uid40863	NC_013849	chromosome		arCOG04792	9	(-)	L		Transposase			
===								5	Ferroglobus_placidus_DSM_10642_uid40863	NC_013849	chromosome										
288930807	hypothetical protein Ferp_0415	No	0		360381	360959	-		Ferroglobus_placidus_DSM_10642_uid40863	NC_013849	chromosome										
288930808	cytoplasmic protein	No	0		361055	361207	+		Ferroglobus_placidus_DSM_10642_uid40863	NC_013849	chromosome		arCOG08100	1	(-)	S		Uncharacterized conserved protein			
288930809	hypothetical protein Ferp_0417	No	1		361241	361762	-		Ferroglobus_placidus_DSM_10642_uid40863	NC_013849	chromosome										
288930810	hypothetical protein Ferp_0418	No	0		361778	361960	-		Ferroglobus_placidus_DSM_10642_uid40863	NC_013849	chromosome										
288930811	hypothetical protein Ferp_0419	No	0		362200	363915	+		Ferroglobus_placidus_DSM_10642_uid40863	NC_013849	chromosome										
===								10	Ferroglobus_placidus_DSM_10642_uid40863	NC_013849	chromosome										
288930817	integrase	No	0	YES	370693	371904	+		Ferroglobus_placidus_DSM_10642_uid40863	NC_013849	chromosome		arCOG01242	14	(-)	L	XerC	Integrase	2E-12	pfam00589	Phage integrase family. Members of this
288930818	hypothetical protein Ferp_0427	No	0	YES	371945	372160	-		Ferroglobus_placidus_DSM_10642_uid40863	NC_013849	chromosome										
288930819	hypothetical protein Ferp_0428	No	0	YES	372259	373179	+		Ferroglobus_placidus_DSM_10642_uid40863	NC_013849	chromosome										
288930820	hypothetical protein Ferp_0429	No	0	YES	373200	373604	+		Ferroglobus_placidus_DSM_10642_uid40863	NC_013849	chromosome										
288930821	peptidase M10A and M12B matrixin and adama	Yes	1	YES	374026	374724	+		Ferroglobus_placidus_DSM_10642_uid40863	NC_013849	chromosome										
288930822	hypothetical protein Ferp_0432	No	0	YES	374807	375364	+		Ferroglobus_placidus_DSM_10642_uid40863	NC_013849	chromosome										
288930823	hypothetical protein Ferp_0433	No	1	YES	375484	375699	+		Ferroglobus_placidus_DSM_10642_uid40863	NC_013849	chromosome										
288930824	binding-protein-dependent transporters inner m	No	2	YES	375696	375884	+		Ferroglobus_placidus_DSM_10642_uid40863	NC_013849	chromosome										
288930825	hypothetical protein Ferp_0435	No	0	YES	375886	376671	+		Ferroglobus_placidus_DSM_10642_uid40863	NC_013849	chromosome										
288930826	hypothetical protein Ferp_0436	No	0	YES	376676	378376	+		Ferroglobus_placidus_DSM_10642_uid40863	NC_013849	chromosome	arCOG03181,a	arCOG03181	1	(-)	K	MarR	Transcriptional regulator, MarR family	2E-10	pfam12846	AAA-like domain. This family of domains
===								8	Ferroglobus_placidus_DSM_10642_uid40863	NC_013849	chromosome										
288930846	hypothetical protein Ferp_0456	No	0		395838	395978	-		Ferroglobus_placidus_DSM_10642_uid40863	NC_013849	chromosome										
288930847	hypothetical protein Ferp_0458	No	1		396953	397441	-		Ferroglobus_placidus_DSM_10642_uid40863	NC_013849	chromosome		arCOG02416	3	(-)	N		Predicted flagellin FlaG			
288930848	hypothetical protein Ferp_0459	Yes	3		397523	397825	+		Ferroglobus_placidus_DSM_10642_uid40863	NC_013849	chromosome										
288930849	hypothetical protein Ferp_0460	No	0		397840	397950	+		Ferroglobus_placidus_DSM_10642_uid40863	NC_013849	chromosome										
288930850	hypothetical protein Ferp_0461	No	0		397947	398981	+		Ferroglobus_placidus_DSM_10642_uid40863	NC_013849	chromosome										
288930851	hypothetical protein Ferp_0462	No	0		398978	399187	+		Ferroglobus_placidus_DSM_10642_uid40863	NC_013849	chromosome										
288930852	hypothetical protein Ferp_0463	No	0		399194	399547	+		Ferroglobus_placidus_DSM_10642_uid40863	NC_013849	chromosome										
288930853	thioredoxin	No	0		399580	399945	+		Ferroglobus_placidus_DSM_10642_uid40863	NC_013849	chromosome		arCOG01972	5	(-)	O	TrxA	Thiol-disulfide isomerase or thioredoxin	0.00000002	COG0526	Thiol-disulfide isomerase and thioredoxi
===								5	Ferroglobus_placidus_DSM_10642_uid40863	NC_013849	chromosome										
288930860	hypothetical protein Ferp_0471	No	3		407944	408255	+		Ferroglobus_placidus_DSM_10642_uid40863	NC_013849	chromosome		arCOG10228	2		S		Uncharacterized conserved protein			
288930861	hypothetical protein Ferp_0472	Yes	0		408579	409004	-		Ferroglobus_placidus_DSM_10642_uid40863	NC_013849	chromosome								0.00000002	COG3042	Putative hemolysin
288930862	nuclease (SNase domain protein)	Yes	0		409085	409888	-		Ferroglobus_placidus_DSM_10642_uid40863	NC_013849	chromosome		arCOG03192	6	(-)	L		Micrococcal nuclease (thermonuclease)	1E-13	COG1525	Micrococcal nuclease (thermonuclease) ho
288930863	hypothetical protein Ferp_0474	No	0		409997	410308	-		Ferroglobus_placidus_DSM_10642_uid40863	NC_013849	chromosome		arCOG11171	1		S		Uncharacterized conserved protein			
288930864	hypothetical protein Ferp_0475	No	0		410562	410789	-		Ferroglobus_placidus_DSM_10642_uid40863	NC_013849	chromosome										
===								8	Ferroglobus_placidus_DSM_10642_uid40863	NC_013849	chromosome										
288930866	hypothetical protein Ferp_0477	No	0		411883	412440	+		Ferroglobus_placidus_DSM_10642_uid40863	NC_013849	chromosome		arCOG03112	1	(-)	S		Fic family protein			
288930867	hypothetical protein Ferp_0478	No	0		413385	414170	+		Ferroglobus_placidus_DSM_10642_uid40863	NC_013849	chromosome								0.0000002	COG0419	ATPase involved in DNA repair
288930868	hypothetical protein Ferp_0479	No	0		414226	414666	+		Ferroglobus_placidus_DSM_10642_uid40863	NC_013849	chromosome										
288930869	hypothetical protein Ferp_0480	Yes	5		414816	415319	+		Ferroglobus_placidus_DSM_10642_uid40863	NC_013849	chromosome		arCOG11284	1		S		Uncharacterized conserved protein			
288930870	hypothetical protein Ferp_0481	No	0		416352	418448	+		Ferroglobus_placidus_DSM_10642_uid40863	NC_013849	chromosome										
288930871	hypothetical protein Ferp_0482	No	0		418493	419176	-		Ferroglobus_placidus_DSM_10642_uid40863	NC_013849	chromosome										
288930872	hypothetical protein Ferp_0483	No	0		419180	419275	-		Ferroglobus_placidus_DSM_10642_uid40863	NC_013849	chromosome										
288930873	hypothetical protein Ferp_0484	No	3		419288	419677	-		Ferroglobus_placidus_DSM_10642_uid40863	NC_013849	chromosome		arCOG06161	1	(-)	S		Uncharacterized conserved protein			
===								9	Ferroglobus_placidus_DSM_10642_uid40863	NC_013849	chromosome										
288930876	hypothetical protein Ferp_0487	No	1		426851	428071	-		Ferroglobus_placidus_DSM_10642_uid40863	NC_013849	chromosome		arCOG03450	1	(-)	R		Predicted surface protease of transglutaminase family			
288930877	hypothetical protein Ferp_0488	Yes	0		428101	428688	-		Ferroglobus_placidus_DSM_10642_uid40863	NC_013849	chromosome										
288930878	hypothetical protein Ferp_0489	No	11		428666	430453	-		Ferroglobus_placidus_DSM_10642_uid40863	NC_013849	chromosome								0.00007	pfam04535	Domain of unknown function (DUF588). Thi
288930879	hypothetical protein Ferp_0490	No	0		430630	430953	-		Ferroglobus_placidus_DSM_10642_uid40863	NC_013849	chromosome										
288930880	hypothetical protein Ferp_0491	No	0		431071	431346	+		Ferroglobus_placidus_DSM_10642_uid40863	NC_013849	chromosome										
288930881	methyltransferase	No	0		432639	432803	-		Ferroglobus_placidus_DSM_10642_uid40863	NC_013849	chromosome										
288930882	hypothetical protein Ferp_0493	Yes	1		434660	436021	+		Ferroglobus_placidus_DSM_10642_uid40863	NC_013849	chromosome		arCOG00368	3	(-)	L	SbcC	ATPase involved in DNA repair, SbcC	5E-13	COG1196	Chromosome segregation ATPases
288930883	hypothetical protein Ferp_0494	Yes	1	YES	436078	436362	+		Ferroglobus_placidus_DSM_10642_uid40863	NC_013849	chromosome										
288930884	hypothetical protein Ferp_0495	No	4	YES	436790	437338	+		Ferroglobus_placidus_DSM_10642_uid40863	NC_013849	chromosome										
===								16	Ferroglobus_placidus_DSM_10642_uid40863	NC_013849	chromosome										
288930886	hypothetical protein Ferp_0497	No	0	YES	438595	440031	+		Ferroglobus_placidus_DSM_10642_uid40863	NC_013849	chromosome		arCOG01055	9	(-)	K		Predicted transcriptional regulator			
288930887	hypothetical protein Ferp_0498	No	0	YES	440182	440433	+		Ferroglobus_placidus_DSM_10642_uid40863	NC_013849	chromosome										
288930888	hypothetical protein Ferp_0499	No	0	YES	440438	440758	+		Ferroglobus_placidus_DSM_10642_uid40863	NC_013849	chromosome										
288930889	hypothetical protein Ferp_0500	No	1	YES	442018	442179	+		Ferroglobus_placidus_DSM_10642_uid40863	NC_013849	chromosome										
288930890	hypothetical protein Ferp_0501	No	0	YES	442330	442737	+		Ferroglobus_placidus_DSM_10642_uid40863	NC_013849	chromosome										
288930891	hypothetical protein Ferp_0502	No	0	YES	442734	443252	+		Ferroglobus_placidus_DSM_10642_uid40863	NC_013849	chromosome										
288930892	hypothetical protein Ferp_0503	No	0	YES	443307	443972	+		Ferroglobus_placidus_DSM_10642_uid40863	NC_013849	chromosome										
288930893	hypothetical protein Ferp_0504	No	0	YES	444028	444696	+		Ferroglobus_placidus_DSM_10642_uid40863	NC_013849	chromosome										
288930894	hypothetical protein Ferp_0505	No	0	YES	444745	445443	+		Ferroglobus_placidus_DSM_10642_uid40863	NC_013849	chromosome										
288930895	hypothetical protein Ferp_0506	No	0	YES	445445	445972	-		Ferroglobus_placidus_DSM_10642_uid40863	NC_013849	chromosome										
288930896	hypothetical protein Ferp_0507	No	0	YES	446028	446603	-		Ferroglobus_placidus_DSM_10642_uid40863	NC_013849	chromosome										
288930897	hypothetical protein Ferp_0508	No	0	YES	446690	447010	+		Ferroglobus_placidus_DSM_10642_uid40863	NC_013849	chromosome										
288930898	CRISPR-associated protein, Csx3 family	No	0	YES	447064	447372	+		Ferroglobus_placidus_DSM_10642_uid40863	NC_013849	chromosome		arCOG07528	1	(-)	V		CRISPR-associated protein Csx3	3E-15	pfam09620	CRISPR-associated protein (Cas_csx3). Th
288930899	hypothetical protein Ferp_0510	No	0	YES	447546	448475	+		Ferroglobus_placidus_DSM_10642_uid40863	NC_013849	chromosome										
288930900	hypothetical protein Ferp_0511	No	0	YES	448518	449225	+		Ferroglobus_placidus_DSM_10642_uid40863	NC_013849	chromosome										
288930901	hypothetical protein Ferp_0512	No	2	YES	449236	449457	+		Ferroglobus_placidus_DSM_10642_uid40863	NC_013849	chromosome										
===								26	Ferroglobus_placidus_DSM_10642_uid40863	NC_013849	chromosome										
288930904	hypothetical protein Ferp_0515	No	0		452258	452698	+		Ferroglobus_placidus_DSM_10642_uid40863	NC_013849	chromosome										
288930905	hypothetical protein Ferp_0516	No	0		452713	453174	+		Ferroglobus_placidus_DSM_10642_uid40863	NC_013849	chromosome								0.00001	pfam02463	RecF/RecN/SMC N terminal domain. This do
288930906	hypothetical protein Ferp_0517	No	0		453234	453563	+		Ferroglobus_placidus_DSM_10642_uid40863	NC_013849	chromosome										
288930907	ATPase associated with various cellular activiti	No	0		453603	454871	+		Ferroglobus_placidus_DSM_10642_uid40863	NC_013849	chromosome		arCOG04159	1	(-)	R		MoxR-like ATPase			
288930908	hypothetical protein Ferp_0519	No	0		454904	455317	+		Ferroglobus_placidus_DSM_10642_uid40863	NC_013849	chromosome										
288930909	hypothetical protein Ferp_0520	No	0		455340	455825	+		Ferroglobus_placidus_DSM_10642_uid40863	NC_013849	chromosome										
288930910	hypothetical protein Ferp_0521	No	0		455919	457325	+		Ferroglobus_placidus_DSM_10642_uid40863	NC_013849	chromosome		arCOG01334	4	(-)	O	HtpX	Zn-dependent protease with chaperone function	4E-20	COG3864	Uncharacterized protein conserved in bac
288930911	hypothetical protein Ferp_0522	No	0		457362	457886	+		Ferroglobus_placidus_DSM_10642_uid40863	NC_013849	chromosome										
288930912	hypothetical protein Ferp_0523	No	0		457904	458962	+		Ferroglobus_placidus_DSM_10642_uid40863	NC_013849	chromosome										
288930913	hypothetical protein Ferp_0524	No	0		459031	459510	+		Ferroglobus_placidus_DSM_10642_uid40863	NC_013849	chromosome										
288930914	hypothetical protein Ferp_0525	No	0		459507	459629	+		Ferroglobus_placidus_DSM_10642_uid40863	NC_013849	chromosome										
288930915	hypothetical protein Ferp_0526	No	3		459741	460040	+		Ferroglobus_placidus_DSM_10642_uid40863	NC_013849	chromosome										
288930916	hypothetical protein Ferp_0527	No	0		460085	460537	+		Ferroglobus_placidus_DSM_10642_uid40863	NC_013849	chromosome										
288930917	hypothetical protein Ferp_0528	No	0		460552	460806	+		Ferroglobus_placidus_DSM_10642_uid40863	NC_013849	chromosome										
288930918	hypothetical protein Ferp_0529	No	0		460862	461245	+		Ferroglobus_placidus_DSM_10642_uid40863	NC_013849	chromosome										
288930919	hypothetical protein Ferp_0530	No	0		461287	461541	+		Ferroglobus_placidus_DSM_10642_uid40863	NC_013849	chromosome										
288930920	hypothetical protein Ferp_0531	No	0		461613	461858	+		Ferroglobus_placidus_DSM_10642_uid40863	NC_013849	chromosome										
288930921	hypothetical protein Ferp_0532	No	0		461893	462621	+		Ferroglobus_placidus_DSM_10642_uid40863	NC_013849	chromosome		arCOG01917	12	(-)	S		Zn-ribbon domain containing protein			
288930922	hypothetical protein Ferp_0533	No	0		462701	463303	+		Ferroglobus_placidus_DSM_10642_uid40863	NC_013849	chromosome										
288930923	hypothetical protein Ferp_0534	No	0		463339	464586	+		Ferroglobus_placidus_DSM_10642_uid40863	NC_013849	chromosome										
288930924	hypothetical protein Ferp_0535	No	0		464654	465298	+		Ferroglobus_placidus_DSM_10642_uid40863	NC_013849	chromosome										
288930925	hypothetical protein Ferp_0536	No	0		465323	465457	+		Ferroglobus_placidus_DSM_10642_uid40863	NC_013849	chromosome										
288930926	hypothetical protein Ferp_0537	No	0		465445	465894	+		Ferroglobus_placidus_DSM_10642_uid40863	NC_013849	chromosome										
288930927	hypothetical protein Ferp_0538	No	0		466091	466378	+		Ferroglobus_placidus_DSM_10642_uid40863	NC_013849	chromosome										
288930928	propeptide PepSY amd peptidase M4	Yes	1		466533	467024	+		Ferroglobus_placidus_DSM_10642_uid40863	NC_013849	chromosome		arCOG10270	1		S		Uncharacterized conserved protein	0.00000005	pfam03413	Peptidase propeptide and YPEB domain. Th
288930929	hypothetical protein Ferp_0540	No	3		467102	467371	+		Ferroglobus_placidus_DSM_10642_uid40863	NC_013849	chromosome		arCOG08000	1	(-)	S		Membrane protein			
===								10	Ferroglobus_placidus_DSM_10642_uid40863	NC_013849	chromosome										
288930933	hypothetical protein Ferp_0545	No	0		470938	471759	+		Ferroglobus_placidus_DSM_10642_uid40863	NC_013849	chromosome										
288930934	haloacid dehalogenase type 3	No	0		471830	472192	+		Ferroglobus_placidus_DSM_10642_uid40863	NC_013849	chromosome		arCOG04221	2	(-)	G	NagD	Phosphatase of the HAD superfamily			
288930935	hypothetical protein Ferp_0547	No	0		472240	472719	+		Ferroglobus_placidus_DSM_10642_uid40863	NC_013849	chromosome		arCOG10345	1		S		Uncharacterized conserved protein			
288930936	hypothetical protein Ferp_0548	No	0		472721	472858	+		Ferroglobus_placidus_DSM_10642_uid40863	NC_013849	chromosome										
288930937	hypothetical protein Ferp_0549	No	0		472862	473161	+		Ferroglobus_placidus_DSM_10642_uid40863	NC_013849	chromosome										
288930938	hypothetical protein Ferp_0550	No	0		473259	473519	+		Ferroglobus_placidus_DSM_10642_uid40863	NC_013849	chromosome										
288930939	hypothetical protein Ferp_0551	No	0		473564	473701	+		Ferroglobus_placidus_DSM_10642_uid40863	NC_013849	chromosome										
288930940	hypothetical protein Ferp_0552	No	0		473764	474738	+		Ferroglobus_placidus_DSM_10642_uid40863	NC_013849	chromosome										
288930941	hypothetical protein Ferp_0553	No	0		474814	475449	+		Ferroglobus_placidus_DSM_10642_uid40863	NC_013849	chromosome										
288930942	hypothetical protein Ferp_0554	Yes	1		475522	476652	-		Ferroglobus_placidus_DSM_10642_uid40863	NC_013849	chromosome		arCOG02416	3	(-)	N		Predicted flagellin FlaG	0.000006	pfam07174	Fibronectin-attachment protein (FAP). Th
===								9	Ferroglobus_placidus_DSM_10642_uid40863	NC_013849	chromosome										
288930944	zinc finger C2H2-type domain protein	No	0		477838	478194	+		Ferroglobus_placidus_DSM_10642_uid40863	NC_013849	chromosome		arCOG07930	3	(-)	S		Zinc finger C2H2-type			
288930945	hypothetical protein Ferp_0557	No	0		478235	478663	+		Ferroglobus_placidus_DSM_10642_uid40863	NC_013849	chromosome										
288930946	hypothetical protein Ferp_0558	No	0		478691	479608	+		Ferroglobus_placidus_DSM_10642_uid40863	NC_013849	chromosome										
288930947	hypothetical protein Ferp_0559	No	0		479646	480101	+		Ferroglobus_placidus_DSM_10642_uid40863	NC_013849	chromosome										
288930948	hypothetical protein Ferp_0560	No	0		480145	480594	+		Ferroglobus_placidus_DSM_10642_uid40863	NC_013849	chromosome										
288930949	hypothetical protein Ferp_0561	No	0		480598	481383	+		Ferroglobus_placidus_DSM_10642_uid40863	NC_013849	chromosome										
288930950	hypothetical protein Ferp_0562	No	0		481376	481567	+		Ferroglobus_placidus_DSM_10642_uid40863	NC_013849	chromosome										
288930951	hypothetical protein Ferp_0563	No	0		481595	481948	+		Ferroglobus_placidus_DSM_10642_uid40863	NC_013849	chromosome										
288930952	hypothetical protein Ferp_0564	No	0		481953	482231	+		Ferroglobus_placidus_DSM_10642_uid40863	NC_013849	chromosome		arCOG03898	7	(-)	V	McrA	Restriction endonuclease, McrA/HNH family	0.00008	pfam01844	HNH endonuclease.
===								8	Ferroglobus_placidus_DSM_10642_uid40863	NC_013849	chromosome										
288930954	hypothetical protein Ferp_0566	No	0		483270	483797	+		Ferroglobus_placidus_DSM_10642_uid40863	NC_013849	chromosome										
288930955	ribonuclease H	No	0		483794	484426	+		Ferroglobus_placidus_DSM_10642_uid40863	NC_013849	chromosome		arCOG02942	1	(-)	L	RnhA	Ribonuclease HI	1E-11	pfam13456	Reverse transcriptase-like. This domain
288930956	hypothetical protein Ferp_0568	No	0		484484	484984	+		Ferroglobus_placidus_DSM_10642_uid40863	NC_013849	chromosome										
288930957	hypothetical protein Ferp_0569	No	0		485011	485337	+		Ferroglobus_placidus_DSM_10642_uid40863	NC_013849	chromosome										

288930958	hypothetical protein Ferp_0570	No	0			485458	486681	+		Ferroglobus_placidus_DSM_10642_uid40863	NC_013849	chromosome									
288930959	hypothetical protein Ferp_0571	No	0			486721	487701	+		Ferroglobus_placidus_DSM_10642_uid40863	NC_013849	chromosome									
288930960	hypothetical protein Ferp_0572	No	2			487756	488460	+		Ferroglobus_placidus_DSM_10642_uid40863	NC_013849	chromosome		arCOG07030	1 (-)	S		Uncharacterized conserved protein			
288930961	hypothetical protein Ferp_0573	No	0			488491	488976	+		Ferroglobus_placidus_DSM_10642_uid40863	NC_013849	chromosome									
===									9	Ferroglobus_placidus_DSM_10642_uid40863	NC_013849	chromosome									
288931019	hypothetical protein Ferp_0633	Yes	0			545159	547570	+		Ferroglobus_placidus_DSM_10642_uid40863	NC_013849	chromosome		arCOG07534	1 (-)	M		Halomucin			
288931020	hypothetical protein Ferp_0634	No	3			547570	547851	+		Ferroglobus_placidus_DSM_10642_uid40863	NC_013849	chromosome									
288931021	hypothetical protein Ferp_0635	No	0			547975	548565	+		Ferroglobus_placidus_DSM_10642_uid40863	NC_013849	chromosome		arCOG10887	3	S		Uncharacterized conserved protein			
288931022	hypothetical protein Ferp_0636	No	5			548571	549149	+		Ferroglobus_placidus_DSM_10642_uid40863	NC_013849	chromosome	clust_177								
288931023	hypothetical protein Ferp_0637	No	0			549190	549471	+		Ferroglobus_placidus_DSM_10642_uid40863	NC_013849	chromosome									
288931024	hypothetical protein Ferp_0638	No	0			549432	549554	+		Ferroglobus_placidus_DSM_10642_uid40863	NC_013849	chromosome		arCOG10887	3	S		Uncharacterized conserved protein			
288931025	hypothetical protein Ferp_0639	No	5			549551	550135	+		Ferroglobus_placidus_DSM_10642_uid40863	NC_013849	chromosome	clust_177								
288931026	hypothetical protein Ferp_0640	No	0			550205	550387	+		Ferroglobus_placidus_DSM_10642_uid40863	NC_013849	chromosome		arCOG10887	3	S		Uncharacterized conserved protein			
288931027	hypothetical protein Ferp_0641	No	6			550494	551171	+		Ferroglobus_placidus_DSM_10642_uid40863	NC_013849	chromosome		arCOG06126	1 (-)	R		Uncharacterized membrane protein, possible Na	3E-69	pfam04474	Protein of unknown function (DUF554). Fa
===									13	Ferroglobus_placidus_DSM_10642_uid40863	NC_013849	chromosome									
288931051	hypothetical protein Ferp_0665	No	6			571843	572619	-		Ferroglobus_placidus_DSM_10642_uid40863	NC_013849	chromosome									
288931052	ISA1214-5 transposase	No	0			572707	573675	-		Ferroglobus_placidus_DSM_10642_uid40863	NC_013849	chromosome	arCOG07732,a	arCOG07732	1 (-)	L		Transposase	2E-178	COG5558	Transposase
288931053	hypothetical protein Ferp_0667	No	0			573712	573885	+		Ferroglobus_placidus_DSM_10642_uid40863	NC_013849	chromosome		arCOG03883	2	S	ISA1214	Putative transposon-encoded protein	6E-27	COG3466	Putative transposon-encoded protein
288931054	hypothetical protein Ferp_0668	Yes	1			573961	574707	+		Ferroglobus_placidus_DSM_10642_uid40863	NC_013849	chromosome		arCOG10904	5	S		Uncharacterized conserved protein			
288931055	hypothetical protein Ferp_0669	Yes	1			574808	575566	+		Ferroglobus_placidus_DSM_10642_uid40863	NC_013849	chromosome		arCOG10904	5	S		Uncharacterized conserved protein			
288931056	hypothetical protein Ferp_0670	Yes	0			575658	580856	+		Ferroglobus_placidus_DSM_10642_uid40863	NC_013849	chromosome		arCOG08705	1 (-)	R		Large extracellular alpha-helical protein			
288931057	hypothetical protein Ferp_0671	Yes	0			580879	582003	-		Ferroglobus_placidus_DSM_10642_uid40863	NC_013849	chromosome									
288931058	hypothetical protein Ferp_0672	Yes	1			582111	587030	+		Ferroglobus_placidus_DSM_10642_uid40863	NC_013849	chromosome	arCOG09418,a	arCOG09418	1 (-)	C		Multiheme cytochrome			
288931059	hypothetical protein Ferp_0673	No	1			587059	587787	+		Ferroglobus_placidus_DSM_10642_uid40863	NC_013849	chromosome		arCOG10904	5	S		Uncharacterized conserved protein			
288931060	hypothetical protein Ferp_0674	Yes	1			587778	588404	+		Ferroglobus_placidus_DSM_10642_uid40863	NC_013849	chromosome		arCOG10904	5	S		Uncharacterized conserved protein			
288931061	hypothetical protein Ferp_0675	Yes	1			588401	589555	+		Ferroglobus_placidus_DSM_10642_uid40863	NC_013849	chromosome	arCOG10904,a	arCOG10904	5	S		Uncharacterized conserved protein			
288931062	carbohydrate-binding CenC domain protein	Yes	0			589612	595206	+		Ferroglobus_placidus_DSM_10642_uid40863	NC_013849	chromosome	arCOG10826,a	arCOG10826	1 (-)	C		Cytochrome c554 family protein			
288931063	hypothetical protein Ferp_0677	No	0			595181	595678	+		Ferroglobus_placidus_DSM_10642_uid40863	NC_013849	chromosome									
===									6	Ferroglobus_placidus_DSM_10642_uid40863	NC_013849	chromosome									
288931486	hypothetical protein Ferp_1112	Yes	0	YES		969704	970510	-		Ferroglobus_placidus_DSM_10642_uid40863	NC_013849	chromosome		arCOG07630	1	S		Uncharacterized conserved protein			
288931487	hypothetical protein Ferp_1113	No	0	YES		970783	971250	-		Ferroglobus_placidus_DSM_10642_uid40863	NC_013849	chromosome									
288931488	hypothetical protein Ferp_1114	No	0	YES		971265	971465	-		Ferroglobus_placidus_DSM_10642_uid40863	NC_013849	chromosome									
288931489	hypothetical protein Ferp_1115	No	0	YES		972235	972687	-		Ferroglobus_placidus_DSM_10642_uid40863	NC_013849	chromosome		arCOG09059	1 (-)	V		PIN domain containing protein			
288931490	AbrB family transcriptional regulator	No	0	YES		972687	972959	-		Ferroglobus_placidus_DSM_10642_uid40863	NC_013849	chromosome							0.00002	COG4013	Uncharacterized protein conserved in arc
288931491	hypothetical protein Ferp_1117	No	0	YES		973502	974056	-		Ferroglobus_placidus_DSM_10642_uid40863	NC_013849	chromosome									
===									6	Ferroglobus_placidus_DSM_10642_uid40863	NC_013849	chromosome									
288931565	hypothetical protein Ferp_1192	No	0			1031421	1032095	+		Ferroglobus_placidus_DSM_10642_uid40863	NC_013849	chromosome		arCOG09808	2	S		Uncharacterized conserved protein			
288931566	hypothetical protein Ferp_1193	No	0			1032097	1032552	+		Ferroglobus_placidus_DSM_10642_uid40863	NC_013849	chromosome									
288931567	hypothetical protein Ferp_1194	No	0			1032579	1032926	+		Ferroglobus_placidus_DSM_10642_uid40863	NC_013849	chromosome									
288931568	transposase IS4 family protein	No	0			1032919	1034193	-		Ferroglobus_placidus_DSM_10642_uid40863	NC_013849	chromosome		arCOG07671	1 (-)	R		Transposase related protein			
288931569	hypothetical protein Ferp_1196	No	0			1034472	1035182	+		Ferroglobus_placidus_DSM_10642_uid40863	NC_013849	chromosome									
288931570	hypothetical protein Ferp_1197	No	0			1035179	1035313	+		Ferroglobus_placidus_DSM_10642_uid40863	NC_013849	chromosome									
===									21	Ferroglobus_placidus_DSM_10642_uid40863	NC_013849	chromosome									
288931574	peptidase domain protein	Yes	0	YES		1039003	1039368	-		Ferroglobus_placidus_DSM_10642_uid40863	NC_013849	chromosome		arCOG04987	6 (-)	S		Uncharacterized conserved protein			
288931575	hypothetical protein Ferp_1202	Yes	1	YES		1039713	1040618	+		Ferroglobus_placidus_DSM_10642_uid40863	NC_013849	chromosome									
288931576	hypothetical protein Ferp_1203	No	1	YES		1040633	1040737	+		Ferroglobus_placidus_DSM_10642_uid40863	NC_013849	chromosome									
288931577	hypothetical protein Ferp_1204	No	0	YES		1040744	1040944	+		Ferroglobus_placidus_DSM_10642_uid40863	NC_013849	chromosome									
288931578	hypothetical protein Ferp_1205	No	0	YES		1041008	1041112	+		Ferroglobus_placidus_DSM_10642_uid40863	NC_013849	chromosome									
288931579	hypothetical protein Ferp_1206	Yes	1	YES		1041303	1042199	+		Ferroglobus_placidus_DSM_10642_uid40863	NC_013849	chromosome									
288931580	hypothetical protein Ferp_1207	No	3	YES		1042242	1042502	+		Ferroglobus_placidus_DSM_10642_uid40863	NC_013849	chromosome		arCOG11172	1	S		Uncharacterized conserved protein			
288931581	phospholipase C zinc-binding protein	No	1	YES		1042549	1043508	+		Ferroglobus_placidus_DSM_10642_uid40863	NC_013849	chromosome		arCOG04005	2 (-)	I		Zinc dependent phospholipase C			
288931582	hypothetical protein Ferp_1209	No	6	YES		1043513	1044076	+		Ferroglobus_placidus_DSM_10642_uid40863	NC_013849	chromosome									
288931583	hypothetical protein Ferp_1210	Yes	0	YES		1044302	1045315	+		Ferroglobus_placidus_DSM_10642_uid40863	NC_013849	chromosome		arCOG10238	4	S		Uncharacterized conserved protein			
288931584	hypothetical protein Ferp_1211	Yes	0	YES		1045291	1046175	+		Ferroglobus_placidus_DSM_10642_uid40863	NC_013849	chromosome									
288931585	hypothetical protein Ferp_1212	No	3	YES		1046195	1046914	+		Ferroglobus_placidus_DSM_10642_uid40863	NC_013849	chromosome									
288931586	hypothetical protein Ferp_1213	No	0	YES		1046954	1047826	+		Ferroglobus_placidus_DSM_10642_uid40863	NC_013849	chromosome									
288931587	hypothetical protein Ferp_1216	Yes	1	YES		1048907	1049644	+		Ferroglobus_placidus_DSM_10642_uid40863	NC_013849	chromosome									
288931588	hypothetical protein Ferp_1217	Yes	1	YES		1049649	1050287	+		Ferroglobus_placidus_DSM_10642_uid40863	NC_013849	chromosome		arCOG10988	1	S		Uncharacterized conserved protein			
288931589	hypothetical protein Ferp_1218	Yes	1	YES		1050315	1051454	+		Ferroglobus_placidus_DSM_10642_uid40863	NC_013849	chromosome		arCOG04005	2 (-)	I		Zinc dependent phospholipase C			
288931590	hypothetical protein Ferp_1219	No	3	YES		1051456	1051809	+		Ferroglobus_placidus_DSM_10642_uid40863	NC_013849	chromosome									
288931591	RND family efflux transporter MFP subunit	Yes	1	YES		1051814	1052041	+		Ferroglobus_placidus_DSM_10642_uid40863	NC_013849	chromosome									
288931592	hypothetical protein Ferp_1221	No	0	YES		1052029	1052652	+		Ferroglobus_placidus_DSM_10642_uid40863	NC_013849	chromosome									
288931593	double-stranded RNA-binding protein	No	0	YES		1052894	1053139	+		Ferroglobus_placidus_DSM_10642_uid40863	NC_013849	chromosome									
288931594	hypothetical protein Ferp_1224	No	0	YES		1053967	1054188	+		Ferroglobus_placidus_DSM_10642_uid40863	NC_013849	chromosome		arCOG02681	1 (-)	V		Predicted antitoxin, copG family	4E-21	COG1753	Predicted antotoxin, copG family
===									6	Ferroglobus_placidus_DSM_10642_uid40863	NC_013849	chromosome									
288931837	L-carnitine dehydratase/bile acid-inducible prot	No	0			1259310	1260740	+		Ferroglobus_placidus_DSM_10642_uid40863	NC_013849	chromosome		arCOG02304	1 (-)	C	Mct/CaiB	Succinyl-CoA:mesaconate CoA-transferase or pro	9E-37	COG1804	Predicted acyl-CoA transferases/carnitin
288931838	hypothetical protein Ferp_1472	No	0			1260754	1261254	+		Ferroglobus_placidus_DSM_10642_uid40863	NC_013849	chromosome									
288931839	hypothetical protein Ferp_1473	No	0			1261260	1261520	+		Ferroglobus_placidus_DSM_10642_uid40863	NC_013849	chromosome									
288931840	hypothetical protein Ferp_1474	No	0			1261554	1261682	+		Ferroglobus_placidus_DSM_10642_uid40863	NC_013849	chromosome									
288931841	hemolysin B	No	0			1261672	1261926	+		Ferroglobus_placidus_DSM_10642_uid40863	NC_013849	chromosome									
288931842	hypothetical protein Ferp_1476	No	0			1261919	1263010	-		Ferroglobus_placidus_DSM_10642_uid40863	NC_013849	chromosome		arCOG01015	1 (-)	J		Pseudouridylate synthase	4E-91	COG1258	Predicted pseudouridylate synthase
===									11	Ferroglobus_placidus_DSM_10642_uid40863	NC_013849	chromosome									
288932388	SSV1 integrase, C-fragment	No	0	YES		1758938	1759930	+		Ferroglobus_placidus_DSM_10642_uid40863	NC_013849	chromosome		arCOG01244	9 (-)	L		Intergrase/Recombinase	1E-21	COG4342	Uncharacterized protein conserved in arc
288932389	hypothetical protein Ferp_2039	No	2	YES		1760539	1760799	+		Ferroglobus_placidus_DSM_10642_uid40863	NC_013849	chromosome		arCOG11131	4	S		Uncharacterized conserved protein			
288932390	hypothetical protein Ferp_2040	No	0	YES		1760958	1761200	-		Ferroglobus_placidus_DSM_10642_uid40863	NC_013849	chromosome									
288932391	hypothetical protein Ferp_2041	No	0	YES		1761197	1761364	-		Ferroglobus_placidus_DSM_10642_uid40863	NC_013849	chromosome									
288932392	hypothetical protein Ferp_2043	No	0	YES		1761567	1761794	-		Ferroglobus_placidus_DSM_10642_uid40863	NC_013849	chromosome									
288932393	hypothetical protein Ferp_2044	No	0	YES		1761812	1762018	-		Ferroglobus_placidus_DSM_10642_uid40863	NC_013849	chromosome		arCOG11281	2	S		Uncharacterized conserved protein			
288932394	hypothetical protein Ferp_2045	No	0	YES		1762015	1762236	-		Ferroglobus_placidus_DSM_10642_uid40863	NC_013849	chromosome									
288932395	hypothetical protein Ferp_2046	No	0	YES		1762243	1762473	-		Ferroglobus_placidus_DSM_10642_uid40863	NC_013849	chromosome	clust_410								
288932396	hypothetical protein Ferp_2047	No	0	YES		1762457	1762903	-		Ferroglobus_placidus_DSM_10642_uid40863	NC_013849	chromosome		arCOG10366	7	L		PD-(D/E)XK superfamily nuclease			
288932397	Bifunctional DNA primase/polymerase	No	0	YES		1762903	1765551	-		Ferroglobus_placidus_DSM_10642_uid40863	NC_013849	chromosome		arCOG08578	4 (-)	Y		RepA plasmid replication protein	0.00000001	pfam09250	Bifunctional DNA primase/polymerase, N-t
288932398	hypothetical protein Ferp_2049	No	0	YES		1765708	1765917	-		Ferroglobus_placidus_DSM_10642_uid40863	NC_013849	chromosome	clust_442								
===									5	Ferroglobus_placidus_DSM_10642_uid40863	NC_013849	chromosome									
288932636	hypothetical protein Ferp_2297	No	2	YES		1974186	1974668	+		Ferroglobus_placidus_DSM_10642_uid40863	NC_013849	chromosome									
288932637	hypothetical protein Ferp_2298	No	0	YES		1974642	1974941	-		Ferroglobus_placidus_DSM_10642_uid40863	NC_013849	chromosome									
288932638	pyrophosphatase	No	0	YES		1975121	1975423	-		Ferroglobus_placidus_DSM_10642_uid40863	NC_013849	chromosome		arCOG01084	1 (-)	R	MazG	Predicted pyrophosphatase	0.00000002	COG1694	Predicted pyrophosphatase
288932639	integrase catalytic region	No	0	YES		1975417	1975599	-		Ferroglobus_placidus_DSM_10642_uid40863	NC_013849	chromosome									
288932640	hypothetical protein Ferp_2301	No	1	YES		1975604	1975759	-		Ferroglobus_placidus_DSM_10642_uid40863	NC_013849	chromosome									
===									5	Fervidicoccus_fontis_Kam940_uid162201	NC_017461	chromosome									
385805052	hypothetical protein FFONT_0001	No	0			826	2625	-		Fervidicoccus_fontis_Kam940_uid162201	NC_017461	chromosome									
385805053	hypothetical protein FFONT_0002	No	0			2860	2985	-		Fervidicoccus_fontis_Kam940_uid162201	NC_017461	chromosome									
385805054	hypothetical protein FFONT_0004	No	0			4253	4381	-		Fervidicoccus_fontis_Kam940_uid162201	NC_017461	chromosome									
385805055	hypothetical protein FFONT_0005	No	4			4394	4834	+		Fervidicoccus_fontis_Kam940_uid162201	NC_017461	chromosome		arCOG05371	4 (-)	S		Uncharacterized conserved protein			
385805056	hypothetical protein FFONT_0006	No	0			5450	5614	-		Fervidicoccus_fontis_Kam940_uid162201	NC_017461	chromosome									
===									7	Fervidicoccus_fontis_Kam940_uid162201	NC_017461	chromosome									
385805066	hypothetical protein FFONT_0016	No	0			12690	12920	-		Fervidicoccus_fontis_Kam940_uid162201	NC_017461	chromosome		arCOG02217	2 (-)	V		RHH/CopG DNA binding protein			
385805067	hypothetical protein FFONT_0017	No	0			13427	13579	-		Fervidicoccus_fontis_Kam940_uid162201	NC_017461	chromosome									
385805068	hypothetical protein FFONT_0018	No	0			13642	13758	-		Fervidicoccus_fontis_Kam940_uid162201	NC_017461	chromosome									
385805069	hypothetical protein FFONT_0019	No	0			14018	14149	-		Fervidicoccus_fontis_Kam940_uid162201	NC_017461	chromosome									
385805070	hypothetical protein FFONT_0020	No	0			14175	14348	-		Fervidicoccus_fontis_Kam940_uid162201	NC_017461	chromosome									
385805071	hypothetical protein FFONT_0021	No	0			14581	15900	+		Fervidicoccus_fontis_Kam940_uid162201	NC_017461	chromosome		arCOG07237	1 (-)	V		Uncharacterized protein contains PIN domain			
385805072	hypothetical protein FFONT_0022	No	0			15912	16028	-		Fervidicoccus_fontis_Kam940_uid162201	NC_017461	chromosome									
===									8	Fervidicoccus_fontis_Kam940_uid162201	NC_017461	chromosome									
385805142	hypothetical protein FFONT_0095	No	1			85201	85686	+		Fervidicoccus_fontis_Kam940_uid162201	NC_017461	chromosome		arCOG04805	1 (-)	P	KdpC	K+-transporting ATPase, c chain	0.00002	COG2156	K+-transporting ATPase, c chain
385805143	hypothetical protein FFONT_0096	No	0			85803	85922	+		Fervidicoccus_fontis_Kam940_uid162201	NC_017461	chromosome									
385805144	hypothetical protein FFONT_0097	No	1			86019	87881	-		Fervidicoccus_fontis_Kam940_uid162201	NC_017461	chromosome									
385805145	hypothetical protein FFONT_0098	No	1			87884	88318	-		Fervidicoccus_fontis_Kam940_uid162201	NC_017461	chromosome									
385805146	hypothetical protein FFONT_0099	No	3			88315	88695	-		Fervidicoccus_fontis_Kam940_uid162201	NC_017461	chromosome									
385805147	hypothetical protein FFONT_0100	No	4			88850	89461	+		Fervidicoccus_fontis_Kam940_uid162201	NC_017461	chromosome									
385805148	putative TrbE protein	No	0			89770	91629	+		Fervidicoccus_fontis_Kam940_uid162201	NC_017461	chromosome	arCOG04035,a	arCOG04035	5 (-)	U	VirB4	Type IV secretory pathway, VirB4 component	3E-13	pfam12846	AAA-like domain. This family of domains
385805149	hypothetical protein FFONT_0102	No	2			91626	91865	+		Fervidicoccus_fontis_Kam940_uid162201	NC_017461	chromosome									
===									5	Fervidicoccus_fontis_Kam940_uid162201	NC_017461	chromosome									
385805278	hypothetical protein FFONT_0233	No	0			226446	226991	-		Fervidicoccus_fontis_Kam940_uid162201	NC_017461	chromosome									
385805279	hypothetical protein FFONT_0234	No	0			227068	227211	-		Fervidicoccus_fontis_Kam940_uid162201	NC_017461	chromosome									
385805280	tRNA pseudouridine synthase D	No	0			227398	228363	+		Fervidicoccus_fontis_Kam940_uid162201	NC_017461	chromosome		arCOG04252	4 (-)	J		tRNA pseudouridine synthase D	3E-11	pfam01142	tRNA pseudouridine synthase D (TruD). Tr
385805281	hypothetical protein FFONT_0236	No	0			228889	229041	+		Fervidicoccus_fontis_Kam940_uid162201	NC_017461	chromosome									
385805282	hypothetical protein FFONT_0237	No	6			229077	229592	-		Fervidicoccus_fontis_Kam940_uid162201	NC_017461	chromosome							0.0000008	pfam02293	AmiS/UreI family transporter. This famil
===									7	Fervidicoccus_fontis_Kam940_uid162201	NC_017461	chromosome									
385805413	hypothetical protein FFONT_0369	No	1			351661	352218	-		Fervidicoccus_fontis_Kam940_uid162201	NC_017461	chromosome									
385805414	hypothetical protein FFONT_0370	No	10			352667	353776	+		Fervidicoccus_fontis_Kam940_uid162201	NC_017461	chromosome		arCOG00131	1 (-)	G	ProP	Permease of the major facilitator superfamily			
385805415	hypothetical protein FFONT_0371	No	0			354062	354184	-		Fervidicoccus_fontis_Kam940_uid162201	NC_017461	chromosome									
385805416	hypothetical protein FFONT_0372	No	0			354173	354286	+		Fervidicoccus_fontis_Kam940_uid162201	NC_017461	chromosome									
385805417	hypothetical protein FFONT_0373	No	5			354283	355044	+		Fervidicoccus_fontis_Kam940_uid162201	NC_017461	chromosome									
385805418	hypothetical protein FFONT_0374	No	0			355186	355323	-		Fervidicoccus_fontis_Kam940_uid162201	NC_017461	chromosome									
385805419	binding-protein-dependent transport system in	No	12			355368	356954	-		Fervidicoccus_fontis_Kam940_uid162201	NC_017461	chromosome		arCOG00174	2 (-)	P		ABC-type anion transport system, duplicated per	2E-67	COG4986	ABC-type anion transport system, duplica
===									5	Fervidicoccus_fontis_Kam940_uid162201	NC_017461	chromosome									
385805687	hypothetical protein FFONT_0643	No	0			604116	605306	+		Fervidicoccus_fontis_Kam940_uid162201	NC_017461	chromosome									
385805688	coenzyme F420-reducing hydrogenase subuni	No	0			605303	606616	+		Fervidicoccus_fontis_Kam940_uid162201	NC_017461	chromosome	arCOG01543,a	arCOG01543	2 (-)	C	NuoI	NADH dehydrogenase subunit I	1E-25	COG1035	Coenzyme F420-reducing hydrogenase, beta
385805689	hypothetical protein FFONT_0645	No	0			607005	607139	+		Fervidicoccus_fontis_Kam940_uid162201	NC_017461	chromosome									
385805690	hypothetical protein FFONT_0646	No	0			607994	608233	+		Fervidicoccus_fontis_Kam940_uid162201	NC_017461	chromosome									
385805691	hypothetical protein FFONT_0647	No	0			608241	608837	+		Fervidicoccus_fontis_Kam940_uid162201	NC_017461	chromosome									
===									5	Fervidicoccus_fontis_Kam940_uid162201	NC_017461	chromosome									
385805734	hypothetical protein FFONT_0692	No	0			645885	646226	-		Fervidicoccus_fontis_Kam940_uid162201	NC_017461	chromosome									
385805735	hypothetical protein FFONT_0693	No	2			646236	646832	-		Fervidicoccus_fontis_Kam940_uid162201	NC_017461	chromosome									
385805736	hypothetical protein FFONT_0694	No	1			646840	648732	-		Fervidicoccus_fontis_Kam940_uid162201	NC_017461	chromosome									
385805737	putative, serine protease	No	1			648793	649266	-		Fervidicoccus_fontis_Kam940_uid162201	NC_017461	chromosome		arCOG03871	6 (-)	N		Flagellar protein G	0.00008	COG3430	Uncharacterized protein conserved in arc
385805738	hypothetical protein FFONT_0696	No	1			649274	649420	-		Fervidicoccus_fontis_Kam940_uid162201	NC_017461	chromosome									
===									16	Halalkalicoccus_jeotgali_B3_uid50305	NC_014297	chromosome									
300709870	hypothetical protein HacjB3_02495	No	0			485536	486177	-		Halalkalicoccus_jeotgali_B3_uid50305	NC_014297	chromosome							0.000000003	pfam10446	Protein of unknown function (DUF2457). T
300709871	geranylgeranylglyceryl phosphate synthase fa	No	0			486891	487631	-		Halalkalicoccus_jeotgali_B3_uid50305	NC_014297	chromosome		arCOG01085	1 (-)	I	PcrB	(S)-3-O-geranylgeranylglyceryl phosphate syntha	1E-20	COG1646	Predicted phosphate-binding enzymes, TIM
300709872	hypothetical protein HacjB3_02505	No	0			488243	488419	-		Halalkalicoccus_jeotgali_B3_uid50305	NC_014297	chromosome									
300709873	hypothetical protein HacjB3_02510	No	0			490286	491509	-		Halalkalicoccus_jeotgali_B3_uid50305	NC_014297	chromosome		arCOG09176	8	S		Uncharacterized conserved protein			
300709874	hypothetical protein HacjB3_02515	Yes	0			491565	492233	-		Halalkalicoccus_jeotgali_B3_uid50305	NC_014297	chromosome									
300709875	hypothetical protein HacjB3_02520	No	0			493275	493403	+		Halalkalicoccus_jeotgali_B3_uid50305	NC_014297	chromosome									
300709876	hypothetical protein HacjB3_02525	No	1			493932	494024	+		Halalkalicoccus_jeotgali_B3_uid50305	NC_014297	chromosome									
300709877	hypothetical protein HacjB3_02530	No	0			494193	494609	+		Halalkalicoccus_jeotgali_B3_uid50305	NC_014297	chromosome									
300709878	hypothetical protein HacjB3_02535	No	0			494985	495200	+		Halalkalicoccus_jeotgali_B3_uid50305	NC_014297	chromosome									
300709879	ABC transporter ATP-binding protein	No	0			495980	496675	-		Halalkalicoccus_jeotgali_B3_uid50305	NC_014297	chromosome		arCOG00922	4 (-)	V	SalX	ABC-type antimicrobial peptide transport system,	3E-52	COG1136	ABC-type antimicrobial peptide transport
300709880	NAD-dependent epimerase/dehydratase	No	0			497309	498244	+		Halalkalicoccus_jeotgali_B3_uid50305	NC_014297	chromosome		arCOG01369	5 (-)	M	WcaG	Nucleoside-diphosphate-sugar epimerase	3E-39	COG0451	Nucleoside-diphosphate-sugar epimerases
300709881	glycosyl hydrolase BNR repeat-containing prot	No	0			499016	500290	-		Halalkalicoccus_jeotgali_B3_uid50305	NC_014297	chromosome		arCOG11134	1	S		Uncharacterized conserved protein			
300709882	hypothetical protein HacjB3_02555	No	0			501006	502070	+		Halalkalicoccus_jeotgali_B3_uid50305	NC_014297	chromosome							4E-11	pfam04006	Mpp10 protein. This family includes prot
300709883	hypothetical protein HacjB3_02560	Yes	0			502943	503536	+		Halalkalicoccus_jeotgali_B3_uid50305	NC_014297	chromosome									
300709884	glycosyl hydrolase BNR repeat-containing prot	Yes	0			504647	505927	-		Halalkalicoccus_jeotgali_B3_uid50305	NC_014297	chromosome									
300709885	hypothetical protein HacjB3_02570	No	1			506211	506447	-		Halalkalicoccus_jeotgali_B3_uid50305	NC_014297	chromosome		arCOG07779	1 (-)	S		Uncharacterized conserved protein	0.0000007	pfam11127	Protein of unknown function (DUF2892). T
===									6	Halalkalicoccus_jeotgali_B3_uid50305	NC_014297	chromosome									
300709888	hypothetical protein HacjB3_02585	No	0			508861	509106	+		Halalkalicoccus_jeotgali_B3_uid50305	NC_014297	chromosome		arCOG10959	8	S		Uncharacterized conserved protein			
300709889	hypothetical protein HacjB3_02590	No	0			509106	509717	+		Halalkalicoccus_jeotgali_B3_uid50305	NC_014297	chromosome									
300709890	hypothetical protein HacjB3_02595	No	0			509780	509959	+		Halalkalicoccus_jeotgali_B3_uid50305	NC_014297	chromosome									
300709891	cold-shock DNA-binding domain-containing pro	No	0			509969	510175	-		Halalkalicoccus_jeotgali_B3_uid50305	NC_014297	chromosome		arCOG02984	1 (-)	K	CspC	Cold shock protein, CspA family	2E-12	COG1278	Cold shock proteins
300709892	hypothetical protein HacjB3_02605	No	2			510501	510713	+		Halalkalicoccus_jeotgali_B3_uid50305	NC_014297	chromosome		arCOG11231	2	S		Uncharacterized conserved protein			
300709893	hypothetical protein HacjB3_02610	No	1			510718	510846	-		Halalkalicoccus_jeotgali_B3_uid50305	NC_014297	chromosome									
===									6	Halalkalicoccus_jeotgali_B3_uid50305	NC_014297	chromosome									
300710002	carboxyvinyl-carboxyphosphonate phosphoryl	No	0			637041	637979	+		Halalkalicoccus_jeotgali_B3_uid50305	NC_014297	chromosome		arCOG00581	1 (-)	G	PrpB	PEP phosphonomutase or related enzyme	2E-48	COG2513	PEP phosphonomutase and related enzymes
300710003	hypothetical protein HacjB3_03160	Yes	0			638010	639350	-		Halalkalicoccus_jeotgali_B3_uid50305	NC_014297	chromosome							6E-15	pfam07263	Dentin matrix protein 1 (DMP1). This fam
300710004	hypothetical protein HacjB3_03165	No	0			639698	640513	-		Halalkalicoccus_jeotgali_B3_uid50305	NC_014297	chromosome		arCOG09176	8	S		Uncharacterized conserved protein			
300710005	hypothetical protein HacjB3_03170	No	0			640586	640888	-		Halalkalicoccus_jeotgali_B3_uid50305	NC_014297	chromosome									
300710006	CHRD domain containing protein	No	0			641820	642128	+		Halalkalicoccus_jeotgali_B3_uid50305	NC_014297	chromosome									
300710007	hypothetical protein HacjB3_03180	No	0			642309	643052	+		Halalkalicoccus_jeotgali_B3_uid50305	NC_014297	chromosome		arCOG00500	2 (-)	R	ElaC	Metal-dependent hydrolase of the beta-lactamase	3E-23	COG1234	Metal-dependent hydrolases of the beta-l
===									9	Halalkalicoccus_jeotgali_B3_uid50305	NC_014297	chromosome									
300710400	hypothetical protein HacjB3_05155	No	0			987447	987815	-		Halalkalicoccus_jeotgali_B3_uid50305	NC_014297	chromosome									
300710401	holliday junction resolvase	No	0			987805	988170	-		Halalkalicoccus_jeotgali_B3_uid50305	NC_014297	chromosome		arCOG00919	2 (-)	L		Holliday junction resolvase	0.000002	pfam01870	Archaeal holliday junction resolvase (hj
300710402	hypothetical protein HacjB3_05165	No	0			988167	988817	-		Halalkalicoccus_jeotgali_B3_uid50305	NC_014297	chromosome									
300710403	hypothetical protein HacjB3_05170	No	0			988814	989185	-		Halalkalicoccus_jeotgali_B3_uid50305	NC_014297	chromosome		arCOG07776	1	S		Uncharacterized conserved protein			
300710404	hypothetical protein HacjB3_05175	No	0			989182	989403	-		Halalkalicoccus_jeotgali_B3_uid50305	NC_014297	chromosome									
300710405	hypothetical protein HacjB3_05180	No	1			989400	989600	-		Halalkalicoccus_jeotgali_B3_uid50305	NC_014297	chromosome									
300710406	hypothetical protein HacjB3_05185	No	1			989796	989897	-		Halalkalicoccus_jeotgali_B3_uid50305	NC_014297	chromosome									
300710407	hypothetical protein HacjB3_05190	No	0			990010	990237	-		Halalkalicoccus_jeotgali_B3_uid50305	NC_014297	chromosome									
300710408	hypothetical protein HacjB3_05195	No	0			990234	990611	-		Halalkalicoccus_jeotgali_B3_uid50305	NC_014297	chromosome		arCOG03828	5 (-)	S		Uncharacterized conserved protein			
===									25	Halalkalicoccus_jeotgali_B3_uid50305	NC_014297	chromosome									
300710415	hypothetical protein HacjB3_05230	No	0		Virus_Caudovi	994004	994231	+		Halalkalicoccus_jeotgali_B3_uid50305	NC_014297	chromosome		arCOG09396	1 (-)	K		Predicted transcriptional regulator	0.00005	pfam08220	DeoR-like helix-turn-helix domain.
300710416	hypothetical protein HacjB3_05235	No	0		Virus_Caudovi	994239	994604	-		Halalkalicoccus_jeotgali_B3_uid50305	NC_014297	chromosome									
300710417	hypothetical protein HacjB3_05240	No	0		Virus_Caudovi	994668	994856	-		Halalkalicoccus_jeotgali_B3_uid50305	NC_014297	chromosome									
300710418	hypothetical protein HacjB3_05245	No	0		Virus_Caudovi	994853	995206	-		Halalkalicoccus_jeotgali_B3_uid50305	NC_014297	chromosome									
300710419	hypothetical protein HacjB3_05250	No	1		Virus_Caudovi	995380	995484	+		Halalkalicoccus_jeotgali_B3_uid50305	NC_014297	chromosome									
300710420	hypothetical protein HacjB3_05255	No	0		Virus_Caudovi	995644	996114	+		Halalkalicoccus_jeotgali_B3_uid50305	NC_014297	chromosome									
300710421	hypothetical protein HacjB3_05260	No	0		Virus_Caudovi	996111	996239	+		Halalkalicoccus_jeotgali_B3_uid50305	NC_014297	chromosome									
300710422	hypothetical protein HacjB3_05265	No	0		Virus_Caudovi	996239	996448	+		Halalkalicoccus_jeotgali_B3_uid50305	NC_014297	chromosome									
300710423	hypothetical protein HacjB3_05270	No	0		Virus_Caudovi	996450	996734	+		Halalkalicoccus_jeotgali_B3_uid50305	NC_014297	chromosome									
300710424	hypothetical protein HacjB3_05275	No	0		Virus_Caudovi	996731	997051	+		Halalkalicoccus_jeotgali_B3_uid50305	NC_014297	chromosome									
300710425	hypothetical protein HacjB3_05280	No	0		Virus_Caudovi	997233	997739	+		Halalkalicoccus_jeotgali_B3_uid50305	NC_014297	chromosome									
300710426	hypothetical protein HacjB3_05285	No	4		Virus_Caudovi	997825	998379	-		Halalkalicoccus_jeotgali_B3_uid50305	NC_014297	chromosome									
300710427	hypothetical protein HacjB3_05290	No	2		Virus_Caudovi	998357	998614	-		Halalkalicoccus_jeotgali_B3_uid50305	NC_014297	chromosome									
300710428	hypothetical protein HacjB3_05295	No	3		Virus_Caudovi	998757	999347	-		Halalkalicoccus_jeotgali_B3_uid50305	NC_014297	chromosome									
300710429	hypothetical protein HacjB3_05300	No	0		Virus_Caudovi	999357	1000268	-		Halalkalicoccus_jeotgali_B3_uid50305	NC_014297	chromosome		arCOG09176	8	S		Uncharacterized conserved protein			
300710430	hypothetical protein HacjB3_05305	No	0		Virus_Caudovi	1000379	1000606	+		Halalkalicoccus_jeotgali_B3_uid50305	NC_014297	chromosome									
300710431	hypothetical protein HacjB3_05310	No	0		Virus_Caudovi	1000625	1001389	-		Halalkalicoccus_jeotgali_B3_uid50305	NC_014297	chromosome									
300710432	integrase family protein	No	0		Virus_Caudovi	1001828	1002349	+		Halalkalicoccus_jeotgali_B3_uid50305	NC_014297	chromosome		arCOG06451	4 (-)	L	XerC	Integrase			
300710433	asparagine synthase	No	0		Virus_Caudovi	1002434	1004044	-		Halalkalicoccus_jeotgali_B3_uid50305	NC_014297	chromosome		arCOG00121	3 (-)	E	AsnB	Asparagine synthase (glutamine-hydrolyzing)			
300710434	hypothetical protein HacjB3_05325	No	0		Virus_Caudovi	1004091	1005875	-		Halalkalicoccus_jeotgali_B3_uid50305	NC_014297	chromosome							0.00001	pfam13229	Right handed beta helix region. This reg
300710435	hypothetical protein HacjB3_05330	No	0		Virus_Caudovi	1005892	1008099	-		Halalkalicoccus_jeotgali_B3_uid50305	NC_014297	chromosome									
300710436	hypothetical protein HacjB3_05335	No	0		Virus_Caudovi	1008118	1008951	-		Halalkalicoccus_jeotgali_B3_uid50305	NC_014297	chromosome									
300710437	hypothetical protein HacjB3_05340	No	0		Virus_Caudovi	1008958	1009446	-		Halalkalicoccus_jeotgali_B3_uid50305	NC_014297	chromosome									
300710438	hypothetical protein HacjB3_05345	No	0		Virus_Caudovi	1009486	1010373	-		Halalkalicoccus_jeotgali_B3_uid50305	NC_014297	chromosome		arCOG10698	3 (-)	S		Uncharacterized conserved protein			
300710439	hypothetical protein HacjB3_05350	No	0		Virus_Caudovi	1010380	1010835	-		Halalkalicoccus_jeotgali_B3_uid50305	NC_014297	chromosome									
===									5	Halalkalicoccus_jeotgali_B3_uid50305	NC_014297	chromosome									
300710441	hypothetical protein HacjB3_05360	No	0		Virus_Caudovi	1012218	1012742	-		Halalkalicoccus_jeotgali_B3_uid50305	NC_014297	chromosome									
300710442	hypothetical protein HacjB3_05365	No	0		Virus_Caudovi	1012754	1013815	-		Halalkalicoccus_jeotgali_B3_uid50305	NC_014297	chromosome		arCOG11245	4	S		Uncharacterized conserved protein			
300710443	hypothetical protein HacjB3_05370	No	0		Virus_Caudovi	1013831	1014748	-		Halalkalicoccus_jeotgali_B3_uid50305	NC_014297	chromosome		arCOG10308	5 (-)	S		Uncharacterized conserved protein			
300710444	hypothetical protein HacjB3_05375	No	0		Virus_Caudovi	1014751	1015200	-		Halalkalicoccus_jeotgali_B3_uid50305	NC_014297	chromosome									
300710445	hypothetical protein HacjB3_05380	No	0		Virus_Caudovi	1015203	1015757	-		Halalkalicoccus_jeotgali_B3_uid50305	NC_014297	chromosome									
===									37	Halalkalicoccus_jeotgali_B3_uid50305	NC_014297	chromosome									
300710447	hypothetical protein HacjB3_05390	No	0		Virus_Caudovi	1019431	1019610	-		Halalkalicoccus_jeotgali_B3_uid50305	NC_014297	chromosome		arCOG03953	4 (-)	S		Uncharacterized conserved protein			
300710448	hypothetical protein HacjB3_05395	No	0		Virus_Caudovi	1019659	1020102	-		Halalkalicoccus_jeotgali_B3_uid50305	NC_014297	chromosome									
300710449	hypothetical protein HacjB3_05400	No	0		Virus_Caudovi	1020099	1020326	-		Halalkalicoccus_jeotgali_B3_uid50305	NC_014297	chromosome									
300710450	hypothetical protein HacjB3_05405	No	0		Virus_Caudovi	1021351	1021497	+		Halalkalicoccus_jeotgali_B3_uid50305	NC_014297	chromosome									
300710451	hypothetical protein HacjB3_05410	No	0		Virus_Caudovi	1021767	1022168	-		Halalkalicoccus_jeotgali_B3_uid50305	NC_014297	chromosome									
300710452	hypothetical protein HacjB3_05415	No	0		Virus_Caudovi	1022188	1023561	-		Halalkalicoccus_jeotgali_B3_uid50305	NC_014297	chromosome									
300710453	hypothetical protein HacjB3_05420	No	0		Virus_Caudovi	1023633	1024358	-		Halalkalicoccus_jeotgali_B3_uid50305	NC_014297	chromosome									
300710454	hypothetical protein HacjB3_05425	No	0		Virus_Caudovi	1024369	1024815	-		Halalkalicoccus_jeotgali_B3_uid50305	NC_014297	chromosome									
300710455	hypothetical protein HacjB3_05430	No	0		Virus_Caudovi	1024819	1025259	-		Halalkalicoccus_jeotgali_B3_uid50305	NC_014297	chromosome									
300710456	hypothetical protein HacjB3_05435	No	0		Virus_Caudovi	1025266	1025871	-		Halalkalicoccus_jeotgali_B3_uid50305	NC_014297	chromosome									
300710457	hypothetical protein HacjB3_05440	No	0		Virus_Caudovi	1025889	1026284	-		Halalkalicoccus_jeotgali_B3_uid50305	NC_014297	chromosome									
300710458	hypothetical protein HacjB3_05445	No	0		Virus_Caudovi	1026296	1026895	-		Halalkalicoccus_jeotgali_B3_uid50305	NC_014297	chromosome									
300710459	Linocin_M18 bacteriocin protein	No	0		Virus_Caudovi	1026902	1027972	-		Halalkalicoccus_jeotgali_B3_uid50305	NC_014297	chromosome		arCOG05908	2 (-)	R		Ferritin-like domain			
300710460	hypothetical protein HacjB3_05455	No	0		Virus_Caudovi	1027973	1028497	-		Halalkalicoccus_jeotgali_B3_uid50305	NC_014297	chromosome									
300710461	hypothetical protein HacjB3_05460	No	0		Virus_Caudovi	1028542	1030860	-		Halalkalicoccus_jeotgali_B3_uid50305	NC_014297	chromosome									
300710462	hypothetical protein HacjB3_05465	No	0		Virus_Caudovi	1030873	1031865	-		Halalkalicoccus_jeotgali_B3_uid50305	NC_014297	chromosome		arCOG09510	5 (-)	T		NAD+--asparagine ADP-ribosyltransferase			
300710463	hypothetical protein HacjB3_05470	No	0		Virus_Caudovi	1031887	1032291	-		Halalkalicoccus_jeotgali_B3_uid50305	NC_014297	chromosome									
300710464	hypothetical protein HacjB3_05475	No	0		Virus_Caudovi	1032368	1032475	-		Halalkalicoccus_jeotgali_B3_uid50305	NC_014297	chromosome									
300710465	hypothetical protein HacjB3_05480	No	0		Virus_Caudovi	1032468	1033928	-		Halalkalicoccus_jeotgali_B3_uid50305	NC_014297	chromosome									
300710466	hypothetical protein HacjB3_05485	No	0		Virus_Caudovi	1034175	1035788	-		Halalkalicoccus_jeotgali_B3_uid50305	NC_014297	chromosome		arCOG09550	17	Y	TerL	Phage terminase large subunit			
300710467	hypothetical protein HacjB3_05490	No	0		Virus_Caudovi	1035788	1035985	-		Halalkalicoccus_jeotgali_B3_uid50305	NC_014297	chromosome									
300710468	hypothetical protein HacjB3_05495	No	0		Virus_Caudovi	1035993	1036643	-		Halalkalicoccus_jeotgali_B3_uid50305	NC_014297	chromosome		arCOG09210	6 (-)	S		Uncharacterized conserved protein	0.00004	COG5484	Uncharacterized conserved protein
300710469	hypothetical protein HacjB3_05500	No	0		Virus_Caudovi	1036711	1037148	-		Halalkalicoccus_jeotgali_B3_uid50305	NC_014297	chromosome									
300710470	HNH endonuclease	No	0		Virus_Caudovi	1037167	1037457	-		Halalkalicoccus_jeotgali_B3_uid50305	NC_014297	chromosome		arCOG03899	2 (-)	V		Predicted restriction endonuclease, HNH family			
300710471	hypothetical protein HacjB3_05510	No	0		Virus_Caudovi	1037457	1037645	-		Halalkalicoccus_jeotgali_B3_uid50305	NC_014297	chromosome		arCOG11198	4	S		Uncharacterized conserved protein			
300710472	hypothetical protein HacjB3_05515	No	1		Virus_Caudovi	1037654	1037785	-		Halalkalicoccus_jeotgali_B3_uid50305	NC_014297	chromosome									
300710473	hypothetical protein HacjB3_05520	No	0		Virus_Caudovi	1037778	1038005	-		Halalkalicoccus_jeotgali_B3_uid50305	NC_014297	chromosome									
300710474	hypothetical protein HacjB3_05525	No	0		Virus_Caudovi	1038005	1038289	-		Halalkalicoccus_jeotgali_B3_uid50305	NC_014297	chromosome									
300710475	hypothetical protein HacjB3_05530	No	0		Virus_Caudovi	1038329	1038544	-		Halalkalicoccus_jeotgali_B3_uid50305	NC_014297	chromosome									
300710476	hypothetical protein HacjB3_05535	No	0		Virus_Caudovi	1038814	1038909	-		Halalkalicoccus_jeotgali_B3_uid50305	NC_014297	chromosome									
300710477	hypothetical protein HacjB3_05540	No	0		Virus_Caudovi	1038906	1039103	-		Halalkalicoccus_jeotgali_B3_uid50305	NC_014297	chromosome									
300710478	hypothetical protein HacjB3_05545	No	0		Virus_Caudovi	1039100	1039297	-		Halalkalicoccus_jeotgali_B3_uid50305	NC_014297	chromosome									
300710479	hypothetical protein HacjB3_05550	No	0		Virus_Caudovi	1039275	1039931	-		Halalkalicoccus_jeotgali_B3_uid50305	NC_014297	chromosome									
300710480	hypothetical protein HacjB3_05555	No	0		Virus_Caudovi	1039930	1040190	+		Halalkalicoccus_jeotgali_B3_uid50305	NC_014297	chromosome									
300710481	hypothetical protein HacjB3_05560	No	0		Virus_Caudovi	1040637	1040843	+		Halalkalicoccus_jeotgali_B3_uid50305	NC_014297	chromosome		arCOG08095	3 (-)	K		Predicted transcriptional regulator, contains HTH domain			
300710482	hypothetical protein HacjB3_05565	No	0		Virus_Caudovi	1040840	1041454	+		Halalkalicoccus_jeotgali_B3_uid50305	NC_014297	chromosome									
300710483	hypothetical protein HacjB3_05570	Yes	0		Virus_Caudovi	1041457	1042665	-		Halalkalicoccus_jeotgali_B3_uid50305	NC_014297	chromosome		arCOG09176	8	S		Uncharacterized conserved protein			
===									11	Halalkalicoccus_jeotgali_B3_uid50305	NC_014297	chromosome									
300710495	hypothetical protein HacjB3_05630	No	0			1056238	1056807	+		Halalkalicoccus_jeotgali_B3_uid50305	NC_014297	chromosome									
300710496	ArsR family transcriptional regulator	No	0			1056892	1057134	-		Halalkalicoccus_jeotgali_B3_uid50305	NC_014297	chromosome		arCOG00394	7 (-)	K	ArsR	Transcriptional regulator containing HTH domain, ArsR family			
300710497	hypothetical protein HacjB3_05640	No	0			1057121	1057357	-		Halalkalicoccus_jeotgali_B3_uid50305	NC_014297	chromosome									
300710498	hypothetical protein HacjB3_05645	No	0			1057381	1057503	+		Halalkalicoccus_jeotgali_B3_uid50305	NC_014297	chromosome									
300710499	hypothetical protein HacjB3_05650	No	0			1057598	1057804	-		Halalkalicoccus_jeotgali_B3_uid50305	NC_014297	chromosome									
300710500	hypothetical protein HacjB3_05655	No	0			1057804	1057914	-		Halalkalicoccus_jeotgali_B3_uid50305	NC_014297	chromosome									
300710501	hypothetical protein HacjB3_05660	No	0			1057941	1058315	-		Halalkalicoccus_jeotgali_B3_uid50305	NC_014297	chromosome									
300710502	hypothetical protein HacjB3_05665	No	0			1058521	1058757	-		Halalkalicoccus_jeotgali_B3_uid50305	NC_014297	chromosome									
300710503	hypothetical protein HacjB3_05670	No	0			1058754	1061669	-		Halalkalicoccus_jeotgali_B3_uid50305	NC_014297	chromosome		arCOG04495	4 (-)	L	RepH	Plasmid replication protein, contains HTH domain			
300710504	hypothetical protein HacjB3_05675	No	0			1062112	1062456	-		Halalkalicoccus_jeotgali_B3_uid50305	NC_014297	chromosome									
300710505	hypothetical protein HacjB3_05680	Yes	1			1062904	1063095	-		Halalkalicoccus_jeotgali_B3_uid50305	NC_014297	chromosome									
===									5	Halalkalicoccus_jeotgali_B3_uid50305	NC_014297	chromosome									
300710549	hypothetical protein HacjB3_05900	No	0			1107135	1107410	+		Halalkalicoccus_jeotgali_B3_uid50305	NC_014297	chromosome									
300710550	hypothetical protein HacjB3_05905	No	0			1107434	1107721	-		Halalkalicoccus_jeotgali_B3_uid50305	NC_014297	chromosome									
300710551	hypothetical protein HacjB3_05910	No	0			1109017	1109364	+		Halalkalicoccus_jeotgali_B3_uid50305	NC_014297	chromosome									
300710552	filamentation induced by cAMP protein Fic	No	0			1109427	1110035	-		Halalkalicoccus_jeotgali_B3_uid50305	NC_014297	chromosome		arCOG03110	5 (-)	K		Predicted transcriptional regulator, contains Fic ar	1E-26	COG3177	Fic family protein
300710553	hypothetical protein HacjB3_05920	No	0			1110047	1110154	-		Halalkalicoccus_jeotgali_B3_uid50305	NC_014297	chromosome									
===									5	Halalkalicoccus_jeotgali_B3_uid50305	NC_014297	chromosome									
300710744	hypothetical protein HacjB3_06895	No	0			1289484	1289789	+		Halalkalicoccus_jeotgali_B3_uid50305	NC_014297	chromosome									
300710745	hypothetical protein HacjB3_06900	Yes	4			1290870	1291223	+		Halalkalicoccus_jeotgali_B3_uid50305	NC_014297	chromosome									
300710746	zinc finger SWIM domain-containing protein	No	0			1291605	1292111	-		Halalkalicoccus_jeotgali_B3_uid50305	NC_014297	chromosome									
300710747	phage integrase family protein	No	0			1292569	1293330	-		Halalkalicoccus_jeotgali_B3_uid50305	NC_014297	chromosome		arCOG01241	31 (-)	L	XerC	Integrase	4E-19	COG4974	Site-specific recombinase XerD
300710748	hypothetical protein HacjB3_06915	No	0			1293567	1294076	-		Halalkalicoccus_jeotgali_B3_uid50305	NC_014297	chromosome									
===									16	Halalkalicoccus_jeotgali_B3_uid50305	NC_014297	chromosome									
300710804	oxidoreductase	No	0			1342957	1344042	+		Halalkalicoccus_jeotgali_B3_uid50305	NC_014297	chromosome		arCOG01622	3 (-)	R	MviM	Predicted dehydrogenase	4E-43	COG0673	Predicted dehydrogenases and related pro
300710805	hypothetical protein HacjB3_07210	No	0			1344090	1344374	-		Halalkalicoccus_jeotgali_B3_uid50305	NC_014297	chromosome									
300710806	hypothetical protein HacjB3_07215	No	0			1344431	1344826	-		Halalkalicoccus_jeotgali_B3_uid50305	NC_014297	chromosome									
300710807	hypothetical protein HacjB3_07220	No	0			1345806	1345997	-		Halalkalicoccus_jeotgali_B3_uid50305	NC_014297	chromosome									
300710808	hypothetical protein HacjB3_07225	No	0			1346093	1346302	+		Halalkalicoccus_jeotgali_B3_uid50305	NC_014297	chromosome									
300710809	hypothetical protein HacjB3_07230	No	0			1347021	1347311	+		Halalkalicoccus_jeotgali_B3_uid50305	NC_014297	chromosome									
300710810	hypothetical protein HacjB3_07235	No	0			1347441	1347587	+		Halalkalicoccus_jeotgali_B3_uid50305	NC_014297	chromosome									
300710811	hypothetical protein HacjB3_07240	No	2			1347677	1347892	+		Halalkalicoccus_jeotgali_B3_uid50305	NC_014297	chromosome									
300710812	hypothetical protein HacjB3_07245	No	0			1347976	1348260	+		Halalkalicoccus_jeotgali_B3_uid50305	NC_014297	chromosome									
300710813	phage tail tape measure protein, TP901 family	No	4			1348451	1351072	+		Halalkalicoccus_jeotgali_B3_uid50305	NC_014297	chromosome		arCOG11109	8 (-)	Y		Phage tail tape measure protein, TP901 family	9E-32	pfam10145	Phage-related minor tail protein. Member ; Phage-related protein
300710814	hypothetical protein HacjB3_07255	No	0			1351156	1352988	+		Halalkalicoccus_jeotgali_B3_uid50305	NC_014297	chromosome									
300710815	hypothetical protein HacjB3_07260	No	0			1353761	1354099	+		Halalkalicoccus_jeotgali_B3_uid50305	NC_014297	chromosome									
300710816	hypothetical protein HacjB3_07265	No	0			1354653	1355393	+		Halalkalicoccus_jeotgali_B3_uid50305	NC_014297	chromosome									
300710817	hypothetical protein HacjB3_07270	No	1			1355674	1356051	-		Halalkalicoccus_jeotgali_B3_uid50305	NC_014297	chromosome									
300710818	hypothetical protein HacjB3_07275	No	0			1356407	1356505	+		Halalkalicoccus_jeotgali_B3_uid50305	NC_014297	chromosome									
300710819	hypothetical protein HacjB3_07280	No	0			1356502	1358874	+		Halalkalicoccus_jeotgali_B3_uid50305	NC_014297	chromosome		arCOG07786	2 (-)	K		Predicted Helicase fused to HTH domain			
===									5	Halalkalicoccus_jeotgali_B3_uid50305	NC_014297	chromosome									
300711595	hypothetical protein HacjB3_11170	No	2			2073746	2074060	+		Halalkalicoccus_jeotgali_B3_uid50305	NC_014297	chromosome									
300711596	hypothetical protein HacjB3_11175	No	0			2074242	2074625	-		Halalkalicoccus_jeotgali_B3_uid50305	NC_014297	chromosome									
300711597	methionine sulfoxide reductase A	No	0			2074783	2075322	+		Halalkalicoccus_jeotgali_B3_uid50305	NC_014297	chromosome		arCOG02816	1 (-)	O	MsrA	Peptide methionine sulfoxide reductase	2E-66	COG0225	Peptide methionine sulfoxide reductase [
300711598	hypothetical protein HacjB3_11185	No	0			2075324	2075530	-		Halalkalicoccus_jeotgali_B3_uid50305	NC_014297	chromosome									
300711599	hypothetical protein HacjB3_11190	No	0			2075713	2075847	+		Halalkalicoccus_jeotgali_B3_uid50305	NC_014297	chromosome									
===									5	Halalkalicoccus_jeotgali_B3_uid50305	NC_014297	chromosome									
300711824	hypothetical protein HacjB3_12315	No	0			2281387	2281482	+		Halalkalicoccus_jeotgali_B3_uid50305	NC_014297	chromosome									
300711825	hypothetical protein HacjB3_12320	No	6			2281479	2282096	+		Halalkalicoccus_jeotgali_B3_uid50305	NC_014297	chromosome							0.000003	pfam11139	Protein of unknown function (DUF2910). S
300711826	hypothetical protein HacjB3_12325	No	4			2282316	2282720	+		Halalkalicoccus_jeotgali_B3_uid50305	NC_014297	chromosome									
300711827	hypothetical protein HacjB3_12330	No	7			2282812	2283843	+		Halalkalicoccus_jeotgali_B3_uid50305	NC_014297	chromosome		arCOG02642	3 (-)	R	yhhT	Predicted permease, member of the PurR regul	2E-29	COG0628	Predicted permease, member of the PurR r
300711828	hypothetical protein HacjB3_12335	No	0			2284042	2284416	-		Halalkalicoccus_jeotgali_B3_uid50305	NC_014297	chromosome									
===									5	Halalkalicoccus_jeotgali_B3_uid50305	NC_014298	plasmid 1									
300712482	hypothetical protein HacjB3_15721	No	0		plasmid	69741	69890	-		Halalkalicoccus_jeotgali_B3_uid50305	NC_014298	plasmid 1									
300712483	hypothetical protein HacjB3_15726	No	0		plasmid	69963	70070	+		Halalkalicoccus_jeotgali_B3_uid50305	NC_014298	plasmid 1									
300712484	hypothetical protein HacjB3_15731	No	0		plasmid	70191	70514	+		Halalkalicoccus_jeotgali_B3_uid50305	NC_014298	plasmid 1		arCOG10874	2	S		Uncharacterized conserved protein			
300712485	hypothetical protein HacjB3_15736	No	0		plasmid	70584	70748	-		Halalkalicoccus_jeotgali_B3_uid50305	NC_014298	plasmid 1		arCOG01641	1 (-)	R		Predicted RNA-binding protein, contains TRAM d	2E-12	COG3269	Predicted RNA-binding protein, contains
300712486	hypothetical protein HacjB3_15741	No	0		plasmid	71292	71501	+		Halalkalicoccus_jeotgali_B3_uid50305	NC_014298	plasmid 1		arCOG10889	1	S		Uncharacterized conserved protein			
===									7	Halalkalicoccus_jeotgali_B3_uid50305	NC_014298	plasmid 1									
300712521	glycerol dehydratase reactivation factor, small	No	0		plasmid	108008	108400	-		Halalkalicoccus_jeotgali_B3_uid50305	NC_014298	plasmid 1							4E-11	pfam02288	Dehydratase medium subunit. This family
300712522	glycerol dehydratase reactivation factor, large	No	0		plasmid	108423	110330	-		Halalkalicoccus_jeotgali_B3_uid50305	NC_014298	plasmid 1		arCOG04280	1 (-)	K	NagC	Transcriptional regulator/sugar kinase	2E-119	pfam08841	Diol dehydratase reactivase ATPase-like
300712523	dehydratase small subunit	No	0		plasmid	110434	110838	-		Halalkalicoccus_jeotgali_B3_uid50305	NC_014298	plasmid 1							3E-41	pfam02287	Dehydratase small subunit. This family c
300712524	dehydratase medium subunit	No	0		plasmid	110852	111424	-		Halalkalicoccus_jeotgali_B3_uid50305	NC_014298	plasmid 1							6E-31	pfam02288	Dehydratase medium subunit. This family
300712525	glycerol dehydratase large subunit	No	0		plasmid	111494	113215	-		Halalkalicoccus_jeotgali_B3_uid50305	NC_014298	plasmid 1								pfam02286	Dehydratase large subunit. This family c
300712526	short-chain dehydrogenase/reductase SDR	No	0		plasmid	113629	114402	+		Halalkalicoccus_jeotgali_B3_uid50305	NC_014298	plasmid 1		arCOG01259	2 (-)	I	FabG	Short-chain alcohol dehydrogenase	7E-44	COG1028	Dehydrogenases with different specificit
300712527	hypothetical protein HacjB3_15956	Yes	0		plasmid	114474	115325	+		Halalkalicoccus_jeotgali_B3_uid50305	NC_014298	plasmid 1									
===									5	Halalkalicoccus_jeotgali_B3_uid50305	NC_014298	plasmid 1									
300712547	Hypothetical transthyretin-like protein	No	0		plasmid	135104	135454	-		Halalkalicoccus_jeotgali_B3_uid50305	NC_014298	plasmid 1	clust_304						1E-33	COG2351	Transthyretin-like protein
300712548	Uricase	No	0		plasmid	135451	136425	-		Halalkalicoccus_jeotgali_B3_uid50305	NC_014298	plasmid 1	clust_99						2E-56	COG3648	Uricase (urate oxidase)
300712549	Uricase	No	0		plasmid	136493	137023	-		Halalkalicoccus_jeotgali_B3_uid50305	NC_014298	plasmid 1	clust_198						2E-49	COG3195	Uncharacterized protein conserved in bac
300712550	N-carbamoyl-L-amino acid amidohydrolase	No	0		plasmid	137259	138587	+		Halalkalicoccus_jeotgali_B3_uid50305	NC_014298	plasmid 1		arCOG01109	1 (-)	E	ArgE	Acetylornithine deacetylase/Succinyl-diaminopim	0.0001	pfam01546	Peptidase family M20/M25/M40. This famil
300712551	hypothetical protein HacjB3_16076	No	0		plasmid	138604	139023	-		Halalkalicoccus_jeotgali_B3_uid50305	NC_014298	plasmid 1		arCOG10646	1	S		Uncharacterized conserved protein	3E-12	pfam12903	Protein of unknown function (DUF3830). T
===									8	Halalkalicoccus_jeotgali_B3_uid50305	NC_014298	plasmid 1									
300712564	hypothetical protein HacjB3_16141	No	0		plasmid	155392	155511	+		Halalkalicoccus_jeotgali_B3_uid50305	NC_014298	plasmid 1									
300712565	hypothetical protein HacjB3_16146	Yes	0		plasmid	155750	156619	-		Halalkalicoccus_jeotgali_B3_uid50305	NC_014298	plasmid 1									
300712566	transducer protein htr15	No	0		plasmid	156902	158410	-		Halalkalicoccus_jeotgali_B3_uid50305	NC_014298	plasmid 1	arCOG02318,a	arCOG02318	1 (-)	T	Tar	Sensory protein; contains PAS, HAMP and Methy	2E-47	COG0840	Methyl-accepting chemotaxis protein
300712567	reactivating factor for ethanolamine ammonia ly	No	0		plasmid	158961	160436	-		Halalkalicoccus_jeotgali_B3_uid50305	NC_014298	plasmid 1							4E-113	pfam06277	Ethanolamine utilisation protein EutA. T
300712568	ethanolamine ammonia-lyase small subunit	No	0		plasmid	160433	161167	-		Halalkalicoccus_jeotgali_B3_uid50305	NC_014298	plasmid 1							2E-71	COG4302	Ethanolamine ammonia-lyase, small subuni
300712569	ethanolamine ammonia lyase large subunit	No	0		plasmid	161160	162536	-		Halalkalicoccus_jeotgali_B3_uid50305	NC_014298	plasmid 1								COG4303	Ethanolamine ammonia-lyase, large subuni
300712570	dimethylmenaquinone methyltransferase	No	0		plasmid	162641	163189	-		Halalkalicoccus_jeotgali_B3_uid50305	NC_014298	plasmid 1		arCOG00117	1 (-)	H	MenG	Demethylmenaquinone methyltransferase	1E-31	COG0684	Demethylmenaquinone methyltransferase
300712571	hypothetical protein HacjB3_16176	No	0		plasmid	163954	164526	+		Halalkalicoccus_jeotgali_B3_uid50305	NC_014298	plasmid 1									
===									11	Halalkalicoccus_jeotgali_B3_uid50305	NC_014298	plasmid 1									
300712644	Phytanoyl-CoA dioxygenase	No	0		plasmid	249666	250157	-		Halalkalicoccus_jeotgali_B3_uid50305	NC_014298	plasmid 1		arCOG09157	1	S		Uncharacterized conserved protein	3E-15	pfam05721	Phytanoyl-CoA dioxygenase (PhyH). This f
300712645	hypothetical protein HacjB3_16586	No	0		plasmid	250173	250433	+		Halalkalicoccus_jeotgali_B3_uid50305	NC_014298	plasmid 1									
300712646	hypothetical protein HacjB3_16591	No	0		plasmid	250526	250702	-		Halalkalicoccus_jeotgali_B3_uid50305	NC_014298	plasmid 1									

300712647	deoxyribodipyrimidine photolyase	No	0	plasmid	250811	251401	-		Halalkalicoccus_jeotgali_B3_uid50305	NC_014298	plasmid 1		arCOG02840	1 (-)	L	PhrB	Deoxyribodipyrimidine photolyase	7E-23	pfam03441	FAD binding domain of DNA photolyase.
300712648	hypothetical protein HacjB3_16601	No	0	plasmid	251783	252130	+		Halalkalicoccus_jeotgali_B3_uid50305	NC_014298	plasmid 1									
300712649	peptidase M14 carboxypeptidase A	No	0	plasmid	253248	255971	-		Halalkalicoccus_jeotgali_B3_uid50305	NC_014298	plasmid 1									
300712650	hypothetical protein HacjB3_16611	No	0	plasmid	256448	256660	-		Halalkalicoccus_jeotgali_B3_uid50305	NC_014298	plasmid 1									
300712651	hypothetical protein HacjB3_16616	No	0	plasmid	256847	257068	-		Halalkalicoccus_jeotgali_B3_uid50305	NC_014298	plasmid 1									
300712652	hypothetical protein HacjB3_16621	Yes	0	plasmid	258698	258907	+		Halalkalicoccus_jeotgali_B3_uid50305	NC_014298	plasmid 1									
300712653	hypothetical protein HacjB3_16626	No	0	plasmid	259166	259459	+		Halalkalicoccus_jeotgali_B3_uid50305	NC_014298	plasmid 1									
300712654	protein N-acetyltransferase-like protein	No	0	plasmid	260349	260912	+		Halalkalicoccus_jeotgali_B3_uid50305	NC_014298	plasmid 1		arCOG00842	2 (-)	J	RimL	Acetyltransferase, RimL family	8E-17	COG1670	Acetyltransferases, including N-acetylas
===								5	Halalkalicoccus_jeotgali_B3_uid50305	NC_014298	plasmid 1									
300712687	hypothetical protein HacjB3_16796	No	0	plasmid	310358	310528	+		Halalkalicoccus_jeotgali_B3_uid50305	NC_014298	plasmid 1									
300712688	hypothetical protein HacjB3_16801	No	0	plasmid	310702	310866	+		Halalkalicoccus_jeotgali_B3_uid50305	NC_014298	plasmid 1									
300712689	low temperature requirement protein A	No	12	plasmid	310964	312079	+		Halalkalicoccus_jeotgali_B3_uid50305	NC_014298	plasmid 1		arCOG10808	2	S		Uncharacterized conserved protein	4E-51	pfam06772	Bacterial low temperature requirement A
300712690	hypothetical protein HacjB3_16811	No	0	plasmid	312436	312666	+		Halalkalicoccus_jeotgali_B3_uid50305	NC_014298	plasmid 1		arCOG08216	1 (-)	V		Antitoxin MazE superfamily protein			
300712691	hypothetical protein HacjB3_16816	No	0	plasmid	312799	313053	-		Halalkalicoccus_jeotgali_B3_uid50305	NC_014298	plasmid 1									
===								5	Halalkalicoccus_jeotgali_B3_uid50305	NC_014298	plasmid 1									
300712719	hypothetical protein HacjB3_16956	No	0	plasmid	351259	351576	+		Halalkalicoccus_jeotgali_B3_uid50305	NC_014298	plasmid 1									
300712720	hypothetical protein HacjB3_16961	No	0	plasmid	351710	351919	+		Halalkalicoccus_jeotgali_B3_uid50305	NC_014298	plasmid 1									
300712721	glycoside hydrolase family 2 sugar binding pro	No	0	plasmid	352177	355743	-		Halalkalicoccus_jeotgali_B3_uid50305	NC_014298	plasmid 1	arCOG07337,a	arCOG07337	1 (-)	G	LacZ	Beta-galactosidase/beta-glucuronidase	3E-35	COG3250	Beta-galactosidase/beta-glucuronidase
300712722	hypothetical protein HacjB3_16971	No	0	plasmid	355921	356175	+		Halalkalicoccus_jeotgali_B3_uid50305	NC_014298	plasmid 1									
300712723	hypothetical protein HacjB3_16976	No	0	plasmid	356308	356448	+		Halalkalicoccus_jeotgali_B3_uid50305	NC_014298	plasmid 1									
===								5	Halalkalicoccus_jeotgali_B3_uid50305	NC_014298	plasmid 1									
300712764	hypothetical protein HacjB3_17216	No	0	plasmid	402772	403131	-		Halalkalicoccus_jeotgali_B3_uid50305	NC_014298	plasmid 1							0.000009	COG1196	Chromosome segregation ATPases
300712765	hypothetical protein HacjB3_17221	No	0	plasmid	403226	403417	-		Halalkalicoccus_jeotgali_B3_uid50305	NC_014298	plasmid 1		arCOG08988	1	S		Uncharacterized conserved protein			
300712766	hypothetical protein HacjB3_17226	No	14	plasmid	403740	405662	-		Halalkalicoccus_jeotgali_B3_uid50305	NC_014298	plasmid 1		arCOG03188	2 (-)	S		Uncharacterized conserved protein			
300712767	hypothetical protein HacjB3_17231	No	0	plasmid	405884	406006	-		Halalkalicoccus_jeotgali_B3_uid50305	NC_014298	plasmid 1									
300712768	hypothetical protein HacjB3_17236	No	1	plasmid	406126	406272	+		Halalkalicoccus_jeotgali_B3_uid50305	NC_014298	plasmid 1									
===								17	Halalkalicoccus_jeotgali_B3_uid50305	NC_014299	plasmid 2									
300712781	hypothetical protein HacjB3_17298	No	0	plasmid	14162	14527	-		Halalkalicoccus_jeotgali_B3_uid50305	NC_014299	plasmid 2	clust_292								
300712782	hypothetical protein HacjB3_17303	No	0	plasmid	14763	15320	-		Halalkalicoccus_jeotgali_B3_uid50305	NC_014299	plasmid 2		arCOG07773	5 (-)	S		SWIM zinc finger containing protein			
300712783	hypothetical protein HacjB3_17308	No	0	plasmid	15526	15645	+		Halalkalicoccus_jeotgali_B3_uid50305	NC_014299	plasmid 2	clust_509								
300712784	hypothetical protein HacjB3_17313	No	0	plasmid	15910	16479	-		Halalkalicoccus_jeotgali_B3_uid50305	NC_014299	plasmid 2	clust_185								
300712785	hypothetical protein HacjB3_17318	No	0	plasmid	16549	16701	+		Halalkalicoccus_jeotgali_B3_uid50305	NC_014299	plasmid 2	clust_497								
300712786	hypothetical protein HacjB3_17323	No	0	plasmid	16894	17346	+		Halalkalicoccus_jeotgali_B3_uid50305	NC_014299	plasmid 2	clust_233								
300712787	hypothetical protein HacjB3_17328	No	0	plasmid	17343	17441	+		Halalkalicoccus_jeotgali_B3_uid50305	NC_014299	plasmid 2	clust_515								
300712788	hypothetical protein HacjB3_17333	No	0	plasmid	17438	18109	+		Halalkalicoccus_jeotgali_B3_uid50305	NC_014299	plasmid 2		arCOG04609	2 (-)	S		Predicted lipoprotein	0.0000003	COG1196	Chromosome segregation ATPases
300712789	hypothetical protein HacjB3_17338	No	0	plasmid	19077	19580	+		Halalkalicoccus_jeotgali_B3_uid50305	NC_014299	plasmid 2	clust_213								
300712790	hypothetical protein HacjB3_17343	No	0	plasmid	19571	20857	-		Halalkalicoccus_jeotgali_B3_uid50305	NC_014299	plasmid 2		arCOG09176	8	S		Uncharacterized conserved protein			
300712791	hypothetical protein HacjB3_17348	No	2	plasmid	20872	21081	-		Halalkalicoccus_jeotgali_B3_uid50305	NC_014299	plasmid 2	clust_443								
300712792	hypothetical protein HacjB3_17353	No	2	plasmid	21617	21961	+		Halalkalicoccus_jeotgali_B3_uid50305	NC_014299	plasmid 2		arCOG10938	2	S		Uncharacterized conserved protein			
300712793	hypothetical protein HacjB3_17358	No	6	plasmid	22204	23415	+		Halalkalicoccus_jeotgali_B3_uid50305	NC_014299	plasmid 2	clust_82						0.00002	pfam05802	Enterobacterial EspB protein. EspB is a
300712794	transfer complex protein, putative	No	2	plasmid	23418	26696	+		Halalkalicoccus_jeotgali_B3_uid50305	NC_014299	plasmid 2	arCOG04035,a	arCOG04035	5 (-)	U	VirB4	Type IV secretory pathway, VirB4 component	2E-11	pfam12846	AAA-like domain. This family of domains
300712795	hypothetical protein HacjB3_17368	No	0	plasmid	28187	28693	+		Halalkalicoccus_jeotgali_B3_uid50305	NC_014299	plasmid 2	clust_211								
300712796	hypothetical protein HacjB3_17373	No	0	plasmid	29116	29634	+		Halalkalicoccus_jeotgali_B3_uid50305	NC_014299	plasmid 2		arCOG10343	6	S		Uncharacterized conserved protein			
300712797	relaxase/mobilization nuclease domain-contair	No	0	plasmid	29637	30161	+		Halalkalicoccus_jeotgali_B3_uid50305	NC_014299	plasmid 2		arCOG10214	7	S		Uncharacterized conserved protein			
===								5	Halalkalicoccus_jeotgali_B3_uid50305	NC_014299	plasmid 2									
300712917	hypothetical protein HacjB3_17978	No	0	plasmid	153534	153659	-		Halalkalicoccus_jeotgali_B3_uid50305	NC_014299	plasmid 2									
300712918	hypothetical protein HacjB3_17983	Yes	1	plasmid	153961	154971	+		Halalkalicoccus_jeotgali_B3_uid50305	NC_014299	plasmid 2		arCOG06249	3 (-)	S		Uncharacterized conserved protein			
300712919	hypothetical protein HacjB3_17988	No	1	plasmid	155231	155731	+		Halalkalicoccus_jeotgali_B3_uid50305	NC_014299	plasmid 2		arCOG11205	1	S		Uncharacterized conserved protein			
300712920	hypothetical protein HacjB3_17993	No	3	plasmid	157022	157309	-		Halalkalicoccus_jeotgali_B3_uid50305	NC_014299	plasmid 2									
300712921	hypothetical protein HacjB3_17998	No	0	plasmid	157726	157974	-		Halalkalicoccus_jeotgali_B3_uid50305	NC_014299	plasmid 2									
===								17	Halalkalicoccus_jeotgali_B3_uid50305	NC_014299	plasmid 2									
300712929	hypothetical protein HacjB3_18038	No	0	plasmid	163075	163278	+		Halalkalicoccus_jeotgali_B3_uid50305	NC_014299	plasmid 2		arCOG03953	4 (-)	S		Uncharacterized conserved protein			
300712930	hypothetical protein HacjB3_18043	No	1	plasmid	163292	163477	+		Halalkalicoccus_jeotgali_B3_uid50305	NC_014299	plasmid 2	clust_471								
300712931	hypothetical protein HacjB3_18048	No	2	plasmid	164025	164195	+		Halalkalicoccus_jeotgali_B3_uid50305	NC_014299	plasmid 2	clust_487								
300712932	hypothetical protein HacjB3_18053	No	4	plasmid	164713	165132	-		Halalkalicoccus_jeotgali_B3_uid50305	NC_014299	plasmid 2		arCOG10943	2	S		Uncharacterized conserved protein			
300712933	hypothetical protein HacjB3_18058	No	0	plasmid	165550	166134	+		Halalkalicoccus_jeotgali_B3_uid50305	NC_014299	plasmid 2	clust_178								
300712934	hypothetical protein HacjB3_18063	No	0	plasmid	166139	166372	+		Halalkalicoccus_jeotgali_B3_uid50305	NC_014299	plasmid 2	clust_414								
300712935	hypothetical protein HacjB3_18068	No	0	plasmid	166452	166718	+		Halalkalicoccus_jeotgali_B3_uid50305	NC_014299	plasmid 2		arCOG10959	8	S		Uncharacterized conserved protein			
300712936	hypothetical protein HacjB3_18073	No	0	plasmid	166715	166879	+		Halalkalicoccus_jeotgali_B3_uid50305	NC_014299	plasmid 2	clust_490								
300712937	hypothetical protein HacjB3_18078	No	0	plasmid	166876	167061	+		Halalkalicoccus_jeotgali_B3_uid50305	NC_014299	plasmid 2	clust_472								
300712938	hypothetical protein HacjB3_18083	No	0	plasmid	167058	167300	+		Halalkalicoccus_jeotgali_B3_uid50305	NC_014299	plasmid 2	clust_402								
300712939	hypothetical protein HacjB3_18088	No	0	plasmid	167297	167683	+		Halalkalicoccus_jeotgali_B3_uid50305	NC_014299	plasmid 2	clust_275								
300712940	hypothetical protein HacjB3_18093	No	0	plasmid	167680	167883	+		Halalkalicoccus_jeotgali_B3_uid50305	NC_014299	plasmid 2	clust_451								
300712941	hypothetical protein HacjB3_18098	No	0	plasmid	167880	168350	+		Halalkalicoccus_jeotgali_B3_uid50305	NC_014299	plasmid 2	clust_223								
300712942	hypothetical protein HacjB3_18103	No	0	plasmid	169156	169623	+		Halalkalicoccus_jeotgali_B3_uid50305	NC_014299	plasmid 2		arCOG10343	6	S		Uncharacterized conserved protein			
300712943	relaxase/mobilization nuclease domain-contair	No	0	plasmid	169620	170141	+		Halalkalicoccus_jeotgali_B3_uid50305	NC_014299	plasmid 2		arCOG10214	7	S		Uncharacterized conserved protein	0.00006	COG3843	Type IV secretory pathway, VirD2 compone
300712944	Type IV secretory pathway VirD4 protein-like p	Yes	3	plasmid	170143	171246	+		Halalkalicoccus_jeotgali_B3_uid50305	NC_014299	plasmid 2		arCOG00280	21 (-)	L		HerA helicase	0.00000002	pfam10412	Type IV secretion-system coupling protei
300712945	Type IV secretory pathway VirD4 protein-like p	No	0	plasmid	171231	171737	+		Halalkalicoccus_jeotgali_B3_uid50305	NC_014299	plasmid 2	clust_212						0.0000002	pfam12696	TraM recognition site of TraD and TraG.
===								17	Halalkalicoccus_jeotgali_B3_uid50305	NC_014299	plasmid 2									
300712960	hypothetical protein HacjB3_18193	No	0	plasmid	181842	182045	+		Halalkalicoccus_jeotgali_B3_uid50305	NC_014299	plasmid 2		arCOG03953	4 (-)	S		Uncharacterized conserved protein			
300712961	hypothetical protein HacjB3_18198	No	1	plasmid	182059	182244	+		Halalkalicoccus_jeotgali_B3_uid50305	NC_014299	plasmid 2	clust_471								
300712962	hypothetical protein HacjB3_18203	No	2	plasmid	182792	182962	+		Halalkalicoccus_jeotgali_B3_uid50305	NC_014299	plasmid 2	clust_487								
300712963	hypothetical protein HacjB3_18208	No	4	plasmid	183480	183899	-		Halalkalicoccus_jeotgali_B3_uid50305	NC_014299	plasmid 2		arCOG10943	2	S		Uncharacterized conserved protein			
300712964	hypothetical protein HacjB3_18213	No	0	plasmid	184317	184901	+		Halalkalicoccus_jeotgali_B3_uid50305	NC_014299	plasmid 2	clust_178								
300712965	hypothetical protein HacjB3_18218	No	0	plasmid	184906	185139	+		Halalkalicoccus_jeotgali_B3_uid50305	NC_014299	plasmid 2	clust_414								
300712966	hypothetical protein HacjB3_18223	No	0	plasmid	185219	185485	+		Halalkalicoccus_jeotgali_B3_uid50305	NC_014299	plasmid 2		arCOG10959	8	S		Uncharacterized conserved protein			
300712967	hypothetical protein HacjB3_18228	No	0	plasmid	185482	185646	+		Halalkalicoccus_jeotgali_B3_uid50305	NC_014299	plasmid 2	clust_490								
300712968	hypothetical protein HacjB3_18233	No	0	plasmid	185643	185828	+		Halalkalicoccus_jeotgali_B3_uid50305	NC_014299	plasmid 2	clust_472								
300712969	hypothetical protein HacjB3_18238	No	0	plasmid	185825	186067	+		Halalkalicoccus_jeotgali_B3_uid50305	NC_014299	plasmid 2	clust_402								
300712970	hypothetical protein HacjB3_18243	No	0	plasmid	186064	186450	+		Halalkalicoccus_jeotgali_B3_uid50305	NC_014299	plasmid 2	clust_275								
300712971	hypothetical protein HacjB3_18248	No	0	plasmid	186447	186650	+		Halalkalicoccus_jeotgali_B3_uid50305	NC_014299	plasmid 2	clust_451								
300712972	hypothetical protein HacjB3_18253	No	0	plasmid	186647	187117	+		Halalkalicoccus_jeotgali_B3_uid50305	NC_014299	plasmid 2	clust_223								
300712973	hypothetical protein HacjB3_18258	No	0	plasmid	187923	188390	+		Halalkalicoccus_jeotgali_B3_uid50305	NC_014299	plasmid 2		arCOG10343	6	S		Uncharacterized conserved protein			
300712974	relaxase/mobilization nuclease domain-contair	No	0	plasmid	188387	188908	+		Halalkalicoccus_jeotgali_B3_uid50305	NC_014299	plasmid 2		arCOG10214	7	S		Uncharacterized conserved protein	0.00006	COG3843	Type IV secretory pathway, VirD2 compone
300712975	Type IV secretory pathway VirD4 protein-like p	Yes	3	plasmid	188910	190013	+		Halalkalicoccus_jeotgali_B3_uid50305	NC_014299	plasmid 2		arCOG00280	21 (-)	L		HerA helicase	0.00000002	pfam10412	Type IV secretion-system coupling protei
300712976	Type IV secretory pathway VirD4 protein-like p	No	0	plasmid	189998	190504	+		Halalkalicoccus_jeotgali_B3_uid50305	NC_014299	plasmid 2	clust_212						0.0000002	pfam12696	TraM recognition site of TraD and TraG.
===								5	Halalkalicoccus_jeotgali_B3_uid50305	NC_014299	plasmid 2									
300712993	hypothetical protein HacjB3_18358	No	1	plasmid	203922	204239	+		Halalkalicoccus_jeotgali_B3_uid50305	NC_014299	plasmid 2									
300712994	hypothetical protein HacjB3_18363	No	2	plasmid	206177	206377	+		Halalkalicoccus_jeotgali_B3_uid50305	NC_014299	plasmid 2									
300712995	hypothetical protein HacjB3_18368	Yes	5	plasmid	207355	208146	-		Halalkalicoccus_jeotgali_B3_uid50305	NC_014299	plasmid 2		arCOG06311	1 (-)	S		Uncharacterized conserved protein			
300712996	hypothetical protein HacjB3_18373	No	4	plasmid	208699	209061	+		Halalkalicoccus_jeotgali_B3_uid50305	NC_014299	plasmid 2		arCOG11369	1	S		Uncharacterized conserved protein			
300712997	hypothetical protein HacjB3_18378	No	3	plasmid	209281	210183	-		Halalkalicoccus_jeotgali_B3_uid50305	NC_014299	plasmid 2	arCOG02673	arCOG02673	2	U		OxaA/SpoJ/YigC translocase/secretase, sec-inde	5E-28	COG1422	Predicted membrane protein
===								6	Halalkalicoccus_jeotgali_B3_uid50305	NC_014299	plasmid 2									
300713108	hypothetical protein HacjB3_18948	No	0	plasmid	333747	333947	-		Halalkalicoccus_jeotgali_B3_uid50305	NC_014299	plasmid 2									
300713109	hypothetical protein HacjB3_18953	No	0	plasmid	334313	335371	+		Halalkalicoccus_jeotgali_B3_uid50305	NC_014299	plasmid 2		arCOG09176	8	S		Uncharacterized conserved protein			
300713110	hypothetical protein HacjB3_18958	Yes	0	plasmid	335361	335978	-		Halalkalicoccus_jeotgali_B3_uid50305	NC_014299	plasmid 2		arCOG10172	3 (-)	S		Uncharacterized conserved protein			
300713111	hypothetical protein HacjB3_18963	No	2	plasmid	336030	336272	-		Halalkalicoccus_jeotgali_B3_uid50305	NC_014299	plasmid 2									
300713112	hypothetical protein HacjB3_18968	No	0	plasmid	336363	336587	-		Halalkalicoccus_jeotgali_B3_uid50305	NC_014299	plasmid 2									
300713113	hypothetical protein HacjB3_18973	Yes	3	plasmid	336870	337247	+		Halalkalicoccus_jeotgali_B3_uid50305	NC_014299	plasmid 2									
===								7	Halalkalicoccus_jeotgali_B3_uid50305	NC_014300	plasmid 3									
299883414	hypothetical protein HacjB3_19193	No	2	plasmid	27737	28105	-		Halalkalicoccus_jeotgali_B3_uid50305	NC_014300	plasmid 3									
299883415	hypothetical protein HacjB3_19198	Yes	7	plasmid	28095	29621	-		Halalkalicoccus_jeotgali_B3_uid50305	NC_014300	plasmid 3		arCOG02043	1 (-)	M	AglB	Oligosaccharyltransferase membrane subunit			
299883416	hypothetical protein HacjB3_19203	No	3	plasmid	29633	30076	-		Halalkalicoccus_jeotgali_B3_uid50305	NC_014300	plasmid 3									
299883417	hypothetical protein HacjB3_19208	No	0	plasmid	30209	30454	+		Halalkalicoccus_jeotgali_B3_uid50305	NC_014300	plasmid 3									
299883418	hypothetical protein HacjB3_19213	No	2	plasmid	30447	30671	+		Halalkalicoccus_jeotgali_B3_uid50305	NC_014300	plasmid 3									
299883419	hypothetical protein HacjB3_19218	No	0	plasmid	30676	31998	+		Halalkalicoccus_jeotgali_B3_uid50305	NC_014300	plasmid 3		arCOG09176	8	S		Uncharacterized conserved protein			
299883420	hypothetical protein HacjB3_19223	No	0	plasmid	31995	32528	-		Halalkalicoccus_jeotgali_B3_uid50305	NC_014300	plasmid 3		arCOG10172	3 (-)	S		Uncharacterized conserved protein			
===								17	Halalkalicoccus_jeotgali_B3_uid50305	NC_014301	plasmid 4									
299883435	hypothetical protein HacjB3_19298	No	0	plasmid	6998	7363	-		Halalkalicoccus_jeotgali_B3_uid50305	NC_014301	plasmid 4	clust_292								
299883436	hypothetical protein HacjB3_19303	No	0	plasmid	7599	8156	-		Halalkalicoccus_jeotgali_B3_uid50305	NC_014301	plasmid 4		arCOG07773	5 (-)	S		SWIM zinc finger containing protein			
299883437	hypothetical protein HacjB3_19308	No	0	plasmid	8362	8481	+		Halalkalicoccus_jeotgali_B3_uid50305	NC_014301	plasmid 4	clust_509								
299883438	hypothetical protein HacjB3_19313	No	0	plasmid	8746	9315	-		Halalkalicoccus_jeotgali_B3_uid50305	NC_014301	plasmid 4	clust_185								
299883439	hypothetical protein HacjB3_19318	No	0	plasmid	9385	9537	+		Halalkalicoccus_jeotgali_B3_uid50305	NC_014301	plasmid 4	clust_497								
299883440	hypothetical protein HacjB3_19323	No	0	plasmid	9730	10182	+		Halalkalicoccus_jeotgali_B3_uid50305	NC_014301	plasmid 4	clust_233								
299883441	hypothetical protein HacjB3_19328	No	0	plasmid	10179	10277	+		Halalkalicoccus_jeotgali_B3_uid50305	NC_014301	plasmid 4	clust_515								
299883442	hypothetical protein HacjB3_19333	No	0	plasmid	10274	10945	+		Halalkalicoccus_jeotgali_B3_uid50305	NC_014301	plasmid 4		arCOG04609	2 (-)	S		Predicted lipoprotein	0.0000003	COG1196	Chromosome segregation ATPases
299883443	hypothetical protein HacjB3_19338	No	0	plasmid	11913	12416	+		Halalkalicoccus_jeotgali_B3_uid50305	NC_014301	plasmid 4	clust_213								
299883444	hypothetical protein HacjB3_19343	No	0	plasmid	12407	13693	-		Halalkalicoccus_jeotgali_B3_uid50305	NC_014301	plasmid 4		arCOG09176	8	S		Uncharacterized conserved protein			
299883445	hypothetical protein HacjB3_19348	No	2	plasmid	13708	13917	-		Halalkalicoccus_jeotgali_B3_uid50305	NC_014301	plasmid 4	clust_443								
299883446	hypothetical protein HacjB3_19353	No	2	plasmid	14453	14797	+		Halalkalicoccus_jeotgali_B3_uid50305	NC_014301	plasmid 4		arCOG10938	2	S		Uncharacterized conserved protein			
299883447	hypothetical protein HacjB3_19358	No	6	plasmid	15040	16251	+		Halalkalicoccus_jeotgali_B3_uid50305	NC_014301	plasmid 4	clust_82						0.00002	pfam05802	Enterobacterial EspB protein. EspB is a
299883448	transfer complex protein, putative	No	2	plasmid	16254	19532	+		Halalkalicoccus_jeotgali_B3_uid50305	NC_014301	plasmid 4	arCOG04035,a	arCOG04035	5 (-)	U	VirB4	Type IV secretory pathway, VirB4 component	2E-11	pfam12846	AAA-like domain. This family of domains
299883449	hypothetical protein HacjB3_19368	No	0	plasmid	21023	21529	+		Halalkalicoccus_jeotgali_B3_uid50305	NC_014301	plasmid 4	clust_211								
299883450	hypothetical protein HacjB3_19373	No	0	plasmid	21952	22470	+		Halalkalicoccus_jeotgali_B3_uid50305	NC_014301	plasmid 4		arCOG10343	6	S		Uncharacterized conserved protein			
299883451	relaxase/mobilization nuclease domain-contair	No	0	plasmid	22473	22997	+		Halalkalicoccus_jeotgali_B3_uid50305	NC_014301	plasmid 4		arCOG10214	7	S		Uncharacterized conserved protein			
===								12	Halalkalicoccus_jeotgali_B3_uid50305	NC_014302	plasmid 5									
299883475	hypothetical protein HacjB3_19498	No	4	plasmid	1057	1725	-		Halalkalicoccus_jeotgali_B3_uid50305	NC_014302	plasmid 5									
299883476	hypothetical protein HacjB3_19503	No	0	plasmid	1778	1963	-		Halalkalicoccus_jeotgali_B3_uid50305	NC_014302	plasmid 5									
299883477	hypothetical protein HacjB3_19508	No	0	plasmid	2301	2843	+		Halalkalicoccus_jeotgali_B3_uid50305	NC_014302	plasmid 5									
299883478	hypothetical protein HacjB3_19513	No	0	plasmid	3151	3351	+		Halalkalicoccus_jeotgali_B3_uid50305	NC_014302	plasmid 5									
299883479	hypothetical protein HacjB3_19518	No	0	plasmid	4051	5076	+		Halalkalicoccus_jeotgali_B3_uid50305	NC_014302	plasmid 5		arCOG10679	2	S		Uncharacterized conserved protein			
299883480	hypothetical protein HacjB3_19523	No	0	plasmid	5760	6575	+		Halalkalicoccus_jeotgali_B3_uid50305	NC_014302	plasmid 5		arCOG07807	3	S		Uncharacterized conserved protein			
299883481	hypothetical protein HacjB3_19528	No	0	plasmid	7227	9017	-		Halalkalicoccus_jeotgali_B3_uid50305	NC_014302	plasmid 5		arCOG06914	10 (-)	R		Phage or plasmid associated DNA primase	2E-23	COG3378	Phage associated DNA primase
299883482	hypothetical protein HacjB3_19533	No	0	plasmid	9125	9265	-		Halalkalicoccus_jeotgali_B3_uid50305	NC_014302	plasmid 5									
299883483	hypothetical protein HacjB3_19538	No	0	plasmid	9265	9423	-		Halalkalicoccus_jeotgali_B3_uid50305	NC_014302	plasmid 5									
299883484	hypothetical protein HacjB3_19543	No	0	plasmid	9497	11455	-		Halalkalicoccus_jeotgali_B3_uid50305	NC_014302	plasmid 5		arCOG04143	1 (-)	L		DNA topoisomerase VI, subunit A			
299883485	hypothetical protein HacjB3_19548	No	1	plasmid	11672	12172	+		Halalkalicoccus_jeotgali_B3_uid50305	NC_014302	plasmid 5									
299883486	hypothetical protein HacjB3_19553	No	1	plasmid	14544	14642	-		Halalkalicoccus_jeotgali_B3_uid50305	NC_014302	plasmid 5									
===								5	Halalkalicoccus_jeotgali_B3_uid50305	NC_014302	plasmid 5									
299883494	hypothetical protein HacjB3_19593	No	0	plasmid	20142	20369	-		Halalkalicoccus_jeotgali_B3_uid50305	NC_014302	plasmid 5		arCOG08906	1 (-)	V		Predicted antitoxins containing the HTH domain			
299883495	hypothetical protein HacjB3_19598	No	2	plasmid	21465	21959	-		Halalkalicoccus_jeotgali_B3_uid50305	NC_014302	plasmid 5									
299883496	hypothetical protein HacjB3_19603	No	0	plasmid	22029	22196	-		Halalkalicoccus_jeotgali_B3_uid50305	NC_014302	plasmid 5									
299883497	hypothetical protein HacjB3_19608	No	0	plasmid	22584	22793	-		Halalkalicoccus_jeotgali_B3_uid50305	NC_014302	plasmid 5									
299883498	hypothetical protein HacjB3_19613	No	1	plasmid	23092	23247	+		Halalkalicoccus_jeotgali_B3_uid50305	NC_014302	plasmid 5									
===								8	Halalkalicoccus_jeotgali_B3_uid50305	NC_014303	plasmid 6									
299883500	hypothetical protein HacjB3_19623	No	0	plasmid	2173	2907	+		Halalkalicoccus_jeotgali_B3_uid50305	NC_014303	plasmid 6									
299883501	hypothetical protein HacjB3_19628	No	0	plasmid	2904	3368	+		Halalkalicoccus_jeotgali_B3_uid50305	NC_014303	plasmid 6									
299883502	hypothetical protein HacjB3_19633	No	0	plasmid	3579	3860	+		Halalkalicoccus_jeotgali_B3_uid50305	NC_014303	plasmid 6		arCOG10959	8	S		Uncharacterized conserved protein			
299883503	hypothetical protein HacjB3_19638	No	0	plasmid	3857	4156	+		Halalkalicoccus_jeotgali_B3_uid50305	NC_014303	plasmid 6									
299883504	hypothetical protein HacjB3_19643	No	0	plasmid	5172	5414	-		Halalkalicoccus_jeotgali_B3_uid50305	NC_014303	plasmid 6									
299883505	hypothetical protein HacjB3_19648	No	0	plasmid	5584	5895	+		Halalkalicoccus_jeotgali_B3_uid50305	NC_014303	plasmid 6									
299883506	hypothetical protein HacjB3_19653	No	1	plasmid	5995	6138	-		Halalkalicoccus_jeotgali_B3_uid50305	NC_014303	plasmid 6									
299883507	hypothetical protein HacjB3_19618	No	0	plasmid	6663	503	+		Halalkalicoccus_jeotgali_B3_uid50305	NC_014303	plasmid 6							0.000005	COG1196	Chromosome segregation ATPases
===								9	Haloarcula_hispanica_ATCC_33960_uid72475	NC_015943	chromosome II									
344209510	hypothetical protein HAH_4028	No	2		26868	27074	-		Haloarcula_hispanica_ATCC_33960_uid72475	NC_015943	chromosome II									
344209511	hypothetical protein HAH_4029	No	0		27090	27293	-		Haloarcula_hispanica_ATCC_33960_uid72475	NC_015943	chromosome II		arCOG00471	1 (-)	L	CDC6	Cdc6-related protein, AAA superfamily ATPase			
344209512	glyoxalase/bleomycin resistance protein/dioxy	No	0		27566	27892	-		Haloarcula_hispanica_ATCC_33960_uid72475	NC_015943	chromosome II		arCOG10673	1	S		Uncharacterized conserved protein			
344209513	hypothetical protein HAH_4031	No	0		28423	28683	-		Haloarcula_hispanica_ATCC_33960_uid72475	NC_015943	chromosome II	clust_387								
344209514	hypothetical protein HAH_4032	No	0		28769	29014	+		Haloarcula_hispanica_ATCC_33960_uid72475	NC_015943	chromosome II	clust_57								
344209515	hypothetical protein HAH_4033	No	0		29328	30257	-		Haloarcula_hispanica_ATCC_33960_uid72475	NC_015943	chromosome II		arCOG09239	2 (-)	T		GAF domain-containing protein fused to SAM (St	0.00001	COG1196	Chromosome segregation ATPases
344209516	hypothetical protein HAH_4034	No	0		30475	30687	-		Haloarcula_hispanica_ATCC_33960_uid72475	NC_015943	chromosome II		arCOG07540	2	S		Uncharacterized conserved protein			
344209517	hypothetical protein HAH_4035	No	0		30687	30986	-		Haloarcula_hispanica_ATCC_33960_uid72475	NC_015943	chromosome II	clust_350								
344209518	hypothetical protein HAH_4036	No	0		30983	32158	-		Haloarcula_hispanica_ATCC_33960_uid72475	NC_015943	chromosome II		arCOG10908	2	S		Uncharacterized conserved protein			
===								5	Haloarcula_hispanica_ATCC_33960_uid72475	NC_015943	chromosome II									
344209554	hypothetical protein HAH_4072	No	0		62276	62734	-		Haloarcula_hispanica_ATCC_33960_uid72475	NC_015943	chromosome II		arCOG09163	1	S		Uncharacterized conserved protein			
344209555	hypothetical protein HAH_4073	No	0		62956	63093	+		Haloarcula_hispanica_ATCC_33960_uid72475	NC_015943	chromosome II	clust_63								
344209556	hypothetical protein HAH_4074	No	0		63090	63458	+		Haloarcula_hispanica_ATCC_33960_uid72475	NC_015943	chromosome II									
344209557	hypothetical protein HAH_4075	No	0		63591	64079	+		Haloarcula_hispanica_ATCC_33960_uid72475	NC_015943	chromosome II		arCOG08892	1 (-)	S		Uncharacterized conserved protein			
344209558	hypothetical protein HAH_4076	Yes	1		64218	64943	-		Haloarcula_hispanica_ATCC_33960_uid72475	NC_015943	chromosome II									
===								10	Haloarcula_hispanica_ATCC_33960_uid72475	NC_015943	chromosome II									
344209585	hypothetical protein HAH_4103	No	0		92279	93124	+		Haloarcula_hispanica_ATCC_33960_uid72475	NC_015943	chromosome II		arCOG03726	4 (-)	S		Uncharacterized conserved protein			
344209586	hypothetical protein HAH_4104	No	0		93226	93906	+		Haloarcula_hispanica_ATCC_33960_uid72475	NC_015943	chromosome II		arCOG10879	5	S		Uncharacterized conserved protein			
344209587	hypothetical protein HAH_4105	No	0		93942	94352	+		Haloarcula_hispanica_ATCC_33960_uid72475	NC_015943	chromosome II		arCOG10879	5	S		Uncharacterized conserved protein			
344209588	hypothetical protein HAH_4106	No	0		95392	95508	+		Haloarcula_hispanica_ATCC_33960_uid72475	NC_015943	chromosome II									
344209589	hypothetical protein HAH_4107	No	0		95684	95974	+		Haloarcula_hispanica_ATCC_33960_uid72475	NC_015943	chromosome II	clust_359								
344209590	hypothetical protein HAH_4108	No	0		95971	96285	+		Haloarcula_hispanica_ATCC_33960_uid72475	NC_015943	chromosome II	clust_340								
344209591	hypothetical protein HAH_4109	No	0		96479	96718	+		Haloarcula_hispanica_ATCC_33960_uid72475	NC_015943	chromosome II		arCOG06176	2 (-)	S		Uncharacterized conserved protein			
344209592	hypothetical protein HAH_4110	No	0		96776	97141	+		Haloarcula_hispanica_ATCC_33960_uid72475	NC_015943	chromosome II	clust_294								
344209593	hypothetical protein HAH_4111	No	0		97375	97578	+		Haloarcula_hispanica_ATCC_33960_uid72475	NC_015943	chromosome II	clust_452								
344209594	hypothetical protein HAH_4112	No	0		97571	97786	+		Haloarcula_hispanica_ATCC_33960_uid72475	NC_015943	chromosome II	clust_436								
===								11	Haloarcula_hispanica_ATCC_33960_uid72475	NC_015943	chromosome II									
344209620	hypothetical protein HAH_4138	No	0		116756	116971	-		Haloarcula_hispanica_ATCC_33960_uid72475	NC_015943	chromosome II	clust_436								
344209621	hypothetical protein HAH_4139	No	0		116964	117167	-		Haloarcula_hispanica_ATCC_33960_uid72475	NC_015943	chromosome II	clust_452								
344209622	hypothetical protein HAH_4140	No	0		117401	117766	-		Haloarcula_hispanica_ATCC_33960_uid72475	NC_015943	chromosome II	clust_294								
344209623	hypothetical protein HAH_4141	No	0		117824	118063	-		Haloarcula_hispanica_ATCC_33960_uid72475	NC_015943	chromosome II		arCOG06176	2 (-)	S		Uncharacterized conserved protein			
344209624	hypothetical protein HAH_4142	No	0		118257	118571	-		Haloarcula_hispanica_ATCC_33960_uid72475	NC_015943	chromosome II	clust_340								
344209625	hypothetical protein HAH_4143	No	0		118568	118858	-		Haloarcula_hispanica_ATCC_33960_uid72475	NC_015943	chromosome II	clust_359								
344209626	hypothetical protein HAH_4144	No	0		119328	119855	-		Haloarcula_hispanica_ATCC_33960_uid72475	NC_015943	chromosome II									
344209627	hypothetical protein HAH_4145	No	0		119852	120619	-		Haloarcula_hispanica_ATCC_33960_uid72475	NC_015943	chromosome II									
344209628	hypothetical protein HAH_4146	No	0		120718	121122	-		Haloarcula_hispanica_ATCC_33960_uid72475	NC_015943	chromosome II		arCOG10879	5	S		Uncharacterized conserved protein			
344209629	hypothetical protein HAH_4147	No	0		121158	121838	-		Haloarcula_hispanica_ATCC_33960_uid72475	NC_015943	chromosome II		arCOG10879	5	S		Uncharacterized conserved protein			
344209630	hypothetical protein HAH_4148	No	0		121939	122784	-		Haloarcula_hispanica_ATCC_33960_uid72475	NC_015943	chromosome II		arCOG03726	4 (-)	S		Uncharacterized conserved protein			
===								6	Haloarcula_hispanica_ATCC_33960_uid72475	NC_015943	chromosome II									
344209663	ribbon-helix-helix protein	No	0		161782	162159	+		Haloarcula_hispanica_ATCC_33960_uid72475	NC_015943	chromosome II	clust_283								
344209664	ribbon-helix-helix protein	No	0		162156	162629	+		Haloarcula_hispanica_ATCC_33960_uid72475	NC_015943	chromosome II		arCOG10343	6	S		Uncharacterized conserved protein			
344209665	relaxase/mobilization nuclease domain-contair	No	0		162629	163381	+		Haloarcula_hispanica_ATCC_33960_uid72475	NC_015943	chromosome II		arCOG10214	7	S		Uncharacterized conserved protein			
344209666	putative conjugative transfer protein TraD	No	4		163378	165594	+		Haloarcula_hispanica_ATCC_33960_uid72475	NC_015943	chromosome II	arCOG02841,a	arCOG02841	4 (-)	V	MdlB	ABC-type multidrug transport system, ATPase an	6E-18	pfam10412	Type IV secretion-system coupling protei ; Nop14-like family.
344209667	hypothetical protein HAH_4185	No	0		166686	167069	+		Haloarcula_hispanica_ATCC_33960_uid72475	NC_015943	chromosome II	clust_278								
344209668	hypothetical protein HAH_4186	No	0		167806	168828	+		Haloarcula_hispanica_ATCC_33960_uid72475	NC_015943	chromosome II		arCOG09152	2	S		Uncharacterized conserved protein	0.00002	COG0419	ATPase involved in DNA repair
===								5	Haloarcula_hispanica_ATCC_33960_uid72475	NC_015943	chromosome II									
344209674	hypothetical protein HAH_4192	No	0		174661	174798	-		Haloarcula_hispanica_ATCC_33960_uid72475	NC_015943	chromosome II	clust_63								
344209675	hypothetical protein HAH_4193	No	0		175469	175591	-		Haloarcula_hispanica_ATCC_33960_uid72475	NC_015943	chromosome II									
344209676	hypothetical protein HAH_4194	No	0		175847	176524	+		Haloarcula_hispanica_ATCC_33960_uid72475	NC_015943	chromosome II		arCOG10882	2 (-)	S		Uncharacterized conserved protein			
344209677	hypothetical protein HAH_4195	No	0		177011	177148	-		Haloarcula_hispanica_ATCC_33960_uid72475	NC_015943	chromosome II	clust_63								
344209678	hypothetical protein HAH_4196	No	0		177394	177543	-		Haloarcula_hispanica_ATCC_33960_uid72475	NC_015943	chromosome II									
===								12	Haloarcula_hispanica_ATCC_33960_uid72475	NC_015943	chromosome II									
344209681	hypothetical protein HAH_4199	No	0		182095	182220	+		Haloarcula_hispanica_ATCC_33960_uid72475	NC_015943	chromosome II									
344209682	putative ISH4 transposase	No	0		182224	183108	-		Haloarcula_hispanica_ATCC_33960_uid72475	NC_015943	chromosome II		arCOG02132	5 (-)	L		Transposase	2E-16	pfam12762	ISXO2-like transposase domain. This doma ; Transposase and
344209683	hypothetical protein HAH_4201	No	0		183661	183795	+		Haloarcula_hispanica_ATCC_33960_uid72475	NC_015943	chromosome II									
344209684	DNA/RNA helicase, superfamily I	No	0		184188	186350	+		Haloarcula_hispanica_ATCC_33960_uid72475	NC_015943	chromosome II	clust_67						9E-62	COG3972	Superfamily I DNA and RNA helicases
344209685	hypothetical protein HAH_4203	No	3		186651	188474	-		Haloarcula_hispanica_ATCC_33960_uid72475	NC_015943	chromosome II									
344209686	hypothetical protein HAH_4204	No	0		188791	189054	-		Haloarcula_hispanica_ATCC_33960_uid72475	NC_015943	chromosome II									
344209687	hypothetical protein HAH_4205	No	0		189051	189380	-		Haloarcula_hispanica_ATCC_33960_uid72475	NC_015943	chromosome II	clust_317								
344209688	hypothetical protein HAH_4206	No	2		189414	189977	+		Haloarcula_hispanica_ATCC_33960_uid72475	NC_015943	chromosome II									
344209689	hypothetical protein HAH_4207	No	0		190008	190286	+		Haloarcula_hispanica_ATCC_33960_uid72475	NC_015943	chromosome II		arCOG10909	4	S		Uncharacterized conserved protein			
344209690	hypothetical protein HAH_4208	No	0		190567	191487	+		Haloarcula_hispanica_ATCC_33960_uid72475	NC_015943	chromosome II		arCOG09239	2 (-)	T		GAF domain-containing protein fused to SAM (Sterile alpha motif) domain			
344209691	hypothetical protein HAH_4209	No	0		191812	192057	-		Haloarcula_hispanica_ATCC_33960_uid72475	NC_015943	chromosome II	clust_57								
344209692	hypothetical protein HAH_4210	No	0		192143	192403	+		Haloarcula_hispanica_ATCC_33960_uid72475	NC_015943	chromosome II	clust_387								
===								9	Haloarcula_hispanica_ATCC_33960_uid72475	NC_015943	chromosome II									
344209729	transposase (ISH3)	No	0		232938	234131	+		Haloarcula_hispanica_ATCC_33960_uid72475	NC_015943	chromosome II		arCOG06160	11 (-)	L		Transposase			
344209730	hypothetical protein HAH_4252	No	0		234267	234812	-		Haloarcula_hispanica_ATCC_33960_uid72475	NC_015943	chromosome II									
344209731	hypothetical protein HAH_4253	No	0		234814	235968	-		Haloarcula_hispanica_ATCC_33960_uid72475	NC_015943	chromosome II		arCOG10906	5	S		Uncharacterized conserved protein			
344209732	hypothetical protein HAH_4254	No	0		236243	236506	-		Haloarcula_hispanica_ATCC_33960_uid72475	NC_015943	chromosome II									
344209733	hypothetical protein HAH_4255	No	0		236917	237246	-		Haloarcula_hispanica_ATCC_33960_uid72475	NC_015943	chromosome II							1E-12	pfam11429	Colicin D. Colicin D is a tRNase which k
344209734	hypothetical protein HAH_4256	No	0		237499	238128	-		Haloarcula_hispanica_ATCC_33960_uid72475	NC_015943	chromosome II		arCOG10906	5	S		Uncharacterized conserved protein			
344209735	hypothetical protein HAH_4257	No	0		238187	239302	-		Haloarcula_hispanica_ATCC_33960_uid72475	NC_015943	chromosome II		arCOG09139	5	S		Uncharacterized conserved protein			
344209736	putative calcium-binding protein	No	0		239305	241293	-		Haloarcula_hispanica_ATCC_33960_uid72475	NC_015943	chromosome II		arCOG10906	5	S		Uncharacterized conserved protein			
344209737	hypothetical protein HAH_4259	No	0		241284	241739	-		Haloarcula_hispanica_ATCC_33960_uid72475	NC_015943	chromosome II		arCOG02129	2 (-)	L		Transposase	2E-18	pfam13358	DDE superfamily endonuclease. This famil
===								6	Haloarcula_hispanica_ATCC_33960_uid72475	NC_015943	chromosome II									
344209906	hypothetical protein HAH_4428	No	2		447279	447773	+		Haloarcula_hispanica_ATCC_33960_uid72475	NC_015943	chromosome II		arCOG08111	1 (-)	S		Uncharacterized conserved protein			
344209907	hypothetical protein HAH_4429	No	0		447906	448958	+		Haloarcula_hispanica_ATCC_33960_uid72475	NC_015943	chromosome II		arCOG07572	1	S		Uncharacterized conserved protein			
344209908	hypothetical protein HAH_4430	No	0		449645	453826	+		Haloarcula_hispanica_ATCC_33960_uid72475	NC_015943	chromosome II	clust_10								
344209909	hypothetical protein HAH_4431	No	0		453848	454165	+		Haloarcula_hispanica_ATCC_33960_uid72475	NC_015943	chromosome II	clust_336								
344209910	hypothetical protein HAH_4432	No	0		454189	454443	-		Haloarcula_hispanica_ATCC_33960_uid72475	NC_015943	chromosome II	clust_22								
344209911	hypothetical protein HAH_4433	No	0		454645	455871	-		Haloarcula_hispanica_ATCC_33960_uid72475	NC_015943	chromosome II		arCOG09140	1 (-)	S		Uncharacterized conserved protein	6E-160	pfam06792	Uncharacterized protein family (UPF0261)
===								5	Haloarcula_hispanica_ATCC_33960_uid72475	NC_015948	chromosome I									
344211966	hypothetical protein HAH_1695	No	0		1644369	1644653	+		Haloarcula_hispanica_ATCC_33960_uid72475	NC_015948	chromosome I									
344211967	site-specific recombinase XerD	No	0		1644650	1645567	+		Haloarcula_hispanica_ATCC_33960_uid72475	NC_015948	chromosome I		arCOG01241	31 (-)	L	XerC	Integrase	5E-26	COG4974	Site-specific recombinase XerD
344211968	hypothetical protein HAH_1697	Yes	0		1645549	1645740	+		Haloarcula_hispanica_ATCC_33960_uid72475	NC_015948	chromosome I									
344211969	hypothetical protein HAH_1698	No	0		1645770	1646084	-		Haloarcula_hispanica_ATCC_33960_uid72475	NC_015948	chromosome I									
344211970	hypothetical protein HAH_1699	No	0		1646081	1646377	-		Haloarcula_hispanica_ATCC_33960_uid72475	NC_015948	chromosome I									
===								5	Haloarcula_hispanica_ATCC_33960_uid72475	NC_015948	chromosome I									
344212179	hypothetical protein HAH_1912	No	0		1854360	1854494	+		Haloarcula_hispanica_ATCC_33960_uid72475	NC_015948	chromosome I		arCOG10862	2	S		Uncharacterized conserved protein			
344212180	hypothetical protein HAH_1913	Yes	0		1854554	1855771	-		Haloarcula_hispanica_ATCC_33960_uid72475	NC_015948	chromosome I		arCOG04512	2 (-)	P		Predicted iron-dependent peroxidase			
344212181	hypothetical protein HAH_1914	No	1		1855868	1856017	+		Haloarcula_hispanica_ATCC_33960_uid72475	NC_015948	chromosome I	clust_499								
344212182	hypothetical protein HAH_1915	No	2		1856017	1856292	+		Haloarcula_hispanica_ATCC_33960_uid72475	NC_015948	chromosome I	clust_370								
344212183	hypothetical protein HAH_1916	Yes	2		1856285	1856518	+		Haloarcula_hispanica_ATCC_33960_uid72475	NC_015948	chromosome I	clust_413								
===								18	Haloarcula_marismortui_ATCC_43049_uid57719	NC_006389	plasmid pNG100									
55376108	hypothetical protein pNG1001	No	4 YES	plasmid	167	817	-		Haloarcula_marismortui_ATCC_43049_uid57719	NC_006389	plasmid pNG100		arCOG06152	1 (-)	S		Uncharacterized conserved protein			
55376109	hypothetical protein pNG1002	No	0 YES	plasmid	824	1456	-		Haloarcula_marismortui_ATCC_43049_uid57719	NC_006389	plasmid pNG100									
55376110	hypothetical protein pNG1003	No	0 YES	plasmid	1960	2172	-		Haloarcula_marismortui_ATCC_43049_uid57719	NC_006389	plasmid pNG100									
55376111	hypothetical protein pNG1004	No	0 YES	plasmid	3779	4057	-		Haloarcula_marismortui_ATCC_43049_uid57719	NC_006389	plasmid pNG100									
55376112	hypothetical protein pNG1005	No	0 YES	plasmid	4121	4849	-		Haloarcula_marismortui_ATCC_43049_uid57719	NC_006389	plasmid pNG100									
55376113	hypothetical protein pNG1006	No	0 YES	plasmid	5143	5688	+		Haloarcula_marismortui_ATCC_43049_uid57719	NC_006389	plasmid pNG100									
55376114	hypothetical protein pNG1007	No	1 YES	plasmid	6009	6350	+		Haloarcula_marismortui_ATCC_43049_uid57719	NC_006389	plasmid pNG100									
55376115	hypothetical protein pNG1008	No	0 YES	plasmid	7033	7386	-		Haloarcula_marismortui_ATCC_43049_uid57719	NC_006389	plasmid pNG100									
55376116	hypothetical protein pNG1009	No	0	plasmid	7660	8523	-		Haloarcula_marismortui_ATCC_43049_uid57719	NC_006389	plasmid pNG100		arCOG01510	2 (-)	L	RFA1	Single-stranded DNA-binding replication protein A (RPA), large (70 kD) subunit or related ssDNA-binding protein			
55376117	hypothetical protein pNG1011	No	0	plasmid	9116	9487	+		Haloarcula_marismortui_ATCC_43049_uid57719	NC_006389	plasmid pNG100		arCOG04493	1 (-)	V		PIN domain containing protein			
55376118	hypothetical protein pNG1012	No	0	plasmid	10248	10985	+		Haloarcula_marismortui_ATCC_43049_uid57719	NC_006389	plasmid pNG100									
55376119	hypothetical protein pNG1013	Yes	3	plasmid	11066	11311	-		Haloarcula_marismortui_ATCC_43049_uid57719	NC_006389	plasmid pNG100									
55376120	hypothetical protein pNG1014	No	1	plasmid	11311	11520	-		Haloarcula_marismortui_ATCC_43049_uid57719	NC_006389	plasmid pNG100									
55376122	transcription regulator	No	0	plasmid	11675	12016	-		Haloarcula_marismortui_ATCC_43049_uid57719	NC_006389	plasmid pNG100	clust_310								
55376121	hypothetical protein pNG1016	No	0	plasmid	11849	12424	+		Haloarcula_marismortui_ATCC_43049_uid57719	NC_006389	plasmid pNG100									
55376123	hypothetical protein pNG1017	No	0	plasmid	12472	14019	-		Haloarcula_marismortui_ATCC_43049_uid57719	NC_006389	plasmid pNG100	clust_26								
55376124	hypothetical protein pNG1018	No	0	plasmid	14550	14936	+		Haloarcula_marismortui_ATCC_43049_uid57719	NC_006389	plasmid pNG100	clust_263								
55376125	hypothetical protein pNG1019	No	0	plasmid	15086	15238	+		Haloarcula_marismortui_ATCC_43049_uid57719	NC_006389	plasmid pNG100		arCOG04494	2 (-)	S		Uncharacterized conserved protein			
===								11	Haloarcula_marismortui_ATCC_43049_uid57719	NC_006389	plasmid pNG100									
55376132	flagellin A protein	No	1	plasmid	22035	23426	+		Haloarcula_marismortui_ATCC_43049_uid57719	NC_006389	plasmid pNG100		arCOG01829	3 (-)	N	FlaB	Archaeal flagellins	5E-18	pfam01917	Archaebacterial flagellin. Members of th ; Archaeal flagellins
55376133	hypothetical protein pNG1027	No	0 YES	plasmid	23766	24896	-		Haloarcula_marismortui_ATCC_43049_uid57719	NC_006389	plasmid pNG100		arCOG07967	1	S		Uncharacterized protein			
55376134	hypothetical protein pNG1030	No	0 YES	plasmid	25470	25748	-		Haloarcula_marismortui_ATCC_43049_uid57719	NC_006389	plasmid pNG100									
55376135	hypothetical protein pNG1028	No	0 YES	plasmid	25741	25881	-		Haloarcula_marismortui_ATCC_43049_uid57719	NC_006389	plasmid pNG100									
55376136	hypothetical protein pNG1029	No	0 YES	plasmid	25871	26083	-		Haloarcula_marismortui_ATCC_43049_uid57719	NC_006389	plasmid pNG100									
55376137	hypothetical protein pNG1032	No	0 YES	plasmid	26080	26586	-		Haloarcula_marismortui_ATCC_43049_uid57719	NC_006389	plasmid pNG100									
55376138	hypothetical protein pNG1033	No	0 YES	plasmid	26587	27087	-		Haloarcula_marismortui_ATCC_43049_uid57719	NC_006389	plasmid pNG100									
55376139	hypothetical protein pNG1034	No	0 YES	plasmid	27077	27205	-		Haloarcula_marismortui_ATCC_43049_uid57719	NC_006389	plasmid pNG100									
55376140	hypothetical protein pNG1035	No	0 YES	plasmid	27202	27519	-		Haloarcula_marismortui_ATCC_43049_uid57719	NC_006389	plasmid pNG100	clust_332								
55376141	hypothetical protein pNG1039	No	0 YES	plasmid	27746	29482	-		Haloarcula_marismortui_ATCC_43049_uid57719	NC_006389	plasmid pNG100		arCOG10909	4	S		Uncharacterized conserved protein			
55376142	putative helicase	No	0 YES	plasmid	30098	32686	+		Haloarcula_marismortui_ATCC_43049_uid57719	NC_006389	plasmid pNG100		arCOG00874	2 (-)	K	xpb	ssDNA-dependent ATPase, helicase superfamily II			
===								17	Haloarcula_marismortui_ATCC_43049_uid57719	NC_006390	plasmid pNG200									
55376159	ParB-like nuclease	No	0 YES	plasmid	9464	10045	-		Haloarcula_marismortui_ATCC_43049_uid57719	NC_006390	plasmid pNG200									
55376160	hypothetical protein pNG2017	No	0 YES	plasmid	10267	10479	-		Haloarcula_marismortui_ATCC_43049_uid57719	NC_006390	plasmid pNG200									
55376161	60S ribosomal protein L36-like protein	No	1	plasmid	10774	11718	-		Haloarcula_marismortui_ATCC_43049_uid57719	NC_006390	plasmid pNG200		arCOG02471	1 (-)	R		Predicted acyltransferase	0.00002	pfam04519	Polymer-forming cytoskeletal. This is a
55376162	hypothetical protein pNG2019	Yes	3	plasmid	11885	13003	-		Haloarcula_marismortui_ATCC_43049_uid57719	NC_006390	plasmid pNG200	clust_80						0.000002	pfam06682	Protein of unknown function (DUF1183). T
55376163	hypothetical protein pNG2020	Yes	1	plasmid	13082	13684	-		Haloarcula_marismortui_ATCC_43049_uid57719	NC_006390	plasmid pNG200		arCOG11334	5	S		Uncharacterized conserved protein			
55376164	hypothetical protein pNG2021	No	1	plasmid	13858	14577	+		Haloarcula_marismortui_ATCC_43049_uid57719	NC_006390	plasmid pNG200		arCOG11334	5	S		Uncharacterized conserved protein			
55376165	pNRC100 replication protein H-like	No	0	plasmid	14675	17527	-		Haloarcula_marismortui_ATCC_43049_uid57719	NC_006390	plasmid pNG200		arCOG04495	4 (-)	L	RepH	Plasmid replication protein, contains HTH domain			
55376166	hypothetical protein pNG2023	No	0	plasmid	17837	18160	-		Haloarcula_marismortui_ATCC_43049_uid57719	NC_006390	plasmid pNG200	clust_322								
55376167	hypothetical protein pNG2024	No	0	plasmid	18132	18386	-		Haloarcula_marismortui_ATCC_43049_uid57719	NC_006390	plasmid pNG200		arCOG06155	6	S		Uncharacterized conserved protein			
55376168	hypothetical protein pNG2025	No	0	plasmid	18383	19003	-		Haloarcula_marismortui_ATCC_43049_uid57719	NC_006390	plasmid pNG200									
55376169	hypothetical protein pNG2026	No	0	plasmid	18993	19211	-		Haloarcula_marismortui_ATCC_43049_uid57719	NC_006390	plasmid pNG200									
55376170	hypothetical protein pNG2027	No	0	plasmid	19208	19525	-		Haloarcula_marismortui_ATCC_43049_uid57719	NC_006390	plasmid pNG200	clust_332								
55376171	hypothetical protein pNG2028	No	0	plasmid	19751	20113	-		Haloarcula_marismortui_ATCC_43049_uid57719	NC_006390	plasmid pNG200		arCOG10909	4	S		Uncharacterized conserved protein			
55376172	hypothetical protein pNG2029	No	0	plasmid	20168	20557	+		Haloarcula_marismortui_ATCC_43049_uid57719	NC_006390	plasmid pNG200									
55376173	hypothetical protein pNG2030	No	0	plasmid	20714	21043	+		Haloarcula_marismortui_ATCC_43049_uid57719	NC_006390	plasmid pNG200	clust_317								
55376174	hypothetical protein pNG2032	No	0	plasmid	21810	22367	+		Haloarcula_marismortui_ATCC_43049_uid57719	NC_006390	plasmid pNG200		arCOG10879	5	S		Uncharacterized conserved protein			
55376175	putative membrane-bound metal-dependent hy	No	4	plasmid	22596	23081	-		Haloarcula_marismortui_ATCC_43049_uid57719	NC_006390	plasmid pNG200		arCOG01744	1 (-)	R		Membrane-bound metal-dependent hydrolase	0.000000004	pfam04307	Predicted membrane-bound metal-dependent
===								8	Haloarcula_marismortui_ATCC_43049_uid57719	NC_006390	plasmid pNG200									
55376179	hypothetical protein pNG2037	No	0	plasmid	28017	28121	-		Haloarcula_marismortui_ATCC_43049_uid57719	NC_006390	plasmid pNG200									
55376180	hypothetical protein pNG2038	No	0	plasmid	28125	29099	-		Haloarcula_marismortui_ATCC_43049_uid57719	NC_006390	plasmid pNG200		arCOG07538	2 (-)	S		Uncharacterized conserved protein			
55376181	ribbon-helix-helix protein	No	0	plasmid	29568	30272	-		Haloarcula_marismortui_ATCC_43049_uid57719	NC_006390	plasmid pNG200							4E-13	COG1196	Chromosome segregation ATPases
55376182	hypothetical protein pNG2040	No	0	plasmid	30540	30683	-		Haloarcula_marismortui_ATCC_43049_uid57719	NC_006390	plasmid pNG200									
55376183	hypothetical protein pNG2041	No	0	plasmid	30767	30913	+		Haloarcula_marismortui_ATCC_43049_uid57719	NC_006390	plasmid pNG200									
55376184	transcription regulator	No	0	plasmid	30963	31277	-		Haloarcula_marismortui_ATCC_43049_uid57719	NC_006390	plasmid pNG200	clust_310								
55376185	hypothetical protein pNG2043	No	0	plasmid	31422	31691	+		Haloarcula_marismortui_ATCC_43049_uid57719	NC_006390	plasmid pNG200									
55376186	hypothetical protein pNG2044	No	0	plasmid	31733	33280	-		Haloarcula_marismortui_ATCC_43049_uid57719	NC_006390	plasmid pNG200	clust_26								
===								22	Haloarcula_marismortui_ATCC_43049_uid57719	NC_006391	plasmid pNG300									
55376189	hypothetical protein pNG3005	No	2	plasmid	4437	4742	+		Haloarcula_marismortui_ATCC_43049_uid57719	NC_006391	plasmid pNG300									
55376190	transcription regulator	No	0	plasmid	4742	5065	+		Haloarcula_marismortui_ATCC_43049_uid57719	NC_006391	plasmid pNG300		arCOG00002	1 (-)	K		Predicted transcriptional regulator, PadR family			
55376191	hypothetical protein pNG3008	No	5	plasmid	5275	5808	+		Haloarcula_marismortui_ATCC_43049_uid57719	NC_006391	plasmid pNG300									
55376192	hypothetical protein pNG3009	No	1	plasmid	5805	5942	-		Haloarcula_marismortui_ATCC_43049_uid57719	NC_006391	plasmid pNG300	clust_502								
55376193	helix-turn-helix motif-containing DNA-binding pr	No	0	plasmid	5972	6142	+		Haloarcula_marismortui_ATCC_43049_uid57719	NC_006391	plasmid pNG300									
55376194	hypothetical protein pNG3011	No	0	plasmid	7360	7503	-		Haloarcula_marismortui_ATCC_43049_uid57719	NC_006391	plasmid pNG300		arCOG07894	2 (-)	S		Uncharacterized protein			
55376195	hypothetical protein pNG3012	No	0	plasmid	7870	8991	+		Haloarcula_marismortui_ATCC_43049_uid57719	NC_006391	plasmid pNG300		arCOG10971	1	S		Uncharacterized conserved protein			
55376196	cell division control protein 6-like	No	0	plasmid	9079	10134	+		Haloarcula_marismortui_ATCC_43049_uid57719	NC_006391	plasmid pNG300		arCOG00467	13 (-)	L	CDC6	Cdc6-related protein, AAA superfamily ATPase	1E-31	COG1474	Cdc6-related protein, AAA superfamily AT
55376197	hypothetical protein pNG3014	No	0	plasmid	10299	10469	+		Haloarcula_marismortui_ATCC_43049_uid57719	NC_006391	plasmid pNG300									
55376198	hypothetical protein pNG3015	No	0	plasmid	10470	10937	-		Haloarcula_marismortui_ATCC_43049_uid57719	NC_006391	plasmid pNG300		arCOG07766	2	S		Uncharacterized conserved protein			
55376199	hypothetical protein pNG3017	No	0	plasmid	11102	12847	+		Haloarcula_marismortui_ATCC_43049_uid57719	NC_006391	plasmid pNG300		arCOG10909	4	S		Uncharacterized conserved protein			
55376200	hypothetical protein pNG3018	No	0	plasmid	12849	12983	+		Haloarcula_marismortui_ATCC_43049_uid57719	NC_006391	plasmid pNG300									
55376201	hypothetical protein pNG3019	No	0	plasmid	12980	13498	+		Haloarcula_marismortui_ATCC_43049_uid57719	NC_006391	plasmid pNG300									
55376202	hypothetical protein pNG3021	No	0	plasmid	13770	14126	+		Haloarcula_marismortui_ATCC_43049_uid57719	NC_006391	plasmid pNG300									
55376203	hypothetical protein pNG3023	No	0	plasmid	14123	14683	+		Haloarcula_marismortui_ATCC_43049_uid57719	NC_006391	plasmid pNG300									
55376204	hypothetical protein pNG3024	No	0	plasmid	14676	14909	+		Haloarcula_marismortui_ATCC_43049_uid57719	NC_006391	plasmid pNG300									
55376205	hypothetical protein pNG3025	No	0	plasmid	14902	15462	+		Haloarcula_marismortui_ATCC_43049_uid57719	NC_006391	plasmid pNG300									
55376206	hypothetical protein pNG3026	No	0	plasmid	15452	15655	+		Haloarcula_marismortui_ATCC_43049_uid57719	NC_006391	plasmid pNG300									
55376207	hypothetical protein pNG3027	No	0	plasmid	15757	15912	+		Haloarcula_marismortui_ATCC_43049_uid57719	NC_006391	plasmid pNG300		arCOG06155	6	S		Uncharacterized conserved protein			
55376208	hypothetical protein pNG3028	No	0	plasmid	15884	16207	+		Haloarcula_marismortui_ATCC_43049_uid57719	NC_006391	plasmid pNG300	clust_322								
55376209	pNRC100 replication protein H-like	No	0	plasmid	16660	20064	+		Haloarcula_marismortui_ATCC_43049_uid57719	NC_006391	plasmid pNG300		arCOG04495	4 (-)	L	RepH	Plasmid replication protein, contains HTH domain			
55376210	hypothetical protein pNG3030	No	0	plasmid	21773	21979	+		Haloarcula_marismortui_ATCC_43049_uid57719	NC_006391	plasmid pNG300									
===								13	Haloarcula_marismortui_ATCC_43049_uid57719	NC_006391	plasmid pNG300									
55376213	putative plasmid partitioning protein Soj	No	0	plasmid	27132	28001	+		Haloarcula_marismortui_ATCC_43049_uid57719	NC_006391	plasmid pNG300		arCOG00586	8 (-)	D	Soj	ATPase involved in chromosome partitioning, Pa	3E-26	COG1192	ATPases involved in chromosome partition
55376214	hypothetical protein pNG3039	No	0	plasmid	27994	28464	+		Haloarcula_marismortui_ATCC_43049_uid57719	NC_006391	plasmid pNG300									
55376215	hypothetical protein pNG3040	No	0	plasmid	28475	28876	-		Haloarcula_marismortui_ATCC_43049_uid57719	NC_006391	plasmid pNG300	clust_263								
55376216	hypothetical protein pNG3041	No	0	plasmid	29080	29259	+		Haloarcula_marismortui_ATCC_43049_uid57719	NC_006391	plasmid pNG300									
55376217	hypothetical protein pNG3042	No	0	plasmid	29469	29882	+		Haloarcula_marismortui_ATCC_43049_uid57719	NC_006391	plasmid pNG300									
55376218	hypothetical protein pNG3043	Yes	2	plasmid	29879	30457	+		Haloarcula_marismortui_ATCC_43049_uid57719	NC_006391	plasmid pNG300									

	55376219	hypothetical protein pNG3044	No	2		plasmid	30464	30949	+		Haloarcula_marismortui_ATCC_43049_uid57719	NC_006391	plasmid pNG300									
	55376220	hypothetical protein pNG3046	No	2		plasmid	31303	31737	+		Haloarcula_marismortui_ATCC_43049_uid57719	NC_006391	plasmid pNG300									
	55376221	hypothetical protein pNG3048	No	0		plasmid	31898	32488	-		Haloarcula_marismortui_ATCC_43049_uid57719	NC_006391	plasmid pNG300									
	55376222	hypothetical protein pNG3049	No	0		plasmid	32492	32956	-		Haloarcula_marismortui_ATCC_43049_uid57719	NC_006391	plasmid pNG300									
	55376223	hypothetical protein pNG3050	No	4		plasmid	33140	34864	+		Haloarcula_marismortui_ATCC_43049_uid57719	NC_006391	plasmid pNG300									
	55376224	hypothetical protein pNG3051	No	0		plasmid	34851	35657	+		Haloarcula_marismortui_ATCC_43049_uid57719	NC_006391	plasmid pNG300									
	55376225	hypothetical protein pNG3052	No	0		plasmid	35657	35986	+		Haloarcula_marismortui_ATCC_43049_uid57719	NC_006391	plasmid pNG300		arCOG00551	1 (-)	L		DNA replication initiation complex subunit, GINS15 family			
===										9	Haloarcula_marismortui_ATCC_43049_uid57719	NC_006392	plasmid pNG400									
	55376230	hypothetical protein pNG4002	No	0	YES	plasmid	1484	1945	-		Haloarcula_marismortui_ATCC_43049_uid57719	NC_006392	plasmid pNG400									
	55376231	putative transposase	No	0	YES	plasmid	1994	2845	-		Haloarcula_marismortui_ATCC_43049_uid57719	NC_006392	plasmid pNG400		arCOG02751	5 (-)	L		Transposase, IS5 family			
	55376232	hypothetical protein pNG4004	No	0		plasmid	3054	3602	+		Haloarcula_marismortui_ATCC_43049_uid57719	NC_006392	plasmid pNG400									
	55376233	hypothetical protein pNG4006	No	0	YES	plasmid	3646	4101	-		Haloarcula_marismortui_ATCC_43049_uid57719	NC_006392	plasmid pNG400									
	55376234	hypothetical protein pNG4005	No	0	YES	plasmid	4282	4557	+		Haloarcula_marismortui_ATCC_43049_uid57719	NC_006392	plasmid pNG400									
	55376235	hypothetical protein pNG4007	No	1	YES	plasmid	4652	5065	+		Haloarcula_marismortui_ATCC_43049_uid57719	NC_006392	plasmid pNG400		arCOG08996	1	S		Uncharacterized conserved protein			
	55376236	hypothetical protein pNG4008	No	0	YES	plasmid	5078	6334	-		Haloarcula_marismortui_ATCC_43049_uid57719	NC_006392	plasmid pNG400									
	55376237	hypothetical protein pNG4009	No	0	YES	plasmid	6300	6794	-		Haloarcula_marismortui_ATCC_43049_uid57719	NC_006392	plasmid pNG400									
	55376238	hypothetical protein pNG4010	No	0	YES	plasmid	6802	7137	-		Haloarcula_marismortui_ATCC_43049_uid57719	NC_006392	plasmid pNG400		arCOG00724	7 (-)	K		Predicted transcriptional regulator, PadR family			
===										5	Haloarcula_marismortui_ATCC_43049_uid57719	NC_006392	plasmid pNG400									
	55376242	hypothetical protein pNG4014	No	0	YES	plasmid	9743	10453	-		Haloarcula_marismortui_ATCC_43049_uid57719	NC_006392	plasmid pNG400									
	55376243	hypothetical protein pNG4015	No	0	YES	plasmid	10453	10743	-		Haloarcula_marismortui_ATCC_43049_uid57719	NC_006392	plasmid pNG400									
	55376244	hypothetical protein pNG4016	No	0	YES	plasmid	10990	11811	+		Haloarcula_marismortui_ATCC_43049_uid57719	NC_006392	plasmid pNG400		arCOG02454	1	S		Uncharacterized conserved protein			
	55376245	phycocyanin alpha phycocyanobilin lyase-like	No	0		plasmid	11821	12945	-		Haloarcula_marismortui_ATCC_43049_uid57719	NC_006392	plasmid pNG400		arCOG02966	7 (-)	S		HEAT repeats containing protein	1E-28	COG1413	FOG: HEAT repeat
	55376246	hypothetical protein pNG4018	No	0		plasmid	13257	13529	+		Haloarcula_marismortui_ATCC_43049_uid57719	NC_006392	plasmid pNG400		arCOG11155	1	S		Uncharacterized conserved protein			
===										7	Haloarcula_marismortui_ATCC_43049_uid57719	NC_006392	plasmid pNG400									
	55376252	hypothetical protein pNG4024	No	0		plasmid	16325	16729	-		Haloarcula_marismortui_ATCC_43049_uid57719	NC_006392	plasmid pNG400		arCOG02756	1 (-)	K		Predicted transcriptional regulator	1E-33	COG4190	Predicted transcriptional regulator
	55376253	transcription regulator	No	0		plasmid	16907	17275	-		Haloarcula_marismortui_ATCC_43049_uid57719	NC_006392	plasmid pNG400									
	55376254	hypothetical protein pNG4026	Yes	0		plasmid	17268	17384	-		Haloarcula_marismortui_ATCC_43049_uid57719	NC_006392	plasmid pNG400									
	55376255	hypothetical protein pNG4027	No	0		plasmid	17961	19499	+		Haloarcula_marismortui_ATCC_43049_uid57719	NC_006392	plasmid pNG400	clust_26								
	55376256	hypothetical protein pNG4028	No	0		plasmid	19557	20027	-		Haloarcula_marismortui_ATCC_43049_uid57719	NC_006392	plasmid pNG400									
	55376257	hypothetical protein pNG4029	No	0		plasmid	20053	20310	+		Haloarcula_marismortui_ATCC_43049_uid57719	NC_006392	plasmid pNG400		arCOG07539	2 (-)	S		Uncharacterized conserved protein			
	55376258	hypothetical protein pNG4030	No	0		plasmid	20371	20877	-		Haloarcula_marismortui_ATCC_43049_uid57719	NC_006392	plasmid pNG400									
===										7	Haloarcula_marismortui_ATCC_43049_uid57719	NC_006392	plasmid pNG400									
	55376263	pNRC100 replication protein H-like	No	0		plasmid	25511	28915	-		Haloarcula_marismortui_ATCC_43049_uid57719	NC_006392	plasmid pNG400		arCOG04495	4 (-)	L	RepH	Plasmid replication protein, contains HTH domain			
	55376264	hypothetical protein pNG4037	No	0		plasmid	29352	29840	-		Haloarcula_marismortui_ATCC_43049_uid57719	NC_006392	plasmid pNG400									
	55376265	hypothetical protein pNG4038	No	0		plasmid	29837	30049	-		Haloarcula_marismortui_ATCC_43049_uid57719	NC_006392	plasmid pNG400		arCOG07540	2	S		Uncharacterized conserved protein			
	55376266	hypothetical protein pNG4039	No	0		plasmid	30049	30348	-		Haloarcula_marismortui_ATCC_43049_uid57719	NC_006392	plasmid pNG400	clust_350								
	55376267	hypothetical protein pNG4041	No	0		plasmid	30345	32537	-		Haloarcula_marismortui_ATCC_43049_uid57719	NC_006392	plasmid pNG400		arCOG10908	2	S		Uncharacterized conserved protein			
	55376268	DNA binding protein	No	0		plasmid	32537	32851	-		Haloarcula_marismortui_ATCC_43049_uid57719	NC_006392	plasmid pNG400		arCOG00005	4 (-)	K		Predicted transcriptional regulator, PadR family			
	55376269	transcription regulator	No	0		plasmid	32848	33528	-		Haloarcula_marismortui_ATCC_43049_uid57719	NC_006392	plasmid pNG400									
===										5	Haloarcula_marismortui_ATCC_43049_uid57719	NC_006393	plasmid pNG500									
	55376319	hypothetical protein pNG5046	No	0	YES	plasmid	38508	38696	-		Haloarcula_marismortui_ATCC_43049_uid57719	NC_006393	plasmid pNG500									
	55376320	terminal quinol oxidase subunit	No	4	YES	plasmid	38737	39153	-		Haloarcula_marismortui_ATCC_43049_uid57719	NC_006393	plasmid pNG500		arCOG03070	1 (-)	S		Predicted membrane protein, DoxD family	6E-11	COG2259	Predicted membrane protein
	55376321	hypothetical protein pNG5048	No	0	YES	plasmid	39342	39548	+		Haloarcula_marismortui_ATCC_43049_uid57719	NC_006393	plasmid pNG500									
	55376322	hypothetical protein pNG5049	Yes	0	YES	plasmid	39545	39826	+		Haloarcula_marismortui_ATCC_43049_uid57719	NC_006393	plasmid pNG500									
	55376323	hypothetical protein pNG5050	No	1	YES	plasmid	40146	40283	+		Haloarcula_marismortui_ATCC_43049_uid57719	NC_006393	plasmid pNG500	clust_502								
===										22	Haloarcula_marismortui_ATCC_43049_uid57719	NC_006393	plasmid pNG500									
	55376358	ribbon-helix-helix protein	No	0		plasmid	80708	81085	+		Haloarcula_marismortui_ATCC_43049_uid57719	NC_006393	plasmid pNG500	clust_283								
	55376359	ribbon-helix-helix protein	No	0		plasmid	81082	81555	+		Haloarcula_marismortui_ATCC_43049_uid57719	NC_006393	plasmid pNG500		arCOG10343	6	S		Uncharacterized conserved protein			
	55376360	relaxase/mobilization nuclease domain-contair	No	0		plasmid	81552	82307	+		Haloarcula_marismortui_ATCC_43049_uid57719	NC_006393	plasmid pNG500		arCOG10214	7	S		Uncharacterized conserved protein	0.00005	pfam13868	Tumour suppressor, Mitostatin. Trichople
	55376361	TraD protein	No	3		plasmid	82403	84520	+		Haloarcula_marismortui_ATCC_43049_uid57719	NC_006393	plasmid pNG500		arCOG07873	1 (-)	R	PfoR	Uncharacterized conserved membrane protein	1E-18	pfam10412	Type IV secretion-system coupling protei ; Dentin matrix protein 1 (DMP1). This fam
	55376362	hypothetical protein pNG5092	No	0		plasmid	85636	86001	+		Haloarcula_marismortui_ATCC_43049_uid57719	NC_006393	plasmid pNG500	clust_278								
	55376363	hypothetical protein pNG5093	Yes	0		plasmid	86032	86289	+		Haloarcula_marismortui_ATCC_43049_uid57719	NC_006393	plasmid pNG500									
	55376364	hypothetical protein pNG5094	No	0		plasmid	86273	86536	-		Haloarcula_marismortui_ATCC_43049_uid57719	NC_006393	plasmid pNG500									
	55376365	hypothetical protein pNG5095	No	0		plasmid	87030	88181	-		Haloarcula_marismortui_ATCC_43049_uid57719	NC_006393	plasmid pNG500		arCOG01510	2 (-)	L	RFA1	Single-stranded DNA-binding replication protein A (RPA), large (70 kD) subunit or related ssDNA-binding protein			
	55376366	hypothetical protein pNG5096	No	0	YES	plasmid	88505	89320	+		Haloarcula_marismortui_ATCC_43049_uid57719	NC_006393	plasmid pNG500									
	55376367	hypothetical protein pNG5099	No	0	YES	plasmid	89645	90133	-		Haloarcula_marismortui_ATCC_43049_uid57719	NC_006393	plasmid pNG500		arCOG07544	3 (-)	S		Zn-finger protein			
	55376368	hypothetical protein pNG5101	No	0	YES	plasmid	90401	90871	+		Haloarcula_marismortui_ATCC_43049_uid57719	NC_006393	plasmid pNG500									
	55376369	hypothetical protein pNG5102	No	0	YES	plasmid	90868	91044	+		Haloarcula_marismortui_ATCC_43049_uid57719	NC_006393	plasmid pNG500									
	55376370	hypothetical protein pNG5103	No	0	YES	plasmid	91017	91277	-		Haloarcula_marismortui_ATCC_43049_uid57719	NC_006393	plasmid pNG500									
	55376371	hypothetical protein pNG5104	No	0	YES	plasmid	91656	92771	-		Haloarcula_marismortui_ATCC_43049_uid57719	NC_006393	plasmid pNG500		arCOG06163	1	S		Uncharacterized conserved protein			
	55376372	hypothetical protein pNG5105	No	0	YES	plasmid	92951	93196	-		Haloarcula_marismortui_ATCC_43049_uid57719	NC_006393	plasmid pNG500	clust_57								
	55376373	hypothetical protein pNG5106	No	0	YES	plasmid	93282	93455	+		Haloarcula_marismortui_ATCC_43049_uid57719	NC_006393	plasmid pNG500									
	55376374	hypothetical protein pNG5107	No	0	YES	plasmid	93452	93562	-		Haloarcula_marismortui_ATCC_43049_uid57719	NC_006393	plasmid pNG500									
	55376375	hypothetical protein pNG5108	No	0	YES	plasmid	93643	95487	-		Haloarcula_marismortui_ATCC_43049_uid57719	NC_006393	plasmid pNG500		arCOG07768	1	S		Uncharacterized conserved protein			
	55376376	hypothetical protein pNG5109	No	0	YES	plasmid	95902	96882	+		Haloarcula_marismortui_ATCC_43049_uid57719	NC_006393	plasmid pNG500									
	55376377	hypothetical protein pNG5110	No	0	YES	plasmid	96985	98115	+		Haloarcula_marismortui_ATCC_43049_uid57719	NC_006393	plasmid pNG500									
	55376378	hypothetical protein pNG5111	No	0	YES	plasmid	98832	99851	-		Haloarcula_marismortui_ATCC_43049_uid57719	NC_006393	plasmid pNG500		arCOG07544	3 (-)	S		Zn-finger protein			
	55376379	hypothetical protein pNG5112	No	0	YES	plasmid	99915	100496	-		Haloarcula_marismortui_ATCC_43049_uid57719	NC_006393	plasmid pNG500									
===										6	Haloarcula_marismortui_ATCC_43049_uid57719	NC_006394	plasmid pNG600									
	55376499	exonuclease VII small subunit	No	0		plasmid	75435	75617	+		Haloarcula_marismortui_ATCC_43049_uid57719	NC_006394	plasmid pNG600		arCOG06170	1 (-)	L		Exonuclease VII small subunit	0.000001	pfam02609	Exonuclease VII small subunit. This fami
	55376500	hypothetical protein pNG6096	No	3		plasmid	75765	76139	+		Haloarcula_marismortui_ATCC_43049_uid57719	NC_006394	plasmid pNG600		arCOG06171	3	S		Uncharacterized conserved protein			
	55376501	hypothetical protein pNG6097	No	0		plasmid	76248	76547	+		Haloarcula_marismortui_ATCC_43049_uid57719	NC_006394	plasmid pNG600									
	55376502	hypothetical protein pNG6098	No	0		plasmid	76568	76771	-		Haloarcula_marismortui_ATCC_43049_uid57719	NC_006394	plasmid pNG600									
	55376503	hypothetical protein pNG6099	No	3		plasmid	76911	77333	+		Haloarcula_marismortui_ATCC_43049_uid57719	NC_006394	plasmid pNG600		arCOG09002	1	S		Uncharacterized conserved protein			
	55376504	hypothetical protein pNG6101	No	1		plasmid	77359	79014	+		Haloarcula_marismortui_ATCC_43049_uid57719	NC_006394	plasmid pNG600		arCOG06162	1 (-)	S		Cell suface protein			
===										26	Haloarcula_marismortui_ATCC_43049_uid57719	NC_006396	chromosome I									
	55377457	phage integrase	No	0	YES	Virus_Sphaerc	519012	519857	-		Haloarcula_marismortui_ATCC_43049_uid57719	NC_006396	chromosome I		arCOG02282	8 (-)	K		Predicted transcriptional regulator, C-terminal HTH-like domain			
	55377456	hypothetical protein rrnAC0590	No	0	YES	Virus_Sphaerc	519848	520351	+		Haloarcula_marismortui_ATCC_43049_uid57719	NC_006396	chromosome I									
	55377458	hypothetical protein rrnAC0591	No	0	YES	Virus_Sphaerc	520435	521274	-		Haloarcula_marismortui_ATCC_43049_uid57719	NC_006396	chromosome I		arCOG09035	3	S		Uncharacterized conserved protein			
	55377459	hypothetical protein rrnAC0592	No	0	YES	Virus_Sphaerc	521276	521785	-		Haloarcula_marismortui_ATCC_43049_uid57719	NC_006396	chromosome I									
	55377460	hypothetical protein rrnAC0593	No	0	YES	Virus_Sphaerc	521807	521932	-		Haloarcula_marismortui_ATCC_43049_uid57719	NC_006396	chromosome I									
	55377461	hypothetical protein rrnAC0594	No	0	YES	Virus_Sphaerc	522350	522565	-		Haloarcula_marismortui_ATCC_43049_uid57719	NC_006396	chromosome I		arCOG09036	3	S		Uncharacterized conserved protein			
	55377462	hypothetical protein rrnAC0595	No	1	YES	Virus_Sphaerc	522562	523014	-		Haloarcula_marismortui_ATCC_43049_uid57719	NC_006396	chromosome I									
	55377463	hypothetical protein rrnAC0596	No	0	YES	Virus_Sphaerc	523002	523544	-		Haloarcula_marismortui_ATCC_43049_uid57719	NC_006396	chromosome I									
	55377464	hypothetical protein rrnAC0597	No	0	YES	Virus_Sphaerc	523541	524383	-		Haloarcula_marismortui_ATCC_43049_uid57719	NC_006396	chromosome I									
	55377465	hypothetical protein rrnAC0598	No	0	YES	Virus_Sphaerc	524385	524888	-		Haloarcula_marismortui_ATCC_43049_uid57719	NC_006396	chromosome I									
	55377466	hypothetical protein rrnAC0599	No	0	YES	Virus_Sphaerc	525127	525222	-		Haloarcula_marismortui_ATCC_43049_uid57719	NC_006396	chromosome I									
	55377467	hypothetical protein rrnAC0600	No	1	YES	Virus_Sphaerc	525212	525409	-		Haloarcula_marismortui_ATCC_43049_uid57719	NC_006396	chromosome I									
	55377468	hypothetical protein rrnAC0601	No	0	YES	Virus_Sphaerc	525409	525921	-		Haloarcula_marismortui_ATCC_43049_uid57719	NC_006396	chromosome I									
	55377469	hypothetical protein rrnAC0602	No	1	YES	Virus_Sphaerc	525921	526184	-		Haloarcula_marismortui_ATCC_43049_uid57719	NC_006396	chromosome I									
	55377470	hypothetical protein rrnAC0603	No	0	YES	Virus_Sphaerc	526199	526924	-		Haloarcula_marismortui_ATCC_43049_uid57719	NC_006396	chromosome I		arCOG09037	5	Y		VP4/VP17-like Major capsid protein			
	55377471	hypothetical protein rrnAC0604	No	0	YES	Virus_Sphaerc	526930	527460	-		Haloarcula_marismortui_ATCC_43049_uid57719	NC_006396	chromosome I		arCOG09038	6	Y		VP7/VP16-like Major capsid protein			
	55377472	hypothetical protein rrnAC0605	No	0	YES	Virus_Sphaerc	527809	527949	-		Haloarcula_marismortui_ATCC_43049_uid57719	NC_006396	chromosome I		arCOG09039	3	S		Uncharacterized conserved protein			
	55377473	hypothetical protein rrnAC0606	No	0	YES	Virus_Sphaerc	527946	528686	-		Haloarcula_marismortui_ATCC_43049_uid57719	NC_006396	chromosome I		arCOG00280	21 (-)	L		HerA helicase			
	55377474	hypothetical protein rrnAC0607	Yes	0	YES	Virus_Sphaerc	528967	529212	-		Haloarcula_marismortui_ATCC_43049_uid57719	NC_006396	chromosome I		arCOG10925	3	S		Uncharacterized conserved protein			
	55377475	hypothetical protein rrnAC0608	No	0	YES	Virus_Sphaerc	529209	529781	-		Haloarcula_marismortui_ATCC_43049_uid57719	NC_006396	chromosome I		arCOG06228	5	S		Uncharacterized conserved protein			
	55377476	hypothetical protein rrnAC0609	No	0	YES	Virus_Sphaerc	529786	530346	-		Haloarcula_marismortui_ATCC_43049_uid57719	NC_006396	chromosome I		arCOG09040	3	S		Uncharacterized conserved protein	0.00001	pfam04006	Mpp10 protein. This family includes prot
	55377477	hypothetical protein rrnAC0610	No	1	YES	Virus_Sphaerc	530340	530954	-		Haloarcula_marismortui_ATCC_43049_uid57719	NC_006396	chromosome I		arCOG09041	4	S		Uncharacterized conserved protein	0.000004	pfam05297	Herpesvirus latent membrane protein 1 (L
	55377478	hypothetical protein rrnAC0612	No	0	YES	Virus_Sphaerc	530951	531928	-		Haloarcula_marismortui_ATCC_43049_uid57719	NC_006396	chromosome I		arCOG09042	7	Y		Viral protein, VP1 family			
	55377479	hypothetical protein rrnAC0613	No	0	YES	Virus_Sphaerc	532386	532553	-		Haloarcula_marismortui_ATCC_43049_uid57719	NC_006396	chromosome I									
	55377480	hypothetical protein rrnAC0614	No	0	YES	Virus_Sphaerc	532546	533001	-		Haloarcula_marismortui_ATCC_43049_uid57719	NC_006396	chromosome I		arCOG09043	4	S		Uncharacterized conserved protein			
	55377481	hypothetical protein rrnAC0615	No	0	YES	Virus_Sphaerc	533141	533695	-		Haloarcula_marismortui_ATCC_43049_uid57719	NC_006396	chromosome I		arCOG09044	5 (-)	R		SWIM zinc finger			
===										6	Haloarcula_marismortui_ATCC_43049_uid57719	NC_006396	chromosome I									
	55377826	inositol-1-monophosphatase	No	0			882280	883119	-		Haloarcula_marismortui_ATCC_43049_uid57719	NC_006396	chromosome I		arCOG01349	1 (-)	G	SuhB	Archaeal fructose-1,6-bisphosphatase or related (	3E-37	COG0483	Archaeal fructose-1,6-bisphosphatase and
	55377827	hypothetical protein rrnAC1006	No	0			883191	883406	-		Haloarcula_marismortui_ATCC_43049_uid57719	NC_006396	chromosome I		arCOG09056	1	S		Uncharacterized conserved protein			
	55377828	hypothetical protein rrnAC1007	No	0			884016	884117	+		Haloarcula_marismortui_ATCC_43049_uid57719	NC_006396	chromosome I									
	55377829	hypothetical protein rrnAC1008	No	0			884204	884308	+		Haloarcula_marismortui_ATCC_43049_uid57719	NC_006396	chromosome I									
	55377830	hypothetical protein rrnAC1009	No	0			884544	884756	-		Haloarcula_marismortui_ATCC_43049_uid57719	NC_006396	chromosome I									
	55377831	dTDP-glucose 4-6-dehydratase	No	0			885451	886512	+		Haloarcula_marismortui_ATCC_43049_uid57719	NC_006396	chromosome I		arCOG01369	5 (-)	M	WcaG	Nucleoside-diphosphate-sugar epimerase	8E-39	COG1088	dTDP-D-glucose 4,6-dehydratase
===										7	Haloarcula_marismortui_ATCC_43049_uid57719	NC_006396	chromosome I									
	55378112	hypothetical protein rrnAC1324	No	0			1178991	1179125	+		Haloarcula_marismortui_ATCC_43049_uid57719	NC_006396	chromosome I		arCOG10862	2	S		Uncharacterized conserved protein			
	55378113	hypothetical protein rrnAC1325	Yes	0			1179186	1180403	-		Haloarcula_marismortui_ATCC_43049_uid57719	NC_006396	chromosome I		arCOG04512	2 (-)	P		Predicted iron-dependent peroxidase			
	55378114	hypothetical protein rrnAC1326	No	1			1180500	1180649	+		Haloarcula_marismortui_ATCC_43049_uid57719	NC_006396	chromosome I	clust_499								
	55378115	hypothetical protein rrnAC1327	No	2			1180649	1180924	+		Haloarcula_marismortui_ATCC_43049_uid57719	NC_006396	chromosome I	clust_370								
	55378117	hypothetical protein rrnAC1329	No	0			1180745	1181116	-		Haloarcula_marismortui_ATCC_43049_uid57719	NC_006396	chromosome I									
	55378116	hypothetical protein rrnAC1328	Yes	2			1180917	1181150	+		Haloarcula_marismortui_ATCC_43049_uid57719	NC_006396	chromosome I	clust_413								
	55378118	cytoplasmic chaperone TorD	No	0			1181159	1181785	-		Haloarcula_marismortui_ATCC_43049_uid57719	NC_006396	chromosome I		arCOG01506	1 (-)	R	TorD	Uncharacterized component of anaerobic dehydr	0.00000002	pfam02613	Nitrate reductase delta subunit. This fa
===										8	Haloarcula_marismortui_ATCC_43049_uid57719	NC_006396	chromosome I									
	55378241	hypothetical protein rrnAC1460	No	0			1302131	1302400	+		Haloarcula_marismortui_ATCC_43049_uid57719	NC_006396	chromosome I		arCOG06275	1 (-)	S		Uncharacterized conserved protein			
	55378242	hypothetical protein rrnAC1461	No	0			1302957	1303829	-		Haloarcula_marismortui_ATCC_43049_uid57719	NC_006396	chromosome I									
	55378243	hypothetical protein rrnAC1462	No	0			1303913	1304351	-		Haloarcula_marismortui_ATCC_43049_uid57719	NC_006396	chromosome I									
	55378244	hypothetical protein rrnAC1463	No	0			1304155	1304289	-		Haloarcula_marismortui_ATCC_43049_uid57719	NC_006396	chromosome I									
	55378245	hypothetical protein rrnAC1464	No	0			1304717	1305553	+		Haloarcula_marismortui_ATCC_43049_uid57719	NC_006396	chromosome I									
	55378246	hypothetical protein rrnAC1465	No	0			1305661	1305774	+		Haloarcula_marismortui_ATCC_43049_uid57719	NC_006396	chromosome I									
	55378247	hypothetical protein rrnAC1466	No	0			1305771	1306244	-		Haloarcula_marismortui_ATCC_43049_uid57719	NC_006396	chromosome I									
	55378248	hypothetical protein rrnAC1467	No	0			1306241	1307290	-		Haloarcula_marismortui_ATCC_43049_uid57719	NC_006396	chromosome I		arCOG06276	3 (-)	L		Primase-polymerase (primpol) domain containing protein			
===										8	Haloarcula_marismortui_ATCC_43049_uid57719	NC_006396	chromosome I									
	55378322	hypothetical protein rrnAC1547	No	0	YES		1379108	1379668	+		Haloarcula_marismortui_ATCC_43049_uid57719	NC_006396	chromosome I		arCOG06279	1 (-)	S		Uncharacterized conserved protein			
	55378323	hypothetical protein rrnAC1548	No	0	YES		1379896	1380885	+		Haloarcula_marismortui_ATCC_43049_uid57719	NC_006396	chromosome I		arCOG06280	1	S		Uncharacterized conserved protein			
	55378324	hypothetical protein rrnAC1549	No	0	YES		1380882	1381010	+		Haloarcula_marismortui_ATCC_43049_uid57719	NC_006396	chromosome I									
	55378325	hypothetical protein rrnAC1550	No	0	YES		1381097	1381585	-		Haloarcula_marismortui_ATCC_43049_uid57719	NC_006396	chromosome I									
	55378326	hypothetical protein rrnAC1551	No	0	YES		1381659	1382201	-		Haloarcula_marismortui_ATCC_43049_uid57719	NC_006396	chromosome I									
	55378327	hypothetical protein rrnAC1552	No	0	YES		1382222	1382743	-		Haloarcula_marismortui_ATCC_43049_uid57719	NC_006396	chromosome I		arCOG05147	2	S		Uncharacterized conserved protein			
	55378328	hypothetical protein rrnAC1553	No	0	YES		1382843	1383364	-		Haloarcula_marismortui_ATCC_43049_uid57719	NC_006396	chromosome I									
	55378329	hypothetical protein rrnAC1555	No	0	YES		1383507	1384043	-		Haloarcula_marismortui_ATCC_43049_uid57719	NC_006396	chromosome I		arCOG03429	2 (-)	R		SWIM zinc finger containing protein	8E-21	COG4715	Uncharacterized conserved protein
===										5	Haloarcula_marismortui_ATCC_43049_uid57719	NC_006396	chromosome I									
	55378967	hypothetical protein rrnAC2286	No	5			2033166	2034107	-		Haloarcula_marismortui_ATCC_43049_uid57719	NC_006396	chromosome I									
	55378968	hypothetical protein rrnAC2287	No	3			2034463	2034996	-		Haloarcula_marismortui_ATCC_43049_uid57719	NC_006396	chromosome I									
	55378969	hypothetical protein rrnAC2288	No	7			2035259	2036074	+		Haloarcula_marismortui_ATCC_43049_uid57719	NC_006396	chromosome I		arCOG08987	2	S		Uncharacterized conserved protein			
	55378970	hypothetical protein rrnAC2289	No	4			2036112	2036570	+		Haloarcula_marismortui_ATCC_43049_uid57719	NC_006396	chromosome I		arCOG09098	1 (-)	S		Uncharacterized conserved protein			
	55378971	hypothetical protein rrnAC2290	No	1			2036576	2036806	+		Haloarcula_marismortui_ATCC_43049_uid57719	NC_006396	chromosome I		arCOG10957	1	S		Uncharacterized conserved protein			
===										7	Haloarcula_marismortui_ATCC_43049_uid57719	NC_006396	chromosome I									
	55379561	hypothetical protein rrnAC2973	No	0			2637498	2637755	+		Haloarcula_marismortui_ATCC_43049_uid57719	NC_006396	chromosome I									
	55379562	hypothetical protein rrnAC2974	Yes	2			2637950	2639209	+		Haloarcula_marismortui_ATCC_43049_uid57719	NC_006396	chromosome I		arCOG04474	1 (-)	S		Uncharacterized conserved protein			
	55379563	hypothetical protein rrnAC2976	Yes	4			2639220	2640524	-		Haloarcula_marismortui_ATCC_43049_uid57719	NC_006396	chromosome I	clust_80						0.00000004	pfam07172	Glycine rich protein family. This family
	55379564	hypothetical protein rrnAC2977	Yes	1			2640597	2641205	-		Haloarcula_marismortui_ATCC_43049_uid57719	NC_006396	chromosome I		arCOG11334	5	S		Uncharacterized conserved protein			
	55379565	hypothetical protein rrnAC2978	No	1			2641378	2642046	+		Haloarcula_marismortui_ATCC_43049_uid57719	NC_006396	chromosome I		arCOG11334	5	S		Uncharacterized conserved protein			
	55379566	hypothetical protein rrnAC2979	No	2			2642714	2643052	+		Haloarcula_marismortui_ATCC_43049_uid57719	NC_006396	chromosome I		arCOG03828	5 (-)	S		Uncharacterized conserved protein			
	55379567	hypothetical protein rrnAC2980	No	0			2643049	2644584	+		Haloarcula_marismortui_ATCC_43049_uid57719	NC_006396	chromosome I		arCOG11334	5	S		Uncharacterized conserved protein			
===										12	Haloarcula_marismortui_ATCC_43049_uid57719	NC_006397	chromosome II									
	55380087	hypothetical protein rrnB0016	No	0	YES		17711	18109	-		Haloarcula_marismortui_ATCC_43049_uid57719	NC_006397	chromosome II									
	55380088	hypothetical protein rrnB0017	No	0	YES		18285	18470	-		Haloarcula_marismortui_ATCC_43049_uid57719	NC_006397	chromosome II									
	55380089	flagellin A precursor	Yes	0			18736	18990	+		Haloarcula_marismortui_ATCC_43049_uid57719	NC_006397	chromosome II		arCOG01829	3 (-)	N	FlaB	Archaeal flagellins	3E-15	COG1681	Archaeal flagellins
	55380090	hypothetical protein rrnB0019	Yes	2			19435	19881	+		Haloarcula_marismortui_ATCC_43049_uid57719	NC_006397	chromosome II		arCOG09133	2	S		Uncharacterized conserved protein			
	55380091	hypothetical protein rrnB0020	Yes	0			20762	20911	-		Haloarcula_marismortui_ATCC_43049_uid57719	NC_006397	chromosome II									
	55380092	hypothetical protein rrnB0021	No	2			21036	21506	+		Haloarcula_marismortui_ATCC_43049_uid57719	NC_006397	chromosome II		arCOG09133	2	S		Uncharacterized conserved protein			
	55380093	hypothetical protein rrnB0022	Yes	0			21638	22249	+		Haloarcula_marismortui_ATCC_43049_uid57719	NC_006397	chromosome II		arCOG07571	1 (-)	S		Uncharacterized conserved membrane protein			
	55380094	hypothetical protein rrnB0023	No	2			22552	22854	-		Haloarcula_marismortui_ATCC_43049_uid57719	NC_006397	chromosome II		arCOG10837	1	S		Uncharacterized conserved protein			
	55380095	hypothetical protein rrnB0024	No	2			23087	23263	-		Haloarcula_marismortui_ATCC_43049_uid57719	NC_006397	chromosome II		arCOG11231	2	S		Uncharacterized conserved protein			
	55380096	hypothetical protein rrnB0025	No	0			24679	24867	+		Haloarcula_marismortui_ATCC_43049_uid57719	NC_006397	chromosome II									
	55380097	hypothetical protein rrnB0026	No	1			24975	25250	-		Haloarcula_marismortui_ATCC_43049_uid57719	NC_006397	chromosome II									
	55380098	acetyltransferase	No	0			25657	26232	+		Haloarcula_marismortui_ATCC_43049_uid57719	NC_006397	chromosome II		arCOG00834	1 (-)	K	WecD	Acetyltransferase (GNAT) family			
===										8	Haloarcula_marismortui_ATCC_43049_uid57719	NC_006397	chromosome II									
	55380206	hypothetical protein rrnB0161	No	0			122650	122937	+		Haloarcula_marismortui_ATCC_43049_uid57719	NC_006397	chromosome II		arCOG00005	4 (-)	K		Predicted transcriptional regulator, PadR family			
	55380207	hypothetical protein rrnB0162	No	0			123204	123509	-		Haloarcula_marismortui_ATCC_43049_uid57719	NC_006397	chromosome II		arCOG09139	5	S		Uncharacterized conserved protein			
	55380208	hypothetical protein rrnB0163	No	0			123755	124405	-		Haloarcula_marismortui_ATCC_43049_uid57719	NC_006397	chromosome II		arCOG09139	5	S		Uncharacterized conserved protein			
	55380209	hypothetical protein rrnB0164	No	0			124402	125616	-		Haloarcula_marismortui_ATCC_43049_uid57719	NC_006397	chromosome II		arCOG10906	5	S		Uncharacterized conserved protein	0.00007	pfam07093	SGT1 protein. This family consists of se
	55380210	hypothetical protein rrnB0166	No	0			126085	127200	-		Haloarcula_marismortui_ATCC_43049_uid57719	NC_006397	chromosome II		arCOG09139	5	S		Uncharacterized conserved protein			
	55380211	calcium-binding protein-like	Yes	1			127203	131891	-		Haloarcula_marismortui_ATCC_43049_uid57719	NC_006397	chromosome II	arCOG02903,a	arCOG02903	1 (-)	R		Protein comtaining Bacterial Ig-like domain and v	7E-10	COG2304	Uncharacterized protein containing a von
	55380212	hypothetical protein rrnB0168	Yes	2			131974	132876	+		Haloarcula_marismortui_ATCC_43049_uid57719	NC_006397	chromosome II							0.00000004	pfam11596	Protein of unknown function (DUF3246). T
	55380213	hypothetical protein rrnB0169	No	0			133070	133219	+		Haloarcula_marismortui_ATCC_43049_uid57719	NC_006397	chromosome II									
===										8	Halobacterium_NRC_1_uid57769	NC_002607	chromosome									
	15789352	hypothetical protein VNG0016H	No	0	YES		13809	14180	-		Halobacterium_NRC_1_uid57769	NC_002607	chromosome		arCOG02750	2 (-)	K		Predicted transcriptional regulator, HTH			
	15789353	hypothetical protein VNG0017H	No	0	YES		14484	14780	+		Halobacterium_NRC_1_uid57769	NC_002607	chromosome		arCOG09184	2	S		Uncharacterized conserved protein			
	15789354	hypothetical protein VNG0018H	No	0	YES		14868	15131	+		Halobacterium_NRC_1_uid57769	NC_002607	chromosome		arCOG09185	3	S		Uncharacterized conserved protein			
	15789355	hypothetical protein VNG0019H	No	0	YES		15310	15408	+		Halobacterium_NRC_1_uid57769	NC_002607	chromosome		arCOG09186	2	S		Uncharacterized conserved protein			
	15789356	hypothetical protein VNG0020H	No	0	YES		15419	16051	-		Halobacterium_NRC_1_uid57769	NC_002607	chromosome		arCOG09187	2	S		Uncharacterized conserved protein			
	15789357	hypothetical protein VNG0021H	No	0	YES		16342	17706	+		Halobacterium_NRC_1_uid57769	NC_002607	chromosome		arCOG04792	9 (-)	L		Transposase	7E-16	pfam03050	Transposase IS66 family. Transposase pro
	15789358	hypothetical protein VNG0022H	No	0	YES		18357	18902	+		Halobacterium_NRC_1_uid57769	NC_002607	chromosome		arCOG09188	2	S		Uncharacterized conserved protein			
	15789359	hypothetical protein VNG0023H	No	0	YES		18895	19530	+		Halobacterium_NRC_1_uid57769	NC_002607	chromosome		arCOG09189	2	S		Uncharacterized conserved protein			
===										7	Halobacterium_NRC_1_uid57769	NC_002607	chromosome									
	15789363	hypothetical protein VNG0027H	No	0	YES		22103	22750	-		Halobacterium_NRC_1_uid57769	NC_002607	chromosome		arCOG09191	2	S		Uncharacterized conserved protein			
	15789364	hypothetical protein VNG0028C	No	0	YES		23336	23890	+		Halobacterium_NRC_1_uid57769	NC_002607	chromosome		arCOG02128	6 (-)	L		Transposase	9E-14	COG3415	Transposase and inactivated derivatives
	15789365	hypothetical protein VNG0029H	No	0	YES		24109	24564	-		Halobacterium_NRC_1_uid57769	NC_002607	chromosome		arCOG09192	2	S		Uncharacterized conserved protein			
	15789366	hypothetical protein VNG0030H	No	0	YES		26201	26596	+		Halobacterium_NRC_1_uid57769	NC_002607	chromosome		arCOG09193	2	S		Uncharacterized conserved protein			
	15789367	hypothetical protein VNG0031H	No	0	YES		26648	29008	+		Halobacterium_NRC_1_uid57769	NC_002607	chromosome		arCOG06281	2	S		Uncharacterized conserved protein			
	15789368	hypothetical protein VNG0032H	No	0	YES		29046	29429	+		Halobacterium_NRC_1_uid57769	NC_002607	chromosome		arCOG09194	2	S		Uncharacterized conserved protein			
	15789369	hypothetical protein VNG0033H	No	0	YES		29476	29913	-		Halobacterium_NRC_1_uid57769	NC_002607	chromosome		arCOG06153	3 (-)	S		Uncharacterized conserved protein			
===										7	Halobacterium_NRC_1_uid57769	NC_002607	chromosome									
	15789435	oxidoreductase	No	0			98226	99008	+		Halobacterium_NRC_1_uid57769	NC_002607	chromosome		arCOG01265	2 (-)	R		Short-chain alcohol dehydrogenase	3E-62	COG4221	Short-chain alcohol dehydrogenase of unk
	15789436	hypothetical protein VNG0116H	No	1			100891	101715	-		Halobacterium_NRC_1_uid57769	NC_002607	chromosome		arCOG09199	2	S		Uncharacterized conserved protein			
	15789437	hypothetical protein VNG0117H	No	0			102034	102309	-		Halobacterium_NRC_1_uid57769	NC_002607	chromosome		arCOG09200	2	S		Uncharacterized conserved protein			
	15789438	hypothetical protein VNG0118H	No	0			102309	102692	-		Halobacterium_NRC_1_uid57769	NC_002607	chromosome		arCOG09201	3	S		Uncharacterized conserved protein			
	15789439	hypothetical protein VNG0119H	No	0			102863	103021	-		Halobacterium_NRC_1_uid57769	NC_002607	chromosome		arCOG09202	2	S		Uncharacterized conserved protein			
	15789440	hypothetical protein VNG0120H	No	0			103021	103146	-		Halobacterium_NRC_1_uid57769	NC_002607	chromosome		arCOG09203	2	S		Uncharacterized conserved protein			
	15789441	hypothetical protein VNG0121H	Yes	0			103514	104005	+		Halobacterium_NRC_1_uid57769	NC_002607	chromosome		arCOG03116	2 (-)	S		Uncharacterized conserved membrane protein	7E-13	pfam02643	Uncharacterized ACR, COG1430. Two struct
===										5	Halobacterium_NRC_1_uid57769	NC_002608	plasmid pNRC200									
	16120103	hypothetical protein VNG6155H	No	0		plasmid	132066	132377	+		Halobacterium_NRC_1_uid57769	NC_002608	plasmid pNRC200		arCOG09340	1	S		Uncharacterized conserved protein			
	16120104	hypothetical protein VNG6156H	No	0		plasmid	132832	133239	+		Halobacterium_NRC_1_uid57769	NC_002608	plasmid pNRC200		arCOG08887	3	S		Uncharacterized conserved protein			
	16120105	hypothetical protein VNG6157H	No	1		plasmid	135571	135987	-		Halobacterium_NRC_1_uid57769	NC_002608	plasmid pNRC200		arCOG09341	3 (-)	S		Uncharacterized conserved protein			
	16120106	hypothetical protein VNG6158H	No	0		plasmid	136124	136285	-		Halobacterium_NRC_1_uid57769	NC_002608	plasmid pNRC200		arCOG09342	1	S		Uncharacterized conserved protein			
	16120107	hypothetical protein VNG6159H	No	1		plasmid	136593	136724	-		Halobacterium_NRC_1_uid57769	NC_002608	plasmid pNRC200		arCOG09343	1	S		Uncharacterized conserved protein			
===										9	Halobacterium_NRC_1_uid57769	NC_002608	plasmid pNRC200									
	16120242	hypothetical protein VNG6339H	No	0	YES	plasmid	269921	270106	+		Halobacterium_NRC_1_uid57769	NC_002608	plasmid pNRC200		arCOG09362	1	S		Uncharacterized conserved protein			
	16120243	hypothetical protein VNG6340H	No	3	YES	plasmid	270141	270422	-		Halobacterium_NRC_1_uid57769	NC_002608	plasmid pNRC200		arCOG09363	1	S		Uncharacterized conserved protein			
	16120244	hypothetical protein VNG6341H	No	0	YES	plasmid	270646	271986	+		Halobacterium_NRC_1_uid57769	NC_002608	plasmid pNRC200		arCOG04499	1 (-)	S		Uncharacterized conserved protein			
	16120245	hypothetical protein VNG6343H	No	4	YES	plasmid	275186	275623	-		Halobacterium_NRC_1_uid57769	NC_002608	plasmid pNRC200		arCOG09364	2	S		Uncharacterized conserved protein			
	16120246	hypothetical protein VNG6344H	No	0	YES	plasmid	275995	276105	-		Halobacterium_NRC_1_uid57769	NC_002608	plasmid pNRC200									
	16120247	hypothetical protein VNG6345H	No	0	YES	plasmid	276748	278022	+		Halobacterium_NRC_1_uid57769	NC_002608	plasmid pNRC200		arCOG04496	1 (-)	L		Transposase	6E-14	COG3385	FOG: Transposase and inactivated derivat
	16120248	hypothetical protein VNG6346H	No	0	YES	plasmid	278023	278499	-		Halobacterium_NRC_1_uid57769	NC_002608	plasmid pNRC200		arCOG09365	2	S		Uncharacterized conserved protein			
	16120249	hypothetical protein VNG6347H	No	0	YES	plasmid	278559	279104	+		Halobacterium_NRC_1_uid57769	NC_002608	plasmid pNRC200		arCOG09366	4	S		Uncharacterized conserved protein			
	16120250	hypothetical protein VNG6348H	No	0	YES	plasmid	279159	279653	-		Halobacterium_NRC_1_uid57769	NC_002608	plasmid pNRC200		arCOG09367	2	S		Uncharacterized conserved protein			
===										6	Halobacterium_NRC_1_uid57769	NC_002608	plasmid pNRC200									
	16120303	hypothetical protein VNG6424H	No	0		plasmid	325010	325516	-		Halobacterium_NRC_1_uid57769	NC_002608	plasmid pNRC200									
	16120304	hypothetical protein VNG6427H	No	0		plasmid	325607	326050	-		Halobacterium_NRC_1_uid57769	NC_002608	plasmid pNRC200									
	16120305	hypothetical protein VNG6429H	No	0		plasmid	326500	326877	-		Halobacterium_NRC_1_uid57769	NC_002608	plasmid pNRC200		arCOG09372	2	S		Uncharacterized conserved protein			
	16120306	hypothetical protein VNG6430C	No	0		plasmid	327051	327602	+		Halobacterium_NRC_1_uid57769	NC_002608	plasmid pNRC200		arCOG02134	15 (-)	L		Transposase	1E-13	COG3316	Transposase and inactivated derivatives
	16120307	hypothetical protein VNG6431H	No	0		plasmid	327699	327929	-		Halobacterium_NRC_1_uid57769	NC_002608	plasmid pNRC200		arCOG09373	2	S		Uncharacterized conserved protein			
	16120308	hypothetical protein VNG6432H	No	0		plasmid	328134	328547	+		Halobacterium_NRC_1_uid57769	NC_002608	plasmid pNRC200		arCOG09374	2	S		Uncharacterized conserved protein			
===										8	Halobacterium_salinarum_R1_uid61571	NC_010364	chromosome									
	169235060	hypothetical protein OE1023R	No	0	YES		13809	14201	-		Halobacterium_salinarum_R1_uid61571	NC_010364	chromosome		arCOG02750	2 (-)	K		Predicted transcriptional regulator, HTH			
	169235061	hypothetical protein OE1025F	No	0	YES		14484	14780	+		Halobacterium_salinarum_R1_uid61571	NC_010364	chromosome		arCOG09184	2	S		Uncharacterized conserved protein			
	169235062	hypothetical protein OE1026F	No	0	YES		14868	15131	+		Halobacterium_salinarum_R1_uid61571	NC_010364	chromosome		arCOG09185	3	S		Uncharacterized conserved protein			
	169235063	hypothetical protein OE1028F	No	0	YES		15310	15408	+		Halobacterium_salinarum_R1_uid61571	NC_010364	chromosome		arCOG09186	2	S		Uncharacterized conserved protein			
	169235064	hypothetical protein OE1029R	No	0	YES		15419	16051	-		Halobacterium_salinarum_R1_uid61571	NC_010364	chromosome		arCOG09187	2	S		Uncharacterized conserved protein			
	169235065	transposase (ISH10)	No	0	YES		16282	17706	+		Halobacterium_salinarum_R1_uid61571	NC_010364	chromosome		arCOG04792	9 (-)	L		Transposase	8E-16	pfam03050	Transposase IS66 family. Transposase pro ; Chromosome segregation ATPases
	169235066	hypothetical protein OE1036F	No	0	YES		18357	18902	+		Halobacterium_salinarum_R1_uid61571	NC_010364	chromosome		arCOG09188	2	S		Uncharacterized conserved protein			
	169235067	hypothetical protein OE1037F	No	0	YES		18895	19530	+		Halobacterium_salinarum_R1_uid61571	NC_010364	chromosome		arCOG09189	2	S		Uncharacterized conserved protein			
===										9	Halobacterium_salinarum_R1_uid61571	NC_010364	chromosome									
	169235071	hypothetical protein OE1041R	No	0	YES		22103	22750	-		Halobacterium_salinarum_R1_uid61571	NC_010364	chromosome		arCOG09191	2	S		Uncharacterized conserved protein			
	169235072	hypothetical protein OE1044F	No	0	YES		23103	23336	+		Halobacterium_salinarum_R1_uid61571	NC_010364	chromosome									
	169235073	transposase (TCE39)	No	0	YES		23336	23890	+		Halobacterium_salinarum_R1_uid61571	NC_010364	chromosome		arCOG02128	6 (-)	L		Transposase	9E-14	COG3415	Transposase and inactivated derivatives
	169235074	hypothetical protein OE1047R	No	0	YES		24109	24564	-		Halobacterium_salinarum_R1_uid61571	NC_010364	chromosome		arCOG09192	2	S		Uncharacterized conserved protein			
	169235075	hypothetical protein OE1048F	No	0	YES		24892	25227	+		Halobacterium_salinarum_R1_uid61571	NC_010364	chromosome									
	169235076	hypothetical protein OE1052F	No	0	YES		26201	26596	+		Halobacterium_salinarum_R1_uid61571	NC_010364	chromosome		arCOG09193	2	S		Uncharacterized conserved protein			
	169235077	hypothetical protein OE1053F	No	0	YES		26648	29008	+		Halobacterium_salinarum_R1_uid61571	NC_010364	chromosome		arCOG06281	2	S		Uncharacterized conserved protein			
	169235078	hypothetical protein OE1054F	No	0	YES		29046	29429	+		Halobacterium_salinarum_R1_uid61571	NC_010364	chromosome		arCOG09194	2	S		Uncharacterized conserved protein			
	169235079	hypothetical protein OE1055R	No	0	YES		29476	29913	-		Halobacterium_salinarum_R1_uid61571	NC_010364	chromosome		arCOG06153	3 (-)	S		Uncharacterized conserved protein			
===										8	Halobacterium_salinarum_R1_uid61571	NC_010364	chromosome									
	169235149	oxidoreductase	No	0			99251	100033	+		Halobacterium_salinarum_R1_uid61571	NC_010364	chromosome		arCOG01265	2 (-)	R		Short-chain alcohol dehydrogenase	3E-62	COG4221	Short-chain alcohol dehydrogenase of unk
	169235150	hypothetical protein OE1192R	No	1			100522	101346	-		Halobacterium_salinarum_R1_uid61571	NC_010364	chromosome		arCOG09199	2	S		Uncharacterized conserved protein			
	169235151	hypothetical protein OE1193F	No	0			101365	101649	+		Halobacterium_salinarum_R1_uid61571	NC_010364	chromosome									
	169235152	hypothetical protein OE1194R	No	0			101665	101940	-		Halobacterium_salinarum_R1_uid61571	NC_010364	chromosome		arCOG09200	2	S		Uncharacterized conserved protein			
	169235153	hypothetical protein OE1196R	No	0			101940	102323	-		Halobacterium_salinarum_R1_uid61571	NC_010364	chromosome		arCOG09201	3	S		Uncharacterized conserved protein			
	169235154	hypothetical protein OE1197R	No	0			102494	102652	-		Halobacterium_salinarum_R1_uid61571	NC_010364	chromosome		arCOG09202	2	S		Uncharacterized conserved protein			
	169235155	hypothetical protein OE1198R	No	0			102652	102777	-		Halobacterium_salinarum_R1_uid61571	NC_010364	chromosome		arCOG09203	2	S		Uncharacterized conserved protein			
	169235156	hypothetical protein OE1201F	No	0			103208	103636	+		Halobacterium_salinarum_R1_uid61571	NC_010364	chromosome		arCOG03116	2 (-)	S		Uncharacterized conserved membrane protein	5E-13	pfam02643	Uncharacterized ACR, COG1430. Two struct
===										6	Halobacterium_salinarum_R1_uid61571	NC_010364	chromosome									
	169235534	prenyltransferase	No	5			454230	455060	-		Halobacterium_salinarum_R1_uid61571	NC_010364	chromosome		arCOG00476	2 (-)	H	UbiA	4-hydroxybenzoate polyprenyltransferase or relat	4E-24	COG0382	4-hydroxybenzoate polyprenyltransferase
	169235535	hypothetical protein OE1924F	No	0			455798	456778	+		Halobacterium_salinarum_R1_uid61571	NC_010364	chromosome		arCOG09231	1	S		Uncharacterized conserved protein			
	169235536	hypothetical protein OE1925F	Yes	0			457131	457526	+		Halobacterium_salinarum_R1_uid61571	NC_010364	chromosome									
	169235537	hypothetical protein OE1926R	No	0			457664	457858	-		Halobacterium_salinarum_R1_uid61571	NC_010364	chromosome		arCOG09232	3	S		Uncharacterized conserved protein			
	169235538	hypothetical protein OE1928A1F	No	2			458245	458496	+		Halobacterium_salinarum_R1_uid61571	NC_010364	chromosome		arCOG09383	2	S		Uncharacterized conserved protein			
	169235539	transducer protein htr16	Yes	2			458665	461043	-		Halobacterium_salinarum_R1_uid61571	NC_010364	chromosome		arCOG02320	4 (-)	N	Tar	Methyl-accepting chemotaxis protein	1E-47	COG0840	Methyl-accepting chemotaxis protein
===										8	Halobacterium_salinarum_R1_uid61571	NC_010364	chromosome									
	169236752	helicase	No	0			1594454	1596508	+		Halobacterium_salinarum_R1_uid61571	NC_010364	chromosome		arCOG00554	1 (-)	R		Superfamily II helicase, archaea-specific		COG1202	Superfamily II helicase, archaea-specifi
	169236753	hypothetical protein OE4042R	No	0			1596996	1597142	-		Halobacterium_salinarum_R1_uid61571	NC_010364	chromosome		arCOG09298	1	S		Uncharacterized conserved protein			
	169236754	hypothetical protein OE4043R	No	1			1597203	1597790	-		Halobacterium_salinarum_R1_uid61571	NC_010364	chromosome		arCOG09299	1	S		Uncharacterized conserved protein			
	169236755	hypothetical protein OE4044R	No	2			1597783	1598160	-		Halobacterium_salinarum_R1_uid61571	NC_010364	chromosome		arCOG09300	1	S		Uncharacterized conserved protein			
	169236756	hypothetical protein OE4045R	No	1			1598350	1598601	-		Halobacterium_salinarum_R1_uid61571	NC_010364	chromosome									
	169236757	phage PhiH1 repressor protein	No	0			1598617	1598895	-		Halobacterium_salinarum_R1_uid61571	NC_010364	chromosome		arCOG03923	1 (-)	K		PhiH1 repressor family protein, contains HTH domain			
	169236758	hypothetical protein OE4049R	No	0			1598935	1599330	-		Halobacterium_salinarum_R1_uid61571	NC_010364	chromosome		arCOG07981	1	S		Uncharacterized protein			
	169236759	hypothetical protein OE4050F	Yes	1			1599434	1599811	+		Halobacterium_salinarum_R1_uid61571	NC_010364	chromosome		arCOG09301	1	S		Uncharacterized conserved protein	5E-18	COG3430	Uncharacterized protein conserved in arc
===										8	Halobacterium_salinarum_R1_uid61571	NC_010368	plasmid PHS3									
	169237524	hypothetical protein OE5290R	No	1	YES	plasmid	182123	182434	-		Halobacterium_salinarum_R1_uid61571	NC_010368	plasmid PHS3									
	169237525	hypothetical protein OE5291F	No	4	YES	plasmid	182592	182981	+		Halobacterium_salinarum_R1_uid61571	NC_010368	plasmid PHS3									
	169237526	hypothetical protein OE5293F	No	8	YES	plasmid	183667	184917	+		Halobacterium_salinarum_R1_uid61571	NC_010368	plasmid PHS3		arCOG00135	3 (-)	G	ProP	Permease of the major facilitator superfamily	4E-10	pfam05977	Transmembrane secretion effector. This i
	169237527	hypothetical protein OE5294R	No	0	YES	plasmid	185067	185285	-		Halobacterium_salinarum_R1_uid61571	NC_010368	plasmid PHS3									
	169237528	hypothetical protein OE5295F	No	0	YES	plasmid	185299	185616	+		Halobacterium_salinarum_R1_uid61571	NC_010368	plasmid PHS3									
	169237529	hypothetical protein OE5296F	No	0	YES	plasmid	185710	186024	+		Halobacterium_salinarum_R1_uid61571	NC_010368	plasmid PHS3									
	169237530	hypothetical protein OE5297F	No	0	YES	plasmid	186188	186670	+		Halobacterium_salinarum_R1_uid61571	NC_010368	plasmid PHS3									
	169237531	trk-type transport system (substrate potassium	No	0		plasmid	186742	188076	+		Halobacterium_salinarum_R1_uid61571	NC_010368	plasmid PHS3		arCOG01959	1 (-)	P	TrkA	K+ transport system, NAD-binding component	8E-28	COG0569	K+ transport systems, NAD-binding compon ; K+ transport systems, NAD-binding compon
===										5	Halobacterium_salinarum_R1_uid61571	NC_010368	plasmid PHS3									
	169237537	hypothetical protein OE5311R	No	0	YES	plasmid	194464	194874	-		Halobacterium_salinarum_R1_uid61571	NC_010368	plasmid PHS3									
	169237538	hypothetical protein OE5314F	Yes	0	YES	plasmid	195244	196917	+		Halobacterium_salinarum_R1_uid61571	NC_010368	plasmid PHS3		arCOG09122	1 (-)	S		Uncharacterized conserved protein			
	169237539	hypothetical protein OE5315R	No	0	YES	plasmid	197014	197358	-		Halobacterium_salinarum_R1_uid61571	NC_010368	plasmid PHS3									
	169237540	hypothetical protein OE5318F	No	0	YES	plasmid	198852	199706	+		Halobacterium_salinarum_R1_uid61571	NC_010368	plasmid PHS3		arCOG10603	1	S		Uncharacterized conserved protein			
	169237541	hypothetical protein OE5322R	Yes	0	YES	plasmid	200483	200803	-		Halobacterium_salinarum_R1_uid61571	NC_010368	plasmid PHS3									
===										7	Halobacterium_salinarum_R1_uid61571	NC_010369	plasmid PHS2									
	169237611	cysteine-rich protein	No	0		plasmid	6747	7769	+		Halobacterium_salinarum_R1_uid61571	NC_010369	plasmid PHS2		arCOG02846	3 (-)	O	DnaJ	DnaJ-class molecular chaperone	0.00000002	pfam03302	Giardia variant-specific surface protein
	169237612	hypothetical protein OE6018R	No	4	YES	plasmid	8355	8792	-		Halobacterium_salinarum_R1_uid61571	NC_010369	plasmid PHS2		arCOG09364	2	S		Uncharacterized conserved protein			
	169237613	hypothetical protein OE6020R	No	0	YES	plasmid	9164	10255	-		Halobacterium_salinarum_R1_uid61571	NC_010369	plasmid PHS2		arCOG09365	2	S		Uncharacterized conserved protein			
	169237614	hypothetical protein OE6021F	No	0	YES	plasmid	10315	10860	+		Halobacterium_salinarum_R1_uid61571	NC_010369	plasmid PHS2		arCOG09366	4	S		Uncharacterized conserved protein			
	169237615	hypothetical protein OE6022R	No	0	YES	plasmid	10915	11409	-		Halobacterium_salinarum_R1_uid61571	NC_010369	plasmid PHS2		arCOG09367	2	S		Uncharacterized conserved protein			
	169237616	hypothetical protein OE6022A1R	No	0	YES	plasmid	11959	12120	-		Halobacterium_salinarum_R1_uid61571	NC_010369	plasmid PHS2									
	169237617	hypothetical protein OE6023R	No	0	YES	plasmid	12432	13706	-		Halobacterium_salinarum_R1_uid61571	NC_010369	plasmid PHS2		arCOG03110	5 (-)	K		Predicted transciptional regulator, contains Fic ar	2E-47	COG3177	Fic family protein
===										6	Halobacterium_salinarum_R1_uid61571	NC_010369	plasmid PHS2									
	169237671	hypothetical protein OE6113F	No	0	YES	plasmid	57250	57525	+		Halobacterium_salinarum_R1_uid61571	NC_010369	plasmid PHS2		arCOG08978	1	S		Uncharacterized conserved protein			
	169237672	hypothetical protein OE6114R	No	0	YES	plasmid	57725	58117	-		Halobacterium_salinarum_R1_uid61571	NC_010369	plasmid PHS2		arCOG09372	2	S		Uncharacterized conserved protein			
	169237673	IS240-type transposase (ISH102)	No	0	YES	plasmid	58192	58827	+		Halobacterium_salinarum_R1_uid61571	NC_010369	plasmid PHS2		arCOG02134	15 (-)	L		Transposase	3E-14	COG3316	Transposase and inactivated derivatives
	169237674	hypothetical protein OE6116R	No	0	YES	plasmid	58924	59154	-		Halobacterium_salinarum_R1_uid61571	NC_010369	plasmid PHS2		arCOG09373	2	S		Uncharacterized conserved protein			
	169237675	hypothetical protein OE6118F	No	0	YES	plasmid	59359	59772	+		Halobacterium_salinarum_R1_uid61571	NC_010369	plasmid PHS2		arCOG09374	2	S		Uncharacterized conserved protein			
	169237676	hypothetical protein OE6120F	No	0	YES	plasmid	59940	60227	+		Halobacterium_salinarum_R1_uid61571	NC_010369	plasmid PHS2									
===										5	Halobacterium_salinarum_R1_uid61571	NC_010369	plasmid PHS2									
	169237771	transposase (TCE33), nonfunctional N-terminal	No	0		plasmid	148944	149252	-		Halobacterium_salinarum_R1_uid61571	NC_010369	plasmid PHS2									
	169237772	PQQ repeat-containing protein	No	0		plasmid	149433	150548	-		Halobacterium_salinarum_R1_uid61571	NC_010369	plasmid PHS2		arCOG02556	2 (-)	S		WD40-like repeat	8E-29	COG1520	FOG: WD40-like repeat
	169237773	hypothetical protein OE6279R	No	0		plasmid	150849	151565	-		Halobacterium_salinarum_R1_uid61571	NC_010369	plasmid PHS2									
	169237774	hypothetical protein OE6280R	No	0		plasmid	151562	151819	-		Halobacterium_salinarum_R1_uid61571	NC_010369	plasmid PHS2									
	169237775	hypothetical protein OE6281R	No	0		plasmid	152049	152282	-		Halobacterium_salinarum_R1_uid61571	NC_010369	plasmid PHS2									
===										5	Halobacterium_salinarum_R1_uid61571	NC_010369	plasmid PHS2									
	169237790	hypothetical protein OE6323R	Yes	1		plasmid	175314	176303	-		Halobacterium_salinarum_R1_uid61571	NC_010369	plasmid PHS2									
	169237791	hypothetical protein OE6325R	No	1		plasmid	176306	179014	-		Halobacterium_salinarum_R1_uid61571	NC_010369	plasmid PHS2							0.000002	COG5295	Autotransporter adhesin
	169237792	hypothetical protein OE6329R	No	0		plasmid	179062	180243	-		Halobacterium_salinarum_R1_uid61571	NC_010369	plasmid PHS2		arCOG02202	4 (-)	D	FtsZ	Cell division GTPase	0.0000002	pfam00091	Tubulin/FtsZ family, GTPase domain. This
	169237793	coiled-coil protein	No	2		plasmid	180287	182359	-		Halobacterium_salinarum_R1_uid61571	NC_010369	plasmid PHS2							0.000001	COG1196	Chromosome segregation ATPases
	169237794	hypothetical protein OE6335R	Yes	2		plasmid	182356	183402	-		Halobacterium_salinarum_R1_uid61571	NC_010369	plasmid PHS2									
===										6	Haloferax_mediterranei_ATCC_33500_uid167315	NC_017941	chromosome									
	389846301	transcription regulator SirR	No	0			787465	787917	+		Haloferax_mediterranei_ATCC_33500_uid167315	NC_017941	chromosome		arCOG02100	2 (-)	K	TroR	Mn-dependent transcriptional regulator (DtxR fami	5E-22	COG1321	Mn-dependent transcriptional regulator [
	389846302	hypothetical protein HFX_0833	No	0			788446	788661	+		Haloferax_mediterranei_ATCC_33500_uid167315	NC_017941	chromosome									
	389846303	hypothetical protein HFX_0834	Yes	0			788708	789196	+		Haloferax_mediterranei_ATCC_33500_uid167315	NC_017941	chromosome							0.000001	pfam01456	Mucin-like glycoprotein. This family of
	389846304	hypothetical protein HFX_0835	No	0			789165	790646	+		Haloferax_mediterranei_ATCC_33500_uid167315	NC_017941	chromosome									
	389846305	hypothetical protein HFX_0836	No	1			790967	791119	+		Haloferax_mediterranei_ATCC_33500_uid167315	NC_017941	chromosome									
	389846306	sodium-dependent transporter	No	7			791257	792039	+		Haloferax_mediterranei_ATCC_33500_uid167315	NC_017941	chromosome		arCOG02191	1 (-)	R		Predicted Na+-dependent transporter	3E-15	COG0385	Predicted Na+-dependent transporter
===										5	Haloferax_mediterranei_ATCC_33500_uid167315	NC_017941	chromosome									
	389846337	hypothetical protein HFX_0868	No	0			823962	824726	-		Haloferax_mediterranei_ATCC_33500_uid167315	NC_017941	chromosome									
	389846338	lantibiotic modifying enzyme	No	0			824824	827967	-		Haloferax_mediterranei_ATCC_33500_uid167315	NC_017941	chromosome							2E-60	COG4403	Lantibiotic modifying enzyme
	389846339	acyl-CoA synthetase	No	0			828809	830797	+		Haloferax_mediterranei_ATCC_33500_uid167315	NC_017941	chromosome		arCOG01529	1 (-)	I	Acs	Acyl-coenzyme A synthetase/AMP-(fatty) acid liga	4E-129	COG0365	Acyl-coenzyme A synthetases/AMP-(fatty)
	389846340	hypothetical protein HFX_0871	No	0			830810	831055	-		Haloferax_mediterranei_ATCC_33500_uid167315	NC_017941	chromosome									
	389846341	hypothetical protein HFX_0872	No	0			831460	831819	-		Haloferax_mediterranei_ATCC_33500_uid167315	NC_017941	chromosome									
===										8	Haloferax_mediterranei_ATCC_33500_uid167315	NC_017941	chromosome									
	389846367	hypothetical protein HFX_0898	No	2			858146	858511	+		Haloferax_mediterranei_ATCC_33500_uid167315	NC_017941	chromosome									
	389846368	phage integrase	No	0			858912	860219	+		Haloferax_mediterranei_ATCC_33500_uid167315	NC_017941	chromosome		arCOG01243	4 (-)	L	XerC	Site-specific recombinase XerC	0.000001	pfam00589	Phage integrase family. Members of this
	389846369	hypothetical protein HFX_0900	No	0			860177	860338	-		Haloferax_mediterranei_ATCC_33500_uid167315	NC_017941	chromosome									
	389846370	hypothetical protein HFX_0901	No	0			860335	860604	-		Haloferax_mediterranei_ATCC_33500_uid167315	NC_017941	chromosome									
	389846371	hypothetical protein HFX_0903	No	0			860601	860933	-		Haloferax_mediterranei_ATCC_33500_uid167315	NC_017941	chromosome									
	389846372	hypothetical protein HFX_0902	No	0			860830	861072	+		Haloferax_mediterranei_ATCC_33500_uid167315	NC_017941	chromosome									
	389846373	hypothetical protein HFX_0904	No	0			860926	861132	-		Haloferax_mediterranei_ATCC_33500_uid167315	NC_017941	chromosome	clust_445								
	389846374	phage PhiH1 repressor protein	No	0			861352	861618	+		Haloferax_mediterranei_ATCC_33500_uid167315	NC_017941	chromosome		arCOG03924	10 (-)	K	MarR	Transcriptional regulator, MarR family			
===										12	Haloferax_mediterranei_ATCC_33500_uid167315	NC_017941	chromosome									
	389846376	cell division control protein cdc6-like protein	No	0			863681	864670	-		Haloferax_mediterranei_ATCC_33500_uid167315	NC_017941	chromosome		arCOG00467	13 (-)	L	CDC6	Cdc6-related protein, AAA superfamily ATPase	3E-33	COG1474	Cdc6-related protein, AAA superfamily AT
	389846377	hypothetical protein HFX_0908	No	0			865511	865750	-		Haloferax_mediterranei_ATCC_33500_uid167315	NC_017941	chromosome									
	389846378	hypothetical protein HFX_0909	No	2			866057	866452	+		Haloferax_mediterranei_ATCC_33500_uid167315	NC_017941	chromosome									
	389846379	hypothetical protein HFX_0910	Yes	2			866439	867929	+		Haloferax_mediterranei_ATCC_33500_uid167315	NC_017941	chromosome									
	389846380	hypothetical protein HFX_0911	No	3			867934	868392	+		Haloferax_mediterranei_ATCC_33500_uid167315	NC_017941	chromosome									
	389846381	hypothetical protein HFX_0912	No	2			868462	868818	+		Haloferax_mediterranei_ATCC_33500_uid167315	NC_017941	chromosome									
	389846382	hypothetical protein HFX_0913	No	2			868812	869276	+		Haloferax_mediterranei_ATCC_33500_uid167315	NC_017941	chromosome									

389846383	hypothetical protein HFX_0914	No	2		869273	869548	+		Haloferax_mediterranei_ATCC_33500_uid167315	NC_017941	chromosome										
389846384	hypothetical protein HFX_0915	No	4		869603	870025	+		Haloferax_mediterranei_ATCC_33500_uid167315	NC_017941	chromosome										
389846385	hypothetical protein HFX_0916	Yes	6		870038	871813	+		Haloferax_mediterranei_ATCC_33500_uid167315	NC_017941	chromosome										
389846386	hypothetical protein HFX_0917	No	0		871806	872579	+		Haloferax_mediterranei_ATCC_33500_uid167315	NC_017941	chromosome										
389846387	hypothetical protein HFX_0919	No	0		872690	874102	+		Haloferax_mediterranei_ATCC_33500_uid167315	NC_017941	chromosome			arCOG00438	2 (-)	R		AAA family ATPase			
===								29	Haloferax_mediterranei_ATCC_33500_uid167315	NC_017941	chromosome										
389846749	hypothetical protein HFX_1289	No	0	integrated_ele	1222387	1222947	+		Haloferax_mediterranei_ATCC_33500_uid167315	NC_017941	chromosome										
389846750	hypothetical protein HFX_1290	No	0	integrated_ele	1223160	1223525	+		Haloferax_mediterranei_ATCC_33500_uid167315	NC_017941	chromosome										
389846751	hypothetical protein HFX_1291	No	0	integrated_ele	1223522	1225648	+		Haloferax_mediterranei_ATCC_33500_uid167315	NC_017941	chromosome			arCOG06319	6 (-)	R		Protein containing HTH domain			
389846752	hypothetical protein HFX_1292	No	1	integrated_ele	1226029	1226550	+		Haloferax_mediterranei_ATCC_33500_uid167315	NC_017941	chromosome										
389846753	hypothetical protein HFX_1293	No	1	integrated_ele	1226617	1226820	-		Haloferax_mediterranei_ATCC_33500_uid167315	NC_017941	chromosome										
389846754	phage integrase	No	0	integrated_ele	1226915	1227535	-		Haloferax_mediterranei_ATCC_33500_uid167315	NC_017941	chromosome			arCOG02282	8 (-)	K		Predicted transcriptional regulator, C-terminal HTH-like domain			
389846755	hypothetical protein HFX_1295	No	0	integrated_ele	1227716	1229266	-		Haloferax_mediterranei_ATCC_33500_uid167315	NC_017941	chromosome										
389846756	hypothetical protein HFX_1296	No	0	integrated_ele	1231011	1231271	+		Haloferax_mediterranei_ATCC_33500_uid167315	NC_017941	chromosome										
389846757	hypothetical protein HFX_1297	No	0	integrated_ele	1231477	1231665	-		Haloferax_mediterranei_ATCC_33500_uid167315	NC_017941	chromosome										
389846758	hypothetical protein HFX_1298	No	2	integrated_ele	1231881	1232168	-		Haloferax_mediterranei_ATCC_33500_uid167315	NC_017941	chromosome										
389846759	hypothetical protein HFX_1299	No	0	integrated_ele	1232401	1232925	-		Haloferax_mediterranei_ATCC_33500_uid167315	NC_017941	chromosome			arCOG04347	4 (-)	Q		SAM-dependent methyltransferase	0.000004	pfam08241	Methyltransferase domain. Members of thi
389846760	hypothetical protein HFX_1300	No	0	integrated_ele	1233088	1234041	-		Haloferax_mediterranei_ATCC_33500_uid167315	NC_017941	chromosome			arCOG07960	16 (-)	R		ATPase (AAA+ superfamily)			
389846761	hypothetical protein HFX_1301	No	0	integrated_ele	1234038	1234604	-		Haloferax_mediterranei_ATCC_33500_uid167315	NC_017941	chromosome										
389846762	hypothetical protein HFX_1302	No	0	integrated_ele	1234594	1234845	-		Haloferax_mediterranei_ATCC_33500_uid167315	NC_017941	chromosome										
389846763	hypothetical protein HFX_1303	No	2	integrated_ele	1235032	1235529	+		Haloferax_mediterranei_ATCC_33500_uid167315	NC_017941	chromosome										
389846764	hypothetical protein HFX_1304	Yes	2	integrated_ele	1235577	1236005	+		Haloferax_mediterranei_ATCC_33500_uid167315	NC_017941	chromosome										
389846765	hypothetical protein HFX_1305	No	3	integrated_ele	1236002	1236301	+		Haloferax_mediterranei_ATCC_33500_uid167315	NC_017941	chromosome										
389846766	hypothetical protein HFX_1306	No	0	integrated_ele	1236301	1236891	+		Haloferax_mediterranei_ATCC_33500_uid167315	NC_017941	chromosome										
389846767	hypothetical protein HFX_1307	No	3	integrated_ele	1236898	1237764	+		Haloferax_mediterranei_ATCC_33500_uid167315	NC_017941	chromosome										
389846768	hypothetical protein HFX_1308	No	4	integrated_ele	1237765	1238223	+		Haloferax_mediterranei_ATCC_33500_uid167315	NC_017941	chromosome										
389846769	hypothetical protein HFX_1309	No	0	integrated_ele	1238224	1241004	+		Haloferax_mediterranei_ATCC_33500_uid167315	NC_017941	chromosome								5E-13	COG1196	Chromosome segregation ATPases
389846770	hypothetical protein HFX_1310	No	1	integrated_ele	1241004	1241258	+		Haloferax_mediterranei_ATCC_33500_uid167315	NC_017941	chromosome										
389846771	hypothetical protein HFX_1312	Yes	1	integrated_ele	1241530	1242156	+		Haloferax_mediterranei_ATCC_33500_uid167315	NC_017941	chromosome										
389846772	hypothetical protein HFX_1313	No	2	integrated_ele	1242223	1244286	+		Haloferax_mediterranei_ATCC_33500_uid167315	NC_017941	chromosome			arCOG02544	1 (-)	S		NosD-like periplasmic protein fused to PKD repe	0.000008	pfam13753	Putative flagellar system-associated rep
389846773	hypothetical protein HFX_1314	No	3	integrated_ele	1244658	1244951	+		Haloferax_mediterranei_ATCC_33500_uid167315	NC_017941	chromosome										
389846774	hypothetical protein HFX_1316	No	0	integrated_ele	1244783	1245088	-		Haloferax_mediterranei_ATCC_33500_uid167315	NC_017941	chromosome										
389846775	hypothetical protein HFX_1315	No	2	integrated_ele	1244948	1245193	+		Haloferax_mediterranei_ATCC_33500_uid167315	NC_017941	chromosome										
389846776	hypothetical protein HFX_1317	No	2	integrated_ele	1245190	1245543	+		Haloferax_mediterranei_ATCC_33500_uid167315	NC_017941	chromosome										
389846777	hypothetical protein HFX_1318	Yes	0	integrated_ele	1246636	1247463	+		Haloferax_mediterranei_ATCC_33500_uid167315	NC_017941	chromosome			arCOG06417	1 (-)	S		Uncharacterized conserved protein			
===								7	Haloferax_mediterranei_ATCC_33500_uid167315	NC_017941	chromosome										
389846789	hypothetical protein HFX_1330	No	0		1254715	1254918	+		Haloferax_mediterranei_ATCC_33500_uid167315	NC_017941	chromosome	clust_445									
389846790	DNA binding domain-containing protein	No	0		1254922	1255677	+		Haloferax_mediterranei_ATCC_33500_uid167315	NC_017941	chromosome			arCOG00006	2 (-)	K		Predicted transcriptional regulator, PadR family			
389846791	hypothetical protein HFX_1332	No	0		1255731	1256447	+		Haloferax_mediterranei_ATCC_33500_uid167315	NC_017941	chromosome										
389846792	hypothetical protein HFX_1333	No	0		1256444	1256761	+		Haloferax_mediterranei_ATCC_33500_uid167315	NC_017941	chromosome										
389846793	hypothetical protein HFX_1334	No	0		1256754	1257053	+		Haloferax_mediterranei_ATCC_33500_uid167315	NC_017941	chromosome										
389846794	phage integrase/site-specific recombinase	No	0		1257041	1258084	+		Haloferax_mediterranei_ATCC_33500_uid167315	NC_017941	chromosome			arCOG01250	7 (-)	L	XerC	Integrase	1E-11	COG4974	Site-specific recombinase XerD
389846795	hypothetical protein HFX_1336	No	0		1260496	1260699	+		Haloferax_mediterranei_ATCC_33500_uid167315	NC_017941	chromosome										
===								5	Haloferax_mediterranei_ATCC_33500_uid167315	NC_017941	chromosome										
389846951	hypothetical protein HFX_1495	No	2		1411752	1411970	-		Haloferax_mediterranei_ATCC_33500_uid167315	NC_017941	chromosome										
389846952	phage PhiH1 repressor protein	No	0		1412254	1412493	+		Haloferax_mediterranei_ATCC_33500_uid167315	NC_017941	chromosome			arCOG03924	10 (-)	K	MarR	Transcriptional regulator, MarR family			
389846953	hypothetical protein HFX_1497	No	0		1412593	1413681	+		Haloferax_mediterranei_ATCC_33500_uid167315	NC_017941	chromosome										
389846954	hypothetical protein HFX_1498	No	0		1414026	1414214	+		Haloferax_mediterranei_ATCC_33500_uid167315	NC_017941	chromosome										
389846955	hypothetical protein HFX_1499	No	0		1414709	1414951	-		Haloferax_mediterranei_ATCC_33500_uid167315	NC_017941	chromosome										
===								6	Haloferax_mediterranei_ATCC_33500_uid167315	NC_017941	chromosome										
389847130	hypothetical protein HFX_1676	No	0		1597988	1598674	+		Haloferax_mediterranei_ATCC_33500_uid167315	NC_017941	chromosome			arCOG02784	1 (-)	S		Uncharacterized conserved protein	1E-59	COG2135	Uncharacterized conserved protein
389847131	hypothetical protein HFX_1677	No	0		1598795	1600465	+		Haloferax_mediterranei_ATCC_33500_uid167315	NC_017941	chromosome										
389847132	hypothetical protein HFX_1678	No	0		1600527	1601723	+		Haloferax_mediterranei_ATCC_33500_uid167315	NC_017941	chromosome										
389847133	hypothetical protein HFX_1679	No	0		1601859	1603373	+		Haloferax_mediterranei_ATCC_33500_uid167315	NC_017941	chromosome								0.0001	COG2931	RTX toxins and related Ca2+-binding prot
389847134	hypothetical protein HFX_1680	No	0		1603433	1603633	+		Haloferax_mediterranei_ATCC_33500_uid167315	NC_017941	chromosome										
389847135	hypothetical protein HFX_1681	No	0		1605080	1605526	+		Haloferax_mediterranei_ATCC_33500_uid167315	NC_017941	chromosome			arCOG06216	1 (-)	S		Uncharacterized conserved protein			
===								6	Haloferax_mediterranei_ATCC_33500_uid167315	NC_017941	chromosome										
389847931	hypothetical protein HFX_2500	No	2		2447803	2448039	+		Haloferax_mediterranei_ATCC_33500_uid167315	NC_017941	chromosome										
389847932	hypothetical protein HFX_2501	No	0		2448119	2448541	+		Haloferax_mediterranei_ATCC_33500_uid167315	NC_017941	chromosome										
389847933	hypothetical protein HFX_2502	No	0		2448589	2449338	+		Haloferax_mediterranei_ATCC_33500_uid167315	NC_017941	chromosome										
389847934	hypothetical protein HFX_2503	No	0		2449580	2450500	-		Haloferax_mediterranei_ATCC_33500_uid167315	NC_017941	chromosome			arCOG02132	5 (-)	L		Transposase	0.0001	pfam12762	ISXO2-like transposase domain. This doma
389847935	hypothetical protein HFX_2504	No	0		2453516	2453809	-		Haloferax_mediterranei_ATCC_33500_uid167315	NC_017941	chromosome										
389847936	hypothetical protein HFX_2506	No	1		2455770	2456051	+		Haloferax_mediterranei_ATCC_33500_uid167315	NC_017941	chromosome										
===								11	Haloferax_mediterranei_ATCC_33500_uid167315	NC_017941	chromosome										
389848402	hypothetical protein HFX_2990	No	0		2899254	2899844	+		Haloferax_mediterranei_ATCC_33500_uid167315	NC_017941	chromosome										
389848403	site-specific recombinase XerD	No	0		2899845	2900897	+		Haloferax_mediterranei_ATCC_33500_uid167315	NC_017941	chromosome			arCOG01241	31 (-)	L	XerC	Integrase	4E-12	COG4974	Site-specific recombinase XerD
389848404	hypothetical protein HFX_2992	No	0		2900915	2901133	-		Haloferax_mediterranei_ATCC_33500_uid167315	NC_017941	chromosome										
389848405	hypothetical protein HFX_2993	No	0		2901379	2901537	+		Haloferax_mediterranei_ATCC_33500_uid167315	NC_017941	chromosome										
389848406	hypothetical protein HFX_2994	No	1		2901707	2901898	+		Haloferax_mediterranei_ATCC_33500_uid167315	NC_017941	chromosome										
389848407	hypothetical protein HFX_2995	No	2		2902038	2902256	+		Haloferax_mediterranei_ATCC_33500_uid167315	NC_017941	chromosome										
389848408	hypothetical protein HFX_2996	No	0		2902444	2903031	+		Haloferax_mediterranei_ATCC_33500_uid167315	NC_017941	chromosome										
389848409	hypothetical protein HFX_2997	No	0		2903590	2904153	+		Haloferax_mediterranei_ATCC_33500_uid167315	NC_017941	chromosome			arCOG02282	8 (-)	K		Predicted transcriptional regulator, C-terminal HTH-like domain			
389848410	hypothetical protein HFX_2998	No	0		2904411	2905412	-		Haloferax_mediterranei_ATCC_33500_uid167315	NC_017941	chromosome	clust_31									
389848411	hypothetical protein HFX_2999	No	0		2905417	2906094	-		Haloferax_mediterranei_ATCC_33500_uid167315	NC_017941	chromosome										
389848412	hypothetical protein HFX_3000	No	0		2906120	2908186	-		Haloferax_mediterranei_ATCC_33500_uid167315	NC_017941	chromosome								0.00009	COG0326	Molecular chaperone, HSP90 family
===								5	Haloferax_mediterranei_ATCC_33500_uid167315	NC_017941	chromosome										
389848415	hypothetical protein HFX_3003	No	0		2910768	2911793	-		Haloferax_mediterranei_ATCC_33500_uid167315	NC_017941	chromosome	clust_31									
389848416	hypothetical protein HFX_3004	No	0		2911793	2913877	-		Haloferax_mediterranei_ATCC_33500_uid167315	NC_017941	chromosome										
389848417	hypothetical protein HFX_3005	No	0		2914221	2914595	-		Haloferax_mediterranei_ATCC_33500_uid167315	NC_017941	chromosome			arCOG03828	5 (-)	S		Uncharacterized conserved protein			
389848418	hypothetical protein HFX_3006	No	0		2914641	2914877	-		Haloferax_mediterranei_ATCC_33500_uid167315	NC_017941	chromosome										
389848419	hypothetical protein HFX_3007	No	0		2915141	2915293	-		Haloferax_mediterranei_ATCC_33500_uid167315	NC_017941	chromosome										
===								6	Haloferax_mediterranei_ATCC_33500_uid167315	NC_017942	plasmid pHM100										
389848474	transposase	No	0	plasmid	28365	28514	-		Haloferax_mediterranei_ATCC_33500_uid167315	NC_017942	plasmid pHM100			arCOG02134	15 (-)	L		Transposase			
389848475	hypothetical protein HFX_4031	No	0	plasmid	28983	29228	-		Haloferax_mediterranei_ATCC_33500_uid167315	NC_017942	plasmid pHM100										
389848476	hypothetical protein HFX_4032	No	1	plasmid	29528	30115	+		Haloferax_mediterranei_ATCC_33500_uid167315	NC_017942	plasmid pHM100								0.0001	COG1196	Chromosome segregation ATPases
389848477	hypothetical protein HFX_4033	No	0	plasmid	30209	30376	-		Haloferax_mediterranei_ATCC_33500_uid167315	NC_017942	plasmid pHM100										
389848478	hypothetical protein HFX_4034	Yes	1	plasmid	31136	31960	+		Haloferax_mediterranei_ATCC_33500_uid167315	NC_017942	plasmid pHM100										
389848479	hypothetical protein HFX_4035	No	2	plasmid	32086	32901	+		Haloferax_mediterranei_ATCC_33500_uid167315	NC_017942	plasmid pHM100			arCOG06218	3 (-)	S		Uncharacterized conserved protein			
===								7	Haloferax_mediterranei_ATCC_33500_uid167315	NC_017942	plasmid pHM100										
389848518	hypothetical protein HFX_4075	No	0	plasmid	72778	72936	-		Haloferax_mediterranei_ATCC_33500_uid167315	NC_017942	plasmid pHM100										
389848519	hypothetical protein HFX_4076	No	3	plasmid	72999	74243	-		Haloferax_mediterranei_ATCC_33500_uid167315	NC_017942	plasmid pHM100		arCOG06218,a	arCOG06218	3 (-)	S		Uncharacterized conserved protein			
389848520	hypothetical protein HFX_4077	No	0	plasmid	75041	75334	+		Haloferax_mediterranei_ATCC_33500_uid167315	NC_017942	plasmid pHM100										
389848521	hypothetical protein HFX_4078	No	0	plasmid	75636	76559	+		Haloferax_mediterranei_ATCC_33500_uid167315	NC_017942	plasmid pHM100	clust_105							0.000000001	pfam14423	Immunity protein Imm5. A predicted Immun
389848522	hypothetical protein HFX_4079	No	1	plasmid	76665	76844	+		Haloferax_mediterranei_ATCC_33500_uid167315	NC_017942	plasmid pHM100										
389848523	hypothetical protein HFX_4080	No	2	plasmid	77026	77793	+		Haloferax_mediterranei_ATCC_33500_uid167315	NC_017942	plasmid pHM100			arCOG06218	3 (-)	S		Uncharacterized conserved protein			
389848524	hypothetical protein HFX_4083	No	0	plasmid	78299	78553	-		Haloferax_mediterranei_ATCC_33500_uid167315	NC_017942	plasmid pHM100			arCOG10932	3	S		Uncharacterized conserved protein			
===								8	Haloferax_mediterranei_ATCC_33500_uid167315	NC_017943	plasmid pHM300										
389848605	glycerol-3-phosphate ABC transporter periplas	Yes	0	plasmid	19162	20580	-		Haloferax_mediterranei_ATCC_33500_uid167315	NC_017943	plasmid pHM300			arCOG00153	1 (-)	G	UgpB	ABC-type trehalose transport system, periplasmi	4E-26	COG1653	ABC-type sugar transport system, peripla
389848606	hypothetical protein HFX_5019	No	0	plasmid	21697	21924	-		Haloferax_mediterranei_ATCC_33500_uid167315	NC_017943	plasmid pHM300										
389848607	hypothetical protein HFX_5020	No	0	plasmid	21921	22076	-		Haloferax_mediterranei_ATCC_33500_uid167315	NC_017943	plasmid pHM300										
389848608	G8 domain-containing protein	Yes	0	plasmid	22149	24296	+		Haloferax_mediterranei_ATCC_33500_uid167315	NC_017943	plasmid pHM300								3E-20	pfam10162	G8 domain. This domain is found in disea
389848609	hypothetical protein HFX_5022	No	0	plasmid	24399	24830	+		Haloferax_mediterranei_ATCC_33500_uid167315	NC_017943	plasmid pHM300										
389848610	hypothetical protein HFX_5023	No	2	plasmid	25195	25350	+		Haloferax_mediterranei_ATCC_33500_uid167315	NC_017943	plasmid pHM300										
389848611	hypothetical protein HFX_5024	No	0	plasmid	25225	25395	-		Haloferax_mediterranei_ATCC_33500_uid167315	NC_017943	plasmid pHM300										
389848612	sugar ABC transporter ATP-binding protein	No	0	plasmid	25887	27017	-		Haloferax_mediterranei_ATCC_33500_uid167315	NC_017943	plasmid pHM300			arCOG00177	2 (-)	E	PotA	ABC-type spermidine/putrescine transport systen	4E-115	COG3839	ABC-type sugar transport systems, ATPase
===								7	Haloferax_mediterranei_ATCC_33500_uid167315	NC_017943	plasmid pHM500										
389848940	hypothetical protein HFX_6062	No	0	plasmid	70075	70341	-		Haloferax_mediterranei_ATCC_33500_uid167315	NC_017944	plasmid pHM500			arCOG08021	1 (-)	K		Predicted transcriptional regulator containing an HTH domain			
389848941	hypothetical protein HFX_6063	No	3	plasmid	73265	73954	+		Haloferax_mediterranei_ATCC_33500_uid167315	NC_017944	plasmid pHM500										
389848942	hypothetical protein HFX_6064	No	0	plasmid	74494	78717	+		Haloferax_mediterranei_ATCC_33500_uid167315	NC_017944	plasmid pHM500	clust_10									
389848943	hypothetical protein HFX_6065	No	0	plasmid	78773	82900	-		Haloferax_mediterranei_ATCC_33500_uid167315	NC_017944	plasmid pHM500										
389848944	hypothetical protein HFX_6066	No	0	plasmid	83221	83556	+		Haloferax_mediterranei_ATCC_33500_uid167315	NC_017944	plasmid pHM500	clust_40									
389848945	hypothetical protein HFX_6067	Yes	0	plasmid	83775	85019	+		Haloferax_mediterranei_ATCC_33500_uid167315	NC_017944	plasmid pHM500			arCOG02545	3 (-)	S		NosD-like periplasmic protein fused to PKD repeat domain			
389848946	hypothetical protein HFX_6068	No	0	plasmid	85499	85759	-		Haloferax_mediterranei_ATCC_33500_uid167315	NC_017944	plasmid pHM500	clust_22									
===								8	Haloferax_volcanii_DS2_uid46845	NC_013964	plasmid pHV3										
292494228	ABC-type transport system ATP-binding protei	No	0	plasmid	343855	344988	+		Haloferax_volcanii_DS2_uid46845	NC_013964	plasmid pHV3			arCOG00177	2 (-)	E	PotA	ABC-type spermidine/putrescine transport systen	1E-117	COG3839	ABC-type sugar transport systems, ATPase
292494229	hypothetical protein HVO_B0296	No	0	plasmid	345036	345437	-		Haloferax_volcanii_DS2_uid46845	NC_013964	plasmid pHV3										
292494230	hypothetical protein HVO_B0297	No	0	plasmid	345549	345725	-		Haloferax_volcanii_DS2_uid46845	NC_013964	plasmid pHV3										
292494231	hypothetical protein HVO_B0298	No	0	plasmid	345684	345833	+		Haloferax_volcanii_DS2_uid46845	NC_013964	plasmid pHV3										
292494232	Hypothetical transthyretin-like protein	No	0	plasmid	346327	346677	-		Haloferax_volcanii_DS2_uid46845	NC_013964	plasmid pHV3	clust_304							2E-33	COG2351	Transthyretin-like protein
292494233	Uricase	No	0	plasmid	346679	347605	-		Haloferax_volcanii_DS2_uid46845	NC_013964	plasmid pHV3	clust_99							2E-52	COG3648	Uricase (urate oxidase)
292494234	Uricase	No	0	plasmid	347763	348269	-		Haloferax_volcanii_DS2_uid46845	NC_013964	plasmid pHV3	clust_198							2E-51	COG3195	Uncharacterized protein conserved in bac
292494235	D-hydantoinase	No	0	plasmid	348353	349735	-		Haloferax_volcanii_DS2_uid46845	NC_013964	plasmid pHV3			arCOG00689	1 (-)	F	PyrC	Dihydroorotase or related cyclic amidohydrolase	7E-72	COG0044	Dihydroorotase and related cyclic amidoh
===								5	Haloferax_volcanii_DS2_uid46845	NC_013966	plasmid pHV4										
292653585	hypothetical protein HVO_A0020	No	3	plasmid	22306	23010	+		Haloferax_volcanii_DS2_uid46845	NC_013966	plasmid pHV4			arCOG10633	1	S		Uncharacterized conserved protein			
292653586	hypothetical protein HVO_A0021	No	0	plasmid	23099	23494	-		Haloferax_volcanii_DS2_uid46845	NC_013966	plasmid pHV4			arCOG10820	1	S		Uncharacterized conserved protein			
292653587	hypothetical protein HVO_A0022	No	0	plasmid	23617	24615	-		Haloferax_volcanii_DS2_uid46845	NC_013966	plasmid pHV4										
292653588	hypothetical protein HVO_A0023	No	0	plasmid	24692	24859	-		Haloferax_volcanii_DS2_uid46845	NC_013966	plasmid pHV4			arCOG02989	1 (-)	S		Uncharacterized conserved protein			
292653589	hypothetical protein HVO_A0024	No	0	plasmid	25023	25706	+		Haloferax_volcanii_DS2_uid46845	NC_013966	plasmid pHV4										
===								7	Haloferax_volcanii_DS2_uid46845	NC_013966	plasmid pHV4										
292653670	hypothetical protein HVO_A0107	No	0	plasmid	109523	109765	-		Haloferax_volcanii_DS2_uid46845	NC_013966	plasmid pHV4										
292653671	hypothetical protein HVO_A0108	No	0	plasmid	109784	110275	-		Haloferax_volcanii_DS2_uid46845	NC_013966	plasmid pHV4			arCOG08894	1	S		Uncharacterized conserved protein			
292653672	lipoprotein, putative	Yes	1	plasmid	110549	110767	+		Haloferax_volcanii_DS2_uid46845	NC_013966	plasmid pHV4										
292653673	transposase (ISH51)	No	0	plasmid	110914	112080	-		Haloferax_volcanii_DS2_uid46845	NC_013966	plasmid pHV4			arCOG06160	11 (-)	L		Transposase			
292653674	hypothetical protein HVO_A0111	No	0	plasmid	112252	112407	-		Haloferax_volcanii_DS2_uid46845	NC_013966	plasmid pHV4										
292653675	hypothetical protein HVO_A0112	No	1	plasmid	112701	112814	-		Haloferax_volcanii_DS2_uid46845	NC_013966	plasmid pHV4										
292653676	hypothetical protein HVO_A0113	No	0	plasmid	112878	113051	-		Haloferax_volcanii_DS2_uid46845	NC_013966	plasmid pHV4										
===								5	Haloferax_volcanii_DS2_uid46845	NC_013966	plasmid pHV4										
292653726	hypothetical protein HVO_A0166	No	0	plasmid	168752	169117	+		Haloferax_volcanii_DS2_uid46845	NC_013966	plasmid pHV4										
292653727	hypothetical protein HVO_A0167	No	0	plasmid	169148	169276	+		Haloferax_volcanii_DS2_uid46845	NC_013966	plasmid pHV4										
292653728	bacterio-opsin activator-like protein	No	0	plasmid	169386	170126	-		Haloferax_volcanii_DS2_uid46845	NC_013966	plasmid pHV4			arCOG02274	3 (-)	K		Predicted transcriptional regulator, C-terminal HTI	6E-16	COG3413	Predicted DNA binding protein
292653729	HTR-like protein	No	3	plasmid	170366	171283	+		Haloferax_volcanii_DS2_uid46845	NC_013966	plasmid pHV4		arCOG02673	arCOG02673	2	U		OxaA/SpoJ/YigC translocase/secretase, sec-inde	8E-42	COG1422	Predicted membrane protein
292653730	hypothetical protein HVO_A0170	No	0	plasmid	171389	171826	+		Haloferax_volcanii_DS2_uid46845	NC_013966	plasmid pHV4										
===								7	Haloferax_volcanii_DS2_uid46845	NC_013966	plasmid pHV4										
292653759	hypothetical protein HVO_A0199	No	0	plasmid	202720	202926	-		Haloferax_volcanii_DS2_uid46845	NC_013966	plasmid pHV4										
292653760	Asparagine synthase family	No	0	plasmid	202975	204816	+		Haloferax_volcanii_DS2_uid46845	NC_013966	plasmid pHV4			arCOG00121	3 (-)	E	AsnB	Asparagine synthase (glutamine-hydrolyzing)	4E-10	COG0367	Asparagine synthase (glutamine-hydrolyzi
292653761	hypothetical protein HVO_A0201	No	0	plasmid	204978	205235	-		Haloferax_volcanii_DS2_uid46845	NC_013966	plasmid pHV4										
292653762	hypothetical protein HVO_A0202	No	0	plasmid	205969	206082	-		Haloferax_volcanii_DS2_uid46845	NC_013966	plasmid pHV4										
292653763	hypothetical protein HVO_A0203	No	0	plasmid	206782	207343	-		Haloferax_volcanii_DS2_uid46845	NC_013966	plasmid pHV4										
292653764	hypothetical protein HVO_A0204	No	0	plasmid	207464	207595	-		Haloferax_volcanii_DS2_uid46845	NC_013966	plasmid pHV4										
292653765	CRISPR-associated protein Cas6	No	0	plasmid	207954	208772	+		Haloferax_volcanii_DS2_uid46845	NC_013966	plasmid pHV4			arCOG04342	2 (-)	V		CRISPR system related protein Cas6, RAMP superfamily			
===								6	Haloferax_volcanii_DS2_uid46845	NC_013966	plasmid pHV4										
292653791	hypothetical protein HVO_A0231	No	0	plasmid	235086	236375	-		Haloferax_volcanii_DS2_uid46845	NC_013966	plasmid pHV4			arCOG06786	9	Y		Rolling circle replication protein Rep			
292653792	hypothetical protein HVO_A0232	No	0	plasmid	236463	236645	-		Haloferax_volcanii_DS2_uid46845	NC_013966	plasmid pHV4										
292653793	hypothetical protein HVO_A0233	No	0	plasmid	237010	237321	-		Haloferax_volcanii_DS2_uid46845	NC_013966	plasmid pHV4										
292653794	hypothetical protein HVO_A0234	No	0	plasmid	237346	237771	-		Haloferax_volcanii_DS2_uid46845	NC_013966	plasmid pHV4			arCOG05829	1 (-)	K		Predicted transcriptional regulator, contains HTH domain			
292653795	hypothetical protein HVO_A0235	No	0	plasmid	237783	238007	-		Haloferax_volcanii_DS2_uid46845	NC_013966	plasmid pHV4										
292653796	hypothetical protein HVO_A0236	No	0	plasmid	238018	238752	-		Haloferax_volcanii_DS2_uid46845	NC_013966	plasmid pHV4										
===								8	Haloferax_volcanii_DS2_uid46845	NC_013967	chromosome										
292654447	hypothetical protein HVO_0266	No	0	Virus_Pleolipo	237743	237988	+		Haloferax_volcanii_DS2_uid46845	NC_013967	chromosome			arCOG03087	3 (-)	R		Predicted RNA binding protein (dsRBD-like fold),	0.0000002	COG1724	Predicted RNA binding protein (dsRBD-lik
292654448	hypothetical protein HVO_0267	No	0	Virus_Pleolipo	237992	238198	-		Haloferax_volcanii_DS2_uid46845	NC_013967	chromosome										
292654449	hypothetical protein HVO_0268	No	4	Virus_Pleolipo	238338	238736	+		Haloferax_volcanii_DS2_uid46845	NC_013967	chromosome										
292654450	hypothetical protein HVO_0269	No	0	Virus_Pleolipo	238733	238948	+		Haloferax_volcanii_DS2_uid46845	NC_013967	chromosome										
292654451	hypothetical protein HVO_0270	Yes	2	Virus_Pleolipo	238945	239475	+		Haloferax_volcanii_DS2_uid46845	NC_013967	chromosome										
292654452	hypothetical protein HVO_0271	No	3	Virus_Pleolipo	239482	241095	+		Haloferax_volcanii_DS2_uid46845	NC_013967	chromosome			arCOG07782	7 (-)	S		Uncharacterized conserved protein			
292654453	hypothetical protein HVO_0272	Yes	2	Virus_Pleolipo	241129	241704	+		Haloferax_volcanii_DS2_uid46845	NC_013967	chromosome										
292654454	hypothetical protein HVO_0273	No	0	Virus_Pleolipo	241698	242492	+		Haloferax_volcanii_DS2_uid46845	NC_013967	chromosome										
===								18	Haloferax_volcanii_DS2_uid46845	NC_013967	chromosome										
292654538	30S ribosomal protein S10	No	0		328944	329252	+		Haloferax_volcanii_DS2_uid46845	NC_013967	chromosome			arCOG01758	2 (-)	J	RpsJ	Ribosomal protein S10	4E-20	COG0051	Ribosomal protein S10
292654539	hypothetical protein HVO_0362	No	0		329856	330458	+		Haloferax_volcanii_DS2_uid46845	NC_013967	chromosome										
292654540	hypothetical protein HVO_0364	No	0		330652	331095	+		Haloferax_volcanii_DS2_uid46845	NC_013967	chromosome										
292654541	hypothetical protein HVO_0363	No	1		331092	331232	-		Haloferax_volcanii_DS2_uid46845	NC_013967	chromosome										
292654542	hypothetical protein HVO_0365	No	1		331222	331422	-		Haloferax_volcanii_DS2_uid46845	NC_013967	chromosome										
292654543	hypothetical protein HVO_0366	No	1		331415	331801	-		Haloferax_volcanii_DS2_uid46845	NC_013967	chromosome										
292654544	hypothetical protein HVO_0367	No	1		331803	332225	-		Haloferax_volcanii_DS2_uid46845	NC_013967	chromosome										
292654545	hypothetical protein HVO_0368	No	1		332222	332788	-		Haloferax_volcanii_DS2_uid46845	NC_013967	chromosome								0.00003	COG2931	RTX toxins and related Ca2+-binding prot
292654546	hypothetical protein HVO_0369	No	0		332788	332943	-		Haloferax_volcanii_DS2_uid46845	NC_013967	chromosome										
292654547	hypothetical protein HVO_0370	No	0		332943	333431	-		Haloferax_volcanii_DS2_uid46845	NC_013967	chromosome										
292654548	hypothetical protein HVO_0371	No	0		333431	333871	-		Haloferax_volcanii_DS2_uid46845	NC_013967	chromosome										
292654549	hypothetical protein HVO_0372	No	0		333882	334388	-		Haloferax_volcanii_DS2_uid46845	NC_013967	chromosome										
292654550	sugar kinase	No	1		334392	334589	-		Haloferax_volcanii_DS2_uid46845	NC_013967	chromosome										
292654551	hypothetical protein HVO_0374	No	0		334719	334901	+		Haloferax_volcanii_DS2_uid46845	NC_013967	chromosome										
292654552	hypothetical protein HVO_0375	No	0		335021	335359	+		Haloferax_volcanii_DS2_uid46845	NC_013967	chromosome										
292654553	hypothetical protein HVO_0376	No	0		335352	335792	+		Haloferax_volcanii_DS2_uid46845	NC_013967	chromosome										
292654554	hypothetical protein HVO_0377	No	0		335789	336409	+		Haloferax_volcanii_DS2_uid46845	NC_013967	chromosome			arCOG00280	21 (-)	L		HerA helicase			
292654555	hypothetical protein HVO_0378	No	0		336406	336630	+		Haloferax_volcanii_DS2_uid46845	NC_013967	chromosome										
===								5	Haloferax_volcanii_DS2_uid46845	NC_013967	chromosome										
292654967	hypothetical protein HVO_0804	No	0		723854	724015	-		Haloferax_volcanii_DS2_uid46845	NC_013967	chromosome										
292654968	hypothetical protein HVO_0805	No	1		724281	724529	-		Haloferax_volcanii_DS2_uid46845	NC_013967	chromosome										
292654969	pyruvate kinase	No	0		724574	726331	-		Haloferax_volcanii_DS2_uid46845	NC_013967	chromosome		arCOG01112,a	arCOG01112	1 (-)	G		PEP-utilising enzyme associated, "swivelling" be	7E-136	COG0469	Pyruvate kinase
292654970	hypothetical protein HVO_0807	Yes	0		726446	726733	-		Haloferax_volcanii_DS2_uid46845	NC_013967	chromosome	clust_348									
292654971	hypothetical protein HVO_0808	Yes	1		726805	727110	-		Haloferax_volcanii_DS2_uid46845	NC_013967	chromosome	clust_348									
===								6	Haloferax_volcanii_DS2_uid46845	NC_013967	chromosome										
292655582	hypothetical protein HVO_1429	Yes	2		1301654	1302247	-		Haloferax_volcanii_DS2_uid46845	NC_013967	chromosome										
292655583	hypothetical protein HVO_1430	No	1		1302282	1302455	-		Haloferax_volcanii_DS2_uid46845	NC_013967	chromosome										
292655584	hypothetical protein HVO_1431	No	0		1302457	1303896	-		Haloferax_volcanii_DS2_uid46845	NC_013967	chromosome			arCOG07782	7 (-)	S		Uncharacterized conserved protein			
292655585	fibronectin type III domain-containing protein	No	4		1304083	1304490	-		Haloferax_volcanii_DS2_uid46845	NC_013967	chromosome			arCOG08954	3	S		Uncharacterized conserved protein			
292655586	hypothetical protein HVO_1433	No	0		1304490	1304843	-		Haloferax_volcanii_DS2_uid46845	NC_013967	chromosome										
292655587	hypothetical protein HVO_1434	No	0		1304945	1306849	-		Haloferax_volcanii_DS2_uid46845	NC_013967	chromosome			arCOG06786	9	Y		Rolling circle replication protein Rep			
===								10	Haloferax_volcanii_DS2_uid46845	NC_013967	chromosome										
292656384	hypothetical protein HVO_2257	No	0		2116907	2117077	+		Haloferax_volcanii_DS2_uid46845	NC_013967	chromosome										
292656385	hypothetical protein HVO_2258	No	0		2117186	2117695	+		Haloferax_volcanii_DS2_uid46845	NC_013967	chromosome										
292656386	XerC/D-like integrase	No	0		2117697	2118632	+		Haloferax_volcanii_DS2_uid46845	NC_013967	chromosome			arCOG01241	31 (-)	L	XerC	Integrase	6E-27	COG4974	Site-specific recombinase XerD
292656387	hypothetical protein HVO_2260	No	0		2118646	2118960	+		Haloferax_volcanii_DS2_uid46845	NC_013967	chromosome										
292656388	hypothetical protein HVO_2261	No	0		2119332	2119541	+		Haloferax_volcanii_DS2_uid46845	NC_013967	chromosome										
292656389	hypothetical protein HVO_2262	No	0		2119545	2119757	+		Haloferax_volcanii_DS2_uid46845	NC_013967	chromosome										
292656390	hypothetical protein HVO_2263	No	0		2119911	2122316	+		Haloferax_volcanii_DS2_uid46845	NC_013967	chromosome			arCOG07786	2 (-)	K		Predicted Helicase fused to HTH domain			
292656391	hypothetical protein HVO_2264	No	1		2122480	2122746	-		Haloferax_volcanii_DS2_uid46845	NC_013967	chromosome										
292656392	hypothetical protein HVO_2265	No	0		2122933	2123286	+		Haloferax_volcanii_DS2_uid46845	NC_013967	chromosome										
292656393	hypothetical protein HVO_2266	No	0		2123279	2124760	+		Haloferax_volcanii_DS2_uid46845	NC_013967	chromosome										
===								7	Haloferax_volcanii_DS2_uid46845	NC_013967	chromosome										
292656409	transfer complex protein N-terminal part-like pr	No	1		2146377	2146628	-		Haloferax_volcanii_DS2_uid46845	NC_013967	chromosome										
292656410	hypothetical protein HVO_2283	Yes	7		2146625	2147704	-		Haloferax_volcanii_DS2_uid46845	NC_013967	chromosome			arCOG07767	1 (-)	S		Uncharacterized conserved protein			
292656411	hypothetical protein HVO_2284	Yes	2		2147711	2148052	-		Haloferax_volcanii_DS2_uid46845	NC_013967	chromosome										
292656412	hypothetical protein HVO_2285	No	0		2149317	2149598	-		Haloferax_volcanii_DS2_uid46845	NC_013967	chromosome										
292656413	hypothetical protein HVO_2286	Yes	0		2150003	2151682	-		Haloferax_volcanii_DS2_uid46845	NC_013967	chromosome										
292656414	hypothetical protein HVO_2287	No	0		2151899	2152816	+		Haloferax_volcanii_DS2_uid46845	NC_013967	chromosome	clust_91									
292656415	hypothetical protein HVO_2288	No	2		2153749	2155278	+		Haloferax_volcanii_DS2_uid46845	NC_013967	chromosome			arCOG05096	3 (-)	S		Uncharacterized conserved membrane protein			
===								6	Haloferax_volcanii_DS2_uid46845	NC_013968	plasmid pHV1										
292657131	hypothetical protein HVO_C0007	No	0	plasmid	5743	6006	-		Haloferax_volcanii_DS2_uid46845	NC_013968	plasmid pHV1			arCOG02413	1 (-)	R		Predicted nuclease of the RNAse H fold, HicB family			
292657132	hypothetical protein HVO_C0008	No	0	plasmid	6138	6419	-		Haloferax_volcanii_DS2_uid46845	NC_013968	plasmid pHV1										
292657133	hypothetical protein HVO_C0009	No	0	plasmid	6521	6970	+		Haloferax_volcanii_DS2_uid46845	NC_013968	plasmid pHV1										
292657134	hypothetical protein HVO_C0010	No	0	plasmid	7142	7501	+		Haloferax_volcanii_DS2_uid46845	NC_013968	plasmid pHV1										
292657135	hypothetical protein HVO_C0011	No	0	plasmid	7721	9085	+		Haloferax_volcanii_DS2_uid46845	NC_013968	plasmid pHV1										
292657136	hypothetical protein HVO_C0012	No	0	plasmid	9296	9616	+		Haloferax_volcanii_DS2_uid46845	NC_013968	plasmid pHV1			arCOG03939	1 (-)	K		RHH-fold DNA-binding ptotein			
===								8	Haloferax_volcanii_DS2_uid46845	NC_013968	plasmid pHV1										
292657142	hypothetical protein HVO_C0019	No	0	plasmid	13959	14078	-		Haloferax_volcanii_DS2_uid46845	NC_013968	plasmid pHV1										
292657143	transposase (ISH51)	No	0	plasmid	15191	16360	+		Haloferax_volcanii_DS2_uid46845	NC_013968	plasmid pHV1			arCOG06160	11 (-)	L		Transposase			
292657144	hypothetical protein HVO_C0022	No	0	plasmid	16507	16875	-		Haloferax_volcanii_DS2_uid46845	NC_013968	plasmid pHV1										
292657145	hypothetical protein HVO_C0023	No	0	plasmid	17018	17512	-		Haloferax_volcanii_DS2_uid46845	NC_013968	plasmid pHV1										
292657146	hypothetical protein HVO_C0025	No	0	plasmid	17716	17973	-		Haloferax_volcanii_DS2_uid46845	NC_013968	plasmid pHV1										
292657147	hypothetical protein HVO_C0026	No	0	plasmid	18022	18489	-		Haloferax_volcanii_DS2_uid46845	NC_013968	plasmid pHV1										
292657148	hypothetical protein HVO_C0027	No	0	plasmid	18658	18852	+		Haloferax_volcanii_DS2_uid46845	NC_013968	plasmid pHV1			arCOG02625	3 (-)	R		Predicted metal-dependent hydrolase	0.000000001	COG1451	Predicted metal-dependent hydrolase
292657149	hypothetical protein HVO_C0028	No	0	plasmid	18908	19882	+		Haloferax_volcanii_DS2_uid46845	NC_013968	plasmid pHV1										
===								9	Haloferax_volcanii_DS2_uid46845	NC_013968	plasmid pHV1										
292657194	chromosome segregation protein, putative	No	0	plasmid	81271	81825	-		Haloferax_volcanii_DS2_uid46845	NC_013968	plasmid pHV1			arCOG00374	2 (-)	K		Predicted membrane-associated trancriptional re	0.0000002	COG1196	Chromosome segregation ATPases
292657195	hypothetical protein HVO_C0082	No	0	plasmid	81876	82394	-		Haloferax_volcanii_DS2_uid46845	NC_013968	plasmid pHV1										
292657196	hypothetical protein HVO_C0083	No	0	plasmid	82451	82960	+		Haloferax_volcanii_DS2_uid46845	NC_013968	plasmid pHV1										
292657197	hypothetical protein HVO_C0084	No	0	plasmid	83151	83267	-		Haloferax_volcanii_DS2_uid46845	NC_013968	plasmid pHV1										
292657198	hypothetical protein HVO_C0085	No	0	plasmid	83320	83664	-		Haloferax_volcanii_DS2_uid46845	NC_013968	plasmid pHV1										
292657199	hypothetical protein HVO_C0086	No	0	plasmid	83661	83855	-		Haloferax_volcanii_DS2_uid46845	NC_013968	plasmid pHV1										
292657200	hypothetical protein HVO_C0087	No	0	plasmid	83852	84292	-		Haloferax_volcanii_DS2_uid46845	NC_013968	plasmid pHV1										
292657201	hypothetical protein HVO_C0088	No	1	plasmid	84301	84525	-		Haloferax_volcanii_DS2_uid46845	NC_013968	plasmid pHV1										
292657202	hypothetical protein HVO_C0089	No	0	plasmid	84868	85038	-		Haloferax_volcanii_DS2_uid46845	NC_013968	plasmid pHV1										
===								9	Halogeometricum_borinquense_DSM_11551_uid	NC_014729	chromosome										
313124813	hypothetical protein Hbor_00250	No	3		17390	17983	-		Halogeometricum_borinquense_DSM_11551_uid	NC_014729	chromosome										
313124814	monomeric archaeal DNA polymerase sliding (	No	0		18104	18847	-		Halogeometricum_borinquense_DSM_11551_uid	NC_014729	chromosome			arCOG00488	4 (-)	L	DnaN	DNA polymerase sliding clamp subunit (PCNA hc	6E-12	COG0592	DNA polymerase sliding clamp subunit (PC
313124815	hypothetical protein Hbor_00270	No	0		19068	19520	-		Halogeometricum_borinquense_DSM_11551_uid	NC_014729	chromosome										
313124816	hypothetical protein Hbor_00280	Yes	1		19852	20103	-		Halogeometricum_borinquense_DSM_11551_uid	NC_014729	chromosome	clust_8									
313124817	hypothetical protein Hbor_00290	Yes	1		20108	20335	-		Halogeometricum_borinquense_DSM_11551_uid	NC_014729	chromosome	clust_8									
313124818	hypothetical protein Hbor_00300	Yes	1		20362	20583	-		Halogeometricum_borinquense_DSM_11551_uid	NC_014729	chromosome	clust_8									
313124819	hypothetical protein Hbor_00310	Yes	0		20609	20836	-		Halogeometricum_borinquense_DSM_11551_uid	NC_014729	chromosome	clust_8									
313124820	hypothetical protein Hbor_00320	Yes	1		20862	21086	-		Halogeometricum_borinquense_DSM_11551_uid	NC_014729	chromosome	clust_8									
313124821	hypothetical protein Hbor_00330	No	7		22575	24245	-		Halogeometricum_borinquense_DSM_11551_uid	NC_014729	chromosome		arCOG02142,a	arCOG02142	1 (-)	S		Uncharacterized homolog of PrgY (pheromone s	7E-33	COG1916	Uncharacterized homolog of PrgY (pheromo ; Uncharacterized homolog of PrgY (pheromo
===								9	Halogeometricum_borinquense_DSM_11551_uid	NC_014729	chromosome										
313125468	hypothetical protein Hbor_06950	No	0		642271	642438	+		Halogeometricum_borinquense_DSM_11551_uid	NC_014729	chromosome										
313125469	hypothetical protein Hbor_06960	No	1		642740	643573	-		Halogeometricum_borinquense_DSM_11551_uid	NC_014729	chromosome			arCOG08109	2 (-)	S		Uncharacterized conserved protein			
313125470	hypothetical protein Hbor_06970	No	0		644184	644423	-		Halogeometricum_borinquense_DSM_11551_uid	NC_014729	chromosome										
313125471	hypothetical protein Hbor_06980	No	0		644992	645252	+		Halogeometricum_borinquense_DSM_11551_uid	NC_014729	chromosome										
313125472	hypothetical protein Hbor_06990	No	0		645245	645658	+		Halogeometricum_borinquense_DSM_11551_uid	NC_014729	chromosome			arCOG10381	2	S		Uncharacterized conserved protein			
313125473	hypothetical protein Hbor_07000	No	0		645662	646111	-		Halogeometricum_borinquense_DSM_11551_uid	NC_014729	chromosome										
313125474	hypothetical protein Hbor_07010	No	0		646108	647817	-		Halogeometricum_borinquense_DSM_11551_uid	NC_014729	chromosome		arCOG00385,a	arCOG00385	1 (-)	S		Uncharacterized conserved protein	3E-30	COG1196	Chromosome segregation ATPases
313125475	hypothetical protein Hbor_07020	No	4		647807	648322	-		Halogeometricum_borinquense_DSM_11551_uid	NC_014729	chromosome										
313125476	hypothetical protein Hbor_07030	No	0		648402	652601	-		Halogeometricum_borinquense_DSM_11551_uid	NC_014729	chromosome								0.0000001	COG1196	Chromosome segregation ATPases
===								5	Halogeometricum_borinquense_DSM_11551_uid	NC_014729	chromosome										
313125539	hypothetical protein Hbor_07670	No	0		710799	711227	-		Halogeometricum_borinquense_DSM_11551_uid	NC_014729	chromosome	clust_244									
313125540	hypothetical protein Hbor_07680	No	0		711232	711984	-		Halogeometricum_borinquense_DSM_11551_uid	NC_014729	chromosome										
313125541	hypothetical protein Hbor_07690	No	0		712757	714499	-		Halogeometricum_borinquense_DSM_11551_uid	NC_014729	chromosome			arCOG10869	2 (-)	S		Uncharacterized conserved protein	8E-16	pfam13699	Domain of unknown function (DUF4157). Th
313125542	hypothetical protein Hbor_07700	No	3		714755	715138	-		Halogeometricum_borinquense_DSM_11551_uid	NC_014729	chromosome	clust_277									
313125543	hypothetical protein Hbor_07710	Yes	3		715135	715494	-		Halogeometricum_borinquense_DSM_11551_uid	NC_014729	chromosome	clust_299									
===								6	Halogeometricum_borinquense_DSM_11551_uid	NC_014729	chromosome										
313126098	hypothetical protein Hbor_13400	No	0		1254608	1254988	-		Halogeometricum_borinquense_DSM_11551_uid	NC_014729	chromosome										
313126099	hypothetical protein Hbor_13420	No	0		1255252	1256013	-		Halogeometricum_borinquense_DSM_11551_uid	NC_014729	chromosome										
313126100	hypothetical protein Hbor_13430	No	0		1256013	1257785	-		Halogeometricum_borinquense_DSM_11551_uid	NC_014729	chromosome			arCOG03900	5 (-)	L		Transposase			
313126101	hypothetical protein Hbor_13440	No	0		1258315	1258467	-		Halogeometricum_borinquense_DSM_11551_uid	NC_014729	chromosome										
313126102	hypothetical protein Hbor_13450	No	0		1258528	1258752	+		Halogeometricum_borinquense_DSM_11551_uid	NC_014729	chromosome										
313126103	hypothetical protein Hbor_13460	No	3		1258756	1259490	-		Halogeometricum_borinquense_DSM_11551_uid	NC_014729	chromosome										
===								5	Halogeometricum_borinquense_DSM_11551_uid	NC_014729	chromosome										
313126520	hypothetical protein Hbor_17770	Yes	0		1706745	1707992	+		Halogeometricum_borinquense_DSM_11551_uid	NC_014729	chromosome										
313126521	hypothetical protein Hbor_17780	No	0		1707995	1708999	+		Halogeometricum_borinquense_DSM_11551_uid	NC_014729	chromosome			arCOG09358	1	S		Uncharacterized conserved protein			
313126522	hypothetical protein Hbor_17790	No	1		1709393	1709506	+		Halogeometricum_borinquense_DSM_11551_uid	NC_014729	chromosome										
313126523	hypothetical protein Hbor_17800	No	0		1709546	1710424	-		Halogeometricum_borinquense_DSM_11551_uid	NC_014729	chromosome			arCOG04640	1 (-)	S		Uncharacterized conserved protein	0.0001	pfam13200	Putative glycosyl hydrolase domain. This
313126524	hypothetical protein Hbor_17810	No	1		1710534	1710638	-		Halogeometricum_borinquense_DSM_11551_uid	NC_014729	chromosome										
===								7	Halogeometricum_borinquense_DSM_11551_uid	NC_014729	chromosome										
313126686	ppox class probable f420-dependent enzyme	No	0		1866487	1866888	-		Halogeometricum_borinquense_DSM_11551_uid	NC_014729	chromosome			arCOG00516	6 (-)	R		Predicted flavin-nucleotide-binding protein	0.000001	pfam01243	Pyridoxamine 5'-phosphate oxidase.
313126687	hypothetical protein Hbor_19450	No	0		1867212	1867511	+		Halogeometricum_borinquense_DSM_11551_uid	NC_014729	chromosome										
313126688	hypothetical protein Hbor_19460	No	0		1867694	1868113	+		Halogeometricum_borinquense_DSM_11551_uid	NC_014729	chromosome										
313126689	hypothetical protein Hbor_19470	No	0		1868113	1868595	+		Halogeometricum_borinquense_DSM_11551_uid	NC_014729	chromosome			arCOG10632	1	S		Uncharacterized conserved protein			
313126690	hypothetical protein Hbor_19480	No	2		1868742	1868960	+		Halogeometricum_borinquense_DSM_11551_uid	NC_014729	chromosome			arCOG11265	1	S		Uncharacterized conserved protein			
313126691	hypothetical protein Hbor_19490	No	3		1868957	1869367	+		Halogeometricum_borinquense_DSM_11551_uid	NC_014729	chromosome			arCOG09311	1 (-)	S		Uncharacterized conserved membrane protein			
313126692	hypothetical protein Hbor_19510	No	0		1870415	1870510	-		Halogeometricum_borinquense_DSM_11551_uid	NC_014729	chromosome										
===								9	Halogeometricum_borinquense_DSM_11551_uid	NC_014729	chromosome										
313126741	hypothetical protein Hbor_20000	No	5		1913537	1914712	+		Halogeometricum_borinquense_DSM_11551_uid	NC_014729	chromosome		arCOG03919,a	arCOG03919	1 (-)	R		ABC-type uncharacterized transport system, perm	4E-25	pfam05403	Plasmodium histidine-rich protein (HRPII ; ABC-type uncharacterized transport syste
313126742	hypothetical protein Hbor_20010	No	0		1914884	1915792	+		Halogeometricum_borinquense_DSM_11551_uid	NC_014729	chromosome										
313126743	hypothetical protein Hbor_20020	No	0		1916030	1916650	-		Halogeometricum_borinquense_DSM_11551_uid	NC_014729	chromosome										
313126744	hypothetical protein Hbor_20030	No	0		1916647	1916868	-		Halogeometricum_borinquense_DSM_11551_uid	NC_014729	chromosome										
313126745	hypothetical protein Hbor_20040	No	0		1916865	1917161	-		Halogeometricum_borinquense_DSM_11551_uid	NC_014729	chromosome										
313126746	hypothetical protein Hbor_20050	No	0		1917161	1917340	-		Halogeometricum_borinquense_DSM_11551_uid	NC_014729	chromosome										
313126747	hypothetical protein Hbor_20060	Yes	0		1917983	1918090	-		Halogeometricum_borinquense_DSM_11551_uid	NC_014729	chromosome										
313126748	site-specific recombinase xerd	No	0		1918530	1919465	-		Halogeometricum_borinquense_DSM_11551_uid	NC_014729	chromosome			arCOG01241	31 (-)	L	XerC	Integrase	5E-31	COG4974	Site-specific recombinase XerD
313126749	hypothetical protein Hbor_20080	No	0		1919458	1919979	-		Halogeometricum_borinquense_DSM_11551_uid	NC_014729	chromosome										
===								18	Halogeometricum_borinquense_DSM_11551_uid	NC_014729	chromosome										
313127612	hypothetical protein Hbor_28980	No	0	integrated_ele	2762479	2762901	-		Halogeometricum_borinquense_DSM_11551_uid	NC_014729	chromosome	clust_244									
313127613	hypothetical protein Hbor_28990	No	0	integrated_ele	2762908	2764287	-		Halogeometricum_borinquense_DSM_11551_uid	NC_014729	chromosome			arCOG10869	2 (-)	S		Uncharacterized conserved protein	4E-16	pfam13699	Domain of unknown function (DUF4157). Th
313127614	hypothetical protein Hbor_29000	No	3	integrated_ele	2764543	2764926	-		Halogeometricum_borinquense_DSM_11551_uid	NC_014729	chromosome	clust_277									
313127615	hypothetical protein Hbor_29010	Yes	3	integrated_ele	2764923	2765282	-		Halogeometricum_borinquense_DSM_11551_uid	NC_014729	chromosome	clust_299									
313127616	hypothetical protein Hbor_29020	Yes	2	integrated_ele	2765279	2765659	-		Halogeometricum_borinquense_DSM_11551_uid	NC_014729	chromosome										
313127617	hypothetical protein Hbor_29030	Yes	3	integrated_ele	2765656	2768751	-		Halogeometricum_borinquense_DSM_11551_uid	NC_014729	chromosome			arCOG06742	1 (-)	S		NosD-like cell surface protein			
313127618	hypothetical protein Hbor_29040	No	2	integrated_ele	2768748	2769041	-		Halogeometricum_borinquense_DSM_11551_uid	NC_014729	chromosome										
313127619	hypothetical protein Hbor_29050	No	2	integrated_ele	2769045	2769341	-		Halogeometricum_borinquense_DSM_11551_uid	NC_014729	chromosome										
313127620	hypothetical protein Hbor_29060	No	0	integrated_ele	2769338	2772046	-		Halogeometricum_borinquense_DSM_11551_uid	NC_014729	chromosome			arCOG08949	1 (-)	S		Uncharacterized conserved protein	0.000002	pfam06682	Protein of unknown function (DUF1183). T
313127621	hypothetical protein Hbor_29070	No	0	integrated_ele	2772123	2772452	+		Halogeometricum_borinquense_DSM_11551_uid	NC_014729	chromosome										
313127622	hypothetical protein Hbor_29080	No	2	integrated_ele	2772524	2773528	-		Halogeometricum_borinquense_DSM_11551_uid	NC_014729	chromosome										
313127623	hypothetical protein Hbor_29090	No	0	integrated_ele	2773604	2774164	-		Halogeometricum_borinquense_DSM_11551_uid	NC_014729	chromosome										
313127624	hypothetical protein Hbor_29100	No	1	integrated_ele	2774167	2774412	-		Halogeometricum_borinquense_DSM_11551_uid	NC_014729	chromosome										
313127625	hypothetical protein Hbor_29110	Yes	2	integrated_ele	2774521	2774997	-		Halogeometricum_borinquense_DSM_11551_uid	NC_014729	chromosome										
313127626	hypothetical protein Hbor_29120	No	2	integrated_ele	2775047	2775625	-		Halogeometricum_borinquense_DSM_11551_uid	NC_014729	chromosome										
313127627	hypothetical protein Hbor_29130	No	0	integrated_ele	2775740	2776012	+		Halogeometricum_borinquense_DSM_11551_uid	NC_014729	chromosome										
313127628	hypothetical protein Hbor_29140	No	0	integrated_ele	2776009	2776632	+		Halogeometricum_borinquense_DSM_11551_uid	NC_014729	chromosome										
313127629	zonular occludens toxin (zot)	No	0	integrated_ele	2776648	2777547	+		Halogeometricum_borinquense_DSM_11551_uid	NC_014729	chromosome			arCOG07960	16 (-)	R		ATPase (AAA+ superfamily)			
===								10	Halogeometricum_borinquense_DSM_11551_uid	NC_014729	chromosome										
313127631	hypothetical protein Hbor_29170	No	0	integrated_ele	2778888	2779073	+		Halogeometricum_borinquense_DSM_11551_uid	NC_014729	chromosome										
313127632	von willebrand factor type a-like protein	No	0	integrated_ele	2779140	2780348	+		Halogeometricum_borinquense_DSM_11551_uid	NC_014729	chromosome			arCOG02900	3 (-)	R		Uncharacterized protein containing a von Willebrand factor type A (vWA) domain			
313127633	hypothetical protein Hbor_29190	No	0	integrated_ele	2780361	2780690	-		Halogeometricum_borinquense_DSM_11551_uid	NC_014729	chromosome										
313127634	hypothetical protein Hbor_29200	No	2	integrated_ele	2780687	2780920	-		Halogeometricum_borinquense_DSM_11551_uid	NC_014729	chromosome										
313127635	hypothetical protein Hbor_29210	No	3	integrated_ele	2780917	2781318	-		Halogeometricum_borinquense_DSM_11551_uid	NC_014729	chromosome										
313127636	hypothetical protein Hbor_29220	No	0	integrated_ele	2782030	2782263	+		Halogeometricum_borinquense_DSM_11551_uid	NC_014729	chromosome										
313127637	phage integrase family protein	No	0	integrated_ele	2782577	2783182	+		Halogeometricum_borinquense_DSM_11551_uid	NC_014729	chromosome			arCOG02282	8 (-)	K		Predicted transcriptional regulator, C-terminal HTH-like domain			
313127638	hypothetical protein Hbor_29240	No	0	integrated_ele	2783502	2783636	+		Halogeometricum_borinquense_DSM_11551_uid	NC_014729	chromosome										
313127639	hypothetical protein Hbor_29250	No	0	integrated_ele	2783867	2784067	-		Halogeometricum_borinquense_DSM_11551_uid	NC_014729	chromosome										
313127640	hypothetical protein Hbor_29260	No	0	integrated_ele	2784064	2784264	-		Halogeometricum_borinquense_DSM_11551_uid	NC_014729	chromosome								0.0001	pfam09954	Uncharacterized protein conserved in bac
===								10	Halogeometricum_borinquense_DSM_11551_uid	NC_014731	plasmid pHBOR02										
313116834	HEAT repeat protein	No	0	plasmid	8868	10904	+		Halogeometricum_borinquense_DSM_11551_uid	NC_014731	plasmid pHBOR02			arCOG02967	3 (-)	T		NACHT family NTPase fused to HEAT repeats do	1E-16	COG1413	FOG: HEAT repeat
313116835	uncharacterized conserved protein (DUF2262)	No	0	plasmid	10919	11077	-		Halogeometricum_borinquense_DSM_11551_uid	NC_014731	plasmid pHBOR02										
313116836	phage integrase family protein	No	0	plasmid	11281	11529	-		Halogeometricum_borinquense_DSM_11551_uid	NC_014731	plasmid pHBOR02										
313116837	DNA/RNA helicase, superfamily I	No	0	plasmid	12538	14343	-		Halogeometricum_borinquense_DSM_11551_uid	NC_014731	plasmid pHBOR02	clust_67							4E-52	COG3972	Superfamily I DNA and RNA helicases

313116838	hypothetical protein Hbor_33000	No	2		plasmid	15873	16073	+		Halogeometricum_borinquense_DSM_11551_uid	NC_014731	plasmid pHBOR02										
313116839	hypothetical protein Hbor_33010	No	0		plasmid	16375	16635	+		Halogeometricum_borinquense_DSM_11551_uid	NC_014731	plasmid pHBOR02	clust_22									
313116840	hypothetical protein Hbor_33020	Yes	0		plasmid	16694	16927	-		Halogeometricum_borinquense_DSM_11551_uid	NC_014731	plasmid pHBOR02										
313116841	transposase	No	0		plasmid	16976	17653	-		Halogeometricum_borinquense_DSM_11551_uid	NC_014731	plasmid pHBOR02			arCOG02134	15 (-)	L		Transposase	2E-14	COG3316	Transposase and inactivated derivatives
313116842	hypothetical protein Hbor_33040	No	0		plasmid	17898	18302	-		Halogeometricum_borinquense_DSM_11551_uid	NC_014731	plasmid pHBOR02	clust_40									
313116843	hypothetical protein Hbor_33050	No	0		plasmid	18556	20139	+		Halogeometricum_borinquense_DSM_11551_uid	NC_014731	plasmid pHBOR02										
===									5	Halogeometricum_borinquense_DSM_11551_uid	NC_014731	plasmid pHBOR02										
313116859	hypothetical protein Hbor_33220	No	0		plasmid	40650	41426	-		Halogeometricum_borinquense_DSM_11551_uid	NC_014731	plasmid pHBOR02	clust_128									
313116860	hypothetical protein Hbor_33230	Yes	1		plasmid	41429	42211	-		Halogeometricum_borinquense_DSM_11551_uid	NC_014731	plasmid pHBOR02	clust_127									
313116861	hypothetical protein Hbor_33240	No	0		plasmid	42638	43429	+		Halogeometricum_borinquense_DSM_11551_uid	NC_014731	plasmid pHBOR02			arCOG02809	5 (-)	S		Uncharacterized conserved protein			
313116862	hypothetical protein Hbor_33250	No	0		plasmid	43770	43886	+		Halogeometricum_borinquense_DSM_11551_uid	NC_014731	plasmid pHBOR02										
313116863	hypothetical protein Hbor_33260	Yes	6		plasmid	43931	44575	-		Halogeometricum_borinquense_DSM_11551_uid	NC_014731	plasmid pHBOR02			arCOG10849	1	S		Uncharacterized conserved protein			
===									6	Halogeometricum_borinquense_DSM_11551_uid	NC_014731	plasmid pHBOR02										
313116949	hypothetical protein Hbor_34150	Yes	1		plasmid	136862	138280	-		Halogeometricum_borinquense_DSM_11551_uid	NC_014731	plasmid pHBOR02								1E-14	pfam04006	Mpp10 protein. This family includes prot ; Nop14-like family. Emg1 and Nop14 are no
313116950	hypothetical protein Hbor_34160	No	0		plasmid	138807	139136	+		Halogeometricum_borinquense_DSM_11551_uid	NC_014731	plasmid pHBOR02										
313116951	Sugar-specific transcriptional regulator TrmB	No	0		plasmid	139311	140105	+		Halogeometricum_borinquense_DSM_11551_uid	NC_014731	plasmid pHBOR02			arCOG02809	5 (-)	S		Uncharacterized conserved protein	8E-11	COG4742	Predicted transcriptional regulator
313116952	hypothetical protein Hbor_34180	No	0		plasmid	140331	141233	+		Halogeometricum_borinquense_DSM_11551_uid	NC_014731	plasmid pHBOR02										
313116953	hypothetical protein Hbor_34190	Yes	1		plasmid	141721	142497	-		Halogeometricum_borinquense_DSM_11551_uid	NC_014731	plasmid pHBOR02								5E-12	pfam04006	Mpp10 protein. This family includes prot
313116954	hypothetical protein Hbor_34200	No	0		plasmid	142579	142830	+		Halogeometricum_borinquense_DSM_11551_uid	NC_014731	plasmid pHBOR02										
===									5	Halogeometricum_borinquense_DSM_11551_uid	NC_014731	plasmid pHBOR02										
313117099	hypothetical protein Hbor_35670	No	0		plasmid	301862	302497	+		Halogeometricum_borinquense_DSM_11551_uid	NC_014731	plasmid pHBOR02										
313117100	hypothetical protein Hbor_35680	No	2		plasmid	302688	302903	+		Halogeometricum_borinquense_DSM_11551_uid	NC_014731	plasmid pHBOR02			arCOG10947	2	S		Uncharacterized conserved protein			
313117101	transposase family protein	No	0		plasmid	304026	305615	+		Halogeometricum_borinquense_DSM_11551_uid	NC_014731	plasmid pHBOR02		arCOG03900,a	arCOG03900	5 (-)	L		Transposase			
313117102	hypothetical protein Hbor_35700	No	2		plasmid	306268	306474	-		Halogeometricum_borinquense_DSM_11551_uid	NC_014731	plasmid pHBOR02										
313117103	hypothetical protein Hbor_35710	No	0		plasmid	306513	306668	-		Halogeometricum_borinquense_DSM_11551_uid	NC_014731	plasmid pHBOR02										
===									17	Halogeometricum_borinquense_DSM_11551_uid	NC_014731	plasmid pHBOR02										
313117116	hypothetical protein Hbor_35840	No	0		plasmid	322833	323165	+		Halogeometricum_borinquense_DSM_11551_uid	NC_014731	plasmid pHBOR02	clust_316									
313117117	nucleotidyltransferase family protein	No	0		plasmid	323432	323830	+		Halogeometricum_borinquense_DSM_11551_uid	NC_014731	plasmid pHBOR02			arCOG02105	3 (-)	V		Minimal nucleotidyltransferase			
313117118	hypothetical protein Hbor_35860	No	3		plasmid	323868	324140	+		Halogeometricum_borinquense_DSM_11551_uid	NC_014731	plasmid pHBOR02										
313117119	hypothetical protein Hbor_35870	No	0		plasmid	324163	324291	+		Halogeometricum_borinquense_DSM_11551_uid	NC_014731	plasmid pHBOR02										
313117120	hypothetical protein Hbor_35880	No	0		plasmid	324301	324888	+		Halogeometricum_borinquense_DSM_11551_uid	NC_014731	plasmid pHBOR02										
313117121	hypothetical protein Hbor_35890	No	0		plasmid	325850	326635	+		Halogeometricum_borinquense_DSM_11551_uid	NC_014731	plasmid pHBOR02			arCOG02809	5 (-)	S		Uncharacterized conserved protein			
313117122	hypothetical protein Hbor_35900	No	0		plasmid	326640	327131	-		Halogeometricum_borinquense_DSM_11551_uid	NC_014731	plasmid pHBOR02										
313117123	hypothetical protein Hbor_35910	No	0		plasmid	327676	328386	+		Halogeometricum_borinquense_DSM_11551_uid	NC_014731	plasmid pHBOR02										
313117124	hypothetical protein Hbor_35940	No	0		plasmid	331324	331881	+		Halogeometricum_borinquense_DSM_11551_uid	NC_014731	plasmid pHBOR02										
313117125	hypothetical protein Hbor_35950	No	0		plasmid	332376	332606	+		Halogeometricum_borinquense_DSM_11551_uid	NC_014731	plasmid pHBOR02										
313117126	hypothetical protein Hbor_35960	No	0		plasmid	332703	333182	-		Halogeometricum_borinquense_DSM_11551_uid	NC_014731	plasmid pHBOR02										
313117127	hypothetical protein Hbor_35970	No	0		plasmid	333408	333530	+		Halogeometricum_borinquense_DSM_11551_uid	NC_014731	plasmid pHBOR02										
313117128	ferrichrome-binding protein	No	0		plasmid	334016	335224	-		Halogeometricum_borinquense_DSM_11551_uid	NC_014731	plasmid pHBOR02			arCOG06180	2 (-)	P	FepB	ABC-type Fe3+-hydroxamate transport system, p	0.000009	COG0614	ABC-type Fe3+-hydroxamate transport syst
313117129	hypothetical protein Hbor_35990	No	0		plasmid	335744	335977	-		Halogeometricum_borinquense_DSM_11551_uid	NC_014731	plasmid pHBOR02										
313117130	hypothetical protein Hbor_36000	No	0		plasmid	336649	337752	+		Halogeometricum_borinquense_DSM_11551_uid	NC_014731	plasmid pHBOR02										
313117131	hypothetical protein Hbor_36010	No	3		plasmid	337792	338076	+		Halogeometricum_borinquense_DSM_11551_uid	NC_014731	plasmid pHBOR02										
313117132	hypothetical protein Hbor_36020	No	2		plasmid	338567	339010	-		Halogeometricum_borinquense_DSM_11551_uid	NC_014731	plasmid pHBOR02			arCOG06379	1	S		Uncharacterized conserved protein			
===									5	Halogeometricum_borinquense_DSM_11551_uid	NC_014732	plasmid pHBOR04										
313122413	hypothetical protein Hbor_36470	No	0		plasmid	45708	45896	-		Halogeometricum_borinquense_DSM_11551_uid	NC_014732	plasmid pHBOR04			arCOG11148	1	S		Uncharacterized conserved protein			
313122414	hypothetical protein Hbor_36480	No	0		plasmid	45991	46593	-		Halogeometricum_borinquense_DSM_11551_uid	NC_014732	plasmid pHBOR04			arCOG01861	3 (-)	H	MptB	Methanopterin biosynthesis cyclic phosphodiesterase, HD-superfamily hydrolase			
313122415	hypothetical protein Hbor_36490	No	0		plasmid	46586	46690	-		Halogeometricum_borinquense_DSM_11551_uid	NC_014732	plasmid pHBOR04										
313122416	hypothetical protein Hbor_36500	No	0		plasmid	46872	47096	-		Halogeometricum_borinquense_DSM_11551_uid	NC_014732	plasmid pHBOR04										
313122417	hypothetical protein Hbor_36510	No	0		plasmid	48399	48626	+		Halogeometricum_borinquense_DSM_11551_uid	NC_014732	plasmid pHBOR04										
===									6	Halogeometricum_borinquense_DSM_11551_uid	NC_014732	plasmid pHBOR04										
313122506	hypothetical protein Hbor_37400	No	0		plasmid	149626	149952	-		Halogeometricum_borinquense_DSM_11551_uid	NC_014732	plasmid pHBOR04										
313122507	hypothetical protein Hbor_37410	No	0		plasmid	149949	150311	-		Halogeometricum_borinquense_DSM_11551_uid	NC_014732	plasmid pHBOR04										
313122508	transposase	No	0		plasmid	150430	151107	+		Halogeometricum_borinquense_DSM_11551_uid	NC_014732	plasmid pHBOR04			arCOG02134	15 (-)	L		Transposase	2E-14	COG3316	Transposase and inactivated derivatives
313122509	hypothetical protein Hbor_37430	No	1		plasmid	151325	151504	+		Halogeometricum_borinquense_DSM_11551_uid	NC_014732	plasmid pHBOR04										
313122510	hypothetical protein Hbor_37440	No	2		plasmid	151524	151745	+		Halogeometricum_borinquense_DSM_11551_uid	NC_014732	plasmid pHBOR04										
313122511	hypothetical protein Hbor_37450	Yes	0		plasmid	151973	152407	-		Halogeometricum_borinquense_DSM_11551_uid	NC_014732	plasmid pHBOR04										
===									9	Halogeometricum_borinquense_DSM_11551_uid	NC_014735	plasmid pHBOR01										
313117187	hypothetical protein Hbor_29700	No	0		plasmid	3008	3394	+		Halogeometricum_borinquense_DSM_11551_uid	NC_014735	plasmid pHBOR01										
313117188	hypothetical protein Hbor_29710	No	0		plasmid	3950	4255	+		Halogeometricum_borinquense_DSM_11551_uid	NC_014735	plasmid pHBOR01			arCOG02134	15 (-)	L		Transposase			
313117189	hypothetical protein Hbor_29720	No	4		plasmid	4595	5041	+		Halogeometricum_borinquense_DSM_11551_uid	NC_014735	plasmid pHBOR01										
313117190	hypothetical protein Hbor_29730	No	0		plasmid	5135	5284	-		Halogeometricum_borinquense_DSM_11551_uid	NC_014735	plasmid pHBOR01										
313117191	hypothetical protein Hbor_29740	No	1		plasmid	5438	5995	+		Halogeometricum_borinquense_DSM_11551_uid	NC_014735	plasmid pHBOR01										
313117192	hypothetical protein Hbor_29750	Yes	1		plasmid	5992	6654	+		Halogeometricum_borinquense_DSM_11551_uid	NC_014735	plasmid pHBOR01										
313117193	hypothetical protein Hbor_29760	No	1		plasmid	6651	7127	+		Halogeometricum_borinquense_DSM_11551_uid	NC_014735	plasmid pHBOR01										
313117194	hypothetical protein Hbor_29770	No	1		plasmid	7105	7401	+		Halogeometricum_borinquense_DSM_11551_uid	NC_014735	plasmid pHBOR01										
313117195	universal stress protein UspA-like protein	No	0		plasmid	7585	8031	+		Halogeometricum_borinquense_DSM_11551_uid	NC_014735	plasmid pHBOR01			arCOG02053	7 (-)	T	UspA	Nucleotide-binding protein, UspA family	2E-20	pfam00582	Universal stress protein family. The uni
===									6	Halogeometricum_borinquense_DSM_11551_uid	NC_014735	plasmid pHBOR01										
313117345	CARDB domain-containing protein	No	0		plasmid	176498	182056	+		Halogeometricum_borinquense_DSM_11551_uid	NC_014735	plasmid pHBOR01		arCOG02532,a	arCOG02532	2 (-)	S		beta-propeller repeat protein fused to CARDB-lik	0.00002	pfam13249	Prenyltransferase-like. ; Putative flagellar system-associated rep
313117346	dockerin-like protein	Yes	1		plasmid	182034	183275	+		Halogeometricum_borinquense_DSM_11551_uid	NC_014735	plasmid pHBOR01										
313117347	hypothetical protein Hbor_31340	Yes	1		plasmid	183289	184149	+		Halogeometricum_borinquense_DSM_11551_uid	NC_014735	plasmid pHBOR01								7E-10	pfam01034	Syndecan domain. Syndecans are transmemb ; Uncharacterized protein conserved in bac
313117348	hypothetical protein Hbor_31350	No	0		plasmid	184429	184665	+		Halogeometricum_borinquense_DSM_11551_uid	NC_014735	plasmid pHBOR01										
313117349	hypothetical protein Hbor_31360	Yes	1		plasmid	184816	185925	-		Halogeometricum_borinquense_DSM_11551_uid	NC_014735	plasmid pHBOR01			arCOG11256	2	S		Uncharacterized conserved protein	0.0000007	pfam01456	Mucin-like glycoprotein. This family of
313117350	hypothetical protein Hbor_31370	Yes	2		plasmid	186234	189053	+		Halogeometricum_borinquense_DSM_11551_uid	NC_014735	plasmid pHBOR01			arCOG06273	2 (-)	S		S-layer protein			
===									8	Halogeometricum_borinquense_DSM_11551_uid	NC_014736	plasmid pHBOR03										
313122586	transposase	No	0		plasmid	44692	46041	-		Halogeometricum_borinquense_DSM_11551_uid	NC_014736	plasmid pHBOR03			arCOG03900	5 (-)	L		Transposase			
313122587	hypothetical protein Hbor_38440	No	0		plasmid	46327	46566	+		Halogeometricum_borinquense_DSM_11551_uid	NC_014736	plasmid pHBOR03										
313122588	hypothetical protein Hbor_38450	No	0		plasmid	46611	46829	-		Halogeometricum_borinquense_DSM_11551_uid	NC_014736	plasmid pHBOR03										
313122589	hypothetical protein Hbor_38460	Yes	1		plasmid	46999	47886	+		Halogeometricum_borinquense_DSM_11551_uid	NC_014736	plasmid pHBOR03										
313122590	hypothetical protein Hbor_38470	Yes	1		plasmid	47966	48490	+		Halogeometricum_borinquense_DSM_11551_uid	NC_014736	plasmid pHBOR03										
313122591	hypothetical protein Hbor_38480	No	3		plasmid	49212	49526	+		Halogeometricum_borinquense_DSM_11551_uid	NC_014736	plasmid pHBOR03										
313122592	nucleoside-diphosphate-sugar pyrophosphory	No	0		plasmid	49750	50457	-		Halogeometricum_borinquense_DSM_11551_uid	NC_014736	plasmid pHBOR03										
313122593	bacterial regulatory protein, arsR family	No	0		plasmid	51264	51446	-		Halogeometricum_borinquense_DSM_11551_uid	NC_014736	plasmid pHBOR03			arCOG03403	5 (-)	V		PemK/MazF family toxin			
===									9	Halogeometricum_borinquense_DSM_11551_uid	NC_014736	plasmid pHBOR03										
313122624	hypothetical protein Hbor_38810	No	0		plasmid	91860	93107	+		Halogeometricum_borinquense_DSM_11551_uid	NC_014736	plasmid pHBOR03			arCOG02320	4 (-)	N	Tar	Methyl-accepting chemotaxis protein	0.000001	COG0419	ATPase involved in DNA repair
313122625	phage tail sheath protein FI	No	0		plasmid	93380	95104	+		Halogeometricum_borinquense_DSM_11551_uid	NC_014736	plasmid pHBOR03	clust_11							2E-30	COG3497	Phage tail sheath protein FI
313122626	cysteine protease	No	0		plasmid	95154	95603	+		Halogeometricum_borinquense_DSM_11551_uid	NC_014736	plasmid pHBOR03	clust_19							6E-12	pfam06841	T4-like virus tail tube protein gp19. Th
313122627	hypothetical protein Hbor_38840	No	0		plasmid	95603	95755	+		Halogeometricum_borinquense_DSM_11551_uid	NC_014736	plasmid pHBOR03										
313122628	hypothetical protein Hbor_38850	No	0		plasmid	95759	96295	+		Halogeometricum_borinquense_DSM_11551_uid	NC_014736	plasmid pHBOR03	clust_16									
313122629	hypothetical protein Hbor_38860	No	0		plasmid	96292	96561	+		Halogeometricum_borinquense_DSM_11551_uid	NC_014736	plasmid pHBOR03	clust_21									
313122630	acetyltransferase, ribosomal protein N-acetylas	No	0		plasmid	96586	97050	+		Halogeometricum_borinquense_DSM_11551_uid	NC_014736	plasmid pHBOR03	clust_6							1E-29	pfam06841	T4-like virus tail tube protein gp19. Th
313122631	hypothetical protein Hbor_38880	No	0		plasmid	97063	97656	-		Halogeometricum_borinquense_DSM_11551_uid	NC_014736	plasmid pHBOR03	clust_170							5E-14	pfam14065	Protein of unknown function (DUF4255). T
313122632	AAA+ family ATPase	No	0		plasmid	97751	99931	-		Halogeometricum_borinquense_DSM_11551_uid	NC_014736	plasmid pHBOR03			arCOG01308	10 (-)	O	Cdc48	ATPase of the AAA+ class , CDC48 family	2E-17	COG0464	ATPases of the AAA+ class
===									6	Halogeometricum_borinquense_DSM_11551_uid	NC_014736	plasmid pHBOR03										
313122653	acyl-CoA dehydrogenase	No	0		plasmid	119884	120342	+		Halogeometricum_borinquense_DSM_11551_uid	NC_014736	plasmid pHBOR03			arCOG02802	1	S		Uncharacterized conserved protein			
313122654	aminoglycoside phosphotransferase	No	4		plasmid	120599	120964	-		Halogeometricum_borinquense_DSM_11551_uid	NC_014736	plasmid pHBOR03										
313122655	ABC-type multidrug transport system, ATPase	No	7		plasmid	121228	122448	+		Halogeometricum_borinquense_DSM_11551_uid	NC_014736	plasmid pHBOR03			arCOG03920	1 (-)	S		Uncharacterized conserved protein			
313122656	hypothetical protein Hbor_39130	No	0		plasmid	122548	122694	+		Halogeometricum_borinquense_DSM_11551_uid	NC_014736	plasmid pHBOR03										
313122657	transposase	No	1		plasmid	122754	122987	+		Halogeometricum_borinquense_DSM_11551_uid	NC_014736	plasmid pHBOR03										
313122658	acyl-CoA dehydrogenase	No	2		plasmid	123234	123449	-		Halogeometricum_borinquense_DSM_11551_uid	NC_014736	plasmid pHBOR03			arCOG10947	2	S		Uncharacterized conserved protein			
===									8	Halogeometricum_borinquense_DSM_11551_uid	NC_014736	plasmid pHBOR03										
313122682	fibronectin type III domain-containing protein	No	0		plasmid	145706	149542	-		Halogeometricum_borinquense_DSM_11551_uid	NC_014736	plasmid pHBOR03			arCOG05978	2 (-)	M		Extracellular protein containing Kelch and FN3 dc	0.000002	pfam00041	Fibronectin type III domain. ; Fibronectin type III domain.
313122683	hypothetical protein Hbor_39400	No	0		plasmid	149543	150562	-		Halogeometricum_borinquense_DSM_11551_uid	NC_014736	plasmid pHBOR03			arCOG10640	5	Y		Phage associated protein			
313122684	hypothetical protein Hbor_39410	No	0		plasmid	150681	151307	-		Halogeometricum_borinquense_DSM_11551_uid	NC_014736	plasmid pHBOR03			arCOG10639	4	Y		Phage associated protein			
313122685	hypothetical protein Hbor_39420	No	2		plasmid	151544	151864	-		Halogeometricum_borinquense_DSM_11551_uid	NC_014736	plasmid pHBOR03										
313122686	ABC-type dipeptide transport system, periplas	No	0		plasmid	151951	152445	-		Halogeometricum_borinquense_DSM_11551_uid	NC_014736	plasmid pHBOR03										
313122687	transcriptional regulator, ArsR family	No	0		plasmid	152600	152854	-		Halogeometricum_borinquense_DSM_11551_uid	NC_014736	plasmid pHBOR03			arCOG00394	7 (-)	K	ArsR	Transcriptional regulator containing HTH domain, ArsR family			
313122688	ABC-type dipeptide/oligopeptide/nickel transp	No	0		plasmid	152851	153177	-		Halogeometricum_borinquense_DSM_11551_uid	NC_014736	plasmid pHBOR03										
313122689	ABC-type dipeptide/oligopeptide/nickel transp	No	0		plasmid	153612	154385	-		Halogeometricum_borinquense_DSM_11551_uid	NC_014736	plasmid pHBOR03								0.0000002	COG1196	Chromosome segregation ATPases
===									6	Halogeometricum_borinquense_DSM_11551_uid	NC_014737	plasmid pHBOR05										
313122741	hypothetical protein Hbor_37850	No	0		plasmid	1660	2463	-		Halogeometricum_borinquense_DSM_11551_uid	NC_014737	plasmid pHBOR05										
313122742	hypothetical protein Hbor_37860	No	0		plasmid	2460	3248	-		Halogeometricum_borinquense_DSM_11551_uid	NC_014737	plasmid pHBOR05										
313122743	hypothetical protein Hbor_37870	No	0		plasmid	3245	4396	-		Halogeometricum_borinquense_DSM_11551_uid	NC_014737	plasmid pHBOR05			arCOG10137	1 (-)	S		Uncharacterized conserved protein	0.0001	pfam13654	AAA domain. This family includes a wide
313122744	hypothetical protein Hbor_37880	No	0		plasmid	4811	5074	+		Halogeometricum_borinquense_DSM_11551_uid	NC_014737	plasmid pHBOR05										
313122745	hypothetical protein Hbor_37890	No	0		plasmid	5071	5313	+		Halogeometricum_borinquense_DSM_11551_uid	NC_014737	plasmid pHBOR05										
313122746	hypothetical protein Hbor_37900	No	0		plasmid	5630	6943	+		Halogeometricum_borinquense_DSM_11551_uid	NC_014737	plasmid pHBOR05								0.0000008	pfam09528	Ehrlichia tandem repeat (Ehrlichia_rpt).
===									6	Halogeometricum_borinquense_DSM_11551_uid	NC_014737	plasmid pHBOR05										
313122757	cytotoxic translational repressor of toxin-antitox	No	0		plasmid	15152	15436	+		Halogeometricum_borinquense_DSM_11551_uid	NC_014737	plasmid pHBOR05			arCOG01664	2 (-)	V	RelE	Cytotoxic translational repressor of toxin-antitoxin stability system			
313122758	hypothetical protein Hbor_38020	No	0		plasmid	15433	15999	-		Halogeometricum_borinquense_DSM_11551_uid	NC_014737	plasmid pHBOR05										
313122759	hypothetical protein Hbor_38030	No	0		plasmid	16166	16594	+		Halogeometricum_borinquense_DSM_11551_uid	NC_014737	plasmid pHBOR05										
313122760	hypothetical protein Hbor_38040	No	0		plasmid	16581	16715	+		Halogeometricum_borinquense_DSM_11551_uid	NC_014737	plasmid pHBOR05										
313122761	hypothetical protein Hbor_38050	No	0		plasmid	16720	16938	+		Halogeometricum_borinquense_DSM_11551_uid	NC_014737	plasmid pHBOR05										
313122762	hypothetical protein Hbor_38060	No	0		plasmid	16986	17246	+		Halogeometricum_borinquense_DSM_11551_uid	NC_014737	plasmid pHBOR05										
===									6	Halomicrobium_mukohataei_DSM_12286_uid591	NC_013201	plasmid pHmuk01										
257372951	hypothetical protein Hmuk_3267	No	0		plasmid	68453	68728	+		Halomicrobium_mukohataei_DSM_12286_uid591	NC_013201	plasmid pHmuk01										
257372952	hypothetical protein Hmuk_3269	No	2		plasmid	69793	70032	-		Halomicrobium_mukohataei_DSM_12286_uid591	NC_013201	plasmid pHmuk01			arCOG11094	1	S		Uncharacterized conserved protein			
257372953	hypothetical protein Hmuk_3270	No	0		plasmid	70266	70940	+		Halomicrobium_mukohataei_DSM_12286_uid591	NC_013201	plasmid pHmuk01								0.000002	COG1196	Chromosome segregation ATPases
257372954	iron dependent repressor	No	0		plasmid	71073	71537	-		Halomicrobium_mukohataei_DSM_12286_uid591	NC_013201	plasmid pHmuk01			arCOG02100	2 (-)	K	TroR	Mn-dependent transcriptional regulator (DtxR fami	0.000000009	COG1321	Mn-dependent transcriptional regulator [
257372955	hypothetical protein Hmuk_3272	No	8		plasmid	71691	72563	+		Halomicrobium_mukohataei_DSM_12286_uid591	NC_013201	plasmid pHmuk01			arCOG11095	1	S		Uncharacterized conserved protein			
257372956	hypothetical protein Hmuk_3273	No	0		plasmid	72615	73496	-		Halomicrobium_mukohataei_DSM_12286_uid591	NC_013201	plasmid pHmuk01										
===									9	Halomicrobium_mukohataei_DSM_12286_uid591	NC_013201	plasmid pHmuk01										
257373024	branched-chain amino acid ABC transporter	No	3	YES	plasmid	159572	159883	-		Halomicrobium_mukohataei_DSM_12286_uid591	NC_013201	plasmid pHmuk01			arCOG06625	1 (-)	S		Uncharacterized membrane protein	3E-17	COG4541	Predicted membrane protein
257373025	hypothetical protein Hmuk_3355	No	2	YES	plasmid	160288	161004	+		Halomicrobium_mukohataei_DSM_12286_uid591	NC_013201	plasmid pHmuk01										
257373026	hypothetical protein Hmuk_3356	No	0	YES	plasmid	161284	161427	-		Halomicrobium_mukohataei_DSM_12286_uid591	NC_013201	plasmid pHmuk01										
257373027	hypothetical protein Hmuk_3357	No	0	YES	plasmid	161453	162103	-		Halomicrobium_mukohataei_DSM_12286_uid591	NC_013201	plasmid pHmuk01										
257373028	hypothetical protein Hmuk_3358	No	0	YES	plasmid	162211	162885	-		Halomicrobium_mukohataei_DSM_12286_uid591	NC_013201	plasmid pHmuk01	clust_154									
257373029	hypothetical protein Hmuk_3359	No	0	YES	plasmid	162925	163068	-		Halomicrobium_mukohataei_DSM_12286_uid591	NC_013201	plasmid pHmuk01										
257373030	hypothetical protein Hmuk_3360	No	0	YES	plasmid	163226	163894	-		Halomicrobium_mukohataei_DSM_12286_uid591	NC_013201	plasmid pHmuk01	clust_154									
257373031	hypothetical protein Hmuk_3361	No	0	YES	plasmid	163896	164558	-		Halomicrobium_mukohataei_DSM_12286_uid591	NC_013201	plasmid pHmuk01										
257373032	hypothetical protein Hmuk_3362	No	0	YES	plasmid	164567	165664	-		Halomicrobium_mukohataei_DSM_12286_uid591	NC_013201	plasmid pHmuk01			arCOG07561	7 (-)	K		Predicted regulatory protein containing HTH domain			
===									25	Halomicrobium_mukohataei_DSM_12286_uid591	NC_013202	chromosome										
257386539	integrase family protein	No	0	YES	Virus_Sphaerc	449776	450435	-		Halomicrobium_mukohataei_DSM_12286_uid591	NC_013202	chromosome			arCOG02282	8 (-)	K		Predicted transcriptional regulator, C-terminal HTH-like domain			
257386540	hypothetical protein Hmuk_0472	Yes	1	YES	Virus_Sphaerc	450792	450989	-		Halomicrobium_mukohataei_DSM_12286_uid591	NC_013202	chromosome			arCOG09036	3	S		Uncharacterized conserved protein			
257386541	hypothetical protein Hmuk_0473	No	1	YES	Virus_Sphaerc	451022	451234	-		Halomicrobium_mukohataei_DSM_12286_uid591	NC_013202	chromosome	clust_438									
257386542	hypothetical protein Hmuk_0474	No	1	YES	Virus_Sphaerc	451234	451677	-		Halomicrobium_mukohataei_DSM_12286_uid591	NC_013202	chromosome	clust_237									
257386543	hypothetical protein Hmuk_0475	No	1	YES	Virus_Sphaerc	451674	452246	-		Halomicrobium_mukohataei_DSM_12286_uid591	NC_013202	chromosome	clust_183									
257386544	hypothetical protein Hmuk_0476	No	0	YES	Virus_Sphaerc	452337	452876	-		Halomicrobium_mukohataei_DSM_12286_uid591	NC_013202	chromosome										
257386545	hypothetical protein Hmuk_0477	No	0	YES	Virus_Sphaerc	452883	453767	-		Halomicrobium_mukohataei_DSM_12286_uid591	NC_013202	chromosome										
257386546	hypothetical protein Hmuk_0478	No	0	YES	Virus_Sphaerc	453770	454225	-		Halomicrobium_mukohataei_DSM_12286_uid591	NC_013202	chromosome	clust_18									
257386547	hypothetical protein Hmuk_0479	No	1	YES	Virus_Sphaerc	454284	454547	-		Halomicrobium_mukohataei_DSM_12286_uid591	NC_013202	chromosome	clust_382									
257386548	hypothetical protein Hmuk_0480	No	0	YES	Virus_Sphaerc	454552	455277	-		Halomicrobium_mukohataei_DSM_12286_uid591	NC_013202	chromosome			arCOG09037	5	Y		VP4/VP17-like Major capsid protein			
257386549	hypothetical protein Hmuk_0481	No	0	YES	Virus_Sphaerc	455282	455764	-		Halomicrobium_mukohataei_DSM_12286_uid591	NC_013202	chromosome			arCOG09038	6	Y		VP7/VP16-like Major capsid protein			
257386550	hypothetical protein Hmuk_0482	No	0	YES	Virus_Sphaerc	455764	456027	-		Halomicrobium_mukohataei_DSM_12286_uid591	NC_013202	chromosome	clust_383									
257386551	hypothetical protein Hmuk_0483	No	0	YES	Virus_Sphaerc	456111	456254	-		Halomicrobium_mukohataei_DSM_12286_uid591	NC_013202	chromosome			arCOG09039	3	S		Uncharacterized conserved protein			
257386552	hypothetical protein Hmuk_0484	No	0	YES	Virus_Sphaerc	456251	457051	-		Halomicrobium_mukohataei_DSM_12286_uid591	NC_013202	chromosome			arCOG00280	21 (-)	L		HerA helicase			
257386553	hypothetical protein Hmuk_0485	No	1	YES	Virus_Sphaerc	457170	457448	-		Halomicrobium_mukohataei_DSM_12286_uid591	NC_013202	chromosome			arCOG10925	3	S		Uncharacterized conserved protein			
257386554	hypothetical protein Hmuk_0486	No	0	YES	Virus_Sphaerc	457445	458020	-		Halomicrobium_mukohataei_DSM_12286_uid591	NC_013202	chromosome			arCOG06228	5	S		Uncharacterized conserved protein			
257386555	hypothetical protein Hmuk_0487	No	0	YES	Virus_Sphaerc	458017	458622	-		Halomicrobium_mukohataei_DSM_12286_uid591	NC_013202	chromosome			arCOG09040	3	S		Uncharacterized conserved protein	0.000001	pfam03344	Daxx Family. The Daxx protein (also know
257386556	hypothetical protein Hmuk_0488	No	0	YES	Virus_Sphaerc	458619	459200	-		Halomicrobium_mukohataei_DSM_12286_uid591	NC_013202	chromosome			arCOG09041	4	S		Uncharacterized conserved protein	0.00001	pfam05432	Bone sialoprotein II (BSP-II). Bone sial
257386557	hypothetical protein Hmuk_0489	No	0	YES	Virus_Sphaerc	459197	460657	-		Halomicrobium_mukohataei_DSM_12286_uid591	NC_013202	chromosome			arCOG09042	7	Y		Viral protein, VP1 family			
257386558	hypothetical protein Hmuk_0490	No	0	YES	Virus_Sphaerc	460759	461385	-		Halomicrobium_mukohataei_DSM_12286_uid591	NC_013202	chromosome										
257386559	hypothetical protein Hmuk_0491	No	0	YES	Virus_Sphaerc	461534	461875	-		Halomicrobium_mukohataei_DSM_12286_uid591	NC_013202	chromosome										
257386560	hypothetical protein Hmuk_0492	No	0	YES	Virus_Sphaerc	461868	461999	-		Halomicrobium_mukohataei_DSM_12286_uid591	NC_013202	chromosome										
257386561	hypothetical protein Hmuk_0493	No	0	YES	Virus_Sphaerc	461996	462397	-		Halomicrobium_mukohataei_DSM_12286_uid591	NC_013202	chromosome			arCOG09043	4	S		Uncharacterized conserved protein			
257386562	hypothetical protein Hmuk_0494	No	0	YES	Virus_Sphaerc	462537	462680	-		Halomicrobium_mukohataei_DSM_12286_uid591	NC_013202	chromosome										
257386563	zinc finger SWIM domain protein	No	0	YES	Virus_Sphaerc	462681	463160	-		Halomicrobium_mukohataei_DSM_12286_uid591	NC_013202	chromosome			arCOG09044	5 (-)	R		SWIM zinc finger			
===									10	Halomicrobium_mukohataei_DSM_12286_uid591	NC_013202	chromosome										
257386868	malate dehydrogenase	No	0			777203	778117	+		Halomicrobium_mukohataei_DSM_12286_uid591	NC_013202	chromosome			arCOG00246	1 (-)	C	Mdh	Malate/lactate dehydrogenase	4E-68	COG0039	Malate/lactate dehydrogenases
257386869	hypothetical protein Hmuk_0804	No	3			778211	778537	-		Halomicrobium_mukohataei_DSM_12286_uid591	NC_013202	chromosome										
257386870	hypothetical protein Hmuk_0805	No	0			778764	778901	-		Halomicrobium_mukohataei_DSM_12286_uid591	NC_013202	chromosome										
257386871	hypothetical protein Hmuk_0806	No	0			778936	779082	+		Halomicrobium_mukohataei_DSM_12286_uid591	NC_013202	chromosome										
257386872	hypothetical protein Hmuk_0807	No	4			779144	779548	-		Halomicrobium_mukohataei_DSM_12286_uid591	NC_013202	chromosome			arCOG09321	3	S		Uncharacterized conserved protein			
257386873	hypothetical protein Hmuk_0808	No	4			779567	779974	-		Halomicrobium_mukohataei_DSM_12286_uid591	NC_013202	chromosome	clust_257									
257386874	hypothetical protein Hmuk_0809	No	4			779991	780386	-		Halomicrobium_mukohataei_DSM_12286_uid591	NC_013202	chromosome	clust_257									
257386875	hypothetical protein Hmuk_0810	No	4			780403	780822	-		Halomicrobium_mukohataei_DSM_12286_uid591	NC_013202	chromosome			arCOG09321	3	S		Uncharacterized conserved protein			
257386876	hypothetical protein Hmuk_0811	Yes	0			780923	781915	-		Halomicrobium_mukohataei_DSM_12286_uid591	NC_013202	chromosome										
257386877	hypothetical protein Hmuk_0812	No	0			782136	782855	-		Halomicrobium_mukohataei_DSM_12286_uid591	NC_013202	chromosome		arCOG04417,a	arCOG04417	1 (-)	R		Zn-ribbon containing protein	2E-11	COG3364	Zn-ribbon containing protein
===									9	Halomicrobium_mukohataei_DSM_12286_uid591	NC_013202	chromosome										
257387012	hypothetical protein Hmuk_0949	No	0			912093	912386	+		Halomicrobium_mukohataei_DSM_12286_uid591	NC_013202	chromosome			arCOG08170	1 (-)	S		Uncharacterized conserved protein			
257387013	hypothetical protein Hmuk_0950	No	0			912903	913052	+		Halomicrobium_mukohataei_DSM_12286_uid591	NC_013202	chromosome										
257387014	hypothetical protein Hmuk_0951	No	0			913049	913378	+		Halomicrobium_mukohataei_DSM_12286_uid591	NC_013202	chromosome										
257387015	hypothetical protein Hmuk_0952	No	0			913426	915189	+		Halomicrobium_mukohataei_DSM_12286_uid591	NC_013202	chromosome								1E-14	pfam07232	Putative rep protein (DUF1424). This fam
257387016	hypothetical protein Hmuk_0953	No	0			915365	916039	+		Halomicrobium_mukohataei_DSM_12286_uid591	NC_013202	chromosome										
257387017	hypothetical protein Hmuk_0954	No	0			917181	917507	+		Halomicrobium_mukohataei_DSM_12286_uid591	NC_013202	chromosome										
257387018	hypothetical protein Hmuk_0955	No	2			917504	917920	+		Halomicrobium_mukohataei_DSM_12286_uid591	NC_013202	chromosome			arCOG08954	3	S		Uncharacterized conserved protein			
257387019	hypothetical protein Hmuk_0956	Yes	2			917925	919769	+		Halomicrobium_mukohataei_DSM_12286_uid591	NC_013202	chromosome			arCOG07782	7 (-)	S		Uncharacterized conserved protein			
257387020	hypothetical protein Hmuk_0957	No	0			919769	919957	+		Halomicrobium_mukohataei_DSM_12286_uid591	NC_013202	chromosome										
===									22	Halomicrobium_mukohataei_DSM_12286_uid591	NC_013202	chromosome										
257387758	AbrB family transcriptional regulator	No	0	YES	Virus_Sphaerc	1635004	1635249	-		Halomicrobium_mukohataei_DSM_12286_uid591	NC_013202	chromosome			arCOG04501	1 (-)	K		SpoVT / AbrB like domain			
257387759	hypothetical protein Hmuk_1711	No	1	YES	Virus_Sphaerc	1635316	1635543	-		Halomicrobium_mukohataei_DSM_12286_uid591	NC_013202	chromosome			arCOG09036	3	S		Uncharacterized conserved protein			
257387760	hypothetical protein Hmuk_1712	Yes	1	YES	Virus_Sphaerc	1635543	1635752	-		Halomicrobium_mukohataei_DSM_12286_uid591	NC_013202	chromosome	clust_438									
257387761	hypothetical protein Hmuk_1713	No	1	YES	Virus_Sphaerc	1635752	1636195	-		Halomicrobium_mukohataei_DSM_12286_uid591	NC_013202	chromosome	clust_237									
257387762	hypothetical protein Hmuk_1714	No	1	YES	Virus_Sphaerc	1636192	1636755	-		Halomicrobium_mukohataei_DSM_12286_uid591	NC_013202	chromosome	clust_183									
257387763	hypothetical protein Hmuk_1715	No	0	YES	Virus_Sphaerc	1636829	1637476	-		Halomicrobium_mukohataei_DSM_12286_uid591	NC_013202	chromosome										
257387764	hypothetical protein Hmuk_1716	No	0	YES	Virus_Sphaerc	1637481	1638371	-		Halomicrobium_mukohataei_DSM_12286_uid591	NC_013202	chromosome										
257387765	hypothetical protein Hmuk_1717	No	0	YES	Virus_Sphaerc	1638373	1638828	-		Halomicrobium_mukohataei_DSM_12286_uid591	NC_013202	chromosome	clust_18									
257387766	hypothetical protein Hmuk_1718	No	1	YES	Virus_Sphaerc	1638860	1639123	-		Halomicrobium_mukohataei_DSM_12286_uid591	NC_013202	chromosome	clust_382									
257387767	hypothetical protein Hmuk_1719	No	0	YES	Virus_Sphaerc	1639128	1639856	-		Halomicrobium_mukohataei_DSM_12286_uid591	NC_013202	chromosome			arCOG09037	5	Y		VP4/VP17-like Major capsid protein			
257387768	hypothetical protein Hmuk_1720	No	0	YES	Virus_Sphaerc	1639861	1640343	-		Halomicrobium_mukohataei_DSM_12286_uid591	NC_013202	chromosome			arCOG09038	6	Y		VP7/VP16-like Major capsid protein			
257387769	hypothetical protein Hmuk_1721	No	0	YES	Virus_Sphaerc	1640343	1640606	-		Halomicrobium_mukohataei_DSM_12286_uid591	NC_013202	chromosome	clust_383									
257387770	hypothetical protein Hmuk_1722	No	0	YES	Virus_Sphaerc	1640699	1640839	-		Halomicrobium_mukohataei_DSM_12286_uid591	NC_013202	chromosome			arCOG09039	3	S		Uncharacterized conserved protein			
257387771	hypothetical protein Hmuk_1723	Yes	0	YES	Virus_Sphaerc	1640836	1641633	-		Halomicrobium_mukohataei_DSM_12286_uid591	NC_013202	chromosome										
257387772	hypothetical protein Hmuk_1724	Yes	0	YES	Virus_Sphaerc	1641700	1641978	-		Halomicrobium_mukohataei_DSM_12286_uid591	NC_013202	chromosome			arCOG10925	3	S		Uncharacterized conserved protein			
257387773	hypothetical protein Hmuk_1725	No	0	YES	Virus_Sphaerc	1641975	1642553	-		Halomicrobium_mukohataei_DSM_12286_uid591	NC_013202	chromosome			arCOG06228	5	S		Uncharacterized conserved protein			
257387774	hypothetical protein Hmuk_1726	No	0	YES	Virus_Sphaerc	1642550	1643122	-		Halomicrobium_mukohataei_DSM_12286_uid591	NC_013202	chromosome			arCOG09040	3	S		Uncharacterized conserved protein	0.000005	pfam04147	Nop14-like family. Emg1 and Nop14 are no
257387775	hypothetical protein Hmuk_1727	No	0	YES	Virus_Sphaerc	1643122	1643703	-		Halomicrobium_mukohataei_DSM_12286_uid591	NC_013202	chromosome			arCOG09041	4	S		Uncharacterized conserved protein	0.0001	pfam09073	BUD22. BUD22 has been shown in yeast to
257387776	hypothetical protein Hmuk_1728	No	0	YES	Virus_Sphaerc	1643700	1644998	-		Halomicrobium_mukohataei_DSM_12286_uid591	NC_013202	chromosome			arCOG09042	7	Y		Viral protein, VP1 family			
257387777	hypothetical protein Hmuk_1729	No	0	YES	Virus_Sphaerc	1645121	1645390	+		Halomicrobium_mukohataei_DSM_12286_uid591	NC_013202	chromosome										
257387778	DNA methyltransferase	No	0	YES	Virus_Sphaerc	1645374	1646093	-		Halomicrobium_mukohataei_DSM_12286_uid591	NC_013202	chromosome			arCOG04157	7 (-)	L	Dcm	Site-specific DNA methylase			
257387779	hypothetical protein Hmuk_1731	No	0	YES	Virus_Sphaerc	1646086	1646553	-		Halomicrobium_mukohataei_DSM_12286_uid591	NC_013202	chromosome										
===									6	Halomicrobium_mukohataei_DSM_12286_uid591	NC_013202	chromosome										
257389158	von Willebrand factor A	No	0			2997104	2998285	-		Halomicrobium_mukohataei_DSM_12286_uid591	NC_013202	chromosome			arCOG02900	3 (-)	R		Uncharacterized protein containing a von Willebra	2E-14	COG2304	Uncharacterized protein containing a von
257389159	hypothetical protein Hmuk_3120	No	2			2999118	2999402	+		Halomicrobium_mukohataei_DSM_12286_uid591	NC_013202	chromosome			arCOG10824	1	S		Uncharacterized conserved protein			
257389160	hypothetical protein Hmuk_3121	No	0			2999399	2999680	+		Halomicrobium_mukohataei_DSM_12286_uid591	NC_013202	chromosome										
257389161	hypothetical protein Hmuk_3122	No	4			2999683	3000129	+		Halomicrobium_mukohataei_DSM_12286_uid591	NC_013202	chromosome										
257389162	hypothetical protein Hmuk_3123	No	2			3000190	3000354	+		Halomicrobium_mukohataei_DSM_12286_uid591	NC_013202	chromosome										
257389163	phosphoadenosine phosphosulfate reductase	No	0			3000389	3001357	-		Halomicrobium_mukohataei_DSM_12286_uid591	NC_013202	chromosome			arCOG04586	1 (-)	E	CysH	3'-phosphoadenosine 5'-phosphosulfate sulfotrar	0.00000002	COG0175	3'-phosphoadenosine 5'-phosphosulfate su
===									11	Halopiger_xanaduensis_SH_6_uid68105	NC_015659	plasmid pHALXA03										
336251463	capsule polysaccharide biosynthesis protein	No	0		plasmid	1285	3015	-		Halopiger_xanaduensis_SH_6_uid68105	NC_015659	plasmid pHALXA03										
336251464	sulfatase	No	0		plasmid	3110	4468	-		Halopiger_xanaduensis_SH_6_uid68105	NC_015659	plasmid pHALXA03			arCOG02785	3 (-)	P	AslA	Arylsulfatase A or related enzyme	6E-22	pfam00884	Sulfatase.
336251465	hypothetical protein Halxa_0211	No	0		plasmid	4566	4877	-		Halopiger_xanaduensis_SH_6_uid68105	NC_015659	plasmid pHALXA03										
336251466	hypothetical protein Halxa_0212	No	0		plasmid	4874	4999	-		Halopiger_xanaduensis_SH_6_uid68105	NC_015659	plasmid pHALXA03										
336251467	hypothetical protein Halxa_0213	No	0		plasmid	5017	5445	-		Halopiger_xanaduensis_SH_6_uid68105	NC_015659	plasmid pHALXA03										
336251468	AAA ATPase	No	0		plasmid	5810	6916	+		Halopiger_xanaduensis_SH_6_uid68105	NC_015659	plasmid pHALXA03			arCOG00467	13 (-)	L	CDC6	Cdc6-related protein, AAA superfamily ATPase	3E-35	COG1474	Cdc6-related protein, AAA superfamily AT
336251469	hypothetical protein Halxa_0215	No	0		plasmid	6913	7170	-		Halopiger_xanaduensis_SH_6_uid68105	NC_015659	plasmid pHALXA03										
336251470	hypothetical protein Halxa_0216	No	0		plasmid	7241	7342	-		Halopiger_xanaduensis_SH_6_uid68105	NC_015659	plasmid pHALXA03										
336251471	hypothetical protein Halxa_0217	No	0		plasmid	7518	7838	-		Halopiger_xanaduensis_SH_6_uid68105	NC_015659	plasmid pHALXA03			arCOG03924	10 (-)	K	MarR	Transcriptional regulator, MarR family			
336251472	hypothetical protein Halxa_0218	No	0		plasmid	8111	8545	+		Halopiger_xanaduensis_SH_6_uid68105	NC_015659	plasmid pHALXA03										
336251473	hypothetical protein Halxa_0219	No	0		plasmid	8896	9495	+		Halopiger_xanaduensis_SH_6_uid68105	NC_015659	plasmid pHALXA03										
===									59	Halopiger_xanaduensis_SH_6_uid68105	NC_015659	plasmid pHALXA03										
336251479	hypothetical protein Halxa_0225	No	0		plasmid	15930	16253	+		Halopiger_xanaduensis_SH_6_uid68105	NC_015659	plasmid pHALXA03			arCOG03065	1 (-)	K	ArsR	Transcriptional regulator containing HTH domain, ArsR family			
336251480	hypothetical protein Halxa_0226	No	0		plasmid	16250	16471	+		Halopiger_xanaduensis_SH_6_uid68105	NC_015659	plasmid pHALXA03										
336251481	hypothetical protein Halxa_0227	No	0		plasmid	16468	17004	+		Halopiger_xanaduensis_SH_6_uid68105	NC_015659	plasmid pHALXA03	clust_193									
336251482	hypothetical protein Halxa_0228	No	0		plasmid	17001	17096	+		Halopiger_xanaduensis_SH_6_uid68105	NC_015659	plasmid pHALXA03										
336251483	hypothetical protein Halxa_0229	No	0		plasmid	17093	17587	+		Halopiger_xanaduensis_SH_6_uid68105	NC_015659	plasmid pHALXA03										
336251484	hypothetical protein Halxa_0230	No	0		plasmid	17580	19271	+		Halopiger_xanaduensis_SH_6_uid68105	NC_015659	plasmid pHALXA03			arCOG06224	4 (-)	L		HerA helicase	4E-15	COG1196	Chromosome segregation ATPases
336251485	hypothetical protein Halxa_0231	No	0		plasmid	19690	20268	+		Halopiger_xanaduensis_SH_6_uid68105	NC_015659	plasmid pHALXA03										
336251486	PadR family transcriptional regulator	No	0		plasmid	20441	20758	+		Halopiger_xanaduensis_SH_6_uid68105	NC_015659	plasmid pHALXA03			arCOG00005	4 (-)	K		Predicted transcriptional regulator, PadR family			
336251487	hypothetical protein Halxa_0233	No	0		plasmid	20758	21027	+		Halopiger_xanaduensis_SH_6_uid68105	NC_015659	plasmid pHALXA03										
336251488	hypothetical protein Halxa_0234	No	0		plasmid	21024	21479	+		Halopiger_xanaduensis_SH_6_uid68105	NC_015659	plasmid pHALXA03										
336251489	hypothetical protein Halxa_0235	No	0		plasmid	21480	21992	+		Halopiger_xanaduensis_SH_6_uid68105	NC_015659	plasmid pHALXA03										
336251490	hypothetical protein Halxa_0236	No	0		plasmid	21989	22144	+		Halopiger_xanaduensis_SH_6_uid68105	NC_015659	plasmid pHALXA03										
336251491	hypothetical protein Halxa_0237	No	0		plasmid	22141	22842	+		Halopiger_xanaduensis_SH_6_uid68105	NC_015659	plasmid pHALXA03										
336251492	hypothetical protein Halxa_0238	No	0		plasmid	22843	23088	+		Halopiger_xanaduensis_SH_6_uid68105	NC_015659	plasmid pHALXA03										
336251493	hypothetical protein Halxa_0239	No	0		plasmid	23081	23296	+		Halopiger_xanaduensis_SH_6_uid68105	NC_015659	plasmid pHALXA03										
336251494	hypothetical protein Halxa_0240	No	0		plasmid	23286	23573	+		Halopiger_xanaduensis_SH_6_uid68105	NC_015659	plasmid pHALXA03										
336251495	hypothetical protein Halxa_0241	No	0		plasmid	23570	24094	+		Halopiger_xanaduensis_SH_6_uid68105	NC_015659	plasmid pHALXA03										
336251496	hypothetical protein Halxa_0242	No	2		plasmid	24084	24509	+		Halopiger_xanaduensis_SH_6_uid68105	NC_015659	plasmid pHALXA03										
336251497	hypothetical protein Halxa_0243	No	0		plasmid	24496	24636	+		Halopiger_xanaduensis_SH_6_uid68105	NC_015659	plasmid pHALXA03										
336251498	chromosome partitioning-like ATPase	No	0		plasmid	25029	25868	+		Halopiger_xanaduensis_SH_6_uid68105	NC_015659	plasmid pHALXA03			arCOG00586	8 (-)	D	Soj	ATPase involved in chromosome partitioning, Pa	6E-16	COG1192	ATPases involved in chromosome partition
336251499	hypothetical protein Halxa_0245	No	0		plasmid	25872	26318	+		Halopiger_xanaduensis_SH_6_uid68105	NC_015659	plasmid pHALXA03			arCOG06156	1 (-)	S		Uncharacterized conserved protein	0.0001	pfam09444	MRC1-like domain. This putative domain i
336251500	hypothetical protein Halxa_0246	No	0		plasmid	26493	27482	-		Halopiger_xanaduensis_SH_6_uid68105	NC_015659	plasmid pHALXA03										
336251501	hypothetical protein Halxa_0247	No	0		plasmid	27475	28074	-		Halopiger_xanaduensis_SH_6_uid68105	NC_015659	plasmid pHALXA03										
336251502	hypothetical protein Halxa_0248	No	0		plasmid	28071	28241	-		Halopiger_xanaduensis_SH_6_uid68105	NC_015659	plasmid pHALXA03	clust_474									
336251503	hypothetical protein Halxa_0249	No	2		plasmid	28234	28503	-		Halopiger_xanaduensis_SH_6_uid68105	NC_015659	plasmid pHALXA03	clust_375									
336251504	hypothetical protein Halxa_0250	No	0		plasmid	28493	28699	-		Halopiger_xanaduensis_SH_6_uid68105	NC_015659	plasmid pHALXA03										
336251505	CopG-like domain-containing protein DNA-bind	No	0		plasmid	28692	28922	-		Halopiger_xanaduensis_SH_6_uid68105	NC_015659	plasmid pHALXA03			arCOG01009	8 (-)	V		Ribbon-helix-helix protein, copG family	0.0000004	COG3609	Predicted transcriptional regulators con
336251506	hypothetical protein Halxa_0252	No	0		plasmid	29173	29451	-		Halopiger_xanaduensis_SH_6_uid68105	NC_015659	plasmid pHALXA03										
336251507	hypothetical protein Halxa_0253	No	0		plasmid	29454	29690	-		Halopiger_xanaduensis_SH_6_uid68105	NC_015659	plasmid pHALXA03	clust_392									
336251508	hypothetical protein Halxa_0254	No	0		plasmid	29687	29932	-		Halopiger_xanaduensis_SH_6_uid68105	NC_015659	plasmid pHALXA03										
336251509	hypothetical protein Halxa_0255	No	0		plasmid	29922	30155	-		Halopiger_xanaduensis_SH_6_uid68105	NC_015659	plasmid pHALXA03										
336251510	hypothetical protein Halxa_0256	No	0		plasmid	30193	30609	-		Halopiger_xanaduensis_SH_6_uid68105	NC_015659	plasmid pHALXA03										
336251511	hypothetical protein Halxa_0257	No	0		plasmid	30602	31042	-		Halopiger_xanaduensis_SH_6_uid68105	NC_015659	plasmid pHALXA03			arCOG02105	3 (-)	V		Minimal nucleotidyltransferase			
336251512	hypothetical protein Halxa_0258	No	0		plasmid	31032	31247	-		Halopiger_xanaduensis_SH_6_uid68105	NC_015659	plasmid pHALXA03										
336251513	hypothetical protein Halxa_0259	No	0		plasmid	31237	31536	-		Halopiger_xanaduensis_SH_6_uid68105	NC_015659	plasmid pHALXA03										
336251514	hypothetical protein Halxa_0260	No	0		plasmid	31747	32406	+		Halopiger_xanaduensis_SH_6_uid68105	NC_015659	plasmid pHALXA03			arCOG00328	8 (-)	L	PolB	DNA polymerase elongation subunit (family B)			
336251515	hypothetical protein Halxa_0261	No	0		plasmid	32461	32742	+		Halopiger_xanaduensis_SH_6_uid68105	NC_015659	plasmid pHALXA03	clust_369									
336251516	hypothetical protein Halxa_0262	No	0		plasmid	32802	33149	+		Halopiger_xanaduensis_SH_6_uid68105	NC_015659	plasmid pHALXA03										
336251517	hypothetical protein Halxa_0263	No	0		plasmid	33189	33365	+		Halopiger_xanaduensis_SH_6_uid68105	NC_015659	plasmid pHALXA03			arCOG11163	4	S		Uncharacterized conserved protein			
336251518	hypothetical protein Halxa_0264	No	1		plasmid	33375	33521	-		Halopiger_xanaduensis_SH_6_uid68105	NC_015659	plasmid pHALXA03										
336251519	hypothetical protein Halxa_0265	No	1		plasmid	33518	33808	-		Halopiger_xanaduensis_SH_6_uid68105	NC_015659	plasmid pHALXA03										
336251520	hypothetical protein Halxa_0266	No	2		plasmid	33805	34611	-		Halopiger_xanaduensis_SH_6_uid68105	NC_015659	plasmid pHALXA03										
336251521	hypothetical protein Halxa_0267	No	0		plasmid	34608	34847	-		Halopiger_xanaduensis_SH_6_uid68105	NC_015659	plasmid pHALXA03										
336251522	hypothetical protein Halxa_0268	No	0		plasmid	34844	35365	-		Halopiger_xanaduensis_SH_6_uid68105	NC_015659	plasmid pHALXA03			arCOG09408	1	S		Uncharacterized conserved protein	4E-43	pfam07799	Protein of unknown function (DUF1643). T
336251523	hypothetical protein Halxa_0269	No	0		plasmid	35362	35742	-		Halopiger_xanaduensis_SH_6_uid68105	NC_015659	plasmid pHALXA03								9E-11	pfam14216	Domain of unknown function (DUF4326). Th
336251524	hypothetical protein Halxa_0270	No	0		plasmid	35735	36184	-		Halopiger_xanaduensis_SH_6_uid68105	NC_015659	plasmid pHALXA03										
336251525	hypothetical protein Halxa_0271	No	0		plasmid	36293	36604	-		Halopiger_xanaduensis_SH_6_uid68105	NC_015659	plasmid pHALXA03										
336251526	hypothetical protein Halxa_0272	No	0		plasmid	36952	37620	+		Halopiger_xanaduensis_SH_6_uid68105	NC_015659	plasmid pHALXA03			arCOG00790	6 (-)	V	Cas4	CRISPR-associated protein Cas4, RecB family exonuclease			
336251527	hypothetical protein Halxa_0273	No	0		plasmid	37765	38121	+		Halopiger_xanaduensis_SH_6_uid68105	NC_015659	plasmid pHALXA03										
336251528	hypothetical protein Halxa_0274	No	0		plasmid	38206	38715	+		Halopiger_xanaduensis_SH_6_uid68105	NC_015659	plasmid pHALXA03			arCOG03726	4 (-)	S		Uncharacterized conserved protein			
336251529	hypothetical protein Halxa_0275	No	0		plasmid	38719	39426	+		Halopiger_xanaduensis_SH_6_uid68105	NC_015659	plasmid pHALXA03										
336251530	hypothetical protein Halxa_0276	No	0		plasmid	39414	39791	+		Halopiger_xanaduensis_SH_6_uid68105	NC_015659	plasmid pHALXA03										
336251531	hypothetical protein Halxa_0277	No	0		plasmid	39788	40495	+		Halopiger_xanaduensis_SH_6_uid68105	NC_015659	plasmid pHALXA03										
336251532	hypothetical protein Halxa_0278	No	0		plasmid	40492	41265	+		Halopiger_xanaduensis_SH_6_uid68105	NC_015659	plasmid pHALXA03										
336251533	hypothetical protein Halxa_0279	No	2		plasmid	41327	41509	+		Halopiger_xanaduensis_SH_6_uid68105	NC_015659	plasmid pHALXA03										
336251534	hypothetical protein Halxa_0280	No	0		plasmid	41909	42082	+		Halopiger_xanaduensis_SH_6_uid68105	NC_015659	plasmid pHALXA03										
336251535	integrase family protein	No	0		plasmid	42057	43298	+		Halopiger_xanaduensis_SH_6_uid68105	NC_015659	plasmid pHALXA03			arCOG08889	1 (-)	L		XerD/XerC family integrase			
336251536	hypothetical protein Halxa_0282	No	0		plasmid	43295	43744	-		Halopiger_xanaduensis_SH_6_uid68105	NC_015659	plasmid pHALXA03										
336251537	hypothetical protein Halxa_0283	No	2		plasmid	43864	44124	-		Halopiger_xanaduensis_SH_6_uid68105	NC_015659	plasmid pHALXA03										
===									19	Halopiger_xanaduensis_SH_6_uid68105	NC_015659	plasmid pHALXA03										
336251541	hypothetical protein Halxa_0287	No	0		plasmid	47329	49692	-		Halopiger_xanaduensis_SH_6_uid68105	NC_015659	plasmid pHALXA03			arCOG00280	21 (-)	L		HerA helicase			
336251542	hypothetical protein Halxa_0288	No	5		plasmid	49714	51330	-		Halopiger_xanaduensis_SH_6_uid68105	NC_015659	plasmid pHALXA03	clust_72									
336251543	hypothetical protein Halxa_0290	No	3		plasmid	52586	53617	-		Halopiger_xanaduensis_SH_6_uid68105	NC_015659	plasmid pHALXA03	clust_94									
336251544	hypothetical protein Halxa_0291	No	0		plasmid	53614	54198	-		Halopiger_xanaduensis_SH_6_uid68105	NC_015659	plasmid pHALXA03	clust_179									
336251545	hypothetical protein Halxa_0292	No	0		plasmid	54195	54410	-		Halopiger_xanaduensis_SH_6_uid68105	NC_015659	plasmid pHALXA03	clust_434									
336251546	hypothetical protein Halxa_0293	No	3		plasmid	54571	54879	-		Halopiger_xanaduensis_SH_6_uid68105	NC_015659	plasmid pHALXA03	clust_346									
336251547	hypothetical protein Halxa_0294	No	1		plasmid	54876	55115	-		Halopiger_xanaduensis_SH_6_uid68105	NC_015659	plasmid pHALXA03	clust_404									
336251548	hypothetical protein Halxa_0295	Yes	1		plasmid	55112	56053	-		Halopiger_xanaduensis_SH_6_uid68105	NC_015659	plasmid pHALXA03	clust_98									
336251549	hypothetical protein Halxa_0296	No	1		plasmid	56056	56508	-		Halopiger_xanaduensis_SH_6_uid68105	NC_015659	plasmid pHALXA03	clust_234									
336251550	hypothetical protein Halxa_0297	Yes	0		plasmid	56718	57419	-		Halopiger_xanaduensis_SH_6_uid68105	NC_015659	plasmid pHALXA03	clust_143									
336251551	hypothetical protein Halxa_0298	No	0		plasmid	57609	57998	-		Halopiger_xanaduensis_SH_6_uid68105	NC_015659	plasmid pHALXA03										
336251552	hypothetical protein Halxa_0299	Yes	1		plasmid	58100	59122	-		Halopiger_xanaduensis_SH_6_uid68105	NC_015659	plasmid pHALXA03										
336251553	hypothetical protein Halxa_0300	No	0		plasmid	59282	60325	-		Halopiger_xanaduensis_SH_6_uid68105	NC_015659	plasmid pHALXA03			arCOG10338	2 (-)	S		Uncharacterized conserved protein			
336251554	hypothetical protein Halxa_0301	No	0		plasmid	60397	61155	-		Halopiger_xanaduensis_SH_6_uid68105	NC_015659	plasmid pHALXA03			arCOG10931	2	S		Uncharacterized conserved protein			
336251555	hypothetical protein Halxa_0302	No	1		plasmid	61160	62224	-		Halopiger_xanaduensis_SH_6_uid68105	NC_015659	plasmid pHALXA03	clust_89									
336251556	hypothetical protein Halxa_0303	No	0		plasmid	62238	63056	-		Halopiger_xanaduensis_SH_6_uid68105	NC_015659	plasmid pHALXA03	clust_119									
336251557	peptidase S8 and S53 subtilisin kexin sedolisi	No	0		plasmid	63059	64627	-		Halopiger_xanaduensis_SH_6_uid68105	NC_015659	plasmid pHALXA03			arCOG00702	6 (-)	O	AprE	Subtilisin-like serine protease	2E-15	COG1404	Subtilisin-like serine proteases
336251558	hypothetical protein Halxa_0305	Yes	2		plasmid	64638	67985	-		Halopiger_xanaduensis_SH_6_uid68105	NC_015659	plasmid pHALXA03	clust_64									
336251559	hypothetical protein Halxa_0306	No	0		plasmid	67999	68658	-		Halopiger_xanaduensis_SH_6_uid68105	NC_015659	plasmid pHALXA03										
===									19	Halopiger_xanaduensis_SH_6_uid68105	NC_015666	chromosome										
336252356	hypothetical protein Halxa_0948	No	0			264846	265433	+		Halopiger_xanaduensis_SH_6_uid68105	NC_015666	chromosome										
336252357	integrase family protein	No	0			265426	266463	+		Halopiger_xanaduensis_SH_6_uid68105	NC_015666	chromosome			arCOG01241	31 (-)	L	XerC	Integrase	7E-23	COG4974	Site-specific recombinase XerD
336252358	hypothetical protein Halxa_0950	No	0			266534	266989	+		Halopiger_xanaduensis_SH_6_uid68105	NC_015666	chromosome										
336252359	hypothetical protein Halxa_0951	No	0			266992	267237	+		Halopiger_xanaduensis_SH_6_uid68105	NC_015666	chromosome										
336252360	hypothetical protein Halxa_0952	No	0			267244	267564	-		Halopiger_xanaduensis_SH_6_uid68105	NC_015666	chromosome										
336252361	hypothetical protein Halxa_0953	No	0			267703	267981	-		Halopiger_xanaduensis_SH_6_uid68105	NC_015666	chromosome										
336252362	hypothetical protein Halxa_0954	No	0			267978	268307	-		Halopiger_xanaduensis_SH_6_uid68105	NC_015666	chromosome										
336252363	hypothetical protein Halxa_0955	No	0			268385	268681	-		Halopiger_xanaduensis_SH_6_uid68105	NC_015666	chromosome										
336252364	hypothetical protein Halxa_0956	No	0			268678	269067	-		Halopiger_xanaduensis_SH_6_uid68105	NC_015666	chromosome										
336252365	hypothetical protein Halxa_0957	No	0			269199	269471	-		Halopiger_xanaduensis_SH_6_uid68105	NC_015666	chromosome										
336252366	hypothetical protein Halxa_0958	No	0			269464	269916	-		Halopiger_xanaduensis_SH_6_uid68105	NC_015666	chromosome										
336252367	hypothetical protein Halxa_0959	No	0			270125	271006	-		Halopiger_xanaduensis_SH_6_uid68105	NC_015666	chromosome								4E-32	pfam14022	Protein of unknown function (DUF4238). T
336252368	hypothetical protein Halxa_0960	No	0			271086	271409	-		Halopiger_xanaduensis_SH_6_uid68105	NC_015666	chromosome										
336252369	Resolvase domain-containing protein	No	0			271627	272355	+		Halopiger_xanaduensis_SH_6_uid68105	NC_015666	chromosome			arCOG03162	12 (-)	L	PinR	Site-specific recombinase, DNA invertase Pin ho	1E-15	COG1961	Site-specific recombinases, DNA invertas
336252370	hypothetical protein Halxa_0962	No	0			272446	272676	-		Halopiger_xanaduensis_SH_6_uid68105	NC_015666	chromosome										
336252371	hypothetical protein Halxa_0963	No	0			272986	273297	-		Halopiger_xanaduensis_SH_6_uid68105	NC_015666	chromosome										
336252372	hypothetical protein Halxa_0964	No	0			273459	273656	-		Halopiger_xanaduensis_SH_6_uid68105	NC_015666	chromosome										
336252373	hypothetical protein Halxa_0965	No	0			273702	273863	+		Halopiger_xanaduensis_SH_6_uid68105	NC_015666	chromosome										
336252374	ParB domain-containing protein nuclease	No	0			273882	275291	-		Halopiger_xanaduensis_SH_6_uid68105	NC_015666	chromosome		arCOG00047,a	arCOG00047	1 (-)	L		Predicted DNA modification methylase	0.00000001	COG1475	Stage 0 sporulation protein J (antagonis
===									7	Halopiger_xanaduensis_SH_6_uid68105	NC_015666	chromosome										
336252395	hypothetical protein Halxa_0987	No	0			307451	308089	+		Halopiger_xanaduensis_SH_6_uid68105	NC_015666	chromosome			arCOG02804	1 (-)	S		Uncharacterized conserved protein			
336252396	hypothetical protein Halxa_0988	No	0			308299	309126	+		Halopiger_xanaduensis_SH_6_uid68105	NC_015666	chromosome										
336252397	hypothetical protein Halxa_0989	No	0			309691	309909	+		Halopiger_xanaduensis_SH_6_uid68105	NC_015666	chromosome										
336252398	hypothetical protein Halxa_0990	No	0			309909	310070	+		Halopiger_xanaduensis_SH_6_uid68105	NC_015666	chromosome										
336252399	hypothetical protein Halxa_0991	No	0			310118	310282	-		Halopiger_xanaduensis_SH_6_uid68105	NC_015666	chromosome										
336252400	hypothetical protein Halxa_0992	No	0			310533	311501	+		Halopiger_xanaduensis_SH_6_uid68105	NC_015666	chromosome			arCOG02773	1 (-)	K		HTH-domain containing transcriptional regulator			

336252401	hypothetical protein Halxa_0993	No	0			311829	312563	-		Halopiger_xanaduensis_SH_6_uid68105	NC_015666	chromosome							0.00000002	COG1196	Chromosome segregation ATPases
===									5	Halopiger_xanaduensis_SH_6_uid68105	NC_015666	chromosome									
336252404	hypothetical protein Halxa_0996	No	0			318962	319681	+		Halopiger_xanaduensis_SH_6_uid68105	NC_015666	chromosome							3E-11	COG1196	Chromosome segregation ATPases
336252405	hypothetical protein Halxa_0997	No	0			320008	321447	+		Halopiger_xanaduensis_SH_6_uid68105	NC_015666	chromosome		arCOG03924	10 (-)	K	MarR	Transcriptional regulator, MarR family			
336252406	hypothetical protein Halxa_0998	No	0			321551	321787	+		Halopiger_xanaduensis_SH_6_uid68105	NC_015666	chromosome									
336252407	hypothetical protein Halxa_0999	No	0			321848	322876	+		Halopiger_xanaduensis_SH_6_uid68105	NC_015666	chromosome									
336252408	hypothetical protein Halxa_1000	No	0			323185	324852	-		Halopiger_xanaduensis_SH_6_uid68105	NC_015666	chromosome									
===									7	Halopiger_xanaduensis_SH_6_uid68105	NC_015666	chromosome									
336252411	hypothetical protein Halxa_1003	No	0			327717	328025	+		Halopiger_xanaduensis_SH_6_uid68105	NC_015666	chromosome									
336252412	metallophosphoesterase	No	0			328027	328935	+		Halopiger_xanaduensis_SH_6_uid68105	NC_015666	chromosome		arCOG01141	2 (-)	R		Phosphoesterase			
336252413	hypothetical protein Halxa_1005	No	3			328970	329314	-		Halopiger_xanaduensis_SH_6_uid68105	NC_015666	chromosome									
336252414	hypothetical protein Halxa_1006	No	0			329462	329725	-		Halopiger_xanaduensis_SH_6_uid68105	NC_015666	chromosome									
336252415	hypothetical protein Halxa_1007	No	0			330076	330420	+		Halopiger_xanaduensis_SH_6_uid68105	NC_015666	chromosome									
336252416	hypothetical protein Halxa_1008	No	0			330413	330745	+		Halopiger_xanaduensis_SH_6_uid68105	NC_015666	chromosome									
336252417	preprotein translocase subunit SecY	No	8			330862	332325	-		Halopiger_xanaduensis_SH_6_uid68105	NC_015666	chromosome		arCOG04169	3 (-)	U	SecY	Preprotein translocase subunit SecY	1E-48	COG0201	Preprotein translocase subunit SecY
===									5	Halopiger_xanaduensis_SH_6_uid68105	NC_015666	chromosome									
336252476	hypothetical protein Halxa_1069	Yes	1			378757	379527	-		Halopiger_xanaduensis_SH_6_uid68105	NC_015666	chromosome	clust_130						0.00008	pfam10446	Protein of unknown function (DUF2457). T
336252477	hypothetical protein Halxa_1071	Yes	1			380441	381085	-		Halopiger_xanaduensis_SH_6_uid68105	NC_015666	chromosome	clust_130						0.0001	pfam13332	Haemagluttinin repeat.
336252478	hypothetical protein Halxa_1072	No	0			381840	383321	+		Halopiger_xanaduensis_SH_6_uid68105	NC_015666	chromosome		arCOG10751	2 (-)	S		Uncharacterized conserved protein			
336252479	hypothetical protein Halxa_1073	Yes	1			383570	383917	-		Halopiger_xanaduensis_SH_6_uid68105	NC_015666	chromosome									
336252480	hypothetical protein Halxa_1074	Yes	0			384284	384904	+		Halopiger_xanaduensis_SH_6_uid68105	NC_015666	chromosome									
===									7	Halopiger_xanaduensis_SH_6_uid68105	NC_015666	chromosome									
336252855	phosphoglucomutase/phosphomannomutase	No	0			758267	759667	+		Halopiger_xanaduensis_SH_6_uid68105	NC_015666	chromosome		arCOG00767	3 (-)	G	{ManB}	Phosphomannomutase	4E-72	COG1109	Phosphomannomutase
336252856	hypothetical protein Halxa_1451	No	0			759681	761504	+		Halopiger_xanaduensis_SH_6_uid68105	NC_015666	chromosome		arCOG10906	5	S		Uncharacterized conserved protein			
336252857	hypothetical protein Halxa_1452	No	0			761567	762004	-		Halopiger_xanaduensis_SH_6_uid68105	NC_015666	chromosome									
336252858	hypothetical protein Halxa_1453	No	0			762079	762774	+		Halopiger_xanaduensis_SH_6_uid68105	NC_015666	chromosome									
336252859	hypothetical protein Halxa_1454	No	0			762971	763483	+		Halopiger_xanaduensis_SH_6_uid68105	NC_015666	chromosome									
336252860	hypothetical protein Halxa_1455	No	0			763707	764243	+		Halopiger_xanaduensis_SH_6_uid68105	NC_015666	chromosome									
336252861	hypothetical protein Halxa_1456	No	0			764801	765172	+		Halopiger_xanaduensis_SH_6_uid68105	NC_015666	chromosome		arCOG10793	1 (-)	S		Uncharacterized conserved protein			
===									6	Halopiger_xanaduensis_SH_6_uid68105	NC_015666	chromosome									
336253712	aryl-alcohol dehydrogenase	No	0			1630578	1631585	-		Halopiger_xanaduensis_SH_6_uid68105	NC_015666	chromosome		arCOG01617	4 (-)	C	Tas	Predicted oxidoreductase (related to aryl-alcohol	2E-68	COG0667	Predicted oxidoreductases (related to ar
336253713	hypothetical protein Halxa_2316	No	0			1631687	1631815	-		Halopiger_xanaduensis_SH_6_uid68105	NC_015666	chromosome									
336253714	hypothetical protein Halxa_2317	No	0			1631981	1632103	-		Halopiger_xanaduensis_SH_6_uid68105	NC_015666	chromosome									
336253715	hypothetical protein Halxa_2318	Yes	1			1632094	1632630	-		Halopiger_xanaduensis_SH_6_uid68105	NC_015666	chromosome									
336253716	hypothetical protein Halxa_2319	No	0			1632644	1633072	-		Halopiger_xanaduensis_SH_6_uid68105	NC_015666	chromosome									
336253717	family 2 glycosyl transferase	No	0			1634202	1635026	+		Halopiger_xanaduensis_SH_6_uid68105	NC_015666	chromosome		arCOG01381	4 (-)	M	WcaA	Glycosyltransferase	7E-13	COG1215	Glycosyltransferases, probably involved
===									8	Halopiger_xanaduensis_SH_6_uid68105	NC_015666	chromosome									
336254296	hypothetical protein Halxa_2908	No	0			2232676	2232957	-		Halopiger_xanaduensis_SH_6_uid68105	NC_015666	chromosome									
336254297	hypothetical protein Halxa_2909	No	0			2233667	2235952	+		Halopiger_xanaduensis_SH_6_uid68105	NC_015666	chromosome		arCOG01165	1 (-)	L		DNA topoisomerase VI, subunit B	1E-11	pfam04147	Nop14-like family. Emg1 and Nop14 are no ; Histidine kinase-, DNA gyrase B-, and HS
336254298	hypothetical protein Halxa_2910	No	0			2236402	2236683	+		Halopiger_xanaduensis_SH_6_uid68105	NC_015666	chromosome									
336254299	hypothetical protein Halxa_2911	No	0			2236676	2237008	+		Halopiger_xanaduensis_SH_6_uid68105	NC_015666	chromosome		arCOG09201	3	S		Uncharacterized conserved protein			
336254300	hypothetical protein Halxa_2912	No	0			2237005	2237172	+		Halopiger_xanaduensis_SH_6_uid68105	NC_015666	chromosome									
336254301	hypothetical protein Halxa_2913	No	0			2237186	2237356	+		Halopiger_xanaduensis_SH_6_uid68105	NC_015666	chromosome									
336254302	bacterio-opsin activator-like protein	No	0			2237364	2237984	-		Halopiger_xanaduensis_SH_6_uid68105	NC_015666	chromosome		arCOG02282	8 (-)	K		Predicted transcriptional regulator, C-terminal HTH-like domain			
336254303	hypothetical protein Halxa_2915	No	0			2238447	2239997	+		Halopiger_xanaduensis_SH_6_uid68105	NC_015666	chromosome									
===									9	Halopiger_xanaduensis_SH_6_uid68105	NC_015666	chromosome									
336255147	oligopeptide/dipeptide ABC transporter ATPas	No	0			3097144	3098304	+		Halopiger_xanaduensis_SH_6_uid68105	NC_015666	chromosome		arCOG00184	2 (-)	E	AppF	ABC-type oligopeptide transport system, ATPase	6E-65	COG4608	ABC-type oligopeptide transport system,
336255148	hypothetical protein Halxa_3770	Yes	1			3098454	3098618	-		Halopiger_xanaduensis_SH_6_uid68105	NC_015666	chromosome		arCOG11096	1	S		Uncharacterized conserved protein			
336255149	hypothetical protein Halxa_3771	No	2			3098836	3099084	+		Halopiger_xanaduensis_SH_6_uid68105	NC_015666	chromosome									
336255150	alpha-glucuronidase	No	0			3099191	3101266	-		Halopiger_xanaduensis_SH_6_uid68105	NC_015666	chromosome								COG3661	Alpha-glucuronidase
336255151	glycoside hydrolase family 10	No	1			3101457	3103118	+		Halopiger_xanaduensis_SH_6_uid68105	NC_015666	chromosome		arCOG10668	2	S		Uncharacterized conserved protein	2E-19	pfam00331	Glycosyl hydrolase family 10.
336255152	endo-1,4-beta-xylanase	No	0			3103131	3104105	-		Halopiger_xanaduensis_SH_6_uid68105	NC_015666	chromosome		arCOG10668	2	S		Uncharacterized conserved protein	5E-81	pfam00331	Glycosyl hydrolase family 10.
336255153	hypothetical protein Halxa_3775	No	2			3104252	3104494	+		Halopiger_xanaduensis_SH_6_uid68105	NC_015666	chromosome									
336255154	5,10-methylenetetrahydromethanopterin reduct	No	0			3104520	3105602	-		Halopiger_xanaduensis_SH_6_uid68105	NC_015666	chromosome		arCOG02410	2 (-)	C		Coenzyme F420-dependent N5,N10-methylene te	5E-33	COG2141	Coenzyme F420-dependent N5,N10-methylene
336255155	Uronate isomerase	No	0			3105696	3107060	+		Halopiger_xanaduensis_SH_6_uid68105	NC_015666	chromosome		arCOG08625	1	S		Uncharacterized conserved protein	2E-118	pfam02614	Glucuronate isomerase. This is a family
===									9	Halopiger_xanaduensis_SH_6_uid68105	NC_015667	plasmid pHALXA02									
336251593	hypothetical protein Halxa_0037	No	0		plasmid	36289	36663	+		Halopiger_xanaduensis_SH_6_uid68105	NC_015667	plasmid pHALXA02									
336251594	hypothetical protein Halxa_0038	No	0		plasmid	37464	37772	+		Halopiger_xanaduensis_SH_6_uid68105	NC_015667	plasmid pHALXA02									
336251595	hypothetical protein Halxa_0039	No	0		plasmid	38010	38777	-		Halopiger_xanaduensis_SH_6_uid68105	NC_015667	plasmid pHALXA02									
336251596	hypothetical protein Halxa_0040	No	0		plasmid	39030	39200	+		Halopiger_xanaduensis_SH_6_uid68105	NC_015667	plasmid pHALXA02		arCOG10199	1 (-)	S		Uncharacterized conserved protein			
336251597	hypothetical protein Halxa_0041	No	0		plasmid	39341	39814	+		Halopiger_xanaduensis_SH_6_uid68105	NC_015667	plasmid pHALXA02									
336251598	hypothetical protein Halxa_0042	No	0		plasmid	39993	40295	+		Halopiger_xanaduensis_SH_6_uid68105	NC_015667	plasmid pHALXA02									
336251599	hypothetical protein Halxa_0043	Yes	1		plasmid	40340	41116	+		Halopiger_xanaduensis_SH_6_uid68105	NC_015667	plasmid pHALXA02	clust_127								
336251600	hypothetical protein Halxa_0044	No	0		plasmid	41119	41886	+		Halopiger_xanaduensis_SH_6_uid68105	NC_015667	plasmid pHALXA02	clust_128								
336251601	hypothetical protein Halxa_0045	No	0		plasmid	41997	42521	+		Halopiger_xanaduensis_SH_6_uid68105	NC_015667	plasmid pHALXA02		arCOG06378	1 (-)	S		Uncharacterized conserved protein			
===									5	Haloquadratum_walsbyi_C23_uid162019	NC_017457	plasmid PL100									
385802305	hypothetical protein Hqrw_5002	No	0		plasmid	918	1166	+		Haloquadratum_walsbyi_C23_uid162019	NC_017457	plasmid PL100									
385802306	hypothetical protein Hqrw_5003	Yes	0		plasmid	1254	1478	+		Haloquadratum_walsbyi_C23_uid162019	NC_017457	plasmid PL100									
385802307	hypothetical protein Hqrw_5004	Yes	0		plasmid	1765	2136	+		Haloquadratum_walsbyi_C23_uid162019	NC_017457	plasmid PL100		arCOG09341	3 (-)	S		Uncharacterized conserved protein			
385802308	hypothetical protein Hqrw_5005	No	0		plasmid	2307	2939	+		Haloquadratum_walsbyi_C23_uid162019	NC_017457	plasmid PL100									
385802309	hypothetical protein Hqrw_5006	Yes	0		plasmid	3211	3576	+		Haloquadratum_walsbyi_C23_uid162019	NC_017457	plasmid PL100									
===									14	Haloquadratum_walsbyi_C23_uid162019	NC_017457	plasmid PL100									
385802331	hypothetical protein Hqrw_5033	No	3		plasmid	35726	36610	+		Haloquadratum_walsbyi_C23_uid162019	NC_017457	plasmid PL100		arCOG01917	12 (-)	S		Zn-ribbon domain containing protein			
385802332	hypothetical protein Hqrw_5035	No	0		plasmid	37383	38348	-		Haloquadratum_walsbyi_C23_uid162019	NC_017457	plasmid PL100									
385802333	hypothetical protein Hqrw_5036	No	0		plasmid	38470	38730	+		Haloquadratum_walsbyi_C23_uid162019	NC_017457	plasmid PL100									
385802334	hypothetical protein Hqrw_5037	No	0		plasmid	38902	39180	-		Haloquadratum_walsbyi_C23_uid162019	NC_017457	plasmid PL100									
385802335	hypothetical protein Hqrw_5038	No	0		plasmid	39177	39626	-		Haloquadratum_walsbyi_C23_uid162019	NC_017457	plasmid PL100									
385802336	hypothetical protein Hqrw_5039	No	0		plasmid	40678	41745	+		Haloquadratum_walsbyi_C23_uid162019	NC_017457	plasmid PL100		arCOG07683	1 (-)	L		Predicted nuclease of the RecB family			
385802337	hypothetical protein Hqrw_5040	No	0		plasmid	41922	42356	+		Haloquadratum_walsbyi_C23_uid162019	NC_017457	plasmid PL100									
385802338	hypothetical protein Hqrw_5041	No	0		plasmid	42679	42861	+		Haloquadratum_walsbyi_C23_uid162019	NC_017457	plasmid PL100									
385802339	hypothetical protein Hqrw_5043	No	0		plasmid	45065	45598	-		Haloquadratum_walsbyi_C23_uid162019	NC_017457	plasmid PL100		arCOG00741	1 (-)	K	ArsR	Transcriptional regulator containing HTH domain, ArsR family			
385802340	hypothetical protein Hqrw_5044	No	2		plasmid	45988	46242	-		Haloquadratum_walsbyi_C23_uid162019	NC_017457	plasmid PL100		arCOG09383	2	S		Uncharacterized conserved protein			
385802341	hypothetical protein Hqrw_5045	No	0		plasmid	46397	46651	+		Haloquadratum_walsbyi_C23_uid162019	NC_017457	plasmid PL100									
385802342	hypothetical protein Hqrw_5046	No	0		plasmid	48396	49547	+		Haloquadratum_walsbyi_C23_uid162019	NC_017457	plasmid PL100									
385802343	hypothetical protein Hqrw_5048	No	0		plasmid	50176	50364	-		Haloquadratum_walsbyi_C23_uid162019	NC_017457	plasmid PL100		arCOG04718	1 (-)	S		Uncharacterized conserved protein			
385802344	hypothetical protein Hqrw_5049	No	0		plasmid	50457	51001	-		Haloquadratum_walsbyi_C23_uid162019	NC_017457	plasmid PL100									
===									6	Haloquadratum_walsbyi_C23_uid162019	NC_017459	chromosome									
385802845	NUDIX family hydrolase	No	0			557822	558340	-		Haloquadratum_walsbyi_C23_uid162019	NC_017459	chromosome		arCOG01078	1 (-)	L	MutT	NUDIX family hydrolase			
385802846	hypothetical protein Hqrw_1540	No	2			558758	559642	-		Haloquadratum_walsbyi_C23_uid162019	NC_017459	chromosome		arCOG09100	1	S		Uncharacterized conserved protein			
385802847	hypothetical protein Hqrw_1541	No	2			559639	559872	-		Haloquadratum_walsbyi_C23_uid162019	NC_017459	chromosome		arCOG07567	1	S		Uncharacterized conserved protein			
385802848	hypothetical protein Hqrw_1542	No	0			559995	561101	+		Haloquadratum_walsbyi_C23_uid162019	NC_017459	chromosome									
385802849	hypothetical protein Hqrw_1543	No	0			561209	562273	+		Haloquadratum_walsbyi_C23_uid162019	NC_017459	chromosome									
385802850	hypothetical protein Hqrw_1544	No	0			562283	563248	+		Haloquadratum_walsbyi_C23_uid162019	NC_017459	chromosome		arCOG03382	1 (-)	U	TolB	Periplasmic component of the Tol biopolymer transport system			
===									5	Haloquadratum_walsbyi_C23_uid162019	NC_017459	chromosome									
385802857	coiled-coil protein	No	1			578144	580240	-		Haloquadratum_walsbyi_C23_uid162019	NC_017459	chromosome									
385802858	hypothetical protein Hqrw_1552	No	3			580237	581358	-		Haloquadratum_walsbyi_C23_uid162019	NC_017459	chromosome									
385802859	hypothetical protein Hqrw_1553	Yes	2			581355	581627	-		Haloquadratum_walsbyi_C23_uid162019	NC_017459	chromosome									
385802860	cell division protein FtsZ	No	0			581627	582916	-		Haloquadratum_walsbyi_C23_uid162019	NC_017459	chromosome		arCOG02202	4 (-)	D	FtsZ	Cell division GTPase			
385802861	hypothetical protein Hqrw_1555	No	2			583025	584740	-		Haloquadratum_walsbyi_C23_uid162019	NC_017459	chromosome									
===									9	Haloquadratum_walsbyi_C23_uid162019	NC_017459	chromosome									
385803451	hypothetical protein Hqrw_2240	No	0			1294581	1294937	+		Haloquadratum_walsbyi_C23_uid162019	NC_017459	chromosome		arCOG01957	1 (-)	P	TrkA	K+ transport system, NAD-binding component			
385803452	hypothetical protein Hqrw_2241	No	2			1295075	1295326	+		Haloquadratum_walsbyi_C23_uid162019	NC_017459	chromosome	clust_395								
385803453	hypothetical protein Hqrw_2242	No	0			1295485	1295856	-		Haloquadratum_walsbyi_C23_uid162019	NC_017459	chromosome	clust_287								
385803454	hypothetical protein Hqrw_2243	No	3			1296041	1296337	-		Haloquadratum_walsbyi_C23_uid162019	NC_017459	chromosome									
385803455	hypothetical protein Hqrw_2244	No	0			1296491	1296682	+		Haloquadratum_walsbyi_C23_uid162019	NC_017459	chromosome									
385803456	hypothetical protein Hqrw_2245	No	3			1296660	1296926	-		Haloquadratum_walsbyi_C23_uid162019	NC_017459	chromosome									
385803457	hypothetical protein Hqrw_2246	Yes	2			1297156	1297419	-		Haloquadratum_walsbyi_C23_uid162019	NC_017459	chromosome	clust_376								
385803458	hypothetical protein Hqrw_2247	No	0			1297975	1298301	+		Haloquadratum_walsbyi_C23_uid162019	NC_017459	chromosome	clust_321						0.000007	pfam06044	Dam-replacing family. Dam-replacing prot
385803459	haloviral protein	No	0			1299505	1300539	+		Haloquadratum_walsbyi_C23_uid162019	NC_017459	chromosome	arCOG08947,a	arCOG08947	1 (-)	L		LAGLIDADG family endonuclease			
===									7	Haloquadratum_walsbyi_C23_uid162019	NC_017459	chromosome									
385803476	small CPxCG-related zinc finger protein	No	0			1328360	1328563	+		Haloquadratum_walsbyi_C23_uid162019	NC_017459	chromosome		arCOG06377	1 (-)	S		Uncharacterized conserved protein			
385803477	hypothetical protein Hqrw_2269	No	2			1328580	1329074	+		Haloquadratum_walsbyi_C23_uid162019	NC_017459	chromosome		arCOG10376	1	S		Uncharacterized conserved protein			
385803478	hypothetical protein Hqrw_2270	No	5			1329324	1329941	-		Haloquadratum_walsbyi_C23_uid162019	NC_017459	chromosome		arCOG09321	3	S		Uncharacterized conserved protein			
385803479	hypothetical protein Hqrw_2271	Yes	5			1330197	1330775	-		Haloquadratum_walsbyi_C23_uid162019	NC_017459	chromosome									
385803480	hypothetical protein Hqrw_2272	No	0			1331082	1331462	-		Haloquadratum_walsbyi_C23_uid162019	NC_017459	chromosome	clust_281								
385803481	hypothetical protein Hqrw_2274	Yes	0			1331926	1332291	+		Haloquadratum_walsbyi_C23_uid162019	NC_017459	chromosome		arCOG09341	3 (-)	S		Uncharacterized conserved protein			
385803482	hypothetical protein Hqrw_2275	No	0			1332430	1332810	-		Haloquadratum_walsbyi_C23_uid162019	NC_017459	chromosome	clust_281								
===									6	Haloquadratum_walsbyi_C23_uid162019	NC_017459	chromosome									
385803969	phosphate/phosphonate ABC transporter subs	Yes	0			1936182	1937159	-		Haloquadratum_walsbyi_C23_uid162019	NC_017459	chromosome		arCOG01805	2 (-)	P	PhnD	ABC-type phosphate/phosphonate transport syst	8E-20	COG3221	ABC-type phosphate/phosphonate transport
385803970	phosphonate metabolism protein PhnG	No	0			1937492	1937941	+		Haloquadratum_walsbyi_C23_uid162019	NC_017459	chromosome	clust_235						0.00000007	COG3624	Uncharacterized enzyme of phosphonate me
385803971	phosphonate metabolism protein PhnH	No	0			1937938	1938612	+		Haloquadratum_walsbyi_C23_uid162019	NC_017459	chromosome	clust_153						0.00000002	COG3625	Uncharacterized enzyme of phosphonate me
385803972	phosphonate metabolism protein PhnI	No	0			1938612	1939763	+		Haloquadratum_walsbyi_C23_uid162019	NC_017459	chromosome	clust_84						2E-103	pfam05861	Bacterial phosphonate metabolism protein
385803973	phosphonate metabolism protein PhnJ	No	0			1939756	1940874	+		Haloquadratum_walsbyi_C23_uid162019	NC_017459	chromosome	clust_86						9E-118	pfam06007	Phosphonate metabolism protein PhnJ. Thi
385803974	phosphonate ABC transporter ATP-binding pro	No	0			1940871	1941707	+		Haloquadratum_walsbyi_C23_uid162019	NC_017459	chromosome		arCOG00181	2 (-)	E	DppD	ABC-type dipeptide/oligopeptide/nickel transpor	8E-71	COG4107	ABC-type phosphonate transport system, A
===									7	Haloquadratum_walsbyi_C23_uid162019	NC_017459	chromosome									
385804203	signal-transducing histidine kinase	No	0			2216027	2217037	-		Haloquadratum_walsbyi_C23_uid162019	NC_017459	chromosome		arCOG02331	2 (-)	T		Signal transduction histidine kinase	5E-20	COG0642	Signal transduction histidine kinase
385804204	hypothetical protein Hqrw_3190	No	0			2217551	2217841	-		Haloquadratum_walsbyi_C23_uid162019	NC_017459	chromosome	clust_358								
385804205	hypothetical protein Hqrw_3192	No	0			2218631	2218867	+		Haloquadratum_walsbyi_C23_uid162019	NC_017459	chromosome	clust_407								
385804206	hypothetical protein Hqrw_3193	No	4			2218985	2219350	-		Haloquadratum_walsbyi_C23_uid162019	NC_017459	chromosome	clust_291								
385804207	hypothetical protein Hqrw_3195	No	0			2219786	2220022	+		Haloquadratum_walsbyi_C23_uid162019	NC_017459	chromosome	clust_408								
385804208	hypothetical protein Hqrw_3196	No	0			2220365	2220604	-		Haloquadratum_walsbyi_C23_uid162019	NC_017459	chromosome									
385804209	hypothetical protein Hqrw_3198	No	0			2221290	2221711	+		Haloquadratum_walsbyi_C23_uid162019	NC_017459	chromosome		arCOG06157	1 (-)	S		Uncharacterized conserved protein			
===									8	Haloquadratum_walsbyi_C23_uid162019	NC_017459	chromosome									
385804211	hypothetical protein Hqrw_3200	No	0			2222245	2222550	-		Haloquadratum_walsbyi_C23_uid162019	NC_017459	chromosome		arCOG06398	3 (-)	S		Uncharacterized conserved protein			
385804212	hypothetical protein Hqrw_3201	No	0			2222916	2223098	-		Haloquadratum_walsbyi_C23_uid162019	NC_017459	chromosome									
385804213	hypothetical protein Hqrw_3202	No	0			2223117	2223437	-		Haloquadratum_walsbyi_C23_uid162019	NC_017459	chromosome									
385804214	hypothetical protein Hqrw_3203	No	0			2223767	2224027	-		Haloquadratum_walsbyi_C23_uid162019	NC_017459	chromosome									
385804215	hypothetical protein Hqrw_3204	No	0			2224496	2224840	-		Haloquadratum_walsbyi_C23_uid162019	NC_017459	chromosome									
385804216	hypothetical protein Hqrw_3205	No	0			2224897	2225241	-		Haloquadratum_walsbyi_C23_uid162019	NC_017459	chromosome									
385804217	hypothetical protein Hqrw_3206	No	0			2225277	2225582	-		Haloquadratum_walsbyi_C23_uid162019	NC_017459	chromosome									
385804218	preprotein translocase subunit SecY	No	9			2225757	2227226	-		Haloquadratum_walsbyi_C23_uid162019	NC_017459	chromosome		arCOG04169	3 (-)	U	SecY	Preprotein translocase subunit SecY	3E-48	COG0201	Preprotein translocase subunit SecY
===									8	Haloquadratum_walsbyi_C23_uid162019	NC_017459	chromosome									
385804666	hypothetical protein Hqrw_3739	No	0			2723677	2724234	-		Haloquadratum_walsbyi_C23_uid162019	NC_017459	chromosome									
385804667	small CPxCG-related zinc finger protein	No	0			2724316	2724465	-		Haloquadratum_walsbyi_C23_uid162019	NC_017459	chromosome		arCOG04494	2 (-)	S		Uncharacterized conserved protein			
385804668	hypothetical protein Hqrw_3741	Yes	3			2724737	2725027	-		Haloquadratum_walsbyi_C23_uid162019	NC_017459	chromosome									
385804669	hypothetical protein Hqrw_3742	No	2			2725101	2725277	-		Haloquadratum_walsbyi_C23_uid162019	NC_017459	chromosome									
385804670	hypothetical protein Hqrw_3743	No	0			2725413	2725877	-		Haloquadratum_walsbyi_C23_uid162019	NC_017459	chromosome									
385804671	hypothetical protein Hqrw_3744	Yes	2			2726067	2726336	+		Haloquadratum_walsbyi_C23_uid162019	NC_017459	chromosome	clust_376								
385804672	hypothetical protein Hqrw_3745	No	0			2726805	2727392	+		Haloquadratum_walsbyi_C23_uid162019	NC_017459	chromosome									
385804673	hypothetical protein Hqrw_3746	No	0			2727406	2727947	-		Haloquadratum_walsbyi_C23_uid162019	NC_017459	chromosome		arCOG01764	2 (-)	K	SPT15	TATA-box binding protein (TBP), component of T	2E-17	COG2101	TATA-box binding protein (TBP), componen
===									5	Haloquadratum_walsbyi_DSM_16790_uid58673	NC_008212	chromosome									
110667778	hypothetical protein HQ1823A	No	0			1012002	1012199	+		Haloquadratum_walsbyi_DSM_16790_uid58673	NC_008212	chromosome		arCOG09361	1	S		Uncharacterized conserved protein			
110667779	hypothetical protein HQ1824A	No	2			1013196	1013780	-		Haloquadratum_walsbyi_DSM_16790_uid58673	NC_008212	chromosome									
110667780	hypothetical protein HQ1825A	No	4			1014646	1015119	-		Haloquadratum_walsbyi_DSM_16790_uid58673	NC_008212	chromosome		arCOG06199	1 (-)	S		Uncharacterized conserved protein			
110667781	hypothetical protein HQ1826A	No	0			1015234	1015653	-		Haloquadratum_walsbyi_DSM_16790_uid58673	NC_008212	chromosome									
110667782	hypothetical protein HQ1827A	No	0			1015690	1016082	-		Haloquadratum_walsbyi_DSM_16790_uid58673	NC_008212	chromosome									
===									6	Haloquadratum_walsbyi_DSM_16790_uid58673	NC_008212	chromosome									
110668001	hypothetical protein HQ2056A	No	0			1279161	1279676	+		Haloquadratum_walsbyi_DSM_16790_uid58673	NC_008212	chromosome		arCOG06432	1 (-)	S		Uncharacterized conserved protein			
110668002	hypothetical protein HQ2057A	No	3			1281026	1281415	-		Haloquadratum_walsbyi_DSM_16790_uid58673	NC_008212	chromosome		arCOG06384	1	S		Uncharacterized conserved protein			
110668003	hypothetical protein HQ2058A	Yes	0			1281749	1282339	+		Haloquadratum_walsbyi_DSM_16790_uid58673	NC_008212	chromosome									
110668004	hypothetical protein HQ2059A	No	4			1282509	1283123	+		Haloquadratum_walsbyi_DSM_16790_uid58673	NC_008212	chromosome		arCOG06158	1	S		Uncharacterized conserved protein			
110668005	hypothetical protein HQ2060A	No	0			1283490	1283768	+		Haloquadratum_walsbyi_DSM_16790_uid58673	NC_008212	chromosome									
110668006	hypothetical protein HQ2061A	No	0			1284055	1284249	-		Haloquadratum_walsbyi_DSM_16790_uid58673	NC_008212	chromosome		arCOG04677	1 (-)	I		Methylmalonyl-CoA mutase, N-terminal domain/subunit			
===									5	Haloquadratum_walsbyi_DSM_16790_uid58673	NC_008212	chromosome									
110668015	hypothetical protein HQ2070A	No	2			1290858	1291109	+		Haloquadratum_walsbyi_DSM_16790_uid58673	NC_008212	chromosome	clust_395								
110668016	hypothetical protein HQ2071A	No	0			1291268	1291639	-		Haloquadratum_walsbyi_DSM_16790_uid58673	NC_008212	chromosome	clust_287								
110668017	IS1341-type transposase	No	0			1291769	1293118	-		Haloquadratum_walsbyi_DSM_16790_uid58673	NC_008212	chromosome		arCOG00683	3 (-)	L		Transposase	7E-19	pfam01385	Probable transposase. This family includ ; Putative transposase DNA-binding domain.
110668018	hypothetical protein HQ2073A	No	0			1294080	1294406	+		Haloquadratum_walsbyi_DSM_16790_uid58673	NC_008212	chromosome	clust_321						0.000007	pfam06044	Dam-replacing family. Dam-replacing prot
110668019	hypothetical protein HQ2074A	No	0			1294597	1295067	-		Haloquadratum_walsbyi_DSM_16790_uid58673	NC_008212	chromosome									
===									8	Haloquadratum_walsbyi_DSM_16790_uid58673	NC_008212	chromosome									
110668421	hypothetical protein HQ2512A	No	0			1757468	1757740	+		Haloquadratum_walsbyi_DSM_16790_uid58673	NC_008212	chromosome		arCOG03164	1 (-)	L		Predicted site-specific integrase-resolvase			
110668422	hypothetical protein HQ2513A	No	0			1757733	1758296	+		Haloquadratum_walsbyi_DSM_16790_uid58673	NC_008212	chromosome									
110668423	hypothetical protein HQ2514A	No	0			1758352	1758663	+		Haloquadratum_walsbyi_DSM_16790_uid58673	NC_008212	chromosome									
110668424	IS1341-type transposase-like protein	No	0			1758828	1759088	+		Haloquadratum_walsbyi_DSM_16790_uid58673	NC_008212	chromosome									
110668425	hypothetical protein HQ2516A	No	0			1759219	1759431	+		Haloquadratum_walsbyi_DSM_16790_uid58673	NC_008212	chromosome									
110668426	RNA-associated protein	No	0			1759535	1760260	+		Haloquadratum_walsbyi_DSM_16790_uid58673	NC_008212	chromosome		arCOG04187	2 (-)	J		Predicted exosome subunit	2E-88	COG1500	Predicted exosome subunit
110668427	hypothetical protein HQ2518A	No	1			1760453	1760857	+		Haloquadratum_walsbyi_DSM_16790_uid58673	NC_008212	chromosome									
110668428	TP rich cell surface protein viral Tpx	Yes	0			1761085	1761405	-		Haloquadratum_walsbyi_DSM_16790_uid58673	NC_008212	chromosome							0.000000008	pfam01456	Mucin-like glycoprotein. This family of
===									6	Haloquadratum_walsbyi_DSM_16790_uid58673	NC_008212	chromosome									
110668505	ABC-type phosphate/phosphonate transport sy	No	1			1871784	1872815	-		Haloquadratum_walsbyi_DSM_16790_uid58673	NC_008212	chromosome		arCOG01805	2 (-)	P	PhnD	ABC-type phosphate/phosphonate transport syst	2E-18	COG3221	ABC-type phosphate/phosphonate transport
110668506	phosphonate metabolism protein PhnG	No	0			1873085	1873534	+		Haloquadratum_walsbyi_DSM_16790_uid58673	NC_008212	chromosome	clust_235						0.000000008	COG3624	Uncharacterized enzyme of phosphonate me
110668507	phosphonate metabolism protein PhnH	No	0			1873531	1874205	+		Haloquadratum_walsbyi_DSM_16790_uid58673	NC_008212	chromosome	clust_153						0.000000006	COG3625	Uncharacterized enzyme of phosphonate me
110668508	phosphonate metabolism protein PhnI	No	0			1874205	1875356	+		Haloquadratum_walsbyi_DSM_16790_uid58673	NC_008212	chromosome	clust_84						7E-102	pfam05861	Bacterial phosphonate metabolism protein
110668509	phosphonate metabolism protein PhnJ	No	0			1875349	1876467	+		Haloquadratum_walsbyi_DSM_16790_uid58673	NC_008212	chromosome	clust_86						2E-116	pfam06007	Phosphonate metabolism protein PhnJ. Thi
110668510	phosphonate ABC transporter ATP-binding pro	No	0			1876464	1877300	+		Haloquadratum_walsbyi_DSM_16790_uid58673	NC_008212	chromosome		arCOG00181	2 (-)	E	DppD	ABC-type dipeptide/oligopeptide/nickel transpor	4E-71	COG4107	ABC-type phosphonate transport system, A
===									6	Haloquadratum_walsbyi_DSM_16790_uid58673	NC_008212	chromosome									
110668713	signal-transducing histidine kinase	No	0			2122710	2123723	-		Haloquadratum_walsbyi_DSM_16790_uid58673	NC_008212	chromosome		arCOG02331	2 (-)	T		Signal transduction histidine kinase	2E-20	COG0642	Signal transduction histidine kinase
110668714	hypothetical protein HQ2815A	No	0			2124237	2124527	-		Haloquadratum_walsbyi_DSM_16790_uid58673	NC_008212	chromosome	clust_358								
110668715	hypothetical protein HQ2816A	No	0			2125317	2125553	+		Haloquadratum_walsbyi_DSM_16790_uid58673	NC_008212	chromosome	clust_407								
110668716	hypothetical protein HQ2817A	No	4			2125671	2126036	-		Haloquadratum_walsbyi_DSM_16790_uid58673	NC_008212	chromosome	clust_291								
110668717	hypothetical protein HQ2818A	No	0			2126475	2126711	+		Haloquadratum_walsbyi_DSM_16790_uid58673	NC_008212	chromosome	clust_408								
110668718	preprotein translocase subunit SecY	No	9			2126989	2128458	-		Haloquadratum_walsbyi_DSM_16790_uid58673	NC_008212	chromosome		arCOG04169	3 (-)	U	SecY	Preprotein translocase subunit SecY	1E-47	COG0201	Preprotein translocase subunit SecY
===									11	Haloquadratum_walsbyi_DSM_16790_uid58673	NC_008212	chromosome									
110669158	adenine-specific DNA methyltransferase	No	0	YES		2623995	2625362	-		Haloquadratum_walsbyi_DSM_16790_uid58673	NC_008212	chromosome		arCOG02636	3 (-)	L		Adenine-specific DNA methylase	0.000000002	pfam13659	Methyltransferase domain. This family co
110669159	hypothetical protein HQ3278A	No	0	YES		2625428	2626273	-		Haloquadratum_walsbyi_DSM_16790_uid58673	NC_008212	chromosome		arCOG09411	1	S		Uncharacterized conserved protein			
110669160	hypothetical protein HQ3279A	No	1	YES		2626426	2626653	-		Haloquadratum_walsbyi_DSM_16790_uid58673	NC_008212	chromosome									
110669161	hypothetical protein HQ3280A	No	0			2626854	2629604	-		Haloquadratum_walsbyi_DSM_16790_uid58673	NC_008212	chromosome									
110669162	hypothetical protein HQ3281A	No	0			2630461	2631957	-		Haloquadratum_walsbyi_DSM_16790_uid58673	NC_008212	chromosome									
110669163	hypothetical protein HQ3282A	No	0			2632395	2633450	-		Haloquadratum_walsbyi_DSM_16790_uid58673	NC_008212	chromosome	clust_91								
110669164	hypothetical protein HQ3283A	No	0			2633527	2634612	+		Haloquadratum_walsbyi_DSM_16790_uid58673	NC_008212	chromosome									
110669165	protease HtpX	No	3			2634886	2635743	-		Haloquadratum_walsbyi_DSM_16790_uid58673	NC_008212	chromosome		arCOG01334	4 (-)	O	HtpX	Zn-dependent protease with chaperone function			
110669166	hypothetical protein HQ3285A	No	0			2636491	2636970	-		Haloquadratum_walsbyi_DSM_16790_uid58673	NC_008212	chromosome									
110669167	hypothetical protein HQ3286A	No	0			2637051	2637578	-		Haloquadratum_walsbyi_DSM_16790_uid58673	NC_008212	chromosome									
110669168	hypothetical protein HQ3287A	No	2			2637849	2638088	+		Haloquadratum_walsbyi_DSM_16790_uid58673	NC_008212	chromosome									
===									6	Haloquadratum_walsbyi_DSM_16790_uid58673	NC_008212	chromosome									
110669172	conjugation protein	No	0			2642296	2644203	+		Haloquadratum_walsbyi_DSM_16790_uid58673	NC_008212	chromosome		arCOG04816	4 (-)	U		TraG/TraD/VirD4 family enzyme, ATPase			
110669173	hypothetical protein HQ3292A	No	1			2644502	2645341	+		Haloquadratum_walsbyi_DSM_16790_uid58673	NC_008212	chromosome									
110669174	hypothetical protein HQ3293A	No	0			2645342	2645749	+		Haloquadratum_walsbyi_DSM_16790_uid58673	NC_008212	chromosome		arCOG10381	2	S		Uncharacterized conserved protein			
110669175	hypothetical protein HQ3294A	No	0			2645754	2646494	-		Haloquadratum_walsbyi_DSM_16790_uid58673	NC_008212	chromosome									
110669176	hypothetical protein HQ3295A	No	0			2647199	2648488	-		Haloquadratum_walsbyi_DSM_16790_uid58673	NC_008212	chromosome							0.0000001	COG1196	Chromosome segregation ATPases
110669177	AAA-type ATPase (cell division control protein	No	0			2650476	2651789	+		Haloquadratum_walsbyi_DSM_16790_uid58673	NC_008212	chromosome		arCOG06414	1 (-)	O	SpoVK	ATPase of the AAA+ class	5E-62	COG1222	ATP-dependent 26S proteasome regulatory
===									6	Haloquadratum_walsbyi_DSM_16790_uid58673	NC_008212	chromosome									
110669249	endonuclease	Yes	0			2736485	2737477	-		Haloquadratum_walsbyi_DSM_16790_uid58673	NC_008212	chromosome		arCOG03192	6 (-)	L		Micrococcal nuclease (thermonuclease)	2E-20	pfam00932	Lamin Tail Domain. The lamin-tail domain ; Micrococcal nuclease (thermonuclease) ho
110669250	hypothetical protein HQ3376A	No	0			2737628	2737951	-		Haloquadratum_walsbyi_DSM_16790_uid58673	NC_008212	chromosome		arCOG06185	1	S		Uncharacterized conserved protein			
110669251	hypothetical protein HQ3377A	No	2			2738131	2738322	+		Haloquadratum_walsbyi_DSM_16790_uid58673	NC_008212	chromosome									
110669252	IS1341-type transposase (nonfunctional, centra	No	0			2738784	2738837	-		Haloquadratum_walsbyi_DSM_16790_uid58673	NC_008212	chromosome									
110669253	IS200-type transposase (nonfunctional,C-termir	No	0			2739640	2739714	+		Haloquadratum_walsbyi_DSM_16790_uid58673	NC_008212	chromosome									
110669254	ABC-type spermidine/putrescine transport syst	No	6			2739788	2740609	-		Haloquadratum_walsbyi_DSM_16790_uid58673	NC_008212	chromosome		arCOG00162	1 (-)	E	PotC	ABC-type spermidine/putrescine transport systen	8E-50	COG1177	ABC-type spermidine/putrescine transport
===									11	Haloquadratum_walsbyi_DSM_16790_uid58673	NC_008213	plasmid PL47									
109644368	phage integrase/recombinase xerD	No	0	YES	plasmid	19	573	-		Haloquadratum_walsbyi_DSM_16790_uid58673	NC_008213	plasmid PL47		arCOG06451	4 (-)	L	XerC	Integrase	2E-15	COG4974	Site-specific recombinase XerD
109644369	hypothetical protein HQ4002A	No	0	YES	plasmid	825	1475	-		Haloquadratum_walsbyi_DSM_16790_uid58673	NC_008213	plasmid PL47									
109644370	hypothetical protein HQ4003A	No	0	YES	plasmid	1721	2467	-		Haloquadratum_walsbyi_DSM_16790_uid58673	NC_008213	plasmid PL47									
109644371	hypothetical protein HQ4005A	No	5	YES	plasmid	3882	4634	+		Haloquadratum_walsbyi_DSM_16790_uid58673	NC_008213	plasmid PL47									
109644372	hypothetical protein HQ4006A	No	2	YES	plasmid	4657	5145	+		Haloquadratum_walsbyi_DSM_16790_uid58673	NC_008213	plasmid PL47									
109644373	cell surface glycoprotein precursor	No	0	YES	plasmid	5442	6845	-		Haloquadratum_walsbyi_DSM_16790_uid58673	NC_008213	plasmid PL47		arCOG03906	2 (-)	S		Cell surface protein	0.0001	pfam07263	Dentin matrix protein 1 (DMP1). This fam
109644374	hypothetical protein HQ4008A	No	0	YES	plasmid	7266	7550	-		Haloquadratum_walsbyi_DSM_16790_uid58673	NC_008213	plasmid PL47									
109644375	hypothetical protein HQ4009A	No	0	YES	plasmid	7652	7918	-		Haloquadratum_walsbyi_DSM_16790_uid58673	NC_008213	plasmid PL47									
109644376	hypothetical protein HQ4010A	No	0	YES	plasmid	8398	8799	+		Haloquadratum_walsbyi_DSM_16790_uid58673	NC_008213	plasmid PL47		arCOG07975	1 (-)	V		Ribbon-helix-helix protein, copG family			
109644377	hypothetical protein HQ4011A	No	0	YES	plasmid	9163	9540	+		Haloquadratum_walsbyi_DSM_16790_uid58673	NC_008213	plasmid PL47									
109644378	hypothetical protein HQ4012A	No	2	YES	plasmid	10716	11063	+		Haloquadratum_walsbyi_DSM_16790_uid58673	NC_008213	plasmid PL47									
===									6	Halorhabdus_utahensis_DSM_12940_uid59189	NC_013158	chromosome									
257051193	hypothetical protein Huta_0104	No	0	YES		102502	103155	-		Halorhabdus_utahensis_DSM_12940_uid59189	NC_013158	chromosome									
257051194	hypothetical protein Huta_0105	No	0	YES		103376	104524	-		Halorhabdus_utahensis_DSM_12940_uid59189	NC_013158	chromosome									
257051195	hypothetical protein Huta_0106	No	0	YES		104677	105270	+		Halorhabdus_utahensis_DSM_12940_uid59189	NC_013158	chromosome									
257051196	hypothetical protein Huta_0107	No	0	YES		105286	105507	-		Halorhabdus_utahensis_DSM_12940_uid59189	NC_013158	chromosome		arCOG10165	3 (-)	S		Uncharacterized conserved protein			
257051197	hypothetical protein Huta_0108	No	0	YES		105534	105686	-		Halorhabdus_utahensis_DSM_12940_uid59189	NC_013158	chromosome									
257051198	hypothetical protein Huta_0109	No	0	YES		105694	106080	-		Halorhabdus_utahensis_DSM_12940_uid59189	NC_013158	chromosome									
===									7	Halorhabdus_utahensis_DSM_12940_uid59189	NC_013158	chromosome									
257051323	hypothetical protein Huta_0235	No	0	YES		228412	228930	+		Halorhabdus_utahensis_DSM_12940_uid59189	NC_013158	chromosome		arCOG06448	1 (-)	V		HEPN domain containing protein			
257051324	hypothetical protein Huta_0236	No	0	YES		228952	229341	-		Halorhabdus_utahensis_DSM_12940_uid59189	NC_013158	chromosome									
257051325	hypothetical protein Huta_0237	No	0	YES		229320	229511	-		Halorhabdus_utahensis_DSM_12940_uid59189	NC_013158	chromosome									
257051326	hypothetical protein Huta_0238	No	0	YES		229940	231184	+		Halorhabdus_utahensis_DSM_12940_uid59189	NC_013158	chromosome									
257051327	hypothetical protein Huta_0239	No	0	YES		231387	232166	+		Halorhabdus_utahensis_DSM_12940_uid59189	NC_013158	chromosome									
257051328	HNH endonuclease	No	0	YES		232561	233535	+		Halorhabdus_utahensis_DSM_12940_uid59189	NC_013158	chromosome		arCOG03898	7 (-)	V	McrA	Restriction endonuclease, McrA/HNH family			
257051329	hypothetical protein Huta_0241	No	0	YES		233554	234960	-		Halorhabdus_utahensis_DSM_12940_uid59189	NC_013158	chromosome		arCOG06786	9	Y		Rolling circle replication protein Rep			
===									6	Halorhabdus_utahensis_DSM_12940_uid59189	NC_013158	chromosome									
257051880	deoxyguanosinetriphosphate triphosphohydrol	No	0			776396	777760	-		Halorhabdus_utahensis_DSM_12940_uid59189	NC_013158	chromosome		arCOG07445	3 (-)	F	Dgt	dGTP triphosphohydrolase	2E-52	COG0232	dGTP triphosphohydrolase
257051881	hypothetical protein Huta_0796	No	0			777778	777984	-		Halorhabdus_utahensis_DSM_12940_uid59189	NC_013158	chromosome									
257051882	hypothetical protein Huta_0797	No	4			778353	778745	+		Halorhabdus_utahensis_DSM_12940_uid59189	NC_013158	chromosome									
257051883	hypothetical protein Huta_0798	Yes	1			778754	779509	+		Halorhabdus_utahensis_DSM_12940_uid59189	NC_013158	chromosome									
257051884	hypothetical protein Huta_0799	Yes	2			779526	781265	+		Halorhabdus_utahensis_DSM_12940_uid59189	NC_013158	chromosome									
257051885	hypothetical protein Huta_0800	Yes	2			781265	781804	+		Halorhabdus_utahensis_DSM_12940_uid59189	NC_013158	chromosome		arCOG08168	1 (-)	S		Uncharacterized conserved protein			
===									7	Halorhabdus_utahensis_DSM_12940_uid59189	NC_013158	chromosome									
257051889	TIR protein	No	0			784289	785086	-		Halorhabdus_utahensis_DSM_12940_uid59189	NC_013158	chromosome									
257051890	hypothetical protein Huta_0805	No	0			785444	787063	-		Halorhabdus_utahensis_DSM_12940_uid59189	NC_013158	chromosome		arCOG06319	6 (-)	R		Protein containing HTH domain			
257051891	RNA polymerase, sigma-24 subunit, ECF subfa	No	0			787164	787364	-		Halorhabdus_utahensis_DSM_12940_uid59189	NC_013158	chromosome		arCOG08182	1	S		Uncharacterized conserved protein			
257051892	hypothetical protein Huta_0807	No	0			787361	787525	-		Halorhabdus_utahensis_DSM_12940_uid59189	NC_013158	chromosome									
257051893	hypothetical protein Huta_0808	No	0			787522	787701	-		Halorhabdus_utahensis_DSM_12940_uid59189	NC_013158	chromosome									
257051894	hypothetical protein Huta_0809	No	0			787916	788146	-		Halorhabdus_utahensis_DSM_12940_uid59189	NC_013158	chromosome		arCOG09149	1	S		Uncharacterized conserved protein			
257051895	30S ribosomal protein S10P	No	0			788518	788826	-		Halorhabdus_utahensis_DSM_12940_uid59189	NC_013158	chromosome		arCOG01758	2 (-)	J	RpsJ	Ribosomal protein S10	2E-20	COG0051	Ribosomal protein S10
===									7	Halorhabdus_utahensis_DSM_12940_uid59189	NC_013158	chromosome									
257052910	hypothetical protein Huta_1840	Yes	0			1848357	1849421	-		Halorhabdus_utahensis_DSM_12940_uid59189	NC_013158	chromosome		arCOG08140	1 (-)	S		Uncharacterized conserved protein			
257052911	hypothetical protein Huta_1841	No	2			1850082	1850429	+		Halorhabdus_utahensis_DSM_12940_uid59189	NC_013158	chromosome									
257052912	hypothetical protein Huta_1842	No	2			1850454	1850705	+		Halorhabdus_utahensis_DSM_12940_uid59189	NC_013158	chromosome									
257052913	hypothetical protein Huta_1843	No	2			1850731	1850985	-		Halorhabdus_utahensis_DSM_12940_uid59189	NC_013158	chromosome									
257052914	hypothetical protein Huta_1844	No	4			1851112	1851552	-		Halorhabdus_utahensis_DSM_12940_uid59189	NC_013158	chromosome									
257052915	hypothetical protein Huta_1845	No	1			1851549	1852085	-		Halorhabdus_utahensis_DSM_12940_uid59189	NC_013158	chromosome									
257052916	oligopeptide/dipeptide ABC transporter, ATPa	No	0			1852100	1853452	-		Halorhabdus_utahensis_DSM_12940_uid59189	NC_013158	chromosome		arCOG00184	2 (-)	E	AppF	ABC-type oligopeptide transport system, ATPase	6E-87	COG4608	ABC-type oligopeptide transport system,
===									10	Halorhabdus_utahensis_DSM_12940_uid59189	NC_013158	chromosome									
257053099	hypothetical protein Huta_2031	No	0			2048739	2048864	-		Halorhabdus_utahensis_DSM_12940_uid59189	NC_013158	chromosome									
257053100	hypothetical protein Huta_2032	No	1			2048957	2054683	+		Halorhabdus_utahensis_DSM_12940_uid59189	NC_013158	chromosome	arCOG07561,a	arCOG07561	7 (-)	K		Predicted regulatory protein containing HTH domain			
257053101	hypothetical protein Huta_2033	No	0			2054851	2056074	+		Halorhabdus_utahensis_DSM_12940_uid59189	NC_013158	chromosome		arCOG09139	5	S		Uncharacterized conserved protein			
257053102	hypothetical protein Huta_2034	No	0			2056192	2056503	+		Halorhabdus_utahensis_DSM_12940_uid59189	NC_013158	chromosome									
257053103	hypothetical protein Huta_2035	No	0			2056694	2057785	+		Halorhabdus_utahensis_DSM_12940_uid59189	NC_013158	chromosome									
257053104	hypothetical protein Huta_2036	No	0			2057787	2058803	+		Halorhabdus_utahensis_DSM_12940_uid59189	NC_013158	chromosome									
257053105	hypothetical protein Huta_2037	No	0			2058822	2059094	-		Halorhabdus_utahensis_DSM_12940_uid59189	NC_013158	chromosome									
257053106	hypothetical protein Huta_2038	No	0			2059182	2061131	+		Halorhabdus_utahensis_DSM_12940_uid59189	NC_013158	chromosome									
257053107	hypothetical protein Huta_2039	No	0			2061194	2061532	+		Halorhabdus_utahensis_DSM_12940_uid59189	NC_013158	chromosome									
257053108	hypothetical protein Huta_2040	No	0			2061867	2062799	+		Halorhabdus_utahensis_DSM_12940_uid59189	NC_013158	chromosome		arCOG04518	1 (-)	K		Predicted transcriptional regulator, contains HTH domain			
===									7	Halorhabdus_utahensis_DSM_12940_uid59189	NC_013158	chromosome									
257053127	hypothetical protein Huta_2060	No	3			2088965	2091148	+		Halorhabdus_utahensis_DSM_12940_uid59189	NC_013158	chromosome		arCOG03127	3 (-)	P	Kch	Ion channel fused to ion transporting domain	0.0000008	pfam13576	Pentapeptide repeats (9 copies). ; Pentapeptide repeats (9 copies). ; Pentapeptide repeats (9 copies).
257053128	hypothetical protein Huta_2061	No	2			2091615	2092175	-		Halorhabdus_utahensis_DSM_12940_uid59189	NC_013158	chromosome									
257053129	hypothetical protein Huta_2062	No	4			2092472	2093200	+		Halorhabdus_utahensis_DSM_12940_uid59189	NC_013158	chromosome									
257053130	hypothetical protein Huta_2063	No	0			2093291	2093419	-		Halorhabdus_utahensis_DSM_12940_uid59189	NC_013158	chromosome									
257053131	hypothetical protein Huta_2064	No	1			2093564	2093950	-		Halorhabdus_utahensis_DSM_12940_uid59189	NC_013158	chromosome							0.00000008	COG3874	Uncharacterized conserved protein
257053132	hypothetical protein Huta_2065	No	0			2094179	2094919	+		Halorhabdus_utahensis_DSM_12940_uid59189	NC_013158	chromosome		arCOG08154	1	S		Uncharacterized conserved protein			
257053133	hypothetical protein Huta_2066	No	4			2094959	2095966	-		Halorhabdus_utahensis_DSM_12940_uid59189	NC_013158	chromosome		arCOG08131	1 (-)	S		Uncharacterized conserved protein			
===									5	Halorhabdus_utahensis_DSM_12940_uid59189	NC_013158	chromosome									
257053137	hypothetical protein Huta_2070	No	0			2099467	2099661	+		Halorhabdus_utahensis_DSM_12940_uid59189	NC_013158	chromosome									
257053138	glutamyl-tRNA synthetase	No	0			2099809	2101557	+		Halorhabdus_utahensis_DSM_12940_uid59189	NC_013158	chromosome		arCOG04302	1 (-)	J	GlnS	Glutamyl- or glutaminyl-tRNA synthetase	6E-46	COG0008	Glutamyl- and glutaminyl-tRNA synthetase
257053139	hypothetical protein Huta_2072	No	4			2101744	2102241	-		Halorhabdus_utahensis_DSM_12940_uid59189	NC_013158	chromosome									
257053140	hypothetical protein Huta_2073	No	0			2102242	2103183	-		Halorhabdus_utahensis_DSM_12940_uid59189	NC_013158	chromosome		arCOG09035	3	S		Uncharacterized conserved protein			
257053141	hypothetical protein Huta_2074	No	0			2103184	2103435	-		Halorhabdus_utahensis_DSM_12940_uid59189	NC_013158	chromosome									
===									5	Halorhabdus_utahensis_DSM_12940_uid59189	NC_013158	chromosome									
257053353	hypothetical protein Huta_2286	No	0			2336381	2336626	+		Halorhabdus_utahensis_DSM_12940_uid59189	NC_013158	chromosome									
257053354	peptidylprolyl isomerase FKBP-type	No	0			2336641	2337114	-		Halorhabdus_utahensis_DSM_12940_uid59189	NC_013158	chromosome		arCOG00981	1 (-)	O	SlpA	FKBP-type peptidyl-prolyl cis-trans isomerase 2	2E-27	COG1047	FKBP-type peptidyl-prolyl cis-trans isom
257053355	hypothetical protein Huta_2288	No	3			2337721	2338053	-		Halorhabdus_utahensis_DSM_12940_uid59189	NC_013158	chromosome									
257053356	hypothetical protein Huta_2289	Yes	2			2338206	2338670	+		Halorhabdus_utahensis_DSM_12940_uid59189	NC_013158	chromosome									
257053357	hypothetical protein Huta_2290	No	0			2338868	2339275	+		Halorhabdus_utahensis_DSM_12940_uid59189	NC_013158	chromosome									
===									9	Halorhabdus_utahensis_DSM_12940_uid59189	NC_013158	chromosome									
257053708	hypothetical protein Huta_2647	Yes	0			2718707	2723170	+		Halorhabdus_utahensis_DSM_12940_uid59189	NC_013158	chromosome	arCOG07561,a	arCOG07561	7 (-)	K		Predicted regulatory protein containing HTH domain			
257053709	hypothetical protein Huta_2648	No	1			2723157	2723681	+		Halorhabdus_utahensis_DSM_12940_uid59189	NC_013158	chromosome									
257053710	hypothetical protein Huta_2649	No	0			2723663	2723878	+		Halorhabdus_utahensis_DSM_12940_uid59189	NC_013158	chromosome									
257053711	hypothetical protein Huta_2650	No	0			2723875	2724804	+		Halorhabdus_utahensis_DSM_12940_uid59189	NC_013158	chromosome									
257053712	hypothetical protein Huta_2651	No	0			2724967	2725209	+		Halorhabdus_utahensis_DSM_12940_uid59189	NC_013158	chromosome									
257053713	hypothetical protein Huta_2652	No	0			2725279	2725380	+		Halorhabdus_utahensis_DSM_12940_uid59189	NC_013158	chromosome									
257053714	hypothetical protein Huta_2653	Yes	0			2725548	2725985	+		Halorhabdus_utahensis_DSM_12940_uid59189	NC_013158	chromosome									
257053715	hypothetical protein Huta_2654	No	0			2726030	2726692	+		Halorhabdus_utahensis_DSM_12940_uid59189	NC_013158	chromosome		arCOG09155	1	S		Uncharacterized conserved protein			
257053716	hypothetical protein Huta_2655	No	0			2728255	2728788	+		Halorhabdus_utahensis_DSM_12940_uid59189	NC_013158	chromosome		arCOG04545	1 (-)	S		Uncharacterized conserved protein			
===									5	Halorhabdus_utahensis_DSM_12940_uid59189	NC_013158	chromosome									
257054016	hypothetical protein Huta_2955	No	0			3044491	3045747	+		Halorhabdus_utahensis_DSM_12940_uid59189	NC_013158	chromosome		arCOG08155	1	S		Uncharacterized conserved protein			
257054017	Polyprenyl synthetase	No	0			3045822	3046748	+		Halorhabdus_utahensis_DSM_12940_uid59189	NC_013158	chromosome		arCOG01726	1 (-)	H	IspA	Geranylgeranyl pyrophosphate synthase	1E-41	COG0142	Geranylgeranyl pyrophosphate synthase
257054018	hypothetical protein Huta_2957	No	4			3046825	3047292	+		Halorhabdus_utahensis_DSM_12940_uid59189	NC_013158	chromosome									
257054019	hypothetical protein Huta_2958	No	4			3047294	3047782	+		Halorhabdus_utahensis_DSM_12940_uid59189	NC_013158	chromosome									
257054020	hypothetical protein Huta_2959	No	8			3047866	3049410	+		Halorhabdus_utahensis_DSM_12940_uid59189	NC_013158	chromosome									
===									10	Halorubrum_lacusprofundi_ATCC_49239_uid5880	NC_012028	chromosome 2									
222475700	hypothetical protein Hlac_2764	No	0	YES		21404	21847	+		Halorubrum_lacusprofundi_ATCC_49239_uid5880	NC_012028	chromosome 2		arCOG06153	3 (-)	S		Uncharacterized conserved protein			
222475701	hypothetical protein Hlac_2765	No	0	YES		21917	22300	+		Halorubrum_lacusprofundi_ATCC_49239_uid5880	NC_012028	chromosome 2		arCOG10630	1	S		Uncharacterized conserved protein			
222475702	hypothetical protein Hlac_2766	No	0	YES		22373	24721	-		Halorubrum_lacusprofundi_ATCC_49239_uid5880	NC_012028	chromosome 2									
222475703	hypothetical protein Hlac_2767	No	0	YES		24792	26549	-		Halorubrum_lacusprofundi_ATCC_49239_uid5880	NC_012028	chromosome 2									
222475704	hypothetical protein Hlac_2770	No	0	YES		29101	29832	+		Halorubrum_lacusprofundi_ATCC_49239_uid5880	NC_012028	chromosome 2	clust_33								
222475705	hypothetical protein Hlac_2771	No	0	YES		30318	30599	+		Halorubrum_lacusprofundi_ATCC_49239_uid5880	NC_012028	chromosome 2	clust_367								
222475706	hypothetical protein Hlac_2772	No	0	YES		30856	31266	+		Halorubrum_lacusprofundi_ATCC_49239_uid5880	NC_012028	chromosome 2									
222475707	hypothetical protein Hlac_2773	No	0	YES		31397	31795	-		Halorubrum_lacusprofundi_ATCC_49239_uid5880	NC_012028	chromosome 2		arCOG08887	3	S		Uncharacterized conserved protein			
222475708	hypothetical protein Hlac_2774	No	0	YES		31788	31940	-		Halorubrum_lacusprofundi_ATCC_49239_uid5880	NC_012028	chromosome 2		arCOG10363	7	V		AbrB-like transcriptional regulator			
222475709	transcriptional regulator TrmB	No	0			32249	32842	+		Halorubrum_lacusprofundi_ATCC_49239_uid5880	NC_012028	chromosome 2		arCOG04498	1 (-)	K		Predicted transcriptional regulator related to sugar-specific regulator TrmB			
===									7	Halorubrum_lacusprofundi_ATCC_49239_uid5880	NC_012028	chromosome 2									
222475736	ATPase	No	0	YES		65457	66848	-		Halorubrum_lacusprofundi_ATCC_49239_uid5880	NC_012028	chromosome 2		arCOG03166	9 (-)	R		AAA+ superfamily ATPase fused to HTH and Rec	1E-29	COG1672	Predicted ATPase (AAA+ superfamily)
222475737	hypothetical protein Hlac_2805	No	0	YES		67016	67699	+		Halorubrum_lacusprofundi_ATCC_49239_uid5880	NC_012028	chromosome 2	clust_149								
222475738	hypothetical protein Hlac_2806	No	0	YES		67872	68195	+		Halorubrum_lacusprofundi_ATCC_49239_uid5880	NC_012028	chromosome 2	clust_323								
222475739	hypothetical protein Hlac_2807	No	0	YES		68261	68623	-		Halorubrum_lacusprofundi_ATCC_49239_uid5880	NC_012028	chromosome 2	clust_45								
222475740	hypothetical protein Hlac_2808	No	0	YES		68807	69655	-		Halorubrum_lacusprofundi_ATCC_49239_uid5880	NC_012028	chromosome 2		arCOG11350	3	S		Uncharacterized conserved protein			
222475741	integrase family protein	No	0	YES		69792	70409	+		Halorubrum_lacusprofundi_ATCC_49239_uid5880	NC_012028	chromosome 2		arCOG01241	31 (-)	L	XerC	Integrase	0.000001	COG4974	Site-specific recombinase XerD
222475742	hypothetical protein Hlac_2810	No	0	YES		70507	71067	-		Halorubrum_lacusprofundi_ATCC_49239_uid5880	NC_012028	chromosome 2		arCOG10631	2	S		Uncharacterized conserved protein			
===									5	Halorubrum_lacusprofundi_ATCC_49239_uid5880	NC_012028	chromosome 2									

	222475826	hypothetical protein Hlac_2903	No	0	YES		158505	158900	+		Halorubrum_lacusprofundi_ATCC_49239_uid5880	NC_012028	chromosome 2									
	222475827	transposase (ISH3)	No	0	YES		158921	160087	+		Halorubrum_lacusprofundi_ATCC_49239_uid5880	NC_012028	chromosome 2		arCOG06160	11 (-)	L		Transposase			
	222475828	putative transposase	No	0	YES		160294	160440	-		Halorubrum_lacusprofundi_ATCC_49239_uid5880	NC_012028	chromosome 2									
	222475829	hypothetical protein Hlac_2906	No	0	YES		161076	161228	-		Halorubrum_lacusprofundi_ATCC_49239_uid5880	NC_012028	chromosome 2									
	222475830	hypothetical protein Hlac_2907	No	0	YES		166677	166952	-		Halorubrum_lacusprofundi_ATCC_49239_uid5880	NC_012028	chromosome 2									
===										8	Halorubrum_lacusprofundi_ATCC_49239_uid5880	NC_012028	chromosome 2									
	222475866	hypothetical protein Hlac_2951	No	0			206860	207072	-		Halorubrum_lacusprofundi_ATCC_49239_uid5880	NC_012028	chromosome 2		arCOG08128	7 (-)	S		Uncharacterized conserved protein			
	222475867	hypothetical protein Hlac_2952	No	0			207276	207665	+		Halorubrum_lacusprofundi_ATCC_49239_uid5880	NC_012028	chromosome 2									
	222475868	hypothetical protein Hlac_2953	No	0			207665	207793	+		Halorubrum_lacusprofundi_ATCC_49239_uid5880	NC_012028	chromosome 2									
	222475869	SNF2/RAD54 family helicase	No	0			207786	208187	+		Halorubrum_lacusprofundi_ATCC_49239_uid5880	NC_012028	chromosome 2									
	222475870	hypothetical protein Hlac_2955	No	0			208177	208404	+		Halorubrum_lacusprofundi_ATCC_49239_uid5880	NC_012028	chromosome 2									
	222475871	hypothetical protein Hlac_2956	No	0			208772	208957	+		Halorubrum_lacusprofundi_ATCC_49239_uid5880	NC_012028	chromosome 2									
	222475872	hypothetical protein Hlac_2957	No	0			208962	209123	+		Halorubrum_lacusprofundi_ATCC_49239_uid5880	NC_012028	chromosome 2									
	222475873	orc1/cdc6 family replication initiation protein	No	0			209219	210445	-		Halorubrum_lacusprofundi_ATCC_49239_uid5880	NC_012028	chromosome 2		arCOG00467	13 (-)	L	CDC6	Cdc6-related protein, AAA superfamily ATPase	7E-76	COG1474	Cdc6-related protein, AAA superfamily AT
===										10	Halorubrum_lacusprofundi_ATCC_49239_uid5880	NC_012028	chromosome 2									
	222476101	hypothetical protein Hlac_3202	No	4	YES		448135	448896	-		Halorubrum_lacusprofundi_ATCC_49239_uid5880	NC_012028	chromosome 2									
	222476102	transposase (ISH3)	No	0	YES		448976	450142	+		Halorubrum_lacusprofundi_ATCC_49239_uid5880	NC_012028	chromosome 2		arCOG06160	11 (-)	L		Transposase			
	222476103	hypothetical protein Hlac_3204	No	0	YES		450283	450468	-		Halorubrum_lacusprofundi_ATCC_49239_uid5880	NC_012028	chromosome 2	clust_470								
	222476104	hypothetical protein Hlac_3205	No	0	YES		450461	450625	-		Halorubrum_lacusprofundi_ATCC_49239_uid5880	NC_012028	chromosome 2		arCOG10363	7	V		AbrB-like transcriptional regulator			
	222476105	hypothetical protein Hlac_3206	No	0	YES		450938	451210	-		Halorubrum_lacusprofundi_ATCC_49239_uid5880	NC_012028	chromosome 2									
	222476106	hypothetical protein Hlac_3207	No	0	YES		451243	451734	-		Halorubrum_lacusprofundi_ATCC_49239_uid5880	NC_012028	chromosome 2	clust_218								
	222476107	hypothetical protein Hlac_3208	No	0	YES		451982	452218	-		Halorubrum_lacusprofundi_ATCC_49239_uid5880	NC_012028	chromosome 2		arCOG09232	3	S		Uncharacterized conserved protein			
	222476108	hypothetical protein Hlac_3209	No	0	YES		452445	452567	-		Halorubrum_lacusprofundi_ATCC_49239_uid5880	NC_012028	chromosome 2									
	222476109	transposase (ISH3)	No	0	YES		452947	454113	+		Halorubrum_lacusprofundi_ATCC_49239_uid5880	NC_012028	chromosome 2		arCOG06160	11 (-)	L		Transposase			
	222476110	hypothetical protein Hlac_3211	No	0	YES		454281	454517	+		Halorubrum_lacusprofundi_ATCC_49239_uid5880	NC_012028	chromosome 2									
===										39	Halorubrum_lacusprofundi_ATCC_49239_uid5880	NC_012029	chromosome 1									
	222479171	hypothetical protein Hlac_0736	No	0		Virus_Caudovi	750926	751066	+		Halorubrum_lacusprofundi_ATCC_49239_uid5880	NC_012029	chromosome 1									
	222479172	hypothetical protein Hlac_0738	No	0		Virus_Caudovi	751252	751764	+		Halorubrum_lacusprofundi_ATCC_49239_uid5880	NC_012029	chromosome 1	clust_206								
	222479173	MCM family protein	No	0		Virus_Caudovi	751915	754068	+		Halorubrum_lacusprofundi_ATCC_49239_uid5880	NC_012029	chromosome 1		arCOG00439	14 (-)	L	MCM2	Predicted ATPase involved in replication control,	2E-117	COG1241	Predicted ATPase involved in replication
	222479174	hypothetical protein Hlac_0741	No	0		Virus_Caudovi	754071	754523	+		Halorubrum_lacusprofundi_ATCC_49239_uid5880	NC_012029	chromosome 1									
	222479175	hypothetical protein Hlac_0742	No	0		Virus_Caudovi	754520	754795	+		Halorubrum_lacusprofundi_ATCC_49239_uid5880	NC_012029	chromosome 1									
	222479176	hypothetical protein Hlac_0743	No	0		Virus_Caudovi	754792	754998	+		Halorubrum_lacusprofundi_ATCC_49239_uid5880	NC_012029	chromosome 1									
	222479177	hypothetical protein Hlac_0744	No	0		Virus_Caudovi	754988	755203	+		Halorubrum_lacusprofundi_ATCC_49239_uid5880	NC_012029	chromosome 1									
	222479178	hypothetical protein Hlac_0745	No	0		Virus_Caudovi	755200	755460	+		Halorubrum_lacusprofundi_ATCC_49239_uid5880	NC_012029	chromosome 1		arCOG00394	7 (-)	K	ArsR	Transcriptional regulator containing HTH domain, ArsR family			
	222479179	hypothetical protein Hlac_0746	No	0		Virus_Caudovi	755605	756090	+		Halorubrum_lacusprofundi_ATCC_49239_uid5880	NC_012029	chromosome 1									
	222479180	hypothetical protein Hlac_0747	No	0		Virus_Caudovi	756087	756266	+		Halorubrum_lacusprofundi_ATCC_49239_uid5880	NC_012029	chromosome 1									
	222479181	hypothetical protein Hlac_0748	No	2		Virus_Caudovi	756755	757066	+		Halorubrum_lacusprofundi_ATCC_49239_uid5880	NC_012029	chromosome 1									
	222479182	hypothetical protein Hlac_0749	No	0		Virus_Caudovi	757066	757227	+		Halorubrum_lacusprofundi_ATCC_49239_uid5880	NC_012029	chromosome 1									
	222479183	hypothetical protein Hlac_0750	No	0		Virus_Caudovi	757296	757685	+		Halorubrum_lacusprofundi_ATCC_49239_uid5880	NC_012029	chromosome 1									
	222479184	hypothetical protein Hlac_0751	No	0		Virus_Caudovi	757682	758020	+		Halorubrum_lacusprofundi_ATCC_49239_uid5880	NC_012029	chromosome 1									
	222479185	hypothetical protein Hlac_0752	No	0		Virus_Caudovi	758017	758412	+		Halorubrum_lacusprofundi_ATCC_49239_uid5880	NC_012029	chromosome 1									
	222479186	hypothetical protein Hlac_0753	No	0		Virus_Caudovi	758501	759301	+		Halorubrum_lacusprofundi_ATCC_49239_uid5880	NC_012029	chromosome 1		arCOG09210	6 (-)	S		Uncharacterized conserved protein	0.000009	COG5484	Uncharacterized conserved protein
	222479187	hypothetical protein Hlac_0754	No	0		Virus_Caudovi	759298	759921	+		Halorubrum_lacusprofundi_ATCC_49239_uid5880	NC_012029	chromosome 1									
	222479188	hypothetical protein Hlac_0755	No	0		Virus_Caudovi	759925	761565	+		Halorubrum_lacusprofundi_ATCC_49239_uid5880	NC_012029	chromosome 1		arCOG09550	17	Y	TerL	Phage terminase large subunit			
	222479189	hypothetical protein Hlac_0756	No	0		Virus_Caudovi	761592	763316	+		Halorubrum_lacusprofundi_ATCC_49239_uid5880	NC_012029	chromosome 1									
	222479190	hypothetical protein Hlac_0757	No	0		Virus_Caudovi	763313	763729	+		Halorubrum_lacusprofundi_ATCC_49239_uid5880	NC_012029	chromosome 1									
	222479191	hypothetical protein Hlac_0758	No	0		Virus_Caudovi	763732	764424	+		Halorubrum_lacusprofundi_ATCC_49239_uid5880	NC_012029	chromosome 1									
	222479192	hypothetical protein Hlac_0759	No	0		Virus_Caudovi	764530	765537	+		Halorubrum_lacusprofundi_ATCC_49239_uid5880	NC_012029	chromosome 1									
	222479193	hypothetical protein Hlac_0760	No	0		Virus_Caudovi	765558	766766	+		Halorubrum_lacusprofundi_ATCC_49239_uid5880	NC_012029	chromosome 1									
	222479194	hypothetical protein Hlac_0761	No	0		Virus_Caudovi	766773	766988	+		Halorubrum_lacusprofundi_ATCC_49239_uid5880	NC_012029	chromosome 1									
	222479195	hypothetical protein Hlac_0762	No	0		Virus_Caudovi	766996	767907	+		Halorubrum_lacusprofundi_ATCC_49239_uid5880	NC_012029	chromosome 1									
	222479196	hypothetical protein Hlac_0763	No	0		Virus_Caudovi	767914	768624	+		Halorubrum_lacusprofundi_ATCC_49239_uid5880	NC_012029	chromosome 1									
	222479197	hypothetical protein Hlac_0764	No	2		Virus_Caudovi	768621	768917	+		Halorubrum_lacusprofundi_ATCC_49239_uid5880	NC_012029	chromosome 1									
	222479198	hypothetical protein Hlac_0765	No	0		Virus_Caudovi	768917	769369	+		Halorubrum_lacusprofundi_ATCC_49239_uid5880	NC_012029	chromosome 1	clust_222								
	222479199	hypothetical protein Hlac_0766	No	0		Virus_Caudovi	769366	769884	+		Halorubrum_lacusprofundi_ATCC_49239_uid5880	NC_012029	chromosome 1									
	222479200	hypothetical protein Hlac_0767	No	0		Virus_Caudovi	769922	770839	+		Halorubrum_lacusprofundi_ATCC_49239_uid5880	NC_012029	chromosome 1	clust_106								
	222479201	hypothetical protein Hlac_0768	No	0		Virus_Caudovi	770839	771351	+		Halorubrum_lacusprofundi_ATCC_49239_uid5880	NC_012029	chromosome 1	clust_207								
	222479202	hypothetical protein Hlac_0769	No	0		Virus_Caudovi	771452	772588	+		Halorubrum_lacusprofundi_ATCC_49239_uid5880	NC_012029	chromosome 1									
	222479203	hypothetical protein Hlac_0770	No	2		Virus_Caudovi	772606	772908	+		Halorubrum_lacusprofundi_ATCC_49239_uid5880	NC_012029	chromosome 1									
	222479204	hypothetical protein Hlac_0771	No	0		Virus_Caudovi	772952	773620	+		Halorubrum_lacusprofundi_ATCC_49239_uid5880	NC_012029	chromosome 1		arCOG10639	4	Y		Phage associated protein			
	222479205	hypothetical protein Hlac_0772	No	0		Virus_Caudovi	773624	774811	+		Halorubrum_lacusprofundi_ATCC_49239_uid5880	NC_012029	chromosome 1		arCOG10640	5	Y		Phage associated protein			
	222479206	Fibronectin type III domain protein	No	0		Virus_Caudovi	774812	776476	+		Halorubrum_lacusprofundi_ATCC_49239_uid5880	NC_012029	chromosome 1							0.00009	pfam00041	Fibronectin type III domain.
	222479207	hypothetical protein Hlac_0774	No	0		Virus_Caudovi	776476	779244	+		Halorubrum_lacusprofundi_ATCC_49239_uid5880	NC_012029	chromosome 1									
	222479208	hypothetical protein Hlac_0775	No	0		Virus_Caudovi	779268	779675	+		Halorubrum_lacusprofundi_ATCC_49239_uid5880	NC_012029	chromosome 1									
	222479209	hypothetical protein Hlac_0776	Yes	1		Virus_Caudovi	780099	781208	-		Halorubrum_lacusprofundi_ATCC_49239_uid5880	NC_012029	chromosome 1		arCOG03053	1 (-)	S		Uncharacterized conserved protein	0.000001	pfam04094	Protein of unknown function (DUF390). Th
===										9	Halorubrum_lacusprofundi_ATCC_49239_uid5880	NC_012029	chromosome 1									
	222480147	hypothetical protein Hlac_1731	No	0			1747325	1747627	-		Halorubrum_lacusprofundi_ATCC_49239_uid5880	NC_012029	chromosome 1									
	222480148	PASTA domain containing protein	No	0			1747624	1748916	-		Halorubrum_lacusprofundi_ATCC_49239_uid5880	NC_012029	chromosome 1		arCOG06234	1 (-)	R		Predicted nucleic acid binding protein, containing	0.000009	COG2815	Uncharacterized protein conserved in bac ; Chromosome segregation ATPases
	222480149	hypothetical protein Hlac_1733	No	0			1748913	1749305	-		Halorubrum_lacusprofundi_ATCC_49239_uid5880	NC_012029	chromosome 1		arCOG08952	2	S		Uncharacterized conserved protein			
	222480150	hypothetical protein Hlac_1734	No	0			1749295	1749870	-		Halorubrum_lacusprofundi_ATCC_49239_uid5880	NC_012029	chromosome 1		arCOG08953	3	S		Uncharacterized conserved protein			
	222480151	hypothetical protein Hlac_1735	No	0			1749948	1750472	-		Halorubrum_lacusprofundi_ATCC_49239_uid5880	NC_012029	chromosome 1		arCOG08953	3	S		Uncharacterized conserved protein			
	222480152	hypothetical protein Hlac_1736	No	0			1750739	1750975	+		Halorubrum_lacusprofundi_ATCC_49239_uid5880	NC_012029	chromosome 1									
	222480153	hypothetical protein Hlac_1738	No	2			1752028	1752279	-		Halorubrum_lacusprofundi_ATCC_49239_uid5880	NC_012029	chromosome 1									
	222480154	xylose isomerase	No	0			1752906	1753721	-		Halorubrum_lacusprofundi_ATCC_49239_uid5880	NC_012029	chromosome 1		arCOG01902	1 (-)	G	IolE	Sugar phosphate isomerase/epimerase	0.000002	pfam01261	Xylose isomerase-like TIM barrel. This T
	222480155	hypothetical protein Hlac_1740	No	0			1754088	1754993	+		Halorubrum_lacusprofundi_ATCC_49239_uid5880	NC_012029	chromosome 1		arCOG10645	1	S		Uncharacterized conserved protein			
===										6	Halorubrum_lacusprofundi_ATCC_49239_uid5880	NC_012029	chromosome 1									
	222480166	N-ethylammeline chlorohydrolase	No	0			1768690	1769217	-		Halorubrum_lacusprofundi_ATCC_49239_uid5880	NC_012029	chromosome 1		arCOG00695	1 (-)	F	SsnA	Cytosine deaminase or related metal-dependent	0.00000005	COG0402	Cytosine deaminase and related metal-dep
	222480167	hypothetical protein Hlac_1752	No	1			1769196	1769309	-		Halorubrum_lacusprofundi_ATCC_49239_uid5880	NC_012029	chromosome 1									
	222480168	hypothetical protein Hlac_1753	No	4			1769344	1769745	-		Halorubrum_lacusprofundi_ATCC_49239_uid5880	NC_012029	chromosome 1		arCOG08954	3	S		Uncharacterized conserved protein			
	222480169	hypothetical protein Hlac_1754	No	0			1769745	1770107	-		Halorubrum_lacusprofundi_ATCC_49239_uid5880	NC_012029	chromosome 1									
	222480170	hypothetical protein Hlac_1755	No	0			1770323	1771552	-		Halorubrum_lacusprofundi_ATCC_49239_uid5880	NC_012029	chromosome 1									
	222480171	transposase (ISH3)	No	0			1773018	1774184	+		Halorubrum_lacusprofundi_ATCC_49239_uid5880	NC_012029	chromosome 1		arCOG06160	11 (-)	L		Transposase			
===										7	Halorubrum_lacusprofundi_ATCC_49239_uid5880	NC_012029	chromosome 1									
	222480770	glycine-rich protein	No	0			2356586	2357056	-		Halorubrum_lacusprofundi_ATCC_49239_uid5880	NC_012029	chromosome 1							2E-10	pfam07172	Glycine rich protein family. This family
	222480771	aldo/keto reductase	No	0			2357213	2358187	-		Halorubrum_lacusprofundi_ATCC_49239_uid5880	NC_012029	chromosome 1		arCOG01617	4 (-)	C	Tas	Predicted oxidoreductase (related to aryl-alcohol	3E-73	COG0667	Predicted oxidoreductases (related to ar
	222480772	hypothetical protein Hlac_2363	No	2			2358253	2358450	-		Halorubrum_lacusprofundi_ATCC_49239_uid5880	NC_012029	chromosome 1		arCOG11344	1	S		Uncharacterized conserved protein			
	222480773	hypothetical protein Hlac_2364	No	0			2358582	2358866	-		Halorubrum_lacusprofundi_ATCC_49239_uid5880	NC_012029	chromosome 1									
	222480774	hypothetical protein Hlac_2366	No	2			2360296	2360541	-		Halorubrum_lacusprofundi_ATCC_49239_uid5880	NC_012029	chromosome 1		arCOG10674	1	S		Uncharacterized conserved protein			
	222480775	hypothetical protein Hlac_2367	No	2			2360633	2360812	-		Halorubrum_lacusprofundi_ATCC_49239_uid5880	NC_012029	chromosome 1									
	222480776	translation initiation factor IF-1A	No	0			2360904	2361194	+		Halorubrum_lacusprofundi_ATCC_49239_uid5880	NC_012029	chromosome 1		arCOG01179	2 (-)	J	InfA	Translation initiation factor 1 (IF-1)	4E-14	pfam01176	Translation initiation factor 1A / IF-1.
===										6	Halorubrum_lacusprofundi_ATCC_49239_uid5880	NC_012029	chromosome 1									
	222481080	hypothetical protein Hlac_2676	No	0			2662895	2664058	+		Halorubrum_lacusprofundi_ATCC_49239_uid5880	NC_012029	chromosome 1		arCOG08160	1 (-)	S		Uncharacterized conserved protein			
	222481081	hypothetical protein Hlac_2677	No	8			2664633	2665502	-		Halorubrum_lacusprofundi_ATCC_49239_uid5880	NC_012029	chromosome 1									
	222481082	hypothetical protein Hlac_2678	No	4			2665738	2666232	+		Halorubrum_lacusprofundi_ATCC_49239_uid5880	NC_012029	chromosome 1									
	222481083	hypothetical protein Hlac_2679	No	3			2666338	2666703	-		Halorubrum_lacusprofundi_ATCC_49239_uid5880	NC_012029	chromosome 1		arCOG08976	1	S		Uncharacterized conserved protein			
	222481084	hypothetical protein Hlac_2680	No	0			2666979	2667137	+		Halorubrum_lacusprofundi_ATCC_49239_uid5880	NC_012029	chromosome 1									
	222481085	hypothetical protein Hlac_2681	No	12			2667252	2669891	-		Halorubrum_lacusprofundi_ATCC_49239_uid5880	NC_012029	chromosome 1		arCOG02174	2 (-)	R		Predicted exporter of the RND superfamily	6E-66	COG1033	Predicted exporters of the RND superfami
===										12	Halorubrum_lacusprofundi_ATCC_49239_uid5880	NC_012030	plasmid pHLAC01									
	222481297	hypothetical protein Hlac_3424	No	0		plasmid	171942	172187	-		Halorubrum_lacusprofundi_ATCC_49239_uid5880	NC_012030	plasmid pHLAC01									
	222481298	hypothetical protein Hlac_3425	No	2		plasmid	172218	172811	-		Halorubrum_lacusprofundi_ATCC_49239_uid5880	NC_012030	plasmid pHLAC01		arCOG06383	1 (-)	K		Predicted transcriptional regulator			
	222481299	hypothetical protein Hlac_3426	No	4		plasmid	172945	173457	+		Halorubrum_lacusprofundi_ATCC_49239_uid5880	NC_012030	plasmid pHLAC01		arCOG08986	1	S		Uncharacterized conserved protein			
	222481300	hypothetical protein Hlac_3427	No	7		plasmid	173646	174458	+		Halorubrum_lacusprofundi_ATCC_49239_uid5880	NC_012030	plasmid pHLAC01		arCOG08987	2	S		Uncharacterized conserved protein			
	222481301	hypothetical protein Hlac_3428	No	0		plasmid	174433	174681	+		Halorubrum_lacusprofundi_ATCC_49239_uid5880	NC_012030	plasmid pHLAC01									
	222481302	hypothetical protein Hlac_3429	No	0		plasmid	174781	175866	-		Halorubrum_lacusprofundi_ATCC_49239_uid5880	NC_012030	plasmid pHLAC01									
	222481303	hypothetical protein Hlac_3430	No	0		plasmid	175868	177028	-		Halorubrum_lacusprofundi_ATCC_49239_uid5880	NC_012030	plasmid pHLAC01		arCOG06119	2 (-)	S		Uncharacterized conserved protein			
	222481304	hypothetical protein Hlac_3431	No	0		plasmid	177254	177349	-		Halorubrum_lacusprofundi_ATCC_49239_uid5880	NC_012030	plasmid pHLAC01									
	222481305	hypothetical protein Hlac_3432	No	0		plasmid	177425	177637	-		Halorubrum_lacusprofundi_ATCC_49239_uid5880	NC_012030	plasmid pHLAC01									
	222481306	hypothetical protein Hlac_3433	No	0		plasmid	177735	178031	+		Halorubrum_lacusprofundi_ATCC_49239_uid5880	NC_012030	plasmid pHLAC01									
	222481307	transposase (ISH3)	No	0		plasmid	178439	179605	+		Halorubrum_lacusprofundi_ATCC_49239_uid5880	NC_012030	plasmid pHLAC01		arCOG06160	11 (-)	L		Transposase			
	222481308	hypothetical protein Hlac_3435	No	0		plasmid	180089	180430	-		Halorubrum_lacusprofundi_ATCC_49239_uid5880	NC_012030	plasmid pHLAC01									
===										17	Halorubrum_lacusprofundi_ATCC_49239_uid5880	NC_012030	plasmid pHLAC01									
	222481362	NAD-dependent epimerase/dehydratase	No	0		plasmid	234331	235278	-		Halorubrum_lacusprofundi_ATCC_49239_uid5880	NC_012030	plasmid pHLAC01		arCOG01369	5 (-)	M	WcaG	Nucleoside-diphosphate-sugar epimerase	9E-58	COG0451	Nucleoside-diphosphate-sugar epimerases
	222481363	hypothetical protein Hlac_3492	No	0		plasmid	235751	235966	+		Halorubrum_lacusprofundi_ATCC_49239_uid5880	NC_012030	plasmid pHLAC01		arCOG08144	1	S		Uncharacterized conserved protein			
	222481364	hypothetical protein Hlac_3494	No	0		plasmid	238473	238973	+		Halorubrum_lacusprofundi_ATCC_49239_uid5880	NC_012030	plasmid pHLAC01									
	222481365	hypothetical protein Hlac_3495	No	0		plasmid	239035	239373	-		Halorubrum_lacusprofundi_ATCC_49239_uid5880	NC_012030	plasmid pHLAC01									
	222481366	hypothetical protein Hlac_3496	No	2		plasmid	239736	240410	-		Halorubrum_lacusprofundi_ATCC_49239_uid5880	NC_012030	plasmid pHLAC01									
	222481367	putative CheA signal transduction histidine kina	No	0	YES	plasmid	241213	243582	-		Halorubrum_lacusprofundi_ATCC_49239_uid5880	NC_012030	plasmid pHLAC01							0.0000004	COG0840	Methyl-accepting chemotaxis protein
	222481368	hypothetical protein Hlac_3498	No	0	YES	plasmid	244094	244669	-		Halorubrum_lacusprofundi_ATCC_49239_uid5880	NC_012030	plasmid pHLAC01									
	222481369	hypothetical protein Hlac_3499	No	0	YES	plasmid	244809	245279	+		Halorubrum_lacusprofundi_ATCC_49239_uid5880	NC_012030	plasmid pHLAC01									
	222481370	hypothetical protein Hlac_3500	No	0	YES	plasmid	246340	246459	+		Halorubrum_lacusprofundi_ATCC_49239_uid5880	NC_012030	plasmid pHLAC01									
	222481371	hypothetical protein Hlac_3501	No	0	YES	plasmid	246663	247166	+		Halorubrum_lacusprofundi_ATCC_49239_uid5880	NC_012030	plasmid pHLAC01									
	222481372	phage PhiH1 repressor protein	No	0	YES	plasmid	248244	248498	+		Halorubrum_lacusprofundi_ATCC_49239_uid5880	NC_012030	plasmid pHLAC01		arCOG03924	10 (-)	K	MarR	Transcriptional regulator, MarR family			
	222481373	hypothetical protein Hlac_3503	Yes	0	YES	plasmid	248637	249122	+		Halorubrum_lacusprofundi_ATCC_49239_uid5880	NC_012030	plasmid pHLAC01									
	222481374	hypothetical protein Hlac_3504	No	0	YES	plasmid	249189	249566	-		Halorubrum_lacusprofundi_ATCC_49239_uid5880	NC_012030	plasmid pHLAC01									
	222481375	hypothetical protein Hlac_3505	No	0	YES	plasmid	249563	249814	-		Halorubrum_lacusprofundi_ATCC_49239_uid5880	NC_012030	plasmid pHLAC01									
	222481376	Resolvase domain protein	No	0	YES	plasmid	249984	250694	-		Halorubrum_lacusprofundi_ATCC_49239_uid5880	NC_012030	plasmid pHLAC01		arCOG03162	12 (-)	L	PinR	Site-specific recombinase, DNA invertase Pin homolog			
	222481377	hypothetical protein Hlac_3507	No	0	YES	plasmid	250687	250881	-		Halorubrum_lacusprofundi_ATCC_49239_uid5880	NC_012030	plasmid pHLAC01		arCOG10363	7	V		AbrB-like transcriptional regulator			
	222481378	hypothetical protein Hlac_3508	No	0	YES	plasmid	251023	251319	+		Halorubrum_lacusprofundi_ATCC_49239_uid5880	NC_012030	plasmid pHLAC01									
===										7	Halorubrum_lacusprofundi_ATCC_49239_uid5880	NC_012030	plasmid pHLAC01									
	222481447	CRISPR-associated protein Cas2	No	0	YES	plasmid	333251	333529	+		Halorubrum_lacusprofundi_ATCC_49239_uid5880	NC_012030	plasmid pHLAC01		arCOG04194	3 (-)	V	Cas2	CRISPR-associated protein Cas2	5E-11	pfam09827	CRISPR associated protein Cas2. Members
	222481448	hypothetical protein Hlac_3580	No	0	YES	plasmid	335497	335985	+		Halorubrum_lacusprofundi_ATCC_49239_uid5880	NC_012030	plasmid pHLAC01									
	222481449	hypothetical protein Hlac_3581	No	0	YES	plasmid	336454	336957	-		Halorubrum_lacusprofundi_ATCC_49239_uid5880	NC_012030	plasmid pHLAC01									
	222481450	hypothetical protein Hlac_3582	No	0	YES	plasmid	337120	337326	-		Halorubrum_lacusprofundi_ATCC_49239_uid5880	NC_012030	plasmid pHLAC01									
	222481451	hypothetical protein Hlac_3583	No	0	YES	plasmid	338533	338694	+		Halorubrum_lacusprofundi_ATCC_49239_uid5880	NC_012030	plasmid pHLAC01	clust_491								
	222481452	hypothetical protein Hlac_3584	No	0	YES	plasmid	338827	338988	+		Halorubrum_lacusprofundi_ATCC_49239_uid5880	NC_012030	plasmid pHLAC01	clust_491								
	222481453	PilT protein domain protein	No	0	YES	plasmid	339519	339908	-		Halorubrum_lacusprofundi_ATCC_49239_uid5880	NC_012030	plasmid pHLAC01		arCOG02219	4 (-)	V	VapC	PIN domain containing protein	1E-11	COG1487	Predicted nucleic acid-binding protein,
===										5	Haloterrigena_turkmenica_DSM_5511_uid43501	NC_013743	chromosome									
	284163326	hypothetical protein Htur_0031	No	6			31876	32583	-		Haloterrigena_turkmenica_DSM_5511_uid43501	NC_013743	chromosome									
	284163327	hypothetical protein Htur_0032	Yes	0			32580	33260	-		Haloterrigena_turkmenica_DSM_5511_uid43501	NC_013743	chromosome									
	284163328	alanyl-tRNA synthetase	No	0			33503	36280	+		Haloterrigena_turkmenica_DSM_5511_uid43501	NC_013743	chromosome		arCOG01255	1 (-)	J	AlaS	Alanyl-tRNA synthetase	8E-138	COG0013	Alanyl-tRNA synthetase
	284163329	hypothetical protein Htur_0034	No	0			36327	36647	-		Haloterrigena_turkmenica_DSM_5511_uid43501	NC_013743	chromosome									
	284163330	hypothetical protein Htur_0035	No	0			36881	37573	-		Haloterrigena_turkmenica_DSM_5511_uid43501	NC_013743	chromosome									
===										6	Haloterrigena_turkmenica_DSM_5511_uid43501	NC_013743	chromosome									
	284163735	hypothetical protein Htur_0443	No	0			467913	468263	-		Haloterrigena_turkmenica_DSM_5511_uid43501	NC_013743	chromosome									
	284163736	hypothetical protein Htur_0444	No	0			468378	468554	-		Haloterrigena_turkmenica_DSM_5511_uid43501	NC_013743	chromosome									
	284163737	hypothetical protein Htur_0445	No	0			469743	469877	-		Haloterrigena_turkmenica_DSM_5511_uid43501	NC_013743	chromosome									
	284163738	hypothetical protein Htur_0446	No	0			470109	470555	-		Haloterrigena_turkmenica_DSM_5511_uid43501	NC_013743	chromosome		arCOG03828	5 (-)	S		Uncharacterized conserved protein			
	284163739	hypothetical protein Htur_0447	No	3			471361	471945	-		Haloterrigena_turkmenica_DSM_5511_uid43501	NC_013743	chromosome									
	284163740	hypothetical protein Htur_0448	No	0			472167	472286	+		Haloterrigena_turkmenica_DSM_5511_uid43501	NC_013743	chromosome									
===										6	Haloterrigena_turkmenica_DSM_5511_uid43501	NC_013743	chromosome									
	284164550	peptide ABC transporter periplasmic protein	No	0			1325332	1327209	+		Haloterrigena_turkmenica_DSM_5511_uid43501	NC_013743	chromosome		arCOG01534	2 (-)	E	DdpA	ABC-type transport system, periplasmic compone	2E-15	pfam00496	Bacterial extracellular solute-binding p
	284164551	hypothetical protein Htur_1267	No	0			1327458	1327985	-		Haloterrigena_turkmenica_DSM_5511_uid43501	NC_013743	chromosome									
	284164552	hypothetical protein Htur_1268	No	1			1328177	1328953	-		Haloterrigena_turkmenica_DSM_5511_uid43501	NC_013743	chromosome									
	284164553	Curlin associated repeat protein	Yes	1			1329051	1329788	-		Haloterrigena_turkmenica_DSM_5511_uid43501	NC_013743	chromosome							0.00003	COG5271	AAA ATPase containing von Willebrand fac
	284164554	hypothetical protein Htur_1270	No	0			1330300	1330392	-		Haloterrigena_turkmenica_DSM_5511_uid43501	NC_013743	chromosome									
	284164555	hypothetical protein Htur_1271	Yes	1			1330493	1331173	+		Haloterrigena_turkmenica_DSM_5511_uid43501	NC_013743	chromosome		arCOG02858	1 (-)	R		Membrane associated OB-fold protein			
===										5	Haloterrigena_turkmenica_DSM_5511_uid43501	NC_013743	chromosome									
	284165191	hypothetical protein Htur_1912	No	0			1986433	1986633	-		Haloterrigena_turkmenica_DSM_5511_uid43501	NC_013743	chromosome									
	284165192	ATPase AAA	No	0			1986745	1988238	-		Haloterrigena_turkmenica_DSM_5511_uid43501	NC_013743	chromosome		arCOG00470	1 (-)	L	HolB	ATPase involved in DNA replication HolB, large s	8E-10	COG2812	DNA polymerase III, gamma/tau subunits
	284165193	hypothetical protein Htur_1914	No	6			1988367	1989023	-		Haloterrigena_turkmenica_DSM_5511_uid43501	NC_013743	chromosome									
	284165194	hypothetical protein Htur_1915	No	6			1989025	1989669	-		Haloterrigena_turkmenica_DSM_5511_uid43501	NC_013743	chromosome									
	284165195	hypothetical protein Htur_1916	No	0			1989998	1990828	+		Haloterrigena_turkmenica_DSM_5511_uid43501	NC_013743	chromosome									
===										6	Haloterrigena_turkmenica_DSM_5511_uid43501	NC_013744	plasmid pHTUR01									
	284167218	filamentation induced by cAMP protein Fic	No	0	YES	plasmid	231932	233089	-		Haloterrigena_turkmenica_DSM_5511_uid43501	NC_013744	plasmid pHTUR01		arCOG03110	5 (-)	K		Predicted transciptional regulator, contains Fic ar	4E-39	COG3177	Fic family protein
	284167219	hypothetical protein Htur_3973	No	0	YES	plasmid	233316	233591	+		Haloterrigena_turkmenica_DSM_5511_uid43501	NC_013744	plasmid pHTUR01	clust_51								
	284167220	hypothetical protein Htur_3974	No	0	YES	plasmid	233592	234080	+		Haloterrigena_turkmenica_DSM_5511_uid43501	NC_013744	plasmid pHTUR01		arCOG09038	6	Y		VP7/VP16-like Major capsid protein			
	284167221	hypothetical protein Htur_3975	No	0	YES	plasmid	234234	234824	-		Haloterrigena_turkmenica_DSM_5511_uid43501	NC_013744	plasmid pHTUR01									
	284167222	hypothetical protein Htur_3976	No	0	YES	plasmid	234821	234928	-		Haloterrigena_turkmenica_DSM_5511_uid43501	NC_013744	plasmid pHTUR01									
	284167223	hypothetical protein Htur_3978	No	0	YES	plasmid	235622	236047	+		Haloterrigena_turkmenica_DSM_5511_uid43501	NC_013744	plasmid pHTUR01		arCOG06154	1 (-)	S		Uncharacterized conserved protein			
===										7	Haloterrigena_turkmenica_DSM_5511_uid43501	NC_013745	plasmid pHTUR02									
	284172680	asparagine synthase	No	0		plasmid	181971	183893	-		Haloterrigena_turkmenica_DSM_5511_uid43501	NC_013745	plasmid pHTUR02		arCOG00121	3 (-)	E	AsnB	Asparagine synthase (glutamine-hydrolyzing)	4E-10	COG0367	Asparagine synthase (glutamine-hydrolyzi
	284172681	hypothetical protein Htur_4602	No	0		plasmid	184888	185205	+		Haloterrigena_turkmenica_DSM_5511_uid43501	NC_013745	plasmid pHTUR02									
	284172682	hypothetical protein Htur_4603	No	0		plasmid	185529	185780	-		Haloterrigena_turkmenica_DSM_5511_uid43501	NC_013745	plasmid pHTUR02									
	284172683	hypothetical protein Htur_4604	No	0		plasmid	185868	186392	+		Haloterrigena_turkmenica_DSM_5511_uid43501	NC_013745	plasmid pHTUR02									
	284172684	hypothetical protein Htur_4605	No	0		plasmid	186504	186653	+		Haloterrigena_turkmenica_DSM_5511_uid43501	NC_013745	plasmid pHTUR02									
	284172685	hypothetical protein Htur_4606	No	0		plasmid	186718	187458	-		Haloterrigena_turkmenica_DSM_5511_uid43501	NC_013745	plasmid pHTUR02		arCOG09161	1 (-)	S		Uncharacterized conserved protein			
	284172686	hypothetical protein Htur_4607	No	0		plasmid	187837	188307	-		Haloterrigena_turkmenica_DSM_5511_uid43501	NC_013745	plasmid pHTUR02									
===										7	Haloterrigena_turkmenica_DSM_5511_uid43501	NC_013745	plasmid pHTUR02									
	284172702	membrane-flanked domain protein	No	6		plasmid	203108	204511	+		Haloterrigena_turkmenica_DSM_5511_uid43501	NC_013745	plasmid pHTUR02		arCOG04619	1 (-)	S		Predicted membrane protein	8E-49	COG3428	Predicted membrane protein
	284172703	hypothetical protein Htur_4626	No	0		plasmid	205772	205951	+		Haloterrigena_turkmenica_DSM_5511_uid43501	NC_013745	plasmid pHTUR02									
	284172704	hypothetical protein Htur_4627	No	0		plasmid	206366	206488	+		Haloterrigena_turkmenica_DSM_5511_uid43501	NC_013745	plasmid pHTUR02									
	284172705	hypothetical protein Htur_4628	No	0		plasmid	207942	208103	+		Haloterrigena_turkmenica_DSM_5511_uid43501	NC_013745	plasmid pHTUR02									
	284172706	hypothetical protein Htur_4629	No	0		plasmid	208208	208633	+		Haloterrigena_turkmenica_DSM_5511_uid43501	NC_013745	plasmid pHTUR02									
	284172707	peptidase S8 and S53 subtilisin kexin sedolisi	No	1		plasmid	208675	212892	-		Haloterrigena_turkmenica_DSM_5511_uid43501	NC_013745	plasmid pHTUR02		arCOG00702	6 (-)	O	AprE	Subtilisin-like serine protease	1E-27	pfam00082	Subtilase family. Subtilases are a famil ; FOG: PKD repeat
	284172708	hypothetical protein Htur_4631	No	1		plasmid	213251	213421	+		Haloterrigena_turkmenica_DSM_5511_uid43501	NC_013745	plasmid pHTUR02									
===										6	Haloterrigena_turkmenica_DSM_5511_uid43501	NC_013745	plasmid pHTUR02									
	284172717	Cystathionine gamma-lyase	No	0		plasmid	220340	221524	-		Haloterrigena_turkmenica_DSM_5511_uid43501	NC_013745	plasmid pHTUR02		arCOG00060	1 (-)	E	MetC	Cystathionine beta-lyase/cystathionine gamma-sy	2E-115	pfam01053	Cys/Met metabolism PLP-dependent enzyme.
	284172718	hypothetical protein Htur_4641	No	0		plasmid	221735	222055	+		Haloterrigena_turkmenica_DSM_5511_uid43501	NC_013745	plasmid pHTUR02									
	284172719	hypothetical protein Htur_4642	Yes	0		plasmid	223271	223471	+		Haloterrigena_turkmenica_DSM_5511_uid43501	NC_013745	plasmid pHTUR02									
	284172720	hypothetical protein Htur_4643	No	0		plasmid	224885	225037	+		Haloterrigena_turkmenica_DSM_5511_uid43501	NC_013745	plasmid pHTUR02									
	284172721	hypothetical protein Htur_4644	No	0		plasmid	225032	225265	-		Haloterrigena_turkmenica_DSM_5511_uid43501	NC_013745	plasmid pHTUR02									
	284172722	sulfatase	No	0		plasmid	225680	227065	-		Haloterrigena_turkmenica_DSM_5511_uid43501	NC_013745	plasmid pHTUR02		arCOG02785	3 (-)	P	AslA	Arylsulfatase A or related enzyme	2E-38	COG3119	Arylsulfatase A and related enzymes
===										7	Haloterrigena_turkmenica_DSM_5511_uid43501	NC_013746	plasmid pHTUR03									
	284172927	hypothetical protein Htur_4887	Yes	0		plasmid	78423	78962	-		Haloterrigena_turkmenica_DSM_5511_uid43501	NC_013746	plasmid pHTUR03									
	284172928	Kinetochore-Ndc80 complex, subunit Spc25	No	0		plasmid	81245	82132	-		Haloterrigena_turkmenica_DSM_5511_uid43501	NC_013746	plasmid pHTUR03		arCOG00373	1 (-)	L		DNA sulfur modification protein DndD, ATPase	9E-21	COG1196	Chromosome segregation ATPases
	284172929	hypothetical protein Htur_4891	No	0		plasmid	83056	83586	-		Haloterrigena_turkmenica_DSM_5511_uid43501	NC_013746	plasmid pHTUR03									
	284172930	hypothetical protein Htur_4892	No	0		plasmid	84280	84465	-		Haloterrigena_turkmenica_DSM_5511_uid43501	NC_013746	plasmid pHTUR03									
	284172931	hypothetical protein Htur_4893	No	0		plasmid	84870	85100	-		Haloterrigena_turkmenica_DSM_5511_uid43501	NC_013746	plasmid pHTUR03									
	284172932	hypothetical protein Htur_4894	No	0		plasmid	85263	85376	-		Haloterrigena_turkmenica_DSM_5511_uid43501	NC_013746	plasmid pHTUR03									
	284172933	hypothetical protein Htur_4895	No	0		plasmid	86354	86668	-		Haloterrigena_turkmenica_DSM_5511_uid43501	NC_013746	plasmid pHTUR03		arCOG08980	2 (-)	S		Uncharacterized conserved protein			
===										6	Haloterrigena_turkmenica_DSM_5511_uid43501	NC_013746	plasmid pHTUR03									
	284172958	hypothetical protein Htur_4929	No	0	YES	plasmid	111696	112166	+		Haloterrigena_turkmenica_DSM_5511_uid43501	NC_013746	plasmid pHTUR03		arCOG02801	1 (-)	S		Uncharacterized conserved protein			
	284172959	hypothetical protein Htur_4930	No	0	YES	plasmid	112229	112684	+		Haloterrigena_turkmenica_DSM_5511_uid43501	NC_013746	plasmid pHTUR03									
	284172960	hypothetical protein Htur_4931	No	0	YES	plasmid	113346	113492	-		Haloterrigena_turkmenica_DSM_5511_uid43501	NC_013746	plasmid pHTUR03									
	284172961	hypothetical protein Htur_4932	No	0	YES	plasmid	113633	113821	+		Haloterrigena_turkmenica_DSM_5511_uid43501	NC_013746	plasmid pHTUR03									
	284172962	hypothetical protein Htur_4933	No	0	YES	plasmid	114363	114746	-		Haloterrigena_turkmenica_DSM_5511_uid43501	NC_013746	plasmid pHTUR03									
	284172963	hypothetical protein Htur_4934	No	0	YES	plasmid	115214	116146	-		Haloterrigena_turkmenica_DSM_5511_uid43501	NC_013746	plasmid pHTUR03		arCOG07546	3 (-)	E		Zincin superfamily protease			
===										16	Haloterrigena_turkmenica_DSM_5511_uid43501	NC_013746	plasmid pHTUR03									
	284172965	hypothetical protein Htur_4937	No	0	YES	plasmid	116721	117752	-		Haloterrigena_turkmenica_DSM_5511_uid43501	NC_013746	plasmid pHTUR03		arCOG07544	3 (-)	S		Zn-finger protein			
	284172966	hypothetical protein Htur_4938	No	0	YES	plasmid	118060	118395	-		Haloterrigena_turkmenica_DSM_5511_uid43501	NC_013746	plasmid pHTUR03									
	284172967	hypothetical protein Htur_4939	No	0	YES	plasmid	118742	119020	+		Haloterrigena_turkmenica_DSM_5511_uid43501	NC_013746	plasmid pHTUR03									
	284172968	hypothetical protein Htur_4940	No	0	YES	plasmid	119071	119454	+		Haloterrigena_turkmenica_DSM_5511_uid43501	NC_013746	plasmid pHTUR03									
	284172969	hypothetical protein Htur_4941	No	0	YES	plasmid	119899	120216	-		Haloterrigena_turkmenica_DSM_5511_uid43501	NC_013746	plasmid pHTUR03		arCOG10874	2	S		Uncharacterized conserved protein			
	284172970	hypothetical protein Htur_4942	No	0	YES	plasmid	120603	120758	-		Haloterrigena_turkmenica_DSM_5511_uid43501	NC_013746	plasmid pHTUR03									
	284172971	hypothetical protein Htur_4943	No	0	YES	plasmid	120881	121135	-		Haloterrigena_turkmenica_DSM_5511_uid43501	NC_013746	plasmid pHTUR03		arCOG08980	2 (-)	S		Uncharacterized conserved protein			
	284172972	hypothetical protein Htur_4944	No	0	YES	plasmid	121125	121286	-		Haloterrigena_turkmenica_DSM_5511_uid43501	NC_013746	plasmid pHTUR03		arCOG06449	1 (-)	S		Uncharacterized conserved protein			
	284172973	hypothetical protein Htur_4945	No	0	YES	plasmid	121289	121465	-		Haloterrigena_turkmenica_DSM_5511_uid43501	NC_013746	plasmid pHTUR03									
	284172974	hypothetical protein Htur_4946	No	0	YES	plasmid	121465	121785	-		Haloterrigena_turkmenica_DSM_5511_uid43501	NC_013746	plasmid pHTUR03									
	284172975	hypothetical protein Htur_4947	No	0	YES	plasmid	121886	122272	-		Haloterrigena_turkmenica_DSM_5511_uid43501	NC_013746	plasmid pHTUR03									
	284172976	hypothetical protein Htur_4948	No	0	YES	plasmid	122272	122616	-		Haloterrigena_turkmenica_DSM_5511_uid43501	NC_013746	plasmid pHTUR03									
	284172977	hypothetical protein Htur_4949	No	0	YES	plasmid	122616	123080	-		Haloterrigena_turkmenica_DSM_5511_uid43501	NC_013746	plasmid pHTUR03	clust_224								
	284172978	hypothetical protein Htur_4950	No	0	YES	plasmid	123077	123718	-		Haloterrigena_turkmenica_DSM_5511_uid43501	NC_013746	plasmid pHTUR03									
	284172979	methyltransferase type 12	No	0		plasmid	124083	124697	+		Haloterrigena_turkmenica_DSM_5511_uid43501	NC_013746	plasmid pHTUR03		arCOG03570	1 (-)	Q		SAM-dependent methyltransferase	7E-16	pfam03848	Tellurite resistance protein TehB.
	284172980	hypothetical protein Htur_4952	No	0	YES	plasmid	125103	125483	-		Haloterrigena_turkmenica_DSM_5511_uid43501	NC_013746	plasmid pHTUR03		arCOG09468	2	S		Uncharacterized conserved protein, DUF1428	4E-27	COG5507	Uncharacterized conserved protein
===										25	Haloterrigena_turkmenica_DSM_5511_uid43501	NC_013748	plasmid pHTUR05									
	284176298	hypothetical protein Htur_5013	No	0		plasmid	3425	3604	-		Haloterrigena_turkmenica_DSM_5511_uid43501	NC_013748	plasmid pHTUR05									
	284176299	hypothetical protein Htur_5014	No	0		plasmid	3635	4306	-		Haloterrigena_turkmenica_DSM_5511_uid43501	NC_013748	plasmid pHTUR05		arCOG01400	2 (-)	Q		SAM-dependent methyltransferase			
	284176300	hypothetical protein Htur_5015	No	0		plasmid	4417	4617	-		Haloterrigena_turkmenica_DSM_5511_uid43501	NC_013748	plasmid pHTUR05									
	284176301	hypothetical protein Htur_5016	No	0		plasmid	4941	5537	+		Haloterrigena_turkmenica_DSM_5511_uid43501	NC_013748	plasmid pHTUR05									
	284176302	hypothetical protein Htur_5017	Yes	2		plasmid	5547	8963	+		Haloterrigena_turkmenica_DSM_5511_uid43501	NC_013748	plasmid pHTUR05	clust_64								
	284176303	peptidase S8 and S53 subtilisin kexin sedolisi	No	0		plasmid	8968	10521	+		Haloterrigena_turkmenica_DSM_5511_uid43501	NC_013748	plasmid pHTUR05		arCOG00702	6 (-)	O	AprE	Subtilisin-like serine protease	2E-20	COG1404	Subtilisin-like serine proteases
	284176304	hypothetical protein Htur_5019	No	0		plasmid	10525	11331	+		Haloterrigena_turkmenica_DSM_5511_uid43501	NC_013748	plasmid pHTUR05	clust_119								
	284176305	hypothetical protein Htur_5020	Yes	0		plasmid	11341	12393	+		Haloterrigena_turkmenica_DSM_5511_uid43501	NC_013748	plasmid pHTUR05	clust_89								
	284176306	hypothetical protein Htur_5021	No	0		plasmid	12399	13223	+		Haloterrigena_turkmenica_DSM_5511_uid43501	NC_013748	plasmid pHTUR05		arCOG10931	2	S		Uncharacterized conserved protein			
	284176307	hypothetical protein Htur_5022	No	0		plasmid	13327	14592	+		Haloterrigena_turkmenica_DSM_5511_uid43501	NC_013748	plasmid pHTUR05							0.00002	pfam13229	Right handed beta helix region. This reg
	284176308	hypothetical protein Htur_5023	Yes	2		plasmid	14734	15852	+		Haloterrigena_turkmenica_DSM_5511_uid43501	NC_013748	plasmid pHTUR05									
	284176309	hypothetical protein Htur_5024	No	0		plasmid	15928	16335	+		Haloterrigena_turkmenica_DSM_5511_uid43501	NC_013748	plasmid pHTUR05									
	284176310	hypothetical protein Htur_5025	No	2		plasmid	16336	16587	+		Haloterrigena_turkmenica_DSM_5511_uid43501	NC_013748	plasmid pHTUR05									
	284176311	hypothetical protein Htur_5026	Yes	0		plasmid	16751	17440	+		Haloterrigena_turkmenica_DSM_5511_uid43501	NC_013748	plasmid pHTUR05	clust_143								
	284176312	hypothetical protein Htur_5027	No	0		plasmid	17440	17637	+		Haloterrigena_turkmenica_DSM_5511_uid43501	NC_013748	plasmid pHTUR05									
	284176313	hypothetical protein Htur_5028	No	3		plasmid	17640	18086	+		Haloterrigena_turkmenica_DSM_5511_uid43501	NC_013748	plasmid pHTUR05	clust_234								
	284176314	hypothetical protein Htur_5029	Yes	1		plasmid	18074	19048	+		Haloterrigena_turkmenica_DSM_5511_uid43501	NC_013748	plasmid pHTUR05	clust_98								
	284176315	hypothetical protein Htur_5030	No	1		plasmid	19045	19281	+		Haloterrigena_turkmenica_DSM_5511_uid43501	NC_013748	plasmid pHTUR05	clust_404								
	284176316	hypothetical protein Htur_5031	Yes	3		plasmid	19278	19574	+		Haloterrigena_turkmenica_DSM_5511_uid43501	NC_013748	plasmid pHTUR05	clust_346								
	284176317	hypothetical protein Htur_5032	No	0		plasmid	19735	19950	+		Haloterrigena_turkmenica_DSM_5511_uid43501	NC_013748	plasmid pHTUR05	clust_434								
	284176318	hypothetical protein Htur_5033	No	0		plasmid	19947	20528	+		Haloterrigena_turkmenica_DSM_5511_uid43501	NC_013748	plasmid pHTUR05	clust_179								
	284176319	hypothetical protein Htur_5034	No	3		plasmid	20525	21550	+		Haloterrigena_turkmenica_DSM_5511_uid43501	NC_013748	plasmid pHTUR05	clust_94								
	284176320	hypothetical protein Htur_5035	Yes	2		plasmid	21550	22806	+		Haloterrigena_turkmenica_DSM_5511_uid43501	NC_013748	plasmid pHTUR05									
	284176321	hypothetical protein Htur_5036	No	5		plasmid	22809	24488	+		Haloterrigena_turkmenica_DSM_5511_uid43501	NC_013748	plasmid pHTUR05	clust_72								
	284176322	hypothetical protein Htur_5037	No	0		plasmid	24498	26849	+		Haloterrigena_turkmenica_DSM_5511_uid43501	NC_013748	plasmid pHTUR05		arCOG00280	21 (-)	L		HerA helicase			
===										21	Haloterrigena_turkmenica_DSM_5511_uid43501	NC_013748	plasmid pHTUR05									
	284176330	integrase family protein	No	0	YES	plasmid	37066	38175	-		Haloterrigena_turkmenica_DSM_5511_uid43501	NC_013748	plasmid pHTUR05		arCOG01248	1 (-)	L	XerC	Integrase	1E-12	COG4974	Site-specific recombinase XerD
	284176331	hypothetical protein Htur_5047	No	0	YES	plasmid	38172	38417	-		Haloterrigena_turkmenica_DSM_5511_uid43501	NC_013748	plasmid pHTUR05									
	284176332	hypothetical protein Htur_5048	No	0	YES	plasmid	38667	39044	-		Haloterrigena_turkmenica_DSM_5511_uid43501	NC_013748	plasmid pHTUR05		arCOG11318	1	S		Uncharacterized conserved protein			
	284176333	hypothetical protein Htur_5049	No	0	YES	plasmid	39148	39384	-		Haloterrigena_turkmenica_DSM_5511_uid43501	NC_013748	plasmid pHTUR05		arCOG11163	4	S		Uncharacterized conserved protein			
	284176334	hypothetical protein Htur_5050	No	0	YES	plasmid	39362	39634	-		Haloterrigena_turkmenica_DSM_5511_uid43501	NC_013748	plasmid pHTUR05	clust_369								
	284176335	hypothetical protein Htur_5051	No	0	YES	plasmid	39674	39853	-		Haloterrigena_turkmenica_DSM_5511_uid43501	NC_013748	plasmid pHTUR05									
	284176336	hypothetical protein Htur_5052	No	0	YES	plasmid	39893	40099	-		Haloterrigena_turkmenica_DSM_5511_uid43501	NC_013748	plasmid pHTUR05									
	284176337	hypothetical protein Htur_5053	No	2	YES	plasmid	40096	40428	-		Haloterrigena_turkmenica_DSM_5511_uid43501	NC_013748	plasmid pHTUR05									
	284176338	hypothetical protein Htur_5054	No	0	YES	plasmid	40609	40899	+		Haloterrigena_turkmenica_DSM_5511_uid43501	NC_013748	plasmid pHTUR05									
	284176339	hypothetical protein Htur_5055	No	0	YES	plasmid	40900	41409	+		Haloterrigena_turkmenica_DSM_5511_uid43501	NC_013748	plasmid pHTUR05									
	284176340	hypothetical protein Htur_5056	No	0	YES	plasmid	41402	41977	+		Haloterrigena_turkmenica_DSM_5511_uid43501	NC_013748	plasmid pHTUR05									
	284176341	hypothetical protein Htur_5057	No	0	YES	plasmid	41970	42380	+		Haloterrigena_turkmenica_DSM_5511_uid43501	NC_013748	plasmid pHTUR05									
	284176342	hypothetical protein Htur_5058	No	0	YES	plasmid	42370	42558	+		Haloterrigena_turkmenica_DSM_5511_uid43501	NC_013748	plasmid pHTUR05									
	284176343	hypothetical protein Htur_5059	No	0	YES	plasmid	42555	42809	+		Haloterrigena_turkmenica_DSM_5511_uid43501	NC_013748	plasmid pHTUR05	clust_392								
	284176344	hypothetical protein Htur_5060	No	0	YES	plasmid	42802	43185	+		Haloterrigena_turkmenica_DSM_5511_uid43501	NC_013748	plasmid pHTUR05									
	284176345	hypothetical protein Htur_5061	No	2	YES	plasmid	43182	43445	+		Haloterrigena_turkmenica_DSM_5511_uid43501	NC_013748	plasmid pHTUR05	clust_375								
	284176346	hypothetical protein Htur_5062	No	0	YES	plasmid	43442	43684	+		Haloterrigena_turkmenica_DSM_5511_uid43501	NC_013748	plasmid pHTUR05									
	284176347	hypothetical protein Htur_5063	No	0	YES	plasmid	43677	43859	+		Haloterrigena_turkmenica_DSM_5511_uid43501	NC_013748	plasmid pHTUR05	clust_474								
	284176348	hypothetical protein Htur_5064	No	0	YES	plasmid	43856	44398	+		Haloterrigena_turkmenica_DSM_5511_uid43501	NC_013748	plasmid pHTUR05									
	284176349	hypothetical protein Htur_5065	No	0	YES	plasmid	44954	45160	-		Haloterrigena_turkmenica_DSM_5511_uid43501	NC_013748	plasmid pHTUR05									
	284176350	hypothetical protein Htur_5066	No	0		plasmid	45157	45780	-		Haloterrigena_turkmenica_DSM_5511_uid43501	NC_013748	plasmid pHTUR05		arCOG00115	10 (-)	L		DNA modification methylase			
===										21	Haloterrigena_turkmenica_DSM_5511_uid43501	NC_013748	plasmid pHTUR05									
	284176353	ATPase involved in chromosome partitioning-li	No	0		plasmid	47065	47922	-		Haloterrigena_turkmenica_DSM_5511_uid43501	NC_013748	plasmid pHTUR05		arCOG00586	8 (-)	D	Soj	ATPase involved in chromosome partitioning, Pa	4E-11	COG1192	ATPases involved in chromosome partition
	284176354	hypothetical protein Htur_5070	No	0		plasmid	48411	49217	-		Haloterrigena_turkmenica_DSM_5511_uid43501	NC_013748	plasmid pHTUR05									
	284176355	hypothetical protein Htur_5071	No	0		plasmid	49210	49416	-		Haloterrigena_turkmenica_DSM_5511_uid43501	NC_013748	plasmid pHTUR05									
	284176356	hypothetical protein Htur_5072	No	0		plasmid	49413	49805	-		Haloterrigena_turkmenica_DSM_5511_uid43501	NC_013748	plasmid pHTUR05									
	284176357	hypothetical protein Htur_5073	No	0		plasmid	49802	49984	-		Haloterrigena_turkmenica_DSM_5511_uid43501	NC_013748	plasmid pHTUR05									
	284176358	hypothetical protein Htur_5074	No	0		plasmid	49977	50156	-		Haloterrigena_turkmenica_DSM_5511_uid43501	NC_013748	plasmid pHTUR05									
	284176359	hypothetical protein Htur_5075	No	0		plasmid	50153	50263	-		Haloterrigena_turkmenica_DSM_5511_uid43501	NC_013748	plasmid pHTUR05									
	284176360	hypothetical protein Htur_5076	No	0		plasmid	50264	50515	-		Haloterrigena_turkmenica_DSM_5511_uid43501	NC_013748	plasmid pHTUR05									
	284176361	hypothetical protein Htur_5077	No	0		plasmid	50512	50985	-		Haloterrigena_turkmenica_DSM_5511_uid43501	NC_013748	plasmid pHTUR05									
	284176362	hypothetical protein Htur_5078	No	0		plasmid	50985	51569	-		Haloterrigena_turkmenica_DSM_5511_uid43501	NC_013748	plasmid pHTUR05									
	284176363	transcriptional regulator, PadR-like family	No	0		plasmid	51569	51886	-		Haloterrigena_turkmenica_DSM_5511_uid43501	NC_013748	plasmid pHTUR05		arCOG00005	4 (-)	K		Predicted transcriptional regulator, PadR family			
	284176364	hypothetical protein Htur_5080	No	0		plasmid	52004	52219	-		Haloterrigena_turkmenica_DSM_5511_uid43501	NC_013748	plasmid pHTUR05									
	284176365	heat shock protein DnaJ domain protein	No	0		plasmid	52216	52806	-		Haloterrigena_turkmenica_DSM_5511_uid43501	NC_013748	plasmid pHTUR05		arCOG02845	1 (-)	C	Fdx	DnaJ-like chaperone fused to ferredoxin			
	284176366	hypothetical protein Htur_5082	No	0		plasmid	52803	53288	-		Haloterrigena_turkmenica_DSM_5511_uid43501	NC_013748	plasmid pHTUR05									
	284176367	hypothetical protein Htur_5083	No	1		plasmid	53285	53440	-		Haloterrigena_turkmenica_DSM_5511_uid43501	NC_013748	plasmid pHTUR05									
	284176368	hypothetical protein Htur_5084	No	0		plasmid	53437	53769	-		Haloterrigena_turkmenica_DSM_5511_uid43501	NC_013748	plasmid pHTUR05									
	284176369	hypothetical protein Htur_5085	No	0		plasmid	53766	54176	-		Haloterrigena_turkmenica_DSM_5511_uid43501	NC_013748	plasmid pHTUR05									
	284176370	hypothetical protein Htur_5086	No	0		plasmid	54173	54277	-		Haloterrigena_turkmenica_DSM_5511_uid43501	NC_013748	plasmid pHTUR05									
	284176371	hypothetical protein Htur_5087	No	0		plasmid	54274	54906	-		Haloterrigena_turkmenica_DSM_5511_uid43501	NC_013748	plasmid pHTUR05									
	284176372	hypothetical protein Htur_5088	No	0		plasmid	54903	55448	-		Haloterrigena_turkmenica_DSM_5511_uid43501	NC_013748	plasmid pHTUR05	clust_193								
	284176373	hypothetical protein Htur_5089	No	0		plasmid	55445	55765	-		Haloterrigena_turkmenica_DSM_5511_uid43501	NC_013748	plasmid pHTUR05		arCOG03067	3 (-)	K	ArsR	Transcriptional regulator containing HTH domain, ArsR family			
===										7	Haloterrigena_turkmenica_DSM_5511_uid43501	NC_013749	plasmid pHTUR06									
	284176393	hypothetical protein Htur_5267	No	0	YES	plasmid	474	815	+		Haloterrigena_turkmenica_DSM_5511_uid43501	NC_013749	plasmid pHTUR06		arCOG08120	1 (-)	K		Predicted transcriptional regulator			
	284176394	hypothetical protein Htur_5268	No	0	YES	plasmid	865	1179	+		Haloterrigena_turkmenica_DSM_5511_uid43501	NC_013749	plasmid pHTUR06									
	284176395	hypothetical protein Htur_5269	No	1	YES	plasmid	1261	1488	-		Haloterrigena_turkmenica_DSM_5511_uid43501	NC_013749	plasmid pHTUR06									
	284176396	hypothetical protein Htur_5270	No	0	YES	plasmid	1626	2141	+		Haloterrigena_turkmenica_DSM_5511_uid43501	NC_013749	plasmid pHTUR06							0.00005	pfam05911	Plant protein of unknown function (DUF86
	284176397	hypothetical protein Htur_5271	No	0	YES	plasmid	2111	2467	-		Haloterrigena_turkmenica_DSM_5511_uid43501	NC_013749	plasmid pHTUR06									
	284176398	hypothetical protein Htur_5272	No	0	YES	plasmid	2464	5058	-		Haloterrigena_turkmenica_DSM_5511_uid43501	NC_013749	plasmid pHTUR06		arCOG06342	1 (-)	S		Uncharacterized conserved protein	0.00007	pfam09528	Ehrlichia tandem repeat (Ehrlichia_rpt). ; MT0933-like antitoxin protein. This fami
	284176399	hypothetical protein Htur_5273	No	0		plasmid	5215	5343	-		Haloterrigena_turkmenica_DSM_5511_uid43501	NC_013749	plasmid pHTUR06									
===										11	Haloterrigena_turkmenica_DSM_5511_uid43501	NC_013749	plasmid pHTUR06									
	284176402	hypothetical protein Htur_5276	No	0	YES	plasmid	10840	11487	-		Haloterrigena_turkmenica_DSM_5511_uid43501	NC_013749	plasmid pHTUR06									
	284176403	hypothetical protein Htur_5277	No	0	YES	plasmid	11484	11726	-		Haloterrigena_turkmenica_DSM_5511_uid43501	NC_013749	plasmid pHTUR06		arCOG02866	1 (-)	S		Uncharacterized conserved protein			
	284176404	hypothetical protein Htur_5278	No	0	YES	plasmid	11719	12003	-		Haloterrigena_turkmenica_DSM_5511_uid43501	NC_013749	plasmid pHTUR06									
	284176405	hypothetical protein Htur_5279	No	0	YES	plasmid	12006	12305	-		Haloterrigena_turkmenica_DSM_5511_uid43501	NC_013749	plasmid pHTUR06									
	284176406	hypothetical protein Htur_5281	No	0	YES	plasmid	12946	13128	+		Haloterrigena_turkmenica_DSM_5511_uid43501	NC_013749	plasmid pHTUR06									
	284176407	hypothetical protein Htur_5282	No	2	YES	plasmid	13115	13357	+		Haloterrigena_turkmenica_DSM_5511_uid43501	NC_013749	plasmid pHTUR06									
	284176408	hypothetical protein Htur_5283	No	0	YES	plasmid	13362	13838	+		Haloterrigena_turkmenica_DSM_5511_uid43501	NC_013749	plasmid pHTUR06									
	284176409	hypothetical protein Htur_5284	No	0	YES	plasmid	14187	14456	-		Haloterrigena_turkmenica_DSM_5511_uid43501	NC_013749	plasmid pHTUR06									
	284176410	hypothetical protein Htur_5285	No	0	YES	plasmid	14449	14571	-		Haloterrigena_turkmenica_DSM_5511_uid43501	NC_013749	plasmid pHTUR06									
	284176411	hypothetical protein Htur_5286	No	0	YES	plasmid	14568	14999	-		Haloterrigena_turkmenica_DSM_5511_uid43501	NC_013749	plasmid pHTUR06									
	284176412	integrase family protein	No	0	YES	plasmid	15190	15813	+		Haloterrigena_turkmenica_DSM_5511_uid43501	NC_013749	plasmid pHTUR06		arCOG01241	31 (-)	L	XerC	Integrase	6E-18	COG4973	Site-specific recombinase XerC
===										6	Halovivax_ruber_XH_70_uid184819	NC_019964	chromosome									
	433637071	hypothetical protein Halru_0044	No	0			42182	42322	+		Halovivax_ruber_XH_70_uid184819	NC_019964	chromosome									
	433637072	hypothetical protein Halru_0045	No	0			42324	42551	+		Halovivax_ruber_XH_70_uid184819	NC_019964	chromosome	clust_427								
	433637073	hypothetical protein Halru_0046	No	0			43257	43397	+		Halovivax_ruber_XH_70_uid184819	NC_019964	chromosome									
	433637074	hypothetical protein Halru_0047	No	0			43511	44596	+		Halovivax_ruber_XH_70_uid184819	NC_019964	chromosome		arCOG06119	2 (-)	S		Uncharacterized conserved protein			
	433637075	hypothetical protein Halru_0048	No	0			44598	45749	+		Halovivax_ruber_XH_70_uid184819	NC_019964	chromosome									
	433637076	hypothetical protein Halru_0049	No	0			45856	45948	+		Halovivax_ruber_XH_70_uid184819	NC_019964	chromosome									
===										6	Halovivax_ruber_XH_70_uid184819	NC_019964	chromosome									
	433637383	putative hydrolase or acyltransferase of alpha/l	No	0			393705	394580	-		Halovivax_ruber_XH_70_uid184819	NC_019964	chromosome		arCOG01648	3 (-)	R	MhpC	Alpha/beta superfamily hydrolase	0.0000001	pfam12697	Alpha/beta hydrolase family. This family
	433637384	hypothetical protein Halru_0368	No	0			394708	395064	-		Halovivax_ruber_XH_70_uid184819	NC_019964	chromosome		arCOG11346	1	S		Uncharacterized conserved protein			
	433637385	hypothetical protein Halru_0369	No	2			395230	395412	+		Halovivax_ruber_XH_70_uid184819	NC_019964	chromosome									
	433637386	hypothetical protein Halru_0370	No	2			395599	395838	-		Halovivax_ruber_XH_70_uid184819	NC_019964	chromosome									
	433637387	nicotinate-mononucleotide:5,6-dimethylbenzim	No	0			395895	396683	-		Halovivax_ruber_XH_70_uid184819	NC_019964	chromosome							8E-13	pfam07452	CHRD domain. CHRD (after SWISS-PROT abbr ; Protein of unknown function (DUF2457). T
	433637388	ABC-type branched-chain amino acid transport	No	0			397242	398561	+		Halovivax_ruber_XH_70_uid184819	NC_019964	chromosome		arCOG01021	1 (-)	E	LivK	ABC-type branched-chain amino acid transport sy	7E-16	COG0683	ABC-type branched-chain amino acid trans
===										9	Halovivax_ruber_XH_70_uid184819	NC_019964	chromosome									
	433637891	hypothetical protein Halru_0894	No	0			936155	936298	-		Halovivax_ruber_XH_70_uid184819	NC_019964	chromosome		arCOG08142	1 (-)	S		Uncharacterized conserved protein			
	433637892	hypothetical protein Halru_0896	No	2			936756	936998	-		Halovivax_ruber_XH_70_uid184819	NC_019964	chromosome	clust_389								
	433637893	hypothetical protein Halru_0898	No	0			938002	938931	-		Halovivax_ruber_XH_70_uid184819	NC_019964	chromosome									
	433637894	hypothetical protein Halru_0900	No	0			939793	940068	+		Halovivax_ruber_XH_70_uid184819	NC_019964	chromosome									
	433637895	hypothetical protein Halru_0901	No	0			940070	940297	+		Halovivax_ruber_XH_70_uid184819	NC_019964	chromosome	clust_427								
	433637896	hypothetical protein Halru_0902	No	0			940562	941602	-		Halovivax_ruber_XH_70_uid184819	NC_019964	chromosome									

433637897	hypothetical protein Halru_0903	No	0	942034	942804	+		Halovivax_ruber_XH_70_uid184819	NC_019964	chromosome									
433637898	hypothetical protein Halru_0904	No	0	942801	943235	+		Halovivax_ruber_XH_70_uid184819	NC_019964	chromosome									
433637899	dienelactone hydrolase-like enzyme	No	0	943393	944052	+		Halovivax_ruber_XH_70_uid184819	NC_019964	chromosome		arCOG06847	1 (-)	R		Alpha/beta superfamily hydrolase	0.00003	COG0412	Dienelactone hydrolase and related enzym
===							5	Halovivax_ruber_XH_70_uid184819	NC_019964	chromosome									
433638808	hypothetical protein Halru_1836	No	7	1875388	1876044	+		Halovivax_ruber_XH_70_uid184819	NC_019964	chromosome		arCOG08970	1	S		Uncharacterized conserved protein			
433638809	transcriptional regulator with HTH domain and a	No	0	1876239	1877447	+		Halovivax_ruber_XH_70_uid184819	NC_019964	chromosome		arCOG00492	2 (-)	K	ARO8	Transcriptional regulators containing a DNA-bindir	5E-82	COG1167	Transcriptional regulators containing a
433638810	hypothetical protein Halru_1838	No	5	1877856	1878398	+		Halovivax_ruber_XH_70_uid184819	NC_019964	chromosome									
433638811	hypothetical protein Halru_1839	No	1	1878482	1879483	+		Halovivax_ruber_XH_70_uid184819	NC_019964	chromosome									
433638812	hypothetical protein Halru_1840	No	2	1879787	1880035	+		Halovivax_ruber_XH_70_uid184819	NC_019964	chromosome									
===							6	Halovivax_ruber_XH_70_uid184819	NC_019964	chromosome									
433639609	ABC-type multidrug transport system, ATPase	No	5	2685627	2687540	-		Halovivax_ruber_XH_70_uid184819	NC_019964	chromosome		arCOG02841	4 (-)	V	MdlB	ABC-type multidrug transport system, ATPase an	3E-127	COG1132	ABC-type multidrug transport system, ATP
433639610	hypothetical protein Halru_2660	No	0	2688226	2688483	-		Halovivax_ruber_XH_70_uid184819	NC_019964	chromosome									
433639611	hypothetical protein Halru_2661	No	0	2688857	2689756	+		Halovivax_ruber_XH_70_uid184819	NC_019964	chromosome									
433639612	hypothetical protein Halru_2663	Yes	0	2691326	2692084	-		Halovivax_ruber_XH_70_uid184819	NC_019964	chromosome									
433639613	hypothetical protein Halru_2664	No	0	2692288	2692650	-		Halovivax_ruber_XH_70_uid184819	NC_019964	chromosome									
433639614	thermosome subunit	No	0	2693094	2694758	-		Halovivax_ruber_XH_70_uid184819	NC_019964	chromosome		arCOG01257	3 (-)	O	GroL	Chaperonin GroEL (HSP60 family)	4E-136	pfam00118	TCP-1/cpn60 chaperonin family. This fami
===							9	Halovivax_ruber_XH_70_uid184819	NC_019964	chromosome									
433639633	putative metal-binding protein	No	0	2714063	2714419	+		Halovivax_ruber_XH_70_uid184819	NC_019964	chromosome		arCOG01121	2 (-)	S		SWIM zinc finger			
433639634	conserved hypothetical phage tail region prote	No	0	2714477	2714938	-		Halovivax_ruber_XH_70_uid184819	NC_019964	chromosome	clust_6						3E-32	pfam06841	T4-like virus tail tube protein gp19. Th
433639635	hypothetical protein Halru_2687	No	0	2714976	2715245	-		Halovivax_ruber_XH_70_uid184819	NC_019964	chromosome	clust_21								
433639636	hypothetical protein Halru_2688	No	0	2715242	2715769	-		Halovivax_ruber_XH_70_uid184819	NC_019964	chromosome	clust_16								
433639637	hypothetical protein Halru_2689	No	0	2715766	2715933	-		Halovivax_ruber_XH_70_uid184819	NC_019964	chromosome									
433639638	conserved hypothetical phage tail region prote	No	0	2715930	2716382	-		Halovivax_ruber_XH_70_uid184819	NC_019964	chromosome	clust_19						1E-22	pfam06841	T4-like virus tail tube protein gp19. Th
433639639	phage tail sheath protein FI	No	0	2716425	2718149	-		Halovivax_ruber_XH_70_uid184819	NC_019964	chromosome	clust_11						2E-28	COG3497	Phage tail sheath protein FI
433639640	hypothetical protein Halru_2692	No	0	2718457	2719701	-		Halovivax_ruber_XH_70_uid184819	NC_019964	chromosome	clust_28						0.00007	COG1196	Chromosome segregation ATPases
433639641	hypothetical protein Halru_2693	No	0	2720006	2721016	+		Halovivax_ruber_XH_70_uid184819	NC_019964	chromosome		arCOG10882	2 (-)	S		Uncharacterized conserved protein			
===							8	Halovivax_ruber_XH_70_uid184819	NC_019964	chromosome									
433639742	hypothetical protein Halru_2798	No	0	2826611	2827528	-		Halovivax_ruber_XH_70_uid184819	NC_019964	chromosome		arCOG09035	3	S		Uncharacterized conserved protein			
433639743	hypothetical protein Halru_2799	No	0	2827531	2827770	-		Halovivax_ruber_XH_70_uid184819	NC_019964	chromosome									
433639744	hypothetical protein Halru_2800	No	0	2828186	2828467	+		Halovivax_ruber_XH_70_uid184819	NC_019964	chromosome		arCOG08180	3 (-)	S		Uncharacterized conserved protein			
433639745	hypothetical protein Halru_2801	No	0	2828694	2829725	+		Halovivax_ruber_XH_70_uid184819	NC_019964	chromosome									
433639746	hypothetical protein Halru_2802	No	2	2829725	2831422	+		Halovivax_ruber_XH_70_uid184819	NC_019964	chromosome									
433639747	hypothetical protein Halru_2803	No	1	2831534	2832256	+		Halovivax_ruber_XH_70_uid184819	NC_019964	chromosome									
433639748	hypothetical protein Halru_2804	No	0	2833140	2833817	+		Halovivax_ruber_XH_70_uid184819	NC_019964	chromosome									
433639749	type I restriction enzyme R protein	No	0	2833894	2836851	-		Halovivax_ruber_XH_70_uid184819	NC_019964	chromosome		arCOG00879	2 (-)	V		Type I site-specific restriction-modification systen	5E-34	COG0610	Type I site-specific restriction-modific
===							6	Halovivax_ruber_XH_70_uid184819	NC_019964	chromosome									
433639769	hypothetical protein Halru_2825	No	0	2860317	2860511	-		Halovivax_ruber_XH_70_uid184819	NC_019964	chromosome									
433639770	hypothetical protein Halru_2826	No	0	2860508	2860795	-		Halovivax_ruber_XH_70_uid184819	NC_019964	chromosome									
433639771	hypothetical protein Halru_2827	No	0	2861002	2861220	+		Halovivax_ruber_XH_70_uid184819	NC_019964	chromosome		arCOG08128	7 (-)	S		Uncharacterized conserved protein			
[illegible]																			

305664357	hypothetical protein Igag_1973	No	0		1858108	1859421	+		Ignisphaera_aggregans_DSM_17230_uid51875	NC_014471	chromosome									
305664358	hypothetical protein Igag_1974	No	0		1859726	1860280	+		Ignisphaera_aggregans_DSM_17230_uid51875	NC_014471	chromosome									
305664359	Endonuclease IV	No	0		1860408	1861268	+		Ignisphaera_aggregans_DSM_17230_uid51875	NC_014471	chromosome		arCOG01894	3 (-)	L	Nfo	Endonuclease IV	3E-28	COG0648	Endonuclease IV
305664360	hypothetical protein Igag_1976	No	0		1861590	1861949	+		Ignisphaera_aggregans_DSM_17230_uid51875	NC_014471	chromosome									
305664361	hypothetical protein Igag_1977	No	0		1861951	1862955	+		Ignisphaera_aggregans_DSM_17230_uid51875	NC_014471	chromosome		arCOG01344	1 (-)	J	RPS19A	Ribosomal protein S19E (S16A)			
305664362	hypothetical protein Igag_1978	No	0		1864139	1864414	+		Ignisphaera_aggregans_DSM_17230_uid51875	NC_014471	chromosome									
305664363	hypothetical protein Igag_1979	No	0		1864425	1864790	+		Ignisphaera_aggregans_DSM_17230_uid51875	NC_014471	chromosome									
305664364	hypothetical protein Igag_1980	No	0		1864839	1865126	+		Ignisphaera_aggregans_DSM_17230_uid51875	NC_014471	chromosome		arCOG09893	5	S		Uncharacterized conserved protein			
305664365	hypothetical protein Igag_1981	Yes	1		1865413	1866909	+		Ignisphaera_aggregans_DSM_17230_uid51875	NC_014471	chromosome									
305664366	hypothetical protein Igag_1982	No	1		1867122	1868036	-		Ignisphaera_aggregans_DSM_17230_uid51875	NC_014471	chromosome		arCOG00056	1 (-)	R		Predicted RNA-binding protein, contains THUMP domain			
305664367	hypothetical protein Igag_1983	No	0		1868158	1868568	+		Ignisphaera_aggregans_DSM_17230_uid51875	NC_014471	chromosome									
305664368	hypothetical protein Igag_1985	No	3		1869339	1869605	-		Ignisphaera_aggregans_DSM_17230_uid51875	NC_014471	chromosome									
305664369	hypothetical protein Igag_1986	No	2		1869618	1869794	-		Ignisphaera_aggregans_DSM_17230_uid51875	NC_014471	chromosome									
===								6	Metallosphaera_cuprina_Ar_4_uid66329	NC_015435	chromosome									
330834496	ATP:cob(I)alamin adenosyltransferase	No	0		577330	577854	+		Metallosphaera_cuprina_Ar_4_uid66329	NC_015435	chromosome		arCOG00489	2 (-)	H		cob(I)alamin adenosyltransferase	6E-39	COG2096	cob(I)alamin adenosyltransferase
330834497	hypothetical protein Mcup_0636	No	0		577856	578125	-		Metallosphaera_cuprina_Ar_4_uid66329	NC_015435	chromosome		arCOG10438	1	S		Uncharacterized conserved protein			
330834498	hypothetical protein Mcup_0637	No	0		57	579028	+		Metallosphaera_cuprina_Ar_4_uid66329	NC_015435	chromosome									
330834499	hypothetical protein Mcup_0638	No	3		579618	580163	+		Metallosphaera_cuprina_Ar_4_uid66329	NC_015435	chromosome	clust_37								
330834500	hypothetical protein Mcup_0639	Yes	0		581411	581533	+		Metallosphaera_cuprina_Ar_4_uid66329	NC_015435	chromosome									
330834501	hypothetical protein Mcup_0640	No	4		582081	582470	-		Metallosphaera_cuprina_Ar_4_uid66329	NC_015435	chromosome		arCOG06050	1 (-)	S		Predicted membrane protein	0.00006	COG3431	Predicted membrane protein
===								14	Metallosphaera_cuprina_Ar_4_uid66329	NC_015435	chromosome									
330834553	ammonium transporter	No	11		620645	622015	-		Metallosphaera_cuprina_Ar_4_uid66329	NC_015435	chromosome		arCOG04397	1 (-)	P	AmtB	Ammonia permease	6E-99	COG0004	Ammonia permease
330834554	hypothetical protein Mcup_0693	No	0		622876	623043	+		Metallosphaera_cuprina_Ar_4_uid66329	NC_015435	chromosome									
330834555	hypothetical protein Mcup_0694	No	0		623131	623259	-		Metallosphaera_cuprina_Ar_4_uid66329	NC_015435	chromosome									
330834556	hypothetical protein Mcup_0695	No	0		623750	623866	+		Metallosphaera_cuprina_Ar_4_uid66329	NC_015435	chromosome									
330834557	hypothetical protein Mcup_0696	No	0		624395	624595	+		Metallosphaera_cuprina_Ar_4_uid66329	NC_015435	chromosome									
330834558	hypothetical protein Mcup_0697	No	0		624868	625095	+		Metallosphaera_cuprina_Ar_4_uid66329	NC_015435	chromosome									
330834559	hypothetical protein Mcup_0698	No	0		625288	625647	+		Metallosphaera_cuprina_Ar_4_uid66329	NC_015435	chromosome									
330834560	IS5 inactivated transposase	No	1		625644	625844	+		Metallosphaera_cuprina_Ar_4_uid66329	NC_015435	chromosome		arCOG06508	1 (-)	L		Transposase and inactivated derivatives, IS5 family			
330834561	major facilitator transporter	No	12		626052	627242	+		Metallosphaera_cuprina_Ar_4_uid66329	NC_015435	chromosome		arCOG00135	3 (-)	G	ProP	Permease of the major facilitator superfamily	0.00000004	COG2211	Na+/melibiose symporter and related tran
330834562	hypothetical protein Mcup_0701	No	0		627684	627797	-		Metallosphaera_cuprina_Ar_4_uid66329	NC_015435	chromosome									
330834563	hypothetical protein Mcup_0702	No	0		627947	628063	-		Metallosphaera_cuprina_Ar_4_uid66329	NC_015435	chromosome									
330834564	hypothetical protein Mcup_0703	No	0		628458	628607	-		Metallosphaera_cuprina_Ar_4_uid66329	NC_015435	chromosome									
330834565	hypothetical protein Mcup_0704	No	0		628766	628930	-		Metallosphaera_cuprina_Ar_4_uid66329	NC_015435	chromosome									
330834566	hypothetical protein Mcup_0705	No	1		630128	630538	+		Metallosphaera_cuprina_Ar_4_uid66329	NC_015435	chromosome		arCOG03871	6 (-)	N		Flagellar protein G			
===								6	Metallosphaera_cuprina_Ar_4_uid66329	NC_015435	chromosome									
330834854	hypothetical protein Mcup_0993	No	0		907635	907778	-		Metallosphaera_cuprina_Ar_4_uid66329	NC_015435	chromosome									
330834855	hypothetical protein Mcup_0994	No	0		908075	908242	+		Metallosphaera_cuprina_Ar_4_uid66329	NC_015435	chromosome									
330834856	peptidase A5, thermopsin	Yes	2		908428	911211	-		Metallosphaera_cuprina_Ar_4_uid66329	NC_015435	chromosome	arCOG03671,a	arCOG03671	3 (-)	E		Thermopsin-like protease	2E-45	pfam05317	Thermopsin. This family consists of seve
330834857	hypothetical protein Mcup_0996	No	0		911471	911602	+		Metallosphaera_cuprina_Ar_4_uid66329	NC_015435	chromosome									
330834858	hypothetical protein Mcup_0997	No	0		911903	912055	+		Metallosphaera_cuprina_Ar_4_uid66329	NC_015435	chromosome									
330834859	hypothetical protein Mcup_0998	No	3		912211	912585	+		Metallosphaera_cuprina_Ar_4_uid66329	NC_015435	chromosome		arCOG10299	1	S		Uncharacterized conserved protein			
===								29	Metallosphaera_cuprina_Ar_4_uid66329	NC_015435	chromosome									
330834935	cupin 2 domain-containing protein	No	0		983463	983579	+		Metallosphaera_cuprina_Ar_4_uid66329	NC_015435	chromosome									
330834936	high-affinity nickel-transporter	No	8		984168	985196	+		Metallosphaera_cuprina_Ar_4_uid66329	NC_015435	chromosome		arCOG06023	1 (-)	P	HoxN	High-affinity nickel permease	9E-110	COG3376	High-affinity nickel permease
330834937	hypothetical protein Mcup_1076	No	0		985329	985481	-		Metallosphaera_cuprina_Ar_4_uid66329	NC_015435	chromosome									
330834938	hypothetical protein Mcup_1077	No	0		986313	986483	+		Metallosphaera_cuprina_Ar_4_uid66329	NC_015435	chromosome									
330834939	hypothetical protein Mcup_1079	No	0		986640	986846	-		Metallosphaera_cuprina_Ar_4_uid66329	NC_015435	chromosome									
330834940	hypothetical protein Mcup_1078	No	0		986907	987059	+		Metallosphaera_cuprina_Ar_4_uid66329	NC_015435	chromosome									
330834941	hypothetical protein Mcup_1080	No	0		987176	987355	-		Metallosphaera_cuprina_Ar_4_uid66329	NC_015435	chromosome									
330834942	hypothetical protein Mcup_1081	No	0		988548	988763	+		Metallosphaera_cuprina_Ar_4_uid66329	NC_015435	chromosome									
330834943	hypothetical protein Mcup_1082	No	0		988878	989096	-		Metallosphaera_cuprina_Ar_4_uid66329	NC_015435	chromosome									
330834944	hypothetical protein Mcup_1083	No	0		989396	989536	+		Metallosphaera_cuprina_Ar_4_uid66329	NC_015435	chromosome									
330834945	hypothetical protein Mcup_1084	No	0		990646	990765	-		Metallosphaera_cuprina_Ar_4_uid66329	NC_015435	chromosome									
330834946	hypothetical protein Mcup_1085	No	0		991002	991217	+		Metallosphaera_cuprina_Ar_4_uid66329	NC_015435	chromosome									
330834947	hypothetical protein Mcup_1086	No	0		991196	991348	-		Metallosphaera_cuprina_Ar_4_uid66329	NC_015435	chromosome									
330834948	hypothetical protein Mcup_1087	No	0		991384	991539	-		Metallosphaera_cuprina_Ar_4_uid66329	NC_015435	chromosome									
330834949	hypothetical protein Mcup_1088	No	0		991702	991815	-		Metallosphaera_cuprina_Ar_4_uid66329	NC_015435	chromosome									
330834950	hypothetical protein Mcup_1089	No	0		991949	992065	-		Metallosphaera_cuprina_Ar_4_uid66329	NC_015435	chromosome									
330834951	hypothetical protein Mcup_1090	No	0		992392	992628	-		Metallosphaera_cuprina_Ar_4_uid66329	NC_015435	chromosome									
330834952	hypothetical protein Mcup_1091	No	0		992659	992820	-		Metallosphaera_cuprina_Ar_4_uid66329	NC_015435	chromosome									
330834953	hypothetical protein Mcup_1092	No	1		992829	992951	-		Metallosphaera_cuprina_Ar_4_uid66329	NC_015435	chromosome									
330834954	hypothetical protein Mcup_1093	No	0		993056	993205	+		Metallosphaera_cuprina_Ar_4_uid66329	NC_015435	chromosome									
330834955	hypothetical protein Mcup_1094	No	0		993729	993866	-		Metallosphaera_cuprina_Ar_4_uid66329	NC_015435	chromosome									
330834956	hypothetical protein Mcup_1095	No	0		993892	994113	+		Metallosphaera_cuprina_Ar_4_uid66329	NC_015435	chromosome									
330834957	hypothetical protein Mcup_1096	No	0		994071	994202	+		Metallosphaera_cuprina_Ar_4_uid66329	NC_015435	chromosome									
330834958	hypothetical protein Mcup_1097	No	0		994316	994483	-		Metallosphaera_cuprina_Ar_4_uid66329	NC_015435	chromosome									
330834959	hypothetical protein Mcup_1098	No	1		994812	994934	-		Metallosphaera_cuprina_Ar_4_uid66329	NC_015435	chromosome									
330834960	hypothetical protein Mcup_1099	No	0		995428	995640	-		Metallosphaera_cuprina_Ar_4_uid66329	NC_015435	chromosome									
330834961	hypothetical protein Mcup_1100	No	0		995639	995806	+		Metallosphaera_cuprina_Ar_4_uid66329	NC_015435	chromosome									
330834962	hypothetical protein Mcup_1101	No	0		996509	996637	+		Metallosphaera_cuprina_Ar_4_uid66329	NC_015435	chromosome									
330834963	CRISPR-associated Cas1 family protein	No	0		997850	998740	+		Metallosphaera_cuprina_Ar_4_uid66329	NC_015435	chromosome		arCOG01452	5 (-)	V	Cas1	CRISPR-associated protein Cas1	5E-40	COG1518	CRISPR-associated protein Cas1
===								19	Metallosphaera_cuprina_Ar_4_uid66329	NC_015435	chromosome									
330834974	hypothetical protein Mcup_1113	No	0		1004987	1005217	-		Metallosphaera_cuprina_Ar_4_uid66329	NC_015435	chromosome		arCOG00823	1 (-)	K	AbrB	Transcriptional regulator AbrB			
330834975	hypothetical protein Mcup_1114	No	0		1006833	1006979	+		Metallosphaera_cuprina_Ar_4_uid66329	NC_015435	chromosome									
330834976	hypothetical protein Mcup_1115	No	1		1006933	1007091	-		Metallosphaera_cuprina_Ar_4_uid66329	NC_015435	chromosome									
330834977	hypothetical protein Mcup_1116	No	0		1007439	1007672	-		Metallosphaera_cuprina_Ar_4_uid66329	NC_015435	chromosome									
330834978	hypothetical protein Mcup_1117	No	0		1007927	1008049	+		Metallosphaera_cuprina_Ar_4_uid66329	NC_015435	chromosome									
330834979	hypothetical protein Mcup_1118	No	0		1008361	1008480	-		Metallosphaera_cuprina_Ar_4_uid66329	NC_015435	chromosome									
330834980	hypothetical protein Mcup_1119	No	0		1008636	1008749	+		Metallosphaera_cuprina_Ar_4_uid66329	NC_015435	chromosome									
330834981	hypothetical protein Mcup_1120	No	0		1008811	1008957	-		Metallosphaera_cuprina_Ar_4_uid66329	NC_015435	chromosome									
330834982	hypothetical protein Mcup_1121	No	0		1009152	1009271	+		Metallosphaera_cuprina_Ar_4_uid66329	NC_015435	chromosome									
330834983	hypothetical protein Mcup_1122	No	0		1009978	1010097	+		Metallosphaera_cuprina_Ar_4_uid66329	NC_015435	chromosome									
330834984	hypothetical protein Mcup_1123	No	0		1010790	1010927	+		Metallosphaera_cuprina_Ar_4_uid66329	NC_015435	chromosome									
330834985	hypothetical protein Mcup_1124	No	0		1010973	1011116	+		Metallosphaera_cuprina_Ar_4_uid66329	NC_015435	chromosome									
330834986	hypothetical protein Mcup_1125	No	0		1011187	1011333	+		Metallosphaera_cuprina_Ar_4_uid66329	NC_015435	chromosome									
330834987	hypothetical protein Mcup_1126	No	0		1011620	1011946	-		Metallosphaera_cuprina_Ar_4_uid66329	NC_015435	chromosome									
330834988	hypothetical protein Mcup_1127	No	0		1012269	1012397	-		Metallosphaera_cuprina_Ar_4_uid66329	NC_015435	chromosome									
330834989	hypothetical protein Mcup_1128	No	0		1012445	1012591	-		Metallosphaera_cuprina_Ar_4_uid66329	NC_015435	chromosome									
330834990	hypothetical protein Mcup_1129	No	0		1012735	1013640	+		Metallosphaera_cuprina_Ar_4_uid66329	NC_015435	chromosome		arCOG10846	2	L		PD-DExK family endonuclease			
330834991	glycosyl transferase, group 1	No	0		1014512	1015732	-		Metallosphaera_cuprina_Ar_4_uid66329	NC_015435	chromosome		arCOG01417	2 (-)	M	RfaG	Glycosyltransferase	0.000000001	COG0438	Glycosyltransferase
330834992	hypothetical protein Mcup_1131	No	0		1015744	1015890	+		Metallosphaera_cuprina_Ar_4_uid66329	NC_015435	chromosome									
===								7	Methanobacterium_AL_21_uid63623	NC_015216	chromosome									
325957761	hypothetical protein Metbo_0002	No	6		1643	2533	+		Methanobacterium_AL_21_uid63623	NC_015216	chromosome		arCOG10162	4	S		Uncharacterized conserved protein	0.000006	pfam04893	Yip1 domain. The Yip1 integral membrane
325957762	hypothetical protein Metbo_0003	No	0		2600	3007	+		Methanobacterium_AL_21_uid63623	NC_015216	chromosome		arCOG04279	2 (-)	C		Swiveling domain associated with predicted acor	4E-39	COG1786	Swiveling domain associated with predict
325957763	hypothetical protein Metbo_0004	No	4		3042	3467	-		Methanobacterium_AL_21_uid63623	NC_015216	chromosome	clust_246								
325957764	hypothetical protein Metbo_0005	No	3		3460	3906	-		Methanobacterium_AL_21_uid63623	NC_015216	chromosome							0.00007	pfam04893	Yip1 domain. The Yip1 integral membrane
325957765	hypothetical protein Metbo_0006	No	3		3971	4342	-		Methanobacterium_AL_21_uid63623	NC_015216	chromosome		arCOG10898	4	S		Uncharacterized conserved protein			
325957766	hypothetical protein Metbo_0007	No	3		4542	4934	-		Methanobacterium_AL_21_uid63623	NC_015216	chromosome							0.00007	pfam07694	5TMR of 5TMR-LYT. This entry represents
325957767	amidohydrolase	No	0		5091	6338	+		Methanobacterium_AL_21_uid63623	NC_015216	chromosome		arCOG00696	1 (-)	Q	HutI	Imidazolonepropionase or related amidohydrolas	3E-39	COG1228	Imidazolonepropionase and related amidoh
===								5	Methanobacterium_AL_21_uid63623	NC_015216	chromosome									
325957784	hypothetical protein Metbo_0025	No	1		24374	24679	+		Methanobacterium_AL_21_uid63623	NC_015216	chromosome									
325957785	hypothetical protein Metbo_0026	No	0		24828	24959	-		Methanobacterium_AL_21_uid63623	NC_015216	chromosome									
325957786	transcriptional regulator	No	0		25150	25443	+		Methanobacterium_AL_21_uid63623	NC_015216	chromosome		arCOG00731	3 (-)	K	ArsR	Transcriptional regulator containing HTH domain, ArsR family			
325957787	hypothetical protein Metbo_0028	No	3		25477	25836	+		Methanobacterium_AL_21_uid63623	NC_015216	chromosome		arCOG10459	1	S		Uncharacterized conserved protein			
325957788	hypothetical protein Metbo_0029	Yes	1		25924	26250	+		Methanobacterium_AL_21_uid63623	NC_015216	chromosome									
===								5	Methanobacterium_AL_21_uid63623	NC_015216	chromosome									
325958597	hypothetical protein Metbo_0841	No	1		801822	802433	-		Methanobacterium_AL_21_uid63623	NC_015216	chromosome									
325958598	hypothetical protein Metbo_0842	Yes	1		802488	803123	-		Methanobacterium_AL_21_uid63623	NC_015216	chromosome		arCOG10454	1	S		Uncharacterized conserved protein			
325958599	hypothetical protein Metbo_0843	No	2		803136	803369	-		Methanobacterium_AL_21_uid63623	NC_015216	chromosome									
325958600	hypothetical protein Metbo_0844	No	9		803588	805003	-		Methanobacterium_AL_21_uid63623	NC_015216	chromosome		arCOG02884	2 (-)	S		Predicted membrane protein	6E-79	pfam07760	Protein of unknown function (DUF1616). T ; Protein of unknown function (DUF1616). T
325958601	hypothetical protein Metbo_0845	No	0		805239	806129	+		Methanobacterium_AL_21_uid63623	NC_015216	chromosome									
===								6	Methanobacterium_AL_21_uid63623	NC_015216	chromosome									
325958814	hypothetical protein Metbo_1061	No	0		1056842	1057054	-		Methanobacterium_AL_21_uid63623	NC_015216	chromosome									
325958815	hypothetical protein Metbo_1063	No	3		1057710	1058378	-		Methanobacterium_AL_21_uid63623	NC_015216	chromosome	arCOG10883,a	arCOG10883	5	S		Uncharacterized conserved protein			
325958816	hypothetical protein Metbo_1064	No	2		1058711	1058869	+		Methanobacterium_AL_21_uid63623	NC_015216	chromosome									
325958817	metallophosphoesterase	No	0		1059253	1060521	-		Methanobacterium_AL_21_uid63623	NC_015216	chromosome		arCOG06201	1 (-)	R		Calcineurin family phosphoesterase			
325958818	hypothetical protein Metbo_1066	No	0		1060754	1060957	-		Methanobacterium_AL_21_uid63623	NC_015216	chromosome									
325958819	hypothetical protein Metbo_1067	No	0		1061022	1061285	-		Methanobacterium_AL_21_uid63623	NC_015216	chromosome							0.00006	COG1196	Chromosome segregation ATPases
===								20	Methanobacterium_AL_21_uid63623	NC_015216	chromosome									
325958872	hypothetical protein Metbo_1121	No	2	plasmid	1127922	1128557	-		Methanobacterium_AL_21_uid63623	NC_015216	chromosome		arCOG07652	5 (-)	L		Superfamily II helicase or inactivated derivatives			
325958873	hypothetical protein Metbo_1122	No	1	plasmid	1128884	1129906	+		Methanobacterium_AL_21_uid63623	NC_015216	chromosome									
325958874	hypothetical protein Metbo_1123	No	2	plasmid	1130668	1130985	-		Methanobacterium_AL_21_uid63623	NC_015216	chromosome	clust_334								
325958875	hypothetical protein Metbo_1124	No	4	plasmid	1131132	1131581	-		Methanobacterium_AL_21_uid63623	NC_015216	chromosome									
325958876	hypothetical protein Metbo_1125	No	4	plasmid	1132579	1132929	-		Methanobacterium_AL_21_uid63623	NC_015216	chromosome		arCOG10898	4	S		Uncharacterized conserved protein	0.00001	pfam03553	Na+/H+ antiporter family. This family in
325958877	hypothetical protein Metbo_1127	No	3	plasmid	1133665	1134030	-		Methanobacterium_AL_21_uid63623	NC_015216	chromosome		arCOG11271	3	S		Uncharacterized conserved protein			
325958878	hypothetical protein Metbo_1128	No	3	plasmid	1134077	1134436	-		Methanobacterium_AL_21_uid63623	NC_015216	chromosome									
325958879	hypothetical protein Metbo_1129	No	0	plasmid	1134729	1134914	-		Methanobacterium_AL_21_uid63623	NC_015216	chromosome									
325958880	hypothetical protein Metbo_1130	No	0	plasmid	1134926	1135384	-		Methanobacterium_AL_21_uid63623	NC_015216	chromosome									
325958881	hypothetical protein Metbo_1131	No	5	plasmid	1135806	1136234	+		Methanobacterium_AL_21_uid63623	NC_015216	chromosome									
325958882	hypothetical protein Metbo_1132	No	1	plasmid	1136556	1136960	+		Methanobacterium_AL_21_uid63623	NC_015216	chromosome									
325958883	hypothetical protein Metbo_1133	No	0	plasmid	1137364	1138434	+		Methanobacterium_AL_21_uid63623	NC_015216	chromosome		arCOG09531	2	S		Uncharacterized conserved protein			
325958884	hypothetical protein Metbo_1134	No	0	plasmid	1138548	1139630	-		Methanobacterium_AL_21_uid63623	NC_015216	chromosome							0.00002	pfam01807	CHC2 zinc finger. This domain is princip
325958885	hypothetical protein Metbo_1135	No	0	plasmid	1139631	1139996	-		Methanobacterium_AL_21_uid63623	NC_015216	chromosome									
325958886	hypothetical protein Metbo_1136	No	0	plasmid	1139984	1142119	-		Methanobacterium_AL_21_uid63623	NC_015216	chromosome		arCOG07809	9 (-)	Y		Viral primase fused to AAA DnaA like ATPase and Zn finger domain			
325958887	hypothetical protein Metbo_1137	No	0	plasmid	1142154	1142285	-		Methanobacterium_AL_21_uid63623	NC_015216	chromosome									
325958888	hypothetical protein Metbo_1138	No	0	plasmid	1142266	1142439	-		Methanobacterium_AL_21_uid63623	NC_015216	chromosome									
325958889	hypothetical protein Metbo_1139	No	1	plasmid	1142742	1143386	-		Methanobacterium_AL_21_uid63623	NC_015216	chromosome		arCOG10883	5	S		Uncharacterized conserved protein			
325958890	hypothetical protein Metbo_1140	No	3	plasmid	1143824	1144363	-		Methanobacterium_AL_21_uid63623	NC_015216	chromosome	arCOG10883,a	arCOG10883	5	S		Uncharacterized conserved protein			
325958891	hypothetical protein Metbo_1141	No	6	plasmid	1144612	1145445	-		Methanobacterium_AL_21_uid63623	NC_015216	chromosome		arCOG04907	2 (-)	S		Uncharacterized conserved membrane protein			
===								8	Methanobacterium_AL_21_uid63623	NC_015216	chromosome									
325958896	hypothetical protein Metbo_1146	Yes	0		1151512	1151979	-		Methanobacterium_AL_21_uid63623	NC_015216	chromosome		arCOG10865	9	S		Uncharacterized conserved protein			
325958897	hypothetical protein Metbo_1147	No	0		1152518	1152964	-		Methanobacterium_AL_21_uid63623	NC_015216	chromosome		arCOG10865	9	S		Uncharacterized conserved protein			
325958898	cellulase	No	0		1153574	1154599	-		Methanobacterium_AL_21_uid63623	NC_015216	chromosome		arCOG01518	2 (-)	G	FrvX	Cellulase M or related protein	3E-108	COG1363	Cellulase M and related proteins
325958899	hypothetical protein Metbo_1149	No	2		1154669	1154890	-		Methanobacterium_AL_21_uid63623	NC_015216	chromosome		arCOG10251	2	S		Uncharacterized conserved protein			
325958900	hypothetical protein Metbo_1150	No	1		1154920	1155333	-		Methanobacterium_AL_21_uid63623	NC_015216	chromosome		arCOG10248	3	S		Uncharacterized conserved protein			
325958901	cyclase/dehydrase	No	0		1155723	1156007	+		Methanobacterium_AL_21_uid63623	NC_015216	chromosome									
325958902	hypothetical protein Metbo_1152	No	1		1156400	1157308	+		Methanobacterium_AL_21_uid63623	NC_015216	chromosome		arCOG07613	2 (-)	S		Uncharacterized conserved protein			
325958903	hypothetical protein Metbo_1153	No	1		1157322	1159370	+		Methanobacterium_AL_21_uid63623	NC_015216	chromosome		arCOG10249	3	S		Uncharacterized conserved protein			
===								8	Methanobacterium_AL_21_uid63623	NC_015216	chromosome									
325959067	hypothetical protein Metbo_1319	No	2		1321644	1321928	-		Methanobacterium_AL_21_uid63623	NC_015216	chromosome		arCOG02717	1 (-)	S		Predicted membrane protein	3E-18	COG2034	Predicted membrane protein
325959068	hypothetical protein Metbo_1320	No	2		1322324	1322593	+		Methanobacterium_AL_21_uid63623	NC_015216	chromosome									
325959069	hypothetical protein Metbo_1321	No	3		1322586	1322819	+		Methanobacterium_AL_21_uid63623	NC_015216	chromosome									
325959070	hypothetical protein Metbo_1322	No	1		1322788	1323093	-		Methanobacterium_AL_21_uid63623	NC_015216	chromosome									
325959071	hypothetical protein Metbo_1323	No	0		1323516	1323848	+		Methanobacterium_AL_21_uid63623	NC_015216	chromosome									
325959072	hypothetical protein Metbo_1324	No	0		1324019	1324306	+		Methanobacterium_AL_21_uid63623	NC_015216	chromosome		arCOG10257	2	S		Uncharacterized conserved protein			
325959073	hypothetical protein Metbo_1326	No	0		1325244	1325483	+		Methanobacterium_AL_21_uid63623	NC_015216	chromosome									
325959074	MCP methyltransferase/methylesterase, cheR/	No	0		1325512	1328448	-		Methanobacterium_AL_21_uid63623	NC_015216	chromosome		arCOG04921	1 (-)	N	CheR	Methylase of chemotaxis methyl-accepting protei	1E-48	COG1352	Methylase of chemotaxis methyl-accepting ; CheB methylesterase. ; PAS domain. ; Chromosome segregation ATPases
===								7	Methanobacterium_AL_21_uid63623	NC_015216	chromosome									
325959137	hypothetical protein Metbo_1391	No	0		1394399	1394857	+		Methanobacterium_AL_21_uid63623	NC_015216	chromosome		arCOG00419	4 (-)	F	Hit	HIT family hydrolase	0.0000003	COG0537	Diadenosine tetraphosphate (Ap4A) hydrol
325959138	hypothetical protein Metbo_1392	No	3		1394949	1395386	+		Methanobacterium_AL_21_uid63623	NC_015216	chromosome									
325959139	hypothetical protein Metbo_1393	No	4		1396031	1396435	+		Methanobacterium_AL_21_uid63623	NC_015216	chromosome									
325959140	hypothetical protein Metbo_1394	No	0		1396612	1396734	-		Methanobacterium_AL_21_uid63623	NC_015216	chromosome									
325959141	hypothetical protein Metbo_1395	No	1		1396771	1397109	-		Methanobacterium_AL_21_uid63623	NC_015216	chromosome		arCOG10883	5	S		Uncharacterized conserved protein			
325959142	hypothetical protein Metbo_1396	No	4		1397362	1398021	+		Methanobacterium_AL_21_uid63623	NC_015216	chromosome		arCOG03032	12 (-)	R	NrfG	TPR repeats containing protein	0.000000002	COG0457	FOG: TPR repeat
325959143	hypothetical protein Metbo_1397	No	2		1398068	1398325	-		Methanobacterium_AL_21_uid63623	NC_015216	chromosome									
===								8	Methanobacterium_AL_21_uid63623	NC_015216	chromosome									
325959204	transcriptional regulator TrmB	No	0		1473131	1473931	+		Methanobacterium_AL_21_uid63623	NC_015216	chromosome		arCOG02037	2 (-)	K		Sugar-specific transcriptional regulator TrmB	7E-35	COG1378	Predicted transcriptional regulators
325959205	hypothetical protein Metbo_1462	No	0		1474269	1474583	+		Methanobacterium_AL_21_uid63623	NC_015216	chromosome									
325959206	hypothetical protein Metbo_1463	No	0		1474599	1474958	+		Methanobacterium_AL_21_uid63623	NC_015216	chromosome							3E-47	pfam09862	Protein of unknown function (DUF2089). T
325959207	hypothetical protein Metbo_1464	No	0		1474997	1475125	+		Methanobacterium_AL_21_uid63623	NC_015216	chromosome									
325959208	hypothetical protein Metbo_1465	No	0		1475128	1475514	+		Methanobacterium_AL_21_uid63623	NC_015216	chromosome									
325959209	serine active site containing 1-like protein	No	10		1476727	1477815	+		Methanobacterium_AL_21_uid63623	NC_015216	chromosome									
325959210	SOUL heme-binding protein	No	0		1477917	1478519	+		Methanobacterium_AL_21_uid63623	NC_015216	chromosome		arCOG04820	1 (-)	T		SOUL heme-binding protein	2E-34	pfam04832	SOUL heme-binding protein. This family r
325959211	hypothetical protein Metbo_1469	No	2		1479152	1479367	+		Methanobacterium_AL_21_uid63623	NC_015216	chromosome									
===								5	Methanobacterium_AL_21_uid63623	NC_015216	chromosome									
325959275	hypothetical protein Metbo_1539	No	3		1555546	1555878	+		Methanobacterium_AL_21_uid63623	NC_015216	chromosome		arCOG10948	1	S		Uncharacterized conserved protein			
325959276	hypothetical protein Metbo_1540	No	0		1556212	1556730	-		Methanobacterium_AL_21_uid63623	NC_015216	chromosome									
325959277	deoxyguanosinetriphosphate triphosphohydrol	No	0		1556957	1558942	+		Methanobacterium_AL_21_uid63623	NC_015216	chromosome		arCOG07445	3 (-)	F	Dgt	dGTP triphosphohydrolase	9E-44	COG0232	dGTP triphosphohydrolase
325959278	hypothetical protein Metbo_1542	No	0		1559174	1560055	-		Methanobacterium_AL_21_uid63623	NC_015216	chromosome							6E-10	pfam09509	Protein of unknown function (Hypoth_ymh)
325959279	hypothetical protein Metbo_1543	No	7		1560651	1561532	+		Methanobacterium_AL_21_uid63623	NC_015216	chromosome		arCOG10162	4	S		Uncharacterized conserved protein			
===								10	Methanobacterium_AL_21_uid63623	NC_015216	chromosome									
325959308	hypothetical protein Metbo_1575	Yes	1		1606523	1606999	+		Methanobacterium_AL_21_uid63623	NC_015216	chromosome									
325959309	DNA polymerase B exonuclease	No	0		1607082	1608821	-		Methanobacterium_AL_21_uid63623	NC_015216	chromosome		arCOG00328	8 (-)	L	PolB	DNA polymerase elongation subunit (family B)	1E-106	COG0417	DNA polymerase elongation subunit (famil
325959310	hypothetical protein Metbo_1577	No	0		1608961	1609338	+		Methanobacterium_AL_21_uid63623	NC_015216	chromosome									
325959311	hypothetical protein Metbo_1578	No	0		1609371	1609865	+		Methanobacterium_AL_21_uid63623	NC_015216	chromosome									
325959312	hypothetical protein Metbo_1579	Yes	1		1609891	1610301	-		Methanobacterium_AL_21_uid63623	NC_015216	chromosome									
325959313	hypothetical protein Metbo_1580	Yes	1		1610690	1610992	+		Methanobacterium_AL_21_uid63623	NC_015216	chromosome									
325959314	hypothetical protein Metbo_1581	No	0		1611068	1612261	-		Methanobacterium_AL_21_uid63623	NC_015216	chromosome									
325959315	hypothetical protein Metbo_1582	Yes	1		1612342	1612782	-		Methanobacterium_AL_21_uid63623	NC_015216	chromosome									
325959316	hypothetical protein Metbo_1583	Yes	1		1612934	1613338	-		Methanobacterium_AL_21_uid63623	NC_015216	chromosome		arCOG02488	4 (-)	S		Cell suface protein			
325959317	hypothetical protein Metbo_1584	No	0		1614252	1614548	+		Methanobacterium_AL_21_uid63623	NC_015216	chromosome									
===								5	Methanobacterium_AL_21_uid63623	NC_015216	chromosome									
325959366	hypothetical protein Metbo_1634	No	0		1680520	1680810	-		Methanobacterium_AL_21_uid63623	NC_015216	chromosome									
325959367	hypothetical protein Metbo_1635	No	0		1680928	1681488	-		Methanobacterium_AL_21_uid63623	NC_015216	chromosome		arCOG02589	3 (-)	T	CheY	Rec domain			
325959368	hypothetical protein Metbo_1636	No	0		1682017	1682190	+		Methanobacterium_AL_21_uid63623	NC_015216	chromosome									
325959369	hypothetical protein Metbo_1637	No	0		1682294	1682821	+		Methanobacterium_AL_21_uid63623	NC_015216	chromosome									
325959370	hypothetical protein Metbo_1638	No	0		1683304	1683792	+		Methanobacterium_AL_21_uid63623	NC_015216	chromosome									
===								8	Methanobacterium_AL_21_uid63623	NC_015216	chromosome									
325959375	ion transport 2 domain-containing protein	No	5		1692347	1693099	+		Methanobacterium_AL_21_uid63623	NC_015216	chromosome		arCOG01964	3 (-)	P	Kch	Kef-type K+ transport system, predicted NAD-binding component			
325959376	hypothetical protein Metbo_1644	No	4		1693538	1693888	+		Methanobacterium_AL_21_uid63623	NC_015216	chromosome									
325959377	hypothetical protein Metbo_1645	No	4		1694271	1694618	+		Methanobacterium_AL_21_uid63623	NC_015216	chromosome		arCOG11273	1	S		Uncharacterized conserved protein			
325959378	hypothetical protein Metbo_1646	No	0		1694958	1695386	-		Methanobacterium_AL_21_uid63623	NC_015216	chromosome	clust_241								
325959379	hypothetical protein Metbo_1647	No	0		1695581	1695802	+		Methanobacterium_AL_21_uid63623	NC_015216	chromosome									
325959380	hypothetical protein Metbo_1648	No	0		1696110	1696298	+		Methanobacterium_AL_21_uid63623	NC_015216	chromosome									
325959381	polymorphic membrane protein	Yes	1		1696644	1699124	+		Methanobacterium_AL_21_uid63623	NC_015216	chromosome		arCOG02555	3 (-)	S		NosD-like periplasmic protein	0.00000007	pfam05048	Periplasmic copper-binding protein (NosD ; Periplasmic copper-binding protein (NosD ; Protein of unknown function (DUF1565). T
325959382	proteinase inhibitor i42, chagasin	Yes	1		1699328	1699705	-		Methanobacterium_AL_21_uid63623	NC_015216	chromosome									
===								10	Methanobacterium_AL_21_uid63623	NC_015216	chromosome									
325959501	hypothetical protein Metbo_1772	No	0		1814344	1814439	-		Methanobacterium_AL_21_uid63623	NC_015216	chromosome									
325959502	hypothetical protein Metbo_1773	No	0		1815117	1815248	+		Methanobacterium_AL_21_uid63623	NC_015216	chromosome									
325959503	hypothetical protein Metbo_1774	No	0		1815248	1815406	+		Methanobacterium_AL_21_uid63623	NC_015216	chromosome									
325959504	PAS/PAC sensor signal transduction histidine k	No	0		1815956	1817677	+		Methanobacterium_AL_21_uid63623	NC_015216	chromosome		arCOG06131	1 (-)	T	AtoS	PAS domain	5E-67	COG4251	Bacteriophytochrome (light-regulated sig ; PAS domain.
325959505	hypothetical protein Metbo_1776	No	0		1817759	1818109	-		Methanobacterium_AL_21_uid63623	NC_015216	chromosome									
325959506	hypothetical protein Metbo_1777	No	0		1818379	1818573	-		Methanobacterium_AL_21_uid63623	NC_015216	chromosome									
325959507	hypothetical protein Metbo_1779	No	0		1819734	1819889	+		Methanobacterium_AL_21_uid63623	NC_015216	chromosome									
325959508	transcription initiation factor iiB	No	0		1819988	1820965	+		Methanobacterium_AL_21_uid63623	NC_015216	chromosome		arCOG01981	8 (-)	K	SUA7	Transcription initiation factor TFIIB, Brf1 subunit/Tra	3E-122	COG1405	Transcription initiation factor TFIIIB,
325959509	hypothetical protein Metbo_1781	No	0		1821246	1821350	-		Methanobacterium_AL_21_uid63623	NC_015216	chromosome		arCOG10949	1	S		Uncharacterized conserved protein			
325959510	hypothetical protein Metbo_1782	No	0		1821987	1822283	+		Methanobacterium_AL_21_uid63623	NC_015216	chromosome									
===								5	Methanobacterium_AL_21_uid63623	NC_015216	chromosome									
325959625	hypothetical protein Metbo_1899	No	0		1950039	1950218	-		Methanobacterium_AL_21_uid63623	NC_015216	chromosome									
325959626	hypothetical protein Metbo_1900	No	0		1950303	1950740	-		Methanobacterium_AL_21_uid63623	NC_015216	chromosome									
325959627	thermosome	No	0		1950905	1952644	-		Methanobacterium_AL_21_uid63623	NC_015216	chromosome		arCOG01257	3 (-)	O	GroL	Chaperonin GroEL (HSP60 family)	4E-160	pfam00118	TCP-1/cpn60 chaperonin family. This fami
325959628	hypothetical protein Metbo_1902	No	2		1952886	1953296	-		Methanobacterium_AL_21_uid63623	NC_015216	chromosome		arCOG10883	5	S		Uncharacterized conserved protein			
325959629	hypothetical protein Metbo_1903	No	0		1953375	1953911	-		Methanobacterium_AL_21_uid63623	NC_015216	chromosome		arCOG10254	1	S		Uncharacterized conserved protein	0.00000003	COG0419	ATPase involved in DNA repair
===								6	Methanobacterium_AL_21_uid63623	NC_015216	chromosome									
325959692	NiL domain-containing protein	No	0		2018844	2019230	+		Methanobacterium_AL_21_uid63623	NC_015216	chromosome		arCOG02460	1 (-)	C	NapF	Ferredoxin	0.000000008	COG1142	Fe-S-cluster-containing hydrogenase comp
325959693	hypothetical protein Metbo_1967	Yes	1		2019440	2019871	-		Methanobacterium_AL_21_uid63623	NC_015216	chromosome									
325959694	hypothetical protein Metbo_1968	No	0		2019952	2021079	-		Methanobacterium_AL_21_uid63623	NC_015216	chromosome									
325959695	hypothetical protein Metbo_1969	Yes	1		2021187	2021630	-		Methanobacterium_AL_21_uid63623	NC_015216	chromosome									
325959696	hypothetical protein Metbo_1970	Yes	0		2021744	2022187	-		Methanobacterium_AL_21_uid63623	NC_015216	chromosome									
325959697	cobalt ABC transporter ATPase subunit	No	0		2022673	2023512	-		Methanobacterium_AL_21_uid63623	NC_015216	chromosome		arCOG00203	1 (-)	P	CbiO	ABC-type cobalt transport system, ATPase comp	1E-79	COG1122	ABC-type cobalt transport system, ATPase
===								6	Methanobacterium_AL_21_uid63623	NC_015216	chromosome									
325959720	hypothetical protein Metbo_1994	No	1		2042303	2042920	+		Methanobacterium_AL_21_uid63623	NC_015216	chromosome		arCOG06499	1 (-)	S		Uncharacterized conserved protein			
325959721	hypothetical protein Metbo_1996	No	4		2043329	2043700	+		Methanobacterium_AL_21_uid63623	NC_015216	chromosome									
325959722	hypothetical protein Metbo_1997	No	1		2043769	2043936	-		Methanobacterium_AL_21_uid63623	NC_015216	chromosome									
325959723	hypothetical protein Metbo_1998	Yes	1		2044502	2045503	-		Methanobacterium_AL_21_uid63623	NC_015216	chromosome		arCOG10865	9	S		Uncharacterized conserved protein	0.000003	pfam13287	Fn3 associated. ; Bacterial Ig-like domain.
325959724	hypothetical protein Metbo_1999	Yes	2		2045598	2046155	-		Methanobacterium_AL_21_uid63623	NC_015216	chromosome									
325959725	hypothetical protein Metbo_2000	Yes	1		2046830	2047207	+		Methanobacterium_AL_21_uid63623	NC_015216	chromosome		arCOG05052	1 (-)	R		Predicted RNA-binding protein, contains TRAM domain			
===								12	Methanobacterium_AL_21_uid63623	NC_015216	chromosome									
325959872	hypothetical protein Metbo_2151	No	0		2190167	2190664	+		Methanobacterium_AL_21_uid63623	NC_015216	chromosome									
325959873	hypothetical protein Metbo_2152	No	0		2190673	2191503	-		Methanobacterium_AL_21_uid63623	NC_015216	chromosome									
325959874	type ii site-specific deoxyribonuclease	No	0		2191505	2194570	-		Methanobacterium_AL_21_uid63623	NC_015216	chromosome	arCOG02635,a	arCOG02635	1 (-)	V		Type II restriction enzyme, methylase subunit	8E-10	COG2810	Predicted type IV restriction endonuclea ; N-6 DNA Methylase. Restriction-modificat
325959875	hypothetical protein Metbo_2154	No	0		2194731	2195771	-		Methanobacterium_AL_21_uid63623	NC_015216	chromosome		arCOG11374	2	S		Uncharacterized conserved protein	0.000003	pfam11185	Protein of unknown function (DUF2971). T
325959876	hypothetical protein Metbo_2155	No	0		2196159	2196359	+		Methanobacterium_AL_21_uid63623	NC_015216	chromosome									
325959877	hypothetical protein Metbo_2156	No	0		2196361	2196774	+		Methanobacterium_AL_21_uid63623	NC_015216	chromosome									
325959878	phage/plasmid primase, P4 family	No	0		2197100	2198854	+		Methanobacterium_AL_21_uid63623	NC_015216	chromosome		arCOG06914	10 (-)	R		Phage or plasmid associated DNA primase	4E-29	COG3378	Phage associated DNA primase
325959879	hypothetical protein Metbo_2158	Yes	1		2199367	2199765	+		Methanobacterium_AL_21_uid63623	NC_015216	chromosome									
325959880	polymorphic outer membrane protein	No	0		2199991	2202903	-		Methanobacterium_AL_21_uid63623	NC_015216	chromosome		arCOG02555	3 (-)	S		NosD-like periplasmic protein	1E-12	pfam13205	Bacterial Ig-like domain. ; Fn3 associated. ; Fn3 associated. ; Fn3 associated. ; Fn3 associated. ; Fn3 associated. ; Periplasmic copper-binding protein (NosD
325959881	hypothetical protein Metbo_2160	No	0		2203118	2204155	-		Methanobacterium_AL_21_uid63623	NC_015216	chromosome									
325959882	hypothetical protein Metbo_2161	No	0		2204267	2204656	-		Methanobacterium_AL_21_uid63623	NC_015216	chromosome									
325959883	hypothetical protein Metbo_2162	No	0		2204646	2204798	-		Methanobacterium_AL_21_uid63623	NC_015216	chromosome									
===								14	Methanobacterium_AL_21_uid63623	NC_015216	chromosome									
325960025	polymorphic outer membrane protein	Yes	1		2343360	2345138	-		Methanobacterium_AL_21_uid63623	NC_015216	chromosome		arCOG02555	3 (-)	S		NosD-like periplasmic protein	1E-16	pfam13229	Right handed beta helix region. This reg ; Right handed beta helix region. This reg
325960026	hypothetical protein Metbo_2306	No	2		2346098	2346415	+		Methanobacterium_AL_21_uid63623	NC_015216	chromosome	clust_334								
325960027	hypothetical protein Metbo_2307	No	0		2346483	2346854	+		Methanobacterium_AL_21_uid63623	NC_015216	chromosome									
325960028	hypothetical protein Metbo_2308	No	0		2346945	2347301	-		Methanobacterium_AL_21_uid63623	NC_015216	chromosome									
325960029	hypothetical protein Metbo_2309	No	0		2347406	2347783	-		Methanobacterium_AL_21_uid63623	NC_015216	chromosome									
325960030	hypothetical protein Metbo_2310	Yes	0		2348505	2349446	-		Methanobacterium_AL_21_uid63623	NC_015216	chromosome									
325960031	hypothetical protein Metbo_2311	Yes	1		2349734	2349913	-		Methanobacterium_AL_21_uid63623	NC_015216	chromosome							0.00008	pfam13205	Bacterial Ig-like domain.
325960032	hypothetical protein Metbo_2312	No	0		2350416	2350700	-		Methanobacterium_AL_21_uid63623	NC_015216	chromosome									
325960033	hypothetical protein Metbo_2313	No	1		2351939	2352094	-		Methanobacterium_AL_21_uid63623	NC_015216	chromosome									
325960034	hypothetical protein Metbo_2314	No	4		2352126	2352500	-		Methanobacterium_AL_21_uid63623	NC_015216	chromosome									
325960035	hypothetical protein Metbo_2315	Yes	1		2352608	2358040	-		Methanobacterium_AL_21_uid63623	NC_015216	chromosome	arCOG02526,a	arCOG02526	1 (-)	S		NosD-like periplasmic protein	3E-14	pfam05048	Periplasmic copper-binding protein (NosD ; Periplasmic copper-binding protein (NosD ; Bacterial Ig-like domain. ; Bacterial Ig-like domain. ; Bacterial Ig-like domain. ; Bacterial Ig-like domain. ; Bacterial Ig-like domain.
325960036	hypothetical protein Metbo_2316	Yes	1		2358264	2359583	-		Methanobacterium_AL_21_uid63623	NC_015216	chromosome		arCOG10865	9	S		Uncharacterized conserved protein			
325960037	hypothetical protein Metbo_2317	No	3		2360120	2360719	+		Methanobacterium_AL_21_uid63623	NC_015216	chromosome									
325960038	PASTA domain containing protein	No	3		2360729	2361571	+		Methanobacterium_AL_21_uid63623	NC_015216	chromosome									
===								5	Methanobacterium_AL_21_uid63623	NC_015216	chromosome									
325960137	hypothetical protein Metbo_2418	No	0		2460553	2460807	-		Methanobacterium_AL_21_uid63623	NC_015216	chromosome									
325960138	hypothetical protein Metbo_2419	No	0		2461108	2461362	-		Methanobacterium_AL_21_uid63623	NC_015216	chromosome		arCOG10208	1	S		Uncharacterized conserved protein			
325960139	1-(5-phosphoribosyl)-5-amino-4-imidazole-carb	No	0		2461458	2462222	+		Methanobacterium_AL_21_uid63623	NC_015216	chromosome		arCOG02465	1 (-)	R		NCAIR mutase (PurE)-related protein	5E-82	COG1691	NCAIR mutase (PurE)-related proteins
325960140	hypothetical protein Metbo_2421	No	5		2462251	2462751	-		Methanobacterium_AL_21_uid63623	NC_015216	chromosome		arCOG10684	1	S		Uncharacterized conserved protein			
325960141	hypothetical protein Metbo_2422	No	4		2462797	2463168	-		Methanobacterium_AL_21_uid63623	NC_015216	chromosome		arCOG10440	1	S		Uncharacterized conserved protein			
===								6	Methanobacterium_SWAN_1_uid67359	NC_015574	chromosome									
333986333	hypothetical protein MSWAN_0092	No	4		111032	111400	+		Methanobacterium_SWAN_1_uid67359	NC_015574	chromosome		arCOG10898	4	S		Uncharacterized conserved protein			
333986334	hypothetical protein MSWAN_0093	No	3		111493	111843	+		Methanobacterium_SWAN_1_uid67359	NC_015574	chromosome		arCOG10898	4	S		Uncharacterized conserved protein			
333986335	hypothetical protein MSWAN_0094	No	3		111998	112396	+		Methanobacterium_SWAN_1_uid67359	NC_015574	chromosome	clust_246						0.00002	pfam03866	Hydrophobic abundant protein (HAP). Expr
333986336	hypothetical protein MSWAN_0095	No	0		112561	112977	-		Methanobacterium_SWAN_1_uid67359	NC_015574	chromosome		arCOG04279	2 (-)	C		Swiveling domain associated with predicted acor	5E-45	COG1786	Swiveling domain associated with predict
333986337	hypothetical protein MSWAN_0096	No	6		113259	114164	-		Methanobacterium_SWAN_1_uid67359	NC_015574	chromosome		arCOG10162	4	S		Uncharacterized conserved protein			
333986338	hypothetical protein MSWAN_0097	No	7		114426	115334	-		Methanobacterium_SWAN_1_uid67359	NC_015574	chromosome		arCOG10162	4	S		Uncharacterized conserved protein			
===								5	Methanobacterium_SWAN_1_uid67359	NC_015574	chromosome									
333986512	hypothetical protein MSWAN_0272	No	4		308664	309062	+		Methanobacterium_SWAN_1_uid67359	NC_015574	chromosome									
333986513	hypothetical protein MSWAN_0273	No	0		309562	309735	-		Methanobacterium_SWAN_1_uid67359	NC_015574	chromosome									
333986514	prophage antirepressor	No	0		310826	311590	+		Methanobacterium_SWAN_1_uid67359	NC_015574	chromosome							2E-13	COG3617	Prophage antirepressor
333986515	DNA methylase N-4/N-6 domain-containing pro	No	0		311826	312533	+		Methanobacterium_SWAN_1_uid67359	NC_015574	chromosome		arCOG00115	10 (-)	L		DNA modification methylase	6E-23	COG0863	DNA modification methylase
333986516	hypothetical protein MSWAN_0278	No	0		312720	313082	-		Methanobacterium_SWAN_1_uid67359	NC_015574	chromosome									
===								10	Methanobacterium_SWAN_1_uid67359	NC_015574	chromosome									
333986860	catalase-peroxidase	No	0		680803	682977	-		Methanobacterium_SWAN_1_uid67359	NC_015574	chromosome		arCOG04487	2 (-)	P	KatG	Catalase (peroxidase I)		COG0376	Catalase (peroxidase I)
333986861	hypothetical protein MSWAN_0631	No	0		683012	683191	-		Methanobacterium_SWAN_1_uid67359	NC_015574	chromosome									
333986862	hypothetical protein MSWAN_0632	No	0		683575	685617	+		Methanobacterium_SWAN_1_uid67359	NC_015574	chromosome									
333986863	hypothetical protein MSWAN_0633	No	0		685673	686380	-		Methanobacterium_SWAN_1_uid67359	NC_015574	chromosome							0.000004	pfam09687	Plasmodium RESA N-terminal. The short, f
333986864	hypothetical protein MSWAN_0634	No	0		686487	686651	+		Methanobacterium_SWAN_1_uid67359	NC_015574	chromosome									
333986865	hypothetical protein MSWAN_0635	No	0		686679	687458	-		Methanobacterium_SWAN_1_uid67359	NC_015574	chromosome									
333986866	hypothetical protein MSWAN_0636	No	3		687521	688792	-		Methanobacterium_SWAN_1_uid67359	NC_015574	chromosome		arCOG03127	3 (-)	P	Kch	Ion channel fused to ion transporting domain			
333986867	hypothetical protein MSWAN_0637	No	3		688951	689454	+		Methanobacterium_SWAN_1_uid67359	NC_015574	chromosome									
333986868	hypothetical protein MSWAN_0638	No	0		689455	690261	+		Methanobacterium_SWAN_1_uid67359	NC_015574	chromosome									
333986869	hypothetical protein MSWAN_0639	No	0		690290	690796	+		Methanobacterium_SWAN_1_uid67359	NC_015574	chromosome									
===								10	Methanobacterium_SWAN_1_uid67359	NC_015574	chromosome									
333987065	hypothetical protein MSWAN_0844	No	0		936342	936542	+		Methanobacterium_SWAN_1_uid67359	NC_015574	chromosome									
333987066	hypothetical protein MSWAN_0845	No	0		936815	937054	+		Methanobacterium_SWAN_1_uid67359	NC_015574	chromosome									
333987067	hypothetical protein MSWAN_0846	No	0		937380	937874	+		Methanobacterium_SWAN_1_uid67359	NC_015574	chromosome									
333987068	CDGSH-type iron sulfur domain-containing prot	No	0		938106	938795	+		Methanobacterium_SWAN_1_uid67359	NC_015574	chromosome		arCOG03174	2 (-)	S		Zinc finger domain containing protein (CDGSH-typ	1E-14	COG3369	Zinc finger domain containing protein (C ; Zinc finger domain containing protein (C
333987069	hypothetical protein MSWAN_0848	No	0		939643	939834	+		Methanobacterium_SWAN_1_uid67359	NC_015574	chromosome									
333987070	hypothetical protein MSWAN_0849	No	3		939867	940274	+		Methanobacterium_SWAN_1_uid67359	NC_015574	chromosome	clust_259								
333987071	hypothetical protein MSWAN_0850	No	0		940620	940919	+		Methanobacterium_SWAN_1_uid67359	NC_015574	chromosome									
333987072	hypothetical protein MSWAN_0851	No	4		940992	941375	-		Methanobacterium_SWAN_1_uid67359	NC_015574	chromosome		arCOG11271	3	S		Uncharacterized conserved protein			
333987073	response regulator receiver	No	0		941560	942132	-		Methanobacterium_SWAN_1_uid67359	NC_015574	chromosome		arCOG02589	3 (-)	T	CheY	Rec domain			
333987074	low temperature requirement A	No	10		942391	943590	-		Methanobacterium_SWAN_1_uid67359	NC_015574	chromosome		arCOG10808	2	S		Uncharacterized conserved protein	1E-39	pfam06772	Bacterial low temperature requirement A
===								6	Methanobacterium_SWAN_1_uid67359	NC_015574	chromosome									
333987177	PfkB domain-containing protein	No	0		1045290	1046249	+		Methanobacterium_SWAN_1_uid67359	NC_015574	chromosome		arCOG00014	1 (-)	G	RbsK	Sugar kinase, ribokinase family	4E-34	COG0524	Sugar kinases, ribokinase family
333987178	hypothetical protein MSWAN_0960	No	0		1046659	1046847	+		Methanobacterium_SWAN_1_uid67359	NC_015574	chromosome									
333987179	hypothetical protein MSWAN_0961	No	0		1047119	1047388	+		Methanobacterium_SWAN_1_uid67359	NC_015574	chromosome									
333987180	hypothetical protein MSWAN_0962	No	4		1047430	1047804	-		Methanobacterium_SWAN_1_uid67359	NC_015574	chromosome									
333987181	hypothetical protein MSWAN_0963	No	0		1048097	1048324	+		Methanobacterium_SWAN_1_uid67359	NC_015574	chromosome	clust_423								
333987182	4Fe-4S ferredoxin iron-sulfur binding domain-c	No	0		1048633	1049397	+		Methanobacterium_SWAN_1_uid67359	NC_015574	chromosome		arCOG02179	1 (-)	C	NapF	Polyferredoxin	3E-13	COG1145	Ferredoxin
===								6	Methanobacterium_SWAN_1_uid67359	NC_015574	chromosome									
333987196	SagB-type dehydrogenase domain-containing	Yes	1		1068650	1069375	-		Methanobacterium_SWAN_1_uid67359	NC_015574	chromosome		arCOG00288	8 (-)	C	NfnB	Nitroreductase	2E-13	pfam00881	Nitroreductase family. The nitroreductas
333987197	hypothetical protein MSWAN_0979	No	3		1069671	1069979	+		Methanobacterium_SWAN_1_uid67359	NC_015574	chromosome		arCOG10259	2	S		Uncharacterized conserved protein			
333987198	hypothetical protein MSWAN_0980	No	3		1070023	1070334	+		Methanobacterium_SWAN_1_uid67359	NC_015574	chromosome		arCOG10259	2	S		Uncharacterized conserved protein			
333987199	hypothetical protein MSWAN_0981	Yes	1		1070826	1071860	+		Methanobacterium_SWAN_1_uid67359	NC_015574	chromosome							0.00006	pfam11611	Domain of unknown function (DUF4352). Me
333987200	hypothetical protein MSWAN_0982	No	0		1072802	1073104	+		Methanobacterium_SWAN_1_uid67359	NC_015574	chromosome							0.00000001	pfam09538	Protein of unknown function (FYDLN_acid)
333987201	metal dependent phosphohydrolase	No	0		1073508	1074413	+		Methanobacterium_SWAN_1_uid67359	NC_015574	chromosome		arCOG01861	3 (-)	H	MptB	Methanopterin biosynthesis cyclic phosphodieste	9E-36	COG3481	Predicted HD-superfamily hydrolase
===								9	Methanobacterium_SWAN_1_uid67359	NC_015574	chromosome									
333987203	cyclase/dehydrase	No	0		1075384	1075668	+		Methanobacterium_SWAN_1_uid67359	NC_015574	chromosome		arCOG05403	1 (-)	C	OdsN	Predicted heme-degrading monooxygenase			
333987204	hypothetical protein MSWAN_0986	No	0		1075827	1076054	-		Methanobacterium_SWAN_1_uid67359	NC_015574	chromosome	clust_423								
333987205	hypothetical protein MSWAN_0987	No	0		1076168	1076380	-		Methanobacterium_SWAN_1_uid67359	NC_015574	chromosome									
333987206	hypothetical protein MSWAN_0988	No	0		1076752	1076913	-		Methanobacterium_SWAN_1_uid67359	NC_015574	chromosome									
333987207	hypothetical protein MSWAN_0989	No	0		1077188	1077454	-		Methanobacterium_SWAN_1_uid67359	NC_015574	chromosome									
333987208	hypothetical protein MSWAN_0990	No	0		1077640	1077792	+		Methanobacterium_SWAN_1_uid67359	NC_015574	chromosome									
333987209	hypothetical protein MSWAN_0991	No	0		1078067	1078384	-		Methanobacterium_SWAN_1_uid67359	NC_015574	chromosome									
333987210	hypothetical protein MSWAN_0992	Yes	1		1078870	1079310	+		Methanobacterium_SWAN_1_uid67359	NC_015574	chromosome									
333987211	hypothetical protein MSWAN_0994	No	0		1079904	1080143	-		Methanobacterium_SWAN_1_uid67359	NC_015574	chromosome		arCOG03597	4 (-)	S		Uncharacterized conserved protein	0.0000002	pfam10967	Protein of unknown function (DUF2769). T
===								8	Methanobacterium_SWAN_1_uid67359	NC_015574	chromosome									
333987265	hypothetical protein MSWAN_1050	No	1		1137038	1138129	-		Methanobacterium_SWAN_1_uid67359	NC_015574	chromosome		arCOG10250	2	S		Uncharacterized conserved protein			
333987266	hypothetical protein MSWAN_1051	No	1		1138142	1140283	-		Methanobacterium_SWAN_1_uid67359	NC_015574	chromosome		arCOG10249	3	S		Uncharacterized conserved protein			
333987267	hypothetical protein MSWAN_1052	No	1		1140293	1141213	-		Methanobacterium_SWAN_1_uid67359	NC_015574	chromosome		arCOG07613	2 (-)	S		Uncharacterized conserved protein			

333987268	hypothetical protein MSWAN_1053	No	1			1141434	1141838	+		Methanobacterium_SWAN_1_uid67359	NC_015574	chromosome		arCOG10248	3	S		Uncharacterized conserved protein			
333987269	hypothetical protein MSWAN_1054	No	2			1141901	1142116	+		Methanobacterium_SWAN_1_uid67359	NC_015574	chromosome		arCOG10251	2	S		Uncharacterized conserved protein			
333987270	cellulase	No	0			1142339	1143367	+		Methanobacterium_SWAN_1_uid67359	NC_015574	chromosome		arCOG01518	2 (-)	G	FrvX	Cellulase M or related protein	5E-112	COG1363	Cellulase M and related proteins
333987271	hypothetical protein MSWAN_1056	Yes	1			1143993	1144325	+		Methanobacterium_SWAN_1_uid67359	NC_015574	chromosome		arCOG10865	9	S		Uncharacterized conserved protein			
333987272	hypothetical protein MSWAN_1057	Yes	0			1144444	1144980	+		Methanobacterium_SWAN_1_uid67359	NC_015574	chromosome		arCOG10865	9	S		Uncharacterized conserved protein			
===									8	Methanobacterium_SWAN_1_uid67359	NC_015574	chromosome									
333987313	hypothetical protein MSWAN_1099	No	0			1194314	1194529	-		Methanobacterium_SWAN_1_uid67359	NC_015574	chromosome		arCOG10252	1	S		Uncharacterized conserved protein			
333987314	hypothetical protein MSWAN_1100	No	0			1194748	1195341	+		Methanobacterium_SWAN_1_uid67359	NC_015574	chromosome		arCOG03038	9 (-)	R	NrfG	TPR repeats containing protein	5E-30	COG0457	FOG: TPR repeat
333987315	hypothetical protein MSWAN_1101	No	0			1195664	1196023	+		Methanobacterium_SWAN_1_uid67359	NC_015574	chromosome									
333987316	hypothetical protein MSWAN_1102	No	0			1196261	1196581	-		Methanobacterium_SWAN_1_uid67359	NC_015574	chromosome									
333987317	hypothetical protein MSWAN_1103	No	0			1196988	1197302	+		Methanobacterium_SWAN_1_uid67359	NC_015574	chromosome									
333987318	integrase family protein	No	0			1197502	1198641	-		Methanobacterium_SWAN_1_uid67359	NC_015574	chromosome		arCOG01245	15 (-)	L	XerC	Integrase			
333987319	hypothetical protein MSWAN_1105	No	3			1199148	1199462	+		Methanobacterium_SWAN_1_uid67359	NC_015574	chromosome									
333987320	hypothetical protein MSWAN_1106	No	0			1199523	1199681	+		Methanobacterium_SWAN_1_uid67359	NC_015574	chromosome									
===									5	Methanobacterium_SWAN_1_uid67359	NC_015574	chromosome									
333987324	glutamate--cysteine ligase	No	0			1202768	1204240	-		Methanobacterium_SWAN_1_uid67359	NC_015574	chromosome		arCOG10328	1	S		Uncharacterized conserved protein	6E-111	COG2918	Gamma-glutamylcysteine synthetase
333987325	Ion transport 2 domain-containing protein	No	7			1204548	1205342	-		Methanobacterium_SWAN_1_uid67359	NC_015574	chromosome		arCOG01964	3 (-)	P	Kch	Kef-type K+ transport system, predicted NAD-bin	0.000000005	pfam07885	Ion channel. This family includes the tw
333987326	hypothetical protein MSWAN_1113	No	0			1205484	1205708	+		Methanobacterium_SWAN_1_uid67359	NC_015574	chromosome									
333987327	hypothetical protein MSWAN_1114	No	0			1206077	1206364	+		Methanobacterium_SWAN_1_uid67359	NC_015574	chromosome		arCOG10257	2	S		Uncharacterized conserved protein			
333987328	hypothetical protein MSWAN_1115	Yes	1			1206874	1207314	+		Methanobacterium_SWAN_1_uid67359	NC_015574	chromosome									
===									5	Methanobacterium_SWAN_1_uid67359	NC_015574	chromosome									
333987332	hypothetical protein MSWAN_1119	No	0			1213064	1214227	+		Methanobacterium_SWAN_1_uid67359	NC_015574	chromosome									
333987333	flavodoxin/nitric oxide synthase	No	0			1214371	1215546	+		Methanobacterium_SWAN_1_uid67359	NC_015574	chromosome		arCOG00509	3 (-)	C	FpaA	Uncharacterized flavoprotein	3E-91	COG0426	Uncharacterized flavoproteins
333987334	hypothetical protein MSWAN_1121	No	0			1215683	1215949	-		Methanobacterium_SWAN_1_uid67359	NC_015574	chromosome		arCOG10373	1	S		Uncharacterized conserved protein			
333987335	hypothetical protein MSWAN_1122	No	1			1216836	1217312	-		Methanobacterium_SWAN_1_uid67359	NC_015574	chromosome									
333987336	hypothetical protein MSWAN_1123	No	0			1217749	1218078	+		Methanobacterium_SWAN_1_uid67359	NC_015574	chromosome	clust_43								
===									13	Methanobacterium_SWAN_1_uid67359	NC_015574	chromosome									
333987340	Potassium-transporting ATPase subunit A	No	11			1222859	1224565	-		Methanobacterium_SWAN_1_uid67359	NC_015574	chromosome		arCOG04804	1 (-)	P	KdpA	K+-transporting ATPase, A chain		COG2060	K+-transporting ATPase, A chain
333987341	hypothetical protein MSWAN_1128	No	1			1225401	1225547	+		Methanobacterium_SWAN_1_uid67359	NC_015574	chromosome									
333987342	hypothetical protein MSWAN_1129	No	2			1225972	1226259	+		Methanobacterium_SWAN_1_uid67359	NC_015574	chromosome		arCOG10455	2	S		Uncharacterized conserved protein			
333987343	hypothetical protein MSWAN_1130	No	3			1226429	1226686	+		Methanobacterium_SWAN_1_uid67359	NC_015574	chromosome									
333987344	hypothetical protein MSWAN_1131	No	0			1226909	1227286	-		Methanobacterium_SWAN_1_uid67359	NC_015574	chromosome		arCOG10865	9	S		Uncharacterized conserved protein			
333987345	regulatory protein MarR	No	0			1227872	1228354	-		Methanobacterium_SWAN_1_uid67359	NC_015574	chromosome		arCOG03177	3 (-)	K	MarR	Transcriptional regulator, MarR family	0.00005	COG1846	Transcriptional regulators
333987346	glyoxalase/bleomycin resistance protein/dioxy	No	0			1228377	1228736	-		Methanobacterium_SWAN_1_uid67359	NC_015574	chromosome		arCOG02708	2 (-)	E	GloA	Lactoylglutathione lyase or related enzyme	0.0000001	pfam12681	Glyoxalase-like domain. This domain is r
333987347	hypothetical protein MSWAN_1135	No	0			1230329	1230844	-		Methanobacterium_SWAN_1_uid67359	NC_015574	chromosome									
333987348	hypothetical protein MSWAN_1136	No	1			1230923	1231183	-		Methanobacterium_SWAN_1_uid67359	NC_015574	chromosome									
333987349	hypothetical protein MSWAN_1137	No	2			1231210	1231404	-		Methanobacterium_SWAN_1_uid67359	NC_015574	chromosome									
333987350	hypothetical protein MSWAN_1138	No	0			1231920	1232294	+		Methanobacterium_SWAN_1_uid67359	NC_015574	chromosome	clust_43								
333987351	hypothetical protein MSWAN_1139	No	0			1232504	1233217	-		Methanobacterium_SWAN_1_uid67359	NC_015574	chromosome									
333987352	signal transduction histidine kinase	No	0			1233603	1234649	+		Methanobacterium_SWAN_1_uid67359	NC_015574	chromosome	arCOG02335,a	arCOG02335	2 (-)	T		Signal transduction histidine kinase, contains PAS	9E-27	COG3920	Signal transduction histidine kinase
===									10	Methanobacterium_SWAN_1_uid67359	NC_015574	chromosome									
333987359	phosphonate-transporting ATPase	No	0			1238938	1239633	+		Methanobacterium_SWAN_1_uid67359	NC_015574	chromosome		arCOG00922	4 (-)	V	SalX	ABC-type antimicrobial peptide transport system,	3E-70	COG1136	ABC-type antimicrobial peptide transport
333987360	hypothetical protein MSWAN_1148	No	0			1239971	1240270	+		Methanobacterium_SWAN_1_uid67359	NC_015574	chromosome	clust_349								
333987361	hypothetical protein MSWAN_1149	No	0			1240331	1240603	-		Methanobacterium_SWAN_1_uid67359	NC_015574	chromosome									
333987362	hypothetical protein MSWAN_1150	No	0			1240871	1241137	-		Methanobacterium_SWAN_1_uid67359	NC_015574	chromosome									
333987363	hypothetical protein MSWAN_1151	No	0			1241390	1241695	+		Methanobacterium_SWAN_1_uid67359	NC_015574	chromosome	clust_349								
333987364	hypothetical protein MSWAN_1152	No	0			1241979	1242260	-		Methanobacterium_SWAN_1_uid67359	NC_015574	chromosome									
333987365	hypothetical protein MSWAN_1153	No	0			1242816	1243085	-		Methanobacterium_SWAN_1_uid67359	NC_015574	chromosome									
333987366	hypothetical protein MSWAN_1154	No	0			1243486	1243857	-		Methanobacterium_SWAN_1_uid67359	NC_015574	chromosome									
333987367	hypothetical protein MSWAN_1155	No	0			1243861	1244016	-		Methanobacterium_SWAN_1_uid67359	NC_015574	chromosome									
333987368	major facilitator superfamily protein	No	10			1244631	1245800	-		Methanobacterium_SWAN_1_uid67359	NC_015574	chromosome		arCOG00130	5 (-)	G	ProP	Permease of the major facilitator superfamily	8E-33	pfam07690	Major Facilitator Superfamily.
===									5	Methanobacterium_SWAN_1_uid67359	NC_015574	chromosome									
333987370	hypothetical protein MSWAN_1158	No	0			1246860	1247021	-		Methanobacterium_SWAN_1_uid67359	NC_015574	chromosome									
333987371	hypothetical protein MSWAN_1159	No	3			1247152	1247547	-		Methanobacterium_SWAN_1_uid67359	NC_015574	chromosome	clust_259								
333987372	hypothetical protein MSWAN_1160	No	0			1247573	1248118	-		Methanobacterium_SWAN_1_uid67359	NC_015574	chromosome									
333987373	response regulator receiver	No	0			1248807	1249061	+		Methanobacterium_SWAN_1_uid67359	NC_015574	chromosome		arCOG06537	1 (-)	T	CheY	Rec and PAS domains	0.000000001	COG2197	Response regulator containing a CheY-lik
333987374	hypothetical protein MSWAN_1162	No	0			1249237	1249578	-		Methanobacterium_SWAN_1_uid67359	NC_015574	chromosome									
===									5	Methanobacterium_SWAN_1_uid67359	NC_015574	chromosome									
333987408	hypothetical protein MSWAN_1197	No	0			1278771	1278923	-		Methanobacterium_SWAN_1_uid67359	NC_015574	chromosome									
333987409	hypothetical protein MSWAN_1198	No	5			1279871	1280323	+		Methanobacterium_SWAN_1_uid67359	NC_015574	chromosome									
333987410	hypothetical protein MSWAN_1199	No	3			1280544	1280885	+		Methanobacterium_SWAN_1_uid67359	NC_015574	chromosome									
333987411	nitroreductase	No	0			1281194	1281790	-		Methanobacterium_SWAN_1_uid67359	NC_015574	chromosome		arCOG00288	8 (-)	C	NfnB	Nitroreductase	3E-21	COG0778	Nitroreductase
333987412	hypothetical protein MSWAN_1201	No	0			1282108	1282539	+		Methanobacterium_SWAN_1_uid67359	NC_015574	chromosome	clust_241								
===									6	Methanobacterium_SWAN_1_uid67359	NC_015574	chromosome									
333987535	hypothetical protein MSWAN_1325	No	0			1423415	1423555	-		Methanobacterium_SWAN_1_uid67359	NC_015574	chromosome							0.0001	pfam07295	Protein of unknown function (DUF1451). T
333987536	hypothetical protein MSWAN_1327	No	0			1426734	1427060	+		Methanobacterium_SWAN_1_uid67359	NC_015574	chromosome	clust_319								
333987537	hypothetical protein MSWAN_1328	No	0			1427125	1427454	+		Methanobacterium_SWAN_1_uid67359	NC_015574	chromosome	clust_319								
333987538	polymorphic outer membrane protein	Yes	2			1428816	1430915	+		Methanobacterium_SWAN_1_uid67359	NC_015574	chromosome	arCOG09729,a	arCOG09729	1 (-)	R		Predicted solute binding protein	1E-13	COG3210	Large exoproteins involved in heme utili
333987539	hypothetical protein MSWAN_1330	No	0			1430998	1431123	-		Methanobacterium_SWAN_1_uid67359	NC_015574	chromosome									
333987540	hypothetical protein MSWAN_1331	No	0			1432051	1432797	-		Methanobacterium_SWAN_1_uid67359	NC_015574	chromosome		arCOG10246	1	S		Uncharacterized conserved protein			
===									7	Methanobacterium_SWAN_1_uid67359	NC_015574	chromosome									
333987612	hypothetical protein MSWAN_1404	No	0			1525715	1526071	+		Methanobacterium_SWAN_1_uid67359	NC_015574	chromosome	clust_43								
333987613	hypothetical protein MSWAN_1405	No	5			1526094	1526849	+		Methanobacterium_SWAN_1_uid67359	NC_015574	chromosome		arCOG02929	1 (-)	C	PetE	Plastocyanin			
333987614	hypothetical protein MSWAN_1406	No	0			1527016	1527132	+		Methanobacterium_SWAN_1_uid67359	NC_015574	chromosome									
333987615	hypothetical protein MSWAN_1407	Yes	1			1527205	1527612	+		Methanobacterium_SWAN_1_uid67359	NC_015574	chromosome									
333987616	hypothetical protein MSWAN_1408	Yes	0			1527927	1528274	-		Methanobacterium_SWAN_1_uid67359	NC_015574	chromosome		arCOG11306	1	S		Uncharacterized conserved protein			
333987617	peptidase C1A papain	No	0			1528860	1529774	-		Methanobacterium_SWAN_1_uid67359	NC_015574	chromosome	arCOG10865,a	arCOG10865	9	S		Uncharacterized conserved protein	2E-11	pfam13205	Bacterial Ig-like domain. ; Bacterial Ig-like domain. ; Bacterial Ig-like domain.
333987618	hypothetical protein MSWAN_1410	Yes	1			1529771	1530721	-		Methanobacterium_SWAN_1_uid67359	NC_015574	chromosome		arCOG06477	2 (-)	S		Uncharacterized conserved protein			
===									5	Methanobacterium_SWAN_1_uid67359	NC_015574	chromosome									
333987815	peptidase C39 bacteriocin processing	No	0		Virus_Caudovi	1735874	1736557	-		Methanobacterium_SWAN_1_uid67359	NC_015574	chromosome									
333987816	hypothetical protein MSWAN_1611	No	0		Virus_Caudovi	1736667	1737485	-		Methanobacterium_SWAN_1_uid67359	NC_015574	chromosome									
333987817	hypothetical protein MSWAN_1612	No	1		Virus_Caudovi	1737980	1738117	-		Methanobacterium_SWAN_1_uid67359	NC_015574	chromosome									
333987818	hypothetical protein MSWAN_1613	No	0		Virus_Caudovi	1738167	1738631	-		Methanobacterium_SWAN_1_uid67359	NC_015574	chromosome		arCOG02579	1 (-)	R		Fe-S-cluster containining protein			
333987819	hypothetical protein MSWAN_1614	No	0		Virus_Caudovi	1738669	1739085	-		Methanobacterium_SWAN_1_uid67359	NC_015574	chromosome		arCOG10865	9	S		Uncharacterized conserved protein			
===									76	Methanobacterium_SWAN_1_uid67359	NC_015574	chromosome									
333987821	hypothetical protein MSWAN_1616	No	0		Virus_Caudovi	1740973	1741512	-		Methanobacterium_SWAN_1_uid67359	NC_015574	chromosome									
333987822	hypothetical protein MSWAN_1617	No	0		Virus_Caudovi	1741526	1742707	-		Methanobacterium_SWAN_1_uid67359	NC_015574	chromosome									
333987823	hypothetical protein MSWAN_1618	No	0		Virus_Caudovi	1742700	1743986	-		Methanobacterium_SWAN_1_uid67359	NC_015574	chromosome		arCOG03509	2 (-)	R		Cell suface protein, containing PKD repeats	8E-11	pfam10102	Domain of unknown function (DUF2341). Me
333987824	hypothetical protein MSWAN_1619	No	0		Virus_Caudovi	1743999	1744433	-		Methanobacterium_SWAN_1_uid67359	NC_015574	chromosome									
333987825	hypothetical protein MSWAN_1620	No	0		Virus_Caudovi	1744451	1744792	-		Methanobacterium_SWAN_1_uid67359	NC_015574	chromosome									
333987826	hypothetical protein MSWAN_1621	No	0		Virus_Caudovi	1744789	1745589	-		Methanobacterium_SWAN_1_uid67359	NC_015574	chromosome									
333987827	phage tail tape measure protein, TP901 family	No	0		Virus_Caudovi	1745594	1751341	-		Methanobacterium_SWAN_1_uid67359	NC_015574	chromosome		arCOG11109	8	Y		Phage tail tape measure protein, TP901 family	2E-15	pfam10145	Phage-related minor tail protein. Member
333987828	hypothetical protein MSWAN_1623	Yes	1		Virus_Caudovi	1751439	1752035	+		Methanobacterium_SWAN_1_uid67359	NC_015574	chromosome		arCOG06468	2 (-)	S		Uncharacterized conserved protein			
333987829	hypothetical protein MSWAN_1624	No	0		Virus_Caudovi	1752044	1752250	-		Methanobacterium_SWAN_1_uid67359	NC_015574	chromosome									
333987830	hypothetical protein MSWAN_1625	No	0		Virus_Caudovi	1752256	1752786	-		Methanobacterium_SWAN_1_uid67359	NC_015574	chromosome									
333987831	hypothetical protein MSWAN_1626	No	0		Virus_Caudovi	1752873	1753640	-		Methanobacterium_SWAN_1_uid67359	NC_015574	chromosome									
333987832	hypothetical protein MSWAN_1627	No	0		Virus_Caudovi	1753647	1753841	-		Methanobacterium_SWAN_1_uid67359	NC_015574	chromosome									
333987833	hypothetical protein MSWAN_1628	No	0		Virus_Caudovi	1753929	1754396	-		Methanobacterium_SWAN_1_uid67359	NC_015574	chromosome									
333987834	hypothetical protein MSWAN_1629	No	0		Virus_Caudovi	1754393	1754827	-		Methanobacterium_SWAN_1_uid67359	NC_015574	chromosome									
333987835	hypothetical protein MSWAN_1630	No	0		Virus_Caudovi	1754828	1755217	-		Methanobacterium_SWAN_1_uid67359	NC_015574	chromosome									
333987836	hypothetical protein MSWAN_1631	No	0		Virus_Caudovi	1755219	1755608	-		Methanobacterium_SWAN_1_uid67359	NC_015574	chromosome									
333987837	hypothetical protein MSWAN_1632	No	0		Virus_Caudovi	1755621	1756154	-		Methanobacterium_SWAN_1_uid67359	NC_015574	chromosome									
333987838	hypothetical protein MSWAN_1633	No	0		Virus_Caudovi	1756202	1757086	-		Methanobacterium_SWAN_1_uid67359	NC_015574	chromosome							0.0001	pfam04454	Encapsulating protein for peroxidase. Th
333987839	hypothetical protein MSWAN_1634	No	0		Virus_Caudovi	1757102	1757566	-		Methanobacterium_SWAN_1_uid67359	NC_015574	chromosome									
333987840	hypothetical protein MSWAN_1635	No	0		Virus_Caudovi	1757572	1759527	-		Methanobacterium_SWAN_1_uid67359	NC_015574	chromosome									
333987841	hypothetical protein MSWAN_1636	No	0		Virus_Caudovi	1759566	1760333	-		Methanobacterium_SWAN_1_uid67359	NC_015574	chromosome									
333987842	hypothetical protein MSWAN_1637	No	0		Virus_Caudovi	1760311	1760946	-		Methanobacterium_SWAN_1_uid67359	NC_015574	chromosome									
333987843	hypothetical protein MSWAN_1638	No	0		Virus_Caudovi	1761033	1761314	+		Methanobacterium_SWAN_1_uid67359	NC_015574	chromosome		arCOG08550	2 (-)	V		Predicted antitoxin, copG family			
333987844	hypothetical protein MSWAN_1639	No	0		Virus_Caudovi	1761315	1761545	+		Methanobacterium_SWAN_1_uid67359	NC_015574	chromosome									
333987845	hypothetical protein MSWAN_1640	No	0		Virus_Caudovi	1761542	1761673	+		Methanobacterium_SWAN_1_uid67359	NC_015574	chromosome									
333987846	hypothetical protein MSWAN_1641	No	0		Virus_Caudovi	1761989	1762228	-		Methanobacterium_SWAN_1_uid67359	NC_015574	chromosome									
333987847	hypothetical protein MSWAN_1642	No	0		Virus_Caudovi	1762323	1763753	-		Methanobacterium_SWAN_1_uid67359	NC_015574	chromosome									
333987848	hypothetical protein MSWAN_1643	No	0		Virus_Caudovi	1763758	1765359	-		Methanobacterium_SWAN_1_uid67359	NC_015574	chromosome		arCOG09550	17	Y	TerL	Phage terminase large subunit	8E-10	COG5410	Uncharacterized protein conserved in bac
333987849	hypothetical protein MSWAN_1644	No	0		Virus_Caudovi	1765325	1765906	-		Methanobacterium_SWAN_1_uid67359	NC_015574	chromosome		arCOG11382	5	Y		Phage terminase small subunit			
333987850	hypothetical protein MSWAN_1645	No	0		Virus_Caudovi	1766033	1766287	-		Methanobacterium_SWAN_1_uid67359	NC_015574	chromosome									
333987851	hypothetical protein MSWAN_1646	No	1		Virus_Caudovi	1766281	1766580	-		Methanobacterium_SWAN_1_uid67359	NC_015574	chromosome									
333987852	hypothetical protein MSWAN_1647	No	2		Virus_Caudovi	1766623	1766991	-		Methanobacterium_SWAN_1_uid67359	NC_015574	chromosome									
333987853	hypothetical protein MSWAN_1648	No	0		Virus_Caudovi	1767281	1767499	-		Methanobacterium_SWAN_1_uid67359	NC_015574	chromosome									
333987854	hypothetical protein MSWAN_1649	No	0		Virus_Caudovi	1767655	1768260	-		Methanobacterium_SWAN_1_uid67359	NC_015574	chromosome		arCOG01981	8 (-)	K	SUA7	Transcription initiation factor TFIIB, Brf1 subunit/Transcription initiation factor TFIIB			
333987855	hypothetical protein MSWAN_1650	No	0		Virus_Caudovi	1768388	1769119	-		Methanobacterium_SWAN_1_uid67359	NC_015574	chromosome									
333987856	hypothetical protein MSWAN_1651	No	0		Virus_Caudovi	1769121	1769477	-		Methanobacterium_SWAN_1_uid67359	NC_015574	chromosome									
333987857	hypothetical protein MSWAN_1652	No	0		Virus_Caudovi	1769505	1769840	-		Methanobacterium_SWAN_1_uid67359	NC_015574	chromosome									
333987858	hypothetical protein MSWAN_1653	No	0		Virus_Caudovi	1769857	1769973	-		Methanobacterium_SWAN_1_uid67359	NC_015574	chromosome									
333987859	hypothetical protein MSWAN_1654	No	0		Virus_Caudovi	1770089	1770286	-		Methanobacterium_SWAN_1_uid67359	NC_015574	chromosome									
333987860	hypothetical protein MSWAN_1655	No	0		Virus_Caudovi	1770321	1770488	-		Methanobacterium_SWAN_1_uid67359	NC_015574	chromosome									
333987861	hypothetical protein MSWAN_1656	No	0		Virus_Caudovi	1770500	1770838	-		Methanobacterium_SWAN_1_uid67359	NC_015574	chromosome									
333987862	hypothetical protein MSWAN_1657	No	0		Virus_Caudovi	1770853	1771164	-		Methanobacterium_SWAN_1_uid67359	NC_015574	chromosome									
333987863	hypothetical protein MSWAN_1658	No	0		Virus_Caudovi	1771207	1771596	-		Methanobacterium_SWAN_1_uid67359	NC_015574	chromosome									
333987864	hypothetical protein MSWAN_1659	No	0		Virus_Caudovi	1771600	1771761	-		Methanobacterium_SWAN_1_uid67359	NC_015574	chromosome									
333987865	hypothetical protein MSWAN_1660	No	0		Virus_Caudovi	1771763	1771981	-		Methanobacterium_SWAN_1_uid67359	NC_015574	chromosome									
333987866	hypothetical protein MSWAN_1661	No	0		Virus_Caudovi	1771982	1772203	-		Methanobacterium_SWAN_1_uid67359	NC_015574	chromosome									
333987867	hypothetical protein MSWAN_1662	No	0		Virus_Caudovi	1772209	1772451	-		Methanobacterium_SWAN_1_uid67359	NC_015574	chromosome									
333987868	hypothetical protein MSWAN_1663	No	0		Virus_Caudovi	1772411	1772905	-		Methanobacterium_SWAN_1_uid67359	NC_015574	chromosome									
333987869	hypothetical protein MSWAN_1664	No	0		Virus_Caudovi	1772898	1773206	-		Methanobacterium_SWAN_1_uid67359	NC_015574	chromosome									
333987870	hypothetical protein MSWAN_1665	No	0		Virus_Caudovi	1773203	1773667	-		Methanobacterium_SWAN_1_uid67359	NC_015574	chromosome									
333987871	hypothetical protein MSWAN_1666	No	0		Virus_Caudovi	1773669	1773887	-		Methanobacterium_SWAN_1_uid67359	NC_015574	chromosome									
333987872	hypothetical protein MSWAN_1667	No	0		Virus_Caudovi	1773890	1774579	-		Methanobacterium_SWAN_1_uid67359	NC_015574	chromosome									
333987873	hypothetical protein MSWAN_1668	No	0		Virus_Caudovi	1774563	1774778	-		Methanobacterium_SWAN_1_uid67359	NC_015574	chromosome									
333987874	hypothetical protein MSWAN_1669	No	0		Virus_Caudovi	1774771	1775085	-		Methanobacterium_SWAN_1_uid67359	NC_015574	chromosome									
333987875	hypothetical protein MSWAN_1670	No	0		Virus_Caudovi	1775167	1775538	-		Methanobacterium_SWAN_1_uid67359	NC_015574	chromosome									
333987876	YopX protein	No	0		Virus_Caudovi	1775541	1776062	-		Methanobacterium_SWAN_1_uid67359	NC_015574	chromosome									
333987877	YopX protein	No	0		Virus_Caudovi	1776059	1776529	-		Methanobacterium_SWAN_1_uid67359	NC_015574	chromosome							0.000000009	pfam09643	YopX protein. YopX is a protein that is
333987878	hypothetical protein MSWAN_1673	No	0		Virus_Caudovi	1776542	1777090	-		Methanobacterium_SWAN_1_uid67359	NC_015574	chromosome									
333987879	hypothetical protein MSWAN_1674	No	0		Virus_Caudovi	1777087	1777353	-		Methanobacterium_SWAN_1_uid67359	NC_015574	chromosome									
333987880	hypothetical protein MSWAN_1675	No	0		Virus_Caudovi	1777340	1777720	-		Methanobacterium_SWAN_1_uid67359	NC_015574	chromosome									
333987881	hypothetical protein MSWAN_1676	No	0		Virus_Caudovi	1777750	1778322	-		Methanobacterium_SWAN_1_uid67359	NC_015574	chromosome									
333987882	hypothetical protein MSWAN_1677	No	0		Virus_Caudovi	1778325	1778732	-		Methanobacterium_SWAN_1_uid67359	NC_015574	chromosome							0.000006	pfam02463	RecF/RecN/SMC N terminal domain. This do
333987883	hypothetical protein MSWAN_1678	No	0		Virus_Caudovi	1778742	1778939	-		Methanobacterium_SWAN_1_uid67359	NC_015574	chromosome									
333987884	hypothetical protein MSWAN_1679	No	0		Virus_Caudovi	1778939	1779187	-		Methanobacterium_SWAN_1_uid67359	NC_015574	chromosome									
333987885	hypothetical protein MSWAN_1680	No	0		Virus_Caudovi	1779180	1779551	-		Methanobacterium_SWAN_1_uid67359	NC_015574	chromosome									
333987886	hypothetical protein MSWAN_1681	No	0		Virus_Caudovi	1779710	1779970	-		Methanobacterium_SWAN_1_uid67359	NC_015574	chromosome									
333987887	hypothetical protein MSWAN_1682	No	0		Virus_Caudovi	1779951	1780301	-		Methanobacterium_SWAN_1_uid67359	NC_015574	chromosome									
333987888	hypothetical protein MSWAN_1683	No	0		Virus_Caudovi	1780302	1780511	-		Methanobacterium_SWAN_1_uid67359	NC_015574	chromosome									
333987889	hypothetical protein MSWAN_1684	No	0		Virus_Caudovi	1780512	1780613	-		Methanobacterium_SWAN_1_uid67359	NC_015574	chromosome									
333987890	hypothetical protein MSWAN_1685	No	0		Virus_Caudovi	1780615	1781166	-		Methanobacterium_SWAN_1_uid67359	NC_015574	chromosome									
333987891	hypothetical protein MSWAN_1686	No	0		Virus_Caudovi	1781964	1782470	-		Methanobacterium_SWAN_1_uid67359	NC_015574	chromosome									
333987892	hypothetical protein MSWAN_1687	No	1		Virus_Caudovi	1782470	1782817	-		Methanobacterium_SWAN_1_uid67359	NC_015574	chromosome									
333987893	hypothetical protein MSWAN_1688	No	0		Virus_Caudovi	1783012	1783188	+		Methanobacterium_SWAN_1_uid67359	NC_015574	chromosome									
333987894	hypothetical protein MSWAN_1689	No	0		Virus_Caudovi	1783225	1783377	-		Methanobacterium_SWAN_1_uid67359	NC_015574	chromosome									
333987895	hypothetical protein MSWAN_1690	No	0		Virus_Caudovi	1783726	1784040	+		Methanobacterium_SWAN_1_uid67359	NC_015574	chromosome									
333987896	integrase family protein	No	0		Virus_Caudovi	1784037	1785215	-		Methanobacterium_SWAN_1_uid67359	NC_015574	chromosome		arCOG01245	15 (-)	L	XerC	Integrase	0.0000001	COG4974	Site-specific recombinase XerD
===									8	Methanobacterium_SWAN_1_uid67359	NC_015574	chromosome									
333988014	hypothetical protein MSWAN_1809	No	0			1898252	1898506	-		Methanobacterium_SWAN_1_uid67359	NC_015574	chromosome									
333988015	hypothetical protein MSWAN_1810	No	0			1898503	1898601	-		Methanobacterium_SWAN_1_uid67359	NC_015574	chromosome									
333988016	hypothetical protein MSWAN_1811	Yes	0			1898685	1899080	-		Methanobacterium_SWAN_1_uid67359	NC_015574	chromosome		arCOG10346	1 (-)	S		Uncharacterized conserved protein			
333988017	hypothetical protein MSWAN_1812	No	0			1899113	1899292	-		Methanobacterium_SWAN_1_uid67359	NC_015574	chromosome							0.00008	pfam13248	zinc-ribbon domain. This family consists
333988018	hypothetical protein MSWAN_1813	No	4			1899677	1900396	-		Methanobacterium_SWAN_1_uid67359	NC_015574	chromosome		arCOG10449	1	S		Uncharacterized conserved protein			
333988019	hypothetical protein MSWAN_1814	No	0			1901017	1901322	+		Methanobacterium_SWAN_1_uid67359	NC_015574	chromosome									
333988020	UspA domain-containing protein	No	0			1901517	1901963	+		Methanobacterium_SWAN_1_uid67359	NC_015574	chromosome		arCOG02053	7 (-)	T	UspA	Nucleotide-binding protein, UspA family	4E-23	pfam00582	Universal stress protein family. The uni
333988021	hypothetical protein MSWAN_1816	No	4			1902118	1902540	+		Methanobacterium_SWAN_1_uid67359	NC_015574	chromosome		arCOG11271	3	S		Uncharacterized conserved protein			
===									7	Methanobrevibacter_ruminantium_M1_uid45857	NC_013790	chromosome									
288559278	adhesin-like protein with cysteine protease do	Yes	1			27726	33740	+		Methanobrevibacter_ruminantium_M1_uid45857	NC_013790	chromosome		arCOG02488	4 (-)	S		Cell suface protein	9E-34	COG4870	Cysteine protease
288559279	hypothetical protein mru_0021	No	0	YES		34167	34724	+		Methanobrevibacter_ruminantium_M1_uid45857	NC_013790	chromosome		arCOG10241	1	S		Uncharacterized conserved protein			
288559280	hypothetical protein mru_0022	No	0	YES		34825	35079	+		Methanobrevibacter_ruminantium_M1_uid45857	NC_013790	chromosome									
288559281	hypothetical protein mru_0023	Yes	1	YES		35076	35543	+		Methanobrevibacter_ruminantium_M1_uid45857	NC_013790	chromosome									
288559282	hypothetical protein mru_0024	No	0	YES		35713	36471	+		Methanobrevibacter_ruminantium_M1_uid45857	NC_013790	chromosome									
288559283	hypothetical protein mru_0025	No	0	YES		36461	37042	+		Methanobrevibacter_ruminantium_M1_uid45857	NC_013790	chromosome									
288559284	DNA-cytosine methyltransferase	No	0	YES		37238	38257	-		Methanobrevibacter_ruminantium_M1_uid45857	NC_013790	chromosome		arCOG04157	7 (-)	L	Dcm	Site-specific DNA methylase	9E-86	pfam00145	C-5 cytosine-specific DNA methylase.
===									7	Methanobrevibacter_ruminantium_M1_uid45857	NC_013790	chromosome									
288559298	ATPase	No	0			57287	58522	-		Methanobrevibacter_ruminantium_M1_uid45857	NC_013790	chromosome		arCOG03167	6 (-)	R		Predicted ATPase (AAA+ superfamily)	3E-46	COG1373	Predicted ATPase (AAA+ superfamily)
288559299	hypothetical protein mru_0041	No	0			59627	62761	+		Methanobrevibacter_ruminantium_M1_uid45857	NC_013790	chromosome									
288559300	hypothetical protein mru_0042	No	0			62911	63204	-		Methanobrevibacter_ruminantium_M1_uid45857	NC_013790	chromosome		arCOG11168	2	S		Uncharacterized conserved protein			
288559301	hypothetical protein mru_0043	No	0			63600	64100	+		Methanobrevibacter_ruminantium_M1_uid45857	NC_013790	chromosome							0.0000004	COG1196	Chromosome segregation ATPases
288559302	serine phosphatase	No	6			64346	65155	-		Methanobrevibacter_ruminantium_M1_uid45857	NC_013790	chromosome							4E-24	COG3275	Putative regulator of cell autolysis
288559303	hypothetical protein mru_0045	No	0			65659	65964	-		Methanobrevibacter_ruminantium_M1_uid45857	NC_013790	chromosome									
288559304	MFS transporter	No	14			66494	68038	-		Methanobrevibacter_ruminantium_M1_uid45857	NC_013790	chromosome		arCOG00142	1 (-)	G	ProP	Permease of the major facilitator superfamily	7E-22	pfam07690	Major Facilitator Superfamily.
===									11	Methanobrevibacter_ruminantium_M1_uid45857	NC_013790	chromosome									
288559308	hypothetical protein mru_0050	No	0			76353	76637	-		Methanobrevibacter_ruminantium_M1_uid45857	NC_013790	chromosome									
288559309	hypothetical protein mru_0051	No	0			76641	76898	-		Methanobrevibacter_ruminantium_M1_uid45857	NC_013790	chromosome									
288559310	glycyl-radical enzyme activating protein	No	0			78234	78920	-		Methanobrevibacter_ruminantium_M1_uid45857	NC_013790	chromosome		arCOG00952	1 (-)	O	PflA	Pyruvate-formate lyase-activating enzyme	3E-12	COG1180	Pyruvate-formate lyase-activating enzyme
288559311	hypothetical protein mru_0053	No	0	YES		78913	79392	-		Methanobrevibacter_ruminantium_M1_uid45857	NC_013790	chromosome									
288559312	hypothetical protein mru_0054	No	0	YES		79764	80315	-		Methanobrevibacter_ruminantium_M1_uid45857	NC_013790	chromosome									
288559313	hypothetical protein mru_0055	No	0	YES		80384	80560	-		Methanobrevibacter_ruminantium_M1_uid45857	NC_013790	chromosome									
288559314	hypothetical protein mru_0056	No	0	YES		80782	81000	-		Methanobrevibacter_ruminantium_M1_uid45857	NC_013790	chromosome									
288559315	hypothetical protein mru_0057	No	0	YES		81016	83073	-		Methanobrevibacter_ruminantium_M1_uid45857	NC_013790	chromosome		arCOG09636	4	Y		Phage protein containing C-terminal domain related to phage head morphogenesis Mu gpF-like			
288559316	hypothetical protein mru_0058	No	0	YES		83097	83594	-		Methanobrevibacter_ruminantium_M1_uid45857	NC_013790	chromosome									
288559317	hypothetical protein mru_0059	No	0	YES		84290	85312	-		Methanobrevibacter_ruminantium_M1_uid45857	NC_013790	chromosome		arCOG06861	1 (-)	S		Uncharacterized conserved protein			
288559318	hypothetical protein mru_0060	No	0	YES		85314	85883	-		Methanobrevibacter_ruminantium_M1_uid45857	NC_013790	chromosome									
===									66	Methanobrevibacter_ruminantium_M1_uid45857	NC_013790	chromosome									
288559515	hypothetical protein mru_0257	No	0	YES	Virus_Caudovi	406364	406660	+		Methanobrevibacter_ruminantium_M1_uid45857	NC_013790	chromosome									
288559516	hypothetical protein mru_0258	No	0	YES	Virus_Caudovi	406681	407565	-		Methanobrevibacter_ruminantium_M1_uid45857	NC_013790	chromosome									
288559517	cdc6 family replication initiation protein Cdc6-3	No	0	YES	Virus_Caudovi	407788	408615	+		Methanobrevibacter_ruminantium_M1_uid45857	NC_013790	chromosome		arCOG00467	13 (-)	L	CDC6	Cdc6-related protein, AAA superfamily ATPase	9E-34	COG1474	Cdc6-related protein, AAA superfamily AT
288559518	hypothetical protein mru_0260	No	0	YES	Virus_Caudovi	408644	409501	-		Methanobrevibacter_ruminantium_M1_uid45857	NC_013790	chromosome									
288559519	hypothetical protein mru_0261	No	2	YES	Virus_Caudovi	409761	409979	+		Methanobrevibacter_ruminantium_M1_uid45857	NC_013790	chromosome									
288559520	hypothetical protein mru_0262	No	0	YES	Virus_Caudovi	410142	410309	+		Methanobrevibacter_ruminantium_M1_uid45857	NC_013790	chromosome									
288559521	hypothetical protein mru_0263	No	0	YES	Virus_Caudovi	410309	410464	+		Methanobrevibacter_ruminantium_M1_uid45857	NC_013790	chromosome									
288559522	hypothetical protein mru_0264	No	0	YES	Virus_Caudovi	410474	410698	+		Methanobrevibacter_ruminantium_M1_uid45857	NC_013790	chromosome									
288559523	hypothetical protein mru_0265	No	1	YES	Virus_Caudovi	410695	410856	+		Methanobrevibacter_ruminantium_M1_uid45857	NC_013790	chromosome									
288559524	hypothetical protein mru_0266	No	0	YES	Virus_Caudovi	410857	411309	+		Methanobrevibacter_ruminantium_M1_uid45857	NC_013790	chromosome									
288559525	hypothetical protein mru_0267	No	0	YES	Virus_Caudovi	411306	412061	+		Methanobrevibacter_ruminantium_M1_uid45857	NC_013790	chromosome									
288559526	hypothetical protein mru_0268	No	0	YES	Virus_Caudovi	412054	412284	+		Methanobrevibacter_ruminantium_M1_uid45857	NC_013790	chromosome									
288559527	ATPase involved in DNA replication control MC	No	0	YES	Virus_Caudovi	412343	413347	+		Methanobrevibacter_ruminantium_M1_uid45857	NC_013790	chromosome		arCOG00439	14 (-)	L	MCM2	Predicted ATPase involved in replication control,	1E-23	COG1241	Predicted ATPase involved in replication
288559528	hypothetical protein mru_0270	No	0	YES	Virus_Caudovi	413337	413771	+		Methanobrevibacter_ruminantium_M1_uid45857	NC_013790	chromosome									
288559529	hypothetical protein mru_0271	No	0	YES	Virus_Caudovi	414001	414420	+		Methanobrevibacter_ruminantium_M1_uid45857	NC_013790	chromosome									
288559530	hypothetical protein mru_0272	No	0	YES	Virus_Caudovi	414417	414692	+		Methanobrevibacter_ruminantium_M1_uid45857	NC_013790	chromosome									
288559531	hypothetical protein mru_0273	No	0	YES	Virus_Caudovi	414689	414922	+		Methanobrevibacter_ruminantium_M1_uid45857	NC_013790	chromosome									
288559532	hypothetical protein mru_0274	No	0	YES	Virus_Caudovi	414912	415061	+		Methanobrevibacter_ruminantium_M1_uid45857	NC_013790	chromosome									
288559533	hypothetical protein mru_0275	No	0	YES	Virus_Caudovi	415160	415333	+		Methanobrevibacter_ruminantium_M1_uid45857	NC_013790	chromosome									
288559534	hypothetical protein mru_0276	No	0	YES	Virus_Caudovi	415532	415867	+		Methanobrevibacter_ruminantium_M1_uid45857	NC_013790	chromosome		arCOG10442	2	S		Uncharacterized conserved protein			
288559535	hypothetical protein mru_0277	No	0	YES	Virus_Caudovi	415868	416419	-		Methanobrevibacter_ruminantium_M1_uid45857	NC_013790	chromosome		arCOG01917	12 (-)	S		Zn-ribbon domain containing protein	0.0001	pfam13248	zinc-ribbon domain. This family consists
288559536	hypothetical protein mru_0278	No	2	YES	Virus_Caudovi	416905	417141	+		Methanobrevibacter_ruminantium_M1_uid45857	NC_013790	chromosome									
288559537	hypothetical protein mru_0279	No	2	YES	Virus_Caudovi	417125	417463	+		Methanobrevibacter_ruminantium_M1_uid45857	NC_013790	chromosome									
288559538	ParB-like nuclease domain-containing protein	No	0	YES	Virus_Caudovi	417460	418080	+		Methanobrevibacter_ruminantium_M1_uid45857	NC_013790	chromosome									
288559539	hypothetical protein mru_0281	No	0	YES	Virus_Caudovi	418083	418283	+		Methanobrevibacter_ruminantium_M1_uid45857	NC_013790	chromosome									
288559540	hypothetical protein mru_0282	No	0	YES	Virus_Caudovi	418369	418827	+		Methanobrevibacter_ruminantium_M1_uid45857	NC_013790	chromosome									
288559541	hypothetical protein mru_0283	No	5		Virus_Caudovi	418820	419476	+		Methanobrevibacter_ruminantium_M1_uid45857	NC_013790	chromosome		arCOG04284	1 (-)	R	yhhQ	Uncharacterized member of the PurR regulon	9E-34	pfam02592	Uncharacterized ACR, YhhQ family COG1738
288559542	hypothetical protein mru_0284	No	0		Virus_Caudovi	419480	420070	+		Methanobrevibacter_ruminantium_M1_uid45857	NC_013790	chromosome		arCOG11382	5	Y		Phage terminase small subunit			
288559543	terminase large subunit	No	0		Virus_Caudovi	420081	421556	+		Methanobrevibacter_ruminantium_M1_uid45857	NC_013790	chromosome		arCOG09550	17 (-)	Y	TerL	Phage terminase large subunit	1E-15	COG5410	Uncharacterized protein conserved in bac
288559544	hypothetical protein mru_0286	No	0		Virus_Caudovi	421559	422884	-		Methanobrevibacter_ruminantium_M1_uid45857	NC_013790	chromosome							2E-16	pfam02463	RecF/RecN/SMC N terminal domain. This do
288559545	phage portal protein	No	0		Virus_Caudovi	422996	424654	+		Methanobrevibacter_ruminantium_M1_uid45857	NC_013790	chromosome		arCOG11199	3	S		Uncharacterized conserved protein	9E-15	COG5518	Bacteriophage capsid portal protein
288559546	hypothetical protein mru_0288	No	0		Virus_Caudovi	424560	426887	+		Methanobrevibacter_ruminantium_M1_uid45857	NC_013790	chromosome									
288559547	hypothetical protein mru_0289	No	0		Virus_Caudovi	426955	427218	+		Methanobrevibacter_ruminantium_M1_uid45857	NC_013790	chromosome									
288559548	hypothetical protein mru_0290	No	0		Virus_Caudovi	427920	428459	+		Methanobrevibacter_ruminantium_M1_uid45857	NC_013790	chromosome									
288559549	hypothetical protein mru_0291	No	0		Virus_Caudovi	428491	428781	+		Methanobrevibacter_ruminantium_M1_uid45857	NC_013790	chromosome									
288559550	hypothetical protein mru_0292	No	0		Virus_Caudovi	428769	429098	+		Methanobrevibacter_ruminantium_M1_uid45857	NC_013790	chromosome									
288559551	hypothetical protein mru_0293	No	1		Virus_Caudovi	429095	429661	+		Methanobrevibacter_ruminantium_M1_uid45857	NC_013790	chromosome									
288559552	hypothetical protein mru_0294	No	0		Virus_Caudovi	429689	429880	+		Methanobrevibacter_ruminantium_M1_uid45857	NC_013790	chromosome									
288559553	hypothetical protein mru_0295	No	0		Virus_Caudovi	429881	430162	+		Methanobrevibacter_ruminantium_M1_uid45857	NC_013790	chromosome									
288559554	hypothetical protein mru_0296	No	0		Virus_Caudovi	430175	430546	+		Methanobrevibacter_ruminantium_M1_uid45857	NC_013790	chromosome									
288559555	hypothetical protein mru_0297	No	0		Virus_Caudovi	430558	430755	+		Methanobrevibacter_ruminantium_M1_uid45857	NC_013790	chromosome									
288559556	hypothetical protein mru_0298	No	0		Virus_Caudovi	430756	431106	+		Methanobrevibacter_ruminantium_M1_uid45857	NC_013790	chromosome							0.0000008	pfam11171	Protein of unknown function (DUF2958). S
288559557	hypothetical protein mru_0299	No	0		Virus_Caudovi	431123	431347	+		Methanobrevibacter_ruminantium_M1_uid45857	NC_013790	chromosome									
288559558	hypothetical protein mru_0300	No	0		Virus_Caudovi	431358	431510	+		Methanobrevibacter_ruminantium_M1_uid45857	NC_013790	chromosome									
288559559	hypothetical protein mru_0301	No	0		Virus_Caudovi	431507	431695	+		Methanobrevibacter_ruminantium_M1_uid45857	NC_013790	chromosome									
288559560	hypothetical protein mru_0302	No	0		Virus_Caudovi	431739	431906	+		Methanobrevibacter_ruminantium_M1_uid45857	NC_013790	chromosome									
288559561	hypothetical protein mru_0303	No	0		Virus_Caudovi	431961	432167	+		Methanobrevibacter_ruminantium_M1_uid45857	NC_013790	chromosome									
288559562	hypothetical protein mru_0304	No	0		Virus_Caudovi	432177	432425	+		Methanobrevibacter_ruminantium_M1_uid45857	NC_013790	chromosome									
288559563	hypothetical protein mru_0305	No	0		Virus_Caudovi	433391	434743	-		Methanobrevibacter_ruminantium_M1_uid45857	NC_013790	chromosome		arCOG04006	3 (-)	S		HEAT repeats containing protein			
288559564	hypothetical protein mru_0306	No	1		Virus_Caudovi	434960	435655	-		Methanobrevibacter_ruminantium_M1_uid45857	NC_013790	chromosome		arCOG04208	2 (-)	J	RPL43A	Ribosomal protein L37AE/L43A	2E-18	pfam13868	Tumour suppressor, Mitostatin. Trichople
288559565	hypothetical protein mru_0307	No	0		Virus_Caudovi	435879	436922	+		Methanobrevibacter_ruminantium_M1_uid45857	NC_013790	chromosome							0.00004	COG5271	AAA ATPase containing von Willebrand fac
288559566	hypothetical protein mru_0308	No	0		Virus_Caudovi	436963	438000	+		Methanobrevibacter_ruminantium_M1_uid45857	NC_013790	chromosome									
288559567	hypothetical protein mru_0309	Yes	1		Virus_Caudovi	438005	438409	+		Methanobrevibacter_ruminantium_M1_uid45857	NC_013790	chromosome									
288559568	hypothetical protein mru_0310	No	0		Virus_Caudovi	438426	438839	+		Methanobrevibacter_ruminantium_M1_uid45857	NC_013790	chromosome		arCOG11165	2	S		Uncharacterized conserved protein			
288559569	hypothetical protein mru_0311	No	0		Virus_Caudovi	438836	439279	+		Methanobrevibacter_ruminantium_M1_uid45857	NC_013790	chromosome		arCOG11166	1	S		Uncharacterized conserved protein			
288559570	hypothetical protein mru_0312	No	0		Virus_Caudovi	439276	439785	+		Methanobrevibacter_ruminantium_M1_uid45857	NC_013790	chromosome									
288559571	hypothetical protein mru_0313	No	0		Virus_Caudovi	439797	441077	+		Methanobrevibacter_ruminantium_M1_uid45857	NC_013790	chromosome									
288559572	hypothetical protein mru_0314	No	0		Virus_Caudovi	441083	441523	+		Methanobrevibacter_ruminantium_M1_uid45857	NC_013790	chromosome									
288559573	phage tail tape measure protein	No	4		Virus_Caudovi	441704	445003	+		Methanobrevibacter_ruminantium_M1_uid45857	NC_013790	chromosome							0.00001	COG5412	Phage-related protein
288559574	hypothetical protein mru_0316	No	0		Virus_Caudovi	445005	448043	+		Methanobrevibacter_ruminantium_M1_uid45857	NC_013790	chromosome									
288559575	hypothetical protein mru_0317	No	0		Virus_Caudovi	448046	451354	+		Methanobrevibacter_ruminantium_M1_uid45857	NC_013790	chromosome		arCOG02488	4 (-)	S		Cell suface protein			
288559576	hypothetical protein mru_0318	No	0		Virus_Caudovi	451366	452883	+		Methanobrevibacter_ruminantium_M1_uid45857	NC_013790	chromosome									
288559577	hypothetical protein mru_0319	No	0		Virus_Caudovi	452886	453044	+		Methanobrevibacter_ruminantium_M1_uid45857	NC_013790	chromosome									
288559578	endoisopeptidase PeiR	No	0		Virus_Caudovi	453037	453723	+		Methanobrevibacter_ruminantium_M1_uid45857	NC_013790	chromosome		arCOG03944	1 (-)	S		Uncharacterized conserved protein			
288559579	dnd system-associated protein 3	No	0	YES	Virus_Caudovi	454361	456610	+		Methanobrevibacter_ruminantium_M1_uid45857	NC_013790	chromosome									
288559580	dnd system-associated protein 1	No	0	YES	Virus_Caudovi	456613	457983	+		Methanobrevibacter_ruminantium_M1_uid45857	NC_013790	chromosome							0.0000003	pfam05316	Mitochondrial ribosomal protein (VAR1).
===									6	Methanobrevibacter_ruminantium_M1_uid45857	NC_013790	chromosome									
288559894	SAM-dependent methyltransferase	No	0			838933	839550	+		Methanobrevibacter_ruminantium_M1_uid45857	NC_013790	chromosome		arCOG06507	1 (-)	Q		SAM-dependent methyltransferase	0.000000005	pfam08241	Methyltransferase domain. Members of thi
288559895	hypothetical protein mru_0638	No	0			839647	840144	+		Methanobrevibacter_ruminantium_M1_uid45857	NC_013790	chromosome									
288559896	hypothetical protein mru_0639	No	1			840240	840596	-		Methanobrevibacter_ruminantium_M1_uid45857	NC_013790	chromosome									
288559897	hypothetical protein mru_0640	Yes	1			840939	841424	+		Methanobrevibacter_ruminantium_M1_uid45857	NC_013790	chromosome									
288559898	hypothetical protein mru_0641	No	1			841615	841737	+		Methanobrevibacter_ruminantium_M1_uid45857	NC_013790	chromosome									
288559899	hypothetical protein mru_0642	No	0			841795	842133	+		Methanobrevibacter_ruminantium_M1_uid45857	NC_013790	chromosome		arCOG03572	1 (-)	S		Uncharacterized conserved protein	0.000006	pfam12675	Protein of unknown function (DUF3795). T
===									24	Methanobrevibacter_ruminantium_M1_uid45857	NC_013790	chromosome									
288560004	hypothetical protein mru_0747	No	6			972948	973445	-		Methanobrevibacter_ruminantium_M1_uid45857	NC_013790	chromosome							9E-10	COG3247	Uncharacterized conserved protein
288560005	TPR repeat-containing protein	No	0			973817	974611	-		Methanobrevibacter_ruminantium_M1_uid45857	NC_013790	chromosome									
288560006	ADP-ribosylglycohydrolase family protein	No	0			974668	975681	-		Methanobrevibacter_ruminantium_M1_uid45857	NC_013790	chromosome		arCOG04448	2 (-)	O	DraG	ADP-ribosylglycohydrolase	4E-36	pfam03747	ADP-ribosylglycohydrolase. This family i
288560007	hypothetical protein mru_0750	No	0	YES		975685	976092	-		Methanobrevibacter_ruminantium_M1_uid45857	NC_013790	chromosome									
288560008	hypothetical protein mru_0751	No	0	YES		976097	977062	-		Methanobrevibacter_ruminantium_M1_uid45857	NC_013790	chromosome							0.00003	COG2849	Uncharacterized protein conserved in bac
288560009	hypothetical protein mru_0752	No	0	YES		977127	977693	-		Methanobrevibacter_ruminantium_M1_uid45857	NC_013790	chromosome									
288560010	hypothetical protein mru_0753	No	0	YES		978706	980196	+		Methanobrevibacter_ruminantium_M1_uid45857	NC_013790	chromosome							1E-30	pfam11074	Domain of unknown function(DUF2779). Thi
288560011	hypothetical protein mru_0754	No	0	YES		980458	980805	+		Methanobrevibacter_ruminantium_M1_uid45857	NC_013790	chromosome									
288560012	hypothetical protein mru_0755	No	6	YES		981147	981902	+		Methanobrevibacter_ruminantium_M1_uid45857	NC_013790	chromosome		arCOG02879	3 (-)	S		Uncharacterized conserved protein	0.00002	pfam13197	Protein of unknown function (DUF4013). T
288560013	hypothetical protein mru_0756	No	0	YES		982348	982641	-		Methanobrevibacter_ruminantium_M1_uid45857	NC_013790	chromosome	clust_356								
288560014	hypothetical protein mru_0757	No	0	YES		983247	984068	+		Methanobrevibacter_ruminantium_M1_uid45857	NC_013790	chromosome									
288560015	hypothetical protein mru_0758	No	0	YES		984547	984840	-		Methanobrevibacter_ruminantium_M1_uid45857	NC_013790	chromosome	clust_356								
288560016	hypothetical protein mru_0759	No	1	YES		985057	985842	+		Methanobrevibacter_ruminantium_M1_uid45857	NC_013790	chromosome									
288560017	hypothetical protein mru_0760	No	2	YES		986267	986497	-		Methanobrevibacter_ruminantium_M1_uid45857	NC_013790	chromosome									
288560018	hypothetical protein mru_0761	No	0	YES		986779	987639	-		Methanobrevibacter_ruminantium_M1_uid45857	NC_013790	chromosome									
288560019	hypothetical protein mru_0762	No	0	YES		987953	988096	+		Methanobrevibacter_ruminantium_M1_uid45857	NC_013790	chromosome									
288560020	hypothetical protein mru_0763	No	0	YES		988132	988452	+		Methanobrevibacter_ruminantium_M1_uid45857	NC_013790	chromosome									
288560021	hypothetical protein mru_0764	No	0			988880	990211	+		Methanobrevibacter_ruminantium_M1_uid45857	NC_013790	chromosome		arCOG01917	12 (-)	S		Zn-ribbon domain containing protein			
288560022	hypothetical protein mru_0765	No	0			991435	991752	+		Methanobrevibacter_ruminantium_M1_uid45857	NC_013790	chromosome									
288560023	hypothetical protein mru_0766	No	0			991819	992142	+		Methanobrevibacter_ruminantium_M1_uid45857	NC_013790	chromosome									
288560024	hypothetical protein mru_0767	No	0			992295	992603	+		Methanobrevibacter_ruminantium_M1_uid45857	NC_013790	chromosome									
288560025	hypothetical protein mru_0768	Yes	1			992699	992947	+		Methanobrevibacter_ruminantium_M1_uid45857	NC_013790	chromosome							0.0000004	pfam10446	Protein of unknown function (DUF2457). T
288560026	hypothetical protein mru_0769	No	1			993272	993910	-		Methanobrevibacter_ruminantium_M1_uid45857	NC_013790	chromosome									
288560027	exonuclease	No	0			994054	994839	-		Methanobrevibacter_ruminantium_M1_uid45857	NC_013790	chromosome		arCOG05109	2 (-)	L	DnaQ	DNA polymerase III, epsilon subunit or related 3'-	4E-13	COG2176	DNA polymerase III, alpha subunit (gram-
===									8	Methanobrevibacter_ruminantium_M1_uid45857	NC_013790	chromosome									
288560037	hypothetical protein mru_0780	No	0			1012560	1013189	-		Methanobrevibacter_ruminantium_M1_uid45857	NC_013790	chromosome		arCOG06883	1 (-)	S		Zn-ribbon domain containing protein	0.00000007	pfam12773	Double zinc ribbon. This family consists
288560038	hypothetical protein mru_0781	No	0			1013616	1014155	-		Methanobrevibacter_ruminantium_M1_uid45857	NC_013790	chromosome									
288560039	hypothetical protein mru_0782	No	0			1014152	1014988	-		Methanobrevibacter_ruminantium_M1_uid45857	NC_013790	chromosome									
288560040	hypothetical protein mru_0783	No	0			1015056	1015616	-		Methanobrevibacter_ruminantium_M1_uid45857	NC_013790	chromosome									
288560041	hypothetical protein mru_0784	No	0			1015688	1015807	-		Methanobrevibacter_ruminantium_M1_uid45857	NC_013790	chromosome									
288560042	hypothetical protein mru_0785	No	0			1016012	1017511	-		Methanobrevibacter_ruminantium_M1_uid45857	NC_013790	chromosome		arCOG03271	1 (-)	S		Uncharacterized conserved protein, AIG2 family	3E-21	COG2105	Uncharacterized conserved protein
288560043	hypothetical protein mru_0786	No	0			1017611	1018453	+		Methanobrevibacter_ruminantium_M1_uid45857	NC_013790	chromosome									
288560044	hypothetical protein mru_0787	Yes	1			1018824	1019237	-		Methanobrevibacter_ruminantium_M1_uid45857	NC_013790	chromosome									
===									6	Methanobrevibacter_ruminantium_M1_uid45857	NC_013790	chromosome									
288560198	diaminopimelate aminotransferase DapL	No	0			1201403	1202635	+		Methanobrevibacter_ruminantium_M1_uid45857	NC_013790	chromosome		arCOG01133	2 (-)	E		Diaminopimelate aminotransferase or related asp	1E-39	COG0436	Aspartate/tyrosine/aromatic aminotransfe
288560199	hypothetical protein mru_0942	No	0			1202813	1203493	+		Methanobrevibacter_ruminantium_M1_uid45857	NC_013790	chromosome	clust_144								
288560200	hypothetical protein mru_0943	No	0			1203597	1204223	+		Methanobrevibacter_ruminantium_M1_uid45857	NC_013790	chromosome	clust_163								
288560201	hypothetical protein mru_0944	No	0			1204349	1205017	+		Methanobrevibacter_ruminantium_M1_uid45857	NC_013790	chromosome									
288560202	hypothetical protein mru_0945	No	0			1205111	1205653	+		Methanobrevibacter_ruminantium_M1_uid45857	NC_013790	chromosome									
288560203	adenylosuccinate synthetase PurA	No	0			1206140	1207159	+		Methanobrevibacter_ruminantium_M1_uid45857	NC_013790	chromosome		arCOG04387	1 (-)	F	PurA	Adenylosuccinate synthase	3E-84	COG0104	Adenylosuccinate synthase
===									6	Methanobrevibacter_ruminantium_M1_uid45857	NC_013790	chromosome									
288560228	hypothetical protein mru_0971	No	0			1260519	1260812	-		Methanobrevibacter_ruminantium_M1_uid45857	NC_013790	chromosome							0.0000004	COG1196	Chromosome segregation ATPases
288560229	hypothetical protein mru_0972	No	0			1261107	1262213	-		Methanobrevibacter_ruminantium_M1_uid45857	NC_013790	chromosome		arCOG10868	7	L		PD-(D/E)XK nuclease family transposase	0.000004	pfam05483	Synaptonemal complex protein 1 (SCP-1).
288560230	hypothetical protein mru_0973	No	0			1262856	1263347	-		Methanobrevibacter_ruminantium_M1_uid45857	NC_013790	chromosome									
288560231	DNA primase small subunit PriA	No	0			1263642	1264634	+		Methanobrevibacter_ruminantium_M1_uid45857	NC_013790	chromosome		arCOG04110	4 (-)	L	PRI1	Eukaryotic-type DNA primase, catalytic (small) su	1E-37	COG1467	Eukaryotic-type DNA primase, catalytic (
288560232	hypothetical protein mru_0975	No	1			1264968	1265366	+		Methanobrevibacter_ruminantium_M1_uid45857	NC_013790	chromosome									
288560233	adhesin-like protein	Yes	1			1265784	1266086	+		Methanobrevibacter_ruminantium_M1_uid45857	NC_013790	chromosome									
===									16	Methanobrevibacter_ruminantium_M1_uid45857	NC_013790	chromosome									
288560405	hypothetical protein mru_1149	No	0	YES		1518935	1519582	-		Methanobrevibacter_ruminantium_M1_uid45857	NC_013790	chromosome									
288560406	hypothetical protein mru_1150	No	0	YES		1519658	1521892	-		Methanobrevibacter_ruminantium_M1_uid45857	NC_013790	chromosome		arCOG01917	12 (-)	S		Zn-ribbon domain containing protein	0.000005	COG4547	Cobalamin biosynthesis protein CobT (nic ; SecA Wing and Scaffold domain. SecA prot ; zinc-ribbon domain. This family consists
288560407	hypothetical protein mru_1151	No	0	YES		1522059	1522805	-		Methanobrevibacter_ruminantium_M1_uid45857	NC_013790	chromosome		arCOG10910	4	S		Uncharacterized conserved protein			
288560408	hypothetical protein mru_1152	No	0	YES		1522940	1523734	-		Methanobrevibacter_ruminantium_M1_uid45857	NC_013790	chromosome		arCOG10910	4	S		Uncharacterized conserved protein			
288560409	hypothetical protein mru_1153	No	0	YES		1523777	1524223	-		Methanobrevibacter_ruminantium_M1_uid45857	NC_013790	chromosome		arCOG10910	4	S		Uncharacterized conserved protein			
288560410	hypothetical protein mru_1154	No	0	YES		1524240	1524599	-		Methanobrevibacter_ruminantium_M1_uid45857	NC_013790	chromosome		arCOG06576	5 (-)	V		HEPN domain containing protein			
288560411	hypothetical protein mru_1155	No	0	YES		1524874	1525305	-		Methanobrevibacter_ruminantium_M1_uid45857	NC_013790	chromosome									
288560412	hypothetical protein mru_1156	No	0	YES		1526070	1526471	-		Methanobrevibacter_ruminantium_M1_uid45857	NC_013790	chromosome									
288560413	helicase RecD/TraA family	No	0	YES		1526566	1528401	-		Methanobrevibacter_ruminantium_M1_uid45857	NC_013790	chromosome							0.0000001	pfam05970	PIF1-like helicase. This family includes
288560414	RecF/RecN/SMC N terminal domain-containing	No	0	YES		1528511	1530211	-		Methanobrevibacter_ruminantium_M1_uid45857	NC_013790	chromosome		arCOG03235	4 (-)	L		Predicted ATP-dependent endonuclease of the C	3E-30	pfam13175	AAA ATPase domain. This family of domain
288560415	hypothetical protein mru_1159	No	0	YES		1530521	1531246	-		Methanobrevibacter_ruminantium_M1_uid45857	NC_013790	chromosome							0.0000008	COG2856	Predicted Zn peptidase
288560416	transcriptional regulator	No	0	YES		1531249	1531596	-		Methanobrevibacter_ruminantium_M1_uid45857	NC_013790	chromosome									
288560417	hypothetical protein mru_1161	No	0	YES		1531867	1532988	+		Methanobrevibacter_ruminantium_M1_uid45857	NC_013790	chromosome									
288560418	transposase	No	0	YES		1533217	1534713	+		Methanobrevibacter_ruminantium_M1_uid45857	NC_013790	chromosome	arCOG03514,a	arCOG03514	1 (-)	L		Transposase	0.000002	pfam13751	Transposase DDE domain. Transposase prot ; Transposase and inactivated derivatives

288560419	hypothetical protein mru_1163	No	0	YES	1534981	1536123	+		Methanobrevibacter_ruminantium_M1_uid45857	NC_013790	chromosome											
288560420	hypothetical protein mru_1164	No	0	YES	1536126	1537496	-		Methanobrevibacter_ruminantium_M1_uid45857	NC_013790	chromosome											
===								8	Methanobrevibacter_ruminantium_M1_uid45857	NC_013790	chromosome											
288560543	hypothetical protein mru_1287	No	0		1704833	1707256	-		Methanobrevibacter_ruminantium_M1_uid45857	NC_013790	chromosome											
288560544	serine/threonine protein phosphatase	No	0		1707574	1708368	-		Methanobrevibacter_ruminantium_M1_uid45857	NC_013790	chromosome			arCOG01143	1	(-)	T	ApaH	Serine/threonine protein phosphatase PP2A family			
288560545	diacylglycerol kinase DagK	No	3		1708592	1708951	+		Methanobrevibacter_ruminantium_M1_uid45857	NC_013790	chromosome									5E-18	COG0818	Diacylglycerol kinase
288560546	hypothetical protein mru_1290	No	0		1709381	1709635	+		Methanobrevibacter_ruminantium_M1_uid45857	NC_013790	chromosome									2E-21	pfam10127	Predicted nucleotidyltransferase. Member
288560547	hypothetical protein mru_1291	No	0		1709682	1710125	+		Methanobrevibacter_ruminantium_M1_uid45857	NC_013790	chromosome											
288560548	hypothetical protein mru_1292	No	0		1710386	1711219	-		Methanobrevibacter_ruminantium_M1_uid45857	NC_013790	chromosome											
288560549	glucosamine-fructose-6-phosphate aminotransf	No	0		1711579	1713318	+		Methanobrevibacter_ruminantium_M1_uid45857	NC_013790	chromosome			arCOG00057	3	(-)	M	GlmS	Glucosamine 6-phosphate synthetase	3E-158	COG0449	Glucosamine 6-phosphate synthetase, cont
288560550	hypothetical protein mru_1294	No	0		1713591	1716695	-		Methanobrevibacter_ruminantium_M1_uid45857	NC_013790	chromosome											
===								6	Methanobrevibacter_ruminantium_M1_uid45857	NC_013790	chromosome											
288560770	hypothetical protein mru_1514	No	0		1978174	1979397	-		Methanobrevibacter_ruminantium_M1_uid45857	NC_013790	chromosome			arCOG00120	1	(-)	R		Predicted SAM methyltrasferase	0.00000002	pfam05430	S-adenosyl-L-methionine-dependent methyl
288560771	hypothetical protein mru_1515	No	0		1979484	1979957	-		Methanobrevibacter_ruminantium_M1_uid45857	NC_013790	chromosome											
288560772	hypothetical protein mru_1516	No	0		1980040	1980417	-		Methanobrevibacter_ruminantium_M1_uid45857	NC_013790	chromosome											
288560773	hypothetical protein mru_1517	No	0		1980402	1980692	-		Methanobrevibacter_ruminantium_M1_uid45857	NC_013790	chromosome											
288560774	hypothetical protein mru_1518	No	0		1980708	1981229	-		Methanobrevibacter_ruminantium_M1_uid45857	NC_013790	chromosome											
288560775	pyruvate-formate lyase Pfl	No	0		1981763	1983796	-		Methanobrevibacter_ruminantium_M1_uid45857	NC_013790	chromosome			arCOG06130	1	(-)	C	PflD	Pyruvate-formate lyase	4E-27	pfam02901	Pyruvate formate lyase.
===								9	Methanobrevibacter_ruminantium_M1_uid45857	NC_013790	chromosome											
288560783	glycosyl transferase	No	0		1990260	1991267	-		Methanobrevibacter_ruminantium_M1_uid45857	NC_013790	chromosome									2E-12	pfam00852	Glycosyltransferase family 10 (fucosyltr
288560784	glycosyl transferase	No	0		1991301	1992101	-		Methanobrevibacter_ruminantium_M1_uid45857	NC_013790	chromosome			arCOG09483	1		S		Uncharacterized conserved protein	3E-73	pfam14393	Domain of unknown function (DUF4422). Th
288560785	UDP-galactopyranose mutase Glf	No	0		1992508	1993605	-		Methanobrevibacter_ruminantium_M1_uid45857	NC_013790	chromosome			arCOG01525	1	(-)	M	Glf	UDP-galactopyranose mutase	3E-142	COG0562	UDP-galactopyranose mutase
288560786	hypothetical protein mru_1530	No	0		1994257	1994439	+		Methanobrevibacter_ruminantium_M1_uid45857	NC_013790	chromosome											
288560787	hypothetical protein mru_1531	No	0		1994436	1994591	+		Methanobrevibacter_ruminantium_M1_uid45857	NC_013790	chromosome											
288560788	hypothetical protein mru_1532	No	0		1994667	1995248	+		Methanobrevibacter_ruminantium_M1_uid45857	NC_013790	chromosome											
288560789	hypothetical protein mru_1533	No	0		1995259	1995942	+		Methanobrevibacter_ruminantium_M1_uid45857	NC_013790	chromosome											
288560790	acyltransferase	No	8		1996048	1996875	+		Methanobrevibacter_ruminantium_M1_uid45857	NC_013790	chromosome			arCOG03634	1	(-)	R		Predicted O-acyltransferase	4E-18	pfam01757	Acyltransferase family. This family incl
288560791	hypothetical protein mru_1535	No	2		1996862	1997119	+		Methanobrevibacter_ruminantium_M1_uid45857	NC_013790	chromosome											
===								10	Methanobrevibacter_ruminantium_M1_uid45857	NC_013790	chromosome											
288560846	hypothetical protein mru_1590	No	0		2070069	2070191	+		Methanobrevibacter_ruminantium_M1_uid45857	NC_013790	chromosome											
288560847	hypothetical protein mru_1591	No	0		2070373	2070891	-		Methanobrevibacter_ruminantium_M1_uid45857	NC_013790	chromosome			arCOG11225	1		S		Uncharacterized conserved protein	0.00004	pfam02463	RecF/RecN/SMC N terminal domain. This do
288560848	hypothetical protein mru_1592	No	0		2071165	2071308	-		Methanobrevibacter_ruminantium_M1_uid45857	NC_013790	chromosome											
288560849	von Willebrand factor type A domain-containing	No	0		2071682	2072314	-		Methanobrevibacter_ruminantium_M1_uid45857	NC_013790	chromosome			arCOG07591	1	(-)	R		Von Willebrand factor type A (vWA) domain			
288560850	hypothetical protein mru_1594	No	0		2072717	2074330	-		Methanobrevibacter_ruminantium_M1_uid45857	NC_013790	chromosome											
288560851	hypothetical protein mru_1595	No	0		2074636	2074941	-		Methanobrevibacter_ruminantium_M1_uid45857	NC_013790	chromosome											
288560852	hypothetical protein mru_1596	No	0		2075111	2075350	-		Methanobrevibacter_ruminantium_M1_uid45857	NC_013790	chromosome											
288560853	hypothetical protein mru_1597	No	10		2075617	2076507	+		Methanobrevibacter_ruminantium_M1_uid45857	NC_013790	chromosome			arCOG00271	3	(-)	G	RhaT	Permease of the drug/metabolite transporter (DM	9E-17	COG0697	Permeases of the drug/metabolite transpo
288560854	hypothetical protein mru_1598	No	1		2076556	2077068	+		Methanobrevibacter_ruminantium_M1_uid45857	NC_013790	chromosome											
288560855	hypothetical protein mru_1599	No	0		2077242	2077457	-		Methanobrevibacter_ruminantium_M1_uid45857	NC_013790	chromosome											
===								7	Methanobrevibacter_ruminantium_M1_uid45857	NC_013790	chromosome											
288560918	transposase	No	1		2162457	2163422	+		Methanobrevibacter_ruminantium_M1_uid45857	NC_013790	chromosome			arCOG04442	3	(-)	L		Transposase			
288560919	hypothetical protein mru_1663	No	0		2163617	2163919	+		Methanobrevibacter_ruminantium_M1_uid45857	NC_013790	chromosome											
288560920	hypothetical protein mru_1664	No	0		2163930	2164169	+		Methanobrevibacter_ruminantium_M1_uid45857	NC_013790	chromosome											
288560921	hypothetical protein mru_1665	No	0		2164205	2164354	+		Methanobrevibacter_ruminantium_M1_uid45857	NC_013790	chromosome											
288560922	hypothetical protein mru_1666	No	0		2164718	2165452	-		Methanobrevibacter_ruminantium_M1_uid45857	NC_013790	chromosome											
288560923	HIRAN domain-containing protein	No	0		2165527	2165931	-		Methanobrevibacter_ruminantium_M1_uid45857	NC_013790	chromosome											
288560924	hypothetical protein mru_1668	No	0		2165951	2166709	-		Methanobrevibacter_ruminantium_M1_uid45857	NC_013790	chromosome			arCOG02846	3	(-)	O	DnaJ	DnaJ-class molecular chaperone	0.00000004	COG0419	ATPase involved in DNA repair
===								8	Methanobrevibacter_ruminantium_M1_uid45857	NC_013790	chromosome											
288561048	carbamoyl-phosphate synthase small subunit (	No	0		2315240	2316319	-		Methanobrevibacter_ruminantium_M1_uid45857	NC_013790	chromosome			arCOG00064	1	(-)	E	CarA	Carbamoylphosphate synthase small subunit	6E-120	COG0505	Carbamoylphosphate synthase small subuni
288561049	hypothetical protein mru_1793	No	0		2316727	2317476	-		Methanobrevibacter_ruminantium_M1_uid45857	NC_013790	chromosome											
288561050	hypothetical protein mru_1794	No	4		2317463	2317852	-		Methanobrevibacter_ruminantium_M1_uid45857	NC_013790	chromosome											
288561051	hypothetical protein mru_1795	No	1		2317931	2318158	-		Methanobrevibacter_ruminantium_M1_uid45857	NC_013790	chromosome											
288561052	hypothetical protein mru_1796	No	0		2318432	2318662	+		Methanobrevibacter_ruminantium_M1_uid45857	NC_013790	chromosome									0.00003	pfam13248	zinc-ribbon domain. This family consists
288561053	hypothetical protein mru_1797	No	0		2318757	2319107	+		Methanobrevibacter_ruminantium_M1_uid45857	NC_013790	chromosome											
288561054	adhesin-like protein	No	1		2319411	2320829	+		Methanobrevibacter_ruminantium_M1_uid45857	NC_013790	chromosome			arCOG03946	3	(-)	S		Uncharacterized conserved protein			
288561055	hypothetical protein mru_1799	No	0		2320894	2321823	+		Methanobrevibacter_ruminantium_M1_uid45857	NC_013790	chromosome									0.000000004	COG1196	Chromosome segregation ATPases
===								5	Methanobrevibacter_ruminantium_M1_uid45857	NC_013790	chromosome											
288561379	hypothetical protein mru_2123	No	7		2772665	2773420	-		Methanobrevibacter_ruminantium_M1_uid45857	NC_013790	chromosome											
288561380	hypothetical protein mru_2124	No	2		2773945	2774343	-		Methanobrevibacter_ruminantium_M1_uid45857	NC_013790	chromosome											
288561381	hypothetical protein mru_2125	No	0		2774762	2775538	-		Methanobrevibacter_ruminantium_M1_uid45857	NC_013790	chromosome											
288561382	cell wall biosynthesis protein UDP-glycosyltran	No	0		2776005	2777093	-		Methanobrevibacter_ruminantium_M1_uid45857	NC_013790	chromosome			arCOG07615	1	(-)	M		UDP-N-acetylglucosamine:LPS N-acetylglucosam	0.000003	COG0707	UDP-N-acetylglucosamine:LPS N-acetylgluc
288561383	ribonuclease	Yes	1		2777433	2777891	+		Methanobrevibacter_ruminantium_M1_uid45857	NC_013790	chromosome									0.000005	pfam00545	ribonuclease. This enzyme hydrolyses RNA
===								11	Methanobrevibacter_smithii_ATCC_35061_uid588	NC_009515	chromosome											
148642070	GNAT family N-acetyltransferase	No	0		7976	8551	+		Methanobrevibacter_smithii_ATCC_35061_uid588	NC_009515	chromosome			arCOG00851	1	(-)	K	WecD	Acetyltransferase (GNAT) family			
148642071	hypothetical protein Msm_0011	No	2		8560	9069	-		Methanobrevibacter_smithii_ATCC_35061_uid588	NC_009515	chromosome											
148642072	hypothetical protein Msm_0012	No	0		9331	10137	-		Methanobrevibacter_smithii_ATCC_35061_uid588	NC_009515	chromosome											
148642073	hypothetical protein Msm_0013	No	0		10652	10936	-		Methanobrevibacter_smithii_ATCC_35061_uid588	NC_009515	chromosome											
148642074	putative heat shock related protein	No	0		11089	11382	-		Methanobrevibacter_smithii_ATCC_35061_uid588	NC_009515	chromosome											
148642075	hypothetical protein Msm_0015	No	0		11425	11967	+		Methanobrevibacter_smithii_ATCC_35061_uid588	NC_009515	chromosome											
148642076	hypothetical protein Msm_0016	No	0		11983	12246	+		Methanobrevibacter_smithii_ATCC_35061_uid588	NC_009515	chromosome	clust_381										
148642077	hypothetical protein Msm_0017	No	0		12300	12551	+		Methanobrevibacter_smithii_ATCC_35061_uid588	NC_009515	chromosome											
148642078	hypothetical protein Msm_0018	No	0		12548	12811	+		Methanobrevibacter_smithii_ATCC_35061_uid588	NC_009515	chromosome	clust_381										
148642079	hypothetical protein Msm_0019	No	0		13129	13251	+		Methanobrevibacter_smithii_ATCC_35061_uid588	NC_009515	chromosome											
148642080	O-linked GlnNAc transferase	No	0		13349	13666	-		Methanobrevibacter_smithii_ATCC_35061_uid588	NC_009515	chromosome			arCOG03032	12	(-)	R	NrfG	TPR repeats containing protein			
===								10	Methanobrevibacter_smithii_ATCC_35061_uid588	NC_009515	chromosome											
148642094	hypothetical protein Msm_0034	No	0		26171	26506	-		Methanobrevibacter_smithii_ATCC_35061_uid588	NC_009515	chromosome			arCOG03293	1	(-)	S	XynA	Predicted membrane protein			
148642095	hypothetical protein Msm_0035	Yes	1		26519	27097	-		Methanobrevibacter_smithii_ATCC_35061_uid588	NC_009515	chromosome											
148642096	hypothetical protein Msm_0036	No	0		27314	28018	-		Methanobrevibacter_smithii_ATCC_35061_uid588	NC_009515	chromosome									0.0001	COG1196	Chromosome segregation ATPases
148642097	hypothetical protein Msm_0037	No	0		28082	28510	+		Methanobrevibacter_smithii_ATCC_35061_uid588	NC_009515	chromosome											
148642098	hypothetical protein Msm_0038	No	0		29048	29536	+		Methanobrevibacter_smithii_ATCC_35061_uid588	NC_009515	chromosome											
148642099	hypothetical protein Msm_0039	No	0		30032	30475	+		Methanobrevibacter_smithii_ATCC_35061_uid588	NC_009515	chromosome											
148642100	hypothetical protein Msm_0040	No	0		30477	30749	+		Methanobrevibacter_smithii_ATCC_35061_uid588	NC_009515	chromosome											
148642101	hypothetical protein Msm_0041	No	0		30744	31019	-		Methanobrevibacter_smithii_ATCC_35061_uid588	NC_009515	chromosome											
148642102	hypothetical protein Msm_0042	No	0		31030	31209	-		Methanobrevibacter_smithii_ATCC_35061_uid588	NC_009515	chromosome											
148642103	peptide methionine sulfoxide reductase, PMSI	No	0		31292	32077	+		Methanobrevibacter_smithii_ATCC_35061_uid588	NC_009515	chromosome			arCOG02815	2	(-)	O		Conserved domain frequently associated with pe	9E-28	pfam01625	Peptide methionine sulfoxide reductase. ; Conserved domain frequently associated w
===								6	Methanobrevibacter_smithii_ATCC_35061_uid588	NC_009515	chromosome											
148642307	ribonuclease P protein component 3	No	0		232317	233021	-		Methanobrevibacter_smithii_ATCC_35061_uid588	NC_009515	chromosome			arCOG00307	1	(-)	J	RPP1	RNase P/RNase MRP subunit p30	4E-26	COG1603	RNase P/RNase MRP subunit p30
148642308	hypothetical protein Msm_0248	No	3		233136	233411	-		Methanobrevibacter_smithii_ATCC_35061_uid588	NC_009515	chromosome											
148642309	hypothetical protein Msm_0249	No	1		233634	234353	-		Methanobrevibacter_smithii_ATCC_35061_uid588	NC_009515	chromosome			arCOG07908	3		S		Zn ribbon containing protein			
148642310	hypothetical protein Msm_0250	No	1		234363	234971	-		Methanobrevibacter_smithii_ATCC_35061_uid588	NC_009515	chromosome			arCOG07907	2		S		Zn-ribbons containing protein			
148642311	hypothetical protein Msm_0251	No	1		235049	235582	-		Methanobrevibacter_smithii_ATCC_35061_uid588	NC_009515	chromosome			arCOG07908	3		S		Zn ribbon containing protein			
148642312	Na+-driven multidrug efflux pump, NorM	No	12		235769	237133	-		Methanobrevibacter_smithii_ATCC_35061_uid588	NC_009515	chromosome			arCOG01731	2	(-)	V	NorM	Na+-driven multidrug efflux pump	2E-59	COG0534	Na+-driven multidrug efflux pump
===								7	Methanobrevibacter_smithii_ATCC_35061_uid588	NC_009515	chromosome											
148642395	glutamyl-tRNA(Gln) amidotransferase subunit E	No	0		316992	318857	+		Methanobrevibacter_smithii_ATCC_35061_uid588	NC_009515	chromosome			arCOG01719	3	(-)	J	GatE	Archaeal Glu-tRNAGln amidotransferase subunit E (contains GAI		COG2511	Archaeal Glu-tRNAGln amidotransferase su
148642396	hypothetical protein Msm_0336	No	0		319022	319225	-		Methanobrevibacter_smithii_ATCC_35061_uid588	NC_009515	chromosome											
148642397	putative adhesin-like protein	No	0		319368	319583	+		Methanobrevibacter_smithii_ATCC_35061_uid588	NC_009515	chromosome											
148642398	hypothetical protein Msm_0338	No	0		319693	319947	+		Methanobrevibacter_smithii_ATCC_35061_uid588	NC_009515	chromosome											
148642399	hypothetical protein Msm_0339	No	1		320063	320248	+		Methanobrevibacter_smithii_ATCC_35061_uid588	NC_009515	chromosome											
148642400	thioredoxin reductase (NADPH), TrxB	No	0		320359	321273	+		Methanobrevibacter_smithii_ATCC_35061_uid588	NC_009515	chromosome			arCOG01296	3	(-)	O	TrxB	Thioredoxin reductase	2E-79	COG0492	Thioredoxin reductase
148642401	hypothetical protein Msm_0341	No	1		321472	321960	+		Methanobrevibacter_smithii_ATCC_35061_uid588	NC_009515	chromosome											
===								8	Methanobrevibacter_smithii_ATCC_35061_uid588	NC_009515	chromosome											
148642532	Xaa-Pro aminopeptidase	No	0		448724	449767	-		Methanobrevibacter_smithii_ATCC_35061_uid588	NC_009515	chromosome			arCOG01000	1	(-)	E	PepP	Xaa-Pro aminopeptidase	5E-57	COG0006	Xaa-Pro aminopeptidase
148642533	hypothetical protein Msm_0473	No	5		450036	450788	+		Methanobrevibacter_smithii_ATCC_35061_uid588	NC_009515	chromosome	clust_132										
148642534	hypothetical protein Msm_0474	No	0		451072	451200	+		Methanobrevibacter_smithii_ATCC_35061_uid588	NC_009515	chromosome											
148642535	hypothetical protein Msm_0475	No	7		451214	451933	+		Methanobrevibacter_smithii_ATCC_35061_uid588	NC_009515	chromosome											
148642536	hypothetical protein Msm_0476	No	2		451940	452176	+		Methanobrevibacter_smithii_ATCC_35061_uid588	NC_009515	chromosome	clust_409										
148642537	hypothetical protein Msm_0477	No	5		452199	452957	+		Methanobrevibacter_smithii_ATCC_35061_uid588	NC_009515	chromosome	clust_132										
148642538	hypothetical protein Msm_0478	No	2		452975	453202	+		Methanobrevibacter_smithii_ATCC_35061_uid588	NC_009515	chromosome	clust_409										
148642539	zinc-dependent protease	No	5		453330	453893	+		Methanobrevibacter_smithii_ATCC_35061_uid588	NC_009515	chromosome			arCOG00614	1	(-)	R	SpoIVFB	Zn-dependent protease	4E-10	COG1994	Zn-dependent proteases
===								8	Methanobrevibacter_smithii_ATCC_35061_uid588	NC_009515	chromosome											
148642592	putative IS element (transposase remnant)	No	0		508688	508987	+		Methanobrevibacter_smithii_ATCC_35061_uid588	NC_009515	chromosome											
148642593	putative IS element (transposase remnant)	No	0		509066	509494	+		Methanobrevibacter_smithii_ATCC_35061_uid588	NC_009515	chromosome			arCOG04931	3	(-)	S		Uncharacterized conserved protein			
148642594	putative IS element (transposase remnant)	No	0		509745	509903	+		Methanobrevibacter_smithii_ATCC_35061_uid588	NC_009515	chromosome											
148642595	hypothetical protein Msm_0535	No	0		510121	510648	-		Methanobrevibacter_smithii_ATCC_35061_uid588	NC_009515	chromosome											
148642596	hypothetical protein Msm_0536	No	0		510707	511780	-		Methanobrevibacter_smithii_ATCC_35061_uid588	NC_009515	chromosome											
148642597	TPR repeat-containing protein	No	0		511889	512251	-		Methanobrevibacter_smithii_ATCC_35061_uid588	NC_009515	chromosome											
148642598	pyruvate formate-lyase activating enzyme, PflA	No	0		512568	512882	+		Methanobrevibacter_smithii_ATCC_35061_uid588	NC_009515	chromosome			arCOG02173	4	(-)	O	NrdG	Organic radical activating enzyme	0.0000006	COG1180	Pyruvate-formate lyase-activating enzyme
148642599	putative DNA-directed DNA polymerase	No	0		512962	513861	+		Methanobrevibacter_smithii_ATCC_35061_uid588	NC_009515	chromosome											
===								5	Methanobrevibacter_smithii_ATCC_35061_uid588	NC_009515	chromosome											
148643514	ATPase	No	1		1477908	1479623	-		Methanobrevibacter_smithii_ATCC_35061_uid588	NC_009515	chromosome									7E-11	pfam02463	RecF/RecN/SMC N terminal domain. This do
148643515	L,L-diaminopimelate aminotransferase	No	0		1479806	1481041	+		Methanobrevibacter_smithii_ATCC_35061_uid588	NC_009515	chromosome			arCOG01133	2	(-)	E		Diaminopimelate aminotransferase or related asp	3E-36	COG0436	Aspartate/tyrosine/aromatic aminotransfe
148643516	hypothetical protein Msm_1456	No	0		1481051	1481746	+		Methanobrevibacter_smithii_ATCC_35061_uid588	NC_009515	chromosome	clust_144										
148643517	hypothetical protein Msm_1457	No	0		1481751	1482335	+		Methanobrevibacter_smithii_ATCC_35061_uid588	NC_009515	chromosome	clust_163										
148643518	hypothetical protein Msm_1458	No	0		1482346	1482930	+		Methanobrevibacter_smithii_ATCC_35061_uid588	NC_009515	chromosome											
===								8	Methanobrevibacter_smithii_ATCC_35061_uid588	NC_009515	chromosome											
148643565	hypothetical protein Msm_1505	No	0		1531630	1531779	-		Methanobrevibacter_smithii_ATCC_35061_uid588	NC_009515	chromosome											
148643566	hypothetical protein Msm_1506	No	0		1531769	1531945	-		Methanobrevibacter_smithii_ATCC_35061_uid588	NC_009515	chromosome											
148643567	glycosyl transferase family protein	No	0		1532141	1533163	+		Methanobrevibacter_smithii_ATCC_35061_uid588	NC_009515	chromosome			arCOG01381	4	(-)	M	WcaA	Glycosyltransferase	3E-19	pfam00535	Glycosyl transferase family 2. Diverse f
148643568	hypothetical protein Msm_1508	No	0		1533294	1533503	+		Methanobrevibacter_smithii_ATCC_35061_uid588	NC_009515	chromosome											
148643569	hypothetical protein Msm_1509	Yes	1		1533493	1533903	+		Methanobrevibacter_smithii_ATCC_35061_uid588	NC_009515	chromosome											
148643570	putative SAM-dependent methyltransferase	No	0		1533920	1534195	-		Methanobrevibacter_smithii_ATCC_35061_uid588	NC_009515	chromosome											
148643571	hypothetical protein Msm_1511	No	0		1534427	1534639	-		Methanobrevibacter_smithii_ATCC_35061_uid588	NC_009515	chromosome											
148643572	lipopolysaccharide cholinephosphotransferase	No	0		1534820	1535860	+		Methanobrevibacter_smithii_ATCC_35061_uid588	NC_009515	chromosome			arCOG09737	1	(-)	M		LPS biosynthesis protein	2E-25	pfam04991	LicD family. The LICD family of proteins
===								54	Methanobrevibacter_smithii_ATCC_35061_uid588	NC_009515	chromosome											
148643700	DNA intergrase/recombinase	No	0	Virus_Caudovi	1693321	1694475	+		Methanobrevibacter_smithii_ATCC_35061_uid588	NC_009515	chromosome			arCOG01245	15	(-)	L	XerC	Integrase			
148643701	hypothetical protein Msm_1641	No	0	Virus_Caudovi	1694545	1694820	+		Methanobrevibacter_smithii_ATCC_35061_uid588	NC_009515	chromosome											
148643702	hypothetical protein Msm_1642	No	0	Virus_Caudovi	1694840	1695199	+		Methanobrevibacter_smithii_ATCC_35061_uid588	NC_009515	chromosome			arCOG11168	2		S		Uncharacterized conserved protein			
148643703	hypothetical protein Msm_1643	No	0	Virus_Caudovi	1695204	1695629	+		Methanobrevibacter_smithii_ATCC_35061_uid588	NC_009515	chromosome											
148643704	hypothetical protein Msm_1644	No	0	Virus_Caudovi	1695671	1696003	-		Methanobrevibacter_smithii_ATCC_35061_uid588	NC_009515	chromosome											
148643705	virulence protein	No	0	Virus_Caudovi	1696729	1697160	+		Methanobrevibacter_smithii_ATCC_35061_uid588	NC_009515	chromosome			arCOG04956	2	(-)	R		Virulence protein	4E-12	COG3943	Virulence protein
148643706	ATPase	No	0	Virus_Caudovi	1697221	1698444	+		Methanobrevibacter_smithii_ATCC_35061_uid588	NC_009515	chromosome			arCOG03169	1	(-)	R		Predicted ATPase (AAA+ superfamily)			
148643707	hypothetical protein Msm_1647	No	0	Virus_Caudovi	1698623	1698796	-		Methanobrevibacter_smithii_ATCC_35061_uid588	NC_009515	chromosome											
148643708	hypothetical protein Msm_1648	No	0	Virus_Caudovi	1698975	1699118	+		Methanobrevibacter_smithii_ATCC_35061_uid588	NC_009515	chromosome											
148643709	hypothetical protein Msm_1649	No	0	Virus_Caudovi	1699115	1699339	+		Methanobrevibacter_smithii_ATCC_35061_uid588	NC_009515	chromosome											
148643710	hypothetical protein Msm_1650	No	0	Virus_Caudovi	1699340	1699615	+		Methanobrevibacter_smithii_ATCC_35061_uid588	NC_009515	chromosome											
148643711	hypothetical protein Msm_1651	No	1	Virus_Caudovi	1699612	1699791	+		Methanobrevibacter_smithii_ATCC_35061_uid588	NC_009515	chromosome											
148643712	hypothetical protein Msm_1652	No	0	Virus_Caudovi	1699788	1700087	+		Methanobrevibacter_smithii_ATCC_35061_uid588	NC_009515	chromosome											
148643713	hypothetical protein Msm_1653	No	0	Virus_Caudovi	1700087	1700404	+		Methanobrevibacter_smithii_ATCC_35061_uid588	NC_009515	chromosome											
148643714	Gp40-related protein	No	0	Virus_Caudovi	1700424	1701035	+		Methanobrevibacter_smithii_ATCC_35061_uid588	NC_009515	chromosome									8E-14	pfam04404	ERF superfamily. The DNA single-strand a
148643715	hypothetical protein Msm_1655	No	0	Virus_Caudovi	1701038	1701232	+		Methanobrevibacter_smithii_ATCC_35061_uid588	NC_009515	chromosome											
148643716	hypothetical protein Msm_1656	No	0	Virus_Caudovi	1701229	1701483	+		Methanobrevibacter_smithii_ATCC_35061_uid588	NC_009515	chromosome											
148643717	hypothetical protein Msm_1657	No	0	Virus_Caudovi	1701480	1701899	+		Methanobrevibacter_smithii_ATCC_35061_uid588	NC_009515	chromosome											
148643718	hypothetical protein Msm_1658	No	0	Virus_Caudovi	1702051	1702296	+		Methanobrevibacter_smithii_ATCC_35061_uid588	NC_009515	chromosome											
148643719	hypothetical protein Msm_1659	No	0	Virus_Caudovi	1702293	1702466	+		Methanobrevibacter_smithii_ATCC_35061_uid588	NC_009515	chromosome											
148643720	hypothetical protein Msm_1660	No	0	Virus_Caudovi	1702463	1702648	+		Methanobrevibacter_smithii_ATCC_35061_uid588	NC_009515	chromosome											
148643721	hypothetical protein Msm_1661	No	0	Virus_Caudovi	1702654	1702995	+		Methanobrevibacter_smithii_ATCC_35061_uid588	NC_009515	chromosome			arCOG00398	2	(-)	J		ASCH domain, predicted RNA-binding domain			
148643722	hypothetical protein Msm_1662	No	0	Virus_Caudovi	1703009	1703155	+		Methanobrevibacter_smithii_ATCC_35061_uid588	NC_009515	chromosome											
148643723	hypothetical protein Msm_1663	No	0	Virus_Caudovi	1703327	1703590	+		Methanobrevibacter_smithii_ATCC_35061_uid588	NC_009515	chromosome											
148643724	hypothetical protein Msm_1664	No	0	Virus_Caudovi	1703577	1703807	+		Methanobrevibacter_smithii_ATCC_35061_uid588	NC_009515	chromosome											
148643725	hypothetical protein Msm_1665	No	0	Virus_Caudovi	1703812	1704126	+		Methanobrevibacter_smithii_ATCC_35061_uid588	NC_009515	chromosome			arCOG10442	2		S		Uncharacterized conserved protein			
148643726	hypothetical protein Msm_1666	No	0	Virus_Caudovi	1704123	1704467	+		Methanobrevibacter_smithii_ATCC_35061_uid588	NC_009515	chromosome											
148643727	hypothetical protein Msm_1667	No	0	Virus_Caudovi	1704608	1704859	+		Methanobrevibacter_smithii_ATCC_35061_uid588	NC_009515	chromosome											
148643728	hypothetical protein Msm_1668	No	1	Virus_Caudovi	1705453	1705626	+		Methanobrevibacter_smithii_ATCC_35061_uid588	NC_009515	chromosome											
148643729	hypothetical protein Msm_1669	No	1	Virus_Caudovi	1705616	1705915	+		Methanobrevibacter_smithii_ATCC_35061_uid588	NC_009515	chromosome											
148643730	hypothetical protein Msm_1670	No	0	Virus_Caudovi	1706074	1706667	+		Methanobrevibacter_smithii_ATCC_35061_uid588	NC_009515	chromosome			arCOG11382	5		Y		Phage terminase small subunit			
148643731	large terminase subunit	No	0	Virus_Caudovi	1706664	1708022	+		Methanobrevibacter_smithii_ATCC_35061_uid588	NC_009515	chromosome			arCOG09550	17	(-)	Y	TerL	Phage terminase large subunit			
148643732	bacteriophage capsid portal protein	No	0	Virus_Caudovi	1708025	1709431	+		Methanobrevibacter_smithii_ATCC_35061_uid588	NC_009515	chromosome			arCOG11199	3		S		Uncharacterized conserved protein			
148643733	hypothetical protein Msm_1673	No	0	Virus_Caudovi	1709428	1711056	+		Methanobrevibacter_smithii_ATCC_35061_uid588	NC_009515	chromosome									0.00000005	pfam03496	ADP-ribosyltransferase exoenzyme. This i
148643734	hypothetical protein Msm_1674	No	0	Virus_Caudovi	1711113	1711439	+		Methanobrevibacter_smithii_ATCC_35061_uid588	NC_009515	chromosome											
148643735	putative structural protein (prophage psiM100)	No	0	Virus_Caudovi	1711630	1711977	+		Methanobrevibacter_smithii_ATCC_35061_uid588	NC_009515	chromosome											
148643736	hypothetical protein Msm_1676	No	0	Virus_Caudovi	1711980	1712594	+		Methanobrevibacter_smithii_ATCC_35061_uid588	NC_009515	chromosome											
148643737	putative major capsid protein gp5 (phage psiM	No	0	Virus_Caudovi	1712653	1713663	+		Methanobrevibacter_smithii_ATCC_35061_uid588	NC_009515	chromosome											
148643738	hypothetical protein Msm_1678	No	0	Virus_Caudovi	1713954	1714319	+		Methanobrevibacter_smithii_ATCC_35061_uid588	NC_009515	chromosome			arCOG11165	2		S		Uncharacterized conserved protein			
148643739	hypothetical protein Msm_1679	No	0	Virus_Caudovi	1714359	1714802	+		Methanobrevibacter_smithii_ATCC_35061_uid588	NC_009515	chromosome											
148643740	hypothetical protein Msm_1680	No	0	Virus_Caudovi	1714799	1715260	+		Methanobrevibacter_smithii_ATCC_35061_uid588	NC_009515	chromosome											
148643741	hypothetical protein Msm_1681	No	0	Virus_Caudovi	1715266	1716507	+		Methanobrevibacter_smithii_ATCC_35061_uid588	NC_009515	chromosome											
148643742	hypothetical protein Msm_1682	No	0	Virus_Caudovi	1716590	1717024	+		Methanobrevibacter_smithii_ATCC_35061_uid588	NC_009515	chromosome											
148643743	hypothetical protein Msm_1683	No	0	Virus_Caudovi	1717011	1717244	+		Methanobrevibacter_smithii_ATCC_35061_uid588	NC_009515	chromosome											
148643744	phage-related minor tail protein	No	5	Virus_Caudovi	1717244	1721725	+		Methanobrevibacter_smithii_ATCC_35061_uid588	NC_009515	chromosome									0.0001	COG5412	Phage-related protein
148643745	hypothetical protein Msm_1685	No	0	Virus_Caudovi	1721732	1722469	+		Methanobrevibacter_smithii_ATCC_35061_uid588	NC_009515	chromosome											
148643746	hypothetical protein Msm_1686	No	0	Virus_Caudovi	1722447	1725317	+		Methanobrevibacter_smithii_ATCC_35061_uid588	NC_009515	chromosome											
148643747	hypothetical protein Msm_1687	No	0	Virus_Caudovi	1725323	1727866	+		Methanobrevibacter_smithii_ATCC_35061_uid588	NC_009515	chromosome											
148643748	hypothetical protein Msm_1688	No	0	Virus_Caudovi	1727863	1729512	+		Methanobrevibacter_smithii_ATCC_35061_uid588	NC_009515	chromosome											
148643749	putative collagen-like protein B	Yes	0	Virus_Caudovi	1729521	1730132	+		Methanobrevibacter_smithii_ATCC_35061_uid588	NC_009515	chromosome											
148643750	hypothetical protein Msm_1690	No	0	Virus_Caudovi	1730158	1730310	+		Methanobrevibacter_smithii_ATCC_35061_uid588	NC_009515	chromosome											
148643751	putative PeiW-related protein (phage psiM100)	No	0	Virus_Caudovi	1730303	1731121	+		Methanobrevibacter_smithii_ATCC_35061_uid588	NC_009515	chromosome									9E-39	pfam12386	Pseudomurein endo-isopeptidase Pei. This
148643752	hypothetical protein Msm_1692	No	0	Virus_Caudovi	1731593	1731934	+		Methanobrevibacter_smithii_ATCC_35061_uid588	NC_009515	chromosome											
148643753	ribokinase PfkB family	No	0	Virus_Caudovi	1733043	1733888	+		Methanobrevibacter_smithii_ATCC_35061_uid588	NC_009515	chromosome			arCOG00016	1	(-)	G	RbsK	Sugar kinase, ribokinase family			
===								11	Methanobrevibacter_smithii_ATCC_35061_uid588	NC_009515	chromosome											
148643815	purine/pyrimidine phosphoribosyl transferase	No	0		1818868	1819917	+		Methanobrevibacter_smithii_ATCC_35061_uid588	NC_009515	chromosome											
148643816	Smf protein	No	0		1819928	1820866	+		Methanobrevibacter_smithii_ATCC_35061_uid588	NC_009515	chromosome			arCOG02431	4	(-)	R		Predicted Rossmann fold nucleotide-binding pro	2E-31	pfam02481	DNA recombination-mediator protein A. Th
148643817	hypothetical protein Msm_1757	No	0		1821233	1821883	+		Methanobrevibacter_smithii_ATCC_35061_uid588	NC_009515	chromosome											
148643818	hypothetical protein Msm_1758	No	1		1822048	1822293	+		Methanobrevibacter_smithii_ATCC_35061_uid588	NC_009515	chromosome											
148643819	hypothetical protein Msm_1759	No	1		1822306	1822557	+		Methanobrevibacter_smithii_ATCC_35061_uid588	NC_009515	chromosome											
148643820	hypothetical protein Msm_1760	No	0		1822566	1823030	+		Methanobrevibacter_smithii_ATCC_35061_uid588	NC_009515	chromosome											
148643821	DNA repair ATPase	No	0		1823185	1825746	+		Methanobrevibacter_smithii_ATCC_35061_uid588	NC_009515	chromosome											
148643822	hypothetical protein Msm_1762	No	0		1825749	1826918	+		Methanobrevibacter_smithii_ATCC_35061_uid588	NC_009515	chromosome											
148643823	DNA-directed RNA polymerase, subunit M, Rp	No	0		1826922	1827161	+		Methanobrevibacter_smithii_ATCC_35061_uid588	NC_009515	chromosome			arCOG00579	4	(-)	K	RPB9	DNA-directed RNA polymerase, subunit M/Transc	0.0000002	COG1594	DNA-directed RNA polymerase, subunit M/T
148643824	hypothetical protein Msm_1764	No	0		1827222	1827434	-		Methanobrevibacter_smithii_ATCC_35061_uid588	NC_009515	chromosome											
148643825	hypothetical protein Msm_1765	No	0		1827534	1828403	-		Methanobrevibacter_smithii_ATCC_35061_uid588	NC_009515	chromosome			arCOG10868	7		L		PD-(D/E)XK nuclease family transposase			
===								6	Methanobrevibacter_smithii_ATCC_35061_uid588	NC_009515	chromosome											
148643841	hypothetical protein Msm_1781	No	3	YES	1838754	1839230	+		Methanobrevibacter_smithii_ATCC_35061_uid588	NC_009515	chromosome			arCOG01917	12	(-)	S		Zn-ribbon domain containing protein	0.00005	pfam12773	Double zinc ribbon. This family consists
148643842	hypothetical protein Msm_1782	No	0	YES	1839408	1840097	+		Methanobrevibacter_smithii_ATCC_35061_uid588	NC_009515	chromosome											
148643843	hypothetical protein Msm_1783	No	0	YES	1840101	1840430	-		Methanobrevibacter_smithii_ATCC_35061_uid588	NC_009515	chromosome											
148643844	hypothetical protein Msm_1784	No	0	YES	1840447	1840668	-		Methanobrevibacter_smithii_ATCC_35061_uid588	NC_009515	chromosome											
148643845	hypothetical protein Msm_1785	No	0	YES	1840668	1841570	-		Methanobrevibacter_smithii_ATCC_35061_uid588	NC_009515	chromosome			arCOG10868	7		L		PD-(D/E)XK nuclease family transposase			
148643846	O-linked GlcNAc transferase	No	0		1841810	1842331	+		Methanobrevibacter_smithii_ATCC_35061_uid588	NC_009515	chromosome			arCOG03032	12	(-)	R	NrfG	TPR repeats containing protein	0.000002	COG0457	FOG: TPR repeat
===								11	Methanocaldococcus_FS406_22_uid42499	NC_013887	chromosome											
289192583	hypothetical protein MFS40622_1108	No	0		1099012	1099932	+		Methanocaldococcus_FS406_22_uid42499	NC_013887	chromosome			arCOG08185	1	(-)	S		Uncharacterized conserved protein	0.000002	COG1196	Chromosome segregation ATPases
289192584	hypothetical protein MFS40622_1109	No	0		1099946	1100461	+		Methanocaldococcus_FS406_22_uid42499	NC_013887	chromosome											
289192585	hypothetical protein MFS40622_1110	No	0		1100860	1101108	+		Methanocaldococcus_FS406_22_uid42499	NC_013887	chromosome											
289192586	hypothetical protein MFS40622_1111	No	0		1101135	1101281	+		Methanocaldococcus_FS406_22_uid42499	NC_013887	chromosome											
289192587	hypothetical protein MFS40622_1112	No	0		1101353	1101679	+		Methanocaldococcus_FS406_22_uid42499	NC_013887	chromosome											
289192588	hypothetical protein MFS40622_1113	No	0		1101676	1101951	+		Methanocaldococcus_FS406_22_uid42499	NC_013887	chromosome			arCOG09654	3		Y		Predicted phage protein			
289192589	hypothetical protein MFS40622_1114	No	0		1102003	1102227	+		Methanocaldococcus_FS406_22_uid42499	NC_013887	chromosome											
289192590	hypothetical protein MFS40622_1115	No	0		1102249	1102665	+		Methanocaldococcus_FS406_22_uid42499	NC_013887	chromosome											
289192591	hypothetical protein MFS40622_1116	No	1		1102760	1102876	+		Methanocaldococcus_FS406_22_uid42499	NC_013887	chromosome	clust_506										
289192592	hypothetical protein MFS40622_1117	No	2		1102884	1103105	-		Methanocaldococcus_FS406_22_uid42499	NC_013887	chromosome	clust_430										
289192593	Tetratricopeptide TPR_2 repeat protein	Yes	0		1103551	1104288	+		Methanocaldococcus_FS406_22_uid42499	NC_013887	chromosome			arCOG03038	9	(-)	R	NrfG	TPR repeats containing protein	0.000004	COG0457	FOG: TPR repeat
===								13	Methanocaldococcus_FS406_22_uid42499	NC_013887	chromosome											
289192617	hypothetical protein MFS40622_1142	No	0		1122249	1122620	-		Methanocaldococcus_FS406_22_uid42499	NC_013887	chromosome			arCOG06595	3	(-)	S		Uncharacterized conserved protein			
289192618	hypothetical protein MFS40622_1143	No	0		1123482	1123745	-		Methanocaldococcus_FS406_22_uid42499	NC_013887	chromosome			arCOG10934	3		S		Uncharacterized conserved protein			
289192619	hypothetical protein MFS40622_1144	No	0		1123869	1124135	-		Methanocaldococcus_FS406_22_uid42499	NC_013887	chromosome			arCOG10934	3		S		Uncharacterized conserved protein			
289192620	hypothetical protein MFS40622_1145	No	0		1124186	1124362	-		Methanocaldococcus_FS406_22_uid42499	NC_013887	chromosome			arCOG10934	3		S		Uncharacterized conserved protein			
289192621	hypothetical protein MFS40622_1147	No	0		1124718	1124810	-		Methanocaldococcus_FS406_22_uid42499	NC_013887	chromosome											
289192622	hypothetical protein MFS40622_1148	No	0		1125093	1125263	-		Methanocaldococcus_FS406_22_uid42499	NC_013887	chromosome			arCOG09567	3		S		Uncharacterized conserved protein			
289192623	hypothetical protein MFS40622_1149	No	0		1125303	1125734	-		Methanocaldococcus_FS406_22_uid42499	NC_013887	chromosome			arCOG09630	4		V		Minimal nucleotidyltransferase, toxin-antitoxin system component			
289192624	hypothetical protein MFS40622_1150	Yes	1		1125776	1126693	-		Methanocaldococcus_FS406_22_uid42499	NC_013887	chromosome											
289192625	hypothetical protein MFS40622_1151	No	0		1126815	1126916	-		Methanocaldococcus_FS406_22_uid42499	NC_013887	chromosome											
289192626	hypothetical protein MFS40622_1152	No	0		1126990	1127370	+		Methanocaldococcus_FS406_22_uid42499	NC_013887	chromosome			arCOG09630	4		V		Minimal nucleotidyltransferase, toxin-antitoxin system component			
289192627	hypothetical protein MFS40622_1153	No	0		1127375	1127878	+		Methanocaldococcus_FS406_22_uid42499	NC_013887	chromosome			arCOG06576	5	(-)	V		HEPN domain containing protein			
289192628	hypothetical protein MFS40622_1154	No	0		1128229	1128549	-		Methanocaldococcus_FS406_22_uid42499	NC_013887	chromosome			arCOG09630	4		V		Minimal nucleotidyltransferase, toxin-antitoxin system component			
289192629	hypothetical protein MFS40622_1155	No	0		1128910	1129218	+		Methanocaldococcus_FS406_22_uid42499	NC_013887	chromosome											
===								8	Methanocaldococcus_FS406_22_uid42499	NC_013887	chromosome											
289192846	protein of unknown function DUF95 transmemb	No	5		1354611	1355168	-		Methanocaldococcus_FS406_22_uid42499	NC_013887	chromosome			arCOG01996	17	(-)	S	SpoIIM	Uncharacterized membrane protein	2E-21	COG1300	Uncharacterized membrane protein
289192847	hypothetical protein MFS40622_1376	No	4		1355150	1355635	-		Methanocaldococcus_FS406_22_uid42499	NC_013887	chromosome											
289192848	hypothetical protein MFS40622_1377	Yes	1		1355691	1355972	-		Methanocaldococcus_FS406_22_uid42499	NC_013887	chromosome			arCOG09673	5		S		Uncharacterized conserved protein			
289192849	hypothetical protein MFS40622_1378	No	2		1356246	1356419	-		Methanocaldococcus_FS406_22_uid42499	NC_013887	chromosome			arCOG10810	3		S		Uncharacterized conserved protein			
289192850	hypothetical protein MFS40622_1379	No	4		1356957	1357418	-		Methanocaldococcus_FS406_22_uid42499	NC_013887	chromosome			arCOG10443	4		S		Uncharacterized conserved protein			
289192851	protein of unknown function DUF1112	No	7		1357645	1358394	-		Methanocaldococcus_FS406_22_uid42499	NC_013887	chromosome			arCOG06646	1	(-)	S		Predicted membrane protein	3E-33	COG4760	Predicted membrane protein
289192852	hypothetical protein MFS40622_1381	No	1		1358441	1358932	-		Methanocaldococcus_FS406_22_uid42499	NC_013887	chromosome			arCOG11107	3		S		Uncharacterized conserved protein			
289192853	hypothetical protein MFS40622_1382	No	3		1359335	1359652	-		Methanocaldococcus_FS406_22_uid42499	NC_013887	chromosome			arCOG10305	3		S		Uncharacterized conserved protein			
===								6	Methanocaldococcus_FS406_22_uid42499	NC_013887	chromosome											
289192949	fibrillarin	No	0		1456150	1456839	-		Methanocaldococcus_FS406_22_uid42499	NC_013887	chromosome			arCOG00078	1	(-)	J	NOP1	Fibrillarin-like rRNA methylase	5E-98	COG1889	Fibrillarin-like rRNA methylase
289192950	hypothetical protein MFS40622_1482	No	0		1456906	1457646	-		Methanocaldococcus_FS406_22_uid42499	NC_013887	chromosome									0.00004	COG0419	ATPase involved in DNA repair
289192951	hypothetical protein MFS40622_1483	No	3		1457803	1458327	+		Methanocaldococcus_FS406_22_uid42499	NC_013887	chromosome			arCOG09672	1		S		Uncharacterized conserved protein			
289192952	hypothetical protein MFS40622_1484	No	2		1458332	1458784	+		Methanocaldococcus_FS406_22_uid42499	NC_013887	chromosome			arCOG10950	1		S		Uncharacterized conserved protein			
289192953	hypothetical protein MFS40622_1485	No	1		1458792	1458896	+		Methanocaldococcus_FS406_22_uid42499	NC_013887	chromosome											
289192954	Pre-mRNA processing ribonucleoprotein, bind	No	1		1458897	1460180	-		Methanocaldococcus_FS406_22_uid42499	NC_013887	chromosome			arCOG01923	1	(-)	J	SIK1	Protein implicated in ribosomal biogenesis, Nop5	3E-94	COG1498	Protein implicated in ribosomal biogenes
===								6	Methanocaldococcus_FS406_22_uid42499	NC_013887	chromosome											
289193103	hypothetical protein MFS40622_1637	No	6		1589929	1591122	+		Methanocaldococcus_FS406_22_uid42499	NC_013887	chromosome			arCOG01462	1	(-)	C	NatB	ABC-type Na+ efflux pump, permease componen	1E-34	COG1668	ABC-type Na+ efflux pump, permease compo
289193104	hypothetical protein MFS40622_1638	No	1		1591391	1591537	+		Methanocaldococcus_FS406_22_uid42499	NC_013887	chromosome											
289193105	hypothetical protein MFS40622_1639	No	4		1591545	1592129	+		Methanocaldococcus_FS406_22_uid42499	NC_013887	chromosome											
289193106	hypothetical protein MFS40622_1640	No	6		1592268	1592768	+		Methanocaldococcus_FS406_22_uid42499	NC_013887	chromosome	clust_214										
289193107	hypothetical protein MFS40622_1641	No	4		1592761	1593219	+		Methanocaldococcus_FS406_22_uid42499	NC_013887	chromosome	clust_230										
289193108	protein of unknown function DUF1063	No	0		1593351	1594517	-		Methanocaldococcus_FS406_22_uid42499	NC_013887	chromosome			arCOG05065	1	(-)	E		L-cysteine desulfidase	2E-54	COG3681	L-cysteine desulfidase
===								33	Methanocaldococcus_fervens_AG86_uid59347	NC_013156	chromosome											
256811474	hypothetical protein Mefer_1540	No	0	Virus_Caudovi	1449627	1450058	-		Methanocaldococcus_fervens_AG86_uid59347	NC_013156	chromosome			arCOG09635	2		S		Uncharacterized conserved protein			
256811475	phage head morphogenesis protein, SPP1 gp	No	0	Virus_Caudovi	1450060	1451895	-		Methanocaldococcus_fervens_AG86_uid59347	NC_013156	chromosome			arCOG09636	4		Y		Phage protein containing C-terminal domain relat	0.000000008	pfam04233	Phage Mu protein F like protein. Members ; ATPase involved in DNA repair
256811476	hypothetical protein Mefer_1542	No	0	Virus_Caudovi	1451904	1453499	-		Methanocaldococcus_fervens_AG86_uid59347	NC_013156	chromosome		arCOG09550	arCOG09550	17		Y	TerL	Phage terminase large subunit			
256811477	hypothetical protein Mefer_1543	No	0	Virus_Caudovi	1453499	1454035	-		Methanocaldococcus_fervens_AG86_uid59347	NC_013156	chromosome			arCOG08300	5	(-)	Y		Phage virion morphogenesis family, Mu gpG-like			
256811478	hypothetical protein Mefer_1544	No	0	Virus_Caudovi	1454035	1454346	-		Methanocaldococcus_fervens_AG86_uid59347	NC_013156	chromosome			arCOG09638	2		Y		Predicted phage protein			
256811479	hypothetical protein Mefer_1545	No	2	Virus_Caudovi	1454373	1454753	-		Methanocaldococcus_fervens_AG86_uid59347	NC_013156	chromosome			arCOG09639	2		Y		Predicted phage protein			
256811480	putative addiction module antidote protein	No	0	Virus_Caudovi	1454758	1455033	-		Methanocaldococcus_fervens_AG86_uid59347	NC_013156	chromosome			arCOG09640	2		Y		Transcriptional regulator, contains HTH domain			
256811481	hypothetical protein Mefer_1547	No	0	Virus_Caudovi	1455181	1455477	+		Methanocaldococcus_fervens_AG86_uid59347	NC_013156	chromosome			arCOG08301	4		Y		Predicted phage protein	0.0001	pfam05483	Synaptonemal complex protein 1 (SCP-1).
256811482	hypothetical protein Mefer_1548	No	0	Virus_Caudovi	1455548	1456645	+		Methanocaldococcus_fervens_AG86_uid59347	NC_013156	chromosome			arCOG09508	6		Y		Phage prohead protease			
256811483	hypothetical protein Mefer_1549	No	0	Virus_Caudovi	1456655	1456981	+		Methanocaldococcus_fervens_AG86_uid59347	NC_013156	chromosome			arCOG09641	3		Y		Predicted phage protein			
256811484	hypothetical protein Mefer_1550	No	0	Virus_Caudovi	1456995	1457753	+		Methanocaldococcus_fervens_AG86_uid59347	NC_013156	chromosome			arCOG09642	3		Y		Predicted phage protein			
256811485	hypothetical protein Mefer_1551	No	0	Virus_Caudovi	1457757	1458068	+		Methanocaldococcus_fervens_AG86_uid59347	NC_013156	chromosome			arCOG09643	3		Y		Predicted phage protein			
256811486	hypothetical protein Mefer_1552	No	0	Virus_Caudovi	1458037	1458360	+		Methanocaldococcus_fervens_AG86_uid59347	NC_013156	chromosome			arCOG09644	2		Y		Predicted phage protein			
256811487	hypothetical protein Mefer_1553	No	0	Virus_Caudovi	1458360	1459064	+		Methanocaldococcus_fervens_AG86_uid59347	NC_013156	chromosome			arCOG09645	3		Y		Predicted phage protein			
256811488	hypothetical protein Mefer_1554	No	0	Virus_Caudovi	1459074	1459439	+		Methanocaldococcus_fervens_AG86_uid59347	NC_013156	chromosome			arCOG09646	2		Y		Predicted phage protein			
256811489	hypothetical protein Mefer_1555	No	3	Virus_Caudovi	1459584	1459955	+		Methanocaldococcus_fervens_AG86_uid59347	NC_013156	chromosome			arCOG09647	2		Y		Predicted phage protein			
256811490	hypothetical protein Mefer_1556	No	0	Virus_Caudovi	1459963	1462476	+		Methanocaldococcus_fervens_AG86_uid59347	NC_013156	chromosome			arCOG11380	2	(-)	Y		Phage tail tape measure protein	2E-10	COG1196	Chromosome segregation ATPases
256811491	hypothetical protein Mefer_1557	No	0	Virus_Caudovi	1462488	1463045	+		Methanocaldococcus_fervens_AG86_uid59347	NC_013156	chromosome			arCOG09648	2		Y		Predicted phage protein			
256811492	hypothetical protein Mefer_1558	No	0	Virus_Caudovi	1463184	1464113	+		Methanocaldococcus_fervens_AG86_uid59347	NC_013156	chromosome			arCOG09649	3		Y		Predicted phage protein			
256811493	hypothetical protein Mefer_1559	No	0	Virus_Caudovi	1464110	1464664	+		Methanocaldococcus_fervens_AG86_uid59347	NC_013156	chromosome			arCOG09650	4		Y		Predicted phage protein			
256811494	hypothetical protein Mefer_1560	No	1	Virus_Caudovi	1464796	1467027	+		Methanocaldococcus_fervens_AG86_uid59347	NC_013156	chromosome			arCOG09651	3		Y		Predicted phage protein			
256811495	hypothetical protein Mefer_1561	No	1	Virus_Caudovi	1467070	1467387	-		Methanocaldococcus_fervens_AG86_uid59347	NC_013156	chromosome			arCOG09652	2		Y		Predicted phage protein			
256811496	hypothetical protein Mefer_1562	No	0	Virus_Caudovi	1467455	1467697	-		Methanocaldococcus_fervens_AG86_uid59347	NC_013156	chromosome			arCOG09653	2		Y		Predicted phage protein			
256811497	hypothetical protein Mefer_1563	No	0	Virus_Caudovi	1467737	1468669	-		Methanocaldococcus_fervens_AG86_uid59347	NC_013156	chromosome			arCOG00417	2	(-)	L	RecA	RecA/RadA recombinase			
256811498	hypothetical protein Mefer_1564	No	0	Virus_Caudovi	1468647	1468910	-		Methanocaldococcus_fervens_AG86_uid59347	NC_013156	chromosome			arCOG08302	4		S		Uncharacterized conserved protein			
256811499	hypothetical protein Mefer_1565	No	0	Virus_Caudovi	146	1469037	-		Methanocaldococcus_fervens_AG86_uid59347	NC_013156	chromosome											
256811500	Transcription factor CBF/NF-Y/histone domain	No	0	Virus_Caudovi	1469104	1469388	-		Methanocaldococcus_fervens_AG86_uid59347	NC_013156	chromosome			arCOG02144	3	(-)	L	HHT1	Histones H3 and H4	0.000005	COG2036	Histones H3 and H4
256811501	hypothetical protein Mefer_1567	No	0	Virus_Caudovi	1469385	1469639	-		Methanocaldococcus_fervens_AG86_uid59347	NC_013156	chromosome			arCOG09654	3		Y		Predicted phage protein			
256811502	hypothetical protein Mefer_1568	No	2	Virus_Caudovi	1469977	1470198	+		Methanocaldococcus_fervens_AG86_uid59347	NC_013156	chromosome	clust_430										
256811503	hypothetical protein Mefer_1569	No	1	Virus_Caudovi	1470195	1470320	-		Methanocaldococcus_fervens_AG86_uid59347	NC_013156	chromosome	clust_506										
256811504	hypothetical protein Mefer_1570	No	1	Virus_Caudovi	1470551	1471384	+		Methanocaldococcus_fervens_AG86_uid59347	NC_013156	chromosome											
256811505	hypothetical protein Mefer_1571	No	0	Virus_Caudovi	1471381	1472139	-		Methanocaldococcus_fervens_AG86_uid59347	NC_013156	chromosome			arCOG09655	2		S		Uncharacterized conserved protein			
256811506	hypothetical protein Mefer_1572	No	0	Virus_Caudovi	1472498	1472821	+		Methanocaldococcus_fervens_AG86_uid59347	NC_013156	chromosome			arCOG06614	1	(-)	S		Uncharacterized conserved protein			
===								13	Methanocaldococcus_fervens_AG86_uid59347	NC_013157	plasmid pMEFER01											
256811533	hypothetical protein Mefer_1598	No	0	plasmid	8903	9235	-		Methanocaldococcus_fervens_AG86_uid59347	NC_013157	plasmid pMEFER01			arCOG06595	3	(-)	S		Uncharacterized conserved protein			
256811534	hypothetical protein Mefer_1599	No	0	plasmid	9482	9688	-		Methanocaldococcus_fervens_AG86_uid59347	NC_013157	plasmid pMEFER01											
256811535	hypothetical protein Mefer_1600	No	0	plasmid	9702	9929	-		Methanocaldococcus_fervens_AG86_uid59347	NC_013157	plasmid pMEFER01									0.0001	COG4026	Uncharacterized protein containing TOPRI
256811536	hypothetical protein Mefer_1601	No	0	plasmid	9898	10125	-		Methanocaldococcus_fervens_AG86_uid59347	NC_013157	plasmid pMEFER01											
256811537	hypothetical protein Mefer_1602	No	0	plasmid	10128	10511	-		Methanocaldococcus_fervens_AG86_uid59347	NC_013157	plasmid pMEFER01											
256811538	hypothetical protein Mefer_1603	No	0	plasmid	10562	10894	-		Methanocaldococcus_fervens_AG86_uid59347	NC_013157	plasmid pMEFER01			arCOG09656	2		S		Uncharacterized conserved protein			
256811539	hypothetical protein Mefer_1604	No	0	plasmid	10921	11037	-		Methanocaldococcus_fervens_AG86_uid59347	NC_013157	plasmid pMEFER01											
256811540	hypothetical protein Mefer_1605	No	0	plasmid	11067	11327	-		Methanocaldococcus_fervens_AG86_uid59347	NC_013157	plasmid pMEFER01											
256811541	hypothetical protein Mefer_1606	No	0	plasmid	11380	11907	-		Methanocaldococcus_fervens_AG86_uid59347	NC_013157	plasmid pMEFER01											
256811542	hypothetical protein Mefer_1607	No	0	plasmid	11982	12143	-		Methanocaldococcus_fervens_AG86_uid59347	NC_013157	plasmid pMEFER01											
256811543	hypothetical protein Mefer_1608	No	0	plasmid	12208	12414	-		Methanocaldococcus_fervens_AG86_uid59347	NC_013157	plasmid pMEFER01											
256811544	hypothetical protein Mefer_1609	No	0	plasmid	12706	12876	+		Methanocaldococcus_fervens_AG86_uid59347	NC_013157	plasmid pMEFER01											
256811545	MCM family protein	No	0	plasmid	13645	15765	+		Methanocaldococcus_fervens_AG86_uid59347	NC_013157	plasmid pMEFER01			arCOG00439	14	(-)	L	MCM2	Predicted ATPase involved in replication control,	9E-139	COG1241	Predicted ATPase involved in replication
===								6	Methanocaldococcus_infernus_ME_uid48803	NC_014122	chromosome											
296108717	hypothetical protein Metin_0029	No	0		25686	25904	-		Methanocaldococcus_infernus_ME_uid48803	NC_014122	chromosome	clust_432										
296108718	hypothetical protein Metin_0030	Yes	2		27060	28523	-		Methanocaldococcus_infernus_ME_uid48803	NC_014122	chromosome			arCOG10210	2		S		Uncharacterized conserved protein	0.00002	pfam05316	Mitochondrial ribosomal protein (VAR1).
296108719	hypothetical protein Metin_0032	No	0		29358	29576	+		Methanocaldococcus_infernus_ME_uid48803	NC_014122	chromosome	clust_432										
296108720	oxidoreductase/nitrogenase component 1	No	0		29607	30962	-		Methanocaldococcus_infernus_ME_uid48803	NC_014122	chromosome			arCOG00598	2	(-)	C	NifD	Nitrogenase molybdenum-iron protein, alpha or b	6E-75	COG2710	Nitrogenase molybdenum-iron protein, alp
296108721	hypothetical protein Metin_0034	No	0		30965	31597	-		Methanocaldococcus_infernus_ME_uid48803	NC_014122	chromosome			arCOG10306	1		S		Uncharacterized conserved protein			
296108722	hypothetical protein Metin_0035	No	0		31660	31917	-		Methanocaldococcus_infernus_ME_uid48803	NC_014122	chromosome			arCOG10190	1		S		Uncharacterized conserved protein			
===								14	Methanocaldococcus_infernus_ME_uid48803	NC_014122	chromosome											
296109976	hypothetical protein Metin_1311	No	4		1178171	1178581	+		Methanocaldococcus_infernus_ME_uid48803	NC_014122	chromosome	clust_254										
296109977	protein of unknown function DUF95 transmemb	No	4		1178586	1179122	+		Methanocaldococcus_infernus_ME_uid48803	NC_014122	chromosome			arCOG01996	17	(-)	S	SpoIIM	Uncharacterized membrane protein	1E-27	pfam01944	Integral membrane protein DUF95. Members
296109978	hypothetical protein Metin_1313	Yes	2		1179351	1179614	+		Methanocaldococcus_infernus_ME_uid48803	NC_014122	chromosome			arCOG10897	10		S		Uncharacterized conserved protein			
296109979	SLC47A2; solute carrier family 47, member 2	Yes	2		1180202	1180429	+		Methanocaldococcus_infernus_ME_uid48803	NC_014122	chromosome											
296109980	hypothetical protein Metin_1315	No	4		1180459	1180881	+		Methanocaldococcus_infernus_ME_uid48803	NC_014122	chromosome											
296109981	protein of unknown function DUF95 transmemb	No	5		1180878	1181402	+		Methanocaldococcus_infernus_ME_uid48803	NC_014122	chromosome			arCOG01996	17	(-)	S	SpoIIM	Uncharacterized membrane protein	7E-19	pfam01944	Integral membrane protein DUF95. Members
296109982	hypothetical protein Metin_1317	Yes	3		1181666	1181962	+		Methanocaldococcus_infernus_ME_uid48803	NC_014122	chromosome			arCOG10897	10		S		Uncharacterized conserved protein			
296109983	hypothetical protein Metin_1318	No	3		1182031	1182357	+		Methanocaldococcus_infernus_ME_uid48803	NC_014122	chromosome			arCOG10305	3		S		Uncharacterized conserved protein			
296109984	protein of unknown function DUF95 transmemb	No	4		1182380	1182925	+		Methanocaldococcus_infernus_ME_uid48803	NC_014122	chromosome			arCOG01996	17	(-)	S	SpoIIM	Uncharacterized membrane protein	1E-20	COG1300	Uncharacterized membrane protein
296109985	hypothetical protein Metin_1320	Yes	3		1183191	1183508	+		Methanocaldococcus_infernus_ME_uid48803	NC_014122	chromosome			arCOG09673	5		S		Uncharacterized conserved protein			
296109986	hypothetical protein Metin_1321	No	2		1183571	1183750	+		Methanocaldococcus_infernus_ME_uid48803	NC_014122	chromosome			arCOG10810	3		S		Uncharacterized conserved protein			
296109987	protein of unknown function DUF95 transmemb	No	3		1183734	1184210	+		Methanocaldococcus_infernus_ME_uid48803	NC_014122	chromosome			arCOG01996	17	(-)	S	SpoIIM	Uncharacterized membrane protein	2E-23	pfam01944	Integral membrane protein DUF95. Members
296109988	hypothetical protein Metin_1323	Yes	3		1184478	1184798	+		Methanocaldococcus_infernus_ME_uid48803	NC_014122	chromosome			arCOG10897	10		S		Uncharacterized conserved protein			
296109989	hypothetical protein Metin_1324	No	4		1184850	1185230	+		Methanocaldococcus_infernus_ME_uid48803	NC_014122	chromosome											
===								7	Methanocaldococcus_jannaschii_DSM_2661_uid	NC_000909	chromosome											
15668352	30S ribosomal protein S19P	No	0		181395	181853	+		Methanocaldococcus_jannaschii_DSM_2661_uid	NC_000909	chromosome			arCOG04099	1	(-)	J	RpsS	Ribosomal protein S19	2E-19	COG0185	Ribosomal protein S19
15668353	hypothetical protein MJ_0181	No	1		181912	182652	-		Methanocaldococcus_jannaschii_DSM_2661_uid	NC_000909	chromosome											
15668354	hypothetical protein MJ_0182	No	0		182724	183308	-		Methanocaldococcus_jannaschii_DSM_2661_uid	NC_000909	chromosome									1E-22	COG4928	Predicted P-loop ATPase
15668355	hypothetical protein MJ_0183	No	0		183342	183488	-		Methanocaldococcus_jannaschii_DSM_2661_uid	NC_000909	chromosome											
15668356	hypothetical protein MJ_0184	No	2		183594	183827	+		Methanocaldococcus_jannaschii_DSM_2661_uid	NC_000909	chromosome											
15668357	hypothetical protein MJ_0185	No	1		183883	184032	+		Methanocaldococcus_jannaschii_DSM_2661_uid	NC_000909	chromosome											
15668358	bifunctional ornithine acetyltransferase/N-acetyl	No	0		184215	185423	-		Methanocaldococcus_jannaschii_DSM_2661_uid	NC_000909	chromosome			arCOG04413	1	(-)	E	ArgJ	N-acetylglutamate synthase (N-acetylornithine am	2E-123	COG1364	N-acetylglutamate synthase (N-acetylorni
===								35	Methanocaldococcus_jannaschii_DSM_2661_uid	NC_000909	chromosome											

	15668502	hypothetical protein MJ_0328	No	0	YES	Virus_Caudovi	304312	304752	-		Methanocaldococcus_jannaschii_DSM_2661_uid	NC_000909	chromosome		arCOG09635	2	S		Uncharacterized conserved protein			
	15668503	hypothetical protein MJ_0329	No	0	YES	Virus_Caudovi	304754	306604	-		Methanocaldococcus_jannaschii_DSM_2661_uid	NC_000909	chromosome		arCOG09636	4	Y		Phage protein containing C-terminal domain relat	2E-36	COG2369	Uncharacterized protein, homolog of phag
	15668504	hypothetical protein MJ_0330	No	0	YES	Virus_Caudovi	306614	308263	-		Methanocaldococcus_jannaschii_DSM_2661_uid	NC_000909	chromosome	arCOG09550	arCOG09550	17	Y	TerL	Phage terminase large subunit			
	15668505	hypothetical protein MJ_0331	No	0	YES	Virus_Caudovi	308260	308667	-		Methanocaldococcus_jannaschii_DSM_2661_uid	NC_000909	chromosome		arCOG08300	5 (-)	Y		Phage virion morphogenesis family, Mu gpG-like			
	15668506	hypothetical protein MJ_0332	No	0	YES	Virus_Caudovi	308672	309004	-		Methanocaldococcus_jannaschii_DSM_2661_uid	NC_000909	chromosome		arCOG09638	2	Y		Predicted phage protein			
	15668507	hypothetical protein MJ_0332.1	No	2	YES	Virus_Caudovi	309019	309417	-		Methanocaldococcus_jannaschii_DSM_2661_uid	NC_000909	chromosome		arCOG09639	2	Y		Predicted phage protein			
	15668508	hypothetical protein MJ_0333	No	0	YES	Virus_Caudovi	309404	309694	-		Methanocaldococcus_jannaschii_DSM_2661_uid	NC_000909	chromosome		arCOG09640	2	Y		Transcriptional regulator, contains HTH domain			
	15668509	hypothetical protein MJ_0334	No	0	YES	Virus_Caudovi	309804	310112	+		Methanocaldococcus_jannaschii_DSM_2661_uid	NC_000909	chromosome		arCOG08301	4	Y		Predicted phage protein	0.00006	COG1340	Uncharacterized archaeal coiled-coil pro
	15668510	hypothetical protein MJ_0335	No	0	YES	Virus_Caudovi	310176	310919	+		Methanocaldococcus_jannaschii_DSM_2661_uid	NC_000909	chromosome		arCOG09508	6	Y		Phage prohead protease			
	15668511	hypothetical protein MJ_0336	No	0	YES	Virus_Caudovi	310929	311288	+		Methanocaldococcus_jannaschii_DSM_2661_uid	NC_000909	chromosome		arCOG09641	3	Y		Predicted phage protein			
	15668512	hypothetical protein MJ_0337	No	0	YES	Virus_Caudovi	311296	312084	+		Methanocaldococcus_jannaschii_DSM_2661_uid	NC_000909	chromosome		arCOG09642	3	Y		Predicted phage protein			
	15668513	hypothetical protein MJ_0338	No	0	YES	Virus_Caudovi	312088	312402	+		Methanocaldococcus_jannaschii_DSM_2661_uid	NC_000909	chromosome		arCOG09643	3	Y		Predicted phage protein			
	15668514	hypothetical protein MJ_0339	No	0	YES	Virus_Caudovi	312371	312694	+		Methanocaldococcus_jannaschii_DSM_2661_uid	NC_000909	chromosome		arCOG09644	2	Y		Predicted phage protein			
	15668515	hypothetical protein MJ_0340	No	0	YES	Virus_Caudovi	312694	313398	+		Methanocaldococcus_jannaschii_DSM_2661_uid	NC_000909	chromosome		arCOG09645	3	Y		Predicted phage protein			
	15668516	hypothetical protein MJ_0341	No	0	YES	Virus_Caudovi	313408	313770	+		Methanocaldococcus_jannaschii_DSM_2661_uid	NC_000909	chromosome		arCOG09646	2	Y		Predicted phage protein			
	15668517	hypothetical protein MJ_0342	No	3	YES	Virus_Caudovi	313915	314286	+		Methanocaldococcus_jannaschii_DSM_2661_uid	NC_000909	chromosome		arCOG09647	2	Y		Predicted phage protein			
	15668518	hypothetical protein MJ_0343	No	0		Virus_Caudovi	314258	316807	+		Methanocaldococcus_jannaschii_DSM_2661_uid	NC_000909	chromosome		arCOG11380	2 (-)	Y		Phage tail tape measure protein	2E-10	COG1196	Chromosome segregation ATPases
	15668519	hypothetical protein MJ_0344	No	0		Virus_Caudovi	316817	317359	+		Methanocaldococcus_jannaschii_DSM_2661_uid	NC_000909	chromosome		arCOG09648	2	Y		Predicted phage protein			
	15668520	hypothetical protein MJ_0345	No	0		Virus_Caudovi	317311	318264	+		Methanocaldococcus_jannaschii_DSM_2661_uid	NC_000909	chromosome		arCOG09649	3	Y		Predicted phage protein			
	15668521	hypothetical protein MJ_0346	No	0		Virus_Caudovi	318274	318579	+		Methanocaldococcus_jannaschii_DSM_2661_uid	NC_000909	chromosome									
	15668522	hypothetical protein MJ_0347	No	0		Virus_Caudovi	318590	319045	+		Methanocaldococcus_jannaschii_DSM_2661_uid	NC_000909	chromosome									
	15668523	hypothetical protein MJ_0347.1	No	0		Virus_Caudovi	319063	319614	+		Methanocaldococcus_jannaschii_DSM_2661_uid	NC_000909	chromosome		arCOG09650	4	Y		Predicted phage protein			
	15668524	hypothetical protein MJ_0348	No	0		Virus_Caudovi	319608	321995	+		Methanocaldococcus_jannaschii_DSM_2661_uid	NC_000909	chromosome		arCOG09651	3	Y		Predicted phage protein			
	15668525	hypothetical protein MJ_0349	No	1		Virus_Caudovi	322047	322364	-		Methanocaldococcus_jannaschii_DSM_2661_uid	NC_000909	chromosome		arCOG09652	2	Y		Predicted phage protein			
	15668526	hypothetical protein MJ_0350	No	0		Virus_Caudovi	322412	322678	-		Methanocaldococcus_jannaschii_DSM_2661_uid	NC_000909	chromosome		arCOG09653	2	Y		Predicted phage protein			
	15668527	hypothetical protein MJ_0351	No	0		Virus_Caudovi	322699	323151	-		Methanocaldococcus_jannaschii_DSM_2661_uid	NC_000909	chromosome									
	15668528	hypothetical protein MJ_0352	No	0		Virus_Caudovi	323179	323898	-		Methanocaldococcus_jannaschii_DSM_2661_uid	NC_000909	chromosome		arCOG09665	3	S		Uncharacterized conserved protein	0.00009	COG1196	Chromosome segregation ATPases
	15668529	hypothetical protein MJ_0353	No	0		Virus_Caudovi	323885	324139	-		Methanocaldococcus_jannaschii_DSM_2661_uid	NC_000909	chromosome		arCOG08302	4	S		Uncharacterized conserved protein			
	15668530	hypothetical protein MJ_0354	No	0		Virus_Caudovi	324117	324293	-		Methanocaldococcus_jannaschii_DSM_2661_uid	NC_000909	chromosome		arCOG09666	3	S		Uncharacterized conserved protein			
	15668531	hypothetical protein MJ_0355	No	0		Virus_Caudovi	324368	324697	-		Methanocaldococcus_jannaschii_DSM_2661_uid	NC_000909	chromosome		arCOG09656	2	S		Uncharacterized conserved protein			
	15668532	hypothetical protein MJ_0356	No	0		Virus_Caudovi	324691	324984	-		Methanocaldococcus_jannaschii_DSM_2661_uid	NC_000909	chromosome		arCOG09654	3	Y		Predicted phage protein			
	15668533	hypothetical protein MJ_0357	No	0		Virus_Caudovi	325937	326404	-		Methanocaldococcus_jannaschii_DSM_2661_uid	NC_000909	chromosome							0.000000004	COG1952	Preprotein translocase subunit SecB
	15668534	hypothetical protein MJ_0358	No	0		Virus_Caudovi	326407	326793	-		Methanocaldococcus_jannaschii_DSM_2661_uid	NC_000909	chromosome									
	15668535	hypothetical protein MJ_0359	No	0		Virus_Caudovi	326802	327446	-		Methanocaldococcus_jannaschii_DSM_2661_uid	NC_000909	chromosome									
	15668536	hypothetical protein MJ_0360	Yes	1		Virus_Caudovi	327764	328171	-		Methanocaldococcus_jannaschii_DSM_2661_uid	NC_000909	chromosome		arCOG10172	3 (-)	S		Uncharacterized conserved protein			
===										6	Methanocaldococcus_jannaschii_DSM_2661_uid	NC_000909	chromosome									
	15669469	hypothetical protein MJ_1282.1	No	6			1229746	1230510	-		Methanocaldococcus_jannaschii_DSM_2661_uid	NC_000909	chromosome		arCOG02298	6 (-)	N	FlaK/PulC	Peptidase A24A, prepilin type IV			
	15669470	hypothetical protein MJ_1282.2	No	0			1230523	1231062	-		Methanocaldococcus_jannaschii_DSM_2661_uid	NC_000909	chromosome									
	15669471	hypothetical protein MJ_1283	No	2			1231011	1231673	-		Methanocaldococcus_jannaschii_DSM_2661_uid	NC_000909	chromosome		arCOG08268	1	S		Uncharacterized conserved protein			
	15669472	hypothetical protein MJ_1284	No	1			1231661	1232026	-		Methanocaldococcus_jannaschii_DSM_2661_uid	NC_000909	chromosome		arCOG08269	1	S		Uncharacterized conserved protein			
	15669473	hypothetical protein MJ_1285	No	1			1232023	1232577	-		Methanocaldococcus_jannaschii_DSM_2661_uid	NC_000909	chromosome		arCOG08270	1	S		Uncharacterized conserved protein			
	15669474	hypothetical protein MJ_1286	No	10			1232581	1234368	-		Methanocaldococcus_jannaschii_DSM_2661_uid	NC_000909	chromosome		arCOG01808	1 (-)	N	TadC	Flp pilus assembly protein TadC	2E-23	COG1955	Archaeal flagella assembly protein J
===										5	Methanocaldococcus_jannaschii_DSM_2661_uid	NC_000909	chromosome									
	15669477	hypothetical protein MJ_1288.1	No	1			1235904	1236275	-		Methanocaldococcus_jannaschii_DSM_2661_uid	NC_000909	chromosome		arCOG08271	1	S		Uncharacterized conserved protein			
	15669478	hypothetical protein MJ_1289	No	1			1236278	1236775	-		Methanocaldococcus_jannaschii_DSM_2661_uid	NC_000909	chromosome		arCOG08272	1	S		Uncharacterized conserved protein			
	15669479	hypothetical protein MJ_1290	Yes	2			1236772	1237710	-		Methanocaldococcus_jannaschii_DSM_2661_uid	NC_000909	chromosome		arCOG08273	1	S		Uncharacterized conserved protein			
	15669480	hypothetical protein MJ_1291	No	1			1237723	1238445	-		Methanocaldococcus_jannaschii_DSM_2661_uid	NC_000909	chromosome		arCOG05987	1 (-)	N		Pilin/Flagellin			
	15669481	hypothetical protein MJ_1292	Yes	0			1238659	1240317	+		Methanocaldococcus_jannaschii_DSM_2661_uid	NC_000909	chromosome									
===										6	Methanocaldococcus_jannaschii_DSM_2661_uid	NC_001732	plasmid large ECE									
	10954493	hypothetical protein MJECL01	No	0	YES	plasmid	302	1051	+		Methanocaldococcus_jannaschii_DSM_2661_uid	NC_001732	plasmid large ECE									
	10954494	hypothetical protein MJECL02	Yes	0	YES	plasmid	1082	1471	-		Methanocaldococcus_jannaschii_DSM_2661_uid	NC_001732	plasmid large ECE									
	10954495	hypothetical protein MJECL03	No	0	YES	plasmid	1374	1700	-		Methanocaldococcus_jannaschii_DSM_2661_uid	NC_001732	plasmid large ECE									
	10954496	putative ATPase	No	0	YES	plasmid	1934	3253	+		Methanocaldococcus_jannaschii_DSM_2661_uid	NC_001732	plasmid large ECE		arCOG03166	9 (-)	R		AAA+ superfamily ATPase fused to HTH and Rec	4E-52	COG1672	Predicted ATPase (AAA+ superfamily)
	10954489	hypothetical protein MJECL05	No	0	YES	plasmid	3265	3453	+		Methanocaldococcus_jannaschii_DSM_2661_uid	NC_001732	plasmid large ECE		arCOG10969	1	S		Uncharacterized conserved protein			
	10954497	hypothetical protein MJECL06	No	0	YES	plasmid	3784	4134	-		Methanocaldococcus_jannaschii_DSM_2661_uid	NC_001732	plasmid large ECE		arCOG10970	3	S		Uncharacterized conserved protein			
===										5	Methanocaldococcus_vulcanius_M7_uid41131	NC_013407	chromosome									
	261402408	hypothetical protein Metvu_0283	No	0			273601	274065	+		Methanocaldococcus_vulcanius_M7_uid41131	NC_013407	chromosome									
	261402409	hypothetical protein Metvu_0284	Yes	0			274116	275024	-		Methanocaldococcus_vulcanius_M7_uid41131	NC_013407	chromosome									
	261402410	hypothetical protein Metvu_0285	Yes	0			275012	275185	-		Methanocaldococcus_vulcanius_M7_uid41131	NC_013407	chromosome									
	261402411	histidine triad (HIT) protein	No	0			275450	275839	+		Methanocaldococcus_vulcanius_M7_uid41131	NC_013407	chromosome		arCOG00419	4 (-)	F	Hit	HIT family hydrolase	7E-41	COG0537	Diadenosine tetraphosphate (Ap4A) hydrol
	261402412	hypothetical protein Metvu_0287	No	2			275929	276150	-		Methanocaldococcus_vulcanius_M7_uid41131	NC_013407	chromosome		arCOG11106	1	S		Uncharacterized conserved protein			
===										5	Methanocaldococcus_vulcanius_M7_uid41131	NC_013407	chromosome									
	261402449	hypothetical protein Metvu_0324	No	3			311661	311939	-		Methanocaldococcus_vulcanius_M7_uid41131	NC_013407	chromosome		arCOG10443	4	S		Uncharacterized conserved protein			
	261402450	hypothetical protein Metvu_0325	No	2			312005	312319	-		Methanocaldococcus_vulcanius_M7_uid41131	NC_013407	chromosome		arCOG09673	5	S		Uncharacterized conserved protein			
	261402451	hypothetical protein Metvu_0326	No	2			312720	313007	-		Methanocaldococcus_vulcanius_M7_uid41131	NC_013407	chromosome		arCOG01996	17 (-)	S	SpoIIM	Uncharacterized membrane protein	0.000002	pfam01944	Integral membrane protein DUF95. Members
	261402452	hypothetical protein Metvu_0327	No	4			313012	313410	-		Methanocaldococcus_vulcanius_M7_uid41131	NC_013407	chromosome	clust_254								
	261402453	hypothetical protein Metvu_0328	No	2			313429	313731	-		Methanocaldococcus_vulcanius_M7_uid41131	NC_013407	chromosome		arCOG09673	5	S		Uncharacterized conserved protein			
===										7	Methanocaldococcus_vulcanius_M7_uid41131	NC_013407	chromosome									
	261402456	hypothetical protein Metvu_0331	No	2			316155	316376	-		Methanocaldococcus_vulcanius_M7_uid41131	NC_013407	chromosome									
	261402457	hypothetical protein Metvu_0332	No	2			316509	316700	-		Methanocaldococcus_vulcanius_M7_uid41131	NC_013407	chromosome									
	261402458	hypothetical protein Metvu_0333	No	3			316789	317160	-		Methanocaldococcus_vulcanius_M7_uid41131	NC_013407	chromosome									
	261402459	ATPase	No	0			317710	318819	-		Methanocaldococcus_vulcanius_M7_uid41131	NC_013407	chromosome		arCOG03407	1 (-)	R		Predicted ATPase (AAA+ superfamily)	3E-44	COG1672	Predicted ATPase (AAA+ superfamily)
	261402460	hypothetical protein Metvu_0335	No	3			319047	319382	-		Methanocaldococcus_vulcanius_M7_uid41131	NC_013407	chromosome		arCOG10305	3	S		Uncharacterized conserved protein			
	261402461	hypothetical protein Metvu_0338	No	1			320189	320680	-		Methanocaldococcus_vulcanius_M7_uid41131	NC_013407	chromosome		arCOG11107	3	S		Uncharacterized conserved protein			
	261402462	hypothetical protein Metvu_0339	No	4			320874	321242	-		Methanocaldococcus_vulcanius_M7_uid41131	NC_013407	chromosome									
===										5	Methanocaldococcus_vulcanius_M7_uid41131	NC_013407	chromosome									
	261402580	hypothetical protein Metvu_0463	No	6			446155	446751	-		Methanocaldococcus_vulcanius_M7_uid41131	NC_013407	chromosome		arCOG10681	1	S		Uncharacterized conserved protein			
	261402581	Radical SAM domain protein	No	0			446723	447568	-		Methanocaldococcus_vulcanius_M7_uid41131	NC_013407	chromosome		arCOG02616	1 (-)	R		Radical SAM superfamily enzyme	1E-19	COG0535	Predicted Fe-S oxidoreductases
	261402582	hypothetical protein Metvu_0465	No	1			447698	447850	-		Methanocaldococcus_vulcanius_M7_uid41131	NC_013407	chromosome		arCOG11177	1	S		Uncharacterized conserved protein			
	261402583	hypothetical protein Metvu_0466	No	1			447917	448075	-		Methanocaldococcus_vulcanius_M7_uid41131	NC_013407	chromosome		arCOG11308	1	S		Uncharacterized conserved protein			
	261402584	hypothetical protein Metvu_0467	No	4			448646	449119	+		Methanocaldococcus_vulcanius_M7_uid41131	NC_013407	chromosome									
===										6	Methanocaldococcus_vulcanius_M7_uid41131	NC_013407	chromosome									
	261402675	50S ribosomal protein L15e	No	0		Virus_Caudovi	527067	527651	-		Methanocaldococcus_vulcanius_M7_uid41131	NC_013407	chromosome		arCOG04209	3 (-)	J	RPL15A	Ribosomal protein L15E	2E-95	COG1632	Ribosomal protein L15E
	261402676	hypothetical protein Metvu_0559	No	0		Virus_Caudovi	528109	528447	-		Methanocaldococcus_vulcanius_M7_uid41131	NC_013407	chromosome		arCOG09509	3	S		Uncharacterized conserved protein			
	261402677	hypothetical protein Metvu_0560	No	1		Virus_Caudovi	528449	528703	-		Methanocaldococcus_vulcanius_M7_uid41131	NC_013407	chromosome									
	261402678	hypothetical protein Metvu_0561	No	0		Virus_Caudovi	528939	529379	+		Methanocaldococcus_vulcanius_M7_uid41131	NC_013407	chromosome									
	261402679	hypothetical protein Metvu_0562	No	0		Virus_Caudovi	529385	530698	+		Methanocaldococcus_vulcanius_M7_uid41131	NC_013407	chromosome	clust_78	arCOG09550	17	Y	TerL	Phage terminase large subunit			
	261402680	phage head morphogenesis protein, SPP1 gp	No	0		Virus_Caudovi	530709	532712	+		Methanocaldococcus_vulcanius_M7_uid41131	NC_013407	chromosome		arCOG09510	5 (-)	T		NAD+--asparagine ADP-ribosyltransferase	0.0000003	pfam04233	Phage Mu protein F like protein. Members ; Chromosome segregation ATPases
===										24	Methanocaldococcus_vulcanius_M7_uid41131	NC_013407	chromosome									
	261402683	hypothetical protein Metvu_0566	No	0		Virus_Caudovi	535108	535695	-		Methanocaldococcus_vulcanius_M7_uid41131	NC_013407	chromosome		arCOG09650	4	Y		Predicted phage protein			
	261402684	hypothetical protein Metvu_0567	No	0		Virus_Caudovi	535695	536723	-		Methanocaldococcus_vulcanius_M7_uid41131	NC_013407	chromosome									
	261402685	hypothetical protein Metvu_0568	No	0		Virus_Caudovi	536728	537924	-		Methanocaldococcus_vulcanius_M7_uid41131	NC_013407	chromosome		arCOG03511	3 (-)	S		Uncharacterized conserved protein	2E-19	pfam10102	Domain of unknown function (DUF2341). Me ; Conserved hypothetical protein 2217 (DUF
	261402686	hypothetical protein Metvu_0569	No	0		Virus_Caudovi	537929	538405	-		Methanocaldococcus_vulcanius_M7_uid41131	NC_013407	chromosome									
	261402687	hypothetical protein Metvu_0570	No	0		Virus_Caudovi	538418	538534	-		Methanocaldococcus_vulcanius_M7_uid41131	NC_013407	chromosome									
	261402688	phage tail tape measure protein, TP901 family	No	5		Virus_Caudovi	538540	540732	-		Methanocaldococcus_vulcanius_M7_uid41131	NC_013407	chromosome		arCOG11109	8	Y		Phage tail tape measure protein, TP901 family	1E-35	pfam10145	Phage-related minor tail protein. Member
	261402689	hypothetical protein Metvu_0572	No	0		Virus_Caudovi	540853	541059	-		Methanocaldococcus_vulcanius_M7_uid41131	NC_013407	chromosome	clust_429								
	261402690	hypothetical protein Metvu_0573	No	0		Virus_Caudovi	541064	541432	-		Methanocaldococcus_vulcanius_M7_uid41131	NC_013407	chromosome	clust_285								
	261402691	hypothetical protein Metvu_0574	No	0		Virus_Caudovi	541444	542217	-		Methanocaldococcus_vulcanius_M7_uid41131	NC_013407	chromosome	clust_126								
	261402692	hypothetical protein Metvu_0575	No	0		Virus_Caudovi	542227	542622	-		Methanocaldococcus_vulcanius_M7_uid41131	NC_013407	chromosome	clust_266								
	261402693	hypothetical protein Metvu_0576	No	0		Virus_Caudovi	542633	543436	-		Methanocaldococcus_vulcanius_M7_uid41131	NC_013407	chromosome		arCOG09506	2	S		Uncharacterized conserved protein			
	261402694	hypothetical protein Metvu_0577	No	0		Virus_Caudovi	543447	543830	-		Methanocaldococcus_vulcanius_M7_uid41131	NC_013407	chromosome		arCOG11044	2	S		Uncharacterized conserved protein			
	261402695	hypothetical protein Metvu_0578	No	0		Virus_Caudovi	543841	544281	-		Methanocaldococcus_vulcanius_M7_uid41131	NC_013407	chromosome		arCOG09507	2	S		Uncharacterized conserved protein			
	261402696	hypothetical protein Metvu_0579	No	0		Virus_Caudovi	544253	545071	-		Methanocaldococcus_vulcanius_M7_uid41131	NC_013407	chromosome		arCOG09508	6 (-)	Y		Phage prohead protease			
	261402697	hypothetical protein Metvu_0580	No	0		Virus_Caudovi	545144	545440	-		Methanocaldococcus_vulcanius_M7_uid41131	NC_013407	chromosome		arCOG08301	4	Y		Predicted phage protein			
	261402698	hypothetical protein Metvu_0581	No	0		Virus_Caudovi	545588	546016	-		Methanocaldococcus_vulcanius_M7_uid41131	NC_013407	chromosome		arCOG06629	1 (-)	K		Transcriptional regulator, contains HTH domain			
	261402699	hypothetical protein Metvu_0582	No	0		Virus_Caudovi	545976	546140	-		Methanocaldococcus_vulcanius_M7_uid41131	NC_013407	chromosome									
	261402700	hypothetical protein Metvu_0583	No	0		Virus_Caudovi	546245	546529	-		Methanocaldococcus_vulcanius_M7_uid41131	NC_013407	chromosome									
	261402701	hypothetical protein Metvu_0584	No	0		Virus_Caudovi	546558	547283	-		Methanocaldococcus_vulcanius_M7_uid41131	NC_013407	chromosome		arCOG09665	3	S		Uncharacterized conserved protein			
	261402702	hypothetical protein Metvu_0585	No	0		Virus_Caudovi	547265	547507	-		Methanocaldococcus_vulcanius_M7_uid41131	NC_013407	chromosome		arCOG08302	4	S		Uncharacterized conserved protein			
	261402703	hypothetical protein Metvu_0586	No	0		Virus_Caudovi	547485	547661	-		Methanocaldococcus_vulcanius_M7_uid41131	NC_013407	chromosome		arCOG09666	3	S		Uncharacterized conserved protein			
	261402704	Transcription factor CBF/NF-Y/histone domain [	No	0		Virus_Caudovi	547733	548017	-		Methanocaldococcus_vulcanius_M7_uid41131	NC_013407	chromosome		arCOG02144	3 (-)	L	HHT1	Histones H3 and H4	0.00001	COG2036	Histones H3 and H4
	261402705	hypothetical protein Metvu_0588	No	0	YES	Virus_Caudovi	548022	548639	-		Methanocaldococcus_vulcanius_M7_uid41131	NC_013407	chromosome									
	261402706	hypothetical protein Metvu_0589	No	0	YES	Virus_Caudovi	548987	549187	+		Methanocaldococcus_vulcanius_M7_uid41131	NC_013407	chromosome									
===										7	Methanocaldococcus_vulcanius_M7_uid41131	NC_013407	chromosome									
	261402708	hypothetical protein Metvu_0591	No	0	YES	Virus_Caudovi	549670	550443	-		Methanocaldococcus_vulcanius_M7_uid41131	NC_013407	chromosome		arCOG06842	2 (-)	S		Uncharacterized conserved protein	4E-17	pfam08861	Domain of unknown function DUF1828. This ; Domain of unknown function DUF1829. This
	261402709	putative Lj965 prophage protein	No	0	YES	Virus_Caudovi	550427	550945	-		Methanocaldococcus_vulcanius_M7_uid41131	NC_013407	chromosome									
	261402710	SCP-like extracellular	No	1	YES	Virus_Caudovi	550991	551497	-		Methanocaldococcus_vulcanius_M7_uid41131	NC_013407	chromosome							0.0001	COG2340	Uncharacterized protein with SCP/PR1 dom
	261402711	hypothetical protein Metvu_0594	No	0	YES	Virus_Caudovi	551589	551933	-		Methanocaldococcus_vulcanius_M7_uid41131	NC_013407	chromosome									
	261402712	hypothetical protein Metvu_0595	No	0	YES	Virus_Caudovi	551946	552188	-		Methanocaldococcus_vulcanius_M7_uid41131	NC_013407	chromosome									
	261402713	ArsR family transcriptional regulator	No	0		Virus_Caudovi	552204	552539	-		Methanocaldococcus_vulcanius_M7_uid41131	NC_013407	chromosome		arCOG01057	21 (-)	K		Predicted transcriptional regulator			
	261402714	hypothetical protein Metvu_0597	No	0		Virus_Caudovi	552725	553144	+		Methanocaldococcus_vulcanius_M7_uid41131	NC_013407	chromosome									
===										10	Methanocaldococcus_vulcanius_M7_uid41131	NC_013407	chromosome									
	261402722	branched-chain amino acid aminotransferase	No	0			560967	561833	-		Methanocaldococcus_vulcanius_M7_uid41131	NC_013407	chromosome		arCOG02297	2 (-)	E	IlvE	Branched-chain amino acid aminotransferase/4-a	1E-68	COG0115	Branched-chain amino acid aminotransfera
	261402723	hypothetical protein Metvu_0606	No	0			561940	562083	-		Methanocaldococcus_vulcanius_M7_uid41131	NC_013407	chromosome									
	261402724	hypothetical protein Metvu_0607	No	3			562080	562454	-		Methanocaldococcus_vulcanius_M7_uid41131	NC_013407	chromosome		arCOG10682	5	S		Uncharacterized conserved protein	2E-11	COG4818	Predicted membrane protein
	261402725	hypothetical protein Metvu_0608	No	4			562464	562910	-		Methanocaldococcus_vulcanius_M7_uid41131	NC_013407	chromosome		arCOG10682	5	S		Uncharacterized conserved protein			
	261402726	hypothetical protein Metvu_0609	No	1			562941	563933	-		Methanocaldococcus_vulcanius_M7_uid41131	NC_013407	chromosome		arCOG07116	1	S		Uncharacterized conserved protein			
	261402727	hypothetical protein Metvu_0610	No	3			563930	564316	-		Methanocaldococcus_vulcanius_M7_uid41131	NC_013407	chromosome		arCOG10682	5	S		Uncharacterized conserved protein	0.000000007	COG4818	Predicted membrane protein
	261402728	hypothetical protein Metvu_0611	No	4			564343	564780	-		Methanocaldococcus_vulcanius_M7_uid41131	NC_013407	chromosome	clust_240								
	261402729	hypothetical protein Metvu_0612	No	4			565084	565473	-		Methanocaldococcus_vulcanius_M7_uid41131	NC_013407	chromosome									
	261402730	hypothetical protein Metvu_0613	Yes	1			565519	566397	-		Methanocaldococcus_vulcanius_M7_uid41131	NC_013407	chromosome		arCOG10331	3	S		Uncharacterized conserved protein			
	261402731	Dinitrogenase iron-molybdenum cofactor biosy	No	0			566687	566983	-		Methanocaldococcus_vulcanius_M7_uid41131	NC_013407	chromosome		arCOG02734	5 (-)	S		NifX family protein	0.00000002	COG1433	Uncharacterized conserved protein
===										9	Methanocaldococcus_vulcanius_M7_uid41131	NC_013407	chromosome									
	261402802	hypothetical protein Metvu_0686	No	0			649172	649900	+		Methanocaldococcus_vulcanius_M7_uid41131	NC_013407	chromosome		arCOG09664	1	S		Uncharacterized conserved protein			
	261402803	hypothetical protein Metvu_0687	Yes	1			650226	650951	+		Methanocaldococcus_vulcanius_M7_uid41131	NC_013407	chromosome		arCOG07105	1 (-)	S		Uncharacterized conserved protein			
	261402804	hypothetical protein Metvu_0688	No	4			651006	651443	+		Methanocaldococcus_vulcanius_M7_uid41131	NC_013407	chromosome	clust_240								
	261402805	hypothetical protein Metvu_0689	No	5			651468	651965	+		Methanocaldococcus_vulcanius_M7_uid41131	NC_013407	chromosome									
	261402806	hypothetical protein Metvu_0690	No	4			652100	652549	+		Methanocaldococcus_vulcanius_M7_uid41131	NC_013407	chromosome	clust_230								
	261402807	hypothetical protein Metvu_0691	No	5			652554	653036	+		Methanocaldococcus_vulcanius_M7_uid41131	NC_013407	chromosome	clust_214								
	261402808	hypothetical protein Metvu_0692	No	1			653301	653519	+		Methanocaldococcus_vulcanius_M7_uid41131	NC_013407	chromosome									
	261402809	hypothetical protein Metvu_0693	No	3			653524	653898	+		Methanocaldococcus_vulcanius_M7_uid41131	NC_013407	chromosome		arCOG10682	5	S		Uncharacterized conserved protein	2E-10	COG4818	Predicted membrane protein
	261402810	2-oxoglutarate ferredoxin oxidoreductase subu	No	0			654038	655138	-		Methanocaldococcus_vulcanius_M7_uid41131	NC_013407	chromosome		arCOG01607	1 (-)	C	PorA	Pyruvate:ferredoxin oxidoreductase or related 2-(	4E-61	COG0674	Pyruvate:ferredoxin oxidoreductase and r
===										7	Methanocaldococcus_vulcanius_M7_uid41131	NC_013407	chromosome									
	261402887	translation initiation factor IF-2B subunit delta	No	0			741775	742689	-		Methanocaldococcus_vulcanius_M7_uid41131	NC_013407	chromosome		arCOG01124	1 (-)	J	GCD2	Translation initiation factor 2B subunit, eIF-2B alph	2E-92	COG1184	Translation initiation factor 2B subunit
	261402888	hypothetical protein Metvu_0772	No	0			743225	743323	+		Methanocaldococcus_vulcanius_M7_uid41131	NC_013407	chromosome									
	261402889	hypothetical protein Metvu_0773	No	0			743298	743543	+		Methanocaldococcus_vulcanius_M7_uid41131	NC_013407	chromosome									
	261402890	hypothetical protein Metvu_0774	No	0			743828	743938	+		Methanocaldococcus_vulcanius_M7_uid41131	NC_013407	chromosome									
	261402891	hypothetical protein Metvu_0775	No	0			744028	744339	+		Methanocaldococcus_vulcanius_M7_uid41131	NC_013407	chromosome									
	261402892	hypothetical protein Metvu_0776	No	0			744696	744788	+		Methanocaldococcus_vulcanius_M7_uid41131	NC_013407	chromosome									
	261402893	intracellular protease, PfpI family	Yes	0			745134	745760	-		Methanocaldococcus_vulcanius_M7_uid41131	NC_013407	chromosome		arCOG00769	2 (-)	R	ThiJ	Putative intracellular protease/amidase	4E-30	pfam01965	DJ-1/PfpI family. The family includes th
===										23	Methanocaldococcus_vulcanius_M7_uid41131	NC_013407	chromosome									
	261403138	hypothetical protein Metvu_1022	No	0			1015526	1016008	+		Methanocaldococcus_vulcanius_M7_uid41131	NC_013407	chromosome		arCOG09630	4	V		Minimal nucleotidyltransferase, toxin-antitoxin system component			
	261403139	hypothetical protein Metvu_1023	No	0			1016009	1016542	+		Methanocaldococcus_vulcanius_M7_uid41131	NC_013407	chromosome		arCOG06576	5 (-)	V		HEPN domain containing protein			
	261403140	hypothetical protein Metvu_1024	No	0			1016545	1016685	+		Methanocaldococcus_vulcanius_M7_uid41131	NC_013407	chromosome									
	261403141	hypothetical protein Metvu_1025	No	0			1016700	1016909	+		Methanocaldococcus_vulcanius_M7_uid41131	NC_013407	chromosome		arCOG09567	3	S		Uncharacterized conserved protein			
	261403142	hypothetical protein Metvu_1026	No	0			1016892	1017407	-		Methanocaldococcus_vulcanius_M7_uid41131	NC_013407	chromosome		arCOG09566	2	S		Uncharacterized conserved protein			
	261403143	hypothetical protein Metvu_1027	No	1			1017427	1017618	-		Methanocaldococcus_vulcanius_M7_uid41131	NC_013407	chromosome									
	261403144	hypothetical protein Metvu_1028	No	0			1017887	1018555	-		Methanocaldococcus_vulcanius_M7_uid41131	NC_013407	chromosome		arCOG00280	21 (-)	L		HerA helicase	0.0001	COG0433	HerA helicase
	261403145	hypothetical protein Metvu_1029	No	0			1018565	1018843	-		Methanocaldococcus_vulcanius_M7_uid41131	NC_013407	chromosome									
	261403146	hypothetical protein Metvu_1030	No	0			1018830	1018982	-		Methanocaldococcus_vulcanius_M7_uid41131	NC_013407	chromosome									
	261403147	hypothetical protein Metvu_1031	No	2			1018994	1019554	-		Methanocaldococcus_vulcanius_M7_uid41131	NC_013407	chromosome									
	261403148	hypothetical protein Metvu_1032	No	1			1019790	1020011	+		Methanocaldococcus_vulcanius_M7_uid41131	NC_013407	chromosome									
	261403149	hypothetical protein Metvu_1033	No	1			1020145	1020276	+		Methanocaldococcus_vulcanius_M7_uid41131	NC_013407	chromosome									
	261403150	hypothetical protein Metvu_1034	No	0			1020286	1021149	+		Methanocaldococcus_vulcanius_M7_uid41131	NC_013407	chromosome		arCOG09562	2	S		Uncharacterized conserved protein			
	261403151	hypothetical protein Metvu_1035	No	2			1021150	1021350	+		Methanocaldococcus_vulcanius_M7_uid41131	NC_013407	chromosome									
	261403152	hypothetical protein Metvu_1036	No	1			1021347	1021649	+		Methanocaldococcus_vulcanius_M7_uid41131	NC_013407	chromosome									
	261403153	hypothetical protein Metvu_1037	No	1			1021663	1022625	+		Methanocaldococcus_vulcanius_M7_uid41131	NC_013407	chromosome							0.00004	COG0419	ATPase involved in DNA repair
	261403154	hypothetical protein Metvu_1038	No	1			1022630	1022815	+		Methanocaldococcus_vulcanius_M7_uid41131	NC_013407	chromosome									
	261403155	hypothetical protein Metvu_1039	No	0			1022787	1023038	+		Methanocaldococcus_vulcanius_M7_uid41131	NC_013407	chromosome									
	261403156	hypothetical protein Metvu_1040	No	0			1023025	1023459	+		Methanocaldococcus_vulcanius_M7_uid41131	NC_013407	chromosome									
	261403157	hypothetical protein Metvu_1041	No	0			1023469	1023810	+		Methanocaldococcus_vulcanius_M7_uid41131	NC_013407	chromosome		arCOG09563	2	S		Uncharacterized conserved protein			
	261403158	hypothetical protein Metvu_1042	No	0			1023807	1025312	+		Methanocaldococcus_vulcanius_M7_uid41131	NC_013407	chromosome		arCOG09564	2	S		Uncharacterized conserved protein			
	261403159	hypothetical protein Metvu_1043	No	0			1025322	1025852	+		Methanocaldococcus_vulcanius_M7_uid41131	NC_013407	chromosome		arCOG09565	2	S		Uncharacterized conserved protein			
	261403160	hypothetical protein Metvu_1044	No	0			1025898	1026914	+		Methanocaldococcus_vulcanius_M7_uid41131	NC_013407	chromosome		arCOG03881	5 (-)	S		Uncharacterized conserved protein	0.0000002	COG0419	ATPase involved in DNA repair
===										12	Methanocaldococcus_vulcanius_M7_uid41131	NC_013408	plasmid pMETVU01									
	261403861	plasmid stabilization system	No	0	YES	plasmid	1120	1404	-		Methanocaldococcus_vulcanius_M7_uid41131	NC_013408	plasmid pMETVU01		arCOG01665	6 (-)	V	RelE	Cytotoxic translational repressor of toxin-antitoxin	3E-14	COG2026	Cytotoxic translational repressor of tox
	261403862	hypothetical protein Metvu_1753	No	0	YES	plasmid	1665	1772	+		Methanocaldococcus_vulcanius_M7_uid41131	NC_013408	plasmid pMETVU01									
	261403863	hypothetical protein Metvu_1754	No	0	YES	plasmid	1769	2095	+		Methanocaldococcus_vulcanius_M7_uid41131	NC_013408	plasmid pMETVU01									
	261403864	hypothetical protein Metvu_1755	No	0	YES	plasmid	2110	2547	+		Methanocaldococcus_vulcanius_M7_uid41131	NC_013408	plasmid pMETVU01									
	261403865	hypothetical protein Metvu_1756	No	0	YES	plasmid	2540	2911	+		Methanocaldococcus_vulcanius_M7_uid41131	NC_013408	plasmid pMETVU01									
	261403866	hypothetical protein Metvu_1757	No	0	YES	plasmid	3238	4005	+		Methanocaldococcus_vulcanius_M7_uid41131	NC_013408	plasmid pMETVU01							0.00007	COG0419	ATPase involved in DNA repair
	261403867	hypothetical protein Metvu_1758	No	0	YES	plasmid	5222	5542	+		Methanocaldococcus_vulcanius_M7_uid41131	NC_013408	plasmid pMETVU01									
	261403868	hypothetical protein Metvu_1759	No	0	YES	plasmid	5509	5670	+		Methanocaldococcus_vulcanius_M7_uid41131	NC_013408	plasmid pMETVU01									
	261403869	hypothetical protein Metvu_1760	No	0	YES	plasmid	5646	7661	+		Methanocaldococcus_vulcanius_M7_uid41131	NC_013408	plasmid pMETVU01									
	261403870	UvrD/REP helicase	No	0	YES	plasmid	7654	9429	+		Methanocaldococcus_vulcanius_M7_uid41131	NC_013408	plasmid pMETVU01		arCOG00797	3 (-)	L	UvrD	Superfamily I DNA and RNA helicase	6E-29	COG0210	Superfamily I DNA and RNA helicases
	261403871	hypothetical protein Metvu_1762	No	0	YES	plasmid	9451	9936	+		Methanocaldococcus_vulcanius_M7_uid41131	NC_013408	plasmid pMETVU01									
	261403872	hypothetical protein Metvu_1763	No	0	YES	plasmid	10162	10671	-		Methanocaldococcus_vulcanius_M7_uid41131	NC_013408	plasmid pMETVU01		arCOG11296	2	S		Uncharacterized conserved protein	0.00000005	COG0419	ATPase involved in DNA repair
===										6	Methanocella_arvoryzae_MRE50_uid61623	NC_009464	chromosome									
	147918699	hypothetical protein LRC34	No	0			18348	18833	-		Methanocella_arvoryzae_MRE50_uid61623	NC_009464	chromosome		arCOG06570	1 (-)	S		Uncharacterized conserved protein			
	147918700	hypothetical protein LRC33	No	0			19033	19302	+		Methanocella_arvoryzae_MRE50_uid61623	NC_009464	chromosome									
	147918701	hypothetical protein LRC32	No	1			19387	19959	+		Methanocella_arvoryzae_MRE50_uid61623	NC_009464	chromosome									
	147918702	hypothetical protein LRC31	No	0			19995	20621	-		Methanocella_arvoryzae_MRE50_uid61623	NC_009464	chromosome									
	147918703	hypothetical protein NRC1	No	0			20761	21582	-		Methanocella_arvoryzae_MRE50_uid61623	NC_009464	chromosome									
	147918704	putative cell division GTPase Z	No	0			22195	23376	+		Methanocella_arvoryzae_MRE50_uid61623	NC_009464	chromosome		arCOG02201	4 (-)	D	FtsZ	Cell division GTPase	5E-119	COG0206	Cell division GTPase
===										6	Methanocella_arvoryzae_MRE50_uid61623	NC_009464	chromosome									
	147918907	SUI1 family translation initiation factor	No	0			235425	235730	+		Methanocella_arvoryzae_MRE50_uid61623	NC_009464	chromosome		arCOG04223	2 (-)	J	SUI1	Translation initiation factor 1 (eIF-1/SUI1)	3E-28	COG0023	Translation initiation factor 1 (eIF-1/S
	147918908	hypothetical protein RRC311	No	0	YES		235838	236452	+		Methanocella_arvoryzae_MRE50_uid61623	NC_009464	chromosome									
	147918909	hypothetical protein RRC310	No	0	YES		236705	236941	+		Methanocella_arvoryzae_MRE50_uid61623	NC_009464	chromosome		arCOG11115	4	S		Uncharacterized conserved protein	0.0000001	pfam00477	Small hydrophilic plant seed protein.
	147918910	hypothetical protein RRC309	No	0	YES		237194	237457	+		Methanocella_arvoryzae_MRE50_uid61623	NC_009464	chromosome		arCOG11115	4	S		Uncharacterized conserved protein	0.0000003	pfam00477	Small hydrophilic plant seed protein.
	147918911	hypothetical protein NRC25	No	1	YES		237638	237850	+		Methanocella_arvoryzae_MRE50_uid61623	NC_009464	chromosome									
	147918912	putative transposase (IS4)	No	0	YES		237875	238996	-		Methanocella_arvoryzae_MRE50_uid61623	NC_009464	chromosome		arCOG03902	8 (-)	L		Transposase			
===										5	Methanocella_arvoryzae_MRE50_uid61623	NC_009464	chromosome									
	147918977	hypothetical protein RRC226	No	0			306679	306957	+		Methanocella_arvoryzae_MRE50_uid61623	NC_009464	chromosome									
	147918978	phenylalanyl-tRNA synthetase subunit beta	No	0			307015	308676	-		Methanocella_arvoryzae_MRE50_uid61623	NC_009464	chromosome		arCOG00412	1 (-)	J	PheT	Phenylalanyl-tRNA synthetase beta subunit	2E-63	COG0072	Phenylalanyl-tRNA synthetase beta subuni
	147918979	hypothetical protein NRC23	No	0			309144	309521	+		Methanocella_arvoryzae_MRE50_uid61623	NC_009464	chromosome									
	147918980	hypothetical protein NRC22	No	1			309868	310254	+		Methanocella_arvoryzae_MRE50_uid61623	NC_009464	chromosome									
	147918981	hypothetical protein RRC222	Yes	1			310405	311292	+		Methanocella_arvoryzae_MRE50_uid61623	NC_009464	chromosome									
===										5	Methanocella_arvoryzae_MRE50_uid61623	NC_009464	chromosome									
	147919040	hypothetical protein RRC143	No	0			383054	383347	-		Methanocella_arvoryzae_MRE50_uid61623	NC_009464	chromosome									
	147919041	hypothetical protein RRC142	No	0			383739	384215	+		Methanocella_arvoryzae_MRE50_uid61623	NC_009464	chromosome		arCOG08236	1	S		Uncharacterized conserved protein, DUF1697	3E-24	pfam08002	Protein of unknown function (DUF1697). T
	147919042	hypothetical protein RRC140	Yes	1			384528	385748	+		Methanocella_arvoryzae_MRE50_uid61623	NC_009464	chromosome									
	147919043	LysE family lysine exporter	Yes	6			385901	386527	+		Methanocella_arvoryzae_MRE50_uid61623	NC_009464	chromosome		arCOG01947	2 (-)	E	RhtB	Putative threonine efflux protein	9E-20	pfam01810	LysE type translocator. This family cons
	147919044	hypothetical protein RRC138	No	0	YES		386576	386956	+		Methanocella_arvoryzae_MRE50_uid61623	NC_009464	chromosome							2E-17	COG3502	Uncharacterized protein conserved in bac
===										5	Methanocella_arvoryzae_MRE50_uid61623	NC_009464	chromosome									
	147919243	hypothetical protein RCIA194	No	0			587563	587796	+		Methanocella_arvoryzae_MRE50_uid61623	NC_009464	chromosome									
	147919244	putative indolepyruvate:ferredoxin oxidoreduct	Yes	0			587823	589421	+		Methanocella_arvoryzae_MRE50_uid61623	NC_009464	chromosome		arCOG01612	1 (-)	C		Indolepyruvate ferredoxin oxidoreductase, alpha	2E-76	COG4231	Indolepyruvate ferredoxin oxidoreductase
	147919245	hypothetical protein RCIA193	No	0			589723	590007	-		Methanocella_arvoryzae_MRE50_uid61623	NC_009464	chromosome									
	147919246	hypothetical protein RCIA192	No	0			590100	590315	-		Methanocella_arvoryzae_MRE50_uid61623	NC_009464	chromosome									
	147919247	hypothetical protein RCIA191	No	0			590378	590587	-		Methanocella_arvoryzae_MRE50_uid61623	NC_009464	chromosome									
===										6	Methanocella_arvoryzae_MRE50_uid61623	NC_009464	chromosome									
	147919272	putative N6 adenine-specific DNA methyltransf	No	0	YES		614110	615033	+		Methanocella_arvoryzae_MRE50_uid61623	NC_009464	chromosome		arCOG00115	10 (-)	L		DNA modification methylase	5E-27	pfam01555	DNA methylase. Members of this family ar
	147919273	hypothetical protein RCIX2616	No	0	YES		615044	615355	-		Methanocella_arvoryzae_MRE50_uid61623	NC_009464	chromosome									
	147919274	hypothetical protein RCIX2615	No	0	YES		615357	615854	-		Methanocella_arvoryzae_MRE50_uid61623	NC_009464	chromosome									
	147919275	hypothetical protein RCIX2613	No	0	YES		616052	616279	-		Methanocella_arvoryzae_MRE50_uid61623	NC_009464	chromosome									
	147919276	hypothetical protein RCIX2611	No	9	YES		616372	617229	-		Methanocella_arvoryzae_MRE50_uid61623	NC_009464	chromosome							0.00001	COG1266	Predicted metal-dependent membrane prote
	147919277	HAD family hydrolase	No	0			617405	618073	+		Methanocella_arvoryzae_MRE50_uid61623	NC_009464	chromosome		arCOG02292	1 (-)	R	Gph	HAD superfamily hydrolase	4E-17	COG1011	Predicted hydrolase (HAD superfamily)
===										5	Methanocella_arvoryzae_MRE50_uid61623	NC_009464	chromosome									
	147919422	hypothetical protein RCIX2427	No	0			734166	734630	+		Methanocella_arvoryzae_MRE50_uid61623	NC_009464	chromosome									
	147919423	hypothetical protein RCIX2426	Yes	2			734830	735522	-		Methanocella_arvoryzae_MRE50_uid61623	NC_009464	chromosome									
	147919424	hypothetical protein RCIX2425	Yes	1			735615	736955	-		Methanocella_arvoryzae_MRE50_uid61623	NC_009464	chromosome		arCOG02165	4 (-)	E		Transglutaminase-like enzyme, putative cysteine protease			
	147919425	hypothetical protein RCIA181	No	0			737307	737579	-		Methanocella_arvoryzae_MRE50_uid61623	NC_009464	chromosome									
	147919426	hypothetical protein RCIA180	No	0			737799	738026	-		Methanocella_arvoryzae_MRE50_uid61623	NC_009464	chromosome									
===										6	Methanocella_arvoryzae_MRE50_uid61623	NC_009464	chromosome									
	147919432	hypothetical protein RCIX2412	No	0			743667	744719	+		Methanocella_arvoryzae_MRE50_uid61623	NC_009464	chromosome		arCOG00560	2 (-)	L	RecQ	Superfamily II DNA helicase	6E-24	COG0514	Superfamily II DNA helicase
	147919433	hypothetical protein RCIA179	No	2			744736	744981	-		Methanocella_arvoryzae_MRE50_uid61623	NC_009464	chromosome									
	147919434	hypothetical protein RCIA178	No	2			745106	745375	-		Methanocella_arvoryzae_MRE50_uid61623	NC_009464	chromosome									
	147919435	hypothetical protein RCIX2410	No	4			745372	745758	-		Methanocella_arvoryzae_MRE50_uid61623	NC_009464	chromosome									
	147919436	hypothetical protein RCIA177	No	0			745827	746099	+		Methanocella_arvoryzae_MRE50_uid61623	NC_009464	chromosome									
	147919437	phosphoglucomutase/phosphomannomutase	No	0			746239	747570	-		Methanocella_arvoryzae_MRE50_uid61623	NC_009464	chromosome		arCOG00767	3 (-)	G	{ManB}	Phosphomannomutase	2E-110	COG1109	Phosphomannomutase
===										6	Methanocella_arvoryzae_MRE50_uid61623	NC_009464	chromosome									
	147919512	hypothetical protein RCIA167	No	0			819582	819860	+		Methanocella_arvoryzae_MRE50_uid61623	NC_009464	chromosome									
	147919513	hypothetical protein RCIX2325	No	7			819944	820708	-		Methanocella_arvoryzae_MRE50_uid61623	NC_009464	chromosome									
	147919514	hypothetical protein RCIX2324	No	0			820751	821377	+		Methanocella_arvoryzae_MRE50_uid61623	NC_009464	chromosome		arCOG03032	12 (-)	R	NrfG	TPR repeats containing protein	5E-16	COG0457	FOG: TPR repeat
	147919515	hypothetical protein RCIX2323	No	4			821539	822180	+		Methanocella_arvoryzae_MRE50_uid61623	NC_009464	chromosome									
	147919516	hypothetical protein RCIX2321	No	3			822268	822585	+		Methanocella_arvoryzae_MRE50_uid61623	NC_009464	chromosome									
	147919517	hypothetical protein RCIX2319	No	0			822675	823031	-		Methanocella_arvoryzae_MRE50_uid61623	NC_009464	chromosome	clust_301								
===										5	Methanocella_arvoryzae_MRE50_uid61623	NC_009464	chromosome									
	147919737	hypothetical protein RCIA153	No	1			1044865	1045188	-		Methanocella_arvoryzae_MRE50_uid61623	NC_009464	chromosome									
	147919738	hypothetical protein RCIX2047	No	3			1045244	1045513	-		Methanocella_arvoryzae_MRE50_uid61623	NC_009464	chromosome									
	147919739	hypothetical protein RCIX2046	Yes	4			1045524	1045901	-		Methanocella_arvoryzae_MRE50_uid61623	NC_009464	chromosome									
	147919740	putative thymidylate kinase	No	0			1045997	1046623	+		Methanocella_arvoryzae_MRE50_uid61623	NC_009464	chromosome		arCOG01891	2 (-)	F	Tmk	Thymidylate kinase	6E-39	COG0125	Thymidylate kinase
	147919741	hypothetical protein RCIX2042	No	5			1046647	1047366	-		Methanocella_arvoryzae_MRE50_uid61623	NC_009464	chromosome									
===										5	Methanocella_arvoryzae_MRE50_uid61623	NC_009464	chromosome									
	147919776	hypothetical protein RCIX1993	Yes	0			1084050	1084418	+		Methanocella_arvoryzae_MRE50_uid61623	NC_009464	chromosome									
	147919777	hypothetical protein RCIX1991	No	1			1084473	1085249	+		Methanocella_arvoryzae_MRE50_uid61623	NC_009464	chromosome									
	147919778	2(4Fe-4S) ferredoxin-domain-containing protein	No	0			1085656	1086486	-		Methanocella_arvoryzae_MRE50_uid61623	NC_009464	chromosome		arCOG02189	1 (-)	C	NapF	HTH containing ranscriptional regulator fused to fe	0.000007	COG1142	Fe-S-cluster-containing hydrogenase comp
	147919779	hypothetical protein RCIX1989	No	0			1086613	1086927	-		Methanocella_arvoryzae_MRE50_uid61623	NC_009464	chromosome									
	147919780	hypothetical protein RCIX1988	No	6			1086931	1087704	-		Methanocella_arvoryzae_MRE50_uid61623	NC_009464	chromosome									
===										7	Methanocella_arvoryzae_MRE50_uid61623	NC_009464	chromosome									
	147920023	hypothetical protein RCIX1665	No	0			1349048	1349941	-		Methanocella_arvoryzae_MRE50_uid61623	NC_009464	chromosome		arCOG03260	1 (-)	S		Uncharacterized conserved protein	0.00002	pfam13620	Carboxypeptidase regulatory-like domain.
	147920024	hypothetical protein RCIX1663	No	0			1349942	1351396	-		Methanocella_arvoryzae_MRE50_uid61623	NC_009464	chromosome									
	147920025	hypothetical protein RCIX1662	No	0			1351393	1351614	-		Methanocella_arvoryzae_MRE50_uid61623	NC_009464	chromosome	clust_428								
	147920026	hypothetical protein RCIX1661	No	0			1351607	1351951	-		Methanocella_arvoryzae_MRE50_uid61623	NC_009464	chromosome	clust_302								
	147920027	hypothetical protein RCIX1660	No	0			1351969	1352364	-		Methanocella_arvoryzae_MRE50_uid61623	NC_009464	chromosome									
	147920028	hypothetical protein RCIX1658	No	0			1352377	1353726	-		Methanocella_arvoryzae_MRE50_uid61623	NC_009464	chromosome	clust_76						1E-20	pfam04984	Phage tail sheath protein. This family i
	147920029	putative chemotaxis signal transducer	No	0			1354003	1355103	-		Methanocella_arvoryzae_MRE50_uid61623	NC_009464	chromosome		arCOG02320	4 (-)	N	Tar	Methyl-accepting chemotaxis protein	0.000000003	pfam00015	Methyl-accepting chemotaxis protein (MCP
===										7	Methanocella_arvoryzae_MRE50_uid61623	NC_009464	chromosome									
	147920185	putative chromosome partitioning protein	No	0	YES		1508895	1509923	-		Methanocella_arvoryzae_MRE50_uid61623	NC_009464	chromosome		arCOG11113	1 (-)	L		ParB-like nuclease domain containing protein	0.00000002	pfam02195	ParB-like nuclease domain.
	147920186	hypothetical protein RCIA111	No	0	YES		1510469	1511095	+		Methanocella_arvoryzae_MRE50_uid61623	NC_009464	chromosome									
	147920187	hypothetical protein RCIX1456	Yes	1	YES		1511388	1511894	-		Methanocella_arvoryzae_MRE50_uid61623	NC_009464	chromosome							0.0000002	pfam07174	Fibronectin-attachment protein (FAP). Th
	147920188	hypothetical protein RCIA110	No	0	YES		1512247	1512492	-		Methanocella_arvoryzae_MRE50_uid61623	NC_009464	chromosome									
	147920189	hypothetical protein RCIX1454	No	3	YES		1512764	1513153	+		Methanocella_arvoryzae_MRE50_uid61623	NC_009464	chromosome									
	147920190	hypothetical protein RCIX1453	No	2	YES		1513172	1513438	+		Methanocella_arvoryzae_MRE50_uid61623	NC_009464	chromosome									
	147920191	putative membrane-bound metal-dependent hy	No	2	YES		1513787	1514353	+		Methanocella_arvoryzae_MRE50_uid61623	NC_009464	chromosome		arCOG03391	1 (-)	R		Predicted membrane-bound metal-dependent hydrolase			
===										6	Methanocella_arvoryzae_MRE50_uid61623	NC_009464	chromosome									
	147920272	Fe(II) import system, GTP-binding protein B	No	8			1593820	1595727	+		Methanocella_arvoryzae_MRE50_uid61623	NC_009464	chromosome		arCOG00359	2 (-)	P	FeoB	Fe2+ transport system protein B	4E-134	COG0370	Fe2+ transport system protein B
	147920273	hypothetical protein RCIX1345	Yes	2			1595869	1596894	+		Methanocella_arvoryzae_MRE50_uid61623	NC_009464	chromosome		arCOG11015	1	S		Uncharacterized conserved protein			
	147920274	hypothetical protein RCIX1343	Yes	2			1597072	1597968	+		Methanocella_arvoryzae_MRE50_uid61623	NC_009464	chromosome							0.000004	pfam01456	Mucin-like glycoprotein. This family of
	147920275	hypothetical protein RCIX1342	Yes	2			1598130	1598963	+		Methanocella_arvoryzae_MRE50_uid61623	NC_009464	chromosome							0.000003	pfam05466	Brain acid soluble protein 1 (BASP1 prot
	147920276	hypothetical protein RCIX1341	Yes	2			1598976	1599509	+		Methanocella_arvoryzae_MRE50_uid61623	NC_009464	chromosome									
	147920277	putative metallo-beta-lactamase	No	0			1599512	1600255	-		Methanocella_arvoryzae_MRE50_uid61623	NC_009464	chromosome		arCOG00500	2 (-)	R	ElaC	Metal-dependent hydrolase of the beta-lactamase	7E-16	COG1234	Metal-dependent hydrolases of the beta-l
===										13	Methanocella_arvoryzae_MRE50_uid61623	NC_009464	chromosome									
	147920373	hypothetical protein RCIX1224	No	0			1699867	1700451	+		Methanocella_arvoryzae_MRE50_uid61623	NC_009464	chromosome		arCOG11016	4	S		Uncharacterized conserved protein			
	147920374	exodeoxyribonuclease III	No	0			1700948	1701721	+		Methanocella_arvoryzae_MRE50_uid61623	NC_009464	chromosome		arCOG02207	2 (-)	L	XthA	Exonuclease III	7E-81	COG0708	Exonuclease III
	147920375	hypothetical protein RCIX1221	Yes	1			1701873	1702526	+		Methanocella_arvoryzae_MRE50_uid61623	NC_009464	chromosome		arCOG11016	4	S		Uncharacterized conserved protein	0.0001	pfam14371	Domain of unknown function (DUF4412). Th
	147920376	hypothetical protein RCIX1220	Yes	1			1702710	1703450	+		Methanocella_arvoryzae_MRE50_uid61623	NC_009464	chromosome		arCOG11016	4	S		Uncharacterized conserved protein			
	147920377	hypothetical protein RCIX1218	No	0			1703609	1704028	+		Methanocella_arvoryzae_MRE50_uid61623	NC_009464	chromosome									
	147920378	hypothetical protein RCIX1217	No	0			1704031	1704369	+		Methanocella_arvoryzae_MRE50_uid61623	NC_009464	chromosome		arCOG11016	4	S		Uncharacterized conserved protein			
	147920379	hypothetical protein RCIX1216	Yes	0			1704540	1705874	+		Methanocella_arvoryzae_MRE50_uid61623	NC_009464	chromosome									
	147920380	hypothetical protein RCIA86	No	0			1705880	1706245	-		Methanocella_arvoryzae_MRE50_uid61623	NC_009464	chromosome									
	147920381	hypothetical protein RCIA84	No	0			1707020	1707472	-		Methanocella_arvoryzae_MRE50_uid61623	NC_009464	chromosome									
	147920382	hypothetical protein RCIX1213	No	1			1707506	1707766	+		Methanocella_arvoryzae_MRE50_uid61623	NC_009464	chromosome									
	147920383	hypothetical protein RCIX1212	No	5			1707763	1708500	-		Methanocella_arvoryzae_MRE50_uid61623	NC_009464	chromosome									
	147920384	hypothetical protein RCIX1211	No	0			1708547	1708756	+		Methanocella_arvoryzae_MRE50_uid61623	NC_009464	chromosome									
	147920385	putative ATPase RIL	No	0			1708884	1710656	-		Methanocella_arvoryzae_MRE50_uid61623	NC_009464	chromosome		arCOG00187	1 (-)	J	ABCE1	Fe-S cluster containing ABC ATPase involved in ribosome recy		COG1245	Predicted ATPase, RNase L inhibitor (RLI
===										5	Methanocella_arvoryzae_MRE50_uid61623	NC_009464	chromosome									
	147920447	hypothetical protein RCIX1127	Yes	2			1783774	1784574	-		Methanocella_arvoryzae_MRE50_uid61623	NC_009464	chromosome									
	147920448	thymidine phosphorylase	No	0			1784865	1786376	+		Methanocella_arvoryzae_MRE50_uid61623	NC_009464	chromosome		arCOG02013	1 (-)	F	DeoA	Thymidine phosphorylase	1E-88	COG0213	Thymidine phosphorylase
	147920449	hypothetical protein RCIX1125	No	0			1787141	1787485	-		Methanocella_arvoryzae_MRE50_uid61623	NC_009464	chromosome							0.000007	pfam14446	Prokaryotic RING finger family 1. RING f
	147920450	hypothetical protein RCIX1124	No	0			1787641	1787937	+		Methanocella_arvoryzae_MRE50_uid61623	NC_009464	chromosome									
	147920451	hypothetical protein RCIX1123	No	0			1788013	1788459	-		Methanocella_arvoryzae_MRE50_uid61623	NC_009464	chromosome									
===										7	Methanocella_arvoryzae_MRE50_uid61623	NC_009464	chromosome									
	147920552	TetR family transcriptional regulator	No	0			1885561	1886217	+		Methanocella_arvoryzae_MRE50_uid61623	NC_009464	chromosome		arCOG02644	1 (-)	K	AcrR	Transcriptional regulator, TetR/AcrR family	3E-15	COG1309	Transcriptional regulator
	147920553	hypothetical protein RCIA56	No	0			1887598	1887963	-		Methanocella_arvoryzae_MRE50_uid61623	NC_009464	chromosome									
	147920554	hypothetical protein RCIA55	No	0			1888202	1888441	-		Methanocella_arvoryzae_MRE50_uid61623	NC_009464	chromosome									
	147920555	hypothetical protein RCIX986	Yes	0			1888534	1889217	-		Methanocella_arvoryzae_MRE50_uid61623	NC_009464	chromosome		arCOG10895	5	S		Uncharacterized conserved protein			
	147920556	hypothetical protein RCIX985	No	1			1889241	1889999	-		Methanocella_arvoryzae_MRE50_uid61623	NC_009464	chromosome		arCOG10895	5	S		Uncharacterized conserved protein			
	147920557	GNAT family acetyltransferase	No	0			1890027	1890473	+		Methanocella_arvoryzae_MRE50_uid61623	NC_009464	chromosome		arCOG00826	1 (-)	K	WecD	Acetyltransferase (GNAT) family	0.000007	pfam00583	Acetyltransferase (GNAT) family. This fa
	147920558	hypothetical protein RCIX983	No	3			1890522	1890929	+		Methanocella_arvoryzae_MRE50_uid61623	NC_009464	chromosome									
===										6	Methanocella_arvoryzae_MRE50_uid61623	NC_009464	chromosome									
	147920808	putative molybdopterin biosynthesis protein M	No	0			2148600	2150528	+		Methanocella_arvoryzae_MRE50_uid61623	NC_009464	chromosome		arCOG00217	1 (-)	H	MoeA	Molybdopterin biosynthesis enzyme	2E-110	COG0303	Molybdopterin biosynthesis enzyme
	147920809	hypothetical protein RCIX660	Yes	2			2150955	2152238	+		Methanocella_arvoryzae_MRE50_uid61623	NC_009464	chromosome							2E-19	pfam05109	Herpes virus major outer envelope glycop
	147920810	hypothetical protein RCIX659	No	0			2152950	2155748	+		Methanocella_arvoryzae_MRE50_uid61623	NC_009464	chromosome							0.000000001	COG5295	Autotransporter adhesin
	147920811	hypothetical protein RCIX658	Yes	2			2155745	2156545	+		Methanocella_arvoryzae_MRE50_uid61623	NC_009464	chromosome							0.00000003	pfam01456	Mucin-like glycoprotein. This family of
	147920812	hypothetical protein RCIA29	No	4			2156588	2157166	-		Methanocella_arvoryzae_MRE50_uid61623	NC_009464	chromosome		arCOG10915	1	S		Uncharacterized conserved protein			
	147920813	chemotaxis signal transducer	No	0			2157411	2157890	+		Methanocella_arvoryzae_MRE50_uid61623	NC_009464	chromosome		arCOG02395	1 (-)	N	CheW	Chemotaxis signal transduction protein	3E-35	COG0835	Chemotaxis signal transduction protein [
===										5	Methanocella_arvoryzae_MRE50_uid61623	NC_009464	chromosome									
	147920996	hypothetical protein RCIX433	No	0			2349470	2349934	-		Methanocella_arvoryzae_MRE50_uid61623	NC_009464	chromosome		arCOG10895	5	S		Uncharacterized conserved protein			
	147920997	hypothetical protein RCIX431	No	0			2350096	2351193	-		Methanocella_arvoryzae_MRE50_uid61623	NC_009464	chromosome									
	147920998	hypothetical protein RCIX429	No	0			2351589	2351810	+		Methanocella_arvoryzae_MRE50_uid61623	NC_009464	chromosome		arCOG11019	2	S		Uncharacterized conserved protein			
	147920999	putative pyridoxamine 5\'-phosphate-related pr	No	0			2351860	2352342	+		Methanocella_arvoryzae_MRE50_uid61623	NC_009464	chromosome		arCOG00520	1 (-)	R		Predicted flavin-nucleotide-binding protein	2E-20	pfam12900	Pyridoxamine 5'-phosphate oxidase. Pyrid
	147921000	hypothetical protein RCIX426	No	1			2352522	2353067	+		Methanocella_arvoryzae_MRE50_uid61623	NC_009464	chromosome		arCOG11019	2	S		Uncharacterized conserved protein			
===										5	Methanocella_arvoryzae_MRE50_uid61623	NC_009464	chromosome									
	147921137	hypothetical protein RCIX256	Yes	0			2496074	2498599	-		Methanocella_arvoryzae_MRE50_uid61623	NC_009464	chromosome									
	147921138	hypothetical protein RCIX255	No	14			2498668	2500509	-		Methanocella_arvoryzae_MRE50_uid61623	NC_009464	chromosome		arCOG03188	2 (-)	S		Uncharacterized conserved protein	0.0000003	COG4906	Predicted membrane protein
	147921139	hypothetical protein RCIX254	No	3			2500513	2500857	-		Methanocella_arvoryzae_MRE50_uid61623	NC_009464	chromosome									
	147921140	hypothetical protein RCIX253	No	0			2500923	2501726	-		Methanocella_arvoryzae_MRE50_uid61623	NC_009464	chromosome									
	147921141	hypothetical protein RCIX252	No	0			2502092	2502307	-		Methanocella_arvoryzae_MRE50_uid61623	NC_009464	chromosome									
===										5	Methanocella_arvoryzae_MRE50_uid61623	NC_009464	chromosome									
	147921155	hypothetical protein RCIX235	No	2			2516400	2516615	+		Methanocella_arvoryzae_MRE50_uid61623	NC_009464	chromosome									
	147921156	hypothetical protein NRC15	No	0			2516862	2517113	+		Methanocella_arvoryzae_MRE50_uid61623	NC_009464	chromosome									
	147921157	hypothetical protein RCIX233	No	0			2517366	2518382	+		Methanocella_arvoryzae_MRE50_uid61623	NC_009464	chromosome	clust_92						4E-58	pfam14399	A predicted NlpC/p60-like peptidase. Mem
	147921158	ATP-dependent RNA helicase	No	0			2518754	2521567	+		Methanocella_arvoryzae_MRE50_uid61623	NC_009464	chromosome		arCOG00557	3 (-)	R	Lhr	Lhr-like helicase	1E-175	COG1201	Lhr-like helicases
	147921159	hypothetical protein RCIX230	No	3			2521698	2522051	+		Methanocella_arvoryzae_MRE50_uid61623	NC_009464	chromosome									
===										5	Methanocella_arvoryzae_MRE50_uid61623	NC_009464	chromosome									

147921337	hypothetical protein RCIX1	Yes	1		2712913	2713185	-		Methanocella_arvoryzae_MRE50_uid61623	NC_009464	chromosome									
147921338	hypothetical protein NRC13	Yes	1		2713798	2714490	-		Methanocella_arvoryzae_MRE50_uid61623	NC_009464	chromosome							0.0000005	pfam07174	Fibronectin-attachment protein (FAP). Th
147921339	hypothetical protein LRC597	No	0		2715004	2715267	+		Methanocella_arvoryzae_MRE50_uid61623	NC_009464	chromosome									
147921340	signal transduction histidine kinase	No	0		2715346	2716854	-		Methanocella_arvoryzae_MRE50_uid61623	NC_009464	chromosome	arCOG02352,a	arCOG02352	1 (-)	T		Signal transduction histidine kinase with PAS and	5E-19	COG2202	FOG: PAS/PAC domain
147921341	hypothetical protein LRC593	Yes	1		2717136	2717894	+		Methanocella_arvoryzae_MRE50_uid61623	NC_009464	chromosome							5E-11	pfam06346	Formin Homology Region 1. This region is
===								9	Methanocella_arvoryzae_MRE50_uid61623	NC_009464	chromosome									
147921418	hypothetical protein LRC496	No	1		2807982	2808977	+		Methanocella_arvoryzae_MRE50_uid61623	NC_009464	chromosome									
147921419	hypothetical protein LRC495	No	0		2809297	2809545	+		Methanocella_arvoryzae_MRE50_uid61623	NC_009464	chromosome									
147921420	hypothetical protein LRC494	No	0		2809638	2810093	-		Methanocella_arvoryzae_MRE50_uid61623	NC_009464	chromosome		arCOG01075	2 (-)	F		NUDIX family hydrolase	2E-10	COG1051	ADP-ribose pyrophosphatase
147921421	hypothetical protein LRC493	No	4		2810405	2810935	+		Methanocella_arvoryzae_MRE50_uid61623	NC_009464	chromosome									
147921422	hypothetical protein LRC492	Yes	2		2811132	2811521	-		Methanocella_arvoryzae_MRE50_uid61623	NC_009464	chromosome		arCOG11120	4	S		Uncharacterized conserved protein			
147921423	hypothetical protein LRC491	Yes	1		2811700	2812569	-		Methanocella_arvoryzae_MRE50_uid61623	NC_009464	chromosome		arCOG10916	6	S		Uncharacterized conserved protein			
147921424	hypothetical protein LRC490	Yes	1		2812910	2813782	-		Methanocella_arvoryzae_MRE50_uid61623	NC_009464	chromosome		arCOG10916	6	S		Uncharacterized conserved protein			
147921425	hypothetical protein LRC488	Yes	2		2814412	2814972	+		Methanocella_arvoryzae_MRE50_uid61623	NC_009464	chromosome		arCOG11011	1	S		Uncharacterized conserved protein			
147921426	hypothetical protein LRC487	No	1		2815009	2817066	-		Methanocella_arvoryzae_MRE50_uid61623	NC_009464	chromosome		arCOG02966	7 (-)	S		HEAT repeats containing protein	1E-20	COG1413	FOG: HEAT repeat
===								10	Methanocella_arvoryzae_MRE50_uid61623	NC_009464	chromosome									
147921480	30S ribosomal protein S6e	No	0		2872877	2873305	+		Methanocella_arvoryzae_MRE50_uid61623	NC_009464	chromosome		arCOG01946	4 (-)	J	RPS6A	Ribosomal protein S6E (S10)	6E-42	COG2125	Ribosomal protein S6E (S10)
147921481	hypothetical protein LRC419	No	0		2873357	2873662	-		Methanocella_arvoryzae_MRE50_uid61623	NC_009464	chromosome	clust_47								
147921482	hypothetical protein LRC418	Yes	1		2874011	2874406	+		Methanocella_arvoryzae_MRE50_uid61623	NC_009464	chromosome		arCOG10873	14	S		Uncharacterized conserved protein			
147921483	hypothetical protein LRC417	Yes	1		2874731	2875126	+		Methanocella_arvoryzae_MRE50_uid61623	NC_009464	chromosome		arCOG10873	14	S		Uncharacterized conserved protein			
147921484	hypothetical protein LRC416	No	1		2875300	2875740	+		Methanocella_arvoryzae_MRE50_uid61623	NC_009464	chromosome		arCOG11121	7	S		Uncharacterized conserved protein			
147921485	hypothetical protein LRC414	Yes	8		2876010	2876810	+		Methanocella_arvoryzae_MRE50_uid61623	NC_009464	chromosome	clust_29								
147921486	hypothetical protein LRC412	No	3		2877554	2878138	+		Methanocella_arvoryzae_MRE50_uid61623	NC_009464	chromosome		arCOG11122	4	S		Uncharacterized conserved protein			
147921487	hypothetical protein LRC410	Yes	1		2878554	2879306	+		Methanocella_arvoryzae_MRE50_uid61623	NC_009464	chromosome		arCOG11111	2	S		Uncharacterized conserved protein			
147921488	hypothetical protein LRC409	No	0		2879561	2880277	+		Methanocella_arvoryzae_MRE50_uid61623	NC_009464	chromosome									
147921489	putative small-conductance mechanosensitive	No	3		2880301	2881107	+		Methanocella_arvoryzae_MRE50_uid61623	NC_009464	chromosome		arCOG01568	4 (-)	M	MscS	Small-conductance mechanosensitive channel	4E-24	COG0668	Small-conductance mechanosensitive chann
===								5	Methanocella_arvoryzae_MRE50_uid61623	NC_009464	chromosome									
147921692	hypothetical protein LRC153	No	11		3095901	3097451	-		Methanocella_arvoryzae_MRE50_uid61623	NC_009464	chromosome		arCOG11018	1	S		Uncharacterized conserved protein			
147921693	hypothetical protein LRC151	Yes	0		3097789	3098361	+		Methanocella_arvoryzae_MRE50_uid61623	NC_009464	chromosome		arCOG02510	1 (-)	S		PKD repeats containing protein	3E-17	COG3291	FOG: PKD repeat
147921694	hypothetical protein LRC150	No	2		3098405	3098749	-		Methanocella_arvoryzae_MRE50_uid61623	NC_009464	chromosome		arCOG11022	1	S		Uncharacterized conserved protein			
147921695	hypothetical protein LRC149	Yes	1		3098977	3099642	+		Methanocella_arvoryzae_MRE50_uid61623	NC_009464	chromosome									
147921696	hypothetical protein NRC5	No	2		3100040	3100267	+		Methanocella_arvoryzae_MRE50_uid61623	NC_009464	chromosome									
===								5	Methanocella_arvoryzae_MRE50_uid61623	NC_009464	chromosome									
147921713	hypothetical protein LRC122	Yes	0		3117510	3118073	+		Methanocella_arvoryzae_MRE50_uid61623	NC_009464	chromosome									
147921714	hypothetical protein LRC121	No	0		3118080	3118367	+		Methanocella_arvoryzae_MRE50_uid61623	NC_009464	chromosome									
147921715	signal transduction histidine kinase	No	2		3118435	3120603	-		Methanocella_arvoryzae_MRE50_uid61623	NC_009464	chromosome		arCOG02342	1 (-)	T		Signal transduction histidine kinase, contains Cac	4E-44	COG3920	Signal transduction histidine kinase
147921716	hypothetical protein LRC119	Yes	2		3120814	3121269	-		Methanocella_arvoryzae_MRE50_uid61623	NC_009464	chromosome		arCOG11126	2	S		Uncharacterized conserved protein			
147921717	hypothetical protein LRC118	Yes	1		3121460	3121822	-		Methanocella_arvoryzae_MRE50_uid61623	NC_009464	chromosome		arCOG11110	3	S		Uncharacterized conserved protein			
===								8	Methanocella_arvoryzae_MRE50_uid61623	NC_009464	chromosome									
147921737	metallo-beta-lactamase	No	0		3143850	3145400	-		Methanocella_arvoryzae_MRE50_uid61623	NC_009464	chromosome		arCOG00547	1 (-)	J		mRNA degradation ribonuclease J1/J2 (metallo-b	5E-16	COG0595	mRNA degradation ribonucleases J1/J2 (me
147921738	hypothetical protein LRC94	No	0		3145547	3146122	+		Methanocella_arvoryzae_MRE50_uid61623	NC_009464	chromosome									
147921739	hypothetical protein NRC4	No	0	YES	3146228	3146479	-		Methanocella_arvoryzae_MRE50_uid61623	NC_009464	chromosome									
147921740	hypothetical protein LRC92	No	0	YES	3146633	3147073	-		Methanocella_arvoryzae_MRE50_uid61623	NC_009464	chromosome									
147921741	hypothetical protein LRC91	No	0	YES	3147105	3147581	-		Methanocella_arvoryzae_MRE50_uid61623	NC_009464	chromosome									
147921742	hypothetical protein LRC89	No	0	YES	3148338	3148601	-		Methanocella_arvoryzae_MRE50_uid61623	NC_009464	chromosome									
147921743	hypothetical protein LRC84	No	0	YES	3150676	3151512	-		Methanocella_arvoryzae_MRE50_uid61623	NC_009464	chromosome		arCOG11023	1	S		Uncharacterized conserved protein			
147921744	transposase, orfA	No	0	YES	3151596	3152123	+		Methanocella_arvoryzae_MRE50_uid61623	NC_009464	chromosome		arCOG02128	6 (-)	L		Transposase	0.00000001	pfam13551	Winged helix-turn helix. This helix-turn
===								6	Methanocella_conradii_HZ254_uid157911	NC_017034	chromosome									
383318753	hypothetical protein Mtc_0304	No	0		308710	308871	+		Methanocella_conradii_HZ254_uid157911	NC_017034	chromosome		arCOG01221	1 (-)	F		Inosine/xanthosine triphosphatase			
383318754	hypothetical protein Mtc_0305	No	6		308868	309563	-		Methanocella_conradii_HZ254_uid157911	NC_017034	chromosome	clust_145						0.000004	pfam01925	Sulfite exporter TauE/SafE. This is a fa
383318755	hypothetical protein Mtc_0306	No	2		309655	310377	-		Methanocella_conradii_HZ254_uid157911	NC_017034	chromosome									
383318756	hypothetical protein Mtc_0307	No	0		310388	310696	-		Methanocella_conradii_HZ254_uid157911	NC_017034	chromosome									
383318757	hypothetical protein Mtc_0308	No	6		310698	311372	-		Methanocella_conradii_HZ254_uid157911	NC_017034	chromosome	clust_145						0.0001	pfam01925	Sulfite exporter TauE/SafE. This is a fa
383318758	Necrosis inducing protein (NPP1)	Yes	1		311424	314306	-		Methanocella_conradii_HZ254_uid157911	NC_017034	chromosome	arCOG04998,a	arCOG04998	1 (-)	S		Uncharacterized conserved protein			
===								7	Methanocella_conradii_HZ254_uid157911	NC_017034	chromosome									
383318961	hypothetical protein Mtc_0517	No	0		512513	514183	-		Methanocella_conradii_HZ254_uid157911	NC_017034	chromosome		arCOG06229	1 (-)	S		Uncharacterized conserved protein			
383318962	hypothetical protein Mtc_0518	Yes	1		519819	520373	-		Methanocella_conradii_HZ254_uid157911	NC_017034	chromosome		arCOG10895	5	S		Uncharacterized conserved protein			
383318963	hypothetical protein Mtc_0519	Yes	1		520396	521388	-		Methanocella_conradii_HZ254_uid157911	NC_017034	chromosome									
383318964	hypothetical protein Mtc_0520	No	1		521846	522247	-		Methanocella_conradii_HZ254_uid157911	NC_017034	chromosome									
383318965	hypothetical protein Mtc_0521	Yes	1		522351	522593	-		Methanocella_conradii_HZ254_uid157911	NC_017034	chromosome									
383318966	hypothetical protein Mtc_0522	No	4		522896	523369	+		Methanocella_conradii_HZ254_uid157911	NC_017034	chromosome									
383318967	glutamyl-tRNA(Gln) amidotransferase, subunit E	No	0		523371	525257	-		Methanocella_conradii_HZ254_uid157911	NC_017034	chromosome		arCOG01719	3 (-)	J	GatE	Archaeal Glu-tRNAGln amidotransferase subunit E (contains GAI		COG2511	Archaeal Glu-tRNAGln amidotransferase su
===								5	Methanocella_conradii_HZ254_uid157911	NC_017034	chromosome									
383319026	hypothetical protein Mtc_0583	No	0		586245	586970	+		Methanocella_conradii_HZ254_uid157911	NC_017034	chromosome							0.0001	pfam07263	Dentin matrix protein 1 (DMP1). This fam
383319027	hypothetical protein Mtc_0584	No	0		587141	587332	+		Methanocella_conradii_HZ254_uid157911	NC_017034	chromosome									
383319028	hypothetical protein Mtc_0586	No	1		587743	588021	+		Methanocella_conradii_HZ254_uid157911	NC_017034	chromosome		arCOG03902	8 (-)	L		Transposase			
383319029	hypothetical protein Mtc_0587	No	7		588130	588768	-		Methanocella_conradii_HZ254_uid157911	NC_017034	chromosome									
383319030	hypothetical protein Mtc_0588	No	0		589058	589225	-		Methanocella_conradii_HZ254_uid157911	NC_017034	chromosome									
===								6	Methanocella_conradii_HZ254_uid157911	NC_017034	chromosome									
383319200	pyruvate carboxylase subunit A	No	0		757908	760079	+		Methanocella_conradii_HZ254_uid157911	NC_017034	chromosome	arCOG01590,a	arCOG01590	1 (-)	I	AccC	Biotin carboxylase		COG0439	Biotin carboxylase
383319201	hypothetical protein Mtc_0759	No	0		760315	760599	+		Methanocella_conradii_HZ254_uid157911	NC_017034	chromosome									
383319202	hypothetical protein Mtc_0760	No	0		760615	760881	+		Methanocella_conradii_HZ254_uid157911	NC_017034	chromosome									
383319203	hypothetical protein Mtc_0761	Yes	1		760984	761442	-		Methanocella_conradii_HZ254_uid157911	NC_017034	chromosome									
383319204	hypothetical protein Mtc_0762	No	4		761713	762201	-		Methanocella_conradii_HZ254_uid157911	NC_017034	chromosome									
383319205	hypothetical protein Mtc_0763	No	0		762237	762782	-		Methanocella_conradii_HZ254_uid157911	NC_017034	chromosome		arCOG03097	4 (-)	S		Uncharacterized conserved protein			
===								5	Methanocella_conradii_HZ254_uid157911	NC_017034	chromosome									
383319449	hypothetical protein Mtc_1016	Yes	2		991036	991227	-		Methanocella_conradii_HZ254_uid157911	NC_017034	chromosome									
383319450	hypothetical protein Mtc_1017	Yes	3		991397	991678	-		Methanocella_conradii_HZ254_uid157911	NC_017034	chromosome									
383319451	valyl-tRNA synthetase	No	0		992026	994467	+		Methanocella_conradii_HZ254_uid157911	NC_017034	chromosome		arCOG00808	2 (-)	J	ValS	Valyl-tRNA synthetase		COG0525	Valyl-tRNA synthetase
383319452	hypothetical protein Mtc_1019	No	4		994493	994984	-		Methanocella_conradii_HZ254_uid157911	NC_017034	chromosome		arCOG11123	1	S		Uncharacterized conserved protein	0.0001	COG0477	Permeases of the major facilitator super
383319453	hypothetical protein Mtc_1020	Yes	1		995156	996010	-		Methanocella_conradii_HZ254_uid157911	NC_017034	chromosome		arCOG11012	1	S		Uncharacterized conserved protein			
===								6	Methanocella_conradii_HZ254_uid157911	NC_017034	chromosome									
383319542	transcriptional regulator	No	0		1073627	1074415	+		Methanocella_conradii_HZ254_uid157911	NC_017034	chromosome		arCOG04362	5 (-)	K		Predicted transcriptional regulator	9E-47	COG4742	Predicted transcriptional regulator
383319543	hypothetical protein Mtc_1109	No	0		1074848	1077028	+		Methanocella_conradii_HZ254_uid157911	NC_017034	chromosome									
383319544	hypothetical protein Mtc_1110	Yes	1		1077034	1077609	+		Methanocella_conradii_HZ254_uid157911	NC_017034	chromosome									
383319545	hypothetical protein Mtc_1111	Yes	0		1078524	1078673	+		Methanocella_conradii_HZ254_uid157911	NC_017034	chromosome									
383319546	hypothetical protein Mtc_1112	No	1		1078707	1079195	-		Methanocella_conradii_HZ254_uid157911	NC_017034	chromosome									
383319547	multidrug ABC transporter ATPase	No	0		1079812	1080717	+		Methanocella_conradii_HZ254_uid157911	NC_017034	chromosome		arCOG00194	14 (-)	V	CcmA	ABC-type multidrug transport system, ATPase co	9E-67	COG1131	ABC-type multidrug transport system, ATP
===								6	Methanocella_conradii_HZ254_uid157911	NC_017034	chromosome									
383319619	hypothetical protein Mtc_1187	No	0		1151003	1151422	-		Methanocella_conradii_HZ254_uid157911	NC_017034	chromosome		arCOG02138	2 (-)	L		Transposase			
383319620	hypothetical protein Mtc_1189	No	0		1152571	1154349	+		Methanocella_conradii_HZ254_uid157911	NC_017034	chromosome									
383319621	hypothetical protein Mtc_1190	No	0		1154351	1155322	+		Methanocella_conradii_HZ254_uid157911	NC_017034	chromosome									
383319622	hypothetical protein Mtc_1191	No	0		1155344	1156297	+		Methanocella_conradii_HZ254_uid157911	NC_017034	chromosome									
383319623	hypothetical protein Mtc_1192	No	0		1156299	1157048	+		Methanocella_conradii_HZ254_uid157911	NC_017034	chromosome									
383319624	CRISPR-associated helicase Cas3	No	0		1157038	1159089	+		Methanocella_conradii_HZ254_uid157911	NC_017034	chromosome	arCOG00555,a	arCOG00555	5 (-)	R		Distinct helicase family with a unique C-terminal d	9E-22	COG1203	CRISPR-associated helicase Cas3
===								5	Methanocella_conradii_HZ254_uid157911	NC_017034	chromosome									
383319670	acyltransferase	No	9		1206036	1207139	-		Methanocella_conradii_HZ254_uid157911	NC_017034	chromosome							4E-15	COG1835	Predicted acyltransferases
383319671	RecA-superfamily ATPases implicated in signa	No	0		1207481	1208248	+		Methanocella_conradii_HZ254_uid157911	NC_017034	chromosome		arCOG01171	2 (-)	T	RAD55	RecA-superfamily ATPase implicated in signal tra	6E-43	pfam06745	KaiC. This family represents a conserved
383319672	hypothetical protein Mtc_1241	No	3		1208258	1208542	+		Methanocella_conradii_HZ254_uid157911	NC_017034	chromosome									
383319673	hypothetical protein Mtc_1242	No	3		1208539	1208838	+		Methanocella_conradii_HZ254_uid157911	NC_017034	chromosome									
383319674	hypothetical protein Mtc_1243	No	4		1208918	1209760	+		Methanocella_conradii_HZ254_uid157911	NC_017034	chromosome									
===								14	Methanocella_conradii_HZ254_uid157911	NC_017034	chromosome									
383319797	30D ribosomal protein S6E	No	0		1307261	1307704	+		Methanocella_conradii_HZ254_uid157911	NC_017034	chromosome		arCOG01946	4 (-)	J	RPS6A	Ribosomal protein S6E (S10)	1E-42	COG2125	Ribosomal protein S6E (S10)
383319798	hypothetical protein Mtc_1367	No	0		1307714	1308025	-		Methanocella_conradii_HZ254_uid157911	NC_017034	chromosome	clust_47								
383319799	hypothetical protein Mtc_1368	Yes	1		1308354	1308863	+		Methanocella_conradii_HZ254_uid157911	NC_017034	chromosome		arCOG10873	14	S		Uncharacterized conserved protein			
383319800	hypothetical protein Mtc_1369	Yes	1		1309020	1309517	+		Methanocella_conradii_HZ254_uid157911	NC_017034	chromosome		arCOG10873	14	S		Uncharacterized conserved protein			
383319801	hypothetical protein Mtc_1370	Yes	1		1309676	1310044	+		Methanocella_conradii_HZ254_uid157911	NC_017034	chromosome		arCOG11120	4	S		Uncharacterized conserved protein			
383319802	hypothetical protein Mtc_1371	Yes	1		1310205	1310564	+		Methanocella_conradii_HZ254_uid157911	NC_017034	chromosome		arCOG10873	14	S		Uncharacterized conserved protein			
383319803	hypothetical protein Mtc_1372	Yes	1		1310761	1311129	+		Methanocella_conradii_HZ254_uid157911	NC_017034	chromosome		arCOG11121	7	S		Uncharacterized conserved protein			
383319804	hypothetical protein Mtc_1373	Yes	1		1311264	1311626	+		Methanocella_conradii_HZ254_uid157911	NC_017034	chromosome		arCOG11121	7	S		Uncharacterized conserved protein			
383319805	hypothetical protein Mtc_1374	Yes	3		1311844	1312659	+		Methanocella_conradii_HZ254_uid157911	NC_017034	chromosome	clust_29								
383319806	hypothetical protein Mtc_1375	No	1		1312867	1313022	+		Methanocella_conradii_HZ254_uid157911	NC_017034	chromosome	clust_54								
383319807	hypothetical protein Mtc_1376	No	0		1313370	1313513	+		Methanocella_conradii_HZ254_uid157911	NC_017034	chromosome									
383319808	hypothetical protein Mtc_1377	Yes	1		1313669	1314139	+		Methanocella_conradii_HZ254_uid157911	NC_017034	chromosome		arCOG11122	4	S		Uncharacterized conserved protein			
383319809	hypothetical protein Mtc_1378	No	1		1314569	1314778	+		Methanocella_conradii_HZ254_uid157911	NC_017034	chromosome									
383319810	aspartate/tyrosine/aromatic aminotransferase	No	0		1314759	1315868	-		Methanocella_conradii_HZ254_uid157911	NC_017034	chromosome		arCOG01130	1 (-)	E		Aspartate/tyrosine/aromatic aminotransferase	1E-100	COG0436	Aspartate/tyrosine/aromatic aminotransfe
===								5	Methanocella_conradii_HZ254_uid157911	NC_017034	chromosome									
383319977	hypothetical protein Mtc_1548	No	0		1474154	1474294	+		Methanocella_conradii_HZ254_uid157911	NC_017034	chromosome									
383319978	hypothetical protein Mtc_1549	No	14		1474298	1475545	-		Methanocella_conradii_HZ254_uid157911	NC_017034	chromosome		arCOG04203	1 (-)	P	MgtC	Mg2+ transport system protein	3E-65	COG3174	Predicted membrane protein
383319979	hypothetical protein Mtc_1550	No	0		1475845	1476195	-		Methanocella_conradii_HZ254_uid157911	NC_017034	chromosome									
383319980	hypothetical protein Mtc_1551	No	0		1476286	1476594	-		Methanocella_conradii_HZ254_uid157911	NC_017034	chromosome									
383319981	hypothetical protein Mtc_1552	No	0		1476697	1477020	+		Methanocella_conradii_HZ254_uid157911	NC_017034	chromosome									
===								5	Methanocella_conradii_HZ254_uid157911	NC_017034	chromosome									
383320322	hypothetical protein Mtc_1905	No	2		1819646	1819873	+		Methanocella_conradii_HZ254_uid157911	NC_017034	chromosome									
383320323	hypothetical protein Mtc_1906	No	0		1819887	1820108	-		Methanocella_conradii_HZ254_uid157911	NC_017034	chromosome									
383320324	glutaredoxin and related protein	No	0		1820211	1820450	+		Methanocella_conradii_HZ254_uid157911	NC_017034	chromosome		arCOG02607	2 (-)	O	GrxC	Glutaredoxin	0.000005	COG0695	Glutaredoxin and related proteins
383320325	hypothetical protein Mtc_1908	No	0		1820581	1820727	+		Methanocella_conradii_HZ254_uid157911	NC_017034	chromosome									
383320326	hypothetical protein Mtc_1909	No	2		1820811	1821002	+		Methanocella_conradii_HZ254_uid157911	NC_017034	chromosome	clust_431								
===								5	Methanocella_conradii_HZ254_uid157911	NC_017034	chromosome									
383320473	hypothetical protein Mtc_2057	No	0		1957057	1957986	-		Methanocella_conradii_HZ254_uid157911	NC_017034	chromosome		arCOG04110	4	L	PRI1	Eukaryotic-type DNA primase, catalytic (small) subunit			
383320474	hypothetical protein Mtc_2058	No	1		1958057	1958575	-		Methanocella_conradii_HZ254_uid157911	NC_017034	chromosome	clust_204								
383320475	bacterioferritin	No	0		1958618	1959037	-		Methanocella_conradii_HZ254_uid157911	NC_017034	chromosome		arCOG01094	2 (-)	P	Bfr	Bacterioferritin (cytochrome b1)	5E-15	pfam00210	Ferritin-like domain. This family contai
383320476	hypothetical protein Mtc_2060	Yes	0		1959213	1959569	+		Methanocella_conradii_HZ254_uid157911	NC_017034	chromosome		arCOG11121	7	S		Uncharacterized conserved protein			
383320477	hypothetical protein Mtc_2061	Yes	1		1959697	1960041	+		Methanocella_conradii_HZ254_uid157911	NC_017034	chromosome							0.000002	pfam07172	Glycine rich protein family. This family
===								7	Methanocella_conradii_HZ254_uid157911	NC_017034	chromosome									
383320604	hypothetical protein Mtc_2192	No	0		2084809	2084916	+		Methanocella_conradii_HZ254_uid157911	NC_017034	chromosome									
383320605	DNA topoisomerase I	No	0		2084956	2087058	+		Methanocella_conradii_HZ254_uid157911	NC_017034	chromosome		arCOG01527	2 (-)	L	TopA	Topoisomerase IA	3E-87	COG0550	Topoisomerase IA
383320606	hypothetical protein Mtc_2194	No	0		2087237	2087587	+		Methanocella_conradii_HZ254_uid157911	NC_017034	chromosome	clust_301								
383320607	hypothetical protein Mtc_2195	Yes	0		2087730	2088593	+		Methanocella_conradii_HZ254_uid157911	NC_017034	chromosome		arCOG10916	6	S		Uncharacterized conserved protein			
383320608	hypothetical protein Mtc_2196	Yes	1		2088600	2089682	+		Methanocella_conradii_HZ254_uid157911	NC_017034	chromosome		arCOG10916	6	S		Uncharacterized conserved protein			
383320609	hypothetical protein Mtc_2197	No	0		2089688	2089930	-		Methanocella_conradii_HZ254_uid157911	NC_017034	chromosome		arCOG11115	4	S		Uncharacterized conserved protein	0.0000003	pfam00477	Small hydrophilic plant seed protein.
383320610	translation initiation factor 1 (eIF-1/SUI1)	No	0		2090050	2090358	-		Methanocella_conradii_HZ254_uid157911	NC_017034	chromosome		arCOG04223	2 (-)	J	SUI1	Translation initiation factor 1 (eIF-1/SUI1)	1E-29	COG0023	Translation initiation factor 1 (eIF-1/S
===								6	Methanocella_paludicola_SANAE_uid42887	NC_013665	chromosome									
282162704	putative histidine kinase	No	0		25082	26992	+		Methanocella_paludicola_SANAE_uid42887	NC_013665	chromosome	arCOG05183,a	arCOG05183	1 (-)	T		Signal transduction histidine kinase, contains Poc	4E-23	COG2202	FOG: PAS/PAC domain
282162705	hypothetical protein MCP_0035	No	0		27075	27308	+		Methanocella_paludicola_SANAE_uid42887	NC_013665	chromosome		arCOG11115	4	S		Uncharacterized conserved protein	0.00000001	pfam00477	Small hydrophilic plant seed protein.
282162706	hypothetical protein MCP_0036	Yes	1		27321	27503	+		Methanocella_paludicola_SANAE_uid42887	NC_013665	chromosome									
282162707	hypothetical protein MCP_0037	Yes	2		27508	28233	-		Methanocella_paludicola_SANAE_uid42887	NC_013665	chromosome		arCOG10880	1	S		Uncharacterized conserved protein			
282162708	hypothetical protein MCP_0038	No	0		28331	28759	-		Methanocella_paludicola_SANAE_uid42887	NC_013665	chromosome		arCOG11110	3	S		Uncharacterized conserved protein			
282162709	thioredoxin	No	0		29045	29383	+		Methanocella_paludicola_SANAE_uid42887	NC_013665	chromosome		arCOG01972	5 (-)	O	TrxA	Thiol-disulfide isomerase or thioredoxin	1E-24	COG3118	Thioredoxin domain-containing protein
===								6	Methanocella_paludicola_SANAE_uid42887	NC_013665	chromosome									
282162793	hypothetical protein MCP_0123	No	0		119446	119580	-		Methanocella_paludicola_SANAE_uid42887	NC_013665	chromosome									
282162794	hypothetical protein MCP_0124	No	0		119753	119920	+		Methanocella_paludicola_SANAE_uid42887	NC_013665	chromosome									
282162795	phosphoribosylaminoimidazole carboxylase c	No	0		119927	120316	+		Methanocella_paludicola_SANAE_uid42887	NC_013665	chromosome		arCOG02464	1 (-)	F	PurE	Phosphoribosylcarboxyaminoimidazole (NCAIR)	2E-32	pfam00731	AIR carboxylase. Members of this family
282162796	hypothetical protein MCP_0126	No	2		120313	120513	+		Methanocella_paludicola_SANAE_uid42887	NC_013665	chromosome									
282162797	hypothetical protein MCP_0127	No	0		120517	120750	-		Methanocella_paludicola_SANAE_uid42887	NC_013665	chromosome		arCOG11124	1	S		Uncharacterized conserved protein			
282162798	hypothetical protein MCP_0128	No	0		120934	121983	+		Methanocella_paludicola_SANAE_uid42887	NC_013665	chromosome	clust_92						2E-54	pfam14399	A predicted NlpC/p60-like peptidase. Mem
===								8	Methanocella_paludicola_SANAE_uid42887	NC_013665	chromosome									
282162980	hypothetical protein MCP_0310	Yes	2		310669	312159	+		Methanocella_paludicola_SANAE_uid42887	NC_013665	chromosome		arCOG01917	12 (-)	S		Zn-ribbon domain containing protein	0.00004	pfam12773	Double zinc ribbon. This family consists ; Predicted solute binding protein
282162981	hypothetical protein MCP_0311	No	0		312469	313818	+		Methanocella_paludicola_SANAE_uid42887	NC_013665	chromosome	clust_76						1E-19	pfam04984	Phage tail sheath protein. This family i
282162982	hypothetical protein MCP_0312	No	0		313829	314242	+		Methanocella_paludicola_SANAE_uid42887	NC_013665	chromosome									
282162983	hypothetical protein MCP_0313	No	0		314260	314616	+		Methanocella_paludicola_SANAE_uid42887	NC_013665	chromosome	clust_302								
282162984	hypothetical protein MCP_0314	No	0		314613	314837	+		Methanocella_paludicola_SANAE_uid42887	NC_013665	chromosome	clust_428								
282162985	hypothetical protein MCP_0315	No	0		314912	317083	+		Methanocella_paludicola_SANAE_uid42887	NC_013665	chromosome									
282162986	hypothetical protein MCP_0316	No	0		317090	318280	+		Methanocella_paludicola_SANAE_uid42887	NC_013665	chromosome		arCOG02488	4 (-)	S		Cell suface protein	0.00003	pfam02369	Bacterial Ig-like domain (group 1). This
282162987	hypothetical protein MCP_0317	No	0		318325	319557	+		Methanocella_paludicola_SANAE_uid42887	NC_013665	chromosome									
===								6	Methanocella_paludicola_SANAE_uid42887	NC_013665	chromosome									
282163096	MarR family transcriptional regulator	No	0		427285	427740	+		Methanocella_paludicola_SANAE_uid42887	NC_013665	chromosome		arCOG03177	3 (-)	K	MarR	Transcriptional regulator, MarR family	0.000008	pfam13463	Winged helix DNA-binding domain.
282163097	hypothetical protein MCP_0427	No	6		427833	428579	+		Methanocella_paludicola_SANAE_uid42887	NC_013665	chromosome							6E-21	pfam14329	Domain of unknown function (DUF4386). Th
282163098	hypothetical protein MCP_0428	No	4		428682	429095	+		Methanocella_paludicola_SANAE_uid42887	NC_013665	chromosome									
282163099	hypothetical protein MCP_0429	No	0		429773	430114	-		Methanocella_paludicola_SANAE_uid42887	NC_013665	chromosome									
282163100	hypothetical protein MCP_0430	Yes	0		430609	431226	-		Methanocella_paludicola_SANAE_uid42887	NC_013665	chromosome		arCOG10927	2	S		Uncharacterized conserved protein			
282163101	putative nitrogen regulatory protein P-II	No	0		431524	431853	+		Methanocella_paludicola_SANAE_uid42887	NC_013665	chromosome		arCOG02305	1 (-)	E	GlnK	Nitrogen regulatory protein PII	2E-35	COG0347	Nitrogen regulatory protein PII
===								6	Methanocella_paludicola_SANAE_uid42887	NC_013665	chromosome									
282163712	hypothetical protein MCP_1042	No	2		1044683	1044904	-		Methanocella_paludicola_SANAE_uid42887	NC_013665	chromosome	clust_431								
282163713	hypothetical protein MCP_1043	Yes	0		1044979	1045596	-		Methanocella_paludicola_SANAE_uid42887	NC_013665	chromosome									
282163714	hypothetical protein MCP_1044	No	2		1045694	1046260	-		Methanocella_paludicola_SANAE_uid42887	NC_013665	chromosome							0.0000005	pfam06346	Formin Homology Region 1. This region is
282163715	putative family B DNA polymerase	No	0		1046378	1049128	-		Methanocella_paludicola_SANAE_uid42887	NC_013665	chromosome		arCOG00328	8 (-)	L	PolB	DNA polymerase elongation subunit (family B)	2E-69	COG0417	DNA polymerase elongation subunit (famil
282163716	hypothetical protein MCP_1046	No	2		1049222	1049626	-		Methanocella_paludicola_SANAE_uid42887	NC_013665	chromosome									
282163717	hypothetical protein MCP_1047	No	4		1049722	1050159	-		Methanocella_paludicola_SANAE_uid42887	NC_013665	chromosome									
===								8	Methanocella_paludicola_SANAE_uid42887	NC_013665	chromosome									
282163766	putative ArsR family transcriptional regulator	No	0	YES	1091631	1092242	+		Methanocella_paludicola_SANAE_uid42887	NC_013665	chromosome		arCOG01687	1 (-)	K	ArsR	Transcriptional regulator containing HTH domain,	0.00006	COG1777	Predicted transcriptional regulators
282163767	hypothetical protein MCP_1097	Yes	2	YES	1092368	1092835	+		Methanocella_paludicola_SANAE_uid42887	NC_013665	chromosome		arCOG11122	4	S		Uncharacterized conserved protein			
282163768	hypothetical protein MCP_1098	No	1	YES	1094059	1094493	-		Methanocella_paludicola_SANAE_uid42887	NC_013665	chromosome		arCOG11121	7	S		Uncharacterized conserved protein			
282163769	hypothetical protein MCP_1099	Yes	1	YES	1094658	1095104	-		Methanocella_paludicola_SANAE_uid42887	NC_013665	chromosome		arCOG10873	14	S		Uncharacterized conserved protein			
282163770	hypothetical protein MCP_1100	No	0	YES	1095254	1095481	-		Methanocella_paludicola_SANAE_uid42887	NC_013665	chromosome									
282163771	hypothetical protein MCP_1101	No	0	YES	1095838	1096101	+		Methanocella_paludicola_SANAE_uid42887	NC_013665	chromosome									
282163772	hypothetical protein MCP_1102	No	0		1096114	1096458	-		Methanocella_paludicola_SANAE_uid42887	NC_013665	chromosome		arCOG01060	1 (-)	K	ArsR	Transcriptional regulator containing HTH domain, ArsR family			
282163773	hypothetical protein MCP_1103	Yes	0		1096550	1096798	+		Methanocella_paludicola_SANAE_uid42887	NC_013665	chromosome									
===								6	Methanocella_paludicola_SANAE_uid42887	NC_013665	chromosome									
282163873	hypothetical protein MCP_1203	Yes	2		1189466	1190875	+		Methanocella_paludicola_SANAE_uid42887	NC_013665	chromosome		arCOG02081	3 (-)	S		Predicted membrane protein	4E-30	COG1470	Predicted membrane protein
282163874	hypothetical protein MCP_1204	Yes	1		1191032	1191403	+		Methanocella_paludicola_SANAE_uid42887	NC_013665	chromosome									
282163875	hypothetical protein MCP_1205	Yes	1		1191498	1192550	-		Methanocella_paludicola_SANAE_uid42887	NC_013665	chromosome		arCOG10916	6	S		Uncharacterized conserved protein			
282163876	hypothetical protein MCP_1206	Yes	1		1192958	1194106	+		Methanocella_paludicola_SANAE_uid42887	NC_013665	chromosome		arCOG10916	6	S		Uncharacterized conserved protein			
282163877	hypothetical protein MCP_1207	No	2		1194117	1194716	+		Methanocella_paludicola_SANAE_uid42887	NC_013665	chromosome									
282163878	hypothetical protein MCP_1208	No	0		1194805	1195320	+		Methanocella_paludicola_SANAE_uid42887	NC_013665	chromosome		arCOG02561	1 (-)	S		WD40 repeats containing protein	5E-45	COG2319	FOG: WD40 repeat
===								7	Methanocella_paludicola_SANAE_uid42887	NC_013665	chromosome									
282164067	hypothetical protein MCP_1397	No	2		1399540	1399884	-		Methanocella_paludicola_SANAE_uid42887	NC_013665	chromosome									
282164068	putative site-specific recombinase	No	0		1400518	1401417	+		Methanocella_paludicola_SANAE_uid42887	NC_013665	chromosome		arCOG01241	31 (-)	L	XerC	Integrase	6E-16	pfam00589	Phage integrase family. Members of this
282164069	hypothetical protein MCP_1399	No	0		1401733	1402398	+		Methanocella_paludicola_SANAE_uid42887	NC_013665	chromosome		arCOG06786	9	Y		Rolling circle replication protein Rep			
282164070	hypothetical protein MCP_1400	No	0		1402544	1402813	+		Methanocella_paludicola_SANAE_uid42887	NC_013665	chromosome									
282164071	hypothetical protein MCP_1401	No	5		1402881	1403549	+		Methanocella_paludicola_SANAE_uid42887	NC_013665	chromosome							5E-11	pfam13787	Protein of unknown function with HXXEE m
282164072	hypothetical protein MCP_1402	No	0		1403996	1405057	+		Methanocella_paludicola_SANAE_uid42887	NC_013665	chromosome									
282164073	putative large-conductance mechanosensitive	No	3		1405186	1405491	+		Methanocella_paludicola_SANAE_uid42887	NC_013665	chromosome		arCOG05213	2 (-)	M	MscL	Large-conductance mechanosensitive channel	4E-18	COG1970	Large-conductance mechanosensitive chann
===								8	Methanocella_paludicola_SANAE_uid42887	NC_013665	chromosome									
282164165	putative signal peptide peptidase	No	1		1499210	1500091	-		Methanocella_paludicola_SANAE_uid42887	NC_013665	chromosome		arCOG01311	1 (-)	O	SppA	Periplasmic serine protease (ClpP class)	2E-54	COG0616	Periplasmic serine proteases (ClpP class
282164166	hypothetical protein MCP_1496	No	1		1501055	1501954	+		Methanocella_paludicola_SANAE_uid42887	NC_013665	chromosome									
282164167	hypothetical protein MCP_1497	No	0		1502124	1502357	-		Methanocella_paludicola_SANAE_uid42887	NC_013665	chromosome									
282164168	hypothetical protein MCP_1498	No	0		1502363	1502572	-		Methanocella_paludicola_SANAE_uid42887	NC_013665	chromosome									
282164169	hypothetical protein MCP_1499	No	0		1502618	1502797	+		Methanocella_paludicola_SANAE_uid42887	NC_013665	chromosome									
282164170	hypothetical protein MCP_1500	No	0		1502991	1503158	-		Methanocella_paludicola_SANAE_uid42887	NC_013665	chromosome		arCOG10928	3	S		Uncharacterized conserved protein	0.0001	pfam00477	Small hydrophilic plant seed protein.
282164171	putative chromosomal protein MC1	No	0		1503260	1503748	-		Methanocella_paludicola_SANAE_uid42887	NC_013665	chromosome		arCOG04743	1 (-)	R		Helicase associated uncharacterized terminal dor	2E-18	pfam05854	Non-histone chromosomal protein MC1. Thi
282164172	hypothetical protein MCP_1502	No	0		1503779	1504393	-		Methanocella_paludicola_SANAE_uid42887	NC_013665	chromosome									
===								9	Methanocella_paludicola_SANAE_uid42887	NC_013665	chromosome									
282164187	hypothetical protein MCP_1517	No	0		1515163	1515336	+		Methanocella_paludicola_SANAE_uid42887	NC_013665	chromosome		arCOG10928	3	S		Uncharacterized conserved protein	0.0001	pfam00477	Small hydrophilic plant seed protein.
282164188	hypothetical protein MCP_1518	Yes	2		1515485	1516423	+		Methanocella_paludicola_SANAE_uid42887	NC_013665	chromosome	arCOG11021,a	arCOG11021	1 (-)	S		Uncharacterized conserved protein			
282164189	hypothetical protein MCP_1519	No	0		1517072	1517962	+		Methanocella_paludicola_SANAE_uid42887	NC_013665	chromosome		arCOG10238	4	S		Uncharacterized conserved protein			
282164190	hypothetical protein MCP_1520	No	1		1518029	1518748	+		Methanocella_paludicola_SANAE_uid42887	NC_013665	chromosome									
282164191	hypothetical protein MCP_1521	Yes	1		1519467	1520042	+		Methanocella_paludicola_SANAE_uid42887	NC_013665	chromosome									
282164192	hypothetical protein MCP_1522	No	0		1520070	1520459	+		Methanocella_paludicola_SANAE_uid42887	NC_013665	chromosome									
282164193	hypothetical protein MCP_1523	Yes	1		1520539	1521093	+		Methanocella_paludicola_SANAE_uid42887	NC_013665	chromosome		arCOG10895	5	S		Uncharacterized conserved protein			
282164194	pyruvate kinase	No	0		1526995	1528746	-		Methanocella_paludicola_SANAE_uid42887	NC_013665	chromosome	arCOG01111,a	arCOG01111	1 (-)	G	PpsA	Phosphoenolpyruvate synthase/pyruvate phosph	6E-154	COG0469	Pyruvate kinase
282164195	hypothetical protein MCP_1525	No	6		1528781	1529494	-		Methanocella_paludicola_SANAE_uid42887	NC_013665	chromosome									
===								19	Methanocella_paludicola_SANAE_uid42887	NC_013665	chromosome									
282164352	30S ribosomal protein S6e	No	0		1695299	1695739	+		Methanocella_paludicola_SANAE_uid42887	NC_013665	chromosome		arCOG01946	4 (-)	J	RPS6A	Ribosomal protein S6E (S10)	8E-40	COG2125	Ribosomal protein S6E (S10)
282164353	hypothetical protein MCP_1683	No	0		1695747	1696055	-		Methanocella_paludicola_SANAE_uid42887	NC_013665	chromosome	clust_47								
282164354	hypothetical protein MCP_1684	Yes	1		1696261	1696578	+		Methanocella_paludicola_SANAE_uid42887	NC_013665	chromosome		arCOG10873	14	S		Uncharacterized conserved protein			
282164355	hypothetical protein MCP_1685	Yes	1		1696745	1697185	+		Methanocella_paludicola_SANAE_uid42887	NC_013665	chromosome		arCOG10873	14	S		Uncharacterized conserved protein			
282164356	hypothetical protein MCP_1686	Yes	1		1697393	1697833	+		Methanocella_paludicola_SANAE_uid42887	NC_013665	chromosome		arCOG10873	14	S		Uncharacterized conserved protein			
282164357	hypothetical protein MCP_1687	Yes	1		1698059	1698502	+		Methanocella_paludicola_SANAE_uid42887	NC_013665	chromosome		arCOG10873	14	S		Uncharacterized conserved protein			
282164358	hypothetical protein MCP_1688	Yes	1		1698743	1699183	+		Methanocella_paludicola_SANAE_uid42887	NC_013665	chromosome		arCOG10873	14	S		Uncharacterized conserved protein			
282164359	hypothetical protein MCP_1689	Yes	1		1699334	1699714	+		Methanocella_paludicola_SANAE_uid42887	NC_013665	chromosome		arCOG11120	4	S		Uncharacterized conserved protein			
282164360	hypothetical protein MCP_1690	Yes	1		1699855	1700232	+		Methanocella_paludicola_SANAE_uid42887	NC_013665	chromosome		arCOG10873	14	S		Uncharacterized conserved protein			
282164361	hypothetical protein MCP_1691	Yes	1		1700387	1700749	+		Methanocella_paludicola_SANAE_uid42887	NC_013665	chromosome		arCOG11120	4	S		Uncharacterized conserved protein			
282164362	hypothetical protein MCP_1692	Yes	1		1700916	1701299	+		Methanocella_paludicola_SANAE_uid42887	NC_013665	chromosome		arCOG11121	7	S		Uncharacterized conserved protein			
282164363	hypothetical protein MCP_1693	Yes	3		1701420	1701785	+		Methanocella_paludicola_SANAE_uid42887	NC_013665	chromosome		arCOG11121	7	S		Uncharacterized conserved protein			
282164364	hypothetical protein MCP_1694	Yes	3		1701966	1703030	+		Methanocella_paludicola_SANAE_uid42887	NC_013665	chromosome	clust_29						0.000006	COG4371	Predicted membrane protein
282164365	hypothetical protein MCP_1695	No	2		1703297	1703560	+		Methanocella_paludicola_SANAE_uid42887	NC_013665	chromosome	clust_54						0.000003	pfam11759	Keratin-associated matrix. The major str
282164366	hypothetical protein MCP_1696	No	3		1703663	1704244	+		Methanocella_paludicola_SANAE_uid42887	NC_013665	chromosome		arCOG11122	4	S		Uncharacterized conserved protein			
282164367	hypothetical protein MCP_1697	Yes	3		1704528	1705304	+		Methanocella_paludicola_SANAE_uid42887	NC_013665	chromosome		arCOG11111	2	S		Uncharacterized conserved protein	0.000001	pfam11759	Keratin-associated matrix. The major str
282164368	hypothetical protein MCP_1698	No	2		1705435	1706115	+		Methanocella_paludicola_SANAE_uid42887	NC_013665	chromosome		arCOG10873	14	S		Uncharacterized conserved protein			
282164369	hypothetical protein MCP_1699	No	6		1706888	1707847	-		Methanocella_paludicola_SANAE_uid42887	NC_013665	chromosome									
282164370	putative PadR-like family transcriptional regulat	No	0		1707947	1708288	-		Methanocella_paludicola_SANAE_uid42887	NC_013665	chromosome		arCOG00001	1 (-)	K		Predicted transcriptional regulator, PadR family	7E-15	COG1695	Predicted transcriptional regulators
===								7	Methanocella_paludicola_SANAE_uid42887	NC_013665	chromosome									
282164374	hypothetical protein MCP_1704	No	0		1712360	1712632	+		Methanocella_paludicola_SANAE_uid42887	NC_013665	chromosome									
282164375	putative FKBP-type peptidyl-prolyl cis-trans isc	No	0		1712897	1713607	-		Methanocella_paludicola_SANAE_uid42887	NC_013665	chromosome		arCOG00980	1 (-)	O	SlpA	FKBP-type peptidyl-prolyl cis-trans isomerase 2	7E-30	COG1047	FKBP-type peptidyl-prolyl cis-trans isom
282164376	hypothetical protein MCP_1706	No	0		1713649	1713843	-		Methanocella_paludicola_SANAE_uid42887	NC_013665	chromosome									
282164377	hypothetical protein MCP_1707	Yes	0		1713842	1714414	+		Methanocella_paludicola_SANAE_uid42887	NC_013665	chromosome							0.00000002	pfam07174	Fibronectin-attachment protein (FAP). Th
282164378	hypothetical protein MCP_1708	No	4		1714477	1715016	-		Methanocella_paludicola_SANAE_uid42887	NC_013665	chromosome									
282164379	hypothetical protein MCP_1709	Yes	0		1715067	1715744	-		Methanocella_paludicola_SANAE_uid42887	NC_013665	chromosome									
282164380	adenylate cyclase	No	0		1715811	1716338	+		Methanocella_paludicola_SANAE_uid42887	NC_013665	chromosome		arCOG01723	2 (-)	F	CyaB	Adenylate cyclase, class 2 (thermophilic)	4E-46	COG1437	Adenylate cyclase, class 2 (thermophilic
===								6	Methanocella_paludicola_SANAE_uid42887	NC_013665	chromosome									
282164664	hypothetical protein MCP_1994	Yes	0		2006686	2006946	+		Methanocella_paludicola_SANAE_uid42887	NC_013665	chromosome									
282164665	hypothetical protein MCP_1995	No	0		2007148	2007453	+		Methanocella_paludicola_SANAE_uid42887	NC_013665	chromosome		arCOG10926	2	S		Uncharacterized conserved protein	0.000000001	pfam00477	Small hydrophilic plant seed protein.
282164666	hypothetical protein MCP_1996	No	0		2007963	2008217	+		Methanocella_paludicola_SANAE_uid42887	NC_013665	chromosome		arCOG10926	2	S		Uncharacterized conserved protein	0.0000004	pfam00477	Small hydrophilic plant seed protein.
282164667	hypothetical protein MCP_1997	No	0		2008232	2008426	+		Methanocella_paludicola_SANAE_uid42887	NC_013665	chromosome		arCOG03593	2 (-)	S		Uncharacterized conserved protein	3E-12	pfam11387	Protein of unknown function (DUF2795). T
282164668	hypothetical protein MCP_1998	No	0		2008562	2008777	+		Methanocella_paludicola_SANAE_uid42887	NC_013665	chromosome									
282164669	hypothetical protein MCP_1999	No	0		2008795	2009121	-		Methanocella_paludicola_SANAE_uid42887	NC_013665	chromosome									
===								7	Methanocella_paludicola_SANAE_uid42887	NC_013665	chromosome									
282164704	hypothetical protein MCP_2034	No	0		2041487	2041828	+		Methanocella_paludicola_SANAE_uid42887	NC_013665	chromosome									
282164705	cytosine deaminase	No	0		2041851	2042288	+		Methanocella_paludicola_SANAE_uid42887	NC_013665	chromosome		arCOG01488	1 (-)	F	CumB	Cytosine/adenosine deaminase	8E-20	COG0590	Cytosine/adenosine deaminases
282164706	hypothetical protein MCP_2036	No	0		2042406	2042639	+		Methanocella_paludicola_SANAE_uid42887	NC_013665	chromosome		arCOG09480	2	S		Uncharacterized conserved protein, DUF2164	2E-13	pfam09932	Uncharacterized conserved protein (DUF21
282164707	hypothetical protein MCP_2037	No	1		2042722	2043354	+		Methanocella_paludicola_SANAE_uid42887	NC_013665	chromosome									
282164708	hypothetical protein MCP_2038	No	3		2043447	2044124	-		Methanocella_paludicola_SANAE_uid42887	NC_013665	chromosome									
282164709	hypothetical protein MCP_2039	No	6		2044435	2045232	+		Methanocella_paludicola_SANAE_uid42887	NC_013665	chromosome									
282164710	hypothetical protein MCP_2040	No	0		2045236	2045550	+		Methanocella_paludicola_SANAE_uid42887	NC_013665	chromosome		arCOG02099	2 (-)	K	TroR	Mn-dependent transcriptional regulator (DtxR family)			
===								10	Methanocella_paludicola_SANAE_uid42887	NC_013665	chromosome									
282164769	hypothetical protein MCP_2099	No	5		2091711	2092412	+		Methanocella_paludicola_SANAE_uid42887	NC_013665	chromosome		arCOG03580	2 (-)	O	STE14	Putative protein-S-isoprenylcysteine methyltransf	2E-13	COG2020	Putative protein-S-isoprenylcysteine met
282164770	hypothetical protein MCP_2100	No	2		2092432	2092629	-		Methanocella_paludicola_SANAE_uid42887	NC_013665	chromosome							0.000003	pfam11127	Protein of unknown function (DUF2892). T
282164771	hypothetical protein MCP_2101	No	0		2092699	2092875	-		Methanocella_paludicola_SANAE_uid42887	NC_013665	chromosome		arCOG10686	1	S		Uncharacterized conserved protein	0.00006	pfam06348	Protein of unknown function (DUF1059). T
282164772	hypothetical protein MCP_2102	No	0		2092961	2093302	-		Methanocella_paludicola_SANAE_uid42887	NC_013665	chromosome									
282164773	hypothetical protein MCP_2103	No	0		2093440	2093673	-		Methanocella_paludicola_SANAE_uid42887	NC_013665	chromosome									
282164774	hypothetical protein MCP_2104	No	1		2093854	2094372	-		Methanocella_paludicola_SANAE_uid42887	NC_013665	chromosome	clust_204								
282164775	hypothetical protein MCP_2105	No	0		2094469	2094990	-		Methanocella_paludicola_SANAE_uid42887	NC_013665	chromosome									
282164776	hypothetical protein MCP_2106	No	0		2095121	2095783	-		Methanocella_paludicola_SANAE_uid42887	NC_013665	chromosome									
282164777	hypothetical protein MCP_2107	Yes	1		2096168	2096632	+		Methanocella_paludicola_SANAE_uid42887	NC_013665	chromosome		arCOG06717	1 (-)	S		Uncharacterized conserved protein			
282164778	hypothetical protein MCP_2108	Yes	0		2096692	2097063	-		Methanocella_paludicola_SANAE_uid42887	NC_013665	chromosome							0.0000003	pfam07172	Glycine rich protein family. This family
===								8	Methanocella_paludicola_SANAE_uid42887	NC_013665	chromosome									
282164788	hypothetical protein MCP_2118	No	0		2103530	2103934	+		Methanocella_paludicola_SANAE_uid42887	NC_013665	chromosome		arCOG01094	2 (-)	P	Bfr	Bacterioferritin (cytochrome b1)			
282164789	hypothetical protein MCP_2119	No	6		2103999	2104562	+		Methanocella_paludicola_SANAE_uid42887	NC_013665	chromosome		arCOG03583	3	S	HdeD	Uncharacterized conserved protein	4E-21	COG3247	Uncharacterized conserved protein
282164790	hypothetical protein MCP_2120	No	0		2104674	2105021	-		Methanocella_paludicola_SANAE_uid42887	NC_013665	chromosome									
282164791	hypothetical protein MCP_2121	No	0		2105018	2105182	-		Methanocella_paludicola_SANAE_uid42887	NC_013665	chromosome									
282164792	hypothetical protein MCP_2122	Yes	1		2105265	2105936	-		Methanocella_paludicola_SANAE_uid42887	NC_013665	chromosome									
282164793	hypothetical protein MCP_2123	No	0		2106064	2106393	+		Methanocella_paludicola_SANAE_uid42887	NC_013665	chromosome									
282164794	aldo/keto reductase family protein	No	0		2106390	2107511	-		Methanocella_paludicola_SANAE_uid42887	NC_013665	chromosome		arCOG01624	3 (-)	R		Predicted oxidoreductase of the aldo/keto reduc	4E-145	COG1453	Predicted oxidoreductases of the aldo/ke
282164795	hypothetical protein MCP_2125	No	0		2107937	2108824	-		Methanocella_paludicola_SANAE_uid42887	NC_013665	chromosome		arCOG04110	4	L	PRI1	Eukaryotic-type DNA primase, catalytic (small) su	7E-76	COG3285	Predicted eukaryotic-type DNA primase
===								5	Methanocella_paludicola_SANAE_uid42887	NC_013665	chromosome									
282164813	hypothetical protein MCP_2143	No	0		2124947	2125246	+		Methanocella_paludicola_SANAE_uid42887	NC_013665	chromosome									
282164814	hypothetical protein MCP_2144	No	0		2125367	2125930	-		Methanocella_paludicola_SANAE_uid42887	NC_013665	chromosome		arCOG04693	1 (-)	S		Uncharacterized conserved protein			
282164815	hypothetical protein MCP_2145	No	1		2126205	2126609	-		Methanocella_paludicola_SANAE_uid42887	NC_013665	chromosome									
282164816	hypothetical protein MCP_2146	Yes	1		2126868	2127203	+		Methanocella_paludicola_SANAE_uid42887	NC_013665	chromosome		arCOG11110	3	S		Uncharacterized conserved protein			
282164817	hypothetical protein MCP_2147	Yes	2		2127403	2127840	+		Methanocella_paludicola_SANAE_uid42887	NC_013665	chromosome		arCOG11126	2	S		Uncharacterized conserved protein			
===								9	Methanocella_paludicola_SANAE_uid42887	NC_013665	chromosome									
282164829	putative aminotransferase	No	0		2136889	2138097	-		Methanocella_paludicola_SANAE_uid42887	NC_013665	chromosome		arCOG00492	2 (-)	K	ARO8	Transcriptional regulators containing a DNA-bindi	4E-92	COG1167	Transcriptional regulators containing a
282164830	hypothetical protein MCP_2160	No	0		2139326	2139694	+		Methanocella_paludicola_SANAE_uid42887	NC_013665	chromosome									
282164831	hypothetical protein MCP_2161	No	0		2139698	2140252	+		Methanocella_paludicola_SANAE_uid42887	NC_013665	chromosome									
282164832	hypothetical protein MCP_2162	No	0		2140253	2141023	+		Methanocella_paludicola_SANAE_uid42887	NC_013665	chromosome									
282164833	hypothetical protein MCP_2163	No	0		2141887	2142600	+		Methanocella_paludicola_SANAE_uid42887	NC_013665	chromosome							7E-11	pfam09250	Bifunctional DNA primase/polymerase, N-t
282164834	hypothetical protein MCP_2164	No	0		2142572	2144029	+		Methanocella_paludicola_SANAE_uid42887	NC_013665	chromosome		arCOG01174	1 (-)	T	RAD55	RecA-superfamily ATPase implicated in signal tra	8E-14	pfam13481	AAA domain. This AAA domain is found in
282164835	hypothetical protein MCP_2165	No	0		2144462	2144689	+		Methanocella_paludicola_SANAE_uid42887	NC_013665	chromosome									
282164836	hypothetical protein MCP_2166	No	0		2146003	2146665	+		Methanocella_paludicola_SANAE_uid42887	NC_013665	chromosome									
282164837	hypothetical protein MCP_2167	No	0		2146688	2146927	-		Methanocella_paludicola_SANAE_uid42887	NC_013665	chromosome									
===								5	Methanocella_paludicola_SANAE_uid42887	NC_013665	chromosome									
282164857	hypothetical protein MCP_2187	Yes	1		2161032	2161466	-		Methanocella_paludicola_SANAE_uid42887	NC_013665	chromosome									
282164858	hypothetical protein MCP_2188	Yes	1		2161621	2162196	+		Methanocella_paludicola_SANAE_uid42887	NC_013665	chromosome									
282164859	hypothetical protein MCP_2189	Yes	1		2162239	2162724	+		Methanocella_paludicola_SANAE_uid42887	NC_013665	chromosome									
282164860	putative signaling protein	No	0		2162979	2165834	+		Methanocella_paludicola_SANAE_uid42887	NC_013665	chromosome	arCOG02350,a	arCOG02350	1 (-)	T		PAS domain	5E-25	COG2202	FOG: PAS/PAC domain
282164861	hypothetical protein MCP_2191	Yes	2		2165931	2166176	-		Methanocella_paludicola_SANAE_uid42887	NC_013665	chromosome	clust_54						0.0000007	pfam06403	Lamprin. This family consists of several
===								6	Methanocella_paludicola_SANAE_uid42887	NC_013665	chromosome									
282164992	hypothetical protein MCP_2322	No	0		2276094	2278205	-		Methanocella_paludicola_SANAE_uid42887	NC_013665	chromosome	arCOG00429,a	arCOG00429	1 (-)	L		Archaea-specific RecJ-like exonuclease, contains DnaJ-type Zn		COG1107	Archaea-specific RecJ-like exonuclease,
282164993	hypothetical protein MCP_2323	No	0		2278323	2278610	+		Methanocella_paludicola_SANAE_uid42887	NC_013665	chromosome									
282164994	hypothetical protein MCP_2324	No	2		2278638	2279258	-		Methanocella_paludicola_SANAE_uid42887	NC_013665	chromosome									
282164995	hypothetical protein MCP_2325	No	0		2279499	2279786	+		Methanocella_paludicola_SANAE_uid42887	NC_013665	chromosome									
282164996	hypothetical protein MCP_2326	No	0		2279911	2280456	+		Methanocella_paludicola_SANAE_uid42887	NC_013665	chromosome									
282164997	hypothetical protein MCP_2327	No	3		2281137	2281391	+		Methanocella_paludicola_SANAE_uid42887	NC_013665	chromosome		arCOG05022	1 (-)	S		Uncharacterized conserved protein	2E-13	COG4095	Uncharacterized conserved protein
===								5	Methanocella_paludicola_SANAE_uid42887	NC_013665	chromosome									
282165322	hypothetical protein MCP_2652	No	1		2599492	2600004	-		Methanocella_paludicola_SANAE_uid42887	NC_013665	chromosome									
282165323	hypothetical protein MCP_2653	Yes	1		2600076	2600486	+		Methanocella_paludicola_SANAE_uid42887	NC_013665	chromosome		arCOG10873	14	S		Uncharacterized conserved protein			
282165324	hypothetical protein MCP_2654	Yes	1		2600494	2601192	-		Methanocella_paludicola_SANAE_uid42887	NC_013665	chromosome		arCOG10927	2	S		Uncharacterized conserved protein			
282165325	signal recognition 54 kDa protein	No	0		2601255	2602583	-		Methanocella_paludicola_SANAE_uid42887	NC_013665	chromosome		arCOG01228	1 (-)	U	Ffh	Signal recognition particle GTPase	1E-125	COG0541	Signal recognition particle GTPase
282165326	hypothetical protein MCP_2656	Yes	0		2602702	2603322	+		Methanocella_paludicola_SANAE_uid42887	NC_013665	chromosome		arCOG09484	1	S		Uncharacterized conserved protein			
===								6	Methanococcoides_burtonii_DSM_6242_uid5802	NC_007955	chromosome									
91772668	PAS/PAC sensor signal transduction histidine I	No	2		658646	660016	+		Methanococcoides_burtonii_DSM_6242_uid5802	NC_007955	chromosome		arCOG02358	4 (-)	T		Signal transduction histidine kinase and HAMP dc	7E-48	COG0642	Signal transduction histidine kinase
91772669	hypothetical protein Mbur_0640	No	1		660244	660546	+		Methanococcoides_burtonii_DSM_6242_uid5802	NC_007955	chromosome		arCOG07647	3	S		Uncharacterized conserved protein			
91772670	hypothetical protein Mbur_0641	No	0		660553	660810	+		Methanococcoides_burtonii_DSM_6242_uid5802	NC_007955	chromosome									
91772671	hypothetical protein Mbur_0642	No	2		660917	661156	+		Methanococcoides_burtonii_DSM_6242_uid5802	NC_007955	chromosome									
91772672	hypothetical protein Mbur_0643	No	0		661620	662006	-		Methanococcoides_burtonii_DSM_6242_uid5802	NC_007955	chromosome									
91772673	hypothetical protein Mbur_0644	No	5		662069	662689	-		Methanococcoides_burtonii_DSM_6242_uid5802	NC_007955	chromosome		arCOG03118	1 (-)	S		Membrane protein DegA family			
===								5	Methanococcoides_burtonii_DSM_6242_uid5802	NC_007955	chromosome									
91773657	hypothetical protein Mbur_1706	No	2		1791479	1791955	+		Methanococcoides_burtonii_DSM_6242_uid5802	NC_007955	chromosome							5E-12	pfam14015	Protein of unknown function (DUF4231). T
91773658	A-macroglobulin like protein	Yes	0		1792043	1796254	-		Methanococcoides_burtonii_DSM_6242_uid5802	NC_007955	chromosome		arCOG03396	1 (-)	I	SqhC	Squalene cyclase	1E-45	pfam07678	A-macroglobulin complement component. Th ; Alpha-2-macroglobulin family. This famil ; Large extracellular alpha-helical protei ; A-macroglobulin receptor. This family in ; Alpha-2-macroglobulin family N-terminal ; Alpha-macro-globulin thiol-ester bond-fo
91773659	hypothetical protein Mbur_1709	Yes	2		1796720	1797277	-		Methanococcoides_burtonii_DSM_6242_uid5802	NC_007955	chromosome		arCOG03577	1	S		Uncharacterized conserved protein			
91773660	peptidase	Yes	0		1797569	1798483	-		Methanococcoides_burtonii_DSM_6242_uid5802	NC_007955	chromosome		arCOG06558	10	S		Uncharacterized conserved protein			
91773661	hypothetical protein Mbur_1711	No	1		1798602	1799030	-		Methanococcoides_burtonii_DSM_6242_uid5802	NC_007955	chromosome		arCOG03345	4	S		Uncharacterized conserved protein			
===								6	Methanococcus_aeolicus_Nankai_3_uid58823	NC_009635	chromosome									
150400957	hypothetical protein Maeo_0525	No	4		545096	545518	-		Methanococcus_aeolicus_Nankai_3_uid58823	NC_009635	chromosome	clust_247								

150400958	hypothetical protein Maeo_0526	Yes	2			545570	545890	-		Methanococcus_aeolicus_Nankai_3_uid58823	NC_009635	chromosome		arCOG10897	10	S		Uncharacterized conserved protein			
150400959	hypothetical protein Maeo_0527	No	5			546143	546670	-		Methanococcus_aeolicus_Nankai_3_uid58823	NC_009635	chromosome		arCOG01996	17 (-)	S	SpoIIM	Uncharacterized membrane protein	1E-18	COG1300	Uncharacterized membrane protein
150400960	hypothetical protein Maeo_0528	No	4			546683	547150	-		Methanococcus_aeolicus_Nankai_3_uid58823	NC_009635	chromosome		arCOG10443	4	S		Uncharacterized conserved protein			
150400961	hypothetical protein Maeo_0529	Yes	2			547179	547478	-		Methanococcus_aeolicus_Nankai_3_uid58823	NC_009635	chromosome		arCOG10897	10	S		Uncharacterized conserved protein			
150400962	hypothetical protein Maeo_0530	No	1			547736	547837	-		Methanococcus_aeolicus_Nankai_3_uid58823	NC_009635	chromosome									
===									7	Methanococcus_aeolicus_Nankai_3_uid58823	NC_009635	chromosome									
150401395	4Fe-4S ferredoxin iron-sulfur binding domain-c	No	0			992307	993020	-		Methanococcus_aeolicus_Nankai_3_uid58823	NC_009635	chromosome		arCOG00296	1 (-)	C	NapF	Ferredoxin	0.00000007	COG1145	Ferredoxin
150401396	hypothetical protein Maeo_0972	No	1			993137	993529	-		Methanococcus_aeolicus_Nankai_3_uid58823	NC_009635	chromosome									
150401397	hypothetical protein Maeo_0973	No	0			993585	994232	-		Methanococcus_aeolicus_Nankai_3_uid58823	NC_009635	chromosome									
150401398	hypothetical protein Maeo_0974	No	0			994248	994814	-		Methanococcus_aeolicus_Nankai_3_uid58823	NC_009635	chromosome									
150401399	hypothetical protein Maeo_0975	No	0			995174	995800	-		Methanococcus_aeolicus_Nankai_3_uid58823	NC_009635	chromosome									
150401400	hypothetical protein Maeo_0976	Yes	1			995818	997206	-		Methanococcus_aeolicus_Nankai_3_uid58823	NC_009635	chromosome									
150401401	CopG family transcriptional regulator	No	0	YES		997499	997906	-		Methanococcus_aeolicus_Nankai_3_uid58823	NC_009635	chromosome		arCOG01008	7 (-)	V	NikR	Transcriptional regulator, CopG/Arc/MetJ family (I	2E-17	COG0864	Predicted transcriptional regulators con
===									21	Methanococcus_aeolicus_Nankai_3_uid58823	NC_009635	chromosome									
150401649	hypothetical protein Maeo_1227	No	0		integrated_ele	1297572	1297778	-		Methanococcus_aeolicus_Nankai_3_uid58823	NC_009635	chromosome									
150401650	hypothetical protein Maeo_1228	No	0		integrated_ele	1298065	1298451	+		Methanococcus_aeolicus_Nankai_3_uid58823	NC_009635	chromosome		arCOG01057	21 (-)	K		Predicted transcriptional regulator			
150401651	hypothetical protein Maeo_1229	No	1		integrated_ele	1298788	1299165	+		Methanococcus_aeolicus_Nankai_3_uid58823	NC_009635	chromosome									
150401652	hypothetical protein Maeo_1230	No	1		integrated_ele	1299177	1299386	+		Methanococcus_aeolicus_Nankai_3_uid58823	NC_009635	chromosome		arCOG09561	2	S		Uncharacterized conserved protein			
150401653	hypothetical protein Maeo_1231	No	2		integrated_ele	1299440	1299784	-		Methanococcus_aeolicus_Nankai_3_uid58823	NC_009635	chromosome									
150401654	hypothetical protein Maeo_1232	No	4		integrated_ele	1299794	1300270	-		Methanococcus_aeolicus_Nankai_3_uid58823	NC_009635	chromosome									
150401655	hypothetical protein Maeo_1233	No	0		integrated_ele	1300270	1300869	-		Methanococcus_aeolicus_Nankai_3_uid58823	NC_009635	chromosome		arCOG00280	21 (-)	L		HerA helicase			
150401656	hypothetical protein Maeo_1234	No	0		integrated_ele	1300875	1301120	-		Methanococcus_aeolicus_Nankai_3_uid58823	NC_009635	chromosome									
150401657	hypothetical protein Maeo_1235	No	0		integrated_ele	1301120	1301716	-		Methanococcus_aeolicus_Nankai_3_uid58823	NC_009635	chromosome									
150401658	hypothetical protein Maeo_1236	No	0		integrated_ele	1302354	1303208	+		Methanococcus_aeolicus_Nankai_3_uid58823	NC_009635	chromosome		arCOG09562	2	S		Uncharacterized conserved protein			
150401659	hypothetical protein Maeo_1237	No	2		integrated_ele	1303219	1303446	+		Methanococcus_aeolicus_Nankai_3_uid58823	NC_009635	chromosome									
150401660	hypothetical protein Maeo_1238	No	1		integrated_ele	1303451	1304311	+		Methanococcus_aeolicus_Nankai_3_uid58823	NC_009635	chromosome							0.00000006	pfam07172	Glycine rich protein family. This family
150401661	hypothetical protein Maeo_1239	No	0		integrated_ele	1304434	1304757	+		Methanococcus_aeolicus_Nankai_3_uid58823	NC_009635	chromosome		arCOG09563	2	S		Uncharacterized conserved protein			
150401662	hypothetical protein Maeo_1240	No	0		integrated_ele	1304762	1305079	+		Methanococcus_aeolicus_Nankai_3_uid58823	NC_009635	chromosome									
150401663	hypothetical protein Maeo_1241	No	0		integrated_ele	1305087	1307303	+		Methanococcus_aeolicus_Nankai_3_uid58823	NC_009635	chromosome		arCOG09564	2	S		Uncharacterized conserved protein			
150401664	hypothetical protein Maeo_1242	No	0		integrated_ele	1307308	1307772	+		Methanococcus_aeolicus_Nankai_3_uid58823	NC_009635	chromosome		arCOG09565	2	S		Uncharacterized conserved protein			
150401665	hypothetical protein Maeo_1243	No	0		integrated_ele	1307872	1308303	+		Methanococcus_aeolicus_Nankai_3_uid58823	NC_009635	chromosome									
150401666	hypothetical protein Maeo_1244	No	0		integrated_ele	1308305	1308835	+		Methanococcus_aeolicus_Nankai_3_uid58823	NC_009635	chromosome		arCOG09566	2	S		Uncharacterized conserved protein			
150401667	hypothetical protein Maeo_1245	No	0		integrated_ele	1308812	1309042	-		Methanococcus_aeolicus_Nankai_3_uid58823	NC_009635	chromosome		arCOG09567	3	S		Uncharacterized conserved protein			
150401668	hypothetical protein Maeo_1246	No	0		integrated_ele	1309082	1309270	-		Methanococcus_aeolicus_Nankai_3_uid58823	NC_009635	chromosome									

===									7	Methanococcus_maripaludis_X1_uid70729	NC_015847	chromosome									
	340624104	hypothetical protein GYY_04715	No	0			872599	874095 +		Methanococcus_maripaludis_X1_uid70729	NC_015847	chromosome		arCOG06636	2 (-)	V		PIN domain containing protein			
	340624105	hypothetical protein GYY_04720	No	3			874260	874610 +		Methanococcus_maripaludis_X1_uid70729	NC_015847	chromosome	clust_288								
	340624106	hypothetical protein GYY_04725	No	2			874644	875876 +		Methanococcus_maripaludis_X1_uid70729	NC_015847	chromosome							7E-12	COG3209	Rhs family protein
	340624107	hypothetical protein GYY_04730	Yes	3			875923	876300 +		Methanococcus_maripaludis_X1_uid70729	NC_015847	chromosome									
	340624108	hypothetical protein GYY_04735	No	0			876423	877304 +		Methanococcus_maripaludis_X1_uid70729	NC_015847	chromosome									
	340624109	hypothetical protein GYY_04740	No	0			877306	877710 +		Methanococcus_maripaludis_X1_uid70729	NC_015847	chromosome									
	340624110	coenzyme F420-reducing hydrogenase subuni	No	0			878157	879005 -		Methanococcus_maripaludis_X1_uid70729	NC_015847	chromosome		arCOG02651	1 (-)	C	FrhB	Coenzyme F420-reducing hydrogenase, beta su	2E-101	COG1035	Coenzyme F420-reducing hydrogenase, beta
===									7	Methanococcus_maripaludis_X1_uid70729	NC_015847	chromosome									
	340624266	CRISPR-associated exonuclease Cas4 family		No	0			1029361	1029990 +		Methanococcus_maripaludis_X1_uid70729	NC_015847	chromosome		arCOG00786	4 (-)	V	Cas4	CRISPR-associated protein Cas4, RecB family ex	7E-12	COG1468	CRISPR-associated protein Cas4 (RecB fam
	340624267	hypothetical protein GYY_05560	No	1			1030402	1030593 -		Methanococcus_maripaludis_X1_uid70729	NC_015847	chromosome									
	340624268	hypothetical protein GYY_05565	No	2			1031223	1031468 -		Methanococcus_maripaludis_X1_uid70729	NC_015847	chromosome	clust_399								
	340624269	hypothetical protein GYY_05570	No	2			1032130	1032345 -		Methanococcus_maripaludis_X1_uid70729	NC_015847	chromosome									
	340624270	hypothetical protein GYY_05575	No	1			1033041	1033148 -		Methanococcus_maripaludis_X1_uid70729	NC_015847	chromosome									
	340624271	hypothetical protein GYY_05580	No	2			1034065	1034310 -		Methanococcus_maripaludis_X1_uid70729	NC_015847	chromosome	clust_399								
	340624272	hypothetical protein GYY_05585	No	0			1034664	1034912 +		Methanococcus_maripaludis_X1_uid70729	NC_015847	chromosome		arCOG05071	1 (-)	S		Uncharacterized conserved protein	8E-12	pfam10884	Protein of unknown function (DUF2683). T
===									5	Methanococcus_vannielii_SB_uid58767	NC_009634	chromosome									
	150398859	hypothetical protein Mevan_0100	No	0	YES		110228	110533 -		Methanococcus_vannielii_SB_uid58767	NC_009634	chromosome									
	150398860	hypothetical protein Mevan_0102	No	0	YES		112796	113632 -		Methanococcus_vannielii_SB_uid58767	NC_009634	chromosome		arCOG09614	1	S		Uncharacterized conserved protein			
	150398861	ATPase	No	0	YES		113632	114903 -		Methanococcus_vannielii_SB_uid58767	NC_009634	chromosome		arCOG05410	1 (-)	E	DppD	ABC-type dipeptide/oligopeptide/nickel transpor	6E-25	COG3950	Predicted ATP-binding protein involved i
	150398862	hypothetical protein Mevan_0104	No	0	YES		115690	116049 +		Methanococcus_vannielii_SB_uid58767	NC_009634	chromosome									
	150398863	hypothetical protein Mevan_0105	No	0	YES		116338	117351 +		Methanococcus_vannielii_SB_uid58767	NC_009634	chromosome									
===									7	Methanococcus_vannielii_SB_uid58767	NC_009634	chromosome									
	150398874	hypothetical protein Mevan_0116	No	0	YES		127863	129716 -		Methanococcus_vannielii_SB_uid58767	NC_009634	chromosome	arCOG03233,a	arCOG03233	3 (-)	R		Predicted ATPase	0.000000002	COG4938	Uncharacterized conserved protein
	150398875	hypothetical protein Mevan_0118	No	0	YES		130561	130824 +		Methanococcus_vannielii_SB_uid58767	NC_009634	chromosome									
	150398876	hypothetical protein Mevan_0119	No	0			130858	131397 +		Methanococcus_vannielii_SB_uid58767	NC_009634	chromosome	clust_167								
	150398877	hypothetical protein Mevan_0120	No	0			131470	131727 +		Methanococcus_vannielii_SB_uid58767	NC_009634	chromosome									
	150398878	hypothetical protein Mevan_0121	No	0			131767	132243 +		Methanococcus_vannielii_SB_uid58767	NC_009634	chromosome									
	150398879	hypothetical protein Mevan_0122	No	0			132532	133146 +		Methanococcus_vannielii_SB_uid58767	NC_009634	chromosome	clust_167								
	150398880	SMF family protein	No	0			133136	134194 +		Methanococcus_vannielii_SB_uid58767	NC_009634	chromosome		arCOG02431	4 (-)	R		Predicted Rossmann fold nucleotide-binding prot	2E-31	pfam02481	DNA recombination-mediator protein A. Th
===									6	Methanococcus_vannielii_SB_uid58767	NC_009634	chromosome									
	150400375	hypothetical protein Mevan_1638	No	0		integrated_ele	1655477	1655968 -		Methanococcus_vannielii_SB_uid58767	NC_009634	chromosome		arCOG09540	4	S		Uncharacterized conserved protein			
	150400376	KAP P-loop domain-containing protein	No	0		integrated_ele	1656608	1659196 -		Methanococcus_vannielii_SB_uid58767	NC_009634	chromosome							4E-24	pfam07693	KAP family P-loop domain. The KAP (after
	150400377	MCM family DNA replication protein	No	0		integrated_ele	1659224	1659964 -		Methanococcus_vannielii_SB_uid58767	NC_009634	chromosome		arCOG00439	14 (-)	L	MCM2	Predicted ATPase involved in replication control,	9E-10	COG1241	Predicted ATPase involved in replication
	150400378	hypothetical protein Mevan_1641	No	0		integrated_ele	1660094	1661077 -		Methanococcus_vannielii_SB_uid58767	NC_009634	chromosome									
	150400379	hypothetical protein Mevan_1642	No	0		integrated_ele	1661369	1661737 +		Methanococcus_vannielii_SB_uid58767	NC_009634	chromosome									
	150400380	hypothetical protein Mevan_1643	No	0		integrated_ele	1661873	1663159 -		Methanococcus_vannielii_SB_uid58767	NC_009634	chromosome									
===									17	Methanococcus_voltae_A3_uid49529	NC_014222	chromosome									
	297618535	integrase family protein	No	0		integrated_ele	4634	5296 -		Methanococcus_voltae_A3_uid49529	NC_014222	chromosome		arCOG01241	31 (-)	L	XerC	Integrase	2E-15	pfam00589	Phage integrase family. Members of this
	297618536	hypothetical protein Mvol_0008	No	0		integrated_ele	5689	5919 +		Methanococcus_voltae_A3_uid49529	NC_014222	chromosome									
	297618537	hypothetical protein Mvol_0009	No	0		integrated_ele	5979	7358 +		Methanococcus_voltae_A3_uid49529	NC_014222	chromosome		arCOG06786	9	Y		Rolling circle replication protein Rep			
	297618538	hypothetical protein Mvol_0010	No	0		integrated_ele	7379	7693 +		Methanococcus_voltae_A3_uid49529	NC_014222	chromosome	clust_339								
	297618539	hypothetical protein Mvol_0011	No	0		integrated_ele	7817	8281 +		Methanococcus_voltae_A3_uid49529	NC_014222	chromosome	clust_225								
	297618540	hypothetical protein Mvol_0012	No	0		integrated_ele	8271	9095 +		Methanococcus_voltae_A3_uid49529	NC_014222	chromosome		arCOG06224	4 (-)	L		HerA helicase			
	297618541	hypothetical protein Mvol_0013	No	0		integrated_ele	9251	9562 +		Methanococcus_voltae_A3_uid49529	NC_014222	chromosome	clust_343								
	297618542	hypothetical protein Mvol_0014	No	0		integrated_ele	9572	10030 +		Methanococcus_voltae_A3_uid49529	NC_014222	chromosome	clust_231								
	297618543	hypothetical protein Mvol_0015	No	0		integrated_ele	10032	10436 +		Methanococcus_voltae_A3_uid49529	NC_014222	chromosome	clust_260								
	297618544	hypothetical protein Mvol_0016	Yes	0		integrated_ele	10449	10877 +		Methanococcus_voltae_A3_uid49529	NC_014222	chromosome	clust_243						0.00001	pfam01456	Mucin-like glycoprotein. This family of
	297618545	hypothetical protein Mvol_0017	Yes	0		integrated_ele	10870	11427 +		Methanococcus_voltae_A3_uid49529	NC_014222	chromosome	clust_188								
	297618546	hypothetical protein Mvol_0018	No	0		integrated_ele	11424	11561 +		Methanococcus_voltae_A3_uid49529	NC_014222	chromosome	clust_503								
	297618547	hypothetical protein Mvol_0019	No	0		integrated_ele	11570	11785 +		Methanococcus_voltae_A3_uid49529	NC_014222	chromosome	clust_435								
	297618548	hypothetical protein Mvol_0020	No	0		integrated_ele	11782	12360 +		Methanococcus_voltae_A3_uid49529	NC_014222	chromosome	clust_181								
	297618549	hypothetical protein Mvol_0021	No	0		integrated_ele	12357	12464 +		Methanococcus_voltae_A3_uid49529	NC_014222	chromosome	clust_513								
	297618550	hypothetical protein Mvol_0022	Yes	0		integrated_ele	12586	12870 +		Methanococcus_voltae_A3_uid49529	NC_014222	chromosome		arCOG10939	2	S		Uncharacterized conserved protein	0.00002	pfam11259	Protein of unknown function (DUF3060). S
	297618551	glycine betaine ABC transporter substrate-bind	Yes	1		integrated_ele	13680	14600 -		Methanococcus_voltae_A3_uid49529	NC_014222	chromosome		arCOG02310	2 (-)	E	ProX	ABC-type proline/glycine betaine transport syster	2E-50	COG2113	ABC-type proline/glycine betaine transpo
===									15	Methanococcus_voltae_A3_uid49529	NC_014222	chromosome									
	297618597	hypothetical protein Mvol_0069	No	0			70559	71614 +		Methanococcus_voltae_A3_uid49529	NC_014222	chromosome		arCOG06786	9	Y		Rolling circle replication protein Rep			
	297618598	hypothetical protein Mvol_0070	No	0			71635	71949 +		Methanococcus_voltae_A3_uid49529	NC_014222	chromosome	clust_339								
	297618599	hypothetical protein Mvol_0071	No	0			72073	72537 +		Methanococcus_voltae_A3_uid49529	NC_014222	chromosome	clust_225								
	297618600	hypothetical protein Mvol_0072	No	0			72527	73351 +		Methanococcus_voltae_A3_uid49529	NC_014222	chromosome		arCOG06224	4 (-)	L		HerA helicase			
	297618601	hypothetical protein Mvol_0073	No	0			73507	73818 +		Methanococcus_voltae_A3_uid49529	NC_014222	chromosome	clust_343								
	297618602	hypothetical protein Mvol_0074	No	0			73828	74286 +		Methanococcus_voltae_A3_uid49529	NC_014222	chromosome	clust_231								
	297618603	hypothetical protein Mvol_0075	No	0			74288	74692 +		Methanococcus_voltae_A3_uid49529	NC_014222	chromosome	clust_260								
	297618604	hypothetical protein Mvol_0076	Yes	0			74705	75133 +		Methanococcus_voltae_A3_uid49529	NC_014222	chromosome	clust_243						0.00001	pfam01456	Mucin-like glycoprotein. This family of
	297618605	hypothetical protein Mvol_0077	Yes	0			75126	75683 +		Methanococcus_voltae_A3_uid49529	NC_014222	chromosome	clust_188								
	297618606	hypothetical protein Mvol_0078	No	0			75680	75817 +		Methanococcus_voltae_A3_uid49529	NC_014222	chromosome	clust_503								
	297618607	hypothetical protein Mvol_0079	No	0			75826	76041 +		Methanococcus_voltae_A3_uid49529	NC_014222	chromosome	clust_435								
	297618608	hypothetical protein Mvol_0080	No	0			76038	76616 +		Methanococcus_voltae_A3_uid49529	NC_014222	chromosome	clust_181								
	297618609	hypothetical protein Mvol_0081	No	0			76613	76720 +		Methanococcus_voltae_A3_uid49529	NC_014222	chromosome	clust_513								
	297618610	hypothetical protein Mvol_0082	Yes	0			76842	77126 +		Methanococcus_voltae_A3_uid49529	NC_014222	chromosome		arCOG10939	2	S		Uncharacterized conserved protein	0.00002	pfam11259	Protein of unknown function (DUF3060). S
	297618611	glycine betaine ABC transporter substrate-bind	Yes	1			77936	78856 -		Methanococcus_voltae_A3_uid49529	NC_014222	chromosome		arCOG02310	2 (-)	E	ProX	ABC-type proline/glycine betaine transport syster	2E-50	COG2113	ABC-type proline/glycine betaine transpo
===									11	Methanococcus_voltae_A3_uid49529	NC_014222	chromosome									
	297618825	hypothetical protein Mvol_0297	No	0			324793	325245 +		Methanococcus_voltae_A3_uid49529	NC_014222	chromosome		arCOG09529	2	S		Uncharacterized conserved protein			
	297618826	hypothetical protein Mvol_0298	No	0			325255	325668 +		Methanococcus_voltae_A3_uid49529	NC_014222	chromosome		arCOG09528	2	S		Uncharacterized conserved protein			
	297618827	hypothetical protein Mvol_0299	No	0			325697	327037 +		Methanococcus_voltae_A3_uid49529	NC_014222	chromosome		arCOG07665	6 (-)	S		Uncharacterized conserved protein			
	297618828	hypothetical protein Mvol_0300	No	0			327015	327350 +		Methanococcus_voltae_A3_uid49529	NC_014222	chromosome		arCOG09524	6	S		Uncharacterized conserved protein			
	297618829	hypothetical protein Mvol_0301	No	0			327531	327788 +		Methanococcus_voltae_A3_uid49529	NC_014222	chromosome									
	297618830	hypothetical protein Mvol_0302	No	0			327790	328224 +		Methanococcus_voltae_A3_uid49529	NC_014222	chromosome									
	297618831	hypothetical protein Mvol_0303	No	0			328237	328602 +		Methanococcus_voltae_A3_uid49529	NC_014222	chromosome									
	297618832	hypothetical protein Mvol_0304	No	0			328611	328913 +		Methanococcus_voltae_A3_uid49529	NC_014222	chromosome									
	297618833	hypothetical protein Mvol_0305	No	0			329122	329403 +		Methanococcus_voltae_A3_uid49529	NC_014222	chromosome									
	297618834	hypothetical protein Mvol_0306	Yes	3			329413	330753 +		Methanococcus_voltae_A3_uid49529	NC_014222	chromosome		arCOG09523	5 (-)	S		Uncharacterized conserved protein			
	297618835	hypothetical protein Mvol_0307	No	0			330734	330958 +		Methanococcus_voltae_A3_uid49529	NC_014222	chromosome									
===									6	Methanococcus_voltae_A3_uid49529	NC_014222	chromosome									
	297618837	KAP P-loop domain-containing protein	No	0			331509	333812 -		Methanococcus_voltae_A3_uid49529	NC_014222	chromosome		arCOG10127	2 (-)	R		KAP-like ATPase	2E-21	pfam07693	KAP family P-loop domain. The KAP (after ; Mitochondrial ribosomal protein (VAR1). ; Borrelia ORF-A. This protein is encoded
	297618838	hypothetical protein Mvol_0310	No	0			333971	334201 -		Methanococcus_voltae_A3_uid49529	NC_014222	chromosome									
	297618839	hypothetical protein Mvol_0311	No	4			334367	334816 +		Methanococcus_voltae_A3_uid49529	NC_014222	chromosome		arCOG11026	3	S		Uncharacterized conserved protein			
	297618840	hypothetical protein Mvol_0312	No	4			334878	335237 +		Methanococcus_voltae_A3_uid49529	NC_014222	chromosome		arCOG08251	4	S		Uncharacterized conserved protein			
	297618841	hypothetical protein Mvol_0313	Yes	2			335251	335841 +		Methanococcus_voltae_A3_uid49529	NC_014222	chromosome		arCOG11226	2	S		Uncharacterized conserved protein			
	297618842	hypothetical protein Mvol_0314	Yes	2			335848	337710 +		Methanococcus_voltae_A3_uid49529	NC_014222	chromosome		arCOG06635	3 (-)	S		Uncharacterized conserved protein			
===									9	Methanococcus_voltae_A3_uid49529	NC_014222	chromosome									
	297618847	restriction modification system DNA specificity	No	0			350232	351470 -		Methanococcus_voltae_A3_uid49529	NC_014222	chromosome		arCOG02626	3 (-)	V	HsdS	Restriction endonuclease S subunit	2E-34	COG0732	Restriction endonuclease S subunits
	297618848	hypothetical protein Mvol_0320	No	0			351483	351638 -		Methanococcus_voltae_A3_uid49529	NC_014222	chromosome									
	297618849	hypothetical protein Mvol_0321	No	0			351891	352571 -		Methanococcus_voltae_A3_uid49529	NC_014222	chromosome									
	297618850	hypothetical protein Mvol_0322	No	0			352686	353024 -		Methanococcus_voltae_A3_uid49529	NC_014222	chromosome									
	297618851	hypothetical protein Mvol_0323	No	0			353110	353595 -		Methanococcus_voltae_A3_uid49529	NC_014222	chromosome									
	297618852	hypothetical protein Mvol_0324	No	0			353694	353936 -		Methanococcus_voltae_A3_uid49529	NC_014222	chromosome									
	297618853	hypothetical protein Mvol_0325	No	0			354870	355514 +		Methanococcus_voltae_A3_uid49529	NC_014222	chromosome		arCOG09533	1	S		Uncharacterized conserved protein			
	297618854	integrase family protein	No	0			355551	356438 +		Methanococcus_voltae_A3_uid49529	NC_014222	chromosome		arCOG01241	31 (-)	L	XerC	Integrase	7E-24	COG4973	Site-specific recombinase XerC
	297618855	hypothetical protein Mvol_0327	No	1			356529	356717 +		Methanococcus_voltae_A3_uid49529	NC_014222	chromosome									
===									16	Methanococcus_voltae_A3_uid49529	NC_014222	chromosome									
	297618929	hypothetical protein Mvol_0401	No	0		Virus_Caudovi	434422	435375 -		Methanococcus_voltae_A3_uid49529	NC_014222	chromosome									
	297618930	hypothetical protein Mvol_0402	No	0		Virus_Caudovi	435445	436302 -		Methanococcus_voltae_A3_uid49529	NC_014222	chromosome									
	297618931	hypothetical protein Mvol_0403	No	0		Virus_Caudovi	436363	439185 -		Methanococcus_voltae_A3_uid49529	NC_014222	chromosome		arCOG04500	1 (-)	S		Uncharacterized conserved protein			
	297618932	hypothetical protein Mvol_0404	No	0		Virus_Caudovi	439296	440000 -		Methanococcus_voltae_A3_uid49529	NC_014222	chromosome		arCOG09645	3	Y		Predicted phage protein			
	297618933	hypothetical protein Mvol_0405	No	0		Virus_Caudovi	440052	440393 -		Methanococcus_voltae_A3_uid49529	NC_014222	chromosome									
	297618934	hypothetical protein Mvol_0406	No	0		Virus_Caudovi	440386	440682 -		Methanococcus_voltae_A3_uid49529	NC_014222	chromosome		arCOG09643	3	Y		Predicted phage protein			
	297618935	hypothetical protein Mvol_0407	No	0		Virus_Caudovi	440744	441517 -		Methanococcus_voltae_A3_uid49529	NC_014222	chromosome		arCOG09642	3	Y		Predicted phage protein			
	297618936	hypothetical protein Mvol_0408	No	0		Virus_Caudovi	441673	442011 -		Methanococcus_voltae_A3_uid49529	NC_014222	chromosome		arCOG09641	3	Y		Predicted phage protein			
	297618937	hypothetical protein Mvol_0409	No	0		Virus_Caudovi	442146	442892 -		Methanococcus_voltae_A3_uid49529	NC_014222	chromosome		arCOG09508	6	Y		Phage prohead protease			
	297618938	hypothetical protein Mvol_0410	No	0		Virus_Caudovi	443046	443285 -		Methanococcus_voltae_A3_uid49529	NC_014222	chromosome									
	297618939	hypothetical protein Mvol_0411	No	2		Virus_Caudovi	443627	444061 +		Methanococcus_voltae_A3_uid49529	NC_014222	chromosome									
	297618940	hypothetical protein Mvol_0412	No	0		Virus_Caudovi	444360	444737 +		Methanococcus_voltae_A3_uid49529	NC_014222	chromosome		arCOG09509	3	S		Uncharacterized conserved protein			
	297618941	hypothetical protein Mvol_0413	No	0		Virus_Caudovi	444827	446557 +		Methanococcus_voltae_A3_uid49529	NC_014222	chromosome	arCOG09550	arCOG09550	17	Y	TerL	Phage terminase large subunit			
	297618942	hypothetical protein Mvol_0414	No	0		Virus_Caudovi	446620	448539 +		Methanococcus_voltae_A3_uid49529	NC_014222	chromosome		arCOG09636	4	Y		Phage protein containing C-terminal domain related to phage head morphogenesis Mu gpF-like			
	297618943	hypothetical protein Mvol_0415	No	0		Virus_Caudovi	448542	449054 +		Methanococcus_voltae_A3_uid49529	NC_014222	chromosome							4E-11	pfam05316	Mitochondrial ribosomal protein (VAR1).
	297618944	aldo/keto reductase	No	0		Virus_Caudovi	449069	450313 -		Methanococcus_voltae_A3_uid49529	NC_014222	chromosome		arCOG01624	3 (-)	R		Predicted oxidoreductase of the aldo/keto reduc	1E-127	COG1453	Predicted oxidoreductases of the aldo/ke
===									56	Methanococcus_voltae_A3_uid49529	NC_014222	chromosome									
	297618973	hypothetical protein Mvol_0445	No	0		Virus_Turrivirid	480602	482083 -		Methanococcus_voltae_A3_uid49529	NC_014222	chromosome		arCOG06786	9 (-)	Y		Rolling circle replication protein Rep			
	297618974	hypothetical protein Mvol_0446	No	1		Virus_Turrivirid	482185	482379 -		Methanococcus_voltae_A3_uid49529	NC_014222	chromosome									
	297618975	hypothetical protein Mvol_0447	No	0		Virus_Turrivirid	482383	482715 -		Methanococcus_voltae_A3_uid49529	NC_014222	chromosome	clust_315								
	297618976	hypothetical protein Mvol_0448	No	0		Virus_Turrivirid	482717	482995 -		Methanococcus_voltae_A3_uid49529	NC_014222	chromosome									
	297618977	hypothetical protein Mvol_0449	No	0		Virus_Turrivirid	482992	483129 -		Methanococcus_voltae_A3_uid49529	NC_014222	chromosome									
	297618978	hypothetical protein Mvol_0450	No	0		Virus_Turrivirid	483133	483351 -		Methanococcus_voltae_A3_uid49529	NC_014222	chromosome									
	297618979	hypothetical protein Mvol_0451	No	0		Virus_Turrivirid	483348	483569 -		Methanococcus_voltae_A3_uid49529	NC_014222	chromosome									
	297618980	hypothetical protein Mvol_0452	No	0		Virus_Turrivirid	483566	483676 -		Methanococcus_voltae_A3_uid49529	NC_014222	chromosome									
	297618981	MarR family transcriptional regulator	No	0		Virus_Turrivirid	484094	484504 +		Methanococcus_voltae_A3_uid49529	NC_014222	chromosome		arCOG01057	21 (-)	K		Predicted transcriptional regulator	0.00002	COG1846	Transcriptional regulators
	297618982	hypothetical protein Mvol_0454	No	0		Virus_Turrivirid	484703	484828 -		Methanococcus_voltae_A3_uid49529	NC_014222	chromosome									
	297618983	hypothetical protein Mvol_0455	No	0		Virus_Turrivirid	484825	484926 -		Methanococcus_voltae_A3_uid49529	NC_014222	chromosome									
	297618984	hypothetical protein Mvol_0456	No	0		Virus_Turrivirid	484923	485474 -		Methanococcus_voltae_A3_uid49529	NC_014222	chromosome									
	297618985	outer membrane protein, hemagglutinin-like pro	No	0		Virus_Turrivirid	485471	485737 -		Methanococcus_voltae_A3_uid49529	NC_014222	chromosome									
	297618986	hypothetical protein Mvol_0458	No	0		Virus_Turrivirid	485751	486389 -		Methanococcus_voltae_A3_uid49529	NC_014222	chromosome									
	297618987	hypothetical protein Mvol_0459	No	0		Virus_Turrivirid	486401	486676 -		Methanococcus_voltae_A3_uid49529	NC_014222	chromosome									
	297618988	hypothetical protein Mvol_0460	No	1		Virus_Turrivirid	486729	487856 -		Methanococcus_voltae_A3_uid49529	NC_014222	chromosome									
	297618989	hypothetical protein Mvol_0461	No	2		Virus_Turrivirid	487859	488089 -		Methanococcus_voltae_A3_uid49529	NC_014222	chromosome									
	297618990	hypothetical protein Mvol_0462	No	2		Virus_Turrivirid	488091	488354 -		Methanococcus_voltae_A3_uid49529	NC_014222	chromosome									
	297618991	hypothetical protein Mvol_0463	No	0		Virus_Turrivirid	488365	489390 -		Methanococcus_voltae_A3_uid49529	NC_014222	chromosome		arCOG10335	5 (-)	Y		Major capsid protein, double jelly roll fold protein			
	297618992	hypothetical protein Mvol_0464	No	0		Virus_Turrivirid	489678	490253 -		Methanococcus_voltae_A3_uid49529	NC_014222	chromosome		arCOG06224	4 (-)	L		HerA helicase			
	297618993	hypothetical protein Mvol_0465	No	0		Virus_Turrivirid	490253	490492 -		Methanococcus_voltae_A3_uid49529	NC_014222	chromosome									
	297618994	hypothetical protein Mvol_0466	No	0		Virus_Turrivirid	490470	491195 -		Methanococcus_voltae_A3_uid49529	NC_014222	chromosome							6E-13	COG0419	ATPase involved in DNA repair
	297618995	hypothetical protein Mvol_0467	No	0		Virus_Turrivirid	491185	491376 -		Methanococcus_voltae_A3_uid49529	NC_014222	chromosome									
	297618996	hypothetical protein Mvol_0468	No	2		Virus_Turrivirid	491797	492387 -		Methanococcus_voltae_A3_uid49529	NC_014222	chromosome									
	297618997	hypothetical protein Mvol_0469	No	0		Virus_Turrivirid	492389	493300 -		Methanococcus_voltae_A3_uid49529	NC_014222	chromosome									
	297618998	hypothetical protein Mvol_0470	No	0		Virus_Turrivirid	493939	496938 -		Methanococcus_voltae_A3_uid49529	NC_014222	chromosome	arCOG03013,a	arCOG03013	2 (-)	L	PRI2	Eukaryotic-type DNA primase, large subunit			
	297618999	integrase family protein	No	0		Virus_Turrivirid	496946	497899 -		Methanococcus_voltae_A3_uid49529	NC_014222	chromosome		arCOG01241	31 (-)	L	XerC	Integrase	3E-16	pfam00589	Phage integrase family. Members of this ; Phage integrase, N-terminal SAM-like dom
	297619000	hypothetical protein Mvol_0472	No	0		Virus_Turrivirid	497966	498211 -		Methanococcus_voltae_A3_uid49529	NC_014222	chromosome									
	297619001	hypothetical protein Mvol_0473	No	0		Virus_Turrivirid	498213	498671 -		Methanococcus_voltae_A3_uid49529	NC_014222	chromosome									
	297619002	hypothetical protein Mvol_0474	No	0		Virus_Turrivirid	498655	498981 -		Methanococcus_voltae_A3_uid49529	NC_014222	chromosome									
	297619003	hypothetical protein Mvol_0475	No	0		Virus_Turrivirid	498981	499475 -		Methanococcus_voltae_A3_uid49529	NC_014222	chromosome									
	297619004	hypothetical protein Mvol_0476	No	0		Virus_Turrivirid	499477	499650 -		Methanococcus_voltae_A3_uid49529	NC_014222	chromosome	clust_485								
	297619005	hypothetical protein Mvol_0477	No	0		Virus_Turrivirid	499652	499843 -		Methanococcus_voltae_A3_uid49529	NC_014222	chromosome	clust_61								
	297619006	hypothetical protein Mvol_0478	No	0		Virus_Turrivirid	500053	500469 +		Methanococcus_voltae_A3_uid49529	NC_014222	chromosome		arCOG01057	21 (-)	K		Predicted transcriptional regulator			
	297619007	hypothetical protein Mvol_0479	No	0		Virus_Turrivirid	500658	500789 +		Methanococcus_voltae_A3_uid49529	NC_014222	chromosome									
	297619008	hypothetical protein Mvol_0480	No	2		Virus_Turrivirid	500942	501196 +		Methanococcus_voltae_A3_uid49529	NC_014222	chromosome									
	297619009	Resolvase domain-containing protein	No	0		Virus_Turrivirid	501219	501767 -		Methanococcus_voltae_A3_uid49529	NC_014222	chromosome		arCOG03162	12 (-)	L	PinR	Site-specific recombinase, DNA invertase Pin ho	1E-36	COG1961	Site-specific recombinases, DNA invertas
	297619010	hypothetical protein Mvol_0482	No	0		Virus_Turrivirid	501995	502132 -		Methanococcus_voltae_A3_uid49529	NC_014222	chromosome									
	297619011	hypothetical protein Mvol_0483	No	0		Virus_Turrivirid	502141	502983 -		Methanococcus_voltae_A3_uid49529	NC_014222	chromosome									
	297619012	hypothetical protein Mvol_0484	No	0		Virus_Turrivirid	503116	506835 -		Methanococcus_voltae_A3_uid49529	NC_014222	chromosome									
	297619013	hypothetical protein Mvol_0485	No	3		Virus_Turrivirid	506847	507170 -		Methanococcus_voltae_A3_uid49529	NC_014222	chromosome									
	297619014	hypothetical protein Mvol_0486	Yes	3		Virus_Turrivirid	507170	507544 -		Methanococcus_voltae_A3_uid49529	NC_014222	chromosome									
	297619015	PKD domain-containing protein	Yes	3		Virus_Turrivirid	507537	509717 -		Methanococcus_voltae_A3_uid49529	NC_014222	chromosome		arCOG02508	1 (-)	S		PKD repeats containing protein	0.000008	pfam00801	PKD domain. This domain was first identi ; Periplasmic copper-binding protein (NosD
	297619016	hypothetical protein Mvol_0488	No	2		Virus_Turrivirid	509719	509991 -		Methanococcus_voltae_A3_uid49529	NC_014222	chromosome									
	297619017	hypothetical protein Mvol_0489	No	5		Virus_Turrivirid	509996	510397 -		Methanococcus_voltae_A3_uid49529	NC_014222	chromosome									
	297619018	hypothetical protein Mvol_0490	No	4		Virus_Turrivirid	510400	510861 -		Methanococcus_voltae_A3_uid49529	NC_014222	chromosome									
	297619019	hypothetical protein Mvol_0491	No	0		Virus_Turrivirid	510874	511107 -		Methanococcus_voltae_A3_uid49529	NC_014222	chromosome									
	297619020	hypothetical protein Mvol_0492	No	0		Virus_Turrivirid	511129	511641 -		Methanococcus_voltae_A3_uid49529	NC_014222	chromosome							0.0000006	COG0740	Protease subunit of ATP-dependent Clp pr
	297619021	hypothetical protein Mvol_0493	No	2		Virus_Turrivirid	511642	511917 -		Methanococcus_voltae_A3_uid49529	NC_014222	chromosome									
	297619022	hypothetical protein Mvol_0494	No	2		Virus_Turrivirid	511926	513179 -		Methanococcus_voltae_A3_uid49529	NC_014222	chromosome									
	297619023	hypothetical protein Mvol_0495	No	0		Virus_Turrivirid	513190	513744 -		Methanococcus_voltae_A3_uid49529	NC_014222	chromosome									
	297619024	hypothetical protein Mvol_0496	No	3		Virus_Turrivirid	513750	514013 -		Methanococcus_voltae_A3_uid49529	NC_014222	chromosome									
	297619025	hypothetical protein Mvol_0497	No	2		Virus_Turrivirid	514101	514463 -		Methanococcus_voltae_A3_uid49529	NC_014222	chromosome									
	297619026	hypothetical protein Mvol_0498	No	0		Virus_Turrivirid	514584	515807 -		Methanococcus_voltae_A3_uid49529	NC_014222	chromosome		arCOG00441	3 (-)	R		MoxR-like ATPase			
	297619027	hypothetical protein Mvol_0499	No	0		Virus_Turrivirid	515958	516380 -		Methanococcus_voltae_A3_uid49529	NC_014222	chromosome									
	297619028	hypothetical protein Mvol_0500	No	0		Virus_Turrivirid	516380	516904 -		Methanococcus_voltae_A3_uid49529	NC_014222	chromosome									
===									11	Methanococcus_voltae_A3_uid49529	NC_014222	chromosome									
	297619031	integrase family protein	No	0			521285	522238 -		Methanococcus_voltae_A3_uid49529	NC_014222	chromosome		arCOG01241	31 (-)	L	XerC	Integrase	1E-15	pfam00589	Phage integrase family. Members of this ; Phage integrase, N-terminal SAM-like dom
	297619032	hypothetical protein Mvol_0504	No	0			522270	522719 -		Methanococcus_voltae_A3_uid49529	NC_014222	chromosome									
	297619033	hypothetical protein Mvol_0505	No	0			522740	523021 -		Methanococcus_voltae_A3_uid49529	NC_014222	chromosome	clust_315								
	297619034	hypothetical protein Mvol_0506	No	0			523011	523259 -		Methanococcus_voltae_A3_uid49529	NC_014222	chromosome									
	297619035	hypothetical protein Mvol_0507	No	0			523273	523482 -		Methanococcus_voltae_A3_uid49529	NC_014222	chromosome									
	297619036	hypothetical protein Mvol_0508	No	0			523479	523817 -		Methanococcus_voltae_A3_uid49529	NC_014222	chromosome									
	297619037	hypothetical protein Mvol_0509	No	0			523844	524005 -		Methanococcus_voltae_A3_uid49529	NC_014222	chromosome	clust_492						0.0001	COG3466	Putative transposon-encoded protein
	297619038	hypothetical protein Mvol_0510	No	0			524005	524190 -		Methanococcus_voltae_A3_uid49529	NC_014222	chromosome	clust_61								
	297619039	hypothetical protein Mvol_0511	No	0			524364	524759 +		Methanococcus_voltae_A3_uid49529	NC_014222	chromosome		arCOG01057	21 (-)	K		Predicted transcriptional regulator			
	297619040	hypothetical protein Mvol_0512	No	0			524954	525130 +		Methanococcus_voltae_A3_uid49529	NC_014222	chromosome									
	297619041	hypothetical protein Mvol_0513	No	0			525140	525565 +		Methanococcus_voltae_A3_uid49529	NC_014222	chromosome		arCOG09538	1	S		Uncharacterized conserved protein			
===									5	Methanococcus_voltae_A3_uid49529	NC_014222	chromosome									
	297620057	RelA/SpoT domain-containing protein	No	0			1729444	1730466 -		Methanococcus_voltae_A3_uid49529	NC_014222	chromosome		arCOG10830	3	S		Uncharacterized conserved protein	0.00000004	COG2357	PpGpp synthetase catalytic domain
	297620058	hypothetical protein Mvol_1535	No	0			1730481	1731671 -		Methanococcus_voltae_A3_uid49529	NC_014222	chromosome									
	297620059	hypothetical protein Mvol_1536	No	0			1731771	1732241 -		Methanococcus_voltae_A3_uid49529	NC_014222	chromosome									
	297620060	hypothetical protein Mvol_1537	No	2			1732767	1733144 -		Methanococcus_voltae_A3_uid49529	NC_014222	chromosome		arCOG06644	1 (-)	S		Uncharacterized conserved protein			
	297620061	hypothetical protein Mvol_1538	No	0			1733310	1733636 -		Methanococcus_voltae_A3_uid49529	NC_014222	chromosome									
===									6	Methanococcus_voltae_A3_uid49529	NC_014222	chromosome									
	297620063	hypothetical protein Mvol_1540	No	0			1735361	1735744 -		Methanococcus_voltae_A3_uid49529	NC_014222	chromosome		arCOG01057	21 (-)	K		Predicted transcriptional regulator	0.00002	COG3432	Predicted transcriptional regulator
	297620064	hypothetical protein Mvol_1541	No	0			1735913	1736098 +		Methanococcus_voltae_A3_uid49529	NC_014222	chromosome	clust_61								
	297620065	hypothetical protein Mvol_1542	No	0			1736098	1736259 +		Methanococcus_voltae_A3_uid49529	NC_014222	chromosome	clust_492								
	297620066	hypothetical protein Mvol_1543	No	0			1736273	1736443 +		Methanococcus_voltae_A3_uid49529	NC_014222	chromosome	clust_485								
	297620067	hypothetical protein Mvol_1544	No	0			1736445	1736702 +		Methanococcus_voltae_A3_uid49529	NC_014222	chromosome									
	297620068	hypothetical protein Mvol_1545	No	0			1736772	1737017 +		Methanococcus_voltae_A3_uid49529	NC_014222	chromosome		arCOG05030	2 (-)	S		Uncharacterized conserved protein	1E-17	pfam10802	Protein of unknown function (DUF2540). T
===									5	Methanocorpusculum_labreanum_Z_uid58785	NC_008942	chromosome									
	124484858	serine/arginine repetitive matrix 2	No	1	YES		33106	33744 -		Methanocorpusculum_labreanum_Z_uid58785	NC_008942	chromosome									
	124484859	hypothetical protein Mlab_0030	No	0	YES		34606	34893 -		Methanocorpusculum_labreanum_Z_uid58785	NC_008942	chromosome									
	124484860	hypothetical protein Mlab_0031	No	1	YES		35027	35386 -		Methanocorpusculum_labreanum_Z_uid58785	NC_008942	chromosome									
	124484861	hypothetical protein Mlab_0032	No	0			35450	36055 -		Methanocorpusculum_labreanum_Z_uid58785	NC_008942	chromosome		arCOG01848	1 (-)	R	WbbJ	Acetyltransferase (isoleucine patch superfamily)	2E-21	COG0110	Acetyltransferase (isoleucine patch supe
	124484862	hypothetical protein Mlab_0033	No	0	YES		36164	38395 -		Methanocorpusculum_labreanum_Z_uid58785	NC_008942	chromosome							2E-152	pfam13166	AAA domain. This family of domains conta
===									8	Methanocorpusculum_labreanum_Z_uid58785	NC_008942	chromosome									
	124485514	hypothetical protein Mlab_0691	Yes	1			664371	666065 -		Methanocorpusculum_labreanum_Z_uid58785	NC_008942	chromosome		arCOG03642	1 (-)	N	Tar	Methyl-accepting chemotaxis protein	6E-13	COG4564	Signal transduction histidine kinase
	124485515	hypothetical protein Mlab_0692	No	0			666181	666516 +		Methanocorpusculum_labreanum_Z_uid58785	NC_008942	chromosome									
	124485516	hypothetical protein Mlab_0693	No	0			668019	668384 +		Methanocorpusculum_labreanum_Z_uid58785	NC_008942	chromosome	clust_44								
	124485517	hypothetical protein Mlab_0694	No	0			668494	668748 +		Methanocorpusculum_labreanum_Z_uid58785	NC_008942	chromosome									
	124485518	hypothetical protein Mlab_0695	No	0			668745	669272 +		Methanocorpusculum_labreanum_Z_uid58785	NC_008942	chromosome	clust_17								
	124485519	hypothetical protein Mlab_0696	No	0			669269	669529 +		Methanocorpusculum_labreanum_Z_uid58785	NC_008942	chromosome	clust_53								
	124485520	hypothetical protein Mlab_0697	No	0			669516	670016 +		Methanocorpusculum_labreanum_Z_uid58785	NC_008942	chromosome	clust_38								
	124485521	hypothetical protein Mlab_0698	No	0			670420	671064 +		Methanocorpusculum_labreanum_Z_uid58785	NC_008942	chromosome		arCOG06904	7 (-)	S		Uncharacterized conserved protein			
===									5	Methanocorpusculum_labreanum_Z_uid58785	NC_008942	chromosome									
	124485629	hypothetical protein Mlab_0807	No	0			788622	789125 -		Methanocorpusculum_labreanum_Z_uid58785	NC_008942	chromosome									
	124485630	hypothetical protein Mlab_0808	No	0			789285	789923 -		Methanocorpusculum_labreanum_Z_uid58785	NC_008942	chromosome		arCOG00769	2 (-)	R	ThiJ	Putative intracellular protease/amidase	0.000000006	pfam13278	Putative amidotransferase. This domain c
	124485631	hypothetical protein Mlab_0809	Yes	1			790085	790837 +		Methanocorpusculum_labreanum_Z_uid58785	NC_008942	chromosome									
	124485632	hypothetical protein Mlab_0810	Yes	1			790842	791423 +		Methanocorpusculum_labreanum_Z_uid58785	NC_008942	chromosome									
	124485633	hypothetical protein Mlab_0811	No	0			791440	792645 -		Methanocorpusculum_labreanum_Z_uid58785	NC_008942	chromosome									
===									6	Methanocorpusculum_labreanum_Z_uid58785	NC_008942	chromosome									
	124485725	hypothetical protein Mlab_0903	No	2			895653	896528 -		Methanocorpusculum_labreanum_Z_uid58785	NC_008942	chromosome		arCOG00894	2 (-)	M	WcaA	Glycosyltransferase	0.000005	COG0463	Glycosyltransferases involved in cell wa
	124485726	hypothetical protein Mlab_0904	No	0	YES		897521	897973 +		Methanocorpusculum_labreanum_Z_uid58785	NC_008942	chromosome									
	124485727	hypothetical protein Mlab_0905	No	0	YES		899637	900131 +		Methanocorpusculum_labreanum_Z_uid58785	NC_008942	chromosome	clust_216								
	124485728	hypothetical protein Mlab_0906	No	0	YES		900128	900394 +		Methanocorpusculum_labreanum_Z_uid58785	NC_008942	chromosome									
	124485729	hypothetical protein Mlab_0907	No	0	YES		900391	900879 +		Methanocorpusculum_labreanum_Z_uid58785	NC_008942	chromosome	clust_210								
	124485730	hypothetical protein Mlab_0908	No	0	YES		902136	902627 +		Methanocorpusculum_labreanum_Z_uid58785	NC_008942	chromosome		arCOG06904	7 (-)	S		Uncharacterized conserved protein			
===									6	Methanocorpusculum_labreanum_Z_uid58785	NC_008942	chromosome									
	124486015	hypothetical protein Mlab_1195	No	0	YES		1221014	1221451 +		Methanocorpusculum_labreanum_Z_uid58785	NC_008942	chromosome		arCOG07749	2 (-)	L		PD-(D/E)XK superfamily nuclease	2E-31	pfam13366	PD-(D/E)XK nuclease superfamily. Members
	124486016	hypothetical protein Mlab_1196	No	0	YES		1221840	1222346 -		Methanocorpusculum_labreanum_Z_uid58785	NC_008942	chromosome	clust_210								
	124486017	hypothetical protein Mlab_1197	No	0	YES		1222346	1222867 -		Methanocorpusculum_labreanum_Z_uid58785	NC_008942	chromosome	clust_17								
	124486018	hypothetical protein Mlab_1198	No	0	YES		1222864	1223328 -		Methanocorpusculum_labreanum_Z_uid58785	NC_008942	chromosome	clust_216								
	124486019	phosphate uptake regulator, PhoU	No	0	YES		1224210	1224452 -		Methanocorpusculum_labreanum_Z_uid58785	NC_008942	chromosome									
	124486020	hypothetical protein Mlab_1200	No	0	YES		1224818	1228048 -		Methanocorpusculum_labreanum_Z_uid58785	NC_008942	chromosome		arCOG06887	2 (-)	V		Restriction endonuclease	1E-101	COG3587	Restriction endonuclease
===									6	Methanocorpusculum_labreanum_Z_uid58785	NC_008942	chromosome									
	124486276	glutamine synthetase, beta-grasp	No	1			1496530	1497885 +		Methanocorpusculum_labreanum_Z_uid58785	NC_008942	chromosome		arCOG02416	3 (-)	N		Predicted flagellin FlaG	0.000003	COG3291	FOG: PKD repeat
	124486277	hypothetical protein Mlab_1461	No	0			1500262	1500627 +		Methanocorpusculum_labreanum_Z_uid58785	NC_008942	chromosome	clust_44								
	124486278	hypothetical protein Mlab_1462	No	0			1500991	1501518 +		Methanocorpusculum_labreanum_Z_uid58785	NC_008942	chromosome	clust_17								
	124486279	hypothetical protein Mlab_1463	No	0			1501515	1501775 +		Methanocorpusculum_labreanum_Z_uid58785	NC_008942	chromosome	clust_53								
	124486280	hypothetical protein Mlab_1464	No	0			1501762	1502259 +		Methanocorpusculum_labreanum_Z_uid58785	NC_008942	chromosome	clust_38								
	124486281	hypothetical protein Mlab_1465	No	0			1502793	1503284 +		Methanocorpusculum_labreanum_Z_uid58785	NC_008942	chromosome		arCOG06904	7 (-)	S		Uncharacterized conserved protein			
===									8	Methanocorpusculum_labreanum_Z_uid58785	NC_008942	chromosome									
	124486416	adenine deaminase	No	0	YES		1632760	1633803 -		Methanocorpusculum_labreanum_Z_uid58785	NC_008942	chromosome		arCOG07475	1 (-)	E		Zincin superfamily protease fused to xre like trans	1E-11	COG1396	Predicted transcriptional regulators
	124486417	hypothetical protein Mlab_1604	No	1	YES		1634098	1634508 -		Methanocorpusculum_labreanum_Z_uid58785	NC_008942	chromosome									
	124486418	hypothetical protein Mlab_1605	No	0	YES		1635301	1635666 +		Methanocorpusculum_labreanum_Z_uid58785	NC_008942	chromosome	clust_44								
	124486419	hypothetical protein Mlab_1606	No	0	YES		1635663	1636043 +		Methanocorpusculum_labreanum_Z_uid58785	NC_008942	chromosome									
	124486420	hypothetical protein Mlab_1607	No	0	YES		1636040	1636567 +		Methanocorpusculum_labreanum_Z_uid58785	NC_008942	chromosome	clust_17								
	124486421	hypothetical protein Mlab_1608	No	0	YES		1636564	1636830 +		Methanocorpusculum_labreanum_Z_uid58785	NC_008942	chromosome	clust_53								
	124486422	hypothetical protein Mlab_1609	No	0	YES		1636817	1637302 +		Methanocorpusculum_labreanum_Z_uid58785	NC_008942	chromosome	clust_38								
	124486423	hypothetical protein Mlab_1610	No	0	YES		1637835	1638290 +		Methanocorpusculum_labreanum_Z_uid58785	NC_008942	chromosome		arCOG07749	2 (-)	L		PD-(D/E)XK superfamily nuclease	1E-32	pfam13366	PD-(D/E)XK nuclease superfamily. Members
===									6	Methanocorpusculum_labreanum_Z_uid58785	NC_008942	chromosome									
	124486516	nucleolar GTP-binding 1	No	3			1738781	1739245 -		Methanocorpusculum_labreanum_Z_uid58785	NC_008942	chromosome		arCOG01912	1 (-)	O		Membrane protein implicated in regulation of mer	5E-12	COG1585	Membrane protein implicated in regulatio
	124486517	hypothetical protein Mlab_1705	No	0			1739376	1740287 -		Methanocorpusculum_labreanum_Z_uid58785	NC_008942	chromosome		arCOG10913	3	S		Uncharacterized conserved protein	6E-31	pfam11288	Protein of unknown function (DUF3089). T
	124486518	HAD superfamily hydrolase	No	0			1740406	1741317 -		Methanocorpusculum_labreanum_Z_uid58785	NC_008942	chromosome		arCOG10913	3	S		Uncharacterized conserved protein	3E-27	pfam11288	Protein of unknown function (DUF3089). T
	124486519	DNA repair and recombination protein RadA	Yes	1			1741419	1742465 -		Methanocorpusculum_labreanum_Z_uid58785	NC_008942	chromosome		arCOG10913	3	S		Uncharacterized conserved protein	2E-29	pfam11288	Protein of unknown function (DUF3089). T
	124486520	nucleic acid binding, OB-fold, tRNA/helicase-ty	Yes	1			1742557	1744071 -		Methanocorpusculum_labreanum_Z_uid58785	NC_008942	chromosome							0.000000001	COG1511	Predicted membrane protein
	124486521	hypothetical protein Mlab_1709	No	11			1744073	1746139 -		Methanocorpusculum_labreanum_Z_uid58785	NC_008942	chromosome		arCOG02174	2 (-)	R		Predicted exporter of the RND superfamily	5E-40	COG1033	Predicted exporters of the RND superfami
===									8	Methanocorpusculum_labreanum_Z_uid58785	NC_008942	chromosome									
	124486537	hypothetical protein Mlab_1725	No	0			1773109	1773579 +		Methanocorpusculum_labreanum_Z_uid58785	NC_008942	chromosome		arCOG05141	1 (-)	E	GloA	Lactoylglutathione lyase or related enzyme	3E-10	pfam12681	Glyoxalase-like domain. This domain is r
	124486538	isocitrate/isopropylmalate dehydrogenase	No	6			1773631	1774308 -		Methanocorpusculum_labreanum_Z_uid58785	NC_008942	chromosome									
	124486539	hypothetical protein Mlab_1727	No	1			1774759	1775184 -		Methanocorpusculum_labreanum_Z_uid58785	NC_008942	chromosome		arCOG07521	6	S		Uncharacterized conserved protein			
	124486540	aconitate hydratase domain-containing protein	No	6			1775181	1775870 -		Methanocorpusculum_labreanum_Z_uid58785	NC_008942	chromosome									
	124486541	hypothetical protein Mlab_1729	No	1			1776037	1776435 -		Methanocorpusculum_labreanum_Z_uid58785	NC_008942	chromosome		arCOG07521	6	S		Uncharacterized conserved protein			
	124486542	hypothetical protein Mlab_1730	Yes	1			1776739	1777170 -		Methanocorpusculum_labreanum_Z_uid58785	NC_008942	chromosome									
	124486543	hypothetical protein Mlab_1731	No	1			1777381	1778061 -		Methanocorpusculum_labreanum_Z_uid58785	NC_008942	chromosome									
	124486544	hypothetical protein Mlab_1732	Yes	2			1779727	1780173 +		Methanocorpusculum_labreanum_Z_uid58785	NC_008942	chromosome		arCOG04987	6 (-)	S		Uncharacterized conserved protein			
===									6	Methanoculleus_bourgensis_MS2_uid171377	NC_018227	chromosome									
	397779204	cytochrome c-type biogenesis protein CcdA	No	6			35754	36530 +		Methanoculleus_bourgensis_MS2_uid171377	NC_018227	chromosome		arCOG02398	1 (-)	O	CcdA	Cytochrome c biogenesis protein	4E-18	COG0785	Cytochrome c biogenesis protein
	397779205	hypothetical protein BN140_0039	Yes	1			36645	37136 -		Methanoculleus_bourgensis_MS2_uid171377	NC_018227	chromosome	clust_201								
	397779206	hypothetical protein BN140_0040	Yes	1			37142	37357 -		Methanoculleus_bourgensis_MS2_uid171377	NC_018227	chromosome									
	397779207	hypothetical protein BN140_0041	No	0			37896	39059 -		Methanoculleus_bourgensis_MS2_uid171377	NC_018227	chromosome									
	397779208	hypothetical protein BN140_0042	No	0			39182	39754 -		Methanoculleus_bourgensis_MS2_uid171377	NC_018227	chromosome	clust_172								
	397779209	ABC-2 type transporter	No	6			40202	41164 -		Methanoculleus_bourgensis_MS2_uid171377	NC_018227	chromosome		arCOG02436	3 (-)	R	NosY	ABC-type transport system involved in multi-copp	7E-24	COG1277	ABC-type transport system involved in mu
===									6	Methanoculleus_bourgensis_MS2_uid171377	NC_018227	chromosome									
	397779540	hypothetical protein BN140_0374	No	0			381561	381791 -		Methanoculleus_bourgensis_MS2_uid171377	NC_018227	chromosome		arCOG03966	3 (-)	V		CopG family DNA-binding protein			
	397779541	hypothetical protein BN140_0375	No	0			382001	382138 +		Methanoculleus_bourgensis_MS2_uid171377	NC_018227	chromosome									
	397779542	hypothetical protein BN140_0376	No	0			382317	382496 +		Methanoculleus_bourgensis_MS2_uid171377	NC_018227	chromosome									
	397779543	hypothetical protein BN140_0377	Yes	1			382752	383846 +		Methanoculleus_bourgensis_MS2_uid171377	NC_018227	chromosome							0.000001	COG4886	Leucine-rich repeat (LRR) protein
	397779544	hypothetical protein BN140_0378	No	0			383951	384058 -		Methanoculleus_bourgensis_MS2_uid171377	NC_018227	chromosome									
	397779545	Acetyl-hydrolase	No	0			384487	385329 -		Methanoculleus_bourgensis_MS2_uid171377	NC_018227	chromosome		arCOG02638	1 (-)	I	Aes	Esterase/lipase	3E-43	COG0657	Esterase/lipase
===									7	Methanoculleus_bourgensis_MS2_uid171377	NC_018227	chromosome									
	397779566	hypothetical protein BN140_0400	No	0			405755	406801 -		Methanoculleus_bourgensis_MS2_uid171377	NC_018227	chromosome		arCOG08223	1 (-)	S		Uncharacterized conserved protein			
	397779567	hypothetical protein BN140_0401	No	0			407126	407686 -		Methanoculleus_bourgensis_MS2_uid171377	NC_018227	chromosome		arCOG06788	4	S		Uncharacterized conserved protein, DUF1269 family			
	397779569	hypothetical protein BN140_0402	No	3			408033	408386 -		Methanoculleus_bourgensis_MS2_uid171377	NC_018227	chromosome									
	397779568	hypothetical protein BN140_0403	No	0			408369	408524 +		Methanoculleus_bourgensis_MS2_uid171377	NC_018227	chromosome									
	397779570	hypothetical protein BN140_0404	Yes	0			408717	409127 -		Methanoculleus_bourgensis_MS2_uid171377	NC_018227	chromosome	clust_251								
	397779571	hypothetical protein BN140_0405	No	0			409417	409683 +		Methanoculleus_bourgensis_MS2_uid171377	NC_018227	chromosome									
	397779572	hypothetical protein BN140_0406	No	0			409686	410579 -		Methanoculleus_bourgensis_MS2_uid171377	NC_018227	chromosome		arCOG01917	12 (-)	S		Zn-ribbon domain containing protein			
===									5	Methanoculleus_bourgensis_MS2_uid171377	NC_018227	chromosome									
	397779832	hypothetical protein BN140_0666	No	0			695996	696373 +		Methanoculleus_bourgensis_MS2_uid171377	NC_018227	chromosome									
	397779833	hypothetical protein BN140_0667	No	0			696379	697110 +		Methanoculleus_bourgensis_MS2_uid171377	NC_018227	chromosome	clust_136								
	397779834	hypothetical protein BN140_0668	No	0			697286	697540 -		Methanoculleus_bourgensis_MS2_uid171377	NC_018227	chromosome		arCOG02123	2 (-)	V		HEPN domain containing protein			
	397779835	hypothetical protein BN140_0669	No	0			697524	697808 -		Methanoculleus_bourgensis_MS2_uid171377	NC_018227	chromosome									
	397779836	hypothetical protein BN140_0670	No	0			697826	698203 -		Methanoculleus_bourgensis_MS2_uid171377	NC_018227	chromosome		arCOG06937	1	S		Uncharacterized conserved protein			
===									7	Methanoculleus_bourgensis_MS2_uid171377	NC_018227	chromosome									
	397779873	hypothetical protein BN140_0707	No	0			735643	738408 +		Methanoculleus_bourgensis_MS2_uid171377	NC_018227	chromosome		arCOG03287	4 (-)	G	GDB1	Glycogen debranching enzyme	0.00002	pfam03200	Mannosyl oligosaccharide glucosidase. Th
	397779874	hypothetical protein BN140_0708	Yes	0			738927	739346 +		Methanoculleus_bourgensis_MS2_uid171377	NC_018227	chromosome	clust_251								
	397779875	hypothetical protein BN140_0709	No	0			739421	739960 +		Methanoculleus_bourgensis_MS2_uid171377	NC_018227	chromosome		arCOG03995	2	S		Uncharacterized conserved protein	6E-30	COG4894	Uncharacterized conserved protein
	397779876	hypothetical protein BN140_0710	No	0			740274	740426 +		Methanoculleus_bourgensis_MS2_uid171377	NC_018227	chromosome		arCOG06788	4	S		Uncharacterized conserved protein, DUF1269 family			
	397779877	hypothetical protein BN140_0711	No	1			740455	740643 +		Methanoculleus_bourgensis_MS2_uid171377	NC_018227	chromosome									
	397779878	hypothetical protein BN140_0712	No	1			740694	741161 -		Methanoculleus_bourgensis_MS2_uid171377	NC_018227	chromosome									
	397779879	hypothetical protein BN140_0713	No	0			742765	745536 +		Methanoculleus_bourgensis_MS2_uid171377	NC_018227	chromosome		arCOG03287	4 (-)	G	GDB1	Glycogen debranching enzyme	0.0001	pfam03200	Mannosyl oligosaccharide glucosidase. Th
===									5	Methanoculleus_bourgensis_MS2_uid171377	NC_018227	chromosome									
	397780189	hypothetical protein BN140_1023	No	0			1070639	1070755 +		Methanoculleus_bourgensis_MS2_uid171377	NC_018227	chromosome									
	397780190	hypothetical protein BN140_1024	No	1			1071100	1071486 +		Methanoculleus_bourgensis_MS2_uid171377	NC_018227	chromosome		arCOG02424	2 (-)	S		Uncharacterized conserved protein	5E-16	pfam07790	Protein of unknown function (DUF1628). T
	397780191	hypothetical protein BN140_1025	No	4			1071614	1072177 +		Methanoculleus_bourgensis_MS2_uid171377	NC_018227	chromosome		arCOG08234	2	S		Uncharacterized conserved protein			
	397780192	hypothetical protein BN140_1026	No	0			1072551	1072694 +		Methanoculleus_bourgensis_MS2_uid171377	NC_018227	chromosome									
	397780193	hypothetical protein BN140_1027	No	0			1072673	1072957 +		Methanoculleus_bourgensis_MS2_uid171377	NC_018227	chromosome		arCOG09583	7	S		Uncharacterized conserved protein			
===									6	Methanoculleus_bourgensis_MS2_uid171377	NC_018227	chromosome									
	397780271	PemK-like protein	No	1			1138604	1138939 +		Methanoculleus_bourgensis_MS2_uid171377	NC_018227	chromosome		arCOG03943	2 (-)	V	MazF	Toxin-antitoxin addiction module toxin componen	2E-14	COG2337	Growth inhibitor
	397780272	hypothetical protein BN140_1106	No	0			1139061	1139246 -		Methanoculleus_bourgensis_MS2_uid171377	NC_018227	chromosome									
	397780273	hypothetical protein BN140_1107	No	0			1139413	1139679 +		Methanoculleus_bourgensis_MS2_uid171377	NC_018227	chromosome									
	397780274	hypothetical protein BN140_1108	No	0			1140073	1140399 +		Methanoculleus_bourgensis_MS2_uid171377	NC_018227	chromosome									
	397780275	hypothetical protein BN140_1109	No	7			1140884	1142047 -		Methanoculleus_bourgensis_MS2_uid171377	NC_018227	chromosome		arCOG06797	1	S		Uncharacterized conserved protein			
	397780276	periplasmic protein	Yes	1			1142044	1143645 -		Methanoculleus_bourgensis_MS2_uid171377	NC_018227	chromosome	arCOG02527,a	arCOG02527	1 (-)	S		PKD repeats containing protein	0.0000007	pfam08308	PEGA domain. This domain is found in bot ; PEGA domain. This domain is found in bot ; Periplasmic component of the Tol biopoly
===									7	Methanoculleus_bourgensis_MS2_uid171377	NC_018227	chromosome									
	397780389	Tyrosine recombinase xerC	No	0			1256405	1257571 +		Methanoculleus_bourgensis_MS2_uid171377	NC_018227	chromosome		arCOG01242	14 (-)	L	XerC	Integrase	2E-17	COG4973	Site-specific recombinase XerC
	397780390	hypothetical protein BN140_1224	No	0			1257719	1257862 +		Methanoculleus_bourgensis_MS2_uid171377	NC_018227	chromosome	clust_500								
	397780391	hypothetical protein BN140_1225	No	0			1257841	1258122 +		Methanoculleus_bourgensis_MS2_uid171377	NC_018227	chromosome		arCOG09583	7	S		Uncharacterized conserved protein			
	397780392	hypothetical protein BN140_1226	No	0			1258115	1258336 +		Methanoculleus_bourgensis_MS2_uid171377	NC_018227	chromosome									
	397780393	hypothetical protein BN140_1227	No	0			1258377	1258781 -		Methanoculleus_bourgensis_MS2_uid171377	NC_018227	chromosome									
	397780394	hypothetical protein BN140_1228	No	0			1258809	1260119 -		Methanoculleus_bourgensis_MS2_uid171377	NC_018227	chromosome		arCOG03038	9 (-)	R	NrfG	TPR repeats containing protein	0.00001	COG0457	FOG: TPR repeat
	397780395	hypothetical protein BN140_1229	No	0			1260121	1260849 -		Methanoculleus_bourgensis_MS2_uid171377	NC_018227	chromosome	clust_136								
===									5	Methanoculleus_bourgensis_MS2_uid171377	NC_018227	chromosome									
	397781261	hypothetical protein BN140_2095	No	1			2170508	2170753 +		Methanoculleus_bourgensis_MS2_uid171377	NC_018227	chromosome									
	397781262	glucose-6-phosphate isomerase, archaeal	No	0			2170930	2171628 +		Methanoculleus_bourgensis_MS2_uid171377	NC_018227	chromosome		arCOG02602	1 (-)	G		Thermophilic glucose-6-phosphate isomerase or	4E-27	pfam06560	Glucose-6-phosphate isomerase (GPI). Thi
	397781263	hypothetical protein BN140_2097	No	0			2171664	2172239 +		Methanoculleus_bourgensis_MS2_uid171377	NC_018227	chromosome									
	397781264	hypothetical protein BN140_2098	No	0			2172046	2172180 +		Methanoculleus_bourgensis_MS2_uid171377	NC_018227	chromosome									
	397781265	hypothetical protein BN140_2099	No	0			2172249	2172344 +		Methanoculleus_bourgensis_MS2_uid171377	NC_018227	chromosome									
===									6	Methanoculleus_bourgensis_MS2_uid171377	NC_018227	chromosome									
	397781283	hypothetical protein BN140_2117	No	0			2191991	2192116 +		Methanoculleus_bourgensis_MS2_uid171377	NC_018227	chromosome		arCOG01191	3 (-)	V		HEPN domain containing protein			
	397781284	hypothetical protein BN140_2118	No	0			2194669	2194764 +		Methanoculleus_bourgensis_MS2_uid171377	NC_018227	chromosome									
	397781285	hypothetical protein BN140_2119	No	0			2194843	2195022 +		Methanoculleus_bourgensis_MS2_uid171377	NC_018227	chromosome									
	397781286	hypothetical protein BN140_2120	No	0			2195280	2195693 +		Methanoculleus_bourgensis_MS2_uid171377	NC_018227	chromosome									
	397781287	hypothetical protein BN140_2121	No	0			2195322	2195471 -		Methanoculleus_bourgensis_MS2_uid171377	NC_018227	chromosome									

397781288	hypothetical protein BN140_2122	No	0			2195468	2195587	-		Methanoculleus_bourgensis_MS2_uid171377	NC_018227	chromosome		arCOG01195	1 (-)	V		Minimal nucleotidyltransferase	0.00008	pfam01909	Nucleotidyltransferase domain. Members o
===									5	Methanoculleus_bourgensis_MS2_uid171377	NC_018227	chromosome									
397781327	hypothetical protein BN140_2161	Yes	1			2259060	2259305	+		Methanoculleus_bourgensis_MS2_uid171377	NC_018227	chromosome									
397781328	hypothetical protein BN140_2162	No	0			2259130	2259360	+		Methanoculleus_bourgensis_MS2_uid171377	NC_018227	chromosome									
397781329	hypothetical protein BN140_2163	No	0			2259399	2259524	+		Methanoculleus_bourgensis_MS2_uid171377	NC_018227	chromosome									
397781330	transcriptional regulator, TrmB	No	0			2260092	2260463	+		Methanoculleus_bourgensis_MS2_uid171377	NC_018227	chromosome		arCOG01679	2 (-)	K	ArsR	Transcriptional regulator containing HTH domain,	0.000002	COG1777	Predicted transcriptional regulators
397781331	hypothetical protein BN140_2165	No	2			2260450	2260773	+		Methanoculleus_bourgensis_MS2_uid171377	NC_018227	chromosome		arCOG11069	3	S		Uncharacterized conserved protein			
===									6	Methanoculleus_bourgensis_MS2_uid171377	NC_018227	chromosome									
397781619	transporter	No	5			2610101	2611084	+		Methanoculleus_bourgensis_MS2_uid171377	NC_018227	chromosome		arCOG01474	2 (-)	P	MMT1	Predicted Co/Zn/Cd cation transporter	1E-52	COG0053	Predicted Co/Zn/Cd cation transporters [
397781620	hypothetical protein BN140_2454	Yes	1			2611106	2611699	-		Methanoculleus_bourgensis_MS2_uid171377	NC_018227	chromosome									
397781621	hypothetical protein BN140_2455	No	0			2612242	2613642	+		Methanoculleus_bourgensis_MS2_uid171377	NC_018227	chromosome									
397781622	hypothetical protein BN140_2456	No	0			2613599	2613886	-		Methanoculleus_bourgensis_MS2_uid171377	NC_018227	chromosome									
397781623	hypothetical protein BN140_2457	No	0			2613996	2614118	+		Methanoculleus_bourgensis_MS2_uid171377	NC_018227	chromosome									
397781624	hypothetical protein BN140_2458	No	5			2614157	2614873	+		Methanoculleus_bourgensis_MS2_uid171377	NC_018227	chromosome		arCOG01330	2 (-)	S		Predicted membrane protein	0.000001	COG2426	Predicted membrane protein
===									12	Methanoculleus_bourgensis_MS2_uid171377	NC_018227	chromosome									
397781626	hypothetical protein BN140_2460	No	0			2616866	2617003	-		Methanoculleus_bourgensis_MS2_uid171377	NC_018227	chromosome									
397781627	hypothetical protein BN140_2461	No	0			2617565	2620030	+		Methanoculleus_bourgensis_MS2_uid171377	NC_018227	chromosome		arCOG01444	1 (-)	V	Cas3	CRISPR-associated helicase Cas3	5E-27	COG1203	CRISPR-associated helicase Cas3
397781628	hypothetical protein BN140_2462	No	0			2620027	2622432	+		Methanoculleus_bourgensis_MS2_uid171377	NC_018227	chromosome									
397781629	hypothetical protein BN140_2463	No	0			2622459	2623487	+		Methanoculleus_bourgensis_MS2_uid171377	NC_018227	chromosome							7E-28	pfam09617	CRISPR-associated protein GSU0053 (Cas_G
397781630	hypothetical protein BN140_2464	No	0			2623500	2624990	+		Methanoculleus_bourgensis_MS2_uid171377	NC_018227	chromosome							3E-25	pfam09609	CRISPR-associated protein, GSU0054 famil
397781631	hypothetical protein BN140_2465	No	0			2625133	2626818	+		Methanoculleus_bourgensis_MS2_uid171377	NC_018227	chromosome		arCOG00786	4 (-)	V	Cas4	CRISPR-associated protein Cas4, RecB family e›	8E-63	pfam01867	CRISPR associated protein Cas1. Clustere
397781632	hypothetical protein BN140_2466	No	0			2638061	2638390	+		Methanoculleus_bourgensis_MS2_uid171377	NC_018227	chromosome									
397781633	hypothetical protein BN140_2467	No	0			2638547	2638708	+		Methanoculleus_bourgensis_MS2_uid171377	NC_018227	chromosome		arCOG10994	1	S		Uncharacterized conserved protein			
397781634	hypothetical protein BN140_2468	No	0			2638738	2641521	-		Methanoculleus_bourgensis_MS2_uid171377	NC_018227	chromosome		arCOG07809	9 (-)	Y		Viral primase fused to AAA DnaA like ATPAse and Zn finger domain			
397781635	hypothetical protein BN140_2469	No	0			2641518	2641838	-		Methanoculleus_bourgensis_MS2_uid171377	NC_018227	chromosome									
397781636	hypothetical protein BN140_2470	No	0			2641835	2642146	-		Methanoculleus_bourgensis_MS2_uid171377	NC_018227	chromosome		arCOG09583	7	S		Uncharacterized conserved protein			
397781637	hypothetical protein BN140_2471	No	0			2642301	2642438	-		Methanoculleus_bourgensis_MS2_uid171377	NC_018227	chromosome									
===									10	Methanoculleus_marisnigri_JR1_uid58561	NC_009051	chromosome									
126178031	Na+/Pi-cotransporter	No	9			73919	75697	-		Methanoculleus_marisnigri_JR1_uid58561	NC_009051	chromosome		arCOG00233	1 (-)	P	NptA	Na+/phosphate symporter	7E-70	COG1283	Na+/phosphate symporter
126178032	hypothetical protein Memar_0080	No	5			75780	76304	-		Methanoculleus_marisnigri_JR1_uid58561	NC_009051	chromosome									
126178033	hypothetical protein Memar_0081	Yes	5			76371	76937	-		Methanoculleus_marisnigri_JR1_uid58561	NC_009051	chromosome		arCOG09592	2	S		Uncharacterized conserved protein			
126178034	hypothetical protein Memar_0082	No	2			77121	77330	-		Methanoculleus_marisnigri_JR1_uid58561	NC_009051	chromosome									
126178035	hypothetical protein Memar_0083	No	2			77334	77558	-		Methanoculleus_marisnigri_JR1_uid58561	NC_009051	chromosome									
126178036	hypothetical protein Memar_0084	No	1			78157	78678	-		Methanoculleus_marisnigri_JR1_uid58561	NC_009051	chromosome	clust_201								
126178037	hypothetical protein Memar_0085	Yes	1			78699	79205	-		Methanoculleus_marisnigri_JR1_uid58561	NC_009051	chromosome									
126178038	ABC-2 type transporter	No	6			79554	80522	-		Methanoculleus_marisnigri_JR1_uid58561	NC_009051	chromosome		arCOG02436	3 (-)	R	NosY	ABC-type transport system involved in multi-copp	2E-22	COG1277	ABC-type transport system involved in mu
126178039	hypothetical protein Memar_0087	Yes	1			81016	81792	-		Methanoculleus_marisnigri_JR1_uid58561	NC_009051	chromosome									
126178040	hypothetical protein Memar_0088	No	0			81807	82415	-		Methanoculleus_marisnigri_JR1_uid58561	NC_009051	chromosome	clust_172								
===									8	Methanoculleus_marisnigri_JR1_uid58561	NC_009051	chromosome									
126178049	ATP-dependent protease La	No	0			90152	92533	+		Methanoculleus_marisnigri_JR1_uid58561	NC_009051	chromosome		arCOG02161	1 (-)	O	Lon	ATP-dependent Lon protease, bacterial type		COG0466	ATP-dependent Lon protease, bacterial ty
126178050	hypothetical protein Memar_0098	No	1			92740	93498	+		Methanoculleus_marisnigri_JR1_uid58561	NC_009051	chromosome		arCOG06136	2	S		Uncharacterized conserved protein			
126178051	hypothetical protein Memar_0099	No	1			93498	93935	+		Methanoculleus_marisnigri_JR1_uid58561	NC_009051	chromosome									
126178052	hypothetical protein Memar_0100	No	1			93959	94405	+		Methanoculleus_marisnigri_JR1_uid58561	NC_009051	chromosome									
126178053	hypothetical protein Memar_0101	Yes	0			94711	95985	+		Methanoculleus_marisnigri_JR1_uid58561	NC_009051	chromosome									
126178054	Outer membrane lipoprotein-sorting protein-lik	No	0			96059	96835	+		Methanoculleus_marisnigri_JR1_uid58561	NC_009051	chromosome		arCOG02470	4 (-)	M	LolA	Outer membrane lipoprotein-sorting protein	0.000002	COG2834	Outer membrane lipoprotein-sorting prote
126178055	hypothetical protein Memar_0103	Yes	1			97127	98221	+		Methanoculleus_marisnigri_JR1_uid58561	NC_009051	chromosome		arCOG09593	8	S		Uncharacterized conserved protein			
126178056	hypothetical protein Memar_0104	Yes	1			98223	98483	+		Methanoculleus_marisnigri_JR1_uid58561	NC_009051	chromosome									
===									10	Methanoculleus_marisnigri_JR1_uid58561	NC_009051	chromosome									
126178075	hypothetical protein Memar_0123	Yes	1	YES		113898	114860	+		Methanoculleus_marisnigri_JR1_uid58561	NC_009051	chromosome		arCOG03956	2 (-)	O	HypF	Hydrogenase maturation factor			
126178076	hypothetical protein Memar_0124	No	1	YES		114899	115474	+		Methanoculleus_marisnigri_JR1_uid58561	NC_009051	chromosome		arCOG10344	3	S		Uncharacterized conserved protein			
126178077	hypothetical protein Memar_0125	Yes	1	YES		115977	116981	+		Methanoculleus_marisnigri_JR1_uid58561	NC_009051	chromosome		arCOG10238	4	S		Uncharacterized conserved protein			
126178078	hypothetical protein Memar_0126	Yes	0	YES		117001	117375	+		Methanoculleus_marisnigri_JR1_uid58561	NC_009051	chromosome									
126178079	hypothetical protein Memar_0127	No	0	YES		117401	117706	+		Methanoculleus_marisnigri_JR1_uid58561	NC_009051	chromosome									
126178080	hypothetical protein Memar_0128	No	0	YES		117901	118095	-		Methanoculleus_marisnigri_JR1_uid58561	NC_009051	chromosome									
126178081	regulatory protein, ArsR	No	2	YES		118268	118741	-		Methanoculleus_marisnigri_JR1_uid58561	NC_009051	chromosome		arCOG01686	3 (-)	K	ArsR	Transcriptional regulator containing HTH domain,	0.00009	COG0640	Predicted transcriptional regulators
126178082	hypothetical protein Memar_0130	No	1	YES		118861	119676	-		Methanoculleus_marisnigri_JR1_uid58561	NC_009051	chromosome									
126178083	hypothetical protein Memar_0131	Yes	0	YES		119703	120479	-		Methanoculleus_marisnigri_JR1_uid58561	NC_009051	chromosome		arCOG06558	10	S		Uncharacterized conserved protein			
126178084	hypothetical protein Memar_0132	Yes	1	YES		120495	121187	-		Methanoculleus_marisnigri_JR1_uid58561	NC_009051	chromosome		arCOG11246	3	S		Uncharacterized conserved protein			
===									8	Methanoculleus_marisnigri_JR1_uid58561	NC_009051	chromosome									
126178144	HAD superfamily hydrolase-like protein	No	0			176294	178120	+		Methanoculleus_marisnigri_JR1_uid58561	NC_009051	chromosome		arCOG02291	4 (-)	R		HAD superfamily hydrolase	5E-24	COG5610	Predicted hydrolase (HAD superfamily)
126178145	hypothetical protein Memar_0194	No	0			178538	179350	-		Methanoculleus_marisnigri_JR1_uid58561	NC_009051	chromosome									
126178146	hypothetical protein Memar_0195	No	0			179379	179507	-		Methanoculleus_marisnigri_JR1_uid58561	NC_009051	chromosome									
126178147	hypothetical protein Memar_0196	No	0			179594	179710	-		Methanoculleus_marisnigri_JR1_uid58561	NC_009051	chromosome									
126178148	hypothetical protein Memar_0197	No	0			179888	179989	-		Methanoculleus_marisnigri_JR1_uid58561	NC_009051	chromosome									
126178149	hypothetical protein Memar_0198	No	11			180065	182014	-		Methanoculleus_marisnigri_JR1_uid58561	NC_009051	chromosome		arCOG07506	3	S		Uncharacterized conserved protein			
126178150	hypothetical protein Memar_0199	No	6			183065	184057	+		Methanoculleus_marisnigri_JR1_uid58561	NC_009051	chromosome		arCOG00901	1 (-)	S		Uncharacterized conserved membrane protein	3E-24	pfam03706	Uncharacterized protein family (UPF0104)
126178151	hypothetical protein Memar_0200	No	1			184151	184372	-		Methanoculleus_marisnigri_JR1_uid58561	NC_009051	chromosome									
===									6	Methanoculleus_marisnigri_JR1_uid58561	NC_009051	chromosome									
126178389	hypothetical protein Memar_0439	No	0	YES		424055	424357	+		Methanoculleus_marisnigri_JR1_uid58561	NC_009051	chromosome		arCOG05296	1 (-)	S		Uncharacterized conserved protein	3E-10	pfam11950	Protein of unknown function (DUF3467). T
126178390	hypothetical protein Memar_0440	No	0	YES		424709	425947	-		Methanoculleus_marisnigri_JR1_uid58561	NC_009051	chromosome		arCOG04985	2	S		Uncharacterized conserved protein			
126178391	hypothetical protein Memar_0441	No	0	YES		426098	426814	-		Methanoculleus_marisnigri_JR1_uid58561	NC_009051	chromosome									
126178392	hypothetical protein Memar_0442	No	2	YES		426983	427591	-		Methanoculleus_marisnigri_JR1_uid58561	NC_009051	chromosome									
126178393	hypothetical protein Memar_0443	No	0			427615	427857	-		Methanoculleus_marisnigri_JR1_uid58561	NC_009051	chromosome									
126178394	hypothetical protein Memar_0444	No	6			428158	428712	-		Methanoculleus_marisnigri_JR1_uid58561	NC_009051	chromosome		arCOG03582	1 (-)	S	HdeD	Uncharacterized conserved protein	3E-16	COG3247	Uncharacterized conserved protein
===									6	Methanoculleus_marisnigri_JR1_uid58561	NC_009051	chromosome									
126178748	DNA polymerase B region	No	0			779117	781555	+		Methanoculleus_marisnigri_JR1_uid58561	NC_009051	chromosome		arCOG00328	8 (-)	L	PolB	DNA polymerase elongation subunit (family B)	6E-85	COG0417	DNA polymerase elongation subunit (famil
126178749	hypothetical protein Memar_0799	No	0			781694	782164	-		Methanoculleus_marisnigri_JR1_uid58561	NC_009051	chromosome									
126178750	hypothetical protein Memar_0800	Yes	0			782446	783072	-		Methanoculleus_marisnigri_JR1_uid58561	NC_009051	chromosome	clust_156								
126178751	hypothetical protein Memar_0801	Yes	0			783138	783788	-		Methanoculleus_marisnigri_JR1_uid58561	NC_009051	chromosome	clust_156								
126178752	hypothetical protein Memar_0802	No	0			784132	784545	-		Methanoculleus_marisnigri_JR1_uid58561	NC_009051	chromosome									
126178753	hypothetical protein Memar_0803	No	0			784771	785403	+		Methanoculleus_marisnigri_JR1_uid58561	NC_009051	chromosome		arCOG08122	1 (-)	S		Uncharacterized conserved protein			
===									6	Methanoculleus_marisnigri_JR1_uid58561	NC_009051	chromosome									
126178866	hypothetical protein Memar_0916	No	0			898862	899308	+		Methanoculleus_marisnigri_JR1_uid58561	NC_009051	chromosome		arCOG04915	5 (-)	S		Uncharacterized conserved protein			
126178867	hypothetical protein Memar_0917	No	4			899308	899910	+		Methanoculleus_marisnigri_JR1_uid58561	NC_009051	chromosome							0.00000002	pfam07895	Protein of unknown function (DUF1673). T
126178868	hypothetical protein Memar_0918	No	3			899910	900599	+		Methanoculleus_marisnigri_JR1_uid58561	NC_009051	chromosome		arCOG06827	7	S		Uncharacterized conserved protein			
126178869	hypothetical protein Memar_0919	No	2			900596	901075	+		Methanoculleus_marisnigri_JR1_uid58561	NC_009051	chromosome									
126178870	hypothetical protein Memar_0920	No	4			901072	901818	+		Methanoculleus_marisnigri_JR1_uid58561	NC_009051	chromosome		arCOG06827	7	S		Uncharacterized conserved protein			
126178871	ABC transporter-related protein	No	0			901915	902724	+		Methanoculleus_marisnigri_JR1_uid58561	NC_009051	chromosome		arCOG05270	1 (-)	P	ModF	ABC-type molybdenum transport system, ATPas	6E-49	COG1119	ABC-type molybdenum transport system, AT
===									6	Methanoculleus_marisnigri_JR1_uid58561	NC_009051	chromosome									
126180024	hypothetical protein Memar_2082	No	0			2071942	2072139	+		Methanoculleus_marisnigri_JR1_uid58561	NC_009051	chromosome									
126180025	hypothetical protein Memar_2083	Yes	1			2072206	2072577	+		Methanoculleus_marisnigri_JR1_uid58561	NC_009051	chromosome									
126180026	hypothetical protein Memar_2084	No	0			2072574	2073254	+		Methanoculleus_marisnigri_JR1_uid58561	NC_009051	chromosome		arCOG06558	10	S		Uncharacterized conserved protein			
126180027	ABC-type transport system involved in multi-co	No	5			2073277	2074479	+		Methanoculleus_marisnigri_JR1_uid58561	NC_009051	chromosome		arCOG02436	3 (-)	R	NosY	ABC-type transport system involved in multi-copp	3E-23	COG1277	ABC-type transport system involved in mu
126180028	hypothetical protein Memar_2086	Yes	1			2074667	2075230	+		Methanoculleus_marisnigri_JR1_uid58561	NC_009051	chromosome									
126180029	hypothetical protein Memar_2087	Yes	0			2075350	2075829	+		Methanoculleus_marisnigri_JR1_uid58561	NC_009051	chromosome									
===									9	Methanoculleus_marisnigri_JR1_uid58561	NC_009051	chromosome									
126180046	hypothetical protein Memar_2104	No	0			2091716	2092009	+		Methanoculleus_marisnigri_JR1_uid58561	NC_009051	chromosome									
126180047	hypothetical protein Memar_2105	No	0			2092079	2092573	+		Methanoculleus_marisnigri_JR1_uid58561	NC_009051	chromosome									
126180048	endothelin-converting protein 1	Yes	0			2092714	2094684	+		Methanoculleus_marisnigri_JR1_uid58561	NC_009051	chromosome		arCOG05200	1 (-)	O	PepO	Predicted metalloendopeptidase	7E-136	COG3590	Predicted metalloendopeptidase
126180049	hypothetical protein Memar_2107	No	7			2094799	2095770	-		Methanoculleus_marisnigri_JR1_uid58561	NC_009051	chromosome									
126180050	hypothetical protein Memar_2108	No	5			2096270	2097274	+		Methanoculleus_marisnigri_JR1_uid58561	NC_009051	chromosome									
126180051	hypothetical protein Memar_2109	Yes	1			2097271	2100459	+		Methanoculleus_marisnigri_JR1_uid58561	NC_009051	chromosome									
126180052	hypothetical protein Memar_2110	Yes	1			2100697	2101455	+		Methanoculleus_marisnigri_JR1_uid58561	NC_009051	chromosome									
126180053	hypothetical protein Memar_2111	No	0			2101512	2101727	-		Methanoculleus_marisnigri_JR1_uid58561	NC_009051	chromosome									
126180054	major facilitator transporter	Yes	12			2102014	2103240	+		Methanoculleus_marisnigri_JR1_uid58561	NC_009051	chromosome		arCOG00130	5 (-)	G	ProP	Permease of the major facilitator superfamily	3E-30	pfam07690	Major Facilitator Superfamily.
===									6	Methanoculleus_marisnigri_JR1_uid58561	NC_009051	chromosome									
126180059	hypothetical protein Memar_2117	No	0	YES		2107836	2108534	+		Methanoculleus_marisnigri_JR1_uid58561	NC_009051	chromosome		arCOG03215	1 (-)	S		Uncharacterized conserved protein	4E-42	pfam09888	Uncharacterized protein conserved in arc
126180060	hypothetical protein Memar_2119	No	0	YES		2108991	2109188	+		Methanoculleus_marisnigri_JR1_uid58561	NC_009051	chromosome									
126180061	hypothetical protein Memar_2121	No	4	YES		2109535	2110674	+		Methanoculleus_marisnigri_JR1_uid58561	NC_009051	chromosome		arCOG10995	1	S		Uncharacterized conserved protein			
126180062	hypothetical protein Memar_2122	No	0	YES		2110775	2111812	-		Methanoculleus_marisnigri_JR1_uid58561	NC_009051	chromosome									
126180063	hypothetical protein Memar_2123	No	0	YES		2112408	2112788	+		Methanoculleus_marisnigri_JR1_uid58561	NC_009051	chromosome									
126180064	DNA polymerase II large subunit	No	0			2113145	2117002	-		Methanoculleus_marisnigri_JR1_uid58561	NC_009051	chromosome	arCOG03147,a	arCOG03147	1 (-)	L		Intein/homing endonuclease		pfam03833	DNA polymerase II large subunit DP2. ; Archaeal DNA polymerase II, large subunit
===									7	Methanoculleus_marisnigri_JR1_uid58561	NC_009051	chromosome									
126180275	hypothetical protein Memar_2335	No	1			2322364	2323062	+		Methanoculleus_marisnigri_JR1_uid58561	NC_009051	chromosome		arCOG04952	2 (-)	S		Uncharacterized conserved protein			
126180276	hypothetical protein Memar_2336	Yes	0			2323383	2324027	+		Methanoculleus_marisnigri_JR1_uid58561	NC_009051	chromosome	clust_15								
126180277	hypothetical protein Memar_2337	Yes	0			2324080	2324772	+		Methanoculleus_marisnigri_JR1_uid58561	NC_009051	chromosome	clust_15								
126180278	hypothetical protein Memar_2338	Yes	0			2324861	2325448	+		Methanoculleus_marisnigri_JR1_uid58561	NC_009051	chromosome	clust_15								
126180279	hypothetical protein Memar_2339	Yes	1			2325445	2326038	+		Methanoculleus_marisnigri_JR1_uid58561	NC_009051	chromosome	clust_15								
126180280	hypothetical protein Memar_2340	No	0			2326445	2326654	+		Methanoculleus_marisnigri_JR1_uid58561	NC_009051	chromosome									
126180281	lysyl-tRNA synthetase	No	0			2326708	2328234	+		Methanoculleus_marisnigri_JR1_uid58561	NC_009051	chromosome		arCOG00408	1 (-)	J	LysU	Lysyl-tRNA synthetase (class II)		COG1190	Lysyl-tRNA synthetase (class II)
===									5	Methanoculleus_marisnigri_JR1_uid58561	NC_009051	chromosome									
126180352	hypothetical protein Memar_2412	Yes	1			2394327	2395121	-		Methanoculleus_marisnigri_JR1_uid58561	NC_009051	chromosome		arCOG10996	1	S		Uncharacterized conserved protein			
126180353	hypothetical protein Memar_2413	No	4			2395118	2395582	-		Methanoculleus_marisnigri_JR1_uid58561	NC_009051	chromosome		arCOG10421	1	S		Uncharacterized conserved protein			
126180354	FeoA family protein	No	0			2397392	2397631	-		Methanoculleus_marisnigri_JR1_uid58561	NC_009051	chromosome							0.0001	pfam04023	FeoA domain. This family includes FeoA a
126180355	FeoA family protein	No	0			2397636	2397854	-		Methanoculleus_marisnigri_JR1_uid58561	NC_009051	chromosome		arCOG02102	1 (-)	P	FeoA	Fe2+ transport system protein A	0.000001	COG1918	Fe2+ transport system protein A
126180356	hypothetical protein Memar_2418	No	0			2398567	2398767	-		Methanoculleus_marisnigri_JR1_uid58561	NC_009051	chromosome									
===									5	Methanohalobium_evestigatum_Z_7303_uid4985	NC_014253	chromosome									
298674119	hypothetical protein Metev_0142	No	0			143252	143488	-		Methanohalobium_evestigatum_Z_7303_uid4985	NC_014253	chromosome									
298674120	hypothetical protein Metev_0143	No	0			143794	144348	+		Methanohalobium_evestigatum_Z_7303_uid4985	NC_014253	chromosome									
298674121	hypothetical protein Metev_0144	No	0			144397	144612	+		Methanohalobium_evestigatum_Z_7303_uid4985	NC_014253	chromosome									
298674122	hypothetical protein Metev_0145	No	0			144914	148747	-		Methanohalobium_evestigatum_Z_7303_uid4985	NC_014253	chromosome		arCOG00806	1 (-)	L		Superfamily I DNA/RNA helicase fused to RecB f	3E-12	COG1112	Superfamily I DNA and RNA helicases and ; AAA domain. This fam
298674123	hypothetical protein Metev_0146	No	0			148977	149837	+		Methanohalobium_evestigatum_Z_7303_uid4985	NC_014253	chromosome									
===									5	Methanohalobium_evestigatum_Z_7303_uid4985	NC_014253	chromosome									
298674129	hypothetical protein Metev_0152	No	0			155868	156710	+		Methanohalobium_evestigatum_Z_7303_uid4985	NC_014253	chromosome									
298674130	hypothetical protein Metev_0153	No	0			157125	157256	+		Methanohalobium_evestigatum_Z_7303_uid4985	NC_014253	chromosome									
298674131	hypothetical protein Metev_0154	No	0			157367	158944	+		Methanohalobium_evestigatum_Z_7303_uid4985	NC_014253	chromosome		arCOG04113	1 (-)	J	RplP	Ribosomal protein L10AE/L16			
298674132	hypothetical protein Metev_0155	No	0			159004	160839	-		Methanohalobium_evestigatum_Z_7303_uid4985	NC_014253	chromosome	clust_24								
298674133	hypothetical protein Metev_0156	No	1			161501	161740	-		Methanohalobium_evestigatum_Z_7303_uid4985	NC_014253	chromosome									
===									5	Methanohalobium_evestigatum_Z_7303_uid4985	NC_014253	chromosome									
298674320	heterotrimeric replication protein A subunit	No	0			340369	340668	-		Methanohalobium_evestigatum_Z_7303_uid4985	NC_014253	chromosome	clust_353								
298674321	hypothetical protein Metev_0350	No	0			340704	341291	-		Methanohalobium_evestigatum_Z_7303_uid4985	NC_014253	chromosome									
298674322	hypothetical protein Metev_0351	No	0			341293	342201	-		Methanohalobium_evestigatum_Z_7303_uid4985	NC_014253	chromosome									
298674323	hypothetical protein Metev_0352	No	0			342296	344188	-		Methanohalobium_evestigatum_Z_7303_uid4985	NC_014253	chromosome	arCOG00798,a	arCOG00798	1 (-)	L	UvrD	Superfamily I DNA and RNA helicase	0.00000003	COG3972	Superfamily I DNA and RNA helicases
298674324	hypothetical protein Metev_0353	No	2			345153	346013	-		Methanohalobium_evestigatum_Z_7303_uid4985	NC_014253	chromosome									
===									7	Methanohalobium_evestigatum_Z_7303_uid4985	NC_014253	chromosome									
298674427	TATA-box-binding family protein	No	0			462540	463058	+		Methanohalobium_evestigatum_Z_7303_uid4985	NC_014253	chromosome		arCOG01764	2 (-)	K	SPT15	TATA-box binding protein (TBP), component of T	3E-23	COG2101	TATA-box binding protein (TBP), componen
298674428	hypothetical protein Metev_0467	No	0			463061	463789	-		Methanohalobium_evestigatum_Z_7303_uid4985	NC_014253	chromosome		arCOG10685	1	I		Phospholipase D/Transphosphatidylase			
298674429	hypothetical protein Metev_0468	No	0			464437	464601	-		Methanohalobium_evestigatum_Z_7303_uid4985	NC_014253	chromosome									
298674430	hypothetical protein Metev_0469	No	0			464594	464761	-		Methanohalobium_evestigatum_Z_7303_uid4985	NC_014253	chromosome									
298674431	hypothetical protein Metev_0470	No	0			464951	465505	+		Methanohalobium_evestigatum_Z_7303_uid4985	NC_014253	chromosome									
298674432	E1-like enzyme family protein	No	0			465651	465833	-		Methanohalobium_evestigatum_Z_7303_uid4985	NC_014253	chromosome									
298674433	integrase family protein	No	0			466291	467139	-		Methanohalobium_evestigatum_Z_7303_uid4985	NC_014253	chromosome		arCOG01241	31 (-)	L	XerC	Integrase	0.00001	COG4973	Site-specific recombinase XerC
===									17	Methanohalobium_evestigatum_Z_7303_uid4985	NC_014253	chromosome									
298674562	hypothetical protein Metev_0610	No	4			596046	596648	+		Methanohalobium_evestigatum_Z_7303_uid4985	NC_014253	chromosome									
298674563	hypothetical protein Metev_0611	Yes	1			596635	598464	+		Methanohalobium_evestigatum_Z_7303_uid4985	NC_014253	chromosome									
298674564	hypothetical protein Metev_0612	No	0			598468	598788	+		Methanohalobium_evestigatum_Z_7303_uid4985	NC_014253	chromosome		arCOG06608	1 (-)	S		Uncharacterized conserved protein			
298674565	hypothetical protein Metev_0613	Yes	2			598788	599579	+		Methanohalobium_evestigatum_Z_7303_uid4985	NC_014253	chromosome									
298674566	hypothetical protein Metev_0614	No	2			599584	600411	+		Methanohalobium_evestigatum_Z_7303_uid4985	NC_014253	chromosome									
298674567	hypothetical protein Metev_0615	No	0			600411	601376	+		Methanohalobium_evestigatum_Z_7303_uid4985	NC_014253	chromosome							0.000004	pfam05707	Zonular occludens toxin (Zot). This fami
298674568	hypothetical protein Metev_0616	No	0			601360	601824	-		Methanohalobium_evestigatum_Z_7303_uid4985	NC_014253	chromosome									
298674569	hypothetical protein Metev_0617	No	1			601923	602789	-		Methanohalobium_evestigatum_Z_7303_uid4985	NC_014253	chromosome		arCOG02420	1 (-)	S		Uncharacterized conserved protein	0.000000005	pfam07790	Protein of unknown function (DUF1628). T
298674570	hypothetical protein Metev_0618	No	3			602834	603130	-		Methanohalobium_evestigatum_Z_7303_uid4985	NC_014253	chromosome									
298674571	hypothetical protein Metev_0619	Yes	0			603132	603749	-		Methanohalobium_evestigatum_Z_7303_uid4985	NC_014253	chromosome									
298674572	hypothetical protein Metev_0620	No	2			603755	603940	-		Methanohalobium_evestigatum_Z_7303_uid4985	NC_014253	chromosome									
298674573	PEGA domain-containing protein	Yes	1			603937	605250	-		Methanohalobium_evestigatum_Z_7303_uid4985	NC_014253	chromosome	arCOG03264,a	arCOG03264	1 (-)	S		Cell suface protein	0.0000002	pfam08308	PEGA domain. This domain is found in bot
298674574	hypothetical protein Metev_0622	Yes	3			605256	607085	-		Methanohalobium_evestigatum_Z_7303_uid4985	NC_014253	chromosome									
298674575	hypothetical protein Metev_0623	No	1			607082	607243	-		Methanohalobium_evestigatum_Z_7303_uid4985	NC_014253	chromosome									
298674576	hypothetical protein Metev_0624	No	6			607259	607978	-		Methanohalobium_evestigatum_Z_7303_uid4985	NC_014253	chromosome									
298674577	hypothetical protein Metev_0625	No	2			608034	609212	-		Methanohalobium_evestigatum_Z_7303_uid4985	NC_014253	chromosome									
298674578	FG-GAP repeat-containing protein	No	2			609460	610788	-		Methanohalobium_evestigatum_Z_7303_uid4985	NC_014253	chromosome		arCOG10338	2 (-)	S		Uncharacterized conserved protein	0.0001	pfam13517	Family description. This domain of about ; Family description. This
===									13	Methanohalobium_evestigatum_Z_7303_uid4985	NC_014253	chromosome									
298674580	hypothetical protein Metev_0628	Yes	0			611943	612437	-		Methanohalobium_evestigatum_Z_7303_uid4985	NC_014253	chromosome									
298674581	hypothetical protein Metev_0629	Yes	2			612470	612730	-		Methanohalobium_evestigatum_Z_7303_uid4985	NC_014253	chromosome	clust_182								
298674582	hypothetical protein Metev_0630	No	0			613041	613709	-		Methanohalobium_evestigatum_Z_7303_uid4985	NC_014253	chromosome		arCOG03989	1 (-)	L	XerC	Integrase			
298674583	hypothetical protein Metev_0631	No	0			613958	614377	-		Methanohalobium_evestigatum_Z_7303_uid4985	NC_014253	chromosome									
298674584	hypothetical protein Metev_0632	No	0			614517	614654	-		Methanohalobium_evestigatum_Z_7303_uid4985	NC_014253	chromosome									
298674585	hypothetical protein Metev_0633	No	0			614655	614837	-		Methanohalobium_evestigatum_Z_7303_uid4985	NC_014253	chromosome									
298674586	hypothetical protein Metev_0634	No	0			614852	615031	-		Methanohalobium_evestigatum_Z_7303_uid4985	NC_014253	chromosome									
298674587	hypothetical protein Metev_0635	No	0			615162	615299	-		Methanohalobium_evestigatum_Z_7303_uid4985	NC_014253	chromosome									
298674588	hypothetical protein Metev_0636	No	0			615303	615671	-		Methanohalobium_evestigatum_Z_7303_uid4985	NC_014253	chromosome									
298674589	hypothetical protein Metev_0637	No	0			615682	616212	-		Methanohalobium_evestigatum_Z_7303_uid4985	NC_014253	chromosome									
298674590	hypothetical protein Metev_0638	No	0			616212	616409	-		Methanohalobium_evestigatum_Z_7303_uid4985	NC_014253	chromosome									
298674591	hypothetical protein Metev_0639	No	0			616406	616585	-		Methanohalobium_evestigatum_Z_7303_uid4985	NC_014253	chromosome									
298674592	Integrase catalytic region	No	0			616671	617177	-		Methanohalobium_evestigatum_Z_7303_uid4985	NC_014253	chromosome		arCOG02129	2 (-)	L		Transposase	2E-12	COG3335	Transposase and inactivated derivatives
===									17	Methanohalobium_evestigatum_Z_7303_uid4985	NC_014253	chromosome									
298674598	LuxR family transcriptional regulator	No	0			620086	620304	-		Methanohalobium_evestigatum_Z_7303_uid4985	NC_014253	chromosome									
298674599	hypothetical protein Metev_0647	No	0			620304	620936	-		Methanohalobium_evestigatum_Z_7303_uid4985	NC_014253	chromosome									
298674600	hypothetical protein Metev_0648	No	0			620948	621091	-		Methanohalobium_evestigatum_Z_7303_uid4985	NC_014253	chromosome									
298674601	MCM family protein	No	0			621105	623231	-		Methanohalobium_evestigatum_Z_7303_uid4985	NC_014253	chromosome		arCOG00439	14 (-)	L	MCM2	Predicted ATPase involved in replication control,	1E-167	COG1241	Predicted ATPase involved in replication
298674602	hypothetical protein Metev_0650	No	0			623237	623647	-		Methanohalobium_evestigatum_Z_7303_uid4985	NC_014253	chromosome									
298674603	hypothetical protein Metev_0651	No	0			623746	624279	-		Methanohalobium_evestigatum_Z_7303_uid4985	NC_014253	chromosome									
298674604	hypothetical protein Metev_0652	No	0			624276	624647	-		Methanohalobium_evestigatum_Z_7303_uid4985	NC_014253	chromosome									
298674605	hypothetical protein Metev_0653	No	0			624644	624823	-		Methanohalobium_evestigatum_Z_7303_uid4985	NC_014253	chromosome		arCOG10941	4	S		Uncharacterized conserved protein			
298674606	hypothetical protein Metev_0654	No	0			624820	624957	-		Methanohalobium_evestigatum_Z_7303_uid4985	NC_014253	chromosome									
298674607	hypothetical protein Metev_0655	No	0			624954	625148	-		Methanohalobium_evestigatum_Z_7303_uid4985	NC_014253	chromosome		arCOG10941	4	S		Uncharacterized conserved protein			
298674608	hypothetical protein Metev_0656	No	0			625171	625377	-		Methanohalobium_evestigatum_Z_7303_uid4985	NC_014253	chromosome									
298674609	hypothetical protein Metev_0657	No	0			625374	625676	-		Methanohalobium_evestigatum_Z_7303_uid4985	NC_014253	chromosome									
298674610	hypothetical protein Metev_0658	No	0			625677	625859	-		Methanohalobium_evestigatum_Z_7303_uid4985	NC_014253	chromosome									
298674611	hypothetical protein Metev_0659	No	0			625860	626048	-		Methanohalobium_evestigatum_Z_7303_uid4985	NC_014253	chromosome									
298674612	hypothetical protein Metev_0660	No	0			626051	626218	-		Methanohalobium_evestigatum_Z_7303_uid4985	NC_014253	chromosome									
298674613	putative CopG/Arc/MetJ family transcriptional r	No	0			626366	626590	-		Methanohalobium_evestigatum_Z_7303_uid4985	NC_014253	chromosome		arCOG01009	8 (-)	V		Ribbon-helix-helix protein, copG family	0.00004	COG3609	Predicted transcriptional regulators con
298674614	hypothetical protein Metev_0662	No	0			626762	627106	+		Methanohalobium_evestigatum_Z_7303_uid4985	NC_014253	chromosome									
===									9	Methanohalobium_evestigatum_Z_7303_uid4985	NC_014253	chromosome									
298674616	hypothetical protein Metev_0664	No	0			627501	627968	+		Methanohalobium_evestigatum_Z_7303_uid4985	NC_014253	chromosome		arCOG06843	1	S		Uncharacterized conserved protein			
298674617	hypothetical protein Metev_0665	No	0			627978	628775	+		Methanohalobium_evestigatum_Z_7303_uid4985	NC_014253	chromosome		arCOG06842	2 (-)	S		Uncharacterized conserved protein	0.000000005	pfam08861	Domain of unknown function DUF1828. This
298674618	hypothetical protein Metev_0666	No	0			628868	629809	+		Methanohalobium_evestigatum_Z_7303_uid4985	NC_014253	chromosome									
298674619	hypothetical protein Metev_0667	No	0			629901	631439	+		Methanohalobium_evestigatum_Z_7303_uid4985	NC_014253	chromosome									
298674620	hypothetical protein Metev_0668	No	0			631467	632771	-		Methanohalobium_evestigatum_Z_7303_uid4985	NC_014253	chromosome									
298674621	DNA adenine methylase	No	0			632764	633717	-		Methanohalobium_evestigatum_Z_7303_uid4985	NC_014253	chromosome		arCOG03416	1 (-)	L	Dam	Site-specific DNA methylase	7E-57	COG0338	Site-specific DNA methylase
298674622	hypothetical protein Metev_0670	No	0			633905	634393	-		Methanohalobium_evestigatum_Z_7303_uid4985	NC_014253	chromosome									
298674623	hypothetical protein Metev_0671	No	0			634917	635177	-		Methanohalobium_evestigatum_Z_7303_uid4985	NC_014253	chromosome									
298674624	hypothetical protein Metev_0672	No	0			635277	635528	-		Methanohalobium_evestigatum_Z_7303_uid4985	NC_014253	chromosome									
===									5	Methanohalobium_evestigatum_Z_7303_uid4985	NC_014253	chromosome									
298674913	hypothetical protein Metev_0976	No	2			934227	934502	-		Methanohalobium_evestigatum_Z_7303_uid4985	NC_014253	chromosome		arCOG06819	1	S		Uncharacterized conserved protein			
298674914	hypothetical protein Metev_0977	No	0			934719	935345	+		Methanohalobium_evestigatum_Z_7303_uid4985	NC_014253	chromosome									
298674915	hypothetical protein Metev_0978	No	0			935368	935487	-		Methanohalobium_evestigatum_Z_7303_uid4985	NC_014253	chromosome									
298674916	5-oxoprolinase	No	0			935538	939200	-		Methanohalobium_evestigatum_Z_7303_uid4985	NC_014253	chromosome	arCOG01511,a	arCOG01511	3 (-)	E	HyuA	N-methylhydantoinase A/acetone carboxylase, beta subunit		pfam02538	Hydantoinase B/oxoprolinase. This family ; N-methylhydantoinase A/acetone carboxyla
298674917	hypothetical protein Metev_0980	No	4			939334	939753	+		Methanohalobium_evestigatum_Z_7303_uid4985	NC_014253	chromosome									
===									6	Methanohalobium_evestigatum_Z_7303_uid4985	NC_014253	chromosome									
298675377	glutamine amidotransferase class-II	No	0			1481089	1482216	-		Methanohalobium_evestigatum_Z_7303_uid4985	NC_014253	chromosome		arCOG00095	1 (-)	E	GltB	Glutamate synthase domain 1	1E-21	COG0067	Glutamate synthase domain 1
298675378	hypothetical protein Metev_1479	No	0			1482698	1482883	-		Methanohalobium_evestigatum_Z_7303_uid4985	NC_014253	chromosome									
298675379	hypothetical protein Metev_1480	No	1			1482850	1483065	-		Methanohalobium_evestigatum_Z_7303_uid4985	NC_014253	chromosome									
298675380	hypothetical protein Metev_1481	No	1			1483165	1483512	-		Methanohalobium_evestigatum_Z_7303_uid4985	NC_014253	chromosome		arCOG03599	1	S		Uncharacterized conserved protein			
298675381	hypothetical protein Metev_1482	No	1			1483684	1484229	-		Methanohalobium_evestigatum_Z_7303_uid4985	NC_014253	chromosome		arCOG03624	3	S		Uncharacterized conserved protein	1E-18	pfam05727	Uncharacterized protein family (UPF0228)
298675382	hypothetical protein Metev_1483	Yes	1			1484358	1485533	-		Methanohalobium_evestigatum_Z_7303_uid4985	NC_014253	chromosome		arCOG10650	2 (-)	S		Uncharacterized conserved protein			
===									9	Methanohalobium_evestigatum_Z_7303_uid4985	NC_014254	plasmid pMETEV01									
298676196	response regulator receiver protein	No	0		plasmid	110780	111151	+		Methanohalobium_evestigatum_Z_7303_uid4985	NC_014254	plasmid pMETEV01		arCOG02589	3 (-)	T	CheY	Rec domain	8E-24	pfam00072	Response regulator receiver domain. This
298676197	hypothetical protein Metev_2338	No	0		plasmid	111902	112537	+		Methanohalobium_evestigatum_Z_7303_uid4985	NC_014254	plasmid pMETEV01									
298676198	hypothetical protein Metev_2339	No	0		plasmid	112684	113199	+		Methanohalobium_evestigatum_Z_7303_uid4985	NC_014254	plasmid pMETEV01									
298676199	hypothetical protein Metev_2340	No	0		plasmid	113593	113946	+		Methanohalobium_evestigatum_Z_7303_uid4985	NC_014254	plasmid pMETEV01									
298676200	hypothetical protein Metev_2341	No	0		plasmid	114019	114225	+		Methanohalobium_evestigatum_Z_7303_uid4985	NC_014254	plasmid pMETEV01									
298676201	hypothetical protein Metev_2343	No	0		plasmid	115092	115376	-		Methanohalobium_evestigatum_Z_7303_uid4985	NC_014254	plasmid pMETEV01									
298676202	hypothetical protein Metev_2344	No	10		plasmid	115721	116806	+		Methanohalobium_evestigatum_Z_7303_uid4985	NC_014254	plasmid pMETEV01		arCOG04963	1 (-)	G	RhaT	permease of the drug/metabolite transporter (DM	0.000002	COG0697	Permeases of the drug/metabolite transpo
298676203	hypothetical protein Metev_2345	No	0		plasmid	116887	117066	-		Methanohalobium_evestigatum_Z_7303_uid4985	NC_014254	plasmid pMETEV01									
298676204	hypothetical protein Metev_2346	No	1		plasmid	117587	117736	-		Methanohalobium_evestigatum_Z_7303_uid4985	NC_014254	plasmid pMETEV01									
===									7	Methanohalobium_evestigatum_Z_7303_uid4985	NC_014254	plasmid pMETEV01									
298676208	hypothetical protein Metev_2350	No	0		plasmid	120552	120788	-		Methanohalobium_evestigatum_Z_7303_uid4985	NC_014254	plasmid pMETEV01									
298676209	periplasmic copper-binding protein	No	1		plasmid	121141	127029	-		Methanohalobium_evestigatum_Z_7303_uid4985	NC_014254	plasmid pMETEV01	arCOG02542,a	arCOG02542	3 (-)	R		Cell surface protein containing PGF motif	2E-38	pfam05048	Periplasmic copper-binding protein (NosD ; Autotransporter adhesin
298676210	hypothetical protein Metev_2353	No	0		plasmid	129617	130003	+		Methanohalobium_evestigatum_Z_7303_uid4985	NC_014254	plasmid pMETEV01									
298676211	hypothetical protein Metev_2354	Yes	0		plasmid	130089	130451	+		Methanohalobium_evestigatum_Z_7303_uid4985	NC_014254	plasmid pMETEV01									
298676212	hypothetical protein Metev_2355	No	0		plasmid	130451	131029	+		Methanohalobium_evestigatum_Z_7303_uid4985	NC_014254	plasmid pMETEV01	clust_182								
298676213	hypothetical protein Metev_2356	No	1		plasmid	131667	131915	-		Methanohalobium_evestigatum_Z_7303_uid4985	NC_014254	plasmid pMETEV01									
298676214	plasmid stabilization system	No	0		plasmid	132136	132408	-		Methanohalobium_evestigatum_Z_7303_uid4985	NC_014254	plasmid pMETEV01		arCOG01663	2 (-)	V	RelE	Cytotoxic translational repressor of toxin-antitoxin	0.000000001	COG2026	Cytotoxic translational repressor of tox
===									8	Methanohalophilus_mahii_DSM_5219_uid47313	NC_014002	chromosome									
294495769	hypothetical protein Mmah_1110	No	2			1134272	1134667	-		Methanohalophilus_mahii_DSM_5219_uid47313	NC_014002	chromosome									
294495770	hypothetical protein Mmah_1111	No	0			1134672	1135292	-		Methanohalophilus_mahii_DSM_5219_uid47313	NC_014002	chromosome		arCOG02408	2 (-)	S		Uncharacterized conserved protein	0.000000003	pfam01936	NYN domain. These domains are found in t
294495771	hypothetical protein Mmah_1112	No	1			1135450	1136109	+		Methanohalophilus_mahii_DSM_5219_uid47313	NC_014002	chromosome									
294495772	hypothetical protein Mmah_1113	No	0			1136392	1137105	-		Methanohalophilus_mahii_DSM_5219_uid47313	NC_014002	chromosome									
294495773	hypothetical protein Mmah_1114	No	1			1137347	1137727	-		Methanohalophilus_mahii_DSM_5219_uid47313	NC_014002	chromosome									
294495774	hypothetical protein Mmah_1115	No	1			1137945	1138418	+		Methanohalophilus_mahii_DSM_5219_uid47313	NC_014002	chromosome		arCOG07647	3	S		Uncharacterized conserved protein			
294495775	hypothetical protein Mmah_1116	No	0			1138832	1139011	-		Methanohalophilus_mahii_DSM_5219_uid47313	NC_014002	chromosome									
294495776	phosphodiesterase, MJ0936 family	No	0			1139083	1139592	+		Methanohalophilus_mahii_DSM_5219_uid47313	NC_014002	chromosome		arCOG01141	2 (-)	R		Phosphoesterase	2E-17	pfam12850	Calcineurin-like phosphoesterase superfa
===									6	Methanolobus_psychrophilus_R15_uid177925	NC_018876	chromosome									
410669351	hypothetical protein Mpsy_0141	No	0			143267	144586	+		Methanolobus_psychrophilus_R15_uid177925	NC_018876	chromosome		arCOG05102	3 (-)	V	McrC	McrBC 5-methylcytosine restriction system comp	4E-46	pfam10117	McrBC 5-methylcytosine restriction syste
410669352	hypothetical protein Mpsy_0142	No	0			144613	145269	-		Methanolobus_psychrophilus_R15_uid177925	NC_018876	chromosome									
410669353	hypothetical protein Mpsy_0143	No	0			145363	146130	+		Methanolobus_psychrophilus_R15_uid177925	NC_018876	chromosome									
410669354	hypothetical protein Mpsy_0144	No	0			146421	146609	+		Methanolobus_psychrophilus_R15_uid177925	NC_018876	chromosome									
410669355	hypothetical protein Mpsy_0145	No	0			146612	146839	+		Methanolobus_psychrophilus_R15_uid177925	NC_018876	chromosome									
410669356	hypothetical protein Mpsy_0146	No	0			146902	147483	+		Methanolobus_psychrophilus_R15_uid177925	NC_018876	chromosome		arCOG05220	1 (-)	S		Uncharacterized conserved protein	5E-39	pfam08747	Domain of unknown function (DUF1788). Pu
===									6	Methanolobus_psychrophilus_R15_uid177925	NC_018876	chromosome									
410669389	hypothetical protein Mpsy_0179	No	0			191108	191524	-		Methanolobus_psychrophilus_R15_uid177925	NC_018876	chromosome		arCOG07235	1 (-)	V		RHH/CopG DNA binding protein			
410669390	hypothetical protein Mpsy_0180	No	0			191683	191796	-		Methanolobus_psychrophilus_R15_uid177925	NC_018876	chromosome									
410669391	hypothetical protein Mpsy_0181	Yes	2			191795	192040	+		Methanolobus_psychrophilus_R15_uid177925	NC_018876	chromosome									
410669392	hypothetical protein Mpsy_0182	No	0			192132	193544	-		Methanolobus_psychrophilus_R15_uid177925	NC_018876	chromosome									
410669393	hypothetical protein Mpsy_0183	No	0			193531	194472	-		Methanolobus_psychrophilus_R15_uid177925	NC_018876	chromosome									
410669394	CRISPR-associated protein, Cas1 family	No	0			194666	195880	-		Methanolobus_psychrophilus_R15_uid177925	NC_018876	chromosome	arCOG01452,a	arCOG01452	5 (-)	V	Cas1	CRISPR-associated protein Cas1	2E-35	pfam01867	CRISPR associated protein Cas1. Clustere
===									5	Methanolobus_psychrophilus_R15_uid177925	NC_018876	chromosome									
410669456	hypothetical protein Mpsy_0246	No	0			251932	252063	+		Methanolobus_psychrophilus_R15_uid177925	NC_018876	chromosome									
410669457	AbrB family transcriptional regulator	No	0			252329	252574	+		Methanolobus_psychrophilus_R15_uid177925	NC_018876	chromosome		arCOG11378	3	S		Uncharacterized conserved protein			
410669458	transcriptional modulator of MazE/toxin, MazF	No	0			252761	252931	+		Methanolobus_psychrophilus_R15_uid177925	NC_018876	chromosome		arCOG03403	5 (-)	V		PemK/MazF family toxin			
410669459	hypothetical protein Mpsy_0249	No	0			252993	253121	+		Methanolobus_psychrophilus_R15_uid177925	NC_018876	chromosome									
410669460	hypothetical protein Mpsy_0250	No	0			253211	253324	-		Methanolobus_psychrophilus_R15_uid177925	NC_018876	chromosome									
===									5	Methanolobus_psychrophilus_R15_uid177925	NC_018876	chromosome									
410669533	hypothetical protein Mpsy_0325	No	0			318799	318921	-		Methanolobus_psychrophilus_R15_uid177925	NC_018876	chromosome									
410669534	beta strand repeat-containing protein	Yes	1			319103	320626	-		Methanolobus_psychrophilus_R15_uid177925	NC_018876	chromosome		arCOG06531	4	S		Uncharacterized conserved protein			
410669535	hypothetical protein Mpsy_0327	No	1			321132	321341	-		Methanolobus_psychrophilus_R15_uid177925	NC_018876	chromosome									
410669536	ribulose bisphosphate carboxylase	No	0			321502	322941	-		Methanolobus_psychrophilus_R15_uid177925	NC_018876	chromosome		arCOG04443	1 (-)	G	RbcL	Ribulose 1,5-bisphosphate carboxylase, large su	1E-80	COG1850	Ribulose 1,5-bisphosphate carboxylase, l
410669537	hypothetical protein Mpsy_0329	No	0			322991	323134	+		Methanolobus_psychrophilus_R15_uid177925	NC_018876	chromosome									
===									16	Methanolobus_psychrophilus_R15_uid177925	NC_018876	chromosome									
410669762	hypothetical protein Mpsy_0554	No	0			542219	542449	+		Methanolobus_psychrophilus_R15_uid177925	NC_018876	chromosome		arCOG01009	8 (-)	V		Ribbon-helix-helix protein, copG family			
410669763	hypothetical protein Mpsy_0555	No	0			542446	542583	+		Methanolobus_psychrophilus_R15_uid177925	NC_018876	chromosome									
410669764	hypothetical protein Mpsy_0556	No	0			542573	542707	+		Methanolobus_psychrophilus_R15_uid177925	NC_018876	chromosome									
410669765	hypothetical protein Mpsy_0557	No	0			542710	543048	+		Methanolobus_psychrophilus_R15_uid177925	NC_018876	chromosome									
410669766	hypothetical protein Mpsy_0558	No	0			543045	543191	+		Methanolobus_psychrophilus_R15_uid177925	NC_018876	chromosome									
410669767	hypothetical protein Mpsy_0559	No	0			543309	543608	+		Methanolobus_psychrophilus_R15_uid177925	NC_018876	chromosome									
410669768	hypothetical protein Mpsy_0560	No	0			543608	543868	+		Methanolobus_psychrophilus_R15_uid177925	NC_018876	chromosome									
410669769	hypothetical protein Mpsy_0561	No	0			543899	544891	+		Methanolobus_psychrophilus_R15_uid177925	NC_018876	chromosome		arCOG00417	2 (-)	L	RecA	RecA/RadA recombinase			
410669770	phage/plasmid primase, P4 family	No	0			544938	547139	+		Methanolobus_psychrophilus_R15_uid177925	NC_018876	chromosome	arCOG06914,a	arCOG06914	10 (-)	R		Phage or plasmid associated DNA primase	1E-11	COG3378	Phage associated DNA primase
410669771	hypothetical protein Mpsy_0563	No	0			547114	547245	+		Methanolobus_psychrophilus_R15_uid177925	NC_018876	chromosome									
410669773	hypothetical protein Mpsy_0565	No	0			547423	547563	-		Methanolobus_psychrophilus_R15_uid177925	NC_018876	chromosome									
410669772	hypothetical protein Mpsy_0564	No	0			547520	547639	+		Methanolobus_psychrophilus_R15_uid177925	NC_018876	chromosome									
410669774	hypothetical protein Mpsy_0566	No	0			547961	548311	+		Methanolobus_psychrophilus_R15_uid177925	NC_018876	chromosome									
410669775	hypothetical protein Mpsy_0567	No	0			548284	548718	+		Methanolobus_psychrophilus_R15_uid177925	NC_018876	chromosome		arCOG02128	6 (-)	L		Transposase			
410669776	hypothetical protein Mpsy_0568	No	0			548744	549967	+		Methanolobus_psychrophilus_R15_uid177925	NC_018876	chromosome		arCOG09550	17	Y	TerL	Phage terminase large subunit	4E-121	COG5323	Uncharacterized conserved protein
410669777	hypothetical protein Mpsy_0569	No	0			549971	550177	+		Methanolobus_psychrophilus_R15_uid177925	NC_018876	chromosome									
===									6	Methanolobus_psychrophilus_R15_uid177925	NC_018876	chromosome									
410669834	hypothetical protein Mpsy_0628	No	0			602490	603284	-		Methanolobus_psychrophilus_R15_uid177925	NC_018876	chromosome		arCOG03134	4 (-)	K		Predicted transcriptional regulator	1E-59	COG4742	Predicted transcriptional regulator
410669835	hypothetical protein Mpsy_0629	No	0			603505	603816	-		Methanolobus_psychrophilus_R15_uid177925	NC_018876	chromosome									
410669836	hypothetical protein Mpsy_0630	No	4			605314	605715	+		Methanolobus_psychrophilus_R15_uid177925	NC_018876	chromosome	clust_267								
410669837	hypothetical protein Mpsy_0631	No	0			605960	606319	+		Methanolobus_psychrophilus_R15_uid177925	NC_018876	chromosome									
410669838	hypothetical protein Mpsy_0632	No	0			606324	607124	+		Methanolobus_psychrophilus_R15_uid177925	NC_018876	chromosome									
410669839	type I restriction-modification system, M subun	No	0			607282	608796	+		Methanolobus_psychrophilus_R15_uid177925	NC_018876	chromosome		arCOG02632	3 (-)	V	HsdM	Type I restriction-modification system methyltrans	2E-109	COG0286	Type I restriction-modification system m
===									5	Methanolobus_psychrophilus_R15_uid177925	NC_018876	chromosome									
410669844	hypothetical protein Mpsy_0638	No	0			615623	616576	-		Methanolobus_psychrophilus_R15_uid177925	NC_018876	chromosome									
410669845	radical SAM domain-containing protein	No	0			616643	617389	-		Methanolobus_psychrophilus_R15_uid177925	NC_018876	chromosome		arCOG01290	5 (-)	L	SplB	DNA repair photolyase	4E-21	COG1533	DNA repair photolyase
410669846	hypothetical protein Mpsy_0640	No	0			617723	617905	+		Methanolobus_psychrophilus_R15_uid177925	NC_018876	chromosome									
410669847	hypothetical protein Mpsy_0641	No	0			617908	618858	-		Methanolobus_psychrophilus_R15_uid177925	NC_018876	chromosome									
410669848	hypothetical protein Mpsy_0642	No	2			619167	619361	+		Methanolobus_psychrophilus_R15_uid177925	NC_018876	chromosome									
===									12	Methanolobus_psychrophilus_R15_uid177925	NC_018876	chromosome									
410669850	GLUG domain protein	Yes	2			619924	623247	-		Methanolobus_psychrophilus_R15_uid177925	NC_018876	chromosome	arCOG02542,a	arCOG02542	3 (-)	R		Cell surface protein containing PGF motif	0.00003	COG5295	Autotransporter adhesin
410669851	hypothetical protein Mpsy_0645	Yes	1			623413	623550	+		Methanolobus_psychrophilus_R15_uid177925	NC_018876	chromosome									
410669852	hypothetical protein Mpsy_0646	No	0			623635	624249	-		Methanolobus_psychrophilus_R15_uid177925	NC_018876	chromosome									
410669853	hypothetical protein Mpsy_0647	No	0			624760	626619	-		Methanolobus_psychrophilus_R15_uid177925	NC_018876	chromosome									
410669854	hypothetical protein Mpsy_0648	No	0			627033	627614	-		Methanolobus_psychrophilus_R15_uid177925	NC_018876	chromosome	clust_34								
410669855	hypothetical protein Mpsy_0649	No	0			627849	628484	-		Methanolobus_psychrophilus_R15_uid177925	NC_018876	chromosome	clust_34								
410669856	hypothetical protein Mpsy_0650	No	0			628490	629179	-		Methanolobus_psychrophilus_R15_uid177925	NC_018876	chromosome	clust_34								
410669857	hypothetical protein Mpsy_0651	No	0			629369	629788	-		Methanolobus_psychrophilus_R15_uid177925	NC_018876	chromosome									
410669858	hypothetical protein Mpsy_0652	No	0			629794	630015	-		Methanolobus_psychrophilus_R15_uid177925	NC_018876	chromosome									
410669859	hypothetical protein Mpsy_0653	No	0			630364	631362	-		Methanolobus_psychrophilus_R15_uid177925	NC_018876	chromosome									
410669860	hypothetical protein Mpsy_0654	No	8			631753	632688	-		Methanolobus_psychrophilus_R15_uid177925	NC_018876	chromosome		arCOG00480	2 (-)	H	MenA	1,4-dihydroxy-2-naphthoate octaprenyltransferase	2E-18	COG1575	1,4-dihydroxy-2-naphthoate octaprenyltra
410669861	hypothetical protein Mpsy_0655	No	0			632748	632888	-		Methanolobus_psychrophilus_R15_uid177925	NC_018876	chromosome									
===									11	Methanolobus_psychrophilus_R15_uid177925	NC_018876	chromosome									
410669892	hypothetical protein Mpsy_0686	No	0			666139	666282	+		Methanolobus_psychrophilus_R15_uid177925	NC_018876	chromosome		arCOG02734	5 (-)	S		NifX family protein			
410669893	hypothetical protein Mpsy_0687	No	0			666293	666451	+		Methanolobus_psychrophilus_R15_uid177925	NC_018876	chromosome									
410669894	hypothetical protein Mpsy_0688	No	0			666567	666866	-		Methanolobus_psychrophilus_R15_uid177925	NC_018876	chromosome									
410669895	hypothetical protein Mpsy_0689	No	0			666994	667134	+		Methanolobus_psychrophilus_R15_uid177925	NC_018876	chromosome									
410669896	hypothetical protein Mpsy_0690	No	3			667240	667587	-		Methanolobus_psychrophilus_R15_uid177925	NC_018876	chromosome									
410669897	phage shock protein C	No	3			667592	668059	-		Methanolobus_psychrophilus_R15_uid177925	NC_018876	chromosome		arCOG03456	1 (-)	K	PspC	Phage shock protein C	1E-11	COG1983	Putative stress-responsive transcription
410669898	hypothetical protein Mpsy_0692	No	0			668175	668294	+		Methanolobus_psychrophilus_R15_uid177925	NC_018876	chromosome									
410669899	hypothetical protein Mpsy_0693	Yes	1			668302	668928	-		Methanolobus_psychrophilus_R15_uid177925	NC_018876	chromosome		arCOG06531	4	S		Uncharacterized conserved protein			
410669900	carbon monoxide dehydrogenase	No	0			669188	671098	-		Methanolobus_psychrophilus_R15_uid177925	NC_018876	chromosome		arCOG02429	1 (-)	C		6Fe-6S prismane cluster-containing protein	9E-107	COG1151	6Fe-6S prismane cluster-containing prote
410669901	hypothetical protein Mpsy_0695	No	0			671317	671496	-		Methanolobus_psychrophilus_R15_uid177925	NC_018876	chromosome									
410669902	hypothetical protein Mpsy_0696	No	0			671611	671733	+		Methanolobus_psychrophilus_R15_uid177925	NC_018876	chromosome									
===									7	Methanolobus_psychrophilus_R15_uid177925	NC_018876	chromosome									
410670294	hypothetical protein Mpsy_1088	No	0			1085409	1085567	+		Methanolobus_psychrophilus_R15_uid177925	NC_018876	chromosome									

410670295	hypothetical protein Mpsy_1089	No	2		1085737	1086582	-		Methanolobus_psychrophilus_R15_uid177925	NC_018876	chromosome									
410670296	hypothetical protein Mpsy_1090	No	0		1087526	1087663	+		Methanolobus_psychrophilus_R15_uid177925	NC_018876	chromosome									
410670297	hypothetical protein Mpsy_1091	No	4		1087647	1088183	+		Methanolobus_psychrophilus_R15_uid177925	NC_018876	chromosome		arCOG01330	2 (-)	S		Predicted membrane protein	0.000006	COG2426	Predicted membrane protein
410670298	hypothetical protein Mpsy_1092	Yes	1		1088395	1089591	+		Methanolobus_psychrophilus_R15_uid177925	NC_018876	chromosome									
410670299	hypothetical protein Mpsy_1093	No	2		1090261	1090920	-		Methanolobus_psychrophilus_R15_uid177925	NC_018876	chromosome									
410670300	hypothetical protein Mpsy_1094	No	0		1090994	1091110	-		Methanolobus_psychrophilus_R15_uid177925	NC_018876	chromosome									
===								5	Methanolobus_psychrophilus_R15_uid177925	NC_018876	chromosome									
410670304	hypothetical protein Mpsy_1098	No	3		1097687	1098031	+		Methanolobus_psychrophilus_R15_uid177925	NC_018876	chromosome									
410670305	hypothetical protein Mpsy_1099	Yes	0		1098480	1099094	-		Methanolobus_psychrophilus_R15_uid177925	NC_018876	chromosome									
410670306	hypothetical protein Mpsy_1100	No	0		1100820	1102592	-		Methanolobus_psychrophilus_R15_uid177925	NC_018876	chromosome		arCOG07809	9 (-)	Y		Viral primase fused to AAA DnaA like ATPAse and Zn finger domain			
410670307	hypothetical protein Mpsy_1101	No	0		1103004	1103129	-		Methanolobus_psychrophilus_R15_uid177925	NC_018876	chromosome									
410670308	hypothetical protein Mpsy_1102	No	0		1103168	1103305	-		Methanolobus_psychrophilus_R15_uid177925	NC_018876	chromosome									
===								5	Methanolobus_psychrophilus_R15_uid177925	NC_018876	chromosome									
410670451	hypothetical protein Mpsy_1246	No	0		1242363	1242641	+		Methanolobus_psychrophilus_R15_uid177925	NC_018876	chromosome									
410670452	hypothetical protein Mpsy_1247	No	0		1242638	1243027	+		Methanolobus_psychrophilus_R15_uid177925	NC_018876	chromosome		arCOG02140	1 (-)	S		Uncharacterized conserved protein			
410670453	hypothetical protein Mpsy_1248	No	0		1243232	1243603	-		Methanolobus_psychrophilus_R15_uid177925	NC_018876	chromosome									
410670454	hypothetical protein Mpsy_1249	No	0		1243766	1243900	-		Methanolobus_psychrophilus_R15_uid177925	NC_018876	chromosome									
410670455	hypothetical protein Mpsy_1250	No	0		1243921	1244064	-		Methanolobus_psychrophilus_R15_uid177925	NC_018876	chromosome	clust_501								
===								6	Methanolobus_psychrophilus_R15_uid177925	NC_018876	chromosome									
410670568	hypothetical protein Mpsy_1363	No	0		1349940	1350761	-		Methanolobus_psychrophilus_R15_uid177925	NC_018876	chromosome		arCOG03386	1 (-)	S		Uncharacterized conserved protein			
410670569	hypothetical protein Mpsy_1364	No	0		1350760	1350876	+		Methanolobus_psychrophilus_R15_uid177925	NC_018876	chromosome									
410670570	hypothetical protein Mpsy_1365	No	0		1351340	1351522	-		Methanolobus_psychrophilus_R15_uid177925	NC_018876	chromosome									
410670571	hypothetical protein Mpsy_1366	Yes	0		1351498	1352319	+		Methanolobus_psychrophilus_R15_uid177925	NC_018876	chromosome		arCOG03972	2	S		Uncharacterized conserved protein	2E-12	COG0419	ATPase involved in DNA repair
410670572	hypothetical protein Mpsy_1367	Yes	0		1352412	1352720	+		Methanolobus_psychrophilus_R15_uid177925	NC_018876	chromosome									
410670573	hypothetical protein Mpsy_1368	Yes	1		1352813	1353580	-		Methanolobus_psychrophilus_R15_uid177925	NC_018876	chromosome		arCOG05283	1 (-)	S		Uncharacterized conserved protein			
===								5	Methanolobus_psychrophilus_R15_uid177925	NC_018876	chromosome									
410670712	hypothetical protein Mpsy_1508	No	0		1488759	1488875	-		Methanolobus_psychrophilus_R15_uid177925	NC_018876	chromosome									
410670713	radical SAM family Fe-S protein	No	0		1488919	1489746	+		Methanolobus_psychrophilus_R15_uid177925	NC_018876	chromosome		arCOG00938	12 (-)	R		Radical SAM superfamily enzyme	3E-14	COG2896	Molybdenum cofactor biosynthesis enzyme
410670714	hypothetical protein Mpsy_1510	No	0		1489807	1489932	-		Methanolobus_psychrophilus_R15_uid177925	NC_018876	chromosome									
410670715	hypothetical protein Mpsy_1511	No	0		1489895	1490209	+		Methanolobus_psychrophilus_R15_uid177925	NC_018876	chromosome									
410670716	hypothetical protein Mpsy_1512	No	1		1490296	1490451	+		Methanolobus_psychrophilus_R15_uid177925	NC_018876	chromosome									
===								5	Methanolobus_psychrophilus_R15_uid177925	NC_018876	chromosome									
410671039	hypothetical protein Mpsy_1836	No	0		1807441	1807566	-		Methanolobus_psychrophilus_R15_uid177925	NC_018876	chromosome									
410671040	phage integrase	No	0		1807605	1808660	+		Methanolobus_psychrophilus_R15_uid177925	NC_018876	chromosome		arCOG01242	14 (-)	L	XerC	Integrase	0.000000002	pfam00589	Phage integrase family. Members of this
410671041	hypothetical protein Mpsy_1838	No	0		1808823	1809356	+		Methanolobus_psychrophilus_R15_uid177925	NC_018876	chromosome									
410671042	hypothetical protein Mpsy_1839	No	0		1809363	1809506	-		Methanolobus_psychrophilus_R15_uid177925	NC_018876	chromosome									
410671043	hypothetical protein Mpsy_1840	No	0		1809947	1810168	-		Methanolobus_psychrophilus_R15_uid177925	NC_018876	chromosome									
===								11	Methanolobus_psychrophilus_R15_uid177925	NC_018876	chromosome									
410671100	hypothetical protein Mpsy_1897	No	4		1860265	1860666	+		Methanolobus_psychrophilus_R15_uid177925	NC_018876	chromosome	clust_267								
410671101	hypothetical protein Mpsy_1898	No	0		1860741	1860884	+		Methanolobus_psychrophilus_R15_uid177925	NC_018876	chromosome		arCOG03953	4 (-)	S		Uncharacterized conserved protein			
410671102	hypothetical protein Mpsy_1899	No	2		1861063	1861257	+		Methanolobus_psychrophilus_R15_uid177925	NC_018876	chromosome	clust_455								
410671103	putative transmembrane protein	No	4		1861258	1861944	-		Methanolobus_psychrophilus_R15_uid177925	NC_018876	chromosome	clust_147								
410671104	hypothetical protein Mpsy_1901	No	0		1862231	1862692	+		Methanolobus_psychrophilus_R15_uid177925	NC_018876	chromosome	clust_227								
410671105	hypothetical protein Mpsy_1902	No	0		1863461	1864288	-		Methanolobus_psychrophilus_R15_uid177925	NC_018876	chromosome		arCOG03134	4 (-)	K		Predicted transcriptional regulator	2E-56	COG4742	Predicted transcriptional regulator
410671106	hypothetical protein Mpsy_1903	No	8		1864435	1865250	-		Methanolobus_psychrophilus_R15_uid177925	NC_018876	chromosome									
410671107	hypothetical protein Mpsy_1904	No	0		1865330	1865470	+		Methanolobus_psychrophilus_R15_uid177925	NC_018876	chromosome									
410671108	hypothetical protein Mpsy_1905	No	0		1865571	1866356	+		Methanolobus_psychrophilus_R15_uid177925	NC_018876	chromosome		arCOG03134	4 (-)	K		Predicted transcriptional regulator	1E-61	COG4742	Predicted transcriptional regulator
410671109	hypothetical protein Mpsy_1906	No	0		1866393	1866683	-		Methanolobus_psychrophilus_R15_uid177925	NC_018876	chromosome									
410671110	hypothetical protein Mpsy_1907	Yes	1		1866830	1867255	+		Methanolobus_psychrophilus_R15_uid177925	NC_018876	chromosome									
===								8	Methanolobus_psychrophilus_R15_uid177925	NC_018876	chromosome									
410671470	hypothetical protein Mpsy_2269	No	2		2186121	2186324	+		Methanolobus_psychrophilus_R15_uid177925	NC_018876	chromosome									
410671471	nuclease	No	0		2186338	2186637	+		Methanolobus_psychrophilus_R15_uid177925	NC_018876	chromosome		arCOG03192	6 (-)	L		Micrococcal nuclease (thermonuclease)			
410671472	hypothetical protein Mpsy_2271	No	1		2186616	2186795	-		Methanolobus_psychrophilus_R15_uid177925	NC_018876	chromosome									
410671473	hypothetical protein Mpsy_2272	No	0		2187260	2188342	-		Methanolobus_psychrophilus_R15_uid177925	NC_018876	chromosome									
410671474	hypothetical protein Mpsy_2273	No	0		2188339	2188596	-		Methanolobus_psychrophilus_R15_uid177925	NC_018876	chromosome									
410671475	hypothetical protein Mpsy_2274	No	0		2189574	2189693	-		Methanolobus_psychrophilus_R15_uid177925	NC_018876	chromosome									
410671476	hypothetical protein Mpsy_2275	No	0		2189798	2190199	-		Methanolobus_psychrophilus_R15_uid177925	NC_018876	chromosome		arCOG03353	2 (-)	S		Uncharacterized conserved protein	9E-55	pfam08004	Protein of unknown function (DUF1699). T
410671477	hypothetical protein Mpsy_2276	No	0		2190412	2190651	+		Methanolobus_psychrophilus_R15_uid177925	NC_018876	chromosome									
===								8	Methanolobus_psychrophilus_R15_uid177925	NC_018876	chromosome									
410671480	CRISPR-associated protein, Cas1 family	No	0		2192298	2192699	+		Methanolobus_psychrophilus_R15_uid177925	NC_018876	chromosome		arCOG01453	1 (-)	V	Cas1	CRISPR-associated protein Cas1			
410671481	hypothetical protein Mpsy_2280	No	0		2193998	2194126	+		Methanolobus_psychrophilus_R15_uid177925	NC_018876	chromosome									
410671482	hypothetical protein Mpsy_2281	No	0		2194174	2194293	+		Methanolobus_psychrophilus_R15_uid177925	NC_018876	chromosome									
410671483	hypothetical protein Mpsy_2282	No	0		2194336	2194764	-		Methanolobus_psychrophilus_R15_uid177925	NC_018876	chromosome									
410671484	hypothetical protein Mpsy_2283	No	0		2194823	2194966	+		Methanolobus_psychrophilus_R15_uid177925	NC_018876	chromosome									
410671485	hypothetical protein Mpsy_2284	Yes	1		2195874	2196014	+		Methanolobus_psychrophilus_R15_uid177925	NC_018876	chromosome									
410671486	hypothetical protein Mpsy_2285	No	0		2196751	2197503	+		Methanolobus_psychrophilus_R15_uid177925	NC_018876	chromosome									
410671487	hypothetical protein Mpsy_2286	No	0		2197540	2198232	-		Methanolobus_psychrophilus_R15_uid177925	NC_018876	chromosome		arCOG01449	2 (-)	V		Predicted CRISPR related protein, COG1517 family			
===								10	Methanolobus_psychrophilus_R15_uid177925	NC_018876	chromosome									
410671493	hypothetical protein Mpsy_2292	No	0		2205639	2205848	-		Methanolobus_psychrophilus_R15_uid177925	NC_018876	chromosome									
410671494	hypothetical protein Mpsy_2293	No	0		2205861	2207117	-		Methanolobus_psychrophilus_R15_uid177925	NC_018876	chromosome		arCOG04985	2	S		Uncharacterized conserved protein			
410671495	hypothetical protein Mpsy_2294	No	0		2207117	2208322	-		Methanolobus_psychrophilus_R15_uid177925	NC_018876	chromosome		arCOG00467	13 (-)	L	CDC6	Cdc6-related protein, AAA superfamily ATPase	8E-28	COG1474	Cdc6-related protein, AAA superfamily AT
410671496	hypothetical protein Mpsy_2295	No	0		2208488	2208604	-		Methanolobus_psychrophilus_R15_uid177925	NC_018876	chromosome									
410671497	hypothetical protein Mpsy_2296	No	0		2208961	2209146	-		Methanolobus_psychrophilus_R15_uid177925	NC_018876	chromosome									
410671498	hypothetical protein Mpsy_2297	No	0		2209153	2209275	+		Methanolobus_psychrophilus_R15_uid177925	NC_018876	chromosome									
410671499	hypothetical protein Mpsy_2298	No	0		2209240	2209455	-		Methanolobus_psychrophilus_R15_uid177925	NC_018876	chromosome									
410671500	hypothetical protein Mpsy_2299	No	0		2209677	2209820	-		Methanolobus_psychrophilus_R15_uid177925	NC_018876	chromosome									
410671501	hypothetical protein Mpsy_2300	No	0		2210154	2212406	+		Methanolobus_psychrophilus_R15_uid177925	NC_018876	chromosome	clust_24								
410671502	hypothetical protein Mpsy_2301	No	0		2213095	2214558	-		Methanolobus_psychrophilus_R15_uid177925	NC_018876	chromosome		arCOG06636	2 (-)	V		PIN domain containing protein			
===								8	Methanolobus_psychrophilus_R15_uid177925	NC_018876	chromosome									
410671505	hypothetical protein Mpsy_2304	No	0		2218499	2218627	+		Methanolobus_psychrophilus_R15_uid177925	NC_018876	chromosome									
410671506	repair helicase	No	0		2218730	2220292	-		Methanolobus_psychrophilus_R15_uid177925	NC_018876	chromosome		arCOG00797	3 (-)	L	UvrD	Superfamily I DNA and RNA helicase			
410671507	hypothetical protein Mpsy_2306	No	0		2221251	2222489	-		Methanolobus_psychrophilus_R15_uid177925	NC_018876	chromosome									
410671508	hypothetical protein Mpsy_2307	No	0		2222514	2222714	-		Methanolobus_psychrophilus_R15_uid177925	NC_018876	chromosome									
410671509	hypothetical protein Mpsy_2308	No	0		2222719	2222856	-		Methanolobus_psychrophilus_R15_uid177925	NC_018876	chromosome									
410671510	hypothetical protein Mpsy_2309	No	0		2224496	2224678	+		Methanolobus_psychrophilus_R15_uid177925	NC_018876	chromosome									
410671511	hypothetical protein Mpsy_2310	No	0		2224942	2225064	-		Methanolobus_psychrophilus_R15_uid177925	NC_018876	chromosome									
410671512	outer membrane adhesin like proteiin	Yes	1		2225499	2230787	+		Methanolobus_psychrophilus_R15_uid177925	NC_018876	chromosome	arCOG02542,a	arCOG02542	3 (-)	R		Cell surface protein containing PGF motif	2E-17	pfam13306	Leucine rich repeats (6 copies). This fa ; Leucine rich repeats (6 copies). This fa
===								7	Methanolobus_psychrophilus_R15_uid177925	NC_018876	chromosome									
410671592	hypothetical protein Mpsy_2391	No	0		2313895	2314053	+		Methanolobus_psychrophilus_R15_uid177925	NC_018876	chromosome		arCOG01008	7 (-)	V	NikR	Transcriptional regulator, CopG/Arc/MetJ family (DNA-binding and a metal-binding domains)			
410671593	hypothetical protein Mpsy_2392	No	0		2314043	2314273	+		Methanolobus_psychrophilus_R15_uid177925	NC_018876	chromosome									
410671594	hypothetical protein Mpsy_2393	No	0		2314298	2316784	+		Methanolobus_psychrophilus_R15_uid177925	NC_018876	chromosome									
410671595	hypothetical protein Mpsy_2394	No	0		2317558	2318619	+		Methanolobus_psychrophilus_R15_uid177925	NC_018876	chromosome									
410671596	hypothetical protein Mpsy_2395	No	0		2318796	2319113	+		Methanolobus_psychrophilus_R15_uid177925	NC_018876	chromosome									
410671597	hypothetical protein Mpsy_2396	No	0		2319232	2319357	+		Methanolobus_psychrophilus_R15_uid177925	NC_018876	chromosome									
410671598	hypothetical protein Mpsy_2397	No	0		2319455	2320420	+		Methanolobus_psychrophilus_R15_uid177925	NC_018876	chromosome	arCOG04153,a	arCOG04153	1 (-)	K		RNA polymerase III subunit C34 (Lrp/AsnC family)			
===								10	Methanolobus_psychrophilus_R15_uid177925	NC_018876	chromosome									
410671617	hypothetical protein Mpsy_2416	No	0		2336364	2336720	+		Methanolobus_psychrophilus_R15_uid177925	NC_018876	chromosome									
410671618	hypothetical protein Mpsy_2417	No	0		2336775	2337086	+		Methanolobus_psychrophilus_R15_uid177925	NC_018876	chromosome		arCOG10647	2 (-)	S		Uncharacterized conserved protein			
410671619	hypothetical protein Mpsy_2418	No	0		2337091	2337243	+		Methanolobus_psychrophilus_R15_uid177925	NC_018876	chromosome									
410671620	hypothetical protein Mpsy_2419	No	0		2337248	2337523	+		Methanolobus_psychrophilus_R15_uid177925	NC_018876	chromosome									
410671621	hypothetical protein Mpsy_2420	No	0		2337711	2338058	+		Methanolobus_psychrophilus_R15_uid177925	NC_018876	chromosome									
410671622	hypothetical protein Mpsy_2421	No	0		2338055	2338759	+		Methanolobus_psychrophilus_R15_uid177925	NC_018876	chromosome									
410671623	transposase	No	0		2338825	2339862	+		Methanolobus_psychrophilus_R15_uid177925	NC_018876	chromosome		arCOG02128	6 (-)	L		Transposase	2E-13	pfam13358	DDE superfamily endonuclease. This famil ; Transposase and inactivated derivatives
410671624	hypothetical protein Mpsy_2423	No	0		2340068	2340367	+		Methanolobus_psychrophilus_R15_uid177925	NC_018876	chromosome									
410671625	hypothetical protein Mpsy_2424	No	0		2340699	2340839	-		Methanolobus_psychrophilus_R15_uid177925	NC_018876	chromosome									
410671626	hypothetical protein Mpsy_2425	No	0		2340974	2341636	-		Methanolobus_psychrophilus_R15_uid177925	NC_018876	chromosome									
===								5	Methanolobus_psychrophilus_R15_uid177925	NC_018876	chromosome									
410671669	hypothetical protein Mpsy_2468	No	8		2373128	2374189	-		Methanolobus_psychrophilus_R15_uid177925	NC_018876	chromosome		arCOG05230	2	S		Uncharacterized conserved protein			
410671670	hypothetical protein Mpsy_2469	No	0		2374325	2375023	-		Methanolobus_psychrophilus_R15_uid177925	NC_018876	chromosome		arCOG04972	1 (-)	S		Uncharacterized conserved protein	1E-25	COG3874	Uncharacterized conserved protein
410671671	hypothetical protein Mpsy_2470	No	1		2375149	2375352	-		Methanolobus_psychrophilus_R15_uid177925	NC_018876	chromosome									
410671672	hypothetical protein Mpsy_2471	No	0		2375560	2375706	-		Methanolobus_psychrophilus_R15_uid177925	NC_018876	chromosome									
410671673	hypothetical protein Mpsy_2472	No	0		2376227	2376352	+		Methanolobus_psychrophilus_R15_uid177925	NC_018876	chromosome									
===								5	Methanolobus_psychrophilus_R15_uid177925	NC_018876	chromosome									
410671785	hypothetical protein Mpsy_2585	No	0		2482427	2482594	-		Methanolobus_psychrophilus_R15_uid177925	NC_018876	chromosome									
410671786	gluconate transporter	No	3		2482554	2482805	-		Methanolobus_psychrophilus_R15_uid177925	NC_018876	chromosome		arCOG04923	1 (-)	G	GntT	H+/gluconate symporter or related permease	0.00000001	COG2610	H+/gluconate symporter and related perme
410671787	hypothetical protein Mpsy_2587	No	0		2482859	2483476	-		Methanolobus_psychrophilus_R15_uid177925	NC_018876	chromosome									
410671788	hypothetical protein Mpsy_2588	No	0		2483487	2484113	-		Methanolobus_psychrophilus_R15_uid177925	NC_018876	chromosome									
410671789	hypothetical protein Mpsy_2589	No	0		2484235	2484786	-		Methanolobus_psychrophilus_R15_uid177925	NC_018876	chromosome									
===								12	Methanolobus_psychrophilus_R15_uid177925	NC_018876	chromosome									
410671982	hypothetical protein Mpsy_2784	No	0		2663716	2663865	+		Methanolobus_psychrophilus_R15_uid177925	NC_018876	chromosome									
410671983	integrase family protein	No	0		2664026	2665225	+		Methanolobus_psychrophilus_R15_uid177925	NC_018876	chromosome		arCOG01242	14 (-)	L	XerC	Integrase	0.0000001	pfam00589	Phage integrase family. Members of this
410671984	hypothetical protein Mpsy_2786	No	0		2665639	2665782	+		Methanolobus_psychrophilus_R15_uid177925	NC_018876	chromosome									
410671985	hypothetical protein Mpsy_2787	No	0		2665939	2666055	+		Methanolobus_psychrophilus_R15_uid177925	NC_018876	chromosome									
410671986	hypothetical protein Mpsy_2788	No	0		2666203	2667816	+		Methanolobus_psychrophilus_R15_uid177925	NC_018876	chromosome									
410671987	hypothetical protein Mpsy_2789	No	0		2668530	2669246	+		Methanolobus_psychrophilus_R15_uid177925	NC_018876	chromosome		arCOG00905	2 (-)	L		Uracil-DNA glycosylase			
410671988	hypothetical protein Mpsy_2790	No	0		2669272	2670132	+		Methanolobus_psychrophilus_R15_uid177925	NC_018876	chromosome							4E-14	pfam14267	Domain of unknown function (DUF4357). Th
410671989	hypothetical protein Mpsy_2791	No	0		2670189	2670320	+		Methanolobus_psychrophilus_R15_uid177925	NC_018876	chromosome									
410671990	hypothetical protein Mpsy_2792	No	0		2670370	2670495	+		Methanolobus_psychrophilus_R15_uid177925	NC_018876	chromosome									
410671991	hypothetical protein Mpsy_2793	No	0		2671216	2671596	+		Methanolobus_psychrophilus_R15_uid177925	NC_018876	chromosome									
410671992	hypothetical protein Mpsy_2794	No	0		2671911	2672795	+		Methanolobus_psychrophilus_R15_uid177925	NC_018876	chromosome		arCOG00744	1 (-)	K		Transcriptional regulator containing HTH domain			
410671993	hypothetical protein Mpsy_2795	No	0		2672827	2673744	+		Methanolobus_psychrophilus_R15_uid177925	NC_018876	chromosome									
===								15	Methanolobus_psychrophilus_R15_uid177925	NC_018876	chromosome									
410672318	hypothetical protein Mpsy_3122	No	0		3010740	3012101	+		Methanolobus_psychrophilus_R15_uid177925	NC_018876	chromosome		arCOG01245	15 (-)	L	XerC	Integrase			
410672319	hypothetical protein Mpsy_3123	No	0		3012356	3012475	-		Methanolobus_psychrophilus_R15_uid177925	NC_018876	chromosome									
410672320	hypothetical protein Mpsy_3124	No	0		3012529	3012669	+		Methanolobus_psychrophilus_R15_uid177925	NC_018876	chromosome									
410672321	hypothetical protein Mpsy_3125	No	0		3012835	3012975	+		Methanolobus_psychrophilus_R15_uid177925	NC_018876	chromosome									
410672322	hypothetical protein Mpsy_3126	No	0		3012972	3013484	+		Methanolobus_psychrophilus_R15_uid177925	NC_018876	chromosome									
410672323	phage/plasmid primase, P4 family	No	0		3013630	3015282	+		Methanolobus_psychrophilus_R15_uid177925	NC_018876	chromosome		arCOG06914	10 (-)	R		Phage or plasmid associated DNA primase	4E-29	COG3378	Phage associated DNA primase
410672324	hypothetical protein Mpsy_3128	No	1		3015242	3015415	+		Methanolobus_psychrophilus_R15_uid177925	NC_018876	chromosome									
410672325	hypothetical protein Mpsy_3129	No	0		3016361	3016645	+		Methanolobus_psychrophilus_R15_uid177925	NC_018876	chromosome									
410672326	hypothetical protein Mpsy_3130	No	0		3017304	3017810	+		Methanolobus_psychrophilus_R15_uid177925	NC_018876	chromosome									
410672327	hypothetical protein Mpsy_3131	No	0		3017981	3018127	+		Methanolobus_psychrophilus_R15_uid177925	NC_018876	chromosome									
410672328	hypothetical protein Mpsy_3132	No	0		3018117	3018284	+		Methanolobus_psychrophilus_R15_uid177925	NC_018876	chromosome									
410672329	hypothetical protein Mpsy_3133	No	1		3018470	3018619	-		Methanolobus_psychrophilus_R15_uid177925	NC_018876	chromosome									
410672330	GLUG domain protein	No	2		3019151	3020089	-		Methanolobus_psychrophilus_R15_uid177925	NC_018876	chromosome		arCOG10954	1 (-)	S		Uncharacterized conserved protein			
410672331	hypothetical protein Mpsy_3135	No	2		3020263	3020529	+		Methanolobus_psychrophilus_R15_uid177925	NC_018876	chromosome									
410672332	hypothetical protein Mpsy_3136	No	2		3020526	3020732	+		Methanolobus_psychrophilus_R15_uid177925	NC_018876	chromosome									
===								6	Methanolobus_psychrophilus_R15_uid177925	NC_018876	chromosome									
410672341	hypothetical protein Mpsy_3145	No	0		3027845	3027973	-		Methanolobus_psychrophilus_R15_uid177925	NC_018876	chromosome									
410672342	hypothetical protein Mpsy_3146	Yes	0		3027970	3028083	-		Methanolobus_psychrophilus_R15_uid177925	NC_018876	chromosome									
410672343	hypothetical protein Mpsy_3147	No	0		3028275	3029060	+		Methanolobus_psychrophilus_R15_uid177925	NC_018876	chromosome		arCOG03134	4 (-)	K		Predicted transcriptional regulator	6E-52	COG4742	Predicted transcriptional regulator
410672344	hypothetical protein Mpsy_3148	No	0		3029467	3030162	+		Methanolobus_psychrophilus_R15_uid177925	NC_018876	chromosome									
410672345	hypothetical protein Mpsy_3149	No	0		3030425	3031381	-		Methanolobus_psychrophilus_R15_uid177925	NC_018876	chromosome									
410672346	hypothetical protein Mpsy_3150	No	0		3031723	3031866	-		Methanolobus_psychrophilus_R15_uid177925	NC_018876	chromosome	clust_501								
===								13	Methanomethylovorans_hollandica_DSM_15978_	NC_019972	plasmid pMETHO01									
435849695	hypothetical protein Metho_2468	No	0	plasmid	7043	7411	-		Methanomethylovorans_hollandica_DSM_15978_	NC_019972	plasmid pMETHO01									
435849696	hypothetical protein Metho_2469	No	0	plasmid	7516	7935	-		Methanomethylovorans_hollandica_DSM_15978_	NC_019972	plasmid pMETHO01									
435849697	hypothetical protein Metho_2470	No	0	plasmid	8049	8534	-		Methanomethylovorans_hollandica_DSM_15978_	NC_019972	plasmid pMETHO01							9E-11	COG4474	Uncharacterized protein conserved in bac
435849698	ribonuclease HII	No	0	plasmid	9109	10080	-		Methanomethylovorans_hollandica_DSM_15978_	NC_019972	plasmid pMETHO01		arCOG04121	1 (-)	L	RnhB	Ribonuclease HII	1E-36	COG1039	Ribonuclease HIII
435849699	hypothetical protein Metho_2472	No	1	plasmid	10108	10236	-		Methanomethylovorans_hollandica_DSM_15978_	NC_019972	plasmid pMETHO01									
435849700	hypothetical protein Metho_2473	No	0	plasmid	10256	10561	-		Methanomethylovorans_hollandica_DSM_15978_	NC_019972	plasmid pMETHO01									
435849701	hypothetical protein Metho_2474	No	0	plasmid	10690	10995	-		Methanomethylovorans_hollandica_DSM_15978_	NC_019972	plasmid pMETHO01									
435849702	transposase, IS605 OrfB family, central region	No	0	plasmid	11259	12605	-		Methanomethylovorans_hollandica_DSM_15978_	NC_019972	plasmid pMETHO01		arCOG00684	5 (-)	L		Transposase	2E-14	COG0675	Transposase and inactivated derivatives
435849703	hypothetical protein Metho_2476	No	0	plasmid	12829	13074	-		Methanomethylovorans_hollandica_DSM_15978_	NC_019972	plasmid pMETHO01									
435849704	transposase, IS605 OrfB family, central region	No	0	plasmid	13609	14943	-		Methanomethylovorans_hollandica_DSM_15978_	NC_019972	plasmid pMETHO01		arCOG00684	5 (-)	L		Transposase	1E-14	COG0675	Transposase and inactivated derivatives
435849705	hypothetical protein Metho_2478	No	0	plasmid	15532	15762	-		Methanomethylovorans_hollandica_DSM_15978_	NC_019972	plasmid pMETHO01									
435849706	hypothetical protein Metho_2479	No	0	plasmid	15808	16227	-		Methanomethylovorans_hollandica_DSM_15978_	NC_019972	plasmid pMETHO01									
435849707	hypothetical protein Metho_2480	No	0	plasmid	19475	21481	+		Methanomethylovorans_hollandica_DSM_15978_	NC_019972	plasmid pMETHO0	clust_24								
===								20	Methanomethylovorans_hollandica_DSM_15978_	NC_019972	plasmid pMETHO01									
435849710	Cdc6-related protein, AAA superfamily ATPase	No	0	plasmid	25937	27088	-		Methanomethylovorans_hollandica_DSM_15978_	NC_019972	plasmid pMETHO01		arCOG00467	13 (-)	L	CDC6	Cdc6-related protein, AAA superfamily ATPase	4E-32	COG1474	Cdc6-related protein, AAA superfamily AT
435849711	hypothetical protein Metho_2484	No	1	plasmid	27353	27559	-		Methanomethylovorans_hollandica_DSM_15978_	NC_019972	plasmid pMETHO01									
435849712	hypothetical protein Metho_2485	No	1	plasmid	27556	27861	-		Methanomethylovorans_hollandica_DSM_15978_	NC_019972	plasmid pMETHO01									
435849713	hypothetical protein Metho_2486	No	1	plasmid	27870	28094	-		Methanomethylovorans_hollandica_DSM_15978_	NC_019972	plasmid pMETHO01									
435849714	hypothetical protein Metho_2487	No	1	plasmid	28100	28288	-		Methanomethylovorans_hollandica_DSM_15978_	NC_019972	plasmid pMETHO01									
435849715	hypothetical protein Metho_2488	No	1	plasmid	28288	28491	-		Methanomethylovorans_hollandica_DSM_15978_	NC_019972	plasmid pMETHO01									
435849716	hypothetical protein Metho_2489	No	1	plasmid	28488	28721	-		Methanomethylovorans_hollandica_DSM_15978_	NC_019972	plasmid pMETHO01									
435849717	hypothetical protein Metho_2490	No	1	plasmid	28731	28964	-		Methanomethylovorans_hollandica_DSM_15978_	NC_019972	plasmid pMETHO0	clust_50								
435849718	hypothetical protein Metho_2491	No	1	plasmid	29021	29266	-		Methanomethylovorans_hollandica_DSM_15978_	NC_019972	plasmid pMETHO0	clust_50								
435849719	hypothetical protein Metho_2492	No	1	plasmid	29461	29661	-		Methanomethylovorans_hollandica_DSM_15978_	NC_019972	plasmid pMETHO01									
435849720	hypothetical protein Metho_2493	No	1	plasmid	29680	29958	-		Methanomethylovorans_hollandica_DSM_15978_	NC_019972	plasmid pMETHO0	clust_50								
435849721	hypothetical protein Metho_2494	No	0	plasmid	30414	33944	+		Methanomethylovorans_hollandica_DSM_15978_	NC_019972	plasmid pMETHO01		arCOG00524	1 (-)	C	HemG	Flavodoxin			
435849722	hypothetical protein Metho_2495	No	0	plasmid	33941	34879	+		Methanomethylovorans_hollandica_DSM_15978_	NC_019972	plasmid pMETHO01									
435849723	hypothetical protein Metho_2496	No	0	plasmid	34876	35190	+		Methanomethylovorans_hollandica_DSM_15978_	NC_019972	plasmid pMETHO01									
435849724	hypothetical protein Metho_2497	No	14	plasmid	35226	36662	-		Methanomethylovorans_hollandica_DSM_15978_	NC_019972	plasmid pMETHO01									
435849725	hypothetical protein Metho_2498	Yes	1	plasmid	36749	37450	-		Methanomethylovorans_hollandica_DSM_15978_	NC_019972	plasmid pMETHO01									
435849726	hypothetical protein Metho_2499	No	3	plasmid	37450	37848	-		Methanomethylovorans_hollandica_DSM_15978_	NC_019972	plasmid pMETHO01									
435849727	hypothetical protein Metho_2500	No	5	plasmid	37858	38481	-		Methanomethylovorans_hollandica_DSM_15978_	NC_019972	plasmid pMETHO01									
435849728	hypothetical protein Metho_2501	No	1	plasmid	38495	39001	-		Methanomethylovorans_hollandica_DSM_15978_	NC_019972	plasmid pMETHO01									
435849729	hypothetical protein Metho_2502	No	0	plasmid	38994	39581	-		Methanomethylovorans_hollandica_DSM_15978_	NC_019972	plasmid pMETHO01		arCOG00833	1 (-)	R	RimI	Acetyltransferase (GNAT) family			
===								5	Methanomethylovorans_hollandica_DSM_15978_	NC_019972	plasmid pMETHO01									
435849732	hypothetical protein Metho_2505	No	0	plasmid	44027	44545	-		Methanomethylovorans_hollandica_DSM_15978_	NC_019972	plasmid pMETHO01									
435849733	hypothetical protein Metho_2506	No	1	plasmid	44882	45055	+		Methanomethylovorans_hollandica_DSM_15978_	NC_019972	plasmid pMETHO01									
435849734	hypothetical protein Metho_2507	No	1	plasmid	45075	45347	+		Methanomethylovorans_hollandica_DSM_15978_	NC_019972	plasmid pMETHO01									
435849735	topoisomerase IA	No	0	plasmid	45353	47500	-		Methanomethylovorans_hollandica_DSM_15978_	NC_019972	plasmid pMETHO01		arCOG01527	2 (-)	L	TopA	Topoisomerase IA	1E-20	COG0550	Topoisomerase IA
435849736	hypothetical protein Metho_2509	Yes	1	plasmid	47554	48825	-		Methanomethylovorans_hollandica_DSM_15978_	NC_019972	plasmid pMETHO01									
===								13	Methanomethylovorans_hollandica_DSM_15978_	NC_019972	plasmid pMETHO01									
435849739	hypothetical protein Metho_2512	No	8	plasmid	51317	53230	+		Methanomethylovorans_hollandica_DSM_15978_	NC_019972	plasmid pMETHO01									
435849740	hypothetical protein Metho_2514	No	0	plasmid	54320	54469	-		Methanomethylovorans_hollandica_DSM_15978_	NC_019972	plasmid pMETHO01									
435849741	putative ATPase	No	0	plasmid	54614	57706	-		Methanomethylovorans_hollandica_DSM_15978_	NC_019972	plasmid pMETHO01		arCOG00286	1 (-)	L		HerA helicase	3E-11	pfam12846	AAA-like domain. This family of domains
435849742	hypothetical protein Metho_2516	No	5	plasmid	57782	58405	-		Methanomethylovorans_hollandica_DSM_15978_	NC_019972	plasmid pMETHO01									
435849743	hypothetical protein Metho_2517	No	1	plasmid	58496	58672	+		Methanomethylovorans_hollandica_DSM_15978_	NC_019972	plasmid pMETHO01									
435849744	hypothetical protein Metho_2518	No	1	plasmid	58669	58926	+		Methanomethylovorans_hollandica_DSM_15978_	NC_019972	plasmid pMETHO01									
435849745	hypothetical protein Metho_2519	No	1	plasmid	58883	59143	+		Methanomethylovorans_hollandica_DSM_15978_	NC_019972	plasmid pMETHO01									
435849746	hypothetical protein Metho_2520	No	1	plasmid	59155	59742	+		Methanomethylovorans_hollandica_DSM_15978_	NC_019972	plasmid pMETHO01									
435849747	hypothetical protein Metho_2521	No	1	plasmid	59764	60300	+		Methanomethylovorans_hollandica_DSM_15978_	NC_019972	plasmid pMETHO01									
435849748	hypothetical protein Metho_2522	Yes	1	plasmid	60309	61112	+		Methanomethylovorans_hollandica_DSM_15978_	NC_019972	plasmid pMETHO01									
435849749	hypothetical protein Metho_2523	No	1	plasmid	61117	61608	+		Methanomethylovorans_hollandica_DSM_15978_	NC_019972	plasmid pMETHO01									
435849750	ATPase, type IV secretory pathway VirB11 con	No	0	plasmid	61624	64119	+		Methanomethylovorans_hollandica_DSM_15978_	NC_019972	plasmid pMETHO01		arCOG01817	8 (-)	N	VirB11	Type IV secretory pathway, VirB11 component, o	0.00000001	COG4962	Flp pilus assembly protein, ATPase CpaF
435849751	hypothetical protein Metho_2525	No	0	plasmid	64132	64668	+		Methanomethylovorans_hollandica_DSM_15978_	NC_019972	plasmid pMETHO01									
===								5	Methanomethylovorans_hollandica_DSM_15978_	NC_019972	plasmid pMETHO01									
435849779	hypothetical protein Metho_2553	No	2	plasmid	92048	92290	-		Methanomethylovorans_hollandica_DSM_15978_	NC_019972	plasmid pMETHO01									
435849780	hypothetical protein Metho_2554	No	5	plasmid	92399	92872	+		Methanomethylovorans_hollandica_DSM_15978_	NC_019972	plasmid pMETHO01		arCOG03976	1	S		Uncharacterized conserved protein			
435849781	uncharacterized membrane protein	No	4	plasmid	93005	93574	-		Methanomethylovorans_hollandica_DSM_15978_	NC_019972	plasmid pMETHO01		arCOG01996	17 (-)	S	SpoIIM	Uncharacterized membrane protein	1E-13	COG1300	Uncharacterized membrane protein
435849782	hypothetical protein Metho_2556	No	2	plasmid	93855	94055	-		Methanomethylovorans_hollandica_DSM_15978_	NC_019972	plasmid pMETHO0	clust_457								
435849783	hypothetical protein Metho_2557	No	2	plasmid	94453	94653	-		Methanomethylovorans_hollandica_DSM_15978_	NC_019972	plasmid pMETHO0	clust_457								
===								12	Methanomethylovorans_hollandica_DSM_15978_	NC_019972	plasmid pMETHO01									
435849856	hypothetical protein Metho_2639	No	0	plasmid	196989	197591	-		Methanomethylovorans_hollandica_DSM_15978_	NC_019972	plasmid pMETHO01									
435849857	putative HD superfamily hydrolase	No	0	plasmid	197588	198226	-		Methanomethylovorans_hollandica_DSM_15978_	NC_019972	plasmid pMETHO01		arCOG01861	3 (-)	H	MptB	Methanopterin biosynthesis cyclic phosphodiesterase, HD-superfamily hydrolase			
435849858	hypothetical protein Metho_2641	No	0	plasmid	198265	198606	-		Methanomethylovorans_hollandica_DSM_15978_	NC_019972	plasmid pMETHO01									
435849859	hypothetical protein Metho_2642	No	0	plasmid	198674	199393	+		Methanomethylovorans_hollandica_DSM_15978_	NC_019972	plasmid pMETHO01									
435849860	hypothetical protein Metho_2643	No	0	plasmid	199495	199839	-		Methanomethylovorans_hollandica_DSM_15978_	NC_019972	plasmid pMETHO01									
435849861	hypothetical protein Metho_2644	No	0	plasmid	199907	200137	-		Methanomethylovorans_hollandica_DSM_15978_	NC_019972	plasmid pMETHO01									
435849862	outer membrane protein	No	2	plasmid	200177	202258	-		Methanomethylovorans_hollandica_DSM_15978_	NC_019972	plasmid pMETHO0	arCOG02538,a	arCOG02538	1 (-)	S		PKD repeats containing protein			
435849863	hypothetical protein Metho_2646	No	2	plasmid	202523	202756	-		Methanomethylovorans_hollandica_DSM_15978_	NC_019972	plasmid pMETHO01									
435849864	hypothetical protein Metho_2647	No	0	plasmid	202799	203107	-		Methanomethylovorans_hollandica_DSM_15978_	NC_019972	plasmid pMETHO01									
435849865	hypothetical protein Metho_2648	No	0	plasmid	203117	203410	-		Methanomethylovorans_hollandica_DSM_15978_	NC_019972	plasmid pMETHO01									
435849866	hypothetical protein Metho_2649	No	0	plasmid	203460	204116	-		Methanomethylovorans_hollandica_DSM_15978_	NC_019972	plasmid pMETHO01		arCOG02967	3 (-)	T		NACHT family NTPase fused to HEAT repeats domain			
435849867	hypothetical protein Metho_2650	No	0	plasmid	204192	204731	-		Methanomethylovorans_hollandica_DSM_15978_	NC_019972	plasmid pMETHO01									
===								9	Methanomethylovorans_hollandica_DSM_15978_	NC_019972	plasmid pMETHO01									
435849873	hypothetical protein Metho_2656	No	0	plasmid	214770	216056	-		Methanomethylovorans_hollandica_DSM_15978_	NC_019972	plasmid pMETHO01									
435849874	hypothetical protein Metho_2657	No	2	plasmid	216208	216459	-		Methanomethylovorans_hollandica_DSM_15978_	NC_019972	plasmid pMETHO01									
435849875	hypothetical protein Metho_2658	No	0	plasmid	216456	217283	-		Methanomethylovorans_hollandica_DSM_15978_	NC_019972	plasmid pMETHO01		arCOG02610	2 (-)	L		Rec8/ScpA/Scc1-like protein (kleisin family)	6E-16	COG1354	Rec8/ScpA/Scc1-like protein (kleisin fam
435849876	hypothetical protein Metho_2659	No	0	plasmid	217338	217844	-		Methanomethylovorans_hollandica_DSM_15978_	NC_019972	plasmid pMETHO01									
435849877	hypothetical protein Metho_2660	No	0	plasmid	218025	219995	+		Methanomethylovorans_hollandica_DSM_15978_	NC_019972	plasmid pMETHO01		arCOG02974	4	S		HEAT repeats containing protein	0.00000002	COG1196	Chromosome segregation ATPases
435849878	hypothetical protein Metho_2661	No	0	plasmid	220217	220798	+		Methanomethylovorans_hollandica_DSM_15978_	NC_019972	plasmid pMETHO01									
435849879	hypothetical protein Metho_2662	No	0	plasmid	220807	221550	-		Methanomethylovorans_hollandica_DSM_15978_	NC_019972	plasmid pMETHO01									
435849880	hypothetical protein Metho_2663	No	0	plasmid	221561	222166	-		Methanomethylovorans_hollandica_DSM_15978_	NC_019972	plasmid pMETHO01									
435849881	hypothetical protein Metho_2664	No	0	plasmid	222124	223470	-		Methanomethylovorans_hollandica_DSM_15978_	NC_019972	plasmid pMETHO01		arCOG07894	2 (-)	S		Uncharacterized protein			
===								8	Methanomethylovorans_hollandica_DSM_15978_	NC_019972	plasmid pMETHO01									
435849898	hypothetical protein Metho_2681	Yes	0	plasmid	242411	243262	-		Methanomethylovorans_hollandica_DSM_15978_	NC_019972	plasmid pMETHO01									
435849899	Protein of unknown function (DUF1699)	No	0	plasmid	243314	243727	-		Methanomethylovorans_hollandica_DSM_15978_	NC_019972	plasmid pMETHO01		arCOG03353	2 (-)	S		Uncharacterized conserved protein	1E-13	pfam08004	Protein of unknown function (DUF1699). T
435849900	hypothetical protein Metho_2683	No	1	plasmid	243846	244013	-		Methanomethylovorans_hollandica_DSM_15978_	NC_019972	plasmid pMETHO01									
435849901	hypothetical protein Metho_2684	No	1	plasmid	244217	244372	+		Methanomethylovorans_hollandica_DSM_15978_	NC_019972	plasmid pMETHO01									
435849902	hypothetical protein Metho_2685	No	2	plasmid	244354	244590	+		Methanomethylovorans_hollandica_DSM_15978_	NC_019972	plasmid pMETHO01									
435849903	hypothetical protein Metho_2686	Yes	1	plasmid	245077	245949	-		Methanomethylovorans_hollandica_DSM_15978_	NC_019972	plasmid pMETHO01									
435849904	hypothetical protein Metho_2687	No	0	plasmid	246118	246399	-		Methanomethylovorans_hollandica_DSM_15978_	NC_019972	plasmid pMETHO01									
435849905	transposase	No	0	plasmid	246557	247768	-		Methanomethylovorans_hollandica_DSM_15978_	NC_019972	plasmid pMETHO01		arCOG00684	5 (-)	L		Transposase	2E-54	COG0675	Transposase and inactivated derivatives
===								8	Methanomethylovorans_hollandica_DSM_15978_	NC_019972	plasmid pMETHO01									
435849908	hypothetical protein Metho_2691	No	0	plasmid	250241	251554	-		Methanomethylovorans_hollandica_DSM_15978_	NC_019972	plasmid pMETHO01									
435849909	HEAT repeat-containing protein	No	0	plasmid	251853	254372	+		Methanomethylovorans_hollandica_DSM_15978_	NC_019972	plasmid pMETHO0	arCOG00560,a	arCOG00560	2 (-)	L	RecQ	Superfamily II DNA helicase	4E-24	COG1413	FOG: HEAT repeat
435849910	hypothetical protein Metho_2693	No	2	plasmid	254585	254824	-		Methanomethylovorans_hollandica_DSM_15978_	NC_019972	plasmid pMETHO01									
435849911	hypothetical protein Metho_2694	No	1	plasmid	255031	255282	-		Methanomethylovorans_hollandica_DSM_15978_	NC_019972	plasmid pMETHO01									
435849912	Peptidase C13 family	No	0	plasmid	255996	258410	-		Methanomethylovorans_hollandica_DSM_15978_	NC_019972	plasmid pMETHO01									
435849913	hypothetical protein Metho_2696	No	0	plasmid	258461	258796	-		Methanomethylovorans_hollandica_DSM_15978_	NC_019972	plasmid pMETHO01									
435849914	ATPase involved in chromosome partitioning	No	0	plasmid	259705	260481	+		Methanomethylovorans_hollandica_DSM_15978_	NC_019972	plasmid pMETHO01		arCOG00586	8 (-)	D	Soj	ATPase involved in chromosome partitioning, Pa	2E-27	COG1192	ATPases involved in chromosome partition
435849915	hypothetical protein Metho_2698	No	0	plasmid	260474	261337	+		Methanomethylovorans_hollandica_DSM_15978_	NC_019972	plasmid pMETHO01		arCOG09449	1	S		Uncharacterized conserved protein			
===								15	Methanomethylovorans_hollandica_DSM_15978_	NC_019972	plasmid pMETHO01									
435849917	DNA-methyltransferase Dcm	No	0	plasmid	264298	265365	-		Methanomethylovorans_hollandica_DSM_15978_	NC_019972	plasmid pMETHO01		arCOG04157	7 (-)	L	Dcm	Site-specific DNA methylase	2E-50	pfam00145	C-5 cytosine-specific DNA methylase.
435849918	hypothetical protein Metho_2702	No	2	plasmid	265534	265830	-		Methanomethylovorans_hollandica_DSM_15978_	NC_019972	plasmid pMETHO01									
435849919	hypothetical protein Metho_2703	No	0	plasmid	266116	266544	-		Methanomethylovorans_hollandica_DSM_15978_	NC_019972	plasmid pMETHO01									
435849920	hypothetical protein Metho_2704	No	0	plasmid	266705	266989	-		Methanomethylovorans_hollandica_DSM_15978_	NC_019972	plasmid pMETHO01									
435849921	hypothetical protein Metho_2705	No	0	plasmid	267319	267972	-		Methanomethylovorans_hollandica_DSM_15978_	NC_019972	plasmid pMETHO01									
435849922	hypothetical protein Metho_2706	No	0	plasmid	268007	268357	-		Methanomethylovorans_hollandica_DSM_15978_	NC_019972	plasmid pMETHO01									
435849923	hypothetical protein Metho_2707	No	0	plasmid	268396	269088	-		Methanomethylovorans_hollandica_DSM_15978_	NC_019972	plasmid pMETHO01		arCOG09587	1 (-)	S		Uncharacterized conserved protein			
435849924	DNA modification methylase	No	0	plasmid	269148	270062	-		Methanomethylovorans_hollandica_DSM_15978_	NC_019972	plasmid pMETHO01		arCOG00115	10 (-)	L		DNA modification methylase	0.00000009	COG0863	DNA modification methylase
435849925	hypothetical protein Metho_2709	No	0	plasmid	270123	270953	-		Methanomethylovorans_hollandica_DSM_15978_	NC_019972	plasmid pMETHO01									
435849926	hypothetical protein Metho_2710	No	1	plasmid	271202	271411	-		Methanomethylovorans_hollandica_DSM_15978_	NC_019972	plasmid pMETHO01									
435849927	hypothetical protein Metho_2711	No	0	plasmid	271486	271773	-		Methanomethylovorans_hollandica_DSM_15978_	NC_019972	plasmid pMETHO01									
435849928	hypothetical protein Metho_2713	No	0	plasmid	272498	272842	-		Methanomethylovorans_hollandica_DSM_15978_	NC_019972	plasmid pMETHO01									
435849929	hypothetical protein Metho_2714	No	0	plasmid	272877	273113	-		Methanomethylovorans_hollandica_DSM_15978_	NC_019972	plasmid pMETHO01									
435849930	hypothetical protein Metho_2715	No	0	plasmid	273225	274253	+		Methanomethylovorans_hollandica_DSM_15978_	NC_019972	plasmid pMETHO01									
435849931	hypothetical protein Metho_2716	No	0	plasmid	274273	274650	-		Methanomethylovorans_hollandica_DSM_15978_	NC_019972	plasmid pMETHO01		arCOG01009	8 (-)	V		Ribbon-helix-helix protein, copG family			
===								6	Methanomethylovorans_hollandica_DSM_15978_	NC_019977	chromosome									
435850817	copper/silver-translocating P-type ATPase,hea	No	7		624652	627003	+		Methanomethylovorans_hollandica_DSM_15978_	NC_019977	chromosome	arCOG01576,a	arCOG01576	3 (-)	P	ZntA	Cation transport ATPase	1E-179	COG2217	Cation transport ATPase
435850818	hypothetical protein Metho_0615	No	0		626844	627179	-		Methanomethylovorans_hollandica_DSM_15978_	NC_019977	chromosome									
435850819	hypothetical protein Metho_0616	Yes	1		627193	627351	-		Methanomethylovorans_hollandica_DSM_15978_	NC_019977	chromosome									
435850820	hypothetical protein Metho_0617	No	0		627545	627826	-		Methanomethylovorans_hollandica_DSM_15978_	NC_019977	chromosome									
435850821	hypothetical protein Metho_0618	Yes	1		628081	628665	+		Methanomethylovorans_hollandica_DSM_15978_	NC_019977	chromosome									
435850822	26S proteasome subunit P45 family	No	0		629142	630380	-		Methanomethylovorans_hollandica_DSM_15978_	NC_019977	chromosome		arCOG01306	1 (-)	O	RPT1	ATP-dependent 26S proteasome regulatory subu	2E-126	COG1222	ATP-dependent 26S proteasome regulatory
===								9	Methanomethylovorans_hollandica_DSM_15978_	NC_019977	chromosome									
435850877	hypothetical protein Metho_0677	No	0		689828	690022	+		Methanomethylovorans_hollandica_DSM_15978_	NC_019977	chromosome									
435850878	hypothetical protein Metho_0678	No	0		690026	690340	+		Methanomethylovorans_hollandica_DSM_15978_	NC_019977	chromosome		arCOG10647	2 (-)	S		Uncharacterized conserved protein			
435850879	hypothetical protein Metho_0679	No	1		690344	690460	+		Methanomethylovorans_hollandica_DSM_15978_	NC_019977	chromosome									
435850880	hypothetical protein Metho_0680	No	0		690640	690795	+		Methanomethylovorans_hollandica_DSM_15978_	NC_019977	chromosome									
435850881	hypothetical protein Metho_0681	No	0		691035	691295	+		Methanomethylovorans_hollandica_DSM_15978_	NC_019977	chromosome									
435850882	DNA primase	No	0		691292	692341	+		Methanomethylovorans_hollandica_DSM_15978_	NC_019977	chromosome							1E-16	COG0358	DNA primase (bacterial type)
435850883	phage/plasmid primase, P4 family, C-terminal (	No	0		692345	694876	+		Methanomethylovorans_hollandica_DSM_15978_	NC_019977	chromosome		arCOG06914	10 (-)	R		Phage or plasmid associated DNA primase	2E-24	COG3378	Phage associated DNA primase
435850884	hypothetical protein Metho_0685	No	0		695225	695506	+		Methanomethylovorans_hollandica_DSM_15978_	NC_019977	chromosome		arCOG10959	8	S		Uncharacterized conserved protein			
435850885	hypothetical protein Metho_0686	No	0		695512	695805	+		Methanomethylovorans_hollandica_DSM_15978_	NC_019977	chromosome									
===								55	Methanomethylovorans_hollandica_DSM_15978_	NC_019977	chromosome									
435851364	hypothetical protein Metho_1190	No	0	Virus_Caudovi	1194285	1194449	+		Methanomethylovorans_hollandica_DSM_15978_	NC_019977	chromosome		arCOG01009	8 (-)	V		Ribbon-helix-helix protein, copG family			
435851365	hypothetical protein Metho_1191	No	0	Virus_Caudovi	1194427	1194675	+		Methanomethylovorans_hollandica_DSM_15978_	NC_019977	chromosome									
435851366	hypothetical protein Metho_1192	No	0	Virus_Caudovi	1194681	1194878	+		Methanomethylovorans_hollandica_DSM_15978_	NC_019977	chromosome									
435851367	hypothetical protein Metho_1193	No	0	Virus_Caudovi	1194995	1195153	+		Methanomethylovorans_hollandica_DSM_15978_	NC_019977	chromosome									
435851368	transcription initiation factor TFIIIB, Brf1 subunit/	No	0	Virus_Caudovi	1195341	1195658	+		Methanomethylovorans_hollandica_DSM_15978_	NC_019977	chromosome		arCOG10941	4	S		Uncharacterized conserved protein			
435851369	hypothetical protein Metho_1195	No	0	Virus_Caudovi	1195827	1196168	+		Methanomethylovorans_hollandica_DSM_15978_	NC_019977	chromosome									
435851370	hypothetical protein Metho_1196	Yes	3	Virus_Caudovi	1196184	1196447	+		Methanomethylovorans_hollandica_DSM_15978_	NC_019977	chromosome									
435851371	hypothetical protein Metho_1197	No	0	Virus_Caudovi	1196462	1196776	+		Methanomethylovorans_hollandica_DSM_15978_	NC_019977	chromosome									
435851372	hypothetical protein Metho_1198	No	0	Virus_Caudovi	1196773	1197147	+		Methanomethylovorans_hollandica_DSM_15978_	NC_019977	chromosome									
435851373	phage recombination protein Bet	No	0	Virus_Caudovi	1197153	1198037	+		Methanomethylovorans_hollandica_DSM_15978_	NC_019977	chromosome							9E-21	pfam03837	RecT family. The DNA single-strand annea
435851374	hypothetical protein Metho_1200	No	0	Virus_Caudovi	1198190	1198372	+		Methanomethylovorans_hollandica_DSM_15978_	NC_019977	chromosome									
435851375	hypothetical protein Metho_1201	No	0	Virus_Caudovi	1198369	1198557	+		Methanomethylovorans_hollandica_DSM_15978_	NC_019977	chromosome									
435851376	hypothetical protein Metho_1202	No	0	Virus_Caudovi	1198558	1199121	+		Methanomethylovorans_hollandica_DSM_15978_	NC_019977	chromosome									
435851377	hypothetical protein Metho_1203	No	0	Virus_Caudovi	1199394	1199600	+		Methanomethylovorans_hollandica_DSM_15978_	NC_019977	chromosome									
435851378	phage uncharacterized protein TIGR01671	No	0	Virus_Caudovi	1199587	1200006	+		Methanomethylovorans_hollandica_DSM_15978_	NC_019977	chromosome							3E-16	pfam09643	YopX protein. YopX is a protein that is
435851379	hypothetical protein Metho_1205	No	0	Virus_Caudovi	1200003	1200173	+		Methanomethylovorans_hollandica_DSM_15978_	NC_019977	chromosome									
435851380	hypothetical protein Metho_1206	No	0	Virus_Caudovi	1200170	1200442	+		Methanomethylovorans_hollandica_DSM_15978_	NC_019977	chromosome									
435851381	hypothetical protein Metho_1207	No	0	Virus_Caudovi	1200607	1201059	+		Methanomethylovorans_hollandica_DSM_15978_	NC_019977	chromosome									
435851382	hypothetical protein Metho_1208	No	0	Virus_Caudovi	1201056	1201430	+		Methanomethylovorans_hollandica_DSM_15978_	NC_019977	chromosome		arCOG01588	1 (-)	E	LysW	lysine biosynthesis protein LysW			
435851383	hypothetical protein Metho_1209	No	0	Virus_Caudovi	1201427	1201627	+		Methanomethylovorans_hollandica_DSM_15978_	NC_019977	chromosome									
435851384	hypothetical protein Metho_1210	No	0	Virus_Caudovi	1201624	1202073	+		Methanomethylovorans_hollandica_DSM_15978_	NC_019977	chromosome		arCOG11382	5	Y		Phage terminase small subunit			
435851385	putative phage protein, putative large terminas	No	0	Virus_Caudovi	1202018	1204546	+		Methanomethylovorans_hollandica_DSM_15978_	NC_019977	chromosome	arCOG03146,a	arCOG03146	1 (-)	L		Intein/homing endonuclease	2E-13	COG5362	Phage-related terminase
435851386	hypothetical protein Metho_1212	No	0	Virus_Caudovi	1204549	1204782	+		Methanomethylovorans_hollandica_DSM_15978_	NC_019977	chromosome									
435851387	hypothetical protein Metho_1213	No	1	Virus_Caudovi	1204899	1205090	+		Methanomethylovorans_hollandica_DSM_15978_	NC_019977	chromosome									
435851388	hypothetical protein Metho_1214	No	0	Virus_Caudovi	1205098	1206345	+		Methanomethylovorans_hollandica_DSM_15978_	NC_019977	chromosome									
435851389	hypothetical protein Metho_1215	No	0	Virus_Caudovi	1206435	1206728	+		Methanomethylovorans_hollandica_DSM_15978_	NC_019977	chromosome									
435851390	hypothetical protein Metho_1216	No	0	Virus_Caudovi	1206728	1208086	+		Methanomethylovorans_hollandica_DSM_15978_	NC_019977	chromosome									
435851391	hypothetical protein Metho_1217	No	0	Virus_Caudovi	1208091	1208459	+		Methanomethylovorans_hollandica_DSM_15978_	NC_019977	chromosome									
435851392	phage putative head morphogenesis protein, S	No	0	Virus_Caudovi	1208475	1209332	+		Methanomethylovorans_hollandica_DSM_15978_	NC_019977	chromosome		arCOG09510	5 (-)	T		NAD+--asparagine ADP-ribosyltransferase			
435851393	hypothetical protein Metho_1219	No	0	Virus_Caudovi	1209329	1209547	-		Methanomethylovorans_hollandica_DSM_15978_	NC_019977	chromosome									
435851394	hypothetical protein Metho_1220	No	0	Virus_Caudovi	1209611	1210570	+		Methanomethylovorans_hollandica_DSM_15978_	NC_019977	chromosome							0.00007	COG3064	Membrane protein involved in colicin upt
435851395	hypothetical protein Metho_1221	No	0	Virus_Caudovi	1210570	1211034	+		Methanomethylovorans_hollandica_DSM_15978_	NC_019977	chromosome									
435851396	Encapsulating protein for peroxidase	No	0	Virus_Caudovi	1211042	1211962	+		Methanomethylovorans_hollandica_DSM_15978_	NC_019977	chromosome									
435851397	hypothetical protein Metho_1223	No	0	Virus_Caudovi	1211978	1212217	+		Methanomethylovorans_hollandica_DSM_15978_	NC_019977	chromosome									
435851398	hypothetical protein Metho_1224	No	0	Virus_Caudovi	1212223	1212621	+		Methanomethylovorans_hollandica_DSM_15978_	NC_019977	chromosome									
435851399	hypothetical protein Metho_1225	No	0	Virus_Caudovi	1212611	1212961	+		Methanomethylovorans_hollandica_DSM_15978_	NC_019977	chromosome									
435851400	hypothetical protein Metho_1226	No	1	Virus_Caudovi	1212958	1213197	+		Methanomethylovorans_hollandica_DSM_15978_	NC_019977	chromosome									
435851401	phage protein, HK97 gp10 family	No	0	Virus_Caudovi	1213197	1213631	+		Methanomethylovorans_hollandica_DSM_15978_	NC_019977	chromosome									
435851402	Protein of unknown function (DUF3168)	Yes	0	Virus_Caudovi	1213628	1214059	+		Methanomethylovorans_hollandica_DSM_15978_	NC_019977	chromosome									
435851403	hypothetical protein Metho_1229	No	0	Virus_Caudovi	1214097	1214894	+		Methanomethylovorans_hollandica_DSM_15978_	NC_019977	chromosome	clust_125								
435851404	hypothetical protein Metho_1230	No	0	Virus_Caudovi	1214900	1215229	+		Methanomethylovorans_hollandica_DSM_15978_	NC_019977	chromosome									
435851405	hypothetical protein Metho_1231	No	0	Virus_Caudovi	1215262	1215513	+		Methanomethylovorans_hollandica_DSM_15978_	NC_019977	chromosome									
435851406	phage tail tape measure protein, TP901 family	No	2	Virus_Caudovi	1215510	1219529	+		Methanomethylovorans_hollandica_DSM_15978_	NC_019977	chromosome		arCOG11109	8 (-)	Y		Phage tail tape measure protein, TP901 family	1E-36	pfam10145	Phage-related minor tail protein. Member ; Phage-related protein
435851407	hypothetical protein Metho_1233	No	0	Virus_Caudovi	1219526	1219930	+		Methanomethylovorans_hollandica_DSM_15978_	NC_019977	chromosome									
435851408	hypothetical protein Metho_1234	No	0	Virus_Caudovi	1219927	1220049	+		Methanomethylovorans_hollandica_DSM_15978_	NC_019977	chromosome									

435851409	hypothetical protein Metho_1235	Yes	0		Virus_Caudovi	1220049	1220420	+		Methanomethylovorans_hollandica_DSM_15978_	NC_019977	chromosome									
435851410	protein of unknown function (DUF2341)	No	0		Virus_Caudovi	1220434	1222743	+		Methanomethylovorans_hollandica_DSM_15978_	NC_019977	chromosome		arCOG03511	3 (-)	S		Uncharacterized conserved protein	1E-14	pfam10102	Domain of unknown function (DUF2341). Me
435851411	hypothetical protein Metho_1237	No	0		Virus_Caudovi	1222740	1223450	+		Methanomethylovorans_hollandica_DSM_15978_	NC_019977	chromosome									
435851412	hypothetical protein Metho_1238	No	0		Virus_Caudovi	1223461	1225161	+		Methanomethylovorans_hollandica_DSM_15978_	NC_019977	chromosome		arCOG03501	1	S		Cell suface protein, contains right handed beta helix region			
435851413	hypothetical protein Metho_1239	No	0		Virus_Caudovi	1225386	1225790	-		Methanomethylovorans_hollandica_DSM_15978_	NC_019977	chromosome									
435851414	hypothetical protein Metho_1240	No	0		Virus_Caudovi	1225787	1225948	-		Methanomethylovorans_hollandica_DSM_15978_	NC_019977	chromosome									
435851415	hypothetical protein Metho_1241	No	0		Virus_Caudovi	1225945	1226406	-		Methanomethylovorans_hollandica_DSM_15978_	NC_019977	chromosome									
435851416	hypothetical protein Metho_1242	No	0		Virus_Caudovi	1226406	1226597	-		Methanomethylovorans_hollandica_DSM_15978_	NC_019977	chromosome									
435851417	hypothetical protein Metho_1243	No	0		Virus_Caudovi	1226634	1226855	-		Methanomethylovorans_hollandica_DSM_15978_	NC_019977	chromosome									
435851418	site-specific recombinase XerD	No	0		Virus_Caudovi	1226926	1228152	-		Methanomethylovorans_hollandica_DSM_15978_	NC_019977	chromosome		arCOG01245	15 (-)	L	XerC	Integrase	0.0000004	COG4974	Site-specific recombinase XerD
===									5	Methanomethylovorans_hollandica_DSM_15978_	NC_019977	chromosome									
435851481	hypothetical protein Metho_1315	No	0			1309885	1310133	+		Methanomethylovorans_hollandica_DSM_15978_	NC_019977	chromosome									
435851482	ORF6N domain-containing protein	No	0			1310205	1310726	+		Methanomethylovorans_hollandica_DSM_15978_	NC_019977	chromosome							1E-13	pfam10543	ORF6N domain. This domain was identified
435851483	restriction endonuclease S subunit	No	0			1310723	1311865	+		Methanomethylovorans_hollandica_DSM_15978_	NC_019977	chromosome		arCOG02628	3 (-)	V	HsdS	Restriction endonuclease S subunit	9E-21	COG0732	Restriction endonuclease S subunits
435851484	hypothetical protein Metho_1318	No	4			1311865	1312416	+		Methanomethylovorans_hollandica_DSM_15978_	NC_019977	chromosome									
435851485	hypothetical protein Metho_1319	No	0			1312413	1313237	+		Methanomethylovorans_hollandica_DSM_15978_	NC_019977	chromosome									
===									12	Methanomethylovorans_hollandica_DSM_15978_	NC_019977	chromosome									
435851756	phage protein D	No	0			1611647	1612732	-		Methanomethylovorans_hollandica_DSM_15978_	NC_019977	chromosome		arCOG10308	5 (-)	S		Uncharacterized conserved protein			
435851757	LysM domain-containing protein	No	0			1612736	1613383	-		Methanomethylovorans_hollandica_DSM_15978_	NC_019977	chromosome									
435851758	hypothetical protein Metho_1620	No	0			1613384	1614397	-		Methanomethylovorans_hollandica_DSM_15978_	NC_019977	chromosome									
435851759	conserved hypothetical phage tail region prote	No	0			1614411	1614848	-		Methanomethylovorans_hollandica_DSM_15978_	NC_019977	chromosome	clust_6						2E-38	pfam06841	T4-like virus tail tube protein gp19. Th
435851760	hypothetical protein Metho_1622	No	0			1614889	1615074	-		Methanomethylovorans_hollandica_DSM_15978_	NC_019977	chromosome									
435851761	hypothetical protein Metho_1623	No	0			1615071	1615436	-		Methanomethylovorans_hollandica_DSM_15978_	NC_019977	chromosome									
435851762	conserved hypothetical phage tail region prote	No	0			1615455	1615889	-		Methanomethylovorans_hollandica_DSM_15978_	NC_019977	chromosome							3E-37	pfam06841	T4-like virus tail tube protein gp19. Th
435851763	phage tail sheath protein FI	No	0			1615939	1617561	-		Methanomethylovorans_hollandica_DSM_15978_	NC_019977	chromosome							9E-23	COG3497	Phage tail sheath protein FI
435851764	hypothetical protein Metho_1626	No	0			1617707	1618306	-		Methanomethylovorans_hollandica_DSM_15978_	NC_019977	chromosome									
435851765	hypothetical protein Metho_1627	No	0			1618311	1618925	-		Methanomethylovorans_hollandica_DSM_15978_	NC_019977	chromosome							1E-17	pfam14065	Protein of unknown function (DUF4255). T
435851766	AAA+ family ATPase	No	0			1618918	1621143	-		Methanomethylovorans_hollandica_DSM_15978_	NC_019977	chromosome		arCOG01308	10 (-)	O	Cdc48	ATPase of the AAA+ class , CDC48 family	4E-23	COG1222	ATP-dependent 26S proteasome regulatory
435851767	hypothetical protein Metho_1629	No	0			1621166	1621486	-		Methanomethylovorans_hollandica_DSM_15978_	NC_019977	chromosome	clust_303								
===									8	Methanoplanus_petrolearius_DSM_11571_uid526	NC_014507	chromosome									
307352218	hypothetical protein Mpet_0050	No	4			62252	62992	-		Methanoplanus_petrolearius_DSM_11571_uid526	NC_014507	chromosome		arCOG06827	7	S		Uncharacterized conserved protein			
307352219	hypothetical protein Mpet_0051	No	0			62989	63435	-		Methanoplanus_petrolearius_DSM_11571_uid526	NC_014507	chromosome		arCOG04915	5 (-)	S		Uncharacterized conserved protein			
307352220	hypothetical protein Mpet_0052	No	6			63697	64446	-		Methanoplanus_petrolearius_DSM_11571_uid526	NC_014507	chromosome									
307352221	hypothetical protein Mpet_0053	No	0			64597	64803	-		Methanoplanus_petrolearius_DSM_11571_uid526	NC_014507	chromosome		arCOG11069	3	S		Uncharacterized conserved protein			
307352222	hypothetical protein Mpet_0054	No	2			64842	65240	-		Methanoplanus_petrolearius_DSM_11571_uid526	NC_014507	chromosome		arCOG11069	3	S		Uncharacterized conserved protein			
307352223	ArsR family transcriptional regulator	No	0			65230	65544	-		Methanoplanus_petrolearius_DSM_11571_uid526	NC_014507	chromosome		arCOG01679	2 (-)	K	ArsR	Transcriptional regulator containing HTH domain,	0.000002	COG1777	Predicted transcriptional regulators
307352224	hypothetical protein Mpet_0056	No	3			65730	66095	-		Methanoplanus_petrolearius_DSM_11571_uid526	NC_014507	chromosome		arCOG09582	2	S		Uncharacterized conserved protein			
307352225	hypothetical protein Mpet_0057	No	2			66228	66500	-		Methanoplanus_petrolearius_DSM_11571_uid526	NC_014507	chromosome									
===									10	Methanoplanus_petrolearius_DSM_11571_uid526	NC_014507	chromosome									
307352403	hypothetical protein Mpet_0238	Yes	1			255786	256934	-		Methanoplanus_petrolearius_DSM_11571_uid526	NC_014507	chromosome		arCOG09593	8	S		Uncharacterized conserved protein			
307352404	hypothetical protein Mpet_0239	No	3			257497	258306	-		Methanoplanus_petrolearius_DSM_11571_uid526	NC_014507	chromosome		arCOG10344	3	S		Uncharacterized conserved protein			
307352405	hypothetical protein Mpet_0240	No	1			258452	259675	-		Methanoplanus_petrolearius_DSM_11571_uid526	NC_014507	chromosome		arCOG02559	3 (-)	S		Cell suface protein			
307352406	hypothetical protein Mpet_0241	No	0			259662	259769	-		Methanoplanus_petrolearius_DSM_11571_uid526	NC_014507	chromosome									
307352407	hypothetical protein Mpet_0242	No	1			259913	260302	-		Methanoplanus_petrolearius_DSM_11571_uid526	NC_014507	chromosome		arCOG07521	6	S		Uncharacterized conserved protein			
307352408	hypothetical protein Mpet_0243	No	1			260352	260753	-		Methanoplanus_petrolearius_DSM_11571_uid526	NC_014507	chromosome									
307352409	hypothetical protein Mpet_0244	No	5			260901	261476	-		Methanoplanus_petrolearius_DSM_11571_uid526	NC_014507	chromosome									
307352410	hypothetical protein Mpet_0245	No	0			261533	262192	-		Methanoplanus_petrolearius_DSM_11571_uid526	NC_014507	chromosome									
307352411	hypothetical protein Mpet_0246	Yes	1			262350	263009	+		Methanoplanus_petrolearius_DSM_11571_uid526	NC_014507	chromosome									
307352412	tetratricopeptide repeat-containing protein	Yes	0			263021	263482	+		Methanoplanus_petrolearius_DSM_11571_uid526	NC_014507	chromosome		arCOG03032	12 (-)	R	NrfG	TPR repeats containing protein			
===									11	Methanoplanus_petrolearius_DSM_11571_uid526	NC_014507	chromosome									
307352612	ABC transporter-like protein	No	6			459586	461328	-		Methanoplanus_petrolearius_DSM_11571_uid526	NC_014507	chromosome		arCOG02841	4 (-)	V	MdlB	ABC-type multidrug transport system, ATPase an	1E-87	COG1132	ABC-type multidrug transport system, ATP
307352613	hypothetical protein Mpet_0452	No	8			461986	462858	-		Methanoplanus_petrolearius_DSM_11571_uid526	NC_014507	chromosome									
307352614	hypothetical protein Mpet_0453	Yes	1			462879	463160	-		Methanoplanus_petrolearius_DSM_11571_uid526	NC_014507	chromosome									
307352615	hypothetical protein Mpet_0454	Yes	0			463573	464097	-		Methanoplanus_petrolearius_DSM_11571_uid526	NC_014507	chromosome		arCOG06822	4	S		Uncharacterized conserved protein			
307352616	hypothetical protein Mpet_0455	No	1			464416	465051	-		Methanoplanus_petrolearius_DSM_11571_uid526	NC_014507	chromosome									
307352617	hypothetical protein Mpet_0456	No	0			465127	466080	-		Methanoplanus_petrolearius_DSM_11571_uid526	NC_014507	chromosome	clust_93								
307352618	hypothetical protein Mpet_0457	No	5			466190	466627	-		Methanoplanus_petrolearius_DSM_11571_uid526	NC_014507	chromosome									
307352619	hypothetical protein Mpet_0458	Yes	1			466635	467474	-		Methanoplanus_petrolearius_DSM_11571_uid526	NC_014507	chromosome									
307352620	hypothetical protein Mpet_0459	No	0			468047	468241	-		Methanoplanus_petrolearius_DSM_11571_uid526	NC_014507	chromosome									
307352621	hypothetical protein Mpet_0460	Yes	2			468257	468991	+		Methanoplanus_petrolearius_DSM_11571_uid526	NC_014507	chromosome		arCOG02611	4 (-)	K		Predicted transcriptional regulator, containd two H	2E-24	COG3398	Uncharacterized protein conserved in arc
307352622	hypothetical protein Mpet_0461	No	1			469259	469876	-		Methanoplanus_petrolearius_DSM_11571_uid526	NC_014507	chromosome									
===									6	Methanoplanus_petrolearius_DSM_11571_uid526	NC_014507	chromosome									
307353013	ABC transporter-like protein	No	0			868863	869786	-		Methanoplanus_petrolearius_DSM_11571_uid526	NC_014507	chromosome		arCOG00194	14 (-)	V	CcmA	ABC-type multidrug transport system, ATPase co	5E-60	COG1131	ABC-type multidrug transport system, ATP
307353014	hypothetical protein Mpet_0856	Yes	1			870047	871168	-		Methanoplanus_petrolearius_DSM_11571_uid526	NC_014507	chromosome		arCOG06558	10	S		Uncharacterized conserved protein			
307353015	hypothetical protein Mpet_0857	Yes	0			871265	872362	-		Methanoplanus_petrolearius_DSM_11571_uid526	NC_014507	chromosome		arCOG06558	10	S		Uncharacterized conserved protein			
307353016	hypothetical protein Mpet_0858	Yes	1			872474	873613	-		Methanoplanus_petrolearius_DSM_11571_uid526	NC_014507	chromosome		arCOG06558	10	S		Uncharacterized conserved protein			
307353017	hypothetical protein Mpet_0859	Yes	1			873789	874187	-		Methanoplanus_petrolearius_DSM_11571_uid526	NC_014507	chromosome									
307353018	hypothetical protein Mpet_0860	No	0			875291	875713	+		Methanoplanus_petrolearius_DSM_11571_uid526	NC_014507	chromosome		arCOG04987	6 (-)	S		Uncharacterized conserved protein			
===									6	Methanoplanus_petrolearius_DSM_11571_uid526	NC_014507	chromosome									
307353020	alpha-galactosidase	Yes	2			876570	878024	+		Methanoplanus_petrolearius_DSM_11571_uid526	NC_014507	chromosome		arCOG02087	2 (-)	S		Predicted membrane protein	2E-51	COG1470	Predicted membrane protein
307353021	hypothetical protein Mpet_0863	Yes	1			878425	879549	-		Methanoplanus_petrolearius_DSM_11571_uid526	NC_014507	chromosome		arCOG06558	10	S		Uncharacterized conserved protein			
307353022	hypothetical protein Mpet_0864	No	0			879633	880010	+		Methanoplanus_petrolearius_DSM_11571_uid526	NC_014507	chromosome									
307353023	hypothetical protein Mpet_0866	Yes	0			881223	882164	+		Methanoplanus_petrolearius_DSM_11571_uid526	NC_014507	chromosome									
307353024	hypothetical protein Mpet_0867	No	4			882179	882520	+		Methanoplanus_petrolearius_DSM_11571_uid526	NC_014507	chromosome									
307353025	hypothetical protein Mpet_0868	No	0			882550	882807	+		Methanoplanus_petrolearius_DSM_11571_uid526	NC_014507	chromosome		arCOG04182	1 (-)	J	RPS24A	Ribosomal protein S24E	1E-10	COG2004	Ribosomal protein S24E
===									9	Methanoplanus_petrolearius_DSM_11571_uid526	NC_014507	chromosome									
307353029	hypothetical protein Mpet_0873	Yes	1			887604	888068	+		Methanoplanus_petrolearius_DSM_11571_uid526	NC_014507	chromosome		arCOG03345	4	S		Uncharacterized conserved protein			
307353030	hypothetical protein Mpet_0874	No	6			888068	888703	+		Methanoplanus_petrolearius_DSM_11571_uid526	NC_014507	chromosome									
307353031	hypothetical protein Mpet_0875	No	0			888619	888849	-		Methanoplanus_petrolearius_DSM_11571_uid526	NC_014507	chromosome									
307353032	hypothetical protein Mpet_0876	No	1			888999	889703	+		Methanoplanus_petrolearius_DSM_11571_uid526	NC_014507	chromosome		arCOG03974	2 (-)	S		Uncharacterized conserved protein			
307353033	hypothetical protein Mpet_0877	No	5			889741	890292	+		Methanoplanus_petrolearius_DSM_11571_uid526	NC_014507	chromosome									
307353034	hypothetical protein Mpet_0878	Yes	1			890881	891990	+		Methanoplanus_petrolearius_DSM_11571_uid526	NC_014507	chromosome		arCOG09593	8	S		Uncharacterized conserved protein			
307353035	hypothetical protein Mpet_0879	Yes	0			892002	892526	+		Methanoplanus_petrolearius_DSM_11571_uid526	NC_014507	chromosome		arCOG06822	4	S		Uncharacterized conserved protein			
307353036	hypothetical protein Mpet_0881	No	0			892800	893621	+		Methanoplanus_petrolearius_DSM_11571_uid526	NC_014507	chromosome									
307353037	hypothetical protein Mpet_0882	No	0			893804	894235	+		Methanoplanus_petrolearius_DSM_11571_uid526	NC_014507	chromosome		arCOG07958	1 (-)	S		Ketosteroid isomerase homolog	0.000000004	pfam13474	SnoaL-like domain. This family contains
===									6	Methanoplanus_petrolearius_DSM_11571_uid526	NC_014507	chromosome									
307353118	prefoldin subunit beta	No	0			972450	972809	+		Methanoplanus_petrolearius_DSM_11571_uid526	NC_014507	chromosome		arCOG01342	1 (-)	O	GimC	Prefoldin, chaperonin cofactor	4E-31	COG1382	Prefoldin, chaperonin cofactor
307353119	hypothetical protein Mpet_0965	Yes	1			973965	975065	+		Methanoplanus_petrolearius_DSM_11571_uid526	NC_014507	chromosome									
307353120	hypothetical protein Mpet_0966	No	0			975101	975724	+		Methanoplanus_petrolearius_DSM_11571_uid526	NC_014507	chromosome									
307353121	hypothetical protein Mpet_0967	No	0			975753	976292	+		Methanoplanus_petrolearius_DSM_11571_uid526	NC_014507	chromosome									
307353122	hypothetical protein Mpet_0968	No	0			976298	976849	+		Methanoplanus_petrolearius_DSM_11571_uid526	NC_014507	chromosome									
307353123	Radical SAM domain-containing protein	No	0			977326	978279	+		Methanoplanus_petrolearius_DSM_11571_uid526	NC_014507	chromosome		arCOG00953	1 (-)	J		Homolog of Wybutosine (yW) biosynthesis enzyr	4E-40	COG0731	Fe-S oxidoreductases
===									7	Methanoplanus_petrolearius_DSM_11571_uid526	NC_014507	chromosome									
307353273	hypothetical protein Mpet_1120	No	0			1143789	1143911	-		Methanoplanus_petrolearius_DSM_11571_uid526	NC_014507	chromosome									
307353274	hypothetical protein Mpet_1121	No	0			1144099	1146855	-		Methanoplanus_petrolearius_DSM_11571_uid526	NC_014507	chromosome		arCOG07809	9 (-)	Y		Viral primase fused to AAA DnaA like ATPAse and Zn finger domain			
307353275	hypothetical protein Mpet_1122	No	0			1146852	1147205	-		Methanoplanus_petrolearius_DSM_11571_uid526	NC_014507	chromosome									
307353276	hypothetical protein Mpet_1123	No	0			1147195	1147470	-		Methanoplanus_petrolearius_DSM_11571_uid526	NC_014507	chromosome		arCOG09583	7	S		Uncharacterized conserved protein			
307353277	hypothetical protein Mpet_1124	No	0			1147449	1147592	-		Methanoplanus_petrolearius_DSM_11571_uid526	NC_014507	chromosome	clust_500								
307353278	hypothetical protein Mpet_1125	No	7			1147749	1148675	-		Methanoplanus_petrolearius_DSM_11571_uid526	NC_014507	chromosome		arCOG05299	1 (-)	S		Uncharacterized conserved protein	2E-35	COG2899	Uncharacterized protein conserved in bac
307353279	hypothetical protein Mpet_1126	No	2			1148745	1148981	-		Methanoplanus_petrolearius_DSM_11571_uid526	NC_014507	chromosome									
===									12	Methanoplanus_petrolearius_DSM_11571_uid526	NC_014507	chromosome									
307353561	hypothetical protein Mpet_1414	No	1			1444545	1445228	+		Methanoplanus_petrolearius_DSM_11571_uid526	NC_014507	chromosome	arCOG05164,a	arCOG05164	1 (-)	S		Uncharacterized conserved protein			
307353562	hypothetical protein Mpet_1415	No	0			1445722	1445841	+		Methanoplanus_petrolearius_DSM_11571_uid526	NC_014507	chromosome									
307353563	hypothetical protein Mpet_1416	No	0			1445984	1446283	-		Methanoplanus_petrolearius_DSM_11571_uid526	NC_014507	chromosome									
307353564	hypothetical protein Mpet_1417	No	0			1446384	1446572	+		Methanoplanus_petrolearius_DSM_11571_uid526	NC_014507	chromosome									
307353565	hypothetical protein Mpet_1418	No	0			1446569	1446694	+		Methanoplanus_petrolearius_DSM_11571_uid526	NC_014507	chromosome									
307353566	hypothetical protein Mpet_1419	No	0			1446694	1446876	+		Methanoplanus_petrolearius_DSM_11571_uid526	NC_014507	chromosome									
307353567	hypothetical protein Mpet_1420	No	0			1446873	1447274	+		Methanoplanus_petrolearius_DSM_11571_uid526	NC_014507	chromosome		arCOG03732	1 (-)	S		Uncharacterized conserved protein			
307353568	hypothetical protein Mpet_1421	No	0			1447271	1447813	+		Methanoplanus_petrolearius_DSM_11571_uid526	NC_014507	chromosome									
307353569	hypothetical protein Mpet_1422	No	0			1447810	1448028	+		Methanoplanus_petrolearius_DSM_11571_uid526	NC_014507	chromosome									
307353570	hypothetical protein Mpet_1423	No	0			1448217	1448987	+		Methanoplanus_petrolearius_DSM_11571_uid526	NC_014507	chromosome		arCOG08578	4 (-)	Y		RepA plasmid replication protein			
307353571	hypothetical protein Mpet_1424	No	0			1449119	1450591	+		Methanoplanus_petrolearius_DSM_11571_uid526	NC_014507	chromosome		arCOG10891	4	S		Uncharacterized conserved protein			
307353572	hypothetical protein Mpet_1425	No	0			1450863	1451252	+		Methanoplanus_petrolearius_DSM_11571_uid526	NC_014507	chromosome									
===									17	Methanoplanus_petrolearius_DSM_11571_uid526	NC_014507	chromosome									
307353582	hypothetical protein Mpet_1436	No	2			1461094	1462083	+		Methanoplanus_petrolearius_DSM_11571_uid526	NC_014507	chromosome		arCOG06559	1 (-)	S		Uncharacterized conserved protein			
307353583	hypothetical protein Mpet_1437	No	5			1462540	1463169	+		Methanoplanus_petrolearius_DSM_11571_uid526	NC_014507	chromosome									
307353584	hypothetical protein Mpet_1438	No	0			1463166	1463657	+		Methanoplanus_petrolearius_DSM_11571_uid526	NC_014507	chromosome									
307353585	hypothetical protein Mpet_1439	No	0			1463659	1464036	+		Methanoplanus_petrolearius_DSM_11571_uid526	NC_014507	chromosome									
307353586	hypothetical protein Mpet_1440	No	1			1464176	1464454	+		Methanoplanus_petrolearius_DSM_11571_uid526	NC_014507	chromosome									
307353587	hypothetical protein Mpet_1441	No	0			1464454	1464966	+		Methanoplanus_petrolearius_DSM_11571_uid526	NC_014507	chromosome									
307353588	hypothetical protein Mpet_1442	No	0			1464991	1466022	+		Methanoplanus_petrolearius_DSM_11571_uid526	NC_014507	chromosome									
307353589	hypothetical protein Mpet_1443	No	0			1466022	1466411	+		Methanoplanus_petrolearius_DSM_11571_uid526	NC_014507	chromosome									
307353590	hypothetical protein Mpet_1444	No	0			1466411	1466710	+		Methanoplanus_petrolearius_DSM_11571_uid526	NC_014507	chromosome									
307353591	hypothetical protein Mpet_1445	No	0			1466779	1466946	+		Methanoplanus_petrolearius_DSM_11571_uid526	NC_014507	chromosome									
307353592	hypothetical protein Mpet_1446	No	0			1468123	1469559	+		Methanoplanus_petrolearius_DSM_11571_uid526	NC_014507	chromosome									
307353593	hypothetical protein Mpet_1447	No	0			1469565	1470080	+		Methanoplanus_petrolearius_DSM_11571_uid526	NC_014507	chromosome									
307353594	hypothetical protein Mpet_1448	No	0			1470077	1470349	+		Methanoplanus_petrolearius_DSM_11571_uid526	NC_014507	chromosome	clust_361								
307353595	hypothetical protein Mpet_1449	No	0			1470346	1471161	+		Methanoplanus_petrolearius_DSM_11571_uid526	NC_014507	chromosome		arCOG10308	5 (-)	S		Uncharacterized conserved protein			
307353596	hypothetical protein Mpet_1450	No	0			1471158	1471661	+		Methanoplanus_petrolearius_DSM_11571_uid526	NC_014507	chromosome		arCOG11245	4	S		Uncharacterized conserved protein			
307353597	hypothetical protein Mpet_1451	No	0			1471658	1472002	+		Methanoplanus_petrolearius_DSM_11571_uid526	NC_014507	chromosome									
307353598	hypothetical protein Mpet_1452	No	0			1472139	1472489	+		Methanoplanus_petrolearius_DSM_11571_uid526	NC_014507	chromosome									
===									9	Methanoplanus_petrolearius_DSM_11571_uid526	NC_014507	chromosome									
307353600	hypothetical protein Mpet_1454	No	0			1473580	1474284	+		Methanoplanus_petrolearius_DSM_11571_uid526	NC_014507	chromosome		arCOG10698	3 (-)	S		Uncharacterized conserved protein			
307353601	hypothetical protein Mpet_1455	No	0			1474299	1475459	+		Methanoplanus_petrolearius_DSM_11571_uid526	NC_014507	chromosome									
307353602	hypothetical protein Mpet_1456	No	0			1475461	1475847	+		Methanoplanus_petrolearius_DSM_11571_uid526	NC_014507	chromosome									
307353603	hypothetical protein Mpet_1457	No	0			1476236	1476607	+		Methanoplanus_petrolearius_DSM_11571_uid526	NC_014507	chromosome									
307353604	hypothetical protein Mpet_1458	No	0			1476609	1477538	+		Methanoplanus_petrolearius_DSM_11571_uid526	NC_014507	chromosome									
307353605	hypothetical protein Mpet_1459	No	1			1477587	1477913	+		Methanoplanus_petrolearius_DSM_11571_uid526	NC_014507	chromosome									
307353606	hypothetical protein Mpet_1460	Yes	1			1478256	1478570	+		Methanoplanus_petrolearius_DSM_11571_uid526	NC_014507	chromosome									
307353607	hypothetical protein Mpet_1461	No	0			1478579	1478779	+		Methanoplanus_petrolearius_DSM_11571_uid526	NC_014507	chromosome									
307353608	hypothetical protein Mpet_1462	No	2			1478996	1480186	-		Methanoplanus_petrolearius_DSM_11571_uid526	NC_014507	chromosome		arCOG03127	3 (-)	P	Kch	Ion channel fused to ion transporting domain			
===									8	Methanoplanus_petrolearius_DSM_11571_uid526	NC_014507	chromosome									
307353685	hypothetical protein Mpet_1541	No	0			1571379	1573235	+		Methanoplanus_petrolearius_DSM_11571_uid526	NC_014507	chromosome									
307353686	hypothetical protein Mpet_1542	No	2			1573363	1573644	+		Methanoplanus_petrolearius_DSM_11571_uid526	NC_014507	chromosome		arCOG04983	2 (-)	S		Predicted membrane protein	4E-19	COG3326	Predicted membrane protein
307353687	hypothetical protein Mpet_1543	No	1			1574477	1574839	-		Methanoplanus_petrolearius_DSM_11571_uid526	NC_014507	chromosome		arCOG07521	6	S		Uncharacterized conserved protein			
307353688	hypothetical protein Mpet_1544	Yes	1			1574876	1575622	-		Methanoplanus_petrolearius_DSM_11571_uid526	NC_014507	chromosome									
307353689	hypothetical protein Mpet_1545	Yes	1			1575653	1576939	-		Methanoplanus_petrolearius_DSM_11571_uid526	NC_014507	chromosome									
307353690	hypothetical protein Mpet_1546	No	0			1577019	1577210	-		Methanoplanus_petrolearius_DSM_11571_uid526	NC_014507	chromosome									
307353691	hypothetical protein Mpet_1547	No	2			1577531	1577812	+		Methanoplanus_petrolearius_DSM_11571_uid526	NC_014507	chromosome		arCOG04983	2 (-)	S		Predicted membrane protein	8E-19	COG3326	Predicted membrane protein
307353692	hypothetical protein Mpet_1548	Yes	1			1578669	1579766	+		Methanoplanus_petrolearius_DSM_11571_uid526	NC_014507	chromosome		arCOG09593	8	S		Uncharacterized conserved protein			
===									11	Methanoplanus_petrolearius_DSM_11571_uid526	NC_014507	chromosome									
307353695	hypothetical protein Mpet_1551	No	1			1581799	1583148	+		Methanoplanus_petrolearius_DSM_11571_uid526	NC_014507	chromosome		arCOG02491	7 (-)	S		WD40 repeats containing protein			
307353696	hypothetical protein Mpet_1552	Yes	1			1583145	1583636	+		Methanoplanus_petrolearius_DSM_11571_uid526	NC_014507	chromosome	clust_219								
307353697	hypothetical protein Mpet_1553	No	0			1584005	1584217	+		Methanoplanus_petrolearius_DSM_11571_uid526	NC_014507	chromosome									
307353698	hypothetical protein Mpet_1554	Yes	1			1584238	1584600	+		Methanoplanus_petrolearius_DSM_11571_uid526	NC_014507	chromosome		arCOG09593	8	S		Uncharacterized conserved protein			
307353699	hypothetical protein Mpet_1555	No	0			1584742	1585323	+		Methanoplanus_petrolearius_DSM_11571_uid526	NC_014507	chromosome									
307353700	hypothetical protein Mpet_1556	Yes	1			1585360	1585851	+		Methanoplanus_petrolearius_DSM_11571_uid526	NC_014507	chromosome	clust_219								
307353701	hypothetical protein Mpet_1557	Yes	1			1586030	1586524	+		Methanoplanus_petrolearius_DSM_11571_uid526	NC_014507	chromosome									
307353702	hypothetical protein Mpet_1558	Yes	1			1586537	1587112	+		Methanoplanus_petrolearius_DSM_11571_uid526	NC_014507	chromosome		arCOG10344	3	S		Uncharacterized conserved protein			
307353703	hypothetical protein Mpet_1559	No	0			1587440	1587664	+		Methanoplanus_petrolearius_DSM_11571_uid526	NC_014507	chromosome		arCOG09519	1 (-)	S		Uncharacterized conserved protein			
307353704	hypothetical protein Mpet_1560	No	2			1588056	1588286	-		Methanoplanus_petrolearius_DSM_11571_uid526	NC_014507	chromosome									
307353705	hypothetical protein Mpet_1561	Yes	1			1588301	1589098	-		Methanoplanus_petrolearius_DSM_11571_uid526	NC_014507	chromosome		arCOG10331	3	S		Uncharacterized conserved protein			
===									5	Methanoplanus_petrolearius_DSM_11571_uid526	NC_014507	chromosome									
307353732	hypothetical protein Mpet_1588	Yes	0			1610107	1610412	+		Methanoplanus_petrolearius_DSM_11571_uid526	NC_014507	chromosome									
307353733	YD repeat-containing protein	Yes	1			1610409	1614452	+		Methanoplanus_petrolearius_DSM_11571_uid526	NC_014507	chromosome							3E-60	COG3209	Rhs family protein
307353734	hypothetical protein Mpet_1590	No	4			1614524	1615228	+		Methanoplanus_petrolearius_DSM_11571_uid526	NC_014507	chromosome	clust_137								
307353735	metal-dependent phosphohydrolase HD sub d	No	0			1615642	1616835	-		Methanoplanus_petrolearius_DSM_11571_uid526	NC_014507	chromosome		arCOG07445	3 (-)	F	Dgt	dGTP triphosphohydrolase	8E-32	COG0232	dGTP triphosphohydrolase
307353736	hypothetical protein Mpet_1592	Yes	1			1616943	1618034	-		Methanoplanus_petrolearius_DSM_11571_uid526	NC_014507	chromosome		arCOG06558	10	S		Uncharacterized conserved protein			
===									7	Methanoplanus_petrolearius_DSM_11571_uid526	NC_014507	chromosome									
307354016	hypothetical protein Mpet_1877	No	0			1894867	1895772	+		Methanoplanus_petrolearius_DSM_11571_uid526	NC_014507	chromosome		arCOG05191	1 (-)	S		Uncharacterized conserved protein	3E-37	COG4866	Uncharacterized conserved protein
307354017	hypothetical protein Mpet_1878	No	1			1896277	1896675	-		Methanoplanus_petrolearius_DSM_11571_uid526	NC_014507	chromosome	clust_268								
307354018	hypothetical protein Mpet_1879	No	1			1896925	1897656	-		Methanoplanus_petrolearius_DSM_11571_uid526	NC_014507	chromosome		arCOG11246	3	S		Uncharacterized conserved protein			
307354019	hypothetical protein Mpet_1880	Yes	0			1897668	1898501	-		Methanoplanus_petrolearius_DSM_11571_uid526	NC_014507	chromosome		arCOG06558	10	S		Uncharacterized conserved protein			
307354020	hypothetical protein Mpet_1881	No	1			1898736	1899134	-		Methanoplanus_petrolearius_DSM_11571_uid526	NC_014507	chromosome	clust_268								
307354021	hypothetical protein Mpet_1882	No	1			1899122	1900201	-		Methanoplanus_petrolearius_DSM_11571_uid526	NC_014507	chromosome		arCOG10238	4	S		Uncharacterized conserved protein			
307354022	hypothetical protein Mpet_1883	No	0			1901057	1902220	-		Methanoplanus_petrolearius_DSM_11571_uid526	NC_014507	chromosome		arCOG04278	1 (-)	R		Predicted aconitase	3E-128	COG1679	Predicted aconitase
===									12	Methanoplanus_petrolearius_DSM_11571_uid526	NC_014507	chromosome									
307354088	hypothetical protein Mpet_1950	Yes	0			1971895	1972431	+		Methanoplanus_petrolearius_DSM_11571_uid526	NC_014507	chromosome									
307354089	hypothetical protein Mpet_1951	Yes	1			1972469	1973008	+		Methanoplanus_petrolearius_DSM_11571_uid526	NC_014507	chromosome		arCOG09277	1	S		Uncharacterized conserved protein			
307354090	FeoA family protein	No	0			1973068	1973298	+		Methanoplanus_petrolearius_DSM_11571_uid526	NC_014507	chromosome									
307354091	aldehyde dehydrogenase	No	0			1973295	1974728	-		Methanoplanus_petrolearius_DSM_11571_uid526	NC_014507	chromosome		arCOG01252	3 (-)	C	PutA	Lactaldehyde dehydrogenase, Succinate semial	4E-101	pfam00171	Aldehyde dehydrogenase family. This fami
307354092	hypothetical protein Mpet_1954	No	2			1974762	1974959	-		Methanoplanus_petrolearius_DSM_11571_uid526	NC_014507	chromosome									
307354093	major facilitator superfamily protein	No	12			1975104	1976423	-		Methanoplanus_petrolearius_DSM_11571_uid526	NC_014507	chromosome		arCOG00130	5 (-)	G	ProP	Permease of the major facilitator superfamily	1E-31	pfam07690	Major Facilitator Superfamily.
307354094	hypothetical protein Mpet_1956	No	0			1976524	1976820	+		Methanoplanus_petrolearius_DSM_11571_uid526	NC_014507	chromosome									
307354095	hypothetical protein Mpet_1957	No	0			1976969	1977163	+		Methanoplanus_petrolearius_DSM_11571_uid526	NC_014507	chromosome	clust_461								
307354096	hypothetical protein Mpet_1958	No	0			1977160	1977447	-		Methanoplanus_petrolearius_DSM_11571_uid526	NC_014507	chromosome	clust_363								
307354097	hypothetical protein Mpet_1959	No	4			1977805	1978299	+		Methanoplanus_petrolearius_DSM_11571_uid526	NC_014507	chromosome									
307354098	hypothetical protein Mpet_1960	No	2			1978366	1979382	-		Methanoplanus_petrolearius_DSM_11571_uid526	NC_014507	chromosome									
307354099	hypothetical protein Mpet_1962	No	0			1979949	1980872	-		Methanoplanus_petrolearius_DSM_11571_uid526	NC_014507	chromosome		arCOG03576	1 (-)	F		Polyphosphate kinase 2	7E-38	COG2326	Uncharacterized conserved protein
===									6	Methanoplanus_petrolearius_DSM_11571_uid526	NC_014507	chromosome									
307354160	transcriptional modulator of MazE/toxin, MazF	No	0			2038836	2039168	+		Methanoplanus_petrolearius_DSM_11571_uid526	NC_014507	chromosome		arCOG03943	2 (-)	V	MazF	Toxin-antitoxin addiction module toxin componen	0.0000007	pfam02452	PemK-like protein. PemK is a growth inhi
307354161	hypothetical protein Mpet_2026	No	0			2039282	2039521	-		Methanoplanus_petrolearius_DSM_11571_uid526	NC_014507	chromosome									
307354162	hypothetical protein Mpet_2027	No	0			2039671	2039844	+		Methanoplanus_petrolearius_DSM_11571_uid526	NC_014507	chromosome	clust_484								
307354163	hypothetical protein Mpet_2028	No	0			2039944	2040147	-		Methanoplanus_petrolearius_DSM_11571_uid526	NC_014507	chromosome									
307354164	hypothetical protein Mpet_2029	No	3			2040449	2041126	-		Methanoplanus_petrolearius_DSM_11571_uid526	NC_014507	chromosome									
307354165	orc1/cdc6 family replication initiation protein	No	0			2041916	2043037	-		Methanoplanus_petrolearius_DSM_11571_uid526	NC_014507	chromosome		arCOG00467	13 (-)	L	CDC6	Cdc6-related protein, AAA superfamily ATPase	1E-40	COG1474	Cdc6-related protein, AAA superfamily AT
===									5	Methanoplanus_petrolearius_DSM_11571_uid526	NC_014507	chromosome									
307354175	hypothetical protein Mpet_2040	No	2			2049567	2049806	+		Methanoplanus_petrolearius_DSM_11571_uid526	NC_014507	chromosome									
307354176	hypothetical protein Mpet_2041	No	0			2049803	2051401	+		Methanoplanus_petrolearius_DSM_11571_uid526	NC_014507	chromosome		arCOG03032	12 (-)	R	NrfG	TPR repeats containing protein			
307354177	hypothetical protein Mpet_2042	No	0			2051693	2052523	-		Methanoplanus_petrolearius_DSM_11571_uid526	NC_014507	chromosome							2E-43	COG4227	Antirestriction protein
307354178	hypothetical protein Mpet_2043	No	0			2052523	2052771	-		Methanoplanus_petrolearius_DSM_11571_uid526	NC_014507	chromosome									
307354179	hypothetical protein Mpet_2044	No	0			2053360	2054715	+		Methanoplanus_petrolearius_DSM_11571_uid526	NC_014507	chromosome		arCOG00367	3	L		NurA 5'-3' nuclease			
===									28	Methanoplanus_petrolearius_DSM_11571_uid526	NC_014507	chromosome									
307354434	MarR family transcriptional regulator	No	0		Virus_Turrivirid	2312955	2313800	-		Methanoplanus_petrolearius_DSM_11571_uid526	NC_014507	chromosome		arCOG02242	2 (-)	K		Transcriptional regulator, contains HTH domain	0.00004	pfam12802	MarR family. The Mar proteins are involv
307354435	hypothetical protein Mpet_2301	No	2		Virus_Turrivirid	2313996	2314238	-		Methanoplanus_petrolearius_DSM_11571_uid526	NC_014507	chromosome									
307354436	hypothetical protein Mpet_2302	No	0		Virus_Turrivirid	2314235	2314534	-		Methanoplanus_petrolearius_DSM_11571_uid526	NC_014507	chromosome									
307354437	hypothetical protein Mpet_2303	No	0		Virus_Turrivirid	2314531	2314962	-		Methanoplanus_petrolearius_DSM_11571_uid526	NC_014507	chromosome									
307354438	hypothetical protein Mpet_2304	No	0		Virus_Turrivirid	2314962	2315291	-		Methanoplanus_petrolearius_DSM_11571_uid526	NC_014507	chromosome									
307354439	transporter cation channel family	No	0		Virus_Turrivirid	2315339	2315572	+		Methanoplanus_petrolearius_DSM_11571_uid526	NC_014507	chromosome									
307354440	hypothetical protein Mpet_2306	No	0		Virus_Turrivirid	2316517	2316669	+		Methanoplanus_petrolearius_DSM_11571_uid526	NC_014507	chromosome									
307354441	hypothetical protein Mpet_2307	No	1		Virus_Turrivirid	2316745	2317050	-		Methanoplanus_petrolearius_DSM_11571_uid526	NC_014507	chromosome									
307354442	hypothetical protein Mpet_2308	No	1		Virus_Turrivirid	2317159	2317692	+		Methanoplanus_petrolearius_DSM_11571_uid526	NC_014507	chromosome									
307354443	hypothetical protein Mpet_2309	No	0		Virus_Turrivirid	2317701	2317898	+		Methanoplanus_petrolearius_DSM_11571_uid526	NC_014507	chromosome									
307354444	hypothetical protein Mpet_2310	No	1		Virus_Turrivirid	2317891	2318445	+		Methanoplanus_petrolearius_DSM_11571_uid526	NC_014507	chromosome									
307354445	hypothetical protein Mpet_2311	No	2		Virus_Turrivirid	2318442	2318693	+		Methanoplanus_petrolearius_DSM_11571_uid526	NC_014507	chromosome									
307354446	hypothetical protein Mpet_2312	No	2		Virus_Turrivirid	2318694	2318930	+		Methanoplanus_petrolearius_DSM_11571_uid526	NC_014507	chromosome									
307354447	ATPase	No	0		Virus_Turrivirid	2318947	2319615	+		Methanoplanus_petrolearius_DSM_11571_uid526	NC_014507	chromosome		arCOG00280	21 (-)	L		HerA helicase	0.00003	COG0433	HerA helicase
307354448	hypothetical protein Mpet_2314	No	1		Virus_Turrivirid	2319692	2319826	+		Methanoplanus_petrolearius_DSM_11571_uid526	NC_014507	chromosome									
307354449	hypothetical protein Mpet_2315	No	0		Virus_Turrivirid	2319842	2320810	+		Methanoplanus_petrolearius_DSM_11571_uid526	NC_014507	chromosome		arCOG10335	5 (-)	Y		Major capsid protein, double jelly roll fold protein			
307354450	hypothetical protein Mpet_2316	Yes	1		Virus_Turrivirid	2320839	2323124	+		Methanoplanus_petrolearius_DSM_11571_uid526	NC_014507	chromosome							0.0001	COG5295	Autotransporter adhesin
307354451	hypothetical protein Mpet_2317	No	1		Virus_Turrivirid	2323141	2323284	+		Methanoplanus_petrolearius_DSM_11571_uid526	NC_014507	chromosome									
307354452	hypothetical protein Mpet_2318	No	2		Virus_Turrivirid	2323291	2323641	+		Methanoplanus_petrolearius_DSM_11571_uid526	NC_014507	chromosome									
307354453	hypothetical protein Mpet_2319	No	0		Virus_Turrivirid	2323642	2323818	+		Methanoplanus_petrolearius_DSM_11571_uid526	NC_014507	chromosome									
307354454	hypothetical protein Mpet_2320	No	1		Virus_Turrivirid	2323823	2323984	+		Methanoplanus_petrolearius_DSM_11571_uid526	NC_014507	chromosome									
307354455	hypothetical protein Mpet_2321	No	0		Virus_Turrivirid	2324027	2324413	+		Methanoplanus_petrolearius_DSM_11571_uid526	NC_014507	chromosome									
307354456	hypothetical protein Mpet_2322	No	0		Virus_Turrivirid	2324459	2325040	+		Methanoplanus_petrolearius_DSM_11571_uid526	NC_014507	chromosome									
307354457	hypothetical protein Mpet_2323	No	0		Virus_Turrivirid	2325040	2325852	+		Methanoplanus_petrolearius_DSM_11571_uid526	NC_014507	chromosome									
307354458	hypothetical protein Mpet_2324	No	0		Virus_Turrivirid	2325864	2326469	+		Methanoplanus_petrolearius_DSM_11571_uid526	NC_014507	chromosome									
307354459	RelE/StbE family addiction module toxin	No	0		Virus_Turrivirid	2326528	2326815	+		Methanoplanus_petrolearius_DSM_11571_uid526	NC_014507	chromosome	clust_363								
307354460	hypothetical protein Mpet_2326	No	0		Virus_Turrivirid	2326812	2327000	-		Methanoplanus_petrolearius_DSM_11571_uid526	NC_014507	chromosome	clust_461								
307354461	integrase family protein	No	0		Virus_Turrivirid	2327058	2328371	-		Methanoplanus_petrolearius_DSM_11571_uid526	NC_014507	chromosome		arCOG01242	14 (-)	L	XerC	Integrase	1E-11	pfam00589	Phage integrase family. Members of this
===									10	Methanoplanus_petrolearius_DSM_11571_uid526	NC_014507	chromosome									
307354627	hypothetical protein Mpet_2496	No	0			2497146	2497310	-		Methanoplanus_petrolearius_DSM_11571_uid526	NC_014507	chromosome									
307354628	GCN5-like N-acetyltransferase	No	0			2497616	2498146	+		Methanoplanus_petrolearius_DSM_11571_uid526	NC_014507	chromosome		arCOG00842	2 (-)	J	RimL	Acetyltransferase, RimL family	1E-17	COG1670	Acetyltransferases, including N-acetylas
307354629	hypothetical protein Mpet_2498	No	0			2498278	2499687	+		Methanoplanus_petrolearius_DSM_11571_uid526	NC_014507	chromosome									
307354630	hypothetical protein Mpet_2499	No	2			2500002	2501411	-		Methanoplanus_petrolearius_DSM_11571_uid526	NC_014507	chromosome	clust_12								
307354631	hypothetical protein Mpet_2500	Yes	0			2501497	2502861	-		Methanoplanus_petrolearius_DSM_11571_uid526	NC_014507	chromosome	clust_12								
307354632	hypothetical protein Mpet_2501	Yes	1			2502890	2504218	-		Methanoplanus_petrolearius_DSM_11571_uid526	NC_014507	chromosome	clust_12								
307354633	hypothetical protein Mpet_2502	Yes	1			2504247	2505680	-		Methanoplanus_petrolearius_DSM_11571_uid526	NC_014507	chromosome	clust_12								
307354634	hypothetical protein Mpet_2503	Yes	1			2506038	2506808	+		Methanoplanus_petrolearius_DSM_11571_uid526	NC_014507	chromosome									
307354635	hypothetical protein Mpet_2504	Yes	1			2507073	2507645	+		Methanoplanus_petrolearius_DSM_11571_uid526	NC_014507	chromosome									
307354636	hypothetical protein Mpet_2505	Yes	0			2507903	2508607	+		Methanoplanus_petrolearius_DSM_11571_uid526	NC_014507	chromosome		arCOG04987	6 (-)	S		Uncharacterized conserved protein			
===									5	Methanoplanus_petrolearius_DSM_11571_uid526	NC_014507	chromosome									
307354866	hypothetical protein Mpet_2736	Yes	2			2747303	2748232	-		Methanoplanus_petrolearius_DSM_11571_uid526	NC_014507	chromosome									
307354867	hypothetical protein Mpet_2737	No	3			2748350	2748769	-		Methanoplanus_petrolearius_DSM_11571_uid526	NC_014507	chromosome		arCOG03630	1 (-)	S		Uncharacterized conserved protein	3E-11	COG2881	Uncharacterized protein conserved in arc
307354868	hypothetical protein Mpet_2738	No	2			2748779	2749165	-		Methanoplanus_petrolearius_DSM_11571_uid526	NC_014507	chromosome									
307354869	hypothetical protein Mpet_2739	No	2			2749216	2749509	+		Methanoplanus_petrolearius_DSM_11571_uid526	NC_014507	chromosome									
307354870	hypothetical protein Mpet_2740	No	5			2749627	2750217	-		Methanoplanus_petrolearius_DSM_11571_uid526	NC_014507	chromosome							2E-15	COG2881	Uncharacterized protein conserved in arc
===									11	Methanopyrus_kandleri_AV19_uid57883	NC_003551	chromosome									
20093738	hypothetical protein MK0298	No	0			287326	287943	-		Methanopyrus_kandleri_AV19_uid57883	NC_003551	chromosome	clust_1						2E-93	pfam07913	Protein of unknown function (DUF1678). T
20093739	hypothetical protein MK0299	Yes	0			288089	289126	-		Methanopyrus_kandleri_AV19_uid57883	NC_003551	chromosome	clust_30								
20093740	hypothetical protein MK0300	Yes	0			289372	290193	-		Methanopyrus_kandleri_AV19_uid57883	NC_003551	chromosome									
20093741	RNA-binding protein	No	0			290810	291202	+		Methanopyrus_kandleri_AV19_uid57883	NC_003551	chromosome		arCOG07759	2 (-)	V	VapC	PIN domain containing protein			
20093742	RNA-binding protein	Yes	0			291417	292477	+		Methanopyrus_kandleri_AV19_uid57883	NC_003551	chromosome									
20093743	transglutaminase-like supefamily cysteine prote	No	0			292704	293645	+		Methanopyrus_kandleri_AV19_uid57883	NC_003551	chromosome		arCOG02165	4 (-)	E		Transglutaminase-like enzyme, putative cysteine protease			
20093744	hypothetical protein MK0304	No	0			293608	294210	+		Methanopyrus_kandleri_AV19_uid57883	NC_003551	chromosome									
20093745	hypothetical protein MK0305	No	1			294271	295311	+		Methanopyrus_kandleri_AV19_uid57883	NC_003551	chromosome									
20093746	hypothetical protein MK0306	No	2			295669	296193	+		Methanopyrus_kandleri_AV19_uid57883	NC_003551	chromosome									
20093747	formylmethanofuran dehydrogenase subunit F,	No	0			296467	297540	+		Methanopyrus_kandleri_AV19_uid57883	NC_003551	chromosome		arCOG02180	2 (-)	C	NapF	Polyferredoxin	2E-15	COG1145	Ferredoxin
20093748	hypothetical protein MK0308	No	0	YES		297654	298370	-		Methanopyrus_kandleri_AV19_uid57883	NC_003551	chromosome									
===									6	Methanopyrus_kandleri_AV19_uid57883	NC_003551	chromosome									
20093750	hypothetical protein MK0310	No	0	YES		299623	300867	-		Methanopyrus_kandleri_AV19_uid57883	NC_003551	chromosome		arCOG03158	1 (-)	L		Intein/homing endonuclease	2E-12	COG1372	Intein/homing endonuclease
20093751	hypothetical protein MK0311	No	0	YES		302118	302261	-		Methanopyrus_kandleri_AV19_uid57883	NC_003551	chromosome									
20093752	hypothetical protein MK0312	No	0	YES		302397	303113	+		Methanopyrus_kandleri_AV19_uid57883	NC_003551	chromosome									
20093753	hypothetical protein MK0313	Yes	0	YES		303210	303731	+		Methanopyrus_kandleri_AV19_uid57883	NC_003551	chromosome									
20093754	hypothetical protein MK0316	No	0	YES		307991	308224	+		Methanopyrus_kandleri_AV19_uid57883	NC_003551	chromosome									
20093755	DNA methylase containing a Zn-ribbon	No	0	YES		309026	309403	-		Methanopyrus_kandleri_AV19_uid57883	NC_003551	chromosome		arCOG00889	6 (-)	L		Adenine-specific DNA methylase containing a Zn	5E-17	COG1743	Adenine-specific DNA methylase containin
===									10	Methanopyrus_kandleri_AV19_uid57883	NC_003551	chromosome									
20093761	formylmethanofuran dehydrogenase subunit F,	No	0			315226	315678	+		Methanopyrus_kandleri_AV19_uid57883	NC_003551	chromosome		arCOG02180	2 (-)	C	NapF	Polyferredoxin	4E-11	COG2768	Uncharacterized Fe-S center protein
20093762	fragment of dehydrogenase related to phosph	No	0			315855	316253	-		Methanopyrus_kandleri_AV19_uid57883	NC_003551	chromosome									
20093763	hypothetical protein MK0325	No	0			316385	316765	-		Methanopyrus_kandleri_AV19_uid57883	NC_003551	chromosome							1E-48	pfam07913	Protein of unknown function (DUF1678). T
20093764	hypothetical protein MK0326	No	0			316791	318491	+		Methanopyrus_kandleri_AV19_uid57883	NC_003551	chromosome									
20093765	hypothetical protein MK0327	No	6			318525	319349	+		Methanopyrus_kandleri_AV19_uid57883	NC_003551	chromosome									
20093766	hypothetical protein MK0328	No	3			319527	320099	+		Methanopyrus_kandleri_AV19_uid57883	NC_003551	chromosome									
20093767	hypothetical protein MK0329	No	2			320696	321142	+		Methanopyrus_kandleri_AV19_uid57883	NC_003551	chromosome									
20093768	hypothetical protein MK0330	Yes	0			321611	322570	-		Methanopyrus_kandleri_AV19_uid57883	NC_003551	chromosome	clust_97								
20093769	hypothetical protein MK0331	No	0			323201	323818	+		Methanopyrus_kandleri_AV19_uid57883	NC_003551	chromosome	clust_1						3E-97	pfam07913	Protein of unknown function (DUF1678). T
20093770	hypothetical protein MK0332	No	0			324061	324486	-		Methanopyrus_kandleri_AV19_uid57883	NC_003551	chromosome		arCOG04901	1 (-)	S		Uncharacterized conserved protein	1E-65	COG4029	Uncharacterized protein conserved in arc
===									7	Methanopyrus_kandleri_AV19_uid57883	NC_003551	chromosome									
20094027	fused hydroxymethylpyrimidine/phosphomethy	No	0			547818	549116	+		Methanopyrus_kandleri_AV19_uid57883	NC_003551	chromosome		arCOG00020	2 (-)	H	ThiD	Hydroxymethylpyrimidine/phosphomethylpyrimid	1E-49	COG1992	Uncharacterized conserved protein
20094028	hypothetical protein MK0590	No	0			549121	549732	+		Methanopyrus_kandleri_AV19_uid57883	NC_003551	chromosome									
20094029	hypothetical protein MK0591	Yes	0			549969	550763	+		Methanopyrus_kandleri_AV19_uid57883	NC_003551	chromosome									
20094030	hypothetical protein MK0592	No	0			550754	551515	+		Methanopyrus_kandleri_AV19_uid57883	NC_003551	chromosome									
20094031	hypothetical protein MK0593	No	0			551518	551976	+		Methanopyrus_kandleri_AV19_uid57883	NC_003551	chromosome									
20094032	hypothetical protein MK0594	No	0			552664	552933	+		Methanopyrus_kandleri_AV19_uid57883	NC_003551	chromosome									
20094033	methyltransferase	No	0			553054	553923	+		Methanopyrus_kandleri_AV19_uid57883	NC_003551	chromosome		arCOG00054	1 (-)	R		Predicted archaeal methyltransferase	2E-88	COG2521	Predicted archaeal methyltransferase
===									7	Methanopyrus_kandleri_AV19_uid57883	NC_003551	chromosome									
20094081	hypothetical protein MK0643	No	2			602066	602473	-		Methanopyrus_kandleri_AV19_uid57883	NC_003551	chromosome									
20094082	ATPase	No	0			602534	603211	+		Methanopyrus_kandleri_AV19_uid57883	NC_003551	chromosome		arCOG00035	3 (-)	R		Predicted ATPase of PP-loop superfamily	4E-83	COG2102	Predicted ATPases of PP-loop superfamily
20094083	hypothetical protein MK0645	No	0			603358	604410	+		Methanopyrus_kandleri_AV19_uid57883	NC_003551	chromosome									
20094084	hypothetical protein MK0646	No	1			604733	604954	-		Methanopyrus_kandleri_AV19_uid57883	NC_003551	chromosome									
20094085	hypothetical protein MK0647	No	0			605491	606189	+		Methanopyrus_kandleri_AV19_uid57883	NC_003551	chromosome							8E-51	pfam07913	Protein of unknown function (DUF1678). T
20094086	hydrogenase maturation factor	No	0			606223	608511	-		Methanopyrus_kandleri_AV19_uid57883	NC_003551	chromosome		arCOG01187	1 (-)	O	HypF	Hydrogenase maturation factor		COG0068	Hydrogenase maturation factor
20094087	hypothetical protein MK0649	No	0			608508	609632	-		Methanopyrus_kandleri_AV19_uid57883	NC_003551	chromosome									
===									11	Methanopyrus_kandleri_AV19_uid57883	NC_003551	chromosome									
20094131	hypothetical protein MK0694	No	1			657749	658219	-		Methanopyrus_kandleri_AV19_uid57883	NC_003551	chromosome									
20094132	hypothetical protein MK0695	No	1			658227	658802	-		Methanopyrus_kandleri_AV19_uid57883	NC_003551	chromosome		arCOG03926	1 (-)	S		Uncharacterized conserved protein			
20094133	hypothetical protein MK0696	No	1			658768	659217	-		Methanopyrus_kandleri_AV19_uid57883	NC_003551	chromosome							0.000001	COG1991	Uncharacterized conserved protein
20094134	hypothetical protein MK0697	No	0			659236	661821	+		Methanopyrus_kandleri_AV19_uid57883	NC_003551	chromosome									
20094135	hypothetical protein MK0698	No	1			661961	663658	-		Methanopyrus_kandleri_AV19_uid57883	NC_003551	chromosome									
20094136	hypothetical protein MK0699	No	1			663655	664569	-		Methanopyrus_kandleri_AV19_uid57883	NC_003551	chromosome									
20094137	hypothetical protein MK0700	No	1			664566	664736	-		Methanopyrus_kandleri_AV19_uid57883	NC_003551	chromosome									
20094138	hypothetical protein MK0701	No	1			664747	664935	-		Methanopyrus_kandleri_AV19_uid57883	NC_003551	chromosome									
20094139	hypothetical protein MK0702	No	1			664932	665126	-		Methanopyrus_kandleri_AV19_uid57883	NC_003551	chromosome									
20094140	prepilin-cleaving (type IV) signal peptidase	No	6			665111	666085	-		Methanopyrus_kandleri_AV19_uid57883	NC_003551	chromosome		arCOG02300	1 (-)	N	EppA/Pul	Type II secretory pathway, prepilin signal peptida	0.00000002	pfam06819	Archaeal Peptidase A24 C-terminal Domain ; Flp pilus assembly protein, protease Cpa
20094141	hypothetical protein MK0704	No	0			666091	667089	-		Methanopyrus_kandleri_AV19_uid57883	NC_003551	chromosome									
===									8	Methanopyrus_kandleri_AV19_uid57883	NC_003551	chromosome									
20094346	hypothetical protein MK0910	No	3			864567	866477	-		Methanopyrus_kandleri_AV19_uid57883	NC_003551	chromosome									
20094347	carbamoylphosphate synthase large subunit	No	0			866594	868288	-		Methanopyrus_kandleri_AV19_uid57883	NC_003551	chromosome		arCOG01594	1 (-)	E	CarB	Carbamoylphosphate synthase large subunit		COG0458	Carbamoylphosphate synthase large subuni ; Carbamoyl-phosphate synthetase large cha
20094348	hypothetical protein MK0912	No	0			868674	869447	+		Methanopyrus_kandleri_AV19_uid57883	NC_003551	chromosome									
20094349	hypothetical protein MK0913	No	7			869366	870883	+		Methanopyrus_kandleri_AV19_uid57883	NC_003551	chromosome									
20094350	hypothetical protein MK0914	No	12			870784	873003	-		Methanopyrus_kandleri_AV19_uid57883	NC_003551	chromosome									
20094351	hypothetical protein MK0915	No	0			872967	873524	-		Methanopyrus_kandleri_AV19_uid57883	NC_003551	chromosome									
20094352	hypothetical protein MK0916	No	5			873521	874090	-		Methanopyrus_kandleri_AV19_uid57883	NC_003551	chromosome									
20094353	translation initiation factor eIF2B subunit	No	0			874490	875560	-		Methanopyrus_kandleri_AV19_uid57883	NC_003551	chromosome		arCOG00666	1 (-)	M	GCD1	N-acetylglucosamine-1-phosphate uridyltransfera	2E-79	COG1208	Nucleoside-diphosphate-sugar pyrophospho
===									5	Methanopyrus_kandleri_AV19_uid57883	NC_003551	chromosome									
20094381	hypothetical protein MK0945	No	0			904797	905420	-		Methanopyrus_kandleri_AV19_uid57883	NC_003551	chromosome	clust_1						2E-91	pfam07913	Protein of unknown function (DUF1678). T
20094382	hypothetical protein MK0946	No	2			905879	906190	+		Methanopyrus_kandleri_AV19_uid57883	NC_003551	chromosome		arCOG06689	1 (-)	S		Uncharacterized conserved protein			
20094383	hypothetical protein MK0947	Yes	0			906696	908201	+		Methanopyrus_kandleri_AV19_uid57883	NC_003551	chromosome									
20094384	hypothetical protein MK0948	No	0			908194	910293	+		Methanopyrus_kandleri_AV19_uid57883	NC_003551	chromosome									
20094385	hypothetical protein MK0949	No	8			910269	911270	+		Methanopyrus_kandleri_AV19_uid57883	NC_003551	chromosome									
===									11	Methanopyrus_kandleri_AV19_uid57883	NC_003551	chromosome									
20094528	hypothetical protein MK1092	No	0			1061983	1062237	+		Methanopyrus_kandleri_AV19_uid57883	NC_003551	chromosome									
20094529	N-methylhydantoinase A	No	0			1062427	1063875	-		Methanopyrus_kandleri_AV19_uid57883	NC_003551	chromosome		arCOG01511	3 (-)	E	HyuA	N-methylhydantoinase A/acetone carboxylase, b	5E-28	COG0145	N-methylhydantoinase A/acetone carboxyla
20094530	hypothetical protein MK1094	No	0			1063943	1064371	-		Methanopyrus_kandleri_AV19_uid57883	NC_003551	chromosome									
20094531	hypothetical protein MK1095	No	0			1064771	1065691	-		Methanopyrus_kandleri_AV19_uid57883	NC_003551	chromosome									
20094532	hypothetical protein MK1096	No	0			1066239	1067360	-		Methanopyrus_kandleri_AV19_uid57883	NC_003551	chromosome	clust_85								
20094533	hypothetical protein MK1097	No	0			1067565	1067867	-		Methanopyrus_kandleri_AV19_uid57883	NC_003551	chromosome									
20094534	hypothetical protein MK1098	Yes	2			1067881	1068231	-		Methanopyrus_kandleri_AV19_uid57883	NC_003551	chromosome									
20094535	hypothetical protein MK1099	No	0			1068430	1069563	-		Methanopyrus_kandleri_AV19_uid57883	NC_003551	chromosome	clust_85								
20094536	extracellular polysaccharide hydrolase	No	0			1070068	1071114	+		Methanopyrus_kandleri_AV19_uid57883	NC_003551	chromosome		arCOG07957	3 (-)	R		Uncharacterized TIM barrel protein	6E-59	COG2342	Predicted extracellular endo alpha-1,4 p
20094537	hypothetical protein MK1101	No	0			1071283	1072530	+		Methanopyrus_kandleri_AV19_uid57883	NC_003551	chromosome									
20094538	transcription regulator	No	0			1072764	1073159	-		Methanopyrus_kandleri_AV19_uid57883	NC_003551	chromosome									
===									13	Methanopyrus_kandleri_AV19_uid57883	NC_003551	chromosome									
20094545	hypothetical protein MK1109	No	0			1079336	1080184	-		Methanopyrus_kandleri_AV19_uid57883	NC_003551	chromosome									

20094546	hypothetical protein MK1110	No	1		1080370	1081089	-		Methanopyrus_kandleri_AV19_uid57883	NC_003551	chromosome									
20094547	hypothetical protein MK1111	No	0		1081197	1082513	+		Methanopyrus_kandleri_AV19_uid57883	NC_003551	chromosome									
20094548	hypothetical protein MK1112	Yes	0		1082635	1084164	-		Methanopyrus_kandleri_AV19_uid57883	NC_003551	chromosome		arCOG03832	1 (-)	S		Uncharacterized conserved protein	0.000003	COG1196	Chromosome segregation ATPases
20094549	hypothetical protein MK1113	Yes	0		1084374	1084985	-		Methanopyrus_kandleri_AV19_uid57883	NC_003551	chromosome									
20094550	hypothetical protein MK1114	Yes	0		1085323	1086447	-		Methanopyrus_kandleri_AV19_uid57883	NC_003551	chromosome									
20094551	hypothetical protein MK1115	No	0		1086530	1088314	-		Methanopyrus_kandleri_AV19_uid57883	NC_003551	chromosome	arCOG02827,a	arCOG02827	1 (-)	S		Uncharacterized conserved protein	0.00002	COG4932	Predicted outer membrane protein
20094552	hypothetical protein MK1116	No	0		1088392	1090035	-		Methanopyrus_kandleri_AV19_uid57883	NC_003551	chromosome									
20094553	hypothetical protein MK1117	No	0		1090497	1090760	-		Methanopyrus_kandleri_AV19_uid57883	NC_003551	chromosome									
20094554	hypothetical protein MK1118	No	1		1090917	1091960	-		Methanopyrus_kandleri_AV19_uid57883	NC_003551	chromosome									
20094555	hypothetical protein MK1119	Yes	0		1091917	1092153	-		Methanopyrus_kandleri_AV19_uid57883	NC_003551	chromosome									
20094556	ATPase	No	0		1092364	1093884	-		Methanopyrus_kandleri_AV19_uid57883	NC_003551	chromosome		arCOG00439	14 (-)	L	MCM2	Predicted ATPase involved in replication control,	2E-55	COG1241	Predicted ATPase involved in replication
20094557	hypothetical protein MK1121	No	0		1095025	1095999	+		Methanopyrus_kandleri_AV19_uid57883	NC_003551	chromosome									
===								5	Methanopyrus_kandleri_AV19_uid57883	NC_003551	chromosome									
20094566	hypothetical protein MK1130	No	0	YES	1105400	1105687	+		Methanopyrus_kandleri_AV19_uid57883	NC_003551	chromosome									
20094567	hypothetical protein MK1131	No	0	YES	1107532	1108419	-		Methanopyrus_kandleri_AV19_uid57883	NC_003551	chromosome									
20094568	hypothetical protein MK1132	No	0	YES	1109620	1110027	+		Methanopyrus_kandleri_AV19_uid57883	NC_003551	chromosome		arCOG01191	3 (-)	V		HEPN domain containing protein	2E-13	pfam05168	HEPN domain.
20094569	hypothetical protein MK1133	No	1	YES	1110240	1110470	-		Methanopyrus_kandleri_AV19_uid57883	NC_003551	chromosome									
20094570	hypothetical protein MK1134	No	0	YES	1113424	1114281	+		Methanopyrus_kandleri_AV19_uid57883	NC_003551	chromosome									
===								51	Methanopyrus_kandleri_AV19_uid57883	NC_003551	chromosome									
20094574	hypothetical protein MK1138	No	0	YES	1116684	1116905	+		Methanopyrus_kandleri_AV19_uid57883	NC_003551	chromosome		arCOG01191	3 (-)	V		HEPN domain containing protein	0.0000007	COG2250	Uncharacterized conserved protein relate
20094575	hypothetical protein MK1139	No	0	YES	1116898	1117071	+		Methanopyrus_kandleri_AV19_uid57883	NC_003551	chromosome									
20094576	hypothetical protein MK1140	No	1	YES	1117134	1117373	-		Methanopyrus_kandleri_AV19_uid57883	NC_003551	chromosome									
20094577	hypothetical protein MK1141	No	3	YES	1117370	1117810	-		Methanopyrus_kandleri_AV19_uid57883	NC_003551	chromosome									
20094578	hypothetical protein MK1142	No	0	YES	1117919	1118431	-		Methanopyrus_kandleri_AV19_uid57883	NC_003551	chromosome									
20094579	hypothetical protein MK1143	No	0	YES	1119001	1119915	-		Methanopyrus_kandleri_AV19_uid57883	NC_003551	chromosome									
20094580	hypothetical protein MK1144	No	5	YES	1120281	1121489	-		Methanopyrus_kandleri_AV19_uid57883	NC_003551	chromosome									
20094581	hypothetical protein MK1145	No	4	YES	1122067	1122807	+		Methanopyrus_kandleri_AV19_uid57883	NC_003551	chromosome									
20094582	hypothetical protein MK1146	Yes	3	YES	1122763	1123665	-		Methanopyrus_kandleri_AV19_uid57883	NC_003551	chromosome									
20094583	hypothetical protein MK1147	No	0	YES	1125171	1125659	-		Methanopyrus_kandleri_AV19_uid57883	NC_003551	chromosome									
20094584	hypothetical protein MK1148	Yes	0	YES	1125923	1130821	+		Methanopyrus_kandleri_AV19_uid57883	NC_003551	chromosome									
20094585	hypothetical protein MK1149	Yes	1	YES	1130814	1136363	+		Methanopyrus_kandleri_AV19_uid57883	NC_003551	chromosome									
20094586	hypothetical protein MK1150	Yes	5	YES	1136364	1137101	+		Methanopyrus_kandleri_AV19_uid57883	NC_003551	chromosome									
20094587	hypothetical protein MK1151	Yes	5	YES	1137105	1137752	+		Methanopyrus_kandleri_AV19_uid57883	NC_003551	chromosome									
20094588	hypothetical protein MK1152	No	3	YES	1138095	1138991	+		Methanopyrus_kandleri_AV19_uid57883	NC_003551	chromosome									
20094589	hypothetical protein MK1153	No	4	YES	1139217	1139651	+		Methanopyrus_kandleri_AV19_uid57883	NC_003551	chromosome									
20094590	hypothetical protein MK1154	No	6	YES	1139945	1141204	+		Methanopyrus_kandleri_AV19_uid57883	NC_003551	chromosome	clust_81								
20094591	hypothetical protein MK1155	No	3	YES	1141640	1142470	+		Methanopyrus_kandleri_AV19_uid57883	NC_003551	chromosome									
20094592	hypothetical protein MK1156	Yes	1	YES	1142499	1142942	+		Methanopyrus_kandleri_AV19_uid57883	NC_003551	chromosome									
20094593	hypothetical protein MK1157	No	0	YES	1143512	1144135	-		Methanopyrus_kandleri_AV19_uid57883	NC_003551	chromosome	clust_1						7E-95	pfam07913	Protein of unknown function (DUF1678). T
20094594	hypothetical protein MK1158	No	5	YES	1144383	1145600	-		Methanopyrus_kandleri_AV19_uid57883	NC_003551	chromosome	clust_81								
20094595	hypothetical protein MK1159	No	4	YES	1145844	1146677	+		Methanopyrus_kandleri_AV19_uid57883	NC_003551	chromosome									
20094596	hypothetical protein MK1160	No	6	YES	1146822	1147688	+		Methanopyrus_kandleri_AV19_uid57883	NC_003551	chromosome									
20094597	hypothetical protein MK1161	No	2	YES	1148015	1148680	+		Methanopyrus_kandleri_AV19_uid57883	NC_003551	chromosome									
20094598	hypothetical protein MK1162	No	5	YES	1148705	1149403	+		Methanopyrus_kandleri_AV19_uid57883	NC_003551	chromosome									
20094599	hypothetical protein MK1163	No	0	YES	1149695	1150318	-		Methanopyrus_kandleri_AV19_uid57883	NC_003551	chromosome	clust_1						8E-94	pfam07913	Protein of unknown function (DUF1678). T
20094600	nuclease	No	1	YES	1151111	1151647	-		Methanopyrus_kandleri_AV19_uid57883	NC_003551	chromosome		arCOG03192	6 (-)	L		Micrococcal nuclease (thermonuclease)			
20094601	hypothetical protein MK1165	No	0	YES	1151966	1152913	-		Methanopyrus_kandleri_AV19_uid57883	NC_003551	chromosome									
20094602	hypothetical protein MK1166	No	0	YES	1152967	1154208	-		Methanopyrus_kandleri_AV19_uid57883	NC_003551	chromosome		arCOG02838	1 (-)	T		FIST_N domain containing protein	2E-67	COG3287	Uncharacterized conserved protein
20094603	hypothetical protein MK1167	No	0	YES	1155432	1156157	+		Methanopyrus_kandleri_AV19_uid57883	NC_003551	chromosome									
20094604	hypothetical protein MK1168	Yes	1	YES	1156220	1157155	+		Methanopyrus_kandleri_AV19_uid57883	NC_003551	chromosome									
20094605	hypothetical protein MK1169	No	0	YES	1158073	1158933	-		Methanopyrus_kandleri_AV19_uid57883	NC_003551	chromosome									
20094606	hypothetical protein MK1170	No	7	YES	1160085	1161410	-		Methanopyrus_kandleri_AV19_uid57883	NC_003551	chromosome									
20094607	membrane-bound metal-dependent hydrolase	No	0		1161703	1162374	-		Methanopyrus_kandleri_AV19_uid57883	NC_003551	chromosome							0.000001	pfam04307	Predicted membrane-bound metal-dependent
20094608	hypothetical protein MK1172	No	0		1162560	1163432	+		Methanopyrus_kandleri_AV19_uid57883	NC_003551	chromosome									
20094609	hypothetical protein MK1173	Yes	1		1163540	1164262	+		Methanopyrus_kandleri_AV19_uid57883	NC_003551	chromosome									
20094610	hypothetical protein MK1174	No	3		1165552	1166187	+		Methanopyrus_kandleri_AV19_uid57883	NC_003551	chromosome									
20094611	hypothetical protein MK1175	No	0		1167028	1167396	-		Methanopyrus_kandleri_AV19_uid57883	NC_003551	chromosome									
20094612	hypothetical protein MK1176	Yes	0		1167393	1167758	-		Methanopyrus_kandleri_AV19_uid57883	NC_003551	chromosome									
20094613	metal-binding domain-containing protein	Yes	0		1168689	1171121	+		Methanopyrus_kandleri_AV19_uid57883	NC_003551	chromosome	arCOG00765,a	arCOG00765	1 (-)	R		Protein containing a metal-binding domain sharec	2E-105	COG5643	Protein containing a metal-binding domai
20094614	hypothetical protein MK1178	Yes	0		1171194	1174100	+		Methanopyrus_kandleri_AV19_uid57883	NC_003551	chromosome		arCOG02556	2 (-)	S		WD40-like repeat			
20094615	hypothetical protein MK1179	No	0		1174103	1174543	-		Methanopyrus_kandleri_AV19_uid57883	NC_003551	chromosome									
20094616	hypothetical protein MK1180	No	0		1174740	1175693	-		Methanopyrus_kandleri_AV19_uid57883	NC_003551	chromosome									
20094617	hypothetical protein MK1181	No	0		1176046	1176945	+		Methanopyrus_kandleri_AV19_uid57883	NC_003551	chromosome									
20094618	hypothetical protein MK1182	Yes	0		1177071	1177787	-		Methanopyrus_kandleri_AV19_uid57883	NC_003551	chromosome									
20094619	polyferredoxin	No	5		1178571	1179359	-		Methanopyrus_kandleri_AV19_uid57883	NC_003551	chromosome		arCOG02772	2 (-)	C	NapH	Polyferredoxin	2E-17	COG0348	Polyferredoxin
20094620	hypothetical protein MK1184	Yes	0		1179463	1179858	-		Methanopyrus_kandleri_AV19_uid57883	NC_003551	chromosome							0.000001	pfam12810	Glycine rich protein. This family of pro
20094621	hypothetical protein MK1185	Yes	2		1179906	1180262	-		Methanopyrus_kandleri_AV19_uid57883	NC_003551	chromosome									
20094622	hypothetical protein MK1186	No	0		1181791	1182024	+		Methanopyrus_kandleri_AV19_uid57883	NC_003551	chromosome									
20094623	extracellular polysaccharide hydrolase	Yes	1		1182514	1183490	+		Methanopyrus_kandleri_AV19_uid57883	NC_003551	chromosome		arCOG07957	3 (-)	R		Uncharacterized TIM barrel protein	2E-65	COG2342	Predicted extracellular endo alpha-1,4 p
20094624	hypothetical protein MK1188	No	0		1183487	1183930	+		Methanopyrus_kandleri_AV19_uid57883	NC_003551	chromosome									
===								9	Methanopyrus_kandleri_AV19_uid57883	NC_003551	chromosome									
20094685	rubrerythrin	No	0		1231755	1232132	-		Methanopyrus_kandleri_AV19_uid57883	NC_003551	chromosome		arCOG01097	1 (-)	C		Rubrerythrin	6E-13	COG1592	Rubrerythrin
20094686	hypothetical protein MK1250	Yes	2		1232451	1232984	-		Methanopyrus_kandleri_AV19_uid57883	NC_003551	chromosome									
20094687	hypothetical protein MK1251	No	0		1234371	1235411	-		Methanopyrus_kandleri_AV19_uid57883	NC_003551	chromosome									
20094688	hypothetical protein MK1252	No	0		1236233	1236910	-		Methanopyrus_kandleri_AV19_uid57883	NC_003551	chromosome	clust_1						1E-89	pfam07913	Protein of unknown function (DUF1678). T
20094689	hypothetical protein MK1253	Yes	0		1237175	1240579	+		Methanopyrus_kandleri_AV19_uid57883	NC_003551	chromosome									
20094690	hypothetical protein MK1254	No	0		1241043	1241195	+		Methanopyrus_kandleri_AV19_uid57883	NC_003551	chromosome									
20094691	RNA-binding protein	No	0		1241416	1241982	+		Methanopyrus_kandleri_AV19_uid57883	NC_003551	chromosome		arCOG07759	2 (-)	V	VapC	PIN domain containing protein			
20094692	hypothetical protein MK1256	No	0		1241966	1242934	-		Methanopyrus_kandleri_AV19_uid57883	NC_003551	chromosome	clust_100								
20094693	hypothetical protein MK1257	No	0		1243554	1244471	-		Methanopyrus_kandleri_AV19_uid57883	NC_003551	chromosome									
===								23	Methanopyrus_kandleri_AV19_uid57883	NC_003551	chromosome									
20094697	Fe2+/Zn2+ uptake regulator	No	0	YES	1253762	1254154	+		Methanopyrus_kandleri_AV19_uid57883	NC_003551	chromosome									
20094698	ATPase involved in chromosome partitioning	No	0	YES	1254244	1255155	+		Methanopyrus_kandleri_AV19_uid57883	NC_003551	chromosome		arCOG00586	8 (-)	D	Soj	ATPase involved in chromosome partitioning, ParA family			
20094699	hypothetical protein MK1263	No	0	YES	1255170	1255841	+		Methanopyrus_kandleri_AV19_uid57883	NC_003551	chromosome									
20094700	hypothetical protein MK1264	No	0	YES	1255904	1257532	+		Methanopyrus_kandleri_AV19_uid57883	NC_003551	chromosome									
20094701	hypothetical protein MK1265	No	0	YES	1257546	1258277	+		Methanopyrus_kandleri_AV19_uid57883	NC_003551	chromosome									
20094702	hypothetical protein MK1266	No	0	YES	1258311	1259615	+		Methanopyrus_kandleri_AV19_uid57883	NC_003551	chromosome	clust_77								
20094703	hypothetical protein MK1267	No	0	YES	1259840	1261165	+		Methanopyrus_kandleri_AV19_uid57883	NC_003551	chromosome	clust_77						0.00003	COG0419	ATPase involved in DNA repair
20094704	hypothetical protein MK1268	Yes	0	YES	1261784	1263256	-		Methanopyrus_kandleri_AV19_uid57883	NC_003551	chromosome									
20094705	hypothetical protein MK1269	No	0	YES	1264021	1264473	+		Methanopyrus_kandleri_AV19_uid57883	NC_003551	chromosome							1E-75	pfam07913	Protein of unknown function (DUF1678). T
20094706	hypothetical protein MK1270	No	0	YES	1264935	1265888	-		Methanopyrus_kandleri_AV19_uid57883	NC_003551	chromosome									
20094707	hypothetical protein MK1271	No	0	YES	1266112	1267695	-		Methanopyrus_kandleri_AV19_uid57883	NC_003551	chromosome							9E-11	pfam03157	High molecular weight glutenin subunit.
20094708	hypothetical protein MK1272	No	0	YES	1267711	1269366	-		Methanopyrus_kandleri_AV19_uid57883	NC_003551	chromosome									
20094709	hypothetical protein MK1273	Yes	0	YES	1269348	1270529	-		Methanopyrus_kandleri_AV19_uid57883	NC_003551	chromosome									
20094710	metallo-beta-lactamase superfamily hydrolase	No	0		1270586	1271590	-		Methanopyrus_kandleri_AV19_uid57883	NC_003551	chromosome		arCOG00545	2 (-)	J	YSH1	Predicted exonuclease of the beta-lactamase fold	0.000000003	COG1236	Predicted exonuclease of the beta-lactam
20094711	hypothetical protein MK1275	No	0		1271731	1272240	-		Methanopyrus_kandleri_AV19_uid57883	NC_003551	chromosome		arCOG00104	1 (-)	C		Formaldehyde-activating enzyme nesessary for r	2E-67	COG1795	Formaldehyde-activating enzyme nesessary
20094712	hypothetical protein MK1276	No	3	YES	1272292	1273644	-		Methanopyrus_kandleri_AV19_uid57883	NC_003551	chromosome									
20094713	hypothetical protein MK1277	No	1	YES	1274035	1274772	+		Methanopyrus_kandleri_AV19_uid57883	NC_003551	chromosome									
20094714	hypothetical protein MK1278	No	0	YES	1275808	1277502	-		Methanopyrus_kandleri_AV19_uid57883	NC_003551	chromosome									
20094715	hypothetical protein MK1279	Yes	0	YES	1277672	1278295	+		Methanopyrus_kandleri_AV19_uid57883	NC_003551	chromosome									
20094716	hypothetical protein MK1280	No	0	YES	1278820	1279008	+		Methanopyrus_kandleri_AV19_uid57883	NC_003551	chromosome									
20094717	hypothetical protein MK1281	No	1	YES	1279599	1280219	-		Methanopyrus_kandleri_AV19_uid57883	NC_003551	chromosome									
20094718	hypothetical protein MK1282	No	0	YES	1280956	1281933	-		Methanopyrus_kandleri_AV19_uid57883	NC_003551	chromosome		arCOG06477	2 (-)	S		Uncharacterized conserved protein			
20094719	hypothetical protein MK1283	Yes	0	YES	1282214	1283809	-		Methanopyrus_kandleri_AV19_uid57883	NC_003551	chromosome									
===								6	Methanopyrus_kandleri_AV19_uid57883	NC_003551	chromosome									
20094722	hypothetical protein MK1286	No	0	YES	1285068	1286045	+		Methanopyrus_kandleri_AV19_uid57883	NC_003551	chromosome	clust_97								
20094723	hypothetical protein MK1287	No	0	YES	1286185	1286763	-		Methanopyrus_kandleri_AV19_uid57883	NC_003551	chromosome	clust_1						2E-92	pfam07913	Protein of unknown function (DUF1678). T
20094724	hypothetical protein MK1288	Yes	0	YES	1287009	1287983	-		Methanopyrus_kandleri_AV19_uid57883	NC_003551	chromosome	clust_30								
20094725	adenine-specific DNA methylase containing a Z	No	0	YES	1288128	1290386	+		Methanopyrus_kandleri_AV19_uid57883	NC_003551	chromosome		arCOG00889	6 (-)	L		Adenine-specific DNA methylase containing a Zn	9E-91	COG1743	Adenine-specific DNA methylase containin
20094726	hypothetical protein MK1290	No	0	YES	1290370	1291122	+		Methanopyrus_kandleri_AV19_uid57883	NC_003551	chromosome									
20094727	hypothetical protein MK1291	No	0	YES	1291279	1291923	-		Methanopyrus_kandleri_AV19_uid57883	NC_003551	chromosome	clust_1						2E-92	pfam07913	Protein of unknown function (DUF1678). T
===								14	Methanopyrus_kandleri_AV19_uid57883	NC_003551	chromosome									
20094736	hypothetical protein MK1300	No	0	YES	1308020	1308490	-		Methanopyrus_kandleri_AV19_uid57883	NC_003551	chromosome									
20094737	Terpene cyclase/mutase family protein	Yes	0	YES	1308525	1310213	-		Methanopyrus_kandleri_AV19_uid57883	NC_003551	chromosome		arCOG07147	1 (-)	I	SqhC	Squalene cyclase	5E-17	COG1657	Squalene cyclase
20094738	hypothetical protein MK1302	No	0	YES	1311974	1312216	+		Methanopyrus_kandleri_AV19_uid57883	NC_003551	chromosome									
20094739	hypothetical protein MK1303	Yes	0	YES	1312185	1313237	-		Methanopyrus_kandleri_AV19_uid57883	NC_003551	chromosome									
20094740	hypothetical protein MK1304	No	3	YES	1313373	1314599	-		Methanopyrus_kandleri_AV19_uid57883	NC_003551	chromosome							0.00008	pfam07172	Glycine rich protein family. This family
20094741	hypothetical protein MK1305	No	12	YES	1314596	1316125	-		Methanopyrus_kandleri_AV19_uid57883	NC_003551	chromosome									
20094742	glycosyltransferase	No	0		1316132	1317607	-		Methanopyrus_kandleri_AV19_uid57883	NC_003551	chromosome		arCOG01403	4 (-)	M	RfaG	Glycosyltransferase	2E-28	COG0438	Glycosyltransferase
20094743	DNA polymerase beta family nucleotidyltransfe	No	0	YES	1319237	1319530	-		Methanopyrus_kandleri_AV19_uid57883	NC_003551	chromosome		arCOG01205	1 (-)	V		Minimal nucleotidyltransferase	6E-11	COG1708	Predicted nucleotidyltransferases
20094744	P-loop ATPase	No	0	YES	1319573	1321492	-		Methanopyrus_kandleri_AV19_uid57883	NC_003551	chromosome									
20094745	hypothetical protein MK1309	No	0	YES	1322642	1323265	+		Methanopyrus_kandleri_AV19_uid57883	NC_003551	chromosome	clust_1						3E-94	pfam07913	Protein of unknown function (DUF1678). T
20094746	hypothetical protein MK1310	No	0	YES	1324335	1324640	-		Methanopyrus_kandleri_AV19_uid57883	NC_003551	chromosome									
20094747	the argonaute protein, implicated in translation	No	0	YES	1324652	1326787	-		Methanopyrus_kandleri_AV19_uid57883	NC_003551	chromosome		arCOG03890	1	V		Argonaute homolog, implicated in RNA metabolis	4E-168	COG1431	Argonaute homolog, implicated in RNA met
20094748	hypothetical protein MK1312	No	0	YES	1326771	1327766	-		Methanopyrus_kandleri_AV19_uid57883	NC_003551	chromosome		arCOG01452	5 (-)	V	Cas1	CRISPR-associated protein Cas1	2E-39	pfam01867	CRISPR associated protein Cas1. Clustere
20094749	hypothetical protein MK1313	Yes	0	YES	1329452	1330918	-		Methanopyrus_kandleri_AV19_uid57883	NC_003551	chromosome	clust_74								
===								38	Methanopyrus_kandleri_AV19_uid57883	NC_003551	chromosome									
20094760	hypothetical protein MK1324	No	4		1342074	1342703	+		Methanopyrus_kandleri_AV19_uid57883	NC_003551	chromosome									
20094761	Ras-like GTPase activity regulator	No	0		1342985	1343332	+		Methanopyrus_kandleri_AV19_uid57883	NC_003551	chromosome		arCOG02603	1 (-)	R		Roadblock/LC7 domain	8E-30	COG2018	Uncharacterized distant relative of home
20094762	hypothetical protein MK1326	No	0		1344045	1344728	+		Methanopyrus_kandleri_AV19_uid57883	NC_003551	chromosome									
20094763	hypothetical protein MK1327	No	4		1344701	1345228	+		Methanopyrus_kandleri_AV19_uid57883	NC_003551	chromosome									
20094764	hypothetical protein MK1328	No	0		1345308	1345556	-		Methanopyrus_kandleri_AV19_uid57883	NC_003551	chromosome									
20094765	hypothetical protein MK1329	No	0		1345608	1346639	-		Methanopyrus_kandleri_AV19_uid57883	NC_003551	chromosome									
20094766	hypothetical protein MK1330	Yes	0		1346857	1349094	-		Methanopyrus_kandleri_AV19_uid57883	NC_003551	chromosome									
20094767	hypothetical protein MK1331	Yes	0		1349240	1350568	-		Methanopyrus_kandleri_AV19_uid57883	NC_003551	chromosome	clust_74								
20094768	hypothetical protein MK1332	No	1		1351003	1351692	+		Methanopyrus_kandleri_AV19_uid57883	NC_003551	chromosome									
20094769	hypothetical protein MK1333	No	3		1351717	1352718	+		Methanopyrus_kandleri_AV19_uid57883	NC_003551	chromosome									
20094770	membrane-bound metal-dependent hydrolase	Yes	0		1352753	1353799	-		Methanopyrus_kandleri_AV19_uid57883	NC_003551	chromosome							0.0000003	COG1988	Predicted membrane-bound metal-dependent
20094771	Zn-dependent hydrolase	No	0		1353804	1354355	-		Methanopyrus_kandleri_AV19_uid57883	NC_003551	chromosome									
20094772	hypothetical protein MK1336	Yes	0		1354689	1355963	-		Methanopyrus_kandleri_AV19_uid57883	NC_003551	chromosome									
20094773	hypothetical protein MK1337	No	0		1356271	1356459	-		Methanopyrus_kandleri_AV19_uid57883	NC_003551	chromosome									
20094774	hypothetical protein MK1338	No	0		1356793	1357287	-		Methanopyrus_kandleri_AV19_uid57883	NC_003551	chromosome									
20094775	hypothetical protein MK1339	Yes	2		1357826	1360414	-		Methanopyrus_kandleri_AV19_uid57883	NC_003551	chromosome									
20094776	hypothetical protein MK1340	No	0		1360653	1361492	+		Methanopyrus_kandleri_AV19_uid57883	NC_003551	chromosome									
20094777	hypothetical protein MK1341	No	0		1361489	1361719	+		Methanopyrus_kandleri_AV19_uid57883	NC_003551	chromosome									
20094778	hypothetical protein MK1342	No	0		1361829	1362332	+		Methanopyrus_kandleri_AV19_uid57883	NC_003551	chromosome									
20094779	hypothetical protein MK1343	No	0		1364466	1365077	+		Methanopyrus_kandleri_AV19_uid57883	NC_003551	chromosome	clust_1						2E-97	pfam07913	Protein of unknown function (DUF1678). T
20094780	hypothetical protein MK1344	No	0		1365140	1366013	+		Methanopyrus_kandleri_AV19_uid57883	NC_003551	chromosome	clust_100								
20094781	Fe-S oxidoreductase	No	0		1366319	1367176	-		Methanopyrus_kandleri_AV19_uid57883	NC_003551	chromosome		arCOG00938	12 (-)	R		Radical SAM superfamily enzyme	9E-21	COG0535	Predicted Fe-S oxidoreductases
20094782	hypothetical protein MK1346	Yes	0		1367297	1368256	-		Methanopyrus_kandleri_AV19_uid57883	NC_003551	chromosome	clust_30								
20094783	hypothetical protein MK1347	No	2		1368270	1368527	-		Methanopyrus_kandleri_AV19_uid57883	NC_003551	chromosome									
20094784	hypothetical protein MK1348	No	6		1369122	1369865	-		Methanopyrus_kandleri_AV19_uid57883	NC_003551	chromosome									
20094785	hypothetical protein MK1349	No	0		1369858	1370589	-		Methanopyrus_kandleri_AV19_uid57883	NC_003551	chromosome									
20094786	transglutaminase-like supefamily cysteine prote	No	0		1370729	1371478	-		Methanopyrus_kandleri_AV19_uid57883	NC_003551	chromosome		arCOG02165	4 (-)	E		Transglutaminase-like enzyme, putative cysteine	0.000007	pfam01841	Transglutaminase-like superfamily. This
20094787	CobN/Mg-chelatase family protein	No	0		1371767	1375339	-		Methanopyrus_kandleri_AV19_uid57883	NC_003551	chromosome		arCOG03022	5 (-)	H	CobN	Cobalamin biosynthesis protein CobN or related Mg-chelatase		pfam02514	CobN/Magnesium Chelatase. This family co
20094788	hypothetical protein MK1352	No	5		1375488	1376102	+		Methanopyrus_kandleri_AV19_uid57883	NC_003551	chromosome									
20094789	hypothetical protein MK1353	No	0		1376114	1376947	+		Methanopyrus_kandleri_AV19_uid57883	NC_003551	chromosome									
20094790	hypothetical protein MK1354	No	4		1376796	1377713	+		Methanopyrus_kandleri_AV19_uid57883	NC_003551	chromosome									
20094791	hypothetical protein MK1355	No	5		1378052	1378888	+		Methanopyrus_kandleri_AV19_uid57883	NC_003551	chromosome									
20094792	hypothetical protein MK1356	No	4		1379071	1380000	+		Methanopyrus_kandleri_AV19_uid57883	NC_003551	chromosome									
20094793	hypothetical protein MK1357	No	4		1380143	1380862	+		Methanopyrus_kandleri_AV19_uid57883	NC_003551	chromosome									
20094794	putative component of a threonine efflux syster	No	5		1381069	1381686	+		Methanopyrus_kandleri_AV19_uid57883	NC_003551	chromosome		arCOG01947	2 (-)	E	RhtB	Putative threonine efflux protein	4E-15	COG1280	Putative threonine efflux protein
20094795	hypothetical protein MK1359	No	0		1381905	1382150	-		Methanopyrus_kandleri_AV19_uid57883	NC_003551	chromosome									
20094796	hypothetical protein MK1360	No	4		1382453	1383180	+		Methanopyrus_kandleri_AV19_uid57883	NC_003551	chromosome									
20094797	hypothetical protein MK1361	Yes	0		1384064	1385821	+		Methanopyrus_kandleri_AV19_uid57883	NC_003551	chromosome									
===								5	Methanopyrus_kandleri_AV19_uid57883	NC_003551	chromosome									
20094855	hypothetical protein MK1419	No	3		1447622	1447993	+		Methanopyrus_kandleri_AV19_uid57883	NC_003551	chromosome									
20094856	hypothetical protein MK1420	No	0		1447990	1448730	+		Methanopyrus_kandleri_AV19_uid57883	NC_003551	chromosome									
20094857	hypothetical protein MK1421	No	0		1448743	1449780	+		Methanopyrus_kandleri_AV19_uid57883	NC_003551	chromosome									
20094858	dihydrodipicolinate reductase	No	0		1449777	1450604	+		Methanopyrus_kandleri_AV19_uid57883	NC_003551	chromosome		arCOG04393	1 (-)	E	DapB	Dihydrodipicolinate reductase	3E-85	COG0289	Dihydrodipicolinate reductase
20094859	hypothetical protein MK1423	Yes	0		1450639	1451508	+		Methanopyrus_kandleri_AV19_uid57883	NC_003551	chromosome									
===								8	Methanoregula_boonei_6A8_uid58815	NC_009712	chromosome									
154149923	hypothetical protein Mboo_0378	Yes	2	YES	368753	370075	+		Methanoregula_boonei_6A8_uid58815	NC_009712	chromosome		arCOG02491	7 (-)	S		WD40 repeats containing protein			
154149924	hypothetical protein Mboo_0379	No	0	YES	370341	370703	+		Methanoregula_boonei_6A8_uid58815	NC_009712	chromosome									
154149925	hypothetical protein Mboo_0380	No	3	YES	370933	371301	+		Methanoregula_boonei_6A8_uid58815	NC_009712	chromosome		arCOG09582	2	S		Uncharacterized conserved protein			
154149926	hypothetical protein Mboo_0381	No	0	YES	371850	372119	+		Methanoregula_boonei_6A8_uid58815	NC_009712	chromosome		arCOG09583	7	S		Uncharacterized conserved protein			
154149927	hypothetical protein Mboo_0382	No	0	YES	372116	372520	+		Methanoregula_boonei_6A8_uid58815	NC_009712	chromosome									
154149928	hypothetical protein Mboo_0383	No	0	YES	373178	373681	+		Methanoregula_boonei_6A8_uid58815	NC_009712	chromosome		arCOG06904	7 (-)	S		Uncharacterized conserved protein			
154149929	hypothetical protein Mboo_0384	No	0	YES	373974	374450	+		Methanoregula_boonei_6A8_uid58815	NC_009712	chromosome	clust_5								
154149930	hypothetical protein Mboo_0385	No	5	YES	374443	375027	+		Methanoregula_boonei_6A8_uid58815	NC_009712	chromosome	clust_35								
===								5	Methanoregula_boonei_6A8_uid58815	NC_009712	chromosome									
154149938	hypothetical protein Mboo_0393	No	0		380731	381030	+		Methanoregula_boonei_6A8_uid58815	NC_009712	chromosome	clust_342								
154149939	acyltransferase 3	No	10		381429	382559	-		Methanoregula_boonei_6A8_uid58815	NC_009712	chromosome							0.000000006	pfam01757	Acyltransferase family. This family incl
154149940	hypothetical protein Mboo_0395	No	5		382556	383128	-		Methanoregula_boonei_6A8_uid58815	NC_009712	chromosome		arCOG09487	2	S		Uncharacterized conserved protein			
154149941	MarR family transcriptional regulator	No	0		383125	383622	-		Methanoregula_boonei_6A8_uid58815	NC_009712	chromosome		arCOG03182	3 (-)	K	MarR	Transcriptional regulator, MarR family	0.0000002	COG1846	Transcriptional regulators
154149942	hypothetical protein Mboo_0397	No	0	YES	383930	384169	+		Methanoregula_boonei_6A8_uid58815	NC_009712	chromosome									
===								5	Methanoregula_boonei_6A8_uid58815	NC_009712	chromosome									
154149956	hypothetical protein Mboo_0411	No	1		395740	395910	+		Methanoregula_boonei_6A8_uid58815	NC_009712	chromosome	clust_62								
154149957	hypothetical protein Mboo_0412	No	0		395894	396394	+		Methanoregula_boonei_6A8_uid58815	NC_009712	chromosome	clust_5								
154149958	hypothetical protein Mboo_0413	No	5		396387	396971	+		Methanoregula_boonei_6A8_uid58815	NC_009712	chromosome	clust_35								
154149959	hypothetical protein Mboo_0415	Yes	5		399902	400891	+		Methanoregula_boonei_6A8_uid58815	NC_009712	chromosome		arCOG09466	1 (-)	P		Cation transporter family protein			
154149960	hypothetical protein Mboo_0416	No	0		400996	401445	-		Methanoregula_boonei_6A8_uid58815	NC_009712	chromosome									
===								7	Methanoregula_boonei_6A8_uid58815	NC_009712	chromosome									
154150049	phage integrase family protein	No	0	YES	487967	489241	+		Methanoregula_boonei_6A8_uid58815	NC_009712	chromosome		arCOG01242	14 (-)	L	XerC	Integrase	1E-14	pfam00589	Phage integrase family. Members of this
154150050	hypothetical protein Mboo_0507	No	0	YES	489366	490007	-		Methanoregula_boonei_6A8_uid58815	NC_009712	chromosome									
154150051	hypothetical protein Mboo_0508	No	0	YES	490577	491635	+		Methanoregula_boonei_6A8_uid58815	NC_009712	chromosome									
154150052	hypothetical protein Mboo_0509	No	0	YES	491792	492130	+		Methanoregula_boonei_6A8_uid58815	NC_009712	chromosome									
154150053	hypothetical protein Mboo_0510	No	0	YES	492706	492969	-		Methanoregula_boonei_6A8_uid58815	NC_009712	chromosome									
154150054	hypothetical protein Mboo_0511	No	1	YES	493090	493425	+		Methanoregula_boonei_6A8_uid58815	NC_009712	chromosome									
154150055	hypothetical protein Mboo_0512	No	1	YES	493425	494294	+		Methanoregula_boonei_6A8_uid58815	NC_009712	chromosome		arCOG07264	1 (-)	S		Predicted membrane protein with C-terminal Zn ribbon domain			
===								6	Methanoregula_boonei_6A8_uid58815	NC_009712	chromosome									
154150315	bifunctional formaldehyde-activating enzyme/3-	No	0		743704	744885	+		Methanoregula_boonei_6A8_uid58815	NC_009712	chromosome		arCOG00103	1 (-)	G		D-arabino 3-hexulose 6-phosphate formaldehyde	1E-64	COG1795	Formaldehyde-activating enzyme nesessary ; 3-hexulose-6-phosphate synthase and rela
154150316	hypothetical protein Mboo_0773	No	0		744985	745443	+		Methanoregula_boonei_6A8_uid58815	NC_009712	chromosome									
154150317	hypothetical protein Mboo_0774	No	0		745418	745702	-		Methanoregula_boonei_6A8_uid58815	NC_009712	chromosome									
154150318	hypothetical protein Mboo_0775	No	0		745933	746307	+		Methanoregula_boonei_6A8_uid58815	NC_009712	chromosome									
154150319	hypothetical protein Mboo_0776	No	0		746339	746806	+		Methanoregula_boonei_6A8_uid58815	NC_009712	chromosome									
154150320	alpha/beta hydrolase fold	Yes	1		747042	747896	-		Methanoregula_boonei_6A8_uid58815	NC_009712	chromosome		arCOG01648	3 (-)	R	MhpC	Alpha/beta superfamily hydrolase	2E-16	COG0596	Predicted hydrolases or acyltransferases
===								5	Methanoregula_boonei_6A8_uid58815	NC_009712	chromosome									
154150503	hypothetical protein Mboo_0960	Yes	2		946120	948231	-		Methanoregula_boonei_6A8_uid58815	NC_009712	chromosome							0.0000001	COG5295	Autotransporter adhesin
154150504	hypothetical protein Mboo_0961	Yes	3		948228	949232	-		Methanoregula_boonei_6A8_uid58815	NC_009712	chromosome		arCOG04932	1 (-)	S		Cell suface protein			
154150505	hypothetical protein Mboo_0962	No	0		949284	949700	-		Methanoregula_boonei_6A8_uid58815	NC_009712	chromosome									
154150506	hypothetical protein Mboo_0963	Yes	1		949905	950462	-		Methanoregula_boonei_6A8_uid58815	NC_009712	chromosome									
154150507	hypothetical protein Mboo_0964	No	1		950742	951980	+		Methanoregula_boonei_6A8_uid58815	NC_009712	chromosome									
===								6	Methanoregula_boonei_6A8_uid58815	NC_009712	chromosome									
154150801	nitroreductase	No	0		1283155	1283817	+		Methanoregula_boonei_6A8_uid58815	NC_009712	chromosome		arCOG00301	1 (-)	C	NfnB	Nitroreductase	0.00001	COG0778	Nitroreductase
154150802	hypothetical protein Mboo_1259	No	0		1284008	1284709	-		Methanoregula_boonei_6A8_uid58815	NC_009712	chromosome									
154150803	hypothetical protein Mboo_1260	No	4		1285505	1285858	+		Methanoregula_boonei_6A8_uid58815	NC_009712	chromosome									
154150804	hypothetical protein Mboo_1261	No	3		1285951	1286289	-		Methanoregula_boonei_6A8_uid58815	NC_009712	chromosome							0.000001	COG1320	Multisubunit Na+/H+ antiporter, MnhG sub
154150805	hypothetical protein Mboo_1262	No	2		1286286	1286549	-		Methanoregula_boonei_6A8_uid58815	NC_009712	chromosome									
154150806	hypothetical protein Mboo_1263	No	0		1286738	1288132	+		Methanoregula_boonei_6A8_uid58815	NC_009712	chromosome		arCOG00073	2 (-)	E	CysH	PAPS reductase related enzyme fused to RNA-b	2E-39	COG5270	PUA domain (predicted RNA-binding domain ; 3'-phosphoadenosine 5'-phosphosulfate su
===								7	Methanoregula_boonei_6A8_uid58815	NC_009712	chromosome									
154151033	large-conductance mechanosensitive channel-	No	2		1543704	1544081	+		Methanoregula_boonei_6A8_uid58815	NC_009712	chromosome		arCOG05213	2 (-)	M	MscL	Large-conductance mechanosensitive channel	2E-16	COG1970	Large-conductance mechanosensitive chann
154151034	hypothetical protein Mboo_1491	No	1		1544678	1548229	+		Methanoregula_boonei_6A8_uid58815	NC_009712	chromosome							7E-65	pfam11999	Protein of unknown function (DUF3494). T ; Protein of unknown function (DUF3494). T ; Protein of unknown function (DUF3494). T ; Protein of unknown function (DUF3494). T
154151035	anti-sigma-factor antagonist	No	0		1548840	1549688	+		Methanoregula_boonei_6A8_uid58815	NC_009712	chromosome		arCOG09390	2	S		Uncharacterized conserved protein	2E-18	COG1366	Anti-anti-sigma regulatory factor (antag ; RsbT co-antagonist protein rsbRD N-termi
154151036	anti-sigma-factor antagonist	No	0		1549714	1550118	+		Methanoregula_boonei_6A8_uid58815	NC_009712	chromosome		arCOG09390	2	S		Uncharacterized conserved protein	0.00000001	COG1366	Anti-anti-sigma regulatory factor (antag
154151037	putative anti-sigma regulatory factor, serine/thre	No	0		1550090	1550494	+		Methanoregula_boonei_6A8_uid58815	NC_009712	chromosome		arCOG09391	1	S		Uncharacterized conserved protein	1E-10	COG2172	Anti-sigma regulatory factor (Ser/Thr pr
154151038	stage II sporulation E family protein	No	0		1550485	1551525	+		Methanoregula_boonei_6A8_uid58815	NC_009712	chromosome		arCOG09392	1	S		Uncharacterized conserved protein			
154151039	signal transduction histidine kinase	No	0		1551522	1555283	+		Methanoregula_boonei_6A8_uid58815	NC_009712	chromosome	arCOG02330,a	arCOG02330	2 (-)	T	AtoS	Bacterio-opsin activator protein (contains PAS, G	3E-37	COG3920	Signal transduction histidine kinase
===								9	Methanoregula_boonei_6A8_uid58815	NC_009712	chromosome									
154151047	pyridine nucleotide-disulphide oxidoreductase	No	1		1567269	1568657	+		Methanoregula_boonei_6A8_uid58815	NC_009712	chromosome		arCOG01068	1 (-)	C	Lpd	Pyruvate/2-oxoglutarate dehydrogenase comple	3E-91	COG1249	Pyruvate/2-oxoglutarate dehydrogenase co
154151048	hypothetical protein Mboo_1505	Yes	1		1568799	1569695	+		Methanoregula_boonei_6A8_uid58815	NC_009712	chromosome		arCOG08235	1	S		Uncharacterized conserved protein	6E-15	COG4315	Uncharacterized protein conserved in bac ; Uncharacterized protein conserved in bac
154151049	hypothetical protein Mboo_1506	No	1		1569764	1570039	+		Methanoregula_boonei_6A8_uid58815	NC_009712	chromosome									
154151050	hypothetical protein Mboo_1507	No	0		1570060	1570305	-		Methanoregula_boonei_6A8_uid58815	NC_009712	chromosome									
154151051	hypothetical protein Mboo_1508	No	0		1570315	1570857	+		Methanoregula_boonei_6A8_uid58815	NC_009712	chromosome									
154151052	hypothetical protein Mboo_1509	No	0		1570996	1571388	-		Methanoregula_boonei_6A8_uid58815	NC_009712	chromosome									
154151053	Serine/threonine protein phosphatase-like prot	No	0		1571659	1572387	+		Methanoregula_boonei_6A8_uid58815	NC_009712	chromosome							6E-10	COG0631	Serine/threonine protein phosphatase
154151054	hypothetical protein Mboo_1511	No	0		1572642	1572896	+		Methanoregula_boonei_6A8_uid58815	NC_009712	chromosome		arCOG03714	1 (-)	F	NrdD	ATP cone domain			
154151055	hypothetical protein Mboo_1512	No	0		1572920	1573132	+		Methanoregula_boonei_6A8_uid58815	NC_009712	chromosome		arCOG09596	1	S		Uncharacterized conserved protein			
===								8	Methanoregula_boonei_6A8_uid58815	NC_009712	chromosome									
154151089	hypothetical protein Mboo_1547	No	0		1617702	1618079	-		Methanoregula_boonei_6A8_uid58815	NC_009712	chromosome									
154151090	hypothetical protein Mboo_1548	No	0		1618618	1618929	-		Methanoregula_boonei_6A8_uid58815	NC_009712	chromosome	clust_342								
154151091	putative transcriptional regulator	No	0		1619271	1620119	-		Methanoregula_boonei_6A8_uid58815	NC_009712	chromosome	arCOG01686,a	arCOG01686	3 (-)	K	ArsR	Transcriptional regulator containing HTH domain,	0.0000002	COG2202	FOG: PAS/PAC domain
154151092	hypothetical protein Mboo_1550	No	0		1620098	1620430	-		Methanoregula_boonei_6A8_uid58815	NC_009712	chromosome									
154151093	hypothetical protein Mboo_1551	Yes	1		1620853	1621764	+		Methanoregula_boonei_6A8_uid58815	NC_009712	chromosome		arCOG09597	1	S		Uncharacterized conserved protein	4E-44	pfam12863	Domain of unknown function (DUF3821). Th
154151094	CBS domain-containing protein	No	6		1621848	1622984	-		Methanoregula_boonei_6A8_uid58815	NC_009712	chromosome		arCOG00607	2 (-)	R		Zn-dependent protease fused to CBS domain	7E-20	COG1994	Zn-dependent proteases
154151095	hypothetical protein Mboo_1553	No	0		1623103	1623372	-		Methanoregula_boonei_6A8_uid58815	NC_009712	chromosome									
154151096	hypothetical protein Mboo_1554	No	0		1623550	1623858	+		Methanoregula_boonei_6A8_uid58815	NC_009712	chromosome									
===								5	Methanoregula_boonei_6A8_uid58815	NC_009712	chromosome									
154151141	hypothetical protein Mboo_1599	No	2		1670737	1670925	+		Methanoregula_boonei_6A8_uid58815	NC_009712	chromosome									
154151142	hypothetical protein Mboo_1600	No	1		1671494	1671814	-		Methanoregula_boonei_6A8_uid58815	NC_009712	chromosome									
154151143	NHL repeat-containing protein	Yes	1		1672601	1674076	+		Methanoregula_boonei_6A8_uid58815	NC_009712	chromosome	arCOG02514,a	arCOG02514	1 (-)	S		PKD repeats containing protein	5E-18	COG3291	FOG: PKD repeat
154151144	hypothetical protein Mboo_1602	No	0		1675149	1676042	+		Methanoregula_boonei_6A8_uid58815	NC_009712	chromosome		arCOG09571	1	S		Uncharacterized conserved protein			
154151145	hypothetical protein Mboo_1603	No	0		1676212	1676376	+		Methanoregula_boonei_6A8_uid58815	NC_009712	chromosome									
===								5	Methanoregula_boonei_6A8_uid58815	NC_009712	chromosome									
154151406	hypothetical protein Mboo_1866	No	0		1940459	1941061	-		Methanoregula_boonei_6A8_uid58815	NC_009712	chromosome									
154151407	hypothetical protein Mboo_1867	No	2		1941145	1941396	+		Methanoregula_boonei_6A8_uid58815	NC_009712	chromosome									
154151408	putative methylase	No	0		1941393	1941950	-		Methanoregula_boonei_6A8_uid58815	NC_009712	chromosome		arCOG00109	2 (-)	J	HemK	Methylase of polypeptide chain release factors	9E-25	COG2890	Methylase of polypeptide chain release f
154151409	hypothetical protein Mboo_1869	No	2		1942037	1942309	+		Methanoregula_boonei_6A8_uid58815	NC_009712	chromosome		arCOG04003	2	S		Uncharacterized conserved protein			
154151410	hypothetical protein Mboo_1870	No	2		1942445	1942744	-		Methanoregula_boonei_6A8_uid58815	NC_009712	chromosome									
===								6	Methanoregula_boonei_6A8_uid58815	NC_009712	chromosome									
154151949	hypothetical protein Mboo_2410	No	0		2480160	2480774	-		Methanoregula_boonei_6A8_uid58815	NC_009712	chromosome		arCOG04148	1 (-)	N	FlaH	Predicted ATPase involved in biogenesis of arch	0.00001	COG0467	RecA-superfamily ATPases implicated in s
154151950	hypothetical protein Mboo_2411	No	4		2481043	2481489	+		Methanoregula_boonei_6A8_uid58815	NC_009712	chromosome		arCOG09548	1	S		Uncharacterized conserved protein			
154151951	hypothetical protein Mboo_2412	No	3		2481956	2482360	-		Methanoregula_boonei_6A8_uid58815	NC_009712	chromosome									
154151952	hypothetical protein Mboo_2413	Yes	0		2482556	2483239	+		Methanoregula_boonei_6A8_uid58815	NC_009712	chromosome									
154151953	hypothetical protein Mboo_2414	No	2		2483330	2483659	+		Methanoregula_boonei_6A8_uid58815	NC_009712	chromosome									
154151954	exodeoxyribonuclease III Xth	No	0		2483673	2484485	+		Methanoregula_boonei_6A8_uid58815	NC_009712	chromosome		arCOG02207	2 (-)	L	XthA	Exonuclease III	1E-55	COG0708	Exonuclease III
===								6	Methanoregula_formicicum_SMSP_uid184406	NC_019943	chromosome									
432329553	transglutaminase-like enzyme, predicted cyste	Yes	1		97619	98902	-		Methanoregula_formicicum_SMSP_uid184406	NC_019943	chromosome		arCOG02165	4 (-)	E		Transglutaminase-like enzyme, putative cysteine	0.000005	COG1305	Transglutaminase-like enzymes, putative
432329554	hypothetical protein Metfor_0104	No	5		99140	100063	-		Methanoregula_formicicum_SMSP_uid184406	NC_019943	chromosome									
432329555	hypothetical protein Metfor_0105	No	5		100209	101147	-		Methanoregula_formicicum_SMSP_uid184406	NC_019943	chromosome									
432329556	Peptidase propeptide domain-containing prote	Yes	1		101159	101992	-		Methanoregula_formicicum_SMSP_uid184406	NC_019943	chromosome		arCOG06558	10	S		Uncharacterized conserved protein			
432329557	hypothetical protein Metfor_0107	Yes	1		102006	102680	-		Methanoregula_formicicum_SMSP_uid184406	NC_019943	chromosome		arCOG11246	3	S		Uncharacterized conserved protein			
432329558	hypothetical protein Metfor_0108	Yes	1		103205	103654	+		Methanoregula_formicicum_SMSP_uid184406	NC_019943	chromosome		arCOG04987	6 (-)	S		Uncharacterized conserved protein			
===								6	Methanoregula_formicicum_SMSP_uid184406	NC_019943	chromosome									
432329760	hypothetical protein Metfor_0319	No	3		316567	317052	-		Methanoregula_formicicum_SMSP_uid184406	NC_019943	chromosome		arCOG07551	1	S		Uncharacterized conserved protein			
432329761	hypothetical protein Metfor_0320	No	0		317208	317522	+		Methanoregula_formicicum_SMSP_uid184406	NC_019943	chromosome									
432329762	rRNA large subunit m3Psi methyltransferase Rl	No	0		317601	318080	+		Methanoregula_formicicum_SMSP_uid184406	NC_019943	chromosome		arCOG05111	1 (-)	R		SPOUT methyltransferase superfamily protein	3E-50	pfam02590	Predicted SPOUT methyltransferase. This
432329763	hypothetical protein Metfor_0322	No	3		318164	318505	+		Methanoregula_formicicum_SMSP_uid184406	NC_019943	chromosome									
432329764	hypothetical protein Metfor_0323	No	3		318506	318838	+		Methanoregula_formicicum_SMSP_uid184406	NC_019943	chromosome									
432329765	hypothetical protein Metfor_0324	No	0		318835	319059	+		Methanoregula_formicicum_SMSP_uid184406	NC_019943	chromosome									
===								7	Methanoregula_formicicum_SMSP_uid184406	NC_019943	chromosome									
432330310	magnesium chelatase, H subunit	No	0		879197	883006	-		Methanoregula_formicicum_SMSP_uid184406	NC_019943	chromosome		arCOG03022	5 (-)	H	CobN	Cobalamin biosynthesis protein CobN or related Mg-chelatase		pfam02514	CobN/Magnesium Chelatase. This family co
432330311	hypothetical protein Metfor_0891	No	0		883345	883863	-		Methanoregula_formicicum_SMSP_uid184406	NC_019943	chromosome									
432330312	hypothetical protein Metfor_0892	No	0		883973	884236	-		Methanoregula_formicicum_SMSP_uid184406	NC_019943	chromosome		arCOG10960	2	S		Uncharacterized conserved protein			
432330313	hypothetical protein Metfor_0893	No	0		884253	884750	-		Methanoregula_formicicum_SMSP_uid184406	NC_019943	chromosome									
432330314	hypothetical protein Metfor_0894	No	0		884877	885701	-		Methanoregula_formicicum_SMSP_uid184406	NC_019943	chromosome									
432330315	hypothetical protein Metfor_0896	No	0		886357	887214	-		Methanoregula_formicicum_SMSP_uid184406	NC_019943	chromosome		arCOG03439	2 (-)	S		vWFa domain containing protein			
432330316	hypothetical protein Metfor_0897	No	0		887216	888274	-		Methanoregula_formicicum_SMSP_uid184406	NC_019943	chromosome		arCOG10960	2	S		Uncharacterized conserved protein			
===								6	Methanoregula_formicicum_SMSP_uid184406	NC_019943	chromosome									
432330460	hypothetical protein Metfor_1047	Yes	1		1026417	1028063	-		Methanoregula_formicicum_SMSP_uid184406	NC_019943	chromosome		arCOG09580	1	S		Uncharacterized conserved protein			
432330461	hypothetical protein Metfor_1048	Yes	4		1028187	1028612	-		Methanoregula_formicicum_SMSP_uid184406	NC_019943	chromosome		arCOG08981	1	S		Uncharacterized conserved protein			
432330462	putative transcriptional regulator	No	0		1028618	1028917	-		Methanoregula_formicicum_SMSP_uid184406	NC_019943	chromosome		arCOG00731	3 (-)	K	ArsR	Transcriptional regulator containing HTH domain, ArsR family			
432330463	hypothetical protein Metfor_1050	No	1		1029254	1029403	-		Methanoregula_formicicum_SMSP_uid184406	NC_019943	chromosome									
432330464	hypothetical protein Metfor_1051	Yes	1		1029557	1029886	-		Methanoregula_formicicum_SMSP_uid184406	NC_019943	chromosome									
432330465	hypothetical protein Metfor_1052	No	4		1030060	1030578	-		Methanoregula_formicicum_SMSP_uid184406	NC_019943	chromosome									
===								5	Methanoregula_formicicum_SMSP_uid184406	NC_019943	chromosome									
432330468	hypothetical protein Metfor_1055	Yes	5		1034045	1034641	+		Methanoregula_formicicum_SMSP_uid184406	NC_019943	chromosome		arCOG09592	2	S		Uncharacterized conserved protein			
432330469	hypothetical protein Metfor_1056	Yes	1		1034845	1035402	+		Methanoregula_formicicum_SMSP_uid184406	NC_019943	chromosome									
432330470	hypothetical protein Metfor_1057	Yes	1		1035624	1036439	-		Methanoregula_formicicum_SMSP_uid184406	NC_019943	chromosome									
432330471	hypothetical protein Metfor_1058	No	5		1036695	1037342	+		Methanoregula_formicicum_SMSP_uid184406	NC_019943	chromosome		arCOG02879	3 (-)	S		Uncharacterized conserved protein	1E-28	pfam13197	Protein of unknown function (DUF4013). T
432330472	hypothetical protein Metfor_1059	No	2		1037520	1037711	-		Methanoregula_formicicum_SMSP_uid184406	NC_019943	chromosome									
===								6	Methanoregula_formicicum_SMSP_uid184406	NC_019943	chromosome									
432330535	hypothetical protein Metfor_1123	No	0		1089407	1089754	-		Methanoregula_formicicum_SMSP_uid184406	NC_019943	chromosome		arCOG03390	1 (-)	S		Uncharacterized conserved protein			
432330536	hypothetical protein Metfor_1124	No	0		1089770	1089913	-		Methanoregula_formicicum_SMSP_uid184406	NC_019943	chromosome									
432330537	hypothetical protein Metfor_1125	No	0		1089961	1090338	-		Methanoregula_formicicum_SMSP_uid184406	NC_019943	chromosome									
432330538	hypothetical protein Metfor_1126	No	0		1090521	1090709	+		Methanoregula_formicicum_SMSP_uid184406	NC_019943	chromosome									
432330539	hypothetical protein Metfor_1127	No	0		1091125	1091283	+		Methanoregula_formicicum_SMSP_uid184406	NC_019943	chromosome									
432330540	hypothetical protein Metfor_1128	No	0		1092147	1093253	-		Methanoregula_formicicum_SMSP_uid184406	NC_019943	chromosome		arCOG03110	5 (-)	K		Predicted transciptional regulator, contains Fic ar	2E-25	COG3177	Fic family protein
===								23	Methanoregula_formicicum_SMSP_uid184406	NC_019943	chromosome									
432330765	hypothetical protein Metfor_1362	No	0		1320168	1320314	+		Methanoregula_formicicum_SMSP_uid184406	NC_019943	chromosome									
432330766	acetyltransferase	No	0		1320315	1320896	+		Methanoregula_formicicum_SMSP_uid184406	NC_019943	chromosome		arCOG04971	3 (-)	K	WecD	Acetyltransferase (GNAT) family			
432330767	hypothetical protein Metfor_1364	No	0		1321139	1321321	-		Methanoregula_formicicum_SMSP_uid184406	NC_019943	chromosome									
432330768	hypothetical protein Metfor_1365	No	0		1321508	1321972	-		Methanoregula_formicicum_SMSP_uid184406	NC_019943	chromosome									
432330769	hypothetical protein Metfor_1366	No	0		1321942	1322217	-		Methanoregula_formicicum_SMSP_uid184406	NC_019943	chromosome									
432330770	hypothetical protein Metfor_1367	No	0		1322220	1322417	-		Methanoregula_formicicum_SMSP_uid184406	NC_019943	chromosome									
432330771	hypothetical protein Metfor_1368	No	0		1322414	1322773	-		Methanoregula_formicicum_SMSP_uid184406	NC_019943	chromosome									
432330772	hypothetical protein Metfor_1369	No	0		1322775	1323014	-		Methanoregula_formicicum_SMSP_uid184406	NC_019943	chromosome									
432330773	hypothetical protein Metfor_1370	No	0		1323239	1323511	+		Methanoregula_formicicum_SMSP_uid184406	NC_019943	chromosome									
432330774	hypothetical protein Metfor_1371	Yes	0		1323382	1323987	-		Methanoregula_formicicum_SMSP_uid184406	NC_019943	chromosome									
432330775	hypothetical protein Metfor_1372	No	0		1324011	1324187	+		Methanoregula_formicicum_SMSP_uid184406	NC_019943	chromosome	clust_484								
432330776	hypothetical protein Metfor_1373	No	0		1324585	1325652	+		Methanoregula_formicicum_SMSP_uid184406	NC_019943	chromosome		arCOG01917	12 (-)	S		Zn-ribbon domain containing protein			
432330777	hypothetical protein Metfor_1374	No	0		1326331	1326933	+		Methanoregula_formicicum_SMSP_uid184406	NC_019943	chromosome									
432330778	hypothetical protein Metfor_1375	No	0		1327161	1327385	-		Methanoregula_formicicum_SMSP_uid184406	NC_019943	chromosome									
432330779	hypothetical protein Metfor_1376	No	0		1327388	1327843	-		Methanoregula_formicicum_SMSP_uid184406	NC_019943	chromosome									
432330780	hypothetical protein Metfor_1377	No	0		1328044	1328367	-		Methanoregula_formicicum_SMSP_uid184406	NC_019943	chromosome									
432330781	hypothetical protein Metfor_1378	No	0		1328364	1328870	-		Methanoregula_formicicum_SMSP_uid184406	NC_019943	chromosome									
432330782	hypothetical protein Metfor_1379	No	0		1328867	1330570	-		Methanoregula_formicicum_SMSP_uid184406	NC_019943	chromosome		arCOG06914	10 (-)	R		Phage or plasmid associated DNA primase	1E-21	COG3378	Phage associated DNA primase
432330783	hypothetical protein Metfor_1380	No	0		1330567	1330752	-		Methanoregula_formicicum_SMSP_uid184406	NC_019943	chromosome									
432330784	hypothetical protein Metfor_1381	No	0		1330752	1330985	-		Methanoregula_formicicum_SMSP_uid184406	NC_019943	chromosome									
432330785	hypothetical protein Metfor_1382	No	0		1330982	1331179	-		Methanoregula_formicicum_SMSP_uid184406	NC_019943	chromosome									
432330786	hypothetical protein Metfor_1383	No	0		1331157	1331369	-		Methanoregula_formicicum_SMSP_uid184406	NC_019943	chromosome									
432330787	hypothetical protein Metfor_1384	No	0		1331627	1332364	+		Methanoregula_formicicum_SMSP_uid184406	NC_019943	chromosome		arCOG04362	5 (-)	K		Predicted transcriptional regulator			
===								5	Methanoregula_formicicum_SMSP_uid184406	NC_019943	chromosome									
432330892	hypothetical protein Metfor_1495	No	0		1450174	1450284	-		Methanoregula_formicicum_SMSP_uid184406	NC_019943	chromosome									
432330893	hypothetical protein Metfor_1496	No	0		1450366	1450740	-		Methanoregula_formicicum_SMSP_uid184406	NC_019943	chromosome									

432330894	hypothetical protein Metfor_1497	No	4		1450835	1451359	-		Methanoregula_formicicum_SMSP_uid184406	NC_019943	chromosome			arCOG08234	2		S		Uncharacterized conserved protein			
432330895	archaeal flagellin-like protein	No	1		1451625	1452074	-		Methanoregula_formicicum_SMSP_uid184406	NC_019943	chromosome			arCOG02424	2	(-)	S		Uncharacterized conserved protein	1E-13	pfam07790	Protein of unknown function (DUF1628). T
432330896	hypothetical protein Metfor_1499	No	0		1452273	1452476	-		Methanoregula_formicicum_SMSP_uid184406	NC_019943	chromosome											
===								5	Methanoregula_formicicum_SMSP_uid184406	NC_019943	chromosome											
432330977	hypothetical protein Metfor_1583	No	0		1525773	1526201	-		Methanoregula_formicicum_SMSP_uid184406	NC_019943	chromosome			arCOG09441	8		L		HJR family endonuclease			
432330978	hypothetical protein Metfor_1584	No	0		1526555	1526719	+		Methanoregula_formicicum_SMSP_uid184406	NC_019943	chromosome											
432330979	hypothetical protein Metfor_1585	No	4		1526764	1527492	-		Methanoregula_formicicum_SMSP_uid184406	NC_019943	chromosome	clust_137										
432330980	RHS repeat-associated core domain protein	Yes	1		1527564	1531991	-		Methanoregula_formicicum_SMSP_uid184406	NC_019943	chromosome		arCOG02560,a	arCOG02560	1	(-)	S		Beta-propeller repeat containing protein	5E-53	COG3209	Rhs family protein
432330981	hypothetical protein Metfor_1587	Yes	0		1533035	1533415	+		Methanoregula_formicicum_SMSP_uid184406	NC_019943	chromosome											
===								5	Methanoregula_formicicum_SMSP_uid184406	NC_019943	chromosome											
432331082	periplasmic protease	Yes	2		1660698	1662377	-		Methanoregula_formicicum_SMSP_uid184406	NC_019943	chromosome									0.000000004	COG0793	Periplasmic protease
432331083	hypothetical protein Metfor_1692	No	0		1662461	1662652	-		Methanoregula_formicicum_SMSP_uid184406	NC_019943	chromosome											
432331084	hypothetical protein Metfor_1693	No	6		1662809	1663612	+		Methanoregula_formicicum_SMSP_uid184406	NC_019943	chromosome			arCOG02879	3	(-)	S		Uncharacterized conserved protein	2E-20	pfam13197	Protein of unknown function (DUF4013). T
432331085	hypothetical protein Metfor_1694	Yes	1		1663944	1664540	-		Methanoregula_formicicum_SMSP_uid184406	NC_019943	chromosome	clust_166										
432331086	hypothetical protein Metfor_1695	No	0		1664638	1664808	+		Methanoregula_formicicum_SMSP_uid184406	NC_019943	chromosome											
===								13	Methanoregula_formicicum_SMSP_uid184406	NC_019943	chromosome											
432331303	ATPase with chaperone activity, ATP-binding s	No	0		1876814	1879207	+		Methanoregula_formicicum_SMSP_uid184406	NC_019943	chromosome		arCOG03947,a	arCOG03947	2	(-)	O	clpA	ATP-binding subunits of Clp protease and DnaK/I	1E-170	COG0542	ATP-binding subunits of Clp protease and
432331304	hypothetical protein Metfor_1922	No	2		1879990	1880193	-		Methanoregula_formicicum_SMSP_uid184406	NC_019943	chromosome											
432331305	hypothetical protein Metfor_1923	No	1		1880294	1880509	-		Methanoregula_formicicum_SMSP_uid184406	NC_019943	chromosome											
432331306	hypothetical protein Metfor_1924	Yes	1		1880561	1881469	-		Methanoregula_formicicum_SMSP_uid184406	NC_019943	chromosome											
432331307	hypothetical protein Metfor_1925	No	0		1882269	1882796	+		Methanoregula_formicicum_SMSP_uid184406	NC_019943	chromosome											
432331308	competence protein	No	0		1882613	1883755	-		Methanoregula_formicicum_SMSP_uid184406	NC_019943	chromosome									0.00001	pfam06054	Competence protein CoiA-like family. Man
432331309	hypothetical protein Metfor_1927	No	0		1883756	1883938	+		Methanoregula_formicicum_SMSP_uid184406	NC_019943	chromosome											
432331310	hypothetical protein Metfor_1928	Yes	1		1883945	1884562	+		Methanoregula_formicicum_SMSP_uid184406	NC_019943	chromosome	clust_166										
432331311	hypothetical protein Metfor_1929	No	0		1884397	1884831	-		Methanoregula_formicicum_SMSP_uid184406	NC_019943	chromosome											
432331312	hypothetical protein Metfor_1930	Yes	1		1885318	1885830	-		Methanoregula_formicicum_SMSP_uid184406	NC_019943	chromosome			arCOG06822	4		S		Uncharacterized conserved protein			
432331313	hypothetical protein Metfor_1931	Yes	0		1885869	1886447	-		Methanoregula_formicicum_SMSP_uid184406	NC_019943	chromosome			arCOG06822	4		S		Uncharacterized conserved protein			
432331314	hypothetical protein Metfor_1932	Yes	1		1886470	1887693	-		Methanoregula_formicicum_SMSP_uid184406	NC_019943	chromosome			arCOG09593	8		S		Uncharacterized conserved protein			
432331315	hypothetical protein Metfor_1933	No	1		1888047	1888769	-		Methanoregula_formicicum_SMSP_uid184406	NC_019943	chromosome			arCOG04952	2	(-)	S		Uncharacterized conserved protein			
===								7	Methanoregula_formicicum_SMSP_uid184406	NC_019943	chromosome											
432331387	hypothetical protein Metfor_2009	No	0		1958462	1958725	-		Methanoregula_formicicum_SMSP_uid184406	NC_019943	chromosome											
432331388	hypothetical protein Metfor_2010	No	1		1958880	1960148	+		Methanoregula_formicicum_SMSP_uid184406	NC_019943	chromosome			arCOG01917	12	(-)	S		Zn-ribbon domain containing protein	0.000003	pfam05466	Brain acid soluble protein 1 (BASP1 prot
432331389	hypothetical protein Metfor_2011	No	0		1960118	1960264	-		Methanoregula_formicicum_SMSP_uid184406	NC_019943	chromosome											
432331390	hypothetical protein Metfor_2012	No	0		1961568	1961783	+		Methanoregula_formicicum_SMSP_uid184406	NC_019943	chromosome											
432331391	hypothetical protein Metfor_2013	No	0		1961786	1962238	+		Methanoregula_formicicum_SMSP_uid184406	NC_019943	chromosome											
432331392	putative transcriptional regulator with HTH doma	No	0		1962411	1963820	+		Methanoregula_formicicum_SMSP_uid184406	NC_019943	chromosome		arCOG03296,a	arCOG03296	2	(-)	K		Predicted transcriptional regulator containing an H	4E-58	COG2865	Predicted transcriptional regulator cont
432331393	hypothetical protein Metfor_2015	No	0		1964235	1966256	-		Methanoregula_formicicum_SMSP_uid184406	NC_019943	chromosome											
===								6	Methanoregula_formicicum_SMSP_uid184406	NC_019943	chromosome											
432331664	putative nucleotidyltransferase	No	0		2264062	2264352	-		Methanoregula_formicicum_SMSP_uid184406	NC_019943	chromosome			arCOG01206	1	(-)	V		Minimal nucleotidyltransferase	1E-24	COG1669	Predicted nucleotidyltransferases
432331665	hypothetical protein Metfor_2297	No	0		2264424	2264852	-		Methanoregula_formicicum_SMSP_uid184406	NC_019943	chromosome			arCOG09441	8		L		HJR family endonuclease			
432331666	hypothetical protein Metfor_2298	No	0		2264849	2265253	-		Methanoregula_formicicum_SMSP_uid184406	NC_019943	chromosome											
432331667	hypothetical protein Metfor_2299	Yes	1		2265837	2266361	+		Methanoregula_formicicum_SMSP_uid184406	NC_019943	chromosome											
432331668	hypothetical protein Metfor_2300	No	0		2266367	2267557	-		Methanoregula_formicicum_SMSP_uid184406	NC_019943	chromosome											
432331669	putative membrane protein/domain protein	No	2		2267704	2268270	+		Methanoregula_formicicum_SMSP_uid184406	NC_019943	chromosome			arCOG03633	2	(-)	S		Predicted membrane protein/domain			
===								18	Methanoregula_formicicum_SMSP_uid184406	NC_019943	chromosome											
432331699	uncharacterized conserved small protein	No	0		2298354	2298581	-		Methanoregula_formicicum_SMSP_uid184406	NC_019943	chromosome			arCOG03885	1	(-)	S		Uncharacterized conserved small protein	0.0000005	COG5559	Uncharacterized conserved small protein
432331700	hypothetical protein Metfor_2332	No	0		2298687	2298917	-		Methanoregula_formicicum_SMSP_uid184406	NC_019943	chromosome											
432331701	hypothetical protein Metfor_2333	No	2		2298914	2299201	-		Methanoregula_formicicum_SMSP_uid184406	NC_019943	chromosome											
432331702	hypothetical protein Metfor_2334	No	0		2299410	2299883	-		Methanoregula_formicicum_SMSP_uid184406	NC_019943	chromosome	clust_5										
432331703	hypothetical protein Metfor_2335	No	1		2299873	2300037	-		Methanoregula_formicicum_SMSP_uid184406	NC_019943	chromosome	clust_62										
432331704	hypothetical protein Metfor_2336	No	0		2300450	2300953	-		Methanoregula_formicicum_SMSP_uid184406	NC_019943	chromosome			arCOG06904	7	(-)	S		Uncharacterized conserved protein			
432331705	hypothetical protein Metfor_2337	No	0		2301753	2302106	-		Methanoregula_formicicum_SMSP_uid184406	NC_019943	chromosome											
432331706	hypothetical protein Metfor_2338	No	0		2302103	2302366	-		Methanoregula_formicicum_SMSP_uid184406	NC_019943	chromosome			arCOG09583	7		S		Uncharacterized conserved protein			
432331707	hypothetical protein Metfor_2339	No	0		2302344	2302679	-		Methanoregula_formicicum_SMSP_uid184406	NC_019943	chromosome											
432331708	hypothetical protein Metfor_2340	Yes	1		2302973	2304022	-		Methanoregula_formicicum_SMSP_uid184406	NC_019943	chromosome	clust_93										
432331709	hypothetical protein Metfor_2341	Yes	1		2304057	2304617	-		Methanoregula_formicicum_SMSP_uid184406	NC_019943	chromosome											
432331710	hypothetical protein Metfor_2342	No	5		2305165	2305659	-		Methanoregula_formicicum_SMSP_uid184406	NC_019943	chromosome											
432331711	transposase	No	0		2306017	2306934	+		Methanoregula_formicicum_SMSP_uid184406	NC_019943	chromosome			arCOG02125	5	(-)	L	Tra5	Transposase	8E-13	pfam00665	Integrase core domain. Integrase mediate ; Winged helix-turn helix. This helix-turn
432331712	hypothetical protein Metfor_2344	No	0		2307469	2307759	-		Methanoregula_formicicum_SMSP_uid184406	NC_019943	chromosome											
432331713	hypothetical protein Metfor_2345	No	0		2307821	2308369	+		Methanoregula_formicicum_SMSP_uid184406	NC_019943	chromosome											
432331714	hypothetical protein Metfor_2346	Yes	1		2308457	2309407	-		Methanoregula_formicicum_SMSP_uid184406	NC_019943	chromosome											
432331715	outer membrane lipoprotein-sorting protein	No	1		2309929	2310738	-		Methanoregula_formicicum_SMSP_uid184406	NC_019943	chromosome			arCOG02470	4	(-)	M	LolA	Outer membrane lipoprotein-sorting protein	4E-10	COG2834	Outer membrane lipoprotein-sorting prote
432331716	hypothetical protein Metfor_2348	No	1		2310788	2311426	-		Methanoregula_formicicum_SMSP_uid184406	NC_019943	chromosome											
===								8	Methanoregula_formicicum_SMSP_uid184406	NC_019943	chromosome											
432331718	hypothetical protein Metfor_2350	Yes	0		2312446	2313468	-		Methanoregula_formicicum_SMSP_uid184406	NC_019943	chromosome											
432331719	hypothetical protein Metfor_2351	Yes	1		2313477	2314619	-		Methanoregula_formicicum_SMSP_uid184406	NC_019943	chromosome			arCOG09593	8		S		Uncharacterized conserved protein			
432331720	hypothetical protein Metfor_2352	Yes	2		2314921	2315571	-		Methanoregula_formicicum_SMSP_uid184406	NC_019943	chromosome			arCOG03906	2	(-)	S		Cell surface protein			
432331721	hypothetical protein Metfor_2353	Yes	1		2315702	2316793	-		Methanoregula_formicicum_SMSP_uid184406	NC_019943	chromosome			arCOG09593	8		S		Uncharacterized conserved protein			
432331722	hypothetical protein Metfor_2354	No	1		2317349	2317561	-		Methanoregula_formicicum_SMSP_uid184406	NC_019943	chromosome											
432331723	Yip1 domain protein	No	5		2318134	2318679	-		Methanoregula_formicicum_SMSP_uid184406	NC_019943	chromosome											
432331724	hypothetical protein Metfor_2356	Yes	1		2318707	2319597	-		Methanoregula_formicicum_SMSP_uid184406	NC_019943	chromosome											
432331725	hypothetical protein Metfor_2357	Yes	1		2319843	2320349	+		Methanoregula_formicicum_SMSP_uid184406	NC_019943	chromosome			arCOG04987	6	(-)	S		Uncharacterized conserved protein			
===								8	Methanoregula_formicicum_SMSP_uid184406	NC_019943	chromosome											
432331753	hypothetical protein Metfor_2387	No	4		2346372	2346893	-		Methanoregula_formicicum_SMSP_uid184406	NC_019943	chromosome											
432331754	hypothetical protein Metfor_2388	No	0		2347102	2347707	+		Methanoregula_formicicum_SMSP_uid184406	NC_019943	chromosome											
432331755	hypothetical protein Metfor_2389	No	0		2347723	2347923	-		Methanoregula_formicicum_SMSP_uid184406	NC_019943	chromosome			arCOG03966	3	(-)	V		CopG family DNA-binding protein			
432331756	hypothetical protein Metfor_2390	No	0		2347947	2348435	+		Methanoregula_formicicum_SMSP_uid184406	NC_019943	chromosome											
432331757	hypothetical protein Metfor_2391	No	0		2348700	2349014	-		Methanoregula_formicicum_SMSP_uid184406	NC_019943	chromosome											
432331758	hypothetical protein Metfor_2392	No	2		2349011	2350462	-		Methanoregula_formicicum_SMSP_uid184406	NC_019943	chromosome											
432331759	dsRNA-specific ribonuclease	No	0		2350790	2351320	+		Methanoregula_formicicum_SMSP_uid184406	NC_019943	chromosome			arCOG03457	1	(-)	K	Rnc	dsRNA-specific ribonuclease	0.000002	COG0571	dsRNA-specific ribonuclease
432331760	hypothetical protein Metfor_2394	No	0		2351481	2351780	+		Methanoregula_formicicum_SMSP_uid184406	NC_019943	chromosome											
===								6	Methanoregula_formicicum_SMSP_uid184406	NC_019943	chromosome											
432331796	hypothetical protein Metfor_2432	No	4		2391366	2391818	-		Methanoregula_formicicum_SMSP_uid184406	NC_019943	chromosome											
432331797	hypothetical protein Metfor_2433	No	0		2391811	2392293	-		Methanoregula_formicicum_SMSP_uid184406	NC_019943	chromosome	clust_5										
432331798	hypothetical protein Metfor_2434	No	0		2392283	2392393	-		Methanoregula_formicicum_SMSP_uid184406	NC_019943	chromosome											
432331799	hypothetical protein Metfor_2435	No	0		2392533	2393036	-		Methanoregula_formicicum_SMSP_uid184406	NC_019943	chromosome			arCOG06904	7	(-)	S		Uncharacterized conserved protein			
432331800	hypothetical protein Metfor_2436	No	0		2393160	2393699	+		Methanoregula_formicicum_SMSP_uid184406	NC_019943	chromosome											
432331801	hypothetical protein Metfor_2437	No	2		2393942	2394247	+		Methanoregula_formicicum_SMSP_uid184406	NC_019943	chromosome											
===								9	Methanoregula_formicicum_SMSP_uid184406	NC_019943	chromosome											
432331840	DNA N-6-adenine-methyltransferase (Dam)	No	0		2429840	2430385	-		Methanoregula_formicicum_SMSP_uid184406	NC_019943	chromosome									0.0000002	pfam05869	DNA N-6-adenine-methyltransferase (Dam).
432331841	hypothetical protein Metfor_2482	No	0		2430382	2433447	-		Methanoregula_formicicum_SMSP_uid184406	NC_019943	chromosome			arCOG07809	9	(-)	Y		Viral primase fused to AAA DnaA like ATPase and Zn finger domain			
432331842	hypothetical protein Metfor_2483	No	0		2433437	2433667	-		Methanoregula_formicicum_SMSP_uid184406	NC_019943	chromosome											
432331843	hypothetical protein Metfor_2484	No	0		2433657	2434001	-		Methanoregula_formicicum_SMSP_uid184406	NC_019943	chromosome											
432331844	hypothetical protein Metfor_2485	No	0		2433998	2434147	-		Methanoregula_formicicum_SMSP_uid184406	NC_019943	chromosome											
432331845	hypothetical protein Metfor_2486	No	0		2434132	2434266	-		Methanoregula_formicicum_SMSP_uid184406	NC_019943	chromosome											
432331846	site-specific recombinase XerD	No	0		2434380	2435603	-		Methanoregula_formicicum_SMSP_uid184406	NC_019943	chromosome			arCOG01242	14	(-)	L	XerC	Integrase	5E-17	pfam00589	Phage integrase family. Members of this
432331847	hypothetical protein Metfor_2489	No	0		2435839	2436312	-		Methanoregula_formicicum_SMSP_uid184406	NC_019943	chromosome											
432331848	hypothetical protein Metfor_2490	No	0		2437180	2437530	+		Methanoregula_formicicum_SMSP_uid184406	NC_019943	chromosome											
===								5	Methanoregula_formicicum_SMSP_uid184406	NC_019943	chromosome											
432332091	hypothetical protein Metfor_2742	No	1		2659139	2659300	+		Methanoregula_formicicum_SMSP_uid184406	NC_019943	chromosome	clust_62										
432332092	hypothetical protein Metfor_2743	No	0		2659293	2659772	+		Methanoregula_formicicum_SMSP_uid184406	NC_019943	chromosome	clust_5										
432332093	hypothetical protein Metfor_2744	No	5		2659816	2660400	+		Methanoregula_formicicum_SMSP_uid184406	NC_019943	chromosome	clust_35										
432332094	Kef-type K+ transport system, membrane com	No	12		2660531	2662528	-		Methanoregula_formicicum_SMSP_uid184406	NC_019943	chromosome			arCOG01955	3	(-)	P	KefB	Kef-type K+ transport system, membrane compo	5E-60	COG0475	Kef-type K+ transport systems, membrane ; TrkA-N domain. This domain is found in a ; TrkA-C domain. This domain is often foun
432332095	hypothetical protein Metfor_2746	No	2		2662620	2662814	-		Methanoregula_formicicum_SMSP_uid184406	NC_019943	chromosome											
===								6	Methanoregula_formicicum_SMSP_uid184406	NC_019943	chromosome											
432332098	hypothetical protein Metfor_2749	No	3		2666281	2666883	+		Methanoregula_formicicum_SMSP_uid184406	NC_019943	chromosome			arCOG06902	2	(-)	S		Uncharacterized conserved protein	7E-26	COG4843	Uncharacterized protein conserved in bac
432332099	hypothetical protein Metfor_2750	No	1		2668015	2668209	+		Methanoregula_formicicum_SMSP_uid184406	NC_019943	chromosome											
432332100	hypothetical protein Metfor_2751	No	0		2668301	2668642	+		Methanoregula_formicicum_SMSP_uid184406	NC_019943	chromosome											
432332101	hypothetical protein Metfor_2752	No	0		2668655	2669029	+		Methanoregula_formicicum_SMSP_uid184406	NC_019943	chromosome											
432332102	hypothetical protein Metfor_2753	No	0		2668995	2669186	+		Methanoregula_formicicum_SMSP_uid184406	NC_019943	chromosome											
432332103	hypothetical protein Metfor_2754	No	3		2669781	2670335	+		Methanoregula_formicicum_SMSP_uid184406	NC_019943	chromosome			arCOG06902	2	(-)	S		Uncharacterized conserved protein	1E-23	COG4843	Uncharacterized protein conserved in bac
===								6	Methanoregula_formicicum_SMSP_uid184406	NC_019943	chromosome											
432332107	putative transcriptional regulator	No	0		2671807	2672178	-		Methanoregula_formicicum_SMSP_uid184406	NC_019943	chromosome			arCOG04377	1	(-)	K		Predicted transcriptional regulator	4E-24	COG4738	Predicted transcriptional regulator
432332108	hypothetical protein Metfor_2761	No	0		2672658	2673011	+		Methanoregula_formicicum_SMSP_uid184406	NC_019943	chromosome											
432332109	hypothetical protein Metfor_2762	No	0		2673008	2673157	+		Methanoregula_formicicum_SMSP_uid184406	NC_019943	chromosome											
432332110	hypothetical protein Metfor_2763	No	0		2673157	2673414	+		Methanoregula_formicicum_SMSP_uid184406	NC_019943	chromosome			arCOG09583	7		S		Uncharacterized conserved protein			
432332111	hypothetical protein Metfor_2764	No	0		2673411	2673878	+		Methanoregula_formicicum_SMSP_uid184406	NC_019943	chromosome			arCOG09441	8		L		HJR family endonuclease			
432332112	ATPase involved in chromosome partitioning	No	0		2674697	2675590	-		Methanoregula_formicicum_SMSP_uid184406	NC_019943	chromosome			arCOG00585	1	(-)	D	Mrp	Mrp family protein, ATPase, contains iron-sulfur c	5E-56	COG0489	ATPases involved in chromosome partition
===								11	Methanosaeta_concilii_GP6_uid66207	NC_015416	chromosome											
330506359	hypothetical protein MCON_0047	No	0		47274	47435	+		Methanosaeta_concilii_GP6_uid66207	NC_015416	chromosome											
330506360	hypothetical protein MCON_0048	No	0		47726	47926	+		Methanosaeta_concilii_GP6_uid66207	NC_015416	chromosome			arCOG02607	2	(-)	O	GrxC	Glutaredoxin			
330506361	hypothetical protein MCON_0049	No	0		48034	48147	+		Methanosaeta_concilii_GP6_uid66207	NC_015416	chromosome											
330506362	hypothetical protein MCON_0050	No	0		48122	48250	+		Methanosaeta_concilii_GP6_uid66207	NC_015416	chromosome											
330506363	hypothetical protein MCON_0051	No	0		48252	48455	+		Methanosaeta_concilii_GP6_uid66207	NC_015416	chromosome											
330506364	aldo/keto reductase	No	0		48759	49667	+		Methanosaeta_concilii_GP6_uid66207	NC_015416	chromosome			arCOG06306	1	(-)	C	Tas	Predicted oxidoreductase (related to aryl-alcohol	0.00000004	COG1453	Predicted oxidoreductases of the aldo/ke
330506365	hypothetical protein MCON_0054	Yes	0		49792	50838	+		Methanosaeta_concilii_GP6_uid66207	NC_015416	chromosome			arCOG10863	1		S		Uncharacterized conserved protein			
330506366	hypothetical protein MCON_0056	No	0		51461	51664	+		Methanosaeta_concilii_GP6_uid66207	NC_015416	chromosome											
330506367	hypothetical protein MCON_0057	No	0		51670	52173	+		Methanosaeta_concilii_GP6_uid66207	NC_015416	chromosome											
330506368	nitrogenase MoFe cofactor biosynthesis doma	No	0		53709	54551	-		Methanosaeta_concilii_GP6_uid66207	NC_015416	chromosome			arCOG00598	2	(-)	C	NifD	Nitrogenase molybdenum-iron protein, alpha or b	4E-36	COG2710	Nitrogenase molybdenum-iron protein, alp
330506369	hypothetical protein MCON_0061	No	0		54665	54847	-		Methanosaeta_concilii_GP6_uid66207	NC_015416	chromosome											
===								5	Methanosaeta_concilii_GP6_uid66207	NC_015416	chromosome											
330506401	hypothetical protein MCON_0100	No	0		97399	97608	-		Methanosaeta_concilii_GP6_uid66207	NC_015416	chromosome			arCOG09589	1		S		Uncharacterized conserved protein			
330506402	hypothetical protein MCON_0101	No	0		97598	98116	-		Methanosaeta_concilii_GP6_uid66207	NC_015416	chromosome			arCOG09590	1	(-)	V		PIN domain containing protein			
330506403	hypothetical protein MCON_0102	No	0		98170	98322	-		Methanosaeta_concilii_GP6_uid66207	NC_015416	chromosome											
330506404	hypothetical protein MCON_0103	No	0		98555	98752	+		Methanosaeta_concilii_GP6_uid66207	NC_015416	chromosome											
330506405	hypothetical protein MCON_0104	No	0		98792	99082	+		Methanosaeta_concilii_GP6_uid66207	NC_015416	chromosome									0.0000001	pfam09954	Uncharacterized protein conserved in bac
===								6	Methanosaeta_concilii_GP6_uid66207	NC_015416	chromosome											
330506419	fasciclin domain-containing protein	Yes	1		118224	119165	+		Methanosaeta_concilii_GP6_uid66207	NC_015416	chromosome		arCOG03335,a	arCOG03335	1	(-)	M		Secreted and surface protein containing fasciclin-	2E-18	pfam02469	Fasciclin domain. This extracellular dom ; Fasciclin domain. This extracellular dom
330506420	hypothetical protein MCON_0120	No	1		119268	120008	-		Methanosaeta_concilii_GP6_uid66207	NC_015416	chromosome			arCOG10866	8		S		Uncharacterized conserved protein			
330506421	hypothetical protein MCON_0122	No	0		120072	120479	-		Methanosaeta_concilii_GP6_uid66207	NC_015416	chromosome											
330506422	lipoprotein	Yes	1		120729	121481	+		Methanosaeta_concilii_GP6_uid66207	NC_015416	chromosome											
330506423	hypothetical protein MCON_0124	No	1		121646	121825	+		Methanosaeta_concilii_GP6_uid66207	NC_015416	chromosome											
330506424	radical SAM domain-containing protein	No	0		121983	122540	+		Methanosaeta_concilii_GP6_uid66207	NC_015416	chromosome			arCOG02173	4	(-)	O	NrdG	Organic radical activating enzyme	2E-14	COG0602	Organic radical activating enzymes
===								5	Methanosaeta_concilii_GP6_uid66207	NC_015416	chromosome											
330506503	hypothetical protein MCON_0216	No	0		200694	200840	-		Methanosaeta_concilii_GP6_uid66207	NC_015416	chromosome											
330506504	hypothetical protein MCON_0217	No	1		200851	200997	-		Methanosaeta_concilii_GP6_uid66207	NC_015416	chromosome											
330506505	hypothetical protein MCON_0218	No	1		200994	201218	-		Methanosaeta_concilii_GP6_uid66207	NC_015416	chromosome											
330506506	hypothetical protein MCON_0219	No	0		201604	202074	+		Methanosaeta_concilii_GP6_uid66207	NC_015416	chromosome			arCOG07802	1	(-)	R		Predicted flavin-nucleotide-binding protein			
330506507	hypothetical protein MCON_0221	Yes	0		202381	202692	+		Methanosaeta_concilii_GP6_uid66207	NC_015416	chromosome											
===								6	Methanosaeta_concilii_GP6_uid66207	NC_015416	chromosome											
330506590	transposase, IS605 OrfB family	No	0		281616	282791	-		Methanosaeta_concilii_GP6_uid66207	NC_015416	chromosome			arCOG00679	4	(-)	L		Transposase	4E-22	COG0675	Transposase and inactivated derivatives
330506591	hypothetical protein MCON_0331	No	0		283003	283347	-		Methanosaeta_concilii_GP6_uid66207	NC_015416	chromosome											
330506592	hypothetical protein MCON_0332	No	0		283364	283834	-		Methanosaeta_concilii_GP6_uid66207	NC_015416	chromosome											
330506593	hypothetical protein MCON_0333	No	0		283831	284052	-		Methanosaeta_concilii_GP6_uid66207	NC_015416	chromosome											
330506594	hypothetical protein MCON_0334	No	0		284144	284437	+		Methanosaeta_concilii_GP6_uid66207	NC_015416	chromosome											
330506595	hypothetical protein MCON_0335	No	0		284486	284602	+		Methanosaeta_concilii_GP6_uid66207	NC_015416	chromosome			arCOG03086	2	(-)	R		Predicted RNA binding protein (dsRBD-like fold), HicA family			
===								5	Methanosaeta_concilii_GP6_uid66207	NC_015416	chromosome											
330506844	hypothetical protein MCON_0626	No	6		558659	559288	-		Methanosaeta_concilii_GP6_uid66207	NC_015416	chromosome											
330506845	hypothetical protein MCON_0627	No	0		559357	559605	-		Methanosaeta_concilii_GP6_uid66207	NC_015416	chromosome			arCOG10339	4		S		Uncharacterized conserved protein			
330506846	transposase	No	0		559626	560936	+		Methanosaeta_concilii_GP6_uid66207	NC_015416	chromosome			arCOG04931	3	(-)	S		Uncharacterized conserved protein			
330506847	transposase IS3 family protein integrase	No	0		561103	561858	-		Methanosaeta_concilii_GP6_uid66207	NC_015416	chromosome			arCOG10339	4		S		Uncharacterized conserved protein	2E-18	COG2801	Transposase and inactivated derivatives
330506848	IS3/IS66 family element, Orf1 protein	No	0		561855	562184	-		Methanosaeta_concilii_GP6_uid66207	NC_015416	chromosome	clust_318								0.0000003	COG2963	Transposase and inactivated derivatives
===								7	Methanosaeta_concilii_GP6_uid66207	NC_015416	chromosome											
330506970	hypothetical protein MCON_0781	No	0		693051	693620	-		Methanosaeta_concilii_GP6_uid66207	NC_015416	chromosome			arCOG01866	1	(-)	K	HipB	Predicted transcriptional regulator, xre family	2E-11	pfam07883	Cupin domain. This family represents the ; Helix-turn-helix domain. Members of this
330506971	hypothetical protein MCON_0784	No	0		694070	694435	-		Methanosaeta_concilii_GP6_uid66207	NC_015416	chromosome											
330506972	hypothetical protein MCON_0785	No	1		694669	696621	+		Methanosaeta_concilii_GP6_uid66207	NC_015416	chromosome									4E-20	pfam04747	Protein of unknown function, DUF612. Thi
330506973	hypothetical protein MCON_0786	No	0		696667	697140	-		Methanosaeta_concilii_GP6_uid66207	NC_015416	chromosome											
330506974	hypothetical protein MCON_0787	No	0		697257	698150	-		Methanosaeta_concilii_GP6_uid66207	NC_015416	chromosome											
330506975	hypothetical protein MCON_0788	No	1		698484	698720	-		Methanosaeta_concilii_GP6_uid66207	NC_015416	chromosome											
330506976	hypothetical protein MCON_0793	No	0		700336	700890	+		Methanosaeta_concilii_GP6_uid66207	NC_015416	chromosome			arCOG00724	7	(-)	K		Predicted transcriptional regulator, PadR family			
===								5	Methanosaeta_concilii_GP6_uid66207	NC_015416	chromosome											
330507144	hypothetical protein MCON_1000	No	0		862156	862380	+		Methanosaeta_concilii_GP6_uid66207	NC_015416	chromosome											
330507145	RelE/StbE family addiction module toxin	No	0		862457	862753	-		Methanosaeta_concilii_GP6_uid66207	NC_015416	chromosome			arCOG02414	1	(-)	V	RelE	Cytotoxic translational repressor of toxin-antitoxin stability system			
330507146	hypothetical protein MCON_1002	No	0		862759	862935	-		Methanosaeta_concilii_GP6_uid66207	NC_015416	chromosome											
330507147	hypothetical protein MCON_1003	No	0		863081	863233	+		Methanosaeta_concilii_GP6_uid66207	NC_015416	chromosome	clust_498										
330507148	hypothetical protein MCON_1004	No	0		863348	863506	+		Methanosaeta_concilii_GP6_uid66207	NC_015416	chromosome											
===								7	Methanosaeta_concilii_GP6_uid66207	NC_015416	chromosome											
330507156	lipoprotein	Yes	0		868906	869535	+		Methanosaeta_concilii_GP6_uid66207	NC_015416	chromosome			arCOG09394	1	(-)	S		Uncharacterized conserved protein			
330507157	hypothetical protein MCON_1015	No	0		869701	869853	+		Methanosaeta_concilii_GP6_uid66207	NC_015416	chromosome	clust_498										
330507158	hypothetical protein MCON_1016	No	0		869834	870016	+		Methanosaeta_concilii_GP6_uid66207	NC_015416	chromosome											
330507159	hypothetical protein MCON_1017	No	0		870092	870307	-		Methanosaeta_concilii_GP6_uid66207	NC_015416	chromosome											
330507160	hypothetical protein MCON_1018	Yes	0		870361	871140	-		Methanosaeta_concilii_GP6_uid66207	NC_015416	chromosome			arCOG10866	8		S		Uncharacterized conserved protein			
330507161	hypothetical protein MCON_1020	No	0		871428	873002	-		Methanosaeta_concilii_GP6_uid66207	NC_015416	chromosome			arCOG03473	2	(-)	L		Transposase	3E-21	COG5421	Transposase
330507162	membrane protein	No	10		873438	874643	+		Methanosaeta_concilii_GP6_uid66207	NC_015416	chromosome											
===								9	Methanosaeta_concilii_GP6_uid66207	NC_015416	chromosome											
330507251	hypothetical protein MCON_1134	Yes	1		974017	974727	-		Methanosaeta_concilii_GP6_uid66207	NC_015416	chromosome			arCOG10866	8		S		Uncharacterized conserved protein			
330507252	hypothetical protein MCON_1135	Yes	0		974996	976177	-		Methanosaeta_concilii_GP6_uid66207	NC_015416	chromosome			arCOG02081	3	(-)	S		Predicted membrane protein			
330507253	hypothetical protein MCON_1136	Yes	1		976225	977001	-		Methanosaeta_concilii_GP6_uid66207	NC_015416	chromosome											
330507254	hypothetical protein MCON_1137	Yes	1		977313	978095	-		Methanosaeta_concilii_GP6_uid66207	NC_015416	chromosome			arCOG10866	8		S		Uncharacterized conserved protein			
330507255	hypothetical protein MCON_1138	No	0		978157	978357	-		Methanosaeta_concilii_GP6_uid66207	NC_015416	chromosome											
330507256	hypothetical protein MCON_1139	Yes	1		978596	979318	-		Methanosaeta_concilii_GP6_uid66207	NC_015416	chromosome			arCOG10866	8		S		Uncharacterized conserved protein			
330507257	hypothetical protein MCON_1140	No	0		979380	979685	-		Methanosaeta_concilii_GP6_uid66207	NC_015416	chromosome											
330507258	hypothetical protein MCON_1141	Yes	1		979824	980546	-		Methanosaeta_concilii_GP6_uid66207	NC_015416	chromosome			arCOG10866	8		S		Uncharacterized conserved protein			
330507259	hypothetical protein MCON_1145	No	0		981351	982349	+		Methanosaeta_concilii_GP6_uid66207	NC_015416	chromosome			arCOG02710	1	(-)	K		Repressor of nif and glnA expression	3E-91	COG1693	Repressor of nif and glnA expression
===								7	Methanosaeta_concilii_GP6_uid66207	NC_015416	chromosome											
330507833	transposase	No	0		1588072	1588989	-		Methanosaeta_concilii_GP6_uid66207	NC_015416	chromosome			arCOG02125	5	(-)	L	Tra5	Transposase	1E-17	COG2801	Transposase and inactivated derivatives
330507834	hypothetical protein MCON_1853	No	0		1589837	1591093	+		Methanosaeta_concilii_GP6_uid66207	NC_015416	chromosome											
330507835	hypothetical protein MCON_1854	No	0		1591194	1592669	+		Methanosaeta_concilii_GP6_uid66207	NC_015416	chromosome											
330507836	hypothetical protein MCON_1855	No	0		1592700	1592891	-		Methanosaeta_concilii_GP6_uid66207	NC_015416	chromosome			arCOG10339	4		S		Uncharacterized conserved protein			
330507837	hypothetical protein MCON_1856	Yes	1		1592891	1593799	+		Methanosaeta_concilii_GP6_uid66207	NC_015416	chromosome											
330507838	hypothetical protein MCON_1858	No	0		1594067	1594231	-		Methanosaeta_concilii_GP6_uid66207	NC_015416	chromosome			arCOG04449	1	(-)	J	TruA	Pseudouridylate synthase			
330507839	hypothetical protein MCON_1859	No	0		1594419	1594964	+		Methanosaeta_concilii_GP6_uid66207	NC_015416	chromosome											
===								17	Methanosaeta_concilii_GP6_uid66207	NC_015416	chromosome											
330507934	site-specific integrase/recombinase	No	0		1686148	1686987	+		Methanosaeta_concilii_GP6_uid66207	NC_015416	chromosome			arCOG01241	31	(-)	L	XerC	Integrase	1E-23	COG4974	Site-specific recombinase XerD
330507935	hypothetical protein MCON_1979	No	0		1687216	1687557	+		Methanosaeta_concilii_GP6_uid66207	NC_015416	chromosome											
330507936	hypothetical protein MCON_1981	No	0		1687764	1688069	+		Methanosaeta_concilii_GP6_uid66207	NC_015416	chromosome											
330507937	hypothetical protein MCON_1983	No	0		1689359	1690246	+		Methanosaeta_concilii_GP6_uid66207	NC_015416	chromosome											
330507938	hypothetical protein MCON_1985	No	0		1690395	1690646	+		Methanosaeta_concilii_GP6_uid66207	NC_015416	chromosome											
330507939	hypothetical protein MCON_1987	No	0		1690880	1691065	+		Methanosaeta_concilii_GP6_uid66207	NC_015416	chromosome											
330507940	hypothetical protein MCON_1988	No	0		1691062	1691862	+		Methanosaeta_concilii_GP6_uid66207	NC_015416	chromosome											
330507941	hypothetical protein MCON_1990	No	0		1692414	1692590	+		Methanosaeta_concilii_GP6_uid66207	NC_015416	chromosome											
330507942	hypothetical protein MCON_1991	No	0		1692657	1692812	+		Methanosaeta_concilii_GP6_uid66207	NC_015416	chromosome											
330507943	hypothetical protein MCON_1992	No	0		1692891	1693064	+		Methanosaeta_concilii_GP6_uid66207	NC_015416	chromosome											
330507944	hypothetical protein MCON_1993	No	0		1693111	1693335	+		Methanosaeta_concilii_GP6_uid66207	NC_015416	chromosome											
330507945	phage/plasmid primase, P4 family	No	0		1693618	1694754	+		Methanosaeta_concilii_GP6_uid66207	NC_015416	chromosome			arCOG06914	10	(-)	R		Phage or plasmid associated DNA primase	1E-25	COG3378	Phage associated DNA primase
330507946	hypothetical protein MCON_1996	No	0		1695175	1695531	+		Methanosaeta_concilii_GP6_uid66207	NC_015416	chromosome											
330507947	phage terminase, large subunit, PBSx family	No	0		1695531	1696799	+		Methanosaeta_concilii_GP6_uid66207	NC_015416	chromosome			arCOG09550	17		Y	TerL	Phage terminase large subunit			
330507948	portal protein	No	0		1696849	1698405	+		Methanosaeta_concilii_GP6_uid66207	NC_015416	chromosome											
330507949	phage putative head morphogenesis protein d	No	0		1698600	1699100	+		Methanosaeta_concilii_GP6_uid66207	NC_015416	chromosome			arCOG09510	5	(-)	T		NAD+--asparagine ADP-ribosyltransferase	0.000002	pfam04233	Phage Mu protein F like protein. Members
330507950	lipoprotein	Yes	0		1699351	1700586	+		Methanosaeta_concilii_GP6_uid66207	NC_015416	chromosome											
===								6	Methanosaeta_concilii_GP6_uid66207	NC_015416	chromosome											
330508203	TPR-repeat-containing protein	No	0		1988932	1991100	+		Methanosaeta_concilii_GP6_uid66207	NC_015416	chromosome			arCOG03038	9	(-)	R	NrfG	TPR repeats containing protein	7E-24	COG0457	FOG: TPR repeat
330508204	hypothetical protein MCON_2341	No	2		1991167	1991382	+		Methanosaeta_concilii_GP6_uid66207	NC_015416	chromosome											
330508205	hypothetical protein MCON_2342	No	0		1991496	1991834	+		Methanosaeta_concilii_GP6_uid66207	NC_015416	chromosome											
330508206	hypothetical protein MCON_2343	No	0		1992109	1993029	-		Methanosaeta_concilii_GP6_uid66207	NC_015416	chromosome											
330508207	hypothetical protein MCON_2346	Yes	1		1993657	1994256	-		Methanosaeta_concilii_GP6_uid66207	NC_015416	chromosome											
330508208	hypothetical protein MCON_2347	No	0		1994368	1994823	+		Methanosaeta_concilii_GP6_uid66207	NC_015416	chromosome			arCOG00463	1	(-)	L		Uri superfamily endonuclease	6E-19	pfam01986	Domain of unknown function DUF123. This
===								7	Methanosaeta_concilii_GP6_uid66207	NC_015416	chromosome											
330508217	hypothetical protein MCON_2357	No	0		2008259	200	+		Methanosaeta_concilii_GP6_uid66207	NC_015416	chromosome			arCOG00041	1	(-)	R		Predicted phosphoribosyltransferase	3E-51	COG1926	Predicted phosphoribosyltransferases
330508218	hypothetical protein MCON_2358	No	0		2008949	2011420	-		Methanosaeta_concilii_GP6_uid66207	NC_015416	chromosome			arCOG05250	6		S		Uncharacterized conserved protein			
330508219	hypothetical protein MCON_2359	No	3		2011480	2011890	-		Methanosaeta_concilii_GP6_uid66207	NC_015416	chromosome											
330508220	hypothetical protein MCON_2360	No	2		2011880	2012170	-		Methanosaeta_concilii_GP6_uid66207	NC_015416	chromosome											
330508221	hypothetical protein MCON_2361	No	0		2012416	2012946	+		Methanosaeta_concilii_GP6_uid66207	NC_015416	chromosome											
330508222	hypothetical protein MCON_2362	No	2		2012943	2013839	+		Methanosaeta_concilii_GP6_uid66207	NC_015416	chromosome											
330508223	SPFH domain/band 7 family protein	No	1		2013920	2014801	+		Methanosaeta_concilii_GP6_uid66207	NC_015416	chromosome			arCOG01915	1	(-)	O	HflC	Membrane protease subunit, stomatin/prohibitin h	4E-20	pfam01145	SPFH domain / Band 7 family. This family
===								5	Methanosaeta_concilii_GP6_uid66207	NC_015416	chromosome											
330508306	hypothetical protein MCON_2466	No	0		2119427	2119861	-		Methanosaeta_concilii_GP6_uid66207	NC_015416	chromosome											
330508307	hypothetical protein MCON_2467	No	0		2119929	2120300	-		Methanosaeta_concilii_GP6_uid66207	NC_015416	chromosome											
330508308	transposase, IS4	No	0		2120585	2121979	+		Methanosaeta_concilii_GP6_uid66207	NC_015416	chromosome			arCOG03473	2	(-)	L		Transposase	0.00000002	COG5421	Transposase
330508309	hypothetical protein MCON_2471	No	0		2122227	2122541	+		Methanosaeta_concilii_GP6_uid66207	NC_015416	chromosome											
330508310	hypothetical protein MCON_2474	Yes	1		2124572	2125156	+		Methanosaeta_concilii_GP6_uid66207	NC_015416	chromosome											
===								5	Methanosaeta_concilii_GP6_uid66207	NC_015416	chromosome											
330508761	hypothetical protein MCON_3048	Yes	1		2577025	2577597	-		Methanosaeta_concilii_GP6_uid66207	NC_015416	chromosome											
330508762	hypothetical protein MCON_3050	No	0		2578037	2578360	+		Methanosaeta_concilii_GP6_uid66207	NC_015416	chromosome									0.000001	pfam10771	Protein of unknown function (DUF2582). T
330508763	hypothetical protein MCON_3052	No	5		2578663	2579256	+		Methanosaeta_concilii_GP6_uid66207	NC_015416	chromosome			arCOG00009	2	(-)	E	PotE	Amino acid transporter	7E-20	COG0531	Amino acid transporters
330508764	hypothetical protein MCON_3053	No	0		2579267	2579764	-		Methanosaeta_concilii_GP6_uid66207	NC_015416	chromosome			arCOG10608	1		S		Uncharacterized conserved protein			
330508765	hypothetical protein MCON_3054	No	0		2579795	2580925	-		Methanosaeta_concilii_GP6_uid66207	NC_015416	chromosome									0.000001	COG4995	Uncharacterized protein conserved in bac
===								14	Methanosaeta_concilii_GP6_uid66207	NC_015416	chromosome											
330509022	hypothetical protein MCON_3384	No	0		2850475	2850942	-		Methanosaeta_concilii_GP6_uid66207	NC_015416	chromosome			arCOG02138	2	(-)	L		Transposase	0.00009	COG3677	Transposase and inactivated derivatives
330509023	hypothetical protein MCON_3386	No	0		2851053	2851790	+		Methanosaeta_concilii_GP6_uid66207	NC_015416	chromosome											
330509024	hypothetical protein MCON_3387	No	0		2851888	2852679	+		Methanosaeta_concilii_GP6_uid66207	NC_015416	chromosome											
330509025	IS3/IS66 family element, Orf1 protein	No	0		2852942	2853271	+		Methanosaeta_concilii_GP6_uid66207	NC_015416	chromosome	clust_318								0.0000003	COG2963	Transposase and inactivated derivatives
330509026	transposase IS3 family protein integrase	No	0		2853268	2854146	+		Methanosaeta_concilii_GP6_uid66207	NC_015416	chromosome			arCOG10339	4		S		Uncharacterized conserved protein	1E-22	COG2801	Transposase and inactivated derivatives
330509027	hypothetical protein MCON_3393	No	0		2855305	2855520	+		Methanosaeta_concilii_GP6_uid66207	NC_015416	chromosome											
330509028	transposase, Mutator family	No	0		2855670	2856794	+		Methanosaeta_concilii_GP6_uid66207	NC_015416	chromosome			arCOG04918	1	(-)	L		Transposase	2E-85	pfam00872	Transposase, Mutator family.
330509029	hypothetical protein MCON_3395	No	0		2856932	2857129	+		Methanosaeta_concilii_GP6_uid66207	NC_015416	chromosome											
330509030	hypothetical protein MCON_3396	No	0		2857236	2857517	+		Methanosaeta_concilii_GP6_uid66207	NC_015416	chromosome											
330509031	hypothetical protein MCON_3397	No	1		2857760	2858032	+		Methanosaeta_concilii_GP6_uid66207	NC_015416	chromosome											
330509032	hypothetical protein MCON_3398	No	0		2858037	2858318	+		Methanosaeta_concilii_GP6_uid66207	NC_015416	chromosome											
330509033	hypothetical protein MCON_3405	No	0		2859549	2859974	+		Methanosaeta_concilii_GP6_uid66207	NC_015416	chromosome											
330509034	hypothetical protein MCON_3406	No	2		2860074	2860307	+		Methanosaeta_concilii_GP6_uid66207	NC_015416	chromosome											
330509035	hypothetical protein MCON_3407	No	0		2861029	2861202	+		Methanosaeta_concilii_GP6_uid66207	NC_015416	chromosome			arCOG02412	1	(-)	R		Predicted nuclease of the RNAse H fold, HicB fa	0.000002	pfam03681	Uncharacterized protein family (UPF0150)
===								5	Methanosaeta_concilii_GP6_uid66207	NC_015416	chromosome											
330509131	hypothetical protein MCON_3526	No	0		2982991	2983560	-		Methanosaeta_concilii_GP6_uid66207	NC_015416	chromosome											
330509132	IS3/IS66 family element, Orf1 protein	No	0		2983960	2984181	+		Methanosaeta_concilii_GP6_uid66207	NC_015416	chromosome											
330509133	hypothetical protein MCON_3528	No	0		2984537	2984788	+		Methanosaeta_concilii_GP6_uid66207	NC_015416	chromosome											
330509134	TPR-repeat-containing protein	No	1		2985091	2986035	+		Methanosaeta_concilii_GP6_uid66207	NC_015416	chromosome			arCOG03038	9	(-)	R	NrfG	TPR repeats containing protein	0.000000002	COG0457	FOG: TPR repeat
330509135	hypothetical protein MCON_3530	No	0		2986216	2987118	-		Methanosaeta_concilii_GP6_uid66207	NC_015416	chromosome											
===								21	Methanosaeta_concilii_GP6_uid66207	NC_015430	plasmid pGP6											
330506029	hypothetical protein MCON_3548	No	0	plasmid	47	367	+		Methanosaeta_concilii_GP6_uid66207	NC_015430	plasmid pGP6											
330506030	hypothetical protein MCON_3549	No	0	plasmid	552	1205	+		Methanosaeta_concilii_GP6_uid66207	NC_015430	plasmid pGP6			arCOG10891	4		S		Uncharacterized conserved protein			
330506031	hypothetical protein MCON_3550	No	0	plasmid	1535	2206	-		Methanosaeta_concilii_GP6_uid66207	NC_015430	plasmid pGP6	clust_155										
330506032	hypothetical protein MCON_3551	No	1	plasmid	2404	2730	-		Methanosaeta_concilii_GP6_uid66207	NC_015430	plasmid pGP6											
330506033	hypothetical protein MCON_3552	No	1	plasmid	2765	3088	+		Methanosaeta_concilii_GP6_uid66207	NC_015430	plasmid pGP6											
330506034	hypothetical protein MCON_3553	No	0	plasmid	3689	5662	-		Methanosaeta_concilii_GP6_uid66207	NC_015430	plasmid pGP6			arCOG10891	4		S		Uncharacterized conserved protein			
330506035	hypothetical protein MCON_3554	No	0	plasmid	5924	6574	-		Methanosaeta_concilii_GP6_uid66207	NC_015430	plasmid pGP6									0.000001	pfam11523	Protein of unknown function (DUF3223). T
330506036	hypothetical protein MCON_3555	No	0	plasmid	7192	7470	-		Methanosaeta_concilii_GP6_uid66207	NC_015430	plasmid pGP6											
330506037	hypothetical protein MCON_3556	No	0	plasmid	7642	9879	-		Methanosaeta_concilii_GP6_uid66207	NC_015430	plasmid pGP6											
330506038	hypothetical protein MCON_3557	No	0	plasmid	10089	10586	+		Methanosaeta_concilii_GP6_uid66207	NC_015430	plasmid pGP6			arCOG03730	1	(-)	S		Uncharacterized conserved protein	0.00000001	COG1196	Chromosome segregation ATPases
330506039	hypothetical protein MCON_3558	No	0	plasmid	10812	11318	-		Methanosaeta_concilii_GP6_uid66207	NC_015430	plasmid pGP6											
330506040	hypothetical protein MCON_3559	No	0	plasmid	11503	11814	-		Methanosaeta_concilii_GP6_uid66207	NC_015430	plasmid pGP6			arCOG03182	3	(-)	K	MarR	Transcriptional regulator, MarR family			
330506041	hypothetical protein MCON_3560	No	0	plasmid	12352	12597	-		Methanosaeta_concilii_GP6_uid66207	NC_015430	plasmid pGP6											
330506042	hypothetical protein MCON_3561	No	0	plasmid	12584	12979	-		Methanosaeta_concilii_GP6_uid66207	NC_015430	plasmid pGP6			arCOG11289	1		S		Uncharacterized conserved protein			
330506043	hypothetical protein MCON_3562	No	0	plasmid	13059	13688	+		Methanosaeta_concilii_GP6_uid66207	NC_015430	plasmid pGP6											
330506044	hypothetical protein MCON_3563	No	0	plasmid	13685	14092	+		Methanosaeta_concilii_GP6_uid66207	NC_015430	plasmid pGP6											
330506045	hypothetical protein MCON_3564	No	0	plasmid	14136	14807	-		Methanosaeta_concilii_GP6_uid66207	NC_015430	plasmid pGP6	clust_155										
330506046	hypothetical protein MCON_3565	No	1	plasmid	15420	15692	+		Methanosaeta_concilii_GP6_uid66207	NC_015430	plasmid pGP6											
330506047	hypothetical protein MCON_3566	No	0	plasmid	16005	16634	+		Methanosaeta_concilii_GP6_uid66207	NC_015430	plasmid pGP6			arCOG10891	4		S		Uncharacterized conserved protein			
330506048	hypothetical protein MCON_3567	No	0	plasmid	16615	17061	+		Methanosaeta_concilii_GP6_uid66207	NC_015430	plasmid pGP6											
330506049	hypothetical protein MCON_3568	No	0	plasmid	17170	17652	+		Methanosaeta_concilii_GP6_uid66207	NC_015430	plasmid pGP6			arCOG00810	1	(-)	J	MetG	Methionyl-tRNA synthetase	5E-36	COG0143	Methionyl-tRNA synthetase
===								16	Methanosaeta_harundinacea_6Ac_uid81199	NC_016637	plasmid pH6Ac											
386003072	hypothetical protein Mhar_2397	No	0	plasmid	240	353	+		Methanosaeta_harundinacea_6Ac_uid81199	NC_016637	plasmid pH6Ac											
386003073	hypothetical protein Mhar_2398	No	0	plasmid	694	834	-		Methanosaeta_harundinacea_6Ac_uid81199	NC_016637	plasmid pH6Ac											
386003074	hypothetical protein Mhar_2399	No	0	plasmid	1197	1367	+		Methanosaeta_harundinacea_6Ac_uid81199	NC_016637	plasmid pH6Ac											
386003075	hypothetical protein Mhar_2400	No	0	plasmid	1460	1606	+		Methanosaeta_harundinacea_6Ac_uid81199	NC_016637	plasmid pH6Ac											
386003076	hypothetical protein Mhar_2401	No	0	plasmid	1608	4982	+		Methanosaeta_harundinacea_6Ac_uid81199	NC_016637	plasmid pH6Ac											
386003077	hypothetical protein Mhar_2402	No	0	plasmid	5393	5557	+		Methanosaeta_harundinacea_6Ac_uid81199	NC_016637	plasmid pH6Ac											
386003078	hypothetical protein Mhar_2403	No	0	plasmid	5851	6024	+		Methanosaeta_harundinacea_6Ac_uid81199	NC_016637	plasmid pH6Ac											
386003079	hypothetical protein Mhar_2404	No	0	plasmid	6173	6319	+		Methanosaeta_harundinacea_6Ac_uid81199	NC_016637	plasmid pH6Ac											
386003080	hypothetical protein Mhar_2405	No	0	plasmid	6303	6494	+		Methanosaeta_harundinacea_6Ac_uid81199	NC_016637	plasmid pH6Ac											
386003081	hypothetical protein Mhar_2406	No	0	plasmid	6562	6681	+		Methanosaeta_harundinacea_6Ac_uid81199	NC_016637	plasmid pH6Ac											
386003082	hypothetical protein Mhar_2407	No	0	plasmid	6866	7378	+		Methanosaeta_harundinacea_6Ac_uid81199	NC_016637	plasmid pH6Ac			arCOG01008	7	(-)	V	NikR	Transcriptional regulator, CopG/Arc/MetJ family (I	0.00003	COG0864	Predicted transcriptional regulators con
386003083	hypothetical protein Mhar_2408	Yes	0	plasmid	7410	7829	-		Methanosaeta_harundinacea_6Ac_uid81199	NC_016637	plasmid pH6Ac											
386003084	hypothetical protein Mhar_2409	No	0	plasmid	7938	8189	+		Methanosaeta_harundinacea_6Ac_uid81199	NC_016637	plasmid pH6Ac											
386003085	hypothetical protein Mhar_2410	No	0	plasmid	8715	8876	-		Methanosaeta_harundinacea_6Ac_uid81199	NC_016637	plasmid pH6Ac											
386003086	Extracellular nuclease	No	0	plasmid	9096	9725	+		Methanosaeta_harundinacea_6Ac_uid81199	NC_016637	plasmid pH6Ac		arCOG03192,a	arCOG03192	6	(-)	L		Micrococcal nuclease (thermonuclease)			
386003087	hypothetical protein Mhar_2412	No	0	plasmid	10161	10772	-		Methanosaeta_harundinacea_6Ac_uid81199	NC_016637	plasmid pH6Ac											
===								5	Methanosaeta_harundinacea_6Ac_uid81199	NC_017527	chromosome											
386000756	hypothetical protein Mhar_0039	Yes	0		48776	49090	+		Methanosaeta_harundinacea_6Ac_uid81199	NC_017527	chromosome											
386000757	hypothetical protein Mhar_0040	No	0		49199	49687	-		Methanosaeta_harundinacea_6Ac_uid81199	NC_017527	chromosome											
386000758	hypothetical protein Mhar_0041	No	0		49802	50095	-		Methanosaeta_harundinacea_6Ac_uid81199	NC_017527	chromosome											
386000759	hypothetical protein Mhar_0042	No	0		50115	52136	-		Methanosaeta_harundinacea_6Ac_uid81199	NC_017527	chromosome		arCOG03045,a	arCOG03045	2	(-)	R	NrfG	TPR repeats containing protein	8E-13	COG0457	FOG: TPR repeat
386000760	Signal recognition particle GTPase	No	0		52118	53380	-		Methanosaeta_harundinacea_6Ac_uid81199	NC_017527	chromosome											
===								5	Methanosaeta_harundinacea_6Ac_uid81199	NC_017527	chromosome											
386002083	hypothetical protein Mhar_1395	No	0		1501958	1502419	+		Methanosaeta_harundinacea_6Ac_uid81199	NC_017527	chromosome											
386002084	hypothetical protein Mhar_1396	No	1		1502492	1504849	-		Methanosaeta_harundinacea_6Ac_uid81199	NC_017527	chromosome		arCOG07611,a	arCOG07611	1	(-)	M		Cell surface protein, contains DUF11 domains			
386002085	hypothetical protein Mhar_1397	Yes	1		1504973	1505524	-		Methanosaeta_harundinacea_6Ac_uid81199	NC_017527	chromosome											
386002086	hypothetical protein Mhar_1398	Yes	0		1505545	1506237	-		Methanosaeta_harundinacea_6Ac_uid81199	NC_017527	chromosome	clust_146										
386002087	hypothetical protein Mhar_1399	Yes	0		1506608	1507303	-		Methanosaeta_harundinacea_6Ac_uid81199	NC_017527	chromosome	clust_146										
===								5	Methanosaeta_harundinacea_6Ac_uid81199	NC_017527	chromosome											
386002134	hypothetical protein Mhar_1446	No	0		1555743	1556051	-		Methanosaeta_harundinacea_6Ac_uid81199	NC_017527	chromosome											
386002135	hypothetical protein Mhar_1447	No	3		1556053	1556406	-		Methanosaeta_harundinacea_6Ac_uid81199	NC_017527	chromosome											
386002136	hypothetical protein Mhar_1448	Yes	4		1556394	1557332	-		Methanosaeta_harundinacea_6Ac_uid81199	NC_017527	chromosome			arCOG02400	1	(-)	O	CcdA	Cytochrome c biogenesis protein			
386002137	hypothetical protein Mhar_1449	No	0		1558102	1558521	+		Methanosaeta_harundinacea_6Ac_uid81199	NC_017527	chromosome											
386002138	hypothetical protein Mhar_1450	No	0		1558905	1559186	-		Methanosaeta_harundinacea_6Ac_uid81199	NC_017527	chromosome											
===								5	Methanosaeta_harundinacea_6Ac_uid81199	NC_017527	chromosome											
386002345	hypothetical protein Mhar_1661	Yes	1		1791197	1793161	+		Methanosaeta_harundinacea_6Ac_uid81199	NC_017527	chromosome											
386002346	hypothetical protein Mhar_1662	Yes	1		1793164	1794615	+		Methanosaeta_harundinacea_6Ac_uid81199	NC_017527	chromosome											
386002347	hypothetical protein Mhar_1663	No	5		1794612	1795295	+		Methanosaeta_harundinacea_6Ac_uid81199	NC_017527	chromosome											
386002348	hypothetical protein Mhar_1664	No	0		1795292	1796818	+		Methanosaeta_harundinacea_6Ac_uid81199	NC_017527	chromosome			arCOG02202	4	(-)	D	FtsZ	Cell division GTPase			
386002349	hypothetical protein Mhar_1665	Yes	1		1796841	1797422	+		Methanosaeta_harundinacea_6Ac_uid81199	NC_017527	chromosome			arCOG09555	1		S		Uncharacterized conserved protein			
===								8	Methanosaeta_harundinacea_6Ac_uid81199	NC_017527	chromosome											
386002746	Formate dehydrogenase subunit alpha	No	0		2227985	2230012	-		Methanosaeta_harundinacea_6Ac_uid81199	NC_017527	chromosome			arCOG01492	1	(-)	R		Uncharacterized anaerobic dehydrogenase		COG3383	Uncharacterized anaerobic dehydrogenase
386002747	hypothetical protein Mhar_2067	Yes	0		2230200	2231459	+		Methanosaeta_harundinacea_6Ac_uid81199	NC_017527	chromosome											
386002748	hypothetical protein Mhar_2068	No	1		2231561	2231944	+		Methanosaeta_harundinacea_6Ac_uid81199	NC_017527	chromosome											

386002749	hypothetical protein Mhar_2069	Yes	0			2232083	2235148	+		Methanosaeta_harundinacea_6Ac_uid81199	NC_017527	chromosome							3E-18	COG5295	Autotransporter adhesin
386002750	hypothetical protein Mhar_2070	Yes	1			2235149	2236036	+		Methanosaeta_harundinacea_6Ac_uid81199	NC_017527	chromosome		arCOG10866	8	S		Uncharacterized conserved protein			
386002751	hypothetical protein Mhar_2071	No	0			2236133	2237224	+		Methanosaeta_harundinacea_6Ac_uid81199	NC_017527	chromosome									
386002752	hypothetical protein Mhar_2072	Yes	1			2237227	2238120	+		Methanosaeta_harundinacea_6Ac_uid81199	NC_017527	chromosome		arCOG10866	8	S		Uncharacterized conserved protein			
386002753	GTP-binding protein	No	0			2238326	2239339	+		Methanosaeta_harundinacea_6Ac_uid81199	NC_017527	chromosome		arCOG00355	1 (-)	R		GTP-binding protein YscX, possible signaling fac	1E-14	COG0218	Predicted GTPase
===									5	Methanosaeta_harundinacea_6Ac_uid81199	NC_017527	chromosome									
386002824	hypothetical protein Mhar_2147	No	0			2316909	2317562	+		Methanosaeta_harundinacea_6Ac_uid81199	NC_017527	chromosome							6E-13	pfam11655	Protein of unknown function (DUF2589). T
386002825	hypothetical protein Mhar_2148	No	0			2317684	2318433	+		Methanosaeta_harundinacea_6Ac_uid81199	NC_017527	chromosome							0.000005	pfam02463	RecF/RecN/SMC N terminal domain. This do
386002826	hypothetical protein Mhar_2149	No	0			2318446	2319003	+		Methanosaeta_harundinacea_6Ac_uid81199	NC_017527	chromosome									
386002827	hypothetical protein Mhar_2150	Yes	0			2319093	2319698	+		Methanosaeta_harundinacea_6Ac_uid81199	NC_017527	chromosome		arCOG06468	2 (-)	S		Uncharacterized conserved protein			
386002828	hypothetical protein Mhar_2151	No	1			2319778	2319990	-		Methanosaeta_harundinacea_6Ac_uid81199	NC_017527	chromosome									
===									8	Methanosaeta_harundinacea_6Ac_uid81199	NC_017527	chromosome									
386002851	hypothetical protein Mhar_2175	No	0			2337357	2337674	+		Methanosaeta_harundinacea_6Ac_uid81199	NC_017527	chromosome									
386002852	hypothetical protein Mhar_2176	Yes	0			2337724	2338182	-		Methanosaeta_harundinacea_6Ac_uid81199	NC_017527	chromosome									
386002853	Universal stress protein	No	0			2338623	2339054	-		Methanosaeta_harundinacea_6Ac_uid81199	NC_017527	chromosome		arCOG02053	7 (-)	T	UspA	Nucleotide-binding protein, UspA family	2E-25	pfam00582	Universal stress protein family. The uni
386002854	hypothetical protein Mhar_2178	No	0			2339458	2339724	+		Methanosaeta_harundinacea_6Ac_uid81199	NC_017527	chromosome									
386002855	hypothetical protein Mhar_2179	Yes	0			2339855	2340181	+		Methanosaeta_harundinacea_6Ac_uid81199	NC_017527	chromosome									
386002856	hypothetical protein Mhar_2180	No	0			2340715	2340909	+		Methanosaeta_harundinacea_6Ac_uid81199	NC_017527	chromosome		arCOG11378	3	S		Uncharacterized conserved protein			
386002857	hypothetical protein Mhar_2181	No	0			2340924	2341226	+		Methanosaeta_harundinacea_6Ac_uid81199	NC_017527	chromosome		arCOG03403	5 (-)	V		PemK/MazF family toxin			
386002858	hypothetical protein Mhar_2182	No	0			2341625	2341825	-		Methanosaeta_harundinacea_6Ac_uid81199	NC_017527	chromosome		arCOG10004	1	S		Uncharacterized conserved protein			
===									5	Methanosaeta_thermophila_PT_uid58469	NC_008553	chromosome									
116753737	hypothetical protein Mthe_0423	Yes	0			431332	432945	+		Methanosaeta_thermophila_PT_uid58469	NC_008553	chromosome									
116753738	hypothetical protein Mthe_0424	Yes	1			432951	434393	+		Methanosaeta_thermophila_PT_uid58469	NC_008553	chromosome									
116753739	hypothetical protein Mthe_0425	No	5			434390	434971	+		Methanosaeta_thermophila_PT_uid58469	NC_008553	chromosome									
116753740	hypothetical protein Mthe_0426	No	0			434968	436497	+		Methanosaeta_thermophila_PT_uid58469	NC_008553	chromosome		arCOG02202	4 (-)	D	FtsZ	Cell division GTPase			
116753741	hypothetical protein Mthe_0427	Yes	1			436631	437515	+		Methanosaeta_thermophila_PT_uid58469	NC_008553	chromosome									
===									6	Methanosaeta_thermophila_PT_uid58469	NC_008553	chromosome									
116754179	hypothetical protein Mthe_0869	No	0			938879	939166	-		Methanosaeta_thermophila_PT_uid58469	NC_008553	chromosome									
116754180	hypothetical protein Mthe_0870	No	4			939315	939926	-		Methanosaeta_thermophila_PT_uid58469	NC_008553	chromosome									
116754181	hypothetical protein Mthe_0871	Yes	0			941262	941654	-		Methanosaeta_thermophila_PT_uid58469	NC_008553	chromosome									
116754182	aminotransferase, class V	No	0			941790	942938	+		Methanosaeta_thermophila_PT_uid58469	NC_008553	chromosome		arCOG00066	2 (-)	E	NifS	Cysteine sulfinate desulfinase/cysteine desulfura	8E-167	COG1104	Cysteine sulfinate desulfinase/cysteine
116754183	hypothetical protein Mthe_0873	Yes	0			943011	943985	+		Methanosaeta_thermophila_PT_uid58469	NC_008553	chromosome									
116754184	hypothetical protein Mthe_0874	Yes	0			943982	945154	+		Methanosaeta_thermophila_PT_uid58469	NC_008553	chromosome									
===									8	Methanosalsum_zhilinae_DSM_4017_uid68249	NC_015676	chromosome									
336476076	hypothetical protein Mzhil_0118	No	2			132807	133007	+		Methanosalsum_zhilinae_DSM_4017_uid68249	NC_015676	chromosome									
336476077	hypothetical protein Mzhil_0119	No	0			133426	135102	+		Methanosalsum_zhilinae_DSM_4017_uid68249	NC_015676	chromosome							2E-16	pfam14311	Domain of unknown function (DUF4379). Th ; Domain of unknown function (DUF4379). Th ; Domain of unknown function (DUF4379). Th ; Domain of unknown function (DUF4379). Th ; Domain of unknown function (DUF4379). Th ; Domain of unknown function (DUF4379). Th
336476078	hypothetical protein Mzhil_0120	No	0			135203	135466	+		Methanosalsum_zhilinae_DSM_4017_uid68249	NC_015676	chromosome		arCOG01055	9 (-)	K		Predicted transcriptional regulator	0.000000004	COG3432	Predicted transcriptional regulator
336476079	hypothetical protein Mzhil_0121	No	0			135517	136614	-		Methanosalsum_zhilinae_DSM_4017_uid68249	NC_015676	chromosome							0.000003	pfam14281	PD-(D/E)XK nuclease superfamily. Members
336476080	hypothetical protein Mzhil_0122	No	2			137148	137900	-		Methanosalsum_zhilinae_DSM_4017_uid68249	NC_015676	chromosome									
336476081	hypothetical protein Mzhil_0123	No	0			138343	138585	-		Methanosalsum_zhilinae_DSM_4017_uid68249	NC_015676	chromosome									
336476082	transposase IS4 family protein	No	0			140034	140549	-		Methanosalsum_zhilinae_DSM_4017_uid68249	NC_015676	chromosome		arCOG02751	5 (-)	L		Transposase, IS5 family			
336476083	hypothetical protein Mzhil_0125	No	0			140751	141032	-		Methanosalsum_zhilinae_DSM_4017_uid68249	NC_015676	chromosome	clust_353								
===									7	Methanosalsum_zhilinae_DSM_4017_uid68249	NC_015676	chromosome									
336476679	hypothetical protein Mzhil_0736	No	0			804951	805445	-		Methanosalsum_zhilinae_DSM_4017_uid68249	NC_015676	chromosome									
336476680	phage/plasmid primase, P4 family	No	0			805670	807832	-		Methanosalsum_zhilinae_DSM_4017_uid68249	NC_015676	chromosome	arCOG06914,a	arCOG06914	10 (-)	R		Phage or plasmid associated DNA primase	9E-27	COG3378	Phage associated DNA primase
336476681	hypothetical protein Mzhil_0738	No	0			807829	807987	-		Methanosalsum_zhilinae_DSM_4017_uid68249	NC_015676	chromosome									
336476682	hypothetical protein Mzhil_0739	No	0			807984	808286	-		Methanosalsum_zhilinae_DSM_4017_uid68249	NC_015676	chromosome									
336476683	hypothetical protein Mzhil_0740	No	0			808704	809420	+		Methanosalsum_zhilinae_DSM_4017_uid68249	NC_015676	chromosome									
336476684	hypothetical protein Mzhil_0741	No	0			809886	811733	+		Methanosalsum_zhilinae_DSM_4017_uid68249	NC_015676	chromosome		arCOG05223	1 (-)	L		ParB-like nuclease domain containing protein fus	3E-13	COG1479	Uncharacterized conserved protein
336476685	hypothetical protein Mzhil_0742	No	0			811742	812797	+		Methanosalsum_zhilinae_DSM_4017_uid68249	NC_015676	chromosome									
===									9	Methanosalsum_zhilinae_DSM_4017_uid68249	NC_015676	chromosome									
336477238	hypothetical protein Mzhil_1309	No	0			1425013	1427130	+		Methanosalsum_zhilinae_DSM_4017_uid68249	NC_015676	chromosome		arCOG00280	21 (-)	L		HerA helicase	0.00000001	COG0433	HerA helicase
336477239	hypothetical protein Mzhil_1310	No	0			1427175	1427507	+		Methanosalsum_zhilinae_DSM_4017_uid68249	NC_015676	chromosome									
336477240	50S ribosomal protein L22	No	2			1427720	1427920	-		Methanosalsum_zhilinae_DSM_4017_uid68249	NC_015676	chromosome	clust_455								
336477241	Activator of Hsp90 ATPase 1 family protein	No	0			1428702	1429157	+		Methanosalsum_zhilinae_DSM_4017_uid68249	NC_015676	chromosome	clust_227								
336477242	hypothetical protein Mzhil_1313	No	0			1429195	1429398	+		Methanosalsum_zhilinae_DSM_4017_uid68249	NC_015676	chromosome		arCOG11378	3	S		Uncharacterized conserved protein			
336477243	putative transmembrane protein	No	4			1429741	1430430	-		Methanosalsum_zhilinae_DSM_4017_uid68249	NC_015676	chromosome	clust_147						0.000002	COG4758	Predicted membrane protein
336477244	ABC transporter	No	0			1430586	1430777	-		Methanosalsum_zhilinae_DSM_4017_uid68249	NC_015676	chromosome									
336477245	hypothetical protein Mzhil_1317	No	0			1431739	1432017	+		Methanosalsum_zhilinae_DSM_4017_uid68249	NC_015676	chromosome		arCOG05148	1 (-)	S		Uncharacterized conserved protein			
336477246	hypothetical protein Mzhil_1318	No	0			1432014	1432139	+		Methanosalsum_zhilinae_DSM_4017_uid68249	NC_015676	chromosome									
===									6	Methanosarcina_acetivorans_C2A_uid57879	NC_003552	chromosome									
20088942	arginyl-tRNA synthetase	No	0			54809	56512	+		Methanosarcina_acetivorans_C2A_uid57879	NC_003552	chromosome		arCOG00487	1 (-)	J	ArgS	Arginyl-tRNA synthetase			
20088943	hypothetical protein MA0044	No	1			56493	56717	+		Methanosarcina_acetivorans_C2A_uid57879	NC_003552	chromosome									
20088944	hypothetical protein MA0045	No	0			56925	57329	+		Methanosarcina_acetivorans_C2A_uid57879	NC_003552	chromosome		arCOG03486	1	S		Uncharacterized conserved protein			
20088945	hypothetical protein MA0046	No	0			57569	58264	+		Methanosarcina_acetivorans_C2A_uid57879	NC_003552	chromosome		arCOG03487	2	S		Uncharacterized conserved protein			
20088946	hypothetical protein MA0047	No	0			58313	59023	+		Methanosarcina_acetivorans_C2A_uid57879	NC_003552	chromosome		arCOG03487	2	S		Uncharacterized conserved protein			
20088947	hypothetical protein MA0048	No	0			59191	59415	+		Methanosarcina_acetivorans_C2A_uid57879	NC_003552	chromosome		arCOG03966	3 (-)	V		CopG family DNA-binding protein			
===									6	Methanosarcina_acetivorans_C2A_uid57879	NC_003552	chromosome									
20089124	hypothetical protein MA0226	No	0			272895	273161	-		Methanosarcina_acetivorans_C2A_uid57879	NC_003552	chromosome									
20089125	hypothetical protein MA0227	No	0			273162	273887	-		Methanosarcina_acetivorans_C2A_uid57879	NC_003552	chromosome		arCOG03968	2	S		Uncharacterized conserved protein	3E-75	pfam07900	Protein of unknown function (DUF1670). T
20089126	hypothetical protein MA0228	No	0			273899	274654	-		Methanosarcina_acetivorans_C2A_uid57879	NC_003552	chromosome		arCOG03969	2	S		Uncharacterized conserved protein	7E-66	pfam07900	Protein of unknown function (DUF1670). T
20089127	hypothetical protein MA0229	No	1			275092	276009	-		Methanosarcina_acetivorans_C2A_uid57879	NC_003552	chromosome		arCOG02911	3 (-)	S		Uncharacterized conserved membrane protein			
20089128	hypothetical protein MA0230	No	1			276030	276950	-		Methanosarcina_acetivorans_C2A_uid57879	NC_003552	chromosome		arCOG02419	2	S		Uncharacterized conserved protein	8E-12	pfam07790	Protein of unknown function (DUF1628). T
20089129	hypothetical protein MA0231	No	0			277044	277403	-		Methanosarcina_acetivorans_C2A_uid57879	NC_003552	chromosome		arCOG02419	2	S		Uncharacterized conserved protein			
===									6	Methanosarcina_acetivorans_C2A_uid57879	NC_003552	chromosome									
20089285	hypothetical protein MA0388	No	0			470816	472120	-		Methanosarcina_acetivorans_C2A_uid57879	NC_003552	chromosome		arCOG04931	3 (-)	S		Uncharacterized conserved protein			
20089286	hypothetical protein MA0389	No	0			472290	472499	+		Methanosarcina_acetivorans_C2A_uid57879	NC_003552	chromosome		arCOG07804	1	S		Uncharacterized conserved protein			
20089287	hypothetical protein MA0390	No	0			474212	474688	-		Methanosarcina_acetivorans_C2A_uid57879	NC_003552	chromosome									
20089288	hypothetical protein MA0391	No	3			474855	475295	-		Methanosarcina_acetivorans_C2A_uid57879	NC_003552	chromosome									
20089289	hypothetical protein MA0392	No	0			475296	475538	-		Methanosarcina_acetivorans_C2A_uid57879	NC_003552	chromosome									
20089290	cobalt ABC transporter, permease protein	No	5			475733	476515	+		Methanosarcina_acetivorans_C2A_uid57879	NC_003552	chromosome		arCOG02249	1 (-)	P	CbiQ	ABC-type cobalt transport system, permease cor	1E-10	pfam02361	Cobalt transport protein. This family co
===									13	Methanosarcina_acetivorans_C2A_uid57879	NC_003552	chromosome									
20089355	hypothetical protein MA0464	No	0			542972	544933	-		Methanosarcina_acetivorans_C2A_uid57879	NC_003552	chromosome		arCOG05147	2	S		Uncharacterized conserved protein	5E-158	COG4715	Uncharacterized conserved protein
20089356	hypothetical protein MA0465	No	0			544992	546491	-		Methanosarcina_acetivorans_C2A_uid57879	NC_003552	chromosome		arCOG00755	1 (-)	E	DadA	Glycine/D-amino acid oxidase (deaminating)	8E-15	pfam01266	FAD dependent oxidoreductase. This famil ; Rieske Fe-S protein
20089357	hypothetical protein MA0468	No	0			548980	549450	-		Methanosarcina_acetivorans_C2A_uid57879	NC_003552	chromosome									
20089358	hypothetical protein MA0469	Yes	1			550418	551323	+		Methanosarcina_acetivorans_C2A_uid57879	NC_003552	chromosome		arCOG03972	2	S		Uncharacterized conserved protein			
20089359	hypothetical protein MA0470	No	1			551760	551984	-		Methanosarcina_acetivorans_C2A_uid57879	NC_003552	chromosome									
20089360	hypothetical protein MA0471	No	0			552436	552756	+		Methanosarcina_acetivorans_C2A_uid57879	NC_003552	chromosome									
20089361	hypothetical protein MA0472	No	0			552863	554347	-		Methanosarcina_acetivorans_C2A_uid57879	NC_003552	chromosome		arCOG03513	2 (-)	L		Transposase	3E-31	COG3666	Transposase and inactivated derivatives ; Transposase DDE domain. Transposase prot
20089362	hypothetical protein MA0473	Yes	1			554425	554664	+		Methanosarcina_acetivorans_C2A_uid57879	NC_003552	chromosome									
20089363	hypothetical protein MA0474	No	0			554684	555496	+		Methanosarcina_acetivorans_C2A_uid57879	NC_003552	chromosome		arCOG07622	1	S		Uncharacterized conserved protein			
20089364	hypothetical protein MA0475	No	1			555563	556177	+		Methanosarcina_acetivorans_C2A_uid57879	NC_003552	chromosome									
20089365	hypothetical protein MA0476	No	1			556226	556669	+		Methanosarcina_acetivorans_C2A_uid57879	NC_003552	chromosome		arCOG03344	1	S		Uncharacterized conserved protein			
20089366	hypothetical protein MA0477	No	0			557853	558233	+		Methanosarcina_acetivorans_C2A_uid57879	NC_003552	chromosome		arCOG02156	1 (-)	R		Protein implicated in RNA metabolism, contains PRC-barrel domain			
20089367	hypothetical protein MA0478	No	0			558739	558948	-		Methanosarcina_acetivorans_C2A_uid57879	NC_003552	chromosome									
===									13	Methanosarcina_acetivorans_C2A_uid57879	NC_003552	chromosome									
20089381	hypothetical protein MA0492	No	6			576556	577233	-		Methanosarcina_acetivorans_C2A_uid57879	NC_003552	chromosome		arCOG08955	1	S		Uncharacterized conserved protein			
20089382	hypothetical protein MA0493	No	0			577531	578181	+		Methanosarcina_acetivorans_C2A_uid57879	NC_003552	chromosome		arCOG04950	1 (-)	R		Predicted enzyme with a TIM-barrel fold	9E-52	COG0325	Predicted enzyme with a TIM-barrel fold
20089383	hypothetical protein MA0494	No	1			578794	579201	+		Methanosarcina_acetivorans_C2A_uid57879	NC_003552	chromosome		arCOG03345	4	S		Uncharacterized conserved protein			
20089384	hypothetical protein MA0495	No	0			579198	579422	+		Methanosarcina_acetivorans_C2A_uid57879	NC_003552	chromosome									
20089385	hypothetical protein MA0496	No	0			579515	579841	+		Methanosarcina_acetivorans_C2A_uid57879	NC_003552	chromosome									
20089386	hypothetical protein MA0497	No	1			579937	582003	-		Methanosarcina_acetivorans_C2A_uid57879	NC_003552	chromosome		arCOG06708	1	S		Cell suface protein	0.000001	pfam05048	Periplasmic copper-binding protein (NosD ; Carboxypeptidase regulatory-like domain.
20089387	hypothetical protein MA0498	No	0			582140	582343	+		Methanosarcina_acetivorans_C2A_uid57879	NC_003552	chromosome									
20089388	hypothetical protein MA0499	No	0			582771	583250	+		Methanosarcina_acetivorans_C2A_uid57879	NC_003552	chromosome		arCOG03200	1 (-)	K		Transcriptional regulator, effector-binding domain	3E-28	COG4978	Transcriptional regulator, effector-bind
20089389	hypothetical protein MA0500	Yes	0	YES		583586	584113	+		Methanosarcina_acetivorans_C2A_uid57879	NC_003552	chromosome		arCOG03345	4	S		Uncharacterized conserved protein			
20089390	hypothetical protein MA0501	Yes	0	YES		584232	584459	+		Methanosarcina_acetivorans_C2A_uid57879	NC_003552	chromosome									
20089391	hypothetical protein MA0502	No	6	YES		584615	585280	+		Methanosarcina_acetivorans_C2A_uid57879	NC_003552	chromosome									
20089392	hypothetical protein MA0503	No	0	YES		585418	585648	-		Methanosarcina_acetivorans_C2A_uid57879	NC_003552	chromosome									
20089393	ArsR family transcriptional regulator	No	2	YES		585765	586307	-		Methanosarcina_acetivorans_C2A_uid57879	NC_003552	chromosome		arCOG01683	3 (-)	K	ArsR	Transcriptional regulator containing HTH domain,	0.00004	pfam12840	Helix-turn-helix domain. This domain rep
===									11	Methanosarcina_acetivorans_C2A_uid57879	NC_003552	chromosome									
20089442	hypothetical protein MA0553	No	0			649482	649766	+		Methanosarcina_acetivorans_C2A_uid57879	NC_003552	chromosome									
20089443	hypothetical protein MA0554	Yes	1			649933	650793	+		Methanosarcina_acetivorans_C2A_uid57879	NC_003552	chromosome		arCOG03974	2 (-)	S		Uncharacterized conserved protein			
20089444	hypothetical protein MA0555	Yes	0			651520	652707	+		Methanosarcina_acetivorans_C2A_uid57879	NC_003552	chromosome	arCOG03378,a	arCOG03378	1	S		Uncharacterized conserved protein			
20089445	hypothetical protein MA0556	No	0			652958	655138	+		Methanosarcina_acetivorans_C2A_uid57879	NC_003552	chromosome		arCOG03316	7	S		Uncharacterized conserved protein		pfam11369	Protein of unknown function (DUF3160). T
20089446	hypothetical protein MA0557	No	0			655538	657511	+		Methanosarcina_acetivorans_C2A_uid57879	NC_003552	chromosome		arCOG03316	7	S		Uncharacterized conserved protein		pfam11369	Protein of unknown function (DUF3160). T
20089447	hypothetical protein MA0558	No	0			657701	659947	+		Methanosarcina_acetivorans_C2A_uid57879	NC_003552	chromosome		arCOG03316	7	S		Uncharacterized conserved protein		pfam11369	Protein of unknown function (DUF3160). T
20089448	hypothetical protein MA0559	Yes	0			660130	660654	+		Methanosarcina_acetivorans_C2A_uid57879	NC_003552	chromosome		arCOG05151	4	S		Uncharacterized conserved protein			
20089449	hypothetical protein MA0560	No	0			661579	663963	+		Methanosarcina_acetivorans_C2A_uid57879	NC_003552	chromosome		arCOG03316	7	S		Uncharacterized conserved protein		pfam11369	Protein of unknown function (DUF3160). T
20089450	hypothetical protein MA0561	No	0			664015	666390	+		Methanosarcina_acetivorans_C2A_uid57879	NC_003552	chromosome		arCOG03316	7	S		Uncharacterized conserved protein		pfam11369	Protein of unknown function (DUF3160). T
20089451	hypothetical protein MA0562	Yes	0			666579	667085	+		Methanosarcina_acetivorans_C2A_uid57879	NC_003552	chromosome		arCOG05151	4	S		Uncharacterized conserved protein			
20089452	hypothetical protein MA0563	Yes	1			667136	668335	+		Methanosarcina_acetivorans_C2A_uid57879	NC_003552	chromosome		arCOG02470	4 (-)	M	LolA	Outer membrane lipoprotein-sorting protein	1E-13	COG2834	Outer membrane lipoprotein-sorting prote
===									7	Methanosarcina_acetivorans_C2A_uid57879	NC_003552	chromosome									
20089455	hypothetical protein MA0566	Yes	1			671739	672695	+		Methanosarcina_acetivorans_C2A_uid57879	NC_003552	chromosome		arCOG03312	1 (-)	E		Peptidase family C39			
20089456	hypothetical protein MA0567	No	5			672806	673219	+		Methanosarcina_acetivorans_C2A_uid57879	NC_003552	chromosome		arCOG04951	2	S		Uncharacterized conserved protein			
20089457	hypothetical protein MA0568	No	0			673580	673876	+		Methanosarcina_acetivorans_C2A_uid57879	NC_003552	chromosome									
20089458	hypothetical protein MA0569	No	1			674025	674435	+		Methanosarcina_acetivorans_C2A_uid57879	NC_003552	chromosome		arCOG03343	2	S		Uncharacterized conserved protein			
20089459	hypothetical protein MA0570	No	0			674723	675076	+		Methanosarcina_acetivorans_C2A_uid57879	NC_003552	chromosome									
20089460	hypothetical protein MA0571	No	4			675365	675823	+		Methanosarcina_acetivorans_C2A_uid57879	NC_003552	chromosome		arCOG03975	2 (-)	S		Uncharacterized conserved protein			
20089461	hypothetical protein MA0572	Yes	1			676187	675676	+		Methanosarcina_acetivorans_C2A_uid57879	NC_003552	chromosome		arCOG03343	2	S		Uncharacterized conserved protein			
===									6	Methanosarcina_acetivorans_C2A_uid57879	NC_003552	chromosome									
20089622	hypothetical protein MA0737	No	9			856749	857921	-		Methanosarcina_acetivorans_C2A_uid57879	NC_003552	chromosome									
20089623	hypothetical protein MA0738	No	0			858220	858504	-		Methanosarcina_acetivorans_C2A_uid57879	NC_003552	chromosome									
20089624	hypothetical protein MA0739	No	0			858898	859557	-		Methanosarcina_acetivorans_C2A_uid57879	NC_003552	chromosome	arCOG00528,a	arCOG00528	1 (-)	R		Pyridoxamine 5'-phosphate oxidase related prot	5E-51	COG5015	Uncharacterized conserved protein
20089625	hypothetical protein MA0740	No	3			859801	860394	-		Methanosarcina_acetivorans_C2A_uid57879	NC_003552	chromosome		arCOG06719	1	S		Uncharacterized conserved protein	4E-12	pfam11667	Protein of unknown function (DUF3267). T
20089626	hypothetical protein MA0741	No	0			860637	860936	+		Methanosarcina_acetivorans_C2A_uid57879	NC_003552	chromosome									
20089627	hypothetical protein MA0742	Yes	2			861669	862058	+		Methanosarcina_acetivorans_C2A_uid57879	NC_003552	chromosome		arCOG05198	1	S		Uncharacterized conserved protein			
===									5	Methanosarcina_acetivorans_C2A_uid57879	NC_003552	chromosome									
20089755	hypothetical protein MA0871	Yes	8			1034157	1035809	-		Methanosarcina_acetivorans_C2A_uid57879	NC_003552	chromosome		arCOG05162	3	S		Uncharacterized conserved protein			
20089756	cobaltochelatase subunit CobN	No	0			1035751	1039659	-		Methanosarcina_acetivorans_C2A_uid57879	NC_003552	chromosome		arCOG03022	5 (-)	H	CobN	Cobalamin biosynthesis protein CobN or related Mg-chelatase		pfam02514	CobN/Magnesium Chelatase. This family co
20089757	PQQ repeat-containing protein	Yes	0			1039669	1041381	-		Methanosarcina_acetivorans_C2A_uid57879	NC_003552	chromosome		arCOG02489	6	S		WD40 repeats containing protein			
20089758	hypothetical protein MA0874	Yes	1			1041386	1042909	-		Methanosarcina_acetivorans_C2A_uid57879	NC_003552	chromosome		arCOG02489	6	S		WD40 repeats containing protein	0.00001	COG2319	FOG: WD40 repeat
20089759	hypothetical protein MA0875	No	1			1042949	1043542	-		Methanosarcina_acetivorans_C2A_uid57879	NC_003552	chromosome		arCOG05163	3	S		Uncharacterized conserved protein			
===									5	Methanosarcina_acetivorans_C2A_uid57879	NC_003552	chromosome									
20090164	hypothetical protein MA1300	No	2	YES		1559875	1560264	+		Methanosarcina_acetivorans_C2A_uid57879	NC_003552	chromosome									
20090165	hypothetical protein MA1301	No	0	YES		1560795	1561019	-		Methanosarcina_acetivorans_C2A_uid57879	NC_003552	chromosome									
20090166	tungsten-containing aldehyde ferredoxin oxido	No	0			1561216	1562556	-		Methanosarcina_acetivorans_C2A_uid57879	NC_003552	chromosome		arCOG00938	12 (-)	R		Radical SAM superfamily enzyme	1E-35	COG0535	Predicted Fe-S oxidoreductases
20090167	hypothetical protein MA1303	No	0	YES		1562845	1563189	-		Methanosarcina_acetivorans_C2A_uid57879	NC_003552	chromosome									
20090168	hypothetical protein MA1304	No	0	YES		1563240	1563626	+		Methanosarcina_acetivorans_C2A_uid57879	NC_003552	chromosome		arCOG07807	3	S		Uncharacterized conserved protein			
===									7	Methanosarcina_acetivorans_C2A_uid57879	NC_003552	chromosome									
20090262	hypothetical protein MA1402	No	1			1669311	1669769	+		Methanosarcina_acetivorans_C2A_uid57879	NC_003552	chromosome									
20090263	hypothetical protein MA1403	No	0			1669947	1670669	-		Methanosarcina_acetivorans_C2A_uid57879	NC_003552	chromosome		arCOG05179	1 (-)	R		Haloacid dehalogenase (HAD) superfamily phosphatase			
20090264	hypothetical protein MA1404	No	1			1671280	1671753	+		Methanosarcina_acetivorans_C2A_uid57879	NC_003552	chromosome		arCOG07808	1	S		Uncharacterized conserved protein			
20090265	hypothetical protein MA1405	Yes	5			1671761	1672312	+		Methanosarcina_acetivorans_C2A_uid57879	NC_003552	chromosome		arCOG06729	1	S		Uncharacterized conserved membrane protein	0.00000001	COG0398	Uncharacterized conserved protein
20090266	hypothetical protein MA1406	Yes	1			1672472	1672834	+		Methanosarcina_acetivorans_C2A_uid57879	NC_003552	chromosome									
20090267	hypothetical protein MA1407	No	0			1673495	1673872	-		Methanosarcina_acetivorans_C2A_uid57879	NC_003552	chromosome		arCOG06730	1	S		Uncharacterized conserved protein			
20090268	hypothetical protein MA1408	No	6			1674455	1675708	+		Methanosarcina_acetivorans_C2A_uid57879	NC_003552	chromosome		arCOG04151	1 (-)	R		ABC-type multidrug transport system, permease	1E-98	COG2237	Predicted membrane protein
===									9	Methanosarcina_acetivorans_C2A_uid57879	NC_003552	chromosome									
20090415	hypothetical protein MA1557	No	0			1842711	1843025	-		Methanosarcina_acetivorans_C2A_uid57879	NC_003552	chromosome									
20090416	hypothetical protein MA1558	No	0			1843643	1844185	+		Methanosarcina_acetivorans_C2A_uid57879	NC_003552	chromosome									
20090417	hypothetical protein MA1559	No	0			1844182	1845138	+		Methanosarcina_acetivorans_C2A_uid57879	NC_003552	chromosome		arCOG07960	16 (-)	R		ATPase (AAA+ superfamily)			
20090418	hypothetical protein MA1560	No	0			1846035	1846298	-		Methanosarcina_acetivorans_C2A_uid57879	NC_003552	chromosome									
20090419	hypothetical protein MA1561	No	3			1846401	1846946	-		Methanosarcina_acetivorans_C2A_uid57879	NC_003552	chromosome									
20090420	hypothetical protein MA1562	No	0			1846958	1847947	-		Methanosarcina_acetivorans_C2A_uid57879	NC_003552	chromosome		arCOG07810	2 (-)	T	CyaA	Adenylate cyclase, family 3			
20090421	hypothetical protein MA1563	No	1			1849937	1850524	-		Methanosarcina_acetivorans_C2A_uid57879	NC_003552	chromosome		arCOG07647	3	S		Uncharacterized conserved protein			
20090422	hypothetical protein MA1564	Yes	3			1851088	1851807	+		Methanosarcina_acetivorans_C2A_uid57879	NC_003552	chromosome									
20090423	hypothetical protein MA1565	No	0			1852634	1854316	-		Methanosarcina_acetivorans_C2A_uid57879	NC_003552	chromosome	clust_71								
===									9	Methanosarcina_acetivorans_C2A_uid57879	NC_003552	chromosome									
20090733	IstB helper protein	No	0			2319615	2320388	+		Methanosarcina_acetivorans_C2A_uid57879	NC_003552	chromosome		arCOG06699	1 (-)	L	IstB	IstB transposon helper protein, DNA replication p	2E-89	COG1484	DNA replication protein
20090734	hypothetical protein MA1884	No	5			2321007	2322068	-		Methanosarcina_acetivorans_C2A_uid57879	NC_003552	chromosome		arCOG06750	1	S		Uncharacterized conserved protein			
20090735	hypothetical protein MA1885	No	2			2322273	2322890	+		Methanosarcina_acetivorans_C2A_uid57879	NC_003552	chromosome		arCOG03996	1	S		Zn finger protein			
20090736	hypothetical protein MA1886	No	0			2323237	2323674	-		Methanosarcina_acetivorans_C2A_uid57879	NC_003552	chromosome									
20090737	hypothetical protein MA1887	No	0			2323667	2324554	-		Methanosarcina_acetivorans_C2A_uid57879	NC_003552	chromosome									
20090738	hypothetical protein MA1888	No	0			2325061	2325420	+		Methanosarcina_acetivorans_C2A_uid57879	NC_003552	chromosome									
20090739	hypothetical protein MA1889	No	0			2325429	2326154	+		Methanosarcina_acetivorans_C2A_uid57879	NC_003552	chromosome									
20090740	transposase	No	0			2326246	2327163	-		Methanosarcina_acetivorans_C2A_uid57879	NC_003552	chromosome		arCOG02125	5 (-)	L	Tra5	Transposase	1E-19	COG2801	Transposase and inactivated derivatives
20090741	hypothetical protein MA1891	No	0			2327639	2329846	-		Methanosarcina_acetivorans_C2A_uid57879	NC_003552	chromosome									
===									9	Methanosarcina_acetivorans_C2A_uid57879	NC_003552	chromosome									
20090743	hypothetical protein MA1893	No	0			2331951	2332805	-		Methanosarcina_acetivorans_C2A_uid57879	NC_003552	chromosome									
20090744	hypothetical protein MA1894	No	1			2332802	2333113	-		Methanosarcina_acetivorans_C2A_uid57879	NC_003552	chromosome									
20090745	hypothetical protein MA1895	No	0			2334449	2336368	-		Methanosarcina_acetivorans_C2A_uid57879	NC_003552	chromosome		arCOG07652	5 (-)	L		Superfamily II helicase or inactivated derivatives	1E-48	COG5519	Superfamily II helicase and inactivated
20090746	hypothetical protein MA1896	No	0			2336620	2337906	-		Methanosarcina_acetivorans_C2A_uid57879	NC_003552	chromosome	clust_2								
20090747	hypothetical protein MA1897	No	0			2337932	2338687	-		Methanosarcina_acetivorans_C2A_uid57879	NC_003552	chromosome									
20090748	hypothetical protein MA1898	No	0			2338897	2339142	-		Methanosarcina_acetivorans_C2A_uid57879	NC_003552	chromosome									
20090749	hypothetical protein MA1899	No	0			2339303	2339674	-		Methanosarcina_acetivorans_C2A_uid57879	NC_003552	chromosome									
20090750	hypothetical protein MA1900	No	0			2340293	2341579	-		Methanosarcina_acetivorans_C2A_uid57879	NC_003552	chromosome	clust_2								
20090751	alcohol dehydrogenase (NADP+)	No	1			2341794	2342864	+		Methanosarcina_acetivorans_C2A_uid57879	NC_003552	chromosome		arCOG01459	1 (-)	E	Tdh	Threonine dehydrogenase or related Zn-depende	1E-45	COG1063	Threonine dehydrogenase and related Zn-d
===									11	Methanosarcina_acetivorans_C2A_uid57879	NC_003552	chromosome									
20090882	potassium channel protein	No	6			2526960	2527799	+		Methanosarcina_acetivorans_C2A_uid57879	NC_003552	chromosome		arCOG01964	3 (-)	P	Kch	Kef-type K+ transport system, predicted NAD-bin	3E-19	pfam00520	Ion transport protein. This family conta
20090883	hypothetical protein MA2035	No	0			2527970	2528227	-		Methanosarcina_acetivorans_C2A_uid57879	NC_003552	chromosome									
20090884	hypothetical protein MA2036	No	0			2528432	2528890	-		Methanosarcina_acetivorans_C2A_uid57879	NC_003552	chromosome									
20090885	hypothetical protein MA2038	No	0			2530357	2530686	-		Methanosarcina_acetivorans_C2A_uid57879	NC_003552	chromosome									
20090886	hypothetical protein MA2039	No	0			2530687	2530986	-		Methanosarcina_acetivorans_C2A_uid57879	NC_003552	chromosome									
20090887	hypothetical protein MA2040	No	0			2531003	2531278	-		Methanosarcina_acetivorans_C2A_uid57879	NC_003552	chromosome									
20090888	hypothetical protein MA2041	No	0			2531271	2531972	-		Methanosarcina_acetivorans_C2A_uid57879	NC_003552	chromosome							5E-22	COG5529	Pyocin large subunit
20090889	hypothetical protein MA2042	No	0			2532087	2532491	-		Methanosarcina_acetivorans_C2A_uid57879	NC_003552	chromosome									
20090890	hypothetical protein MA2043	No	0			2532488	2533810	-		Methanosarcina_acetivorans_C2A_uid57879	NC_003552	chromosome							7E-14	COG3209	Rhs family protein
20090891	hypothetical protein MA2044	No	0			2533814	2534485	-		Methanosarcina_acetivorans_C2A_uid57879	NC_003552	chromosome									
20090892	hypothetical protein MA2045	Yes	0			2534487	2541140	-		Methanosarcina_acetivorans_C2A_uid57879	NC_003552	chromosome		arCOG03509	2 (-)	R		Cell suface protein, containing PKD repeats	1E-34	COG3209	Rhs family protein
===									7	Methanosarcina_acetivorans_C2A_uid57879	NC_003552	chromosome									
20090911	Met-10+ related protein	No	0			2557595	2558626	-		Methanosarcina_acetivorans_C2A_uid57879	NC_003552	chromosome		arCOG10124	1 (-)	J	TYW2	Wybutosine (yW) biosynthesis enzyme, TYW2 tra	4E-84	COG2520	Predicted methyltransferase
20090912	hypothetical protein MA2065	No	0			2559097	2559450	+		Methanosarcina_acetivorans_C2A_uid57879	NC_003552	chromosome	clust_303								
20090913	hypothetical protein MA2066	No	0			2559494	2560531	+		Methanosarcina_acetivorans_C2A_uid57879	NC_003552	chromosome									
20090914	hypothetical protein MA2067	No	0			2560562	2560894	+		Methanosarcina_acetivorans_C2A_uid57879	NC_003552	chromosome									
20090915	hypothetical protein MA2069	No	2			2561769	2562611	+		Methanosarcina_acetivorans_C2A_uid57879	NC_003552	chromosome									
20090916	hypothetical protein MA2070	No	0			2563061	2563345	+		Methanosarcina_acetivorans_C2A_uid57879	NC_003552	chromosome		arCOG06758	1	S		Uncharacterized conserved protein	0.0001	COG4104	Uncharacterized conserved protein
20090917	transposase	No	0			2563549	2563989	-		Methanosarcina_acetivorans_C2A_uid57879	NC_003552	chromosome		arCOG03585	6 (-)	L		Transposase	2E-13	pfam02371	Transposase IS116/IS110/IS902 family. Tr
===									5	Methanosarcina_acetivorans_C2A_uid57879	NC_003552	chromosome									
20090944	hypothetical protein MA2100	No	0	YES		2601180	2601404	+		Methanosarcina_acetivorans_C2A_uid57879	NC_003552	chromosome		arCOG05204	2	S		Uncharacterized conserved protein			
20090945	hypothetical protein MA2101	No	0	YES		2601882	2602109	+		Methanosarcina_acetivorans_C2A_uid57879	NC_003552	chromosome									
20090946	hypothetical protein MA2102	No	0	YES		2603087	2604778	-		Methanosarcina_acetivorans_C2A_uid57879	NC_003552	chromosome									
20090947	type I site-specific deoxyribonuclease	No	0	YES		2605359	2606231	-		Methanosarcina_acetivorans_C2A_uid57879	NC_003552	chromosome		arCOG02626	3 (-)	V	HsdS	Restriction endonuclease S subunit	4E-19	COG0732	Restriction endonuclease S subunits
20090948	hypothetical protein MA2104	No	0	YES		2606340	2607626	+		Methanosarcina_acetivorans_C2A_uid57879	NC_003552	chromosome	clust_2								
===									9	Methanosarcina_acetivorans_C2A_uid57879	NC_003552	chromosome									
20090965	type I site-specific deoxyribonuclease	No	0	YES		2627964	2630717	-		Methanosarcina_acetivorans_C2A_uid57879	NC_003552	chromosome		arCOG00880	2 (-)	V	HsdR	Type I site-specific restriction-modification systen	4E-85	COG4096	Type I site-specific restriction-modific
20090966	hypothetical protein MA2123	No	0	YES		2631809	2632033	+		Methanosarcina_acetivorans_C2A_uid57879	NC_003552	chromosome									
20090967	hypothetical protein MA2124	No	0	YES		2632077	2633009	+		Methanosarcina_acetivorans_C2A_uid57879	NC_003552	chromosome									
20090968	hypothetical protein MA2125	No	0	YES		2633820	2634038	-		Methanosarcina_acetivorans_C2A_uid57879	NC_003552	chromosome									
20090969	hypothetical protein MA2126	No	2	YES		2634380	2634649	+		Methanosarcina_acetivorans_C2A_uid57879	NC_003552	chromosome									
20090970	hypothetical protein MA2127	No	1	YES		2634628	2634864	+		Methanosarcina_acetivorans_C2A_uid57879	NC_003552	chromosome									
20090971	hypothetical protein MA2128	No	0	YES		2634828	2635259	+		Methanosarcina_acetivorans_C2A_uid57879	NC_003552	chromosome									
20090972	hypothetical protein MA2129	No	0	YES		2635561	2635884	-		Methanosarcina_acetivorans_C2A_uid57879	NC_003552	chromosome		arCOG05205	2	S		Uncharacterized conserved protein			
20090973	hypothetical protein MA2130	No	0	YES		2636168	2636461	-		Methanosarcina_acetivorans_C2A_uid57879	NC_003552	chromosome		arCOG05024	1 (-)	V		HEPN domain containing protein	1E-35	COG2361	Uncharacterized conserved protein
===									8	Methanosarcina_acetivorans_C2A_uid57879	NC_003552	chromosome									
20091064	transposase A	No	0			2734866	2736029	+		Methanosarcina_acetivorans_C2A_uid57879	NC_003552	chromosome		arCOG01242	14 (-)	L	XerC	Integrase	6E-10	pfam00589	Phage integrase family. Members of this
20091065	hypothetical protein MA2225	No	0			2736566	2736826	+		Methanosarcina_acetivorans_C2A_uid57879	NC_003552	chromosome									
20091066	hypothetical protein MA2226	No	0			2737031	2737294	+		Methanosarcina_acetivorans_C2A_uid57879	NC_003552	chromosome									
20091067	hypothetical protein MA2227	No	0			2737559	2737846	-		Methanosarcina_acetivorans_C2A_uid57879	NC_003552	chromosome									
20091068	hypothetical protein MA2228	No	7			2737873	2738901	-		Methanosarcina_acetivorans_C2A_uid57879	NC_003552	chromosome		arCOG11077	1	S		Uncharacterized conserved protein			
20091069	hypothetical protein MA2229	No	0			2738913	2739329	+		Methanosarcina_acetivorans_C2A_uid57879	NC_003552	chromosome									
20091070	hypothetical protein MA2230	No	0			2739326	2739664	-		Methanosarcina_acetivorans_C2A_uid57879	NC_003552	chromosome									
20091071	site-specific recombinase	No	0			2739683	2740627	-		Methanosarcina_acetivorans_C2A_uid57879	NC_003552	chromosome		arCOG01241	31 (-)	L	XerC	Integrase	2E-13	pfam00589	Phage integrase family. Members of this
===									5	Methanosarcina_acetivorans_C2A_uid57879	NC_003552	chromosome									
20091073	hypothetical protein MA2233	No	0			2743489	2744352	+		Methanosarcina_acetivorans_C2A_uid57879	NC_003552	chromosome							0.00000003	pfam05701	Plant protein of unknown function (DUF82
20091074	hypothetical protein MA2234	No	0			2745242	2745676	+		Methanosarcina_acetivorans_C2A_uid57879	NC_003552	chromosome									
20091075	hypothetical protein MA2235	No	0			2746659	2748899	-		Methanosarcina_acetivorans_C2A_uid57879	NC_003552	chromosome		arCOG07652	5 (-)	L		Superfamily II helicase or inactivated derivatives	2E-79	COG5519	Superfamily II helicase and inactivated
20091076	hypothetical protein MA2236	No	2			2749240	2749650	-		Methanosarcina_acetivorans_C2A_uid57879	NC_003552	chromosome									
20091077	hypothetical protein MA2237	No	1			2750009	2750341	+		Methanosarcina_acetivorans_C2A_uid57879	NC_003552	chromosome									
===									6	Methanosarcina_acetivorans_C2A_uid57879	NC_003552	chromosome									
20091133	flavoredoxin Flr	No	0			2824137	2824703	-		Methanosarcina_acetivorans_C2A_uid57879	NC_003552	chromosome		arCOG02017	1 (-)	R		Conserved protein/domain typically associated w	2E-18	pfam01613	Flavin reductase like domain. This is a
20091134	hypothetical protein MA2296	No	1			2824935	2825150	+		Methanosarcina_acetivorans_C2A_uid57879	NC_003552	chromosome		arCOG05214	3	S		Uncharacterized conserved protein			
20091135	hypothetical protein MA2297	No	1			2825407	2825925	+		Methanosarcina_acetivorans_C2A_uid57879	NC_003552	chromosome		arCOG05214	3	S		Uncharacterized conserved protein			
20091136	hypothetical protein MA2298	No	1			2826229	2826564	+		Methanosarcina_acetivorans_C2A_uid57879	NC_003552	chromosome		arCOG06768	1	S		Uncharacterized conserved protein			
20091137	hypothetical protein MA2299	No	1			2826586	2827098	+		Methanosarcina_acetivorans_C2A_uid57879	NC_003552	chromosome		arCOG05214	3	S		Uncharacterized conserved protein			
20091138	5-oxoprolinase (ATP-hydrolyzing)	No	0			2827266	2831120	-		Methanosarcina_acetivorans_C2A_uid57879	NC_003552	chromosome		arCOG01511	3 (-)	E	HyuA	N-methylhydantoinase A/acetone carboxylase, beta subunit		pfam02538	Hydantoinase B/oxoprolinase. This family ; N-methylhydantoinase A/acetone carboxyla
===									8	Methanosarcina_acetivorans_C2A_uid57879	NC_003552	chromosome									
20091150	hypothetical protein MA2312	No	0			2846894	2847184	-		Methanosarcina_acetivorans_C2A_uid57879	NC_003552	chromosome		arCOG06771	1	S		Uncharacterized conserved protein			
20091151	polysaccharide biosynthesis protein	No	14			2848117	2849664	-		Methanosarcina_acetivorans_C2A_uid57879	NC_003552	chromosome		arCOG02209	4 (-)	R	RfbX	Polysaccharide biosynthesis protein, Mvin family	1E-42	COG2244	Membrane protein involved in the export
20091152	hypothetical protein MA2314	No	0			2849680	2850138	-		Methanosarcina_acetivorans_C2A_uid57879	NC_003552	chromosome		arCOG06772	1	S		Uncharacterized conserved protein			
20091153	hypothetical protein MA2315	No	0			2850125	2850514	-		Methanosarcina_acetivorans_C2A_uid57879	NC_003552	chromosome									
20091154	hypothetical protein MA2318	No	0			2851959	2852192	+		Methanosarcina_acetivorans_C2A_uid57879	NC_003552	chromosome		arCOG06773	1	S		Uncharacterized conserved protein			
20091155	hypothetical protein MA2319	No	0			2852717	2853898	-		Methanosarcina_acetivorans_C2A_uid57879	NC_003552	chromosome		arCOG03574	1	S		Uncharacterized conserved protein	3E-22	pfam06245	Protein of unknown function (DUF1015). F
20091156	metallo-beta-lactamase	No	0			2854167	2855540	-		Methanosarcina_acetivorans_C2A_uid57879	NC_003552	chromosome	arCOG00517,a	arCOG00517	1 (-)	R	GloB	Rhodanese Homology Domain fused to Zn-depe	2E-17	COG0607	Rhodanese-related sulfurtransferase
20091157	hypothetical protein MA2321	No	0			2855788	2856351	+		Methanosarcina_acetivorans_C2A_uid57879	NC_003552	chromosome		arCOG05215	1	S		Uncharacterized conserved protein			
===									7	Methanosarcina_acetivorans_C2A_uid57879	NC_003552	chromosome									
20091161	hypothetical protein MA2325	No	0			2860946	2863105	+		Methanosarcina_acetivorans_C2A_uid57879	NC_003552	chromosome		arCOG05285	2 (-)	S		Uncharacterized conserved protein	2E-67	COG5361	Uncharacterized conserved protein
20091162	hypothetical protein MA2327	Yes	1			2864308	2865918	+		Methanosarcina_acetivorans_C2A_uid57879	NC_003552	chromosome									
20091163	hypothetical protein MA2328	Yes	1			2866121	2867065	+		Methanosarcina_acetivorans_C2A_uid57879	NC_003552	chromosome									
20091164	hypothetical protein MA2329	No	0			2867342	2868058	+		Methanosarcina_acetivorans_C2A_uid57879	NC_003552	chromosome									
20091165	hypothetical protein MA2330	No	0			2868046	2868312	-		Methanosarcina_acetivorans_C2A_uid57879	NC_003552	chromosome									
20091166	hypothetical protein MA2331	No	0			2868824	2869219	+		Methanosarcina_acetivorans_C2A_uid57879	NC_003552	chromosome		arCOG03595	1	S		Uncharacterized conserved protein	0.000000002	pfam11387	Protein of unknown function (DUF2795). T ; Protein of unknown function (DUF2795). T
20091167	hypothetical protein MA2332	No	0			2869512	2870009	-		Methanosarcina_acetivorans_C2A_uid57879	NC_003552	chromosome		arCOG03459	1 (-)	S		Uncharacterized conserved protein			
===									7	Methanosarcina_acetivorans_C2A_uid57879	NC_003552	chromosome									
20091222	hypothetical protein MA2391	No	0			2961250	2962959	+		Methanosarcina_acetivorans_C2A_uid57879	NC_003552	chromosome		arCOG03575	1 (-)	F		Polyphosphate kinase 2	5E-64	pfam03976	Polyphosphate kinase 2 (PPK2). Inorganic ; Polyphosphate kinase 2 (PPK2). Inorganic
20091223	hypothetical protein MA2392	No	0			2963117	2963344	+		Methanosarcina_acetivorans_C2A_uid57879	NC_003552	chromosome									
20091224	hypothetical protein MA2393	No	0			2963638	2964924	-		Methanosarcina_acetivorans_C2A_uid57879	NC_003552	chromosome	clust_2								
20091225	hypothetical protein MA2394	No	0			2965711	2965992	+		Methanosarcina_acetivorans_C2A_uid57879	NC_003552	chromosome									
20091226	hypothetical protein MA2395	No	0			2966285	2966692	+		Methanosarcina_acetivorans_C2A_uid57879	NC_003552	chromosome		arCOG07656	3	S		Uncharacterized conserved protein			
20091227	hypothetical protein MA2396	No	0			2966840	2967061	+		Methanosarcina_acetivorans_C2A_uid57879	NC_003552	chromosome									
20091228	sensory transduction histidine kinase	No	0			2967830	2969869	+		Methanosarcina_acetivorans_C2A_uid57879	NC_003552	chromosome	arCOG02330,a	arCOG02330	2 (-)	T	AtoS	Bacterio-opsin activator protein (contains PAS, G	3E-13	COG2202	FOG: PAS/PAC domain
===									5	Methanosarcina_acetivorans_C2A_uid57879	NC_003552	chromosome									
20091354	beta/gama crystallin family protein	Yes	1			3140436	3140798	-		Methanosarcina_acetivorans_C2A_uid57879	NC_003552	chromosome		arCOG07640	1	S		beta/gamma crystallin family protein	2E-14	pfam00030	Beta/Gamma crystallin. The alignment com
20091355	hypothetical protein MA2527	Yes	1			3142648	3142872	+		Methanosarcina_acetivorans_C2A_uid57879	NC_003552	chromosome		arCOG05225	2	S		Uncharacterized conserved protein			
20091356	cellulosomal protein	No	0			3143359	3144510	-		Methanosarcina_acetivorans_C2A_uid57879	NC_003552	chromosome		arCOG07654	1 (-)	M	CotH	Spore coat assembly protein	2E-16	pfam08757	CotH protein. Members of this family inc
20091357	extracellular protease	No	0			3145301	3146443	+		Methanosarcina_acetivorans_C2A_uid57879	NC_003552	chromosome							2E-87	COG3227	Zinc metalloprotease (elastase)
20091358	hypothetical protein MA2530	No	0			3146650	3146976	+		Methanosarcina_acetivorans_C2A_uid57879	NC_003552	chromosome									
===									25	Methanosarcina_acetivorans_C2A_uid57879	NC_003552	chromosome									
20091410	hypothetical protein MA2584	No	0		integrated_ele	3213869	3216559	-		Methanosarcina_acetivorans_C2A_uid57879	NC_003552	chromosome									
20091411	hypothetical protein MA2585	No	1		integrated_ele	3216813	3217253	-		Methanosarcina_acetivorans_C2A_uid57879	NC_003552	chromosome									
20091412	hypothetical protein MA2586	Yes	2		integrated_ele	3217253	3219289	-		Methanosarcina_acetivorans_C2A_uid57879	NC_003552	chromosome		arCOG07813	7 (-)	G		Glycosyl hydrolases family 43	0.00006	pfam02973	Sialidase, N-terminal domain.
20091413	hypothetical protein MA2587	No	3		integrated_ele	3219531	3219836	-		Methanosarcina_acetivorans_C2A_uid57879	NC_003552	chromosome									
20091414	hypothetical protein MA2588	No	2		integrated_ele	3219833	3220102	-		Methanosarcina_acetivorans_C2A_uid57879	NC_003552	chromosome									
20091415	hypothetical protein MA2589	Yes	2		integrated_ele	3220201	3222315	-		Methanosarcina_acetivorans_C2A_uid57879	NC_003552	chromosome									
20091416	hypothetical protein MA2590	Yes	2		integrated_ele	3222315	3222551	-		Methanosarcina_acetivorans_C2A_uid57879	NC_003552	chromosome									
20091417	hypothetical protein MA2591	No	0		integrated_ele	3222552	3223391	-		Methanosarcina_acetivorans_C2A_uid57879	NC_003552	chromosome									
20091418	hypothetical protein MA2592	No	0		integrated_ele	3223391	3223804	-		Methanosarcina_acetivorans_C2A_uid57879	NC_003552	chromosome									
20091419	hypothetical protein MA2593	No	2		integrated_ele	3223820	3224479	-		Methanosarcina_acetivorans_C2A_uid57879	NC_003552	chromosome									
20091420	hypothetical protein MA2594	No	0		integrated_ele	3224476	3225060	-		Methanosarcina_acetivorans_C2A_uid57879	NC_003552	chromosome									
20091421	hypothetical protein MA2595	No	1		integrated_ele	3225097	3225681	+		Methanosarcina_acetivorans_C2A_uid57879	NC_003552	chromosome									
20091422	hypothetical protein MA2596	No	0		integrated_ele	3225849	3226130	+		Methanosarcina_acetivorans_C2A_uid57879	NC_003552	chromosome									
20091423	hypothetical protein MA2597	No	1		integrated_ele	3226127	3226417	+		Methanosarcina_acetivorans_C2A_uid57879	NC_003552	chromosome									
20091424	hypothetical protein MA2598	Yes	2		integrated_ele	3226414	3226809	+		Methanosarcina_acetivorans_C2A_uid57879	NC_003552	chromosome		arCOG02298	6 (-)	N	FlaK/PulC	Peptidase A24A, prepilin type IV	0.00002	COG4960	Flp pilus assembly protein, protease Cpa
20091425	phage integrase	No	2		integrated_ele	3226951	3227526	-		Methanosarcina_acetivorans_C2A_uid57879	NC_003552	chromosome		arCOG01241	31 (-)	L	XerC	Integrase			
20091426	hypothetical protein MA2600	No	9		integrated_ele	3227880	3228956	-		Methanosarcina_acetivorans_C2A_uid57879	NC_003552	chromosome									
20091427	hypothetical protein MA2601	No	0		integrated_ele	3229629	3231125	+		Methanosarcina_acetivorans_C2A_uid57879	NC_003552	chromosome		arCOG07814	1	S		Uncharacterized conserved protein			
20091428	hypothetical protein MA2602	No	0		integrated_ele	3232687	3234723	-		Methanosarcina_acetivorans_C2A_uid57879	NC_003552	chromosome									
20091429	hypothetical protein MA2603	No	2		integrated_ele	3235205	3235744	-		Methanosarcina_acetivorans_C2A_uid57879	NC_003552	chromosome									
20091430	hypothetical protein MA2604	No	0		integrated_ele	3235948	3237261	-		Methanosarcina_acetivorans_C2A_uid57879	NC_003552	chromosome		arCOG06786	9	Y		Rolling circle replication protein Rep			
20091431	hypothetical protein MA2605	No	0		integrated_ele	3237620	3238069	-		Methanosarcina_acetivorans_C2A_uid57879	NC_003552	chromosome									
20091432	hypothetical protein MA2606	No	0		integrated_ele	3238066	3238551	-		Methanosarcina_acetivorans_C2A_uid57879	NC_003552	chromosome									
20091433	hypothetical protein MA2607	No	0		integrated_ele	3238973	3239425	-		Methanosarcina_acetivorans_C2A_uid57879	NC_003552	chromosome		arCOG06787	4 (-)	L		PD-(D/E)XK superfamily nuclease			
20091434	hypothetical protein MA2608	No	0		integrated_ele	3239582	3239917	+		Methanosarcina_acetivorans_C2A_uid57879	NC_003552	chromosome									
===									11	Methanosarcina_acetivorans_C2A_uid57879	NC_003552	chromosome									
20091457	hypothetical protein MA2634	No	0			3263773	3265059	-		Methanosarcina_acetivorans_C2A_uid57879	NC_003552	chromosome	clust_2								
20091458	hypothetical protein MA2635	No	8			3265831	3266904	+		Methanosarcina_acetivorans_C2A_uid57879	NC_003552	chromosome		arCOG02642	3 (-)	R	yhhT	Predicted permease, member of the PurR regulc	7E-36	COG0628	Predicted permease, member of the PurR r
20091459	hypothetical protein MA2636	No	2			3267238	3267735	+		Methanosarcina_acetivorans_C2A_uid57879	NC_003552	chromosome							0.000000001	COG4803	Predicted membrane protein
20091460	hypothetical protein MA2637	No	0			3268085	3268378	-		Methanosarcina_acetivorans_C2A_uid57879	NC_003552	chromosome									
20091461	hypothetical protein MA2638	Yes	3			3268419	3268811	+		Methanosarcina_acetivorans_C2A_uid57879	NC_003552	chromosome									
20091462	hypothetical protein MA2639	No	0			3269097	3269714	+		Methanosarcina_acetivorans_C2A_uid57879	NC_003552	chromosome		arCOG06788	4	S		Uncharacterized conserved protein, DUF1269 fan	0.00000003	COG4803	Predicted membrane protein
20091463	hypothetical protein MA2640	No	0			3270346	3270882	+		Methanosarcina_acetivorans_C2A_uid57879	NC_003552	chromosome		arCOG06788	4	S		Uncharacterized conserved protein, DUF1269 fan	4E-10	COG4803	Predicted membrane protein
20091464	hypothetical protein MA2641	No	2			3271080	3271400	+		Methanosarcina_acetivorans_C2A_uid57879	NC_003552	chromosome									
20091465	hypothetical protein MA2642	No	1			3271551	3271973	+		Methanosarcina_acetivorans_C2A_uid57879	NC_003552	chromosome		arCOG01568	4 (-)	M	MscS	Small-conductance mechanosensitive channel	8E-19	pfam00924	Mechanosensitive ion channel. Two member
20091466	hypothetical protein MA2643	No	0			3272023	3272250	-		Methanosarcina_acetivorans_C2A_uid57879	NC_003552	chromosome									
20091467	hypothetical protein MA2644	No	2			3273086	3273601	+		Methanosarcina_acetivorans_C2A_uid57879	NC_003552	chromosome		arCOG05227	1	S		Predicted membrane protein	2E-46	COG4803	Predicted membrane protein
===									5	Methanosarcina_acetivorans_C2A_uid57879	NC_003552	chromosome									
20091477	hypothetical protein MA2654	No	0			3287813	3288196	-		Methanosarcina_acetivorans_C2A_uid57879	NC_003552	chromosome									
20091478	MuT/NUDIX protein	No	0			3288317	3288703	-		Methanosarcina_acetivorans_C2A_uid57879	NC_003552	chromosome		arCOG01080	1 (-)	L	MutT	NUDIX family hydrolase	0.0000002	pfam00293	NUDIX domain.
20091479	hypothetical protein MA2656	Yes	1			3289045	3290082	-		Methanosarcina_acetivorans_C2A_uid57879	NC_003552	chromosome	arCOG03624,a	arCOG03624	3	S		Uncharacterized conserved protein	8E-38	pfam05727	Uncharacterized protein family (UPF0228) ; Uncharacterized protein family (UPF0228)
20091480	hypothetical protein MA2657	No	9			3291281	3292435	+		Methanosarcina_acetivorans_C2A_uid57879	NC_003552	chromosome		arCOG05230	2	S		Uncharacterized conserved protein			
20091481	hypothetical protein MA2658	No	0			3292834	3293094	+		Methanosarcina_acetivorans_C2A_uid57879	NC_003552	chromosome		arCOG03632	3	S		Uncharacterized conserved protein			
===									6	Methanosarcina_acetivorans_C2A_uid57879	NC_003552	chromosome									
20091609	hypothetical protein MA2786	No	0			3473927	3474154	+		Methanosarcina_acetivorans_C2A_uid57879	NC_003552	chromosome		arCOG02139	1 (-)	L	InsB	Transposase and inactivated derivatives, IS1 fam	2E-15	COG1662	Transposase and inactivated derivatives,
20091610	hypothetical protein MA2787	No	0			3474258	3474545	+		Methanosarcina_acetivorans_C2A_uid57879	NC_003552	chromosome									
20091611	hypothetical protein MA2788	No	1			3474738	3475175	+		Methanosarcina_acetivorans_C2A_uid57879	NC_003552	chromosome	clust_239								
20091612	hypothetical protein MA2789	No	0			3475866	3476162	-		Methanosarcina_acetivorans_C2A_uid57879	NC_003552	chromosome									
20091613	hypothetical protein MA2790	No	0			3476230	3476481	+		Methanosarcina_acetivorans_C2A_uid57879	NC_003552	chromosome									
20091614	hypothetical protein MA2791	No	0			3476450	3476842	+		Methanosarcina_acetivorans_C2A_uid57879	NC_003552	chromosome		arCOG03744	1 (-)	O		Predicted aspartyl protease			
===									14	Methanosarcina_acetivorans_C2A_uid57879	NC_003552	chromosome									
20091623	hypothetical protein MA2801	No	0	YES		3496308	3496586	+		Methanosarcina_acetivorans_C2A_uid57879	NC_003552	chromosome									
20091624	hypothetical protein MA2802	No	0	YES		3496596	3496931	+		Methanosarcina_acetivorans_C2A_uid57879	NC_003552	chromosome									
20091625	hypothetical protein MA2803	Yes	0	YES		3497016	3497246	+		Methanosarcina_acetivorans_C2A_uid57879	NC_003552	chromosome									
20091626	hypothetical protein MA2804	No	0	YES		3497414	3498685	+		Methanosarcina_acetivorans_C2A_uid57879	NC_003552	chromosome		arCOG07665	6 (-)	S		Uncharacterized conserved protein			
20091627	hypothetical protein MA2805	No	1	YES		3498689	3499045	+		Methanosarcina_acetivorans_C2A_uid57879	NC_003552	chromosome									
20091628	hypothetical protein MA2806	No	7	YES		3499048	3500187	-		Methanosarcina_acetivorans_C2A_uid57879	NC_003552	chromosome		arCOG06798	2	S		Uncharacterized conserved protein			
20091629	hypothetical protein MA4678	No	0	YES		3500702	3500980	+		Methanosarcina_acetivorans_C2A_uid57879	NC_003552	chromosome									
20091630	hypothetical protein MA2807	No	0			3501180	3503174	+		Methanosarcina_acetivorans_C2A_uid57879	NC_003552	chromosome		arCOG02966	7 (-)	S		HEAT repeats containing protein	5E-22	COG1413	FOG: HEAT repeat

20091631	hypothetical protein MA4677	Yes	2			3503250	3503657	+		Methanosarcina_acetivorans_C2A_uid57879	NC_003552	chromosome									
20091632	hypothetical protein MA2808	Yes	2			3503976	3505892	+		Methanosarcina_acetivorans_C2A_uid57879	NC_003552	chromosome									
20091633	hypothetical protein MA2809	Yes	3			3505892	3506275	+		Methanosarcina_acetivorans_C2A_uid57879	NC_003552	chromosome									
20091634	hypothetical protein MA2810	No	1			3506272	3506892	+		Methanosarcina_acetivorans_C2A_uid57879	NC_003552	chromosome									
20091635	site-specific recombinase	No	0			3508611	3509579	+		Methanosarcina_acetivorans_C2A_uid57879	NC_003552	chromosome		arCOG01241	31 (-)	L	XerC	Integrase	2E-20	COG4974	Site-specific recombinase XerD
20091636	hypothetical protein MA2812	No	4			3509598	3510404	-		Methanosarcina_acetivorans_C2A_uid57879	NC_003552	chromosome		arCOG06799	2	S		Uncharacterized conserved protein			
===									5	Methanosarcina_acetivorans_C2A_uid57879	NC_003552	chromosome									
20091747	hypothetical protein MA2928	No	0			3656077	3657363	-		Methanosarcina_acetivorans_C2A_uid57879	NC_003552	chromosome	clust_2								
20091748	hypothetical protein MA2929	Yes	1			3657750	3658106	-		Methanosarcina_acetivorans_C2A_uid57879	NC_003552	chromosome									
20091749	hypothetical protein MA2930	No	0			3658443	3658919	-		Methanosarcina_acetivorans_C2A_uid57879	NC_003552	chromosome									
20091750	transposase	No	0			3658991	3659908	+		Methanosarcina_acetivorans_C2A_uid57879	NC_003552	chromosome		arCOG02125	5 (-)	L	Tra5	Transposase	1E-19	COG2801	Transposase and inactivated derivatives
20091751	hypothetical protein MA2932	No	0			3659898	3660155	+		Methanosarcina_acetivorans_C2A_uid57879	NC_003552	chromosome									
===									7	Methanosarcina_acetivorans_C2A_uid57879	NC_003552	chromosome									
20091791	hypothetical protein MA2973	No	0			3709189	3709377	-		Methanosarcina_acetivorans_C2A_uid57879	NC_003552	chromosome		arCOG03597	4 (-)	S		Uncharacterized conserved protein	2E-16	pfam10967	Protein of unknown function (DUF2769). T
20091792	hypothetical protein MA2974	No	1			3709748	3710200	-		Methanosarcina_acetivorans_C2A_uid57879	NC_003552	chromosome									
20091793	hypothetical protein MA2975	No	0			3710459	3711307	+		Methanosarcina_acetivorans_C2A_uid57879	NC_003552	chromosome		arCOG03538	4	S		Uncharacterized conserved protein			
20091794	hypothetical protein MA2976	No	0			3711459	3712295	+		Methanosarcina_acetivorans_C2A_uid57879	NC_003552	chromosome		arCOG03539	3	S		Uncharacterized conserved protein			
20091795	hypothetical protein MA2977	No	0			3712309	3712716	-		Methanosarcina_acetivorans_C2A_uid57879	NC_003552	chromosome									
20091796	hypothetical protein MA2978	No	0			3713230	3713490	+		Methanosarcina_acetivorans_C2A_uid57879	NC_003552	chromosome		arCOG02974	4	S		HEAT repeats containing protein			
20091797	cell surface protein	No	3			3713764	3715458	-		Methanosarcina_acetivorans_C2A_uid57879	NC_003552	chromosome		arCOG02497	1 (-)	S		S-layer protein	1E-29	pfam05048	Periplasmic copper-binding protein (NosD
===									5	Methanosarcina_acetivorans_C2A_uid57879	NC_003552	chromosome									
20091799	hypothetical protein MA2981	Yes	1			3716220	3716630	-		Methanosarcina_acetivorans_C2A_uid57879	NC_003552	chromosome		arCOG07667	1	S		Uncharacterized conserved protein			
20091800	hypothetical protein MA2982	No	0			3717611	3718567	+		Methanosarcina_acetivorans_C2A_uid57879	NC_003552	chromosome		arCOG03536	3	S		Uncharacterized conserved protein			
20091801	transposase	No	0			3718775	3719692	+		Methanosarcina_acetivorans_C2A_uid57879	NC_003552	chromosome		arCOG02125	5 (-)	L	Tra5	Transposase	1E-19	COG2801	Transposase and inactivated derivatives
20091802	hypothetical protein MA2984	No	0			3720050	3720649	+		Methanosarcina_acetivorans_C2A_uid57879	NC_003552	chromosome		arCOG03538	4	S		Uncharacterized conserved protein			
20091803	hypothetical protein MA2985	No	0			3721618	3722352	+		Methanosarcina_acetivorans_C2A_uid57879	NC_003552	chromosome		arCOG03540	2	S		Uncharacterized conserved protein			
===									6	Methanosarcina_acetivorans_C2A_uid57879	NC_003552	chromosome									
20091923	hypothetical protein MA3105	No	0			3861459	3861746	+		Methanosarcina_acetivorans_C2A_uid57879	NC_003552	chromosome		arCOG03592	2	S		Uncharacterized conserved protein	0.000005	pfam11387	Protein of unknown function (DUF2795). T
20091924	hypothetical protein MA3106	No	1			3861888	3862112	+		Methanosarcina_acetivorans_C2A_uid57879	NC_003552	chromosome									
20091925	hypothetical protein MA3107	No	0			3862275	3862517	+		Methanosarcina_acetivorans_C2A_uid57879	NC_003552	chromosome		arCOG03592	2	S		Uncharacterized conserved protein	0.000002	pfam11387	Protein of unknown function (DUF2795). T
20091926	glutathione-regulated potassium-efflux system	No	10			3862878	3864863	-		Methanosarcina_acetivorans_C2A_uid57879	NC_003552	chromosome		arCOG01955	3 (-)	P	KefB	Kef-type K+ transport system, membrane compo	2E-70	COG0475	Kef-type K+ transport systems, membrane ; Kef-type K+ transport systems, predicted ; TrkA-C domain. This domain is often foun
20091927	hypothetical protein MA3109	No	0			3865215	3866846	-		Methanosarcina_acetivorans_C2A_uid57879	NC_003552	chromosome	clust_71								
20091928	hypothetical protein MA3110	No	1			3867455	3867709	-		Methanosarcina_acetivorans_C2A_uid57879	NC_003552	chromosome									
===									5	Methanosarcina_acetivorans_C2A_uid57879	NC_003552	chromosome									
20091933	hypothetical protein MA3115	No	0			3869747	3871033	+		Methanosarcina_acetivorans_C2A_uid57879	NC_003552	chromosome	clust_2								
20091934	hypothetical protein MA3116	Yes	0			3871060	3871437	-		Methanosarcina_acetivorans_C2A_uid57879	NC_003552	chromosome									
20091935	hypothetical protein MA3117	Yes	1			3871961	3872506	+		Methanosarcina_acetivorans_C2A_uid57879	NC_003552	chromosome		arCOG03623	1	S		Uncharacterized conserved protein	3E-38	pfam05727	Uncharacterized protein family (UPF0228)
20091936	surface antigen gene	Yes	1			3872784	3873818	+		Methanosarcina_acetivorans_C2A_uid57879	NC_003552	chromosome		arCOG02505	1 (-)	S		beta-propeller repeat protein	4E-49	COG3391	Uncharacterized conserved protein
20091937	hypothetical protein MA3119	Yes	1			3874125	3874814	+		Methanosarcina_acetivorans_C2A_uid57879	NC_003552	chromosome		arCOG03624	3	S		Uncharacterized conserved protein	4E-44	pfam05727	Uncharacterized protein family (UPF0228) ; Schizosaccharomyces pombe repeat of unkn
===									7	Methanosarcina_acetivorans_C2A_uid57879	NC_003552	chromosome									
20092025	hypothetical protein MA3209	No	0			3973421	3973750	+		Methanosarcina_acetivorans_C2A_uid57879	NC_003552	chromosome		arCOG03277	1	S		Uncharacterized conserved protein			
20092026	hypothetical protein MA3210	No	0			3973902	3975188	-		Methanosarcina_acetivorans_C2A_uid57879	NC_003552	chromosome	clust_2								
20092027	hypothetical protein MA3211	No	0			3975606	3976013	-		Methanosarcina_acetivorans_C2A_uid57879	NC_003552	chromosome		arCOG03355	2	S		Uncharacterized conserved protein	3E-67	pfam08004	Protein of unknown function (DUF1699). T
20092028	thioredoxin	No	0			3976647	3976928	-		Methanosarcina_acetivorans_C2A_uid57879	NC_003552	chromosome		arCOG01972	5 (-)	O	TrxA	Thiol-disulfide isomerase or thioredoxin	5E-20	COG0526	Thiol-disulfide isomerase and thioredoxi
20092029	hypothetical protein MA3213	No	0			3976977	3977342	-		Methanosarcina_acetivorans_C2A_uid57879	NC_003552	chromosome									
20092030	hypothetical protein MA3214	No	0			3978321	3979349	+		Methanosarcina_acetivorans_C2A_uid57879	NC_003552	chromosome							7E-10	pfam05572	Pregnancy-associated plasma protein-A. P
20092031	hypothetical protein MA3215	No	0			3979615	3980037	-		Methanosarcina_acetivorans_C2A_uid57879	NC_003552	chromosome									
===									14	Methanosarcina_acetivorans_C2A_uid57879	NC_003552	chromosome									
20092038	hypothetical protein MA3222	No	0			3985691	3986125	-		Methanosarcina_acetivorans_C2A_uid57879	NC_003552	chromosome		arCOG02132	5 (-)	L		Transposase	2E-22	COG3677	Transposase and inactivated derivatives
20092039	hypothetical protein MA3223	No	0			3986142	3986876	-		Methanosarcina_acetivorans_C2A_uid57879	NC_003552	chromosome									
20092040	hypothetical protein MA3224	No	0			3986878	3987381	-		Methanosarcina_acetivorans_C2A_uid57879	NC_003552	chromosome									
20092041	hypothetical protein MA3225	No	3			3987956	3988303	+		Methanosarcina_acetivorans_C2A_uid57879	NC_003552	chromosome									
20092042	hypothetical protein MA3226	No	0			3988317	3988781	+		Methanosarcina_acetivorans_C2A_uid57879	NC_003552	chromosome									
20092043	hypothetical protein MA3227	No	2			3988783	3989640	+		Methanosarcina_acetivorans_C2A_uid57879	NC_003552	chromosome									
20092044	hypothetical protein MA3228	No	2			3989650	3989940	+		Methanosarcina_acetivorans_C2A_uid57879	NC_003552	chromosome									
20092045	cell surface protein	Yes	4			3989937	3992813	+		Methanosarcina_acetivorans_C2A_uid57879	NC_003552	chromosome		arCOG06836	1 (-)	R		PKD repeats containing protein	6E-48	COG3291	FOG: PKD repeat
20092046	hypothetical protein MA3230	No	3			3992915	3993355	+		Methanosarcina_acetivorans_C2A_uid57879	NC_003552	chromosome									
20092047	hypothetical protein MA3231	No	0			3993588	3994694	+		Methanosarcina_acetivorans_C2A_uid57879	NC_003552	chromosome									
20092048	hypothetical protein MA3232	No	0			3994761	3996173	+		Methanosarcina_acetivorans_C2A_uid57879	NC_003552	chromosome									
20092049	hypothetical protein MA3233	Yes	3			3996183	3996563	+		Methanosarcina_acetivorans_C2A_uid57879	NC_003552	chromosome									
20092050	hypothetical protein MA3234	No	2			3996713	3996961	+		Methanosarcina_acetivorans_C2A_uid57879	NC_003552	chromosome	clust_397								
20092051	hypothetical protein MA3235	No	0			3997412	3997690	-		Methanosarcina_acetivorans_C2A_uid57879	NC_003552	chromosome		arCOG04941	1 (-)	T		ACT domain-containing protein	6E-31	COG3830	ACT domain-containing protein
===									6	Methanosarcina_acetivorans_C2A_uid57879	NC_003552	chromosome									
20092088	hypothetical protein MA3272	No	3			4038330	4038740	-		Methanosarcina_acetivorans_C2A_uid57879	NC_003552	chromosome		arCOG02485	4	S		WD40-like repeat			
20092089	hypothetical protein MA3274	No	0			4039700	4040038	-		Methanosarcina_acetivorans_C2A_uid57879	NC_003552	chromosome		arCOG02485	4	S		WD40-like repeat			
20092090	hypothetical protein MA3275	Yes	2			4040221	4041528	-		Methanosarcina_acetivorans_C2A_uid57879	NC_003552	chromosome		arCOG03375	2 (-)	S		HEAT repeats containing protein	7E-29	COG1413	FOG: HEAT repeat
20092091	hypothetical protein MA3276	No	1			4041577	4042077	-		Methanosarcina_acetivorans_C2A_uid57879	NC_003552	chromosome		arCOG07668	2	S		Uncharacterized conserved protein			
20092092	hypothetical protein MA3277	Yes	1			4042338	4042802	+		Methanosarcina_acetivorans_C2A_uid57879	NC_003552	chromosome		arCOG03997	2	S		Uncharacterized conserved protein			
20092093	hypothetical protein MA3278	No	4			4042885	4043472	-		Methanosarcina_acetivorans_C2A_uid57879	NC_003552	chromosome		arCOG06805	1	S		Uncharacterized conserved protein	0.00004	pfam13197	Protein of unknown function (DUF4013). T
===									6	Methanosarcina_acetivorans_C2A_uid57879	NC_003552	chromosome									
20092197	hypothetical protein MA3383	No	0			4182760	4183080	-		Methanosarcina_acetivorans_C2A_uid57879	NC_003552	chromosome		arCOG02287	2 (-)	S		Uncharacterized beta/alpha-propeller protein, like	3E-30	COG0393	Uncharacterized conserved protein
20092198	hypothetical protein MA3384	No	2			4183089	4183310	-		Methanosarcina_acetivorans_C2A_uid57879	NC_003552	chromosome									
20092199	hypothetical protein MA3385	No	0			4183734	4184420	+		Methanosarcina_acetivorans_C2A_uid57879	NC_003552	chromosome									
20092200	hypothetical protein MA3386	No	0			4184814	4185341	+		Methanosarcina_acetivorans_C2A_uid57879	NC_003552	chromosome									
20092201	hypothetical protein MA3387	No	0			4185724	4186977	-		Methanosarcina_acetivorans_C2A_uid57879	NC_003552	chromosome		arCOG05250	6	S		Uncharacterized conserved protein			
20092202	serine protease inhibitor	Yes	0			4187242	4188516	+		Methanosarcina_acetivorans_C2A_uid57879	NC_003552	chromosome		arCOG04933	4 (-)	O		Serine protease inhibitor		COG4826	Serine protease inhibitor
===									7	Methanosarcina_acetivorans_C2A_uid57879	NC_003552	chromosome									
20092447	hypothetical protein MA3647	No	0	YES		4497021	4497437	-		Methanosarcina_acetivorans_C2A_uid57879	NC_003552	chromosome		arCOG01157	1 (-)	R		Metallophosphoesterase superfamily enzyme	1E-16	COG1408	Predicted phosphohydrolases
20092448	hypothetical protein MA3648	No	1	YES		4497496	4497813	-		Methanosarcina_acetivorans_C2A_uid57879	NC_003552	chromosome									
20092449	hypothetical protein MA3649	No	3	YES		4500355	4500684	+		Methanosarcina_acetivorans_C2A_uid57879	NC_003552	chromosome									
20092450	hypothetical protein MA3650	No	0	YES		4501637	4502437	-		Methanosarcina_acetivorans_C2A_uid57879	NC_003552	chromosome									
20092451	hypothetical protein MA3651	No	0	YES		4502721	4503044	+		Methanosarcina_acetivorans_C2A_uid57879	NC_003552	chromosome									
20092452	amidinotransferase	No	0			4503602	4504606	-		Methanosarcina_acetivorans_C2A_uid57879	NC_003552	chromosome		arCOG03109	1 (-)	E		N-Dimethylarginine dimethylaminohydrolase	4E-25	COG1834	N-Dimethylarginine dimethylaminohydrolas
20092453	hypothetical protein MA3653	Yes	0			4504737	4505186	+		Methanosarcina_acetivorans_C2A_uid57879	NC_003552	chromosome									
===									39	Methanosarcina_acetivorans_C2A_uid57879	NC_003552	chromosome									
20092597	hypothetical protein MA3801	No	0		Virus_Caudovi	4688183	4688680	-		Methanosarcina_acetivorans_C2A_uid57879	NC_003552	chromosome		arCOG03511	3 (-)	S		Uncharacterized conserved protein	0.00000002	pfam10102	Domain of unknown function (DUF2341). Me
20092598	hypothetical protein MA3802	No	0		Virus_Caudovi	4689069	4689704	-		Methanosarcina_acetivorans_C2A_uid57879	NC_003552	chromosome									
20092599	hypothetical protein MA3803	No	0		Virus_Caudovi	4689706	4690077	-		Methanosarcina_acetivorans_C2A_uid57879	NC_003552	chromosome									
20092600	hypothetical protein MA3804	No	0		Virus_Caudovi	4690165	4690506	-		Methanosarcina_acetivorans_C2A_uid57879	NC_003552	chromosome									
20092601	hypothetical protein MA3805	No	3		Virus_Caudovi	4690506	4692983	-		Methanosarcina_acetivorans_C2A_uid57879	NC_003552	chromosome		arCOG11109	8	Y		Phage tail tape measure protein, TP901 family	4E-94	COG5280	Phage-related minor tail protein
20092602	hypothetical protein MA3806	No	0		Virus_Caudovi	4693155	4693391	-		Methanosarcina_acetivorans_C2A_uid57879	NC_003552	chromosome									
20092603	hypothetical protein MA3807	No	0		Virus_Caudovi	4693398	4693823	+		Methanosarcina_acetivorans_C2A_uid57879	NC_003552	chromosome									
20092604	hypothetical protein MA3808	No	0		Virus_Caudovi	4693789	4694574	-		Methanosarcina_acetivorans_C2A_uid57879	NC_003552	chromosome	clust_125								
20092605	hypothetical protein MA3809	No	0		Virus_Caudovi	4694599	4694973	-		Methanosarcina_acetivorans_C2A_uid57879	NC_003552	chromosome									
20092606	hypothetical protein MA3810	No	0		Virus_Caudovi	4694970	4695401	-		Methanosarcina_acetivorans_C2A_uid57879	NC_003552	chromosome									
20092607	hypothetical protein MA3811	No	1		Virus_Caudovi	4695402	4695680	-		Methanosarcina_acetivorans_C2A_uid57879	NC_003552	chromosome									
20092608	hypothetical protein MA3812	No	0		Virus_Caudovi	4695795	4696523	-		Methanosarcina_acetivorans_C2A_uid57879	NC_003552	chromosome									
20092609	hypothetical protein MA3813	No	0		Virus_Caudovi	4696520	4697140	-		Methanosarcina_acetivorans_C2A_uid57879	NC_003552	chromosome									
20092610	hypothetical protein MA3814	No	0		Virus_Caudovi	4697147	4697539	-		Methanosarcina_acetivorans_C2A_uid57879	NC_003552	chromosome									
20092611	hypothetical protein MA3815	No	0		Virus_Caudovi	4697536	4697799	-		Methanosarcina_acetivorans_C2A_uid57879	NC_003552	chromosome									
20092612	hypothetical protein MA3816	No	0		Virus_Caudovi	4697810	4698718	-		Methanosarcina_acetivorans_C2A_uid57879	NC_003552	chromosome									
20092613	hypothetical protein MA3817	No	0		Virus_Caudovi	4698730	4699191	-		Methanosarcina_acetivorans_C2A_uid57879	NC_003552	chromosome									
20092614	hypothetical protein MA3818	No	0		Virus_Caudovi	4699176	4699799	-		Methanosarcina_acetivorans_C2A_uid57879	NC_003552	chromosome									
20092615	hypothetical protein MA3819	No	0		Virus_Caudovi	4699820	4700152	-		Methanosarcina_acetivorans_C2A_uid57879	NC_003552	chromosome									
20092616	hypothetical protein MA3820	No	0		Virus_Caudovi	4700665	4702293	+		Methanosarcina_acetivorans_C2A_uid57879	NC_003552	chromosome		arCOG03470	2 (-)	L		Transposase		COG5421	Transposase
20092617	hypothetical protein MA3821	No	5		Virus_Caudovi	4702453	4703355	+		Methanosarcina_acetivorans_C2A_uid57879	NC_003552	chromosome		arCOG06798	2	S		Uncharacterized conserved protein			
20092618	hypothetical protein MA3822	No	0		Virus_Caudovi	4703494	4703754	-		Methanosarcina_acetivorans_C2A_uid57879	NC_003552	chromosome									
20092619	hypothetical protein MA3823	No	0		Virus_Caudovi	4703860	4705266	-		Methanosarcina_acetivorans_C2A_uid57879	NC_003552	chromosome		arCOG09510	5 (-)	T		NAD+--asparagine ADP-ribosyltransferase	1E-25	COG5585	NAD+--asparagine ADP-ribosyltransferase
20092620	hypothetical protein MA3824	No	0		Virus_Caudovi	4705435	4706667	-		Methanosarcina_acetivorans_C2A_uid57879	NC_003552	chromosome									
20092621	hypothetical protein MA3825	No	1		Virus_Caudovi	4706673	4707002	-		Methanosarcina_acetivorans_C2A_uid57879	NC_003552	chromosome									
20092622	terminase	No	0		Virus_Caudovi	4707089	4708570	-		Methanosarcina_acetivorans_C2A_uid57879	NC_003552	chromosome		arCOG09550	17 (-)	Y	TerL	Phage terminase large subunit	5E-39	COG5362	Phage-related terminase
20092623	hypothetical protein MA3827	No	0		Virus_Caudovi	4708583	4708804	-		Methanosarcina_acetivorans_C2A_uid57879	NC_003552	chromosome									
20092624	hypothetical protein MA3828	No	0		Virus_Caudovi	4708831	4709313	-		Methanosarcina_acetivorans_C2A_uid57879	NC_003552	chromosome		arCOG11382	5	Y		Phage terminase small subunit			
20092625	Mcm protein	No	0		Virus_Caudovi	4709398	4711506	-		Methanosarcina_acetivorans_C2A_uid57879	NC_003552	chromosome		arCOG00439	14 (-)	L	MCM2	Predicted ATPase involved in replication control,	3E-180	COG1241	Predicted ATPase involved in replication
20092626	hypothetical protein MA3830	No	0		Virus_Caudovi	4711499	4711891	-		Methanosarcina_acetivorans_C2A_uid57879	NC_003552	chromosome									
20092627	hypothetical protein MA3831	No	0		Virus_Caudovi	4712231	4712575	-		Methanosarcina_acetivorans_C2A_uid57879	NC_003552	chromosome									
20092628	hypothetical protein MA3832	No	0		Virus_Caudovi	4712576	4712983	-		Methanosarcina_acetivorans_C2A_uid57879	NC_003552	chromosome		arCOG06787	4 (-)	L		PD-(D/E)XK superfamily nuclease			
20092629	hypothetical protein MA3833	No	0		Virus_Caudovi	4713083	4713646	-		Methanosarcina_acetivorans_C2A_uid57879	NC_003552	chromosome									
20092630	hypothetical protein MA3834	No	0		Virus_Caudovi	4713762	4714460	-		Methanosarcina_acetivorans_C2A_uid57879	NC_003552	chromosome									
20092631	hypothetical protein MA3835	No	0		Virus_Caudovi	4714457	4715062	-		Methanosarcina_acetivorans_C2A_uid57879	NC_003552	chromosome									
20092632	hypothetical protein MA3836	No	1		Virus_Caudovi	4715265	4715927	+		Methanosarcina_acetivorans_C2A_uid57879	NC_003552	chromosome									
20092633	hypothetical protein MA3837	No	0		Virus_Caudovi	4716474	4716686	-		Methanosarcina_acetivorans_C2A_uid57879	NC_003552	chromosome									
20092634	hypothetical protein MA3838	Yes	0		Virus_Caudovi	4716847	4717701	+		Methanosarcina_acetivorans_C2A_uid57879	NC_003552	chromosome									
20092635	hypothetical protein MA3839	No	0		Virus_Caudovi	4718197	4718607	+		Methanosarcina_acetivorans_C2A_uid57879	NC_003552	chromosome		arCOG03032	12 (-)	R	NrfG	TPR repeats containing protein	0.000008	COG0457	FOG: TPR repeat
===									6	Methanosarcina_acetivorans_C2A_uid57879	NC_003552	chromosome									
20092920	site-specific recombinase	No	0	YES		5044148	5045044	-		Methanosarcina_acetivorans_C2A_uid57879	NC_003552	chromosome		arCOG01241	31 (-)	L	XerC	Integrase	1E-16	COG4974	Site-specific recombinase XerD
20092921	hypothetical protein MA4128	No	2	YES		5045217	5045465	-		Methanosarcina_acetivorans_C2A_uid57879	NC_003552	chromosome	clust_397								
20092922	hypothetical protein MA4129	No	1	YES		5046162	5046599	+		Methanosarcina_acetivorans_C2A_uid57879	NC_003552	chromosome	clust_239								
20092923	hypothetical protein MA4130	No	0	YES		5046568	5046858	-		Methanosarcina_acetivorans_C2A_uid57879	NC_003552	chromosome									
20092924	hypothetical protein MA4131	No	0	YES		5047389	5048819	+		Methanosarcina_acetivorans_C2A_uid57879	NC_003552	chromosome									
20092925	transposase	No	0	YES		5049499	5050743	+		Methanosarcina_acetivorans_C2A_uid57879	NC_003552	chromosome		arCOG02126	1 (-)	L		Transposase	5E-71	COG4584	Transposase and inactivated derivatives
===									11	Methanosarcina_acetivorans_C2A_uid57879	NC_003552	chromosome									
20092927	hypothetical protein MA4134	No	0	YES		5051833	5052066	+		Methanosarcina_acetivorans_C2A_uid57879	NC_003552	chromosome									
20092928	hypothetical protein MA4135	No	0	YES		5053011	5054297	+		Methanosarcina_acetivorans_C2A_uid57879	NC_003552	chromosome	clust_2								
20092929	hypothetical protein MA4136	No	1	YES		5054324	5054731	-		Methanosarcina_acetivorans_C2A_uid57879	NC_003552	chromosome									
20092930	hypothetical protein MA4137	No	0	YES		5054882	5056855	-		Methanosarcina_acetivorans_C2A_uid57879	NC_003552	chromosome		arCOG07652	5 (-)	L		Superfamily II helicase or inactivated derivatives	2E-68	COG5519	Superfamily II helicase and inactivated
20092931	hypothetical protein MA4138	No	0	YES		5057025	5057351	-		Methanosarcina_acetivorans_C2A_uid57879	NC_003552	chromosome									
20092932	hypothetical protein MA4139	No	0	YES		5057437	5058006	-		Methanosarcina_acetivorans_C2A_uid57879	NC_003552	chromosome		arCOG06787	4 (-)	L		PD-(D/E)XK superfamily nuclease			
20092933	hypothetical protein MA4140	No	0	YES		5058003	5058491	-		Methanosarcina_acetivorans_C2A_uid57879	NC_003552	chromosome		arCOG07656	3	S		Uncharacterized conserved protein			
20092934	hypothetical protein MA4141	No	3	YES		5058488	5058892	-		Methanosarcina_acetivorans_C2A_uid57879	NC_003552	chromosome									
20092935	hypothetical protein MA4142	No	0	YES		5059128	5059394	-		Methanosarcina_acetivorans_C2A_uid57879	NC_003552	chromosome		arCOG10941	4	S		Uncharacterized conserved protein	0.0001	COG1997	Ribosomal protein L37AE/L43A
20092936	hypothetical protein MA4143	No	0	YES		5059391	5059810	-		Methanosarcina_acetivorans_C2A_uid57879	NC_003552	chromosome									
20092937	ADP-ribosylglycohydrolase (dinitrogenase red	No	0			5060572	5061516	+		Methanosarcina_acetivorans_C2A_uid57879	NC_003552	chromosome		arCOG04448	2 (-)	O	DraG	ADP-ribosylglycohydrolase	3E-55	COG1397	ADP-ribosylglycohydrolase
===									5	Methanosarcina_acetivorans_C2A_uid57879	NC_003552	chromosome									
20093225	hypothetical protein MA4439	No	0			5482610	5482819	+		Methanosarcina_acetivorans_C2A_uid57879	NC_003552	chromosome									
20093226	polysaccharide deacetylase	No	0			5483251	5484156	-		Methanosarcina_acetivorans_C2A_uid57879	NC_003552	chromosome		arCOG02876	1 (-)	G	CDA1	Predicted xylanase/chitin deacetylase	1E-11	COG0726	Predicted xylanase/chitin deacetylase
20093227	hypothetical protein MA4441	No	0			5484479	5484796	-		Methanosarcina_acetivorans_C2A_uid57879	NC_003552	chromosome									
20093228	hypothetical protein MA4442	Yes	1			5486695	5488488	+		Methanosarcina_acetivorans_C2A_uid57879	NC_003552	chromosome	arCOG03500,a	arCOG03500	3	S		Cell suface protein	6E-89	pfam06848	Disaggregatase related repeat. This fami ; Mucin-like glycoprotein. This family of ; Pectate lyase superfamily protein. This
20093229	hypothetical protein MA4443	No	0			5488936	5489211	-		Methanosarcina_acetivorans_C2A_uid57879	NC_003552	chromosome									
===									16	Methanosarcina_acetivorans_C2A_uid57879	NC_003552	chromosome									
20093270	hypothetical protein MA4485	No	0	YES		5533711	5534166	+		Methanosarcina_acetivorans_C2A_uid57879	NC_003552	chromosome		arCOG04915	5 (-)	S		Uncharacterized conserved protein			
20093271	hypothetical protein MA4486	No	4	YES		5534201	5534809	+		Methanosarcina_acetivorans_C2A_uid57879	NC_003552	chromosome		arCOG03255	2	S		Uncharacterized conserved protein	9E-28	pfam07895	Protein of unknown function (DUF1673). T
20093272	hypothetical protein MA4487	No	5	YES		5534806	5535525	+		Methanosarcina_acetivorans_C2A_uid57879	NC_003552	chromosome		arCOG03254	3	S		Uncharacterized conserved protein	4E-33	pfam07895	Protein of unknown function (DUF1673). T
20093273	hypothetical protein MA4488	No	4	YES		5535558	5536145	+		Methanosarcina_acetivorans_C2A_uid57879	NC_003552	chromosome		arCOG03252	4	S		Uncharacterized conserved protein	1E-45	pfam07895	Protein of unknown function (DUF1673). T
20093274	hypothetical protein MA4489	No	4	YES		5536175	5536747	+		Methanosarcina_acetivorans_C2A_uid57879	NC_003552	chromosome		arCOG03250	2	S		Uncharacterized conserved protein	7E-55	pfam07895	Protein of unknown function (DUF1673). T
20093275	hypothetical protein MA4490	No	4	YES		5536748	5537377	+		Methanosarcina_acetivorans_C2A_uid57879	NC_003552	chromosome		arCOG03252	4	S		Uncharacterized conserved protein	5E-48	pfam07895	Protein of unknown function (DUF1673). T
20093276	hypothetical protein MA4491	No	4	YES		5537407	5538006	+		Methanosarcina_acetivorans_C2A_uid57879	NC_003552	chromosome		arCOG03253	4	S		Uncharacterized conserved protein	5E-49	pfam07895	Protein of unknown function (DUF1673). T
20093277	hypothetical protein MA4492	No	4	YES		5538003	5538614	+		Methanosarcina_acetivorans_C2A_uid57879	NC_003552	chromosome		arCOG03249	4 (-)	S		Uncharacterized conserved protein	8E-61	pfam07895	Protein of unknown function (DUF1673). T
20093278	hypothetical protein MA4493	No	4	YES		5538615	5539238	+		Methanosarcina_acetivorans_C2A_uid57879	NC_003552	chromosome		arCOG03251	3	S		Uncharacterized conserved protein	6E-67	pfam07895	Protein of unknown function (DUF1673). T
20093279	hypothetical protein MA4494	No	3	YES		5539239	5539865	+		Methanosarcina_acetivorans_C2A_uid57879	NC_003552	chromosome		arCOG03249	4 (-)	S		Uncharacterized conserved protein	2E-65	pfam07895	Protein of unknown function (DUF1673). T
20093280	hypothetical protein MA4495	No	1	YES		5539805	5540152	-		Methanosarcina_acetivorans_C2A_uid57879	NC_003552	chromosome									
20093281	hypothetical protein MA4496	No	2	YES		5540142	5540600	+		Methanosarcina_acetivorans_C2A_uid57879	NC_003552	chromosome		arCOG06845	3	S		Uncharacterized conserved protein			
20093282	hypothetical protein MA4497	No	4	YES		5540602	5541339	+		Methanosarcina_acetivorans_C2A_uid57879	NC_003552	chromosome		arCOG06827	7	S		Uncharacterized conserved protein			
20093283	hypothetical protein MA4498	No	4	YES		5541481	5542242	+		Methanosarcina_acetivorans_C2A_uid57879	NC_003552	chromosome		arCOG06827	7	S		Uncharacterized conserved protein	0.00008	pfam07895	Protein of unknown function (DUF1673). T
20093284	hypothetical protein MA4499	No	4	YES		5542444	5543094	+		Methanosarcina_acetivorans_C2A_uid57879	NC_003552	chromosome		arCOG06827	7	S		Uncharacterized conserved protein			
20093285	hypothetical protein MA4500	No	0	YES		5543178	5543651	-		Methanosarcina_acetivorans_C2A_uid57879	NC_003552	chromosome		arCOG03097	4 (-)	S		Uncharacterized conserved protein	0.000001	COG0419	ATPase involved in DNA repair
===									5	Methanosarcina_barkeri_Fusaro_uid57715	NC_007355	chromosome									
73667771	hypothetical protein Mbar_A0222	No	0	YES		262451	262960	+		Methanosarcina_barkeri_Fusaro_uid57715	NC_007355	chromosome									
73667772	hypothetical protein Mbar_A0223	No	0	YES		263361	263627	-		Methanosarcina_barkeri_Fusaro_uid57715	NC_007355	chromosome		arCOG01665	6 (-)	V	RelE	Cytotoxic translational repressor of toxin-antitoxin	0.00003	COG2026	Cytotoxic translational repressor of tox
73667773	hypothetical protein Mbar_A0224	No	1	YES		263617	263901	-		Methanosarcina_barkeri_Fusaro_uid57715	NC_007355	chromosome									
73667774	hypothetical protein Mbar_A0225	No	0	YES		264264	265067	+		Methanosarcina_barkeri_Fusaro_uid57715	NC_007355	chromosome	clust_123								
73667775	hypothetical protein Mbar_A0226	No	0	YES		265126	265926	+		Methanosarcina_barkeri_Fusaro_uid57715	NC_007355	chromosome	clust_123								
===									5	Methanosarcina_barkeri_Fusaro_uid57715	NC_007355	chromosome									
73667903	hypothetical protein Mbar_A0354	No	0			412196	412624	-		Methanosarcina_barkeri_Fusaro_uid57715	NC_007355	chromosome		arCOG05250	6	S		Uncharacterized conserved protein			
73667904	transposase	No	1			413474	415111	-		Methanosarcina_barkeri_Fusaro_uid57715	NC_007355	chromosome		arCOG03470	2 (-)	L		Transposase	2E-171	COG5421	Transposase
73667905	hypothetical protein Mbar_A0356	Yes	1			415576	415908	+		Methanosarcina_barkeri_Fusaro_uid57715	NC_007355	chromosome									
73667906	hypothetical protein Mbar_A0357	No	0			416338	416526	-		Methanosarcina_barkeri_Fusaro_uid57715	NC_007355	chromosome									
73667907	hypothetical protein Mbar_A0358	No	0			416848	417210	+		Methanosarcina_barkeri_Fusaro_uid57715	NC_007355	chromosome		arCOG06808	1	S		Uncharacterized conserved protein			
===									6	Methanosarcina_barkeri_Fusaro_uid57715	NC_007355	chromosome									
73668656	transporter	No	12			1380116	1381582	-		Methanosarcina_barkeri_Fusaro_uid57715	NC_007355	chromosome		arCOG02209	4 (-)	R	RfbX	Polysaccharide biosynthesis protein, Mvin family	2E-40	COG2244	Membrane protein involved in the export
73668657	hypothetical protein Mbar_A1124	Yes	0			1382363	1384969	-		Methanosarcina_barkeri_Fusaro_uid57715	NC_007355	chromosome	arCOG03504,a	arCOG03504	2	S		Cell suface protein	1E-98	pfam08480	Disaggregatase related. This domain is f ; Disaggregatase related repeat. This fami ; Disaggregatase related repeat. This fami ; Right handed beta helix region. This reg
73668658	hypothetical protein Mbar_A1125	Yes	0			1386028	1388001	-		Methanosarcina_barkeri_Fusaro_uid57715	NC_007355	chromosome		arCOG03500	3	S		Cell suface protein	1E-95	pfam08480	Disaggregatase related. This domain is f
73668659	hypothetical protein Mbar_A1126	Yes	0			1389030	1391003	-		Methanosarcina_barkeri_Fusaro_uid57715	NC_007355	chromosome		arCOG03500	3	S		Cell suface protein	2E-96	pfam08480	Disaggregatase related. This domain is f
73668660	hypothetical protein Mbar_A1127	Yes	0			1391967	1393436	-		Methanosarcina_barkeri_Fusaro_uid57715	NC_007355	chromosome		arCOG03504	2	S		Cell suface protein	8E-94	pfam08480	Disaggregatase related. This domain is f
73668661	glycosyltransferase	No	0			1394436	1395620	-		Methanosarcina_barkeri_Fusaro_uid57715	NC_007355	chromosome		arCOG01410	1 (-)	M	RfaG	Glycosyltransferase	8E-32	COG0438	Glycosyltransferase
===									5	Methanosarcina_barkeri_Fusaro_uid57715	NC_007355	chromosome									
73668685	hypothetical protein Mbar_A1152	No	0			1424269	1424679	-		Methanosarcina_barkeri_Fusaro_uid57715	NC_007355	chromosome		arCOG04999	1	S		Uncharacterized conserved protein			
73668686	hypothetical protein Mbar_A1153	No	0			1424930	1425379	+		Methanosarcina_barkeri_Fusaro_uid57715	NC_007355	chromosome		arCOG04915	5 (-)	S		Uncharacterized conserved protein			
73668687	hypothetical protein Mbar_A1154	No	4			1425420	1426001	+		Methanosarcina_barkeri_Fusaro_uid57715	NC_007355	chromosome		arCOG03255	2	S		Uncharacterized conserved protein	9E-31	pfam07895	Protein of unknown function (DUF1673). T
73668688	hypothetical protein Mbar_A1155	No	5			1425998	1426717	+		Methanosarcina_barkeri_Fusaro_uid57715	NC_007355	chromosome		arCOG03254	3	S		Uncharacterized conserved protein	4E-24	pfam07895	Protein of unknown function (DUF1673). T
73668689	hypothetical protein Mbar_A1156	No	4			1426731	1427348	+		Methanosarcina_barkeri_Fusaro_uid57715	NC_007355	chromosome		arCOG03253	4	S		Uncharacterized conserved protein	5E-47	pfam07895	Protein of unknown function (DUF1673). T
===									8	Methanosarcina_barkeri_Fusaro_uid57715	NC_007355	chromosome									
73668692	hypothetical protein Mbar_A1159	No	4			1428553	1429179	+		Methanosarcina_barkeri_Fusaro_uid57715	NC_007355	chromosome		arCOG03253	4	S		Uncharacterized conserved protein	2E-46	pfam07895	Protein of unknown function (DUF1673). T
73668693	hypothetical protein Mbar_A1160	No	4			1429176	1429775	+		Methanosarcina_barkeri_Fusaro_uid57715	NC_007355	chromosome		arCOG03249	4 (-)	S		Uncharacterized conserved protein	3E-54	pfam07895	Protein of unknown function (DUF1673). T
73668694	hypothetical protein Mbar_A1161	No	4			1429772	1430377	+		Methanosarcina_barkeri_Fusaro_uid57715	NC_007355	chromosome		arCOG03250	2	S		Uncharacterized conserved protein	2E-56	pfam07895	Protein of unknown function (DUF1673). T
73668695	hypothetical protein Mbar_A1162	No	3			1430378	1431007	+		Methanosarcina_barkeri_Fusaro_uid57715	NC_007355	chromosome		arCOG03252	4	S		Uncharacterized conserved protein	2E-52	pfam07895	Protein of unknown function (DUF1673). T
73668696	hypothetical protein Mbar_A1163	No	4			1431004	1431603	+		Methanosarcina_barkeri_Fusaro_uid57715	NC_007355	chromosome		arCOG03251	3	S		Uncharacterized conserved protein	1E-60	pfam07895	Protein of unknown function (DUF1673). T
73668697	hypothetical protein Mbar_A1164	No	4			1431604	1432245	+		Methanosarcina_barkeri_Fusaro_uid57715	NC_007355	chromosome		arCOG06845	3	S		Uncharacterized conserved protein			
73668698	hypothetical protein Mbar_A1165	No	4			1432407	1433156	+		Methanosarcina_barkeri_Fusaro_uid57715	NC_007355	chromosome		arCOG06827	7	S		Uncharacterized conserved protein			
73668699	hypothetical protein Mbar_A1166	No	0			1433192	1433665	-		Methanosarcina_barkeri_Fusaro_uid57715	NC_007355	chromosome		arCOG03097	4 (-)	S		Uncharacterized conserved protein	0.000005	COG0466	ATP-dependent Lon protease, bacterial ty
===									6	Methanosarcina_barkeri_Fusaro_uid57715	NC_007355	chromosome									
73668963	hypothetical protein Mbar_A1440	Yes	1			1773978	1774253	+		Methanosarcina_barkeri_Fusaro_uid57715	NC_007355	chromosome		arCOG03316	7	S		Uncharacterized conserved protein			
73668964	hypothetical protein Mbar_A1441	No	0			1774419	1776275	+		Methanosarcina_barkeri_Fusaro_uid57715	NC_007355	chromosome		arCOG03316	7	S		Uncharacterized conserved protein		pfam11369	Protein of unknown function (DUF3160). T
73668965	hypothetical protein Mbar_A1442	No	1			1776457	1776993	+		Methanosarcina_barkeri_Fusaro_uid57715	NC_007355	chromosome		arCOG05151	4	S		Uncharacterized conserved protein			
73668966	hypothetical protein Mbar_A1443	No	4			1777264	1777716	+		Methanosarcina_barkeri_Fusaro_uid57715	NC_007355	chromosome		arCOG03975	2 (-)	S		Uncharacterized conserved protein			
73668967	hypothetical protein Mbar_A1444	No	5			1777722	1778204	+		Methanosarcina_barkeri_Fusaro_uid57715	NC_007355	chromosome		arCOG04951	2	S		Uncharacterized conserved protein			
73668968	hypothetical protein Mbar_A1445	Yes	0			1778902	1779408	+		Methanosarcina_barkeri_Fusaro_uid57715	NC_007355	chromosome		arCOG05151	4	S		Uncharacterized conserved protein			
===									5	Methanosarcina_barkeri_Fusaro_uid57715	NC_007355	chromosome									
73669276	hypothetical protein Mbar_A1770	Yes	7			2169828	2171405	-		Methanosarcina_barkeri_Fusaro_uid57715	NC_007355	chromosome		arCOG05162	3	S		Uncharacterized conserved protein			
73669277	cobaltochelatase subunit CobN	No	0			2171422	2175453	-		Methanosarcina_barkeri_Fusaro_uid57715	NC_007355	chromosome		arCOG03022	5 (-)	H	CobN	Cobalamin biosynthesis protein CobN or related	7E-136	pfam02514	CobN/Magnesium Chelatase. This family co ; CobN/Magnesium Chelatase. This family co
73669278	PQQ repeat-containing protein	Yes	0			2175440	2177158	-		Methanosarcina_barkeri_Fusaro_uid57715	NC_007355	chromosome		arCOG02489	6	S		WD40 repeats containing protein	0.0000005	COG2319	FOG: WD40 repeat
73669279	hypothetical protein Mbar_A1773	Yes	0			2177163	2178713	-		Methanosarcina_barkeri_Fusaro_uid57715	NC_007355	chromosome		arCOG02489	6	S		WD40 repeats containing protein			
73669280	hypothetical protein Mbar_A1774	No	1			2178743	2179324	-		Methanosarcina_barkeri_Fusaro_uid57715	NC_007355	chromosome		arCOG05163	3	S		Uncharacterized conserved protein			
===									5	Methanosarcina_barkeri_Fusaro_uid57715	NC_007355	chromosome									
73669320	hypothetical protein Mbar_A1814	Yes	1			2266007	2266396	-		Methanosarcina_barkeri_Fusaro_uid57715	NC_007355	chromosome		arCOG02563	1	S		PKD repeats containing protein	6E-18	COG3391	Uncharacterized conserved protein
73669321	hypothetical protein Mbar_A1815	Yes	1			2266923	2268776	-		Methanosarcina_barkeri_Fusaro_uid57715	NC_007355	chromosome	arCOG03268,a	arCOG03268	2	S		Cell suface protein	4E-21	pfam07752	Protein of unknown function (DUF1608). T ; Protein of unknown function (DUF1608). T ; Protein of unknown function (DUF1608). T
73669322	hypothetical protein Mbar_A1816	Yes	0			2269205	2270722	-		Methanosarcina_barkeri_Fusaro_uid57715	NC_007355	chromosome		arCOG03268	2	S		Cell suface protein	5E-18	pfam07752	Protein of unknown function (DUF1608). T ; Protein of unknown function (DUF1608). T
73669323	phycocyanin alpha phycocyanobilin lyase	Yes	1			2270807	2272288	-		Methanosarcina_barkeri_Fusaro_uid57715	NC_007355	chromosome		arCOG02966	7 (-)	S		HEAT repeats containing protein	3E-38	COG1413	FOG: HEAT repeat
73669324	hypothetical protein Mbar_A1818	No	1			2272288	2273136	-		Methanosarcina_barkeri_Fusaro_uid57715	NC_007355	chromosome		arCOG04955	1	S		Uncharacterized conserved protein			
===									12	Methanosarcina_barkeri_Fusaro_uid57715	NC_007355	chromosome									
73669726	hypothetical protein Mbar_A2236	No	0			2842885	2843325	+		Methanosarcina_barkeri_Fusaro_uid57715	NC_007355	chromosome									
73669727	hypothetical protein Mbar_A2237	No	0			2843855	2844238	+		Methanosarcina_barkeri_Fusaro_uid57715	NC_007355	chromosome		arCOG06787	4 (-)	L		PD-(D/E)XK superfamily nuclease			
73669728	hypothetical protein Mbar_A2238	No	0			2844205	2844519	+		Methanosarcina_barkeri_Fusaro_uid57715	NC_007355	chromosome		arCOG07656	3	S		Uncharacterized conserved protein			
73669729	hypothetical protein Mbar_A2239	No	0			2844661	2846775	+		Methanosarcina_barkeri_Fusaro_uid57715	NC_007355	chromosome	arCOG06786,a	arCOG06786	9	Y		Rolling circle replication protein Rep			
73669730	hypothetical protein Mbar_A2240	Yes	2			2848251	2848580	+		Methanosarcina_barkeri_Fusaro_uid57715	NC_007355	chromosome									
73669731	hypothetical protein Mbar_A2241	Yes	2			2848974	2851109	+		Methanosarcina_barkeri_Fusaro_uid57715	NC_007355	chromosome		arCOG02088	1	S		Uncharacterized conserved protein			
73669732	hypothetical protein Mbar_A2242	Yes	1			2851112	2852056	+		Methanosarcina_barkeri_Fusaro_uid57715	NC_007355	chromosome									
73669733	hypothetical protein Mbar_A2243	No	1			2852071	2852508	+		Methanosarcina_barkeri_Fusaro_uid57715	NC_007355	chromosome									
73669734	hypothetical protein Mbar_A2244	No	0			2853062	2853478	+		Methanosarcina_barkeri_Fusaro_uid57715	NC_007355	chromosome									
73669735	hypothetical protein Mbar_A2245	No	0			2853465	2853854	+		Methanosarcina_barkeri_Fusaro_uid57715	NC_007355	chromosome									
73669736	hypothetical protein Mbar_A2246	No	0			2854024	2855019	+		Methanosarcina_barkeri_Fusaro_uid57715	NC_007355	chromosome									
73669737	site-specific recombinase	No	0			2855539	2856486	+		Methanosarcina_barkeri_Fusaro_uid57715	NC_007355	chromosome		arCOG01241	31 (-)	L	XerC	Integrase	6E-19	COG4974	Site-specific recombinase XerD
===									9	Methanosarcina_barkeri_Fusaro_uid57715	NC_007355	chromosome									
73670127	Na(+)-linked D-alanine glycine permease	No	10			3366189	3367469	-		Methanosarcina_barkeri_Fusaro_uid57715	NC_007355	chromosome		arCOG05006	1 (-)	E	AlsT	Na+/alanine symporter	2E-131	COG1115	Na+/alanine symporter
73670128	hypothetical protein Mbar_A2657	No	2	YES		3367842	3368099	+		Methanosarcina_barkeri_Fusaro_uid57715	NC_007355	chromosome									
73670129	hypothetical protein Mbar_A2658	No	0	YES		3368258	3368851	-		Methanosarcina_barkeri_Fusaro_uid57715	NC_007355	chromosome		arCOG06859	2	S		Uncharacterized conserved protein			
73670130	hypothetical protein Mbar_A2659	No	0	YES		3369234	3369497	-		Methanosarcina_barkeri_Fusaro_uid57715	NC_007355	chromosome		arCOG03632	3	S		Uncharacterized conserved protein			
73670131	hypothetical protein Mbar_A2660	No	0	YES		3369590	3369808	-		Methanosarcina_barkeri_Fusaro_uid57715	NC_007355	chromosome		arCOG03631	7	S		Uncharacterized conserved protein			
73670132	hypothetical protein Mbar_A2661	No	0	YES		3369805	3369945	-		Methanosarcina_barkeri_Fusaro_uid57715	NC_007355	chromosome		arCOG03631	7	S		Uncharacterized conserved protein			
73670133	putative type I restriction enzyme R protein	No	0	YES		3370021	3373251	-		Methanosarcina_barkeri_Fusaro_uid57715	NC_007355	chromosome		arCOG00878	2 (-)	V		Type I site-specific restriction-modification systen	1E-108	COG0610	Type I site-specific restriction-modific
73670134	hypothetical protein Mbar_A2663	No	0	YES		3373408	3374172	+		Methanosarcina_barkeri_Fusaro_uid57715	NC_007355	chromosome									
73670135	hypothetical protein Mbar_A2664	No	0	YES		3374459	3375031	-		Methanosarcina_barkeri_Fusaro_uid57715	NC_007355	chromosome		arCOG03631	7	S		Uncharacterized conserved protein			
===									5	Methanosarcina_barkeri_Fusaro_uid57715	NC_007355	chromosome									
73670182	phosphorylase	No	0			3446945	3447280	+		Methanosarcina_barkeri_Fusaro_uid57715	NC_007355	chromosome		arCOG02974	4	S		HEAT repeats containing protein			
73670183	hypothetical protein Mbar_A2714	Yes	1			3448433	3448852	+		Methanosarcina_barkeri_Fusaro_uid57715	NC_007355	chromosome		arCOG03970	1	S		Uncharacterized conserved protein			
73670184	hypothetical protein Mbar_A2715	No	0			3449249	3449590	-		Methanosarcina_barkeri_Fusaro_uid57715	NC_007355	chromosome									
73670185	hypothetical protein Mbar_A2716	No	0			3450022	3452739	-		Methanosarcina_barkeri_Fusaro_uid57715	NC_007355	chromosome		arCOG03038	9 (-)	R	NrfG	TPR repeats containing protein	6E-15	COG0457	FOG: TPR repeat
73670186	hypothetical protein Mbar_A2717	No	0			3452757	3453161	-		Methanosarcina_barkeri_Fusaro_uid57715	NC_007355	chromosome									
===									5	Methanosarcina_barkeri_Fusaro_uid57715	NC_007355	chromosome									
73670415	hypothetical protein Mbar_A2955	No	0			3801081	3801209	-		Methanosarcina_barkeri_Fusaro_uid57715	NC_007355	chromosome									
73670416	hypothetical protein Mbar_A2956	No	0			3801295	3801429	-		Methanosarcina_barkeri_Fusaro_uid57715	NC_007355	chromosome									
73670417	putative acetyltransferase	No	0			3802452	3802949	+		Methanosarcina_barkeri_Fusaro_uid57715	NC_007355	chromosome		arCOG00830	1 (-)	M		Sortase or related acyltransferase	4E-17	COG1247	Sortase and related acyltransferases
73670418	hypothetical protein Mbar_A2958	No	0			3803654	3805024	-		Methanosarcina_barkeri_Fusaro_uid57715	NC_007355	chromosome		arCOG05250	6	S		Uncharacterized conserved protein	0.00004	COG0419	ATPase involved in DNA repair
73670419	hypothetical protein Mbar_A2959	No	0			3805718	3808459	+		Methanosarcina_barkeri_Fusaro_uid57715	NC_007355	chromosome		arCOG02975	2	S		HEAT repeats containing protein			
===									7	Methanosarcina_barkeri_Fusaro_uid57715	NC_007355	chromosome									
73670602	hypothetical protein Mbar_A3151	No	0			4060508	4060798	+		Methanosarcina_barkeri_Fusaro_uid57715	NC_007355	chromosome									
73670603	hypothetical protein Mbar_A3152	Yes	1			4061828	4063123	+		Methanosarcina_barkeri_Fusaro_uid57715	NC_007355	chromosome		arCOG03375	2 (-)	S		HEAT repeats containing protein	3E-25	COG1413	FOG: HEAT repeat
73670604	hypothetical protein Mbar_A3153	Yes	4			4063163	4065076	+		Methanosarcina_barkeri_Fusaro_uid57715	NC_007355	chromosome		arCOG02485	4	S		WD40-like repeat	1E-13	pfam13360	PQQ-like domain. This domain contains se ; FOG: WD40-like repeat
73670605	hypothetical protein Mbar_A3154	Yes	4			4065141	4067036	+		Methanosarcina_barkeri_Fusaro_uid57715	NC_007355	chromosome		arCOG02485	4	S		WD40-like repeat	7E-12	pfam13360	PQQ-like domain. This domain contains se
73670606	hypothetical protein Mbar_A3155	Yes	0			4067244	4067732	-		Methanosarcina_barkeri_Fusaro_uid57715	NC_007355	chromosome		arCOG03997	2	S		Uncharacterized conserved protein			
73670607	hypothetical protein Mbar_A3156	No	1			4068023	4068397	+		Methanosarcina_barkeri_Fusaro_uid57715	NC_007355	chromosome		arCOG07668	2	S		Uncharacterized conserved protein			
73670608	FtsJ-like methyltransferase	No	0			4070500	4071210	+		Methanosarcina_barkeri_Fusaro_uid57715	NC_007355	chromosome		arCOG00080	1 (-)	J		Predicted rRNA methylase	2E-21	COG1189	Predicted rRNA methylase
===									6	Methanosarcina_barkeri_Fusaro_uid57715	NC_007355	chromosome									
73670612	hypothetical protein Mbar_A3163	No	0			4073342	4073800	+		Methanosarcina_barkeri_Fusaro_uid57715	NC_007355	chromosome									
73670613	hypothetical protein Mbar_A3164	No	0			4073811	4074146	+		Methanosarcina_barkeri_Fusaro_uid57715	NC_007355	chromosome									
73670614	Fe-S oxidoreductase	No	0			4074139	4075518	+		Methanosarcina_barkeri_Fusaro_uid57715	NC_007355	chromosome		arCOG01356	2 (-)	C		Radical SAM superfamily enzyme	1E-30	COG1032	Fe-S oxidoreductase
73670615	hypothetical protein Mbar_A3166	No	0			4076414	4076842	+		Methanosarcina_barkeri_Fusaro_uid57715	NC_007355	chromosome									
73670616	tRNA nucleotidyltransferase	No	0			4077110	4078324	+		Methanosarcina_barkeri_Fusaro_uid57715	NC_007355	chromosome							2E-33	COG0617	tRNA nucleotidyltransferase/poly(A) poly
73670617	hypothetical protein Mbar_A3168	No	0			4079233	4079703	-		Methanosarcina_barkeri_Fusaro_uid57715	NC_007355	chromosome									
===									7	Methanosarcina_barkeri_Fusaro_uid57715	NC_007355	chromosome									
73670719	hypothetical protein Mbar_A3273	No	3	YES		4207135	4207806	-		Methanosarcina_barkeri_Fusaro_uid57715	NC_007355	chromosome		arCOG06799	2	S		Uncharacterized conserved protein			
73670720	type I site-specific deoxyribonuclease	No	0	YES		4208140	4210893	-		Methanosarcina_barkeri_Fusaro_uid57715	NC_007355	chromosome		arCOG00880	2 (-)	V	HsdR	Type I site-specific restriction-modification systen	7E-75	COG4096	Type I site-specific restriction-modific
73670721	hypothetical protein Mbar_A3275	Yes	1	YES		4211041	4212171	-		Methanosarcina_barkeri_Fusaro_uid57715	NC_007355	chromosome							0.00007	pfam09073	BUD22. BUD22 has been shown in yeast to
73670722	hypothetical protein Mbar_A3276	No	1	YES		4213033	4213305	+		Methanosarcina_barkeri_Fusaro_uid57715	NC_007355	chromosome		arCOG05225	2	S		Uncharacterized conserved protein			
73670723	hypothetical protein Mbar_A3277	No	0	YES		4214018	4214323	-		Methanosarcina_barkeri_Fusaro_uid57715	NC_007355	chromosome		arCOG06809	1	S		Uncharacterized conserved protein			
73670724	hypothetical protein Mbar_A3278	No	0	YES		4214456	4214821	+		Methanosarcina_barkeri_Fusaro_uid57715	NC_007355	chromosome									
73670725	aminotransferase	No	0			4215131	4216336	-		Methanosarcina_barkeri_Fusaro_uid57715	NC_007355	chromosome		arCOG00914	1 (-)	E	ArgD	Ornithine/acetylornithine aminotransferase	1E-105	COG4992	Ornithine/acetylornithine aminotransfera
===									5	Methanosarcina_barkeri_Fusaro_uid57715	NC_007355	chromosome									
73670938	hypothetical protein Mbar_A3502	No	0			4492465	4492719	-		Methanosarcina_barkeri_Fusaro_uid57715	NC_007355	chromosome									
73670939	tryptophan synthase subunit beta	No	0			4492772	4494070	+		Methanosarcina_barkeri_Fusaro_uid57715	NC_007355	chromosome		arCOG01432	1 (-)	E		Tryptophan synthase beta-subunit		COG1350	Predicted alternative tryptophan synthas
73670940	hypothetical protein Mbar_A3504	No	0			4494668	4494895	-		Methanosarcina_barkeri_Fusaro_uid57715	NC_007355	chromosome									
73670941	hypothetical protein Mbar_A3505	No	0			4495060	4495467	-		Methanosarcina_barkeri_Fusaro_uid57715	NC_007355	chromosome		arCOG03354	1	S		Uncharacterized conserved protein	3E-69	pfam08004	Protein of unknown function (DUF1699). T
73670942	hypothetical protein Mbar_A3506	No	0			4495954	4496361	-		Methanosarcina_barkeri_Fusaro_uid57715	NC_007355	chromosome		arCOG03356	1	S		Uncharacterized conserved protein	2E-71	pfam08004	Protein of unknown function (DUF1699). T
===									5	Methanosarcina_barkeri_Fusaro_uid57715	NC_007355	chromosome									
73671058	hypothetical protein Mbar_A3628	No	0			4661974	4662720	-		Methanosarcina_barkeri_Fusaro_uid57715	NC_007355	chromosome		arCOG03540	2	S		Uncharacterized conserved protein	0.000004	COG0419	ATPase involved in DNA repair
73671059	hypothetical protein Mbar_A3629	No	0			4663562	4664455	-		Methanosarcina_barkeri_Fusaro_uid57715	NC_007355	chromosome		arCOG03536	3	S		Uncharacterized conserved protein			
73671060	cell surface glycoprotein	Yes	1			4664790	4666292	-		Methanosarcina_barkeri_Fusaro_uid57715	NC_007355	chromosome	arCOG01974,a	arCOG01974	1 (-)	O	TrxA	Thiol-disulfide isomerase or thioredoxin	6E-40	pfam05048	Periplasmic copper-binding protein (NosD ; Thiol-disulfide isomerase and thioredoxi
73671061	hypothetical protein Mbar_A3631	No	0			4666625	4667263	-		Methanosarcina_barkeri_Fusaro_uid57715	NC_007355	chromosome		arCOG03539	3	S		Uncharacterized conserved protein			
73671062	hypothetical protein Mbar_A3632	No	0			4667617	4668462	-		Methanosarcina_barkeri_Fusaro_uid57715	NC_007355	chromosome		arCOG03538	4	S		Uncharacterized conserved protein			
===									7	Methanosarcina_barkeri_Fusaro_uid57715	NC_007355	chromosome									
73671084	hypothetical protein Mbar_A3655	Yes	1			4711771	4712166	-		Methanosarcina_barkeri_Fusaro_uid57715	NC_007355	chromosome									
73671085	transposase	No	0			4713326	4714921	-		Methanosarcina_barkeri_Fusaro_uid57715	NC_007355	chromosome		arCOG03469	1 (-)	L		Transposase	1E-92	COG5421	Transposase
73671086	hypothetical protein Mbar_A3657	No	8			4714988	4716073	-		Methanosarcina_barkeri_Fusaro_uid57715	NC_007355	chromosome		arCOG07506	3	S		Uncharacterized conserved protein			
73671087	hypothetical protein Mbar_A3658	Yes	1			4717042	4718244	-		Methanosarcina_barkeri_Fusaro_uid57715	NC_007355	chromosome							0.00000007	pfam04615	Utp14 protein. This protein is found to
73671088	hypothetical protein Mbar_A3659	Yes	2			4718417	4719202	-		Methanosarcina_barkeri_Fusaro_uid57715	NC_007355	chromosome									
73671089	hypothetical protein Mbar_A3660	No	0			4719957	4721219	+		Methanosarcina_barkeri_Fusaro_uid57715	NC_007355	chromosome		arCOG05241	3	S		Uncharacterized conserved protein			
73671090	integral membrane protein	Yes	6			4721473	4722105	-		Methanosarcina_barkeri_Fusaro_uid57715	NC_007355	chromosome		arCOG01731	2 (-)	V	NorM	Na+-driven multidrug efflux pump	5E-12	pfam01554	MatE. The MatE domain
===									6	Methanosarcina_mazei_Go1_uid57893	NC_003901	chromosome									
21226111	hypothetical protein MM_0009	No	7			9608	10900	-		Methanosarcina_mazei_Go1_uid57893	NC_003901	chromosome		arCOG02264	1 (-)	S		Predicted membrane protein	4E-45	COG1808	Predicted membrane protein
21226112	hypothetical protein MM_0010	No	2			11087	11299	-		Methanosarcina_mazei_Go1_uid57893	NC_003901	chromosome									
21226113	hypothetical protein MM_0011	No	0			12748	13155	+		Methanosarcina_mazei_Go1_uid57893	NC_003901	chromosome		arCOG03355	2	S		Uncharacterized conserved protein	8E-67	pfam08004	Protein of unknown function (DUF1699). T
21226114	RNA signal recognition particle 4.5S RNA	No	0			14436	14801	+		Methanosarcina_mazei_Go1_uid57893	NC_003901	chromosome		arCOG09468	2	S		Uncharacterized conserved protein, DUF1428	2E-32	COG5507	Uncharacterized conserved protein
21226115	hypothetical protein MM_0013	No	0			15146	15637	+		Methanosarcina_mazei_Go1_uid57893	NC_003901	chromosome		arCOG06856	1	S		Uncharacterized conserved protein			
21226116	undecaprenyl pyrophosphate synthetase	No	0			16177	16548	+		Methanosarcina_mazei_Go1_uid57893	NC_003901	chromosome		arCOG01532	2 (-)	I	UppS	Undecaprenyl pyrophosphate synthase	9E-12	pfam01255	Putative undecaprenyl diphosphate syntha
===									6	Methanosarcina_mazei_Go1_uid57893	NC_003901	chromosome									
21226134	hypothetical protein MM_0032	No	0	YES		36605	36829	+		Methanosarcina_mazei_Go1_uid57893	NC_003901	chromosome		arCOG03086	2 (-)	R		Predicted RNA binding protein (dsRBD-like fold),	2E-10	pfam07927	YcfA-like protein. The viral, archaeal a
21226135	hypothetical protein MM_0033	No	0	YES		36987	37358	-		Methanosarcina_mazei_Go1_uid57893	NC_003901	chromosome		arCOG05205	2	S		Uncharacterized conserved protein			
21226136	hypothetical protein MM_0034	No	4	YES		37836	38780	-		Methanosarcina_mazei_Go1_uid57893	NC_003901	chromosome									
21226137	hypothetical protein MM_0035	No	2			39205	41862	-		Methanosarcina_mazei_Go1_uid57893	NC_003901	chromosome		arCOG02974	4	S		HEAT repeats containing protein			
21226138	hypothetical protein MM_0036	No	0			42333	44348	+		Methanosarcina_mazei_Go1_uid57893	NC_003901	chromosome									
161485685	argininosuccinate synthase	No	0			44603	45787	-		Methanosarcina_mazei_Go1_uid57893	NC_003901	chromosome		arCOG00112	1 (-)	E	ArgG	Argininosuccinate synthase	2E-167	pfam00764	Arginosuccinate synthase. This family co
===									8	Methanosarcina_mazei_Go1_uid57893	NC_003901	chromosome									
21226451	hypothetical protein MM_0349	No	0			456929	457147	+		Methanosarcina_mazei_Go1_uid57893	NC_003901	chromosome									
21226452	putative molybdopterin biosynthesis protein	No	0			457470	458210	+		Methanosarcina_mazei_Go1_uid57893	NC_003901	chromosome		arCOG02021	2 (-)	P	PspE	Rhodanese-related sulfurtransferase	1E-18	COG0607	Rhodanese-related sulfurtransferase
21226453	hypothetical protein MM_0351	No	0			458507	459502	+		Methanosarcina_mazei_Go1_uid57893	NC_003901	chromosome		arCOG03496	1	S		Uncharacterized conserved protein			
21226454	hypothetical protein MM_0352	No	0			459465	459740	+		Methanosarcina_mazei_Go1_uid57893	NC_003901	chromosome									
21226455	hypothetical protein MM_0353	No	0			460059	460760	-		Methanosarcina_mazei_Go1_uid57893	NC_003901	chromosome		arCOG02536	1	S		PKD repeats containing protein			
21226456	hypothetical protein MM_0354	Yes	1			461765	462097	+		Methanosarcina_mazei_Go1_uid57893	NC_003901	chromosome		arCOG05241	3	S		Uncharacterized conserved protein			
21226457	transposase	No	0			462142	462606	-		Methanosarcina_mazei_Go1_uid57893	NC_003901	chromosome		arCOG02759	1 (-)	L		Transposase	1E-21	COG1943	Transposase and inactivated derivatives
21226458	hypothetical protein MM_0356	No	0			462764	463789	+		Methanosarcina_mazei_Go1_uid57893	NC_003901	chromosome		arCOG05241	3	S		Uncharacterized conserved protein			
===									5	Methanosarcina_mazei_Go1_uid57893	NC_003901	chromosome									
21226621	hypothetical protein MM_0519	No	0			647462	648451	-		Methanosarcina_mazei_Go1_uid57893	NC_003901	chromosome							3E-14	pfam14249	Tocopherol cyclase. This family contains
21226622	hypothetical protein MM_0520	No	0			648444	650030	-		Methanosarcina_mazei_Go1_uid57893	NC_003901	chromosome							0.0000002	COG0419	ATPase involved in DNA repair
21226623	hypothetical protein MM_0521	No	0			649989	650228	+		Methanosarcina_mazei_Go1_uid57893	NC_003901	chromosome									
21226624	transposase	No	0			650298	651542	+		Methanosarcina_mazei_Go1_uid57893	NC_003901	chromosome		arCOG03585	6 (-)	L		Transposase	5E-15	COG3547	Transposase and inactivated derivatives
21226625	hypothetical protein MM_0523	No	0			651555	651902	-		Methanosarcina_mazei_Go1_uid57893	NC_003901	chromosome									
===									10	Methanosarcina_mazei_Go1_uid57893	NC_003901	chromosome									
21226649	hypothetical protein MM_0547	No	8	YES		675845	676567	+		Methanosarcina_mazei_Go1_uid57893	NC_003901	chromosome									
21226650	hypothetical protein MM_0548	No	0	YES		676642	676866	+		Methanosarcina_mazei_Go1_uid57893	NC_003901	chromosome									
21226651	hypothetical protein MM_0549	No	0	YES		676921	677325	-		Methanosarcina_mazei_Go1_uid57893	NC_003901	chromosome		arCOG06831	2 (-)	R	Doc	Prophage maintenance system killer protein	0.00000004	COG3654	Prophage maintenance system killer prote
21226652	hypothetical protein MM_0550	No	0	YES		677312	677563	-		Methanosarcina_mazei_Go1_uid57893	NC_003901	chromosome		arCOG03967	1	S		Uncharacterized conserved protein			
21226653	hypothetical protein MM_0551	No	0	YES		678340	678564	-		Methanosarcina_mazei_Go1_uid57893	NC_003901	chromosome		arCOG05204	2	S		Uncharacterized conserved protein			
21226654	hypothetical protein MM_0552	No	0	YES		678610	678858	-		Methanosarcina_mazei_Go1_uid57893	NC_003901	chromosome		arCOG06755	1	S		Uncharacterized conserved protein			
21226655	hypothetical protein MM_0553	No	0	YES		679897	680190	+		Methanosarcina_mazei_Go1_uid57893	NC_003901	chromosome									
21226656	hypothetical protein MM_0554	No	0	YES		680167	680421	-		Methanosarcina_mazei_Go1_uid57893	NC_003901	chromosome									

	21226657	hypothetical protein MM_0555	No	0	YES	680804	681043	+		Methanosarcina_mazei_Go1_uid57893	NC_003901	chromosome									
	21226659	hypothetical protein MM_0557	No	0	YES	682642	683241	-		Methanosarcina_mazei_Go1_uid57893	NC_003901	chromosome		arCOG00786	4 (-)	V	Cas4	CRISPR-associated protein Cas4, RecB family ex	0.00000001	COG1468	CRISPR-associated protein Cas4 (RecB fam
===									5	Methanosarcina_mazei_Go1_uid57893	NC_003901	chromosome									
	21226811	hypothetical protein MM_0709	No	0		854842	855057	-		Methanosarcina_mazei_Go1_uid57893	NC_003901	chromosome		arCOG07504	1	S		Uncharacterized conserved protein			
	21226812	hypothetical protein MM_0710	No	0		855304	855588	-		Methanosarcina_mazei_Go1_uid57893	NC_003901	chromosome									
	21226813	transposase	No	0		856718	858148	+		Methanosarcina_mazei_Go1_uid57893	NC_003901	chromosome		arCOG03474	2 (-)	L		Transposase	2E-34	COG5421	Transposase
	21226814	hypothetical protein MM_0712	No	0		858345	859103	+		Methanosarcina_mazei_Go1_uid57893	NC_003901	chromosome		arCOG03969	2	S		Uncharacterized conserved protein	6E-54	pfam07900	Protein of unknown function (DUF1670). T
	21226815	hypothetical protein MM_0713	No	0		859100	859888	+		Methanosarcina_mazei_Go1_uid57893	NC_003901	chromosome		arCOG03968	2	S		Uncharacterized conserved protein	2E-73	pfam07900	Protein of unknown function (DUF1670). T
===									7	Methanosarcina_mazei_Go1_uid57893	NC_003901	chromosome									
	21227306	hypothetical protein MM_1204	No	0		1429466	1429921	+		Methanosarcina_mazei_Go1_uid57893	NC_003901	chromosome		arCOG04915	5 (-)	S		Uncharacterized conserved protein			
	21227307	hypothetical protein MM_1205	No	5		1429957	1430676	+		Methanosarcina_mazei_Go1_uid57893	NC_003901	chromosome		arCOG03254	3	S		Uncharacterized conserved protein	7E-32	pfam07895	Protein of unknown function (DUF1673). T
	21227308	hypothetical protein MM_1206	No	4		1430673	1431290	+		Methanosarcina_mazei_Go1_uid57893	NC_003901	chromosome		arCOG03251	3	S		Uncharacterized conserved protein	1E-65	pfam07895	Protein of unknown function (DUF1673). T
	21227309	hypothetical protein MM_1207	No	4		1431291	1431917	+		Methanosarcina_mazei_Go1_uid57893	NC_003901	chromosome		arCOG03252	4	S		Uncharacterized conserved protein	2E-44	pfam07895	Protein of unknown function (DUF1673). T
	21227310	hypothetical protein MM_1208	No	4		1431914	1432546	+		Methanosarcina_mazei_Go1_uid57893	NC_003901	chromosome		arCOG03253	4	S		Uncharacterized conserved protein	3E-50	pfam07895	Protein of unknown function (DUF1673). T
	21227311	hypothetical protein MM_1209	No	4		1432543	1433163	+		Methanosarcina_mazei_Go1_uid57893	NC_003901	chromosome		arCOG03249	4 (-)	S		Uncharacterized conserved protein	2E-66	pfam07895	Protein of unknown function (DUF1673). T
	21227312	hypothetical protein MM_1210	No	4		1433164	1433883	+		Methanosarcina_mazei_Go1_uid57893	NC_003901	chromosome		arCOG06845	3	S		Uncharacterized conserved protein	0.000005	pfam07895	Protein of unknown function (DUF1673). T
===									5	Methanosarcina_mazei_Go1_uid57893	NC_003901	chromosome									
	21228099	hypothetical protein MM_1997	Yes	7		2375266	2376840	-		Methanosarcina_mazei_Go1_uid57893	NC_003901	chromosome		arCOG05162	3	S		Uncharacterized conserved protein			
	21228100	cobaltochelatase subunit CobN	No	0		2376852	2380766	-		Methanosarcina_mazei_Go1_uid57893	NC_003901	chromosome		arCOG03022	5 (-)	H	CobN	Cobalamin biosynthesis protein CobN or related Mg-chelatase		pfam02514	CobN/Magnesium Chelatase. This family co
	21228101	hypothetical protein MM_1999	Yes	0		2380776	2382494	-		Methanosarcina_mazei_Go1_uid57893	NC_003901	chromosome		arCOG02489	6	S		WD40 repeats containing protein			
	21228102	hypothetical protein MM_2000	Yes	1		2382499	2384019	-		Methanosarcina_mazei_Go1_uid57893	NC_003901	chromosome		arCOG02489	6	S		WD40 repeats containing protein	0.00005	COG2319	FOG: WD40 repeat
	21228103	hypothetical protein MM_2001	Yes	1		2384073	2384666	-		Methanosarcina_mazei_Go1_uid57893	NC_003901	chromosome		arCOG05163	3	S		Uncharacterized conserved protein			
===									6	Methanosarcina_mazei_Go1_uid57893	NC_003901	chromosome									
	21228126	hypothetical protein MM_2024	No	1		2416103	2416585	-		Methanosarcina_mazei_Go1_uid57893	NC_003901	chromosome		arCOG02981	1 (-)	S		Uncharacterized conserved protein			
	21228127	hypothetical protein MM_2025	No	3		2418771	2419166	+		Methanosarcina_mazei_Go1_uid57893	NC_003901	chromosome									
	21228128	hypothetical protein MM_2026	No	0		2419617	2420204	+		Methanosarcina_mazei_Go1_uid57893	NC_003901	chromosome		arCOG09655	2	S		Uncharacterized conserved protein			
	21228129	hypothetical protein MM_2027	No	0		2420223	2420648	-		Methanosarcina_mazei_Go1_uid57893	NC_003901	chromosome									
	21228130	hypothetical protein MM_2028	No	0		2421736	2424741	-		Methanosarcina_mazei_Go1_uid57893	NC_003901	chromosome	arCOG02973,a	arCOG02973	1	S		HEAT repeats containing protein	2E-13	COG1413	FOG: HEAT repeat
	21228131	hypothetical protein MM_2029	No	0		2425620	2425835	+		Methanosarcina_mazei_Go1_uid57893	NC_003901	chromosome		arCOG06871	1 (-)	S		Uncharacterized conserved protein			
===									8	Methanosarcina_mazei_Go1_uid57893	NC_003901	chromosome									
	21228218	hypothetical protein MM_2116	No	0		2528368	2528616	+		Methanosarcina_mazei_Go1_uid57893	NC_003901	chromosome									
	21228219	hypothetical protein MM_2117	No	5		2528662	2531085	+		Methanosarcina_mazei_Go1_uid57893	NC_003901	chromosome		arCOG02884	2 (-)	S		Predicted membrane protein	2E-28	COG4743	Predicted membrane protein
	21228220	hypothetical protein MM_2118	No	0		2531905	2532393	+		Methanosarcina_mazei_Go1_uid57893	NC_003901	chromosome		arCOG06531	4	S		Uncharacterized conserved protein			
	21228221	hypothetical protein MM_2119	No	1		2533190	2533672	+		Methanosarcina_mazei_Go1_uid57893	NC_003901	chromosome		arCOG06531	4	S		Uncharacterized conserved protein			
	21228222	hypothetical protein MM_2120	No	0		2534493	2535068	+		Methanosarcina_mazei_Go1_uid57893	NC_003901	chromosome		arCOG08953	3	S		Uncharacterized conserved protein			
	21228223	hypothetical protein MM_2121	No	0		2535233	2535637	+		Methanosarcina_mazei_Go1_uid57893	NC_003901	chromosome		arCOG08952	2	S		Uncharacterized conserved protein			
	21228224	putative serine/threonine protein kinase	No	0		2536074	2537264	+		Methanosarcina_mazei_Go1_uid57893	NC_003901	chromosome	arCOG05197,a	arCOG05197	1 (-)	T		PASTA domain	9E-23	COG2815	Uncharacterized protein conserved in bac ; PASTA domain. This domain is found at th
	21228225	hypothetical protein MM_2123	No	0		2537280	2537543	+		Methanosarcina_mazei_Go1_uid57893	NC_003901	chromosome		arCOG07645	1	S		Uncharacterized conserved protein			
===									6	Methanosarcina_mazei_Go1_uid57893	NC_003901	chromosome									
	21228775	hypothetical protein MM_2673	No	0		3196278	3196583	-		Methanosarcina_mazei_Go1_uid57893	NC_003901	chromosome		arCOG04979	1	S		Uncharacterized conserved protein			
	21228776	hypothetical protein MM_2674	No	0		3196660	3199650	-		Methanosarcina_mazei_Go1_uid57893	NC_003901	chromosome		arCOG02975	2	S		HEAT repeats containing protein	0.00006	COG1413	FOG: HEAT repeat
	21228777	Serine protease inhibitor	Yes	0		3199762	3201042	-		Methanosarcina_mazei_Go1_uid57893	NC_003901	chromosome		arCOG04933	4 (-)	O		Serine protease inhibitor		COG4826	Serine protease inhibitor
	21228778	hypothetical protein MM_2676	No	0	YES	3201412	3202752	+		Methanosarcina_mazei_Go1_uid57893	NC_003901	chromosome		arCOG05250	6	S		Uncharacterized conserved protein			
	21228779	hypothetical protein MM_2677	No	0	YES	3202956	3203135	-		Methanosarcina_mazei_Go1_uid57893	NC_003901	chromosome									
	21228780	hypothetical protein MM_2678	Yes	0	YES	3203467	3203703	+		Methanosarcina_mazei_Go1_uid57893	NC_003901	chromosome							1E-12	COG3237	Uncharacterized protein conserved in bac
===									8	Methanosarcina_mazei_Go1_uid57893	NC_003901	chromosome									
	21228834	hypothetical protein MM_2732	No	0	YES	3269558	3270181	-		Methanosarcina_mazei_Go1_uid57893	NC_003901	chromosome		arCOG06859	2	S		Uncharacterized conserved protein			
	21228835	hypothetical protein MM_2733	No	0	YES	3270295	3273501	-		Methanosarcina_mazei_Go1_uid57893	NC_003901	chromosome		arCOG00878	2 (-)	V		Type I site-specific restriction-modification systen	3E-105	COG0610	Type I site-specific restriction-modific
	21228836	hypothetical protein MM_2734	No	0	YES	3273727	3274029	-		Methanosarcina_mazei_Go1_uid57893	NC_003901	chromosome		arCOG03632	3	S		Uncharacterized conserved protein			
	21228837	hypothetical protein MM_2735	No	0	YES	3274005	3274283	-		Methanosarcina_mazei_Go1_uid57893	NC_003901	chromosome		arCOG03631	7	S		Uncharacterized conserved protein			
	21228838	hypothetical protein MM_2736	No	0	YES	3274362	3274913	-		Methanosarcina_mazei_Go1_uid57893	NC_003901	chromosome		arCOG03631	7	S		Uncharacterized conserved protein			
	21228839	hypothetical protein MM_2737	No	0	YES	3275116	3275709	-		Methanosarcina_mazei_Go1_uid57893	NC_003901	chromosome		arCOG03631	7	S		Uncharacterized conserved protein			
	21228840	hypothetical protein MM_2738	No	0	YES	3275786	3276301	-		Methanosarcina_mazei_Go1_uid57893	NC_003901	chromosome		arCOG03631	7	S		Uncharacterized conserved protein			
	21228841	type I restriction-modification system specificity	No	0	YES	3276324	3277544	-		Methanosarcina_mazei_Go1_uid57893	NC_003901	chromosome		arCOG02628	3 (-)	V	HsdS	Restriction endonuclease S subunit	2E-16	COG0732	Restriction endonuclease S subunits
===									15	Methanosarcina_mazei_Go1_uid57893	NC_003901	chromosome									
	21228856	hypothetical protein MM_2754	No	0		3300817	3303024	+		Methanosarcina_mazei_Go1_uid57893	NC_003901	chromosome		arCOG07652	5 (-)	L		Superfamily II helicase or inactivated derivatives	4E-22	COG5519	Superfamily II helicase and inactivated
	21228857	hypothetical protein MM_2755	No	0		3304162	3304656	+		Methanosarcina_mazei_Go1_uid57893	NC_003901	chromosome									
	21228858	hypothetical protein MM_2756	No	0		3304813	3305430	+		Methanosarcina_mazei_Go1_uid57893	NC_003901	chromosome							0.00007	COG0419	ATPase involved in DNA repair
	21228859	hypothetical protein MM_2757	No	0		3305636	3305932	+		Methanosarcina_mazei_Go1_uid57893	NC_003901	chromosome									
	21228860	hypothetical protein MM_2758	No	0		3305933	3306463	+		Methanosarcina_mazei_Go1_uid57893	NC_003901	chromosome									
	21228861	hypothetical protein MM_2759	Yes	0		3306939	3307256	+		Methanosarcina_mazei_Go1_uid57893	NC_003901	chromosome									
	21228862	hypothetical protein MM_2760	No	0		3307425	3312317	+		Methanosarcina_mazei_Go1_uid57893	NC_003901	chromosome									
	21228863	hypothetical protein MM_2761	No	0		3312354	3312944	+		Methanosarcina_mazei_Go1_uid57893	NC_003901	chromosome									
	21228864	hypothetical protein MM_2762	No	2		3313280	3313537	+		Methanosarcina_mazei_Go1_uid57893	NC_003901	chromosome									
	21228865	putative DNA recombinase	No	0		3314653	3315192	+		Methanosarcina_mazei_Go1_uid57893	NC_003901	chromosome		arCOG03162	12 (-)	L	PinR	Site-specific recombinase, DNA invertase Pin ho	7E-17	pfam00239	Resolvase, N terminal domain. The N-term
	21228866	hypothetical protein MM_2764	No	0		3315583	3316029	+		Methanosarcina_mazei_Go1_uid57893	NC_003901	chromosome									
	21228867	hypothetical protein MM_2765	No	0		3316030	3316398	+		Methanosarcina_mazei_Go1_uid57893	NC_003901	chromosome									
	21228868	hypothetical protein MM_2766	No	0		3316406	3316870	+		Methanosarcina_mazei_Go1_uid57893	NC_003901	chromosome									
	21228869	hypothetical protein MM_2767	No	0		3316871	3317779	+		Methanosarcina_mazei_Go1_uid57893	NC_003901	chromosome		arCOG07960	16 (-)	R		ATPase (AAA+ superfamily)			
	21228870	hypothetical protein MM_2768	No	0		3318706	3319026	-		Methanosarcina_mazei_Go1_uid57893	NC_003901	chromosome		arCOG03995	2	S		Uncharacterized conserved protein	0.00000008	COG4894	Uncharacterized conserved protein
===									5	Methanosarcina_mazei_Go1_uid57893	NC_003901	chromosome									
	21229133	hypothetical protein MM_3031	No	2		3669405	3669626	-		Methanosarcina_mazei_Go1_uid57893	NC_003901	chromosome									
	21229134	hypothetical protein MM_3032	No	0		3669949	3670674	-		Methanosarcina_mazei_Go1_uid57893	NC_003901	chromosome		arCOG03174	2 (-)	S		Zinc finger domain containing protein (CDGSH-typ	1E-13	COG3369	Zinc finger domain containing protein (C ; Zinc finger domain containing protein (C
	21229135	hypothetical protein MM_3033	No	0		3670695	3670823	+		Methanosarcina_mazei_Go1_uid57893	NC_003901	chromosome									
	21229136	hypothetical protein MM_3034	No	3		3671240	3671680	+		Methanosarcina_mazei_Go1_uid57893	NC_003901	chromosome		arCOG05168	1	S		Uncharacterized conserved protein			
	21229137	hypothetical protein MM_3035	No	0		3671718	3671975	+		Methanosarcina_mazei_Go1_uid57893	NC_003901	chromosome									
===									6	Methanosarcina_mazei_Go1_uid57893	NC_003901	chromosome									
	21229162	hypothetical protein MM_3060	No	0		3702004	3702525	-		Methanosarcina_mazei_Go1_uid57893	NC_003901	chromosome									
	21229163	hypothetical protein MM_3061	No	0		3702633	3705143	-		Methanosarcina_mazei_Go1_uid57893	NC_003901	chromosome									
	21229164	hypothetical protein MM_3062	No	0		3705182	3706237	-		Methanosarcina_mazei_Go1_uid57893	NC_003901	chromosome							3E-24	pfam08291	Peptidase M15.
	21229165	calcium dependent protease	No	0		3706265	3708568	-		Methanosarcina_mazei_Go1_uid57893	NC_003901	chromosome		arCOG00702	6 (-)	O	AprE	Subtilisin-like serine protease	2E-23	pfam00082	Subtilase family. Subtilases are a famil
	21229166	hypothetical protein MM_3064	No	0		3708804	3712136	-		Methanosarcina_mazei_Go1_uid57893	NC_003901	chromosome									
	21229167	hypothetical protein MM_3065	No	0		3712269	3714686	-		Methanosarcina_mazei_Go1_uid57893	NC_003901	chromosome									
===									7	Methanosarcina_mazei_Go1_uid57893	NC_003901	chromosome									
	21229263	transposase	No	0		3843673	3843957	-		Methanosarcina_mazei_Go1_uid57893	NC_003901	chromosome		arCOG04792	9 (-)	L		Transposase			
	21229264	hypothetical protein MM_3162	No	0		3844614	3844925	+		Methanosarcina_mazei_Go1_uid57893	NC_003901	chromosome									
	21229265	hypothetical protein MM_3163	No	0		3845071	3845232	-		Methanosarcina_mazei_Go1_uid57893	NC_003901	chromosome									
	21229266	hypothetical protein MM_3164	No	0		3845853	3846722	+		Methanosarcina_mazei_Go1_uid57893	NC_003901	chromosome		arCOG03275	1	S		Uncharacterized conserved protein	1E-24	pfam07796	Protein of unknown function (DUF1638). T
	21229267	hypothetical protein MM_3165	No	0		3848698	3848967	+		Methanosarcina_mazei_Go1_uid57893	NC_003901	chromosome									
	21229268	hypothetical protein MM_3166	No	0		3848998	3849381	-		Methanosarcina_mazei_Go1_uid57893	NC_003901	chromosome									
	21229269	transposase	No	0		3849993	3851423	+		Methanosarcina_mazei_Go1_uid57893	NC_003901	chromosome		arCOG03474	2 (-)	L		Transposase	2E-34	COG5421	Transposase
===									8	Methanosarcina_mazei_Go1_uid57893	NC_003901	chromosome									
	21229340	hypothetical protein MM_3238	No	6		3933749	3934315	+		Methanosarcina_mazei_Go1_uid57893	NC_003901	chromosome		arCOG03583	3	S	HdeD	Uncharacterized conserved protein	2E-20	COG3247	Uncharacterized conserved protein
	21229341	hypothetical protein MM_3239	No	0		3934903	3935265	+		Methanosarcina_mazei_Go1_uid57893	NC_003901	chromosome									
	21229342	putative transcriptional regulator	No	0		3935262	3936635	+		Methanosarcina_mazei_Go1_uid57893	NC_003901	chromosome		arCOG00945	1 (-)	R	AslB	Radical SAM superfamily enzyme	1E-18	COG0641	Arylsulfatase regulator (Fe-S oxidoreduc
	21229343	hypothetical protein MM_3241	No	6	YES	3937034	3937600	+		Methanosarcina_mazei_Go1_uid57893	NC_003901	chromosome		arCOG03583	3	S	HdeD	Uncharacterized conserved protein	4E-20	COG3247	Uncharacterized conserved protein
	21229344	hypothetical protein MM_3242	No	4	YES	3937845	3938360	+		Methanosarcina_mazei_Go1_uid57893	NC_003901	chromosome									
	21229345	hypothetical protein MM_3243	No	8	YES	3938456	3939343	-		Methanosarcina_mazei_Go1_uid57893	NC_003901	chromosome		arCOG06746	1	S		Uncharacterized conserved protein			
	21229346	hypothetical protein MM_3244	No	0	YES	3939697	3942306	-		Methanosarcina_mazei_Go1_uid57893	NC_003901	chromosome		arCOG05250	6	S		Uncharacterized conserved protein	0.0000002	COG0419	ATPase involved in DNA repair
	21229347	transposase	No	0	YES	3944678	3945811	-		Methanosarcina_mazei_Go1_uid57893	NC_003901	chromosome		arCOG04792	9 (-)	L		Transposase	2E-21	pfam03050	Transposase IS66 family. Transposase pro
===									6	Methanosarcina_mazei_Go1_uid57893	NC_003901	chromosome									
	21229439	hypothetical protein MM_3337	No	0		4064895	4065140	-		Methanosarcina_mazei_Go1_uid57893	NC_003901	chromosome		arCOG03597	4 (-)	S		Uncharacterized conserved protein	2E-12	pfam10967	Protein of unknown function (DUF2769). T
	21229440	hypothetical protein MM_3338	No	0		4065728	4066555	+		Methanosarcina_mazei_Go1_uid57893	NC_003901	chromosome		arCOG03538	4	S		Uncharacterized conserved protein			
	21229441	hypothetical protein MM_3339	No	0		4066716	4067549	+		Methanosarcina_mazei_Go1_uid57893	NC_003901	chromosome		arCOG03539	3	S		Uncharacterized conserved protein			
	21229442	hypothetical protein MM_3340	No	0		4068085	4068972	+		Methanosarcina_mazei_Go1_uid57893	NC_003901	chromosome		arCOG03536	3	S		Uncharacterized conserved protein			
	21229443	hypothetical protein MM_3341	No	0		4069737	4069937	-		Methanosarcina_mazei_Go1_uid57893	NC_003901	chromosome									
	21229444	hypothetical protein MM_3342	No	0		4070261	4071514	+		Methanosarcina_mazei_Go1_uid57893	NC_003901	chromosome		arCOG03037	1 (-)	R	NrfG	TPR repeats containing protein	2E-29	COG0457	FOG: TPR repeat
===									7	Methanosarcina_mazei_Go1_uid57893	NC_003901	chromosome									
	21229447	hypothetical protein MM_3345	No	2	YES	4073159	4073485	+		Methanosarcina_mazei_Go1_uid57893	NC_003901	chromosome		arCOG07653	1 (-)	S		Uncharacterized conserved protein			
	21229448	hypothetical protein MM_3346	No	0	YES	4074201	4074683	-		Methanosarcina_mazei_Go1_uid57893	NC_003901	chromosome		arCOG05193	1	S		Uncharacterized conserved protein			
	21229449	hypothetical protein MM_3347	No	0	YES	4074963	4075412	+		Methanosarcina_mazei_Go1_uid57893	NC_003901	chromosome									
	21229450	hypothetical protein MM_3348	No	0	YES	4076448	4076627	-		Methanosarcina_mazei_Go1_uid57893	NC_003901	chromosome									
	21229451	hypothetical protein MM_3349	No	0	YES	4076806	4077003	-		Methanosarcina_mazei_Go1_uid57893	NC_003901	chromosome									
	21229452	hypothetical protein MM_3350	No	0	YES	4077279	4077845	-		Methanosarcina_mazei_Go1_uid57893	NC_003901	chromosome		arCOG03560	2 (-)	S		Uncharacterized conserved protein	8E-69	pfam07929	Plasmid pRiA4b ORF-3-like protein. Membe
	21229453	hypothetical protein MM_3351	No	0	YES	4077979	4078113	-		Methanosarcina_mazei_Go1_uid57893	NC_003901	chromosome		arCOG06753	1	S		Uncharacterized conserved protein			
===									11	Methanosarcina_mazei_Go1_uid57893	NC_003901	chromosome									
	21229462	hypothetical protein MM_3360	No	0	YES	4088643	4089089	-		Methanosarcina_mazei_Go1_uid57893	NC_003901	chromosome		arCOG03318	1 (-)	L		Retron-type reverse transcriptase			
	21229463	hypothetical protein MM_3361	No	0	YES	4089787	4090137	+		Methanosarcina_mazei_Go1_uid57893	NC_003901	chromosome									
	21229464	hypothetical protein MM_3362	No	0	YES	4090734	4090970	+		Methanosarcina_mazei_Go1_uid57893	NC_003901	chromosome									
	21229465	hypothetical protein MM_3363	No	0	YES	4091213	4091428	+		Methanosarcina_mazei_Go1_uid57893	NC_003901	chromosome									
	21229466	hypothetical protein MM_3364	No	0	YES	4091910	4092077	+		Methanosarcina_mazei_Go1_uid57893	NC_003901	chromosome									
	21229467	hypothetical protein MM_3365	No	0	YES	4092285	4092488	+		Methanosarcina_mazei_Go1_uid57893	NC_003901	chromosome									
	21229468	hypothetical protein MM_3366	No	0	YES	4092488	4092661	+		Methanosarcina_mazei_Go1_uid57893	NC_003901	chromosome									
	21229470	hypothetical protein MM_3368	No	0	YES	4093354	4093542	+		Methanosarcina_mazei_Go1_uid57893	NC_003901	chromosome									
	21229471	hypothetical protein MM_3369	No	0	YES	4093860	4094033	+		Methanosarcina_mazei_Go1_uid57893	NC_003901	chromosome									
	21229472	hypothetical protein MM_3370	Yes	0	YES	4094732	4095157	-		Methanosarcina_mazei_Go1_uid57893	NC_003901	chromosome									
	21229473	hypothetical protein MM_3371	No	0	YES	4095397	4096173	-		Methanosarcina_mazei_Go1_uid57893	NC_003901	chromosome		arCOG06820	1 (-)	R		Acetyltransferase (GNAT) family	0.000003	pfam14268	YoaP-like. The YoaP-like domain is found
===									10	Methanosphaera_stadtmanae_DSM_3091_uid584	NC_007681	chromosome									
	84489268	hypothetical protein Msp_0457	No	0	YES	536464	536691	+		Methanosphaera_stadtmanae_DSM_3091_uid584	NC_007681	chromosome									
	84489269	hypothetical protein Msp_0458	No	0		536839	537234	+		Methanosphaera_stadtmanae_DSM_3091_uid584	NC_007681	chromosome		arCOG05262	1 (-)	L	XthA	Exonuclease III			
	84489270	hypothetical protein Msp_0459	No	1		537267	537875	+		Methanosphaera_stadtmanae_DSM_3091_uid584	NC_007681	chromosome									
	84489271	hypothetical protein Msp_0460	No	0		538545	539144	+		Methanosphaera_stadtmanae_DSM_3091_uid584	NC_007681	chromosome									
	84489272	hypothetical protein Msp_0461	No	0		539141	539500	+		Methanosphaera_stadtmanae_DSM_3091_uid584	NC_007681	chromosome									
	84489273	hypothetical protein Msp_0462	No	0		539764	539958	+		Methanosphaera_stadtmanae_DSM_3091_uid584	NC_007681	chromosome									
	84489274	hypothetical protein Msp_0463	No	0	YES	540261	540884	-		Methanosphaera_stadtmanae_DSM_3091_uid584	NC_007681	chromosome									
	84489275	hypothetical protein Msp_0464	No	0	YES	541177	542250	-		Methanosphaera_stadtmanae_DSM_3091_uid584	NC_007681	chromosome		arCOG11374	2	S		Uncharacterized conserved protein	0.00002	pfam11185	Protein of unknown function (DUF2971). T
	84489276	hypothetical protein Msp_0465	No	0	YES	542494	546243	-		Methanosphaera_stadtmanae_DSM_3091_uid584	NC_007681	chromosome	arCOG06886,a	arCOG06886	1	S		SWIM/SEC-C metal-binding motif protein	0.00007	pfam13175	AAA ATPase domain. This family of domain ; SEC-C motif. The SEC-C motif found in th
	84489277	hypothetical protein Msp_0466	No	0	YES	546355	546873	-		Methanosphaera_stadtmanae_DSM_3091_uid584	NC_007681	chromosome		arCOG09740	1 (-)	S		Uncharacterized conserved protein	8E-18	pfam08819	Domain of unknown function (DUF1802). Th
===									5	Methanosphaera_stadtmanae_DSM_3091_uid584	NC_007681	chromosome									
	84489283	hypothetical protein Msp_0472	No	0	YES	553904	554671	+		Methanosphaera_stadtmanae_DSM_3091_uid584	NC_007681	chromosome									
	84489284	type III restriction-modification system, restrictic	No	0	YES	554686	557673	+		Methanosphaera_stadtmanae_DSM_3091_uid584	NC_007681	chromosome		arCOG06887	2 (-)	V		Restriction endonuclease	6E-152	COG3587	Restriction endonuclease
	84489285	hypothetical protein Msp_0474	Yes	0	YES	557688	557963	+		Methanosphaera_stadtmanae_DSM_3091_uid584	NC_007681	chromosome							0.000000002	pfam05048	Periplasmic copper-binding protein (NosD
	84489286	hypothetical protein Msp_0475	No	3	YES	557996	558508	+		Methanosphaera_stadtmanae_DSM_3091_uid584	NC_007681	chromosome									
	84489287	hypothetical protein Msp_0476	No	1	YES	558611	558835	+		Methanosphaera_stadtmanae_DSM_3091_uid584	NC_007681	chromosome									
===									9	Methanosphaera_stadtmanae_DSM_3091_uid584	NC_007681	chromosome									
	84489513	hypothetical protein Msp_0704	No	0		813111	813368	+		Methanosphaera_stadtmanae_DSM_3091_uid584	NC_007681	chromosome									
	84489514	hypothetical protein Msp_0705	No	0		814033	814188	+		Methanosphaera_stadtmanae_DSM_3091_uid584	NC_007681	chromosome									
	84489515	hypothetical protein Msp_0706	No	0		814325	814882	+		Methanosphaera_stadtmanae_DSM_3091_uid584	NC_007681	chromosome		arCOG07602	1 (-)	T		Histidine kinase-like ATPase			
	84489516	hypothetical protein Msp_0707	No	0		814879	815139	+		Methanosphaera_stadtmanae_DSM_3091_uid584	NC_007681	chromosome		arCOG07603	1	S		Uncharacterized conserved protein			
	84489517	hypothetical protein Msp_0708	No	0		815586	816218	-		Methanosphaera_stadtmanae_DSM_3091_uid584	NC_007681	chromosome									
	84489518	hypothetical protein Msp_0709	No	0		816357	817133	+		Methanosphaera_stadtmanae_DSM_3091_uid584	NC_007681	chromosome									
	84489519	hypothetical protein Msp_0710	No	0		817126	817584	+		Methanosphaera_stadtmanae_DSM_3091_uid584	NC_007681	chromosome		arCOG10910	4	S		Uncharacterized conserved protein			
	84489520	hypothetical protein Msp_0711	No	0		817588	817944	+		Methanosphaera_stadtmanae_DSM_3091_uid584	NC_007681	chromosome		arCOG06576	5 (-)	V		HEPN domain containing protein			
	84489521	hypothetical protein Msp_0712	Yes	1		818033	818338	+		Methanosphaera_stadtmanae_DSM_3091_uid584	NC_007681	chromosome									
===									8	Methanosphaera_stadtmanae_DSM_3091_uid584	NC_007681	chromosome									
	84489537	hypothetical protein Msp_0728	No	0	YES	841016	841357	+		Methanosphaera_stadtmanae_DSM_3091_uid584	NC_007681	chromosome									
	84489538	hypothetical protein Msp_0729	No	0	YES	841679	842098	-		Methanosphaera_stadtmanae_DSM_3091_uid584	NC_007681	chromosome		arCOG02219	4 (-)	V	VapC	PIN domain containing protein			
	84489539	hypothetical protein Msp_0730	No	0	YES	842095	842325	-		Methanosphaera_stadtmanae_DSM_3091_uid584	NC_007681	chromosome									
	84489540	hypothetical protein Msp_0731	No	3	YES	842674	843015	-		Methanosphaera_stadtmanae_DSM_3091_uid584	NC_007681	chromosome									
	84489541	hypothetical protein Msp_0732	No	0	YES	843214	843543	+		Methanosphaera_stadtmanae_DSM_3091_uid584	NC_007681	chromosome									
	84489542	hypothetical protein Msp_0733	No	4	YES	843543	844040	+		Methanosphaera_stadtmanae_DSM_3091_uid584	NC_007681	chromosome									
	84489543	hypothetical protein Msp_0734	No	0	YES	844026	844379	-		Methanosphaera_stadtmanae_DSM_3091_uid584	NC_007681	chromosome		arCOG03167	6 (-)	R		Predicted ATPase (AAA+ superfamily)	0.0000001	COG1373	Predicted ATPase (AAA+ superfamily)
	84489544	hypothetical protein Msp_0735	No	1	YES	844318	844557	-		Methanosphaera_stadtmanae_DSM_3091_uid584	NC_007681	chromosome									
===									7	Methanosphaera_stadtmanae_DSM_3091_uid584	NC_007681	chromosome									
	84489555	hypothetical protein Msp_0750	No	1	YES	864516	865307	-		Methanosphaera_stadtmanae_DSM_3091_uid584	NC_007681	chromosome									
	84489556	hypothetical protein Msp_0752	No	3	YES	866428	866787	+		Methanosphaera_stadtmanae_DSM_3091_uid584	NC_007681	chromosome		arCOG05100	2 (-)	S		Predicted membrane protein	0.00001	COG2364	Predicted membrane protein
	84489557	hypothetical protein Msp_0753	No	0	YES	867018	867419	+		Methanosphaera_stadtmanae_DSM_3091_uid584	NC_007681	chromosome							0.0001	COG3392	Adenine-specific DNA methylase
	84489558	hypothetical protein Msp_0754	No	0	YES	867426	867821	+		Methanosphaera_stadtmanae_DSM_3091_uid584	NC_007681	chromosome									
	84489559	hypothetical protein Msp_0755	No	0	YES	867953	868111	+		Methanosphaera_stadtmanae_DSM_3091_uid584	NC_007681	chromosome									
	84489560	hypothetical protein Msp_0756	No	0	YES	868421	868801	-		Methanosphaera_stadtmanae_DSM_3091_uid584	NC_007681	chromosome									
	84489561	ATPase	No	0	YES	869250	870470	+		Methanosphaera_stadtmanae_DSM_3091_uid584	NC_007681	chromosome		arCOG03167	6 (-)	R		Predicted ATPase (AAA+ superfamily)	2E-44	COG1373	Predicted ATPase (AAA+ superfamily)
===									9	Methanosphaera_stadtmanae_DSM_3091_uid584	NC_007681	chromosome									
	84489583	hypothetical protein Msp_0779	No	0		912539	913117	-		Methanosphaera_stadtmanae_DSM_3091_uid584	NC_007681	chromosome		arCOG02572	1 (-)	R	WrbA	Multimeric flavodoxin WrbA	1E-17	COG0655	Multimeric flavodoxin WrbA
	84489584	hypothetical protein Msp_0780	No	1		913224	913919	-		Methanosphaera_stadtmanae_DSM_3091_uid584	NC_007681	chromosome									
	84489585	D-tyrosyl-tRNA(Tyr) deacylase	No	0		914148	914594	+		Methanosphaera_stadtmanae_DSM_3091_uid584	NC_007681	chromosome							2E-48	COG1490	D-Tyr-tRNAtyr deacylase
	84489586	hypothetical protein Msp_0782	No	0		914608	914811	+		Methanosphaera_stadtmanae_DSM_3091_uid584	NC_007681	chromosome									
	84489587	hypothetical protein Msp_0783	No	6		914823	915458	+		Methanosphaera_stadtmanae_DSM_3091_uid584	NC_007681	chromosome							0.00009	COG3371	Predicted membrane protein
	84489588	hypothetical protein Msp_0784	No	0		915483	915797	+		Methanosphaera_stadtmanae_DSM_3091_uid584	NC_007681	chromosome		arCOG09728	1	S		Uncharacterized conserved protein			
	84489589	hypothetical protein Msp_0785	No	0		915809	917206	+		Methanosphaera_stadtmanae_DSM_3091_uid584	NC_007681	chromosome									
	84489590	hypothetical protein Msp_0786	No	0		917232	918149	-		Methanosphaera_stadtmanae_DSM_3091_uid584	NC_007681	chromosome									
	84489591	FprA	No	0		918254	919492	-		Methanosphaera_stadtmanae_DSM_3091_uid584	NC_007681	chromosome		arCOG00509	3 (-)	C	FpaA	Uncharacterized flavoprotein	8E-89	COG0426	Uncharacterized flavoproteins
===									5	Methanosphaera_stadtmanae_DSM_3091_uid584	NC_007681	chromosome									
	84489625	hypothetical protein Msp_0825	No	0		957309	957620	+		Methanosphaera_stadtmanae_DSM_3091_uid584	NC_007681	chromosome		arCOG11376	1	S		Uncharacterized conserved protein			
	84489626	cation transport ATPase	No	0		957685	959802	+		Methanosphaera_stadtmanae_DSM_3091_uid584	NC_007681	chromosome		arCOG01576	3 (-)	P	ZntA	Cation transport ATPase	1E-102	COG2217	Cation transport ATPase
	84489627	hypothetical protein Msp_0827	No	1		959907	960224	+		Methanosphaera_stadtmanae_DSM_3091_uid584	NC_007681	chromosome									
	84489628	hypothetical protein Msp_0828	No	0		960488	960958	+		Methanosphaera_stadtmanae_DSM_3091_uid584	NC_007681	chromosome									
	84489629	hypothetical protein Msp_0829	No	4		961432	961857	+		Methanosphaera_stadtmanae_DSM_3091_uid584	NC_007681	chromosome									
===									7	Methanosphaera_stadtmanae_DSM_3091_uid584	NC_007681	chromosome									
	84489637	glutamylcysteine synthetase	No	0	YES	974491	975903	-		Methanosphaera_stadtmanae_DSM_3091_uid584	NC_007681	chromosome									
	84489638	glutamine amidotransferase	No	0		975875	976705	-		Methanosphaera_stadtmanae_DSM_3091_uid584	NC_007681	chromosome		arCOG03639	1 (-)	R		Predicted glutamine amidotransferase	5E-30	pfam13230	Glutamine amidotransferases class-II. Th
	84489639	hypothetical protein Msp_0839	No	0		977307	977633	-		Methanosphaera_stadtmanae_DSM_3091_uid584	NC_007681	chromosome	clust_320								
	84489640	hypothetical protein Msp_0840	No	0		977657	977899	-		Methanosphaera_stadtmanae_DSM_3091_uid584	NC_007681	chromosome									
	84489641	hypothetical protein Msp_0841	No	0		977950	978270	-		Methanosphaera_stadtmanae_DSM_3091_uid584	NC_007681	chromosome	clust_320								
	84489642	hypothetical protein Msp_0842	No	0		978374	978877	-		Methanosphaera_stadtmanae_DSM_3091_uid584	NC_007681	chromosome									
	84489643	hypothetical protein Msp_0843	No	0		979226	979630	-		Methanosphaera_stadtmanae_DSM_3091_uid584	NC_007681	chromosome		arCOG00519	1 (-)	C	FldA	Flavodoxin			
===									10	Methanosphaera_stadtmanae_DSM_3091_uid584	NC_007681	chromosome									
	84489731	hypothetical protein Msp_0932	No	9	YES	1075695	1077602	+		Methanosphaera_stadtmanae_DSM_3091_uid584	NC_007681	chromosome							0.00003	COG1277	ABC-type transport system involved in mu
	84489732	hypothetical protein Msp_0933	Yes	1	YES	1077644	1078318	-		Methanosphaera_stadtmanae_DSM_3091_uid584	NC_007681	chromosome	clust_152								
	84489733	hypothetical protein Msp_0934	No	3	YES	1078322	1078663	-		Methanosphaera_stadtmanae_DSM_3091_uid584	NC_007681	chromosome		arCOG03949	1 (-)	S		Predicted membrane protein	2E-25	pfam09858	Predicted membrane protein (DUF2085). Th
	84489734	hypothetical protein Msp_0935	No	1	YES	1078726	1079376	-		Methanosphaera_stadtmanae_DSM_3091_uid584	NC_007681	chromosome	clust_152								
	84489735	hypothetical protein Msp_0936	No	0	YES	1079468	1079953	-		Methanosphaera_stadtmanae_DSM_3091_uid584	NC_007681	chromosome									
	84489736	hypothetical protein Msp_0937	No	0	YES	1080290	1080661	-		Methanosphaera_stadtmanae_DSM_3091_uid584	NC_007681	chromosome									
	84489737	hypothetical protein Msp_0938	No	0	YES	1080680	1080934	-		Methanosphaera_stadtmanae_DSM_3091_uid584	NC_007681	chromosome									
	84489738	hypothetical protein Msp_0939	No	0	YES	1081114	1081323	-		Methanosphaera_stadtmanae_DSM_3091_uid584	NC_007681	chromosome									
	84489739	hypothetical protein Msp_0940	No	0	YES	1082044	1084110	+		Methanosphaera_stadtmanae_DSM_3091_uid584	NC_007681	chromosome							0.0000005	pfam05316	Mitochondrial ribosomal protein (VAR1). ; CRISPR-associated protein TM1802 (cas_TM
	84489740	hypothetical protein Msp_0941	No	0	YES	1084115	1085038	+		Methanosphaera_stadtmanae_DSM_3091_uid584	NC_007681	chromosome		arCOG02757	1 (-)	V	Cas7	CRISPR system related protein Cas7, RAMP sup	4E-26	pfam05107	Family of unknown function (DUF694). Fam
===									12	Methanosphaera_stadtmanae_DSM_3091_uid584	NC_007681	chromosome									
	84489746	hypothetical protein Msp_0947	No	0	YES	1090897	1091160	+		Methanosphaera_stadtmanae_DSM_3091_uid584	NC_007681	chromosome		arCOG04194	3 (-)	V	Cas2	CRISPR-associated protein Cas2	3E-18	COG1343	CRISPR-associated protein Cas2
	84489747	hypothetical protein Msp_0948	No	1	YES	1091296	1091460	-		Methanosphaera_stadtmanae_DSM_3091_uid584	NC_007681	chromosome									
	84489748	hypothetical protein Msp_0949	No	1	YES	1091479	1091592	-		Methanosphaera_stadtmanae_DSM_3091_uid584	NC_007681	chromosome									
	84489749	hypothetical protein Msp_0950	No	1	YES	1091785	1092069	-		Methanosphaera_stadtmanae_DSM_3091_uid584	NC_007681	chromosome									
	84489750	hypothetical protein Msp_0951	No	0	YES	1092269	1092412	-		Methanosphaera_stadtmanae_DSM_3091_uid584	NC_007681	chromosome									
	84489751	hypothetical protein Msp_0952	No	2	YES	1092984	1093358	-		Methanosphaera_stadtmanae_DSM_3091_uid584	NC_007681	chromosome									
	84489752	hypothetical protein Msp_0953	No	1	YES	1093339	1093557	-		Methanosphaera_stadtmanae_DSM_3091_uid584	NC_007681	chromosome	clust_421								
	84489753	hypothetical protein Msp_0954	No	2	YES	1094314	1094556	-		Methanosphaera_stadtmanae_DSM_3091_uid584	NC_007681	chromosome									
	84489754	hypothetical protein Msp_0955	No	2	YES	1094462	1094689	-		Methanosphaera_stadtmanae_DSM_3091_uid584	NC_007681	chromosome	clust_421								
	84489755	hypothetical protein Msp_0956	No	0	YES	1095123	1095392	+		Methanosphaera_stadtmanae_DSM_3091_uid584	NC_007681	chromosome									
	84489756	hypothetical protein Msp_0957	No	0	YES	1095389	1095667	+		Methanosphaera_stadtmanae_DSM_3091_uid584	NC_007681	chromosome									
	84489757	polar amino acid ABC transporter ATP-binding	No	0		1096241	1096924	-		Methanosphaera_stadtmanae_DSM_3091_uid584	NC_007681	chromosome		arCOG00923	1 (-)	E	GlnQ	ABC-type polar amino acid transport system, ATF	1E-90	COG1126	ABC-type polar amino acid transport syst
===									7	Methanosphaerula_palustris_E1_9c_uid59193	NC_011832	chromosome									
	219851032	glycosyl transferase group 1	No	0		387038	388180	+		Methanosphaerula_palustris_E1_9c_uid59193	NC_011832	chromosome		arCOG01403	4 (-)	M	RfaG	Glycosyltransferase	1E-40	COG0438	Glycosyltransferase
	219851033	hypothetical protein Mpal_0363	No	0		388630	388833	-		Methanosphaerula_palustris_E1_9c_uid59193	NC_011832	chromosome									
	219851034	hypothetical protein Mpal_0365	No	0		389451	389603	+		Methanosphaerula_palustris_E1_9c_uid59193	NC_011832	chromosome									
	219851035	hypothetical protein Mpal_0367	No	0		390457	390678	-		Methanosphaerula_palustris_E1_9c_uid59193	NC_011832	chromosome									
	219851036	hypothetical protein Mpal_0368	No	0		390759	391100	+		Methanosphaerula_palustris_E1_9c_uid59193	NC_011832	chromosome									
	219851037	hypothetical protein Mpal_0369	No	0		391110	391499	+		Methanosphaerula_palustris_E1_9c_uid59193	NC_011832	chromosome		arCOG09441	8	L		HJR family endonuclease			
	219851038	hypothetical protein Mpal_0370	No	0		391483	394659	+		Methanosphaerula_palustris_E1_9c_uid59193	NC_011832	chromosome	arCOG01543,a	arCOG01543	2 (-)	C	NuoI	NADH dehydrogenase subunit I			
===									5	Methanosphaerula_palustris_E1_9c_uid59193	NC_011832	chromosome									
	219851045	hypothetical protein Mpal_0377	No	0	YES	398696	398800	-		Methanosphaerula_palustris_E1_9c_uid59193	NC_011832	chromosome									
	219851046	hypothetical protein Mpal_0378	No	1	YES	398823	399650	+		Methanosphaerula_palustris_E1_9c_uid59193	NC_011832	chromosome		arCOG09443	1	S		Uncharacterized conserved protein			
	219851047	protein of unknown function UPF0150	No	0	YES	399660	399902	-		Methanosphaerula_palustris_E1_9c_uid59193	NC_011832	chromosome		arCOG02411	1 (-)	R		Predicted nuclease of the RNAse H fold, HicB fai	0.000001	COG1598	Predicted nuclease of the RNAse H fold,
	219851048	hypothetical protein Mpal_0382	No	0	YES	400775	401125	-		Methanosphaerula_palustris_E1_9c_uid59193	NC_011832	chromosome									
	219851049	hypothetical protein Mpal_0383	No	4		401477	402058	+		Methanosphaerula_palustris_E1_9c_uid59193	NC_011832	chromosome							0.00007	pfam04307	Predicted membrane-bound metal-dependent
===									5	Methanosphaerula_palustris_E1_9c_uid59193	NC_011832	chromosome									
	219851286	hypothetical protein Mpal_0624	No	0		619874	620092	-		Methanosphaerula_palustris_E1_9c_uid59193	NC_011832	chromosome									
	219851287	UvrD/REP helicase	No	0		620224	623283	-		Methanosphaerula_palustris_E1_9c_uid59193	NC_011832	chromosome		arCOG00787	3 (-)	L		UvrD/Rep family helicase fused to exonuclease f	8E-77	COG0210	Superfamily I DNA and RNA helicases
	219851288	hypothetical protein Mpal_0626	Yes	1		623431	624636	-		Methanosphaerula_palustris_E1_9c_uid59193	NC_011832	chromosome									
	219851289	hypothetical protein Mpal_0627	No	0		625013	625600	+		Methanosphaerula_palustris_E1_9c_uid59193	NC_011832	chromosome									
	219851290	hypothetical protein Mpal_0628	No	0		625700	626383	+		Methanosphaerula_palustris_E1_9c_uid59193	NC_011832	chromosome									
===									5	Methanosphaerula_palustris_E1_9c_uid59193	NC_011832	chromosome									
	219851513	hypothetical protein Mpal_0862	No	0		890562	890666	+		Methanosphaerula_palustris_E1_9c_uid59193	NC_011832	chromosome									
	219851514	hypothetical protein Mpal_0863	No	0		890818	890973	-		Methanosphaerula_palustris_E1_9c_uid59193	NC_011832	chromosome									
	219851515	transcription factor	No	0		891747	892298	+		Methanosphaerula_palustris_E1_9c_uid59193	NC_011832	chromosome		arCOG01765	1 (-)	K	SPT15	TATA-box binding protein (TBP), component of T	2E-52	COG2101	TATA-box binding protein (TBP), componen
	219851516	Glyoxalase/bleomycin resistance protein/dioxy	No	0		892686	893189	+		Methanosphaerula_palustris_E1_9c_uid59193	NC_011832	chromosome							4E-18	COG2764	Uncharacterized protein conserved in bac
	219851517	hypothetical protein Mpal_0867	No	0		894426	894812	+		Methanosphaerula_palustris_E1_9c_uid59193	NC_011832	chromosome									
===									9	Methanosphaerula_palustris_E1_9c_uid59193	NC_011832	chromosome									
	219851534	hypothetical protein Mpal_0885	No	1	YES	914967	916415	-		Methanosphaerula_palustris_E1_9c_uid59193	NC_011832	chromosome									
	219851535	hypothetical protein Mpal_0886	No	0	YES	916412	916522	-		Methanosphaerula_palustris_E1_9c_uid59193	NC_011832	chromosome									
	219851536	hypothetical protein Mpal_0887	No	0	YES	916519	916722	-		Methanosphaerula_palustris_E1_9c_uid59193	NC_011832	chromosome									
	219851537	hypothetical protein Mpal_0888	No	0	YES	917158	917430	-		Methanosphaerula_palustris_E1_9c_uid59193	NC_011832	chromosome		arCOG01665	6 (-)	V	RelE	Cytotoxic translational repressor of toxin-antitoxin stability system			
	219851538	hypothetical protein Mpal_0889	No	0	YES	917469	917630	-		Methanosphaerula_palustris_E1_9c_uid59193	NC_011832	chromosome		arCOG10629	1	S		Uncharacterized conserved protein			
	219851539	hypothetical protein Mpal_0890	No	0	YES	918131	918352	+		Methanosphaerula_palustris_E1_9c_uid59193	NC_011832	chromosome									
	219851540	hypothetical protein Mpal_0893	No	0	YES	918916	919044	+		Methanosphaerula_palustris_E1_9c_uid59193	NC_011832	chromosome									
	219851541	periplasmic copper-binding	Yes	1		919060	920361	+		Methanosphaerula_palustris_E1_9c_uid59193	NC_011832	chromosome		arCOG02545	3 (-)	S		NosD-like periplasmic protein fused to PKD repe	2E-26	pfam05048	Periplasmic copper-binding protein (NosD ; FOG: PKD repeat
	219851542	hypothetical protein Mpal_0895	Yes	0	YES	920438	920542	+		Methanosphaerula_palustris_E1_9c_uid59193	NC_011832	chromosome									
===									7	Methanosphaerula_palustris_E1_9c_uid59193	NC_011832	chromosome									
	219851635	hypothetical protein Mpal_0993	No	0	YES	1022043	1024523	+		Methanosphaerula_palustris_E1_9c_uid59193	NC_011832	chromosome									
	219851636	PAS/PAC sensor signal transduction histidine I	No	0		1024681	1026603	+		Methanosphaerula_palustris_E1_9c_uid59193	NC_011832	chromosome		arCOG02358	4 (-)	T		Signal transduction histidine kinase and HAMP d	8E-67	COG4251	Bacteriophytochrome (light-regulated sig ; KaiB domain. The cyanobacterial clock pr ; FOG: PAS/PAC domain
	219851637	hypothetical protein Mpal_0996	No	0		1027222	1027422	-		Methanosphaerula_palustris_E1_9c_uid59193	NC_011832	chromosome									
	219851638	hypothetical protein Mpal_0997	No	5		1027593	1028231	+		Methanosphaerula_palustris_E1_9c_uid59193	NC_011832	chromosome							0.00001	COG2426	Predicted membrane protein
	219851639	hypothetical protein Mpal_0998	No	0		1028263	1028523	-		Methanosphaerula_palustris_E1_9c_uid59193	NC_011832	chromosome									
	219851640	Carboxymuconolactone decarboxylase	No	0		1028553	1028870	+		Methanosphaerula_palustris_E1_9c_uid59193	NC_011832	chromosome		arCOG02151	1 (-)	S		Uncharacterized homolog of gamma-carboxymuconolactone decarboxylase subunit			
	219851641	hypothetical protein Mpal_1000	No	0		1028912	1029154	+		Methanosphaerula_palustris_E1_9c_uid59193	NC_011832	chromosome									
===									7	Methanosphaerula_palustris_E1_9c_uid59193	NC_011832	chromosome									
	219851799	hypothetical protein Mpal_1165	No	0		1197168	1197383	-		Methanosphaerula_palustris_E1_9c_uid59193	NC_011832	chromosome									
	219851800	multi-sensor signal transduction histidine kinas	No	0		1198748	1200382	+		Methanosphaerula_palustris_E1_9c_uid59193	NC_011832	chromosome		arCOG02385	2 (-)	T	CheY	Rec domain	8E-15	COG2204	Response regulator containing CheY-like ; Osmosensitive K+ channel histidine kinas
	219851801	hypothetical protein Mpal_1168	No	0		1200629	1200727	-		Methanosphaerula_palustris_E1_9c_uid59193	NC_011832	chromosome									
	219851802	hypothetical protein Mpal_1169	No	0		1200724	1201044	-		Methanosphaerula_palustris_E1_9c_uid59193	NC_011832	chromosome	clust_328								
	219851803	hypothetical protein Mpal_1170	No	0		1201472	1201792	-		Methanosphaerula_palustris_E1_9c_uid59193	NC_011832	chromosome	clust_328								
	219851804	hypothetical protein Mpal_1171	No	0		1202379	1202573	-		Methanosphaerula_palustris_E1_9c_uid59193	NC_011832	chromosome									
	219851805	histidine kinase	No	0		1202856	1203722	+		Methanosphaerula_palustris_E1_9c_uid59193	NC_011832	chromosome		arCOG06193	1 (-)	T		Signal transduction histidine kinase and PAS dor	0.00000004	COG3290	Signal transduction histidine kinase reg
===									6	Methanosphaerula_palustris_E1_9c_uid59193	NC_011832	chromosome									
	219851865	hypothetical protein Mpal_1239	No	3		1269412	1269792	+		Methanosphaerula_palustris_E1_9c_uid59193	NC_011832	chromosome		arCOG01467	2 (-)	V		ABC-type multidrug transport system, permease	0.000008	COG0842	ABC-type multidrug transport system, per
	219851866	hypothetical protein Mpal_1240	No	2		1269851	1270120	-		Methanosphaerula_palustris_E1_9c_uid59193	NC_011832	chromosome									
	219851867	hypothetical protein Mpal_1241	No	0		1270352	1270684	-		Methanosphaerula_palustris_E1_9c_uid59193	NC_011832	chromosome									
	219851868	hypothetical protein Mpal_1242	No	1		1270875	1272038	+		Methanosphaerula_palustris_E1_9c_uid59193	NC_011832	chromosome									
	219851869	hypothetical protein Mpal_1243	Yes	2		1272128	1272475	+		Methanosphaerula_palustris_E1_9c_uid59193	NC_011832	chromosome									
	219851870	metal-binding protein-like protein	No	0		1272561	1273211	+		Methanosphaerula_palustris_E1_9c_uid59193	NC_011832	chromosome		arCOG04361	1 (-)	S		Predicted metal-binding protein	1E-39	pfam10050	Predicted metal-binding protein (DUF2284
===									7	Methanosphaerula_palustris_E1_9c_uid59193	NC_011832	chromosome									
	219851897	hypothetical protein Mpal_1272	No	0	YES	1300616	1301098	+		Methanosphaerula_palustris_E1_9c_uid59193	NC_011832	chromosome									
	219851898	NHL repeat containing protein	Yes	1	YES	1301140	1302303	+		Methanosphaerula_palustris_E1_9c_uid59193	NC_011832	chromosome	arCOG03561,a	arCOG03561	1 (-)	S		Uncharacterized conserved protein	2E-11	COG3391	Uncharacterized conserved protein
	219851899	hypothetical protein Mpal_1274	No	0	YES	1302456	1303070	+		Methanosphaerula_palustris_E1_9c_uid59193	NC_011832	chromosome									
	219851900	hypothetical protein Mpal_1275	No	0	YES	1303677	1303799	+		Methanosphaerula_palustris_E1_9c_uid59193	NC_011832	chromosome									
	219851901	hypothetical protein Mpal_1276	No	0	YES	1303789	1303950	+		Methanosphaerula_palustris_E1_9c_uid59193	NC_011832	chromosome									
	219851902	hypothetical protein Mpal_1277	No	0	YES	1303985	1304122	+		Methanosphaerula_palustris_E1_9c_uid59193	NC_011832	chromosome		arCOG09460	1	S		Uncharacterized conserved protein			
	219851903	drug resistance transporter, EmrB/QacA subfar	No	14		1304731	1306149	+		Methanosphaerula_palustris_E1_9c_uid59193	NC_011832	chromosome		arCOG00143	1 (-)	G	ProP	Permease of the major facilitator superfamily	1E-21	pfam07690	Major Facilitator Superfamily.
===									7	Methanosphaerula_palustris_E1_9c_uid59193	NC_011832	chromosome									
	219851961	hypothetical protein Mpal_1337	No	0		1380016	1380666	+		Methanosphaerula_palustris_E1_9c_uid59193	NC_011832	chromosome									
	219851962	prenyltransferase	No	7		1380741	1381598	-		Methanosphaerula_palustris_E1_9c_uid59193	NC_011832	chromosome		arCOG00476	2 (-)	H	UbiA	4-hydroxybenzoate polyprenyltransferase or rela	7E-31	pfam01040	UbiA prenyltransferase family.
	219851963	hypothetical protein Mpal_1339	No	0		1382398	1382937	-		Methanosphaerula_palustris_E1_9c_uid59193	NC_011832	chromosome									
	219851964	hypothetical protein Mpal_1340	No	1		1383647	1383796	+		Methanosphaerula_palustris_E1_9c_uid59193	NC_011832	chromosome									
	219851965	hypothetical protein Mpal_1341	No	0		1383859	1383972	-		Methanosphaerula_palustris_E1_9c_uid59193	NC_011832	chromosome									
	219851966	periplasmic copper-binding	Yes	1		1384256	1387015	+		Methanosphaerula_palustris_E1_9c_uid59193	NC_011832	chromosome		arCOG02545	3 (-)	S		NosD-like periplasmic protein fused to PKD repe	2E-26	pfam05048	Periplasmic copper-binding protein (NosD ; Carbohydrate binding module (family 6). ; Carbohydrate binding module (family 6). ; FOG: PKD repeat
	219851967	hypothetical protein Mpal_1343	No	0		1387024	1387350	-		Methanosphaerula_palustris_E1_9c_uid59193	NC_011832	chromosome									
===									7	Methanosphaerula_palustris_E1_9c_uid59193	NC_011832	chromosome									
	219852003	hypothetical protein Mpal_1382	No	0		1422686	1422940	+		Methanosphaerula_palustris_E1_9c_uid59193	NC_011832	chromosome									
	219852004	hypothetical protein Mpal_1383	No	0		1423040	1426183	-		Methanosphaerula_palustris_E1_9c_uid59193	NC_011832	chromosome		arCOG07809	9 (-)	Y		Viral primase fused to AAA DnaA like ATPAse and Zn finger domain			
	219852005	hypothetical protein Mpal_1384	No	0		1426167	1426556	-		Methanosphaerula_palustris_E1_9c_uid59193	NC_011832	chromosome		arCOG09441	8	L		HJR family endonuclease			
	219852006	hypothetical protein Mpal_1385	No	0		1426566	1426880	-		Methanosphaerula_palustris_E1_9c_uid59193	NC_011832	chromosome	clust_338								
	219852007	hypothetical protein Mpal_1386	No	0		1426972	1428456	+		Methanosphaerula_palustris_E1_9c_uid59193	NC_011832	chromosome									
	219852008	hypothetical protein Mpal_1387	Yes	1		1429038	1429508	+		Methanosphaerula_palustris_E1_9c_uid59193	NC_011832	chromosome		arCOG09465	1 (-)	S		Uncharacterized conserved protein			
	219852009	hypothetical protein Mpal_1388	No	4		1429585	1430307	-		Methanosphaerula_palustris_E1_9c_uid59193	NC_011832	chromosome									
===									5	Methanosphaerula_palustris_E1_9c_uid59193	NC_011832	chromosome									
	219852011	hypothetical protein Mpal_1390	No	3		1433716	1434198	+		Methanosphaerula_palustris_E1_9c_uid59193	NC_011832	chromosome									
	219852012	hypothetical protein Mpal_1391	No	4		1434452	1435159	+		Methanosphaerula_palustris_E1_9c_uid59193	NC_011832	chromosome									
	219852013	Radical SAM domain protein	No	0		1435839	1436972	+		Methanosphaerula_palustris_E1_9c_uid59193	NC_011832	chromosome		arCOG00938	12 (-)	R		Radical SAM superfamily enzyme	4E-19	COG0535	Predicted Fe-S oxidoreductases
	219852014	hypothetical protein Mpal_1393	No	0		1437352	1437471	+		Methanosphaerula_palustris_E1_9c_uid59193	NC_011832	chromosome									
	219852015	Fibronectin type III domain protein	Yes	1		1437682	1441500	+		Methanosphaerula_palustris_E1_9c_uid59193	NC_011832	chromosome									
===									6	Methanosphaerula_palustris_E1_9c_uid59193	NC_011832	chromosome									
	219852251	hypothetical protein Mpal_1648	No	0		1698357	1698665	-		Methanosphaerula_palustris_E1_9c_uid59193	NC_011832	chromosome		arCOG08094	3 (-)	S		Uncharacterized conserved protein	4E-13	pfam13619	KTSC domain. This short domain is named
	219852252	hypothetical protein Mpal_1649	No	0		1698968	1699213	-		Methanosphaerula_palustris_E1_9c_uid59193	NC_011832	chromosome									
	219852253	hypothetical protein Mpal_1650	No	0		1699338	1699676	+		Methanosphaerula_palustris_E1_9c_uid59193	NC_011832	chromosome									
	219852254	hypothetical protein Mpal_1651	No	0		1699673	1700080	+		Methanosphaerula_palustris_E1_9c_uid59193	NC_011832	chromosome									
	219852255	hypothetical protein Mpal_1652	No	1		1700957	1701172	+		Methanosphaerula_palustris_E1_9c_uid59193	NC_011832	chromosome									
	219852256	aldo/keto reductase	No	0		1701418	1702563	+		Methanosphaerula_palustris_E1_9c_uid59193	NC_011832	chromosome		arCOG01624	3 (-)	R		Predicted oxidoreductase of the aldo/keto reduc	2E-144	COG1453	Predicted oxidoreductases of the aldo/ke
===									7	Methanosphaerula_palustris_E1_9c_uid59193	NC_011832	chromosome									
	219852493	hypothetical protein Mpal_1897	No	0		2011701	2011964	+		Methanosphaerula_palustris_E1_9c_uid59193	NC_011832	chromosome									
	219852494	transferase hexapeptide repeat containing prot	No	0		2012114	2012713	+		Methanosphaerula_palustris_E1_9c_uid59193	NC_011832	chromosome		arCOG01851	1 (-)	E	CysE	Serine acetyltransferase	1E-17	COG1045	Serine acetyltransferase
	219852495	hypothetical protein Mpal_1899	No	0		2012802	2013224	-		Methanosphaerula_palustris_E1_9c_uid59193	NC_011832	chromosome									
	219852496	hypothetical protein Mpal_1900	No	1		2013478	2014344	+		Methanosphaerula_palustris_E1_9c_uid59193	NC_011832	chromosome		arCOG08230	1	S		Uncharacterized conserved protein			
	219852497	hypothetical protein Mpal_1901	No	0		2014536	2014766	-		Methanosphaerula_palustris_E1_9c_uid59193	NC_011832	chromosome		arCOG09480	2	S		Uncharacterized conserved protein, DUF2164	6E-14	pfam09932	Uncharacterized conserved protein (DUF21
	219852498	hypothetical protein Mpal_1902	No	0		2015052	2015384	+		Methanosphaerula_palustris_E1_9c_uid59193	NC_011832	chromosome		arCOG09481	1	S		Uncharacterized conserved protein			
	219852499	4Fe-4S ferredoxin iron-sulfur binding domain p	No	0		2015437	2016234	+		Methanosphaerula_palustris_E1_9c_uid59193	NC_011832	chromosome		arCOG02449	1 (-)	C	NapF	Flavodoxin fused to ferredoxin domain	0.0001	COG2768	Uncharacterized Fe-S center protein
===									6	Methanosphaerula_palustris_E1_9c_uid59193	NC_011832	chromosome									
	219852621	hypothetical protein Mpal_2031	No	0		2153864	2154064	+		Methanosphaerula_palustris_E1_9c_uid59193	NC_011832	chromosome	clust_338								
	219852622	hypothetical protein Mpal_2032	No	0		2154074	2154463	+		Methanosphaerula_palustris_E1_9c_uid59193	NC_011832	chromosome		arCOG09441	8	L		HJR family endonuclease			
	219852623	hypothetical protein Mpal_2033	No	0		2154447	2157548	+		Methanosphaerula_palustris_E1_9c_uid59193	NC_011832	chromosome		arCOG07809	9 (-)	Y		Viral primase fused to AAA DnaA like ATPAse and Zn finger domain			
	219852624	hypothetical protein Mpal_2034	No	0		2157688	2158035	+		Methanosphaerula_palustris_E1_9c_uid59193	NC_011832	chromosome									
	219852625	hypothetical protein Mpal_2035	No	0		2158385	2158513	+		Methanosphaerula_palustris_E1_9c_uid59193	NC_011832	chromosome									
	219852626	hypothetical protein Mpal_2036	No	0		2158979	2159218	-		Methanosphaerula_palustris_E1_9c_uid59193	NC_011832	chromosome									
===									8	Methanosphaerula_palustris_E1_9c_uid59193	NC_011832	chromosome									
	219852629	hypothetical protein Mpal_2039	No	0	YES	2161234	2161452	+		Methanosphaerula_palustris_E1_9c_uid59193	NC_011832	chromosome									
	219852630	hypothetical protein Mpal_2040	No	0	YES	2161590	2161832	+		Methanosphaerula_palustris_E1_9c_uid59193	NC_011832	chromosome									
	219852631	hypothetical protein Mpal_2041	No	0	YES	2161846	2162898	+		Methanosphaerula_palustris_E1_9c_uid59193	NC_011832	chromosome		arCOG04956	2 (-)	R		Virulence protein	2E-117	pfam13310	Virulence protein RhuM family. There are
	219852632	hypothetical protein Mpal_2042	No	1	YES	2162888	2163115	+		Methanosphaerula_palustris_E1_9c_uid59193	NC_011832	chromosome									

219852633	hypothetical protein Mpal_2043	No	0	YES	2163160	2164515	+		Methanosphaerula_palustris_E1_9c_uid59193	NC_011832	chromosome			arCOG09531	2	S		Uncharacterized conserved protein			
219852634	protein of unknown function DUF1016	No	0	YES	2164516	2165031	+		Methanosphaerula_palustris_E1_9c_uid59193	NC_011832	chromosome			arCOG03361	1 (-)	R		Predicted nuclease of restriction endonuclease-li	5E-18	pfam06250	Protein of unknown function (DUF1016). F
219852635	hypothetical protein Mpal_2045	No	2	YES	2165091	2165267	-		Methanosphaerula_palustris_E1_9c_uid59193	NC_011832	chromosome										
219852636	hypothetical protein Mpal_2046	No	0	YES	2165337	2165483	-		Methanosphaerula_palustris_E1_9c_uid59193	NC_011832	chromosome										
===								8	Methanosphaerula_palustris_E1_9c_uid59193	NC_011832	chromosome										
219852868	hypothetical protein Mpal_2291	Yes	0		2441691	2441879	+		Methanosphaerula_palustris_E1_9c_uid59193	NC_011832	chromosome										
219852869	hypothetical protein Mpal_2292	No	0		2441962	2442294	+		Methanosphaerula_palustris_E1_9c_uid59193	NC_011832	chromosome			arCOG02516	1 (-)	S		PKD repeats containing protein	0.0000001	COG3291	FOG: PKD repeat
219852870	hypothetical protein Mpal_2293	No	4		2443247	2443756	+		Methanosphaerula_palustris_E1_9c_uid59193	NC_011832	chromosome			arCOG09487	2	S		Uncharacterized conserved protein			
219852871	hypothetical protein Mpal_2294	No	0		2444365	2444628	-		Methanosphaerula_palustris_E1_9c_uid59193	NC_011832	chromosome										
219852872	hypothetical protein Mpal_2295	No	0		2444966	2445082	-		Methanosphaerula_palustris_E1_9c_uid59193	NC_011832	chromosome										
219852873	hypothetical protein Mpal_2296	No	0		2445082	2445297	-		Methanosphaerula_palustris_E1_9c_uid59193	NC_011832	chromosome										
219852874	hypothetical protein Mpal_2297	No	0		2445304	2445402	-		Methanosphaerula_palustris_E1_9c_uid59193	NC_011832	chromosome										
219852875	Exonuclease RNase T and DNA polymerase II	No	0		2445791	2446372	+		Methanosphaerula_palustris_E1_9c_uid59193	NC_011832	chromosome			arCOG05109	2 (-)	L	DnaQ	DNA polymerase III, epsilon subunit or related 3'-	0.000000003	COG0847	DNA polymerase III, epsilon subunit and
===								5	Methanosphaerula_palustris_E1_9c_uid59193	NC_011832	chromosome										
219852998	hypothetical protein Mpal_2428	No	0		2566565	2567194	-		Methanosphaerula_palustris_E1_9c_uid59193	NC_011832	chromosome										
219852999	membrane protein-like protein	No	5		2567200	2567709	-		Methanosphaerula_palustris_E1_9c_uid59193	NC_011832	chromosome			arCOG09488	1	S		Uncharacterized conserved membrane protein, D	0.00006	pfam13515	Fusaric acid resistance protein-like.
219853000	hypothetical protein Mpal_2430	No	4		2567837	2568220	+		Methanosphaerula_palustris_E1_9c_uid59193	NC_011832	chromosome			arCOG09489	1	S		Uncharacterized conserved protein			
219853001	hypothetical protein Mpal_2431	No	0		2568441	2568716	-		Methanosphaerula_palustris_E1_9c_uid59193	NC_011832	chromosome			arCOG01055	9 (-)	K		Predicted transcriptional regulator	0.000002	COG3432	Predicted transcriptional regulator
219853002	hypothetical protein Mpal_2432	No	4		2568713	2569435	-		Methanosphaerula_palustris_E1_9c_uid59193	NC_011832	chromosome			arCOG09490	1	S		Uncharacterized conserved protein			
===								5	Methanosphaerula_palustris_E1_9c_uid59193	NC_011832	chromosome										
219853318	hypothetical protein Mpal_2771	No	0		2899493	2899639	+		Methanosphaerula_palustris_E1_9c_uid59193	NC_011832	chromosome										
219853319	GPR1/FUN34/yaaH family protein	No	6		2899927	2900568	+		Methanosphaerula_palustris_E1_9c_uid59193	NC_011832	chromosome			arCOG03176	1 (-)	S		Predicted membrane protein	7E-47	COG1584	Predicted membrane protein
219853320	hypothetical protein Mpal_2773	No	0		2901347	2901535	+		Methanosphaerula_palustris_E1_9c_uid59193	NC_011832	chromosome										
219853321	hypothetical protein Mpal_2774	No	0		2901562	2902137	+		Methanosphaerula_palustris_E1_9c_uid59193	NC_011832	chromosome										
219853322	hypothetical protein Mpal_2775	No	0		2903045	2903428	+		Methanosphaerula_palustris_E1_9c_uid59193	NC_011832	chromosome										
===								8	Methanospirillum_hungatei_JF_1_uid58181	NC_007796	chromosome										
88601611	hypothetical protein Mhun_0299	Yes	1		337958	338656	-		Methanospirillum_hungatei_JF_1_uid58181	NC_007796	chromosome			arCOG02421	1 (-)	S		Uncharacterized conserved protein	2E-17	pfam07790	Protein of unknown function (DUF1628). T
88601612	hypothetical protein Mhun_0300	Yes	1		338833	339552	+		Methanospirillum_hungatei_JF_1_uid58181	NC_007796	chromosome										
88601613	hypothetical protein Mhun_0301	No	0		340124	340372	+		Methanospirillum_hungatei_JF_1_uid58181	NC_007796	chromosome			arCOG03941	1	S		Uncharacterized conserved protein			
88601614	hypothetical protein Mhun_0302	No	1		340475	340720	-		Methanospirillum_hungatei_JF_1_uid58181	NC_007796	chromosome										
88601615	hypothetical protein Mhun_0303	No	0		340967	341344	+		Methanospirillum_hungatei_JF_1_uid58181	NC_007796	chromosome										
88601616	zinc finger domain-containing protein	No	0		344124	344480	-		Methanospirillum_hungatei_JF_1_uid58181	NC_007796	chromosome			arCOG01121	2 (-)	S		SWIM zinc finger			
88601617	hypothetical protein Mhun_0306	No	0		344480	346060	-		Methanospirillum_hungatei_JF_1_uid58181	NC_007796	chromosome	clust_25									
88601618	hypothetical protein Mhun_0307	No	0		346456	346698	-		Methanospirillum_hungatei_JF_1_uid58181	NC_007796	chromosome										
===								5	Methanospirillum_hungatei_JF_1_uid58181	NC_007796	chromosome										
88601989	glycoside hydrolase family protein	Yes	1		804720	806120	-		Methanospirillum_hungatei_JF_1_uid58181	NC_007796	chromosome								5E-11	pfam01229	Glycosyl hydrolases family 39.
88601990	hypothetical protein Mhun_0696	No	0		806117	807004	-		Methanospirillum_hungatei_JF_1_uid58181	NC_007796	chromosome										
88601991	transposase, IS4	No	0		807043	808653	-		Methanospirillum_hungatei_JF_1_uid58181	NC_007796	chromosome			arCOG03513	2 (-)	L		Transposase	3E-10	COG3666	Transposase and inactivated derivatives ; Transposase DDE domain. Transposase prot ; Chromosome segregation ATPases
88601992	hypothetical protein Mhun_0698	No	1		808691	809068	-		Methanospirillum_hungatei_JF_1_uid58181	NC_007796	chromosome										
88601993	hypothetical protein Mhun_0699	No	0		809187	809690	+		Methanospirillum_hungatei_JF_1_uid58181	NC_007796	chromosome										
===								5	Methanospirillum_hungatei_JF_1_uid58181	NC_007796	chromosome										
88602288	hypothetical protein Mhun_0997	No	0		1122915	1123532	-		Methanospirillum_hungatei_JF_1_uid58181	NC_007796	chromosome										
88602289	hypothetical protein Mhun_0998	No	0		1124307	1124600	-		Methanospirillum_hungatei_JF_1_uid58181	NC_007796	chromosome			arCOG10959	8	S		Uncharacterized conserved protein			
88602290	hypothetical protein Mhun_0999	No	0		1124769	1125212	+		Methanospirillum_hungatei_JF_1_uid58181	NC_007796	chromosome										
88602291	Phage/plasmid primase P4-like protein	No	0		1125628	1127799	-		Methanospirillum_hungatei_JF_1_uid58181	NC_007796	chromosome			arCOG06914	10 (-)	R		Phage or plasmid associated DNA primase	4E-39	COG3378	Phage associated DNA primase
88602292	hypothetical protein Mhun_1001	No	0		1127792	1128172	-		Methanospirillum_hungatei_JF_1_uid58181	NC_007796	chromosome										
===								7	Methanospirillum_hungatei_JF_1_uid58181	NC_007796	chromosome										
88602373	hypothetical protein Mhun_1083	No	0	YES	1223800	1227300	-		Methanospirillum_hungatei_JF_1_uid58181	NC_007796	chromosome			arCOG05219	1 (-)	S		Uncharacterized conserved protein			
88602374	hypothetical protein Mhun_1084	No	0	YES	1227287	1227841	-		Methanospirillum_hungatei_JF_1_uid58181	NC_007796	chromosome										
88602375	hypothetical protein Mhun_1085	No	1	YES	1227838	1228530	-		Methanospirillum_hungatei_JF_1_uid58181	NC_007796	chromosome										
88602376	hypothetical protein Mhun_1086	No	0	YES	1229330	1229620	-		Methanospirillum_hungatei_JF_1_uid58181	NC_007796	chromosome										
88602377	hypothetical protein Mhun_1087	Yes	0	YES	1229795	1230355	-		Methanospirillum_hungatei_JF_1_uid58181	NC_007796	chromosome										
88602378	hypothetical protein Mhun_1088	No	0	YES	1230381	1230884	-		Methanospirillum_hungatei_JF_1_uid58181	NC_007796	chromosome			arCOG06904	7 (-)	S		Uncharacterized conserved protein			
88602379	hypothetical protein Mhun_1089	No	0	YES	1230992	1231555	-		Methanospirillum_hungatei_JF_1_uid58181	NC_007796	chromosome										
===								9	Methanospirillum_hungatei_JF_1_uid58181	NC_007796	chromosome										
88602507	transposase IS66	No	0		1389957	1390892	-		Methanospirillum_hungatei_JF_1_uid58181	NC_007796	chromosome			arCOG04792	9 (-)	L		Transposase	0.000000006	pfam03050	Transposase IS66 family. Transposase pro
88602508	hypothetical protein Mhun_1221	No	0		1392129	1393163	-		Methanospirillum_hungatei_JF_1_uid58181	NC_007796	chromosome	clust_4									
88602509	hypothetical protein Mhun_1222	No	0		1393278	1394261	-		Methanospirillum_hungatei_JF_1_uid58181	NC_007796	chromosome	clust_4									
88602510	hypothetical protein Mhun_1223	No	0		1394376	1395395	-		Methanospirillum_hungatei_JF_1_uid58181	NC_007796	chromosome	clust_4									
88602511	hypothetical protein Mhun_1224	No	0		1395510	1396517	-		Methanospirillum_hungatei_JF_1_uid58181	NC_007796	chromosome	clust_4									
88602512	hypothetical protein Mhun_1225	No	0		1396617	1397636	-		Methanospirillum_hungatei_JF_1_uid58181	NC_007796	chromosome	clust_4									
88602513	hypothetical protein Mhun_1226	No	0		1397756	1398775	-		Methanospirillum_hungatei_JF_1_uid58181	NC_007796	chromosome	clust_4									
88602514	hypothetical protein Mhun_1227	No	0		1398891	1399979	-		Methanospirillum_hungatei_JF_1_uid58181	NC_007796	chromosome	clust_4									
88602515	hypothetical protein Mhun_1228	No	1		1400062	1400670	-		Methanospirillum_hungatei_JF_1_uid58181	NC_007796	chromosome			arCOG05300	1 (-)	S		Uncharacterized conserved protein			
===								6	Methanospirillum_hungatei_JF_1_uid58181	NC_007796	chromosome										
88602661	hypothetical protein Mhun_1380	No	0	YES	1577739	1579337	-		Methanospirillum_hungatei_JF_1_uid58181	NC_007796	chromosome			arCOG04928	1 (-)	V	Cas8/Cse	CRISPR system related protein, large subunit of (	5E-70	pfam09481	CRISPR-associated protein Cse1 (CRISPR_c
88602662	hypothetical protein Mhun_1383	No	0	YES	1583020	1583580	+		Methanospirillum_hungatei_JF_1_uid58181	NC_007796	chromosome										
88602663	hypothetical protein Mhun_1384	No	0	YES	1584064	1584384	-		Methanospirillum_hungatei_JF_1_uid58181	NC_007796	chromosome										
88602664	hypothetical protein Mhun_1385	No	0	YES	1584548	1584856	+		Methanospirillum_hungatei_JF_1_uid58181	NC_007796	chromosome										
88602665	hypothetical protein Mhun_1386	No	0	YES	1585235	1585495	+		Methanospirillum_hungatei_JF_1_uid58181	NC_007796	chromosome										
88602666	PilT protein-like protein	No	0	YES	1585577	1585987	-		Methanospirillum_hungatei_JF_1_uid58181	NC_007796	chromosome			arCOG00713	1 (-)	V		PIN domain containing protein	0.0000002	COG1848	Predicted nucleic acid-binding protein,
===								9	Methanospirillum_hungatei_JF_1_uid58181	NC_007796	chromosome										
88602672	hypothetical protein Mhun_1393	Yes	0	YES	1590591	1591142	+		Methanospirillum_hungatei_JF_1_uid58181	NC_007796	chromosome										
88602673	hypothetical protein Mhun_1394	No	0	YES	1592044	1592373	+		Methanospirillum_hungatei_JF_1_uid58181	NC_007796	chromosome			arCOG09441	8	L		HJR family endonuclease			
88602674	hypothetical protein Mhun_1395	No	0	YES	1592370	1592633	+		Methanospirillum_hungatei_JF_1_uid58181	NC_007796	chromosome	clust_357									
88602675	hypothetical protein Mhun_1396	No	0		1592643	1595885	+		Methanospirillum_hungatei_JF_1_uid58181	NC_007796	chromosome		arCOG07809,a	arCOG07809	9 (-)	Y		Viral primase fused to AAA DnaA like ATPase and Zn finger domain			
88602676	hypothetical protein Mhun_1397	No	0	YES	1595875	1596213	+		Methanospirillum_hungatei_JF_1_uid58181	NC_007796	chromosome	clust_312									
88602677	putative transposase	No	0	YES	1596267	1597136	+		Methanospirillum_hungatei_JF_1_uid58181	NC_007796	chromosome								3E-23	pfam04754	Putative transposase, YhgA-like. This fa
88602678	putative transposase	No	0	YES	1597344	1598225	+		Methanospirillum_hungatei_JF_1_uid58181	NC_007796	chromosome			arCOG10868	7	L		PD-(D/E)XK nuclease family transposase	8E-24	pfam04754	Putative transposase, YhgA-like. This fa
88602679	putative transposase	No	0	YES	1598434	1599339	+		Methanospirillum_hungatei_JF_1_uid58181	NC_007796	chromosome			arCOG10868	7	L		PD-(D/E)XK nuclease family transposase	2E-23	pfam04754	Putative transposase, YhgA-like. This fa
88602680	ATPase	No	0	YES	1599604	1600194	+		Methanospirillum_hungatei_JF_1_uid58181	NC_007796	chromosome			arCOG03167	6 (-)	R		Predicted ATPase (AAA+ superfamily)	7E-26	pfam13173	AAA domain. This family of domains conta
===								7	Methanospirillum_hungatei_JF_1_uid58181	NC_007796	chromosome										
88602848	hypothetical protein Mhun_1576	No	2		1802999	1803268	-		Methanospirillum_hungatei_JF_1_uid58181	NC_007796	chromosome										
88602849	hypothetical protein Mhun_1577	Yes	1		1803355	1804479	+		Methanospirillum_hungatei_JF_1_uid58181	NC_007796	chromosome		arCOG03259,a	arCOG03259	1 (-)	S		Cell suface protein			
88602850	hypothetical protein Mhun_1578	No	3		1804484	1804807	-		Methanospirillum_hungatei_JF_1_uid58181	NC_007796	chromosome										
88602851	hypothetical protein Mhun_1579	Yes	1		1804820	1805383	-		Methanospirillum_hungatei_JF_1_uid58181	NC_007796	chromosome										
88602852	hypothetical protein Mhun_1580	Yes	0		1805441	1806082	-		Methanospirillum_hungatei_JF_1_uid58181	NC_007796	chromosome										
88602853	hypothetical protein Mhun_1581	No	0		1807086	1807787	+		Methanospirillum_hungatei_JF_1_uid58181	NC_007796	chromosome										
88602854	NUDIX hydrolase	No	0		1807784	1808569	-		Methanospirillum_hungatei_JF_1_uid58181	NC_007796	chromosome			arCOG01076	1 (-)	L	NPY1	NTP pyrophosphohydrolase containing a Zn-finge	7E-55	COG2816	NTP pyrophosphohydrolases containing a Z
===								7	Methanospirillum_hungatei_JF_1_uid58181	NC_007796	chromosome										
88602929	hypothetical protein Mhun_1657	No	0	YES	1878195	1878806	+		Methanospirillum_hungatei_JF_1_uid58181	NC_007796	chromosome			arCOG02123	2 (-)	V		HEPN domain containing protein	2E-16	COG1895	Uncharacterized conserved protein relate
88602930	hypothetical protein Mhun_1658	No	0	YES	1878994	1881078	-		Methanospirillum_hungatei_JF_1_uid58181	NC_007796	chromosome										
88602931	hypothetical protein Mhun_1659	No	0	YES	1881075	1882049	-		Methanospirillum_hungatei_JF_1_uid58181	NC_007796	chromosome										
88602932	hypothetical protein Mhun_1660	No	0	YES	1882326	1883219	+		Methanospirillum_hungatei_JF_1_uid58181	NC_007796	chromosome										
88602933	hypothetical protein Mhun_1661	No	0	YES	1883409	1884704	-		Methanospirillum_hungatei_JF_1_uid58181	NC_007796	chromosome										
88602934	hypothetical protein Mhun_1662	No	0	YES	1885613	1885888	+		Methanospirillum_hungatei_JF_1_uid58181	NC_007796	chromosome										
88602935	PilT protein-like protein	No	0	YES	1885878	1886240	+		Methanospirillum_hungatei_JF_1_uid58181	NC_007796	chromosome			arCOG02225	2 (-)	V	VapC	PIN domain containing protein	0.00000003	COG1487	Predicted nucleic acid-binding protein,
===								5	Methanospirillum_hungatei_JF_1_uid58181	NC_007796	chromosome										
88603107	hypothetical protein Mhun_1849	No	0		2085498	2085764	+		Methanospirillum_hungatei_JF_1_uid58181	NC_007796	chromosome										
88603108	shikimate kinase	No	0		2086364	2086888	+		Methanospirillum_hungatei_JF_1_uid58181	NC_007796	chromosome			arCOG01047	1 (-)	E	AroK	Shikimate kinase	2E-26	COG0703	Shikimate kinase
88603109	hypothetical protein Mhun_1851	No	0	YES	2086851	2088569	-		Methanospirillum_hungatei_JF_1_uid58181	NC_007796	chromosome	clust_69							5E-37	pfam09820	Predicted AAA-ATPase. This family contai
88603110	hypothetical protein Mhun_1852	No	0	YES	2089653	2089937	-		Methanospirillum_hungatei_JF_1_uid58181	NC_007796	chromosome										
88603111	hypothetical protein Mhun_1853	No	0	YES	2090328	2090591	-		Methanospirillum_hungatei_JF_1_uid58181	NC_007796	chromosome										
===								9	Methanospirillum_hungatei_JF_1_uid58181	NC_007796	chromosome										
88603139	hypothetical protein Mhun_1881	No	0	YES	2126201	2126515	-		Methanospirillum_hungatei_JF_1_uid58181	NC_007796	chromosome	clust_312									
88603140	hypothetical protein Mhun_1882	No	0		2126515	2129691	-		Methanospirillum_hungatei_JF_1_uid58181	NC_007796	chromosome		arCOG07809,a	arCOG07809	9 (-)	Y		Viral primase fused to AAA DnaA like ATPase and Zn finger domain			
88603141	hypothetical protein Mhun_1883	No	0		2129688	2129978	-		Methanospirillum_hungatei_JF_1_uid58181	NC_007796	chromosome	clust_357									
88603142	hypothetical protein Mhun_1884	No	0		2129975	2130307	-		Methanospirillum_hungatei_JF_1_uid58181	NC_007796	chromosome			arCOG09441	8	L		HJR family endonuclease			
88603143	hypothetical protein Mhun_1885	No	0		2130428	2130721	-		Methanospirillum_hungatei_JF_1_uid58181	NC_007796	chromosome										
88603144	hypothetical protein Mhun_1886	No	0		2131080	2132822	+		Methanospirillum_hungatei_JF_1_uid58181	NC_007796	chromosome	clust_69							8E-42	pfam09820	Predicted AAA-ATPase. This family contai ; Protein of unknown function (DUF1703). T
88603145	tetratricopeptide TPR_2	No	0		2133028	2133351	-		Methanospirillum_hungatei_JF_1_uid58181	NC_007796	chromosome			arCOG03032	12 (-)	R	NrfG	TPR repeats containing protein	5E-11	COG0457	FOG: TPR repeat
88603146	hypothetical protein Mhun_1888	No	0	YES	2133448	2133729	-		Methanospirillum_hungatei_JF_1_uid58181	NC_007796	chromosome										
88603147	hypothetical protein Mhun_1889	No	0	YES	2133889	2134482	-		Methanospirillum_hungatei_JF_1_uid58181	NC_007796	chromosome										
===								6	Methanospirillum_hungatei_JF_1_uid58181	NC_007796	chromosome										
88603303	extracellular solute-binding protein	Yes	1		2319628	2320404	+		Methanospirillum_hungatei_JF_1_uid58181	NC_007796	chromosome			arCOG01799	1 (-)	E	HisJ	ABC-type amino acid transport/signal transductio	8E-34	pfam00497	Bacterial extracellular solute-binding p
88603304	hypothetical protein Mhun_2055	No	0		2320360	2320602	-		Methanospirillum_hungatei_JF_1_uid58181	NC_007796	chromosome										
88603305	hypothetical protein Mhun_2056	Yes	0		2320750	2321310	+		Methanospirillum_hungatei_JF_1_uid58181	NC_007796	chromosome										
88603306	hypothetical protein Mhun_2057	No	0		2321745	2323331	+		Methanospirillum_hungatei_JF_1_uid58181	NC_007796	chromosome	clust_25									
88603307	hypothetical protein Mhun_2058	No	1		2323460	2325163	+		Methanospirillum_hungatei_JF_1_uid58181	NC_007796	chromosome	clust_25									
88603308	excisionase/Xis, DNA-binding	No	0		2326519	2326836	+		Methanospirillum_hungatei_JF_1_uid58181	NC_007796	chromosome			arCOG09496	1 (-)	K		Helix-Turn-Helix DNA binding domain of truncated MerR-like family			
===								5	Methanospirillum_hungatei_JF_1_uid58181	NC_007796	chromosome										
88603367	hypothetical protein Mhun_2119	No	0		2391801	2392124	-		Methanospirillum_hungatei_JF_1_uid58181	NC_007796	chromosome										
88603368	hypothetical protein Mhun_2120	No	0		2392238	2392384	+		Methanospirillum_hungatei_JF_1_uid58181	NC_007796	chromosome										
88603369	ABC transporter-like protein	No	6		2392980	2394758	+		Methanospirillum_hungatei_JF_1_uid58181	NC_007796	chromosome			arCOG02841	4 (-)	V	MdlB	ABC-type multidrug transport system, ATPase an	3E-90	COG1132	ABC-type multidrug transport system, ATP
88603370	NDP-hexose 3-C-methyltransferase TylCIII	No	0		2395543	2396772	+		Methanospirillum_hungatei_JF_1_uid58181	NC_007796	chromosome								9E-33	pfam08484	C-methyltransferase C-terminal domain. T ; Putative zinc binding domain. This domai
88603371	hypothetical protein Mhun_2123	No	0		2396899	2398161	+		Methanospirillum_hungatei_JF_1_uid58181	NC_007796	chromosome										
===								6	Methanospirillum_hungatei_JF_1_uid58181	NC_007796	chromosome										
88603670	hypothetical protein Mhun_2428	No	1		2686419	2687936	-		Methanospirillum_hungatei_JF_1_uid58181	NC_007796	chromosome			arCOG02911	3 (-)	S		Uncharacterized conserved membrane protein			
88603671	hypothetical protein Mhun_2429	No	0		2688341	2689261	-		Methanospirillum_hungatei_JF_1_uid58181	NC_007796	chromosome	clust_14							5E-45	pfam12784	PD-(D/E)XK nuclease family transposase.
88603672	hypothetical protein Mhun_2430	No	0		2689826	2690701	-		Methanospirillum_hungatei_JF_1_uid58181	NC_007796	chromosome			arCOG10868	7	L		PD-(D/E)XK nuclease family transposase			
88603673	hypothetical protein Mhun_2431	No	0		2690748	2691002	-		Methanospirillum_hungatei_JF_1_uid58181	NC_007796	chromosome										
88603674	hypothetical protein Mhun_2432	No	0		2691201	2691980	-		Methanospirillum_hungatei_JF_1_uid58181	NC_007796	chromosome			arCOG10868	7	L		PD-(D/E)XK nuclease family transposase	1E-26	pfam12784	PD-(D/E)XK nuclease family transposase.
88603675	catalase/peroxidase HPI	No	0		2692142	2694298	+		Methanospirillum_hungatei_JF_1_uid58181	NC_007796	chromosome			arCOG04487	2 (-)	P	KatG	Catalase (peroxidase I)		COG0376	Catalase (peroxidase I)
===								5	Methanospirillum_hungatei_JF_1_uid58181	NC_007796	chromosome										
88603899	hypothetical protein Mhun_2661	Yes	1		2933969	2934298	-		Methanospirillum_hungatei_JF_1_uid58181	NC_007796	chromosome										
88603900	hypothetical protein Mhun_2662	No	2		2934309	2934560	-		Methanospirillum_hungatei_JF_1_uid58181	NC_007796	chromosome			arCOG04003	2	S		Uncharacterized conserved protein			
88603901	ATPase, E1-E2 type	No	9		2934823	2937414	+		Methanospirillum_hungatei_JF_1_uid58181	NC_007796	chromosome			arCOG01578	1 (-)	P	MgtA	Cation transport ATPase	3E-152	COG0474	Cation transport ATPase
88603902	hypothetical protein Mhun_2664	Yes	1	YES	2937426	2937749	-		Methanospirillum_hungatei_JF_1_uid58181	NC_007796	chromosome										
88603903	hypothetical protein Mhun_2665	Yes	1	YES	2937755	2938030	-		Methanospirillum_hungatei_JF_1_uid58181	NC_007796	chromosome										
===								8	Methanospirillum_hungatei_JF_1_uid58181	NC_007796	chromosome										
88603928	hypothetical protein Mhun_2692	No	6		2965549	2966187	+		Methanospirillum_hungatei_JF_1_uid58181	NC_007796	chromosome										
88603929	PAS/PAC sensor signal transduction histidine l	No	0		2966386	2968569	+		Methanospirillum_hungatei_JF_1_uid58181	NC_007796	chromosome		arCOG02329,a	arCOG02329	1 (-)	T		Signal transduction histidine kinase with PAS and	4E-24	COG2202	FOG: PAS/PAC domain
88603930	hypothetical protein Mhun_2694	No	0	YES	2968657	2969538	+		Methanospirillum_hungatei_JF_1_uid58181	NC_007796	chromosome	clust_14							3E-43	pfam12784	PD-(D/E)XK nuclease family transposase.
88603931	hypothetical protein Mhun_2695	No	0	YES	2969834	2970748	+		Methanospirillum_hungatei_JF_1_uid58181	NC_007796	chromosome	clust_14							4E-43	pfam12784	PD-(D/E)XK nuclease family transposase.
88603932	hypothetical protein Mhun_2696	No	0	YES	2971055	2971930	+		Methanospirillum_hungatei_JF_1_uid58181	NC_007796	chromosome	clust_14							1E-43	pfam12784	PD-(D/E)XK nuclease family transposase.
88603933	hypothetical protein Mhun_2697	No	0	YES	2971983	2972633	+		Methanospirillum_hungatei_JF_1_uid58181	NC_007796	chromosome										
88603934	hypothetical protein Mhun_2698	No	1	YES	2973952	2975730	+		Methanospirillum_hungatei_JF_1_uid58181	NC_007796	chromosome			arCOG02911	3 (-)	S		Uncharacterized conserved membrane protein			
88603935	hypothetical protein Mhun_2699	No	1	YES	2975730	2976383	+		Methanospirillum_hungatei_JF_1_uid58181	NC_007796	chromosome										
===								5	Methanospirillum_hungatei_JF_1_uid58181	NC_007796	chromosome										
88603957	DNA polymerase, beta-like region	No	0	YES	2992561	2992842	+		Methanospirillum_hungatei_JF_1_uid58181	NC_007796	chromosome										
88603958	hypothetical protein Mhun_2722	No	0	YES	2992839	2993291	+		Methanospirillum_hungatei_JF_1_uid58181	NC_007796	chromosome										
88603959	hypothetical protein Mhun_2723	No	0	YES	2993326	2993802	-		Methanospirillum_hungatei_JF_1_uid58181	NC_007796	chromosome								5E-15	pfam09820	Predicted AAA-ATPase. This family contai
88603960	5'-nucleotidase-like protein	No	1		2993842	2995587	+		Methanospirillum_hungatei_JF_1_uid58181	NC_007796	chromosome			arCOG02832	1 (-)	F	UshA	5'-nucleotidase/2',3'-cyclic phosphodiesterase or	8E-38	COG0737	5'-nucleotidase/2',3'-cyclic phosphodies
88603961	protein kinase/helix-hairpin-helix DNA-binding (	No	0		2995624	2998989	-		Methanospirillum_hungatei_JF_1_uid58181	NC_007796	chromosome								2E-28	COG4248	Uncharacterized protein with protein kin
===								6	Methanospirillum_hungatei_JF_1_uid58181	NC_007796	chromosome										
88603980	HEAT repeat-containing PBS lyase	No	0		3012674	3013039	-		Methanospirillum_hungatei_JF_1_uid58181	NC_007796	chromosome			arCOG04006	3 (-)	S		HEAT repeats containing protein	2E-14	pfam13646	HEAT repeats. This family includes multi
88603981	hypothetical protein Mhun_2745	No	0		3013550	3013963	+		Methanospirillum_hungatei_JF_1_uid58181	NC_007796	chromosome	clust_39									
88603982	hypothetical protein Mhun_2746	No	0		3014307	3014717	+		Methanospirillum_hungatei_JF_1_uid58181	NC_007796	chromosome	clust_39									
88603983	hypothetical protein Mhun_2747	No	0		3014842	3015726	+		Methanospirillum_hungatei_JF_1_uid58181	NC_007796	chromosome										
88603984	hypothetical protein Mhun_2748	No	0		3015956	3016366	+		Methanospirillum_hungatei_JF_1_uid58181	NC_007796	chromosome	clust_39									
88603985	response regulator receiver domain-containing	No	0		3016562	3017872	+		Methanospirillum_hungatei_JF_1_uid58181	NC_007796	chromosome			arCOG02378	1 (-)	T		Signal transduction histidine kinase, contains RE(	8E-22	pfam00072	Response regulator receiver domain. This
===								8	Methanospirillum_hungatei_JF_1_uid58181	NC_007796	chromosome										
88604074	hypothetical protein Mhun_2841	No	0		3121635	3122423	-		Methanospirillum_hungatei_JF_1_uid58181	NC_007796	chromosome										
88604075	hypothetical protein Mhun_2842	No	0		3122571	3123167	+		Methanospirillum_hungatei_JF_1_uid58181	NC_007796	chromosome			arCOG06915	3 (-)	L		Primase-polymerase (primpol) domain			
88604076	hypothetical protein Mhun_2843	No	0		3123254	3124981	+		Methanospirillum_hungatei_JF_1_uid58181	NC_007796	chromosome										
88604077	AsnC family transcriptional regulator	No	4		3125461	3126159	+		Methanospirillum_hungatei_JF_1_uid58181	NC_007796	chromosome								6E-11	pfam10935	Protein of unknown function (DUF2637). T
88604078	hypothetical protein Mhun_2845	No	0		3126278	3126538	+		Methanospirillum_hungatei_JF_1_uid58181	NC_007796	chromosome			arCOG10959	8	S		Uncharacterized conserved protein			
88604079	hypothetical protein Mhun_2846	No	0		3128201	3128719	+		Methanospirillum_hungatei_JF_1_uid58181	NC_007796	chromosome	clust_203							0.000002	COG0419	ATPase involved in DNA repair
88604080	hypothetical protein Mhun_2847	No	0		3130169	3130687	+		Methanospirillum_hungatei_JF_1_uid58181	NC_007796	chromosome	clust_203							0.000002	COG0419	ATPase involved in DNA repair
88604081	HAD family hydrolase	No	0		3131046	3131819	+		Methanospirillum_hungatei_JF_1_uid58181	NC_007796	chromosome			arCOG04221	2 (-)	G	NagD	Phosphatase of the HAD superfamily	8E-28	COG0647	Predicted sugar phosphatases of the HAD
===								7	Methanothermobacter_marburgensis_Marburg_ui	NC_014408	chromosome										
304314494	nickel responsive regulator	No	0		665806	666234	+		Methanothermobacter_marburgensis_Marburg_ui	NC_014408	chromosome			arCOG01008	7 (-)	V	NikR	Transcriptional regulator, CopG/Arc/MetJ family (I	2E-43	COG0864	Predicted transcriptional regulators con
304314495	hypothetical protein MTBMA_c07340	No	0		666535	666750	-		Methanothermobacter_marburgensis_Marburg_ui	NC_014408	chromosome	clust_199									
304314496	hypothetical protein MTBMA_c07350	No	1		666758	667285	-		Methanothermobacter_marburgensis_Marburg_ui	NC_014408	chromosome										
304314497	hypothetical protein MTBMA_c07360	No	1		667299	667784	-		Methanothermobacter_marburgensis_Marburg_ui	NC_014408	chromosome										
304314498	hypothetical protein MTBMA_c07370	Yes	1		668024	668464	-		Methanothermobacter_marburgensis_Marburg_ui	NC_014408	chromosome										
304314499	hypothetical protein MTBMA_c07380	No	1		668550	669017	+		Methanothermobacter_marburgensis_Marburg_ui	NC_014408	chromosome										
304314500	hypothetical protein MTBMA_c07390	No	0		669085	670959	-		Methanothermobacter_marburgensis_Marburg_ui	NC_014408	chromosome			arCOG10127	2 (-)	R		KAP-like ATPase	3E-32	pfam07693	KAP family P-loop domain. The KAP (after
===								14	Methanothermobacter_marburgensis_Marburg_ui	NC_014408	chromosome										
304314861	hypothetical protein MTBMA_c11010	No	0		1021254	1021439	+		Methanothermobacter_marburgensis_Marburg_ui	NC_014408	chromosome										
304314862	methionine-sulfoxide reductase	No	0		1021554	1021961	+		Methanothermobacter_marburgensis_Marburg_ui	NC_014408	chromosome			arCOG02815	2 (-)	O		Conserved domain frequently associated with pe	5E-48	pfam01641	SelR domain. Methionine sulfoxide reduct
304314863	hypothetical protein MTBMA_c11030	No	2		1021992	1022222	+		Methanothermobacter_marburgensis_Marburg_ui	NC_014408	chromosome			arCOG10455	2	S		Uncharacterized conserved protein			
304314864	hypothetical protein MTBMA_c11040	No	0		1022368	1022727	-		Methanothermobacter_marburgensis_Marburg_ui	NC_014408	chromosome										
304314865	hypothetical protein MTBMA_c11050	No	2		1022952	1023197	-		Methanothermobacter_marburgensis_Marburg_ui	NC_014408	chromosome										
304314866	hypothetical protein MTBMA_c11060	No	0		1023471	1023731	-		Methanothermobacter_marburgensis_Marburg_ui	NC_014408	chromosome										
304314867	hypothetical protein MTBMA_c11070	No	1		1023849	1024232	-		Methanothermobacter_marburgensis_Marburg_ui	NC_014408	chromosome			arCOG10864	11	M		Peptidase C39			
304314868	hypothetical protein MTBMA_c11080	No	3		1024387	1024626	-		Methanothermobacter_marburgensis_Marburg_ui	NC_014408	chromosome	clust_400									
304314869	hypothetical protein MTBMA_c11090	No	0		1024643	1025041	-		Methanothermobacter_marburgensis_Marburg_ui	NC_014408	chromosome			arCOG10864	11	M		Peptidase C39			
304314870	hypothetical protein MTBMA_c11100	No	3		1025162	1025407	-		Methanothermobacter_marburgensis_Marburg_ui	NC_014408	chromosome	clust_7									
304314871	hypothetical protein MTBMA_c11110	No	2		1025520	1026167	-		Methanothermobacter_marburgensis_Marburg_ui	NC_014408	chromosome			arCOG10864	11	M		Peptidase C39			
304314872	tetratricopeptide repeat domain-containing prot	No	0		1026328	1027548	-		Methanothermobacter_marburgensis_Marburg_ui	NC_014408	chromosome			arCOG03038	9 (-)	R	NrfG	TPR repeats containing protein	4E-50	COG0457	FOG: TPR repeat
304314873	hypothetical protein MTBMA_c11130	No	0		1027545	1028495	-		Methanothermobacter_marburgensis_Marburg_ui	NC_014408	chromosome	clust_102									
304314874	hypothetical protein MTBMA_c11140	No	3		1028512	1028751	-		Methanothermobacter_marburgensis_Marburg_ui	NC_014408	chromosome	clust_7									
===								7	Methanothermobacter_marburgensis_Marburg_ui	NC_014408	chromosome										
304315209	hypothetical protein MTBMA_c14640	No	0		1346764	1347471	+		Methanothermobacter_marburgensis_Marburg_ui	NC_014408	chromosome			arCOG04855	1 (-)	Q		SAM-dependent methyltransferase			
304315210	hypothetical protein MTBMA_c14650	Yes	1		1347492	1347845	+		Methanothermobacter_marburgensis_Marburg_ui	NC_014408	chromosome										
304315211	hypothetical protein MTBMA_c14660	No	0		1348012	1348311	+		Methanothermobacter_marburgensis_Marburg_ui	NC_014408	chromosome										
304315212	hypothetical protein MTBMA_c14670	No	0		1348455	1348616	-		Methanothermobacter_marburgensis_Marburg_ui	NC_014408	chromosome										
304315213	hypothetical protein MTBMA_c14680	No	0		1348776	1348967	-		Methanothermobacter_marburgensis_Marburg_ui	NC_014408	chromosome										
304315214	type I restriction-modification enzyme, subunit	No	0		1349186	1352074	-		Methanothermobacter_marburgensis_Marburg_ui	NC_014408	chromosome			arCOG00879	2 (-)	V		Type I site-specific restriction-modification systen	6E-46	COG0610	Type I site-specific restriction-modific
304315215	hypothetical protein MTBMA_c14700	No	0		1352123	1352983	-		Methanothermobacter_marburgensis_Marburg_ui	NC_014408	chromosome										
===								5	Methanothermobacter_marburgensis_Marburg_ui	NC_014408	chromosome										
304315333	hypothetical protein MTBMA_c15880	No	3		1464101	1464517	+		Methanothermobacter_marburgensis_Marburg_ui	NC_014408	chromosome	clust_252									
304315334	hypothetical protein MTBMA_c15890	No	3		1464612	1464848	+		Methanothermobacter_marburgensis_Marburg_ui	NC_014408	chromosome	clust_7									
304315335	hypothetical protein MTBMA_c15900	No	0		1464983	1465546	+		Methanothermobacter_marburgensis_Marburg_ui	NC_014408	chromosome										
304315336	tetratricopeptide repeat domain-containing prot	No	1		1465548	1466171	+		Methanothermobacter_marburgensis_Marburg_ui	NC_014408	chromosome			arCOG03038	9 (-)	R	NrfG	TPR repeats containing protein	2E-24	COG0457	FOG: TPR repeat
304315337	hypothetical protein MTBMA_c15920	No	0		1466196	1466639	+		Methanothermobacter_marburgensis_Marburg_ui	NC_014408	chromosome										
===								8	Methanothermobacter_marburgensis_Marburg_ui	NC_014408	chromosome										
304315463	hypothetical protein MTBMA_c17290	No	3		1566036	1566428	+		Methanothermobacter_marburgensis_Marburg_ui	NC_014408	chromosome										
304315464	hypothetical protein MTBMA_c17300	Yes	1		1566692	1567549	+		Methanothermobacter_marburgensis_Marburg_ui	NC_014408	chromosome			arCOG06761	2 (-)	R		Predicted double-glycine peptidase	1E-21	pfam03412	Peptidase C39 family. Lantibiotic and no
304315465	hypothetical protein MTBMA_c17310	No	1		1567555	1568034	+		Methanothermobacter_marburgensis_Marburg_ui	NC_014408	chromosome										
304315466	hypothetical protein MTBMA_c17320	No	4		1568121	1568552	+		Methanothermobacter_marburgensis_Marburg_ui	NC_014408	chromosome	clust_232									
304315467	hypothetical protein MTBMA_c17330	No	0		1568642	1568803	+		Methanothermobacter_marburgensis_Marburg_ui	NC_014408	chromosome										
304315468	hypothetical protein MTBMA_c17340	No	3		1568804	1569136	+		Methanothermobacter_marburgensis_Marburg_ui	NC_014408	chromosome										
304315469	hypothetical protein MTBMA_c17350	No	4		1569315	1569671	+		Methanothermobacter_marburgensis_Marburg_ui	NC_014408	chromosome										
304315470	FwdE-related protein	No	0		1569781	1570296	-		Methanothermobacter_marburgensis_Marburg_ui	NC_014408	chromosome			arCOG00762	1 (-)	C		Formylmethanofuran dehydrogenase subunit E	8E-41	COG2191	Formylmethanofuran dehydrogenase subunit
===								5	Methanothermobacter_thermautotrophicus_Delta_	NC_000916	chromosome										
15678086	hypothetical protein MTH57	No	2		35825	36208	-		Methanothermobacter_thermautotrophicus_Delta_	NC_000916	chromosome										
15678087	hypothetical protein MTH58	No	1		36384	36641	-		Methanothermobacter_thermautotrophicus_Delta_	NC_000916	chromosome										
15678088	hypothetical protein MTH59	Yes	1		36638	37219	-		Methanothermobacter_thermautotrophicus_Delta_	NC_000916	chromosome			arCOG02566	1 (-)	S		Uncharacterized conserved protein			
15678089	hypothetical protein MTH60	Yes	1		37308	37838	-		Methanothermobacter_thermautotrophicus_Delta_	NC_000916	chromosome			arCOG10348	1	S		Uncharacterized conserved protein			
15678090	hypothetical protein MTH61	Yes	1		38015	38500	-		Methanothermobacter_thermautotrophicus_Delta_	NC_000916	chromosome			arCOG10347	1	S		Uncharacterized conserved protein			
===								14	Methanothermobacter_thermautotrophicus_Delta_	NC_000916	chromosome										
15678097	hypothetical protein MTH69	No	3		42385	42642	-		Methanothermobacter_thermautotrophicus_Delta_	NC_000916	chromosome										
15678098	hypothetical protein MTH70	No	0		42661	42846	-		Methanothermobacter_thermautotrophicus_Delta_	NC_000916	chromosome			arCOG10864	11	M		Peptidase C39			
15678099	hypothetical protein MTH71	No	1		42843	43109	-		Methanothermobacter_thermautotrophicus_Delta_	NC_000916	chromosome										
15678100	O-linked GlcNAc transferase	No	0		43227	44438	-		Methanothermobacter_thermautotrophicus_Delta_	NC_000916	chromosome			arCOG03042	2 (-)	R	NrfG	TPR repeats containing protein	4E-42	COG0457	FOG: TPR repeat
15678101	hypothetical protein MTH73	No	0		44435	45385	-		Methanothermobacter_thermautotrophicus_Delta_	NC_000916	chromosome	clust_102									
15678102	hypothetical protein MTH74	No	3		45402	45677	-		Methanothermobacter_thermautotrophicus_Delta_	NC_000916	chromosome	clust_7									
15678103	surface protease related protein	Yes	1		45658	48171	-		Methanothermobacter_thermautotrophicus_Delta_	NC_000916	chromosome			arCOG03946	3 (-)	S		Uncharacterized conserved protein	2E-10	COG3291	FOG: PKD repeat
15678104	hypothetical protein MTH76	No	0		48369	48749	-		Methanothermobacter_thermautotrophicus_Delta_	NC_000916	chromosome										
15678105	hypothetical protein MTH77	No	2		48867	49274	-		Methanothermobacter_thermautotrophicus_Delta_	NC_000916	chromosome			arCOG10864	11	M		Peptidase C39			
15678106	hypothetical protein MTH78	No	3		49405	49647	-		Methanothermobacter_thermautotrophicus_Delta_	NC_000916	chromosome	clust_400									
15678107	hypothetical protein MTH79	No	2		49661	50164	-		Methanothermobacter_thermautotrophicus_Delta_	NC_000916	chromosome			arCOG10864	11	M		Peptidase C39			
15678108	hypothetical protein MTH80	No	3		50180	50425	-		Methanothermobacter_thermautotrophicus_Delta_	NC_000916	chromosome	clust_7									
15678109	hypothetical protein MTH81	No	2		50538	51182	-		Methanothermobacter_thermautotrophicus_Delta_	NC_000916	chromosome			arCOG10864	11	M		Peptidase C39			
15678110	hypothetical protein MTH82	No	0		51302	51484	-		Methanothermobacter_thermautotrophicus_Delta_	NC_000916	chromosome			arCOG03032	12 (-)	R	NrfG	TPR repeats containing protein			
===								17	Methanothermobacter_thermautotrophicus_Delta_	NC_000916	chromosome										
15678112	hypothetical protein MTH84	No	0		52562	53164	-		Methanothermobacter_thermautotrophicus_Delta_	NC_000916	chromosome										
15678113	hypothetical protein MTH85	No	1		53284	53409	+		Methanothermobacter_thermautotrophicus_Delta_	NC_000916	chromosome										
15678114	hypothetical protein MTH86	No	3		53629	54045	-		Methanothermobacter_thermautotrophicus_Delta_	NC_000916	chromosome	clust_252									
15678115	surface protease related protein	Yes	1		54150	56672	-		Methanothermobacter_thermautotrophicus_Delta_	NC_000916	chromosome			arCOG03946	3 (-)	S		Uncharacterized conserved protein	2E-10	COG3291	FOG: PKD repeat
15678116	hypothetical protein MTH88	No	0		56672	56779	-		Methanothermobacter_thermautotrophicus_Delta_	NC_000916	chromosome										
15678117	hypothetical protein MTH89	No	0		56940	57119	-		Methanothermobacter_thermautotrophicus_Delta_	NC_000916	chromosome										
15678118	hypothetical protein MTH90	No	4		57204	57596	-		Methanothermobacter_thermautotrophicus_Delta_	NC_000916	chromosome										
15678119	hypothetical protein MTH91	No	1		57581	57808	-		Methanothermobacter_thermautotrophicus_Delta_	NC_000916	chromosome										
15678120	hypothetical protein MTH92	No	2		57999	58211	-		Methanothermobacter_thermautotrophicus_Delta_	NC_000916	chromosome										
15678121	hypothetical protein MTH93	No	4		58390	58758	-		Methanothermobacter_thermautotrophicus_Delta_	NC_000916	chromosome										
15678122	hypothetical protein MTH94	No	1		58795	59067	-		Methanothermobacter_thermautotrophicus_Delta_	NC_000916	chromosome										
15678123	hypothetical protein MTH95	Yes	4		59128	59583	-		Methanothermobacter_thermautotrophicus_Delta_	NC_000916	chromosome	clust_232									
15678124	hypothetical protein MTH96	No	0		59620	60189	-		Methanothermobacter_thermautotrophicus_Delta_	NC_000916	chromosome			arCOG10864	11	M		Peptidase C39	4E-18	COG3271	Predicted double-glycine peptidase
15678125	hypothetical protein MTH97	Yes	1		60246	60524	-		Methanothermobacter_thermautotrophicus_Delta_	NC_000916	chromosome										
15678126	hypothetical protein MTH98	No	1		60818	60943	-		Methanothermobacter_thermautotrophicus_Delta_	NC_000916	chromosome										
15678127	hypothetical protein MTH99	No	0		61183	61362	-		Methanothermobacter_thermautotrophicus_Delta_	NC_000916	chromosome										
15678128	hypothetical protein MTH100	No	0		61443	61754	-		Methanothermobacter_thermautotrophicus_Delta_	NC_000916	chromosome			arCOG03597	4 (-)	S		Uncharacterized conserved protein			
===								6	Methanothermobacter_thermautotrophicus_Delta_	NC_000916	chromosome										
15678135	hypothetical protein MTH107	No	0	YES	68751	69311	+		Methanothermobacter_thermautotrophicus_Delta_	NC_000916	chromosome			arCOG01446	5 (-)	VK	Csa3	CRISPR-Cas assicated transcriptional regulator, contains CARF and HTH domain			
15678136	hypothetical protein MTH108	No	0	YES	69355	69588	-		Methanothermobacter_thermautotrophicus_Delta_	NC_000916	chromosome			arCOG10448	1	S		Uncharacterized conserved protein			
15678137	hypothetical protein MTH109	No	0	YES	69681	70118	+		Methanothermobacter_thermautotrophicus_Delta_	NC_000916	chromosome										
15678138	hypothetical protein MTH110	No	1	YES	70128	70376	+		Methanothermobacter_thermautotrophicus_Delta_	NC_000916	chromosome										
15678139	hypothetical protein MTH111	Yes	0	YES	70599	71165	+		Methanothermobacter_thermautotrophicus_Delta_	NC_000916	chromosome			arCOG10205	1	S		Uncharacterized conserved protein			
15678140	hypothetical protein MTH112	No	5	YES	71214	71708	-		Methanothermobacter_thermautotrophicus_Delta_	NC_000916	chromosome			arCOG01096	1 (-)	S		Ferritin-like domain containing protein	0.0001	COG1814	Uncharacterized membrane protein
===								8	Methanothermobacter_thermautotrophicus_Delta_	NC_000916	chromosome										
15678313	hypothetical protein MTH285	No	0		220771	221826	+		Methanothermobacter_thermautotrophicus_Delta_	NC_000916	chromosome										
15678314	hypothetical protein MTH286	No	0		221950	222390	+		Methanothermobacter_thermautotrophicus_Delta_	NC_000916	chromosome										
15678315	anaerobic ribonucleoside-triphosphate reducta	No	0		222406	223119	+		Methanothermobacter_thermautotrophicus_Delta_	NC_000916	chromosome			arCOG02173	4 (-)	O	NrdG	Organic radical activating enzyme	8E-20	pfam13353	4Fe-4S single cluster domain. This famil
15678316	hypothetical protein MTH288	No	0		223109	224122	+		Methanothermobacter_thermautotrophicus_Delta_	NC_000916	chromosome										
15678317	hypothetical protein MTH289	No	0		224123	225622	+		Methanothermobacter_thermautotrophicus_Delta_	NC_000916	chromosome										
15678318	hypothetical protein MTH290	No	1		225689	226198	-		Methanothermobacter_thermautotrophicus_Delta_	NC_000916	chromosome	clust_199									
15678319	hypothetical protein MTH291	No	1		226236	226682	-		Methanothermobacter_thermautotrophicus_Delta_	NC_000916	chromosome										
15678320	sensory transduction histidine kinase	No	8		227212	228906	+		Methanothermobacter_thermautotrophicus_Delta_	NC_000916	chromosome			arCOG02335	2 (-)	T		Signal transduction histidine kinase, contains PA(	3E-25	COG3920	Signal transduction histidine kinase
===								7	Methanothermobacter_thermautotrophicus_Delta_	NC_000916	chromosome										
15678452	hypothetical protein MTH424	No	1		362016	362399	-		Methanothermobacter_thermautotrophicus_Delta_	NC_000916	chromosome			arCOG10248	3	S		Uncharacterized conserved protein			
15678453	hypothetical protein MTH425	No	1		362490	362849	+		Methanothermobacter_thermautotrophicus_Delta_	NC_000916	chromosome			arCOG05788	1 (-)	S		Uncharacterized conserved protein			
15678454	hypothetical protein MTH426	No	0		362886	363374	+		Methanothermobacter_thermautotrophicus_Delta_	NC_000916	chromosome										
15678455	hypothetical protein MTH427	No	1		363375	365378	+		Methanothermobacter_thermautotrophicus_Delta_	NC_000916	chromosome			arCOG10249	3	S		Uncharacterized conserved protein			
15678456	hypothetical protein MTH428	No	2		365451	366515	+		Methanothermobacter_thermautotrophicus_Delta_	NC_000916	chromosome			arCOG10250	2	S		Uncharacterized conserved protein			
15678457	hypothetical protein MTH429	No	1		366546	366938	+		Methanothermobacter_thermautotrophicus_Delta_	NC_000916	chromosome			arCOG03821	1 (-)	S		Uncharacterized conserved protein			
15678458	hypothetical protein MTH430	No	1		367033	367437	+		Methanothermobacter_thermautotrophicus_Delta_	NC_000916	chromosome			arCOG10451	1	S		Uncharacterized conserved protein			
===								5	Methanothermobacter_thermautotrophicus_Delta_	NC_000916	chromosome										
15678549	hypothetical protein MTH521	No	1	YES	467632	468126	+		Methanothermobacter_thermautotrophicus_Delta_	NC_000916	chromosome			arCOG07907	2	S		Zn-ribbons containing protein			
15678550	hypothetical protein MTH522	No	0	YES	468147	468329	+		Methanothermobacter_thermautotrophicus_Delta_	NC_000916	chromosome										
15678551	hypothetical protein MTH523	No	1	YES	468322	468879	+		Methanothermobacter_thermautotrophicus_Delta_	NC_000916	chromosome			arCOG07908	3	S		Zn ribbon containing protein			
15678552	hypothetical protein MTH524	Yes	1	YES	469019	469720	+		Methanothermobacter_thermautotrophicus_Delta_	NC_000916	chromosome			arCOG06479	1 (-)	S		Uncharacterized conserved protein			
15678553	hypothetical protein MTH525	No	0	YES	469822	470004	+		Methanothermobacter_thermautotrophicus_Delta_	NC_000916	chromosome										
===								6	Methanothermobacter_thermautotrophicus_Delta_	NC_000916	chromosome										
15679329	methyltransferase related protein	No	0		1196831	1197421	+		Methanothermobacter_thermautotrophicus_Delta_	NC_000916	chromosome			arCOG00109	2 (-)	J	HemK	Methylase of polypeptide chain release factors	2E-25	COG2890	Methylase of polypeptide chain release f
15679330	hypothetical protein MTH1330	Yes	1		1197608	1198567	+		Methanothermobacter_thermautotrophicus_Delta_	NC_000916	chromosome			arCOG10864	11	M		Peptidase C39	4E-21	pfam03412	Peptidase C39 family. Lantibiotic and no
15679331	hypothetical protein MTH1331	No	3		1198614	1198979	+		Methanothermobacter_thermautotrophicus_Delta_	NC_000916	chromosome	clust_290									
15679332	hypothetical protein MTH1332	No	0		1199007	1199576	+		Methanothermobacter_thermautotrophicus_Delta_	NC_000916	chromosome								5E-10	COG3271	Predicted double-glycine peptidase
15679333	hypothetical protein MTH1333	No	1		1200106	1200276	+		Methanothermobacter_thermautotrophicus_Delta_	NC_000916	chromosome										
15679334	diaminopimelate epimerase	No	0		1200942	1201811	-		Methanothermobacter_thermautotrophicus_Delta_	NC_000916	chromosome			arCOG02255	1 (-)	E	DapF	Diaminopimelate epimerase	5E-82	COG0253	Diaminopimelate epimerase
===								6	Methanothermobacter_thermautotrophicus_Delta_	NC_000916	chromosome										
15679644	hydrogenase expression/formation protein Hyp	No	0		1515136	1515384	+		Methanothermobacter_thermautotrophicus_Delta_	NC_000916	chromosome			arCOG04427	1 (-)	O	HypC	Hydrogenase maturation factor	1E-19	COG0298	Hydrogenase maturation factor
15679645	hypothetical protein MTH1650	No	0		1515701	1515922	+		Methanothermobacter_thermautotrophicus_Delta_	NC_000916	chromosome										
15679646	hypothetical protein MTH1651	No	0		1516205	1516723	-		Methanothermobacter_thermautotrophicus_Delta_	NC_000916	chromosome			arCOG10864	11	M		Peptidase C39	7E-17	COG3271	Predicted double-glycine peptidase
15679647	hypothetical protein MTH1652	No	3		1516720	1517085	-		Methanothermobacter_thermautotrophicus_Delta_	NC_000916	chromosome	clust_290									
15679648	hypothetical protein MTH1653	Yes	1		1517132	1518097	-		Methanothermobacter_thermautotrophicus_Delta_	NC_000916	chromosome			arCOG10864	11	M		Peptidase C39	6E-21	pfam03412	Peptidase C39 family. Lantibiotic and no
15679649	hypothetical protein MTH1654	No	0		1518389	1518916	-		Methanothermobacter_thermautotrophicus_Delta_	NC_000916	chromosome			arCOG02316	1 (-)	R		Predicted regulator of amino acid metabolism, cc	7E-53	COG2150	Predicted regulator of amino acid metabo
===								10	Methanothermococcus_okinawensis_IH1_uid515	NC_015636	chromosome										
336121287	hypothetical protein Metok_0294	Yes	0		316994	317221	+		Methanothermococcus_okinawensis_IH1_uid515	NC_015636	chromosome										
336121288	hypothetical protein Metok_0295	No	0		317308	317601	+		Methanothermococcus_okinawensis_IH1_uid515	NC_015636	chromosome										
336121289	hypothetical protein Metok_0296	No	0		317877	318920	-		Methanothermococcus_okinawensis_IH1_uid515	NC_015636	chromosome										
336121290	hypothetical protein Metok_0297	No	0		318917	319468	-		Methanothermococcus_okinawensis_IH1_uid515	NC_015636	chromosome			arCOG00724	7 (-)	K		Predicted transcriptional regulator, PadR family			
336121291	hypothetical protein Metok_0298	No	0		319461	319976	-		Methanothermococcus_okinawensis_IH1_uid515	NC_015636	chromosome										
336121292	hypothetical protein Metok_0299	No	0		320335	320709	+		Methanothermococcus_okinawensis_IH1_uid515	NC_015636	chromosome										
336121293	hypothetical protein Metok_0300	No	0		320706	320927	+		Methanothermococcus_okinawensis_IH1_uid515	NC_015636	chromosome										
336121294	hypothetical protein Metok_0301	No	0		320976	321155	+		Methanothermococcus_okinawensis_IH1_uid515	NC_015636	chromosome										
336121295	hypothetical protein Metok_0302	No	0		321152	321331	+		Methanothermococcus_okinawensis_IH1_uid515	NC_015636	chromosome										
336121296	hypothetical protein Metok_0303	No	0		321341	321577	+		Methanothermococcus_okinawensis_IH1_uid515	NC_015636	chromosome			arCOG08240	3 (-)	S		Uncharacterized conserved protein			
===								15	Methanothermococcus_okinawensis_IH1_uid515	NC_015636	chromosome										
336121301	hypothetical protein Metok_0308	No	0		323442	323759	+		Methanothermococcus_okinawensis_IH1_uid515	NC_015636	chromosome			arCOG08301	4	Y		Predicted phage protein			
336121302	hypothetical protein Metok_0309	No	0		323825	324694	+		Methanothermococcus_okinawensis_IH1_uid515	NC_015636	chromosome			arCOG09508	6 (-)	Y		Phage prohead protease			
336121303	hypothetical protein Metok_0310	No	0		324704	325030	+		Methanothermococcus_okinawensis_IH1_uid515	NC_015636	chromosome								0.000000002	pfam02924	Bacteriophage lambda head decoration pro
336121304	hypothetical protein Metok_0311	No	0		325040	325990	+		Methanothermococcus_okinawensis_IH1_uid515	NC_015636	chromosome										
336121305	hypothetical protein Metok_0312	No	0		326001	326402	+		Methanothermococcus_okinawensis_IH1_uid515	NC_015636	chromosome	clust_266									
336121306	hypothetical protein Metok_0313	No	0		326408	327193	+		Methanothermococcus_okinawensis_IH1_uid515	NC_015636	chromosome	clust_126									
336121307	hypothetical protein Metok_0314	No	0		327202	327576	+		Methanothermococcus_okinawensis_IH1_uid515	NC_015636	chromosome	clust_285									
336121308	hypothetical protein Metok_0315	No	0		327560	327784	+		Methanothermococcus_okinawensis_IH1_uid515	NC_015636	chromosome	clust_429									
336121309	hypothetical protein Metok_0316	No	1		327774	327899	+		Methanothermococcus_okinawensis_IH1_uid515	NC_015636	chromosome										
336121310	phage tail tape measure protein, TP901 family	No	5		327904	330087	+		Methanothermococcus_okinawensis_IH1_uid515	NC_015636	chromosome			arCOG11109	8 (-)	Y		Phage tail tape measure protein, TP901 family	8E-31	pfam10145	Phage-related minor tail protein. Member
336121311	hypothetical protein Metok_0318	No	0		330093	330656	+		Methanothermococcus_okinawensis_IH1_uid515	NC_015636	chromosome										
336121312	hypothetical protein Metok_0319	No	0		330692	331498	+		Methanothermococcus_okinawensis_IH1_uid515	NC_015636	chromosome			arCOG09649	3	Y		Predicted phage protein			
336121313	hypothetical protein Metok_0320	No	0		331495	332934	+		Methanothermococcus_okinawensis_IH1_uid515	NC_015636	chromosome			arCOG03508	1 (-)	S		Uncharacterized conserved protein			
336121314	hypothetical protein Metok_0321	No	0		332971	333471	+		Methanothermococcus_okinawensis_IH1_uid515	NC_015636	chromosome			arCOG09650	4	Y		Predicted phage protein			
336121315	hypothetical protein Metok_0322	No	0		333536	335683	+		Methanothermococcus_okinawensis_IH1_uid515	NC_015636	chromosome			arCOG09651	3	Y		Predicted phage protein			
===								16	Methanothermococcus_okinawensis_IH1_uid515	NC_015636	chromosome										
336121357	hypothetical protein Metok_0365	No	2		385030	386133	-		Methanothermococcus_okinawensis_IH1_uid515	NC_015636	chromosome			arCOG00368	3 (-)	L	SbcC	ATPase involved in DNA repair, SbcC	1E-12	COG0419	ATPase involved in DNA repair
336121358	hypothetical protein Metok_0366	Yes	2		386133	386846	-		Methanothermococcus_okinawensis_IH1_uid515	NC_015636	chromosome	clust_140									
336121359	hypothetical protein Metok_0367	No	2		387093	387359	-		Methanothermococcus_okinawensis_IH1_uid515	NC_015636	chromosome	clust_374									
336121360	hypothetical protein Metok_0368	Yes	1		387371	388159	-		Methanothermococcus_okinawensis_IH1_uid515	NC_015636	chromosome								0.000001	pfam10446	Protein of unknown function (DUF2457). T
336121361	hypothetical protein Metok_0369	No	4		388249	388623	-		Methanothermococcus_okinawensis_IH1_uid515	NC_015636	chromosome	clust_273									
336121362	hypothetical protein Metok_0370	No	3		388625	389530	-		Methanothermococcus_okinawensis_IH1_uid515	NC_015636	chromosome										
336121363	hypothetical protein Metok_0371	No	0		389547	389843	-		Methanothermococcus_okinawensis_IH1_uid515	NC_015636	chromosome			arCOG09539	3	S		Uncharacterized conserved protein			
336121364	hypothetical protein Metok_0372	No	0		389848	391134	-		Methanothermococcus_okinawensis_IH1_uid515	NC_015636	chromosome			arCOG07665	6 (-)	S		Uncharacterized conserved protein			

336121365	hypothetical protein Metok_0373	No	0			391124	391537	-		Methanothermococcus_okinawensis_IH1_uid515	NC_015636	chromosome		arCOG09528	2	S		Uncharacterized conserved protein			
336121366	hypothetical protein Metok_0374	No	0			391539	391958	-		Methanothermococcus_okinawensis_IH1_uid515	NC_015636	chromosome		arCOG09529	2	S		Uncharacterized conserved protein			
336121367	hypothetical protein Metok_0375	No	0			391955	392215	-		Methanothermococcus_okinawensis_IH1_uid515	NC_015636	chromosome		arCOG09530	1 (-)	S		Uncharacterized conserved protein			
336121368	hypothetical protein Metok_0376	No	0			392212	392433	-		Methanothermococcus_okinawensis_IH1_uid515	NC_015636	chromosome		arCOG08302	4	S		Uncharacterized conserved protein			
336121369	hypothetical protein Metok_0377	No	0			392445	392681	-		Methanothermococcus_okinawensis_IH1_uid515	NC_015636	chromosome									
336121370	hypothetical protein Metok_0378	No	0			392692	393024	-		Methanothermococcus_okinawensis_IH1_uid515	NC_015636	chromosome									
336121371	hypothetical protein Metok_0379	No	0			393026	393187	-		Methanothermococcus_okinawensis_IH1_uid515	NC_015636	chromosome									
336121372	regulatory protein MarR	No	0			393355	393720	+		Methanothermococcus_okinawensis_IH1_uid515	NC_015636	chromosome		arCOG01057	21 (-)	K		Predicted transcriptional regulator			
===									10	Methanothermococcus_okinawensis_IH1_uid515	NC_015636	chromosome									
336121422	hypothetical protein Metok_0435	No	0			440232	440597	+		Methanothermococcus_okinawensis_IH1_uid515	NC_015636	chromosome		arCOG00761	1 (-)	S		Uncharacterized conserved protein	6E-21	COG1720	Uncharacterized conserved protein
336121423	hypothetical protein Metok_0436	Yes	1			441206	442084	+		Methanothermococcus_okinawensis_IH1_uid515	NC_015636	chromosome		arCOG10331	3	S		Uncharacterized conserved protein			
336121424	hypothetical protein Metok_0437	No	3			442157	442582	+		Methanothermococcus_okinawensis_IH1_uid515	NC_015636	chromosome		arCOG10682	5	S		Uncharacterized conserved protein			
336121425	hypothetical protein Metok_0438	No	3			442690	443052	-		Methanothermococcus_okinawensis_IH1_uid515	NC_015636	chromosome	clust_261								
336121426	hypothetical protein Metok_0439	No	2			443328	443639	+		Methanothermococcus_okinawensis_IH1_uid515	NC_015636	chromosome									
336121427	hypothetical protein Metok_0440	No	5			443665	444219	+		Methanothermococcus_okinawensis_IH1_uid515	NC_015636	chromosome									
336121428	hypothetical protein Metok_0441	No	4			444245	444649	+		Methanothermococcus_okinawensis_IH1_uid515	NC_015636	chromosome	clust_261								
336121429	hypothetical protein Metok_0442	Yes	2			444649	444864	+		Methanothermococcus_okinawensis_IH1_uid515	NC_015636	chromosome									
336121430	hypothetical protein Metok_0444	Yes	1			446702	448207	+		Methanothermococcus_okinawensis_IH1_uid515	NC_015636	chromosome		arCOG10210	2	S		Uncharacterized conserved protein	0.000000005	pfam05316	Mitochondrial ribosomal protein (VAR1).
336121431	coenzyme F420-reducing hydrogenase subuni	No	0			448255	448746	-		Methanothermococcus_okinawensis_IH1_uid515	NC_015636	chromosome		arCOG04429	1 (-)	C	HyaD	Ni,Fe-hydrogenase maturation factor	2E-26	COG0680	Ni,Fe-hydrogenase maturation factor
===									21	Methanothermococcus_okinawensis_IH1_uid515	NC_015636	chromosome									
336121441	hypothetical protein Metok_0456	No	4			458867	459280	-		Methanothermococcus_okinawensis_IH1_uid515	NC_015636	chromosome	clust_247								
336121442	hypothetical protein Metok_0457	Yes	2			459341	459661	-		Methanothermococcus_okinawensis_IH1_uid515	NC_015636	chromosome		arCOG10897	10	S		Uncharacterized conserved protein			
336121443	hypothetical protein Metok_0458	No	4			459946	460485	-		Methanothermococcus_okinawensis_IH1_uid515	NC_015636	chromosome		arCOG01996	17 (-)	S	SpoIIM	Uncharacterized membrane protein	1E-22	COG1300	Uncharacterized membrane protein
336121444	hypothetical protein Metok_0459	No	3			460543	460890	-		Methanothermococcus_okinawensis_IH1_uid515	NC_015636	chromosome									
336121445	hypothetical protein Metok_0460	Yes	3			460943	461242	-		Methanothermococcus_okinawensis_IH1_uid515	NC_015636	chromosome		arCOG10897	10	S		Uncharacterized conserved protein			
336121446	hypothetical protein Metok_0461	No	4			461518	462042	-		Methanothermococcus_okinawensis_IH1_uid515	NC_015636	chromosome		arCOG01996	17 (-)	S	SpoIIM	Uncharacterized membrane protein	1E-18	COG1300	Uncharacterized membrane protein
336121447	hypothetical protein Metok_0462	No	4			462055	462510	-		Methanothermococcus_okinawensis_IH1_uid515	NC_015636	chromosome		arCOG10443	4	S		Uncharacterized conserved protein			
336121448	hypothetical protein Metok_0463	Yes	2			462550	462858	-		Methanothermococcus_okinawensis_IH1_uid515	NC_015636	chromosome		arCOG10897	10	S		Uncharacterized conserved protein			
336121449	hypothetical protein Metok_0464	No	4			463098	463613	-		Methanothermococcus_okinawensis_IH1_uid515	NC_015636	chromosome		arCOG01996	17 (-)	S	SpoIIM	Uncharacterized membrane protein	4E-22	COG1300	Uncharacterized membrane protein
336121450	hypothetical protein Metok_0465	No	1			463663	464250	-		Methanothermococcus_okinawensis_IH1_uid515	NC_015636	chromosome		arCOG11107	3	S		Uncharacterized conserved protein			
336121451	hypothetical protein Metok_0466	Yes	1			464416	464646	-		Methanothermococcus_okinawensis_IH1_uid515	NC_015636	chromosome		arCOG10897	10	S		Uncharacterized conserved protein			
336121452	hypothetical protein Metok_0467	No	4			464930	465484	-		Methanothermococcus_okinawensis_IH1_uid515	NC_015636	chromosome		arCOG01996	17 (-)	S	SpoIIM	Uncharacterized membrane protein	2E-24	COG1300	Uncharacterized membrane protein
336121453	hypothetical protein Metok_0468	No	2			465527	465814	-		Methanothermococcus_okinawensis_IH1_uid515	NC_015636	chromosome									
336121454	hypothetical protein Metok_0469	Yes	2			465901	466173	-		Methanothermococcus_okinawensis_IH1_uid515	NC_015636	chromosome		arCOG10897	10	S		Uncharacterized conserved protein			
336121455	hypothetical protein Metok_0470	No	3			466453	466992	-		Methanothermococcus_okinawensis_IH1_uid515	NC_015636	chromosome		arCOG01996	17 (-)	S	SpoIIM	Uncharacterized membrane protein	5E-23	COG1300	Uncharacterized membrane protein
336121456	hypothetical protein Metok_0471	No	4			466997	467416	-		Methanothermococcus_okinawensis_IH1_uid515	NC_015636	chromosome									
336121457	hypothetical protein Metok_0472	No	2			467536	467712	-		Methanothermococcus_okinawensis_IH1_uid515	NC_015636	chromosome									
336121458	hypothetical protein Metok_0473	No	2			467744	467980	-		Methanothermococcus_okinawensis_IH1_uid515	NC_015636	chromosome									
336121459	hypothetical protein Metok_0474	No	3			468037	468369	-		Methanothermococcus_okinawensis_IH1_uid515	NC_015636	chromosome		arCOG10809	2	S		Uncharacterized conserved protein			
336121460	hypothetical protein Metok_0475	No	2			468733	468912	-		Methanothermococcus_okinawensis_IH1_uid515	NC_015636	chromosome		arCOG10810	3	S		Uncharacterized conserved protein			
336121461	coenzyme F420 hydrogenase	No	0			469174	471054	+		Methanothermococcus_okinawensis_IH1_uid515	NC_015636	chromosome		arCOG02061	1 (-)	C	FrhB	Coenzyme F420-reducing hydrogenase, beta su	7E-74	COG2221	Dissimilatory sulfite reductase (desulfo ; Coenzyme F420-reducing hydrogenase, beta
===									8	Methanothermococcus_okinawensis_IH1_uid515	NC_015636	chromosome									
336122511	2-hydroxyglutaryl-CoA dehydratase D-compon	No	0			1581565	1582686	+		Methanothermococcus_okinawensis_IH1_uid515	NC_015636	chromosome		arCOG04464	1 (-)	E	HgdB	Benzoyl-CoA reductase/2-hydroxyglutaryl-CoA d	2E-115	COG1775	Benzoyl-CoA reductase/2-hydroxyglutaryl-
336122512	hypothetical protein Metok_1546	No	0			1582803	1583378	-		Methanothermococcus_okinawensis_IH1_uid515	NC_015636	chromosome									
336122513	hypothetical protein Metok_1547	No	0			1583480	1583911	-		Methanothermococcus_okinawensis_IH1_uid515	NC_015636	chromosome									
336122514	hypothetical protein Metok_1548	No	1			1583999	1585000	-		Methanothermococcus_okinawensis_IH1_uid515	NC_015636	chromosome									
336122515	KAP P-loop domain-containing protein	No	0			1584997	1585983	-		Methanothermococcus_okinawensis_IH1_uid515	NC_015636	chromosome							4E-19	pfam07693	KAP family P-loop domain. The KAP (after
336122516	hypothetical protein Metok_1550	No	3			1585970	1586317	-		Methanothermococcus_okinawensis_IH1_uid515	NC_015636	chromosome									
336122517	hypothetical protein Metok_1551	No	4			1586311	1586754	-		Methanothermococcus_okinawensis_IH1_uid515	NC_015636	chromosome									
336122518	8-amino-7-oxononanoate synthase	No	0			1586793	1587911	-		Methanothermococcus_okinawensis_IH1_uid515	NC_015636	chromosome		arCOG00113	1 (-)	H	BioF	7-keto-8-aminopelargonate synthetase or related	2E-97	COG0156	7-keto-8-aminopelargonate synthetase and
===									5	Methanothermus_fervidus_DSM_2088_uid60167	NC_014658	chromosome									
312137162	hypothetical protein Mfer_0939	No	1			892902	893177	+		Methanothermus_fervidus_DSM_2088_uid60167	NC_014658	chromosome									
312137163	peptidase c39 bacteriocin processing	Yes	1			893280	894047	+		Methanothermus_fervidus_DSM_2088_uid60167	NC_014658	chromosome		arCOG06761	2 (-)	R		Predicted double-glycine peptidase	2E-22	pfam03412	Peptidase C39 family. Lantibiotic and no
312137164	hypothetical protein Mfer_0941	No	7			894124	894891	+		Methanothermus_fervidus_DSM_2088_uid60167	NC_014658	chromosome		arCOG11253	1	S		Uncharacterized conserved protein			
312137165	hypothetical protein Mfer_0942	Yes	1			895168	895479	+		Methanothermus_fervidus_DSM_2088_uid60167	NC_014658	chromosome									
312137166	hypothetical protein Mfer_0943	No	0			895590	896492	-		Methanothermus_fervidus_DSM_2088_uid60167	NC_014658	chromosome		arCOG10846	2	L		PD-DExK family endonuclease			
===									6	Methanotorris_igneus_Kol_5_uid67321	NC_015562	chromosome									
333910393	hypothetical protein Metig_0505	No	0			509741	510514	+		Methanotorris_igneus_Kol_5_uid67321	NC_015562	chromosome		arCOG05103	4 (-)	S		Uncharacterized conserved protein			
333910394	hypothetical protein Metig_0506	No	0			510498	510827	+		Methanotorris_igneus_Kol_5_uid67321	NC_015562	chromosome		arCOG10661	1	S		Uncharacterized conserved protein			
333910395	hypothetical protein Metig_0507	No	0			510829	511308	+		Methanotorris_igneus_Kol_5_uid67321	NC_015562	chromosome		arCOG10290	1	S		Uncharacterized conserved protein	0.00003	COG1196	Chromosome segregation ATPases
333910396	hypothetical protein Metig_0508	No	0			511339	511722	-		Methanotorris_igneus_Kol_5_uid67321	NC_015562	chromosome									
333910397	hypothetical protein Metig_0509	No	0			511885	512370	-		Methanotorris_igneus_Kol_5_uid67321	NC_015562	chromosome									
333910398	integrase family protein	No	0			512671	513222	+		Methanotorris_igneus_Kol_5_uid67321	NC_015562	chromosome		arCOG06578	1 (-)	L	XerC	Integrase			
===									7	Methanotorris_igneus_Kol_5_uid67321	NC_015562	chromosome									
333910400	hypothetical protein Metig_0512	Yes	1			513750	514523	-		Methanotorris_igneus_Kol_5_uid67321	NC_015562	chromosome		arCOG03038	9 (-)	R	NrfG	TPR repeats containing protein	1E-15	COG0457	FOG: TPR repeat
333910401	hypothetical protein Metig_0513	No	0			514838	515050	-		Methanotorris_igneus_Kol_5_uid67321	NC_015562	chromosome							0.00003	COG1340	Uncharacterized archaeal coiled-coil pro
333910402	hypothetical protein Metig_0514	No	0			515088	515276	-		Methanotorris_igneus_Kol_5_uid67321	NC_015562	chromosome		arCOG09666	3	S		Uncharacterized conserved protein			
333910403	hypothetical protein Metig_0515	No	0			515294	515914	-		Methanotorris_igneus_Kol_5_uid67321	NC_015562	chromosome									
333910404	hypothetical protein Metig_0516	No	0			515936	516121	-		Methanotorris_igneus_Kol_5_uid67321	NC_015562	chromosome									
333910405	hypothetical protein Metig_0517	No	0			516512	517729	+		Methanotorris_igneus_Kol_5_uid67321	NC_015562	chromosome		arCOG06586	2 (-)	S		Uncharacterized conserved protein			
333910406	hypothetical protein Metig_0518	No	0			517710	519038	+		Methanotorris_igneus_Kol_5_uid67321	NC_015562	chromosome		arCOG06585	2	L		PD-(D/E)XK superfamily nuclease			
===									6	Methanotorris_igneus_Kol_5_uid67321	NC_015562	chromosome									
333910990	hypothetical protein Metig_1119	No	0			1119277	1120272	+		Methanotorris_igneus_Kol_5_uid67321	NC_015562	chromosome		arCOG09679	1	S		Uncharacterized conserved protein			
333910991	hypothetical protein Metig_1120	No	0			1120319	1120570	+		Methanotorris_igneus_Kol_5_uid67321	NC_015562	chromosome									
333910992	hypothetical protein Metig_1121	No	3			1120647	1120988	+		Methanotorris_igneus_Kol_5_uid67321	NC_015562	chromosome		arCOG10809	2	S		Uncharacterized conserved protein			
333910993	hypothetical protein Metig_1122	No	2			1121024	1121284	+		Methanotorris_igneus_Kol_5_uid67321	NC_015562	chromosome		arCOG01996	17 (-)	S	SpoIIM	Uncharacterized membrane protein			
333910994	hypothetical protein Metig_1123	No	6			1121700	1122281	-		Methanotorris_igneus_Kol_5_uid67321	NC_015562	chromosome									
333910995	hypothetical protein Metig_1124	Yes	1			1122337	1123149	-		Methanotorris_igneus_Kol_5_uid67321	NC_015562	chromosome		arCOG09673	5	S		Uncharacterized conserved protein			
===									7	Nanoarchaeum_equitans_Kin4_M_uid58009	NC_005213	chromosome									
41614827	putative RNA-processing protein	No	0			28722	29234	-		Nanoarchaeum_equitans_Kin4_M_uid58009	NC_005213	chromosome		arCOG04150	1 (-)	R		Predicted RNA-binding protein (contains KH dom	1E-22	COG1094	Predicted RNA-binding protein (contains
41614828	hypothetical protein NEQ032	No	0			29432	30583	+		Nanoarchaeum_equitans_Kin4_M_uid58009	NC_005213	chromosome									
41614829	hypothetical protein NEQ033	Yes	1			30576	32363	+		Nanoarchaeum_equitans_Kin4_M_uid58009	NC_005213	chromosome									
41614830	hypothetical protein NEQ034	No	1			32363	32998	+		Nanoarchaeum_equitans_Kin4_M_uid58009	NC_005213	chromosome									
41614831	hypothetical protein NEQ035	Yes	1			33020	34420	+		Nanoarchaeum_equitans_Kin4_M_uid58009	NC_005213	chromosome									
41614832	hypothetical protein NEQ036	No	0			34449	34733	+		Nanoarchaeum_equitans_Kin4_M_uid58009	NC_005213	chromosome									
41614833	50S ribosomal protein L37Ae	No	0			34755	35015	+		Nanoarchaeum_equitans_Kin4_M_uid58009	NC_005213	chromosome		arCOG04208	2 (-)	J	RPL43A	Ribosomal protein L37AE/L43A	2E-23	pfam01780	Ribosomal L37ae protein family. This rib
===									10	Nanoarchaeum_equitans_Kin4_M_uid58009	NC_005213	chromosome									
41614929	cell division protein FtsZ	No	0			120868	121935	+		Nanoarchaeum_equitans_Kin4_M_uid58009	NC_005213	chromosome		arCOG02201	4 (-)	D	FtsZ	Cell division GTPase	5E-104	COG0206	Cell division GTPase
41614930	hypothetical protein NEQ132	No	1			121928	122527	-		Nanoarchaeum_equitans_Kin4_M_uid58009	NC_005213	chromosome									
41614931	hypothetical protein NEQ134	No	1			122482	123342	-		Nanoarchaeum_equitans_Kin4_M_uid58009	NC_005213	chromosome									
41614932	hypothetical protein NEQ135	No	1			123318	124844	-		Nanoarchaeum_equitans_Kin4_M_uid58009	NC_005213	chromosome									
41614933	hypothetical protein NEQ136	No	1			124805	125575	-		Nanoarchaeum_equitans_Kin4_M_uid58009	NC_005213	chromosome									
41614934	hypothetical protein NEQ137	No	1			125554	127263	-		Nanoarchaeum_equitans_Kin4_M_uid58009	NC_005213	chromosome									
41614935	hypothetical protein NEQ139	No	2			127297	128406	+		Nanoarchaeum_equitans_Kin4_M_uid58009	NC_005213	chromosome									
41614936	hypothetical protein NEQ138	No	4			128285	128998	-		Nanoarchaeum_equitans_Kin4_M_uid58009	NC_005213	chromosome									
41614937	hypothetical protein NEQ140	No	1			128980	129858	-		Nanoarchaeum_equitans_Kin4_M_uid58009	NC_005213	chromosome									
41614938	hypothetical protein NEQ141	No	0			129888	131510	-		Nanoarchaeum_equitans_Kin4_M_uid58009	NC_005213	chromosome		arCOG01257	3 (-)	O	GroL	Chaperonin GroEL (HSP60 family)	2E-162	pfam00118	TCP-1/cpn60 chaperonin family. This fami
===									5	Nanoarchaeum_equitans_Kin4_M_uid58009	NC_005213	chromosome									
41614956	hypothetical protein NEQ160	No	7			147299	148270	+		Nanoarchaeum_equitans_Kin4_M_uid58009	NC_005213	chromosome							0.00000009	pfam01944	Integral membrane protein DUF95. Members
41614957	hypothetical protein NEQ162	No	7			148267	149250	+		Nanoarchaeum_equitans_Kin4_M_uid58009	NC_005213	chromosome		arCOG02642	3 (-)	R	yhhT	Predicted permease, member of the PurR regulo	1E-36	pfam01594	Domain of unknown function DUF20. This t
41614958	hypothetical protein NEQ161	No	0			149202	149369	-		Nanoarchaeum_equitans_Kin4_M_uid58009	NC_005213	chromosome							0.0000005	pfam11081	Protein of unknown function (DUF2890). T
41614959	hypothetical protein NEQ163	Yes	0			149411	149716	-		Nanoarchaeum_equitans_Kin4_M_uid58009	NC_005213	chromosome									
41614960	hypothetical protein NEQ164	No	10			149706	150581	-		Nanoarchaeum_equitans_Kin4_M_uid58009	NC_005213	chromosome							0.0000002	COG0530	Ca2+/Na+ antiporter
===									5	Nanoarchaeum_equitans_Kin4_M_uid58009	NC_005213	chromosome									
41615015	hypothetical protein NEQ221	No	0			195532	195828	+		Nanoarchaeum_equitans_Kin4_M_uid58009	NC_005213	chromosome									
41615016	hypothetical protein NEQ222	Yes	8			195858	197864	+		Nanoarchaeum_equitans_Kin4_M_uid58009	NC_005213	chromosome									
41615017	hypothetical protein NEQ223	No	0			197875	198699	-		Nanoarchaeum_equitans_Kin4_M_uid58009	NC_005213	chromosome		arCOG01700	2 (-)	E	SpeB	Arginase family enzyme	3E-15	pfam00491	Arginase family.
41615018	hypothetical protein NEQ224	No	0			198788	199201	+		Nanoarchaeum_equitans_Kin4_M_uid58009	NC_005213	chromosome									
41615019	hypothetical protein NEQ225	No	0			199209	199997	+		Nanoarchaeum_equitans_Kin4_M_uid58009	NC_005213	chromosome									
===									5	Nanoarchaeum_equitans_Kin4_M_uid58009	NC_005213	chromosome									
41615043	hypothetical protein NEQ249	No	4			220568	221104	-		Nanoarchaeum_equitans_Kin4_M_uid58009	NC_005213	chromosome									
41615044	hypothetical protein NEQ250	No	5			221225	221737	-		Nanoarchaeum_equitans_Kin4_M_uid58009	NC_005213	chromosome									
41615045	valyl-tRNA synthetase	No	0			221873	224128	+		Nanoarchaeum_equitans_Kin4_M_uid58009	NC_005213	chromosome		arCOG00808	2 (-)	J	ValS	Valyl-tRNA synthetase	7E-139	COG0525	Valyl-tRNA synthetase
41615046	hypothetical protein NEQ254	No	1			224014	225156	+		Nanoarchaeum_equitans_Kin4_M_uid58009	NC_005213	chromosome									
41615047	hypothetical protein NEQ253	No	4			225113	225577	-		Nanoarchaeum_equitans_Kin4_M_uid58009	NC_005213	chromosome									
===									6	Nanoarchaeum_equitans_Kin4_M_uid58009	NC_005213	chromosome									
41615077	hypothetical protein NEQ288	No	0			260106	260354	+		Nanoarchaeum_equitans_Kin4_M_uid58009	NC_005213	chromosome		arCOG02144	3 (-)	L	HHT1	Histones H3 and H4	1E-12	COG2036	Histones H3 and H4
41615078	hypothetical protein NEQ290	No	2			260680	260988	+		Nanoarchaeum_equitans_Kin4_M_uid58009	NC_005213	chromosome									
41615079	hypothetical protein NEQ289	No	1			260968	261327	-		Nanoarchaeum_equitans_Kin4_M_uid58009	NC_005213	chromosome									
41615080	hypothetical protein NEQ291	No	1			261339	265511	-		Nanoarchaeum_equitans_Kin4_M_uid58009	NC_005213	chromosome									
41615081	hypothetical protein NEQ292	No	0			265508	265672	-		Nanoarchaeum_equitans_Kin4_M_uid58009	NC_005213	chromosome									
41615082	hypothetical protein NEQ293	No	0			265757	266881	-		Nanoarchaeum_equitans_Kin4_M_uid58009	NC_005213	chromosome		arCOG04252	4 (-)	J		tRNA pseudouridine synthase D	2E-39	COG0585	Uncharacterized conserved protein
===									7	Nanoarchaeum_equitans_Kin4_M_uid58009	NC_005213	chromosome									
41615226	hypothetical protein NEQ442	Yes	0			389084	391411	+		Nanoarchaeum_equitans_Kin4_M_uid58009	NC_005213	chromosome									
41615227	hypothetical protein NEQ441	No	0			391408	391950	-		Nanoarchaeum_equitans_Kin4_M_uid58009	NC_005213	chromosome		arCOG01336	1 (-)	S	AMMECR	Uncharacterized conserved protein	1E-54	pfam01871	AMMECR1. This family consists of several
41615228	hypothetical protein NEQ443	No	0			391916	392485	-		Nanoarchaeum_equitans_Kin4_M_uid58009	NC_005213	chromosome									
41615229	hypothetical protein NEQ444	Yes	1			392551	393300	+		Nanoarchaeum_equitans_Kin4_M_uid58009	NC_005213	chromosome									
41615230	hypothetical protein NEQ445	No	1			393310	393882	+		Nanoarchaeum_equitans_Kin4_M_uid58009	NC_005213	chromosome									
41615231	30S ribosomal protein S9P	No	0			397016	397426	+		Nanoarchaeum_equitans_Kin4_M_uid58009	NC_005213	chromosome		arCOG04243	1 (-)	J	RpsI	Ribosomal protein S9	5E-15	pfam00380	Ribosomal protein S9/S16. This family in
41615232	hypothetical protein NEQ448	No	1			397402	397779	+		Nanoarchaeum_equitans_Kin4_M_uid58009	NC_005213	chromosome									
===									5	Natrialba_magadii_ATCC_43099_uid46245	NC_013922	chromosome									
289579742	hypothetical protein Nmag_0047	No	0			57609	57821	-		Natrialba_magadii_ATCC_43099_uid46245	NC_013922	chromosome		arCOG09093	1	S		Uncharacterized conserved protein			
289579743	hypothetical protein Nmag_0048	No	0			58273	58443	-		Natrialba_magadii_ATCC_43099_uid46245	NC_013922	chromosome									
289579744	hypothetical protein Nmag_0049	No	2			58547	59698	+		Natrialba_magadii_ATCC_43099_uid46245	NC_013922	chromosome		arCOG09252	1 (-)	S		Uncharacterized conserved protein			
289579745	hypothetical protein Nmag_0050	No	4			59749	61839	-		Natrialba_magadii_ATCC_43099_uid46245	NC_013922	chromosome									
289579746	hypothetical protein Nmag_0051	No	1			62470	63342	-		Natrialba_magadii_ATCC_43099_uid46245	NC_013922	chromosome									
===									22	Natrialba_magadii_ATCC_43099_uid46245	NC_013922	chromosome									
289579926	hypothetical protein Nmag_0238	No	0	YES		242966	243493	+		Natrialba_magadii_ATCC_43099_uid46245	NC_013922	chromosome									
289579927	HNH endonuclease	No	0	YES		243610	244041	-		Natrialba_magadii_ATCC_43099_uid46245	NC_013922	chromosome		arCOG03898	7 (-)	V	McrA	Restriction endonuclease, McrA/HNH family	0.000000001	pfam01844	HNH endonuclease.
289579928	hypothetical protein Nmag_0240	No	0	YES		244398	244505	+		Natrialba_magadii_ATCC_43099_uid46245	NC_013922	chromosome									
289579929	hypothetical protein Nmag_0241	No	0	YES		244493	244873	+		Natrialba_magadii_ATCC_43099_uid46245	NC_013922	chromosome									
289579930	hypothetical protein Nmag_0242	No	0	YES		244883	245536	-		Natrialba_magadii_ATCC_43099_uid46245	NC_013922	chromosome									
289579931	hypothetical protein Nmag_0243	No	0	YES		245647	245814	+		Natrialba_magadii_ATCC_43099_uid46245	NC_013922	chromosome									
289579932	hypothetical protein Nmag_0244	No	0	YES		245909	246004	+		Natrialba_magadii_ATCC_43099_uid46245	NC_013922	chromosome									
289579933	hypothetical protein Nmag_0245	No	0			246089	246358	+		Natrialba_magadii_ATCC_43099_uid46245	NC_013922	chromosome									
289579934	hypothetical protein Nmag_0246	No	0	YES		246505	246672	+		Natrialba_magadii_ATCC_43099_uid46245	NC_013922	chromosome									
289579935	hypothetical protein Nmag_0247	Yes	2	YES		246722	246952	+		Natrialba_magadii_ATCC_43099_uid46245	NC_013922	chromosome									
289579936	Na(+)/H(+) antiporter subunit	No	2	YES		246949	247170	+		Natrialba_magadii_ATCC_43099_uid46245	NC_013922	chromosome									
289579937	hypothetical protein Nmag_0249	No	0	YES		247480	247857	+		Natrialba_magadii_ATCC_43099_uid46245	NC_013922	chromosome									
289579938	hypothetical protein Nmag_0250	No	0	YES		248048	248626	+		Natrialba_magadii_ATCC_43099_uid46245	NC_013922	chromosome									
289579939	integrase family protein	No	0	YES		248773	249366	+		Natrialba_magadii_ATCC_43099_uid46245	NC_013922	chromosome		arCOG02282	8 (-)	K		Predicted transcriptional regulator, C-terminal HTH-like domain			
289579940	hypothetical protein Nmag_0252	No	0	YES		249462	250133	+		Natrialba_magadii_ATCC_43099_uid46245	NC_013922	chromosome									
289579941	hypothetical protein Nmag_0253	No	0	YES		250253	250441	+		Natrialba_magadii_ATCC_43099_uid46245	NC_013922	chromosome									
289579942	hypothetical protein Nmag_0254	No	0	YES		250467	250697	+		Natrialba_magadii_ATCC_43099_uid46245	NC_013922	chromosome		arCOG10165	3 (-)	S		Uncharacterized conserved protein			
289579943	hypothetical protein Nmag_0255	No	0	YES		250932	251327	+		Natrialba_magadii_ATCC_43099_uid46245	NC_013922	chromosome									
289579944	hypothetical protein Nmag_0256	No	0	YES		251426	251608	+		Natrialba_magadii_ATCC_43099_uid46245	NC_013922	chromosome									
289579945	hypothetical protein Nmag_0257	No	0	YES		251605	251766	+		Natrialba_magadii_ATCC_43099_uid46245	NC_013922	chromosome									
289579946	hypothetical protein Nmag_0258	No	0	YES		251813	251956	+		Natrialba_magadii_ATCC_43099_uid46245	NC_013922	chromosome									
289579947	hypothetical protein Nmag_0259	No	0	YES		251979	252209	-		Natrialba_magadii_ATCC_43099_uid46245	NC_013922	chromosome		arCOG09407	1 (-)	S		Uncharacterized conserved protein			
===									5	Natrialba_magadii_ATCC_43099_uid46245	NC_013922	chromosome									
289579965	CheA signal transduction histidine kinase	No	1			271020	272087	+		Natrialba_magadii_ATCC_43099_uid46245	NC_013922	chromosome									
289579966	hypothetical protein Nmag_0278	No	0			272321	272611	+		Natrialba_magadii_ATCC_43099_uid46245	NC_013922	chromosome		arCOG08180	3 (-)	S		Uncharacterized conserved protein			
289579967	hypothetical protein Nmag_0279	No	0			272614	272826	+		Natrialba_magadii_ATCC_43099_uid46245	NC_013922	chromosome									
289579968	hypothetical protein Nmag_0280	No	0			272823	272966	+		Natrialba_magadii_ATCC_43099_uid46245	NC_013922	chromosome									
289579969	hypothetical protein Nmag_0281	No	0			272963	273145	+		Natrialba_magadii_ATCC_43099_uid46245	NC_013922	chromosome									
===									11	Natrialba_magadii_ATCC_43099_uid46245	NC_013922	chromosome									
289580040	hypothetical protein Nmag_0354	No	2			340010	341254	-		Natrialba_magadii_ATCC_43099_uid46245	NC_013922	chromosome									
289580041	hypothetical protein Nmag_0355	No	0			341623	342741	+		Natrialba_magadii_ATCC_43099_uid46245	NC_013922	chromosome									
289580042	UBA/THIF-type NAD/FAD binding protein	No	0			342761	345076	+		Natrialba_magadii_ATCC_43099_uid46245	NC_013922	chromosome	arCOG01138,a	arCOG01138	1 (-)	R		Predicted metal-dependent protease of the PAD	2E-13	pfam00899	ThiF family. This family contains a repe
289580043	hypothetical protein Nmag_0357	No	0	YES		345117	346337	+		Natrialba_magadii_ATCC_43099_uid46245	NC_013922	chromosome									
289580044	hypothetical protein Nmag_0358	No	0	YES		346952	347965	-		Natrialba_magadii_ATCC_43099_uid46245	NC_013922	chromosome									
289580045	hypothetical protein Nmag_0359	No	0	YES		348081	348968	+		Natrialba_magadii_ATCC_43099_uid46245	NC_013922	chromosome									
289580046	hypothetical protein Nmag_0360	No	0	YES		348965	349540	+		Natrialba_magadii_ATCC_43099_uid46245	NC_013922	chromosome									
289580047	hypothetical protein Nmag_0361	No	2	YES		349556	349996	+		Natrialba_magadii_ATCC_43099_uid46245	NC_013922	chromosome							5E-13	pfam14015	Protein of unknown function (DUF4231). T
289580048	hypothetical protein Nmag_0362	No	0	YES		350041	350511	+		Natrialba_magadii_ATCC_43099_uid46245	NC_013922	chromosome		arCOG06889	2	S		Uncharacterized conserved protein containing TI	2E-18	pfam08937	MTH538 TIR-like domain (DUF1863). This d
289580049	hypothetical protein Nmag_0363	No	0	YES		351108	351308	+		Natrialba_magadii_ATCC_43099_uid46245	NC_013922	chromosome		arCOG10363	7	V		AbrB-like transcriptional regulator			
289580050	resolvase	No	0	YES		351301	352050	+		Natrialba_magadii_ATCC_43099_uid46245	NC_013922	chromosome		arCOG03162	12 (-)	L	PinR	Site-specific recombinase, DNA invertase Pin homolog			
===									14	Natrialba_magadii_ATCC_43099_uid46245	NC_013922	chromosome									
289580052	hypothetical protein Nmag_0366	No	0	YES		353497	353811	+		Natrialba_magadii_ATCC_43099_uid46245	NC_013922	chromosome									
289580053	retinol dehydrogenase 7	No	0	YES		356913	357089	-		Natrialba_magadii_ATCC_43099_uid46245	NC_013922	chromosome									
289580054	orc1/cdc6 family replication initiation protein	No	0	YES		358048	359319	+		Natrialba_magadii_ATCC_43099_uid46245	NC_013922	chromosome		arCOG00467	13 (-)	L	CDC6	Cdc6-related protein, AAA superfamily ATPase	1E-33	COG1474	Cdc6-related protein, AAA superfamily AT
289580055	hypothetical protein Nmag_0369	No	0	YES		359792	359953	-		Natrialba_magadii_ATCC_43099_uid46245	NC_013922	chromosome									
289580056	hypothetical protein Nmag_0370	No	1	YES		360141	360245	+		Natrialba_magadii_ATCC_43099_uid46245	NC_013922	chromosome									
289580057	hypothetical protein Nmag_0371	No	0	YES		360292	360414	-		Natrialba_magadii_ATCC_43099_uid46245	NC_013922	chromosome									
289580058	resolvase	No	0	YES		360458	360844	-		Natrialba_magadii_ATCC_43099_uid46245	NC_013922	chromosome		arCOG03162	12 (-)	L	PinR	Site-specific recombinase, DNA invertase Pin ho	0.00002	COG1961	Site-specific recombinases, DNA invertas
289580059	hypothetical protein Nmag_0373	No	0	YES		361375	361572	-		Natrialba_magadii_ATCC_43099_uid46245	NC_013922	chromosome									
289580060	hypothetical protein Nmag_0374	No	0	YES		361623	361811	-		Natrialba_magadii_ATCC_43099_uid46245	NC_013922	chromosome		arCOG06395	1 (-)	S		Uncharacterized conserved protein			
289580061	hypothetical protein Nmag_0375	No	0	YES		362137	362256	-		Natrialba_magadii_ATCC_43099_uid46245	NC_013922	chromosome									
289580062	hypothetical protein Nmag_0376	No	0	YES		362703	363197	-		Natrialba_magadii_ATCC_43099_uid46245	NC_013922	chromosome									
289580063	hypothetical protein Nmag_0377	No	0	YES		363286	363399	+		Natrialba_magadii_ATCC_43099_uid46245	NC_013922	chromosome									
289580064	hypothetical protein Nmag_0378	No	0	YES		363417	364022	+		Natrialba_magadii_ATCC_43099_uid46245	NC_013922	chromosome									
289580065	ATPase	No	0	YES		364638	365870	+		Natrialba_magadii_ATCC_43099_uid46245	NC_013922	chromosome		arCOG01307	3 (-)	D	Vps4	Cell division ATPase of the AAA+ class, ESCRT	3E-10	COG0714	MoxR-like ATPases
===									12	Natrialba_magadii_ATCC_43099_uid46245	NC_013922	chromosome									
289580436	ATPase AAA	No	0			752904	755081	+		Natrialba_magadii_ATCC_43099_uid46245	NC_013922	chromosome		arCOG01308	10 (-)	O	Cdc48	ATPase of the AAA+ class , CDC48 family	6E-18	COG0464	ATPases of the AAA+ class
289580437	hypothetical protein Nmag_0756	No	0			755112	755771	+		Natrialba_magadii_ATCC_43099_uid46245	NC_013922	chromosome							2E-10	pfam14065	Protein of unknown function (DUF4255). T
289580438	hypothetical protein Nmag_0757	No	0			755809	756261	-		Natrialba_magadii_ATCC_43099_uid46245	NC_013922	chromosome	clust_6						3E-35	pfam06841	T4-like virus tail tube protein gp19. Th
289580439	hypothetical protein Nmag_0758	No	0			756301	756558	-		Natrialba_magadii_ATCC_43099_uid46245	NC_013922	chromosome	clust_21								
289580440	hypothetical protein Nmag_0759	No	0			756555	757085	-		Natrialba_magadii_ATCC_43099_uid46245	NC_013922	chromosome	clust_16								
289580441	hypothetical protein Nmag_0760	No	0			757082	757234	-		Natrialba_magadii_ATCC_43099_uid46245	NC_013922	chromosome									
289580442	hypothetical protein Nmag_0761	No	0			757231	757683	-		Natrialba_magadii_ATCC_43099_uid46245	NC_013922	chromosome	clust_19						1E-21	pfam06841	T4-like virus tail tube protein gp19. Th
289580443	tail sheath protein	No	0			757732	759489	-		Natrialba_magadii_ATCC_43099_uid46245	NC_013922	chromosome	clust_11						3E-26	COG3497	Phage tail sheath protein FI
289580444	hypothetical protein Nmag_0763	No	0			759767	761011	-		Natrialba_magadii_ATCC_43099_uid46245	NC_013922	chromosome	clust_28								
289580445	phage tail protein	No	0			761261	763444	-		Natrialba_magadii_ATCC_43099_uid46245	NC_013922	chromosome									
289580446	hypothetical protein Nmag_0765	No	0			763441	765390	-		Natrialba_magadii_ATCC_43099_uid46245	NC_013922	chromosome									
289580447	hypothetical protein Nmag_0766	No	0			765395	768700	-		Natrialba_magadii_ATCC_43099_uid46245	NC_013922	chromosome		arCOG10307	1 (-)	S		Uncharacterized conserved protein			
===									7	Natrialba_magadii_ATCC_43099_uid46245	NC_013922	chromosome									
289580477	transposase IS4 family protein	No	0			806120	806941	-		Natrialba_magadii_ATCC_43099_uid46245	NC_013922	chromosome		arCOG02751	5 (-)	L		Transposase, IS5 family			
289580478	hypothetical protein Nmag_0798	No	0			807227	807916	+		Natrialba_magadii_ATCC_43099_uid46245	NC_013922	chromosome		arCOG10872	5	S		Uncharacterized conserved protein			
289580479	hypothetical protein Nmag_0799	No	0			807921	808673	+		Natrialba_magadii_ATCC_43099_uid46245	NC_013922	chromosome		arCOG10872	5	S		Uncharacterized conserved protein			
289580480	hypothetical protein Nmag_0800	No	0			808696	809337	+		Natrialba_magadii_ATCC_43099_uid46245	NC_013922	chromosome		arCOG10872	5	S		Uncharacterized conserved protein			
289580481	hypothetical protein Nmag_0801	No	0			809342	810106	+		Natrialba_magadii_ATCC_43099_uid46245	NC_013922	chromosome		arCOG10872	5	S		Uncharacterized conserved protein			
289580482	hypothetical protein Nmag_0802	No	0			810096	811508	+		Natrialba_magadii_ATCC_43099_uid46245	NC_013922	chromosome	arCOG10872,a	arCOG10872	5	S		Uncharacterized conserved protein			
289580483	eRF1 domain 2 protein	No	0			811845	812933	+		Natrialba_magadii_ATCC_43099_uid46245	NC_013922	chromosome		arCOG01742	1 (-)	J	eRF1	Peptide chain release factor 1 (eRF1)	1E-56	COG1503	Peptide chain release factor 1 (eRF1)
===									15	Natrialba_magadii_ATCC_43099_uid46245	NC_013922	chromosome									
289581277	integrase family protein	No	0			1630329	1631690	+		Natrialba_magadii_ATCC_43099_uid46245	NC_013922	chromosome		arCOG01243	4 (-)	L	XerC	Site-specific recombinase XerC			
289581278	kinesin protein family member	No	0			1631654	1631824	-		Natrialba_magadii_ATCC_43099_uid46245	NC_013922	chromosome									
289581279	hypothetical protein Nmag_1607	No	0			1631821	1632144	-		Natrialba_magadii_ATCC_43099_uid46245	NC_013922	chromosome									
289581280	hypothetical protein Nmag_1608	No	1			1632141	1632443	-		Natrialba_magadii_ATCC_43099_uid46245	NC_013922	chromosome									
289581281	hypothetical protein Nmag_1609	No	0			1632436	1632834	-		Natrialba_magadii_ATCC_43099_uid46245	NC_013922	chromosome									
289581282	hypothetical protein Nmag_1610	No	0			1632831	1633133	-		Natrialba_magadii_ATCC_43099_uid46245	NC_013922	chromosome									
289581283	hypothetical protein Nmag_1611	No	0			1633133	1634101	-		Natrialba_magadii_ATCC_43099_uid46245	NC_013922	chromosome									
289581284	hypothetical protein Nmag_1612	No	0	YES		1634098	1634472	-		Natrialba_magadii_ATCC_43099_uid46245	NC_013922	chromosome									
289581285	hypothetical protein Nmag_1613	No	0	YES		1634444	1634677	-		Natrialba_magadii_ATCC_43099_uid46245	NC_013922	chromosome									
289581286	hypothetical protein Nmag_1614	No	0	YES		1634797	1635006	-		Natrialba_magadii_ATCC_43099_uid46245	NC_013922	chromosome									
289581287	hypothetical protein Nmag_1615	No	0	YES		1635087	1635278	+		Natrialba_magadii_ATCC_43099_uid46245	NC_013922	chromosome									
289581288	hypothetical protein Nmag_1616	No	0	YES		1635665	1636420	-		Natrialba_magadii_ATCC_43099_uid46245	NC_013922	chromosome									
289581289	hypothetical protein Nmag_1617	No	0	YES		1636410	1637624	-		Natrialba_magadii_ATCC_43099_uid46245	NC_013922	chromosome									
289581290	hypothetical protein Nmag_1618	No	0	YES		1637703	1639088	-		Natrialba_magadii_ATCC_43099_uid46245	NC_013922	chromosome									
289581291	hypothetical protein Nmag_1619	No	0	YES		1639088	1641253	-		Natrialba_magadii_ATCC_43099_uid46245	NC_013922	chromosome		arCOG00280	21 (-)	L		HerA helicase			
===									7	Natrialba_magadii_ATCC_43099_uid46245	NC_013922	chromosome									
289581296	hypothetical protein Nmag_1624	No	4	YES	Virus_Caudovi	1645612	1646016	-		Natrialba_magadii_ATCC_43099_uid46245	NC_013922	chromosome									
289581297	hypothetical protein Nmag_1625	Yes	1	YES	Virus_Caudovi	1646181	1647047	-		Natrialba_magadii_ATCC_43099_uid46245	NC_013922	chromosome		arCOG02696	1 (-)	S		Uncharacterized conserved protein, DUF1102 family			
289581298	hypothetical protein Nmag_1626	No	0	YES	Virus_Caudovi	1647202	1647303	+		Natrialba_magadii_ATCC_43099_uid46245	NC_013922	chromosome									
289581299	hypothetical protein Nmag_1627	No	0	YES	Virus_Caudovi	1647306	1647509	-		Natrialba_magadii_ATCC_43099_uid46245	NC_013922	chromosome									
289581300	hypothetical protein Nmag_1628	No	0	YES	Virus_Caudovi	1649453	1649710	-		Natrialba_magadii_ATCC_43099_uid46245	NC_013922	chromosome									
289581301	hypothetical protein Nmag_1629	No	0	YES	Virus_Caudovi	1650248	1650877	+		Natrialba_magadii_ATCC_43099_uid46245	NC_013922	chromosome									
289581302	hypothetical protein Nmag_1630	Yes	1	YES	Virus_Caudovi	1651338	1653842	+		Natrialba_magadii_ATCC_43099_uid46245	NC_013922	chromosome		arCOG06273	2 (-)	S		S-layer protein	0.000008	COG5271	AAA ATPase containing von Willebrand fac ; Uncharacterized conserved protein
===									15	Natrialba_magadii_ATCC_43099_uid46245	NC_013922	chromosome									
289581306	hypothetical protein Nmag_1634	No	0	YES	Virus_Caudovi	1658756	1659904	-		Natrialba_magadii_ATCC_43099_uid46245	NC_013922	chromosome		arCOG10640	5	Y		Phage associated protein			
289581307	hypothetical protein Nmag_1635	No	0	YES	Virus_Caudovi	1659914	1660576	-		Natrialba_magadii_ATCC_43099_uid46245	NC_013922	chromosome		arCOG10639	4	Y		Phage associated protein			
289581308	rhomboid family protein	No	4		Virus_Caudovi	1660647	1661297	+		Natrialba_magadii_ATCC_43099_uid46245	NC_013922	chromosome		arCOG01768	2 (-)	E	GlpG	Membrane associated serine protease	0.0000007	COG0705	Membrane associated serine protease
289581309	hypothetical protein Nmag_1637	No	0	YES	Virus_Caudovi	1661303	1662520	-		Natrialba_magadii_ATCC_43099_uid46245	NC_013922	chromosome									
289581310	hypothetical protein Nmag_1638	No	0	YES	Virus_Caudovi	1662597	1663097	-		Natrialba_magadii_ATCC_43099_uid46245	NC_013922	chromosome	clust_207								
289581311	hypothetical protein Nmag_1639	No	0	YES	Virus_Caudovi	1663097	1663990	-		Natrialba_magadii_ATCC_43099_uid46245	NC_013922	chromosome	clust_106								
289581312	hypothetical protein Nmag_1640	No	0	YES	Virus_Caudovi	1664022	1664570	-		Natrialba_magadii_ATCC_43099_uid46245	NC_013922	chromosome	clust_191								
289581313	hypothetical protein Nmag_1641	No	0	YES	Virus_Caudovi	1664567	1665037	-		Natrialba_magadii_ATCC_43099_uid46245	NC_013922	chromosome	clust_222								
289581314	hypothetical protein Nmag_1642	No	0	YES	Virus_Caudovi	1665034	1665774	-		Natrialba_magadii_ATCC_43099_uid46245	NC_013922	chromosome									
289581315	hypothetical protein Nmag_1643	No	0	YES	Virus_Caudovi	1665778	1666038	-		Natrialba_magadii_ATCC_43099_uid46245	NC_013922	chromosome									
289581316	hypothetical protein Nmag_1644	No	0	YES	Virus_Caudovi	1666041	1666571	-		Natrialba_magadii_ATCC_43099_uid46245	NC_013922	chromosome									
289581317	hypothetical protein Nmag_1645	No	0	YES	Virus_Caudovi	1666585	1667922	-		Natrialba_magadii_ATCC_43099_uid46245	NC_013922	chromosome									
289581318	peptidase U35 phage prohead HK97	No	0	YES	Virus_Caudovi	1667925	1669130	-		Natrialba_magadii_ATCC_43099_uid46245	NC_013922	chromosome									
289581319	hypothetical protein Nmag_1647	No	0	YES	Virus_Caudovi	1669222	1670655	-		Natrialba_magadii_ATCC_43099_uid46245	NC_013922	chromosome									
289581320	hypothetical protein Nmag_1648	No	0	YES	Virus_Caudovi	1670657	1671001	-		Natrialba_magadii_ATCC_43099_uid46245	NC_013922	chromosome		arCOG08094	3 (-)	S		Uncharacterized conserved protein	0.0001	pfam13619	KTSC domain. This short domain is named
===									8	Natrialba_magadii_ATCC_43099_uid46245	NC_013922	chromosome									
289581322	hypothetical protein Nmag_1650	No	0	YES	Virus_Caudovi	1672811	1673386	-		Natrialba_magadii_ATCC_43099_uid46245	NC_013922	chromosome		arCOG09210	6 (-)	S		Uncharacterized conserved protein			
289581323	hypothetical protein Nmag_1651	No	0	YES	Virus_Caudovi	1673711	1673902	-		Natrialba_magadii_ATCC_43099_uid46245	NC_013922	chromosome		arCOG11198	4	S		Uncharacterized conserved protein			
289581324	hypothetical protein Nmag_1652	No	2	YES	Virus_Caudovi	1673904	1674179	-		Natrialba_magadii_ATCC_43099_uid46245	NC_013922	chromosome									
289581325	hypothetical protein Nmag_1653	No	0	YES	Virus_Caudovi	1674176	1674385	-		Natrialba_magadii_ATCC_43099_uid46245	NC_013922	chromosome									
289581326	hypothetical protein Nmag_1654	No	2	YES	Virus_Caudovi	1674375	1674728	-		Natrialba_magadii_ATCC_43099_uid46245	NC_013922	chromosome	clust_46								
289581327	hypothetical protein Nmag_1655	No	0	YES	Virus_Caudovi	1674721	1675161	-		Natrialba_magadii_ATCC_43099_uid46245	NC_013922	chromosome	clust_238								
289581328	hypothetical protein Nmag_1656	No	0	YES	Virus_Caudovi	1675165	1675674	-		Natrialba_magadii_ATCC_43099_uid46245	NC_013922	chromosome	clust_195								
289581329	hypothetical protein Nmag_1657	No	0	YES	Virus_Caudovi	1675852	1676097	-		Natrialba_magadii_ATCC_43099_uid46245	NC_013922	chromosome		arCOG00394	7 (-)	K	ArsR	Transcriptional regulator containing HTH domain, ArsR family			
===									6	Natrialba_magadii_ATCC_43099_uid46245	NC_013922	chromosome									
289581557	hypothetical protein Nmag_1888	No	0			1917161	1918138	-		Natrialba_magadii_ATCC_43099_uid46245	NC_013922	chromosome		arCOG09132	1 (-)	S		Uncharacterized conserved protein			
289581558	hypothetical protein Nmag_1889	No	4			1918497	1918952	-		Natrialba_magadii_ATCC_43099_uid46245	NC_013922	chromosome									
289581559	hypothetical protein Nmag_1891	No	0			1920136	1920546	+		Natrialba_magadii_ATCC_43099_uid46245	NC_013922	chromosome									
289581560	hypothetical protein Nmag_1892	No	0			1920612	1920782	+		Natrialba_magadii_ATCC_43099_uid46245	NC_013922	chromosome									
289581561	hypothetical protein Nmag_1893	No	0			1920775	1921581	+		Natrialba_magadii_ATCC_43099_uid46245	NC_013922	chromosome									
289581562	phosphoribosyl-AMP cyclohydrolase	No	0			1922968	1923408	+		Natrialba_magadii_ATCC_43099_uid46245	NC_013922	chromosome		arCOG02676	1 (-)	E	HisI	Phosphoribosyl-AMP cyclohydrolase	3E-45	COG0139	Phosphoribosyl-AMP cyclohydrolase
===									7	Natrialba_magadii_ATCC_43099_uid46245	NC_013922	chromosome									
289581564	hypothetical protein Nmag_1897	No	0			1925616	1925822	+		Natrialba_magadii_ATCC_43099_uid46245	NC_013922	chromosome									
289581565	hypothetical protein Nmag_1898	No	2			1925878	1926048	+		Natrialba_magadii_ATCC_43099_uid46245	NC_013922	chromosome									
289581566	polysaccharide deacetylase	Yes	0			1926342	1927631	-		Natrialba_magadii_ATCC_43099_uid46245	NC_013922	chromosome									
289581567	acetyltransferase (isoleucine patch superfamily	No	0			1928019	1928729	+		Natrialba_magadii_ATCC_43099_uid46245	NC_013922	chromosome		arCOG01854	1 (-)	R	WbbJ	Acetyltransferase (isoleucine patch superfamily)	6E-18	COG0110	Acetyltransferase (isoleucine patch supe
289581568	hypothetical protein Nmag_1901	Yes	2			1928706	1928954	-		Natrialba_magadii_ATCC_43099_uid46245	NC_013922	chromosome									
289581569	hypothetical protein Nmag_1902	No	0			1929093	1929287	+		Natrialba_magadii_ATCC_43099_uid46245	NC_013922	chromosome									
289581570	hypothetical protein Nmag_1903	No	0			1929299	1929814	+		Natrialba_magadii_ATCC_43099_uid46245	NC_013922	chromosome									
===									5	Natrialba_magadii_ATCC_43099_uid46245	NC_013922	chromosome									
289581870	hypothetical protein Nmag_2207	No	2			2263993	2264181	-		Natrialba_magadii_ATCC_43099_uid46245	NC_013922	chromosome									
289581871	CCA-adding enzyme	No	0			2264260	2265753	-		Natrialba_magadii_ATCC_43099_uid46245	NC_013922	chromosome		arCOG04249	1 (-)	J	CCA1	tRNA nucleotidyltransferase (CCA-adding enzyme	5E-109	COG1746	tRNA nucleotidyltransferase (CCA-adding
289581872	hypothetical protein Nmag_2209	No	0			2266192	2266653	+		Natrialba_magadii_ATCC_43099_uid46245	NC_013922	chromosome		arCOG10716	1	S		Uncharacterized conserved protein			
289581873	hypothetical protein Nmag_2210	Yes	0			2266892	2268040	-		Natrialba_magadii_ATCC_43099_uid46245	NC_013922	chromosome							1E-11	pfam05908	Protein of unknown function (DUF867). Th
289581874	hypothetical protein Nmag_2211	No	1			2268248	2269342	-		Natrialba_magadii_ATCC_43099_uid46245	NC_013922	chromosome							0.00000002	pfam05908	Protein of unknown function (DUF867). Th
===									7	Natrialba_magadii_ATCC_43099_uid46245	NC_013922	chromosome									
289582826	restriction endonuclease-like protein	No	0	YES		3297044	3297685	-		Natrialba_magadii_ATCC_43099_uid46245	NC_013922	chromosome		arCOG03898	7 (-)	V	McrA	Restriction endonuclease, McrA/HNH family	9E-17	COG3440	Predicted restriction endonuclease
289582827	hypothetical protein Nmag_3179	No	2	YES		3297889	3298074	-		Natrialba_magadii_ATCC_43099_uid46245	NC_013922	chromosome									
289582828	hypothetical protein Nmag_3180	No	0	YES		3298197	3298337	-		Natrialba_magadii_ATCC_43099_uid46245	NC_013922	chromosome									
289582829	hypothetical protein Nmag_3182	No	0	YES		3299236	3300129	-		Natrialba_magadii_ATCC_43099_uid46245	NC_013922	chromosome									
289582830	hypothetical protein Nmag_3183	No	2	YES		3300332	3300523	-		Natrialba_magadii_ATCC_43099_uid46245	NC_013922	chromosome									
289582831	hypothetical protein Nmag_3185	No	2	YES		3301638	3302189	+		Natrialba_magadii_ATCC_43099_uid46245	NC_013922	chromosome									
289582832	transposase, IS605 OrfB family	No	0	YES		3302261	3303583	-		Natrialba_magadii_ATCC_43099_uid46245	NC_013922	chromosome		arCOG00682	1 (-)	L		Transposase	2E-13	COG0675	Transposase and inactivated derivatives
===									5	Natrialba_magadii_ATCC_43099_uid46245	NC_013923	plasmid pNMAG01									
289583325	hypothetical protein Nmag_3624	No	0		plasmid	10381	10608	-		Natrialba_magadii_ATCC_43099_uid46245	NC_013923	plasmid pNMAG01									
289583326	PhiH1 repressor-like protein	No	0		plasmid	10807	11103	+		Natrialba_magadii_ATCC_43099_uid46245	NC_013923	plasmid pNMAG01		arCOG03924	10 (-)	K	MarR	Transcriptional regulator, MarR family			
289583327	hypothetical protein Nmag_3626	No	0		plasmid	11387	12028	+		Natrialba_magadii_ATCC_43099_uid46245	NC_013923	plasmid pNMAG01							6E-13	pfam13643	Domain of unknown function (DUF4145). Th
289583328	hypothetical protein Nmag_3627	No	0		plasmid	12103	12273	-		Natrialba_magadii_ATCC_43099_uid46245	NC_013923	plasmid pNMAG01									
289583329	hypothetical protein Nmag_3628	Yes	0		plasmid	12524	13024	+		Natrialba_magadii_ATCC_43099_uid46245	NC_013923	plasmid pNMAG01									
===									6	Natrialba_magadii_ATCC_43099_uid46245	NC_013923	plasmid pNMAG01									
289583382	hypothetical protein Nmag_3686	No	0		plasmid	71083	71832	+		Natrialba_magadii_ATCC_43099_uid46245	NC_013923	plasmid pNMAG01									
289583383	hypothetical protein Nmag_3687	No	0		plasmid	72075	72227	+		Natrialba_magadii_ATCC_43099_uid46245	NC_013923	plasmid pNMAG01									
289583384	hypothetical protein Nmag_3688	No	0		plasmid	72553	72657	+		Natrialba_magadii_ATCC_43099_uid46245	NC_013923	plasmid pNMAG01									
289583385	hypothetical protein Nmag_3689	No	0		plasmid	72846	73307	-		Natrialba_magadii_ATCC_43099_uid46245	NC_013923	plasmid pNMAG01		arCOG01207	2 (-)	K		Predicted transcriptional regulator, MarR family			
289583386	hypothetical protein Nmag_3690	No	0	YES	plasmid	73417	73704	+		Natrialba_magadii_ATCC_43099_uid46245	NC_013923	plasmid pNMAG01									
289583387	hypothetical protein Nmag_3691	Yes	0	YES	plasmid	73711	73878	+		Natrialba_magadii_ATCC_43099_uid46245	NC_013923	plasmid pNMAG01									
===									5	Natrialba_magadii_ATCC_43099_uid46245	NC_013924	plasmid pNMAG02									
289937605	hypothetical protein Nmag_4126	Yes	4		plasmid	177738	178154	+		Natrialba_magadii_ATCC_43099_uid46245	NC_013924	plasmid pNMAG02									
289937606	hypothetical protein Nmag_4127	No	1		plasmid	178151	178339	+		Natrialba_magadii_ATCC_43099_uid46245	NC_013924	plasmid pNMAG02									
289937607	transposase, IS605 OrfB family	No	0		plasmid	178336	179529	-		Natrialba_magadii_ATCC_43099_uid46245	NC_013924	plasmid pNMAG02		arCOG00679	4 (-)	L		Transposase	2E-13	COG0675	Transposase and inactivated derivatives
289937608	hypothetical protein Nmag_4129	No	2		plasmid	179781	180086	+		Natrialba_magadii_ATCC_43099_uid46245	NC_013924	plasmid pNMAG02									
289937609	hypothetical protein Nmag_4130	No	0		plasmid	180188	180352	-		Natrialba_magadii_ATCC_43099_uid46245	NC_013924	plasmid pNMAG02									
===									7	Natrialba_magadii_ATCC_43099_uid46245	NC_013924	plasmid pNMAG02									
289937632	hypothetical protein Nmag_4157	No	0		plasmid	204697	204843	+		Natrialba_magadii_ATCC_43099_uid46245	NC_013924	plasmid pNMAG02									
289937633	hypothetical protein Nmag_4158	No	0		plasmid	204854	205696	-		Natrialba_magadii_ATCC_43099_uid46245	NC_013924	plasmid pNMAG02		arCOG08149	2 (-)	S		Uncharacterized conserved protein			
289937634	hypothetical protein Nmag_4159	No	0		plasmid	205932	206384	-		Natrialba_magadii_ATCC_43099_uid46245	NC_013924	plasmid pNMAG02									
289937635	hypothetical protein Nmag_4160	No	0		plasmid	206381	207631	-		Natrialba_magadii_ATCC_43099_uid46245	NC_013924	plasmid pNMAG02									
289937636	hypothetical protein Nmag_4161	No	0		plasmid	207947	208393	+		Natrialba_magadii_ATCC_43099_uid46245	NC_013924	plasmid pNMAG02									
289937637	hypothetical protein Nmag_4162	No	2		plasmid	208867	209178	+		Natrialba_magadii_ATCC_43099_uid46245	NC_013924	plasmid pNMAG02									
289937638	Transposase and inactivated derivatives-like p	No	0		plasmid	209570	210585	-		Natrialba_magadii_ATCC_43099_uid46245	NC_013924	plasmid pNMAG02		arCOG02128	6 (-)	L		Transposase	2E-13	pfam13358	DDE superfamily endonuclease. This famil ; Winged helix-turn helix. This helix-turn
===									39	Natrialba_magadii_ATCC_43099_uid46245	NC_013925	plasmid pNMAG03									
289594274	Proliferating cell nuclear antigen, PCNA	No	0	YES	plasmid	11	754	+		Natrialba_magadii_ATCC_43099_uid46245	NC_013925	plasmid pNMAG03		arCOG00488	4 (-)	L	DnaN	DNA polymerase sliding clamp subunit (PCNA ho	1E-10	COG0592	DNA polymerase sliding clamp subunit (PC
289594275	hypothetical protein Nmag_4212	No	0	YES	plasmid	764	1195	+		Natrialba_magadii_ATCC_43099_uid46245	NC_013925	plasmid pNMAG03									
289594276	hypothetical protein Nmag_4213	No	0	YES	plasmid	1195	1410	+		Natrialba_magadii_ATCC_43099_uid46245	NC_013925	plasmid pNMAG03									
289594277	hypothetical protein Nmag_4214	No	0	YES	plasmid	1388	1630	+		Natrialba_magadii_ATCC_43099_uid46245	NC_013925	plasmid pNMAG03									
289594278	hypothetical protein Nmag_4215	No	0	YES	plasmid	1623	1925	+		Natrialba_magadii_ATCC_43099_uid46245	NC_013925	plasmid pNMAG03									
289594279	hypothetical protein Nmag_4216	No	0	YES	plasmid	1915	2115	+		Natrialba_magadii_ATCC_43099_uid46245	NC_013925	plasmid pNMAG03									
289594280	hypothetical protein Nmag_4217	No	0	YES	plasmid	2105	2332	+		Natrialba_magadii_ATCC_43099_uid46245	NC_013925	plasmid pNMAG03									
289594281	hypothetical protein Nmag_4218	No	0	YES	plasmid	2332	2496	+		Natrialba_magadii_ATCC_43099_uid46245	NC_013925	plasmid pNMAG03									
289594282	hypothetical protein Nmag_4219	No	0	YES	plasmid	2493	2909	+		Natrialba_magadii_ATCC_43099_uid46245	NC_013925	plasmid pNMAG03									
289594283	hypothetical protein Nmag_4220	No	0	YES	plasmid	2899	3279	+		Natrialba_magadii_ATCC_43099_uid46245	NC_013925	plasmid pNMAG03									
289594284	Gp37Gp68 family protein	No	0	YES	plasmid	3269	4141	+		Natrialba_magadii_ATCC_43099_uid46245	NC_013925	plasmid pNMAG03							3E-57	pfam07505	Phage protein Gp37/Gp68. Homologues of p
289594285	hypothetical protein Nmag_4222	No	0	YES	plasmid	4131	4364	+		Natrialba_magadii_ATCC_43099_uid46245	NC_013925	plasmid pNMAG03									
289594286	hypothetical protein Nmag_4223	No	0	YES	plasmid	4361	4795	+		Natrialba_magadii_ATCC_43099_uid46245	NC_013925	plasmid pNMAG03							0.0000001	pfam14216	Domain of unknown function (DUF4326). Th
289594287	hypothetical protein Nmag_4224	No	0	YES	plasmid	4788	5087	+		Natrialba_magadii_ATCC_43099_uid46245	NC_013925	plasmid pNMAG03									
289594288	hypothetical protein Nmag_4225	No	0	YES	plasmid	5084	5521	+		Natrialba_magadii_ATCC_43099_uid46245	NC_013925	plasmid pNMAG03									
289594289	hypothetical protein Nmag_4226	No	0	YES	plasmid	5508	6011	+		Natrialba_magadii_ATCC_43099_uid46245	NC_013925	plasmid pNMAG03									

289594290	hypothetical protein Nmag_4227	No	0	YES	plasmid	6004	6624	+		Natrialba_magadii_ATCC_43099_uid46245	NC_013925	plasmid pNMAG03											
289594291	hypothetical protein Nmag_4228	No	1	YES	plasmid	6614	6841	+		Natrialba_magadii_ATCC_43099_uid46245	NC_013925	plasmid pNMAG03											
289594292	integrin alpha beta-propellor repeat protein	No	0	YES	plasmid	6834	7001	+		Natrialba_magadii_ATCC_43099_uid46245	NC_013925	plasmid pNMAG03											
289594293	hypothetical protein Nmag_4230	No	0	YES	plasmid	6988	7164	+		Natrialba_magadii_ATCC_43099_uid46245	NC_013925	plasmid pNMAG03											
289594294	hypothetical protein Nmag_4231	No	0	YES	plasmid	7157	7702	+		Natrialba_magadii_ATCC_43099_uid46245	NC_013925	plasmid pNMAG03											
289594295	hypothetical protein Nmag_4232	No	0	YES	plasmid	7699	8481	+		Natrialba_magadii_ATCC_43099_uid46245	NC_013925	plasmid pNMAG03											
289594296	DNA-cytosine methyltransferase	No	0	YES	plasmid	8474	9325	+		Natrialba_magadii_ATCC_43099_uid46245	NC_013925	plasmid pNMAG03			arCOG04157	7	(-)	L	Dcm	Site-specific DNA methylase	2E-32	COG0270	Site-specific DNA methylase
289594297	hypothetical protein Nmag_4234	No	0	YES	plasmid	9318	9638	+		Natrialba_magadii_ATCC_43099_uid46245	NC_013925	plasmid pNMAG03											
289594298	DNA methylase N-4/N-6 domain protein	No	0	YES	plasmid	9708	11087	+		Natrialba_magadii_ATCC_43099_uid46245	NC_013925	plasmid pNMAG03			arCOG00115	10	(-)	L		DNA modification methylase	9E-12	pfam01555	DNA methylase. Members of this family ar ; DNA methylase. Members of this family ar
289594299	hypothetical protein Nmag_4236	No	0	YES	plasmid	11084	11659	+		Natrialba_magadii_ATCC_43099_uid46245	NC_013925	plasmid pNMAG03											
289594300	hypothetical protein Nmag_4237	No	0	YES	plasmid	11656	12420	+		Natrialba_magadii_ATCC_43099_uid46245	NC_013925	plasmid pNMAG03											
289594301	hypothetical protein Nmag_4238	No	0	YES	plasmid	12413	12628	+		Natrialba_magadii_ATCC_43099_uid46245	NC_013925	plasmid pNMAG03											
289594302	hypothetical protein Nmag_4239	No	0	YES	plasmid	12621	12902	+		Natrialba_magadii_ATCC_43099_uid46245	NC_013925	plasmid pNMAG03											
289594303	hypothetical protein Nmag_4240	No	0	YES	plasmid	12895	13254	+		Natrialba_magadii_ATCC_43099_uid46245	NC_013925	plasmid pNMAG03											
289594304	hypothetical protein Nmag_4241	No	0	YES	plasmid	13247	13600	+		Natrialba_magadii_ATCC_43099_uid46245	NC_013925	plasmid pNMAG03											
289594305	hypothetical protein Nmag_4242	No	0	YES	plasmid	13883	14467	+		Natrialba_magadii_ATCC_43099_uid46245	NC_013925	plasmid pNMAG03											
289594306	hypothetical protein Nmag_4243	No	0	YES	plasmid	14495	14869	+		Natrialba_magadii_ATCC_43099_uid46245	NC_013925	plasmid pNMAG03											
289594307	hypothetical protein Nmag_4244	No	1	YES	plasmid	15215	15469	+		Natrialba_magadii_ATCC_43099_uid46245	NC_013925	plasmid pNMAG03											
289594308	hypothetical protein Nmag_4245	No	3	YES	plasmid	15453	15785	+		Natrialba_magadii_ATCC_43099_uid46245	NC_013925	plasmid pNMAG03											
289594309	DNA methylase N-4/N-6 domain protein	No	0	YES	plasmid	15862	17121	+		Natrialba_magadii_ATCC_43099_uid46245	NC_013925	plasmid pNMAG03			arCOG00115	10	(-)	L		DNA modification methylase	1E-20	pfam01555	DNA methylase. Members of this family ar
289594310	hypothetical protein Nmag_4247	No	0	YES	plasmid	17096	17650	+		Natrialba_magadii_ATCC_43099_uid46245	NC_013925	plasmid pNMAG03											
289594311	hypothetical protein Nmag_4248	No	0	YES	plasmid	17665	18003	+		Natrialba_magadii_ATCC_43099_uid46245	NC_013925	plasmid pNMAG03											
289594312	hypothetical protein Nmag_4249	No	0	YES	plasmid	18069	18278	+		Natrialba_magadii_ATCC_43099_uid46245	NC_013925	plasmid pNMAG03			arCOG11198	4		S		Uncharacterized conserved protein			
===									20	Natrialba_magadii_ATCC_43099_uid46245	NC_013925	plasmid pNMAG03											
289594314	hypothetical protein Nmag_4251	No	0	YES	plasmid	18635	19237	+		Natrialba_magadii_ATCC_43099_uid46245	NC_013925	plasmid pNMAG03			arCOG09210	6	(-)	S		Uncharacterized conserved protein			
289594315	hypothetical protein Nmag_4252	No	0	YES	plasmid	19230	20894	+		Natrialba_magadii_ATCC_43099_uid46245	NC_013925	plasmid pNMAG03			arCOG09550	17		Y	TerL	Phage terminase large subunit			
289594316	hypothetical protein Nmag_4253	No	0	YES	plasmid	20900	21025	+		Natrialba_magadii_ATCC_43099_uid46245	NC_013925	plasmid pNMAG03											
289594317	hypothetical protein Nmag_4254	No	0	YES	plasmid	21012	21317	+		Natrialba_magadii_ATCC_43099_uid46245	NC_013925	plasmid pNMAG03											
289594318	hypothetical protein Nmag_4255	No	0	YES	plasmid	21310	21495	+		Natrialba_magadii_ATCC_43099_uid46245	NC_013925	plasmid pNMAG03											
289594319	hypothetical protein Nmag_4256	No	0	YES	plasmid	21492	21770	+		Natrialba_magadii_ATCC_43099_uid46245	NC_013925	plasmid pNMAG03											
289594320	portal protein	No	0	YES	plasmid	21767	23314	+		Natrialba_magadii_ATCC_43099_uid46245	NC_013925	plasmid pNMAG03			arCOG11199	3		S		Uncharacterized conserved protein	0.00000003	COG5518	Bacteriophage capsid portal protein
289594321	putative head morphogenesis protein SPP1 gp	No	0	YES	plasmid	23321	24268	+		Natrialba_magadii_ATCC_43099_uid46245	NC_013925	plasmid pNMAG03											
289594322	hypothetical protein Nmag_4259	No	0	YES	plasmid	24347	26233	+		Natrialba_magadii_ATCC_43099_uid46245	NC_013925	plasmid pNMAG03									0.00006	pfam04006	Mpp10 protein. This family includes prot
289594323	hypothetical protein Nmag_4260	No	0	YES	plasmid	26277	27242	+		Natrialba_magadii_ATCC_43099_uid46245	NC_013925	plasmid pNMAG03											
289594324	hypothetical protein Nmag_4261	No	0	YES	plasmid	27252	27695	+		Natrialba_magadii_ATCC_43099_uid46245	NC_013925	plasmid pNMAG03									0.000007	pfam04147	Nop14-like family. Emg1 and Nop14 are no
289594325	hypothetical protein Nmag_4262	No	0	YES	plasmid	27702	28298	+		Natrialba_magadii_ATCC_43099_uid46245	NC_013925	plasmid pNMAG03											
289594326	hypothetical protein Nmag_4263	No	0	YES	plasmid	28295	28675	+		Natrialba_magadii_ATCC_43099_uid46245	NC_013925	plasmid pNMAG03											
289594327	hypothetical protein Nmag_4264	No	0	YES	plasmid	28677	28958	+		Natrialba_magadii_ATCC_43099_uid46245	NC_013925	plasmid pNMAG03											
289594328	hypothetical protein Nmag_4265	No	0	YES	plasmid	28958	29395	+		Natrialba_magadii_ATCC_43099_uid46245	NC_013925	plasmid pNMAG03			arCOG08300	5	(-)	Y		Phage virion morphogenesis family, Mu gpG-like			
289594329	hypothetical protein Nmag_4266	No	0	YES	plasmid	29412	30008	+		Natrialba_magadii_ATCC_43099_uid46245	NC_013925	plasmid pNMAG03											
289594330	hypothetical protein Nmag_4267	No	0	YES	plasmid	30045	31325	+		Natrialba_magadii_ATCC_43099_uid46245	NC_013925	plasmid pNMAG03											
289594331	hypothetical protein Nmag_4268	No	0	YES	plasmid	31342	31734	+		Natrialba_magadii_ATCC_43099_uid46245	NC_013925	plasmid pNMAG03											
289594332	hypothetical protein Nmag_4269	No	0	YES	plasmid	31734	32114	+		Natrialba_magadii_ATCC_43099_uid46245	NC_013925	plasmid pNMAG03											
289594333	HNH nuclease	No	0	YES	plasmid	32438	33214	+		Natrialba_magadii_ATCC_43099_uid46245	NC_013925	plasmid pNMAG03			arCOG03898	7	(-)	V	McrA	Restriction endonuclease, McrA/HNH family			
===									5	Natrialba_magadii_ATCC_43099_uid46245	NC_013925	plasmid pNMAG03											
289594336	hypothetical protein Nmag_4273	No	0	YES	plasmid	36017	36571	+		Natrialba_magadii_ATCC_43099_uid46245	NC_013925	plasmid pNMAG03									0.00004	pfam07423	Protein of unknown function (DUF1510). T
289594337	hypothetical protein Nmag_4274	No	0	YES	plasmid	36572	36916	+		Natrialba_magadii_ATCC_43099_uid46245	NC_013925	plasmid pNMAG03											
289594338	hypothetical protein Nmag_4275	No	0	YES	plasmid	36919	37761	+		Natrialba_magadii_ATCC_43099_uid46245	NC_013925	plasmid pNMAG03			arCOG10308	5	(-)	S		Uncharacterized conserved protein			
289594339	hypothetical protein Nmag_4276	No	0	YES	plasmid	37767	38327	+		Natrialba_magadii_ATCC_43099_uid46245	NC_013925	plasmid pNMAG03			arCOG11245	4		S		Uncharacterized conserved protein			
289594340	hypothetical protein Nmag_4277	No	0	YES	plasmid	38351	38707	+		Natrialba_magadii_ATCC_43099_uid46245	NC_013925	plasmid pNMAG03											
===									11	Natrialba_magadii_ATCC_43099_uid46245	NC_013925	plasmid pNMAG03											
289594342	hypothetical protein Nmag_4279	No	0	YES	plasmid	39975	40583	+		Natrialba_magadii_ATCC_43099_uid46245	NC_013925	plasmid pNMAG03			arCOG10698	3	(-)	S		Uncharacterized conserved protein			
289594343	hypothetical protein Nmag_4280	No	0	YES	plasmid	40587	41171	+		Natrialba_magadii_ATCC_43099_uid46245	NC_013925	plasmid pNMAG03											
289594344	hypothetical protein Nmag_4281	No	0	YES	plasmid	41168	41776	+		Natrialba_magadii_ATCC_43099_uid46245	NC_013925	plasmid pNMAG03											
289594345	hypothetical protein Nmag_4282	No	0	YES	plasmid	41769	43025	+		Natrialba_magadii_ATCC_43099_uid46245	NC_013925	plasmid pNMAG03											
289594346	hypothetical protein Nmag_4283	No	0	YES	plasmid	43029	43310	+		Natrialba_magadii_ATCC_43099_uid46245	NC_013925	plasmid pNMAG03	clust_362										
289594347	integrase family protein	No	0	YES	plasmid	43348	44022	+		Natrialba_magadii_ATCC_43099_uid46245	NC_013925	plasmid pNMAG03			arCOG06451	4	(-)	L	XerC	Integrase			
289594348	hypothetical protein Nmag_4285	No	0	YES	plasmid	44033	44320	-		Natrialba_magadii_ATCC_43099_uid46245	NC_013925	plasmid pNMAG03	clust_362										
289594349	hypothetical protein Nmag_4286	No	0	YES	plasmid	44325	45725	-		Natrialba_magadii_ATCC_43099_uid46245	NC_013925	plasmid pNMAG03											
289594350	hypothetical protein Nmag_4287	No	1	YES	plasmid	45759	46484	+		Natrialba_magadii_ATCC_43099_uid46245	NC_013925	plasmid pNMAG03											
289594351	hypothetical protein Nmag_4288	No	0	YES	plasmid	46494	46769	-		Natrialba_magadii_ATCC_43099_uid46245	NC_013925	plasmid pNMAG03			arCOG08095	3	(-)	K		Predicted transcriptional regulator, contains HTH domain			
289594352	hypothetical protein Nmag_4289	No	0	YES	plasmid	46816	47100	-		Natrialba_magadii_ATCC_43099_uid46245	NC_013925	plasmid pNMAG03											
===									12	Natrinema_J7_uid171337	NC_018224	chromosome											
397771809	AAA family ATPase	No	0			26567	28687	+		Natrinema_J7_uid171337	NC_018224	chromosome			arCOG01308	10	(-)	O	Cdc48	ATPase of the AAA+ class , CDC48 family	1E-16	COG1222	ATP-dependent 26S proteasome regulatory
397771810	hypothetical protein NJ7G_0024	No	0			28820	29431	+		Natrinema_J7_uid171337	NC_018224	chromosome	clust_170								5E-13	pfam14065	Protein of unknown function (DUF4255). T
397771811	hypothetical protein NJ7G_0025	Yes	6			29473	30534	-		Natrinema_J7_uid171337	NC_018224	chromosome											
397771812	hypothetical protein NJ7G_0027	No	0			30578	30742	+		Natrinema_J7_uid171337	NC_018224	chromosome											
397771813	hypothetical protein NJ7G_0026	No	0			30739	30882	-		Natrinema_J7_uid171337	NC_018224	chromosome											
397771814	hypothetical protein NJ7G_0028	No	0			30932	31387	-		Natrinema_J7_uid171337	NC_018224	chromosome	clust_6								1E-36	pfam06841	T4-like virus tail tube protein gp19. Th
397771815	hypothetical protein NJ7G_0029	No	0			31436	31645	-		Natrinema_J7_uid171337	NC_018224	chromosome	clust_21										
397771816	hypothetical protein NJ7G_0030	No	0			31690	32250	-		Natrinema_J7_uid171337	NC_018224	chromosome	clust_16										
397771817	hypothetical protein NJ7G_0031	No	0			32408	32866	-		Natrinema_J7_uid171337	NC_018224	chromosome	clust_19								1E-16	pfam06841	T4-like virus tail tube protein gp19. Th
397771818	phage tail sheath protein FI	No	0			32921	34657	-		Natrinema_J7_uid171337	NC_018224	chromosome	clust_11								2E-26	COG3497	Phage tail sheath protein FI
397771819	hypothetical protein NJ7G_0033	No	0			35071	36228	+		Natrinema_J7_uid171337	NC_018224	chromosome	clust_28								0.0001	COG0419	ATPase involved in DNA repair
397771820	Dihydrolipoyllysine-residue acetyltransferase	No	0			36291	37946	-		Natrinema_J7_uid171337	NC_018224	chromosome		arCOG01706,a	arCOG01706	1	(-)	C	AceF	Pyruvate/2-oxoglutarate dehydrogenase comple:	2E-76	COG0508	Pyruvate/2-oxoglutarate dehydrogenase co ; Pyruvate/2-oxoglutarate dehydrogenase co
===									6	Natrinema_J7_uid171337	NC_018224	chromosome											
397771884	hypothetical protein NJ7G_0100	No	0			103079	103228	+		Natrinema_J7_uid171337	NC_018224	chromosome											
397771885	hypothetical protein NJ7G_0101	No	0			103736	103999	-		Natrinema_J7_uid171337	NC_018224	chromosome											
397771886	hypothetical protein NJ7G_0102	No	3			104078	104437	-		Natrinema_J7_uid171337	NC_018224	chromosome			arCOG10789	1	(-)	S		Uncharacterized conserved protein			
397771887	hypothetical protein NJ7G_0103	No	0			104680	104865	-		Natrinema_J7_uid171337	NC_018224	chromosome											
397771888	hypothetical protein NJ7G_0104	No	0			104836	104967	+		Natrinema_J7_uid171337	NC_018224	chromosome											
397771889	hypothetical protein NJ7G_0105	No	0			105397	105594	+		Natrinema_J7_uid171337	NC_018224	chromosome											
===									5	Natrinema_J7_uid171337	NC_018224	chromosome											
397771979	hypothetical protein NJ7G_0194	No	1			187254	187400	+		Natrinema_J7_uid171337	NC_018224	chromosome											
397771980	hypothetical protein NJ7G_0195	No	1			187728	189263	-		Natrinema_J7_uid171337	NC_018224	chromosome											
397771981	hypothetical protein NJ7G_0196	No	0			189588	189767	+		Natrinema_J7_uid171337	NC_018224	chromosome											
397771982	translation elongation factor aEF-2	No	0			189788	191974	-		Natrinema_J7_uid171337	NC_018224	chromosome			arCOG01559	2	(-)	J	FusA	Translation elongation factor G, EF-G (GTPase)	7E-114	COG0480	Translation elongation factors (GTPases)
397771983	hypothetical protein NJ7G_0198	No	0			192000	192170	-		Natrinema_J7_uid171337	NC_018224	chromosome											
===									8	Natrinema_J7_uid171337	NC_018224	chromosome											
397772708	CRISPR-associated protein Cas2	No	0			842366	842638	+		Natrinema_J7_uid171337	NC_018224	chromosome			arCOG04194	3	(-)	V	Cas2	CRISPR-associated protein Cas2	4E-12	COG1343	CRISPR-associated protein Cas2
397772709	hypothetical protein NJ7G_0931	No	0			843682	843834	+		Natrinema_J7_uid171337	NC_018224	chromosome											
397772710	hypothetical protein NJ7G_0932	No	0			844022	844237	+		Natrinema_J7_uid171337	NC_018224	chromosome											
397772711	hypothetical protein NJ7G_0933	No	0			844594	844905	+		Natrinema_J7_uid171337	NC_018224	chromosome											
397772712	hypothetical protein NJ7G_0934	No	0			844994	845200	+		Natrinema_J7_uid171337	NC_018224	chromosome											
397772713	hypothetical protein NJ7G_0935	No	0			845667	846047	-		Natrinema_J7_uid171337	NC_018224	chromosome											
397772714	hypothetical protein NJ7G_0936	No	0			846095	846736	+		Natrinema_J7_uid171337	NC_018224	chromosome			arCOG01241	31	(-)	L	XerC	Integrase			
397772715	integrase family protein	No	0			846714	846920	+		Natrinema_J7_uid171337	NC_018224	chromosome											
===									5	Natrinema_J7_uid171337	NC_018224	chromosome											
397772746	hypothetical protein NJ7G_0969	No	0			871967	872095	+		Natrinema_J7_uid171337	NC_018224	chromosome											
397772747	hypothetical protein NJ7G_0970	No	0			872708	873172	-		Natrinema_J7_uid171337	NC_018224	chromosome											
397772748	cell surface glycoprotein	Yes	0			873240	873977	-		Natrinema_J7_uid171337	NC_018224	chromosome		arCOG06180,a	arCOG06180	2	(-)	P	FepB	ABC-type Fe3+-hydroxamate transport system, periplasmic component			
397772749	hypothetical protein NJ7G_0972	No	0			873974	874192	-		Natrinema_J7_uid171337	NC_018224	chromosome											
397772750	hypothetical protein NJ7G_0973	No	0			874287	874541	-		Natrinema_J7_uid171337	NC_018224	chromosome											
===									5	Natrinema_J7_uid171337	NC_018224	chromosome											
397772786	hypothetical protein NJ7G_1009	No	0			907910	908119	-		Natrinema_J7_uid171337	NC_018224	chromosome											
397772787	oxidoreductase domain protein	No	0			910394	910804	-		Natrinema_J7_uid171337	NC_018224	chromosome									0.00000002	COG0673	Predicted dehydrogenases and related pro
397772788	hypothetical protein NJ7G_1011	No	0			910916	911152	-		Natrinema_J7_uid171337	NC_018224	chromosome											
397772789	Aldehyde Dehydrogenase	No	0			911175	912626	-		Natrinema_J7_uid171337	NC_018224	chromosome			arCOG01252	3	(-)	C	PutA	Lactaldehyde dehydrogenase, Succinate semial	9E-147	pfam00171	Aldehyde dehydrogenase family. This fami
397772790	hypothetical protein NJ7G_1013	No	0			912974	913120	-		Natrinema_J7_uid171337	NC_018224	chromosome											
===									6	Natrinema_J7_uid171337	NC_018224	chromosome											
397772801	hypothetical protein NJ7G_1024	No	2			922168	922377	+		Natrinema_J7_uid171337	NC_018224	chromosome			arCOG08206	1	(-)	S		Uncharacterized conserved protein			
397772802	hypothetical protein NJ7G_1025	No	0			922839	922985	+		Natrinema_J7_uid171337	NC_018224	chromosome			arCOG10932	3		S		Uncharacterized conserved protein			
397772803	hypothetical protein NJ7G_1026	No	0			923177	923299	+		Natrinema_J7_uid171337	NC_018224	chromosome											
397772804	phosphatidylglycerophosphatase A-like protei	No	1			923516	923965	-		Natrinema_J7_uid171337	NC_018224	chromosome									0.000000002	pfam04608	Phosphatidylglycerophosphatase A. This f
397772805	hypothetical protein NJ7G_1028	No	1			924266	924421	-		Natrinema_J7_uid171337	NC_018224	chromosome											
397772806	Mn-dependent transcriptional regulator	No	0			924439	925170	-		Natrinema_J7_uid171337	NC_018224	chromosome			arCOG02099	2	(-)	K	TroR	Mn-dependent transcriptional regulator (DtxR fami	2E-20	COG1321	Mn-dependent transcriptional regulator [
===									5	Natrinema_J7_uid171337	NC_018224	chromosome											
397773064	hypothetical protein NJ7G_1287	No	1			1149117	1149260	+		Natrinema_J7_uid171337	NC_018224	chromosome											
397773065	hypothetical protein NJ7G_1288	No	0			1149319	1149546	-		Natrinema_J7_uid171337	NC_018224	chromosome			arCOG08128	7	(-)	S		Uncharacterized conserved protein			
397773066	hypothetical protein NJ7G_1289	No	0			1149833	1150036	+		Natrinema_J7_uid171337	NC_018224	chromosome											
397773067	hypothetical protein NJ7G_1290	No	0			1150036	1150491	+		Natrinema_J7_uid171337	NC_018224	chromosome											
397773068	hypothetical protein NJ7G_1291	No	0			1150481	1150693	+		Natrinema_J7_uid171337	NC_018224	chromosome											
===									5	Natrinema_J7_uid171337	NC_018224	chromosome											
397773140	hypothetical protein NJ7G_1364	No	0			1216649	1216765	+		Natrinema_J7_uid171337	NC_018224	chromosome											
397773141	hypothetical protein NJ7G_1363	No	1			1216729	1217109	-		Natrinema_J7_uid171337	NC_018224	chromosome											
397773142	NAD-dependent epimerase/dehydratase	No	0			1217259	1218248	+		Natrinema_J7_uid171337	NC_018224	chromosome			arCOG01369	5	(-)	M	WcaG	Nucleoside-diphosphate-sugar epimerase	8E-50	COG0451	Nucleoside-diphosphate-sugar epimerases
397773143	hypothetical protein NJ7G_1366	No	0			1218261	1218449	+		Natrinema_J7_uid171337	NC_018224	chromosome											
397773144	hypothetical protein NJ7G_1367	No	0			1218521	1218649	-		Natrinema_J7_uid171337	NC_018224	chromosome											
===									5	Natrinema_J7_uid171337	NC_018224	chromosome											
397773180	hypothetical protein NJ7G_1403	No	0			1252589	1252801	+		Natrinema_J7_uid171337	NC_018224	chromosome											
397773181	hypothetical protein NJ7G_1404	No	0			1252798	1252917	+		Natrinema_J7_uid171337	NC_018224	chromosome											
397773182	hypothetical protein NJ7G_1405	No	2			1253066	1253662	-		Natrinema_J7_uid171337	NC_018224	chromosome			arCOG03828	5	(-)	S		Uncharacterized conserved protein			
397773183	hypothetical protein NJ7G_1406	No	0			1253822	1254004	-		Natrinema_J7_uid171337	NC_018224	chromosome											
397773184	hypothetical protein NJ7G_1407	No	0			1254173	1254370	+		Natrinema_J7_uid171337	NC_018224	chromosome											
===									11	Natrinema_J7_uid171337	NC_018224	chromosome											
397773318	hypothetical protein NJ7G_1542	No	0			1384244	1384360	-		Natrinema_J7_uid171337	NC_018224	chromosome											
397773319	AAA family ATPase, CDC48 subfamily	No	0			1384457	1386688	-		Natrinema_J7_uid171337	NC_018224	chromosome			arCOG01308	10	(-)	O	Cdc48	ATPase of the AAA+ class , CDC48 family		COG0464	ATPases of the AAA+ class
397773320	hypothetical protein NJ7G_1544	No	0			1386833	1387186	-		Natrinema_J7_uid171337	NC_018224	chromosome											
397773321	hypothetical protein NJ7G_1545	Yes	1			1387237	1387854	-		Natrinema_J7_uid171337	NC_018224	chromosome									0.000003	pfam10446	Protein of unknown function (DUF2457). T
397773322	hypothetical protein NJ7G_1546	No	0			1387964	1388377	-		Natrinema_J7_uid171337	NC_018224	chromosome											
397773323	hypothetical protein NJ7G_1548	No	0			1389050	1389271	-		Natrinema_J7_uid171337	NC_018224	chromosome			arCOG09253	1	(-)	S		Uncharacterized conserved protein			
397773324	hypothetical protein NJ7G_1549	No	0			1389516	1389656	+		Natrinema_J7_uid171337	NC_018224	chromosome											
397773325	hypothetical protein NJ7G_1550	Yes	1			1389693	1390454	-		Natrinema_J7_uid171337	NC_018224	chromosome											
397773326	hypothetical protein NJ7G_1551	Yes	1			1391002	1391391	-		Natrinema_J7_uid171337	NC_018224	chromosome											
397773327	hypothetical protein NJ7G_1552	No	0			1391697	1391864	+		Natrinema_J7_uid171337	NC_018224	chromosome											
397773328	hypothetical protein NJ7G_1553	No	0			1392144	1393646	+		Natrinema_J7_uid171337	NC_018224	chromosome			arCOG10751	2	(-)	S		Uncharacterized conserved protein			
===									27	Natrinema_J7_uid171337	NC_018224	chromosome											
397773535	hypothetical protein NJ7G_1763	No	0			1572927	1573115	-		Natrinema_J7_uid171337	NC_018224	chromosome											
397773536	hypothetical protein NJ7G_1764	No	0			1573440	1574432	+		Natrinema_J7_uid171337	NC_018224	chromosome			arCOG00033	1	(-)	J	Trm5	Wybutosine (yW) biosynthesis enzyme, Trm5 me	1E-60	COG2520	Predicted methyltransferase
397773537	hypothetical protein NJ7G_1766	No	0			1574754	1574987	+		Natrinema_J7_uid171337	NC_018224	chromosome			arCOG06155	6		S		Uncharacterized conserved protein			
397773538	hypothetical protein NJ7G_1765	No	0			1574978	1575136	-		Natrinema_J7_uid171337	NC_018224	chromosome											
397773539	hypothetical protein NJ7G_1767	No	0			1575189	1575374	-		Natrinema_J7_uid171337	NC_018224	chromosome											
397773540	hypothetical protein NJ7G_1768	No	0			1575353	1576117	+		Natrinema_J7_uid171337	NC_018224	chromosome									0.0001	COG1196	Chromosome segregation ATPases
397773541	hypothetical protein NJ7G_1769	No	0			1576104	1577651	+		Natrinema_J7_uid171337	NC_018224	chromosome			arCOG05978	2		M		Extracellular protein containing Kelch and FN3 domains			
397773542	hypothetical protein NJ7G_1770	Yes	0			1577826	1578170	-		Natrinema_J7_uid171337	NC_018224	chromosome	clust_309										
397773543	hypothetical protein NJ7G_1771	No	0			1578547	1579416	-		Natrinema_J7_uid171337	NC_018224	chromosome											
397773544	hypothetical protein NJ7G_1772	Yes	0			1579409	1579738	-		Natrinema_J7_uid171337	NC_018224	chromosome											
397773545	hypothetical protein NJ7G_1773	No	0			1579768	1579890	+		Natrinema_J7_uid171337	NC_018224	chromosome											
397773546	hypothetical protein NJ7G_1774	No	0			1579880	1580080	+		Natrinema_J7_uid171337	NC_018224	chromosome			arCOG09335	1		S		Uncharacterized conserved protein			
397773547	hypothetical protein NJ7G_1775	No	0			1580333	1581175	+		Natrinema_J7_uid171337	NC_018224	chromosome			arCOG07585	1	(-)	S		Uncharacterized conserved protein			
397773548	hypothetical protein NJ7G_1776	No	0			1581777	1581905	-		Natrinema_J7_uid171337	NC_018224	chromosome											
397773549	hypothetical protein NJ7G_4361	No	0			1581898	1582128	-		Natrinema_J7_uid171337	NC_018224	chromosome			arCOG08128	7	(-)	S		Uncharacterized conserved protein			
397773550	hypothetical protein NJ7G_1777	No	0			1582347	1582547	+		Natrinema_J7_uid171337	NC_018224	chromosome											
397773551	hypothetical protein NJ7G_1778	No	0			1582544	1582804	+		Natrinema_J7_uid171337	NC_018224	chromosome											
397773552	hypothetical protein NJ7G_1779	No	0			1582801	1583004	+		Natrinema_J7_uid171337	NC_018224	chromosome											
397773553	hypothetical protein NJ7G_1780	No	0			1583004	1583444	+		Natrinema_J7_uid171337	NC_018224	chromosome											
397773554	hypothetical protein NJ7G_1781	No	0			1583444	1583788	+		Natrinema_J7_uid171337	NC_018224	chromosome			arCOG07773	5	(-)	S		SWIM zinc finger containing protein			
397773555	hypothetical protein NJ7G_1782	No	0			1583776	1584024	+		Natrinema_J7_uid171337	NC_018224	chromosome	clust_56										
397773556	hypothetical protein NJ7G_1783	Yes	0			1584025	1584222	+		Natrinema_J7_uid171337	NC_018224	chromosome											
397773557	hypothetical protein NJ7G_1784	No	0			1584219	1584401	+		Natrinema_J7_uid171337	NC_018224	chromosome											
397773558	AAA ATPase	No	0			1584524	1585591	+		Natrinema_J7_uid171337	NC_018224	chromosome			arCOG00467	13	(-)	L	CDC6	Cdc6-related protein, AAA superfamily ATPase	2E-18	COG1474	Cdc6-related protein, AAA superfamily AT
397773559	hypothetical protein NJ7G_1786	No	0			1585702	1586451	-		Natrinema_J7_uid171337	NC_018224	chromosome											
397773560	hypothetical protein NJ7G_1788	No	0			1587512	1587673	+		Natrinema_J7_uid171337	NC_018224	chromosome											
397773561	hypothetical protein NJ7G_1789	No	0			1588512	1588778	+		Natrinema_J7_uid171337	NC_018224	chromosome											
===									6	Natrinema_J7_uid171337	NC_018224	chromosome											
397773563	transcriptional regulator PadR family protein	No	0			1589323	1589814	+		Natrinema_J7_uid171337	NC_018224	chromosome			arCOG00006	2	(-)	K		Predicted transcriptional regulator, PadR family			
397773564	hypothetical protein NJ7G_1792	No	0			1589811	1590518	+		Natrinema_J7_uid171337	NC_018224	chromosome											
397773565	hypothetical protein NJ7G_1793	No	0			1590520	1590744	+		Natrinema_J7_uid171337	NC_018224	chromosome											
397773566	hypothetical protein NJ7G_1794	No	0			1590741	1591484	+		Natrinema_J7_uid171337	NC_018224	chromosome	clust_133										
397773567	hypothetical protein NJ7G_1795	No	0			1591481	1591804	+		Natrinema_J7_uid171337	NC_018224	chromosome											
397773568	integrase family protein	No	0			1592013	1592606	-		Natrinema_J7_uid171337	NC_018224	chromosome			arCOG01243	4	(-)	L	XerC	Site-specific recombinase XerC			
===									8	Natrinema_J7_uid171337	NC_018224	chromosome											
397773613	hypothetical protein NJ7G_1842	No	2			1623827	1624405	-		Natrinema_J7_uid171337	NC_018224	chromosome			arCOG08194	1	(-)	S		Uncharacterized conserved protein			
397773614	hypothetical protein NJ7G_1843	No	0			1624605	1625783	+		Natrinema_J7_uid171337	NC_018224	chromosome											
397773615	hypothetical protein NJ7G_1844	No	0			1625917	1626078	+		Natrinema_J7_uid171337	NC_018224	chromosome			arCOG11163	4		S		Uncharacterized conserved protein			
397773616	hypothetical protein NJ7G_1845	No	0			1626144	1626275	-		Natrinema_J7_uid171337	NC_018224	chromosome											
397773617	hypothetical protein NJ7G_1846	No	1			1626507	1626629	-		Natrinema_J7_uid171337	NC_018224	chromosome											
397773618	hypothetical protein NJ7G_1847	No	0			1626822	1626980	+		Natrinema_J7_uid171337	NC_018224	chromosome											
397773619	PhiH1 repressor-like protein	No	0			1627214	1627459	-		Natrinema_J7_uid171337	NC_018224	chromosome			arCOG03924	10	(-)	K	MarR	Transcriptional regulator, MarR family			
397773620	hypothetical protein NJ7G_1849	No	0			1627600	1627752	-		Natrinema_J7_uid171337	NC_018224	chromosome											
===									7	Natrinema_J7_uid171337	NC_018224	chromosome											
397773691	hypothetical protein NJ7G_1920	No	0			1690186	1690371	+		Natrinema_J7_uid171337	NC_018224	chromosome											
397773692	alpha/beta hydrolase fold protein	No	0			1690598	1691431	+		Natrinema_J7_uid171337	NC_018224	chromosome			arCOG01648	3	(-)	R	MhpC	Alpha/beta superfamily hydrolase	2E-13	COG0596	Predicted hydrolases or acyltransferases
397773693	hypothetical protein NJ7G_1922	No	0			1691548	1691661	-		Natrinema_J7_uid171337	NC_018224	chromosome											
397773694	hypothetical protein NJ7G_1923	No	0			1691901	1692014	-		Natrinema_J7_uid171337	NC_018224	chromosome											
397773695	hypothetical protein NJ7G_1924	No	0			1692355	1692519	-		Natrinema_J7_uid171337	NC_018224	chromosome											
397773696	hypothetical protein NJ7G_1925	No	0			1693517	1693651	+		Natrinema_J7_uid171337	NC_018224	chromosome											
397773697	cobW domain protein	No	0			1694104	1694658	-		Natrinema_J7_uid171337	NC_018224	chromosome			arCOG01233	1	(-)	R		GTPase (G3E type)	4E-14	pfam07683	Cobalamin synthesis protein cobW C-termi
===									6	Natrinema_J7_uid171337	NC_018224	chromosome											
397773729	hypothetical protein NJ7G_1957	No	0			1725901	1726020	+		Natrinema_J7_uid171337	NC_018224	chromosome											
397773730	hypothetical protein NJ7G_1958	No	0			1726194	1726385	-		Natrinema_J7_uid171337	NC_018224	chromosome											
397773731	IclR family transcriptional regulator	No	0			1726482	1727261	+		Natrinema_J7_uid171337	NC_018224	chromosome			arCOG02798	1	(-)	K	IclR	Transcriptional regulator (IclR family)	2E-32	COG1414	Transcriptional regulator
397773732	hypothetical protein NJ7G_1960	No	0			1727364	1727651	-		Natrinema_J7_uid171337	NC_018224	chromosome											
397773733	hypothetical protein NJ7G_1961	No	0			1727648	1727800	-		Natrinema_J7_uid171337	NC_018224	chromosome											
397773734	hypothetical protein NJ7G_1962	No	0			1727816	1727965	+		Natrinema_J7_uid171337	NC_018224	chromosome											
===									5	Natrinema_J7_uid171337	NC_018224	chromosome											
397773815	type I site-specific deoxyribonuclease, HsdR f	No	0			1800334	1800489	+		Natrinema_J7_uid171337	NC_018224	chromosome											
397773816	hypothetical protein NJ7G_2044	No	0			1800486	1800689	+		Natrinema_J7_uid171337	NC_018224	chromosome			arCOG02625	3	(-)	R		Predicted metal-dependent hydrolase			
397773817	hypothetical protein NJ7G_2046	No	0			1801964	1802110	+		Natrinema_J7_uid171337	NC_018224	chromosome											
397773818	hypothetical protein NJ7G_2047	No	0			1802591	1802893	+		Natrinema_J7_uid171337	NC_018224	chromosome											
397773819	hypothetical protein NJ7G_2048	No	0			1803040	1803189	-		Natrinema_J7_uid171337	NC_018224	chromosome											
===									6	Natrinema_J7_uid171337	NC_018224	chromosome											
397773890	restriction endonuclease-like protein	No	0			1852737	1853735	+		Natrinema_J7_uid171337	NC_018224	chromosome			arCOG09398	1	(-)	S		Uncharacterized conserved protein	3E-16	COG3440	Predicted restriction endonuclease
397773891	hypothetical protein NJ7G_2120	No	0			1853886	1854632	-		Natrinema_J7_uid171337	NC_018224	chromosome									0.000007	COG1196	Chromosome segregation ATPases
397773892	hypothetical protein NJ7G_2122	No	0			1854728	1854850	+		Natrinema_J7_uid171337	NC_018224	chromosome											
397773893	hypothetical protein NJ7G_2121	No	0			1854847	1855062	-		Natrinema_J7_uid171337	NC_018224	chromosome											
397773894	hypothetical protein NJ7G_2123	No	0			1855190	1855312	-		Natrinema_J7_uid171337	NC_018224	chromosome											
397773895	hypothetical protein NJ7G_2124	No	0			1855676	1855819	-		Natrinema_J7_uid171337	NC_018224	chromosome			arCOG09142	1	(-)	S		Uncharacterized conserved protein			
===									8	Natrinema_J7_uid171337	NC_018224	chromosome											
397773907	Bacterio-opsin activator HTH domain protein	No	0			1864671	1865321	-		Natrinema_J7_uid171337	NC_018224	chromosome			arCOG02274	3	(-)	K		Predicted transcriptional regulator, C-terminal HT	8E-11	pfam04967	HTH DNA binding domain.
397773908	hypothetical protein NJ7G_2137	No	0			1865318	1865464	-		Natrinema_J7_uid171337	NC_018224	chromosome											
397773909	hypothetical protein NJ7G_2138	No	0			1865461	1865610	-		Natrinema_J7_uid171337	NC_018224	chromosome											
397773910	hypothetical protein NJ7G_2139	No	0			1865617	1865799	-		Natrinema_J7_uid171337	NC_018224	chromosome											
397773911	hypothetical protein NJ7G_2140	Yes	1			1865881	1866492	+		Natrinema_J7_uid171337	NC_018224	chromosome	clust_171										
397773912	hypothetical protein NJ7G_2141	No	3			1866499	1867065	+		Natrinema_J7_uid171337	NC_018224	chromosome	clust_176										
397773913	hypothetical protein NJ7G_2142	Yes	1			1867103	1867945	+		Natrinema_J7_uid171337	NC_018224	chromosome			arCOG08948	1	(-)	R		Ferritin family protein	0.0000003	pfam07263	Dentin matrix protein 1 (DMP1). This fam
397773914	hypothetical protein NJ7G_2143	No	3			1867942	1868388	+		Natrinema_J7_uid171337	NC_018224	chromosome	clust_228										
===									5	Natrinema_J7_uid171337	NC_018224	chromosome											
397773932	hypothetical protein NJ7G_2165	No	0			1883149	1883448	-		Natrinema_J7_uid171337	NC_018224	chromosome											
397773933	hypothetical protein NJ7G_2166	No	0			1883707	1884078	+		Natrinema_J7_uid171337	NC_018224	chromosome											
397773934	threonyl-tRNA synthetase	No	0			1884136	1886082	-		Natrinema_J7_uid171337	NC_018224	chromosome			arCOG00401	1	(-)	J	ThrS	Threonyl-tRNA synthetase		COG0441	Threonyl-tRNA synthetase
397773935	hypothetical protein NJ7G_2168	No	0			1886397	1886522	+		Natrinema_J7_uid171337	NC_018224	chromosome											
397773936	hypothetical protein NJ7G_2169	No	0			1886920	1887240	+		Natrinema_J7_uid171337	NC_018224	chromosome											
===									11	Natrinema_J7_uid171337	NC_018224	chromosome											
397774019	hypothetical protein NJ7G_2251	No	0			1959949	1960068	-		Natrinema_J7_uid171337	NC_018224	chromosome											
397774020	hypothetical protein NJ7G_2252	Yes	1			1960985	1962025	+		Natrinema_J7_uid171337	NC_018224	chromosome			arCOG02918	1	(-)	C	PetE	Plastocyanin			
397774021	hypothetical protein NJ7G_2254	No	3			1962685	1962978	-		Natrinema_J7_uid171337	NC_018224	chromosome											
397774022	hypothetical protein NJ7G_2255	No	3			1963177	1963503	-		Natrinema_J7_uid171337	NC_018224	chromosome											
397774023	hypothetical protein NJ7G_2256	No	4			1963623	1964015	-		Natrinema_J7_uid171337	NC_018224	chromosome			arCOG09216	1		S		Uncharacterized conserved protein			
397774024	hypothetical protein NJ7G_2257	No	0			1964307	1964432	+		Natrinema_J7_uid171337	NC_018224	chromosome											
397774025	hypothetical protein NJ7G_2258	No	0			1964850	1964978	+		Natrinema_J7_uid171337	NC_018224	chromosome											
397774026	hypothetical protein NJ7G_2259	No	0			1965115	1965246	+		Natrinema_J7_uid171337	NC_018224	chromosome											
397774027	hypothetical protein NJ7G_2261	No	0			1965591	1965782	-		Natrinema_J7_uid171337	NC_018224	chromosome											
397774028	hypothetical protein NJ7G_4365	No	0			1965781	1965915	+		Natrinema_J7_uid171337	NC_018224	chromosome											
397774029	hypothetical protein NJ7G_2260	No	0			1966009	1966164	-		Natrinema_J7_uid171337	NC_018224	chromosome			arCOG08096	1	(-)	S		Zn finger containing protein			
===									6	Natrinema_J7_uid171337	NC_018224	chromosome											
397774032	Integrase catalytic region	No	0			1967171	1967554	-		Natrinema_J7_uid171337	NC_018224	chromosome			arCOG02134	15	(-)	L		Transposase			
397774033	hypothetical protein NJ7G_2265	No	0			1968005	1968451	+		Natrinema_J7_uid171337	NC_018224	chromosome											
397774034	hypothetical protein NJ7G_2266	No	0			1968519	1969073	+		Natrinema_J7_uid171337	NC_018224	chromosome											
397774035	hypothetical protein NJ7G_2267	No	0			1969115	1969459	+		Natrinema_J7_uid171337	NC_018224	chromosome											
397774036	hypothetical protein NJ7G_2268	No	0			1969478	1971478	+		Natrinema_J7_uid171337	NC_018224	chromosome											
397774037	hypothetical protein NJ7G_2269	Yes	10			1972047	1972937	+		Natrinema_J7_uid171337	NC_018224	chromosome			arCOG00271	3	(-)	G	RhaT	Permease of the drug/metabolite transporter (DM	6E-25	COG0697	Permeases of the drug/metabolite transpo
===									23	Natrinema_J7_uid171337	NC_018224	chromosome											
397774112	hypothetical protein NJ7G_2348	No	0			2033115	2033732	+		Natrinema_J7_uid171337	NC_018224	chromosome			arCOG08149	2	(-)	S		Uncharacterized conserved protein			
397774113	hypothetical protein NJ7G_2349	No	0			2033998	2034114	-		Natrinema_J7_uid171337	NC_018224	chromosome											
397774114	hypothetical protein NJ7G_2350	No	0			2034301	2034624	+		Natrinema_J7_uid171337	NC_018224	chromosome	clust_324										
397774115	hypothetical protein NJ7G_2352	No	0			2034904	2035380	+		Natrinema_J7_uid171337	NC_018224	chromosome			arCOG09042	7		Y		Viral protein, VP1 family			
397774116	hypothetical protein NJ7G_2353	No	1			2035385	2036032	+		Natrinema_J7_uid171337	NC_018224	chromosome											
397774117	hypothetical protein NJ7G_2354	No	0			2036029	2036574	+		Natrinema_J7_uid171337	NC_018224	chromosome											
397774118	hypothetical protein NJ7G_2355	No	0			2036571	2037149	+		Natrinema_J7_uid171337	NC_018224	chromosome			arCOG06228	5		S		Uncharacterized conserved protein			
397774119	hypothetical protein NJ7G_2356	No	0			2037146	2037934	+		Natrinema_J7_uid171337	NC_018224	chromosome			arCOG00280	21	(-)	L		HerA helicase			
397774120	hypothetical protein NJ7G_2357	No	0			2037941	2038120	+		Natrinema_J7_uid171337	NC_018224	chromosome	clust_475										
397774121	hypothetical protein NJ7G_2358	No	0			2038223	2038498	+		Natrinema_J7_uid171337	NC_018224	chromosome	clust_51										
397774122	hypothetical protein NJ7G_2359	No	0			2038499	2038990	+		Natrinema_J7_uid171337	NC_018224	chromosome			arCOG09038	6		Y		VP7/VP16-like Major capsid protein			
397774123	hypothetical protein NJ7G_2360	No	0			2038990	2039727	+		Natrinema_J7_uid171337	NC_018224	chromosome			arCOG09037	5		Y		VP4/VP17-like Major capsid protein			
397774124	hypothetical protein NJ7G_2361	No	1			2039734	2039976	+		Natrinema_J7_uid171337	NC_018224	chromosome	clust_403										
397774125	putative phage-related minor tail protein	No	1			2040138	2040806	+		Natrinema_J7_uid171337	NC_018224	chromosome									0.00000006	COG2931	RTX toxins and related Ca2+-binding prot
397774126	hypothetical protein NJ7G_2363	No	1			2040808	2041026	+		Natrinema_J7_uid171337	NC_018224	chromosome	clust_433										
397774127	hypothetical protein NJ7G_2364	No	0			2041023	2041499	+		Natrinema_J7_uid171337	NC_018224	chromosome	clust_18										
397774128	hypothetical protein NJ7G_2365	No	0			2041496	2042491	+		Natrinema_J7_uid171337	NC_018224	chromosome											
397774129	hypothetical protein NJ7G_2366	No	0			2042478	2042717	-		Natrinema_J7_uid171337	NC_018224	chromosome											
397774130	hypothetical protein NJ7G_4370	No	0			2042803	2043189	+		Natrinema_J7_uid171337	NC_018224	chromosome											
397774131	hypothetical protein NJ7G_2367	No	0			2043215	2043676	-		Natrinema_J7_uid171337	NC_018224	chromosome			arCOG09043	4		S		Uncharacterized conserved protein			
397774132	hypothetical protein NJ7G_2368	No	0			2043798	2044238	-		Natrinema_J7_uid171337	NC_018224	chromosome			arCOG09044	5	(-)	R		SWIM zinc finger			
397774133	hypothetical protein NJ7G_2369	No	0			2044226	2044342	-		Natrinema_J7_uid171337	NC_018224	chromosome											
397774134	hypothetical protein NJ7G_2370	No	0			2044488	2044661	-		Natrinema_J7_uid171337	NC_018224	chromosome											
===									32	Natrinema_J7_uid171337	NC_018224	chromosome											
397774141	hypothetical protein NJ7G_2378	No	0			2054845	2059236	+		Natrinema_J7_uid171337	NC_018224	chromosome	clust_10										
397774142	HEAT repeat protein	No	0			2059324	2061294	+		Natrinema_J7_uid171337	NC_018224	chromosome			arCOG02966	7	(-)	S		HEAT repeats containing protein	9E-18	COG1413	FOG: HEAT repeat
397774143	HEAT repeat protein	No	0			2061269	2061628	+		Natrinema_J7_uid171337	NC_018224	chromosome											
397774144	hypothetical protein NJ7G_2381	No	0			2061644	2062126	-		Natrinema_J7_uid171337	NC_018224	chromosome									2E-11	pfam09346	SMI1 / KNR4 family (SUKH-1). Proteins in
397774145	hypothetical protein NJ7G_2382	No	0			2062229	2063056	-		Natrinema_J7_uid171337	NC_018224	chromosome									5E-20	pfam10020	Uncharacterized protein conserved in bac
397774146	hypothetical protein NJ7G_2383	No	0			2063238	2063399	+		Natrinema_J7_uid171337	NC_018224	chromosome											
397774147	hypothetical protein NJ7G_2384	No	0			2063449	2063673	-		Natrinema_J7_uid171337	NC_018224	chromosome											
397774148	hypothetical protein NJ7G_2385	No	0			2063688	2064143	+		Natrinema_J7_uid171337	NC_018224	chromosome			arCOG02966	7	(-)	S		HEAT repeats containing protein			
397774149	hypothetical protein NJ7G_2386	No	0			2064145	2064426	-		Natrinema_J7_uid171337	NC_018224	chromosome									0.0000002	pfam05076	Suppressor of fused protein (SUFU). SUFU
397774150	hypothetical protein NJ7G_2387	No	0			2064499	2064711	-		Natrinema_J7_uid171337	NC_018224	chromosome											
397774151	hypothetical protein NJ7G_2388	No	0			2064827	2066014	+		Natrinema_J7_uid171337	NC_018224	chromosome			arCOG02559	3	(-)	S		Cell suface protein			
397774152	hypothetical protein NJ7G_2389	No	0			2066041	2066436	-		Natrinema_J7_uid171337	NC_018224	chromosome	clust_271										
397774153	hypothetical protein NJ7G_2390	No	0			2066450	2066914	-		Natrinema_J7_uid171337	NC_018224	chromosome									6E-19	pfam10020	Uncharacterized protein conserved in bac
397774154	hypothetical protein NJ7G_2391	No	0			2067060	2067428	-		Natrinema_J7_uid171337	NC_018224	chromosome	clust_289										
397774155	hypothetical protein NJ7G_2392	No	0			2067471	2067620	-		Natrinema_J7_uid171337	NC_018224	chromosome											
397774156	hypothetical protein NJ7G_2393	No	0			2067621	2067911	+		Natrinema_J7_uid171337	NC_018224	chromosome											
397774157	hypothetical protein NJ7G_2394	No	0			2068180	2068890	-		Natrinema_J7_uid171337	NC_018224	chromosome			arCOG02966	7	(-)	S		HEAT repeats containing protein			
397774158	hypothetical protein NJ7G_2395	No	0			2068868	2069236	-		Natrinema_J7_uid171337	NC_018224	chromosome											
397774159	hypothetical protein NJ7G_2396	No	0			2069311	2069460	-		Natrinema_J7_uid171337	NC_018224	chromosome											
397774160	hypothetical protein NJ7G_2397	No	0			2069889	2071481	-		Natrinema_J7_uid171337	NC_018224	chromosome			arCOG02967	3	(-)	T		NACHT family NTPase fused to HEAT repeats do	0.000008	COG1413	FOG: HEAT repeat
397774161	hypothetical protein NJ7G_2398	No	0			2071478	2071744	-		Natrinema_J7_uid171337	NC_018224	chromosome											
397774162	hypothetical protein NJ7G_2399	No	0			2071834	2072202	-		Natrinema_J7_uid171337	NC_018224	chromosome	clust_289										
397774163	hypothetical protein NJ7G_2400	No	0			2072329	2072592	+		Natrinema_J7_uid171337	NC_018224	chromosome	clust_22										
397774164	hypothetical protein NJ7G_2401	No	0			2072610	2072927	-		Natrinema_J7_uid171337	NC_018224	chromosome	clust_336										
397774165	hypothetical protein NJ7G_2402	No	0			2072949	2074427	-		Natrinema_J7_uid171337	NC_018224	chromosome											
397774166	hypothetical protein NJ7G_2403	No	0			2074670	2077780	+		Natrinema_J7_uid171337	NC_018224	chromosome			arCOG03042	2	(-)	R	NrfG	TPR repeats containing protein			
397774167	KNR4-like cell wall assembly/cell proliferation (	No	0			2077907	2078428	-		Natrinema_J7_uid171337	NC_018224	chromosome									8E-19	COG4282	Protein involved in beta-1,3-glucan synt
397774168	hypothetical protein NJ7G_2405	No	0			2078600	2079085	+		Natrinema_J7_uid171337	NC_018224	chromosome											
397774169	hypothetical protein NJ7G_2406	No	0			2079150	2079992	-		Natrinema_J7_uid171337	NC_018224	chromosome	clust_105								0.0000004	pfam14423	Immunity protein Imm5. A predicted Immun
397774170	hypothetical protein NJ7G_2407	No	0			2079952	2080368	+		Natrinema_J7_uid171337	NC_018224	chromosome											
397774171	hypothetical protein NJ7G_2408	No	0			2080453	2080857	-		Natrinema_J7_uid171337	NC_018224	chromosome	clust_40										
397774172	Homoserine dehydrogenase	No	0			2081018	2081965	-		Natrinema_J7_uid171337	NC_018224	chromosome			arCOG01351	1	(-)	E	ThrA	Homoserine dehydrogenase	8E-58	COG0460	Homoserine dehydrogenase
===									5	Natrinema_J7_uid171337	NC_018224	chromosome											
397774204	hypothetical protein NJ7G_2441	No	0			2106071	2106460	+		Natrinema_J7_uid171337	NC_018224	chromosome											
397774205	hypothetical protein NJ7G_2443	No	0			2106520	2106639	+		Natrinema_J7_uid171337	NC_018224	chromosome											
397774206	CBS domain containing protein	No	0			2106603	2107007	-		Natrinema_J7_uid171337	NC_018224	chromosome			arCOG00606	4	(-)	R		CBS domain	3E-17	COG0517	FOG: CBS domain
397774207	hypothetical protein NJ7G_2444	No	0			2107612	2107818	-		Natrinema_J7_uid171337	NC_018224	chromosome											
397774208	hypothetical protein NJ7G_2445	No	0			2107808	2107993	+		Natrinema_J7_uid171337	NC_018224	chromosome											
===									6	Natrinema_J7_uid171337	NC_018224	chromosome											
397774445	hypothetical protein NJ7G_2686	No	0			2320548	2320814	-		Natrinema_J7_uid171337	NC_018224	chromosome			arCOG03924	10	(-)	K	MarR	Transcriptional regulator, MarR family			
397774446	hypothetical protein NJ7G_2687	No	0			2320949	2321095	-		Natrinema_J7_uid171337	NC_018224	chromosome											
397774447	hypothetical protein NJ7G_2688	No	0			2321363	2321527	+		Natrinema_J7_uid171337	NC_018224	chromosome											
397774448	hypothetical protein NJ7G_2689	No	0			2321630	2321743	+		Natrinema_J7_uid171337	NC_018224	chromosome											
397774449	hypothetical protein NJ7G_2690	No	0			2321907	2322023	-		Natrinema_J7_uid171337	NC_018224	chromosome											
397774450	nicotinamidase-like amidase	No	0			2322238	2322699	-		Natrinema_J7_uid171337	NC_018224	chromosome			arCOG01943	2	(-)	Q	PncA	Amidase related to nicotinamidase	1E-13	pfam00857	Isochorismatase family. This family are
===									6	Natrinema_J7_uid171337	NC_018224	chromosome											
397774495	hypothetical protein NJ7G_2737	No	0			2366348	2366464	-		Natrinema_J7_uid171337	NC_018224	chromosome											
397774496	hypothetical protein NJ7G_2738	No	0			2366971	2367126	-		Natrinema_J7_uid171337	NC_018224	chromosome											
397774497	hypothetical protein NJ7G_2739	Yes	2			2367251	2367466	-		Natrinema_J7_uid171337	NC_018224	chromosome											
397774498	aldo/keto reductase	No	0			2367495	2368646	+		Natrinema_J7_uid171337	NC_018224	chromosome			arCOG01617	4	(-)	C	Tas	Predicted oxidoreductase (related to aryl-alcohol	4E-42	COG0667	Predicted oxidoreductases (related to ar
397774499	hypothetical protein NJ7G_2741	No	0			2368681	2368929	-		Natrinema_J7_uid171337	NC_018224	chromosome											
397774500	hypothetical protein NJ7G_2742	No	0			2369073	2369234	+		Natrinema_J7_uid171337	NC_018224	chromosome											
===									9	Natrinema_J7_uid171337	NC_018224	chromosome											
397774614	glutamate/valine-rich protein	No	2			2474415	2475194	-		Natrinema_J7_uid171337	NC_018224	chromosome		arCOG07580,a	arCOG07580	1	(-)	S		Uncharacterized conserved protein	0.0000004	pfam06513	Repeat of unknown function (DUF1103). Th
397774615	hypothetical protein NJ7G_2857	No	0			2475248	2475364	+		Natrinema_J7_uid171337	NC_018224	chromosome											
397774616	hypothetical protein NJ7G_2858	No	0			2475632	2475994	-		Natrinema_J7_uid171337	NC_018224	chromosome	clust_45										
397774617	hypothetical protein NJ7G_2859	No	0			2476039	2477040	-		Natrinema_J7_uid171337	NC_018224	chromosome			arCOG08992	1		S		Uncharacterized conserved protein			
397774618	hypothetical protein NJ7G_2860	No	0			2477545	2477922	+		Natrinema_J7_uid171337	NC_018224	chromosome											

397774619	hypothetical protein NJ7G_2861	No	0		2478890	2479414	+		Natrinema_J7_uid171337	NC_018224	chromosome										
397774620	hypothetical protein NJ7G_2862	No	0		2479998	2480273	-		Natrinema_J7_uid171337	NC_018224	chromosome										
397774621	hypothetical protein NJ7G_2863	No	0		2480494	2480856	+		Natrinema_J7_uid171337	NC_018224	chromosome										
397774622	Thioredoxin domain-containing protein	No	0		2480953	2481222	+		Natrinema_J7_uid171337	NC_018224	chromosome			arCOG01972	5 (-)	O	TrxA	Thiol-disulfide isomerase or thioredoxin	1E-15	COG0526	Thiol-disulfide isomerase and thioredoxi
===								5	Natrinema_J7_uid171337	NC_018224	chromosome										
397774682	hypothetical protein NJ7G_2924	No	0		2537192	2537389	+		Natrinema_J7_uid171337	NC_018224	chromosome										
397774683	zinc finger SWIM domain protein	No	0		2538069	2538593	+		Natrinema_J7_uid171337	NC_018224	chromosome			arCOG03429	2 (-)	R		SWIM zinc finger containing protein			
397774684	hypothetical protein NJ7G_2926	No	0		2538590	2538730	+		Natrinema_J7_uid171337	NC_018224	chromosome										
397774685	hypothetical protein NJ7G_2933	No	0		2544619	2544777	-		Natrinema_J7_uid171337	NC_018224	chromosome										
397774686	hypothetical protein NJ7G_2934	No	0		2545352	2545525	-		Natrinema_J7_uid171337	NC_018224	chromosome										
===								33	Natrinema_J7_uid171337	NC_018224	chromosome										
397774936	3-phosphoshikimate 1-carboxyvinyltransferase	No	0		2760947	2762305	+		Natrinema_J7_uid171337	NC_018224	chromosome			arCOG04134	1 (-)	E	AroA	5-enolpyruvylshikimate-3-phosphate synthase	1E-110	COG0128	5-enolpyruvylshikimate-3-phosphate synth
397774937	hypothetical protein NJ7G_3187	No	6		2762729	2763574	+		Natrinema_J7_uid171337	NC_018224	chromosome										
397774938	hypothetical protein NJ7G_3188	No	0		2764504	2764836	+		Natrinema_J7_uid171337	NC_018224	chromosome			arCOG10932	3	S		Uncharacterized conserved protein			
397774939	hypothetical protein NJ7G_3189	No	0		2764870	2765025	+		Natrinema_J7_uid171337	NC_018224	chromosome										
397774940	hypothetical protein NJ7G_3190	No	0		2765391	2765783	-		Natrinema_J7_uid171337	NC_018224	chromosome			arCOG09353	1	S		Uncharacterized conserved protein			
397774941	integrase family protein	No	0		2766738	2767553	+		Natrinema_J7_uid171337	NC_018224	chromosome			arCOG01250	7 (-)	L	XerC	Integrase			
397774942	hypothetical protein NJ7G_3192	No	0		2767857	2768288	+		Natrinema_J7_uid171337	NC_018224	chromosome			arCOG09044	5 (-)	R		SWIM zinc finger			
397774943	hypothetical protein NJ7G_3193	No	0		2768410	2768871	+		Natrinema_J7_uid171337	NC_018224	chromosome			arCOG09043	4	S		Uncharacterized conserved protein			
397774944	hypothetical protein NJ7G_3194	No	0		2768907	2769587	-		Natrinema_J7_uid171337	NC_018224	chromosome										
397774945	hypothetical protein NJ7G_3195	No	0		2769590	2769733	-		Natrinema_J7_uid171337	NC_018224	chromosome										
397774946	hypothetical protein NJ7G_3196	No	0		2769827	2770096	-		Natrinema_J7_uid171337	NC_018224	chromosome										
397774947	hypothetical protein NJ7G_3197	No	0		2770259	2770582	-		Natrinema_J7_uid171337	NC_018224	chromosome										
397774948	hypothetical protein NJ7G_3198	No	0		2770579	2771055	-		Natrinema_J7_uid171337	NC_018224	chromosome	clust_18									
397774949	hypothetical protein NJ7G_3199	No	1		2771055	2771270	-		Natrinema_J7_uid171337	NC_018224	chromosome	clust_433									
397774950	hypothetical protein NJ7G_3200	No	0		2771272	2771487	-		Natrinema_J7_uid171337	NC_018224	chromosome										
397774951	hypothetical protein NJ7G_3201	No	1		2771468	2771956	-		Natrinema_J7_uid171337	NC_018224	chromosome								0.00004	pfam07020	Orthopoxvirus C10L protein. This family
397774952	hypothetical protein NJ7G_3202	No	1		2772097	2772339	-		Natrinema_J7_uid171337	NC_018224	chromosome	clust_403									
397774953	hypothetical protein NJ7G_3203	No	0		2772346	2773083	-		Natrinema_J7_uid171337	NC_018224	chromosome			arCOG09037	5	Y		VP4/VP17-like Major capsid protein			
397774954	hypothetical protein NJ7G_3204	No	0		2773083	2773574	-		Natrinema_J7_uid171337	NC_018224	chromosome			arCOG09038	6	Y		VP7/VP16-like Major capsid protein			
397774955	hypothetical protein NJ7G_3205	No	0		2773575	2773850	-		Natrinema_J7_uid171337	NC_018224	chromosome	clust_51									
397774956	filamentation induced by cAMP protein Fic	No	0		2774076	2775233	+		Natrinema_J7_uid171337	NC_018224	chromosome			arCOG03110	5 (-)	K		Predicted transciptional regulator, contains Fic ar	2E-39	COG3177	Fic family protein
397774957	hypothetical protein NJ7G_3207	No	0		2775252	2775434	-		Natrinema_J7_uid171337	NC_018224	chromosome	clust_475									
397774958	hypothetical protein NJ7G_3208	No	0		2775441	2776229	-		Natrinema_J7_uid171337	NC_018224	chromosome			arCOG00280	21 (-)	L		HerA helicase			
397774959	hypothetical protein NJ7G_3209	No	0		2776226	2776804	-		Natrinema_J7_uid171337	NC_018224	chromosome			arCOG06228	5	S		Uncharacterized conserved protein			
397774960	hypothetical protein NJ7G_3210	No	1		2776801	2776971	-		Natrinema_J7_uid171337	NC_018224	chromosome										
397774961	hypothetical protein NJ7G_3211	No	0		2776990	2777352	-		Natrinema_J7_uid171337	NC_018224	chromosome										
397774962	hypothetical protein NJ7G_3212	No	1		2777336	2777995	-		Natrinema_J7_uid171337	NC_018224	chromosome			arCOG09041	4	S		Uncharacterized conserved protein	0.00006	pfam07263	Dentin matrix protein 1 (DMP1). This fam
397774963	hypothetical protein NJ7G_3213	No	0		2778000	2778554	-		Natrinema_J7_uid171337	NC_018224	chromosome			arCOG09042	7	Y		Viral protein, VP1 family			
397774964	hypothetical protein NJ7G_3214	No	0		2778554	2778877	-		Natrinema_J7_uid171337	NC_018224	chromosome	clust_324									
397774965	hypothetical protein NJ7G_3216	No	0		2778983	2779180	+		Natrinema_J7_uid171337	NC_018224	chromosome			arCOG07539	2 (-)	S		Uncharacterized conserved protein			
397774966	hypothetical protein NJ7G_3215	No	0		2779139	2779264	-		Natrinema_J7_uid171337	NC_018224	chromosome										
397774967	hypothetical protein NJ7G_3217	Yes	0		2779639	2780610	+		Natrinema_J7_uid171337	NC_018224	chromosome										
397774968	hypothetical protein NJ7G_3218	No	0		2780733	2780900	+		Natrinema_J7_uid171337	NC_018224	chromosome										
===								8	Natrinema_J7_uid171337	NC_018224	chromosome										
397775064	transposase	No	0		2860368	2860916	+		Natrinema_J7_uid171337	NC_018224	chromosome			arCOG02134	15 (-)	L		Transposase	0.000000005	COG3316	Transposase and inactivated derivatives
397775065	hypothetical protein NJ7G_3317	No	0		2861182	2861424	+		Natrinema_J7_uid171337	NC_018224	chromosome										
397775066	hypothetical protein NJ7G_3318	No	0		2861421	2861555	+		Natrinema_J7_uid171337	NC_018224	chromosome										
397775067	hypothetical protein NJ7G_3319	No	0		2861552	2862037	+		Natrinema_J7_uid171337	NC_018224	chromosome										
397775068	hypothetical protein NJ7G_3320	No	0		2862000	2862281	+		Natrinema_J7_uid171337	NC_018224	chromosome										
397775069	hypothetical protein NJ7G_3321	No	0		2862284	2862475	+		Natrinema_J7_uid171337	NC_018224	chromosome										
397775070	hypothetical protein NJ7G_3322	Yes	0		2863288	2863632	-		Natrinema_J7_uid171337	NC_018224	chromosome	clust_309									
397775071	HEAT repeat protein	No	0		2863878	2864855	+		Natrinema_J7_uid171337	NC_018224	chromosome			arCOG01917	12 (-)	S		Zn-ribbon domain containing protein			
===								5	Natrinema_J7_uid171337	NC_018224	chromosome										
397775073	hypothetical protein NJ7G_3324	No	0		2866075	2866191	-		Natrinema_J7_uid171337	NC_018224	chromosome										
397775074	hypothetical protein NJ7G_3326	No	0		2866278	2867174	+		Natrinema_J7_uid171337	NC_018224	chromosome			arCOG07538	2 (-)	S		Uncharacterized conserved protein			
397775075	hypothetical protein NJ7G_3327	No	0		2867919	2868044	-		Natrinema_J7_uid171337	NC_018224	chromosome										
397775076	hypothetical protein NJ7G_3328	No	1		2868326	2868496	-		Natrinema_J7_uid171337	NC_018224	chromosome										
397775077	hypothetical protein NJ7G_3329	No	0		2868736	2868858	+		Natrinema_J7_uid171337	NC_018224	chromosome										
===								5	Natrinema_J7_uid171337	NC_018224	chromosome										
397775447	hypothetical protein NJ7G_3700	No	0		3206868	3207170	-		Natrinema_J7_uid171337	NC_018224	chromosome										
397775448	hypothetical protein NJ7G_3702	No	0		3207222	3207341	+		Natrinema_J7_uid171337	NC_018224	chromosome										
397775449	glucosamine/fructose-6-phosphate aminotransf	No	0		3207377	3209185	-		Natrinema_J7_uid171337	NC_018224	chromosome			arCOG00057	3 (-)	M	GlmS	Glucosamine 6-phosphate synthetase	4E-164	COG0449	Glucosamine 6-phosphate synthetase, cont
397775450	hypothetical protein NJ7G_3704	No	0		3209185	3209328	-		Natrinema_J7_uid171337	NC_018224	chromosome										
397775451	hypothetical protein NJ7G_3705	No	0		3209491	3209697	+		Natrinema_J7_uid171337	NC_018224	chromosome										
===								5	Natrinema_J7_uid171337	NC_018224	chromosome										
397775478	integrase family protein	No	0		3236404	3236646	+		Natrinema_J7_uid171337	NC_018224	chromosome										
397775479	hypothetical protein NJ7G_3732	No	0		3236628	3236753	-		Natrinema_J7_uid171337	NC_018224	chromosome										
397775480	hypothetical protein NJ7G_3734	No	3		3237055	3237384	+		Natrinema_J7_uid171337	NC_018224	chromosome										
397775481	regulatory protein	No	0		3237387	3237926	+		Natrinema_J7_uid171337	NC_018224	chromosome			arCOG02611	4 (-)	K		Predicted transcriptional regulator, containd two H	2E-15	COG3398	Uncharacterized protein conserved in arc
397775482	hypothetical protein NJ7G_3735	No	5		3237923	3238471	-		Natrinema_J7_uid171337	NC_018224	chromosome										
===								6	Natrinema_J7_uid171337	NC_018224	chromosome										
397775519	thiamine biosynthesis protein ThiC	No	0		3269626	3271071	+		Natrinema_J7_uid171337	NC_018224	chromosome			arCOG02741	4 (-)	H	ThiC	Thiamine biosynthesis protein ThiC		COG0422	Thiamine biosynthesis protein ThiC
397775520	hypothetical protein NJ7G_3773	No	0		3271202	3271357	-		Natrinema_J7_uid171337	NC_018224	chromosome										
397775521	hypothetical protein NJ7G_3774	No	2		3271415	3271654	-		Natrinema_J7_uid171337	NC_018224	chromosome										
397775522	hypothetical protein NJ7G_3775	No	0		3272018	3272929	-		Natrinema_J7_uid171337	NC_018224	chromosome										
397775523	hypothetical protein NJ7G_3776	No	0		3273248	3273478	+		Natrinema_J7_uid171337	NC_018224	chromosome										
397775524	hypothetical protein NJ7G_3777	No	0		3273632	3274960	+		Natrinema_J7_uid171337	NC_018224	chromosome			arCOG01661	1 (-)	R		alpha/beta superfamily hydrolase			
===								9	Natrinema_J7_uid171337	NC_018224	chromosome										
397775653	hypothetical protein NJ7G_3907	No	0		3392677	3393081	+		Natrinema_J7_uid171337	NC_018224	chromosome			arCOG03097	4 (-)	S		Uncharacterized conserved protein			
397775654	hypothetical protein NJ7G_3906	No	0		3393053	3393169	-		Natrinema_J7_uid171337	NC_018224	chromosome										
397775655	hypothetical protein NJ7G_3908	No	0		3393221	3393412	-		Natrinema_J7_uid171337	NC_018224	chromosome										
397775656	hypothetical protein NJ7G_3909	No	0		3393479	3393781	-		Natrinema_J7_uid171337	NC_018224	chromosome										
397775657	hypothetical protein NJ7G_3910	No	0		3393774	3393905	-		Natrinema_J7_uid171337	NC_018224	chromosome										
397775658	hypothetical protein NJ7G_3911	No	0		3393996	3394340	+		Natrinema_J7_uid171337	NC_018224	chromosome			arCOG07773	5 (-)	S		SWIM zinc finger containing protein			
397775659	hypothetical protein NJ7G_3912	No	0		3394328	3394570	+		Natrinema_J7_uid171337	NC_018224	chromosome	clust_56									
397775660	hypothetical protein NJ7G_3913	No	0		3394645	3394887	-		Natrinema_J7_uid171337	NC_018224	chromosome										
397775661	hypothetical protein NJ7G_3914	No	0		3394884	3395114	-		Natrinema_J7_uid171337	NC_018224	chromosome										
===								7	Natrinema_J7_uid171337	NC_018224	chromosome										
397775692	hypothetical protein NJ7G_3948	No	0		3423730	3423951	-		Natrinema_J7_uid171337	NC_018224	chromosome										
397775693	SAICAR synthetase	No	0		3423966	3424985	+		Natrinema_J7_uid171337	NC_018224	chromosome			arCOG04421	1 (-)	F	PurC	Phosphoribosylaminoimidazolesuccinocarboxam	3E-10	COG0152	Phosphoribosylaminoimidazolesuccinocarbo
397775694	hypothetical protein NJ7G_3950	No	1		3425115	3425321	+		Natrinema_J7_uid171337	NC_018224	chromosome										
397775695	hypothetical protein NJ7G_3951	No	0		3425380	3425499	+		Natrinema_J7_uid171337	NC_018224	chromosome										
397775696	hypothetical protein NJ7G_3952	No	0		3425574	3425807	-		Natrinema_J7_uid171337	NC_018224	chromosome										
397775697	hypothetical protein NJ7G_3953	No	0		3425848	3425979	-		Natrinema_J7_uid171337	NC_018224	chromosome										
397775698	hypothetical protein NJ7G_3954	No	0		3426019	3426189	+		Natrinema_J7_uid171337	NC_018224	chromosome			arCOG06252	1 (-)	S		Uncharacterized conserved protein			
===								7	Natrinema_J7_uid171337	NC_018224	chromosome										
397775717	hypothetical protein NJ7G_3973	No	0		3445428	3445850	-		Natrinema_J7_uid171337	NC_018224	chromosome										
397775718	Glyoxalase/bleomycin resistance protein/dioxy	No	0		3446005	3446286	-		Natrinema_J7_uid171337	NC_018224	chromosome			arCOG06106	1 (-)	R		Predicted ring-cleavage extradiol dioxygenase	0.000008	COG2514	Predicted ring-cleavage extradiol dioxyg
397775719	hypothetical protein NJ7G_3975	No	0		3446323	3446532	-		Natrinema_J7_uid171337	NC_018224	chromosome										
397775720	hypothetical protein NJ7G_3976	No	0		3446638	3446796	+		Natrinema_J7_uid171337	NC_018224	chromosome										
397775721	transposase family protein	No	0		3447461	3447592	-		Natrinema_J7_uid171337	NC_018224	chromosome										
397775722	transposase	No	0		3447783	3448460	+		Natrinema_J7_uid171337	NC_018224	chromosome			arCOG02134	15 (-)	L		Transposase	2E-16	COG3316	Transposase and inactivated derivatives
397775723	hypothetical protein NJ7G_3979	No	0		3448585	3448704	+		Natrinema_J7_uid171337	NC_018224	chromosome										
===								5	Natrinema_J7_uid171337	NC_018224	chromosome										
397775726	hypothetical protein NJ7G_3982	No	0		3450949	3451299	+		Natrinema_J7_uid171337	NC_018224	chromosome										
397775727	hypothetical protein NJ7G_3983	No	0		3451392	3451967	-		Natrinema_J7_uid171337	NC_018224	chromosome										
397775728	hypothetical protein NJ7G_3984	No	0		3451964	3452116	-		Natrinema_J7_uid171337	NC_018224	chromosome										
397775729	hypothetical protein NJ7G_3985	No	0		3452372	3452536	+		Natrinema_J7_uid171337	NC_018224	chromosome			arCOG08128	7 (-)	S		Uncharacterized conserved protein			
397775730	hypothetical protein NJ7G_3986	No	0		3452566	3452694	+		Natrinema_J7_uid171337	NC_018224	chromosome										
===								5	Natrinema_J7_uid171337	NC_018224	chromosome										
397776002	hypothetical protein NJ7G_4257	No	0		3671351	3671497	+		Natrinema_J7_uid171337	NC_018224	chromosome										
397776003	hypothetical protein NJ7G_4258	No	0		3671863	3672372	+		Natrinema_J7_uid171337	NC_018224	chromosome										
397776004	beta-lactamase	No	0		3672383	3673861	-		Natrinema_J7_uid171337	NC_018224	chromosome			arCOG03009	1 (-)	R	ComEC	Metal-dependent hydrolase of the beta-lactamase	5E-14	COG2333	Predicted hydrolase (metallo-beta-lactam
397776005	hypothetical protein NJ7G_4260	No	0		3673858	3674175	-		Natrinema_J7_uid171337	NC_018224	chromosome										
397776006	hypothetical protein NJ7G_4261	No	1		3674492	3674629	-		Natrinema_J7_uid171337	NC_018224	chromosome										
===								6	Natrinema_pellirubrum_DSM_15624_uid74437	NC_019962	chromosome										
433589840	hypothetical protein Natpe_0502	No	0		497368	497775	+		Natrinema_pellirubrum_DSM_15624_uid74437	NC_019962	chromosome										
433589841	hypothetical protein Natpe_0503	No	0		498143	498427	+		Natrinema_pellirubrum_DSM_15624_uid74437	NC_019962	chromosome										
433589842	hypothetical protein Natpe_0504	No	0		498388	499002	+		Natrinema_pellirubrum_DSM_15624_uid74437	NC_019962	chromosome										
433589843	transposase family protein	No	0		499517	500851	-		Natrinema_pellirubrum_DSM_15624_uid74437	NC_019962	chromosome			arCOG06159	1 (-)	L		Transposase	3E-10	COG3385	FOG: Transposase and inactivated derivat
433589844	hypothetical protein Natpe_0506	No	2		500922	501182	+		Natrinema_pellirubrum_DSM_15624_uid74437	NC_019962	chromosome	clust_389									
433589845	hypothetical protein Natpe_0508	No	2		502682	502909	+		Natrinema_pellirubrum_DSM_15624_uid74437	NC_019962	chromosome										
===								6	Natrinema_pellirubrum_DSM_15624_uid74437	NC_019962	chromosome										
433590122	methylase involved in ubiquinone/menaquinon	No	0		786092	786895	+		Natrinema_pellirubrum_DSM_15624_uid74437	NC_019962	chromosome			arCOG01783	1 (-)	Q		SAM-dependent methyltransferase	7E-18	pfam08241	Methyltransferase domain. Members of thi
433590123	hypothetical protein Natpe_0796	No	3		787027	787488	-		Natrinema_pellirubrum_DSM_15624_uid74437	NC_019962	chromosome	clust_228									
433590124	hypothetical protein Natpe_0797	Yes	1		787485	788429	-		Natrinema_pellirubrum_DSM_15624_uid74437	NC_019962	chromosome								0.000002	pfam07263	Dentin matrix protein 1 (DMP1). This fam
433590125	hypothetical protein Natpe_0798	No	3		788467	789054	-		Natrinema_pellirubrum_DSM_15624_uid74437	NC_019962	chromosome	clust_176									
433590126	hypothetical protein Natpe_0799	Yes	1		789061	789672	-		Natrinema_pellirubrum_DSM_15624_uid74437	NC_019962	chromosome	clust_171									
433590127	putative DNA binding protein	No	0		790208	790858	+		Natrinema_pellirubrum_DSM_15624_uid74437	NC_019962	chromosome			arCOG02274	3 (-)	K		Predicted transcriptional regulator, C-terminal HTH	6E-11	pfam04967	HTH DNA binding domain.
===								5	Natrinema_pellirubrum_DSM_15624_uid74437	NC_019962	chromosome										
433591094	hypothetical protein Natpe_1804	No	1		1777377	1777481	+		Natrinema_pellirubrum_DSM_15624_uid74437	NC_019962	chromosome										
433591095	hypothetical protein Natpe_1806	No	0		1779411	1779725	-		Natrinema_pellirubrum_DSM_15624_uid74437	NC_019962	chromosome										
433591096	hypothetical protein Natpe_1807	No	0		1779923	1780255	+		Natrinema_pellirubrum_DSM_15624_uid74437	NC_019962	chromosome										
433591097	hypothetical protein Natpe_1809	No	0		1781982	1782200	+		Natrinema_pellirubrum_DSM_15624_uid74437	NC_019962	chromosome			arCOG09008	1 (-)	S		Uncharacterized conserved protein			
433591098	hypothetical protein Natpe_1810	No	0		1782449	1782661	+		Natrinema_pellirubrum_DSM_15624_uid74437	NC_019962	chromosome										
===								12	Natrinema_pellirubrum_DSM_15624_uid74437	NC_019962	chromosome										
433591107	site-specific recombinase XerD	No	0		1794347	1795357	+		Natrinema_pellirubrum_DSM_15624_uid74437	NC_019962	chromosome			arCOG01250	7 (-)	L	XerC	Integrase	8E-11	COG4974	Site-specific recombinase XerD
433591108	hypothetical protein Natpe_1824	No	0		1795441	1796037	+		Natrinema_pellirubrum_DSM_15624_uid74437	NC_019962	chromosome										
433591109	hypothetical protein Natpe_1825	No	0		1796055	1796207	-		Natrinema_pellirubrum_DSM_15624_uid74437	NC_019962	chromosome										
433591110	hypothetical protein Natpe_1826	No	0		1796500	1796649	-		Natrinema_pellirubrum_DSM_15624_uid74437	NC_019962	chromosome										
433591111	hypothetical protein Natpe_1827	No	1		1796732	1796914	+		Natrinema_pellirubrum_DSM_15624_uid74437	NC_019962	chromosome										
433591112	hypothetical protein Natpe_1828	No	0		1797056	1798264	-		Natrinema_pellirubrum_DSM_15624_uid74437	NC_019962	chromosome										
433591113	site-specific recombinase XerD	No	0		1798416	1799534	+		Natrinema_pellirubrum_DSM_15624_uid74437	NC_019962	chromosome			arCOG01250	7 (-)	L	XerC	Integrase	0.00004	COG4974	Site-specific recombinase XerD
433591114	hypothetical protein Natpe_1830	No	0		1799781	1800338	+		Natrinema_pellirubrum_DSM_15624_uid74437	NC_019962	chromosome										
433591115	hypothetical protein Natpe_1831	No	0		1800331	1801299	+		Natrinema_pellirubrum_DSM_15624_uid74437	NC_019962	chromosome										
433591116	hypothetical protein Natpe_1832	No	0		1801302	1802603	+		Natrinema_pellirubrum_DSM_15624_uid74437	NC_019962	chromosome			arCOG06233	1 (-)	L		Topoisomerase IA associated Zn-finger domain			
433591117	hypothetical protein Natpe_1833	No	0		1803287	1803391	+		Natrinema_pellirubrum_DSM_15624_uid74437	NC_019962	chromosome										
433591118	hypothetical protein Natpe_1834	No	0		1804023	1804211	-		Natrinema_pellirubrum_DSM_15624_uid74437	NC_019962	chromosome										
===								6	Natrinema_pellirubrum_DSM_15624_uid74437	NC_019962	chromosome										
433592013	hypothetical protein Natpe_2806	No	0		2745302	2745613	+		Natrinema_pellirubrum_DSM_15624_uid74437	NC_019962	chromosome			arCOG03924	10 (-)	K	MarR	Transcriptional regulator, MarR family			
433592014	hypothetical protein Natpe_2807	No	1		2746001	2747197	-		Natrinema_pellirubrum_DSM_15624_uid74437	NC_019962	chromosome										
433592015	hypothetical protein Natpe_2808	No	0		2747184	2748044	-		Natrinema_pellirubrum_DSM_15624_uid74437	NC_019962	chromosome										
433592016	hypothetical protein Natpe_2809	No	0		2748041	2748721	-		Natrinema_pellirubrum_DSM_15624_uid74437	NC_019962	chromosome										
433592017	hypothetical protein Natpe_2810	No	1		2748816	2748947	+		Natrinema_pellirubrum_DSM_15624_uid74437	NC_019962	chromosome										
433592018	phage integrase family protein	No	0		2749257	2749919	-		Natrinema_pellirubrum_DSM_15624_uid74437	NC_019962	chromosome			arCOG02282	8 (-)	K		Predicted transcriptional regulator, C-terminal HTH-like domain			
===								6	Natrinema_pellirubrum_DSM_15624_uid74437	NC_019967	plasmid pNATPE01										
433593378	RAMP superfamily protein	No	0	plasmid	65118	65975	-		Natrinema_pellirubrum_DSM_15624_uid74437	NC_019967	plasmid pNATPE01			arCOG08890	1 (-)	S		Uncharacterized conserved protein			
433593379	hypothetical protein Natpe_3951	No	0	plasmid	65986	66120	-		Natrinema_pellirubrum_DSM_15624_uid74437	NC_019967	plasmid pNATPE01										
433593380	hypothetical protein Natpe_3952	No	0	plasmid	66117	66395	-		Natrinema_pellirubrum_DSM_15624_uid74437	NC_019967	plasmid pNATPE01										
433593381	hypothetical protein Natpe_3953	Yes	0	plasmid	66388	66756	-		Natrinema_pellirubrum_DSM_15624_uid74437	NC_019967	plasmid pNATPE01										
433593382	hypothetical protein Natpe_3954	No	0	plasmid	66756	66968	-		Natrinema_pellirubrum_DSM_15624_uid74437	NC_019967	plasmid pNATPE01			arCOG09355	1	S		Uncharacterized conserved protein			
433593383	hypothetical protein Natpe_3955	No	0	plasmid	67162	67386	+		Natrinema_pellirubrum_DSM_15624_uid74437	NC_019967	plasmid pNATPE01			arCOG00393	1 (-)	K		Predicted membrane-associated trancriptional regulator			
===								7	Natrinema_pellirubrum_DSM_15624_uid74437	NC_019967	plasmid pNATPE01										
433593438	hypothetical protein Natpe_4031	No	0	plasmid	132995	133114	+		Natrinema_pellirubrum_DSM_15624_uid74437	NC_019967	plasmid pNATPE01										
433593439	hypothetical protein Natpe_4032	No	0	plasmid	133822	135561	-		Natrinema_pellirubrum_DSM_15624_uid74437	NC_019967	plasmid pNATPE01			arCOG06319	6 (-)	R		Protein containing HTH domain			
433593440	hypothetical protein Natpe_4033	No	0	plasmid	135558	135752	-		Natrinema_pellirubrum_DSM_15624_uid74437	NC_019967	plasmid pNATPE01										
433593441	hypothetical protein Natpe_4035	Yes	1	plasmid	136494	137621	-		Natrinema_pellirubrum_DSM_15624_uid74437	NC_019967	plasmid pNATPE01			arCOG11256	2	S		Uncharacterized conserved protein	0.000006	pfam07596	Protein of unknown function (DUF1559). A
433593442	hypothetical protein Natpe_4036	No	0	plasmid	137717	137818	+		Natrinema_pellirubrum_DSM_15624_uid74437	NC_019967	plasmid pNATPE01										
433593443	hypothetical protein Natpe_4037	No	0	plasmid	138005	138277	+		Natrinema_pellirubrum_DSM_15624_uid74437	NC_019967	plasmid pNATPE01			arCOG07968	1 (-)	S		Uncharacterized protein			
433593444	hypothetical protein Natpe_4038	No	0	plasmid	138394	139305	-		Natrinema_pellirubrum_DSM_15624_uid74437	NC_019967	plasmid pNATPE01										
===								6	Natrinema_pellirubrum_DSM_15624_uid74437	NC_019967	plasmid pNATPE01										
433593500	Calcium binding protein from Anabaena CcbP	No	0	plasmid	195066	195416	-		Natrinema_pellirubrum_DSM_15624_uid74437	NC_019967	plasmid pNATPE01			arCOG10634	1	S		Uncharacterized conserved protein	7E-28	pfam11535	Calcium binding. CcbP is a Ca(2+) bindin
433593501	hypothetical protein Natpe_4102	No	0	plasmid	195647	195958	-		Natrinema_pellirubrum_DSM_15624_uid74437	NC_019967	plasmid pNATPE01			arCOG09366	4	S		Uncharacterized conserved protein			
433593502	transposase family protein	No	0	plasmid	195969	196967	-		Natrinema_pellirubrum_DSM_15624_uid74437	NC_019967	plasmid pNATPE01			arCOG04442	3 (-)	L		Transposase			
433593503	hypothetical protein Natpe_4104	No	0	plasmid	196921	197271	-		Natrinema_pellirubrum_DSM_15624_uid74437	NC_019967	plasmid pNATPE01			arCOG09366	4	S		Uncharacterized conserved protein			
433593504	hypothetical protein Natpe_4105	No	0	plasmid	197507	197767	-		Natrinema_pellirubrum_DSM_15624_uid74437	NC_019967	plasmid pNATPE01										
433593505	hypothetical protein Natpe_4107	No	0	plasmid	198356	199099	-		Natrinema_pellirubrum_DSM_15624_uid74437	NC_019967	plasmid pNATPE01										
===								8	Natronobacterium_gregoryi_SP2_uid74439	NC_019792	chromosome										
429190064	hypothetical protein Natgr_0019	No	2		22973	23197	+		Natronobacterium_gregoryi_SP2_uid74439	NC_019792	chromosome										
429190065	hypothetical protein Natgr_0020	No	0		23419	23550	-		Natronobacterium_gregoryi_SP2_uid74439	NC_019792	chromosome										
429190066	transposase family protein	No	0		23711	25597	+		Natronobacterium_gregoryi_SP2_uid74439	NC_019792	chromosome		arCOG03900,a	arCOG03900	5 (-)	L		Transposase			
429190067	hypothetical protein Natgr_0023	No	0		26541	26990	+		Natronobacterium_gregoryi_SP2_uid74439	NC_019792	chromosome										
429190068	hypothetical protein Natgr_0024	No	0		26935	27243	+		Natronobacterium_gregoryi_SP2_uid74439	NC_019792	chromosome										
429190069	hypothetical protein Natgr_0025	No	0		27277	28161	+		Natronobacterium_gregoryi_SP2_uid74439	NC_019792	chromosome			arCOG02132	5 (-)	L		Transposase	3E-17	pfam12762	ISXO2-like transposase domain. This doma ; Transposase and inactivated derivatives
429190070	hypothetical protein Natgr_0026	No	0		28198	28389	+		Natronobacterium_gregoryi_SP2_uid74439	NC_019792	chromosome										
429190071	hypothetical protein Natgr_0027	No	0		28405	28788	-		Natronobacterium_gregoryi_SP2_uid74439	NC_019792	chromosome										
===								5	Natronobacterium_gregoryi_SP2_uid74439	NC_019792	chromosome										
429190353	hypothetical protein Natgr_0324	No	1		325822	326025	+		Natronobacterium_gregoryi_SP2_uid74439	NC_019792	chromosome										
429190354	hypothetical protein Natgr_0325	No	0		326004	326147	-		Natronobacterium_gregoryi_SP2_uid74439	NC_019792	chromosome										
429190355	dehydrogenase	No	0		326312	327352	-		Natronobacterium_gregoryi_SP2_uid74439	NC_019792	chromosome			arCOG01622	3 (-)	R	MviM	Predicted dehydrogenase	6E-22	COG0673	Predicted dehydrogenases and related pro
429190356	hypothetical protein Natgr_0327	No	1		327467	327736	+		Natronobacterium_gregoryi_SP2_uid74439	NC_019792	chromosome										
429190357	hypothetical protein Natgr_0328	No	2		327736	327945	+		Natronobacterium_gregoryi_SP2_uid74439	NC_019792	chromosome										
===								8	Natronobacterium_gregoryi_SP2_uid74439	NC_019792	chromosome										
429191793	hypothetical protein Natgr_1832	No	1	Virus_Caudovi	1817222	1817458	+		Natronobacterium_gregoryi_SP2_uid74439	NC_019792	chromosome										
429191794	hypothetical protein Natgr_1838	No	0	Virus_Caudovi	1823390	1823620	+		Natronobacterium_gregoryi_SP2_uid74439	NC_019792	chromosome	clust_406									
429191795	hypothetical protein Natgr_1839	No	0	Virus_Caudovi	1823608	1827183	+		Natronobacterium_gregoryi_SP2_uid74439	NC_019792	chromosome		arCOG01445,a	arCOG01445	1 (-)	V	Cas3	CRISPR-associated helicase Cas3			
429191796	hypothetical protein Natgr_1840	No	0	Virus_Caudovi	1827277	1827567	-		Natronobacterium_gregoryi_SP2_uid74439	NC_019792	chromosome										
429191797	hypothetical protein Natgr_1841	No	0	Virus_Caudovi	1827681	1828226	+		Natronobacterium_gregoryi_SP2_uid74439	NC_019792	chromosome	clust_195									
429191798	hypothetical protein Natgr_1842	No	0	Virus_Caudovi	1828234	1828488	+		Natronobacterium_gregoryi_SP2_uid74439	NC_019792	chromosome										
429191799	hypothetical protein Natgr_1843	No	1	Virus_Caudovi	1828497	1828823	+		Natronobacterium_gregoryi_SP2_uid74439	NC_019792	chromosome	clust_46									
429191800	transposase	No	0	Virus_Caudovi	1828827	1829354	+		Natronobacterium_gregoryi_SP2_uid74439	NC_019792	chromosome			arCOG09210	6 (-)	S		Uncharacterized conserved protein			
===								15	Natronobacterium_gregoryi_SP2_uid74439	NC_019792	chromosome										
429191804	hypothetical protein Natgr_1848	No	0	Virus_Caudovi	1833209	1834441	+		Natronobacterium_gregoryi_SP2_uid74439	NC_019792	chromosome								0.000009	pfam09979	Uncharacterized protein conserved in bac
429191805	hypothetical protein Natgr_1849	No	0	Virus_Caudovi	1834444	1834896	+		Natronobacterium_gregoryi_SP2_uid74439	NC_019792	chromosome										
429191806	hypothetical protein Natgr_1850	No	0	Virus_Caudovi	1834893	1835900	+		Natronobacterium_gregoryi_SP2_uid74439	NC_019792	chromosome			arCOG05908	2 (-)	R		Ferritin-like domain			
429191807	hypothetical protein Natgr_1851	No	0	Virus_Caudovi	1835907	1836233	+		Natronobacterium_gregoryi_SP2_uid74439	NC_019792	chromosome										
429191808	hypothetical protein Natgr_1852	No	0	Virus_Caudovi	1836230	1836409	+		Natronobacterium_gregoryi_SP2_uid74439	NC_019792	chromosome										
429191809	hypothetical protein Natgr_1853	No	0	Virus_Caudovi	1836490	1836849	+		Natronobacterium_gregoryi_SP2_uid74439	NC_019792	chromosome										
429191810	hypothetical protein Natgr_1854	No	1	Virus_Caudovi	1836856	1836990	+		Natronobacterium_gregoryi_SP2_uid74439	NC_019792	chromosome										
429191811	hypothetical protein Natgr_1855	No	0	Virus_Caudovi	1836987	1837433	+		Natronobacterium_gregoryi_SP2_uid74439	NC_019792	chromosome										
429191812	hypothetical protein Natgr_1856	No	0	Virus_Caudovi	1837423	1837809	+		Natronobacterium_gregoryi_SP2_uid74439	NC_019792	chromosome			arCOG08300	5 (-)	Y		Phage virion morphogenesis family, Mu gpG-like			
429191813	hypothetical protein Natgr_1857	No	0	Virus_Caudovi	1837806	1838234	+		Natronobacterium_gregoryi_SP2_uid74439	NC_019792	chromosome										
429191814	hypothetical protein Natgr_1858	No	0	Virus_Caudovi	1838238	1839071	+		Natronobacterium_gregoryi_SP2_uid74439	NC_019792	chromosome										
429191815	hypothetical protein Natgr_1859	No	0	Virus_Caudovi	1839075	1839554	+		Natronobacterium_gregoryi_SP2_uid74439	NC_019792	chromosome										
429191816	hypothetical protein Natgr_1860	No	2	Virus_Caudovi	1839697	1839927	-		Natronobacterium_gregoryi_SP2_uid74439	NC_019792	chromosome										
429191817	phage tail tape measure protein, TP901 family	No	3	Virus_Caudovi	1839996	1842074	+		Natronobacterium_gregoryi_SP2_uid74439	NC_019792	chromosome			arCOG00371	3 (-)	D	Smc	Chromosome segregation ATPase	1E-10	COG1196	Chromosome segregation ATPases
429191818	hypothetical protein Natgr_1862	No	0	Virus_Caudovi	1842078	1843196	+		Natronobacterium_gregoryi_SP2_uid74439	NC_019792	chromosome			arCOG10640	5	Y		Phage associated protein			
===								10	Natronobacterium_gregoryi_SP2_uid74439	NC_019792	chromosome										
429191820	hypothetical protein Natgr_1864	No	0	Virus_Caudovi	1844787	1847507	+		Natronobacterium_gregoryi_SP2_uid74439	NC_019792	chromosome		arCOG03175,a	arCOG03175	1 (-)	S		CDGSH-type zinc finger			
429191821	hypothetical protein Natgr_1865	Yes	2	Virus_Caudovi	1847576	1847908	-		Natronobacterium_gregoryi_SP2_uid74439	NC_019792	chromosome										
429191822	hypothetical protein Natgr_1866	No	0	Virus_Caudovi	1847953	1848147	-		Natronobacterium_gregoryi_SP2_uid74439	NC_019792	chromosome										
429191823	hypothetical protein Natgr_1867	No	1	Virus_Caudovi	1848170	1848277	-		Natronobacterium_gregoryi_SP2_uid74439	NC_019792	chromosome										
429191824	hypothetical protein Natgr_1868	No	1	Virus_Caudovi	1848456	1849085	-		Natronobacterium_gregoryi_SP2_uid74439	NC_019792	chromosome										
429191825	hypothetical protein Natgr_1869	No	0	Virus_Caudovi	1849218	1849313	-		Natronobacterium_gregoryi_SP2_uid74439	NC_019792	chromosome										
429191826	hypothetical protein Natgr_1870	No	0	Virus_Caudovi	1849471	1850355	-		Natronobacterium_gregoryi_SP2_uid74439	NC_019792	chromosome			arCOG02132	5 (-)	L		Transposase	6E-13	pfam12762	ISXO2-like transposase domain. This doma ; Transposase and inactivated derivatives
429191827	hypothetical protein Natgr_1871	No	1	Virus_Caudovi	1850497	1850607	-		Natronobacterium_gregoryi_SP2_uid74439	NC_019792	chromosome										
429191828	hypothetical protein Natgr_1872	No	0	Virus_Caudovi	1850657	1850803	+		Natronobacterium_gregoryi_SP2_uid74439	NC_019792	chromosome										
429191829	hypothetical protein Natgr_1873	No	0	Virus_Caudovi	1851187	1851387	-		Natronobacterium_gregoryi_SP2_uid74439	NC_019792	chromosome			arCOG11163	4	S		Uncharacterized conserved protein			
===								9	Natronobacterium_gregoryi_SP2_uid74439	NC_019792	chromosome										
429191832	hypothetical protein Natgr_1876	No	1	Virus_Caudovi	1853292	1853417	+		Natronobacterium_gregoryi_SP2_uid74439	NC_019792	chromosome										
429191833	hypothetical protein Natgr_1877	No	0	Virus_Caudovi	1853700	1853909	-		Natronobacterium_gregoryi_SP2_uid74439	NC_019792	chromosome										
429191834	hypothetical protein Natgr_1878	No	0	Virus_Caudovi	1853902	1854114	-		Natronobacterium_gregoryi_SP2_uid74439	NC_019792	chromosome			arCOG08128	7 (-)	S		Uncharacterized conserved protein			
429191835	hypothetical protein Natgr_1879	No	0	Virus_Caudovi	1854328	1854594	+		Natronobacterium_gregoryi_SP2_uid74439	NC_019792	chromosome										
429191836	hypothetical protein Natgr_1880	No	0	Virus_Caudovi	1854591	1855034	+		Natronobacterium_gregoryi_SP2_uid74439	NC_019792	chromosome										
429191837	hypothetical protein Natgr_1881	No	0	Virus_Caudovi	1855034	1855465	+		Natronobacterium_gregoryi_SP2_uid74439	NC_019792	chromosome										
429191838	hypothetical protein Natgr_1882	No	0	Virus_Caudovi	1855465	1855809	+		Natronobacterium_gregoryi_SP2_uid74439	NC_019792	chromosome			arCOG07773	5 (-)	S		SWIM zinc finger containing protein			
429191839	hypothetical protein Natgr_1883	No	0	Virus_Caudovi	1855797	1856039	+		Natronobacterium_gregoryi_SP2_uid74439	NC_019792	chromosome	clust_56									
429191840	hypothetical protein Natgr_1884	No	0	Virus_Caudovi	1856039	1856272	+		Natronobacterium_gregoryi_SP2_uid74439	NC_019792	chromosome										
===								5	Natronobacterium_gregoryi_SP2_uid74439	NC_019792	chromosome										
429192408	hypothetical protein Natgr_2472	No	2		2408633	2408857	+		Natronobacterium_gregoryi_SP2_uid74439	NC_019792	chromosome										
429192409	transposase	No	0		2410116	2411108	+		Natronobacterium_gregoryi_SP2_uid74439	NC_019792	chromosome			arCOG03585	6 (-)	L		Transposase	5E-25	COG3547	Transposase and inactivated derivatives
429192410	hypothetical protein Natgr_2474	No	0		2411320	2411445	+		Natronobacterium_gregoryi_SP2_uid74439	NC_019792	chromosome										
429192411	hypothetical protein Natgr_2475	No	0		2411458	2411703	-		Natronobacterium_gregoryi_SP2_uid74439	NC_019792	chromosome										
429192412	hypothetical protein Natgr_2476	No	1		2411722	2412297	-		Natronobacterium_gregoryi_SP2_uid74439	NC_019792	chromosome										
===								7	Natronobacterium_gregoryi_SP2_uid74439	NC_019792	chromosome										
429192524	hypothetical protein Natgr_2601	No	0		2529673	2529792	+		Natronobacterium_gregoryi_SP2_uid74439	NC_019792	chromosome										
429192525	sugar metabolism transcriptional regulator	No	0		2529956	2530228	-		Natronobacterium_gregoryi_SP2_uid74439	NC_019792	chromosome			arCOG01446	5 (-)	VK	Csa3	CRISPR-Cas assicated transcriptional regulator, contains CARF and HTH domain			
429192526	hypothetical protein Natgr_2603	No	0		2530461	2530703	+		Natronobacterium_gregoryi_SP2_uid74439	NC_019792	chromosome										
429192527	hypothetical protein Natgr_2604	No	0		2530796	2531101	-		Natronobacterium_gregoryi_SP2_uid74439	NC_019792	chromosome										
429192528	hypothetical protein Natgr_2606	No	0		2532725	2532994	+		Natronobacterium_gregoryi_SP2_uid74439	NC_019792	chromosome										
429192529	hypothetical protein Natgr_2607	No	0		2533526	2535346	-		Natronobacterium_gregoryi_SP2_uid74439	NC_019792	chromosome			arCOG06319	6 (-)	R		Protein containing HTH domain			
429192530	hypothetical protein Natgr_2608	No	0		2535557	2535673	+		Natronobacterium_gregoryi_SP2_uid74439	NC_019792	chromosome										
===								5	Natronobacterium_gregoryi_SP2_uid74439	NC_019792	chromosome										
429192950	hypothetical protein Natgr_3045	No	3		2985196	2985555	+		Natronobacterium_gregoryi_SP2_uid74439	NC_019792	chromosome	clust_300									
429192951	hypothetical protein Natgr_3046	No	4		2985527	2985943	+		Natronobacterium_gregoryi_SP2_uid74439	NC_019792	chromosome										
429192952	hypothetical protein Natgr_3047	No	4		2986261	2986581	-		Natronobacterium_gregoryi_SP2_uid74439	NC_019792	chromosome			arCOG11193	1	S		Uncharacterized conserved protein			
429192953	tyrosine decarboxylase MnfA	No	0		2986903	2987991	-		Natronobacterium_gregoryi_SP2_uid74439	NC_019792	chromosome			arCOG00027	1 (-)	E	MfnA	L-tyrosine decarboxylase, PLP-dependent protei	1E-54	COG0076	Glutamate decarboxylase and related PLP-
429192954	hypothetical protein Natgr_3049	No	0		2988108	2988212	+		Natronobacterium_gregoryi_SP2_uid74439	NC_019792	chromosome										
===								41	Natronobacterium_gregoryi_SP2_uid74439	NC_019792	chromosome										
429193304	hypothetical protein Natgr_3414	No	0	Virus_Caudovi	3384788	3384895	+		Natronobacterium_gregoryi_SP2_uid74439	NC_019792	chromosome										
429193305	hypothetical protein Natgr_3415	No	0	Virus_Caudovi	3384888	3385391	+		Natronobacterium_gregoryi_SP2_uid74439	NC_019792	chromosome	clust_206									
429193306	ATPase	No	0	Virus_Caudovi	3385581	3387710	+		Natronobacterium_gregoryi_SP2_uid74439	NC_019792	chromosome			arCOG00439	14 (-)	L	MCM2	Predicted ATPase involved in replication control,	3E-121	COG1241	Predicted ATPase involved in replication
429193307	hypothetical protein Natgr_3417	No	0	Virus_Caudovi	3387713	3388087	+		Natronobacterium_gregoryi_SP2_uid74439	NC_019792	chromosome										
429193308	hypothetical protein Natgr_3418	No	0	Virus_Caudovi	3388084	3388311	+		Natronobacterium_gregoryi_SP2_uid74439	NC_019792	chromosome			arCOG06155	6	S		Uncharacterized conserved protein			
429193309	hypothetical protein Natgr_3419	No	0	Virus_Caudovi	3388308	3388700	+		Natronobacterium_gregoryi_SP2_uid74439	NC_019792	chromosome										
429193310	hypothetical protein Natgr_3420	No	0	Virus_Caudovi	3388693	3388917	+		Natronobacterium_gregoryi_SP2_uid74439	NC_019792	chromosome			arCOG00394	7 (-)	K	ArsR	Transcriptional regulator containing HTH domain, ArsR family			
429193311	hypothetical protein Natgr_3421	No	0	Virus_Caudovi	3389063	3389605	+		Natronobacterium_gregoryi_SP2_uid74439	NC_019792	chromosome										
429193312	hypothetical protein Natgr_3422	No	0	Virus_Caudovi	3390144	3390578	+		Natronobacterium_gregoryi_SP2_uid74439	NC_019792	chromosome	clust_238									
429193313	hypothetical protein Natgr_3423	No	1	Virus_Caudovi	3390587	3390910	+		Natronobacterium_gregoryi_SP2_uid74439	NC_019792	chromosome	clust_46									
429193314	hypothetical protein Natgr_3424	No	0	Virus_Caudovi	3390987	3391385	+		Natronobacterium_gregoryi_SP2_uid74439	NC_019792	chromosome										
429193315	hypothetical protein Natgr_3425	No	0	Virus_Caudovi	3391382	3391963	+		Natronobacterium_gregoryi_SP2_uid74439	NC_019792	chromosome										
429193316	hypothetical protein Natgr_3426	No	0	Virus_Caudovi	3391974	3392282	+		Natronobacterium_gregoryi_SP2_uid74439	NC_019792	chromosome			arCOG11198	4	S		Uncharacterized conserved protein			
429193317	hypothetical protein Natgr_3427	No	1	Virus_Caudovi	3392257	3392409	-		Natronobacterium_gregoryi_SP2_uid74439	NC_019792	chromosome										
429193318	hypothetical protein Natgr_3428	No	0	Virus_Caudovi	3392545	3393147	+		Natronobacterium_gregoryi_SP2_uid74439	NC_019792	chromosome			arCOG09210	6 (-)	S		Uncharacterized conserved protein			
429193319	hypothetical protein Natgr_3429	No	0	Virus_Caudovi	3393144	3394718	+		Natronobacterium_gregoryi_SP2_uid74439	NC_019792	chromosome			arCOG09550	17	Y	TerL	Phage terminase large subunit			
429193320	hypothetical protein Natgr_3430	No	0	Virus_Caudovi	3394715	3394978	+		Natronobacterium_gregoryi_SP2_uid74439	NC_019792	chromosome										
429193321	bacteriophage capsid portal protein	No	0	Virus_Caudovi	3394971	3396911	+		Natronobacterium_gregoryi_SP2_uid74439	NC_019792	chromosome		arCOG08094,a	arCOG08094	3 (-)	S		Uncharacterized conserved protein	0.000000003	COG5518	Bacteriophage capsid portal protein
429193322	hypothetical protein Natgr_3432	No	1	Virus_Caudovi	3396914	3397225	+		Natronobacterium_gregoryi_SP2_uid74439	NC_019792	chromosome										
429193323	hypothetical protein Natgr_3433	No	0	Virus_Caudovi	3397309	3397428	+		Natronobacterium_gregoryi_SP2_uid74439	NC_019792	chromosome										
429193324	hypothetical protein Natgr_3434	No	0	Virus_Caudovi	3397432	3397623	+		Natronobacterium_gregoryi_SP2_uid74439	NC_019792	chromosome										
429193325	hypothetical protein Natgr_3435	No	0	Virus_Caudovi	3397627	3399306	+		Natronobacterium_gregoryi_SP2_uid74439	NC_019792	chromosome										
429193326	hypothetical protein Natgr_3436	No	0	Virus_Caudovi	3399322	3400404	+		Natronobacterium_gregoryi_SP2_uid74439	NC_019792	chromosome										
429193327	hypothetical protein Natgr_3437	No	0	Virus_Caudovi	3400416	3400601	+		Natronobacterium_gregoryi_SP2_uid74439	NC_019792	chromosome										
429193328	hypothetical protein Natgr_3438	No	0	Virus_Caudovi	3400613	3400984	+		Natronobacterium_gregoryi_SP2_uid74439	NC_019792	chromosome										
429193329	hypothetical protein Natgr_3439	No	0	Virus_Caudovi	3400991	3401836	+		Natronobacterium_gregoryi_SP2_uid74439	NC_019792	chromosome										
429193330	hypothetical protein Natgr_3440	Yes	0	Virus_Caudovi	3401833	3402201	+		Natronobacterium_gregoryi_SP2_uid74439	NC_019792	chromosome										
429193331	hypothetical protein Natgr_3441	No	0	Virus_Caudovi	3402201	3402647	+		Natronobacterium_gregoryi_SP2_uid74439	NC_019792	chromosome										
429193332	hypothetical protein Natgr_3442	No	0	Virus_Caudovi	3402644	3403189	+		Natronobacterium_gregoryi_SP2_uid74439	NC_019792	chromosome	clust_191									
429193333	hypothetical protein Natgr_3443	No	0	Virus_Caudovi	3403192	3403386	+		Natronobacterium_gregoryi_SP2_uid74439	NC_019792	chromosome										
429193334	hypothetical protein Natgr_3444	No	0	Virus_Caudovi	3403379	3403672	+		Natronobacterium_gregoryi_SP2_uid74439	NC_019792	chromosome										
429193335	hypothetical protein Natgr_3445	No	0	Virus_Caudovi	3403669	3404580	+		Natronobacterium_gregoryi_SP2_uid74439	NC_019792	chromosome										
429193336	hypothetical protein Natgr_3446	No	0	Virus_Caudovi	3404582	3405124	+		Natronobacterium_gregoryi_SP2_uid74439	NC_019792	chromosome										
429193337	hypothetical protein Natgr_3447	No	0	Virus_Caudovi	3405121	3405237	+		Natronobacterium_gregoryi_SP2_uid74439	NC_019792	chromosome										
429193338	hypothetical protein Natgr_3448	No	0	Virus_Caudovi	3405234	3406367	+		Natronobacterium_gregoryi_SP2_uid74439	NC_019792	chromosome								0.00001	COG3753	Uncharacterized protein conserved in bac
429193339	hypothetical protein Natgr_3449	No	2	Virus_Caudovi	3406399	3406614	+		Natronobacterium_gregoryi_SP2_uid74439	NC_019792	chromosome										
429193340	hypothetical protein Natgr_3450	No	0	Virus_Caudovi	3406794	3407459	+		Natronobacterium_gregoryi_SP2_uid74439	NC_019792	chromosome			arCOG10639	4	Y		Phage associated protein			
429193341	hypothetical protein Natgr_3451	No	0	Virus_Caudovi	3407463	3408770	+		Natronobacterium_gregoryi_SP2_uid74439	NC_019792	chromosome			arCOG10640	5	Y		Phage associated protein			
429193342	hypothetical protein Natgr_3452	No	0	Virus_Caudovi	3408804	3409523	+		Natronobacterium_gregoryi_SP2_uid74439	NC_019792	chromosome										
429193343	hypothetical protein Natgr_3453	No	0	Virus_Caudovi	3409526	3410278	+		Natronobacterium_gregoryi_SP2_uid74439	NC_019792	chromosome										
429193344	hypothetical protein Natgr_3454	No	0	Virus_Caudovi	3410347	3412749	+		Natronobacterium_gregoryi_SP2_uid74439	NC_019792	chromosome		arCOG03557,a	arCOG03557	1 (-)	M		Cell suface protein			
===								20	Natronobacterium_gregoryi_SP2_uid74439	NC_019792	chromosome										
429193357	hypothetical protein Natgr_3467	No	0		3433221	3433619	+		Natronobacterium_gregoryi_SP2_uid74439	NC_019792	chromosome			arCOG00619	1 (-)	E	GltB	Glutamate synthase domain 2 and ferredoxin domain			
429193358	hypothetical protein Natgr_3468	No	0		3433622	3433816	+		Natronobacterium_gregoryi_SP2_uid74439	NC_019792	chromosome										
429193359	hypothetical protein Natgr_3469	No	0		3433813	3434280	+		Natronobacterium_gregoryi_SP2_uid74439	NC_019792	chromosome	clust_224									
429193360	hypothetical protein Natgr_3470	No	0		3434281	3434886	+		Natronobacterium_gregoryi_SP2_uid74439	NC_019792	chromosome										
429193361	hypothetical protein Natgr_3471	No	0		3434886	3435113	+		Natronobacterium_gregoryi_SP2_uid74439	NC_019792	chromosome										
429193362	hypothetical protein Natgr_3472	No	0		3435422	3435640	-		Natronobacterium_gregoryi_SP2_uid74439	NC_019792	chromosome			arCOG08128	7 (-)	S		Uncharacterized conserved protein			
429193363	hypothetical protein Natgr_3473	No	2		3435651	3435860	+		Natronobacterium_gregoryi_SP2_uid74439	NC_019792	chromosome										
429193364	hypothetical protein Natgr_3474	No	2		3435940	3436149	+		Natronobacterium_gregoryi_SP2_uid74439	NC_019792	chromosome										
429193365	Cdc6-related protein, AAA superfamily ATPase	No	0		3436230	3437315	+		Natronobacterium_gregoryi_SP2_uid74439	NC_019792	chromosome			arCOG00467	13 (-)	L	CDC6	Cdc6-related protein, AAA superfamily ATPase	2E-23	COG1474	Cdc6-related protein, AAA superfamily AT
429193366	hypothetical protein Natgr_3476	No	0		3437338	3437502	-		Natronobacterium_gregoryi_SP2_uid74439	NC_019792	chromosome										
429193367	hypothetical protein Natgr_3477	No	0		3438098	3438397	+		Natronobacterium_gregoryi_SP2_uid74439	NC_019792	chromosome										
429193368	hypothetical protein Natgr_3478	No	0		3438394	3438756	+		Natronobacterium_gregoryi_SP2_uid74439	NC_019792	chromosome										
429193369	hypothetical protein Natgr_3479	No	0		3438753	3439502	+		Natronobacterium_gregoryi_SP2_uid74439	NC_019792	chromosome	clust_133									
429193370	hypothetical protein Natgr_3480	No	0		3439502	3439648	+		Natronobacterium_gregoryi_SP2_uid74439	NC_019792	chromosome										
429193371	hypothetical protein Natgr_3481	No	0		3439641	3440534	+		Natronobacterium_gregoryi_SP2_uid74439	NC_019792	chromosome										
429193372	hypothetical protein Natgr_3482	No	0		3440534	3441214	+		Natronobacterium_gregoryi_SP2_uid74439	NC_019792	chromosome										
429193373	hypothetical protein Natgr_3483	No	1		3441207	3441461	+		Natronobacterium_gregoryi_SP2_uid74439	NC_019792	chromosome										
429193374	hypothetical protein Natgr_3484	No	0		3441458	3441769	+		Natronobacterium_gregoryi_SP2_uid74439	NC_019792	chromosome										
429193375	hypothetical protein Natgr_3485	No	0		3441766	3441930	+		Natronobacterium_gregoryi_SP2_uid74439	NC_019792	chromosome										
429193376	site-specific recombinase XerD	No	0		3441954	3443165	-		Natronobacterium_gregoryi_SP2_uid74439	NC_019792	chromosome			arCOG01243	4 (-)	L	XerC	Site-specific recombinase XerC			
===								20	Natronobacterium_gregoryi_SP2_uid74439	NC_019792	chromosome										
429193388	hypothetical protein Natgr_3498	No	0		3453327	3453536	-		Natronobacterium_gregoryi_SP2_uid74439	NC_019792	chromosome										
429193389	site-specific recombinase XerD	No	0		3453746	3454738	+		Natronobacterium_gregoryi_SP2_uid74439	NC_019792	chromosome			arCOG01241	31 (-)	L	XerC	Integrase	3E-14	COG4974	Site-specific recombinase XerD
429193390	hypothetical protein Natgr_3500	No	0		3454794	3455036	+		Natronobacterium_gregoryi_SP2_uid74439	NC_019792	chromosome										
429193391	hypothetical protein Natgr_3501	No	0		3455037	3455225	+		Natronobacterium_gregoryi_SP2_uid74439	NC_019792	chromosome										
429193392	hypothetical protein Natgr_3502	No	0		3455222	3455596	+		Natronobacterium_gregoryi_SP2_uid74439	NC_019792	chromosome										
429193393	hypothetical protein Natgr_3503	No	0		3455593	3455781	-		Natronobacterium_gregoryi_SP2_uid74439	NC_019792	chromosome										
429193394	site-specific recombinase, DNA invertase Pin	No	0		3455830	3456582	-		Natronobacterium_gregoryi_SP2_uid74439	NC_019792	chromosome			arCOG03162	12 (-)	L	PinR	Site-specific recombinase, DNA invertase Pin ho	0.00000003	COG1961	Site-specific recombinases, DNA invertas
429193395	hypothetical protein Natgr_3505	No	0		3456680	3456880	-		Natronobacterium_gregoryi_SP2_uid74439	NC_019792	chromosome										
429193396	hypothetical protein Natgr_3506	No	2		3456882	3457196	-		Natronobacterium_gregoryi_SP2_uid74439	NC_019792	chromosome										
429193397	hypothetical protein Natgr_3507	No	2		3457183	3457401	-		Natronobacterium_gregoryi_SP2_uid74439	NC_019792	chromosome										
429193398	hypothetical protein Natgr_3508	No	0		3457394	3457654	-		Natronobacterium_gregoryi_SP2_uid74439	NC_019792	chromosome										
429193399	hypothetical protein Natgr_3509	No	0		3457726	3458175	-		Natronobacterium_gregoryi_SP2_uid74439	NC_019792	chromosome										

429193400	type I restriction-modification system methyltra	No	0			3458347	3461286	-		Natronobacterium_gregoryi_SP2_uid74439	NC_019792	chromosome		arCOG02632	3 (-)	V	HsdM	Type I restriction-modification system methyltrans	5E-16	COG0286	Type I restriction-modification system m
429193401	hypothetical protein Natgr_3511	No	0			3461384	3461617	+		Natronobacterium_gregoryi_SP2_uid74439	NC_019792	chromosome		arCOG10165	3 (-)	S		Uncharacterized conserved protein			
429193402	hypothetical protein Natgr_3513	No	2			3461986	3462207	-		Natronobacterium_gregoryi_SP2_uid74439	NC_019792	chromosome									
429193403	hypothetical protein Natgr_3514	No	0			3462665	3462982	-		Natronobacterium_gregoryi_SP2_uid74439	NC_019792	chromosome									
429193404	hypothetical protein Natgr_3515	No	0			3462976	3463176	-		Natronobacterium_gregoryi_SP2_uid74439	NC_019792	chromosome									
429193405	hypothetical protein Natgr_3516	No	0			3463503	3463925	-		Natronobacterium_gregoryi_SP2_uid74439	NC_019792	chromosome									
429193406	hypothetical protein Natgr_3517	Yes	0			3464069	3464569	-		Natronobacterium_gregoryi_SP2_uid74439	NC_019792	chromosome							0.00003	pfam10446	Protein of unknown function (DUF2457). T
429193407	hypothetical protein Natgr_3518	No	0			3464959	3465207	+		Natronobacterium_gregoryi_SP2_uid74439	NC_019792	chromosome		arCOG08928	1 (-)	S		Uncharacterized conserved protein			
===									9	Natronobacterium_gregoryi_SP2_uid74439	NC_019792	chromosome									
429193412	hypothetical protein Natgr_3523	No	2			3468825	3469670	-		Natronobacterium_gregoryi_SP2_uid74439	NC_019792	chromosome									
429193413	hypothetical protein Natgr_3524	Yes	2			3469667	3470221	-		Natronobacterium_gregoryi_SP2_uid74439	NC_019792	chromosome									
429193414	hypothetical protein Natgr_3525	Yes	2			3470286	3470999	-		Natronobacterium_gregoryi_SP2_uid74439	NC_019792	chromosome		arCOG02087	2 (-)	S		Predicted membrane protein	0.000004	pfam04147	Nop14-like family. Emg1 and Nop14 are no
429193415	hypothetical protein Natgr_3526	No	1			3471057	3472970	-		Natronobacterium_gregoryi_SP2_uid74439	NC_019792	chromosome									
429193416	hypothetical protein Natgr_3527	No	2			3473201	3473401	-		Natronobacterium_gregoryi_SP2_uid74439	NC_019792	chromosome									
429193417	hypothetical protein Natgr_3528	No	0			3474691	3475212	+		Natronobacterium_gregoryi_SP2_uid74439	NC_019792	chromosome							0.00001	pfam04156	IncA protein. Chlamydia trachomatis is a
429193418	hypothetical protein Natgr_3529	No	0			3475218	3475340	-		Natronobacterium_gregoryi_SP2_uid74439	NC_019792	chromosome									
429193419	hypothetical protein Natgr_3530	No	0			3475922	3476173	-		Natronobacterium_gregoryi_SP2_uid74439	NC_019792	chromosome									
429193420	hypothetical protein Natgr_3531	No	0			3476390	3476602	+		Natronobacterium_gregoryi_SP2_uid74439	NC_019792	chromosome		arCOG03748	2 (-)	K	MarR	Transcriptional regulator, MarR family			
===									6	Natronococcus_occultus_SP4_uid184863	NC_019974	chromosome									
435846451	site-specific recombinase XerC	No	0			1025523	1026647	-		Natronococcus_occultus_SP4_uid184863	NC_019974	chromosome		arCOG01250	7 (-)	L	XerC	Integrase			
435846452	hypothetical protein Natoc_1070	No	0			1026907	1027023	+		Natronococcus_occultus_SP4_uid184863	NC_019974	chromosome									
435846453	hypothetical protein Natoc_1071	No	3			1027142	1027804	+		Natronococcus_occultus_SP4_uid184863	NC_019974	chromosome									
435846454	hypothetical protein Natoc_1072	No	0			1027801	1028052	+		Natronococcus_occultus_SP4_uid184863	NC_019974	chromosome									
435846455	hypothetical protein Natoc_1073	No	0			1028064	1028684	+		Natronococcus_occultus_SP4_uid184863	NC_019974	chromosome									
435846456	hypothetical protein Natoc_1074	No	0			1028757	1029206	+		Natronococcus_occultus_SP4_uid184863	NC_019974	chromosome		arCOG06276	3 (-)	L		Primase-polymerase (primpol) domain containing	3E-17	COG4983	Uncharacterized conserved protein
===									5	Natronococcus_occultus_SP4_uid184863	NC_019974	chromosome									
435847108	hypothetical protein Natoc_1769	No	0			1717786	1717950	-		Natronococcus_occultus_SP4_uid184863	NC_019974	chromosome									
435847109	hypothetical protein Natoc_1770	No	0			1718186	1718365	+		Natronococcus_occultus_SP4_uid184863	NC_019974	chromosome									
435847110	hypothetical protein Natoc_1771	No	2			1718430	1719275	+		Natronococcus_occultus_SP4_uid184863	NC_019974	chromosome		arCOG08109	2 (-)	S		Uncharacterized conserved protein			
435847111	hypothetical protein Natoc_1772	No	0			1719425	1720156	+		Natronococcus_occultus_SP4_uid184863	NC_019974	chromosome									
435847112	hypothetical protein Natoc_1773	No	0			1720201	1720758	+		Natronococcus_occultus_SP4_uid184863	NC_019974	chromosome									
===									10	Natronococcus_occultus_SP4_uid184863	NC_019974	chromosome									
435847321	hypothetical protein Natoc_1993	No	3			1921630	1921938	-		Natronococcus_occultus_SP4_uid184863	NC_019974	chromosome									
435847322	hypothetical protein Natoc_1995	No	2			1922179	1922445	+		Natronococcus_occultus_SP4_uid184863	NC_019974	chromosome									
435847323	hypothetical protein Natoc_1996	No	0			1923101	1924198	+		Natronococcus_occultus_SP4_uid184863	NC_019974	chromosome		arCOG05724	3 (-)	V		Predicted type IV restriction endonuclease	7E-13	COG4748	Uncharacterized conserved protein
435847324	hypothetical protein Natoc_1997	No	0			1924288	1924935	+		Natronococcus_occultus_SP4_uid184863	NC_019974	chromosome									
435847325	hypothetical protein Natoc_1998	No	0			1924986	1926125	-		Natronococcus_occultus_SP4_uid184863	NC_019974	chromosome							5E-18	pfam14022	Protein of unknown function (DUF4238). T
435847326	hypothetical protein Natoc_2000	No	0			1927224	1928261	+		Natronococcus_occultus_SP4_uid184863	NC_019974	chromosome									
435847327	hypothetical protein Natoc_2001	No	0			1928295	1929062	+		Natronococcus_occultus_SP4_uid184863	NC_019974	chromosome									
435847328	hypothetical protein Natoc_2002	No	2			1929073	1929786	+		Natronococcus_occultus_SP4_uid184863	NC_019974	chromosome									
435847329	hypothetical protein Natoc_2003	No	0			1930196	1930438	-		Natronococcus_occultus_SP4_uid184863	NC_019974	chromosome									
435847330	glycosyl transferase	No	0			1930642	1931307	+		Natronococcus_occultus_SP4_uid184863	NC_019974	chromosome		arCOG01385	2 (-)	M	WcaA	Glycosyltransferase	8E-14	pfam00535	Glycosyl transferase family 2. Diverse f
===									8	Natronococcus_occultus_SP4_uid184863	NC_019974	chromosome									
435847584	hypothetical protein Natoc_2266	No	0			2186882	2186974	+		Natronococcus_occultus_SP4_uid184863	NC_019974	chromosome									
435847585	putative metal-dependent hydrolase	No	0			2186967	2187797	+		Natronococcus_occultus_SP4_uid184863	NC_019974	chromosome		arCOG02625	3 (-)	R		Predicted metal-dependent hydrolase			
435847586	hypothetical protein Natoc_2268	No	0			2188392	2188538	-		Natronococcus_occultus_SP4_uid184863	NC_019974	chromosome									
435847587	hypothetical protein Natoc_2269	No	0			2189838	2191220	-		Natronococcus_occultus_SP4_uid184863	NC_019974	chromosome									
435847588	hypothetical protein Natoc_2270	No	0			2192051	2192161	+		Natronococcus_occultus_SP4_uid184863	NC_019974	chromosome									
435847589	DNA modification methylase	No	0			2192335	2193630	-		Natronococcus_occultus_SP4_uid184863	NC_019974	chromosome		arCOG00115	10 (-)	L		DNA modification methylase	0.00000001	pfam01555	DNA methylase. Members of this family ar ; DNA methylase. Members of this family ar
435847590	hypothetical protein Natoc_2272	No	0			2193746	2194777	-		Natronococcus_occultus_SP4_uid184863	NC_019974	chromosome	clust_31								
435847591	hypothetical protein Natoc_2273	No	0			2194777	2196978	-		Natronococcus_occultus_SP4_uid184863	NC_019974	chromosome							0.00000003	pfam09528	Ehrlichia tandem repeat (Ehrlichia_rpt).
===									19	Natronococcus_occultus_SP4_uid184863	NC_019974	chromosome									
435847595	Protein of unknown function (DUF2971)	No	0			2202098	2202883	-		Natronococcus_occultus_SP4_uid184863	NC_019974	chromosome		arCOG07807	3	S		Uncharacterized conserved protein			
435847596	phage/plasmid primase, P4 family, C-terminal (	No	0			2203055	2205199	-		Natronococcus_occultus_SP4_uid184863	NC_019974	chromosome		arCOG06914	10 (-)	R		Phage or plasmid associated DNA primase	3E-21	COG3378	Phage associated DNA primase
435847597	hypothetical protein Natoc_2279	No	0			2205878	2206015	-		Natronococcus_occultus_SP4_uid184863	NC_019974	chromosome									
435847598	hypothetical protein Natoc_2280	No	1			2206012	2206170	-		Natronococcus_occultus_SP4_uid184863	NC_019974	chromosome									
435847599	hypothetical protein Natoc_2281	Yes	1			2206167	2207084	-		Natronococcus_occultus_SP4_uid184863	NC_019974	chromosome							0.0000007	pfam10446	Protein of unknown function (DUF2457). T
435847600	hypothetical protein Natoc_2282	No	1			2207247	2207342	+		Natronococcus_occultus_SP4_uid184863	NC_019974	chromosome									
435847601	hypothetical protein Natoc_2283	No	0			2207525	2207830	-		Natronococcus_occultus_SP4_uid184863	NC_019974	chromosome									
435847602	hypothetical protein Natoc_2284	No	0			2208175	2208435	-		Natronococcus_occultus_SP4_uid184863	NC_019974	chromosome									
435847603	hypothetical protein Natoc_2285	No	0			2208428	2208811	-		Natronococcus_occultus_SP4_uid184863	NC_019974	chromosome									
435847604	hypothetical protein Natoc_2286	No	0			2208958	2209263	-		Natronococcus_occultus_SP4_uid184863	NC_019974	chromosome									
435847605	twin arginine-targeting protein translocase, TatA	No	1			2209828	2210022	-		Natronococcus_occultus_SP4_uid184863	NC_019974	chromosome		arCOG02694	2 (-)	U	TatA	Sec-independent protein secretion pathway com	0.00000006	pfam02416	mttA/Hcf106 family. Members of this prot
435847606	hypothetical protein Natoc_2288	Yes	1			2210073	2210660	-		Natronococcus_occultus_SP4_uid184863	NC_019974	chromosome									
435847607	hypothetical protein Natoc_2289	No	0			2211881	2212990	-		Natronococcus_occultus_SP4_uid184863	NC_019974	chromosome									
435847608	hypothetical protein Natoc_2290	No	0			2213034	2214128	-		Natronococcus_occultus_SP4_uid184863	NC_019974	chromosome		arCOG08166	1 (-)	R		Predicted ATPase (AAA+ superfamily)			
435847609	hypothetical protein Natoc_2291	No	2			2214125	2214913	-		Natronococcus_occultus_SP4_uid184863	NC_019974	chromosome									
435847610	hypothetical protein Natoc_2292	No	2			2215541	2215753	-		Natronococcus_occultus_SP4_uid184863	NC_019974	chromosome									
435847611	hypothetical protein Natoc_2293	Yes	1			2215750	2217813	-		Natronococcus_occultus_SP4_uid184863	NC_019974	chromosome		arCOG07782	7 (-)	S		Uncharacterized conserved protein			
435847612	hypothetical protein Natoc_2294	No	2			2217957	2218169	-		Natronococcus_occultus_SP4_uid184863	NC_019974	chromosome									
435847613	hypothetical protein Natoc_2295	No	1			2218281	2218982	-		Natronococcus_occultus_SP4_uid184863	NC_019974	chromosome							0.0001	pfam04147	Nop14-like family. Emg1 and Nop14 are no
===									5	Natronococcus_occultus_SP4_uid184863	NC_019974	chromosome									
435848891	hypothetical protein Natoc_3635	No	0			3575268	3576395	+		Natronococcus_occultus_SP4_uid184863	NC_019974	chromosome		arCOG06120	2	S		Uncharacterized conserved protein			
435848892	hypothetical protein Natoc_3636	No	0			3576641	3576757	+		Natronococcus_occultus_SP4_uid184863	NC_019974	chromosome		arCOG08180	3 (-)	S		Uncharacterized conserved protein			
435848893	hypothetical protein Natoc_3637	No	0			3576881	3577033	+		Natronococcus_occultus_SP4_uid184863	NC_019974	chromosome									
435848894	hypothetical protein Natoc_3638	No	0			3577094	3577324	+		Natronococcus_occultus_SP4_uid184863	NC_019974	chromosome									
435848895	hypothetical protein Natoc_3639	No	0			3577455	3577658	-		Natronococcus_occultus_SP4_uid184863	NC_019974	chromosome									
===									21	Natronococcus_occultus_SP4_uid184863	NC_019975	plasmid 1									
435849357	hypothetical protein Natoc_4354	No	0		plasmid	58	297	+		Natronococcus_occultus_SP4_uid184863	NC_019975	plasmid 1	clust_406								
435849358	hypothetical protein Natoc_4355	No	0		plasmid	290	805	+		Natronococcus_occultus_SP4_uid184863	NC_019975	plasmid 1									
435849359	hypothetical protein Natoc_4356	No	0		plasmid	1307	1747	+		Natronococcus_occultus_SP4_uid184863	NC_019975	plasmid 1									
435849360	hypothetical protein Natoc_4357	No	0		plasmid	1753	2397	+		Natronococcus_occultus_SP4_uid184863	NC_019975	plasmid 1									
435849361	hypothetical protein Natoc_4358	No	0		plasmid	2400	2789	+		Natronococcus_occultus_SP4_uid184863	NC_019975	plasmid 1									
435849362	hypothetical protein Natoc_4359	No	0		plasmid	2786	3001	+		Natronococcus_occultus_SP4_uid184863	NC_019975	plasmid 1									
435849363	hypothetical protein Natoc_4360	No	0		plasmid	3074	3331	+		Natronococcus_occultus_SP4_uid184863	NC_019975	plasmid 1		arCOG10959	8	S		Uncharacterized conserved protein			
435849364	hypothetical protein Natoc_4361	No	0		plasmid	3331	3549	+		Natronococcus_occultus_SP4_uid184863	NC_019975	plasmid 1									
435849365	hypothetical protein Natoc_4362	No	0		plasmid	3831	4049	+		Natronococcus_occultus_SP4_uid184863	NC_019975	plasmid 1									
435849366	hypothetical protein Natoc_4363	No	0		plasmid	4039	4146	+		Natronococcus_occultus_SP4_uid184863	NC_019975	plasmid 1									
435849367	hypothetical protein Natoc_4364	No	0		plasmid	5130	5351	-		Natronococcus_occultus_SP4_uid184863	NC_019975	plasmid 1									
435849368	hypothetical protein Natoc_4365	No	0		plasmid	5645	5848	-		Natronococcus_occultus_SP4_uid184863	NC_019975	plasmid 1									
435849369	hypothetical protein Natoc_4366	No	0		plasmid	5848	6033	-		Natronococcus_occultus_SP4_uid184863	NC_019975	plasmid 1									
435849370	hypothetical protein Natoc_4367	No	0		plasmid	6033	6290	-		Natronococcus_occultus_SP4_uid184863	NC_019975	plasmid 1									
435849371	Sugar-specific transcriptional regulator TrmB	No	0		plasmid	6449	6721	+		Natronococcus_occultus_SP4_uid184863	NC_019975	plasmid 1		arCOG08095	3 (-)	K		Predicted transcriptional regulator, contains HTH domain			
435849372	putative transcriptional regulator with CopG/Arc	No	0		plasmid	6775	7119	+		Natronococcus_occultus_SP4_uid184863	NC_019975	plasmid 1		arCOG01008	7 (-)	V	NikR	Transcriptional regulator, CopG/Arc/MetJ family (I	0.00003	pfam01402	Ribbon-helix-helix protein, copG family.
435849373	hypothetical protein Natoc_4370	No	0		plasmid	7265	7645	+		Natronococcus_occultus_SP4_uid184863	NC_019975	plasmid 1		arCOG07766	2	S		Uncharacterized conserved protein			
435849374	hypothetical protein Natoc_4371	No	0		plasmid	7612	7809	+		Natronococcus_occultus_SP4_uid184863	NC_019975	plasmid 1									
435849375	hypothetical protein Natoc_4372	No	0		plasmid	8386	8958	+		Natronococcus_occultus_SP4_uid184863	NC_019975	plasmid 1									
435849376	hypothetical protein Natoc_4373	No	0		plasmid	9331	9558	+		Natronococcus_occultus_SP4_uid184863	NC_019975	plasmid 1									
435849377	hypothetical protein Natoc_4374	No	0		plasmid	9555	12929	+		Natronococcus_occultus_SP4_uid184863	NC_019975	plasmid 1		arCOG06276	3 (-)	L		Primase-polymerase (primpol) domain containing	9E-22	COG4983	Uncharacterized conserved protein
===									8	Natronococcus_occultus_SP4_uid184863	NC_019976	plasmid 2									
435849422	hypothetical protein Natoc_4109	Yes	1		plasmid	33152	34213	+		Natronococcus_occultus_SP4_uid184863	NC_019976	plasmid 2		arCOG06249	3 (-)	S		Uncharacterized conserved protein	0.0000004	pfam09528	Ehrlichia tandem repeat (Ehrlichia_rpt).
435849423	hypothetical protein Natoc_4110	No	0		plasmid	34826	35557	-		Natronococcus_occultus_SP4_uid184863	NC_019976	plasmid 2									
435849424	hypothetical protein Natoc_4111	No	0		plasmid	35569	35691	-		Natronococcus_occultus_SP4_uid184863	NC_019976	plasmid 2									
435849425	hypothetical protein Natoc_4112	No	0		plasmid	36283	36378	+		Natronococcus_occultus_SP4_uid184863	NC_019976	plasmid 2									
435849426	hypothetical protein Natoc_4113	No	0		plasmid	36505	36618	-		Natronococcus_occultus_SP4_uid184863	NC_019976	plasmid 2									
435849427	hypothetical protein Natoc_4115	No	4		plasmid	37079	37825	-		Natronococcus_occultus_SP4_uid184863	NC_019976	plasmid 2		arCOG11073	1	S		Uncharacterized conserved protein			
435849428	hypothetical protein Natoc_4116	No	0		plasmid	37906	38787	-		Natronococcus_occultus_SP4_uid184863	NC_019976	plasmid 2									
435849429	transposase	No	0		plasmid	39494	40129	+		Natronococcus_occultus_SP4_uid184863	NC_019976	plasmid 2		arCOG02134	15 (-)	L		Transposase	2E-15	COG3316	Transposase and inactivated derivatives
===									8	Natronomonas_pharaonis_DSM_2160_uid58435	NC_007426	chromosome									
76801807	hypothetical protein NP2328A	No	0		Virus_Pleolipo	1132794	1133168	+		Natronomonas_pharaonis_DSM_2160_uid58435	NC_007426	chromosome		arCOG09044	5 (-)	R		SWIM zinc finger			
76801808	hypothetical protein NP2330A	No	0		Virus_Pleolipo	1133352	1134032	+		Natronomonas_pharaonis_DSM_2160_uid58435	NC_007426	chromosome									
76801809	hypothetical protein NP2332A	No	0		Virus_Pleolipo	1134221	1134466	+		Natronomonas_pharaonis_DSM_2160_uid58435	NC_007426	chromosome									
76801810	hypothetical protein NP2334A	No	0		Virus_Pleolipo	1134463	1134648	+		Natronomonas_pharaonis_DSM_2160_uid58435	NC_007426	chromosome									
76801811	hypothetical protein NP2336A	No	2		Virus_Pleolipo	1134734	1134934	+		Natronomonas_pharaonis_DSM_2160_uid58435	NC_007426	chromosome									
76801812	hypothetical protein NP2338A	Yes	2		Virus_Pleolipo	1134937	1135521	+		Natronomonas_pharaonis_DSM_2160_uid58435	NC_007426	chromosome									
76801813	hypothetical protein NP2340A	No	1		Virus_Pleolipo	1135521	1135628	+		Natronomonas_pharaonis_DSM_2160_uid58435	NC_007426	chromosome									
76801814	hypothetical protein NP2342A	No	1		Virus_Pleolipo	1135625	1137430	+		Natronomonas_pharaonis_DSM_2160_uid58435	NC_007426	chromosome		arCOG07782	7 (-)	S		Uncharacterized conserved protein			
===									11	Natronomonas_pharaonis_DSM_2160_uid58435	NC_007426	chromosome									
76802257	hypothetical protein NP3234A	No	0	YES	plasmid	1561335	1561478	-		Natronomonas_pharaonis_DSM_2160_uid58435	NC_007426	chromosome									
76802258	hypothetical protein NP3236A	No	0	YES	plasmid	1561909	1562559	-		Natronomonas_pharaonis_DSM_2160_uid58435	NC_007426	chromosome									
76802259	integrase	No	0	YES	plasmid	1562790	1563854	-		Natronomonas_pharaonis_DSM_2160_uid58435	NC_007426	chromosome		arCOG01250	7 (-)	L	XerC	Integrase	2E-10	COG4974	Site-specific recombinase XerD
76802260	hypothetical protein NP3240A	No	0	YES	plasmid	1563851	1564057	-		Natronomonas_pharaonis_DSM_2160_uid58435	NC_007426	chromosome	clust_444								
76802261	hypothetical protein NP3242A	No	0	YES	plasmid	1564047	1564316	-		Natronomonas_pharaonis_DSM_2160_uid58435	NC_007426	chromosome	clust_373								
76802262	hypothetical protein NP3244A	No	0	YES	plasmid	1564313	1564552	-		Natronomonas_pharaonis_DSM_2160_uid58435	NC_007426	chromosome		arCOG06155	6	S		Uncharacterized conserved protein			
76802263	hypothetical protein NP3246A	No	2	YES	plasmid	1564552	1564743	-		Natronomonas_pharaonis_DSM_2160_uid58435	NC_007426	chromosome	clust_463								
76802264	hypothetical protein NP3248A	No	0	YES	plasmid	1564743	1565009	-		Natronomonas_pharaonis_DSM_2160_uid58435	NC_007426	chromosome	clust_377								
76802265	hypothetical protein NP3250A	No	0	YES	plasmid	1565006	1565131	-		Natronomonas_pharaonis_DSM_2160_uid58435	NC_007426	chromosome	clust_505								
76802266	hypothetical protein NP3254A	No	0	YES	plasmid	1565699	1565986	+		Natronomonas_pharaonis_DSM_2160_uid58435	NC_007426	chromosome									
76802267	HNH-type endonuclease	No	0	YES	plasmid	1566035	1566664	+		Natronomonas_pharaonis_DSM_2160_uid58435	NC_007426	chromosome		arCOG03899	2 (-)	V		Predicted restriction endonuclease, HNH family			
===									28	Natronomonas_pharaonis_DSM_2160_uid58435	NC_007426	chromosome									
76802279	lipoprotein	No	0	YES	plasmid	1580988	1581233	-		Natronomonas_pharaonis_DSM_2160_uid58435	NC_007426	chromosome		arCOG03087	3 (-)	R		Predicted RNA binding protein (dsRBD-like fold),	0.000001	COG1724	Predicted RNA binding protein (dsRBD-lik
76802280	hypothetical protein NP3282A	No	0	YES	plasmid	1581235	1581489	-		Natronomonas_pharaonis_DSM_2160_uid58435	NC_007426	chromosome	clust_391								
76802281	hypothetical protein NP3284A	No	0	YES	plasmid	1581761	1582669	-		Natronomonas_pharaonis_DSM_2160_uid58435	NC_007426	chromosome	clust_107								
76802282	hypothetical protein NP3286A	No	0	YES	plasmid	1582672	1583184	-		Natronomonas_pharaonis_DSM_2160_uid58435	NC_007426	chromosome	clust_205								
76802283	hypothetical protein NP3288A	No	0	YES	plasmid	1583174	1583494	-		Natronomonas_pharaonis_DSM_2160_uid58435	NC_007426	chromosome	clust_325								
76802284	hypothetical protein NP3290A	No	2	YES	plasmid	1583544	1583864	+		Natronomonas_pharaonis_DSM_2160_uid58435	NC_007426	chromosome	clust_20								
76802285	hypothetical protein NP3292A	No	2	YES	plasmid	1583868	1584194	+		Natronomonas_pharaonis_DSM_2160_uid58435	NC_007426	chromosome	clust_20								
76802286	hypothetical protein NP3294A	No	2	YES	plasmid	1584248	1584451	+		Natronomonas_pharaonis_DSM_2160_uid58435	NC_007426	chromosome	clust_450								
76802287	hypothetical protein NP3296A	No	3	YES	plasmid	1584457	1584777	+		Natronomonas_pharaonis_DSM_2160_uid58435	NC_007426	chromosome	clust_326								
76802288	hypothetical protein NP3298A	No	0	YES	plasmid	1584770	1585405	+		Natronomonas_pharaonis_DSM_2160_uid58435	NC_007426	chromosome	clust_160								
76802289	hypothetical protein NP3300A	No	2	YES	plasmid	1585402	1585719	+		Natronomonas_pharaonis_DSM_2160_uid58435	NC_007426	chromosome	clust_333								
76802290	hypothetical protein NP3302A	No	2	YES	plasmid	1585712	1586692	+		Natronomonas_pharaonis_DSM_2160_uid58435	NC_007426	chromosome	clust_96								
76802291	hypothetical protein NP3304A	No	2	YES	plasmid	1586694	1586924	+		Natronomonas_pharaonis_DSM_2160_uid58435	NC_007426	chromosome	clust_418								
76802292	hypothetical protein NP3306A	No	2	YES	plasmid	1586926	1587183	+		Natronomonas_pharaonis_DSM_2160_uid58435	NC_007426	chromosome	clust_390								
76802293	hypothetical protein NP3308A	No	0	YES	plasmid	1587180	1587293	+		Natronomonas_pharaonis_DSM_2160_uid58435	NC_007426	chromosome	clust_512								
76802294	hypothetical protein NP3310A	No	0	YES	plasmid	1587286	1587972	+		Natronomonas_pharaonis_DSM_2160_uid58435	NC_007426	chromosome	clust_148								
76802295	hypothetical protein NP3312A	Yes	3	YES	plasmid	1588014	1589714	+		Natronomonas_pharaonis_DSM_2160_uid58435	NC_007426	chromosome	clust_70								
76802296	hypothetical protein NP3314A	Yes	3	YES	plasmid	1589711	1590118	+		Natronomonas_pharaonis_DSM_2160_uid58435	NC_007426	chromosome	clust_256								
76802297	hypothetical protein NP3316A	No	3	YES	plasmid	1590115	1590459	+		Natronomonas_pharaonis_DSM_2160_uid58435	NC_007426	chromosome	clust_308								
76802298	hypothetical protein NP3318A	No	2	YES	plasmid	1590483	1590812	+		Natronomonas_pharaonis_DSM_2160_uid58435	NC_007426	chromosome		arCOG06171	3	S		Uncharacterized conserved protein			
76802299	hypothetical protein NP3320A	No	0	YES	plasmid	1590826	1592229	+		Natronomonas_pharaonis_DSM_2160_uid58435	NC_007426	chromosome		arCOG09042	7	Y		Viral protein, VP1 family			
76802300	hypothetical protein NP3322A	No	0	YES	plasmid	1592226	1595336	+		Natronomonas_pharaonis_DSM_2160_uid58435	NC_007426	chromosome	clust_65						0.00000004	pfam03343	SART-1 family. SART-1 is a protein invol
76802301	hypothetical protein NP3324A	No	2	YES	plasmid	1595379	1595591	+		Natronomonas_pharaonis_DSM_2160_uid58435	NC_007426	chromosome	clust_437								
76802302	hypothetical protein NP3326A	No	0	YES	plasmid	1595730	1595918	-		Natronomonas_pharaonis_DSM_2160_uid58435	NC_007426	chromosome	clust_466						0.00005	pfam11931	Domain of unknown function (DUF3449). Th
76802303	hypothetical protein NP3328A	No	0	YES	plasmid	1596118	1596519	+		Natronomonas_pharaonis_DSM_2160_uid58435	NC_007426	chromosome	clust_264								
76802304	hypothetical protein NP3330A	No	0		plasmid	1596465	1598126	-		Natronomonas_pharaonis_DSM_2160_uid58435	NC_007426	chromosome		arCOG06319	6 (-)	R		Protein containing HTH domain			
76802305	hypothetical protein NP3332A	No	0		plasmid	1598336	1598647	-		Natronomonas_pharaonis_DSM_2160_uid58435	NC_007426	chromosome	clust_341								
76802306	hypothetical protein NP3334A	No	0		plasmid	1598647	1599138	-		Natronomonas_pharaonis_DSM_2160_uid58435	NC_007426	chromosome	clust_217								
===									6	Natronomonas_pharaonis_DSM_2160_uid58435	NC_007426	chromosome									
76802829	hypothetical protein NP4390A	Yes	0			2140305	2141045	+		Natronomonas_pharaonis_DSM_2160_uid58435	NC_007426	chromosome		arCOG06249	3 (-)	S		Uncharacterized conserved protein			
76802830	cell surface glycoprotein	No	0			2141102	2142190	-		Natronomonas_pharaonis_DSM_2160_uid58435	NC_007426	chromosome							0.000000003	pfam05887	Procyclic acidic repetitive protein (PAR
76802831	hypothetical protein NP4394A	No	4			2142279	2142764	-		Natronomonas_pharaonis_DSM_2160_uid58435	NC_007426	chromosome									
76802832	hypothetical protein NP4396A	No	4			2142757	2143185	-		Natronomonas_pharaonis_DSM_2160_uid58435	NC_007426	chromosome		arCOG06302	1	S		Uncharacterized conserved protein			
76802833	hypothetical protein NP4400A	No	1			2143567	2143719	+		Natronomonas_pharaonis_DSM_2160_uid58435	NC_007426	chromosome									
76802834	zinc transporter	No	8			2143720	2144520	-		Natronomonas_pharaonis_DSM_2160_uid58435	NC_007426	chromosome		arCOG00576	1 (-)	P		Predicted divalent heavy-metal cations transporte	4E-31	COG0428	Predicted divalent heavy-metal cations t
===									5	Natronomonas_pharaonis_DSM_2160_uid58435	NC_007427	plasmid PL131									
76803398	hypothetical protein NP6070A	No	0		plasmid	29617	29808	+		Natronomonas_pharaonis_DSM_2160_uid58435	NC_007427	plasmid PL131									
76803399	hypothetical protein NP6072A	Yes	0		plasmid	29956	30375	+		Natronomonas_pharaonis_DSM_2160_uid58435	NC_007427	plasmid PL131									
76803400	hypothetical protein NP6074A	No	0		plasmid	30907	32301	-		Natronomonas_pharaonis_DSM_2160_uid58435	NC_007427	plasmid PL131		arCOG06436	1	S		Uncharacterized conserved protein, YdiU/UPF00	2E-100	COG0397	Uncharacterized conserved protein
76803401	hypothetical protein NP6076A	No	4		plasmid	32587	33123	+		Natronomonas_pharaonis_DSM_2160_uid58435	NC_007427	plasmid PL131		arCOG02861	1 (-)	S		Predicted membrane protein, DoxD family			
76803402	hypothetical protein NP6078A	No	2		plasmid	33134	33421	+		Natronomonas_pharaonis_DSM_2160_uid58435	NC_007427	plasmid PL131		arCOG09000	1	S		Uncharacterized conserved protein			
===									35	Natronomonas_pharaonis_DSM_2160_uid58435	NC_007428	plasmid PL23									
76803319	lipoprotein	No	0	YES	plasmid	2018	2263	-		Natronomonas_pharaonis_DSM_2160_uid58435	NC_007428	plasmid PL23		arCOG03087	3 (-)	R		Predicted RNA binding protein (dsRBD-like fold),	0.000001	COG1724	Predicted RNA binding protein (dsRBD-lik
76803320	hypothetical protein NP7006A	No	0	YES	plasmid	2265	2519	-		Natronomonas_pharaonis_DSM_2160_uid58435	NC_007428	plasmid PL23	clust_391								
76803321	hypothetical protein NP7008A	No	0	YES	plasmid	2791	3699	-		Natronomonas_pharaonis_DSM_2160_uid58435	NC_007428	plasmid PL23	clust_107								
76803322	hypothetical protein NP7010A	No	0	YES	plasmid	3702	4214	-		Natronomonas_pharaonis_DSM_2160_uid58435	NC_007428	plasmid PL23	clust_205								
76803323	hypothetical protein NP7012A	No	0	YES	plasmid	4204	4524	-		Natronomonas_pharaonis_DSM_2160_uid58435	NC_007428	plasmid PL23	clust_325								
76803324	hypothetical protein NP7014A	No	2	YES	plasmid	4574	4894	+		Natronomonas_pharaonis_DSM_2160_uid58435	NC_007428	plasmid PL23	clust_20								
76803325	hypothetical protein NP7016A	No	2	YES	plasmid	4898	5224	+		Natronomonas_pharaonis_DSM_2160_uid58435	NC_007428	plasmid PL23	clust_20								
76803326	hypothetical protein NP7018A	No	2	YES	plasmid	5278	5481	+		Natronomonas_pharaonis_DSM_2160_uid58435	NC_007428	plasmid PL23	clust_450								
76803327	hypothetical protein NP7020A	No	3	YES	plasmid	5487	5807	+		Natronomonas_pharaonis_DSM_2160_uid58435	NC_007428	plasmid PL23	clust_326								
76803328	hypothetical protein NP7022A	No	0	YES	plasmid	5800	6435	+		Natronomonas_pharaonis_DSM_2160_uid58435	NC_007428	plasmid PL23	clust_160								
76803329	hypothetical protein NP7024A	No	2	YES	plasmid	6432	6749	+		Natronomonas_pharaonis_DSM_2160_uid58435	NC_007428	plasmid PL23	clust_333								
76803330	hypothetical protein NP7026A	No	2	YES	plasmid	6742	7722	+		Natronomonas_pharaonis_DSM_2160_uid58435	NC_007428	plasmid PL23	clust_96								
76803331	hypothetical protein NP7028A	No	2	YES	plasmid	7724	7954	+		Natronomonas_pharaonis_DSM_2160_uid58435	NC_007428	plasmid PL23	clust_418								
76803332	hypothetical protein NP7030A	No	2	YES	plasmid	7956	8213	+		Natronomonas_pharaonis_DSM_2160_uid58435	NC_007428	plasmid PL23	clust_390								
76803333	hypothetical protein NP7032A	No	0	YES	plasmid	8210	8323	+		Natronomonas_pharaonis_DSM_2160_uid58435	NC_007428	plasmid PL23	clust_512								
76803334	hypothetical protein NP7034A	No	0	YES	plasmid	8316	9002	+		Natronomonas_pharaonis_DSM_2160_uid58435	NC_007428	plasmid PL23	clust_148								
76803335	hypothetical protein NP7036A	Yes	3	YES	plasmid	9044	10744	+		Natronomonas_pharaonis_DSM_2160_uid58435	NC_007428	plasmid PL23	clust_70								
76803336	hypothetical protein NP7038A	Yes	3	YES	plasmid	10741	11148	+		Natronomonas_pharaonis_DSM_2160_uid58435	NC_007428	plasmid PL23	clust_256								
76803337	hypothetical protein NP7040A	No	3	YES	plasmid	11145	11489	+		Natronomonas_pharaonis_DSM_2160_uid58435	NC_007428	plasmid PL23	clust_308								
76803338	hypothetical protein NP7042A	No	2	YES	plasmid	11513	11842	+		Natronomonas_pharaonis_DSM_2160_uid58435	NC_007428	plasmid PL23		arCOG06171	3	S		Uncharacterized conserved protein			
76803339	hypothetical protein NP7044A	No	0	YES	plasmid	11856	13259	+		Natronomonas_pharaonis_DSM_2160_uid58435	NC_007428	plasmid PL23		arCOG09042	7	Y		Viral protein, VP1 family			
76803340	hypothetical protein NP7046A	No	0	YES	plasmid	13256	16366	+		Natronomonas_pharaonis_DSM_2160_uid58435	NC_007428	plasmid PL23	clust_65						0.00000004	pfam03343	SART-1 family. SART-1 is a protein invol
76803341	hypothetical protein NP7048A	No	2	YES	plasmid	16409	16621	+		Natronomonas_pharaonis_DSM_2160_uid58435	NC_007428	plasmid PL23	clust_437								
76803342	hypothetical protein NP7050A	No	0	YES	plasmid	16760	16948	-		Natronomonas_pharaonis_DSM_2160_uid58435	NC_007428	plasmid PL23	clust_466						0.00005	pfam11931	Domain of unknown function (DUF3449). Th
76803343	hypothetical protein NP7052A	No	0	YES	plasmid	17148	17549	+		Natronomonas_pharaonis_DSM_2160_uid58435	NC_007428	plasmid PL23	clust_264								
76803344	hypothetical protein NP7054A	No	0		plasmid	17495	19156	-		Natronomonas_pharaonis_DSM_2160_uid58435	NC_007428	plasmid PL23		arCOG06319	6 (-)	R		Protein containing HTH domain			
76803345	hypothetical protein NP7056A	No	0	YES	plasmid	19366	19677	-		Natronomonas_pharaonis_DSM_2160_uid58435	NC_007428	plasmid PL23	clust_341								
76803346	hypothetical protein NP7058A	No	0	YES	plasmid	19677	20168	-		Natronomonas_pharaonis_DSM_2160_uid58435	NC_007428	plasmid PL23	clust_217								
76803347	integrase	No	0	YES	plasmid	20656	21720	-		Natronomonas_pharaonis_DSM_2160_uid58435	NC_007428	plasmid PL23		arCOG01250	7 (-)	L	XerC	Integrase	2E-10	COG4974	Site-specific recombinase XerD
76803348	hypothetical protein NP7064A	No	0	YES	plasmid	21717	21923	-		Natronomonas_pharaonis_DSM_2160_uid58435	NC_007428	plasmid PL23	clust_444								
76803349	hypothetical protein NP7066A	No	0	YES	plasmid	21913	22182	-		Natronomonas_pharaonis_DSM_2160_uid58435	NC_007428	plasmid PL23	clust_373								
76803350	hypothetical protein NP7068A	No	0	YES	plasmid	22179	22418	-		Natronomonas_pharaonis_DSM_2160_uid58435	NC_007428	plasmid PL23		arCOG06155	6	S		Uncharacterized conserved protein			
76803351	hypothetical protein NP7070A	No	2	YES	plasmid	22418	22609	-		Natronomonas_pharaonis_DSM_2160_uid58435	NC_007428	plasmid PL23	clust_463								
76803352	hypothetical protein NP7072A	No	0	YES	plasmid	22609	22875	-		Natronomonas_pharaonis_DSM_2160_uid58435	NC_007428	plasmid PL23	clust_377								
76803353	hypothetical protein NP7074A	No	0	YES	plasmid	22872	22997	-		Natronomonas_pharaonis_DSM_2160_uid58435	NC_007428	plasmid PL23	clust_505								
===									5	Nitrosopumilus_maritimus_SCM1_uid58903	NC_010085	chromosome									
161527638	GDSL family lipase	No	1			116426	117607	-		Nitrosopumilus_maritimus_SCM1_uid58903	NC_010085	chromosome									
161527639	carbamoyltransferase	No	0			117608	119311	-		Nitrosopumilus_maritimus_SCM1_uid58903	NC_010085	chromosome		arCOG01188	1 (-)	O		Predicted carbamoyl transferase, NodU family	8E-145	COG2192	Predicted carbamoyl transferase, NodU fa
161527640	hypothetical protein Nmar_0128	No	2			119311	119658	-		Nitrosopumilus_maritimus_SCM1_uid58903	NC_010085	chromosome									
161527641	hypothetical protein Nmar_0129	No	1			119660	119812	-		Nitrosopumilus_maritimus_SCM1_uid58903	NC_010085	chromosome									
161527642	hypothetical protein Nmar_0130	No	0			119962	120735	-		Nitrosopumilus_maritimus_SCM1_uid58903	NC_010085	chromosome									
===									6	Nitrosopumilus_maritimus_SCM1_uid58903	NC_010085	chromosome									
161527962	hypothetical protein Nmar_0454	Yes	1			401255	401536	+		Nitrosopumilus_maritimus_SCM1_uid58903	NC_010085	chromosome									
161527963	hypothetical protein Nmar_0455	No	1			401560	402282	-		Nitrosopumilus_maritimus_SCM1_uid58903	NC_010085	chromosome		arCOG08821	18	S		Uncharacterized conserved protein (expansion in Thaumarchaeota)			
161527964	hypothetical protein Nmar_0456	Yes	3			402319	410613	+		Nitrosopumilus_maritimus_SCM1_uid58903	NC_010085	chromosome		arCOG02565	1 (-)	S		Uncharacterized conserved protein	0.000000003	pfam06933	Special lobe-specific silk protein SSP16
161527965	hypothetical protein Nmar_0457	Yes	1			410757	411020	+		Nitrosopumilus_maritimus_SCM1_uid58903	NC_010085	chromosome									
161527966	hypothetical protein Nmar_0458	No	1			411035	411481	-		Nitrosopumilus_maritimus_SCM1_uid58903	NC_010085	chromosome		arCOG08658	1	S		Uncharacterized conserved protein			
161527967	hypothetical protein Nmar_0459	Yes	0			411535	412170	-		Nitrosopumilus_maritimus_SCM1_uid58903	NC_010085	chromosome		arCOG08819	6	S		Uncharacterized conserved protein (expansion in Thaumarchaeota)			
===									7	Nitrosopumilus_maritimus_SCM1_uid58903	NC_010085	chromosome									
161528256	elongation factor EF-2	No	0			675576	677768	+		Nitrosopumilus_maritimus_SCM1_uid58903	NC_010085	chromosome		arCOG01559	2 (-)	J	FusA	Translation elongation factor G, EF-G (GTPase)	1E-105	COG0480	Translation elongation factors (GTPases)
161528257	hypothetical protein Nmar_0749	No	0			677860	678438	+		Nitrosopumilus_maritimus_SCM1_uid58903	NC_010085	chromosome		arCOG10540	4	S		Uncharacterized conserved protein			
161528258	hypothetical protein Nmar_0750	No	0			678482	678712	+		Nitrosopumilus_maritimus_SCM1_uid58903	NC_010085	chromosome		arCOG10541	3	S		Uncharacterized conserved protein			
161528259	hypothetical protein Nmar_0751	No	0			678747	678977	+		Nitrosopumilus_maritimus_SCM1_uid58903	NC_010085	chromosome		arCOG10542	3	S		Uncharacterized conserved protein			
161528260	hypothetical protein Nmar_0752	No	0			678980	679405	-		Nitrosopumilus_maritimus_SCM1_uid58903	NC_010085	chromosome		arCOG10543	3	S		Uncharacterized conserved protein			
161528261	hypothetical protein Nmar_0753	No	2			679479	679691	-		Nitrosopumilus_maritimus_SCM1_uid58903	NC_010085	chromosome		arCOG10544	3	S		Uncharacterized conserved protein			
161528262	hypothetical protein Nmar_0754	No	0			679820	681301	+		Nitrosopumilus_maritimus_SCM1_uid58903	NC_010085	chromosome		arCOG00371	3 (-)	D	Smc	Chromosome segregation ATPase	1E-21	pfam02463	RecF/RecN/SMC N terminal domain. This do
===									6	Nitrosopumilus_maritimus_SCM1_uid58903	NC_010085	chromosome									
161528265	hypothetical protein Nmar_0757	No	0	YES		682447	682770	+		Nitrosopumilus_maritimus_SCM1_uid58903	NC_010085	chromosome	clust_42								
161528266	hypothetical protein Nmar_0758	No	0	YES		682965	683177	-		Nitrosopumilus_maritimus_SCM1_uid58903	NC_010085	chromosome	clust_59								
161528267	hypothetical protein Nmar_0759	No	0	YES		683694	683966	+		Nitrosopumilus_maritimus_SCM1_uid58903	NC_010085	chromosome		arCOG04290	2 (-)	R		PIN-domain and Zn ribbon			
161528268	hypothetical protein Nmar_0760	No	0	YES		683963	684529	-		Nitrosopumilus_maritimus_SCM1_uid58903	NC_010085	chromosome	clust_186								
161528269	hypothetical protein Nmar_0761	No	0	YES		684539	684772	-		Nitrosopumilus_maritimus_SCM1_uid58903	NC_010085	chromosome		arCOG10545	2	S		Uncharacterized conserved protein			
161528270	hypothetical protein Nmar_0762	No	0	YES		684848	685111	+		Nitrosopumilus_maritimus_SCM1_uid58903	NC_010085	chromosome		arCOG10546	2	S		Uncharacterized conserved protein			
===									7	Nitrosopumilus_maritimus_SCM1_uid58903	NC_010085	chromosome									
161528294	hypothetical protein Nmar_0786	No	0	YES		702588	702908	+		Nitrosopumilus_maritimus_SCM1_uid58903	NC_010085	chromosome	clust_327								
161528295	pyridoxamine 5'-phosphate oxidase-related FM	No	0			702901	703305	+		Nitrosopumilus_maritimus_SCM1_uid58903	NC_010085	chromosome		arCOG00516	6 (-)	R		Predicted flavin-nucleotide-binding protein			
161528296	histone protein	Yes	0			703382	703780	-		Nitrosopumilus_maritimus_SCM1_uid58903	NC_010085	chromosome	clust_41						6E-16	pfam07382	Histone H1-like nucleoprotein HC2. This
161528297	hypothetical protein Nmar_0789	No	1			703882	704370	-		Nitrosopumilus_maritimus_SCM1_uid58903	NC_010085	chromosome		arCOG10550	3	S		Uncharacterized conserved protein			
161528298	hypothetical protein Nmar_0790	Yes	1			704520	705113	+		Nitrosopumilus_maritimus_SCM1_uid58903	NC_010085	chromosome									
161528299	hypothetical protein Nmar_0791	No	0			705832	706281	-		Nitrosopumilus_maritimus_SCM1_uid58903	NC_010085	chromosome									
161528300	hypothetical protein Nmar_0792	No	0			706645	706893	-		Nitrosopumilus_maritimus_SCM1_uid58903	NC_010085	chromosome		arCOG08711	1 (-)	S		Zn finger protein			
===									21	Nitrosopumilus_maritimus_SCM1_uid58903	NC_010085	chromosome									
161528397	hypothetical protein Nmar_0889	No	0			780605	780922	+		Nitrosopumilus_maritimus_SCM1_uid58903	NC_010085	chromosome		arCOG10559	2 (-)	L		GIY-YIG domain containing nuclease			
161528398	hypothetical protein Nmar_0890	No	1			780986	781231	+		Nitrosopumilus_maritimus_SCM1_uid58903	NC_010085	chromosome	clust_58								
161528399	hypothetical protein Nmar_0891	No	0			781540	781806	+		Nitrosopumilus_maritimus_SCM1_uid58903	NC_010085	chromosome		arCOG10560	3	S		Uncharacterized conserved protein			
161528400	hypothetical protein Nmar_0892	No	0			781999	782535	-		Nitrosopumilus_maritimus_SCM1_uid58903	NC_010085	chromosome		arCOG10561	3	S		Uncharacterized conserved protein			
161528401	hypothetical protein Nmar_0893	No	0			782577	782846	-		Nitrosopumilus_maritimus_SCM1_uid58903	NC_010085	chromosome									
161528402	hypothetical protein Nmar_0894	No	0			783332	783580	-		Nitrosopumilus_maritimus_SCM1_uid58903	NC_010085	chromosome		arCOG10562	3	S		Uncharacterized conserved protein			
161528403	hypothetical protein Nmar_0895	No	0			783883	784473	+		Nitrosopumilus_maritimus_SCM1_uid58903	NC_010085	chromosome		arCOG10563	3	S		Uncharacterized conserved protein			
161528404	hypothetical protein Nmar_0896	No	0			784509	784838	+		Nitrosopumilus_maritimus_SCM1_uid58903	NC_010085	chromosome		arCOG10564	3	S		Uncharacterized conserved protein			
161528405	hypothetical protein Nmar_0897	No	0			785551	785784	+		Nitrosopumilus_maritimus_SCM1_uid58903	NC_010085	chromosome		arCOG07442	3 (-)	S		Uncharacterized conserved protein			
161528406	hypothetical protein Nmar_0898	No	0			785852	786319	+		Nitrosopumilus_maritimus_SCM1_uid58903	NC_010085	chromosome		arCOG10565	3	S		Uncharacterized conserved protein			
161528407	hypothetical protein Nmar_0899	No	0			786988	787221	-		Nitrosopumilus_maritimus_SCM1_uid58903	NC_010085	chromosome		arCOG10566	1	S		Uncharacterized conserved protein			
161528408	hypothetical protein Nmar_0900	No	0			787291	787641	+		Nitrosopumilus_maritimus_SCM1_uid58903	NC_010085	chromosome		arCOG07348	2 (-)	S		Uncharacterized conserved protein			
161528409	hypothetical protein Nmar_0901	No	0			788095	788304	+		Nitrosopumilus_maritimus_SCM1_uid58903	NC_010085	chromosome	clust_441								
161528410	hypothetical protein Nmar_0902	No	0			788330	788872	+		Nitrosopumilus_maritimus_SCM1_uid58903	NC_010085	chromosome	clust_196								
161528411	hypothetical protein Nmar_0903	No	0			788897	789313	-		Nitrosopumilus_maritimus_SCM1_uid58903	NC_010085	chromosome		arCOG08754	3 (-)	S		Uncharacterized conserved protein			
161528412	hypothetical protein Nmar_0904	No	0			789419	789718	+		Nitrosopumilus_maritimus_SCM1_uid58903	NC_010085	chromosome	clust_352								
161528413	hypothetical protein Nmar_0905	Yes	1			789744	790391	-		Nitrosopumilus_maritimus_SCM1_uid58903	NC_010085	chromosome									
161528414	hypothetical protein Nmar_0906	No	0			791128	791799	-		Nitrosopumilus_maritimus_SCM1_uid58903	NC_010085	chromosome		arCOG00787	3 (-)	L		UvrD/Rep family helicase fused to exonuclease family domain			
161528415	hypothetical protein Nmar_0907	No	0			791835	792236	-		Nitrosopumilus_maritimus_SCM1_uid58903	NC_010085	chromosome	clust_265								
161528416	hypothetical protein Nmar_0908	No	0			792290	792487	+		Nitrosopumilus_maritimus_SCM1_uid58903	NC_010085	chromosome	clust_458								
161528417	hypothetical protein Nmar_0909	No	0			792948	793280	+		Nitrosopumilus_maritimus_SCM1_uid58903	NC_010085	chromosome	clust_314								
===									5	Nitrosopumilus_maritimus_SCM1_uid58903	NC_010085	chromosome									
161528668	Gp37Gp68 family protein	No	0			1061437	1062276	+		Nitrosopumilus_maritimus_SCM1_uid58903	NC_010085	chromosome							1E-73	COG4422	Bacteriophage protein gp37
161528669	hypothetical protein Nmar_1161	No	1			1062348	1063718	-		Nitrosopumilus_maritimus_SCM1_uid58903	NC_010085	chromosome		arCOG02926	8 (-)	C	PetE	Plastocyanin			
161528670	hypothetical protein Nmar_1162	No	0			1064164	1064547	-		Nitrosopumilus_maritimus_SCM1_uid58903	NC_010085	chromosome									
161528671	hypothetical protein Nmar_1163	No	2			1064544	1064774	-		Nitrosopumilus_maritimus_SCM1_uid58903	NC_010085	chromosome									
161528672	hypothetical protein Nmar_1164	No	0			1065590	1066066	-		Nitrosopumilus_maritimus_SCM1_uid58903	NC_010085	chromosome									
===									7	Nitrosopumilus_maritimus_SCM1_uid58903	NC_010085	chromosome									
161528869	hypothetical protein Nmar_1361	No	0	YES		1247960	1248259	-		Nitrosopumilus_maritimus_SCM1_uid58903	NC_010085	chromosome		arCOG10582	2	S		Uncharacterized conserved protein			
161528870	hypothetical protein Nmar_1362	No	0	YES		1248624	1248965	+		Nitrosopumilus_maritimus_SCM1_uid58903	NC_010085	chromosome		arCOG03067	3 (-)	K	ArsR	Transcriptional regulator containing HTH domain, ArsR family			
161528871	hypothetical protein Nmar_1363	No	2	YES		1249342	1250802	-		Nitrosopumilus_maritimus_SCM1_uid58903	NC_010085	chromosome	clust_75								
161528872	hypothetical protein Nmar_1364	No	0	YES		1251121	1251375	-		Nitrosopumilus_maritimus_SCM1_uid58903	NC_010085	chromosome		arCOG11049	2	S		Uncharacterized conserved protein			
161528873	hypothetical protein Nmar_1365	No	1	YES		1251727	1252203	+		Nitrosopumilus_maritimus_SCM1_uid58903	NC_010085	chromosome		arCOG11327	1	S		Uncharacterized conserved protein			
161528874	methyltransferase type 11	No	0			1252200	1253066	-		Nitrosopumilus_maritimus_SCM1_uid58903	NC_010085	chromosome		arCOG01791	2 (-)	Q		SAM-dependent methyltransferase			
161528875	hypothetical protein Nmar_1367	No	0	YES		1253150	1254346	+		Nitrosopumilus_maritimus_SCM1_uid58903	NC_010085	chromosome		arCOG08650	1	S		Uncharacterized conserved protein			
===									6	Nitrosopumilus_maritimus_SCM1_uid58903	NC_010085	chromosome									
161528880	ArsR family transcriptional regulator	No	0	YES		1257597	1257926	+		Nitrosopumilus_maritimus_SCM1_uid58903	NC_010085	chromosome		arCOG01681	1 (-)	K	ArsR	Transcriptional regulator containing HTH domain,	2E-12	COG0640	Predicted transcriptional regulators
161528881	hypothetical protein Nmar_1373	No	3	YES		1257999	1258292	+		Nitrosopumilus_maritimus_SCM1_uid58903	NC_010085	chromosome									
161528882	hypothetical protein Nmar_1374	No	0	YES		1258585	1258893	-		Nitrosopumilus_maritimus_SCM1_uid58903	NC_010085	chromosome									
161528883	hypothetical protein Nmar_1375	No	0	YES		1258902	1259879	-		Nitrosopumilus_maritimus_SCM1_uid58903	NC_010085	chromosome									
161528884	hypothetical protein Nmar_1376	No	0	YES		1260107	1261366	+		Nitrosopumilus_maritimus_SCM1_uid58903	NC_010085	chromosome									
161528885	hypothetical protein Nmar_1377	No	0	YES		1261372	1262889	-		Nitrosopumilus_maritimus_SCM1_uid58903	NC_010085	chromosome		arCOG01245	15 (-)	L	XerC	Integrase			
===									5	Nitrosopumilus_maritimus_SCM1_uid58903	NC_010085	chromosome									
161528941	hypothetical protein Nmar_1433	Yes	1			1304693	1304905	-		Nitrosopumilus_maritimus_SCM1_uid58903	NC_010085	chromosome									
161528942	hypothetical protein Nmar_1434	No	1			1304938	1305462	-		Nitrosopumilus_maritimus_SCM1_uid58903	NC_010085	chromosome									
161528943	hypothetical protein Nmar_1435	No	1			1305440	1306402	-		Nitrosopumilus_maritimus_SCM1_uid58903	NC_010085	chromosome	clust_101								
161528944	hypothetical protein Nmar_1436	Yes	2			1306399	1308189	-		Nitrosopumilus_maritimus_SCM1_uid58903	NC_010085	chromosome		arCOG03383	1 (-)	U	TolB	Periplasmic component of the Tol biopolymer tra	0.000007	COG0823	Periplasmic component of the Tol biopoly
161528945	hypothetical protein Nmar_1437	Yes	2			1308186	1309121	-		Nitrosopumilus_maritimus_SCM1_uid58903	NC_010085	chromosome									
===									6	Nitrosopumilus_maritimus_SCM1_uid58903	NC_010085	chromosome									
161528965	methyltransferase type 11	No	0			1326250	1327284	-		Nitrosopumilus_maritimus_SCM1_uid58903	NC_010085	chromosome		arCOG01778	1 (-)	Q		SAM-dependent methyltransferase	4E-20	pfam13847	Methyltransferase domain. This family ap
161528966	hypothetical protein Nmar_1458	Yes	1			1327457	1327741	-		Nitrosopumilus_maritimus_SCM1_uid58903	NC_010085	chromosome									
161528967	hypothetical protein Nmar_1459	No	1			1327840	1328232	-		Nitrosopumilus_maritimus_SCM1_uid58903	NC_010085	chromosome									
161528968	hypothetical protein Nmar_1460	No	1			1328229	1329227	-		Nitrosopumilus_maritimus_SCM1_uid58903	NC_010085	chromosome									
161528969	hypothetical protein Nmar_1461	No	0			1329228	1329881	-		Nitrosopumilus_maritimus_SCM1_uid58903	NC_010085	chromosome									
161528970	hypothetical protein Nmar_1462	Yes	1			1329980	1331845	-		Nitrosopumilus_maritimus_SCM1_uid58903	NC_010085	chromosome	arCOG08777,a	arCOG08777	3 (-)	S		Uncharacterized conserved protein			
===									7	Picrophilus_torridus_DSM_9790_uid58041	NC_005877	chromosome									
48477093	O-succinylbenzoate-CoA synthase	No	0			23451	24539	-		Picrophilus_torridus_DSM_9790_uid58041	NC_005877	chromosome		arCOG01168	1 (-)	M		L-alanine-DL-glutamate epimerase or related enz	5E-38	COG4948	L-alanine-DL-glutamate epimerase and rel
48477094	hypothetical protein PTO0022	No	0	YES		24615	25811	+		Picrophilus_torridus_DSM_9790_uid58041	NC_005877	chromosome		arCOG06950	1	S		Uncharacterized conserved protein	1E-94	pfam07075	Protein of unknown function (DUF1343). T
48477095	hypothetical protein PTO0023	No	0	YES		25815	26321	-		Picrophilus_torridus_DSM_9790_uid58041	NC_005877	chromosome		arCOG08351	3	S		Uncharacterized conserved protein, YdcF family			
48477096	hypothetical protein PTO0024	No	0	YES		26538	27236	-		Picrophilus_torridus_DSM_9790_uid58041	NC_005877	chromosome									
48477097	hypothetical protein PTO0025	No	0	YES		27316	27996	-		Picrophilus_torridus_DSM_9790_uid58041	NC_005877	chromosome									
48477098	hypothetical protein PTO0026	No	3	YES		28028	28360	-		Picrophilus_torridus_DSM_9790_uid58041	NC_005877	chromosome		arCOG06951	3	S		Uncharacterized conserved protein			
48477099	hypothetical protein PTO0027	No	0	YES		30695	31348	-		Picrophilus_torridus_DSM_9790_uid58041	NC_005877	chromosome		arCOG05102	3 (-)	V	McrC	McrBC 5-methylcytosine restriction system comp	0.000000002	pfam10117	McrBC 5-methylcytosine restriction syste
===									14	Picrophilus_torridus_DSM_9790_uid58041	NC_005877	chromosome									
48477421	hypothetical protein PTO0349	No	3			384491	385129	+		Picrophilus_torridus_DSM_9790_uid58041	NC_005877	chromosome		arCOG02206	1 (-)	S		Uncharacterized conserved membrane protein			
48477422	hypothetical protein PTO0350	No	0			385373	385630	-		Picrophilus_torridus_DSM_9790_uid58041	NC_005877	chromosome									
48477423	hypothetical protein PTO0351	No	0			385627	385866	-		Picrophilus_torridus_DSM_9790_uid58041	NC_005877	chromosome									
48477424	hypothetical protein PTO0352	No	0			385832	386290	-		Picrophilus_torridus_DSM_9790_uid58041	NC_005877	chromosome									
48477425	hypothetical protein PTO0353	No	0			386287	386811	-		Picrophilus_torridus_DSM_9790_uid58041	NC_005877	chromosome									
48477426	hypothetical protein PTO0354	No	0			386813	387193	-		Picrophilus_torridus_DSM_9790_uid58041	NC_005877	chromosome									
48477427	hypothetical protein PTO0355	No	0			387190	387465	-		Picrophilus_torridus_DSM_9790_uid58041	NC_005877	chromosome		arCOG07965	1	S		Zn-finger protein			
48477428	virulence-associated protein E	No	0			387698	390712	-		Picrophilus_torridus_DSM_9790_uid58041	NC_005877	chromosome	arCOG06915,a	arCOG06915	3 (-)	L		Primase-polymerase (primpol) domain	6E-14	pfam09250	Bifunctional DNA primase/polymerase, N-t ; Phage associated DNA primase
48477429	hypothetical protein PTO0357	No	0			391504	392793	+		Picrophilus_torridus_DSM_9790_uid58041	NC_005877	chromosome									
48477430	hypothetical protein PTO0358	No	2			394309	394830	+		Picrophilus_torridus_DSM_9790_uid58041	NC_005877	chromosome									
48477431	hypothetical protein PTO0359	No	0			394832	395068	-		Picrophilus_torridus_DSM_9790_uid58041	NC_005877	chromosome									
48477432	hypothetical protein PTO0360	No	0			395261	395455	+		Picrophilus_torridus_DSM_9790_uid58041	NC_005877	chromosome									
48477433	hypothetical protein PTO0361	No	3			395452	395742	+		Picrophilus_torridus_DSM_9790_uid58041	NC_005877	chromosome									
48477434	DNA integration/recombination/invertion protei	No	0			395745	397004	-		Picrophilus_torridus_DSM_9790_uid58041	NC_005877	chromosome		arCOG01242	14 (-)	L	XerC	Integrase	1E-15	pfam00589	Phage integrase family. Members of this
===									5	Picrophilus_torridus_DSM_9790_uid58041	NC_005877	chromosome									
48478207	hypothetical protein PTO1135	No	2			1138105	1138296	+		Picrophilus_torridus_DSM_9790_uid58041	NC_005877	chromosome									
48478208	hypothetical protein PTO1136	No	3			1138298	1138648	+		Picrophilus_torridus_DSM_9790_uid58041	NC_005877	chromosome		arCOG05384	1	S		Uncharacterized conserved protein			
48478209	hypothetical protein PTO1137	No	2			1138645	1138866	+		Picrophilus_torridus_DSM_9790_uid58041	NC_005877	chromosome		arCOG05385	1	S		Uncharacterized conserved protein			
48478210	isomerizing glucosamine--fructose-6-phosphat	No	0			1138925	1139959	+		Picrophilus_torridus_DSM_9790_uid58041	NC_005877	chromosome		arCOG00057	3 (-)	M	GlmS	Glucosamine 6-phosphate synthetase	4E-25	COG0449	Glucosamine 6-phosphate synthetase, cont
48478211	hypothetical protein PTO1139	No	3			1140015	1140308	+		Picrophilus_torridus_DSM_9790_uid58041	NC_005877	chromosome									
===									5	Pyrobaculum_1860_uid82379	NC_016645	chromosome									
374325601	hypothetical protein P186_0077	No	0			75742	76698	-		Pyrobaculum_1860_uid82379	NC_016645	chromosome									
374325602	hypothetical protein P186_0078	No	0			76670	77134	-		Pyrobaculum_1860_uid82379	NC_016645	chromosome									
374325603	hypothetical protein P186_0080	No	0			77820	78203	+		Pyrobaculum_1860_uid82379	NC_016645	chromosome		arCOG01075	2 (-)	F		NUDIX family hydrolase	4E-11	COG1051	ADP-ribose pyrophosphatase
374325604	hypothetical protein P186_0081	No	4			78208	78684	+		Pyrobaculum_1860_uid82379	NC_016645	chromosome									
374325605	hypothetical protein P186_0082	No	5			78659	79240	+		Pyrobaculum_1860_uid82379	NC_016645	chromosome									
===									7	Pyrobaculum_1860_uid82379	NC_016645	chromosome									
===																				

374325639	FeS assembly ATPase SufC	No	0		108795	109568	-		Pyrobaculum_1860_uid82379	NC_016645	chromosome		arCOG04236	1 (-)	O	sufC	Cysteine desulfurase activator ATPase	5E-73	COG0396	Cysteine desulfurase activator ATPase
374325640	hypothetical protein P186_0117	No	0		109781	109921	+		Pyrobaculum_1860_uid82379	NC_016645	chromosome									
374325641	hypothetical protein P186_0118	Yes	1		109985	110794	-		Pyrobaculum_1860_uid82379	NC_016645	chromosome	clust_121								
374325642	hypothetical protein P186_0119	Yes	1		110955	111440	-		Pyrobaculum_1860_uid82379	NC_016645	chromosome									
374325643	hypothetical protein P186_0120	No	0		112186	112335	+		Pyrobaculum_1860_uid82379	NC_016645	chromosome									
374325644	hypothetical protein P186_0121	No	0		112355	112480	+		Pyrobaculum_1860_uid82379	NC_016645	chromosome									
374325645	FeS assembly protein SufB	No	0		112507	113922	+		Pyrobaculum_1860_uid82379	NC_016645	chromosome		arCOG01715	1 (-)	O	sufB	Cysteine desulfurase activator SufB	2E-78	COG0719	Cysteine desulfurase activator SufB
===								6	Pyrobaculum_1860_uid82379	NC_016645	chromosome									
374325676	PaREP2b	No	0		148688	149449	-		Pyrobaculum_1860_uid82379	NC_016645	chromosome		arCOG07952	4 (-)	S		PaRep2b protein, some fused to other mobile dc	4E-19	pfam07775	PaRep2b protein. This is a family of pro
374325677	hypothetical protein P186_0164	No	0		149496	149630	+		Pyrobaculum_1860_uid82379	NC_016645	chromosome									
374325678	hypothetical protein P186_0165	Yes	0		149969	152113	+		Pyrobaculum_1860_uid82379	NC_016645	chromosome		arCOG07691	7	S		Uncharacterized conserved protein			
374325679	hypothetical protein P186_0166	No	4		152252	152656	+		Pyrobaculum_1860_uid82379	NC_016645	chromosome		arCOG09771	3	S		Uncharacterized conserved protein			
374325680	hypothetical protein P186_0167	No	1		153314	153442	+		Pyrobaculum_1860_uid82379	NC_016645	chromosome									
374325681	hypothetical protein P186_0168	No	0		153509	153844	+		Pyrobaculum_1860_uid82379	NC_016645	chromosome		arCOG07957	3 (-)	R		Uncharacterized TIM barrel protein			
===								7	Pyrobaculum_1860_uid82379	NC_016645	chromosome									
374325741	hypothetical protein P186_0228	No	0		203412	203693	-		Pyrobaculum_1860_uid82379	NC_016645	chromosome									
374325742	hypothetical protein P186_0229	No	0		203732	204553	+		Pyrobaculum_1860_uid82379	NC_016645	chromosome		arCOG07952	4 (-)	S		PaRep2b protein, some fused to other mobile dc	7E-13	pfam07775	PaRep2b protein. This is a family of pro
374325743	hypothetical protein P186_0230	No	0		204699	204983	-		Pyrobaculum_1860_uid82379	NC_016645	chromosome									
374325744	hypothetical protein P186_0231	No	0		204973	205149	-		Pyrobaculum_1860_uid82379	NC_016645	chromosome									
374325745	hypothetical protein P186_0232	No	0		205343	205741	+		Pyrobaculum_1860_uid82379	NC_016645	chromosome									
374325746	3-isopropylmalate dehydratase small subunit	No	0		205747	206157	-		Pyrobaculum_1860_uid82379	NC_016645	chromosome		arCOG02230	1 (-)	E	LeuD	3-isopropylmalate dehydratase small subunit	3E-20	COG0066	3-isopropylmalate dehydratase small subu
374325747	hypothetical protein P186_0233	No	1		206150	206284	+		Pyrobaculum_1860_uid82379	NC_016645	chromosome									
===								14	Pyrobaculum_1860_uid82379	NC_016645	chromosome									
374325797	hypothetical protein P186_0287	Yes	1		246722	247273	+		Pyrobaculum_1860_uid82379	NC_016645	chromosome		arCOG04702	1 (-)	R		Phospholipid-binding protein	5E-42	COG1881	Phospholipid-binding protein
374325798	hypothetical protein P186_0288	No	1		247250	247375	-		Pyrobaculum_1860_uid82379	NC_016645	chromosome									
374325799	hypothetical protein P186_0289	No	0		247443	247685	-		Pyrobaculum_1860_uid82379	NC_016645	chromosome									
374325800	hypothetical protein P186_0290	No	0		247695	248075	-		Pyrobaculum_1860_uid82379	NC_016645	chromosome	clust_280								
374325801	hypothetical protein P186_0291	No	0		248129	248359	-		Pyrobaculum_1860_uid82379	NC_016645	chromosome									
374325802	hypothetical protein P186_0292	No	0		248382	248645	-		Pyrobaculum_1860_uid82379	NC_016645	chromosome	clust_384								
374325803	hypothetical protein P186_0294	No	0		248642	248764	-		Pyrobaculum_1860_uid82379	NC_016645	chromosome									
374325804	hypothetical protein P186_0293	No	0		248763	248906	+		Pyrobaculum_1860_uid82379	NC_016645	chromosome									
374325805	hypothetical protein P186_0295	No	0		249165	249419	-		Pyrobaculum_1860_uid82379	NC_016645	chromosome	clust_394								
374325806	hypothetical protein P186_0296	No	0		249446	249673	+		Pyrobaculum_1860_uid82379	NC_016645	chromosome	clust_425								
374325807	hypothetical protein P186_0297	No	0		249682	249837	-		Pyrobaculum_1860_uid82379	NC_016645	chromosome									
374325808	hypothetical protein P186_0298	No	0		249923	250087	-		Pyrobaculum_1860_uid82379	NC_016645	chromosome									
374325809	hypothetical protein P186_0300	No	0		250260	250586	-		Pyrobaculum_1860_uid82379	NC_016645	chromosome									
374325810	ATPase	No	0		250583	253321	-		Pyrobaculum_1860_uid82379	NC_016645	chromosome		arCOG00888	1 (-)	R		Predicted ATPase (AAA+ superfamily)	1E-21	COG1483	Predicted ATPase (AAA+ superfamily)
===								6	Pyrobaculum_1860_uid82379	NC_016645	chromosome									
374325829	hypothetical protein P186_0322	No	0		275037	275192	+		Pyrobaculum_1860_uid82379	NC_016645	chromosome									
374325830	hypothetical protein P186_0323	No	3		275211	275750	+		Pyrobaculum_1860_uid82379	NC_016645	chromosome									
374325831	hypothetical protein P186_0324	No	6		275750	276592	+		Pyrobaculum_1860_uid82379	NC_016645	chromosome									
374325832	hypothetical protein P186_0325	No	0		276832	277200	-		Pyrobaculum_1860_uid82379	NC_016645	chromosome		arCOG05421	3 (-)	S		paREP9 family			
374325833	hypothetical protein P186_0326	No	0		278187	278528	-		Pyrobaculum_1860_uid82379	NC_016645	chromosome									
374325834	hypothetical protein P186_0327	No	0		278831	278965	-		Pyrobaculum_1860_uid82379	NC_016645	chromosome									
===								8	Pyrobaculum_1860_uid82379	NC_016645	chromosome									
374325903	fuculose-1-phosphate aldolase (fucA)	No	0		348185	348784	-		Pyrobaculum_1860_uid82379	NC_016645	chromosome		arCOG04226	2 (-)	G		Fuculose-1-phosphate aldolase	7E-37	COG0235	Ribulose-5-phosphate 4-epimerase and rel
374325904	hypothetical protein P186_0398	No	3		348932	349267	-		Pyrobaculum_1860_uid82379	NC_016645	chromosome									
374325905	hypothetical protein P186_0399	No	0		349434	349673	-		Pyrobaculum_1860_uid82379	NC_016645	chromosome		arCOG09761	8	S		Uncharacterized conserved protein			
374325906	hypothetical protein P186_0400	No	1		350143	351765	-		Pyrobaculum_1860_uid82379	NC_016645	chromosome		arCOG09761	8	S		Uncharacterized conserved protein			
374325907	hypothetical protein P186_0401	No	0		351802	351918	-		Pyrobaculum_1860_uid82379	NC_016645	chromosome									
374325908	hypothetical protein P186_0402	No	3		351957	352400	-		Pyrobaculum_1860_uid82379	NC_016645	chromosome									
374325909	hypothetical protein P186_0403	Yes	0		352464	354722	-		Pyrobaculum_1860_uid82379	NC_016645	chromosome		arCOG09761	8	S		Uncharacterized conserved protein			
374325910	geranylgeranyl reductase	No	0		354781	356151	-		Pyrobaculum_1860_uid82379	NC_016645	chromosome		arCOG00570	2 (-)	C	FixC	Dehydrogenase (flavoprotein)	9E-45	COG0644	Dehydrogenases (flavoproteins)
===								5	Pyrobaculum_1860_uid82379	NC_016645	chromosome									
374326026	hypothetical protein P186_0522	No	1		451558	451674	+		Pyrobaculum_1860_uid82379	NC_016645	chromosome									
374326027	Microtubule binding protein	No	0		451755	452369	-		Pyrobaculum_1860_uid82379	NC_016645	chromosome		arCOG01424	2 (-)	R		RecB-nuclease fused to coiled-coil domain	0.000000003	pfam07788	Protein of unknown function (DUF1626). T ; ATPase involved in DNA repair
374326028	hypothetical protein P186_0524	No	0		452568	452696	+		Pyrobaculum_1860_uid82379	NC_016645	chromosome									
374326029	hypothetical protein P186_0525	No	4		453155	453550	-		Pyrobaculum_1860_uid82379	NC_016645	chromosome		arCOG09844	1	S		Uncharacterized conserved protein			
374326030	hypothetical protein P186_0526	No	0		453567	453689	-		Pyrobaculum_1860_uid82379	NC_016645	chromosome									
===								7	Pyrobaculum_1860_uid82379	NC_016645	chromosome									
374326386	putative ABC transporter	No	0		759537	759788	-		Pyrobaculum_1860_uid82379	NC_016645	chromosome		arCOG07131	7 (-)	V	CcmA	ABC-type multidrug transport system, ATPase co	2E-12	COG1120	ABC-type cobalamin/Fe3+-siderophores tra
374326387	hypothetical protein P186_0894	No	3		759775	760203	-		Pyrobaculum_1860_uid82379	NC_016645	chromosome	clust_245								
374326388	hypothetical protein P186_0895	No	9		760181	761197	-		Pyrobaculum_1860_uid82379	NC_016645	chromosome	clust_95								
374326389	hypothetical protein P186_0896	No	3		761198	761602	-		Pyrobaculum_1860_uid82379	NC_016645	chromosome									
374326390	hypothetical protein P186_0897	No	0		762641	762793	+		Pyrobaculum_1860_uid82379	NC_016645	chromosome									
374326391	hypothetical protein P186_0898	No	0		762895	763062	-		Pyrobaculum_1860_uid82379	NC_016645	chromosome									
374326392	CRISPR-associated protein, Csa1 family	No	0		763438	764001	+		Pyrobaculum_1860_uid82379	NC_016645	chromosome		arCOG04195	1 (-)	V		CRISPR-associated protein, RecB family exonucl	1E-44	pfam06023	Archaeal protein of unknown function (DU
===								14	Pyrobaculum_1860_uid82379	NC_016645	chromosome									
374326433	CRISPR-associated protein Cas6	No	0		794007	794801	-		Pyrobaculum_1860_uid82379	NC_016645	chromosome		arCOG01439	2 (-)	V	Cas6	CRISPR system related protein, RAMP superfam	8E-22	COG5551	CRISPR system related protein, RAMP supe
374326434	hypothetical protein P186_0941	No	0		794834	794989	+		Pyrobaculum_1860_uid82379	NC_016645	chromosome									
374326435	hypothetical protein P186_0942	Yes	0		795473	795712	+		Pyrobaculum_1860_uid82379	NC_016645	chromosome									
374326436	hypothetical protein P186_0943	No	0		795765	796094	+		Pyrobaculum_1860_uid82379	NC_016645	chromosome									
374326437	hypothetical protein P186_0944	No	1		796213	796371	-		Pyrobaculum_1860_uid82379	NC_016645	chromosome									
374326438	hypothetical protein P186_0945	No	0		796428	796628	+		Pyrobaculum_1860_uid82379	NC_016645	chromosome									
374326439	hypothetical protein P186_0946	No	0		796637	796756	+		Pyrobaculum_1860_uid82379	NC_016645	chromosome									
374326440	hypothetical protein P186_0947	No	0		797399	797737	-		Pyrobaculum_1860_uid82379	NC_016645	chromosome									
374326441	hypothetical protein P186_0948	No	0		797803	797940	-		Pyrobaculum_1860_uid82379	NC_016645	chromosome									
374326442	hypothetical protein P186_0949	No	0		798246	798371	+		Pyrobaculum_1860_uid82379	NC_016645	chromosome									
374326443	hypothetical protein P186_0950	No	0		798524	798712	+		Pyrobaculum_1860_uid82379	NC_016645	chromosome									
374326444	hypothetical protein P186_0951	No	0		798761	798922	+		Pyrobaculum_1860_uid82379	NC_016645	chromosome									
374326445	hypothetical protein P186_0952	No	0		799001	799291	-		Pyrobaculum_1860_uid82379	NC_016645	chromosome									
374326446	hypothetical protein P186_0953	No	0		799288	799563	-		Pyrobaculum_1860_uid82379	NC_016645	chromosome		arCOG01057	21 (-)	K		Predicted transcriptional regulator			
===								5	Pyrobaculum_1860_uid82379	NC_016645	chromosome									
374326476	hypothetical protein P186_0985	No	0		826608	827192	+		Pyrobaculum_1860_uid82379	NC_016645	chromosome									
374326477	hypothetical protein P186_0986	No	0		827199	827843	+		Pyrobaculum_1860_uid82379	NC_016645	chromosome									
374326478	hypothetical protein P186_0987	No	0		827911	828060	+		Pyrobaculum_1860_uid82379	NC_016645	chromosome									
374326479	nicotinate-nucleotide pyrophosphorylase	No	0		828066	828941	-		Pyrobaculum_1860_uid82379	NC_016645	chromosome		arCOG00254	1 (-)	R		Predicted dinucleotide-utilizing enzyme	5E-30	COG1712	Predicted dinucleotide-utilizing enzyme
374326480	hypothetical protein P186_0989	No	0		829030	829152	+		Pyrobaculum_1860_uid82379	NC_016645	chromosome									
===								6	Pyrobaculum_1860_uid82379	NC_016645	chromosome									
374326491	hypothetical protein P186_1000	No	0		836203	837162	+		Pyrobaculum_1860_uid82379	NC_016645	chromosome		arCOG00586	8 (-)	D	Soj	ATPase involved in chromosome partitioning, P	0.000002	COG1192	ATPases involved in chromosome partition
374326492	hypothetical protein P186_1001	No	0		837332	837529	+		Pyrobaculum_1860_uid82379	NC_016645	chromosome									
374326493	hypothetical protein P186_1002	No	0		837706	837831	+		Pyrobaculum_1860_uid82379	NC_016645	chromosome									
374326494	hypothetical protein P186_1003	No	0		838458	838571	+		Pyrobaculum_1860_uid82379	NC_016645	chromosome									
374326495	hypothetical protein P186_1004	No	0		838761	838925	-		Pyrobaculum_1860_uid82379	NC_016645	chromosome									
374326496	hypothetical protein P186_1005	No	0		839491	840273	-		Pyrobaculum_1860_uid82379	NC_016645	chromosome		arCOG01439	2 (-)	V	Cas6	CRISPR system related protein, RAMP superfam	9E-13	COG5551	CRISPR system related protein, RAMP supe
===								5	Pyrobaculum_1860_uid82379	NC_016645	chromosome									
374326879	hypothetical protein P186_1400	No	0		1175432	1175551	+		Pyrobaculum_1860_uid82379	NC_016645	chromosome									
374326880	hypothetical protein P186_1401	No	0		1175853	1176749	-		Pyrobaculum_1860_uid82379	NC_016645	chromosome									
374326881	hypothetical protein P186_1402	No	0		1176931	1177143	-		Pyrobaculum_1860_uid82379	NC_016645	chromosome									
374326882	hypothetical protein P186_1403	No	1		1177227	1178228	-		Pyrobaculum_1860_uid82379	NC_016645	chromosome		arCOG00280	21 (-)	L		HerA helicase			
374326883	hypothetical protein P186_1404	No	0		1178212	1179021	-		Pyrobaculum_1860_uid82379	NC_016645	chromosome									
===								10	Pyrobaculum_1860_uid82379	NC_016645	chromosome									
374326973	hypothetical protein P186_1500	No	6		1269280	1269792	+		Pyrobaculum_1860_uid82379	NC_016645	chromosome		arCOG07033	1	S		Uncharacterized conserved protein			
374326974	hypothetical protein P186_1501	No	1		1269982	1271574	+		Pyrobaculum_1860_uid82379	NC_016645	chromosome		arCOG03754	1 (-)	S		Uncharacterized conserved protein			
374326975	hypothetical protein P186_1502	No	0		1272364	1272612	+		Pyrobaculum_1860_uid82379	NC_016645	chromosome									
374326976	hypothetical protein P186_1503	No	1		1272837	1273475	+		Pyrobaculum_1860_uid82379	NC_016645	chromosome									
374326977	hypothetical protein P186_1506	No	0		1274847	1274975	-		Pyrobaculum_1860_uid82379	NC_016645	chromosome									
374326978	hypothetical protein P186_1507	No	3		1275621	1275959	+		Pyrobaculum_1860_uid82379	NC_016645	chromosome									
374326979	hypothetical protein P186_1508	No	0		1275956	1276183	+		Pyrobaculum_1860_uid82379	NC_016645	chromosome		arCOG09772	4	S		Uncharacterized conserved protein			
374326980	hypothetical protein P186_1509	Yes	1		1276180	1276971	+		Pyrobaculum_1860_uid82379	NC_016645	chromosome	arCOG09773,a	arCOG09773	4	S		Uncharacterized conserved protein			
374326981	hypothetical protein P186_1510	No	0		1277004	1277171	-		Pyrobaculum_1860_uid82379	NC_016645	chromosome									
374326982	hypothetical protein P186_1511	Yes	1		1277645	1278349	+		Pyrobaculum_1860_uid82379	NC_016645	chromosome		arCOG07697	1 (-)	S		Uncharacterized conserved protein			
===								6	Pyrobaculum_1860_uid82379	NC_016645	chromosome									
374327067	hypothetical protein P186_1597	No	3		1345971	1346324	+		Pyrobaculum_1860_uid82379	NC_016645	chromosome									
374327068	hypothetical protein P186_1598	No	3		1346315	1346608	+		Pyrobaculum_1860_uid82379	NC_016645	chromosome									
374327069	hypothetical protein P186_1599	Yes	1		1346971	1348044	-		Pyrobaculum_1860_uid82379	NC_016645	chromosome		arCOG07483	1 (-)	S		Uncharacterized conserved protein			
374327070	hypothetical protein P186_1600	No	1		1348426	1348548	-		Pyrobaculum_1860_uid82379	NC_016645	chromosome									
374327071	hypothetical protein P186_1601	No	0		1348677	1348823	+		Pyrobaculum_1860_uid82379	NC_016645	chromosome		arCOG09791	2	S		Uncharacterized conserved protein			
374327072	hypothetical protein P186_1602	Yes	2		1349220	1349561	-		Pyrobaculum_1860_uid82379	NC_016645	chromosome									
===								7	Pyrobaculum_1860_uid82379	NC_016645	chromosome									
374327143	hypothetical protein P186_1677	Yes	1		1418028	1419815	+		Pyrobaculum_1860_uid82379	NC_016645	chromosome		arCOG01937	2 (-)	R		Archaeal serine protease	1E-19	COG1750	Archaeal serine proteases
374327144	hypothetical protein P186_1678	No	0		1419968	1420081	-		Pyrobaculum_1860_uid82379	NC_016645	chromosome									
374327145	hypothetical protein P186_1679	No	0		1420113	1420226	-		Pyrobaculum_1860_uid82379	NC_016645	chromosome									
374327146	hypothetical protein P186_1680	No	1		1420275	1421072	+		Pyrobaculum_1860_uid82379	NC_016645	chromosome									
374327147	hypothetical protein P186_1681	No	12		1421097	1422500	+		Pyrobaculum_1860_uid82379	NC_016645	chromosome									
374327148	cytochrome c assembly protein	No	15		1422525	1425185	+		Pyrobaculum_1860_uid82379	NC_016645	chromosome		arCOG00268	1 (-)	O	CcmF	Cytochrome c biogenesis factor	5E-38	COG1138	Cytochrome c biogenesis factor
374327149	hypothetical protein P186_1683	No	1		1425680	1426537	+		Pyrobaculum_1860_uid82379	NC_016645	chromosome		arCOG10933	1	S		Uncharacterized conserved protein			
===								5	Pyrobaculum_1860_uid82379	NC_016645	chromosome									
374327321	hypothetical protein P186_1867	No	1		1570199	1571227	+		Pyrobaculum_1860_uid82379	NC_016645	chromosome		arCOG07050	1	S		Uncharacterized conserved protein			
374327322	hypothetical protein P186_1868	Yes	1		1571229	1572548	+		Pyrobaculum_1860_uid82379	NC_016645	chromosome		arCOG02568	1	S		beta-propeller repeat periplasmic protein			
374327323	hypothetical protein P186_1869	No	8		1572545	1573294	+		Pyrobaculum_1860_uid82379	NC_016645	chromosome									
374327324	flavoprotein reductase	No	0		1573291	1574493	+		Pyrobaculum_1860_uid82379	NC_016645	chromosome		arCOG01066	1 (-)	R	HcaD	NAD(FAD)-dependent dehydrogenase	6E-12	COG0446	Uncharacterized NAD(FAD)-dependent dehyd
374327325	hypothetical protein P186_1871	No	0		1574488	1574814	-		Pyrobaculum_1860_uid82379	NC_016645	chromosome		arCOG07049	2	S		Uncharacterized conserved protein			
===								6	Pyrobaculum_1860_uid82379	NC_016645	chromosome									
374327458	UDP-N-acetylglucosamine 2-epimerase	No	0		1684394	1684525	-		Pyrobaculum_1860_uid82379	NC_016645	chromosome		arCOG01392	1 (-)	M	WecB	UDP-N-acetylglucosamine 2-epimerase			
374327459	hypothetical protein P186_2012	No	2		1684571	1685158	-		Pyrobaculum_1860_uid82379	NC_016645	chromosome									
374327460	hypothetical protein P186_2011	No	0		1685268	1685414	+		Pyrobaculum_1860_uid82379	NC_016645	chromosome									
374327461	hypothetical protein P186_2013	No	4		1685398	1685862	-		Pyrobaculum_1860_uid82379	NC_016645	chromosome									
374327462	hypothetical protein P186_2014	No	4		1685844	1686323	-		Pyrobaculum_1860_uid82379	NC_016645	chromosome									
374327463	glycosyltransferase	No	0		1686670	1687833	-		Pyrobaculum_1860_uid82379	NC_016645	chromosome		arCOG01418	2 (-)	M	RfaG	Glycosyltransferase	0.0001	COG0438	Glycosyltransferase
===								5	Pyrobaculum_1860_uid82379	NC_016645	chromosome									
374327480	hypothetical protein P186_2032	No	0		1700696	1701034	+		Pyrobaculum_1860_uid82379	NC_016645	chromosome									
374327481	hypothetical protein P186_2033	No	0		1701180	1701320	-		Pyrobaculum_1860_uid82379	NC_016645	chromosome									
374327482	hypothetical protein P186_2034	No	0		1701461	1701859	-		Pyrobaculum_1860_uid82379	NC_016645	chromosome									
374327483	hypothetical protein P186_2036	No	0		1702014	1702322	-		Pyrobaculum_1860_uid82379	NC_016645	chromosome		arCOG03408	3 (-)	R		Predicted ATPase (AAA+ superfamily)			
374327484	hypothetical protein P186_2035	No	0		1702614	1702751	+		Pyrobaculum_1860_uid82379	NC_016645	chromosome									
===								5	Pyrobaculum_1860_uid82379	NC_016645	chromosome									
374327820	hypothetical protein P186_2382	No	0		1986456	1986569	-		Pyrobaculum_1860_uid82379	NC_016645	chromosome									
374327821	hypothetical protein P186_2383	No	3		1986692	1986979	-		Pyrobaculum_1860_uid82379	NC_016645	chromosome		arCOG04811	3 (-)	S		Uncharacterized conserved membrane protein, Fun14 family			
374327822	hypothetical protein P186_2384	No	0		1986996	1987385	-		Pyrobaculum_1860_uid82379	NC_016645	chromosome									
374327823	hypothetical protein P186_2385	No	0		1987399	1987566	-		Pyrobaculum_1860_uid82379	NC_016645	chromosome									
374327824	hypothetical protein P186_2386	No	0		1987683	1987982	-		Pyrobaculum_1860_uid82379	NC_016645	chromosome									
===								7	Pyrobaculum_1860_uid82379	NC_016645	chromosome									
374327904	hypothetical protein P186_2466	No	1		2056347	2056508	-		Pyrobaculum_1860_uid82379	NC_016645	chromosome									
374327905	glycerophosphoryl diester phosphodiesterase	No	0		2056561	2057247	+		Pyrobaculum_1860_uid82379	NC_016645	chromosome		arCOG00701	1 (-)	C	UgpQ	Glycerophosphoryl diester phosphodiesterase	5E-28	COG0584	Glycerophosphoryl diester phosphodiester
374327906	hypothetical protein P186_2468	No	0		2057541	2057918	+		Pyrobaculum_1860_uid82379	NC_016645	chromosome									
374327907	hypothetical protein P186_2469	Yes	6		2057993	2058478	-		Pyrobaculum_1860_uid82379	NC_016645	chromosome	clust_215								
374327908	hypothetical protein P186_2470	No	0		2058502	2060592	-		Pyrobaculum_1860_uid82379	NC_016645	chromosome		arCOG07012	6	S		Uncharacterized conserved protein			
374327909	hypothetical protein P186_2471	No	0		2060884	2061000	+		Pyrobaculum_1860_uid82379	NC_016645	chromosome									
374327910	sulfonate transport permease protein	No	11		2061173	2062552	+		Pyrobaculum_1860_uid82379	NC_016645	chromosome		arCOG00174	2 (-)	P		ABC-type anion transport system, duplicated per	4E-79	COG4986	ABC-type anion transport system, duplica
===								6	Pyrobaculum_1860_uid82379	NC_016645	chromosome									
374327916	hypothetical protein P186_2478	No	0		2066871	2067164	-		Pyrobaculum_1860_uid82379	NC_016645	chromosome		arCOG00727	2 (-)	V		PIN domain containing protein	0.000000004	COG4113	Predicted nucleic acid-binding protein,
374327917	hypothetical protein P186_2479	No	1		2067507	2067659	+		Pyrobaculum_1860_uid82379	NC_016645	chromosome									
374327918	hypothetical protein P186_2480	No	5		2067702	2068265	-		Pyrobaculum_1860_uid82379	NC_016645	chromosome		arCOG09775	2	S		Uncharacterized conserved protein			
374327919	hypothetical protein P186_2481	No	0		2068249	2070336	-		Pyrobaculum_1860_uid82379	NC_016645	chromosome		arCOG07691	7	S		Uncharacterized conserved protein			
374327920	hypothetical protein P186_2482	No	0		2070578	2071078	+		Pyrobaculum_1860_uid82379	NC_016645	chromosome									
374327921	hypothetical protein P186_2483	No	0		2071370	2071594	-		Pyrobaculum_1860_uid82379	NC_016645	chromosome		arCOG02217	2 (-)	V		RHH/CopG DNA binding protein			
===								5	Pyrobaculum_1860_uid82379	NC_016645	chromosome									
374327994	hypothetical protein P186_2558	No	6		2134824	2135828	-		Pyrobaculum_1860_uid82379	NC_016645	chromosome		arCOG07074	1	S		Uncharacterized conserved protein			
374327995	hypothetical protein P186_2559	No	4		2136196	2136531	+		Pyrobaculum_1860_uid82379	NC_016645	chromosome									
374327996	hypothetical protein P186_2560	No	1		2136548	2138566	+		Pyrobaculum_1860_uid82379	NC_016645	chromosome		arCOG02090	1 (-)	S		Uncharacterized conserved membrane protein			
374327997	hypothetical protein P186_2561	Yes	0		2138706	2138873	-		Pyrobaculum_1860_uid82379	NC_016645	chromosome									
374327998	hypothetical protein P186_2562	No	1		2139201	2139338	+		Pyrobaculum_1860_uid82379	NC_016645	chromosome									
===								5	Pyrobaculum_1860_uid82379	NC_016645	chromosome									
374328106	hypothetical protein P186_2670	No	0		2234264	2234395	-		Pyrobaculum_1860_uid82379	NC_016645	chromosome									
374328107	hypothetical protein P186_2671	No	1		2234368	2234745	-		Pyrobaculum_1860_uid82379	NC_016645	chromosome		arCOG05421	3 (-)	S		paREP9 family			
374328108	hypothetical protein P186_2672	No	0		2234730	2234966	-		Pyrobaculum_1860_uid82379	NC_016645	chromosome									
374328109	hypothetical protein P186_2673	No	0		2235217	2235330	-		Pyrobaculum_1860_uid82379	NC_016645	chromosome									
374328110	hypothetical protein P186_2674	No	0		2236110	2236307	-		Pyrobaculum_1860_uid82379	NC_016645	chromosome									
===								7	Pyrobaculum_1860_uid82379	NC_016645	chromosome									
374328141	haloacetate dehalogenase	No	0		2265131	2265826	-		Pyrobaculum_1860_uid82379	NC_016645	chromosome		arCOG02291	4 (-)	R		HAD superfamily hydrolase	2E-17	COG1011	Predicted hydrolase (HAD superfamily)
374328142	hypothetical protein P186_2706	Yes	0		2265901	2266956	+		Pyrobaculum_1860_uid82379	NC_016645	chromosome		arCOG10888	7	S		Uncharacterized conserved protein			
374328143	hypothetical protein P186_2707	No	0		2267861	2268085	-		Pyrobaculum_1860_uid82379	NC_016645	chromosome									
374328144	hypothetical protein P186_2708	Yes	0		2268262	2268462	+		Pyrobaculum_1860_uid82379	NC_016645	chromosome		arCOG09758	4	S		Uncharacterized conserved protein			
374328145	hypothetical protein P186_2709	No	1		2268459	2269157	+		Pyrobaculum_1860_uid82379	NC_016645	chromosome	arCOG09800,a	arCOG09800	3	S		Uncharacterized conserved protein			
374328146	hypothetical protein P186_2710	No	0		2269446	2270420	-		Pyrobaculum_1860_uid82379	NC_016645	chromosome		arCOG09849	1	S		Uncharacterized conserved protein			
374328147	hypothetical protein P186_2711	No	0		2270407	2271117	-		Pyrobaculum_1860_uid82379	NC_016645	chromosome		arCOG04037	1 (-)	K		Transcriptional regulator containing HTH domain			
===								9	Pyrobaculum_1860_uid82379	NC_016645	chromosome									
374328211	hypothetical protein P186_2783	Yes	0		2329349	2331628	+		Pyrobaculum_1860_uid82379	NC_016645	chromosome		arCOG07691	7	S		Uncharacterized conserved protein			
374328212	hypothetical protein P186_2784	No	5		2331654	2332154	+		Pyrobaculum_1860_uid82379	NC_016645	chromosome		arCOG09771	3	S		Uncharacterized conserved protein			
374328213	Fis family transcriptional regulator	No	0		2332343	2333287	+		Pyrobaculum_1860_uid82379	NC_016645	chromosome		arCOG00415	7 (-)	L	RecA	RecA/RadA recombinase	2E-14	pfam08423	Rad51. Rad51 is a DNA repair and recombi
374328214	hypothetical protein P186_2786	No	0		2333583	2333924	+		Pyrobaculum_1860_uid82379	NC_016645	chromosome									
374328215	hypothetical protein P186_2787	No	0		2333909	2334889	+		Pyrobaculum_1860_uid82379	NC_016645	chromosome									
374328216	hypothetical protein P186_2788	No	0		2334920	2335153	+		Pyrobaculum_1860_uid82379	NC_016645	chromosome									
374328217	hypothetical protein P186_2789	Yes	3		2335481	2336035	+		Pyrobaculum_1860_uid82379	NC_016645	chromosome									
374328218	hypothetical protein P186_2790	No	6		2336035	2336889	+		Pyrobaculum_1860_uid82379	NC_016645	chromosome									
374328219	PaREP2b	No	8		2336886	2350814	+		Pyrobaculum_1860_uid82379	NC_016645	chromosome		arCOG00368	3 (-)	L	SbcC	ATPase involved in DNA repair, SbcC	2E-15	pfam07775	PaRep2b protein. This is a family of pro ; Chromosome segregation ATPases
===								14	Pyrobaculum_1860_uid82379	NC_016645	chromosome									
374328237	hypothetical protein P186_2809	No	0		2362511	2362627	-		Pyrobaculum_1860_uid82379	NC_016645	chromosome									
374328238	protease	No	0		2362799	2363806	+		Pyrobaculum_1860_uid82379	NC_016645	chromosome		arCOG03377	4 (-)	E		Serine protease			
374328239	hypothetical protein P186_2811	Yes	5		2363803	2365731	+		Pyrobaculum_1860_uid82379	NC_016645	chromosome		arCOG09782	4	S		Uncharacterized conserved protein			
374328240	hypothetical protein P186_2812	Yes	0		2365821	2366177	+		Pyrobaculum_1860_uid82379	NC_016645	chromosome									
374328241	hypothetical protein P186_2813	No	4		2366171	2366731	+		Pyrobaculum_1860_uid82379	NC_016645	chromosome									
374328242	hypothetical protein P186_2814	No	0		2366703	2366990	+		Pyrobaculum_1860_uid82379	NC_016645	chromosome		arCOG01062	2 (-)	K		Predicted transcriptional regulator			
374328243	hypothetical protein P186_2815	No	0		2366987	2367256	-		Pyrobaculum_1860_uid82379	NC_016645	chromosome									
374328244	hypothetical protein P186_2816	No	0		2367255	2367371	+		Pyrobaculum_1860_uid82379	NC_016645	chromosome									
374328245	hypothetical protein P186_2817	No	0		2367429	2367611	-		Pyrobaculum_1860_uid82379	NC_016645	chromosome									
374328246	hypothetical protein P186_2818	Yes	0		2367875	2368597	+		Pyrobaculum_1860_uid82379	NC_016645	chromosome									
374328247	hypothetical protein P186_2819	Yes	1		2368594	2369199	+		Pyrobaculum_1860_uid82379	NC_016645	chromosome		arCOG09799	2	S		Uncharacterized conserved protein			
374328248	hypothetical protein P186_2820	No	1		2369550	2369705	+		Pyrobaculum_1860_uid82379	NC_016645	chromosome									
374328249	hypothetical protein P186_2821	No	2		2369710	2370570	+		Pyrobaculum_1860_uid82379	NC_016645	chromosome		arCOG09781	3	S		Uncharacterized conserved protein			
374328250	Glu-tRNA(Gln) amidotransferase subunit A (gat	No	0		2371041	2372246	+		Pyrobaculum_1860_uid82379	NC_016645	chromosome		arCOG01717	1 (-)	J	GatA	Asp-tRNAAsn/Glu-tRNAGln amidotransferase A s	3E-73	COG0154	Asp-tRNAAsn/Glu-tRNAGln amidotransferase
===								9	Pyrobaculum_1860_uid82379	NC_016645	chromosome									
374328307	hypothetical protein P186_2879	No	0		2429469	2429753	+		Pyrobaculum_1860_uid82379	NC_016645	chromosome		arCOG05523	1 (-)	S		Uncharacterized conserved protein			
374328308	hypothetical protein P186_2880	No	1		2429735	2429992	-		Pyrobaculum_1860_uid82379	NC_016645	chromosome		arCOG09812	2	S		Uncharacterized conserved protein			
374328309	hypothetical protein P186_2881	No	1		2430096	2430344	-		Pyrobaculum_1860_uid82379	NC_016645	chromosome									
374328310	hypothetical protein P186_2882	No	0		2430558	2430935	-		Pyrobaculum_1860_uid82379	NC_016645	chromosome	clust_280								
374328311	hypothetical protein P186_2883	No	0		2431190	2431453	-		Pyrobaculum_1860_uid82379	NC_016645	chromosome	clust_384								
374328312	hypothetical protein P186_2884	No	0		2431532	2431723	+		Pyrobaculum_1860_uid82379	NC_016645	chromosome									
374328313	hypothetical protein P186_2885	No	0		2431982	2432236	-		Pyrobaculum_1860_uid82379	NC_016645	chromosome	clust_394								
374328314	hypothetical protein P186_2886	No	0		2432263	2432490	+		Pyrobaculum_1860_uid82379	NC_016645	chromosome	clust_425								
374328315	hypothetical protein P186_2887	Yes	0		2432497	2434620	-		Pyrobaculum_1860_uid82379	NC_016645	chromosome		arCOG00579	4 (-)	K	RPB9	DNA-directed RNA polymerase, subunit M/Transc	6E-30	COG1112	Superfamily I DNA and RNA helicases and ; Superfamily I DNA and RNA helicases and ; DNA-directed RNA polymerase, subunit M/T
===								6	Pyrobaculum_1860_uid82379	NC_016645	chromosome									
374328321	putative DNA methylase	No	0		2441135	2444119	-		Pyrobaculum_1860_uid82379	NC_016645	chromosome		arCOG00889	6 (-)	L		Adenine-specific DNA methylase containing a Zn	6E-144	COG1743	Adenine-specific DNA methylase containin
374328322	hypothetical protein P186_2894	No	0		2444159	2445727	+		Pyrobaculum_1860_uid82379	NC_016645	chromosome									
374328323	hypothetical protein P186_2895	No	1		2446364	2446486	+		Pyrobaculum_1860_uid82379	NC_016645	chromosome									
374328324	hypothetical protein P186_2896	No	0		2446511	2446762	+		Pyrobaculum_1860_uid82379	NC_016645	chromosome									
374328325	hypothetical protein P186_2897	No	0		2446845	2447675	+		Pyrobaculum_1860_uid82379	NC_016645	chromosome									
374328326	hypothetical protein P186_2898	No	0		2447875	2451960	+		Pyrobaculum_1860_uid82379	NC_016645	chromosome	arCOG03045,a	arCOG03045	2 (-)	R	NrfG	TPR repeats containing protein			
===								5	Pyrobaculum_aerophilum_IM2_uid57727	NC_003364	chromosome									
18311701	hypothetical protein PAE0084	No	1	YES	46700	46903	+		Pyrobaculum_aerophilum_IM2_uid57727	NC_003364	chromosome									
18311702	hypothetical protein PAE0086	No	0	YES	46970	47239	+		Pyrobaculum_aerophilum_IM2_uid57727	NC_003364	chromosome									
18311703	hypothetical protein PAE0087	No	4	YES	47280	47747	+		Pyrobaculum_aerophilum_IM2_uid57727	NC_003364	chromosome									
18311704	hypothetical protein PAE0088	No	0	YES	47753	48766	-		Pyrobaculum_aerophilum_IM2_uid57727	NC_003364	chromosome		arCOG06980	1 (-)	S		vWFA domain containing protein	0.00000008	COG3864	Uncharacterized protein conserved in bac
18311705	hypothetical protein PAE0089	No	0	YES	48829	48975	-		Pyrobaculum_aerophilum_IM2_uid57727	NC_003364	chromosome									
===								8	Pyrobaculum_aerophilum_IM2_uid57727	NC_003364	chromosome									
18311734	hypothetical protein PAE0133	No	3	YES	74890	75330	-		Pyrobaculum_aerophilum_IM2_uid57727	NC_003364	chromosome									
18311735	hypothetical protein PAE0134	No	0	YES	75306	75926	-		Pyrobaculum_aerophilum_IM2_uid57727	NC_003364	chromosome									
18311736	hypothetical protein PAE0135	No	4	YES	75923	76297	-		Pyrobaculum_aerophilum_IM2_uid57727	NC_003364	chromosome		arCOG04907	2 (-)	S		Uncharacterized conserved membrane protein	0.00002	pfam12822	Protein of unknown function (DUF3816). T
18311737	hypothetical protein PAE0137	No	0	YES	76373	76714	-		Pyrobaculum_aerophilum_IM2_uid57727	NC_003364	chromosome									
18311738	hypothetical protein PAE0138	No	0	YES	77136	77405	-		Pyrobaculum_aerophilum_IM2_uid57727	NC_003364	chromosome									
18311739	hypothetical protein PAE0139	No	0	YES	77455	77640	+		Pyrobaculum_aerophilum_IM2_uid57727	NC_003364	chromosome									
18311740	hypothetical protein PAE0141	No	0	YES	77664	77840	-		Pyrobaculum_aerophilum_IM2_uid57727	NC_003364	chromosome									
18311741	paREP5a	No	1	YES	77849	78304	+		Pyrobaculum_aerophilum_IM2_uid57727	NC_003364	chromosome		arCOG03877	1 (-)	S		Uncharacterized conserved protein	0.00000003	COG1196	Chromosome segregation ATPases
===								6	Pyrobaculum_aerophilum_IM2_uid57727	NC_003364	chromosome									
18311828	hypothetical protein PAE0276	No	0	YES	152782	153204	+		Pyrobaculum_aerophilum_IM2_uid57727	NC_003364	chromosome		arCOG05426	1 (-)	S		Uncharacterized conserved protein			
18311829	hypothetical protein PAE0278	Yes	1	YES	153585	155069	-		Pyrobaculum_aerophilum_IM2_uid57727	NC_003364	chromosome									
18311830	hypothetical protein PAE0279	No	1	YES	155066	156349	-		Pyrobaculum_aerophilum_IM2_uid57727	NC_003364	chromosome									
18311831	hypothetical protein PAE0280	Yes	5	YES	156806	157387	-		Pyrobaculum_aerophilum_IM2_uid57727	NC_003364	chromosome	clust_180								
18311832	hypothetical protein PAE0282	Yes	2	YES	157359	159680	-		Pyrobaculum_aerophilum_IM2_uid57727	NC_003364	chromosome		arCOG09761	8	S		Uncharacterized conserved protein			
18311833	hypothetical protein PAE0284	No	0	YES	160080	160505	-		Pyrobaculum_aerophilum_IM2_uid57727	NC_003364	chromosome		arCOG04124	1 (-)	S		Uncharacterized conserved protein			
===								9	Pyrobaculum_aerophilum_IM2_uid57727	NC_003364	chromosome									
18311835	paREP2b	No	0	YES	160952	161050	-		Pyrobaculum_aerophilum_IM2_uid57727	NC_003364	chromosome							0.00007	pfam07775	PaRep2b protein. This is a family of pro
18311836	hypothetical protein PAE0288	No	2	YES	161995	162159	-		Pyrobaculum_aerophilum_IM2_uid57727	NC_003364	chromosome		arCOG09762	4	S		Uncharacterized conserved protein			
18311837	metallo cofactor biosynthesis protein, conjectu	No	0		162146	163159	-		Pyrobaculum_aerophilum_IM2_uid57727	NC_003364	chromosome		arCOG00938	12 (-)	R		Radical SAM superfamily enzyme	0.0000002	COG0535	Predicted Fe-S oxidoreductases
18311838	hypothetical protein PAE0290	No	1		163159	163416	-		Pyrobaculum_aerophilum_IM2_uid57727	NC_003364	chromosome		arCOG09763	2	S		Uncharacterized conserved protein			
18311839	hypothetical protein PAE0292	No	1		163413	163982	-		Pyrobaculum_aerophilum_IM2_uid57727	NC_003364	chromosome		arCOG09764	3	S		Uncharacterized conserved protein			
18311840	hypothetical protein PAE0293	No	2		164013	164492	+		Pyrobaculum_aerophilum_IM2_uid57727	NC_003364	chromosome		arCOG09765	4	S		Uncharacterized conserved protein			
18311841	hypothetical protein PAE0294	No	7		164489	165313	+		Pyrobaculum_aerophilum_IM2_uid57727	NC_003364	chromosome		arCOG02768	1 (-)	R		Metal-dependent membrane protease, CAAX fan	0.00001	pfam02517	CAAX protease self-immunity. Members of
18311842	hypothetical protein PAE0294a	No	0		165416	165508	-		Pyrobaculum_aerophilum_IM2_uid57727	NC_003364	chromosome									
18311843	hypothetical protein PAE0294b	No	0		165922	166062	+		Pyrobaculum_aerophilum_IM2_uid57727	NC_003364	chromosome							8E-10	pfam07903	PaRep2a protein. This is a family of pro
===								9	Pyrobaculum_aerophilum_IM2_uid57727	NC_003364	chromosome									
18311920	paREP8	No	0	YES	225611	225922	+		Pyrobaculum_aerophilum_IM2_uid57727	NC_003364	chromosome		arCOG03721	3 (-)	V		HEPN domain containing protein	1E-13	pfam05942	Archaeal PaREP1/PaREP8 family. This fami
18311921	hypothetical protein PAE0409	No	2	YES	225966	226175	-		Pyrobaculum_aerophilum_IM2_uid57727	NC_003364	chromosome									
18311922	hypothetical protein PAE0410	No	0	YES	226397	226648	+		Pyrobaculum_aerophilum_IM2_uid57727	NC_003364	chromosome		arCOG09767	2	S		Uncharacterized conserved protein			
18311923	hypothetical protein PAE0411	No	0	YES	226651	226935	+		Pyrobaculum_aerophilum_IM2_uid57727	NC_003364	chromosome									
18311924	hypothetical protein PAE0412	No	0	YES	226953	227087	+		Pyrobaculum_aerophilum_IM2_uid57727	NC_003364	chromosome									
18311925	hypothetical protein PAE0413	No	0	YES	227117	227578	+		Pyrobaculum_aerophilum_IM2_uid57727	NC_003364	chromosome		arCOG09768	3	S		Uncharacterized conserved protein			
18311926	hypothetical protein PAE0415	No	1	YES	227611	227850	-		Pyrobaculum_aerophilum_IM2_uid57727	NC_003364	chromosome									
18311927	paREP1	No	0	YES	227875	228441	+		Pyrobaculum_aerophilum_IM2_uid57727	NC_003364	chromosome		arCOG03712	4 (-)	V		HEPN domain containing protein	2E-12	pfam05942	Archaeal PaREP1/PaREP8 family. This fami
18311928	hypothetical protein PAE0418	No	0	YES	228549	228746	+		Pyrobaculum_aerophilum_IM2_uid57727	NC_003364	chromosome		arCOG09428	1	S		Uncharacterized conserved protein			
===								6	Pyrobaculum_aerophilum_IM2_uid57727	NC_003364	chromosome									
18311937	glycosyltransferase (type 1)	No	0		233901	235070	+		Pyrobaculum_aerophilum_IM2_uid57727	NC_003364	chromosome		arCOG01418	2 (-)	M	RfaG	Glycosyltransferase	2E-11	COG0438	Glycosyltransferase
18311938	hypothetical protein PAE0432	No	7		235050	236390	-		Pyrobaculum_aerophilum_IM2_uid57727	NC_003364	chromosome		arCOG05429	1	S		Uncharacterized conserved protein			
18311939	hypothetical protein PAE0433	No	1		236353	236883	+		Pyrobaculum_aerophilum_IM2_uid57727	NC_003364	chromosome		arCOG05430	1	S		Uncharacterized conserved protein			
18311940	hypothetical protein PAE0434	No	2		236871	237203	-		Pyrobaculum_aerophilum_IM2_uid57727	NC_003364	chromosome									
18311941	hypothetical protein PAE0435	No	6		237185	237970	-		Pyrobaculum_aerophilum_IM2_uid57727	NC_003364	chromosome		arCOG07688	1	S		Uncharacterized conserved membrane protein			
18311942	hypothetical protein PAE0436	No	14		237967	239244	-		Pyrobaculum_aerophilum_IM2_uid57727	NC_003364	chromosome		arCOG02214	2 (-)	R	RfbX	Polysaccharide biosynthesis protein, Mvin family	0.000001	pfam04955	HupE / UreJ protein. This family of prot
===								24	Pyrobaculum_aerophilum_IM2_uid57727	NC_003364	chromosome									
18311950	hypothetical protein PAE0445	No	12		246298	249042	+		Pyrobaculum_aerophilum_IM2_uid57727	NC_003364	chromosome									
18311951	glycosyltransferase (type 2)	No	1		249094	249921	+		Pyrobaculum_aerophilum_IM2_uid57727	NC_003364	chromosome		arCOG01381	4 (-)	M	WcaA	Glycosyltransferase	6E-13	pfam00535	Glycosyl transferase family 2. Diverse f
18311952	hypothetical protein PAE0455	No	8		251058	252212	-		Pyrobaculum_aerophilum_IM2_uid57727	NC_003364	chromosome									
18311953	hypothetical protein PAE0457	Yes	1		252334	252462	+		Pyrobaculum_aerophilum_IM2_uid57727	NC_003364	chromosome									
18311954	hypothetical protein PAE0458	No	0		252540	252743	+		Pyrobaculum_aerophilum_IM2_uid57727	NC_003364	chromosome									
18311955	hypothetical protein PAE0459	No	0		252772	254286	+		Pyrobaculum_aerophilum_IM2_uid57727	NC_003364	chromosome									
18311956	hypothetical protein PAE0462	No	1		255103	255459	+		Pyrobaculum_aerophilum_IM2_uid57727	NC_003364	chromosome									
18311957	hypothetical protein PAE0463	No	1		255493	256293	+		Pyrobaculum_aerophilum_IM2_uid57727	NC_003364	chromosome		arCOG07689	2	S		Uncharacterized conserved protein			
18311958	hypothetical protein PAE0465	No	0		256569	257102	+		Pyrobaculum_aerophilum_IM2_uid57727	NC_003364	chromosome									
18311959	hypothetical protein PAE0466	No	1		257226	258146	+		Pyrobaculum_aerophilum_IM2_uid57727	NC_003364	chromosome									
18311960	glycosyltransferase (type 1)	No	0		258622	258960	-		Pyrobaculum_aerophilum_IM2_uid57727	NC_003364	chromosome		arCOG01403	4 (-)	M	RfaG	Glycosyltransferase	0.000000004	COG0438	Glycosyltransferase
18311961	hypothetical protein PAE0468	No	0	YES	259081	259293	-		Pyrobaculum_aerophilum_IM2_uid57727	NC_003364	chromosome									
18311962	hypothetical protein PAE0469	No	0	YES	259434	259526	-		Pyrobaculum_aerophilum_IM2_uid57727	NC_003364	chromosome									
18311963	hypothetical protein PAE0470	No	0	YES	259516	259680	+		Pyrobaculum_aerophilum_IM2_uid57727	NC_003364	chromosome									
18311964	hypothetical protein PAE0471	No	0	YES	259809	259961	+		Pyrobaculum_aerophilum_IM2_uid57727	NC_003364	chromosome									
18311965	hypothetical protein PAE0473	No	0	YES	260079	260423	+		Pyrobaculum_aerophilum_IM2_uid57727	NC_003364	chromosome									
18311966	hypothetical protein PAE0475	No	0	YES	260877	260975	-		Pyrobaculum_aerophilum_IM2_uid57727	NC_003364	chromosome									
18311967	hypothetical protein PAE0476	No	0	YES	261049	261144	-		Pyrobaculum_aerophilum_IM2_uid57727	NC_003364	chromosome									
18311968	hypothetical protein PAE0477	No	1	YES	261302	261634	+		Pyrobaculum_aerophilum_IM2_uid57727	NC_003364	chromosome									
18311969	hypothetical protein PAE0478	Yes	0	YES	261627	264002	+		Pyrobaculum_aerophilum_IM2_uid57727	NC_003364	chromosome									
18311970	hypothetical protein PAE0479	No	0	YES	264309	264434	-		Pyrobaculum_aerophilum_IM2_uid57727	NC_003364	chromosome									
18311971	paREP15, putative coiled-coil protein	No	0	YES	264825	265244	-		Pyrobaculum_aerophilum_IM2_uid57727	NC_003364	chromosome		arCOG03724	3 (-)	S		KID-like repeats containing protein, paREP15 fan	0.0000005	COG1196	Chromosome segregation ATPases
18311972	hypothetical protein PAE0482	No	1	YES	265267	265407	-		Pyrobaculum_aerophilum_IM2_uid57727	NC_003364	chromosome									
18311973	hypothetical protein PAE0485	No	0	YES	266150	266257	+		Pyrobaculum_aerophilum_IM2_uid57727	NC_003364	chromosome									
===								8	Pyrobaculum_aerophilum_IM2_uid57727	NC_003364	chromosome									
18311982	hypothetical protein PAE0499	No	0	YES	271587	271790	+		Pyrobaculum_aerophilum_IM2_uid57727	NC_003364	chromosome									
18311983	hypothetical protein PAE0505	No	0	YES	273325	273663	+		Pyrobaculum_aerophilum_IM2_uid57727	NC_003364	chromosome		arCOG04018	2	S		Uncharacterized conserved protein			
18311984	paREP15, putative coiled-coil protein	No	0		273975	274706	+		Pyrobaculum_aerophilum_IM2_uid57727	NC_003364	chromosome		arCOG03724	3 (-)	S		KID-like repeats containing protein, paREP15 fan	2E-16	COG1196	Chromosome segregation ATPases
18311985	hypothetical protein PAE0507	No	0		275620	275802	+		Pyrobaculum_aerophilum_IM2_uid57727	NC_003364	chromosome									
18311986	hypothetical protein PAE0510	No	0		276135	276323	+		Pyrobaculum_aerophilum_IM2_uid57727	NC_003364	chromosome									
18311987	hypothetical protein PAE0512	No	0		276685	277419	-		Pyrobaculum_aerophilum_IM2_uid57727	NC_003364	chromosome		arCOG06994	2	S		Uncharacterized conserved protein			
18311988	hypothetical protein PAE0513	No	0		277542	278759	-		Pyrobaculum_aerophilum_IM2_uid57727	NC_003364	chromosome		arCOG06995	2 (-)	R		Predicted ATPase			
18311989	hypothetical protein PAE0514	No	0		278747	279382	-		Pyrobaculum_aerophilum_IM2_uid57727	NC_003364	chromosome		arCOG06996	2	S		Uncharacterized conserved protein			
===								7	Pyrobaculum_aerophilum_IM2_uid57727	NC_003364	chromosome									
18312005	hypothetical protein PAE0545	No	0		293608	294663	-		Pyrobaculum_aerophilum_IM2_uid57727	NC_003364	chromosome									
18312006	hypothetical protein PAE0546	Yes	0		294660	295943	-		Pyrobaculum_aerophilum_IM2_uid57727	NC_003364	chromosome		arCOG01522	1 (-)	H	HemY	Protoporphyrinogen oxidase	6E-26	COG1232	Protoporphyrinogen oxidase
18312007	paREP7	No	0		296071	296583	-		Pyrobaculum_aerophilum_IM2_uid57727	NC_003364	chromosome									
18312008	paREP7	No	0		297210	297422	+		Pyrobaculum_aerophilum_IM2_uid57727	NC_003364	chromosome									
18312009	hypothetical protein PAE0551	No	0		297425	297511	+		Pyrobaculum_aerophilum_IM2_uid57727	NC_003364	chromosome									
18312010	hypothetical protein PAE0552	No	0		297519	297857	+		Pyrobaculum_aerophilum_IM2_uid57727	NC_003364	chromosome		arCOG01424	2 (-)	R		RecB-nuclease fused to coiled-coil domain	0.00000004	pfam07788	Protein of unknown function (DUF1626). T
18312011	hypothetical protein PAE0555	No	1		298492	298839	+		Pyrobaculum_aerophilum_IM2_uid57727	NC_003364	chromosome		arCOG09769	1	S		Uncharacterized conserved protein			
===								5	Pyrobaculum_aerophilum_IM2_uid57727	NC_003364	chromosome									
18312336	hypothetical protein PAE0995	No	0		581705	582028	-		Pyrobaculum_aerophilum_IM2_uid57727	NC_003364	chromosome									
18312337	hypothetical protein PAE0996	No	0		582050	582586	-		Pyrobaculum_aerophilum_IM2_uid57727	NC_003364	chromosome									
18312338	hypothetical protein PAE0998	No	0		582758	584212	-		Pyrobaculum_aerophilum_IM2_uid57727	NC_003364	chromosome		arCOG04246	2 (-)	J	RtcB	RNA 3'-P ligase, RtcB family protein	1E-167	pfam01139	Uncharacterized protein family UPF0027.
18312339	hypothetical protein PAE0999	Yes	1		584454	586655	+		Pyrobaculum_aerophilum_IM2_uid57727	NC_003364	chromosome		arCOG07691	7	S		Uncharacterized conserved protein			
18312340	hypothetical protein PAE1000	No	5		586648	587178	+		Pyrobaculum_aerophilum_IM2_uid57727	NC_003364	chromosome		arCOG09771	3	S		Uncharacterized conserved protein			
===								6	Pyrobaculum_aerophilum_IM2_uid57727	NC_003364	chromosome									
18312362	hypothetical protein PAE1031	No	0		604504	605109	+		Pyrobaculum_aerophilum_IM2_uid57727	NC_003364	chromosome		arCOG09772	4	S		Uncharacterized conserved protein			
18312363	hypothetical protein PAE1032	No	1		605091	605519	+		Pyrobaculum_aerophilum_IM2_uid57727	NC_003364	chromosome		arCOG09773	4	S		Uncharacterized conserved protein			
18312364	hypothetical protein PAE1033	No	0		605503	605901	+		Pyrobaculum_aerophilum_IM2_uid57727	NC_003364	chromosome		arCOG09773	4	S		Uncharacterized conserved protein			
18312365	hypothetical protein PAE1034	No	0		605904	606098	-		Pyrobaculum_aerophilum_IM2_uid57727	NC_003364	chromosome		arCOG02734	5 (-)	S		NifX family protein	0.000001	COG1433	Uncharacterized conserved protein
18312366	hypothetical protein PAE1035	Yes	1		606138	606962	+		Pyrobaculum_aerophilum_IM2_uid57727	NC_003364	chromosome		arCOG09772	4	S		Uncharacterized conserved protein			
18312367	hypothetical protein PAE1036	No	1		606944	607762	+		Pyrobaculum_aerophilum_IM2_uid57727	NC_003364	chromosome		arCOG09773	4	S		Uncharacterized conserved protein			
===								7	Pyrobaculum_aerophilum_IM2_uid57727	NC_003364	chromosome									
18312391	paREP2b	No	0		629615	630046	+		Pyrobaculum_aerophilum_IM2_uid57727	NC_003364	chromosome							3E-24	pfam07775	PaRep2b protein. This is a family of pro

	18312392	hypothetical protein PAE1076	No	0		630056	630787	+		Pyrobaculum_aerophilum_IM2_uid57727	NC_003364	chromosome		arCOG05481	2 (-)	L		Transposase			
	18312393	hypothetical protein PAE1077	Yes	1		630955	633108	+		Pyrobaculum_aerophilum_IM2_uid57727	NC_003364	chromosome		arCOG07012	6	S		Uncharacterized conserved protein			
	18312394	hypothetical protein PAE1078	No	6		633095	633655	+		Pyrobaculum_aerophilum_IM2_uid57727	NC_003364	chromosome	clust_36								
	18312395	hypothetical protein PAE1079	Yes	1		633688	636009	+		Pyrobaculum_aerophilum_IM2_uid57727	NC_003364	chromosome		arCOG09761	8	S		Uncharacterized conserved protein			
	18312396	hypothetical protein PAE1081	No	4		636089	636517	+		Pyrobaculum_aerophilum_IM2_uid57727	NC_003364	chromosome									
	18312397	hypothetical protein PAE1082	No	0		636514	637401	-		Pyrobaculum_aerophilum_IM2_uid57727	NC_003364	chromosome		arCOG01290	5 (-)	L	SplB	DNA repair photolyase	2E-58	COG1533	DNA repair photolyase
===									8	Pyrobaculum_aerophilum_IM2_uid57727	NC_003364	chromosome									
	18312399	hypothetical protein PAE1086	No	0		638148	638492	-		Pyrobaculum_aerophilum_IM2_uid57727	NC_003364	chromosome		arCOG09798	1	S		Uncharacterized conserved protein			
	18312400	hypothetical protein PAE1087	No	0		638625	639257	-		Pyrobaculum_aerophilum_IM2_uid57727	NC_003364	chromosome		arCOG01145	2 (-)	R		Icc family phosphoesterase	1E-43	COG2129	Predicted phosphoesterases, related to t
	18312401	hypothetical protein PAE1089	Yes	0		639505	641625	+		Pyrobaculum_aerophilum_IM2_uid57727	NC_003364	chromosome		arCOG07691	7	S		Uncharacterized conserved protein			
	18312402	hypothetical protein PAE1092	No	5		641603	642157	+		Pyrobaculum_aerophilum_IM2_uid57727	NC_003364	chromosome		arCOG09775	2	S		Uncharacterized conserved protein			
	18312403	hypothetical protein PAE1095	Yes	1		642760	644886	+		Pyrobaculum_aerophilum_IM2_uid57727	NC_003364	chromosome		arCOG07012	6	S		Uncharacterized conserved protein			
	18312404	hypothetical protein PAE1096	No	6		644903	645397	+		Pyrobaculum_aerophilum_IM2_uid57727	NC_003364	chromosome	clust_215								
	18312405	integral membrane protein (rhomboid related),	No	6		646298	646990	+		Pyrobaculum_aerophilum_IM2_uid57727	NC_003364	chromosome		arCOG01768	2 (-)	E	GlpG	Membrane associated serine protease	2E-34	COG0705	Membrane associated serine protease
	18312406	hypothetical protein PAE1101	No	0	YES	647086	647733	+		Pyrobaculum_aerophilum_IM2_uid57727	NC_003364	chromosome		arCOG05483	2	S		Uncharacterized conserved protein			
===									8	Pyrobaculum_aerophilum_IM2_uid57727	NC_003364	chromosome									
	18312465	paREP2b	No	0		699818	700714	-		Pyrobaculum_aerophilum_IM2_uid57727	NC_003364	chromosome		arCOG07952	4 (-)	S		PaRep2b protein, some fused to other mobile dc	2E-137	pfam07775	PaRep2b protein. This is a family of pro
	18312466	hypothetical protein PAE1192	No	0		700843	700953	-		Pyrobaculum_aerophilum_IM2_uid57727	NC_003364	chromosome									
	18312467	hypothetical protein PAE1194	No	0		700969	701499	-		Pyrobaculum_aerophilum_IM2_uid57727	NC_003364	chromosome									
	18312468	hypothetical protein PAE1195	No	0		701485	701787	+		Pyrobaculum_aerophilum_IM2_uid57727	NC_003364	chromosome									
	18312469	hypothetical protein PAE1197	No	5		702031	702585	-		Pyrobaculum_aerophilum_IM2_uid57727	NC_003364	chromosome							0.0000001	COG3559	Putative exporter of polyketide antibiot
	18312470	ABC transporter ATP-binding protein, putative	No	0		702708	703394	-		Pyrobaculum_aerophilum_IM2_uid57727	NC_003364	chromosome		arCOG00194	14 (-)	V	CcmA	ABC-type multidrug transport system, ATPase co	2E-33	COG1131	ABC-type multidrug transport system, ATP
	18312471	hypothetical protein PAE1200	No	0		703671	703901	-		Pyrobaculum_aerophilum_IM2_uid57727	NC_003364	chromosome									
	18312472	paREP2a	No	0		704053	704322	+		Pyrobaculum_aerophilum_IM2_uid57727	NC_003364	chromosome	clust_371						6E-43	pfam07903	PaRep2a protein. This is a family of pro
===									17	Pyrobaculum_aerophilum_IM2_uid57727	NC_003364	chromosome									
	18312616	hypothetical protein PAE1406	No	0		838988	839398	+		Pyrobaculum_aerophilum_IM2_uid57727	NC_003364	chromosome		arCOG05501	3 (-)	S		Uncharacterized conserved protein			
	18312617	hypothetical protein PAE1407	Yes	3		839519	840619	+		Pyrobaculum_aerophilum_IM2_uid57727	NC_003364	chromosome		arCOG09779	3	S		Uncharacterized conserved protein			
	18312618	hypothetical protein PAE1408	No	5		840609	841052	+		Pyrobaculum_aerophilum_IM2_uid57727	NC_003364	chromosome	clust_208								
	18312619	hypothetical protein PAE1409	No	4		841049	841765	+		Pyrobaculum_aerophilum_IM2_uid57727	NC_003364	chromosome	clust_139								
	18312620	paREP6, authentic frameshift	No	0		842580	842750	-		Pyrobaculum_aerophilum_IM2_uid57727	NC_003364	chromosome									
	18312621	hypothetical protein PAE1414	No	4		842991	843461	+		Pyrobaculum_aerophilum_IM2_uid57727	NC_003364	chromosome		arCOG07028	1	S		Uncharacterized conserved protein			
	18312622	hypothetical protein PAE1418	No	0		843676	844065	+		Pyrobaculum_aerophilum_IM2_uid57727	NC_003364	chromosome		arCOG03725	1 (-)	S		Uncharacterized conserved protein	0.00000004	pfam04156	IncA protein. Chlamydia trachomatis is a
	18312623	hypothetical protein PAE1419	No	1		844347	844502	-		Pyrobaculum_aerophilum_IM2_uid57727	NC_003364	chromosome									
	18312624	hypothetical protein PAE1420	No	0		844477	844605	-		Pyrobaculum_aerophilum_IM2_uid57727	NC_003364	chromosome									
	18312625	hypothetical protein PAE1421	No	3		844790	845050	+		Pyrobaculum_aerophilum_IM2_uid57727	NC_003364	chromosome									
	18312626	hypothetical protein PAE1422	No	0		845031	845528	+		Pyrobaculum_aerophilum_IM2_uid57727	NC_003364	chromosome									
	18312627	carbon starvation protein A-like protein	Yes	14		845502	847358	-		Pyrobaculum_aerophilum_IM2_uid57727	NC_003364	chromosome		arCOG04286	2 (-)	T	CstA	Carbon starvation protein, predicted membrane p	6E-143	COG1966	Carbon starvation protein, predicted mem
	18312628	hypothetical protein PAE1425	No	0		847432	847758	+		Pyrobaculum_aerophilum_IM2_uid57727	NC_003364	chromosome							0.00009	pfam12008	Type I restriction and modification enzy
	18312629	arsenical pump-driving ATPase	No	0		847981	848883	+		Pyrobaculum_aerophilum_IM2_uid57727	NC_003364	chromosome		arCOG02849	1 (-)	P	ArsA	Oxyanion-translocating ATPase	9E-60	COG0003	Predicted ATPase involved in chromosome
	18312630	hypothetical protein PAE1429	No	0		849158	849253	-		Pyrobaculum_aerophilum_IM2_uid57727	NC_003364	chromosome									
	18312631	hypothetical protein PAE1430	No	2		849549	849719	-		Pyrobaculum_aerophilum_IM2_uid57727	NC_003364	chromosome		arCOG09762	4	S		Uncharacterized conserved protein			
	18312632	hypothetical protein PAE1432	No	2		849883	850365	+		Pyrobaculum_aerophilum_IM2_uid57727	NC_003364	chromosome		arCOG09765	4	S		Uncharacterized conserved protein			
===									5	Pyrobaculum_aerophilum_IM2_uid57727	NC_003364	chromosome									
	18312647	paREP2a	No	0	YES	860907	861059	-		Pyrobaculum_aerophilum_IM2_uid57727	NC_003364	chromosome							2E-15	pfam07903	PaRep2a protein. This is a family of pro
	18312648	paREP2a	No	0	YES	861206	861322	-		Pyrobaculum_aerophilum_IM2_uid57727	NC_003364	chromosome									
	18312649	paREP9, associated with SRSRs, authentic fra	No	0	YES	861572	861769	-		Pyrobaculum_aerophilum_IM2_uid57727	NC_003364	chromosome		arCOG05421	3 (-)	S		paREP9 family			
	18312650	hypothetical protein PAE1461	No	0	YES	861756	862166	-		Pyrobaculum_aerophilum_IM2_uid57727	NC_003364	chromosome									
	18312651	hypothetical protein PAE1463	No	0	YES	862318	862809	+		Pyrobaculum_aerophilum_IM2_uid57727	NC_003364	chromosome		arCOG05506	1	S		Uncharacterized conserved protein			
===									5	Pyrobaculum_aerophilum_IM2_uid57727	NC_003364	chromosome									
	18312677	hypothetical protein PAE1507a	No	0		882379	882468	+		Pyrobaculum_aerophilum_IM2_uid57727	NC_003364	chromosome									
	18312678	hypothetical protein PAE1509	No	0		882559	882759	+		Pyrobaculum_aerophilum_IM2_uid57727	NC_003364	chromosome									
	18312679	hypothetical protein PAE1511	No	0		882804	883133	+		Pyrobaculum_aerophilum_IM2_uid57727	NC_003364	chromosome									
	18312680	translation initiation factor IF-2	No	0		883670	885439	-		Pyrobaculum_aerophilum_IM2_uid57727	NC_003364	chromosome		arCOG01560	1 (-)	J	InfB	Translation initiation factor 2 (IF-2; GTPase)	2E-69	COG0532	Translation initiation factor 2 (IF-2; G
	18312681	paREP2b	No	0		885579	886106	-		Pyrobaculum_aerophilum_IM2_uid57727	NC_003364	chromosome							1E-73	pfam07775	PaRep2b protein. This is a family of pro
===									9	Pyrobaculum_aerophilum_IM2_uid57727	NC_003364	chromosome									
	18312742	hypothetical protein PAE1599	No	5		938361	938972	-		Pyrobaculum_aerophilum_IM2_uid57727	NC_003364	chromosome		arCOG02298	6 (-)	N	FlaK/PulC	Peptidase A24A, prepilin type IV			
	18312743	paREP2b	No	0		939053	939412	-		Pyrobaculum_aerophilum_IM2_uid57727	NC_003364	chromosome		arCOG09780	7	S		Uncharacterized conserved protei, PaRep2b family			
	18312744	paREP2b	No	0		939419	939613	-		Pyrobaculum_aerophilum_IM2_uid57727	NC_003364	chromosome		arCOG09780	7	S		Uncharacterized conserved protei, PaRep2b fam	0.00007	pfam07775	PaRep2b protein. This is a family of pro
	18312745	paREP2b	No	0		939589	939756	-		Pyrobaculum_aerophilum_IM2_uid57727	NC_003364	chromosome							0.0000006	pfam07775	PaRep2b protein. This is a family of pro
	18312746	paREP2b	No	0		939810	939941	-		Pyrobaculum_aerophilum_IM2_uid57727	NC_003364	chromosome									
	18312747	paREP2b	No	0		939963	940070	-		Pyrobaculum_aerophilum_IM2_uid57727	NC_003364	chromosome									
	18312748	paREP2a	No	0		940116	940208	+		Pyrobaculum_aerophilum_IM2_uid57727	NC_003364	chromosome									
	18312749	paREP2a	No	0		940301	940609	+		Pyrobaculum_aerophilum_IM2_uid57727	NC_003364	chromosome	clust_282						5E-31	pfam07903	PaRep2a protein. This is a family of pro
	18312750	bifunctional phosphoglucose/phosphomannos	No	0		940791	941699	-		Pyrobaculum_aerophilum_IM2_uid57727	NC_003364	chromosome		arCOG00052	1 (-)	G	Pgi	Glucose-6-phosphate isomerase	6E-11	pfam10432	Bacterial phospho-glucose isomerase C-te
===									9	Pyrobaculum_aerophilum_IM2_uid57727	NC_003364	chromosome									
	18312828	propionate catabolism protein prpD	No	0		1003035	1004396	+		Pyrobaculum_aerophilum_IM2_uid57727	NC_003364	chromosome		arCOG04285	1 (-)	R	PrpD	Uncharacterized protein involved in propionate c	1E-87	COG2079	Uncharacterized protein involved in prop
	18312829	hypothetical protein PAE1717a	No	0		1004477	1004593	-		Pyrobaculum_aerophilum_IM2_uid57727	NC_003364	chromosome									
	18312830	hypothetical protein PAE1718	Yes	0		1005028	1007094	+		Pyrobaculum_aerophilum_IM2_uid57727	NC_003364	chromosome		arCOG07012	6	S		Uncharacterized conserved protein			
	18312831	hypothetical protein PAE1719	No	6		1007099	1007626	+		Pyrobaculum_aerophilum_IM2_uid57727	NC_003364	chromosome									
	18312832	hypothetical protein PAE1720	Yes	1		1008019	1008204	+		Pyrobaculum_aerophilum_IM2_uid57727	NC_003364	chromosome									
	18312833	hypothetical protein PAE1722	Yes	3		1008195	1009070	+		Pyrobaculum_aerophilum_IM2_uid57727	NC_003364	chromosome		arCOG09781	3	S		Uncharacterized conserved protein			
	18312834	hypothetical protein PAE1723	Yes	0		1009425	1009997	+		Pyrobaculum_aerophilum_IM2_uid57727	NC_003364	chromosome		arCOG09782	4	S		Uncharacterized conserved protein			
	18312835	thioredoxin reductase (trxB)	No	1		1010358	1011341	+		Pyrobaculum_aerophilum_IM2_uid57727	NC_003364	chromosome		arCOG01296	3 (-)	O	TrxB	Thioredoxin reductase	4E-72	COG0492	Thioredoxin reductase
	18312836	hypothetical protein PAE1726	Yes	1		1011361	1012242	-		Pyrobaculum_aerophilum_IM2_uid57727	NC_003364	chromosome		arCOG09782	4	S		Uncharacterized conserved protein			
===									8	Pyrobaculum_aerophilum_IM2_uid57727	NC_003364	chromosome									
	18312859	GTP-binding protein	No	0		1032792	1034345	-		Pyrobaculum_aerophilum_IM2_uid57727	NC_003364	chromosome		arCOG01562	1 (-)	R	GTPBP1	GTPase		COG5258	GTPase
	18312860	hypothetical protein PAE1765	No	1		1034552	1034653	-		Pyrobaculum_aerophilum_IM2_uid57727	NC_003364	chromosome									
	18312861	paREP2a	No	0		1034650	1034772	-		Pyrobaculum_aerophilum_IM2_uid57727	NC_003364	chromosome									
	18312862	paREP2a	No	0		1034745	1034996	-		Pyrobaculum_aerophilum_IM2_uid57727	NC_003364	chromosome									
	18312863	paREP2a	No	0		1034986	1035363	-		Pyrobaculum_aerophilum_IM2_uid57727	NC_003364	chromosome	clust_282						1E-59	pfam07903	PaRep2a protein. This is a family of pro
	18312864	paREP2b	No	0		1035524	1038262	+		Pyrobaculum_aerophilum_IM2_uid57727	NC_003364	chromosome		arCOG09783	2	S		paREP2b family protein			
	18312865	membrane-bound proton-translocating pyrophc	No	16		1038248	1040413	-		Pyrobaculum_aerophilum_IM2_uid57727	NC_003364	chromosome		arCOG04949	1 (-)	C	OVP1	Inorganic pyrophosphatase		COG3808	Inorganic pyrophosphatase
	18312866	paREP2a	No	0		1040512	1040724	+		Pyrobaculum_aerophilum_IM2_uid57727	NC_003364	chromosome							7E-18	pfam07903	PaRep2a protein. This is a family of pro
===									7	Pyrobaculum_aerophilum_IM2_uid57727	NC_003364	chromosome									
	18312888	4-aminobutyrate aminotransferase	No	0		1057424	1058770	-		Pyrobaculum_aerophilum_IM2_uid57727	NC_003364	chromosome		arCOG00915	2 (-)	E	GabT	4-aminobutyrate aminotransferase or related amir	2E-153	COG0160	4-aminobutyrate aminotransferase and rel
	18312889	hypothetical protein PAE1802	Yes	1		1059666	1061771	+		Pyrobaculum_aerophilum_IM2_uid57727	NC_003364	chromosome		arCOG07691	7	S		Uncharacterized conserved protein			
	18312890	hypothetical protein PAE1803	No	4		1061749	1062387	+		Pyrobaculum_aerophilum_IM2_uid57727	NC_003364	chromosome		arCOG09784	2	S		Uncharacterized conserved protein			
	18312891	hypothetical protein PAE1803a	No	1		1062448	1062879	+		Pyrobaculum_aerophilum_IM2_uid57727	NC_003364	chromosome		arCOG09785	1	S		Uncharacterized conserved protein			
	18312892	hypothetical protein PAE1804	No	1		1062882	1063499	+		Pyrobaculum_aerophilum_IM2_uid57727	NC_003364	chromosome		arCOG07695	1	S		Uncharacterized conserved protein			
	18312893	hypothetical protein PAE1805	No	0		1063500	1063745	+		Pyrobaculum_aerophilum_IM2_uid57727	NC_003364	chromosome									
	18312894	hypothetical protein PAE1807	No	0		1064077	1064358	-		Pyrobaculum_aerophilum_IM2_uid57727	NC_003364	chromosome		arCOG00915	2 (-)	E	GabT	4-aminobutyrate aminotransferase or related aminotransferase			
===									8	Pyrobaculum_aerophilum_IM2_uid57727	NC_003364	chromosome									
	18312904	hypothetical protein PAE1819	Yes	1		1071252	1072682	+		Pyrobaculum_aerophilum_IM2_uid57727	NC_003364	chromosome		arCOG03377	4 (-)	E		Serine protease			
	18312905	hypothetical protein PAE1820	No	1		1072651	1073472	+		Pyrobaculum_aerophilum_IM2_uid57727	NC_003364	chromosome		arCOG09786	1	S		Uncharacterized conserved protein			
	18312906	hypothetical protein PAE1823	No	0		1073733	1073909	-		Pyrobaculum_aerophilum_IM2_uid57727	NC_003364	chromosome		arCOG09801	3	S		Uncharacterized conserved protein			
	18312907	hypothetical protein PAE1824	No	0		1074027	1074869	-		Pyrobaculum_aerophilum_IM2_uid57727	NC_003364	chromosome		arCOG09787	1	S		Uncharacterized conserved protein			
	18312908	hypothetical protein PAE1825	Yes	1		1074889	1075476	-		Pyrobaculum_aerophilum_IM2_uid57727	NC_003364	chromosome		arCOG09788	1	S		Uncharacterized conserved protein			
	18312909	hypothetical protein PAE1826	No	0		1075466	1075894	-		Pyrobaculum_aerophilum_IM2_uid57727	NC_003364	chromosome									
	18312910	paREP12	No	0		1076402	1077628	+		Pyrobaculum_aerophilum_IM2_uid57727	NC_003364	chromosome		arCOG00687	3 (-)	L		Transposase	0.00001	COG0675	Transposase and inactivated derivatives
	18312911	paREP13	No	1		1077809	1078321	+		Pyrobaculum_aerophilum_IM2_uid57727	NC_003364	chromosome									
===									6	Pyrobaculum_aerophilum_IM2_uid57727	NC_003364	chromosome									
	18313069	hypothetical protein PAE2059	No	0		1214918	1216144	-		Pyrobaculum_aerophilum_IM2_uid57727	NC_003364	chromosome		arCOG00442	1 (-)	R		Uncharacterized protein containing a von Willebra	4E-29	COG2425	Uncharacterized protein containing a von
	18313070	hypothetical protein PAE2060	No	0		1216201	1217016	-		Pyrobaculum_aerophilum_IM2_uid57727	NC_003364	chromosome		arCOG09791	2	S		Uncharacterized conserved protein			
	18313071	hypothetical protein PAE2062	Yes	1		1217017	1217382	-		Pyrobaculum_aerophilum_IM2_uid57727	NC_003364	chromosome									
	18313072	paREP2b	No	0		1217956	1218432	-		Pyrobaculum_aerophilum_IM2_uid57727	NC_003364	chromosome		arCOG09780	7	S		Uncharacterized conserved protei, PaRep2b fam	0.0000007	pfam07775	PaRep2b protein. This is a family of pro
	18313073	paREP2b	No	0		1218466	1218582	-		Pyrobaculum_aerophilum_IM2_uid57727	NC_003364	chromosome									
	18313074	hypothetical protein PAE2069	No	0		1219141	1219722	-		Pyrobaculum_aerophilum_IM2_uid57727	NC_003364	chromosome		arCOG05554	1 (-)	S		Uncharacterized conserved protein			
===									5	Pyrobaculum_aerophilum_IM2_uid57727	NC_003364	chromosome									
	18313101	paREP2a	No	0		1239626	1239766	+		Pyrobaculum_aerophilum_IM2_uid57727	NC_003364	chromosome									
	18313102	paREP2a	No	0		1239797	1240054	+		Pyrobaculum_aerophilum_IM2_uid57727	NC_003364	chromosome	clust_371						3E-38	pfam07903	PaRep2a protein. This is a family of pro
	18313103	DNA polymerase II	No	0		1240114	1242471	+		Pyrobaculum_aerophilum_IM2_uid57727	NC_003364	chromosome		arCOG00328	8 (-)	L	PolB	DNA polymerase elongation subunit (family B)		COG0417	DNA polymerase elongation subunit (famil
	18313104	hypothetical protein PAE2110	No	0		1242523	1242636	-		Pyrobaculum_aerophilum_IM2_uid57727	NC_003364	chromosome									
	18313105	paREP2a	No	0		1242694	1242858	+		Pyrobaculum_aerophilum_IM2_uid57727	NC_003364	chromosome									
===									9	Pyrobaculum_aerophilum_IM2_uid57727	NC_003364	chromosome									
	18313126	hypothetical protein PAE2138	No	0		1261955	1262848	+		Pyrobaculum_aerophilum_IM2_uid57727	NC_003364	chromosome		arCOG04155	2 (-)	L		HhH-GPD superfamily base excision DNA repair protein			
	18313127	hypothetical protein PAE2138a	No	0		1263016	1263324	-		Pyrobaculum_aerophilum_IM2_uid57727	NC_003364	chromosome									
	18313128	hypothetical protein PAE2140	Yes	1		1263958	1264134	+		Pyrobaculum_aerophilum_IM2_uid57727	NC_003364	chromosome									
	18313129	hypothetical protein PAE2142	No	0		1264390	1264566	+		Pyrobaculum_aerophilum_IM2_uid57727	NC_003364	chromosome									
	18313130	hypothetical protein PAE2144	No	0		1264883	1265122	-		Pyrobaculum_aerophilum_IM2_uid57727	NC_003364	chromosome		arCOG09789	1	S		Uncharacterized conserved protein			
	18313131	hypothetical protein PAE2146	No	0		1265393	1265593	-		Pyrobaculum_aerophilum_IM2_uid57727	NC_003364	chromosome									
	18313132	hypothetical protein PAE2147	No	0		1265617	1266339	-		Pyrobaculum_aerophilum_IM2_uid57727	NC_003364	chromosome		arCOG00938	12 (-)	R		Radical SAM superfamily enzyme	0.00001	COG0535	Predicted Fe-S oxidoreductases
	18313133	hypothetical protein PAE2148	No	0		1266345	1266539	-		Pyrobaculum_aerophilum_IM2_uid57727	NC_003364	chromosome									
	18313134	hypothetical protein PAE2149	No	2		1266794	1267222	+		Pyrobaculum_aerophilum_IM2_uid57727	NC_003364	chromosome		arCOG07043	1	S		Uncharacterized conserved protein			
===									5	Pyrobaculum_aerophilum_IM2_uid57727	NC_003364	chromosome									
	18313841	hypothetical protein PAE3123	No	3		1859111	1859449	-		Pyrobaculum_aerophilum_IM2_uid57727	NC_003364	chromosome		arCOG07062	1	S		Uncharacterized conserved protein			
	18313842	penicillin amidase	No	1		1859433	1861523	-		Pyrobaculum_aerophilum_IM2_uid57727	NC_003364	chromosome		arCOG04082	1 (-)	R		Protein related to penicillin acylase	7E-106	COG2366	Protein related to penicillin acylase
	18313843	hypothetical protein PAE3125	No	5		1861604	1862137	-		Pyrobaculum_aerophilum_IM2_uid57727	NC_003364	chromosome									
	18313844	hypothetical protein PAE3127	No	2		1862107	1863570	-		Pyrobaculum_aerophilum_IM2_uid57727	NC_003364	chromosome		arCOG09761	8	S		Uncharacterized conserved protein			
	18313845	hypothetical protein PAE3129	Yes	0		1863667	1864431	-		Pyrobaculum_aerophilum_IM2_uid57727	NC_003364	chromosome		arCOG09761	8	S		Uncharacterized conserved protein			
===									5	Pyrobaculum_aerophilum_IM2_uid57727	NC_003364	chromosome									
	18313891	hypothetical protein PAE3193	No	1		1902925	1903014	+		Pyrobaculum_aerophilum_IM2_uid57727	NC_003364	chromosome									
	18313892	hypothetical protein PAE3193a	No	2		1903036	1903455	+		Pyrobaculum_aerophilum_IM2_uid57727	NC_003364	chromosome									
	18313893	hypothetical protein PAE3195	No	2		1903367	1903951	+		Pyrobaculum_aerophilum_IM2_uid57727	NC_003364	chromosome		arCOG01331	2 (-)	O	HtpX	Zn-dependent protease with chaperone function	1E-15	COG0501	Zn-dependent protease with chaperone fun
	18313894	hypothetical protein PAE3196	No	0		1904252	1905253	+		Pyrobaculum_aerophilum_IM2_uid57727	NC_003364	chromosome									
	18313895	hypothetical protein PAE3197	No	0		1905244	1907256	-		Pyrobaculum_aerophilum_IM2_uid57727	NC_003364	chromosome									
===									5	Pyrobaculum_aerophilum_IM2_uid57727	NC_003364	chromosome									
	18314115	hypothetical protein PAE3501a	No	0		2101487	2101543	-		Pyrobaculum_aerophilum_IM2_uid57727	NC_003364	chromosome									
	18314116	hypothetical protein PAE3502	Yes	0		2102079	2102543	+		Pyrobaculum_aerophilum_IM2_uid57727	NC_003364	chromosome		arCOG09806	2	S		Uncharacterized conserved protein			
	18314117	hypothetical protein PAE3503	No	4		2102893	2103480	+		Pyrobaculum_aerophilum_IM2_uid57727	NC_003364	chromosome		arCOG09807	2	S		Uncharacterized conserved protein			
	18314118	hypothetical protein PAE3504	No	0		2103456	2103695	+		Pyrobaculum_aerophilum_IM2_uid57727	NC_003364	chromosome		arCOG01061	2 (-)	K		Predicted transcriptional regulator			
	18314119	hypothetical protein PAE3506	No	1		2103787	2103987	+		Pyrobaculum_aerophilum_IM2_uid57727	NC_003364	chromosome									
===									9	Pyrobaculum_aerophilum_IM2_uid57727	NC_003364	chromosome									
	18314135	hypothetical protein PAE3532	No	0		2117092	2118300	-		Pyrobaculum_aerophilum_IM2_uid57727	NC_003364	chromosome		arCOG00281	4 (-)	R		Predicted ATPase	0.000002	pfam12846	AAA-like domain. This family of domains
	18314136	hypothetical protein PAE3533	No	0		2118384	2119985	-		Pyrobaculum_aerophilum_IM2_uid57727	NC_003364	chromosome							0.00000008	pfam06346	Formin Homology Region 1. This region is
	18314137	hypothetical protein PAE3535	Yes	1		2120034	2120825	-		Pyrobaculum_aerophilum_IM2_uid57727	NC_003364	chromosome									
	18314138	hypothetical protein PAE3538	No	0		2121046	2121861	-		Pyrobaculum_aerophilum_IM2_uid57727	NC_003364	chromosome									
	18314139	hypothetical protein PAE3540	Yes	0		2121970	2122689	-		Pyrobaculum_aerophilum_IM2_uid57727	NC_003364	chromosome									
	18314140	hypothetical protein PAE3541	No	0		2122854	2123009	-		Pyrobaculum_aerophilum_IM2_uid57727	NC_003364	chromosome									
	18314141	hypothetical protein PAE3542	No	0		2123333	2123773	+		Pyrobaculum_aerophilum_IM2_uid57727	NC_003364	chromosome									
	18314142	paREP2b	No	0		2124131	2125225	+		Pyrobaculum_aerophilum_IM2_uid57727	NC_003364	chromosome		arCOG09783	2	S		paREP2b family protein			
	18314143	paREP2b	No	0		2125232	2127118	+		Pyrobaculum_aerophilum_IM2_uid57727	NC_003364	chromosome		arCOG07952	4 (-)	S		PaRep2b protein, some fused to other mobile dc	1E-58	pfam07775	PaRep2b protein. This is a family of pro
===									6	Pyrobaculum_arsenaticum_DSM_13514_uid5840	NC_009376	chromosome									
	145590558	hypothetical protein Pars_0295	No	0		260183	260620	+		Pyrobaculum_arsenaticum_DSM_13514_uid5840	NC_009376	chromosome		arCOG01699	1 (-)	S		Uncharacterized conserved protein	3E-33	COG4353	Uncharacterized conserved protein
	145590559	hypothetical protein Pars_0296	Yes	0		260670	261797	-		Pyrobaculum_arsenaticum_DSM_13514_uid5840	NC_009376	chromosome	arCOG09780,a	arCOG09780	7	S		Uncharacterized conserved protei, PaRep2b fam	7E-31	pfam07775	PaRep2b protein. This is a family of pro
	145590560	hypothetical protein Pars_0297	No	0		261914	262108	+		Pyrobaculum_arsenaticum_DSM_13514_uid5840	NC_009376	chromosome									
	145590561	hypothetical protein Pars_0298	No	0		262138	262401	+		Pyrobaculum_arsenaticum_DSM_13514_uid5840	NC_009376	chromosome									
	145590562	hypothetical protein Pars_0299	No	0		262398	262883	+		Pyrobaculum_arsenaticum_DSM_13514_uid5840	NC_009376	chromosome	clust_354								
	145590563	acylphosphatase	No	0		263125	263412	-		Pyrobaculum_arsenaticum_DSM_13514_uid5840	NC_009376	chromosome		arCOG01674	1 (-)	C	AcyP	Acylphosphatase	8E-26	COG1254	Acylphosphatases
===									5	Pyrobaculum_arsenaticum_DSM_13514_uid5840	NC_009376	chromosome									
	145590619	hypothetical protein Pars_0364	No	0	YES	314595	315629	-		Pyrobaculum_arsenaticum_DSM_13514_uid5840	NC_009376	chromosome		arCOG09768	3	S		Uncharacterized conserved protein			
	145590620	hypothetical protein Pars_0365	No	0	YES	315642	316196	-		Pyrobaculum_arsenaticum_DSM_13514_uid5840	NC_009376	chromosome		arCOG09767	2	S		Uncharacterized conserved protein			
	145590621	hypothetical protein Pars_0366	No	0	YES	316430	316789	-		Pyrobaculum_arsenaticum_DSM_13514_uid5840	NC_009376	chromosome		arCOG05428	1 (-)	S		Uncharacterized conserved protein			
	145590622	hypothetical protein Pars_0367	No	0	YES	316927	317169	+		Pyrobaculum_arsenaticum_DSM_13514_uid5840	NC_009376	chromosome									
	145590623	hypothetical protein Pars_0368	No	3	YES	317412	317831	+		Pyrobaculum_arsenaticum_DSM_13514_uid5840	NC_009376	chromosome									
===									5	Pyrobaculum_arsenaticum_DSM_13514_uid5840	NC_009376	chromosome									
	145590667	hypothetical protein Pars_0418	No	1		367817	367957	-		Pyrobaculum_arsenaticum_DSM_13514_uid5840	NC_009376	chromosome		arCOG09762	4	S		Uncharacterized conserved protein			
	145590668	radical SAM domain-containing protein	No	0		367965	368978	-		Pyrobaculum_arsenaticum_DSM_13514_uid5840	NC_009376	chromosome		arCOG00938	12 (-)	R		Radical SAM superfamily enzyme	0.00000005	COG0535	Predicted Fe-S oxidoreductases
	145590669	hypothetical protein Pars_0420	No	2		368978	369235	-		Pyrobaculum_arsenaticum_DSM_13514_uid5840	NC_009376	chromosome		arCOG09763	2	S		Uncharacterized conserved protein			
	145590670	hypothetical protein Pars_0421	No	1		369232	369783	-		Pyrobaculum_arsenaticum_DSM_13514_uid5840	NC_009376	chromosome		arCOG09764	3	S		Uncharacterized conserved protein			
	145590671	hypothetical protein Pars_0422	No	2		369815	370294	+		Pyrobaculum_arsenaticum_DSM_13514_uid5840	NC_009376	chromosome		arCOG09765	4	S		Uncharacterized conserved protein			
===									5	Pyrobaculum_arsenaticum_DSM_13514_uid5840	NC_009376	chromosome									
	145590675	hypothetical protein Pars_0426	No	0	YES	372113	372964	-		Pyrobaculum_arsenaticum_DSM_13514_uid5840	NC_009376	chromosome									
	145590676	hypothetical protein Pars_0427	No	0	YES	372992	373222	-		Pyrobaculum_arsenaticum_DSM_13514_uid5840	NC_009376	chromosome									
	145590677	AsnC family transcriptional regulator	No	0		373219	373737	-		Pyrobaculum_arsenaticum_DSM_13514_uid5840	NC_009376	chromosome		arCOG01586	1 (-)	K	Lrp	Transcriptional regulator (Lrp/AsnC family)			
	145590678	hypothetical protein Pars_0429	No	5	YES	373886	374443	+		Pyrobaculum_arsenaticum_DSM_13514_uid5840	NC_009376	chromosome							0.00001	pfam06157	Protein of unknown function (DUF973). Th
	145590679	hypothetical protein Pars_0430	Yes	1	YES	374594	374869	+		Pyrobaculum_arsenaticum_DSM_13514_uid5840	NC_009376	chromosome									
===									5	Pyrobaculum_arsenaticum_DSM_13514_uid5840	NC_009376	chromosome									
	145590772	hypothetical protein Pars_0527	No	0		475336	476142	+		Pyrobaculum_arsenaticum_DSM_13514_uid5840	NC_009376	chromosome	clust_120								
	145590773	hypothetical protein Pars_0528	No	0		476344	476769	-		Pyrobaculum_arsenaticum_DSM_13514_uid5840	NC_009376	chromosome									
	145590774	hypothetical protein Pars_0529	No	0		477076	478158	-		Pyrobaculum_arsenaticum_DSM_13514_uid5840	NC_009376	chromosome									
	145590775	Rieske (2Fe-2S) domain-containing protein	No	0		478148	478420	-		Pyrobaculum_arsenaticum_DSM_13514_uid5840	NC_009376	chromosome		arCOG02852	1 (-)	P	{NirD}	Ferredoxin subunit of nitrite reductase or ring-hydroxylating dioxygenase			
	145590776	hypothetical protein Pars_0531	No	0		478407	479219	-		Pyrobaculum_arsenaticum_DSM_13514_uid5840	NC_009376	chromosome	clust_120								
===									5	Pyrobaculum_arsenaticum_DSM_13514_uid5840	NC_009376	chromosome									
	145590927	hypothetical protein Pars_0688	No	0		610345	610695	-		Pyrobaculum_arsenaticum_DSM_13514_uid5840	NC_009376	chromosome		arCOG02883	3	S		Uncharacterized conserved protein	0.000003	pfam02624	YcaO-like family.
	145590928	hypothetical protein Pars_0689	No	0		611092	611766	-		Pyrobaculum_arsenaticum_DSM_13514_uid5840	NC_009376	chromosome									
	145590929	hypothetical protein Pars_0690	No	3		611935	612828	-		Pyrobaculum_arsenaticum_DSM_13514_uid5840	NC_009376	chromosome									
	145590930	hypothetical protein Pars_0691	No	0		612821	613105	-		Pyrobaculum_arsenaticum_DSM_13514_uid5840	NC_009376	chromosome		arCOG00194	14 (-)	V	CcmA	ABC-type multidrug transport system, ATPase component			
	145590931	hypothetical protein Pars_0692	No	4		613514	614026	+		Pyrobaculum_arsenaticum_DSM_13514_uid5840	NC_009376	chromosome		arCOG09804	3	S		Uncharacterized conserved protein			
===									5	Pyrobaculum_arsenaticum_DSM_13514_uid5840	NC_009376	chromosome									
	145590993	hypothetical protein Pars_0757	Yes	0		676527	676997	-		Pyrobaculum_arsenaticum_DSM_13514_uid5840	NC_009376	chromosome		arCOG09806	2	S		Uncharacterized conserved protein			
	145590994	hypothetical protein Pars_0758	No	0		677032	677277	-		Pyrobaculum_arsenaticum_DSM_13514_uid5840	NC_009376	chromosome		arCOG01061	2 (-)	K		Predicted transcriptional regulator	0.00001	COG3432	Predicted transcriptional regulator
	145590995	hypothetical protein Pars_0759	Yes	4		677253	677834	-		Pyrobaculum_arsenaticum_DSM_13514_uid5840	NC_009376	chromosome		arCOG09807	2	S		Uncharacterized conserved protein			
	145590996	hypothetical protein Pars_0760	No	5		678062	678655	+		Pyrobaculum_arsenaticum_DSM_13514_uid5840	NC_009376	chromosome									
	145590997	hypothetical protein Pars_0761	No	2		678684	679097	+		Pyrobaculum_arsenaticum_DSM_13514_uid5840	NC_009376	chromosome		arCOG09810	1	S		Uncharacterized conserved protein			
===									6	Pyrobaculum_arsenaticum_DSM_13514_uid5840	NC_009376	chromosome									
	145591398	ATPase	No	0		1067114	1068337	-		Pyrobaculum_arsenaticum_DSM_13514_uid5840	NC_009376	chromosome		arCOG03166	9 (-)	R		AAA+ superfamily ATPase fused to HTH and RecB nuclease domains			
	145591399	hypothetical protein Pars_1178	No	0		1068653	1068901	+		Pyrobaculum_arsenaticum_DSM_13514_uid5840	NC_009376	chromosome									
	145591400	hypothetical protein Pars_1179	No	2		1068949	1069332	+		Pyrobaculum_arsenaticum_DSM_13514_uid5840	NC_009376	chromosome									
	145591401	hypothetical protein Pars_1180	No	5		1069434	1070081	+		Pyrobaculum_arsenaticum_DSM_13514_uid5840	NC_009376	chromosome									
	145591402	hypothetical protein Pars_1181	No	0		1070424	1070624	+		Pyrobaculum_arsenaticum_DSM_13514_uid5840	NC_009376	chromosome									
	145591403	PaREP1 domain-containing protein	No	0		1070963	1071337	-		Pyrobaculum_arsenaticum_DSM_13514_uid5840	NC_009376	chromosome		arCOG03722	3 (-)	V		HEPN domain containing protein			
===									8	Pyrobaculum_arsenaticum_DSM_13514_uid5840	NC_009376	chromosome									
	145591661	hypothetical protein Pars_1453	No	0		1308137	1308376	-		Pyrobaculum_arsenaticum_DSM_13514_uid5840	NC_009376	chromosome									
	145591662	hypothetical protein Pars_1454	No	0		1308656	1309960	-		Pyrobaculum_arsenaticum_DSM_13514_uid5840	NC_009376	chromosome		arCOG07455	1 (-)	S		Uncharacterized conserved protein			
	145591663	hypothetical protein Pars_1455	No	10		1310026	1314339	-		Pyrobaculum_arsenaticum_DSM_13514_uid5840	NC_009376	chromosome									
	145591664	hypothetical protein Pars_1456	No	0		1314593	1314838	-		Pyrobaculum_arsenaticum_DSM_13514_uid5840	NC_009376	chromosome									
	145591665	hypothetical protein Pars_1458	No	0		1316043	1316312	-		Pyrobaculum_arsenaticum_DSM_13514_uid5840	NC_009376	chromosome									
	145591666	hypothetical protein Pars_1459	No	0		1316412	1317197	-		Pyrobaculum_arsenaticum_DSM_13514_uid5840	NC_009376	chromosome		arCOG07064	1 (-)	S		Uncharacterized conserved protein			
	145591667	hypothetical protein Pars_1460	No	0		1317587	1317952	+		Pyrobaculum_arsenaticum_DSM_13514_uid5840	NC_009376	chromosome									
	145591668	hypothetical protein Pars_1461	No	2		1318043	1318399	-		Pyrobaculum_arsenaticum_DSM_13514_uid5840	NC_009376	chromosome									
===									9	Pyrobaculum_arsenaticum_DSM_13514_uid5840	NC_009376	chromosome									
	145591834	hypothetical protein Pars_1631	No	0		1470071	1470322	+		Pyrobaculum_arsenaticum_DSM_13514_uid5840	NC_009376	chromosome									
	145591835	hypothetical protein Pars_1632	No	4		1470357	1471685	+		Pyrobaculum_arsenaticum_DSM_13514_uid5840	NC_009376	chromosome									
	145591836	hypothetical protein Pars_1633	No	0		1472131	1473960	-		Pyrobaculum_arsenaticum_DSM_13514_uid5840	NC_009376	chromosome	arCOG01425,a	arCOG01425	1 (-)	R		RecB family nuclease with coiled-coil N-terminal c	1E-13	COG1196	Chromosome segregation ATPases
	145591837	hypothetical protein Pars_1634	No	1	YES	1474163	1476532	+		Pyrobaculum_arsenaticum_DSM_13514_uid5840	NC_009376	chromosome							7E-37	pfam14323	GxGYxY sequence motif in domain of unkno
	145591838	hypothetical protein Pars_1635	No	0	YES	1477036	1477428	-		Pyrobaculum_arsenaticum_DSM_13514_uid5840	NC_009376	chromosome									
	145591839	hypothetical protein Pars_1636	No	0	YES	1477635	1478549	+		Pyrobaculum_arsenaticum_DSM_13514_uid5840	NC_009376	chromosome									
	145591840	hypothetical protein Pars_1637	No	0	YES	1478546	1479142	+		Pyrobaculum_arsenaticum_DSM_13514_uid5840	NC_009376	chromosome									
	145591841	hypothetical protein Pars_1638	No	0	YES	1479319	1479603	+		Pyrobaculum_arsenaticum_DSM_13514_uid5840	NC_009376	chromosome									
	145591842	metallophosphoesterase	No	1	YES	1480006	1480701	+		Pyrobaculum_arsenaticum_DSM_13514_uid5840	NC_009376	chromosome		arCOG01156	1 (-)	R		Metallophosphoesterase superfamily enzyme	5E-13	COG1408	Predicted phosphohydrolases
===									6	Pyrobaculum_arsenaticum_DSM_13514_uid5840	NC_009376	chromosome									
	145591885	pseudouridylate synthase	No	0		1519476	1520717	-		Pyrobaculum_arsenaticum_DSM_13514_uid5840	NC_009376	chromosome		arCOG04252	4 (-)	J		tRNA pseudouridine synthase D	1E-48	COG0585	Uncharacterized conserved protein
	145591886	hypothetical protein Pars_1685	No	0		1521100	1521288	-		Pyrobaculum_arsenaticum_DSM_13514_uid5840	NC_009376	chromosome		arCOG09801	3	S		Uncharacterized conserved protein			
	145591887	hypothetical protein Pars_1686	No	0		1521296	1521778	-		Pyrobaculum_arsenaticum_DSM_13514_uid5840	NC_009376	chromosome		arCOG09800	3	S		Uncharacterized conserved protein			
	145591888	hypothetical protein Pars_1687	No	4		1522073	1522453	-		Pyrobaculum_arsenaticum_DSM_13514_uid5840	NC_009376	chromosome	clust_279								
	145591889	hypothetical protein Pars_1688	No	1		1523009	1523914	+		Pyrobaculum_arsenaticum_DSM_13514_uid5840	NC_009376	chromosome	clust_108								
	145591890	xylose isomerase domain-containing protein	No	0		1524665	1525507	+		Pyrobaculum_arsenaticum_DSM_13514_uid5840	NC_009376	chromosome		arCOG01894	3 (-)	L	Nfo	Endonuclease IV	9E-33	COG0648	Endonuclease IV
===									5	Pyrobaculum_calidifontis_JCM_11548_uid58787	NC_009073	chromosome									
	126458721	hypothetical protein Pcal_0094	No	1		94691	95044	+		Pyrobaculum_calidifontis_JCM_11548_uid58787	NC_009073	chromosome									
	126458722	hypothetical protein Pcal_0095	No	4		95095	95622	+		Pyrobaculum_calidifontis_JCM_11548_uid58787	NC_009073	chromosome									
	126458723	hypothetical protein Pcal_0096	Yes	0		95652	96278	+		Pyrobaculum_calidifontis_JCM_11548_uid58787	NC_009073	chromosome									
	126458724	hypothetical protein Pcal_0097	No	1		96356	96724	-		Pyrobaculum_calidifontis_JCM_11548_uid58787	NC_009073	chromosome		arCOG03739	2 (-)	S		Uncharacterized conserved protein			
	126458725	hypothetical protein Pcal_0098	Yes	6		96892	98007	+		Pyrobaculum_calidifontis_JCM_11548_uid58787	NC_009073	chromosome									
===									7	Pyrobaculum_calidifontis_JCM_11548_uid58787	NC_009073	chromosome									
	126459365	hypothetical protein Pcal_0751	No	0	YES	709392	710075	+		Pyrobaculum_calidifontis_JCM_11548_uid58787	NC_009073	chromosome		arCOG05605	1 (-)	S		Uncharacterized conserved protein			
	126459366	hypothetical protein Pcal_0752	No	0	YES	710086	710835	+		Pyrobaculum_calidifontis_JCM_11548_uid58787	NC_009073	chromosome									
	126459367	hypothetical protein Pcal_0753	Yes	0	YES	710840	711154	+		Pyrobaculum_calidifontis_JCM_11548_uid58787	NC_009073	chromosome									
	126459368	hypothetical protein Pcal_0754	No	0	YES	711178	711762	+		Pyrobaculum_calidifontis_JCM_11548_uid58787	NC_009073	chromosome									
	126459369	hypothetical protein Pcal_0755	No	0	YES	711759	712160	+		Pyrobaculum_calidifontis_JCM_11548_uid58787	NC_009073	chromosome									
	126459370	hypothetical protein Pcal_0756	No	2	YES	712157	714094	+		Pyrobaculum_calidifontis_JCM_11548_uid58787	NC_009073	chromosome		arCOG04034	1 (-)	L		Type IV secretory pathway, VirB4 component	0.000004	pfam01935	Domain of unknown function DUF87. The fu
	126459371	hypothetical protein Pcal_0757	No	0	YES	714121	714906	+		Pyrobaculum_calidifontis_JCM_11548_uid58787	NC_009073	chromosome									
===									6	Pyrobaculum_calidifontis_JCM_11548_uid58787	NC_009073	chromosome									
	126459813	hypothetical protein Pcal_1200	No	5		1113511	1114146	-		Pyrobaculum_calidifontis_JCM_11548_uid58787	NC_009073	chromosome							0.000004	pfam06195	Protein of unknown function (DUF996). Fa
	126459814	hypothetical protein Pcal_1201	No	0		1114486	1114707	+		Pyrobaculum_calidifontis_JCM_11548_uid58787	NC_009073	chromosome		arCOG04018	2	S		Uncharacterized conserved protein			
	126459815	hypothetical protein Pcal_1202	No	0		1114751	1115413	+		Pyrobaculum_calidifontis_JCM_11548_uid58787	NC_009073	chromosome		arCOG06996	2	S		Uncharacterized conserved protein			
	126459816	hypothetical protein Pcal_1203	No	0		1115410	1116603	+		Pyrobaculum_calidifontis_JCM_11548_uid58787	NC_009073	chromosome		arCOG06995	2 (-)	R		Predicted ATPase			
	126459817	hypothetical protein Pcal_1204	No	0		1116657	1117487	+		Pyrobaculum_calidifontis_JCM_11548_uid58787	NC_009073	chromosome		arCOG06994	2	S		Uncharacterized conserved protein			
	126459818	hypothetical protein Pcal_1205	No	6		1117563	1118120	+		Pyrobaculum_calidifontis_JCM_11548_uid58787	NC_009073	chromosome									
===									11	Pyrobaculum_islandicum_DSM_4184_uid58635	NC_008701	chromosome									
	119872769	GCN5-related N-acetyltransferase	No	1		1155438	1156031	-		Pyrobaculum_islandicum_DSM_4184_uid58635	NC_008701	chromosome		arCOG00845	1 (-)	K	WecD	Acetyltransferase (GNAT) family	0.0000001	pfam00583	Acetyltransferase (GNAT) family. This fa
	119872770	hypothetical protein Pisl_1270	No	0		1156274	1156906	-		Pyrobaculum_islandicum_DSM_4184_uid58635	NC_008701	chromosome									
	119872771	hypothetical protein Pisl_1271	No	0		1156896	1157381	-		Pyrobaculum_islandicum_DSM_4184_uid58635	NC_008701	chromosome									
	119872772	hypothetical protein Pisl_1272	No	4		1157415	1157786	-		Pyrobaculum_islandicum_DSM_4184_uid58635	NC_008701	chromosome		arCOG10902	3	S		Uncharacterized conserved protein			
	119872773	hypothetical protein Pisl_1273	No	0		1158123	1158455	+		Pyrobaculum_islandicum_DSM_4184_uid58635	NC_008701	chromosome									
	119872774	nuclease	Yes	2		1158643	1159578	+		Pyrobaculum_islandicum_DSM_4184_uid58635	NC_008701	chromosome		arCOG03193	1 (-)	L		Micrococcal nuclease (thermonuclease)	0.000000008	pfam01456	Mucin-like glycoprotein. This family of ; Micrococcal nuclease (thermonuclease) ho
	119872775	hypothetical protein Pisl_1275	No	0		1159612	1159932	+		Pyrobaculum_islandicum_DSM_4184_uid58635	NC_008701	chromosome							0.00005	pfam00183	Hsp90 protein.
	119872776	hypothetical protein Pisl_1276	No	2		1159908	1160156	+		Pyrobaculum_islandicum_DSM_4184_uid58635	NC_008701	chromosome									
	119872777	hypothetical protein Pisl_1277	No	0		1160252	1160515	+		Pyrobaculum_islandicum_DSM_4184_uid58635	NC_008701	chromosome									
	119872778	hypothetical protein Pisl_1278	No	3		1160559	1160903	+		Pyrobaculum_islandicum_DSM_4184_uid58635	NC_008701	chromosome		arCOG10902	3	S		Uncharacterized conserved protein			
	119872779	transposase, IS605 OrfB	No	0		1161123	1162394	+		Pyrobaculum_islandicum_DSM_4184_uid58635	NC_008701	chromosome		arCOG00687	3 (-)	L		Transposase	0.00002	pfam07282	Putative transposase DNA-binding domain.
===									5	Pyrobaculum_islandicum_DSM_4184_uid58635	NC_008701	chromosome									
	119872907	hypothetical protein Pisl_1413	No	1		1282498	1283946	+		Pyrobaculum_islandicum_DSM_4184_uid58635	NC_008701	chromosome		arCOG07745	1	S		Uncharacterized conserved protein			
	119872908	transposase, IS605 OrfB	No	0		1283927	1285177	-		Pyrobaculum_islandicum_DSM_4184_uid58635	NC_008701	chromosome		arCOG00687	3 (-)	L		Transposase	0.0000006	COG0675	Transposase and inactivated derivatives
	119872909	hypothetical protein Pisl_1415	No	0		1285503	1285826	+		Pyrobaculum_islandicum_DSM_4184_uid58635	NC_008701	chromosome									
	119872910	hypothetical protein Pisl_1416	No	4		1285889	1286281	+		Pyrobaculum_islandicum_DSM_4184_uid58635	NC_008701	chromosome		arCOG10902	3	S		Uncharacterized conserved protein			
	119872911	hypothetical protein Pisl_1417	No	0		1286350	1286811	+		Pyrobaculum_islandicum_DSM_4184_uid58635	NC_008701	chromosome									
===									5	Pyrobaculum_islandicum_DSM_4184_uid58635	NC_008701	chromosome									
	119872976	hypothetical protein Pisl_1484	No	0	YES	1351495	1352313	+		Pyrobaculum_islandicum_DSM_4184_uid58635	NC_008701	chromosome									
	119872977	hypothetical protein Pisl_1485	No	0	YES	1352560	1353138	-		Pyrobaculum_islandicum_DSM_4184_uid58635	NC_008701	chromosome		arCOG05483	2	S		Uncharacterized conserved protein			
	119872978	hypothetical protein Pisl_1486	No	0	YES	1353425	1353793	-		Pyrobaculum_islandicum_DSM_4184_uid58635	NC_008701	chromosome									
	119872979	hypothetical protein Pisl_1487	No	0	YES	1353944	1354975	+		Pyrobaculum_islandicum_DSM_4184_uid58635	NC_008701	chromosome		arCOG03233	3 (-)	R		Predicted ATPase	8E-10	COG4938	Uncharacterized conserved protein
	119872980	hypothetical protein Pisl_1488	No	0	YES	1354975	1355538	+		Pyrobaculum_islandicum_DSM_4184_uid58635	NC_008701	chromosome		arCOG07454	1	S		Uncharacterized conserved protein			
===									6	Pyrobaculum_islandicum_DSM_4184_uid58635	NC_008701	chromosome									
	119873037	hypothetical protein Pisl_1546	No	0	YES	1411984	1412400	+		Pyrobaculum_islandicum_DSM_4184_uid58635	NC_008701	chromosome									
	119873038	hypothetical protein Pisl_1547	No	0	YES	1412798	1413700	+		Pyrobaculum_islandicum_DSM_4184_uid58635	NC_008701	chromosome		arCOG09845	2	S		Uncharacterized conserved protein			
	119873039	ATPase domain-containing protein	No	0		1415462	1417864	+		Pyrobaculum_islandicum_DSM_4184_uid58635	NC_008701	chromosome	arCOG07675,a	arCOG07675	1 (-)	O	HtpG	Molecular chaperone, HSP90 family	0.00000003	COG0326	Molecular chaperone, HSP90 family
	119873040	hypothetical protein Pisl_1549	No	0		1417857	1418585	+		Pyrobaculum_islandicum_DSM_4184_uid58635	NC_008701	chromosome									
	119873041	hypothetical protein Pisl_1550	No	0		1418820	1419059	-		Pyrobaculum_islandicum_DSM_4184_uid58635	NC_008701	chromosome									
	119873042	hypothetical protein Pisl_1551	No	2		1419133	1419867	-		Pyrobaculum_islandicum_DSM_4184_uid58635	NC_008701	chromosome									
===									5	Pyrobaculum_neutrophilum_V24Sta_uid58421	NC_010525	chromosome									
	171185039	hypothetical protein Tneu_0570	No	0	YES	522351	522758	+		Pyrobaculum_neutrophilum_V24Sta_uid58421	NC_010525	chromosome									
	171185040	hypothetical protein Tneu_0571	No	0	YES	522752	523291	+		Pyrobaculum_neutrophilum_V24Sta_uid58421	NC_010525	chromosome		arCOG09817	1	S		Uncharacterized conserved protein			
	171185041	hypothetical protein Tneu_0572	No	0	YES	523301	525082	+		Pyrobaculum_neutrophilum_V24Sta_uid58421	NC_010525	chromosome		arCOG03433	1 (-)	V		CRISPR system related protein, COG1517 family			
	171185042	hypothetical protein Tneu_0573	Yes	0	YES	525567	525980	+		Pyrobaculum_neutrophilum_V24Sta_uid58421	NC_010525	chromosome									
	171185043	hypothetical protein Tneu_0574	No	0	YES	526021	526344	+		Pyrobaculum_neutrophilum_V24Sta_uid58421	NC_010525	chromosome		arCOG09845	2	S		Uncharacterized conserved protein			
===									7	Pyrobaculum_neutrophilum_V24Sta_uid58421	NC_010525	chromosome									
	171185612	hypothetical protein Tneu_1156	No	0	YES	1049078	1049830	+		Pyrobaculum_neutrophilum_V24Sta_uid58421	NC_010525	chromosome		arCOG07011	3 (-)	S		Uncharacterized phage-associated protein	0.0001	pfam13274	Protein of unknown function (DUF4065). T
	171185613	hypothetical protein Tneu_1157	No	0	YES	1049836	1050669	+		Pyrobaculum_neutrophilum_V24Sta_uid58421	NC_010525	chromosome									
	171185614	hypothetical protein Tneu_1158	Yes	1	YES	1050717	1051001	+		Pyrobaculum_neutrophilum_V24Sta_uid58421	NC_010525	chromosome									
	171185615	WD-40 repeat-containing protein	No	0		1051023	1052024	+		Pyrobaculum_neutrophilum_V24Sta_uid58421	NC_010525	chromosome							0.000005	COG2319	FOG: WD40 repeat
	171185616	hypothetical protein Tneu_1160	Yes	5		1052554	1054554	+		Pyrobaculum_neutrophilum_V24Sta_uid58421	NC_010525	chromosome									
	171185617	hypothetical protein Tneu_1161	No	3		1054536	1055213	+		Pyrobaculum_neutrophilum_V24Sta_uid58421	NC_010525	chromosome									
	171185618	paREP10	Yes	0		1055459	1055647	+		Pyrobaculum_neutrophilum_V24Sta_uid58421	NC_010525	chromosome		arCOG00746	1 (-)	K		Predicted transcriptional regulator, paREP10 subfamily of ArsR family			
===									7	Pyrobaculum_neutrophilum_V24Sta_uid58421	NC_010525	chromosome									
	171185936	hypothetical protein Tneu_1484	No	0		1320763	1321146	-		Pyrobaculum_neutrophilum_V24Sta_uid58421	NC_010525	chromosome		arCOG05592	1 (-)	S		Uncharacterized conserved protein			
	171185937	hypothetical protein Tneu_1486	No	1		1322984	1323454	+		Pyrobaculum_neutrophilum_V24Sta_uid58421	NC_010525	chromosome									
	171185938	hypothetical protein Tneu_1487	No	0		1323473	1323673	+		Pyrobaculum_neutrophilum_V24Sta_uid58421	NC_010525	chromosome									
	171185939	hypothetical protein Tneu_1488	No	0		1324266	1325513	+		Pyrobaculum_neutrophilum_V24Sta_uid58421	NC_010525	chromosome									
	171185940	hypothetical protein Tneu_1489	No	0		1325765	1326682	+		Pyrobaculum_neutrophilum_V24Sta_uid58421	NC_010525	chromosome									
	171185941	hypothetical protein Tneu_1490	No	0		1326683	1327018	+		Pyrobaculum_neutrophilum_V24Sta_uid58421	NC_010525	chromosome									
	171185942	exosome complex exonuclease Rrp41	No	0		1327457	1327609	+		Pyrobaculum_neutrophilum_V24Sta_uid58421	NC_010525	chromosome		arCOG01575	1 (-)	J	Rph	Ribonuclease PH	0.00004	COG2123	RNase PH-related exoribonuclease
===									9	Pyrobaculum_oguniense_TE7_uid84411	NC_016885	chromosome									
	379002963	hypothetical protein Pogu_0001	No	0		1	213	-		Pyrobaculum_oguniense_TE7_uid84411	NC_016885	chromosome		arCOG07479	1 (-)	S		Uncharacterized conserved protein			
	379002964	hypothetical protein Pogu_0002	No	0		210	608	-		Pyrobaculum_oguniense_TE7_uid84411	NC_016885	chromosome	clust_269								
	379002965	hypothetical protein Pogu_0003	No	0		605	1234	-		Pyrobaculum_oguniense_TE7_uid84411	NC_016885	chromosome									
	379002966	hypothetical protein Pogu_0004	No	0		1239	1991	-		Pyrobaculum_oguniense_TE7_uid84411	NC_016885	chromosome	clust_113								
	379002967	hypothetical protein Pogu_0005	No	0		3038	3955	+		Pyrobaculum_oguniense_TE7_uid84411	NC_016885	chromosome									
	379002968	hypothetical protein Pogu_0006	No	0		3978	5345	-		Pyrobaculum_oguniense_TE7_uid84411	NC_016885	chromosome									
	379002969	hypothetical protein Pogu_0007	No	0		5399	5779	-		Pyrobaculum_oguniense_TE7_uid84411	NC_016885	chromosome									
	379002970	hypothetical protein Pogu_0008	No	0		5875	6258	-		Pyrobaculum_oguniense_TE7_uid84411	NC_016885	chromosome									
	379002971	hypothetical protein Pogu_0009	No	0		6533	6715	+		Pyrobaculum_oguniense_TE7_uid84411	NC_016885	chromosome		arCOG06985	1 (-)	S		Zn finger protein			
===									5	Pyrobaculum_oguniense_TE7_uid84411	NC_016885	chromosome									
	379002977	hypothetical protein Pogu_0015	No	0		15700	15837	+		Pyrobaculum_oguniense_TE7_uid84411	NC_016885	chromosome									
	379002978	hypothetical protein Pogu_0016	No	0		15895	16017	+		Pyrobaculum_oguniense_TE7_uid84411	NC_016885	chromosome									
	379002979	distinct helicase family with a unique C-terminal	No	0		16145	19255	+		Pyrobaculum_oguniense_TE7_uid84411	NC_016885	chromosome		arCOG00555	5 (-)	R		Distinct helicase family with a unique C-terminal d	5E-22	COG1205	Distinct helicase family with a unique C
	379002980	hypothetical protein Pogu_0018	No	0		19635	19853	-		Pyrobaculum_oguniense_TE7_uid84411	NC_016885	chromosome									
	379002981	hypothetical protein Pogu_0019	No	0		20094	20414	+		Pyrobaculum_oguniense_TE7_uid84411	NC_016885	chromosome									
===									5	Pyrobaculum_oguniense_TE7_uid84411	NC_016885	chromosome									
	379003162	hypothetical protein Pogu_0200	No	0		169225	169401	+		Pyrobaculum_oguniense_TE7_uid84411	NC_016885	chromosome									
	379003163	hypothetical protein Pogu_0201	No	0		169423	169809	+		Pyrobaculum_oguniense_TE7_uid84411	NC_016885	chromosome									
	379003164	hypothetical protein Pogu_0202	No	0		169848	170606	+		Pyrobaculum_oguniense_TE7_uid84411	NC_016885	chromosome		arCOG03671	3 (-)	E		Thermopsin-like protease			
	379003165	hypothetical protein Pogu_0203	No	0		170614	170844	+		Pyrobaculum_oguniense_TE7_uid84411	NC_016885	chromosome									
	379003166	hypothetical protein Pogu_0204	No	0		170814	171035	+		Pyrobaculum_oguniense_TE7_uid84411	NC_016885	chromosome									
===									7	Pyrobaculum_oguniense_TE7_uid84411	NC_016885	chromosome									
	379003410	Endonuclease IV	No	0		374093	374917	-		Pyrobaculum_oguniense_TE7_uid84411	NC_016885	chromosome		arCOG01894	3 (-)	L	Nfo	Endonuclease IV	8E-33	COG0648	Endonuclease IV
	379003411	hypothetical protein Pogu_0449	No	1		375686	376591	-		Pyrobaculum_oguniense_TE7_uid84411	NC_016885	chromosome	clust_108								
	379003412	hypothetical protein Pogu_0450	No	0		376859	377152	+		Pyrobaculum_oguniense_TE7_uid84411	NC_016885	chromosome									
	379003413	hypothetical protein Pogu_0451	No	4		377149	377529	+		Pyrobaculum_oguniense_TE7_uid84411	NC_016885	chromosome	clust_279								
	379003414	hypothetical protein Pogu_0452	Yes	1		377743	378309	+		Pyrobaculum_oguniense_TE7_uid84411	NC_016885	chromosome		arCOG09800	3	S		Uncharacterized conserved protein			
	379003415	hypothetical protein Pogu_0453	No	0		378317	378505	+		Pyrobaculum_oguniense_TE7_uid84411	NC_016885	chromosome		arCOG09801	3	S		Uncharacterized conserved protein			
	379003416	tRNA pseudouridine synthase, TruD family	No	0		378890	380131	+		Pyrobaculum_oguniense_TE7_uid84411	NC_016885	chromosome		arCOG04252	4 (-)	J		tRNA pseudouridine synthase D	9E-48	COG0585	Uncharacterized conserved protein
===									6	Pyrobaculum_oguniense_TE7_uid84411	NC_016885	chromosome									
	379003473	hypothetical protein Pogu_0511	No	0		428808	430190	+		Pyrobaculum_oguniense_TE7_uid84411	NC_016885	chromosome	arCOG03194,a	arCOG03194	1 (-)	S		Uncharacterized conserved protein			
	379003474	hypothetical protein Pogu_0512	No	0		430187	430921	+		Pyrobaculum_oguniense_TE7_uid84411	NC_016885	chromosome									
	379003475	hypothetical protein Pogu_0513	No	0		430897	431883	+		Pyrobaculum_oguniense_TE7_uid84411	NC_016885	chromosome									

	379003476	hypothetical protein Pogu_0514	No	1			432089	432325	+		Pyrobaculum_oguniense_TE7_uid84411	NC_016885	chromosome									
	379003477	hypothetical protein Pogu_0515	No	0			432426	432656	+		Pyrobaculum_oguniense_TE7_uid84411	NC_016885	chromosome									
	379003478	hypothetical protein Pogu_0516	No	0			432653	433072	+		Pyrobaculum_oguniense_TE7_uid84411	NC_016885	chromosome		arCOG00679	4 (-)	L		Transposase			
===										13	Pyrobaculum_oguniense_TE7_uid84411	NC_016885	chromosome									
	379003549	hypothetical protein Pogu_0587	No	0			487460	487756	-		Pyrobaculum_oguniense_TE7_uid84411	NC_016885	chromosome	clust_354								
	379003550	hypothetical protein Pogu_0588	No	0			487753	488430	-		Pyrobaculum_oguniense_TE7_uid84411	NC_016885	chromosome									
	379003551	hypothetical protein Pogu_0589	No	0			488497	489123	-		Pyrobaculum_oguniense_TE7_uid84411	NC_016885	chromosome		arCOG00488	4 (-)	L	DnaN	DNA polymerase sliding clamp subunit (PCNA homolog)			
	379003552	hypothetical protein Pogu_0590	No	0			489116	489439	-		Pyrobaculum_oguniense_TE7_uid84411	NC_016885	chromosome									
	379003553	hypothetical protein Pogu_0591	No	0			489440	489853	+		Pyrobaculum_oguniense_TE7_uid84411	NC_016885	chromosome									
	379003554	hypothetical protein Pogu_0592	No	0			490331	491365	-		Pyrobaculum_oguniense_TE7_uid84411	NC_016885	chromosome									
	379003555	hypothetical protein Pogu_0593	No	0			491621	492490	+		Pyrobaculum_oguniense_TE7_uid84411	NC_016885	chromosome	clust_113								
	379003556	hypothetical protein Pogu_0594	No	0			492480	493235	+		Pyrobaculum_oguniense_TE7_uid84411	NC_016885	chromosome									
	379003557	hypothetical protein Pogu_0595	No	1			493226	493579	+		Pyrobaculum_oguniense_TE7_uid84411	NC_016885	chromosome	clust_269								
	379003558	hypothetical protein Pogu_0596	No	0			493601	493954	+		Pyrobaculum_oguniense_TE7_uid84411	NC_016885	chromosome									
	379003559	hypothetical protein Pogu_0597	No	0			493960	494319	+		Pyrobaculum_oguniense_TE7_uid84411	NC_016885	chromosome									
	379003560	hypothetical protein Pogu_0598	No	0			494307	494669	+		Pyrobaculum_oguniense_TE7_uid84411	NC_016885	chromosome									
	379003561	ATPase	No	0			494699	495412	+		Pyrobaculum_oguniense_TE7_uid84411	NC_016885	chromosome		arCOG05608	1 (-)	N	flhG	Antiactivator of flagellar biosynthesis FleN, an AT	0.00003	COG0455	Antiactivator of flagellar biosynthesis
===										7	Pyrobaculum_oguniense_TE7_uid84411	NC_016885	chromosome									
	379003589	recombinase A	No	0			515654	516595	+		Pyrobaculum_oguniense_TE7_uid84411	NC_016885	chromosome		arCOG00415	7 (-)	L	RecA	RecA/RadA recombinase	6E-15	pfam08423	Rad51. Rad51 is a DNA repair and recombi
	379003590	hypothetical protein Pogu_0628	No	0			516582	516866	+		Pyrobaculum_oguniense_TE7_uid84411	NC_016885	chromosome									
	379003591	hypothetical protein Pogu_0629	No	0			516859	517203	+		Pyrobaculum_oguniense_TE7_uid84411	NC_016885	chromosome									
	379003592	hypothetical protein Pogu_0630	No	0			517206	517520	+		Pyrobaculum_oguniense_TE7_uid84411	NC_016885	chromosome									
	379003593	hypothetical protein Pogu_0631	Yes	3			517820	518290	+		Pyrobaculum_oguniense_TE7_uid84411	NC_016885	chromosome		arCOG10500	4	S		Uncharacterized conserved protein			
	379003594	hypothetical protein Pogu_0632	No	6			518290	519201	+		Pyrobaculum_oguniense_TE7_uid84411	NC_016885	chromosome									
	379003595	PaRep2b protein	No	6			519201	532319	+		Pyrobaculum_oguniense_TE7_uid84411	NC_016885	chromosome	arCOG02170,a	arCOG02170	1 (-)	S		Uncharacterized conserved protein	2E-62	pfam07775	PaRep2b protein. This is a family of pro ; Chromosome segregation ATPases
===										7	Pyrobaculum_oguniense_TE7_uid84411	NC_016885	chromosome									
	379003899	hypothetical protein Pogu_0937	No	0			797388	797669	+		Pyrobaculum_oguniense_TE7_uid84411	NC_016885	chromosome		arCOG09804	3	S		Uncharacterized conserved protein			
	379003900	hypothetical protein Pogu_0938	No	0			797834	798142	+		Pyrobaculum_oguniense_TE7_uid84411	NC_016885	chromosome		arCOG01062	2 (-)	K		Predicted transcriptional regulator	0.00001	COG3432	Predicted transcriptional regulator
	379003901	hypothetical protein Pogu_0939	No	0			798238	798510	+		Pyrobaculum_oguniense_TE7_uid84411	NC_016885	chromosome									
	379003902	hypothetical protein Pogu_0940	Yes	2			798899	800002	-		Pyrobaculum_oguniense_TE7_uid84411	NC_016885	chromosome		arCOG07061	1	S		Uncharacterized conserved protein			
	379003903	hypothetical protein Pogu_0941	No	0			799992	800609	-		Pyrobaculum_oguniense_TE7_uid84411	NC_016885	chromosome									
	379003904	hypothetical protein Pogu_0942	Yes	1			800609	800785	-		Pyrobaculum_oguniense_TE7_uid84411	NC_016885	chromosome									
	379003905	hypothetical protein Pogu_0943	No	0			801002	801214	-		Pyrobaculum_oguniense_TE7_uid84411	NC_016885	chromosome		arCOG03956	2 (-)	O	HypF	Hydrogenase maturation factor			
===										8	Pyrobaculum_oguniense_TE7_uid84411	NC_016885	chromosome									
	379004007	hypothetical protein Pogu_1045	Yes	0			886055	886972	+		Pyrobaculum_oguniense_TE7_uid84411	NC_016885	chromosome		arCOG02748	1	S		Uncharacterized conserved protein, DUF58 famil	9E-10	COG1721	Uncharacterized conserved protein (some
	379004008	hypothetical protein Pogu_1046	No	5			886969	887466	+		Pyrobaculum_oguniense_TE7_uid84411	NC_016885	chromosome		arCOG07041	1	S		Uncharacterized conserved protein			
	379004009	putative ATPase (AAA+ superfamily)	No	0			887494	888834	+		Pyrobaculum_oguniense_TE7_uid84411	NC_016885	chromosome		arCOG03166	9 (-)	R		AAA+ superfamily ATPase fused to HTH and Rec	9E-33	COG1672	Predicted ATPase (AAA+ superfamily)
	379004010	hypothetical protein Pogu_1048	No	0			888975	21	+		Pyrobaculum_oguniense_TE7_uid84411	NC_016885	chromosome									
	379004011	hypothetical protein Pogu_1049	No	0			18	889750	+		Pyrobaculum_oguniense_TE7_uid84411	NC_016885	chromosome									
	379004012	hypothetical protein Pogu_1050, partial	No	0			890128	890373	+		Pyrobaculum_oguniense_TE7_uid84411	NC_016885	chromosome		arCOG01500	1 (-)	C	HybA	Fe-S-cluster-containing dehydrogenase compone	1E-17	COG0437	Fe-S-cluster-containing hydrogenase comp
	379004013	hypothetical protein Pogu_1051	No	0			890805	890960	+		Pyrobaculum_oguniense_TE7_uid84411	NC_016885	chromosome									
	379004014	hypothetical protein Pogu_1052	No	4			890985	891413	+		Pyrobaculum_oguniense_TE7_uid84411	NC_016885	chromosome									
===										6	Pyrobaculum_oguniense_TE7_uid84411	NC_016885	chromosome									
	379004055	hypothetical protein Pogu_1093	No	3			935336	935563	-		Pyrobaculum_oguniense_TE7_uid84411	NC_016885	chromosome									
	379004056	hypothetical protein Pogu_1094	No	0			935901	936110	+		Pyrobaculum_oguniense_TE7_uid84411	NC_016885	chromosome									
	379004057	hypothetical protein Pogu_1095	No	2			936088	936486	+		Pyrobaculum_oguniense_TE7_uid84411	NC_016885	chromosome	clust_245								
	379004058	hypothetical protein Pogu_1096	No	0			936561	936818	+		Pyrobaculum_oguniense_TE7_uid84411	NC_016885	chromosome		arCOG07131	7 (-)	V	CcmA	ABC-type multidrug transport system, ATPase component			
	379004059	hypothetical protein Pogu_1097	No	0			936815	936964	+		Pyrobaculum_oguniense_TE7_uid84411	NC_016885	chromosome									
	379004060	hypothetical protein Pogu_1098	No	0			937269	937505	-		Pyrobaculum_oguniense_TE7_uid84411	NC_016885	chromosome									
===										6	Pyrobaculum_oguniense_TE7_uid84411	NC_016885	chromosome									
	379004272	universal metal-binding-domain/4Fe-4S-binding	No	0			1127803	1128546	+		Pyrobaculum_oguniense_TE7_uid84411	NC_016885	chromosome		arCOG00035	3 (-)	R		Predicted ATPase of PP-loop superfamily	1E-60	COG2102	Predicted ATPases of PP-loop superfamily
	379004273	hypothetical protein Pogu_1311	No	1			1128546	1128806	+		Pyrobaculum_oguniense_TE7_uid84411	NC_016885	chromosome									
	379004274	hypothetical protein Pogu_1312	No	1			1128803	1128913	+		Pyrobaculum_oguniense_TE7_uid84411	NC_016885	chromosome									
	379004275	hypothetical protein Pogu_1313	No	2			1128910	1129155	+		Pyrobaculum_oguniense_TE7_uid84411	NC_016885	chromosome		arCOG09812	2	S		Uncharacterized conserved protein			
	379004276	hypothetical protein Pogu_1314	No	1			1129128	1129334	-		Pyrobaculum_oguniense_TE7_uid84411	NC_016885	chromosome									
	379004277	methionine aminopeptidase	No	0			1129598	1130473	+		Pyrobaculum_oguniense_TE7_uid84411	NC_016885	chromosome		arCOG01001	1 (-)	J	Map	Methionine aminopeptidase	1E-34	COG0024	Methionine aminopeptidase
===										6	Pyrobaculum_oguniense_TE7_uid84411	NC_016885	chromosome									
	379004519	hypothetical protein Pogu_1557	Yes	1			1353628	1353762	+		Pyrobaculum_oguniense_TE7_uid84411	NC_016885	chromosome									
	379004520	hypothetical protein Pogu_1558	No	0			1353797	1354123	+		Pyrobaculum_oguniense_TE7_uid84411	NC_016885	chromosome									
	379004521	hypothetical protein Pogu_1559	Yes	1			1354391	1355251	+		Pyrobaculum_oguniense_TE7_uid84411	NC_016885	chromosome		arCOG09781	3	S		Uncharacterized conserved protein			
	379004522	Thioredoxin reductase	No	1			1355258	1356223	-		Pyrobaculum_oguniense_TE7_uid84411	NC_016885	chromosome		arCOG01296	3 (-)	O	TrxB	Thioredoxin reductase	4E-70	COG0492	Thioredoxin reductase
	379004523	hypothetical protein Pogu_1561	Yes	3			1356297	1357208	-		Pyrobaculum_oguniense_TE7_uid84411	NC_016885	chromosome		arCOG09782	4	S		Uncharacterized conserved protein			
	379004524	hypothetical protein Pogu_1562	No	0			1357232	1357366	-		Pyrobaculum_oguniense_TE7_uid84411	NC_016885	chromosome									
===										6	Pyrobaculum_oguniense_TE7_uid84411	NC_016885	chromosome									
	379004530	50S ribosomal protein L22(archaeal)/L17(eukar	No	0			1361159	1361716	+		Pyrobaculum_oguniense_TE7_uid84411	NC_016885	chromosome		arCOG04098	1 (-)	J	RplV	Ribosomal protein L22	3E-12	COG0091	Ribosomal protein L22
	379004531	hypothetical protein Pogu_1569	Yes	1			1361977	1362870	+		Pyrobaculum_oguniense_TE7_uid84411	NC_016885	chromosome									
	379004532	hypothetical protein Pogu_1570	No	0			1363166	1363327	-		Pyrobaculum_oguniense_TE7_uid84411	NC_016885	chromosome									
	379004533	hypothetical protein Pogu_1571	Yes	1			1363913	1364524	+		Pyrobaculum_oguniense_TE7_uid84411	NC_016885	chromosome									
	379004534	hypothetical protein Pogu_1572	Yes	1			1364526	1365338	+		Pyrobaculum_oguniense_TE7_uid84411	NC_016885	chromosome	clust_121								
	379004535	30S ribosomal protein S3, eukaryotic/archaeal	No	0			1365752	1366414	+		Pyrobaculum_oguniense_TE7_uid84411	NC_016885	chromosome		arCOG04097	1 (-)	J	RpsC	Ribosomal protein S3	2E-28	COG0092	Ribosomal protein S3
===										5	Pyrobaculum_oguniense_TE7_uid84411	NC_016885	chromosome									
	379004599	hypothetical protein Pogu_1637	No	0			1424537	1424836	-		Pyrobaculum_oguniense_TE7_uid84411	NC_016885	chromosome									
	379004600	hypothetical protein Pogu_1638	Yes	2			1425249	1425653	+		Pyrobaculum_oguniense_TE7_uid84411	NC_016885	chromosome									
	379004601	hypothetical protein Pogu_1639	No	3			1425655	1426275	+		Pyrobaculum_oguniense_TE7_uid84411	NC_016885	chromosome		arCOG07527	5 (-)	S		Predicted metal-binding protein			
	379004602	hypothetical protein Pogu_1640	No	4			1426302	1426661	+		Pyrobaculum_oguniense_TE7_uid84411	NC_016885	chromosome									
	379004603	hypothetical protein Pogu_1641	No	1			1426684	1426932	+		Pyrobaculum_oguniense_TE7_uid84411	NC_016885	chromosome									
===										15	Pyrobaculum_oguniense_TE7_uid84411	NC_016885	chromosome									
	379004631	hypothetical protein Pogu_1669	No	4			1453694	1454212	-		Pyrobaculum_oguniense_TE7_uid84411	NC_016885	chromosome		arCOG09804	3	S		Uncharacterized conserved protein			
	379004632	hypothetical protein Pogu_1670	No	0			1454423	1454854	+		Pyrobaculum_oguniense_TE7_uid84411	NC_016885	chromosome		arCOG00194	14 (-)	V	CcmA	ABC-type multidrug transport system, ATPase co	0.000001	COG1131	ABC-type multidrug transport system, ATP
	379004633	hypothetical protein Pogu_1671	Yes	0			1454909	1455037	+		Pyrobaculum_oguniense_TE7_uid84411	NC_016885	chromosome									
	379004634	hypothetical protein Pogu_1672	No	4			1455019	1455735	+		Pyrobaculum_oguniense_TE7_uid84411	NC_016885	chromosome									
	379004635	hypothetical protein Pogu_1673	No	2			1455684	1455920	+		Pyrobaculum_oguniense_TE7_uid84411	NC_016885	chromosome									
	379004636	Serine protease inhibitor	No	0			1456114	1456821	-		Pyrobaculum_oguniense_TE7_uid84411	NC_016885	chromosome		arCOG04933	4 (-)	O		Serine protease inhibitor	2E-23	COG4826	Serine protease inhibitor
	379004637	hypothetical protein Pogu_1675	Yes	1			1456948	1457520	-		Pyrobaculum_oguniense_TE7_uid84411	NC_016885	chromosome		arCOG09758	4	S		Uncharacterized conserved protein			
	379004638	hypothetical protein Pogu_1676	No	1			1457635	1457760	+		Pyrobaculum_oguniense_TE7_uid84411	NC_016885	chromosome									
	379004639	hypothetical protein Pogu_1677	No	0			1458240	1458641	-		Pyrobaculum_oguniense_TE7_uid84411	NC_016885	chromosome									
	379004640	hypothetical protein Pogu_1678	No	6			1458628	1459209	-		Pyrobaculum_oguniense_TE7_uid84411	NC_016885	chromosome	clust_36								
	379004641	hypothetical protein Pogu_1679	Yes	1			1459206	1461371	-		Pyrobaculum_oguniense_TE7_uid84411	NC_016885	chromosome		arCOG07012	6	S		Uncharacterized conserved protein			
	379004642	hypothetical protein Pogu_1680	Yes	1			1461533	1461991	-		Pyrobaculum_oguniense_TE7_uid84411	NC_016885	chromosome		arCOG09758	4	S		Uncharacterized conserved protein			
	379004643	hypothetical protein Pogu_1681	No	0			1462074	1462244	-		Pyrobaculum_oguniense_TE7_uid84411	NC_016885	chromosome									
	379004644	hypothetical protein Pogu_1682	No	0			1462699	1463025	-		Pyrobaculum_oguniense_TE7_uid84411	NC_016885	chromosome		arCOG07049	2	S		Uncharacterized conserved protein			
	379004645	hypothetical protein Pogu_1683	No	4			1463096	1463491	+		Pyrobaculum_oguniense_TE7_uid84411	NC_016885	chromosome		arCOG05546	1 (-)	S		Uncharacterized conserved protein			
===										7	Pyrobaculum_oguniense_TE7_uid84411	NC_016885	chromosome									
	379004832	hypothetical protein Pogu_1870	No	2			1626612	1627046	-		Pyrobaculum_oguniense_TE7_uid84411	NC_016885	chromosome									
	379004833	multidrug ABC transporter ATPase	No	0			1627043	1627630	-		Pyrobaculum_oguniense_TE7_uid84411	NC_016885	chromosome		arCOG07131	7 (-)	V	CcmA	ABC-type multidrug transport system, ATPase co	9E-18	COG1131	ABC-type multidrug transport system, ATP
	379004834	hypothetical protein Pogu_1872	No	1			1627617	1627958	-		Pyrobaculum_oguniense_TE7_uid84411	NC_016885	chromosome									
	379004835	hypothetical protein Pogu_1873	No	8			1628026	1629045	-		Pyrobaculum_oguniense_TE7_uid84411	NC_016885	chromosome	clust_95								
	379004836	hypothetical protein Pogu_1874	No	3			1629045	1629506	-		Pyrobaculum_oguniense_TE7_uid84411	NC_016885	chromosome									
	379004837	small GTP-binding protein domain-containing p	No	7			1629648	1631360	-		Pyrobaculum_oguniense_TE7_uid84411	NC_016885	chromosome		arCOG00359	2 (-)	P	FeoB	Fe2+ transport system protein B	2E-51	COG0370	Fe2+ transport system protein B
	379004838	hypothetical protein Pogu_1876	No	0			1631646	1632134	+		Pyrobaculum_oguniense_TE7_uid84411	NC_016885	chromosome		arCOG07696	1	S		Uncharacterized conserved protein			
===										6	Pyrobaculum_oguniense_TE7_uid84411	NC_016885	chromosome									
	379004842	hypothetical protein Pogu_1880	No	0			1636398	1636835	-		Pyrobaculum_oguniense_TE7_uid84411	NC_016885	chromosome		arCOG01927	1 (-)	C	CoxG	Carbon monoxide dehydrogenase subunit G, Co	5E-19	pfam06240	Carbon monoxide dehydrogenase subunit G
	379004843	PaRep2b protein	No	0			1636988	1637122	-		Pyrobaculum_oguniense_TE7_uid84411	NC_016885	chromosome		arCOG09780	7	S		Uncharacterized conserved protei, PaRep2b family			
	379004844	PaRep2b protein	No	0			1637156	1637284	-		Pyrobaculum_oguniense_TE7_uid84411	NC_016885	chromosome		arCOG09780	7	S		Uncharacterized conserved protei, PaRep2b family			
	379004845	hypothetical protein Pogu_1883	No	0			1637637	1638278	-		Pyrobaculum_oguniense_TE7_uid84411	NC_016885	chromosome		arCOG09760	1	S		Uncharacterized conserved protein			
	379004846	hypothetical protein Pogu_1884	No	0			1638625	1638843	+		Pyrobaculum_oguniense_TE7_uid84411	NC_016885	chromosome									
	379004847	hypothetical protein Pogu_1885	No	0			1639326	1639730	-		Pyrobaculum_oguniense_TE7_uid84411	NC_016885	chromosome		arCOG02108	1 (-)	V		HEPN domain containing protein	0.000003	COG2445	Uncharacterized conserved protein
===										7	Pyrobaculum_oguniense_TE7_uid84411	NC_016885	chromosome									
	379004895	hypothetical protein Pogu_1933	No	0			1680065	1680475	+		Pyrobaculum_oguniense_TE7_uid84411	NC_016885	chromosome		arCOG05501	3 (-)	S		Uncharacterized conserved protein			
	379004896	hypothetical protein Pogu_1934	Yes	2			1680595	1681653	+		Pyrobaculum_oguniense_TE7_uid84411	NC_016885	chromosome		arCOG09779	3	S		Uncharacterized conserved protein			
	379004897	hypothetical protein Pogu_1935	No	6			1681655	1682413	+		Pyrobaculum_oguniense_TE7_uid84411	NC_016885	chromosome									
	379004898	hypothetical protein Pogu_1936	No	5			1682406	1682819	+		Pyrobaculum_oguniense_TE7_uid84411	NC_016885	chromosome	clust_253								
	379004899	hypothetical protein Pogu_1937	No	4			1682904	1683416	+		Pyrobaculum_oguniense_TE7_uid84411	NC_016885	chromosome	clust_208								
	379004900	hypothetical protein Pogu_1938	No	4			1683413	1684075	+		Pyrobaculum_oguniense_TE7_uid84411	NC_016885	chromosome	clust_139								
	379004901	PaREP6	No	0			1684941	1685162	-		Pyrobaculum_oguniense_TE7_uid84411	NC_016885	chromosome		arCOG03756	1 (-)	S		paREP6 family protein			
===										7	Pyrobaculum_oguniense_TE7_uid84411	NC_016885	chromosome									
	379004906	hypothetical protein Pogu_1944	No	0			1687687	1687866	-		Pyrobaculum_oguniense_TE7_uid84411	NC_016885	chromosome		arCOG04009	1	S		Uncharacterized conserved protein			
	379004907	hypothetical protein Pogu_1945	No	0			1687860	1688165	-		Pyrobaculum_oguniense_TE7_uid84411	NC_016885	chromosome		arCOG06988	1 (-)	S		Uncharacterized conserved protein			
	379004908	hypothetical protein Pogu_1946	No	2			1688264	1688425	-		Pyrobaculum_oguniense_TE7_uid84411	NC_016885	chromosome		arCOG09762	4	S		Uncharacterized conserved protein			
	379004909	hypothetical protein Pogu_1947	No	2			1688582	1689061	+		Pyrobaculum_oguniense_TE7_uid84411	NC_016885	chromosome		arCOG09765	4	S		Uncharacterized conserved protein			
	379004910	hypothetical protein Pogu_1948	No	2			1689058	1689456	+		Pyrobaculum_oguniense_TE7_uid84411	NC_016885	chromosome									
	379004911	sugar ABC transporter periplasmic protein	Yes	1			1690209	1691546	-		Pyrobaculum_oguniense_TE7_uid84411	NC_016885	chromosome		arCOG00151	1 (-)	G	UgpB	ABC-type sugar transport system, periplasmic co	5E-15	COG1653	ABC-type sugar transport system, peripla
	379004912	hypothetical protein Pogu_1950	No	1			1691730	1691858	+		Pyrobaculum_oguniense_TE7_uid84411	NC_016885	chromosome									
===										5	Pyrobaculum_oguniense_TE7_uid84411	NC_016885	chromosome									
	379004930	hypothetical protein Pogu_1968	No	0			1706080	1706676	-		Pyrobaculum_oguniense_TE7_uid84411	NC_016885	chromosome									
	379004931	hypothetical protein Pogu_1969	No	0			1706824	1706937	-		Pyrobaculum_oguniense_TE7_uid84411	NC_016885	chromosome									
	379004932	HTH domain protein	No	0			1706967	1707749	-		Pyrobaculum_oguniense_TE7_uid84411	NC_016885	chromosome		arCOG09843	1	S		Uncharacterized conserved protein			
	379004933	hypothetical protein Pogu_1971	No	5			1707862	1708422	+		Pyrobaculum_oguniense_TE7_uid84411	NC_016885	chromosome		arCOG03768	1 (-)	S		Uncharacterized conserved protein	0.000002	pfam06157	Protein of unknown function (DUF973). Th
	379004934	hypothetical protein Pogu_1972	No	3			1708536	1709123	+		Pyrobaculum_oguniense_TE7_uid84411	NC_016885	chromosome									
===										12	Pyrobaculum_oguniense_TE7_uid84411	NC_016885	chromosome									
	379005007	hypothetical protein Pogu_2045	No	1			1783379	1783489	+		Pyrobaculum_oguniense_TE7_uid84411	NC_016885	chromosome		arCOG04189	2 (-)	S		Membrane protein			
	379005008	hypothetical protein Pogu_2046	No	0			1783622	1783870	-		Pyrobaculum_oguniense_TE7_uid84411	NC_016885	chromosome									
	379005009	hypothetical protein Pogu_2047	Yes	0			1785078	1785398	-		Pyrobaculum_oguniense_TE7_uid84411	NC_016885	chromosome									
	379005010	hypothetical protein Pogu_2048	No	1			1785425	1785562	-		Pyrobaculum_oguniense_TE7_uid84411	NC_016885	chromosome									
	379005011	hypothetical protein Pogu_2049	No	0			1785858	1786022	-		Pyrobaculum_oguniense_TE7_uid84411	NC_016885	chromosome									
	379005012	hypothetical protein Pogu_2050	No	0			1786144	1786455	-		Pyrobaculum_oguniense_TE7_uid84411	NC_016885	chromosome		arCOG09758	4	S		Uncharacterized conserved protein			
	379005013	putative protease	Yes	1			1786586	1790449	-		Pyrobaculum_oguniense_TE7_uid84411	NC_016885	chromosome		arCOG00704	1 (-)	O	AprE	Surface layer-associated subtilisin-like serine pro	0.000004	COG4934	Predicted protease
	379005014	hypothetical protein Pogu_2052	No	0			1790791	1791417	+		Pyrobaculum_oguniense_TE7_uid84411	NC_016885	chromosome									
	379005015	hypothetical protein Pogu_2053	No	0			1791488	1791799	+		Pyrobaculum_oguniense_TE7_uid84411	NC_016885	chromosome									
	379005016	hypothetical protein Pogu_2054	No	6			1792270	1793364	-		Pyrobaculum_oguniense_TE7_uid84411	NC_016885	chromosome									
	379005017	hypothetical protein Pogu_2055	No	6			1793364	1794119	-		Pyrobaculum_oguniense_TE7_uid84411	NC_016885	chromosome									
	379005018	glycosyl transferase family 2	No	0			1794222	1794482	-		Pyrobaculum_oguniense_TE7_uid84411	NC_016885	chromosome		arCOG01389	1 (-)	M		Glycosyltransferase			
===										19	Pyrobaculum_oguniense_TE7_uid84411	NC_016885	chromosome									
	379005061	putative nucleic acid-binding protein, contains	No	0			1824682	1825020	-		Pyrobaculum_oguniense_TE7_uid84411	NC_016885	chromosome		arCOG00727	2 (-)	V		PIN domain containing protein	0.000005	COG4113	Predicted nucleic acid-binding protein,
	379005062	hypothetical protein Pogu_2100	No	0			1825253	1825549	+		Pyrobaculum_oguniense_TE7_uid84411	NC_016885	chromosome									
	379005063	hypothetical protein Pogu_2101	No	0			1825760	1826032	+		Pyrobaculum_oguniense_TE7_uid84411	NC_016885	chromosome									
	379005064	hypothetical protein Pogu_2102	No	1			1826170	1826547	-		Pyrobaculum_oguniense_TE7_uid84411	NC_016885	chromosome									
	379005065	hypothetical protein Pogu_2103	No	0			1826672	1826950	+		Pyrobaculum_oguniense_TE7_uid84411	NC_016885	chromosome									
	379005066	hypothetical protein Pogu_2104	No	0			1827095	1827352	+		Pyrobaculum_oguniense_TE7_uid84411	NC_016885	chromosome		arCOG00374	2 (-)	K		Predicted membrane-associated trancriptional regulator			
	379005067	hypothetical protein Pogu_2105	No	0			1827622	1827753	-		Pyrobaculum_oguniense_TE7_uid84411	NC_016885	chromosome									
	379005068	hypothetical protein Pogu_2106	No	0			1827825	1827944	-		Pyrobaculum_oguniense_TE7_uid84411	NC_016885	chromosome									
	379005069	hypothetical protein Pogu_2107	No	0			1828299	1828505	+		Pyrobaculum_oguniense_TE7_uid84411	NC_016885	chromosome									
	379005070	hypothetical protein Pogu_2108	No	2			1828845	1829084	-		Pyrobaculum_oguniense_TE7_uid84411	NC_016885	chromosome									
	379005071	hypothetical protein Pogu_2109	No	0			1829161	1829310	+		Pyrobaculum_oguniense_TE7_uid84411	NC_016885	chromosome									
	379005072	hypothetical protein Pogu_2110	No	1			1829643	1830083	-		Pyrobaculum_oguniense_TE7_uid84411	NC_016885	chromosome									
	379005073	hypothetical protein Pogu_2111	No	0			1830194	1830376	+		Pyrobaculum_oguniense_TE7_uid84411	NC_016885	chromosome									
	379005074	hypothetical protein Pogu_2112	No	0			1830463	1830888	+		Pyrobaculum_oguniense_TE7_uid84411	NC_016885	chromosome									
	379005075	hypothetical protein Pogu_2113	No	0			1831027	1831215	+		Pyrobaculum_oguniense_TE7_uid84411	NC_016885	chromosome									
	379005076	hypothetical protein Pogu_2114	No	0			1831603	1832169	+		Pyrobaculum_oguniense_TE7_uid84411	NC_016885	chromosome									
	379005077	hypothetical protein Pogu_2115	No	1			1832183	1832410	-		Pyrobaculum_oguniense_TE7_uid84411	NC_016885	chromosome									
	379005078	PaRep2b protein	No	0			1832598	1833815	+		Pyrobaculum_oguniense_TE7_uid84411	NC_016885	chromosome		arCOG09780	7	S		Uncharacterized conserved protei, PaRep2b fam	8E-60	pfam07775	PaRep2b protein. This is a family of pro
	379005079	DNA repair photolyase	No	0			1833812	1834648	-		Pyrobaculum_oguniense_TE7_uid84411	NC_016885	chromosome		arCOG01290	5 (-)	L	SplB	DNA repair photolyase	1E-25	COG1533	DNA repair photolyase
===										6	Pyrobaculum_oguniense_TE7_uid84411	NC_016885	chromosome									
	379005144	Putative transposase DNA-binding domain-con	No	0			1895949	1896680	+		Pyrobaculum_oguniense_TE7_uid84411	NC_016885	chromosome		arCOG05481	2 (-)	L		Transposase			
	379005145	hypothetical protein Pogu_2183	Yes	1			1896848	1899001	+		Pyrobaculum_oguniense_TE7_uid84411	NC_016885	chromosome		arCOG07012	6	S		Uncharacterized conserved protein			
	379005146	hypothetical protein Pogu_2184	No	6			1898988	1899548	+		Pyrobaculum_oguniense_TE7_uid84411	NC_016885	chromosome	clust_36								
	379005147	hypothetical protein Pogu_2185	Yes	1			1899580	1901901	+		Pyrobaculum_oguniense_TE7_uid84411	NC_016885	chromosome		arCOG09761	8	S		Uncharacterized conserved protein			
	379005148	hypothetical protein Pogu_2186	No	5			1901861	1902409	+		Pyrobaculum_oguniense_TE7_uid84411	NC_016885	chromosome	clust_180								
	379005149	DNA repair photolyase	No	0			1902509	1903294	-		Pyrobaculum_oguniense_TE7_uid84411	NC_016885	chromosome		arCOG01290	5 (-)	L	SplB	DNA repair photolyase	8E-56	COG1533	DNA repair photolyase
===										6	Pyrobaculum_oguniense_TE7_uid84411	NC_016885	chromosome									
	379005212	hypothetical protein Pogu_2250	No	0			1961022	1961240	-		Pyrobaculum_oguniense_TE7_uid84411	NC_016885	chromosome		arCOG07065	1 (-)	S		Uncharacterized conserved protein			
	379005213	hypothetical protein Pogu_2251	No	6			1961678	1962367	-		Pyrobaculum_oguniense_TE7_uid84411	NC_016885	chromosome									
	379005214	hypothetical protein Pogu_2252	No	4			1962370	1962705	-		Pyrobaculum_oguniense_TE7_uid84411	NC_016885	chromosome									
	379005215	hypothetical protein Pogu_2253	No	4			1962708	1963091	-		Pyrobaculum_oguniense_TE7_uid84411	NC_016885	chromosome	clust_253								
	379005216	hypothetical protein Pogu_2254	Yes	2			1963254	1963583	-		Pyrobaculum_oguniense_TE7_uid84411	NC_016885	chromosome									
	379005217	hypothetical protein Pogu_2255	No	0			1963724	1964104	-		Pyrobaculum_oguniense_TE7_uid84411	NC_016885	chromosome		arCOG05501	3 (-)	S		Uncharacterized conserved protein			
===										8	Pyrobaculum_oguniense_TE7_uid84411	NC_016885	chromosome									
	379005228	hypothetical protein Pogu_2266	No	0			1971301	1971603	-		Pyrobaculum_oguniense_TE7_uid84411	NC_016885	chromosome		arCOG04373	1 (-)	S		Uncharacterized conserved protein	5E-22	COG0011	Uncharacterized conserved protein
	379005229	hypothetical protein Pogu_2267	Yes	1			1971672	1971785	+		Pyrobaculum_oguniense_TE7_uid84411	NC_016885	chromosome									
	379005230	hypothetical protein Pogu_2268	No	0			1971792	1971953	-		Pyrobaculum_oguniense_TE7_uid84411	NC_016885	chromosome									
	379005231	hypothetical protein Pogu_2269	No	0			1972005	1972193	-		Pyrobaculum_oguniense_TE7_uid84411	NC_016885	chromosome									
	379005232	hypothetical protein Pogu_2270	No	1			1972308	1972787	+		Pyrobaculum_oguniense_TE7_uid84411	NC_016885	chromosome		arCOG09779	3	S		Uncharacterized conserved protein			
	379005233	hypothetical protein Pogu_2271	No	0			1972771	1973334	+		Pyrobaculum_oguniense_TE7_uid84411	NC_016885	chromosome									
	379005234	hypothetical protein Pogu_2272	No	4			1973348	1974127	+		Pyrobaculum_oguniense_TE7_uid84411	NC_016885	chromosome									
	379005235	4-alpha-glucanotransferase	No	0			1974327	1975739	-		Pyrobaculum_oguniense_TE7_uid84411	NC_016885	chromosome		arCOG04681	1 (-)	G	MalQ	4-alpha-glucanotransferase	1E-130	pfam02446	4-alpha-glucanotransferase. These enzyme
===										7	Pyrobaculum_oguniense_TE7_uid84411	NC_016885	chromosome									
	379005269	hypothetical protein Pogu_2307	No	0			2003221	2004675	-		Pyrobaculum_oguniense_TE7_uid84411	NC_016885	chromosome		arCOG04246	2 (-)	J	RtcB	RNA 3'-P ligase, RtcB family protein	2E-165	pfam01139	Uncharacterized protein family UPF0027.
	379005270	hypothetical protein Pogu_2308	No	0			2004668	2004895	-		Pyrobaculum_oguniense_TE7_uid84411	NC_016885	chromosome									
	379005271	hypothetical protein Pogu_2309	No	1			2005051	2005254	+		Pyrobaculum_oguniense_TE7_uid84411	NC_016885	chromosome									
	379005272	hypothetical protein Pogu_2310	Yes	1			2005398	2006003	-		Pyrobaculum_oguniense_TE7_uid84411	NC_016885	chromosome		arCOG09799	2	S		Uncharacterized conserved protein			
	379005273	hypothetical protein Pogu_2311	No	0			2006000	2006587	-		Pyrobaculum_oguniense_TE7_uid84411	NC_016885	chromosome		arCOG09772	4	S		Uncharacterized conserved protein			
	379005274	hypothetical protein Pogu_2312	Yes	0			2006562	2006738	-		Pyrobaculum_oguniense_TE7_uid84411	NC_016885	chromosome									
	379005275	phosphoribosylformimino-5-aminoimidazole ca	No	0			2007377	2008063	-		Pyrobaculum_oguniense_TE7_uid84411	NC_016885	chromosome		arCOG00618	1 (-)	E	HisA	Phosphoribosylformimino-5-aminoimidazole carb	1E-36	COG0106	Phosphoribosylformimino-5-aminoimidazole
===										5	Pyrobaculum_oguniense_TE7_uid84411	NC_016885	chromosome									
	379005332	hypothetical protein Pogu_2370	No	1			2059912	2060310	-		Pyrobaculum_oguniense_TE7_uid84411	NC_016885	chromosome		arCOG09840	1	S		Uncharacterized conserved protein			
	379005333	Class III signal peptide	No	1			2060346	2060774	-		Pyrobaculum_oguniense_TE7_uid84411	NC_016885	chromosome		arCOG03739	2 (-)	S		Uncharacterized conserved protein			
	379005334	hypothetical protein Pogu_2372	No	0			2061379	2061534	-		Pyrobaculum_oguniense_TE7_uid84411	NC_016885	chromosome									
	379005335	hypothetical protein Pogu_2373	No	0			2061635	2062012	-		Pyrobaculum_oguniense_TE7_uid84411	NC_016885	chromosome		arCOG09759	1	S		Uncharacterized conserved protein			
	379005336	hypothetical protein Pogu_2374	No	0			2062056	2062202	-		Pyrobaculum_oguniense_TE7_uid84411	NC_016885	chromosome									
===										5	Pyrobaculum_oguniense_TE7_uid84411	NC_016885	chromosome									
	379005608	hypothetical protein Pogu_2646	No	2			2291924	2292436	-		Pyrobaculum_oguniense_TE7_uid84411	NC_016885	chromosome									
	379005609	hypothetical protein Pogu_2647	No	0			2292467	2292865	+		Pyrobaculum_oguniense_TE7_uid84411	NC_016885	chromosome		arCOG09764	3	S		Uncharacterized conserved protein			
	379005610	radical SAM protein	No	0			2292933	2293481	+		Pyrobaculum_oguniense_TE7_uid84411	NC_016885	chromosome		arCOG00938	12 (-)	R		Radical SAM superfamily enzyme	5E-11	COG0535	Predicted Fe-S oxidoreductases
	379005611	hypothetical protein Pogu_2649	No	0			2293492	2293770	+		Pyrobaculum_oguniense_TE7_uid84411	NC_016885	chromosome									
	379005612	hypothetical protein Pogu_2650	No	2			2293772	2293924	+		Pyrobaculum_oguniense_TE7_uid84411	NC_016885	chromosome									
===										6	Pyrobaculum_oguniense_TE7_uid84411	NC_016885	chromosome									
	379005668	phosphoribosylformylglycinamidine synthase I	No	0			2346743	2347378	+		Pyrobaculum_oguniense_TE7_uid84411	NC_016885	chromosome		arCOG00102	1 (-)	F	PurL	Phosphoribosylformylglycinamidine (FGAM) synt	1E-68	COG0047	Phosphoribosylformylglycinamidine (FGAM)
	379005669	hypothetical protein Pogu_2707	Yes	1			2347436	2349502	+		Pyrobaculum_oguniense_TE7_uid84411	NC_016885	chromosome		arCOG07691	7	S		Uncharacterized conserved protein			
	379005670	hypothetical protein Pogu_2708	No	4			2349459	2350010	+		Pyrobaculum_oguniense_TE7_uid84411	NC_016885	chromosome		arCOG09784	2	S		Uncharacterized conserved protein			
	379005671	hypothetical protein Pogu_2709	No	0			2350430	2350987	-		Pyrobaculum_oguniense_TE7_uid84411	NC_016885	chromosome		arCOG05432	1	S		Uncharacterized conserved protein			
	379005672	hypothetical protein Pogu_2710	No	0			2351029	2351361	+		Pyrobaculum_oguniense_TE7_uid84411	NC_016885	chromosome									
	379005673	phosphoribosylformylglycinamidine synthase I	No	0			2351459	2353552	+		Pyrobaculum_oguniense_TE7_uid84411	NC_016885	chromosome		arCOG00641	1 (-)	F	PurL	Phosphoribosylformylglycinamidine (FGAM) synt	3E-177	COG0046	Phosphoribosylformylglycinamidine (FGAM)
===										5	Pyrobaculum_oguniense_TE7_uid84411	NC_016885	chromosome									
	379005758	hypothetical protein Pogu_2796	No	0			2432868	2433965	-		Pyrobaculum_oguniense_TE7_uid84411	NC_016885	chromosome		arCOG08027	1 (-)	S		Uncharacterized protein	0.000007	pfam02063	MARCKS family.
	379005759	hypothetical protein Pogu_2797	Yes	2			2433965	2434465	-		Pyrobaculum_oguniense_TE7_uid84411	NC_016885	chromosome									
	379005760	hypothetical protein Pogu_2798	No	0			2434462	2434827	-		Pyrobaculum_oguniense_TE7_uid84411	NC_016885	chromosome									
	379005761	hypothetical protein Pogu_2799	No	0			2434824	2435252	-		Pyrobaculum_oguniense_TE7_uid84411	NC_016885	chromosome									
	379005762	hypothetical protein Pogu_2800	No	1			2435209	2436012	-		Pyrobaculum_oguniense_TE7_uid84411	NC_016885	chromosome									
===										22	Pyrobaculum_oguniense_TE7_uid84411	NC_016886	chromosome									
	379005768	hypothetical protein	No	0		Virus_Turrivirid	2033	2338	-		Pyrobaculum_oguniense_TE7_uid84411	NC_016886	chromosome		arCOG03924	10 (-)	K	MarR	Transcriptional regulator, MarR family			
	379005769	hypothetical protein	No	0		Virus_Turrivirid	2434	2661	+		Pyrobaculum_oguniense_TE7_uid84411	NC_016886	chromosome									
	379005770	hypothetical protein	No	0		Virus_Turrivirid	2654	2872	+		Pyrobaculum_oguniense_TE7_uid84411	NC_016886	chromosome									
	379005771	hypothetical protein	No	0		Virus_Turrivirid	2883	3200	+		Pyrobaculum_oguniense_TE7_uid84411	NC_016886	chromosome									
	379005772	hypothetical protein	No	0		Virus_Turrivirid	3193	3438	+		Pyrobaculum_oguniense_TE7_uid84411	NC_016886	chromosome									
	379005773	ribbon-helix-helix protein, copG family protein	No	0		Virus_Turrivirid	3435	3668	+		Pyrobaculum_oguniense_TE7_uid84411	NC_016886	chromosome		arCOG01009	8 (-)	V		Ribbon-helix-helix protein, copG family	0.00008	COG3609	Predicted transcriptional regulators con
	379005774	hypothetical protein	No	0		Virus_Turrivirid	3691	4017	+		Pyrobaculum_oguniense_TE7_uid84411	NC_016886	chromosome									
	379005775	hypothetical protein	No	0		Virus_Turrivirid	4253	4567	+		Pyrobaculum_oguniense_TE7_uid84411	NC_016886	chromosome		arCOG05703	1 (-)	S		Uncharacterized conserved protein			
	379005776	hypothetical protein	No	0		Virus_Turrivirid	4682	4798	+		Pyrobaculum_oguniense_TE7_uid84411	NC_016886	chromosome									
	379005777	hypothetical protein	No	1		Virus_Turrivirid	4780	5151	+		Pyrobaculum_oguniense_TE7_uid84411	NC_016886	chromosome								0.0000003	pfam13868	Tumour suppressor, Mitostatin. Trichople
	379005778	hypothetical protein	No	0		Virus_Turrivirid	5148	5864	+		Pyrobaculum_oguniense_TE7_uid84411	NC_016886	chromosome								0.00009	pfam02463	RecF/RecN/SMC N terminal domain. This do
	379005779	hypothetical protein	Yes	0		Virus_Turrivirid	5861	6169	+		Pyrobaculum_oguniense_TE7_uid84411	NC_016886	chromosome									
	379005780	putative ATPase	No	0		Virus_Turrivirid	6179	6850	+		Pyrobaculum_oguniense_TE7_uid84411	NC_016886	chromosome		arCOG00280	21 (-)	L		HerA helicase			
	379005781	hypothetical protein	No	1		Virus_Turrivirid	6887	7051	+		Pyrobaculum_oguniense_TE7_uid84411	NC_016886	chromosome									
	379005782	putative Major Capsid Protein	No	0		Virus_Turrivirid	7060	7986	+		Pyrobaculum_oguniense_TE7_uid84411	NC_016886	chromosome		arCOG10335	5 (-)	Y		Major capsid protein, double jelly roll fold protein			
	379005783	hypothetical protein	No	0		Virus_Turrivirid	8057	8899	+		Pyrobaculum_oguniense_TE7_uid84411	NC_016886	chromosome									
	379005784	hypothetical protein	Yes	1		Virus_Turrivirid	8901	10382	+		Pyrobaculum_oguniense_TE7_uid84411	NC_016886	chromosome								0.00007	COG5651	PPE-repeat proteins
	379005785	hypothetical protein	No	1		Virus_Turrivirid	10379	10492	+		Pyrobaculum_oguniense_TE7_uid84411	NC_016886	chromosome									
	379005786	hypothetical protein	Yes	2		Virus_Turrivirid	10496	11026	+		Pyrobaculum_oguniense_TE7_uid84411	NC_016886	chromosome									
	379005787	hypothetical protein	No	3		Virus_Turrivirid	11090	11377	+		Pyrobaculum_oguniense_TE7_uid84411	NC_016886	chromosome									
	379005788	hypothetical protein	No	0		Virus_Turrivirid	11469	11741	+		Pyrobaculum_oguniense_TE7_uid84411	NC_016886	chromosome		arCOG04146	2 (-)	S		Uncharacterized conserved protein			
	379005789	hypothetical protein	No	1		Virus_Turrivirid	11810	12103	+		Pyrobaculum_oguniense_TE7_uid84411	NC_016886	chromosome									
===										8	Pyrobaculum_oguniense_TE7_uid84411	NC_016886	chromosome									
	379005791	hypothetical protein	No	0		Virus_Turrivirid	13526	13807	+		Pyrobaculum_oguniense_TE7_uid84411	NC_016886	chromosome									
	379005792	hypothetical protein	No	3		Virus_Turrivirid	13831	14178	+		Pyrobaculum_oguniense_TE7_uid84411	NC_016886	chromosome		arCOG05553	1 (-)	S		Uncharacterized conserved protein			
	379005793	hypothetical protein	No	0		Virus_Turrivirid	14190	14372	+		Pyrobaculum_oguniense_TE7_uid84411	NC_016886	chromosome									
	379005794	hypothetical protein	No	0		Virus_Turrivirid	14423	14947	+		Pyrobaculum_oguniense_TE7_uid84411	NC_016886	chromosome									
	379005795	hypothetical protein	No	0		Virus_Turrivirid	14996	15319	+		Pyrobaculum_oguniense_TE7_uid84411	NC_016886	chromosome									
	379005796	hypothetical protein	No	2		Virus_Turrivirid	15309	16403	+		Pyrobaculum_oguniense_TE7_uid84411	NC_016886	chromosome									
	379005797	hypothetical protein	No	0		Virus_Turrivirid	16403	16549	+		Pyrobaculum_oguniense_TE7_uid84411	NC_016886	chromosome									
	379005798	hypothetical protein	No	0		Virus_Turrivirid	16551	16802	+		Pyrobaculum_oguniense_TE7_uid84411	NC_016886	chromosome									
===										16	Pyrococcus_NA2_uid66551	NC_015474	chromosome									
	332158957	hypothetical protein PNA2_1317	No	1			1211353	1212144	+		Pyrococcus_NA2_uid66551	NC_015474	chromosome		arCOG05103	4 (-)	S		Uncharacterized conserved protein			
	332158958	hypothetical protein PNA2_1318	No	0			1212141	1212482	+		Pyrococcus_NA2_uid66551	NC_015474	chromosome									
	332158959	hypothetical protein PNA2_1319	No	2			1212491	1213225	-		Pyrococcus_NA2_uid66551	NC_015474	chromosome									
	332158960	hypothetical protein PNA2_1320	No	2			1213236	1214027	-		Pyrococcus_NA2_uid66551	NC_015474	chromosome		arCOG10043	3	S		Uncharacterized conserved protein			
	332158961	hypothetical protein PNA2_1321	No	0			1214251	1214451	+		Pyrococcus_NA2_uid66551	NC_015474	chromosome	clust_453								
	332158962	hypothetical protein PNA2_1322	No	0			1214725	1214937	+		Pyrococcus_NA2_uid66551	NC_015474	chromosome									
	332158963	hypothetical protein PNA2_1323	No	0			1215400	1215831	+		Pyrococcus_NA2_uid66551	NC_015474	chromosome		arCOG03936	6 (-)	K	AbrB	Transcriptional regulator AbrB			
	332158964	hypothetical protein PNA2_1324	No	0			1216226	1216393	+		Pyrococcus_NA2_uid66551	NC_015474	chromosome	clust_486								
	332158965	hypothetical protein PNA2_1325	No	0			1216400	1216981	+		Pyrococcus_NA2_uid66551	NC_015474	chromosome								0.00001	COG0419	ATPase involved in DNA repair
	332158966	MCM2/3/5 family DNA replication licensing fact	No	0			1216971	1218989	+		Pyrococcus_NA2_uid66551	NC_015474	chromosome		arCOG00439	14 (-)	L	MCM2	Predicted ATPase involved in replication control,	4E-91	COG1241	Predicted ATPase involved in replication
	332158967	hypothetical protein PNA2_1327	No	0			1219029	1219241	+		Pyrococcus_NA2_uid66551	NC_015474	chromosome		arCOG11301	2	S		Uncharacterized conserved protein			
	332158968	hypothetical protein PNA2_1328	No	0			1219244	1219468	+		Pyrococcus_NA2_uid66551	NC_015474	chromosome	clust_405								
	332158969	hypothetical protein PNA2_1329	No	0			1219468	1219998	+		Pyrococcus_NA2_uid66551	NC_015474	chromosome		arCOG07102	9 (-)	L		PD-(D/E)XK superfamily nuclease			
	332158970	hypothetical protein PNA2_1330	No	0			1220004	1220225	+		Pyrococcus_NA2_uid66551	NC_015474	chromosome									
	332158971	malic enzyme	No	0			1220403	1220753	+		Pyrococcus_NA2_uid66551	NC_015474	chromosome									
	332158972	hypothetical protein PNA2_1332	No	0			1221147	1221500	+		Pyrococcus_NA2_uid66551	NC_015474	chromosome		arCOG07101	4	S		Uncharacterized conserved protein			
===										6	Pyrococcus_NA2_uid66551	NC_015474	chromosome									
	332159298	hypothetical protein PNA2_1658	No	0			1549133	1549567	-		Pyrococcus_NA2_uid66551	NC_015474	chromosome									
	332159299	hypothetical protein PNA2_1659	No	0			1549519	1550127	-		Pyrococcus_NA2_uid66551	NC_015474	chromosome									
	332159300	hypothetical protein PNA2_1660	No	0			1550238	1551077	+		Pyrococcus_NA2_uid66551	NC_015474	chromosome									
	332159301	hypothetical protein PNA2_1661	No	0			1551284	1551571	-		Pyrococcus_NA2_uid66551	NC_015474	chromosome		arCOG03807	1 (-)	S		Uncharacterized conserved protein	2E-28	pfam08967	Domain of unknown function (DUF1884). Th
	332159302	hypothetical protein PNA2_1662	No	2			1551879	1552109	+		Pyrococcus_NA2_uid66551	NC_015474	chromosome		arCOG10921	4	S		Uncharacterized conserved protein			
	332159303	hypothetical protein PNA2_1663	No	12			1552112	1553254	+		Pyrococcus_NA2_uid66551	NC_015474	chromosome		arCOG10351	3	S		Uncharacterized conserved protein			
===										6	Pyrococcus_NA2_uid66551	NC_015474	chromosome									
	332159567	hypothetical protein PNA2_1928	No	3			1808522	1808785	-		Pyrococcus_NA2_uid66551	NC_015474	chromosome		arCOG07106	3	S		Uncharacterized conserved protein			
	332159568	hypothetical protein PNA2_1929	No	2			1808810	1809013	-		Pyrococcus_NA2_uid66551	NC_015474	chromosome									
	332159569	hypothetical protein PNA2_1930	No	4			1809010	1809375	-		Pyrococcus_NA2_uid66551	NC_015474	chromosome		arCOG07839	3	S		Uncharacterized conserved protein			
	332159570	hypothetical protein PNA2_1931	No	0			1810610	1810690	-		Pyrococcus_NA2_uid66551	NC_015474	chromosome		arCOG01996	17 (-)	S	SpoIIM	Uncharacterized membrane protein			
	332159571	hypothetical protein PNA2_1932	No	2			1811040	1811228	-		Pyrococcus_NA2_uid66551	NC_015474	chromosome	clust_60								
	332159572	hypothetical protein PNA2_1933	No	1			1811849	1812088	-		Pyrococcus_NA2_uid66551	NC_015474	chromosome									
===										5	Pyrococcus_ST04_uid167261	NC_017946	chromosome									
	389851638	hypothetical protein Py04_0193	Yes	2			184756	184977	+		Pyrococcus_ST04_uid167261	NC_017946	chromosome	clust_60								
	389851639	hypothetical protein Py04_0194	No	4			184982	185536	+		Pyrococcus_ST04_uid167261	NC_017946	chromosome		arCOG01996	17 (-)	S	SpoIIM	Uncharacterized membrane protein	2E-23	COG1300	Uncharacterized membrane protein
	389851640	hypothetical protein Py04_0195	No	4			185837	186193	+		Pyrococcus_ST04_uid167261	NC_017946	chromosome		arCOG07839	3	S		Uncharacterized conserved protein			
	389851641	hypothetical protein Py04_0196	No	3			186452	186721	+		Pyrococcus_ST04_uid167261	NC_017946	chromosome		arCOG07106	3	S		Uncharacterized conserved protein			
	389851642	hypothetical protein Py04_0197	No	2			186734	187024	+		Pyrococcus_ST04_uid167261	NC_017946	chromosome									
===										6	Pyrococcus_ST04_uid167261	NC_017946	chromosome									
	389852312	hypothetical protein Py04_0879	Yes	1			888854	891178	+		Pyrococcus_ST04_uid167261	NC_017946	chromosome	arCOG07560,a	arCOG07560	2 (-)	S		Cell suface protein			
	389852313	hypothetical protein Py04_0884	No	6			893981	894496	+		Pyrococcus_ST04_uid167261	NC_017946	chromosome	clust_202								
	389852314	hypothetical protein Py04_0886	No	4			895599	896117	+		Pyrococcus_ST04_uid167261	NC_017946	chromosome									
	389852315	hypothetical protein Py04_0887	Yes	6			896156	896791	+		Pyrococcus_ST04_uid167261	NC_017946	chromosome	clust_161								
	389852316	hypothetical protein Py04_0888	No	6			897514	898059	+		Pyrococcus_ST04_uid167261	NC_017946	chromosome									
	389852317	leucyl-tRNA synthetase	No	0			898396	901299	+		Pyrococcus_ST04_uid167261	NC_017946	chromosome		arCOG00809	1 (-)	J	LeuS	Leucyl-tRNA synthetase	4E-130	COG0495	Leucyl-tRNA synthetase
===										5	Pyrococcus_furiosus_COM1_uid169620	NC_018092	chromosome									
	397651186	hypothetical protein PFC_02565	Yes	0			494474	495151	+		Pyrococcus_furiosus_COM1_uid169620	NC_018092	chromosome	clust_151								
	397651187	hypothetical protein PFC_02570	No	0			495226	495366	+		Pyrococcus_furiosus_COM1_uid169620	NC_018092	chromosome		arCOG07122	3	S		Uncharacterized conserved protein			
	397651188	hypothetical protein PFC_02575	No	4			495373	495789	+		Pyrococcus_furiosus_COM1_uid169620	NC_018092	chromosome		arCOG07123	4	S		Uncharacterized conserved protein			
	397651189	hypothetical protein PFC_02580	No	0			496207	497127	-		Pyrococcus_furiosus_COM1_uid169620	NC_018092	chromosome		arCOG05724	3 (-)	V		Predicted type IV restriction endonuclease	2E-21	COG2810	Predicted type IV restriction endonuclea
	397651190	hypothetical protein PFC_02585	No	0			497139	497558	-		Pyrococcus_furiosus_COM1_uid169620	NC_018092	chromosome	clust_250								
===										8	Pyrococcus_furiosus_COM1_uid169620	NC_018092	chromosome									
	397651280	transposase	No	0			572701	573402	+		Pyrococcus_furiosus_COM1_uid169620	NC_018092	chromosome		arCOG02134	15 (-)	L		Transposase	1E-16	COG3316	Transposase and inactivated derivatives
	397651281	hypothetical protein PFC_03085	No	0			573394	573672	-		Pyrococcus_furiosus_COM1_uid169620	NC_018092	chromosome									
	397651282	hypothetical protein PFC_03090	No	0			573740	573952	-		Pyrococcus_furiosus_COM1_uid169620	NC_018092	chromosome									
	397651283	hypothetical protein PFC_03095	No	0			574104	574550	+		Pyrococcus_furiosus_COM1_uid169620	NC_018092	chromosome	clust_229								
	397651284	transposase	No	0			574571	574966	+		Pyrococcus_furiosus_COM1_uid169620	NC_018092	chromosome									
	397651285	hypothetical protein PFC_03105	No	0			574947	575327	-		Pyrococcus_furiosus_COM1_uid169620	NC_018092	chromosome	clust_272								
	397651286	glycosyl transferase family protein	No	0			575333	575992	-		Pyrococcus_furiosus_COM1_uid169620	NC_018092	chromosome		arCOG01407	1 (-)	M	RfaG	Glycosyltransferase	5E-37	COG0438	Glycosyltransferase
	397651287	hypothetical protein PFC_03115	No	0			575997	576467	-		Pyrococcus_furiosus_COM1_uid169620	NC_018092	chromosome									
===										7	Pyrococcus_furiosus_COM1_uid169620	NC_018092	chromosome									
	397651593	ABC transporter	No	0			839389	839997	-		Pyrococcus_furiosus_COM1_uid169620	NC_018092	chromosome		arCOG07131	7 (-)	V	CcmA	ABC-type multidrug transport system, ATPase co	3E-28	COG1131	ABC-type multidrug transport system, ATP
	397651594	hypothetical protein PFC_04680	No	3			839994	840422	-		Pyrococcus_furiosus_COM1_uid169620	NC_018092	chromosome	clust_242								
	397651595	hypothetical protein PFC_04685	No	0			840482	840664	+		Pyrococcus_furiosus_COM1_uid169620	NC_018092	chromosome									
	397651596	hypothetical protein PFC_04690	No	2			841551	841826	-		Pyrococcus_furiosus_COM1_uid169620	NC_018092	chromosome		arCOG10921	4	S		Uncharacterized conserved protein			
	397651597	hypothetical protein PFC_04695	No	3			842043	842324	+		Pyrococcus_furiosus_COM1_uid169620	NC_018092	chromosome	clust_366								
	397651598	hypothetical protein PFC_04700	No	2			842321	842632	+		Pyrococcus_furiosus_COM1_uid169620	NC_018092	chromosome		arCOG07137	2	S		Uncharacterized conserved protein			
	397651599	isoleucyl-tRNA ligase	No	0			842826	846026	-		Pyrococcus_furiosus_COM1_uid169620	NC_018092	chromosome		arCOG00807	4 (-)	J	IleS	Isoleucyl-tRNA synthetase		COG0060	Isoleucyl-tRNA synthetase
===										6	Pyrococcus_furiosus_COM1_uid169620	NC_018092	chromosome									
	397652016	ABC transporter	No	0			1220268	1221041	-		Pyrococcus_furiosus_COM1_uid169620	NC_018092	chromosome		arCOG00208	5 (-)	V	CcmA	ABC-type multidrug transport system, ATPase co	4E-34	COG1131	ABC-type multidrug transport system, ATP
	397652017	hypothetical protein PFC_06850	Yes	2			1221013	1221426	-		Pyrococcus_furiosus_COM1_uid169620	NC_018092	chromosome		arCOG09825	2	S		Uncharacterized conserved membrane protein			
	397652018	hypothetical protein PFC_06855	No	1			1222707	1223015	-		Pyrococcus_furiosus_COM1_uid169620	NC_018092	chromosome	clust_344								
	397652019	hypothetical protein PFC_06860	No	0			1223020	1223214	-		Pyrococcus_furiosus_COM1_uid169620	NC_018092	chromosome	clust_460								
	397652020	hypothetical protein PFC_06865	Yes	1			1223192	1223587	-		Pyrococcus_furiosus_COM1_uid169620	NC_018092	chromosome	clust_262								
	397652021	glutamine amidotransferase subunit PdxT	No	0			1223719	1224312	-		Pyrococcus_furiosus_COM1_uid169620	NC_018092	chromosome		arCOG00034	2 (-)	H	PDX2	Predicted glutamine amidotransferase involved ir	4E-66	COG0311	Predicted glutamine amidotransferase inv
===										7	Pyrococcus_furiosus_DSM_3638_uid57873	NC_003413	chromosome									
	18977052	hypothetical protein PF0680	No	0	YES		690764	691177	-		Pyrococcus_furiosus_DSM_3638_uid57873	NC_003413	chromosome		arCOG04214	2 (-)	S		Uncharacterized conserved protein	3E-39	COG0432	Uncharacterized conserved protein
	18977053	hypothetical protein PF0681	Yes	0	YES		691384	692061	+		Pyrococcus_furiosus_DSM_3638_uid57873	NC_003413	chromosome	clust_151								
	18977054	hypothetical protein PF0682	No	0	YES		692075	692257	-		Pyrococcus_furiosus_DSM_3638_uid57873	NC_003413	chromosome									
	18977055	hypothetical protein PF0683	No	0	YES		692106	692276	+		Pyrococcus_furiosus_DSM_3638_uid57873	NC_003413	chromosome		arCOG07122	3	S		Uncharacterized conserved protein			
	18977056	hypothetical protein PF0684	No	4	YES		692283	692699	+		Pyrococcus_furiosus_DSM_3638_uid57873	NC_003413	chromosome		arCOG07123	4	S		Uncharacterized conserved protein			
	18977057	hypothetical protein PF0685	No	0	YES		693117	694037	-		Pyrococcus_furiosus_DSM_3638_uid57873	NC_003413	chromosome		arCOG05724	3 (-)	V		Predicted type IV restriction endonuclease	2E-21	COG2810	Predicted type IV restriction endonuclea
	18977058	hypothetical protein PF0686	No	0	YES		694049	694468	-		Pyrococcus_furiosus_DSM_3638_uid57873	NC_003413	chromosome	clust_250								
===										6	Pyrococcus_furiosus_DSM_3638_uid57873	NC_003413	chromosome									
	18977157	transposase	No	0			771392	772093	+		Pyrococcus_furiosus_DSM_3638_uid57873	NC_003413	chromosome		arCOG02134	15 (-)	L		Transposase	1E-16	COG3316	Transposase and inactivated derivatives
	33359509	hypothetical protein PF0785.2n	No	0			772203	772664	-		Pyrococcus_furiosus_DSM_3638_uid57873	NC_003413	chromosome									

33359510	hypothetical protein PF0785.3n	No	0			772786	773244	+		Pyrococcus_furiosus_DSM_3638_uid57873	NC_003413	chromosome	clust_229									
33359508	transposase	No	0			772789	773660	+		Pyrococcus_furiosus_DSM_3638_uid57873	NC_003413	chromosome										
18977159	hypothetical protein PF0787	No	0			773641	774030	-		Pyrococcus_furiosus_DSM_3638_uid57873	NC_003413	chromosome	clust_272									
18977160	glycosyl transferase	No	0			774027	774686	-		Pyrococcus_furiosus_DSM_3638_uid57873	NC_003413	chromosome			arCOG01411	1 (-)	M	RfaG	Glycosyltransferase	5E-37	COG0438	Glycosyltransferase
===									5	Pyrococcus_furiosus_DSM_3638_uid57873	NC_003413	chromosome										
18977294	hypothetical protein PF0922	No	6			888936	889742	-		Pyrococcus_furiosus_DSM_3638_uid57873	NC_003413	chromosome								0.000005	COG0842	ABC-type multidrug transport system, per
18977295	hypothetical protein PF0923	No	2			889729	890211	-		Pyrococcus_furiosus_DSM_3638_uid57873	NC_003413	chromosome										
18977296	hypothetical protein PF0924	No	0			890208	890636	-		Pyrococcus_furiosus_DSM_3638_uid57873	NC_003413	chromosome										
18977297	heme biosynthesis protein	No	0			890633	891484	-		Pyrococcus_furiosus_DSM_3638_uid57873	NC_003413	chromosome			arCOG00938	12 (-)	R		Radical SAM superfamily enzyme	5E-22	COG0535	Predicted Fe-S oxidoreductases
18977298	hypothetical protein PF0926	No	1			891798	892025	-		Pyrococcus_furiosus_DSM_3638_uid57873	NC_003413	chromosome										
===									8	Pyrococcus_furiosus_DSM_3638_uid57873	NC_003413	chromosome										
18977462	putative ABC transporter	No	0	YES		1037290	1038027	-		Pyrococcus_furiosus_DSM_3638_uid57873	NC_003413	chromosome			arCOG07131	7 (-)	V	CcmA	ABC-type multidrug transport system, ATPase co	8E-28	COG1131	ABC-type multidrug transport system, ATP
18977463	hypothetical protein PF1091	No	3	YES		1037895	1038323	-		Pyrococcus_furiosus_DSM_3638_uid57873	NC_003413	chromosome	clust_242									
18977464	hypothetical protein PF1092	No	10	YES		1038325	1039494	-		Pyrococcus_furiosus_DSM_3638_uid57873	NC_003413	chromosome			arCOG10351	3	S		Uncharacterized conserved protein			
18977465	hypothetical protein PF1093	No	2	YES		1039452	1039727	-		Pyrococcus_furiosus_DSM_3638_uid57873	NC_003413	chromosome			arCOG10921	4	S		Uncharacterized conserved protein			
18977466	hypothetical protein PF1094	No	0	YES		1039877	1040188	-		Pyrococcus_furiosus_DSM_3638_uid57873	NC_003413	chromosome										
33359539	hypothetical protein PF1093.1n	No	3	YES		1039944	1040225	+		Pyrococcus_furiosus_DSM_3638_uid57873	NC_003413	chromosome	clust_366									
18977467	hypothetical protein PF1095	No	2	YES		1040222	1040533	+		Pyrococcus_furiosus_DSM_3638_uid57873	NC_003413	chromosome			arCOG07137	2	S		Uncharacterized conserved protein			
18977468	isoleucyl-tRNA synthetase	No	0			1040727	1043927	-		Pyrococcus_furiosus_DSM_3638_uid57873	NC_003413	chromosome			arCOG00807	4 (-)	J	IleS	Isoleucyl-tRNA synthetase		COG0060	Isoleucyl-tRNA synthetase
===									7	Pyrococcus_furiosus_DSM_3638_uid57873	NC_003413	chromosome										
18977679	hypothetical protein PF1307	No	0			1233501	1233920	-		Pyrococcus_furiosus_DSM_3638_uid57873	NC_003413	chromosome										
18977680	hypothetical protein PF1308	No	0			1234037	1234240	+		Pyrococcus_furiosus_DSM_3638_uid57873	NC_003413	chromosome			arCOG07152	1 (-)	V		DNA-binding protein, potential antitoxin AbrB/MazE fold			
33359557	hypothetical protein PF1308.2n	No	0			1234974	1235243	-		Pyrococcus_furiosus_DSM_3638_uid57873	NC_003413	chromosome	clust_372									
18977681	hypothetical protein PF1309	No	0			1235031	1235333	+		Pyrococcus_furiosus_DSM_3638_uid57873	NC_003413	chromosome										
18977682	hypothetical protein PF1310	No	0			1235314	1236804	+		Pyrococcus_furiosus_DSM_3638_uid57873	NC_003413	chromosome			arCOG10606	2	S		Uncharacterized conserved protein			
18977683	hypothetical protein PF1311	No	0			1237035	1237334	+		Pyrococcus_furiosus_DSM_3638_uid57873	NC_003413	chromosome										
18977684	hypothetical protein PF1312	No	0			1237357	1238064	+		Pyrococcus_furiosus_DSM_3638_uid57873	NC_003413	chromosome			arCOG00579	4 (-)	K	RPB9	DNA-directed RNA polymerase, subunit M/Transc	0.00000005	COG1040	Predicted amidophosphoribosyltransferase
===									7	Pyrococcus_furiosus_DSM_3638_uid57873	NC_003413	chromosome										
18977894	putative ABC transporter	No	0			1421852	1422625	-		Pyrococcus_furiosus_DSM_3638_uid57873	NC_003413	chromosome			arCOG00208	5 (-)	V	CcmA	ABC-type multidrug transport system, ATPase co	4E-34	COG1131	ABC-type multidrug transport system, ATP
18977895	hypothetical protein PF1523	No	2			1422597	1422998	-		Pyrococcus_furiosus_DSM_3638_uid57873	NC_003413	chromosome			arCOG09825	2	S		Uncharacterized conserved membrane protein			
18977896	hypothetical protein PF1524	No	6			1423007	1423798	-		Pyrococcus_furiosus_DSM_3638_uid57873	NC_003413	chromosome			arCOG09826	1	S		Uncharacterized conserved protein			
18977897	hypothetical protein PF1525	No	1			1424291	1424599	-		Pyrococcus_furiosus_DSM_3638_uid57873	NC_003413	chromosome	clust_344									
18977898	hypothetical protein PF1526	No	0			1424604	1424798	-		Pyrococcus_furiosus_DSM_3638_uid57873	NC_003413	chromosome	clust_460									
18977899	hypothetical protein PF1527	No	1			1424776	1425177	-		Pyrococcus_furiosus_DSM_3638_uid57873	NC_003413	chromosome	clust_262									
18977900	glutamine amidotransferase subunit PdxT	No	0			1425303	1425896	-		Pyrococcus_furiosus_DSM_3638_uid57873	NC_003413	chromosome			arCOG00034	2 (-)	H	PDX2	Predicted glutamine amidotransferase involved ir	4E-66	COG0311	Predicted glutamine amidotransferase inv
===									5	Pyrococcus_horikoshii_OT3_uid57753	NC_000961	chromosome										
14590299	hypothetical protein PH0391	No	0			353035	353613	-		Pyrococcus_horikoshii_OT3_uid57753	NC_000961	chromosome										
14590300	hypothetical protein PH0392	No	0			353591	354589	-		Pyrococcus_horikoshii_OT3_uid57753	NC_000961	chromosome			arCOG01400	2 (-)	Q		SAM-dependent methyltransferase			
14590301	hypothetical protein PH0393	Yes	0			354207	354605	+		Pyrococcus_horikoshii_OT3_uid57753	NC_000961	chromosome										
14590302	hypothetical protein PH0394	No	8			354586	355416	-		Pyrococcus_horikoshii_OT3_uid57753	NC_000961	chromosome										
14590303	hypothetical protein PH0395	No	6			355423	356289	-		Pyrococcus_horikoshii_OT3_uid57753	NC_000961	chromosome										
===									5	Pyrococcus_horikoshii_OT3_uid57753	NC_000961	chromosome										
33359283	hypothetical protein PHS008.3n	No	0	YES		366611	366721	+		Pyrococcus_horikoshii_OT3_uid57753	NC_000961	chromosome			arCOG09756	1	S		Uncharacterized conserved protein			
14590318	hypothetical protein PHS009	Yes	0	YES		367015	367212	+		Pyrococcus_horikoshii_OT3_uid57753	NC_000961	chromosome										
14590319	hypothetical protein PHS010	No	0	YES		367456	367656	+		Pyrococcus_horikoshii_OT3_uid57753	NC_000961	chromosome										
33359284	hypothetical protein PHS010.2n	No	0	YES		368100	368336	-		Pyrococcus_horikoshii_OT3_uid57753	NC_000961	chromosome			arCOG09832	1 (-)	V		RelB family antitoxin	0.0000002	COG1196	Chromosome segregation ATPases
33359285	hypothetical protein PHS010.3n	No	0	YES		368633	368881	+		Pyrococcus_horikoshii_OT3_uid57753	NC_000961	chromosome										
===									6	Pyrococcus_horikoshii_OT3_uid57753	NC_000961	chromosome										
14590591	hypothetical protein PH0713	No	0			635424	635897	-		Pyrococcus_horikoshii_OT3_uid57753	NC_000961	chromosome			arCOG04214	2 (-)	S		Uncharacterized conserved protein	6E-39	COG0432	Uncharacterized conserved protein
33359322	hypothetical protein PH0713.1n	No	0			636094	636300	+		Pyrococcus_horikoshii_OT3_uid57753	NC_000961	chromosome			arCOG07122	3	S		Uncharacterized conserved protein			
14590592	hypothetical protein PH0714	No	4			636307	636720	+		Pyrococcus_horikoshii_OT3_uid57753	NC_000961	chromosome			arCOG07123	4	S		Uncharacterized conserved protein			
14590593	hypothetical protein PH0715	No	2			636752	637099	+		Pyrococcus_horikoshii_OT3_uid57753	NC_000961	chromosome			arCOG07123	4	S		Uncharacterized conserved protein			
33359323	hypothetical protein PH0714.1n	No	0			637032	637139	-		Pyrococcus_horikoshii_OT3_uid57753	NC_000961	chromosome										
14590594	hypothetical protein PH0716	No	0			639031	639921	-		Pyrococcus_horikoshii_OT3_uid57753	NC_000961	chromosome			arCOG00021	1 (-)	H		Predicted transcriptional regulator fused phospho	1E-45	COG1992	Uncharacterized conserved protein
===									7	Pyrococcus_horikoshii_OT3_uid57753	NC_000961	chromosome										
14590726	hypothetical protein PH0867	No	2	YES		775061	775369	+		Pyrococcus_horikoshii_OT3_uid57753	NC_000961	chromosome			arCOG09830	1	S		Uncharacterized conserved protein			
14590727	hypothetical protein PH0868	No	4	YES		775405	775779	+		Pyrococcus_horikoshii_OT3_uid57753	NC_000961	chromosome			arCOG07839	3	S		Uncharacterized conserved protein			
14590728	hypothetical protein PH0869	No	0	YES		775767	776252	-		Pyrococcus_horikoshii_OT3_uid57753	NC_000961	chromosome										
14590729	hypothetical protein PH0870	No	4	YES		776339	776836	+		Pyrococcus_horikoshii_OT3_uid57753	NC_000961	chromosome			arCOG01996	17 (-)	S	SpoIIM	Uncharacterized membrane protein	3E-28	COG1300	Uncharacterized membrane protein
33359338	hypothetical protein PH0870.2n	No	3	YES		777220	777456	+		Pyrococcus_horikoshii_OT3_uid57753	NC_000961	chromosome			arCOG07106	3	S		Uncharacterized conserved protein			
14590730	hypothetical protein PH0871	No	4	YES		777753	778115	+		Pyrococcus_horikoshii_OT3_uid57753	NC_000961	chromosome										
33359339	hypothetical protein PH0871.1n	No	2	YES		778184	778396	+		Pyrococcus_horikoshii_OT3_uid57753	NC_000961	chromosome	clust_60									
===									9	Pyrococcus_horikoshii_OT3_uid57753	NC_000961	chromosome										
14591005	hypothetical protein PH1180	No	0	YES	integrated_ele	1067142	1068161	+		Pyrococcus_horikoshii_OT3_uid57753	NC_000961	chromosome			arCOG05286	1	S		Uncharacterized conserved protein			
14591006	hypothetical protein PH1181	No	4	YES	integrated_ele	1068183	1068614	+		Pyrococcus_horikoshii_OT3_uid57753	NC_000961	chromosome			arCOG07162	1	S		Uncharacterized conserved protein			
14591007	hypothetical protein PH1182	No	0	YES	integrated_ele	1068875	1069291	-		Pyrococcus_horikoshii_OT3_uid57753	NC_000961	chromosome			arCOG03403	5 (-)	V		PemK/MazF family toxin			
14591008	hypothetical protein PH1183	No	0		integrated_ele	1069306	1070004	-		Pyrococcus_horikoshii_OT3_uid57753	NC_000961	chromosome			arCOG07163	1	S		Uncharacterized conserved protein	0.0000001	COG0419	ATPase involved in DNA repair
14591009	hypothetical protein PH1184	No	1		integrated_ele	1070145	1070534	-		Pyrococcus_horikoshii_OT3_uid57753	NC_000961	chromosome										
14591010	hypothetical protein PH1185	No	0		integrated_ele	1070200	1070814	+		Pyrococcus_horikoshii_OT3_uid57753	NC_000961	chromosome										
33359371	hypothetical protein PH1185.1n	No	0		integrated_ele	1070820	1071074	-		Pyrococcus_horikoshii_OT3_uid57753	NC_000961	chromosome										
14591011	hypothetical protein PH1186	No	0		integrated_ele	1071081	1071833	-		Pyrococcus_horikoshii_OT3_uid57753	NC_000961	chromosome			arCOG05103	4 (-)	S		Uncharacterized conserved protein			
14591012	hypothetical protein PH1187	Yes	0		integrated_ele	1071250	1071636	+		Pyrococcus_horikoshii_OT3_uid57753	NC_000961	chromosome										
===									7	Pyrococcus_horikoshii_OT3_uid57753	NC_000961	chromosome										
33359389	hypothetical protein PH1297.2n	No	2	YES		1167163	1167495	+		Pyrococcus_horikoshii_OT3_uid57753	NC_000961	chromosome										
14591108	hypothetical protein PH1298	No	0	YES		1167611	1167964	-		Pyrococcus_horikoshii_OT3_uid57753	NC_000961	chromosome			arCOG02225	2 (-)	V	VapC	PIN domain containing protein			
14591109	hypothetical protein PH1299	No	5	YES		1168111	1168599	-		Pyrococcus_horikoshii_OT3_uid57753	NC_000961	chromosome										
14591110	hypothetical protein PH1300	No	4	YES		1168683	1169288	-		Pyrococcus_horikoshii_OT3_uid57753	NC_000961	chromosome										
33359390	hypothetical protein PH1300.1n	No	2	YES		1169203	1169475	-		Pyrococcus_horikoshii_OT3_uid57753	NC_000961	chromosome										
14591111	hypothetical protein PH1301	No	6	YES		1170018	1170605	-		Pyrococcus_horikoshii_OT3_uid57753	NC_000961	chromosome			arCOG07151	3	S		Uncharacterized conserved protein			
14591112	hypothetical protein PH1302	No	0	YES		1170607	1173189	-		Pyrococcus_horikoshii_OT3_uid57753	NC_000961	chromosome		arCOG07710,a	arCOG07710	3 (-)	S		Cell suface protein			
===									6	Pyrococcus_horikoshii_OT3_uid57753	NC_000961	chromosome										
14591605	hypothetical protein PH1858	No	0		integrated_ele	1614454	1615215	+		Pyrococcus_horikoshii_OT3_uid57753	NC_000961	chromosome			arCOG00978	1 (-)	J	GCD14	tRNA(1-methyladenosine) methyltransferase	5E-85	COG2519	tRNA(1-methyladenosine) methyltransferas
14591606	hypothetical protein PH1859	No	0		integrated_ele	1615352	1615738	-		Pyrococcus_horikoshii_OT3_uid57753	NC_000961	chromosome										
14591607	hypothetical protein PH1860	No	0		integrated_ele	1615707	1616243	-		Pyrococcus_horikoshii_OT3_uid57753	NC_000961	chromosome										
14591608	hypothetical protein PH1861	No	2		integrated_ele	1616759	1617097	+		Pyrococcus_horikoshii_OT3_uid57753	NC_000961	chromosome										
14591609	hypothetical protein PH1862	No	0		integrated_ele	1617094	1618299	-		Pyrococcus_horikoshii_OT3_uid57753	NC_000961	chromosome								0.000002	pfam06160	Septation ring formation regulator, EzrA
14591610	hypothetical protein PH1863	No	0		integrated_ele	1618414	1619421	-		Pyrococcus_horikoshii_OT3_uid57753	NC_000961	chromosome			arCOG01244	9 (-)	L		Intergrase/Recombinase	1E-68	COG4342	Uncharacterized protein conserved in arc
===									8	Pyrococcus_horikoshii_OT3_uid57753	NC_000961	chromosome										
14591615	nicotinate phosphoribosyltransferase	No	0			1623137	1624315	+		Pyrococcus_horikoshii_OT3_uid57753	NC_000961	chromosome			arCOG01481	1 (-)	H	PncB	Nicotinic acid phosphoribosyltransferase	2E-61	COG1488	Nicotinic acid phosphoribosyltransferase
33359434	hypothetical protein PH1868.1n	No	0			1624505	1624747	+		Pyrococcus_horikoshii_OT3_uid57753	NC_000961	chromosome										
14591616	hypothetical protein PH1869	No	0			1625439	1625954	+		Pyrococcus_horikoshii_OT3_uid57753	NC_000961	chromosome			arCOG07842	2	S		Uncharacterized conserved protein			
33359435	hypothetical protein PH1869.1n	No	0			1626599	1626712	+		Pyrococcus_horikoshii_OT3_uid57753	NC_000961	chromosome										
14591617	hypothetical protein PH1870	Yes	1			1626902	1627426	+		Pyrococcus_horikoshii_OT3_uid57753	NC_000961	chromosome			arCOG07149	1	S		Uncharacterized conserved protein			
14591618	hypothetical protein PH1871	No	1			1627431	1628297	+		Pyrococcus_horikoshii_OT3_uid57753	NC_000961	chromosome			arCOG07150	1	S		Uncharacterized conserved protein			
14591619	hypothetical protein PH1872	No	0			1628298	1628636	+		Pyrococcus_horikoshii_OT3_uid57753	NC_000961	chromosome										
33359436	4Fe-4S ferredoxin	No	0			1628364	1628576	-		Pyrococcus_horikoshii_OT3_uid57753	NC_000961	chromosome			arCOG00349	1 (-)	C	Fer	Ferredoxin	8E-12	pfam13459	4Fe-4S single cluster domain.
===									9	Pyrococcus_yayanosii_CH1_uid68281	NC_015680	chromosome										
337283542	hypothetical protein PYCH_00310	No	0			29980	31911	+		Pyrococcus_yayanosii_CH1_uid68281	NC_015680	chromosome			arCOG07794	1 (-)	V	McrB	GTPase subunit of restriction endonuclease	5E-19	COG1401	GTPase subunit of restriction endonuclea ; AAA domain.
337283543	hypothetical protein PYCH_00320	No	0			32347	32607	-		Pyrococcus_yayanosii_CH1_uid68281	NC_015680	chromosome			arCOG10871	4	S		Uncharacterized conserved protein			
337283544	hypothetical protein PYCH_00330	No	0			32797	32994	-		Pyrococcus_yayanosii_CH1_uid68281	NC_015680	chromosome			arCOG10871	4	S		Uncharacterized conserved protein			
337283545	hypothetical protein PYCH_00340	No	0			33162	33407	-		Pyrococcus_yayanosii_CH1_uid68281	NC_015680	chromosome			arCOG10871	4	S		Uncharacterized conserved protein			
337283546	hypothetical protein PYCH_00350	No	0			33416	33544	-		Pyrococcus_yayanosii_CH1_uid68281	NC_015680	chromosome			arCOG10871	4	S		Uncharacterized conserved protein			
337283547	hypothetical protein PYCH_00360	No	0			33561	33698	-		Pyrococcus_yayanosii_CH1_uid68281	NC_015680	chromosome										
337283548	hypothetical protein PYCH_00370	No	0			34205	34411	+		Pyrococcus_yayanosii_CH1_uid68281	NC_015680	chromosome										
337283549	hypothetical protein PYCH_00380	No	0			34492	34608	+		Pyrococcus_yayanosii_CH1_uid68281	NC_015680	chromosome										
337283550	putative transposase	No	0			34904	36121	-		Pyrococcus_yayanosii_CH1_uid68281	NC_015680	chromosome			arCOG00683	3 (-)	L		Transposase	3E-49	pfam01385	Probable transposase. This family includ ; Putative transposase DNA-binding domain.
===									8	Pyrococcus_yayanosii_CH1_uid68281	NC_015680	chromosome										
337284029	hypothetical protein PYCH_05410	No	0			449610	449729	+		Pyrococcus_yayanosii_CH1_uid68281	NC_015680	chromosome			arCOG02075	1 (-)	S		Uncharacterized conserved protein			
337284030	hypothetical protein PYCH_05440	No	0			451159	451674	-		Pyrococcus_yayanosii_CH1_uid68281	NC_015680	chromosome			arCOG11322	2	S		Uncharacterized conserved protein			
337284031	hypothetical protein PYCH_05450	No	0			451656	451880	-		Pyrococcus_yayanosii_CH1_uid68281	NC_015680	chromosome										
337284032	hypothetical protein PYCH_05460	No	0			452227	452766	-		Pyrococcus_yayanosii_CH1_uid68281	NC_015680	chromosome			arCOG11323	5	S		Uncharacterized conserved protein			
337284033	hypothetical protein PYCH_05470	No	0			452777	453691	-		Pyrococcus_yayanosii_CH1_uid68281	NC_015680	chromosome			arCOG11323	5	S		Uncharacterized conserved protein			
337284034	hypothetical protein PYCH_05480	No	0			453738	453866	-		Pyrococcus_yayanosii_CH1_uid68281	NC_015680	chromosome										
337284035	hypothetical protein PYCH_05490	Yes	2			454100	454483	-		Pyrococcus_yayanosii_CH1_uid68281	NC_015680	chromosome			arCOG10048	5	S		Uncharacterized conserved protein			
337284036	hydroxylamine reductase	No	0			454708	456063	-		Pyrococcus_yayanosii_CH1_uid68281	NC_015680	chromosome			arCOG02430	1 (-)	C		6Fe-6S prismane cluster-containing protein	5E-105	COG1151	6Fe-6S prismane cluster-containing prote
===									10	Pyrococcus_yayanosii_CH1_uid68281	NC_015680	chromosome										
337284053	hypothetical protein PYCH_05670	No	1			472907	473206	-		Pyrococcus_yayanosii_CH1_uid68281	NC_015680	chromosome			arCOG07090	2 (-)	S		Uncharacterized conserved protein			
337284054	hypothetical protein PYCH_05680	No	0			473371	473514	-		Pyrococcus_yayanosii_CH1_uid68281	NC_015680	chromosome										
337284055	hypothetical protein PYCH_05690	No	3			473513	474703	+		Pyrococcus_yayanosii_CH1_uid68281	NC_015680	chromosome			arCOG10048	5	S		Uncharacterized conserved protein			
337284056	hypothetical protein PYCH_05700	No	0			474709	475737	+		Pyrococcus_yayanosii_CH1_uid68281	NC_015680	chromosome			arCOG11322	2	S		Uncharacterized conserved protein			
337284057	hypothetical protein PYCH_05710	No	0			476311	476643	+		Pyrococcus_yayanosii_CH1_uid68281	NC_015680	chromosome										
337284058	hypothetical protein PYCH_05720	No	1			476684	477475	+		Pyrococcus_yayanosii_CH1_uid68281	NC_015680	chromosome			arCOG11323	5	S		Uncharacterized conserved protein			
337284059	hypothetical protein PYCH_05730	No	0			477516	477782	+		Pyrococcus_yayanosii_CH1_uid68281	NC_015680	chromosome										
337284060	hypothetical protein PYCH_05740	No	0			477908	478168	+		Pyrococcus_yayanosii_CH1_uid68281	NC_015680	chromosome										
337284061	hypothetical protein PYCH_05750	No	0			478227	478343	+		Pyrococcus_yayanosii_CH1_uid68281	NC_015680	chromosome										
337284062	dihydropteroate synthase	No	0			478564	479382	-		Pyrococcus_yayanosii_CH1_uid68281	NC_015680	chromosome			arCOG02817	1 (-)	H	FolC	Folylpolyglutamate synthase and Dihydropteroate	2E-41	COG0294	Dihydropteroate synthase and related enz
===									7	Pyrococcus_yayanosii_CH1_uid68281	NC_015680	chromosome										
337284260	hypothetical protein PYCH_07770	No	0			672922	673071	-		Pyrococcus_yayanosii_CH1_uid68281	NC_015680	chromosome										
337284261	hypothetical protein PYCH_07780	No	0			673241	673363	+		Pyrococcus_yayanosii_CH1_uid68281	NC_015680	chromosome										
337284262	hypothetical protein PYCH_07790	No	0			673541	673744	-		Pyrococcus_yayanosii_CH1_uid68281	NC_015680	chromosome										
337284263	hypothetical protein PYCH_07800	No	0			673744	673986	-		Pyrococcus_yayanosii_CH1_uid68281	NC_015680	chromosome			arCOG03235	4 (-)	L		Predicted ATP-dependent endonuclease of the OLD family			
337284264	hypothetical protein PYCH_07810	No	0			673986	674120	-		Pyrococcus_yayanosii_CH1_uid68281	NC_015680	chromosome										
337284265	hypothetical protein PYCH_07820	No	0			674185	674904	-		Pyrococcus_yayanosii_CH1_uid68281	NC_015680	chromosome										
337284266	hypothetical protein PYCH_07830	No	0			674910	675941	-		Pyrococcus_yayanosii_CH1_uid68281	NC_015680	chromosome										
===									7	Pyrococcus_yayanosii_CH1_uid68281	NC_015680	chromosome										
337284487	RNA 2'-phosphotransferase (kptA)	No	0			882896	883438	-		Pyrococcus_yayanosii_CH1_uid68281	NC_015680	chromosome			arCOG04063	1 (-)	J	KptA	RNA:NAD 2'-phosphotransferase	2E-41	COG1859	RNA:NAD 2'-phosphotransferase
337284488	hypothetical protein PYCH_10070	No	4			883615	884064	+		Pyrococcus_yayanosii_CH1_uid68281	NC_015680	chromosome										
337284489	hypothetical protein PYCH_10080	No	2			884058	884327	+		Pyrococcus_yayanosii_CH1_uid68281	NC_015680	chromosome										
337284490	hypothetical protein PYCH_10090	No	1			884296	884433	-		Pyrococcus_yayanosii_CH1_uid68281	NC_015680	chromosome										
337284491	hypothetical protein PYCH_10100	No	0			884571	884702	-		Pyrococcus_yayanosii_CH1_uid68281	NC_015680	chromosome										
337284492	Heat shock protein/ Zn-dependent protease wi	No	2			884787	885167	-		Pyrococcus_yayanosii_CH1_uid68281	NC_015680	chromosome			arCOG01334	4 (-)	O	HtpX	Zn-dependent protease with chaperone function	8E-10	COG0501	Zn-dependent protease with chaperone fun
337284493	Heat shock protein/ Zn-dependent protease wi	No	5			885194	885739	-		Pyrococcus_yayanosii_CH1_uid68281	NC_015680	chromosome										
===									6	Pyrococcus_yayanosii_CH1_uid68281	NC_015680	chromosome										
337284736	S-layer family protein	No	0			1114916	1116187	-		Pyrococcus_yayanosii_CH1_uid68281	NC_015680	chromosome		arCOG03419,a	arCOG03419	1 (-)	R		Predicted transglutaminase-like protease	0.0001	pfam05124	S-layer like family, C-terminal region.
337284737	hypothetical protein PYCH_12650	Yes	0			1116150	1117685	-		Pyrococcus_yayanosii_CH1_uid68281	NC_015680	chromosome										
337284738	hypothetical protein PYCH_12660	No	0			1118017	1118334	+		Pyrococcus_yayanosii_CH1_uid68281	NC_015680	chromosome	clust_335									
337284739	hypothetical protein PYCH_12680	No	3			1118636	1119112	-		Pyrococcus_yayanosii_CH1_uid68281	NC_015680	chromosome										
337284740	hypothetical protein PYCH_12670	No	0			1119216	1119422	+		Pyrococcus_yayanosii_CH1_uid68281	NC_015680	chromosome										
337284741	quinolinate synthetase	No	0			1119928	1120878	+		Pyrococcus_yayanosii_CH1_uid68281	NC_015680	chromosome			arCOG04459	3 (-)	H	NadA	Quinolinate synthase	2E-103	pfam02445	Quinolinate synthetase A protein. Quinol
===									5	Pyrococcus_yayanosii_CH1_uid68281	NC_015680	chromosome										
337284743	hypothetical protein PYCH_12730	Yes	2			1122053	1122313	+		Pyrococcus_yayanosii_CH1_uid68281	NC_015680	chromosome			arCOG10921	4	S		Uncharacterized conserved protein			
337284744	hypothetical protein PYCH_12740	No	9			1122310	1123509	+		Pyrococcus_yayanosii_CH1_uid68281	NC_015680	chromosome			arCOG10351	3	S		Uncharacterized conserved protein			
337284745	hypothetical protein PYCH_12750	No	1			1123794	1123964	+		Pyrococcus_yayanosii_CH1_uid68281	NC_015680	chromosome										
337284746	putative ABC transporter	No	0			1123954	1124562	+		Pyrococcus_yayanosii_CH1_uid68281	NC_015680	chromosome			arCOG07131	7 (-)	V	CcmA	ABC-type multidrug transport system, ATPase co	2E-23	COG1131	ABC-type multidrug transport system, ATP
337284747	hypothetical protein PYCH_12770	Yes	1			1124748	1125269	+		Pyrococcus_yayanosii_CH1_uid68281	NC_015680	chromosome										
===									5	Pyrococcus_yayanosii_CH1_uid68281	NC_015680	chromosome										
337284889	hypothetical protein PYCH_14210	No	2			1245785	1246042	+		Pyrococcus_yayanosii_CH1_uid68281	NC_015680	chromosome										
337284890	hypothetical protein PYCH_14220	No	1			1246039	1246848	+		Pyrococcus_yayanosii_CH1_uid68281	NC_015680	chromosome			arCOG07165	1	S		Uncharacterized conserved protein			
337284891	hypothetical protein PYCH_14230	No	1			1246860	1247339	+		Pyrococcus_yayanosii_CH1_uid68281	NC_015680	chromosome			arCOG05105	1 (-)	S		Uncharacterized conserved protein			
337284892	hypothetical protein PYCH_14240	No	2			1247349	1247540	+		Pyrococcus_yayanosii_CH1_uid68281	NC_015680	chromosome										
337284893	hypothetical protein PYCH_14250	No	0			1247621	1247980	+		Pyrococcus_yayanosii_CH1_uid68281	NC_015680	chromosome			arCOG10430	1	S		Uncharacterized conserved protein			
===									8	Pyrococcus_yayanosii_CH1_uid68281	NC_015680	chromosome										
337284897	hypothetical protein PYCH_14290	No	0			1251122	1251394	+		Pyrococcus_yayanosii_CH1_uid68281	NC_015680	chromosome	clust_365									
337284898	SpoVT/AbrB family transcriptional regulator	No	0			1252357	1252794	+		Pyrococcus_yayanosii_CH1_uid68281	NC_015680	chromosome			arCOG03936	6 (-)	K	AbrB	Transcriptional regulator AbrB			
337284899	hypothetical protein PYCH_14310	No	0			1253199	1253372	+		Pyrococcus_yayanosii_CH1_uid68281	NC_015680	chromosome	clust_486									
337284900	hypothetical protein PYCH_14320	No	0			1253379	1253954	+		Pyrococcus_yayanosii_CH1_uid68281	NC_015680	chromosome			arCOG07161	3	S		Uncharacterized conserved protein			
337284901	hypothetical protein PYCH_14330	No	0			1253951	1254202	+		Pyrococcus_yayanosii_CH1_uid68281	NC_015680	chromosome			arCOG11301	2	S		Uncharacterized conserved protein			
337284902	hypothetical protein PYCH_14340	No	0			1254207	1254446	+		Pyrococcus_yayanosii_CH1_uid68281	NC_015680	chromosome	clust_405									
337284903	hypothetical protein PYCH_14350	No	0			1254446	1254964	+		Pyrococcus_yayanosii_CH1_uid68281	NC_015680	chromosome			arCOG07102	9 (-)	L		PD-(D/E)XK superfamily nuclease			
337284904	hypothetical protein PYCH_14360	No	0			1255180	1255521	+		Pyrococcus_yayanosii_CH1_uid68281	NC_015680	chromosome			arCOG07101	4	S		Uncharacterized conserved protein			
===									6	Pyrococcus_yayanosii_CH1_uid68281	NC_015680	chromosome										
337284977	site-specific recombinase, phage integrase far	No	0			1321772	1322464	+		Pyrococcus_yayanosii_CH1_uid68281	NC_015680	chromosome			arCOG01241	31 (-)	L	XerC	Integrase	0.0001	pfam00589	Phage integrase family. Members of this
337284978	hypothetical protein PYCH_15120	No	0			1322704	1323789	-		Pyrococcus_yayanosii_CH1_uid68281	NC_015680	chromosome										
337284979	hypothetical protein PYCH_15130	No	0			1324081	1324203	-		Pyrococcus_yayanosii_CH1_uid68281	NC_015680	chromosome										
337284980	hypothetical protein PYCH_15140	No	0			1324489	1324800	-		Pyrococcus_yayanosii_CH1_uid68281	NC_015680	chromosome										
337284981	hypothetical protein PYCH_15150	No	0			1324945	1325340	+		Pyrococcus_yayanosii_CH1_uid68281	NC_015680	chromosome										
337284982	5-methylcytosine restriction system componen	No	0			1325742	1327148	-		Pyrococcus_yayanosii_CH1_uid68281	NC_015680	chromosome			arCOG05102	3 (-)	V	McrC	McrBC 5-methylcytosine restriction system comp	2E-42	pfam10117	McrBC 5-methylcytosine restriction syste
===									7	Pyrococcus_yayanosii_CH1_uid68281	NC_015680	chromosome										
337285108	L-fuculose 1-phosphate aldolase (fucA)	No	0			1454839	1455402	+		Pyrococcus_yayanosii_CH1_uid68281	NC_015680	chromosome			arCOG04226	2 (-)	G		Fuculose-1-phosphate aldolase	2E-34	pfam00596	Class II Aldolase and Adducin N-terminal
337285109	hypothetical protein PYCH_16450	No	0			1455758	1455925	-		Pyrococcus_yayanosii_CH1_uid68281	NC_015680	chromosome										
337285110	hypothetical protein PYCH_16460	No	0			1456171	1456677	+		Pyrococcus_yayanosii_CH1_uid68281	NC_015680	chromosome			arCOG10048	5	S		Uncharacterized conserved protein			
337285111	hypothetical protein PYCH_16470	No	0			1456772	1457443	+		Pyrococcus_yayanosii_CH1_uid68281	NC_015680	chromosome			arCOG11323	5	S		Uncharacterized conserved protein			
337285112	hypothetical protein PYCH_16480	No	1			1457433	1458122	+		Pyrococcus_yayanosii_CH1_uid68281	NC_015680	chromosome										
337285113	hypothetical protein PYCH_16490	No	0			1458417	1458551	+		Pyrococcus_yayanosii_CH1_uid68281	NC_015680	chromosome										
337285114	DNA primase small subunit	No	0			1458632	1459669	-		Pyrococcus_yayanosii_CH1_uid68281	NC_015680	chromosome			arCOG04110	4 (-)	L	PRI1	Eukaryotic-type DNA primase, catalytic (small) su	2E-57	COG1467	Eukaryotic-type DNA primase, catalytic (
===									7	Pyrolobus_fumarii_1A_uid73415	NC_015931	chromosome										
347522841	phosphoenolpyruvate-protein phosphotransfer	No	0			255145	255423	+		Pyrolobus_fumarii_1A_uid73415	NC_015931	chromosome										
347522842	5-methyltetrahydropteroyltriglutamate--homocys	No	0			255506	256525	+		Pyrolobus_fumarii_1A_uid73415	NC_015931	chromosome			arCOG01876	2 (-)	E	MetE	Methionine synthase II (cobalamin-independent)	2E-76	COG0620	Methionine synthase II (cobalamin-indepe
347522843	hypothetical protein Pyrfu_0289	No	0			256534	257082	-		Pyrolobus_fumarii_1A_uid73415	NC_015931	chromosome			arCOG08619	1	S		Metal-binding cluster containing protein			
347522844	hypothetical protein Pyrfu_0290	No	7			257115	257813	-		Pyrolobus_fumarii_1A_uid73415	NC_015931	chromosome										
347522845	hypothetical protein Pyrfu_0291	No	2			257899	258843	-		Pyrolobus_fumarii_1A_uid73415	NC_015931	chromosome										
347522846	3-methyl-2-oxobutanoatehydroxymethyltransfer	No	0			258846	259667	-		Pyrolobus_fumarii_1A_uid73415	NC_015931	chromosome			arCOG00584	1 (-)	H	PanB	Ketopantoate hydroxymethyltransferase	5E-98	COG0413	Ketopantoate hydroxymethyltransferase
347522847	hypothetical protein Pyrfu_0293	Yes	1			260335	262410	-		Pyrolobus_fumarii_1A_uid73415	NC_015931	chromosome										
===									6	Pyrolobus_fumarii_1A_uid73415	NC_015931	chromosome										
347522851	translation factor pelota	No	0			266039	267112	+		Pyrolobus_fumarii_1A_uid73415	NC_015931	chromosome			arCOG01741	1 (-)	J	PelA	Release factor eRF1	2E-73	COG1537	Predicted RNA-binding proteins
347522852	hypothetical protein Pyrfu_0298	No	0			267155	267787	+		Pyrolobus_fumarii_1A_uid73415	NC_015931	chromosome			arCOG07464	1	S		Uncharacterized conserved protein			
347522853	hypothetical protein Pyrfu_0299	No	0			267790	268398	+		Pyrolobus_fumarii_1A_uid73415	NC_015931	chromosome										
347522854	hypothetical protein Pyrfu_0300	No	0			268401	268814	-		Pyrolobus_fumarii_1A_uid73415	NC_015931	chromosome										
347522855	hypothetical protein Pyrfu_0301	No	0			268875	269099	+		Pyrolobus_fumarii_1A_uid73415	NC_015931	chromosome			arCOG06094	1	S		Uncharacterized conserved protein			
347522856	hypothetical protein Pyrfu_0302	No	0			269091	270023	-		Pyrolobus_fumarii_1A_uid73415	NC_015931	chromosome			arCOG00464	2 (-)	L	AlkA	3-methyladenine DNA glycosylase/8-oxoguanine DNA glycosylase			
===									11	Pyrolobus_fumarii_1A_uid73415	NC_015931	chromosome										
347522876	hypothetical protein Pyrfu_0322	No	0			290512	290751	+		Pyrolobus_fumarii_1A_uid73415	NC_015931	chromosome										
347522877	hypothetical protein Pyrfu_0323	No	0			291129	291830	-		Pyrolobus_fumarii_1A_uid73415	NC_015931	chromosome										
347522878	hypothetical protein Pyrfu_0324	No	0			292881	293867	-		Pyrolobus_fumarii_1A_uid73415	NC_015931	chromosome			arCOG01244	9 (-)	L		Intergrase/Recombinase			
347522879	hypothetical protein Pyrfu_0325	No	0			294624	294878	+		Pyrolobus_fumarii_1A_uid73415	NC_015931	chromosome										
347522880	hypothetical protein Pyrfu_0326	No	2			294886	295839	+		Pyrolobus_fumarii_1A_uid73415	NC_015931	chromosome										
347522881	hypothetical protein Pyrfu_0327	No	0			295968	299189	-		Pyrolobus_fumarii_1A_uid73415	NC_015931	chromosome			arCOG04816	4 (-)	U		TraG/TraD/VirD4 family enzyme, ATPase	0.0001	pfam12846	AAA-like domain. This family of domains
347522882	hypothetical protein Pyrfu_0328	No	0			299285	299500	-		Pyrolobus_fumarii_1A_uid73415	NC_015931	chromosome	clust_424									
347522883	hypothetical protein Pyrfu_0329	No	2			299578	299931	+		Pyrolobus_fumarii_1A_uid73415	NC_015931	chromosome										
347522884	hypothetical protein Pyrfu_0330	Yes	1			299936	301168	+		Pyrolobus_fumarii_1A_uid73415	NC_015931	chromosome										
347522885	hypothetical protein Pyrfu_0331	No	1			301155	303404	-		Pyrolobus_fumarii_1A_uid73415	NC_015931	chromosome										
347522886	PilT protein domain containing protein	No	0			303873	304316	-		Pyrolobus_fumarii_1A_uid73415	NC_015931	chromosome			arCOG00730	1 (-)	V		PIN domain containing protein	0.000009	COG1848	Predicted nucleic acid-binding protein,
===									8	Pyrolobus_fumarii_1A_uid73415	NC_015931	chromosome										
347522889	hypothetical protein Pyrfu_0335	No	0			304936	305238	-		Pyrolobus_fumarii_1A_uid73415	NC_015931	chromosome			arCOG03590	2 (-)	V		Addiction module toxin, Txe/YoeB family			
347522890	hypothetical protein Pyrfu_0336	Yes	1			305291	307123	-		Pyrolobus_fumarii_1A_uid73415	NC_015931	chromosome										
347522891	hypothetical protein Pyrfu_0337	No	1			307165	307557	-		Pyrolobus_fumarii_1A_uid73415	NC_015931	chromosome										
347522892	hypothetical protein Pyrfu_0338	No	1			307574	308023	-		Pyrolobus_fumarii_1A_uid73415	NC_015931	chromosome										
347522893	hypothetical protein Pyrfu_0339	No	0			308092	308796	-		Pyrolobus_fumarii_1A_uid73415	NC_015931	chromosome										
347522894	hypothetical protein Pyrfu_0340	No	0			308811	309761	+		Pyrolobus_fumarii_1A_uid73415	NC_015931	chromosome										
347522895	hypothetical protein Pyrfu_0341	No	0			309758	310417	+		Pyrolobus_fumarii_1A_uid73415	NC_015931	chromosome										
347522896	hypothetical protein Pyrfu_0342	No	1			310422	310874	-		Pyrolobus_fumarii_1A_uid73415	NC_015931	chromosome			arCOG03871	6 (-)	N		Flagellar protein G			
===									9	Pyrolobus_fumarii_1A_uid73415	NC_015931	chromosome										
347522903	type II secretion system protein E	No	0			317881	319515	-		Pyrolobus_fumarii_1A_uid73415	NC_015931	chromosome			arCOG01817	8 (-)	N	VirB11	Type IV secretory pathway, VirB11 component, o	6E-63	COG0630	Type IV secretory pathway, VirB11 compon
347522904	hypothetical protein Pyrfu_0350	No	2			319512	319754	-		Pyrolobus_fumarii_1A_uid73415	NC_015931	chromosome										
347522905	hypothetical protein Pyrfu_0351	No	6			319793	320968	-		Pyrolobus_fumarii_1A_uid73415	NC_015931	chromosome								0.000005	COG5373	Predicted membrane protein
347522906	hypothetical protein Pyrfu_0352	No	2			320988	321377	-		Pyrolobus_fumarii_1A_uid73415	NC_015931	chromosome										
347522907	hypothetical protein Pyrfu_0353	No	2			321358	321786	-		Pyrolobus_fumarii_1A_uid73415	NC_015931	chromosome										
347522908	hypothetical protein Pyrfu_0354	Yes	0			321853	321966	-		Pyrolobus_fumarii_1A_uid73415	NC_015931	chromosome										
347522909	Zinc finger, C2H2	No	0			321969	322484	-		Pyrolobus_fumarii_1A_uid73415	NC_015931	chromosome		arCOG07930,a	arCOG07930	3 (-)	S		Zinc finger C2H2-type			
347522910	hypothetical protein Pyrfu_0356	Yes	0			322487	322768	-		Pyrolobus_fumarii_1A_uid73415	NC_015931	chromosome										
347522911	hypothetical protein Pyrfu_0357	No	0			322765	323109	-		Pyrolobus_fumarii_1A_uid73415	NC_015931	chromosome										
===									5	Pyrolobus_fumarii_1A_uid73415	NC_015931	chromosome										
347522915	hypothetical protein Pyrfu_0361	No	0			326032	326712	+		Pyrolobus_fumarii_1A_uid73415	NC_015931	chromosome			arCOG09998	2	S		Uncharacterized conserved protein			
347522916	hypothetical protein Pyrfu_0362	No	2			327328	327987	+		Pyrolobus_fumarii_1A_uid73415	NC_015931	chromosome										
347522917	transcriptional regulator	No	0			327984	328445	-		Pyrolobus_fumarii_1A_uid73415	NC_015931	chromosome			arCOG00724	7 (-)	K		Predicted transcriptional regulator, PadR family			
347522918	putative transporter	No	4			328537	328950	-		Pyrolobus_fumarii_1A_uid73415	NC_015931	chromosome										
347522919	hypothetical protein Pyrfu_0365	No	0			329047	329358	-		Pyrolobus_fumarii_1A_uid73415	NC_015931	chromosome										
===									7	Pyrolobus_fumarii_1A_uid73415	NC_015931	chromosome										
347522968	hypothetical protein Pyrfu_0415	No	0			387851	388132	-		Pyrolobus_fumarii_1A_uid73415	NC_015931	chromosome										
347522969	hypothetical protein Pyrfu_0416	No	2			388231	388500	-		Pyrolobus_fumarii_1A_uid73415	NC_015931	chromosome										
347522970	hypothetical protein Pyrfu_0417	No	0			388600	388722	-		Pyrolobus_fumarii_1A_uid73415	NC_015931	chromosome										
347522971	hypothetical protein Pyrfu_0418	No	0			391647	392096	-		Pyrolobus_fumarii_1A_uid73415	NC_015931	chromosome			arCOG02221	2 (-)	V		PIN domain containing protein			
347522972	hypothetical protein Pyrfu_0419	No	0			392083	392430	-		Pyrolobus_fumarii_1A_uid73415	NC_015931	chromosome										
347522973	hypothetical protein Pyrfu_0420	No	0			392624	392797	+		Pyrolobus_fumarii_1A_uid73415	NC_015931	chromosome										
347522974	hypothetical protein Pyrfu_0421	No	0			392868	393497	-		Pyrolobus_fumarii_1A_uid73415	NC_015931	chromosome										
===									5	Pyrolobus_fumarii_1A_uid73415	NC_015931	chromosome										
347522988	hypothetical protein Pyrfu_0435	No	1			406651	407046	-		Pyrolobus_fumarii_1A_uid73415	NC_015931	chromosome								0.0000007	pfam01920	Prefoldin subunit. This family includes
347522989	hypothetical protein Pyrfu_0436	No	0			407256	407465	-		Pyrolobus_fumarii_1A_uid73415	NC_015931	chromosome										
347522990	hypothetical protein Pyrfu_0437	No	0			407500	407757	-		Pyrolobus_fumarii_1A_uid73415	NC_015931	chromosome			arCOG02270	1 (-)	S		Uncharacterized conserved small protein	3E-12	COG5428	Uncharacterized conserved small protein
347522991	hypothetical protein Pyrfu_0438	No	0			407729	407950	-		Pyrolobus_fumarii_1A_uid73415	NC_015931	chromosome										
347522992	hypothetical protein Pyrfu_0439	No	0			408437	408715	-		Pyrolobus_fumarii_1A_uid73415	NC_015931	chromosome										
===									8	Pyrolobus_fumarii_1A_uid73415	NC_015931	chromosome										
347523019	hypothetical protein Pyrfu_0466	Yes	0			436858	436968	-		Pyrolobus_fumarii_1A_uid73415	NC_015931	chromosome										
347523020	hypothetical protein Pyrfu_0467	No	0			436975	437223	-		Pyrolobus_fumarii_1A_uid73415	NC_015931	chromosome										
347523021	ABC transporter-like protein	No	0			438895	439563	-		Pyrolobus_fumarii_1A_uid73415	NC_015931	chromosome			arCOG00194	14 (-)	V	CcmA	ABC-type multidrug transport system, ATPase co	8E-21	COG1131	ABC-type multidrug transport system, ATP
347523022	hypothetical protein Pyrfu_0469	Yes	0			439565	439921	-		Pyrolobus_fumarii_1A_uid73415	NC_015931	chromosome										
347523023	sodium:galactoside symporter family protein	No	6			439959	440711	-		Pyrolobus_fumarii_1A_uid73415	NC_015931	chromosome										
347523024	hypothetical protein Pyrfu_0471	Yes	3			440714	441505	-		Pyrolobus_fumarii_1A_uid73415	NC_015931	chromosome										
347523025	hypothetical protein Pyrfu_0472	No	1			441517	442176	-		Pyrolobus_fumarii_1A_uid73415	NC_015931	chromosome										
347523026	hypothetical protein Pyrfu_0473	No	0			442528	444180	-		Pyrolobus_fumarii_1A_uid73415	NC_015931	chromosome			arCOG06103	2 (-)	V		Predicted viral immunity protein, COG1517 superfamily, contains C-terminal PIN domain			
===									24	Pyrolobus_fumarii_1A_uid73415	NC_015931	chromosome										
347523071	hypothetical protein Pyrfu_0518	No	0			487577	488017	-		Pyrolobus_fumarii_1A_uid73415	NC_015931	chromosome			arCOG05472	1 (-)	S		Uncharacterized conserved protein			
347523072	hypothetical protein Pyrfu_0519	No	0			488470	488706	-		Pyrolobus_fumarii_1A_uid73415	NC_015931	chromosome										
347523073	hypothetical protein Pyrfu_0520	No	0			488822	489019	-		Pyrolobus_fumarii_1A_uid73415	NC_015931	chromosome										
347523074	hypothetical protein Pyrfu_0521	No	0			489050	489286	-		Pyrolobus_fumarii_1A_uid73415	NC_015931	chromosome										
347523075	hypothetical protein Pyrfu_0522	No	0			489316	489588	-		Pyrolobus_fumarii_1A_uid73415	NC_015931	chromosome										
347523076	hypothetical protein Pyrfu_0523	No	0			489606	489776	+		Pyrolobus_fumarii_1A_uid73415	NC_015931	chromosome										
347523077	death-on-curing family protein	No	0			489751	490158	-		Pyrolobus_fumarii_1A_uid73415	NC_015931	chromosome			arCOG06831	2 (-)	R	Doc	Prophage maintenance system killer protein			
347523078	hypothetical protein Pyrfu_0525	No	2			490162	490365	-		Pyrolobus_fumarii_1A_uid73415	NC_015931	chromosome										
347523079	hypothetical protein Pyrfu_0526	No	3			490542	491090	-		Pyrolobus_fumarii_1A_uid73415	NC_015931	chromosome										
347523080	hypothetical protein Pyrfu_0527	No	2			491174	491518	-		Pyrolobus_fumarii_1A_uid73415	NC_015931	chromosome										
347523081	hypothetical protein Pyrfu_0528	No	0			491508	491690	-		Pyrolobus_fumarii_1A_uid73415	NC_015931	chromosome										
347523082	hypothetical protein Pyrfu_0529	No	0			491672	492112	-		Pyrolobus_fumarii_1A_uid73415	NC_015931	chromosome										
347523083	hypothetical protein Pyrfu_0530	No	0			492093	492458	-		Pyrolobus_fumarii_1A_uid73415	NC_015931	chromosome										
347523084	hypothetical protein Pyrfu_0531	No	0			492773	493216	+		Pyrolobus_fumarii_1A_uid73415	NC_015931	chromosome										
347523085	hypothetical protein Pyrfu_0532	No	5			493129	493839	-		Pyrolobus_fumarii_1A_uid73415	NC_015931	chromosome										
347523086	hypothetical protein Pyrfu_0533	No	0			493931	494551	-		Pyrolobus_fumarii_1A_uid73415	NC_015931	chromosome										
347523087	hypothetical protein Pyrfu_0534	No	1			494703	496424	+		Pyrolobus_fumarii_1A_uid73415	NC_015931	chromosome										
347523088	transcriptional regulator	No	0			496847	497293	-		Pyrolobus_fumarii_1A_uid73415	NC_015931	chromosome			arCOG00724	7 (-)	K		Predicted transcriptional regulator, PadR family			
347523089	hypothetical protein Pyrfu_0536	No	1			497340	497456	-		Pyrolobus_fumarii_1A_uid73415	NC_015931	chromosome										
347523090	hypothetical protein Pyrfu_0537	No	0			497443	497997	-		Pyrolobus_fumarii_1A_uid73415	NC_015931	chromosome										
347523091	hypothetical protein Pyrfu_0538	No	0			498087	498737	+		Pyrolobus_fumarii_1A_uid73415	NC_015931	chromosome										
347523092	hypothetical protein Pyrfu_0539	No	0			498734	499000	+		Pyrolobus_fumarii_1A_uid73415	NC_015931	chromosome			arCOG03590	2 (-)	V		Addiction module toxin, Txe/YoeB family			
347523093	hypothetical protein Pyrfu_0540	No	0			498997	500976	-		Pyrolobus_fumarii_1A_uid73415	NC_015931	chromosome										
347523094	hypothetical protein Pyrfu_0541	No	0			502325	502465	+		Pyrolobus_fumarii_1A_uid73415	NC_015931	chromosome										
===									5	Pyrolobus_fumarii_1A_uid73415	NC_015931	chromosome										
347523134	hypothetical protein Pyrfu_0582	No	1			537037	537717	+		Pyrolobus_fumarii_1A_uid73415	NC_015931	chromosome										
347523135	hypothetical protein Pyrfu_0583	No	1			537757	540558	+		Pyrolobus_fumarii_1A_uid73415	NC_015931	chromosome										
347523136	hypothetical protein Pyrfu_0584	No	1			540949	541107	+		Pyrolobus_fumarii_1A_uid73415	NC_015931	chromosome										
347523137	PaREP1 family protein	No	0			541081	541725	-		Pyrolobus_fumarii_1A_uid73415	NC_015931	chromosome			arCOG03722	3 (-)	V		HEPN domain containing protein			
347523138	hypothetical protein Pyrfu_0586	No	0			541842	542186	+		Pyrolobus_fumarii_1A_uid73415	NC_015931	chromosome										
===									5	Pyrolobus_fumarii_1A_uid73415	NC_015931	chromosome										
347523180	hypothetical protein Pyrfu_0629	No	0			575641	576912	-		Pyrolobus_fumarii_1A_uid73415	NC_015931	chromosome										
347523181	PaREP1 family protein	No	0			577288	577848	+		Pyrolobus_fumarii_1A_uid73415	NC_015931	chromosome			arCOG03712	4 (-)	V		HEPN domain containing protein	2E-11	pfam05942	Archaeal PaREP1/PaREP8 family. This fami
347523182	hypothetical protein Pyrfu_0631	No	0			578653	578817	-		Pyrolobus_fumarii_1A_uid73415	NC_015931	chromosome										
347523183	hypothetical protein Pyrfu_0632	No	0			578987	579376	-		Pyrolobus_fumarii_1A_uid73415	NC_015931	chromosome										
347523184	hypothetical protein Pyrfu_0633	No	1			579324	579674	-		Pyrolobus_fumarii_1A_uid73415	NC_015931	chromosome								0.000008	pfam01456	Mucin-like glycoprotein. This family of
===									6	Pyrolobus_fumarii_1A_uid73415	NC_015931	chromosome										
347523282	biotin/lipoate A/B protein ligase	No	0			678816	679577	+		Pyrolobus_fumarii_1A_uid73415	NC_015931	chromosome			arCOG01939	1 (-)	H	LplA	Lipoate-protein ligase A	7E-42	COG0095	Lipoate-protein ligase A
347523283	hypothetical protein Pyrfu_0732	No	0			679547	679840	+		Pyrolobus_fumarii_1A_uid73415	NC_015931	chromosome										
347523284	hypothetical protein Pyrfu_0733	No	0			679967	680074	-		Pyrolobus_fumarii_1A_uid73415	NC_015931	chromosome										
347523285	hypothetical protein Pyrfu_0734	No	0			680043	681005	-		Pyrolobus_fumarii_1A_uid73415	NC_015931	chromosome			arCOG10956	1	S		Uncharacterized conserved protein			
347523286	hypothetical protein Pyrfu_0735	No	1			681125	681274	+		Pyrolobus_fumarii_1A_uid73415	NC_015931	chromosome										
347523287	50S ribosomal protein L40e	No	0			681650	681832	+		Pyrolobus_fumarii_1A_uid73415	NC_015931	chromosome			arCOG04049	2 (-)	J	RPL40A	Ribosomal protein L40E	9E-12	COG1552	Ribosomal protein L40E
===									6	Pyrolobus_fumarii_1A_uid73415	NC_015931	chromosome										
347523563	ABC transporter-like protein	No	0			954080	954976	-		Pyrolobus_fumarii_1A_uid73415	NC_015931	chromosome			arCOG00194	14 (-)	V	CcmA	ABC-type multidrug transport system, ATPase co	7E-26	COG1131	ABC-type multidrug transport system, ATP
347523564	hypothetical protein Pyrfu_1014	No	0			955177	955518	+		Pyrolobus_fumarii_1A_uid73415	NC_015931	chromosome										
347523565	hypothetical protein Pyrfu_1015	No	1			955638	956750	+		Pyrolobus_fumarii_1A_uid73415	NC_015931	chromosome										
347523566	hypothetical protein Pyrfu_1016	No	2			956753	957133	+		Pyrolobus_fumarii_1A_uid73415	NC_015931	chromosome										
347523567	hypothetical protein Pyrfu_1017	No	7			957123	958088	+		Pyrolobus_fumarii_1A_uid73415	NC_015931	chromosome										
347523568	DNA-cytosine methyltransferase	No	0			958134	959096	-		Pyrolobus_fumarii_1A_uid73415	NC_015931	chromosome			arCOG04157	7 (-)	L	Dcm	Site-specific DNA methylase	1E-75	COG0270	Site-specific DNA methylase
===									5	Pyrolobus_fumarii_1A_uid73415	NC_015931	chromosome										
347523642	hypothetical protein Pyrfu_1092	No	0			1031779	1032168	+		Pyrolobus_fumarii_1A_uid73415	NC_015931	chromosome										
347523643	hypothetical protein Pyrfu_1093	No	0			1032157	1033026	-		Pyrolobus_fumarii_1A_uid73415	NC_015931	chromosome										
347523644	hypothetical protein Pyrfu_1094	No	0			1033183	1033554	+		Pyrolobus_fumarii_1A_uid73415	NC_015931	chromosome			arCOG02734	5 (-)	S		NifX family protein			
347523645	transcription regulator protein	No	0			1033882	1034145	+		Pyrolobus_fumarii_1A_uid73415	NC_015931	chromosome										
347523646	hypothetical protein Pyrfu_1096	No	0			1034164	1034847	+		Pyrolobus_fumarii_1A_uid73415	NC_015931	chromosome										
===									5	Pyrolobus_fumarii_1A_uid73415	NC_015931	chromosome										
347523683	hypothetical protein Pyrfu_1136	No	0			1070904	1071029	+		Pyrolobus_fumarii_1A_uid73415	NC_015931	chromosome										
347523684	hypothetical protein Pyrfu_1137	No	1			1071115	1071495	+		Pyrolobus_fumarii_1A_uid73415	NC_015931	chromosome			arCOG11052	1	S		KID-like repeats containing protein, paREP15 fam	0.000002	COG1196	Chromosome segregation ATPases
347523685	hypothetical protein Pyrfu_1138	No	0			1071532	1071747	-		Pyrolobus_fumarii_1A_uid73415	NC_015931	chromosome										
347523686	AbrB family transcriptional regulator	No	0			1071957	1072205	+		Pyrolobus_fumarii_1A_uid73415	NC_015931	chromosome			arCOG00820	1 (-)	K	AbrB	Transcriptional regulator AbrB	0.00000003	COG2002	Regulators of stationary/sporulation gen
347523687	hypothetical protein Pyrfu_1140	No	0			1072174	1072371	+		Pyrolobus_fumarii_1A_uid73415	NC_015931	chromosome										
===									9	Pyrolobus_fumarii_1A_uid73415	NC_015931	chromosome										
347523723	hypothetical protein Pyrfu_1178	No	0			1102839	1102949	+		Pyrolobus_fumarii_1A_uid73415	NC_015931	chromosome										
347523724	hypothetical protein Pyrfu_1179	No	0			1103360	1103746	-		Pyrolobus_fumarii_1A_uid73415	NC_015931	chromosome			arCOG06398	3 (-)	S		Uncharacterized conserved protein			
347523725	hypothetical protein Pyrfu_1180	No	0			1103760	1104005	-		Pyrolobus_fumarii_1A_uid73415	NC_015931	chromosome										
347523726	hypothetical protein Pyrfu_1181	No	0			1104033	1104368	-		Pyrolobus_fumarii_1A_uid73415	NC_015931	chromosome										
347523727	hypothetical protein Pyrfu_1182	No	0			1104865	1104975	-		Pyrolobus_fumarii_1A_uid73415	NC_015931	chromosome										
347523728	hypothetical protein Pyrfu_1183	No	0			1104978	1105100	-		Pyrolobus_fumarii_1A_uid73415	NC_015931	chromosome										
347523729	hypothetical protein Pyrfu_1184	No	0			1105381	1105557	-		Pyrolobus_fumarii_1A_uid73415	NC_015931	chromosome										
347523730	hypothetical protein Pyrfu_1185	No	0			1105625	1105756	-		Pyrolobus_fumarii_1A_uid73415	NC_015931	chromosome										
347523731	cyclic nucleotide-binding protein	No	0			1106102	1107973	+		Pyrolobus_fumarii_1A_uid73415	NC_015931	chromosome			arCOG01307	3 (-)	D	Vps4	Cell division ATPase of the AAA+ class, ESCRT	0.00000002	COG0714	MoxR-like ATPases
===									5	Pyrolobus_fumarii_1A_uid73415	NC_015931	chromosome										
347523766	hypothetical protein Pyrfu_1224	No	1			1138316	1139545	-		Pyrolobus_fumarii_1A_uid73415	NC_015931	chromosome										

347523767	hypothetical protein Pyrfu_1225	No	0		1139877	1140008	+		Pyrolobus_fumarii_1A_uid73415	NC_015931	chromosome									
347523768	hypothetical protein Pyrfu_1226	No	1		1139974	1140894	-		Pyrolobus_fumarii_1A_uid73415	NC_015931	chromosome									
347523769	SMC domain containing protein	No	0		1141165	1142619	+		Pyrolobus_fumarii_1A_uid73415	NC_015931	chromosome	arCOG03237,a	arCOG03237	1 (-)	R		ATPase, predicted component of phage defense	0.000002	COG4637	Predicted ATPase
347523770	hypothetical protein Pyrfu_1228	No	0		1142616	1143224	+		Pyrolobus_fumarii_1A_uid73415	NC_015931	chromosome									
===								5	Pyrolobus_fumarii_1A_uid73415	NC_015931	chromosome									
347523796	hypothetical protein Pyrfu_1255	No	0		1163738	1164475	-		Pyrolobus_fumarii_1A_uid73415	NC_015931	chromosome									
347523797	hypothetical protein Pyrfu_1256	No	0		1164512	1165024	-		Pyrolobus_fumarii_1A_uid73415	NC_015931	chromosome									
347523798	PaREP1 family protein	No	0		1165117	1165698	-		Pyrolobus_fumarii_1A_uid73415	NC_015931	chromosome		arCOG03712	4 (-)	V		HEPN domain containing protein	2E-14	pfam05942	Archaeal PaREP1/PaREP8 family. This fami
347523799	hypothetical protein Pyrfu_1258	No	2		1165898	1166539	-		Pyrolobus_fumarii_1A_uid73415	NC_015931	chromosome									
347523800	hypothetical protein Pyrfu_1259	No	1		1166671	1167597	-		Pyrolobus_fumarii_1A_uid73415	NC_015931	chromosome									
===								5	Pyrolobus_fumarii_1A_uid73415	NC_015931	chromosome									
347523807	hypothetical protein Pyrfu_1266	No	0		1173282	1173506	+		Pyrolobus_fumarii_1A_uid73415	NC_015931	chromosome									
347523808	hypothetical protein Pyrfu_1267	No	0		1173490	1173663	+		Pyrolobus_fumarii_1A_uid73415	NC_015931	chromosome									
347523809	hypothetical protein Pyrfu_1268	No	0		1173758	1173946	+		Pyrolobus_fumarii_1A_uid73415	NC_015931	chromosome									
347523810	phosphoadenosine phosphosulfate reductase	No	0		1174399	1176408	-		Pyrolobus_fumarii_1A_uid73415	NC_015931	chromosome	arCOG00073,a	arCOG00073	2 (-)	E	CysH	PAPS reductase related enzyme fused to RNA-b	2E-24	COG0175	3'-phosphoadenosine 5'-phosphosulfate su ; PUA domain (predicted RNA-binding domain ; Uncharacterized Fe-S protein
347523811	hypothetical protein Pyrfu_1270	No	0		1176608	1177579	+		Pyrolobus_fumarii_1A_uid73415	NC_015931	chromosome									
===								7	Pyrolobus_fumarii_1A_uid73415	NC_015931	chromosome									
347524103	hypothetical protein Pyrfu_1564	No	0		1465294	1465653	+		Pyrolobus_fumarii_1A_uid73415	NC_015931	chromosome		arCOG03878	1 (-)	S		Uncharacterized conserved protein			
347524104	hypothetical protein Pyrfu_1565	No	1		1465708	1466313	-		Pyrolobus_fumarii_1A_uid73415	NC_015931	chromosome		arCOG10992	1	S		Uncharacterized conserved protein			
347524105	hypothetical protein Pyrfu_1566	No	0		1466373	1466600	+		Pyrolobus_fumarii_1A_uid73415	NC_015931	chromosome									
347524106	selenocysteine-specific translation elongation f	No	0		1466766	1467005	+		Pyrolobus_fumarii_1A_uid73415	NC_015931	chromosome									
347524107	hypothetical protein Pyrfu_1568	No	0		1466982	1467587	-		Pyrolobus_fumarii_1A_uid73415	NC_015931	chromosome									
347524108	hypothetical protein Pyrfu_1569	No	0		1467629	1467985	+		Pyrolobus_fumarii_1A_uid73415	NC_015931	chromosome		arCOG00653	1 (-)	S		Uncharacterized conserved protein			
347524109	hypothetical protein Pyrfu_1570	No	0		1468049	1468483	+		Pyrolobus_fumarii_1A_uid73415	NC_015931	chromosome									
===								8	Pyrolobus_fumarii_1A_uid73415	NC_015931	chromosome									
347524439	quinolinate synthetase complex subunit A	No	0		1776183	1777244	-		Pyrolobus_fumarii_1A_uid73415	NC_015931	chromosome		arCOG04459	3 (-)	H	NadA	Quinolinate synthase	1E-85	pfam02445	Quinolinate synthetase A protein. Quinol
347524440	hypothetical protein Pyrfu_1905	No	0		1777397	1777876	-		Pyrolobus_fumarii_1A_uid73415	NC_015931	chromosome									
347524441	hypothetical protein Pyrfu_1906	No	0		1778157	1778384	-		Pyrolobus_fumarii_1A_uid73415	NC_015931	chromosome	clust_424								
347524442	hypothetical protein Pyrfu_1907	No	3		1778459	1778806	+		Pyrolobus_fumarii_1A_uid73415	NC_015931	chromosome									
347524443	hypothetical protein Pyrfu_1908	No	1		1778803	1778994	+		Pyrolobus_fumarii_1A_uid73415	NC_015931	chromosome									
347524444	hypothetical protein Pyrfu_1909	No	0		1779013	1779228	-		Pyrolobus_fumarii_1A_uid73415	NC_015931	chromosome									
347524445	hypothetical protein Pyrfu_1910	No	0		1779225	1779620	-		Pyrolobus_fumarii_1A_uid73415	NC_015931	chromosome									
347524446	hypothetical protein Pyrfu_1911	No	0		1779602	1780447	-		Pyrolobus_fumarii_1A_uid73415	NC_015931	chromosome		arCOG03216	1 (-)	S		Uncharacterized conserved protein	2E-10	pfam04016	Domain of unknown function (DUF364). Thi
===								9	Staphylothermus_hellenicus_DSM_12710_uid458	NC_014205	chromosome									
297526205	hypothetical protein Shell_0188	No	0		162628	162810	+		Staphylothermus_hellenicus_DSM_12710_uid458	NC_014205	chromosome									
297526206	hypothetical protein Shell_0189	Yes	1		163060	163773	+		Staphylothermus_hellenicus_DSM_12710_uid458	NC_014205	chromosome									
297526207	peptidase S26B, signal peptidase	No	3		163886	165076	+		Staphylothermus_hellenicus_DSM_12710_uid458	NC_014205	chromosome		arCOG01739	2 (-)	U	LepB	Signal peptidase I			
297526208	hypothetical protein Shell_0191	No	4		165069	165761	+		Staphylothermus_hellenicus_DSM_12710_uid458	NC_014205	chromosome									
297526209	hypothetical protein Shell_0192	Yes	1		165791	167329	+		Staphylothermus_hellenicus_DSM_12710_uid458	NC_014205	chromosome									
297526210	hypothetical protein Shell_0193	No	0		168338	169246	+		Staphylothermus_hellenicus_DSM_12710_uid458	NC_014205	chromosome		arCOG04155	2 (-)	L		HhH-GPD superfamily base excision DNA repair protein			
297526211	hypothetical protein Shell_0194	No	0		169243	169698	+		Staphylothermus_hellenicus_DSM_12710_uid458	NC_014205	chromosome									
297526212	hypothetical protein Shell_0195	Yes	3		169854	170753	+		Staphylothermus_hellenicus_DSM_12710_uid458	NC_014205	chromosome									
297526213	hypothetical protein Shell_0196	Yes	0		171259	172521	-		Staphylothermus_hellenicus_DSM_12710_uid458	NC_014205	chromosome									
===								7	Staphylothermus_hellenicus_DSM_12710_uid458	NC_014205	chromosome									
297526490	AAA ATPase central domain-containing protein	No	0		439026	440297	-		Staphylothermus_hellenicus_DSM_12710_uid458	NC_014205	chromosome		arCOG01307	3 (-)	D	Vps4	Cell division ATPase of the AAA+ class, ESCRT	7E-11	COG0464	ATPases of the AAA+ class
297526491	hypothetical protein Shell_0485	No	0		440324	440944	-		Staphylothermus_hellenicus_DSM_12710_uid458	NC_014205	chromosome							0.00002	COG1196	Chromosome segregation ATPases
297526492	hypothetical protein Shell_0486	No	0		440950	441579	-		Staphylothermus_hellenicus_DSM_12710_uid458	NC_014205	chromosome									
297526493	hypothetical protein Shell_0487	No	0		441749	442402	+		Staphylothermus_hellenicus_DSM_12710_uid458	NC_014205	chromosome									
297526494	hypothetical protein Shell_0488	No	6		442427	443476	-		Staphylothermus_hellenicus_DSM_12710_uid458	NC_014205	chromosome									
297526495	hypothetical protein Shell_0489	No	0		443495	444391	-		Staphylothermus_hellenicus_DSM_12710_uid458	NC_014205	chromosome									
297526496	hypothetical protein Shell_0490	No	0		444571	446463	+		Staphylothermus_hellenicus_DSM_12710_uid458	NC_014205	chromosome		arCOG09748	2 (-)	S		Uncharacterized conserved protein	2E-21	COG0419	ATPase involved in DNA repair
===								8	Staphylothermus_hellenicus_DSM_12710_uid458	NC_014205	chromosome									
297526501	hypothetical protein Shell_0495	No	0		453013	453495	+		Staphylothermus_hellenicus_DSM_12710_uid458	NC_014205	chromosome									
297526502	hypothetical protein Shell_0496	Yes	0		453793	454224	-		Staphylothermus_hellenicus_DSM_12710_uid458	NC_014205	chromosome									
297526503	serine/threonine protein kinase	No	0		454246	456078	-		Staphylothermus_hellenicus_DSM_12710_uid458	NC_014205	chromosome		arCOG03682	2 (-)	R	SPS1	Membrane associated serine/threonine protein ki	1E-28	pfam00069	Protein kinase domain.
297526504	hypothetical protein Shell_0498	No	0		456054	456428	-		Staphylothermus_hellenicus_DSM_12710_uid458	NC_014205	chromosome									
297526505	hypothetical protein Shell_0499	No	0		457424	457555	-		Staphylothermus_hellenicus_DSM_12710_uid458	NC_014205	chromosome									
297526506	hypothetical protein Shell_0500	No	0		457704	458846	+		Staphylothermus_hellenicus_DSM_12710_uid458	NC_014205	chromosome									
297526507	hypothetical protein Shell_0501	No	0		459125	459262	-		Staphylothermus_hellenicus_DSM_12710_uid458	NC_014205	chromosome									
297526508	hypothetical protein Shell_0502	No	0		459262	459489	-		Staphylothermus_hellenicus_DSM_12710_uid458	NC_014205	chromosome		arCOG02218	1 (-)	V		RHH/CopG DNA binding protein			
===								6	Staphylothermus_hellenicus_DSM_12710_uid458	NC_014205	chromosome									
297526549	DNA polymerase B region	No	0		496981	498927	+		Staphylothermus_hellenicus_DSM_12710_uid458	NC_014205	chromosome		arCOG00329	1 (-)	L	PolB	DNA polymerase elongation subunit (family B), in	6E-36	COG0417	DNA polymerase elongation subunit (famil
297526550	hypothetical protein Shell_0546	Yes	1		499627	500205	+		Staphylothermus_hellenicus_DSM_12710_uid458	NC_014205	chromosome									
297526551	hypothetical protein Shell_0547	Yes	3		500279	501163	+		Staphylothermus_hellenicus_DSM_12710_uid458	NC_014205	chromosome									
297526552	hypothetical protein Shell_0548	No	6		501164	501931	+		Staphylothermus_hellenicus_DSM_12710_uid458	NC_014205	chromosome							0.0001	COG3307	Lipid A core - O-antigen ligase and rela
297526553	hypothetical protein Shell_0549	No	1		501921	502304	+		Staphylothermus_hellenicus_DSM_12710_uid458	NC_014205	chromosome									
297526554	ABC transporter-like protein	No	0		502295	503095	+		Staphylothermus_hellenicus_DSM_12710_uid458	NC_014205	chromosome		arCOG00194	14 (-)	V	CcmA	ABC-type multidrug transport system, ATPase co	1E-27	COG1131	ABC-type multidrug transport system, ATP
===								6	Staphylothermus_hellenicus_DSM_12710_uid458	NC_014205	chromosome									
297526845	ABC transporter-like protein	No	0		833593	834252	-		Staphylothermus_hellenicus_DSM_12710_uid458	NC_014205	chromosome		arCOG00922	4 (-)	V	SalX	ABC-type antimicrobial peptide transport system,	6E-41	COG1136	ABC-type antimicrobial peptide transport
297526846	hypothetical protein Shell_0861	No	14		834249	835838	-		Staphylothermus_hellenicus_DSM_12710_uid458	NC_014205	chromosome									
297526847	hypothetical protein Shell_0862	No	4		835825	836985	-		Staphylothermus_hellenicus_DSM_12710_uid458	NC_014205	chromosome									
297526848	hypothetical protein Shell_0863	No	3		836982	837839	-		Staphylothermus_hellenicus_DSM_12710_uid458	NC_014205	chromosome									
297526849	hypothetical protein Shell_0864	No	0		838139	838522	-		Staphylothermus_hellenicus_DSM_12710_uid458	NC_014205	chromosome	clust_276								
297526850	hypothetical protein Shell_0865	No	4		838565	839119	-		Staphylothermus_hellenicus_DSM_12710_uid458	NC_014205	chromosome		arCOG02887	1 (-)	S		Predicted membrane protein	2E-27	COG4743	Predicted membrane protein
===								5	Staphylothermus_hellenicus_DSM_12710_uid458	NC_014205	chromosome									
297527027	hypothetical protein Shell_1048	No	14		1008115	1010145	-		Staphylothermus_hellenicus_DSM_12710_uid458	NC_014205	chromosome		arCOG11053	2	S		Uncharacterized conserved membrane protein			
297527028	hypothetical protein Shell_1049	Yes	9		1010250	1014680	-		Staphylothermus_hellenicus_DSM_12710_uid458	NC_014205	chromosome	arCOG02312,a	arCOG02312	2 (-)	V	SalY	ABC-type antimicrobial peptide transport system,	0.0000004	COG4591	ABC-type transport system, involved in l
297527029	hypothetical protein Shell_1050	Yes	2		1014795	1016618	-		Staphylothermus_hellenicus_DSM_12710_uid458	NC_014205	chromosome	clust_68								
297527030	hypothetical protein Shell_1051	No	1		1016732	1017094	-		Staphylothermus_hellenicus_DSM_12710_uid458	NC_014205	chromosome	clust_296								
297527031	hypothetical protein Shell_1052	No	0		1017084	1017935	-		Staphylothermus_hellenicus_DSM_12710_uid458	NC_014205	chromosome	clust_117								
===								5	Staphylothermus_hellenicus_DSM_12710_uid458	NC_014205	chromosome									
297527230	hypothetical protein Shell_1257	No	1		1212451	1213314	-		Staphylothermus_hellenicus_DSM_12710_uid458	NC_014205	chromosome	clust_114								
297527231	hypothetical protein Shell_1258	No	6		1213305	1213904	-		Staphylothermus_hellenicus_DSM_12710_uid458	NC_014205	chromosome	clust_174								
297527232	hypothetical protein Shell_1259	No	0		1213911	1214771	-		Staphylothermus_hellenicus_DSM_12710_uid458	NC_014205	chromosome	clust_116								
297527233	ATPase	No	0		1214794	1215837	-		Staphylothermus_hellenicus_DSM_12710_uid458	NC_014205	chromosome		arCOG00435	2 (-)	R		MoxR-like ATPase	8E-60	COG0714	MoxR-like ATPases
297527234	hypothetical protein Shell_1261	No	0		1215850	1216701	-		Staphylothermus_hellenicus_DSM_12710_uid458	NC_014205	chromosome		arCOG07758	2	S		Uncharacterized conserved protein			
===								7	Staphylothermus_marinus_F1_uid58719	NC_009033	chromosome									
126466099	hypothetical protein Smar_1205	No	0		1172917	1173768	+		Staphylothermus_marinus_F1_uid58719	NC_009033	chromosome		arCOG07758	2	S		Uncharacterized conserved protein			
126466100	ATPase	No	0		1173781	1174824	+		Staphylothermus_marinus_F1_uid58719	NC_009033	chromosome		arCOG00435	2 (-)	R		MoxR-like ATPase	8E-60	COG0714	MoxR-like ATPases
126466101	hypothetical protein Smar_1207	No	0		1174847	1175707	+		Staphylothermus_marinus_F1_uid58719	NC_009033	chromosome	clust_116								
126466102	hypothetical protein Smar_1208	No	6		1175714	1176313	+		Staphylothermus_marinus_F1_uid58719	NC_009033	chromosome	clust_174								
126466103	hypothetical protein Smar_1209	No	1		1176304	1177167	+		Staphylothermus_marinus_F1_uid58719	NC_009033	chromosome	clust_114								
126466104	von Willebrand factor, type A	No	3		1178072	1179001	-		Staphylothermus_marinus_F1_uid58719	NC_009033	chromosome		arCOG02900	3 (-)	R		Uncharacterized protein containing a von Willebrand factor type A (vWA) domain			
126466105	hypothetical protein Smar_1212	No	0		1179006	1179866	-		Staphylothermus_marinus_F1_uid58719	NC_009033	chromosome									
===								5	Staphylothermus_marinus_F1_uid58719	NC_009033	chromosome									
126466285	hypothetical protein Smar_1395	No	0		1358420	1359268	+		Staphylothermus_marinus_F1_uid58719	NC_009033	chromosome	clust_117								
126466286	hypothetical protein Smar_1396	No	0		1359258	1359620	+		Staphylothermus_marinus_F1_uid58719	NC_009033	chromosome	clust_296								
126466287	hypothetical protein Smar_1397	Yes	2		1359723	1361546	+		Staphylothermus_marinus_F1_uid58719	NC_009033	chromosome	clust_68								
126466288	hypothetical protein Smar_1398	Yes	9		1361656	1366086	+		Staphylothermus_marinus_F1_uid58719	NC_009033	chromosome		arCOG02312	2 (-)	V	SalY	ABC-type antimicrobial peptide transport system,	0.0000005	COG4591	ABC-type transport system, involved in l
126466289	hypothetical protein Smar_1399	No	14		1366192	1368222	+		Staphylothermus_marinus_F1_uid58719	NC_009033	chromosome		arCOG11053	2	S		Uncharacterized conserved membrane protein			
===								5	Staphylothermus_marinus_F1_uid58719	NC_009033	chromosome									
126466382	hypothetical protein Smar_1494	No	0		1464519	1464752	+		Staphylothermus_marinus_F1_uid58719	NC_009033	chromosome									
126466383	DEAD/DEAH box helicase domain-containing p	No	0		1464777	1467068	+		Staphylothermus_marinus_F1_uid58719	NC_009033	chromosome		arCOG00555	5 (-)	R		Distinct helicase family with a unique C-terminal d	2E-110	COG1205	Distinct helicase family with a unique C
126466384	hypothetical protein Smar_1496	No	2		1467185	1467952	-		Staphylothermus_marinus_F1_uid58719	NC_009033	chromosome									
126466385	hypothetical protein Smar_1497	No	0		1468042	1468344	-		Staphylothermus_marinus_F1_uid58719	NC_009033	chromosome									
126466386	hypothetical protein Smar_1498	No	0		1468395	1468685	-		Staphylothermus_marinus_F1_uid58719	NC_009033	chromosome									
===								8	Staphylothermus_marinus_F1_uid58719	NC_009033	chromosome									
126466419	hypothetical protein Smar_1533	No	0	YES	1503298	1503510	+		Staphylothermus_marinus_F1_uid58719	NC_009033	chromosome									
126466420	PaREP1/PaREP8 domain-contain protein	No	0	YES	1503485	1503868	+		Staphylothermus_marinus_F1_uid58719	NC_009033	chromosome		arCOG03722	3 (-)	V		HEPN domain containing protein			
126466421	hypothetical protein Smar_1536	No	0	YES	1504742	1504906	-		Staphylothermus_marinus_F1_uid58719	NC_009033	chromosome									
126466422	hypothetical protein Smar_1537	No	0		1505123	1505653	-		Staphylothermus_marinus_F1_uid58719	NC_009033	chromosome							2E-10	COG3551	Uncharacterized protein conserved in bac
126466423	hypothetical protein Smar_1538	No	0		1506258	1506641	+		Staphylothermus_marinus_F1_uid58719	NC_009033	chromosome	clust_276								
126466424	polysaccharide biosynthesis protein	No	12		1506803	1508107	-		Staphylothermus_marinus_F1_uid58719	NC_009033	chromosome		arCOG02214	2 (-)	R	RfbX	Polysaccharide biosynthesis protein, Mvin family	5E-15	COG2244	Membrane protein involved in the export
126466425	hypothetical protein Smar_1540	No	2		1508470	1508712	+		Staphylothermus_marinus_F1_uid58719	NC_009033	chromosome									
126466426	hypothetical protein Smar_1541	No	0		1508753	1509019	-		Staphylothermus_marinus_F1_uid58719	NC_009033	chromosome		arCOG07689	2	S		Uncharacterized conserved protein			
===								9	Sulfolobus_acidocaldarius_DSM_639_uid58379	NC_007181	chromosome									
70606317	conjugative plasmid protein	No	0		407221	407430	-		Sulfolobus_acidocaldarius_DSM_639_uid58379	NC_007181	chromosome									
70606318	conjugative plasmid protein	No	0		407482	407775	-		Sulfolobus_acidocaldarius_DSM_639_uid58379	NC_007181	chromosome		arCOG03423	3 (-)	K	MarR	Transcriptional regulator, MarR family			
70606319	hypothetical protein Saci_0490	No	2		407848	408144	+		Sulfolobus_acidocaldarius_DSM_639_uid58379	NC_007181	chromosome									
70606320	hypothetical protein Saci_0491	No	0		408515	409345	+		Sulfolobus_acidocaldarius_DSM_639_uid58379	NC_007181	chromosome									
70606321	hypothetical protein Saci_0492	No	0		409591	410127	-		Sulfolobus_acidocaldarius_DSM_639_uid58379	NC_007181	chromosome		arCOG06959	1	S		Uncharacterized conserved protein			
70606322	hypothetical protein Saci_0493	No	0		410152	410511	-		Sulfolobus_acidocaldarius_DSM_639_uid58379	NC_007181	chromosome									
70606323	hypothetical protein Saci_0494	No	0		411379	412086	+		Sulfolobus_acidocaldarius_DSM_639_uid58379	NC_007181	chromosome		arCOG09711	1	S		Uncharacterized conserved protein			
70606324	hypothetical protein Saci_0495	No	0		412079	412927	+		Sulfolobus_acidocaldarius_DSM_639_uid58379	NC_007181	chromosome		arCOG07186	2 (-)	V		PIN domain			
70606325	hypothetical protein Saci_0496	No	0		413083	413667	-		Sulfolobus_acidocaldarius_DSM_639_uid58379	NC_007181	chromosome									
===								8	Sulfolobus_acidocaldarius_DSM_639_uid58379	NC_007181	chromosome									
70606334	conjugative plasmid protein	No	2		419327	419875	+		Sulfolobus_acidocaldarius_DSM_639_uid58379	NC_007181	chromosome		arCOG08560	1 (-)	S		Uncharacterized conserved protein			
70606335	conjugative plasmid protein	No	2		419872	420159	-		Sulfolobus_acidocaldarius_DSM_639_uid58379	NC_007181	chromosome		arCOG08561	3	S		Uncharacterized conserved protein			
70606336	conjugative plasmid membrane protein	No	4		420208	420777	+		Sulfolobus_acidocaldarius_DSM_639_uid58379	NC_007181	chromosome		arCOG08562	3	S		Uncharacterized conserved protein			
70606337	hypothetical protein Saci_0508	No	2		420456	420761	-		Sulfolobus_acidocaldarius_DSM_639_uid58379	NC_007181	chromosome									
70606338	conjugative plasmid protein	No	0		420774	420929	-		Sulfolobus_acidocaldarius_DSM_639_uid58379	NC_007181	chromosome		arCOG08563	3	S		Uncharacterized conserved protein			
70606339	conjugative plasmid protein	No	0		421091	421513	+		Sulfolobus_acidocaldarius_DSM_639_uid58379	NC_007181	chromosome		arCOG07296	1 (-)	S		Uncharacterized conserved protein			
70606340	conjugative plasmid protein	No	0		421702	421857	-		Sulfolobus_acidocaldarius_DSM_639_uid58379	NC_007181	chromosome		arCOG08563	3	S		Uncharacterized conserved protein			
70606341	conjugative plasmid protein	No	0		422019	422438	+		Sulfolobus_acidocaldarius_DSM_639_uid58379	NC_007181	chromosome									
===								7	Sulfolobus_acidocaldarius_DSM_639_uid58379	NC_007181	chromosome									
70606347	hypothetical protein Saci_0524	No	5		431941	432435	-		Sulfolobus_acidocaldarius_DSM_639_uid58379	NC_007181	chromosome									
70606348	hypothetical protein Saci_0525	No	2		432501	433196	-		Sulfolobus_acidocaldarius_DSM_639_uid58379	NC_007181	chromosome		arCOG00288	8 (-)	C	NfnB	Nitroreductase	0.000000002	pfam00881	Nitroreductase family. The nitroreductas
70606349	hypothetical protein Saci_0526	No	0		433208	434512	-		Sulfolobus_acidocaldarius_DSM_639_uid58379	NC_007181	chromosome		arCOG02883	3	S		Uncharacterized conserved protein	1E-19	pfam02624	YcaO-like family.
70606350	hypothetical protein Saci_0527	No	0		434491	435201	-		Sulfolobus_acidocaldarius_DSM_639_uid58379	NC_007181	chromosome									
70606351	hypothetical protein Saci_0528	No	1		435210	435341	-		Sulfolobus_acidocaldarius_DSM_639_uid58379	NC_007181	chromosome									
70606352	hypothetical protein Saci_0529	No	6		435514	436173	-		Sulfolobus_acidocaldarius_DSM_639_uid58379	NC_007181	chromosome									
70606353	hypothetical protein Saci_0530	No	6		436289	437026	+		Sulfolobus_acidocaldarius_DSM_639_uid58379	NC_007181	chromosome		arCOG05371	4 (-)	S		Uncharacterized conserved protein			
===								5	Sulfolobus_acidocaldarius_DSM_639_uid58379	NC_007181	chromosome									
70606356	hypothetical protein Saci_0532	No	6		438205	438801	-		Sulfolobus_acidocaldarius_DSM_639_uid58379	NC_007181	chromosome									
70606355	hypothetical protein Saci_0533	No	0		438773	439246	+		Sulfolobus_acidocaldarius_DSM_639_uid58379	NC_007181	chromosome									
70606357	hypothetical protein Saci_0534	No	2		439141	439323	-		Sulfolobus_acidocaldarius_DSM_639_uid58379	NC_007181	chromosome									
70606358	hypothetical protein Saci_0535	No	0		439307	439906	-		Sulfolobus_acidocaldarius_DSM_639_uid58379	NC_007181	chromosome		arCOG07719	1 (-)	R		Radical SAM superfamily enzyme			
70606359	hypothetical protein Saci_0538	No	1		440555	440959	-		Sulfolobus_acidocaldarius_DSM_639_uid58379	NC_007181	chromosome									
===								5	Sulfolobus_acidocaldarius_DSM_639_uid58379	NC_007181	chromosome									
70607684	hypothetical protein Saci_1958	Yes	2		1769620	1770117	+		Sulfolobus_acidocaldarius_DSM_639_uid58379	NC_007181	chromosome									
70607685	hypothetical protein Saci_1959	No	2		1770126	1770809	+		Sulfolobus_acidocaldarius_DSM_639_uid58379	NC_007181	chromosome									
70607686	membrane protein	No	6		1770815	1771582	+		Sulfolobus_acidocaldarius_DSM_639_uid58379	NC_007181	chromosome									
70607687	ABC transporter	No	0		1771579	1772433	+		Sulfolobus_acidocaldarius_DSM_639_uid58379	NC_007181	chromosome		arCOG00208	5 (-)	V	CcmA	ABC-type multidrug transport system, ATPase co	7E-35	COG1131	ABC-type multidrug transport system, ATP
70607688	hypothetical protein Saci_1962	No	6		1772405	1772992	+		Sulfolobus_acidocaldarius_DSM_639_uid58379	NC_007181	chromosome									
===								5	Sulfolobus_islandicus_L_D_8_5_uid43679	NC_013769	chromosome									
284996901	hypothetical protein LD85_0544	No	0		450909	451046	+		Sulfolobus_islandicus_L_D_8_5_uid43679	NC_013769	chromosome							9E-11	pfam04693	Archaeal putative transposase ISC1217.
284996902	ORF in transposon ISC1217	No	1		451226	451453	+		Sulfolobus_islandicus_L_D_8_5_uid43679	NC_013769	chromosome		arCOG09888	1	S		Uncharacterized conserved protein	1E-13	pfam04693	Archaeal putative transposase ISC1217.
284996903	glycerol kinase	No	0		452381	453886	-		Sulfolobus_islandicus_L_D_8_5_uid43679	NC_013769	chromosome		arCOG00024	1 (-)	C	GlpK	Glycerol kinase		COG0554	Glycerol kinase
284996904	hypothetical protein LD85_0550	No	0		455149	455262	+		Sulfolobus_islandicus_L_D_8_5_uid43679	NC_013769	chromosome									
284996905	hypothetical protein LD85_0551	No	0		455341	455478	+		Sulfolobus_islandicus_L_D_8_5_uid43679	NC_013769	chromosome									
===								8	Sulfolobus_islandicus_L_D_8_5_uid43679	NC_013769	chromosome									
284997160	PaREP1 domain containing protein	No	0	YES	713533	714000	-		Sulfolobus_islandicus_L_D_8_5_uid43679	NC_013769	chromosome		arCOG03721	3 (-)	V		HEPN domain containing protein	4E-17	pfam05942	Archaeal PaREP1/PaREP8 family. This fami
284997161	hypothetical protein LD85_0844	No	0	YES	714112	714375	-		Sulfolobus_islandicus_L_D_8_5_uid43679	NC_013769	chromosome									
284997162	hypothetical protein LD85_0845	No	0	YES	714874	715056	-		Sulfolobus_islandicus_L_D_8_5_uid43679	NC_013769	chromosome									
284997163	hypothetical protein LD85_0848	No	1	YES	715956	716921	+		Sulfolobus_islandicus_L_D_8_5_uid43679	NC_013769	chromosome		arCOG10353	2	S		Uncharacterized conserved protein			
284997164	hypothetical protein LD85_0849	No	0	YES	716937	717563	-		Sulfolobus_islandicus_L_D_8_5_uid43679	NC_013769	chromosome		arCOG10265	3	S		Uncharacterized conserved protein			
284997165	hypothetical protein LD85_0850	No	1	YES	718190	718309	+		Sulfolobus_islandicus_L_D_8_5_uid43679	NC_013769	chromosome									
284997166	hypothetical protein LD85_0851	No	1	YES	718353	718469	+		Sulfolobus_islandicus_L_D_8_5_uid43679	NC_013769	chromosome									
284997167	twitching mobility protein PilT	No	0	YES	718462	718875	-		Sulfolobus_islandicus_L_D_8_5_uid43679	NC_013769	chromosome		arCOG02221	2 (-)	V		PIN domain containing protein			
===								10	Sulfolobus_islandicus_L_D_8_5_uid43679	NC_013769	chromosome									
284997786	hypothetical protein LD85_1516	No	0		1328680	1329021	+		Sulfolobus_islandicus_L_D_8_5_uid43679	NC_013769	chromosome		arCOG09893	5	S		Uncharacterized conserved protein			
284997787	hypothetical protein LD85_1517	No	0		1329649	1330392	+		Sulfolobus_islandicus_L_D_8_5_uid43679	NC_013769	chromosome	clust_134								
284997788	hypothetical protein LD85_1518	No	0		1331213	1331767	-		Sulfolobus_islandicus_L_D_8_5_uid43679	NC_013769	chromosome		arCOG03423	3 (-)	K	MarR	Transcriptional regulator, MarR family			
284997789	hypothetical protein LD85_1519	No	0		1332039	1332644	+		Sulfolobus_islandicus_L_D_8_5_uid43679	NC_013769	chromosome	clust_168								
284997790	hypothetical protein LD85_1520	No	0		1332759	1333046	+		Sulfolobus_islandicus_L_D_8_5_uid43679	NC_013769	chromosome	clust_49								
284997791	hypothetical protein LD85_1521	No	3		1333172	1334287	+		Sulfolobus_islandicus_L_D_8_5_uid43679	NC_013769	chromosome		arCOG08469	4	S		Uncharacterized conserved protein			
284997792	hypothetical protein LD85_1522	No	0		1334291	1335016	+		Sulfolobus_islandicus_L_D_8_5_uid43679	NC_013769	chromosome		arCOG08470	4	S		Uncharacterized conserved protein			
284997793	stem cell self-renewal protein Piwi domain prot	No	0		1335013	1336395	-		Sulfolobus_islandicus_L_D_8_5_uid43679	NC_013769	chromosome		arCOG06164	4 (-)	V		Inactivated PIWI domain containing phage-defense protein			
284997794	hypothetical protein LD85_1524	No	0		1336392	1336667	-		Sulfolobus_islandicus_L_D_8_5_uid43679	NC_013769	chromosome									
284997795	hypothetical protein LD85_1525	No	0		1337929	1338300	+		Sulfolobus_islandicus_L_D_8_5_uid43679	NC_013769	chromosome									
===								7	Sulfolobus_islandicus_L_D_8_5_uid43679	NC_013769	chromosome									
284997815	hypothetical protein LD85_1545	No	0		1355517	1355654	+		Sulfolobus_islandicus_L_D_8_5_uid43679	NC_013769	chromosome									
284997816	hypothetical protein LD85_1546	No	10		1355967	1356929	+		Sulfolobus_islandicus_L_D_8_5_uid43679	NC_013769	chromosome		arCOG05344	1 (-)	G	ProP	Permease of the major facilitator superfamily			
284997817	hypothetical protein LD85_1547	No	0		1356970	1357452	+		Sulfolobus_islandicus_L_D_8_5_uid43679	NC_013769	chromosome		arCOG09893	5	S		Uncharacterized conserved protein			
284997818	hypothetical protein LD85_1548	No	0		1358080	1358823	+		Sulfolobus_islandicus_L_D_8_5_uid43679	NC_013769	chromosome	clust_134								
284997819	hypothetical protein LD85_1549	No	0		1359639	1360250	+		Sulfolobus_islandicus_L_D_8_5_uid43679	NC_013769	chromosome	clust_168								
284997820	hypothetical protein LD85_1550	No	0		1360365	1360652	+		Sulfolobus_islandicus_L_D_8_5_uid43679	NC_013769	chromosome	clust_49								
284997821	transposase, IS4 family protein	No	1		1360849	1361886	-		Sulfolobus_islandicus_L_D_8_5_uid43679	NC_013769	chromosome		arCOG03902	8 (-)	L		Transposase	8E-30	pfam14210	Domain of unknown function (DUF4322). Th
===								6	Sulfolobus_islandicus_L_D_8_5_uid43679	NC_013769	chromosome									
284998092	major facilitator superfamily MFS_1	No	10		1587330	1588484	-		Sulfolobus_islandicus_L_D_8_5_uid43679	NC_013769	chromosome		arCOG00130	5 (-)	G	ProP	Permease of the major facilitator superfamily	2E-13	pfam07690	Major Facilitator Superfamily.
284998093	hypothetical protein LD85_1832	No	0	YES	1588481	1589323	-		Sulfolobus_islandicus_L_D_8_5_uid43679	NC_013769	chromosome		arCOG10015	2	S		Uncharacterized conserved protein	3E-88	COG4278	Uncharacterized conserved protein
284998094	hypothetical protein LD85_1833	No	0	YES	1589362	1589601	-		Sulfolobus_islandicus_L_D_8_5_uid43679	NC_013769	chromosome		arCOG10016	2	S		Uncharacterized conserved protein			
284998095	hypothetical protein LD85_1834	No	0	YES	1590438	1590578	+		Sulfolobus_islandicus_L_D_8_5_uid43679	NC_013769	chromosome									
284998096	hypothetical protein LD85_1836	No	0	YES	1591113	1591262	+		Sulfolobus_islandicus_L_D_8_5_uid43679	NC_013769	chromosome		arCOG10002	1	S		Uncharacterized conserved protein			
284998097	hypothetical protein LD85_1837	No	0	YES	1591219	1591620	-		Sulfolobus_islandicus_L_D_8_5_uid43679	NC_013769	chromosome		arCOG01663	2 (-)	V	RelE	Cytotoxic translational repressor of toxin-antitoxin stability system			
===								7	Sulfolobus_islandicus_L_D_8_5_uid43679	NC_013769	chromosome									
284998100	hypothetical protein LD85_1840	No	0	YES	1592682	1592843	-		Sulfolobus_islandicus_L_D_8_5_uid43679	NC_013769	chromosome		arCOG09879	1	S		Uncharacterized conserved protein			
284998101	hypothetical protein LD85_1841	No	0	YES	1593348	1593461	+		Sulfolobus_islandicus_L_D_8_5_uid43679	NC_013769	chromosome		arCOG08540	1 (-)	S		Uncharacterized conserved protein			
284998102	hypothetical protein LD85_1842	No	4	YES	1593552	1593983	+		Sulfolobus_islandicus_L_D_8_5_uid43679	NC_013769	chromosome		arCOG10000	1	S		Uncharacterized conserved protein			
284998103	hypothetical protein LD85_1843	No	0	YES	1594707	1595123	-		Sulfolobus_islandicus_L_D_8_5_uid43679	NC_013769	chromosome									
284998104	hypothetical protein LD85_1844	No	0	YES	1595270	1595626	+		Sulfolobus_islandicus_L_D_8_5_uid43679	NC_013769	chromosome		arCOG10022	2	S		Uncharacterized conserved protein			
284998105	hypothetical protein LD85_1845	No	5	YES	1595851	1596750	+		Sulfolobus_islandicus_L_D_8_5_uid43679	NC_013769	chromosome		arCOG08326	2	S		Uncharacterized conserved protein			
284998106	hypothetical protein LD85_1846	No	0	YES	1596707	1597171	-		Sulfolobus_islandicus_L_D_8_5_uid43679	NC_013769	chromosome		arCOG00724	7 (-)	K		Predicted transcriptional regulator, PadR family			
===								5	Sulfolobus_islandicus_L_D_8_5_uid43679	NC_013769	chromosome									
284998418	hypothetical protein LD85_2166	No	0	YES	1875747	1875920	+		Sulfolobus_islandicus_L_D_8_5_uid43679	NC_013769	chromosome		arCOG09993	2	S		Uncharacterized conserved protein			
284998419	hypothetical protein LD85_2167	No	0	YES	1875913	1876095	+		Sulfolobus_islandicus_L_D_8_5_uid43679	NC_013769	chromosome		arCOG07847	2 (-)	S		Uncharacterized conserved protein			
284998420	CopG domain protein DNA-binding domain pro	No	0	YES	1876107	1876502	+		Sulfolobus_islandicus_L_D_8_5_uid43679	NC_013769	chromosome									
284998421	hypothetical protein LD85_2169	No	0	YES	1876477	1876683	+		Sulfolobus_islandicus_L_D_8_5_uid43679	NC_013769	chromosome									
284998422	hypothetical protein LD85_2172	No	0	YES	1878647	1878904	+		Sulfolobus_islandicus_L_D_8_5_uid43679	NC_013769	chromosome		arCOG08345	1	S		Uncharacterized conserved protein			
===								16	Sulfolobus_islandicus_L_S_2_15_uid58871	NC_012589	chromosome									
227829157	hypothetical protein LS215_0137	No	0	YES	120091	120918	-		Sulfolobus_islandicus_L_S_2_15_uid58871	NC_012589	chromosome		arCOG10009	1	S		Uncharacterized conserved protein	0.000003	COG0419	ATPase involved in DNA repair
227829158	hypothetical protein LS215_0138	No	0	YES	120970	121164	-		Sulfolobus_islandicus_L_S_2_15_uid58871	NC_012589	chromosome									
227829159	hypothetical protein LS215_0139	No	0	YES	121180	121890	-		Sulfolobus_islandicus_L_S_2_15_uid58871	NC_012589	chromosome		arCOG07960	16 (-)	R		ATPase (AAA+ superfamily)			
227829160	hypothetical protein LS215_0140	No	0	YES	121871	122323	-		Sulfolobus_islandicus_L_S_2_15_uid58871	NC_012589	chromosome		arCOG08564	3	Y		Sulfolobus virus associated uncharacterized protein			
227829161	hypothetical protein LS215_0141	No	0	YES	122461	122733	-		Sulfolobus_islandicus_L_S_2_15_uid58871	NC_012589	chromosome									
227829162	hypothetical protein LS215_0142	No	1	YES	122735	123574	-		Sulfolobus_islandicus_L_S_2_15_uid58871	NC_012589	chromosome		arCOG08565	3	Y		Sulfolobus virus associated uncharacterized protein			
227829163	phosphoribosylformylglycinamidine synthase	No	2	YES	123567	123851	-		Sulfolobus_islandicus_L_S_2_15_uid58871	NC_012589	chromosome		arCOG08566	3	Y		Virus associated protein			
227829164	hypothetical protein LS215_0144	No	0	YES	123616	123867	-		Sulfolobus_islandicus_L_S_2_15_uid58871	NC_012589	chromosome									
227829165	phosphoribosylformylglycinamidine synthase I	No	3	YES	123869	124117	-		Sulfolobus_islandicus_L_S_2_15_uid58871	NC_012589	chromosome		arCOG08567	3	Y		Virus associated protein			
227829166	VP1/VP3 family protein	No	2	YES	124158	124421	-		Sulfolobus_islandicus_L_S_2_15_uid58871	NC_012589	chromosome		arCOG08568	3	Y		Virus associated protein, V3 family	1E-24	pfam06281	Protein of unknown function (DUF1035). T
227829167	VP1/VP3 family protein	No	2	YES	124433	124738	-		Sulfolobus_islandicus_L_S_2_15_uid58871	NC_012589	chromosome		arCOG08569	2	Y		Virus associated protein, V1 family	6E-12	pfam06281	Protein of unknown function (DUF1035). T
227829168	phosphoribosylaminoimidazolesuccinocarbox	No	1	YES	124722	125033	-		Sulfolobus_islandicus_L_S_2_15_uid58871	NC_012589	chromosome		arCOG09949	2	S		Uncharacterized conserved protein	0.0000002	COG0640	Predicted transcriptional regulators
227829169	hypothetical protein LS215_0149	Yes	4	YES	125030	125506	-		Sulfolobus_islandicus_L_S_2_15_uid58871	NC_012589	chromosome		arCOG08570	3	Y		Virus associated protein			
227829170	hypothetical protein LS215_0150	No	2	YES	125487	125732	-		Sulfolobus_islandicus_L_S_2_15_uid58871	NC_012589	chromosome		arCOG08571	3	Y		Sulfolobus virus associated uncharacterized membrane protein			
227829171	hypothetical protein LS215_0151	Yes	3	YES	125729	128152	-		Sulfolobus_islandicus_L_S_2_15_uid58871	NC_012589	chromosome		arCOG07848	3 (-)	S		Uncharacterized conserved protein			
227829172	hypothetical protein LS215_0152	No	1	YES	128193	128417	-		Sulfolobus_islandicus_L_S_2_15_uid58871	NC_012589	chromosome									
===								9	Sulfolobus_islandicus_L_S_2_15_uid58871	NC_012589	chromosome									
227829179	CopG domain protein DNA-binding domain pro	No	0	YES	131258	131413	-		Sulfolobus_islandicus_L_S_2_15_uid58871	NC_012589	chromosome		arCOG03845	3 (-)	V		Predicted antitoxin, copG family			
227829180	orotidine 5'-phosphate decarboxylase (OMP d	No	0	YES	131425	131742	-		Sulfolobus_islandicus_L_S_2_15_uid58871	NC_012589	chromosome		arCOG09950	2	S		Uncharacterized conserved protein			
227829181	hypothetical protein LS215_0161	No	0		131842	132288	+		Sulfolobus_islandicus_L_S_2_15_uid58871	NC_012589	chromosome									
227829182	hypothetical protein LS215_0162	No	0		132296	132532	+		Sulfolobus_islandicus_L_S_2_15_uid58871	NC_012589	chromosome									
227829183	site-specific integrase-resolvase-like protein	Yes	0		132456	132719	-		Sulfolobus_islandicus_L_S_2_15_uid58871	NC_012589	chromosome									
227829184	hypothetical protein LS215_0163	No	0		132529	132933	+		Sulfolobus_islandicus_L_S_2_15_uid58871	NC_012589	chromosome		arCOG09999	1	S		Uncharacterized conserved protein	0.0001	pfam07133	Merozoite surface protein (SPAM). This f
227829185	chromosome partition protein	No	0		133135	133566	+		Sulfolobus_islandicus_L_S_2_15_uid58871	NC_012589	chromosome									
227829186	hypothetical protein LS215_0165	No	0		133568	133756	+		Sulfolobus_islandicus_L_S_2_15_uid58871	NC_012589	chromosome		arCOG09951	2	S		Uncharacterized conserved protein			
227829187	hypothetical protein LS215_0166	No	0		133753	134331	+		Sulfolobus_islandicus_L_S_2_15_uid58871	NC_012589	chromosome		arCOG08574	2 (-)	L		Viral/Archaeal nuclease			
===								5	Sulfolobus_islandicus_L_S_2_15_uid58871	NC_012589	chromosome									
227830837	hypothetical protein LS215_1977	No	1	YES	1803941	1804852	+		Sulfolobus_islandicus_L_S_2_15_uid58871	NC_012589	chromosome		arCOG10017	2	S		Uncharacterized conserved protein			
227830838	hypothetical protein LS215_1978	No	0	YES	1805282	1805647	+		Sulfolobus_islandicus_L_S_2_15_uid58871	NC_012589	chromosome		arCOG10018	2	S		Uncharacterized conserved protein			
227830839	transcription factor	No	0	YES	1805672	1805836	-		Sulfolobus_islandicus_L_S_2_15_uid58871	NC_012589	chromosome		arCOG10019	2	S		Uncharacterized conserved protein			
227830840	CRISPR-associated protein Cas4	No	0	YES	1806365	1807018	+		Sulfolobus_islandicus_L_S_2_15_uid58871	NC_012589	chromosome	defense	arCOG00790	6 (-)	V	Cas4	CRISPR-associated protein Cas4, RecB family ex	0.00000004	COG1468	CRISPR-associated protein Cas4 (RecB fam
227830841	hypothetical protein LS215_1981	No	0	YES	1806996	1807388	+		Sulfolobus_islandicus_L_S_2_15_uid58871	NC_012589	chromosome		arCOG10020	2	S		Uncharacterized conserved protein			
===								5	Sulfolobus_islandicus_M_14_25_uid58849	NC_012588	chromosome									
227826874	hypothetical protein M1425_2806	No	0		442033	442308	+		Sulfolobus_islandicus_M_14_25_uid58849	NC_012588	chromosome									
227826875	hypothetical protein M1425_0505	No	0		442451	442591	-		Sulfolobus_islandicus_M_14_25_uid58849	NC_012588	chromosome									
227826876	hypothetical protein M1425_0506	No	0		442665	448661	-		Sulfolobus_islandicus_M_14_25_uid58849	NC_012588	chromosome		arCOG08433	1	S		Uncharacterized conserved protein	0.0000007	COG0419	ATPase involved in DNA repair
227826877	hypothetical protein M1425_0507	No	0		449048	449227	-		Sulfolobus_islandicus_M_14_25_uid58849	NC_012588	chromosome		arCOG07185	2 (-)	S		Uncharacterized conserved protein			
227826878	hypothetical protein M1425_0508	No	0		449638	449913	+		Sulfolobus_islandicus_M_14_25_uid58849	NC_012588	chromosome		arCOG09874	1	S		Uncharacterized conserved protein			
===								5	Sulfolobus_islandicus_M_14_25_uid58849	NC_012588	chromosome									
227827117	hypothetical protein M1425_0788	No	0		735008	735376	+		Sulfolobus_islandicus_M_14_25_uid58849	NC_012588	chromosome		arCOG09977	2	S		Uncharacterized conserved protein			
227827118	hypothetical protein M1425_0789	No	0		735464	736522	-		Sulfolobus_islandicus_M_14_25_uid58849	NC_012588	chromosome		arCOG03035	1 (-)	R	NrfG	TPR repeats containing protein			
227827119	hypothetical protein M1425_0791	No	12		737493	738521	-		Sulfolobus_islandicus_M_14_25_uid58849	NC_012588	chromosome		arCOG07253	3	S		Uncharacterized conserved protein	0.00007	COG4906	Predicted membrane protein
227827120	hypothetical protein M1425_0792	No	0		738933	740615	+		Sulfolobus_islandicus_M_14_25_uid58849	NC_012588	chromosome		arCOG08580	3	E		Uncharacterized conserved protein			
227827121	hypothetical protein M1425_0793	No	0		740625	741167	+		Sulfolobus_islandicus_M_14_25_uid58849	NC_012588	chromosome		arCOG08351	3	S		Uncharacterized conserved protein, YdcF family	5E-12	pfam02698	DUF218 domain. This large family of prot
===								12	Sulfolobus_islandicus_M_14_25_uid58849	NC_012588	chromosome									
227828077	hypothetical protein M1425_1814	No	0	YES	1680031	1680768	-		Sulfolobus_islandicus_M_14_25_uid58849	NC_012588	chromosome		arCOG07960	16 (-)	R		ATPase (AAA+ superfamily)			
227828078	hypothetical protein M1425_1815	No	0	YES	1680749	1681207	-		Sulfolobus_islandicus_M_14_25_uid58849	NC_012588	chromosome		arCOG08564	3	Y		Sulfolobus virus associated uncharacterized protein			
227828079	hypothetical protein M1425_1816	No	1	YES	1681214	1682053	-		Sulfolobus_islandicus_M_14_25_uid58849	NC_012588	chromosome		arCOG08565	3	Y		Sulfolobus virus associated uncharacterized protein			
227828080	hypothetical protein M1425_1817	No	3	YES	1682046	1682324	-		Sulfolobus_islandicus_M_14_25_uid58849	NC_012588	chromosome		arCOG08566	3	Y		Virus associated protein			
227828081	hypothetical protein M1425_1818	No	3	YES	1682343	1682591	-		Sulfolobus_islandicus_M_14_25_uid58849	NC_012588	chromosome		arCOG08567	3	Y		Virus associated protein			
227828082	VP1/VP3 family protein	No	2	YES	1682633	1682908	-		Sulfolobus_islandicus_M_14_25_uid58849	NC_012588	chromosome		arCOG08568	3	Y		Virus associated protein, V3 family	6E-38	pfam06281	Protein of unknown function (DUF1035). T
227828083	hypothetical protein M1425_1821	No	0	YES	1683131	1683508	-		Sulfolobus_islandicus_M_14_25_uid58849	NC_012588	chromosome		arCOG02611	4 (-)	K		Predicted transcriptional regulator, contaIns two H	0.0000002	COG0640	Predicted transcriptional regulators
227828084	hypothetical protein M1425_1822	Yes	4	YES	1683472	1683972	-		Sulfolobus_islandicus_M_14_25_uid58849	NC_012588	chromosome		arCOG08570	3	Y		Virus associated protein			
227828085	hypothetical protein M1425_1823	No	2	YES	1683962	1684204	-		Sulfolobus_islandicus_M_14_25_uid58849	NC_012588	chromosome		arCOG08571	3	Y		Sulfolobus virus associated uncharacterized membrane protein			
227828086	hypothetical protein M1425_1824	Yes	4	YES	1684204	1686618	-		Sulfolobus_islandicus_M_14_25_uid58849	NC_012588	chromosome		arCOG07848	3 (-)	S		Uncharacterized conserved protein			
227828087	hypothetical protein M1425_1825	Yes	1	YES	1686912	1687820	-		Sulfolobus_islandicus_M_14_25_uid58849	NC_012588	chromosome		arCOG08407	1	S		Uncharacterized conserved protein			
227828088	hypothetical protein M1425_2827	Yes	0	YES	1687727	1687840	+		Sulfolobus_islandicus_M_14_25_uid58849	NC_012588	chromosome									
===								12	Sulfolobus_islandicus_M_14_25_uid58849	NC_012588	chromosome									
227828094	CopG family transcripitonal regulator	No	0	YES	1689493	1689738	-		Sulfolobus_islandicus_M_14_25_uid58849	NC_012588	chromosome		arCOG03845	3 (-)	V		Predicted antitoxin, copG family			
227828095	hypothetical protein M1425_1832	No	0	YES	1689751	1690041	-		Sulfolobus_islandicus_M_14_25_uid58849	NC_012588	chromosome									
227828096	hypothetical protein M1425_1833	No	0	YES	1690165	1690386	+		Sulfolobus_islandicus_M_14_25_uid58849	NC_012588	chromosome									
227828097	hypothetical protein M1425_1834	No	1	YES	1690349	1690570	+		Sulfolobus_islandicus_M_14_25_uid58849	NC_012588	chromosome									
227828098	hypothetical protein M1425_1835	No	0	YES	1690617	1690802	+		Sulfolobus_islandicus_M_14_25_uid58849	NC_012588	chromosome									
227828099	regulatory protein MarR	No	0		1690799	1691254	+		Sulfolobus_islandicus_M_14_25_uid58849	NC_012588	chromosome		arCOG03423	3 (-)	K	MarR	Transcriptional regulator, MarR family	0.0000009	pfam07133	Merozoite surface protein (SPAM). This f ; Transcriptional regulators of sugar meta
227828100	hypothetical protein M1425_1837	No	1		1691264	1691617	+		Sulfolobus_islandicus_M_14_25_uid58849	NC_012588	chromosome									
227828101	hypothetical protein M1425_1838	No	0		1691610	1692086	+		Sulfolobus_islandicus_M_14_25_uid58849	NC_012588	chromosome									
227828102	hypothetical protein M1425_1839	No	0		1692088	1692273	+		Sulfolobus_islandicus_M_14_25_uid58849	NC_012588	chromosome		arCOG08586	1	Y		Virus associated protein, contains HTH domain			
227828103	hypothetical protein M1425_1840	No	0		1692279	1692737	+		Sulfolobus_islandicus_M_14_25_uid58849	NC_012588	chromosome							6E-13	pfam08960	Domain of unknown function (DUF1874). Th
227828104	hypothetical protein M1425_1841	No	0		1692954	1693589	+		Sulfolobus_islandicus_M_14_25_uid58849	NC_012588	chromosome		arCOG10008	1	Y		Sulfolobus virus associated uncharacterized prot	7E-116	pfam12187	Viral/Archaeal nuclease. This family of
227828105	ORF D-335 domain protein	No	0		1693912	1694217	+		Sulfolobus_islandicus_M_14_25_uid58849	NC_012588	chromosome		arCOG07277	1 (-)	Y		SSV1_ORF_D-335, predicted GIY-YIG nuclease s	1E-25	pfam07935	ORF D-335-like protein. The sequences fe
===								5	Sulfolobus_islandicus_M_16_27_uid58851	NC_012632	chromosome									
229584285	hypothetical protein M1627_0793	No	0		754214	754582	+		Sulfolobus_islandicus_M_16_27_uid58851	NC_012632	chromosome		arCOG09977	2	S		Uncharacterized conserved protein			
229584286	hypothetical protein M1627_0794	No	0		754670	755728	-		Sulfolobus_islandicus_M_16_27_uid58851	NC_012632	chromosome		arCOG03032	12 (-)	R	NrfG	TPR repeats containing protein			
229584287	hypothetical protein M1627_0796	No	12		756699	757727	-		Sulfolobus_islandicus_M_16_27_uid58851	NC_012632	chromosome		arCOG07253	3	S		Uncharacterized conserved protein	0.00007	COG4906	Predicted membrane protein
229584288	hypothetical protein M1627_0797	No	0		758139	759821	+		Sulfolobus_islandicus_M_16_27_uid58851	NC_012632	chromosome		arCOG08580	3	E		Uncharacterized conserved protein			
229584289	hypothetical protein M1627_0798	No	0		759831	760373	+		Sulfolobus_islandicus_M_16_27_uid58851	NC_012632	chromosome		arCOG08351	3	S		Uncharacterized conserved protein, YdcF family	5E-12	pfam02698	DUF218 domain. This large family of prot
===								5	Sulfolobus_islandicus_M_16_27_uid58851	NC_012632	chromosome									
229584933	hypothetical protein M1627_1508	No	0		1440052	1440351	-		Sulfolobus_islandicus_M_16_27_uid58851	NC_012632	chromosome		arCOG09990	2	S		Uncharacterized conserved protein			
229584934	hypothetical protein M1627_1509	No	0		1440338	1440829	-		Sulfolobus_islandicus_M_16_27_uid58851	NC_012632	chromosome		arCOG08465	3 (-)	S		Uncharacterized conserved protein			
229584935	hypothetical protein M1627_1510	No	0		1440817	1441077	-		Sulfolobus_islandicus_M_16_27_uid58851	NC_012632	chromosome		arCOG09892	2	S		Uncharacterized conserved protein			
229584936	hypothetical protein M1627_1511	No	0		1441074	1441466	-		Sulfolobus_islandicus_M_16_27_uid58851	NC_012632	chromosome									
229584937	hypothetical protein M1627_1512	No	0		1441564	1441761	+		Sulfolobus_islandicus_M_16_27_uid58851	NC_012632	chromosome		arCOG09991	1	S		Uncharacterized conserved protein			
===								10	Sulfolobus_islandicus_M_16_27_uid58851	NC_012632	chromosome									
229584944	hypothetical protein M1627_1519	No	0		1444659	1445000	+		Sulfolobus_islandicus_M_16_27_uid58851	NC_012632	chromosome		arCOG09893	5	S		Uncharacterized conserved protein			
229584945	hypothetical protein M1627_1520	No	0		1444997	1445629	+		Sulfolobus_islandicus_M_16_27_uid58851	NC_012632	chromosome		arCOG08466	4 (-)	S		Uncharacterized conserved protein			found in viruses and conj plasmids
229584946	hypothetical protein M1627_1521	No	0		1445640	1446305	+		Sulfolobus_islandicus_M_16_27_uid58851	NC_012632	chromosome		arCOG08585	1	S		Uncharacterized conserved protein			
229584947	hypothetical protein M1627_1522	No	0		1447032	1447367	+		Sulfolobus_islandicus_M_16_27_uid58851	NC_012632	chromosome									
229584948	hypothetical protein M1627_1523	No	0		1447535	1448104	+		Sulfolobus_islandicus_M_16_27_uid58851	NC_012632	chromosome		arCOG09992	2	S		Uncharacterized conserved protein			
229584949	hypothetical protein M1627_1524	No	0		1448390	1449457	-		Sulfolobus_islandicus_M_16_27_uid58851	NC_012632	chromosome									
229584950	hypothetical protein M1627_2859	No	0		1449734	1449859	-		Sulfolobus_islandicus_M_16_27_uid58851	NC_012632	chromosome		arCOG08468	3	S		Uncharacterized conserved protein			
229584951	hypothetical protein M1627_1525	No	0		1449837	1450004	-		Sulfolobus_islandicus_M_16_27_uid58851	NC_012632	chromosome		arCOG00115	10 (-)	L		DNA modification methylase	0.000003	COG0863	DNA modification methylase
229584952	hypothetical protein M1627_1526	No	2		1450182	1451294	+		Sulfolobus_islandicus_M_16_27_uid58851	NC_012632	chromosome		arCOG08469	4	S		Uncharacterized conserved protein			
229584953	hypothetical protein M1627_1527	No	0		1451298	1452029	+		Sulfolobus_islandicus_M_16_27_uid58851	NC_012632	chromosome		arCOG08470	4	S		Uncharacterized conserved protein			
===								7	Sulfolobus_islandicus_M_16_27_uid58851	NC_012632	chromosome									
229584972	hypothetical protein M1627_1545	No	5		1465902	1466756	-		Sulfolobus_islandicus_M_16_27_uid58851	NC_012632	chromosome		arCOG08326	2	S		Uncharacterized conserved protein			
229584973	hypothetical protein M1627_1546	No	0		1466988	1467272	-		Sulfolobus_islandicus_M_16_27_uid58851	NC_012632	chromosome		arCOG09898	1 (-)	K		Transcriptional regulator, contains wHTH domain			
229584974	hypothetical protein M1627_1547	No	0		1467335	1467607	+		Sulfolobus_islandicus_M_16_27_uid58851	NC_012632	chromosome		arCOG09897	1	S		Uncharacterized conserved protein			
229584975	hypothetical protein M1627_1548	No	0		1467708	1467815	+		Sulfolobus_islandicus_M_16_27_uid58851	NC_012632	chromosome		arCOG11081	1	S		Uncharacterized conserved protein			
229584976	hypothetical protein M1627_1549	No	1		1467860	1468144	-		Sulfolobus_islandicus_M_16_27_uid58851	NC_012632	chromosome									
229584977	hypothetical protein M1627_1550	Yes	2		1468177	1468710	-		Sulfolobus_islandicus_M_16_27_uid58851	NC_012632	chromosome									
229584978	hypothetical protein M1627_1551	Yes	9		1468707	1470692	-		Sulfolobus_islandicus_M_16_27_uid58851	NC_012632	chromosome		arCOG07844	1 (-)	S		PKD domain containing protein			
===								10	Sulfolobus_islandicus_M_16_4_uid58841	NC_012726	chromosome									
238618617	hypothetical protein M164_0140	No	0		121347	121586	+		Sulfolobus_islandicus_M_16_4_uid58841	NC_012726	chromosome		arCOG08459	2 (-)	S		Uncharacterized conserved protein			
238618618	hypothetical protein M164_0141	No	0		121596	121979	+		Sulfolobus_islandicus_M_16_4_uid58841	NC_012726	chromosome		arCOG09855	2	S		Uncharacterized conserved protein			
238618619	hypothetical protein M164_0142	No	0		121966	122262	+		Sulfolobus_islandicus_M_16_4_uid58841	NC_012726	chromosome		arCOG09856	2	S		Uncharacterized conserved protein			
238618620	hypothetical protein M164_0143	No	0		122248	122421	-		Sulfolobus_islandicus_M_16_4_uid58841	NC_012726	chromosome									
238618621	hypothetical protein M164_0144	No	0		122418	126488	-		Sulfolobus_islandicus_M_16_4_uid58841	NC_012726	chromosome		arCOG09857	2	S		Uncharacterized conserved protein	1E-22	pfam06346	Formin Homology Region 1. This region is
238618622	hypothetical protein M164_0145	No	0		126485	126712	-		Sulfolobus_islandicus_M_16_4_uid58841	NC_012726	chromosome		arCOG09858	2	S		Uncharacterized conserved protein			
238618623	hypothetical protein M164_0146	No	0		126751	127092	+		Sulfolobus_islandicus_M_16_4_uid58841	NC_012726	chromosome		arCOG08376	2	S		Uncharacterized conserved protein			
238618624	hypothetical protein M164_0147	No	0		127132	127446	+		Sulfolobus_islandicus_M_16_4_uid58841	NC_012726	chromosome									
238618625	hypothetical protein M164_0148	No	0		127443	127850	+		Sulfolobus_islandicus_M_16_4_uid58841	NC_012726	chromosome									
238618626	hypothetical protein M164_0149	No	0		127991	128164	-		Sulfolobus_islandicus_M_16_4_uid58841	NC_012726	chromosome		arCOG07185	2 (-)	S		Uncharacterized conserved protein			
===								16	Sulfolobus_islandicus_M_16_4_uid58841	NC_012726	chromosome									
238619348	hypothetical protein M164_0893	No	1	YES	846611	847072	-		Sulfolobus_islandicus_M_16_4_uid58841	NC_012726	chromosome		arCOG01446	5 (-)	VK	Csa3	CRISPR-Cas assicated transcriptional regulator, contains CARF and HTH domain			
238619349	integrase family protein	No	0	YES	847379	848689	-		Sulfolobus_islandicus_M_16_4_uid58841	NC_012726	chromosome									
238619350	hypothetical protein M164_0895	No	1	YES	848832	849200	-		Sulfolobus_islandicus_M_16_4_uid58841	NC_012726	chromosome									
238619351	hypothetical protein M164_0896	No	0	YES	849197	849655	-		Sulfolobus_islandicus_M_16_4_uid58841	NC_012726	chromosome									
238619352	hypothetical protein M164_0897	No	0	YES	850163	850438	+		Sulfolobus_islandicus_M_16_4_uid58841	NC_012726	chromosome									
238619353	hypothetical protein M164_0898	No	0	YES	850451	850732	+		Sulfolobus_islandicus_M_16_4_uid58841	NC_012726	chromosome									
238619354	hypothetical protein M164_0899	No	0	YES	850869	851600	+		Sulfolobus_islandicus_M_16_4_uid58841	NC_012726	chromosome									
238619355	hypothetical protein M164_0900	No	0	YES	851575	852246	-		Sulfolobus_islandicus_M_16_4_uid58841	NC_012726	chromosome									
238619356	hypothetical protein M164_0901	No	0	YES	852405	853124	-		Sulfolobus_islandicus_M_16_4_uid58841	NC_012726	chromosome									
238619357	hypothetical protein M164_0902	No	0	YES	853820	854167	+		Sulfolobus_islandicus_M_16_4_uid58841	NC_012726	chromosome									
238619358	hypothetical protein M164_0903	No	0		854188	857715	+		Sulfolobus_islandicus_M_16_4_uid58841	NC_012726	chromosome		arCOG09873	3	Y		RepA plasmid replication protein	0.00009	COG3587	Restriction endonuclease
238619359	hypothetical protein M164_0904	No	1		857821	859401	+		Sulfolobus_islandicus_M_16_4_uid58841	NC_012726	chromosome							0.00001	COG1196	Chromosome segregation ATPases

	238619360	hypothetical protein M164_0905	No	0			859453	860508 +		Sulfolobus_islandicus_M_16_4_uid58841	NC_012726	chromosome										
	238619361	hypothetical protein M164_0906	No	0			860521	861039 -		Sulfolobus_islandicus_M_16_4_uid58841	NC_012726	chromosome										
	238619362	hypothetical protein M164_0907	No	0	YES		861065	861232 -		Sulfolobus_islandicus_M_16_4_uid58841	NC_012726	chromosome										
	238619363	CRISPR locus-related DNA-binding protein	No	0	YES		865648	866259 +		Sulfolobus_islandicus_M_16_4_uid58841	NC_012726	chromosome			arCOG01446	5 (-)	VK	Csa3	CRISPR-Cas assicated transcriptional regulator, contains CARF and HTH domain			
===									6	Sulfolobus_islandicus_M_16_4_uid58841	NC_012726	chromosome										
	238619436	transposase, ISC1234/ST1916	No	0			928371	929309 +		Sulfolobus_islandicus_M_16_4_uid58841	NC_012726	chromosome		arCOG07925,a	arCOG07925	2 (-)	L		Transposase ISC1234			
	238619437	glycosyltransferase,	No	1			929505	929705 -		Sulfolobus_islandicus_M_16_4_uid58841	NC_012726	chromosome										
	238619438	glycosyltransferase,	No	0			929745	929882 -		Sulfolobus_islandicus_M_16_4_uid58841	NC_012726	chromosome										
	238619439	hypothetical protein M164_0989	No	13			930017	932500 +		Sulfolobus_islandicus_M_16_4_uid58841	NC_012726	chromosome			arCOG07850	2	S		Uncharacterized conserved protein			
	238619440	glycosyltransferase	No	0			932475	932651 +		Sulfolobus_islandicus_M_16_4_uid58841	NC_012726	chromosome										
	238619441	glycosyl transferase group 1	No	0			932600	933667 +		Sulfolobus_islandicus_M_16_4_uid58841	NC_012726	chromosome			arCOG01417	2 (-)	M	RfaG	Glycosyltransferase	4E-12	COG0438	Glycosyltransferase
===									6	Sulfolobus_islandicus_M_16_4_uid58841	NC_012726	chromosome										
	238620040	CopG DNA-binding domain protein	No	0			1476320	1476508 -		Sulfolobus_islandicus_M_16_4_uid58841	NC_012726	chromosome								0.000005	pfam01402	Ribbon-helix-helix protein, copG family.
	238620041	hypothetical protein M164_1599	No	0			1476508	1476717 -		Sulfolobus_islandicus_M_16_4_uid58841	NC_012726	chromosome			arCOG08464	2	S		Uncharacterized conserved protein			
	238620042	hypothetical protein M164_1600	No	0			1476710	1477216 -		Sulfolobus_islandicus_M_16_4_uid58841	NC_012726	chromosome			arCOG08465	3 (-)	S		Uncharacterized conserved protein			
	238620043	hypothetical protein M164_1601	No	0			1477204	1477464 -		Sulfolobus_islandicus_M_16_4_uid58841	NC_012726	chromosome			arCOG09892	2	S		Uncharacterized conserved protein			
	238620044	hypothetical protein M164_1602	No	0			1477461	1477688 -		Sulfolobus_islandicus_M_16_4_uid58841	NC_012726	chromosome										
	238620045	hypothetical protein M164_1603	No	0			1477685	1477864 -		Sulfolobus_islandicus_M_16_4_uid58841	NC_012726	chromosome										
===									8	Sulfolobus_islandicus_M_16_4_uid58841	NC_012726	chromosome										
	238620049	hypothetical protein M164_1607	No	0			1479121	1479441 +		Sulfolobus_islandicus_M_16_4_uid58841	NC_012726	chromosome			arCOG09893	5	S		Uncharacterized conserved protein			
	238620050	hypothetical protein M164_1608	No	0			1479455	1480078 +		Sulfolobus_islandicus_M_16_4_uid58841	NC_012726	chromosome			arCOG08466	4 (-)	S		Uncharacterized conserved protein			
	238620051	hypothetical protein M164_1609	No	0			1480091	1482109 +		Sulfolobus_islandicus_M_16_4_uid58841	NC_012726	chromosome			arCOG09873	3	Y		RepA plasmid replication protein			
	238620052	hypothetical protein M164_1610	No	0			1482660	1482947 +		Sulfolobus_islandicus_M_16_4_uid58841	NC_012726	chromosome	clust_49									
	238620053	hypothetical protein M164_2815	No	0			1483094	1483219 -		Sulfolobus_islandicus_M_16_4_uid58841	NC_012726	chromosome			arCOG08468	3	S		Uncharacterized conserved protein			
	238620054	hypothetical protein M164_1611	No	0			1483197	1483364 -		Sulfolobus_islandicus_M_16_4_uid58841	NC_012726	chromosome			arCOG00115	10 (-)	L		DNA modification methylase	0.000003	COG0863	DNA modification methylase
	238620055	hypothetical protein M164_1612	No	2			1483542	1484654 +		Sulfolobus_islandicus_M_16_4_uid58841	NC_012726	chromosome			arCOG08469	4	S		Uncharacterized conserved protein			
	238620056	hypothetical protein M164_1613	No	0			1484658	1485389 +		Sulfolobus_islandicus_M_16_4_uid58841	NC_012726	chromosome			arCOG08470	4	S		Uncharacterized conserved protein			
===									8	Sulfolobus_islandicus_REY15A_uid162071	NC_017276	chromosome										
	385775542	hypothetical protein	No	0			786784	787296 -		Sulfolobus_islandicus_REY15A_uid162071	NC_017276	chromosome			arCOG08547	3	S		Uncharacterized conserved protein			
	385775543	hypothetical protein	No	0			787299	787898 -		Sulfolobus_islandicus_REY15A_uid162071	NC_017276	chromosome			arCOG08548	3	S		Uncharacterized conserved protein			
	385775544	nitroreductase family	No	0			790437	791132 -		Sulfolobus_islandicus_REY15A_uid162071	NC_017276	chromosome			arCOG00288	8 (-)	C	NfnB	Nitroreductase	1E-13	pfam00881	Nitroreductase family. The nitroreductas
	385775545	hypothetical protein	No	0			791142	792440 -		Sulfolobus_islandicus_REY15A_uid162071	NC_017276	chromosome			arCOG02883	3	S		Uncharacterized conserved protein	2E-19	pfam02624	YcaO-like family.
	385775546	hypothetical protein	No	0			792428	792970 -		Sulfolobus_islandicus_REY15A_uid162071	NC_017276	chromosome										
	385775547	hypothetical protein	No	0			793103	793210 -		Sulfolobus_islandicus_REY15A_uid162071	NC_017276	chromosome										
	385775548	hypothetical protein	No	6			793323	793973 +		Sulfolobus_islandicus_REY15A_uid162071	NC_017276	chromosome								0.00004	pfam13346	ABC-2 family transporter protein. This f
	385775549	hypothetical protein	No	6			794188	794925 +		Sulfolobus_islandicus_REY15A_uid162071	NC_017276	chromosome			arCOG05371	4 (-)	S		Uncharacterized conserved protein			
===									6	Sulfolobus_islandicus_Y_G_57_14_uid58923	NC_012622	chromosome										
	229578370	transposase ISC1190	No	0	YES		510050	510235 -		Sulfolobus_islandicus_Y_G_57_14_uid58923	NC_012622	chromosome			arCOG03586	3 (-)	L		Transposase	0.000004	COG3547	Transposase and inactivated derivatives
	229578371	hypothetical protein YG5714_3041	No	0	YES		510252	510353 -		Sulfolobus_islandicus_Y_G_57_14_uid58923	NC_012622	chromosome										
	229578372	hypothetical protein YG5714_0553	No	0	YES		510519	510650 -		Sulfolobus_islandicus_Y_G_57_14_uid58923	NC_012622	chromosome			arCOG09919	3	S		Uncharacterized conserved protein			
	229578373	hypothetical protein YG5714_0554	No	0	YES		510878	511387 +		Sulfolobus_islandicus_Y_G_57_14_uid58923	NC_012622	chromosome			arCOG09920	3	S		Uncharacterized conserved protein			
	229578374	hypothetical protein YG5714_0555	No	0	YES		511413	511940 +		Sulfolobus_islandicus_Y_G_57_14_uid58923	NC_012622	chromosome			arCOG09921	3	S		Uncharacterized conserved protein			
	229578375	hypothetical protein YG5714_0556	No	0	YES		512053	513339 -		Sulfolobus_islandicus_Y_G_57_14_uid58923	NC_012622	chromosome			arCOG04708	2 (-)	V		ATP-dependent helicase fused to N-terminal NEF	2E-25	COG3410	Uncharacterized conserved protein
===									5	Sulfolobus_islandicus_Y_G_57_14_uid58923	NC_012622	chromosome										
	229578422	hypothetical protein YG5714_3040	No	0	YES		566218	566310 -		Sulfolobus_islandicus_Y_G_57_14_uid58923	NC_012622	chromosome										
	229578423	serine/threonine protein kinase	No	0	YES		566648	568078 -		Sulfolobus_islandicus_Y_G_57_14_uid58923	NC_012622	chromosome			arCOG03682	2 (-)	R	SPS1	Membrane associated serine/threonine protein ki	2E-13	pfam00069	Protein kinase domain.
	229578424	hypothetical protein YG5714_0603	No	0	YES		568165	569400 +		Sulfolobus_islandicus_Y_G_57_14_uid58923	NC_012622	chromosome			arCOG09923	2	S		Uncharacterized conserved protein			
	229578425	hypothetical protein YG5714_0604	No	0	YES		569939	570244 -		Sulfolobus_islandicus_Y_G_57_14_uid58923	NC_012622	chromosome										
	229578426	hypothetical protein YG5714_0605	No	4	YES		570765	571577 +		Sulfolobus_islandicus_Y_G_57_14_uid58923	NC_012622	chromosome								0.00005	pfam10318	Serpentine type 7TM GPCR chemoreceptor S
===									7	Sulfolobus_islandicus_Y_G_57_14_uid58923	NC_012622	chromosome										
	229578448	ORF1 in transposon ISC1439	No	0			599658	599924 -		Sulfolobus_islandicus_Y_G_57_14_uid58923	NC_012622	chromosome			arCOG09924	1	S		Uncharacterized conserved protein			
	229578449	glycosyl transferase family 2	No	0			600226	600993 +		Sulfolobus_islandicus_Y_G_57_14_uid58923	NC_012622	chromosome			arCOG01385	2 (-)	M	WcaA	Glycosyltransferase	4E-11	pfam00535	Glycosyl transferase family 2. Diverse f
	229578450	hypothetical protein YG5714_0633	No	0	YES		602042	602353 -		Sulfolobus_islandicus_Y_G_57_14_uid58923	NC_012622	chromosome			arCOG08544	3	V		HEPN domain containing protein, paRep1 subfamily			
	229578451	hypothetical protein YG5714_0634	No	0	YES		602359	602463 -		Sulfolobus_islandicus_Y_G_57_14_uid58923	NC_012622	chromosome			arCOG08544	3	V		HEPN domain containing protein, paRep1 subfamily			
	229578452	conserved hypothetical rRNA adenine N-6-met	No	0	YES		602505	602759 +		Sulfolobus_islandicus_Y_G_57_14_uid58923	NC_012622	chromosome			arCOG08545	2	S		Uncharacterized conserved protein			
	229578453	hypothetical protein YG5714_0636	No	0	YES		602887	603855 +		Sulfolobus_islandicus_Y_G_57_14_uid58923	NC_012622	chromosome			arCOG02658	2 (-)	V	Cas7/Csm	CRISPR system related protein, RAMP superfam	0.00001	COG1337	CRISPR system related protein, RAMP supe
	229578454	hypothetical protein YG5714_0637	No	0	YES		603839	604252 +		Sulfolobus_islandicus_Y_G_57_14_uid58923	NC_012622	chromosome			arCOG08546	1	S		Uncharacterized conserved protein			
===									7	Sulfolobus_islandicus_Y_G_57_14_uid58923	NC_012622	chromosome										
	229579404	hypothetical protein YG5714_1620	No	0	YES		1470430	1471053 +		Sulfolobus_islandicus_Y_G_57_14_uid58923	NC_012622	chromosome			arCOG08466	4 (-)	S		Uncharacterized conserved protein			
	229579405	hypothetical protein YG5714_1621	No	0	YES		1471066	1473084 +		Sulfolobus_islandicus_Y_G_57_14_uid58923	NC_012622	chromosome			arCOG09873	3	Y		RepA plasmid replication protein			
	229579406	hypothetical protein YG5714_1622	No	0	YES		1473707	1476808 +		Sulfolobus_islandicus_Y_G_57_14_uid58923	NC_012622	chromosome			arCOG02632	3	V	HsdM	Type I restriction-modification system methyltransferase subunit			
	229579407	hypothetical protein YG5714_1623	No	0	YES		1477039	1477308 +		Sulfolobus_islandicus_Y_G_57_14_uid58923	NC_012622	chromosome										
	229579408	hypothetical protein YG5714_1624	No	2	YES		1477596	1478708 +		Sulfolobus_islandicus_Y_G_57_14_uid58923	NC_012622	chromosome			arCOG08469	4	S		Uncharacterized conserved protein			
	229579409	hypothetical protein YG5714_1625	No	0	YES		1478712	1479443 +		Sulfolobus_islandicus_Y_G_57_14_uid58923	NC_012622	chromosome			arCOG08470	4	S		Uncharacterized conserved protein			
	229579410	stem cell self-renewal protein Piwi domain prot	No	0	YES		1479440	1480819 -		Sulfolobus_islandicus_Y_G_57_14_uid58923	NC_012622	chromosome			arCOG06164	4 (-)	V		Inactivated PIWI domain containing phage-defense protein			
===									6	Sulfolobus_islandicus_Y_G_57_14_uid58923	NC_012622	chromosome										
	229579416	conserved conjugative plasmid protein	No	2	YES		1486957	1487256 -		Sulfolobus_islandicus_Y_G_57_14_uid58923	NC_012622	chromosome			arCOG08561	3	S		Uncharacterized conserved protein			
	229579417	conserved conjugative plasmid membrane pro	No	3	YES		1487299	1487841 +		Sulfolobus_islandicus_Y_G_57_14_uid58923	NC_012622	chromosome			arCOG08562	3	S		Uncharacterized conserved protein			
	229579418	conserved conjugative plasmid protein	No	0	YES		1487876	1488034 -		Sulfolobus_islandicus_Y_G_57_14_uid58923	NC_012622	chromosome			arCOG08563	3	S		Uncharacterized conserved protein			
	229579419	hypothetical protein YG5714_1635	No	0	YES		1488174	1489070 -		Sulfolobus_islandicus_Y_G_57_14_uid58923	NC_012622	chromosome			arCOG07186	2 (-)	V		PIN domain			
	229579420	hypothetical protein YG5714_1636	No	1	YES		1489045	1489755 -		Sulfolobus_islandicus_Y_G_57_14_uid58923	NC_012622	chromosome			arCOG08323	1	S		Uncharacterized conserved protein	0.0001	COG5101	Importin beta-related nuclear transport
	229579421	hypothetical protein YG5714_1637	No	2	YES		1490009	1490542 +		Sulfolobus_islandicus_Y_G_57_14_uid58923	NC_012622	chromosome										
===									12	Sulfolobus_islandicus_Y_G_57_14_uid58923	NC_012622	chromosome										
	229579532	hypothetical protein YG5714_1752	No	0	YES	Virus_Fusellov	1586904	1587623 -		Sulfolobus_islandicus_Y_G_57_14_uid58923	NC_012622	chromosome			arCOG07960	16 (-)	R		ATPase (AAA+ superfamily)			
	229579533	hypothetical protein YG5714_1753	No	0	YES	Virus_Fusellov	1587610	1588077 -		Sulfolobus_islandicus_Y_G_57_14_uid58923	NC_012622	chromosome			arCOG08564	3	Y		Sulfolobus virus associated uncharacterized protein			
	229579534	hypothetical protein YG5714_1754	No	1	YES	Virus_Fusellov	1588083	1588910 -		Sulfolobus_islandicus_Y_G_57_14_uid58923	NC_012622	chromosome			arCOG08565	3	Y		Sulfolobus virus associated uncharacterized protein			
	229579535	hypothetical protein YG5714_1755	No	3	YES	Virus_Fusellov	1588903	1589187 -		Sulfolobus_islandicus_Y_G_57_14_uid58923	NC_012622	chromosome			arCOG08566	3	Y		Virus associated protein			
	229579536	hypothetical protein YG5714_1756	No	3	YES	Virus_Fusellov	1589203	1589454 -		Sulfolobus_islandicus_Y_G_57_14_uid58923	NC_012622	chromosome			arCOG08567	3	Y		Virus associated protein			
	229579537	VP1/VP3 family protein	No	2	YES	Virus_Fusellov	1589487	1589765 -		Sulfolobus_islandicus_Y_G_57_14_uid58923	NC_012622	chromosome			arCOG08568	3	Y		Virus associated protein, V3 family	3E-34	pfam06281	Protein of unknown function (DUF1035). T
	229579538	VP1/VP3 family protein	No	3	YES	Virus_Fusellov	1589777	1590127 -		Sulfolobus_islandicus_Y_G_57_14_uid58923	NC_012622	chromosome			arCOG08569	2	Y		Virus associated protein, V1 family	6E-12	pfam06281	Protein of unknown function (DUF1035). T
	229579539	hypothetical protein YG5714_1759	No	1	YES	Virus_Fusellov	1590111	1590419 -		Sulfolobus_islandicus_Y_G_57_14_uid58923	NC_012622	chromosome			arCOG09949	2	S		Uncharacterized conserved protein	0.0000002	COG0640	Predicted transcriptional regulators
	229579540	hypothetical protein YG5714_1760	Yes	5	YES	Virus_Fusellov	1590416	1590919 -		Sulfolobus_islandicus_Y_G_57_14_uid58923	NC_012622	chromosome			arCOG08570	3	Y		Virus associated protein			
	229579541	hypothetical protein YG5714_1761	No	2	YES	Virus_Fusellov	1590909	1591148 -		Sulfolobus_islandicus_Y_G_57_14_uid58923	NC_012622	chromosome			arCOG08571	3	Y		Sulfolobus virus associated uncharacterized membrane protein			
	229579542	hypothetical protein YG5714_1762	Yes	4	YES	Virus_Fusellov	1591145	1593592 -		Sulfolobus_islandicus_Y_G_57_14_uid58923	NC_012622	chromosome			arCOG07848	3 (-)	S		Uncharacterized conserved protein			
	229579543	hypothetical protein YG5714_1763	No	1	YES	Virus_Fusellov	1594028	1595032 -		Sulfolobus_islandicus_Y_G_57_14_uid58923	NC_012622	chromosome										
===									8	Sulfolobus_islandicus_Y_G_57_14_uid58923	NC_012622	chromosome										
	229579547	transcriptional regulator, CopG family	No	0	YES	Virus_Fusellov	1596000	1596194 -		Sulfolobus_islandicus_Y_G_57_14_uid58923	NC_012622	chromosome			arCOG08616	1 (-)	V		Ribbon-helix-helix protein, copG family	0.00009	pfam01402	Ribbon-helix-helix protein, copG family.
	229579548	hypothetical protein YG5714_1768	No	0	YES	Virus_Fusellov	1596191	1596370 -		Sulfolobus_islandicus_Y_G_57_14_uid58923	NC_012622	chromosome										
	229579549	hypothetical protein YG5714_1769	No	0	YES	Virus_Fusellov	1596367	1596498 -		Sulfolobus_islandicus_Y_G_57_14_uid58923	NC_012622	chromosome										
	229579550	hypothetical protein YG5714_1770	No	0	YES	Virus_Fusellov	1596510	1596818 -		Sulfolobus_islandicus_Y_G_57_14_uid58923	NC_012622	chromosome			arCOG09950	2	S		Uncharacterized conserved protein			
	229579551	transcriptional regulator, CopG family	No	0	YES	Virus_Fusellov	1596936	1597103 +		Sulfolobus_islandicus_Y_G_57_14_uid58923	NC_012622	chromosome								0.00003	COG0864	Predicted transcriptional regulators con
	229579552	hypothetical protein YG5714_1772	No	0	YES	Virus_Fusellov	1597534	1597821 +		Sulfolobus_islandicus_Y_G_57_14_uid58923	NC_012622	chromosome								1E-20	pfam09645	F-112 protein. F-112 protein is of 70-11
	229579553	hypothetical protein YG5714_1773	No	0	YES	Virus_Fusellov	1597828	1598013 +		Sulfolobus_islandicus_Y_G_57_14_uid58923	NC_012622	chromosome			arCOG09951	2	S		Uncharacterized conserved protein			
	229579554	hypothetical protein YG5714_1774	No	0	YES	Virus_Fusellov	1598010	1598585 +		Sulfolobus_islandicus_Y_G_57_14_uid58923	NC_012622	chromosome			arCOG08574	2 (-)	L		Viral/Archaeal nuclease			
===									5	Sulfolobus_islandicus_Y_G_57_14_uid58923	NC_012622	chromosome										
	229579796	Transposase, ISC1234/ST1916	No	4	YES		1815215	1816144 -		Sulfolobus_islandicus_Y_G_57_14_uid58923	NC_012622	chromosome			arCOG09887	2	S		Uncharacterized conserved protein			
	229579797	ORF1 in transposon ISC1225	No	0	YES		1816260	1816739 -		Sulfolobus_islandicus_Y_G_57_14_uid58923	NC_012622	chromosome			arCOG03902	8 (-)	L		Transposase	1E-31	pfam14210	Domain of unknown function (DUF4322). Th
	229579798	hypothetical protein YG5714_3003	No	0	YES		1816900	1817028 +		Sulfolobus_islandicus_Y_G_57_14_uid58923	NC_012622	chromosome										
	229579799	hypothetical protein YG5714_2021	No	0	YES		1817388	1817879 +		Sulfolobus_islandicus_Y_G_57_14_uid58923	NC_012622	chromosome			arCOG09923	2	S		Uncharacterized conserved protein			
	229579800	hypothetical protein YG5714_3002	No	0	YES		1818073	1818177 +		Sulfolobus_islandicus_Y_G_57_14_uid58923	NC_012622	chromosome										
===									7	Sulfolobus_islandicus_Y_N_15_51_uid58825	NC_012623	chromosome										
	229580838	transposase IS116/IS110/IS902 family protein	No	0			103822	104973 -		Sulfolobus_islandicus_Y_N_15_51_uid58825	NC_012623	chromosome			arCOG03585	6 (-)	L		Transposase	5E-25	COG3547	Transposase and inactivated derivatives
	229580839	hypothetical protein YN1551_0120	No	0			106274	106411 +		Sulfolobus_islandicus_Y_N_15_51_uid58825	NC_012623	chromosome										
	229580840	hypothetical protein YN1551_0121	No	0			106408	106833 +		Sulfolobus_islandicus_Y_N_15_51_uid58825	NC_012623	chromosome								0.0000002	pfam03243	Alkylmercury lyase. Alkylmercury lyase (
	229580841	hypothetical protein YN1551_0122	No	2			106838	107008 +		Sulfolobus_islandicus_Y_N_15_51_uid58825	NC_012623	chromosome										
	229580842	hypothetical protein YN1551_0123	No	7			107029	107790 +		Sulfolobus_islandicus_Y_N_15_51_uid58825	NC_012623	chromosome										
	229580843	amino acid transporter, putative	No	3			108807	109256 -		Sulfolobus_islandicus_Y_N_15_51_uid58825	NC_012623	chromosome			arCOG03462	1 (-)	E	PotE	Amino acid transporter	0.000002	pfam13520	Amino acid permease.
	229580844	hypothetical protein YN1551_0126	No	0			109400	109519 -		Sulfolobus_islandicus_Y_N_15_51_uid58825	NC_012623	chromosome										
===									7	Sulfolobus_islandicus_Y_N_15_51_uid58825	NC_012623	chromosome										
	229581521	hypothetical protein YN1551_0876	No	0	YES		789539	789820 -		Sulfolobus_islandicus_Y_N_15_51_uid58825	NC_012623	chromosome			arCOG01665	6 (-)	V	RelE	Cytotoxic translational repressor of toxin-antitoxin stability system			
	229581522	hypothetical protein YN1551_0878	No	0	YES		790630	791127 -		Sulfolobus_islandicus_Y_N_15_51_uid58825	NC_012623	chromosome			arCOG09957	1	S		Uncharacterized conserved protein			
	229581523	hypothetical protein YN1551_0879	No	0	YES		792150	792329 +		Sulfolobus_islandicus_Y_N_15_51_uid58825	NC_012623	chromosome										
	229581524	Transposase, ISC1234/ST1916	No	4	YES		793552	794481 -		Sulfolobus_islandicus_Y_N_15_51_uid58825	NC_012623	chromosome			arCOG09887	2	S		Uncharacterized conserved protein			
	229581525	hypothetical protein YN1551_0882	No	0	YES		794955	795272 +		Sulfolobus_islandicus_Y_N_15_51_uid58825	NC_012623	chromosome			arCOG09956	1	S		Uncharacterized conserved protein			
	229581526	hypothetical protein YN1551_0883	No	0	YES		795543	795794 -		Sulfolobus_islandicus_Y_N_15_51_uid58825	NC_012623	chromosome			arCOG09955	1	S		Uncharacterized conserved protein			
	229581527	metallophosphoesterase	No	0			796189	797349 -		Sulfolobus_islandicus_Y_N_15_51_uid58825	NC_012623	chromosome			arCOG01154	1 (-)	R		Calcineurin-like phosphoesterase superfamily pro	2E-15	COG4186	Predicted phosphoesterase or phosphohydr
===									6	Sulfolobus_islandicus_Y_N_15_51_uid58825	NC_012623	chromosome										
	229582102	hypothetical protein YN1551_1489	No	0			1299163	1299432 +		Sulfolobus_islandicus_Y_N_15_51_uid58825	NC_012623	chromosome			arCOG04308	1 (-)	S		Uncharacterized conserved protein	2E-31	pfam03685	Uncharacterized protein family (UPF0147)
	229582103	hypothetical protein YN1551_1491	No	4			1300722	1301105 +		Sulfolobus_islandicus_Y_N_15_51_uid58825	NC_012623	chromosome										
	229582104	hypothetical protein YN1551_1492	No	0			1301125	1301301 -		Sulfolobus_islandicus_Y_N_15_51_uid58825	NC_012623	chromosome										
	229582105	hypothetical protein YN1551_1493	No	0			1301298	1301525 -		Sulfolobus_islandicus_Y_N_15_51_uid58825	NC_012623	chromosome			arCOG09858	2	S		Uncharacterized conserved protein			
	229582106	hypothetical protein YN1551_1494	No	0			1301563	1302033 -		Sulfolobus_islandicus_Y_N_15_51_uid58825	NC_012623	chromosome			arCOG08376	2	S		Uncharacterized conserved protein			
	229582107	hypothetical protein YN1551_1495	No	0			1302067	1302399 -		Sulfolobus_islandicus_Y_N_15_51_uid58825	NC_012623	chromosome			arCOG08459	2 (-)	S		Uncharacterized conserved protein			
===									9	Sulfolobus_islandicus_Y_N_15_51_uid58825	NC_012623	chromosome										
	229582563	hypothetical protein YN1551_2003	No	0			1768742	1769143 -		Sulfolobus_islandicus_Y_N_15_51_uid58825	NC_012623	chromosome			arCOG04017	1 (-)	S		Uncharacterized conserved protein			
	229582564	hypothetical protein YN1551_2004	No	0			1769100	1769252 -		Sulfolobus_islandicus_Y_N_15_51_uid58825	NC_012623	chromosome			arCOG09932	1	S		Uncharacterized conserved protein			
	229582565	hypothetical protein YN1551_2005	No	0			1769520	1770056 -		Sulfolobus_islandicus_Y_N_15_51_uid58825	NC_012623	chromosome										
	229582566	hypothetical protein YN1551_2007	No	0			1771058	1772920 -		Sulfolobus_islandicus_Y_N_15_51_uid58825	NC_012623	chromosome			arCOG08580	3	E		Uncharacterized conserved protein			
	229582567	hypothetical protein YN1551_2008	No	0			1773149	1774120 +		Sulfolobus_islandicus_Y_N_15_51_uid58825	NC_012623	chromosome										
	229582568	hypothetical protein YN1551_2009	No	0			1774126	1775808 +		Sulfolobus_islandicus_Y_N_15_51_uid58825	NC_012623	chromosome										
	229582569	hypothetical protein YN1551_2010	No	4			1775825	1776274 +		Sulfolobus_islandicus_Y_N_15_51_uid58825	NC_012623	chromosome										
	229582570	hypothetical protein YN1551_2011	Yes	2			1776455	1778566 +		Sulfolobus_islandicus_Y_N_15_51_uid58825	NC_012623	chromosome		arCOG03671,a	arCOG03671	3 (-)	E		Thermopsin-like protease			
	229582571	hypothetical protein YN1551_2012	No	0			1778650	1778949 +		Sulfolobus_islandicus_Y_N_15_51_uid58825	NC_012623	chromosome										
===									10	Sulfolobus_islandicus_Y_N_15_51_uid58825	NC_012623	chromosome										
	229582787	hypothetical protein YN1551_3273	No	0	YES		2031200	2031337 -		Sulfolobus_islandicus_Y_N_15_51_uid58825	NC_012623	chromosome										
	229582788	Thermopsin	Yes	4	YES		2031460	2032839 -		Sulfolobus_islandicus_Y_N_15_51_uid58825	NC_012623	chromosome			arCOG03672	1 (-)	E		Thermopsin-like protease	2E-66	pfam05317	Thermopsin. This family consists of seve
	229582789	hypothetical protein YN1551_2283	No	0	YES		2033583	2033714 -		Sulfolobus_islandicus_Y_N_15_51_uid58825	NC_012623	chromosome			arCOG09919	3	S		Uncharacterized conserved protein			
	229582790	hypothetical protein YN1551_2284	No	0	YES		2033935	2034447 +		Sulfolobus_islandicus_Y_N_15_51_uid58825	NC_012623	chromosome			arCOG09920	3	S		Uncharacterized conserved protein			Zn finger and something
	229582791	hypothetical protein YN1551_2285	No	0	YES		2034473	2035003 +		Sulfolobus_islandicus_Y_N_15_51_uid58825	NC_012623	chromosome			arCOG09921	3	S		Uncharacterized conserved protein			
	229582792	hypothetical protein YN1551_2287	No	0	YES		2036206	2036667 +		Sulfolobus_islandicus_Y_N_15_51_uid58825	NC_012623	chromosome			arCOG09819	2	S		Uncharacterized conserved protein			
	229582793	hypothetical protein YN1551_2288	No	0	YES		2036657	2037868 +		Sulfolobus_islandicus_Y_N_15_51_uid58825	NC_012623	chromosome			arCOG00890	3 (-)	L		DNA modification methylase	0.00003	COG0863	DNA modification methylase
	229582794	hypothetical protein YN1551_2289	No	0	YES		2037914	2038069 +		Sulfolobus_islandicus_Y_N_15_51_uid58825	NC_012623	chromosome			arCOG11080	2	S		Uncharacterized conserved protein			
	229582795	hypothetical protein YN1551_2290	No	0	YES		2038123	2039325 +		Sulfolobus_islandicus_Y_N_15_51_uid58825	NC_012623	chromosome	clust_83									
	229582796	hypothetical protein YN1551_2291	No	0	YES		2039411	2039599 -		Sulfolobus_islandicus_Y_N_15_51_uid58825	NC_012623	chromosome										
===									6	Sulfolobus_islandicus_Y_N_15_51_uid58825	NC_012623	chromosome										
	229582904	hypothetical protein YN1551_2414	No	0	YES		2164518	2165132 +		Sulfolobus_islandicus_Y_N_15_51_uid58825	NC_012623	chromosome			arCOG08414	1	S		Uncharacterized conserved protein			
	229582905	hypothetical protein YN1551_2415	No	0	YES		2165538	2165963 -		Sulfolobus_islandicus_Y_N_15_51_uid58825	NC_012623	chromosome			arCOG07711	1	S		Uncharacterized conserved protein			
	229582906	hypothetical protein YN1551_2416	No	0	YES		2165936	2166397 -		Sulfolobus_islandicus_Y_N_15_51_uid58825	NC_012623	chromosome			arCOG07712	1	S		Uncharacterized conserved protein			
	229582907	hypothetical protein YN1551_2417	No	0	YES		2166394	2166852 -		Sulfolobus_islandicus_Y_N_15_51_uid58825	NC_012623	chromosome			arCOG07156	1 (-)	S		Uncharacterized conserved protein			
	229582908	hypothetical protein YN1551_2420	No	0	YES		2167416	2167838 -		Sulfolobus_islandicus_Y_N_15_51_uid58825	NC_012623	chromosome			arCOG08544	3	V		HEPN domain containing protein, paRep1 subfamily			
	229582909	hypothetical protein YN1551_2421	No	0	YES		2167880	2168131 +		Sulfolobus_islandicus_Y_N_15_51_uid58825	NC_012623	chromosome			arCOG08545	2	S		Uncharacterized conserved protein			
===									6	Sulfolobus_islandicus_Y_N_15_51_uid58825	NC_012623	chromosome										
	229582916	hypothetical protein YN1551_2429	No	0	YES		2174803	2175183 -		Sulfolobus_islandicus_Y_N_15_51_uid58825	NC_012623	chromosome			arCOG07248	2 (-)	V		PIN domain			
	229582917	hypothetical protein YN1551_2430	No	0	YES		2175183	2175695 -		Sulfolobus_islandicus_Y_N_15_51_uid58825	NC_012623	chromosome			arCOG08547	3	S		Uncharacterized conserved protein			
	229582918	hypothetical protein YN1551_2431	No	0	YES		2175698	2176297 -		Sulfolobus_islandicus_Y_N_15_51_uid58825	NC_012623	chromosome			arCOG08548	3	S		Uncharacterized conserved protein			
	229582919	hypothetical protein YN1551_2432	No	0	YES		2176442	2176576 -		Sulfolobus_islandicus_Y_N_15_51_uid58825	NC_012623	chromosome			arCOG09918	1	S		Uncharacterized conserved protein			
	229582920	hypothetical protein YN1551_2433	No	0	YES		2176640	2176804 +		Sulfolobus_islandicus_Y_N_15_51_uid58825	NC_012623	chromosome			arCOG09943	1	S		Uncharacterized conserved protein			
	229582921	transposase	No	0	YES		2176794	2177876 +		Sulfolobus_islandicus_Y_N_15_51_uid58825	NC_012623	chromosome			arCOG04197	4 (-)	R		Transposase related protein			
===									5	Sulfolobus_islandicus_Y_N_15_51_uid58825	NC_012624	plasmid pYN01										
	228288731	hypothetical protein YN1551_3185	No	0	YES	plasmid	7145	7489 -		Sulfolobus_islandicus_Y_N_15_51_uid58825	NC_012624	plasmid pYN01			arCOG08330	1	S		Uncharacterized conserved protein			
	228288732	hypothetical protein YN1551_3186	No	0	YES	plasmid	7486	7698 -		Sulfolobus_islandicus_Y_N_15_51_uid58825	NC_012624	plasmid pYN01			arCOG08464	2	S		Uncharacterized conserved protein			
	228288733	hypothetical protein YN1551_3187	No	0	YES	plasmid	7691	7981 -		Sulfolobus_islandicus_Y_N_15_51_uid58825	NC_012624	plasmid pYN01			arCOG09990	2	S		Uncharacterized conserved protein			
	228288734	hypothetical protein YN1551_3188	No	0	YES	plasmid	7974	8465 -		Sulfolobus_islandicus_Y_N_15_51_uid58825	NC_012624	plasmid pYN01			arCOG08465	3 (-)	S		Uncharacterized conserved protein			
	228288735	hypothetical protein YN1551_3189	No	0	YES	plasmid	8462	8629 -		Sulfolobus_islandicus_Y_N_15_51_uid58825	NC_012624	plasmid pYN01			arCOG09941	1	S		Uncharacterized conserved protein			
===									7	Sulfolobus_islandicus_Y_N_15_51_uid58825	NC_012624	plasmid pYN01										
	228288739	hypothetical protein YN1551_3194	No	0	YES	plasmid	11394	11675 +		Sulfolobus_islandicus_Y_N_15_51_uid58825	NC_012624	plasmid pYN01										
	228288740	hypothetical protein YN1551_3195	No	0	YES	plasmid	11785	12426 +		Sulfolobus_islandicus_Y_N_15_51_uid58825	NC_012624	plasmid pYN01			arCOG08466	4 (-)	S		Uncharacterized conserved protein			
	228288741	conserved conjugative plasmid protein	No	0	YES	plasmid	12525	12968 +		Sulfolobus_islandicus_Y_N_15_51_uid58825	NC_012624	plasmid pYN01			arCOG09998	2	S		Uncharacterized conserved protein			
	228288742	hypothetical protein YN1551_3197	No	0	YES	plasmid	13406	13744 -		Sulfolobus_islandicus_Y_N_15_51_uid58825	NC_012624	plasmid pYN01			arCOG08468	3	S		Uncharacterized conserved protein			
	228288743	hypothetical protein YN1551_3198	No	0	YES	plasmid	13878	14015 +		Sulfolobus_islandicus_Y_N_15_51_uid58825	NC_012624	plasmid pYN01										
	228288744	hypothetical protein YN1551_3199	No	0	YES	plasmid	14143	14298 +		Sulfolobus_islandicus_Y_N_15_51_uid58825	NC_012624	plasmid pYN01										
	228288745	restriction modification system DNA specificity	No	0	YES	plasmid	14609	16339 -		Sulfolobus_islandicus_Y_N_15_51_uid58825	NC_012624	plasmid pYN01			arCOG00724	7 (-)	K		Predicted transcriptional regulator, PadR family	6E-24	COG0732	Restriction endonuclease S subunits
===									5	Sulfolobus_solfataricus_98_2_uid167998	NC_017274	chromosome										
	384434016	hypothetical protein	No	0			1407961	1408152 +		Sulfolobus_solfataricus_98_2_uid167998	NC_017274	chromosome			arCOG09993	2	S		Uncharacterized conserved protein			
	384434017	hypothetical protein	No	0			1408145	1408327 +		Sulfolobus_solfataricus_98_2_uid167998	NC_017274	chromosome			arCOG07847	2 (-)	S		Uncharacterized conserved protein			
	384434018	hypothetical protein	No	0			1408484	1408657 +		Sulfolobus_solfataricus_98_2_uid167998	NC_017274	chromosome										
	384434019	hypothetical protein	No	4			1408626	1409147 -		Sulfolobus_solfataricus_98_2_uid167998	NC_017274	chromosome			arCOG08562	3	S		Uncharacterized conserved protein			
	384434020	hypothetical protein	No	2			1409182	1409481 +		Sulfolobus_solfataricus_98_2_uid167998	NC_017274	chromosome			arCOG08561	3	S		Uncharacterized conserved protein			
===									8	Sulfolobus_solfataricus_98_2_uid167998	NC_017274	chromosome										
	384434902	hypothetical protein	No	0			2334817	2335071 -		Sulfolobus_solfataricus_98_2_uid167998	NC_017274	chromosome			arCOG01401	2 (-)	Q		SAM-dependent methyltransferase			
	384434903	hypothetical protein	No	0			2335245	2335514 -		Sulfolobus_solfataricus_98_2_uid167998	NC_017274	chromosome										
	384434904	hypothetical protein	No	0			2336063	2336158 +		Sulfolobus_solfataricus_98_2_uid167998	NC_017274	chromosome										
	384434905	hypothetical protein	No	3			2336155	2336496 +		Sulfolobus_solfataricus_98_2_uid167998	NC_017274	chromosome										
	384434906	hypothetical protein	No	0			2337205	2337330 +		Sulfolobus_solfataricus_98_2_uid167998	NC_017274	chromosome										
	384434907	hypothetical protein	No	0			2337946	2338347 -		Sulfolobus_solfataricus_98_2_uid167998	NC_017274	chromosome			arCOG07298	2	S		Uncharacterized conserved protein			
	384434908	hypothetical protein	No	0			2338372	2338881 -		Sulfolobus_solfataricus_98_2_uid167998	NC_017274	chromosome	clust_187									
	384434909	transposase IS116/IS110/IS902 family protein	No	0			2339232	2340182 -		Sulfolobus_solfataricus_98_2_uid167998	NC_017274	chromosome		arCOG03586,a	arCOG03586	3 (-)	L		Transposase	3E-36	COG3547	Transposase and inactivated derivatives
===									6	Sulfolobus_solfataricus_98_2_uid167998	NC_017274	chromosome										
	384434937	hypothetical protein	No	0			2369449	2369889 -		Sulfolobus_solfataricus_98_2_uid167998	NC_017274	chromosome		arCOG09877,a	arCOG09877	1	S		Uncharacterized conserved protein			
	384434938	hypothetical protein	No	0			2370708	2371604 +		Sulfolobus_solfataricus_98_2_uid167998	NC_017274	chromosome			arCOG07190	1	S		Uncharacterized conserved protein			
	384434939	hypothetical protein	No	10			2371636	2372745 +		Sulfolobus_solfataricus_98_2_uid167998	NC_017274	chromosome			arCOG07189	1	S		Uncharacterized conserved protein			
	384434940	major facilitator superfamily protein	No	10			2374634	2375782 -		Sulfolobus_solfataricus_98_2_uid167998	NC_017274	chromosome			arCOG00132	2 (-)	G	ProP	Permease of the major facilitator superfamily	2E-12	pfam07690	Major Facilitator Superfamily.
	384434941	hypothetical protein	No	0			2375779	2376621 -		Sulfolobus_solfataricus_98_2_uid167998	NC_017274	chromosome			arCOG10015	2	S		Uncharacterized conserved protein	2E-102	COG4278	Uncharacterized conserved protein
	384434942	hypothetical protein	No	0			2376657	2376926 -		Sulfolobus_solfataricus_98_2_uid167998	NC_017274	chromosome			arCOG10016	2	S		Uncharacterized conserved protein			
===									7	Sulfolobus_solfataricus_98_2_uid167998	NC_017274	chromosome										
	384435148	hypothetical protein	No	1			2590206	2590307 -		Sulfolobus_solfataricus_98_2_uid167998	NC_017274	chromosome										
	384435149	hypothetical protein	Yes	0			2590297	2590398 -		Sulfolobus_solfataricus_98_2_uid167998	NC_017274	chromosome										
	384435150	hypothetical protein	No	3			2590386	2590697 -		Sulfolobus_solfataricus_98_2_uid167998	NC_017274	chromosome										
	384435151	PaREP1 family protein	No	0			2591406	2591771 +		Sulfolobus_solfataricus_98_2_uid167998	NC_017274	chromosome			arCOG03721	3 (-)	V		HEPN domain containing protein	8E-18	pfam05942	Archaeal PaREP1/PaREP8 family. This fami
	384435152	hypothetical protein	No	1			2591866	2592015 +		Sulfolobus_solfataricus_98_2_uid167998	NC_017274	chromosome			arCOG08446	1	S		Uncharacterized conserved protein			
	384435153	hypothetical protein	No	0			2592232	2593395 -		Sulfolobus_solfataricus_98_2_uid167998	NC_017274	chromosome			arCOG09933	1	S		Uncharacterized conserved protein	0.0000003	COG0497	ATPase involved in DNA repair
	384435154	hypothetical protein	No	0			2593985	2594212 -		Sulfolobus_solfataricus_98_2_uid167998	NC_017274	chromosome										
===									6	Sulfolobus_solfataricus_P2_uid57721	NC_002754	chromosome										
	15898213	hypothetical protein SSO1374	No	4	YES		1209080	1209931 +		Sulfolobus_solfataricus_P2_uid57721	NC_002754	chromosome										
	15898214	hypothetical protein SSO1375	No	2	YES		1210170	1212011 +		Sulfolobus_solfataricus_P2_uid57721	NC_002754	chromosome										
	15898215	hypothetical protein SSO1376	No	4	YES		1212563	1213678 +		Sulfolobus_solfataricus_P2_uid57721	NC_002754	chromosome										
	15898216	transposase ISC1234	No	3	YES		1213832	1214791 -		Sulfolobus_solfataricus_P2_uid57721	NC_002754	chromosome			arCOG07925	2 (-)	L		Transposase ISC1234			
	15898217	hypothetical protein SSO1378	No	0	YES		1214889	1216907 +		Sulfolobus_solfataricus_P2_uid57721	NC_002754	chromosome										
	15898218	hypothetical protein SSO1379	No	3	YES		1216914	1219409 +		Sulfolobus_solfataricus_P2_uid57721	NC_002754	chromosome										
===									5	Sulfolobus_solfataricus_P2_uid57721	NC_002754	chromosome										
	15898314	ISC1043 hypothetical protein	No	1	YES		1341275	1341523 +		Sulfolobus_solfataricus_P2_uid57721	NC_002754	chromosome										
	15898315	second part of one of two inversely orientated	No	0	YES		1341520	1341696 +		Sulfolobus_solfataricus_P2_uid57721	NC_002754	chromosome										
	15898316	third part of one of two inversely orientated orfs	No	0	YES		1341626	1341778 +		Sulfolobus_solfataricus_P2_uid57721	NC_002754	chromosome										
	15898317	transposon ISC1190	No	0	YES		1341997	1342947 +		Sulfolobus_solfataricus_P2_uid57721	NC_002754	chromosome			arCOG03586	3 (-)	L		Transposase	5E-36	COG3547	Transposase and inactivated derivatives
	15898318	hypothetical protein SSO1486	No	0	YES		1343008	1343346 -		Sulfolobus_solfataricus_P2_uid57721	NC_002754	chromosome			arCOG09862	1	S		Uncharacterized conserved protein			
===									6	Sulfolobus_solfataricus_P2_uid57721	NC_002754	chromosome										
	15898777	hypothetical protein SSO1981	No	0	YES		1792375	1794081 +		Sulfolobus_solfataricus_P2_uid57721	NC_002754	chromosome			arCOG07852	1 (-)	R		Predicted regulatory ATPase			
	15898778	hypothetical protein SSO1983	No	1	YES		1794085	1796007 +		Sulfolobus_solfataricus_P2_uid57721	NC_002754	chromosome										
	15898779	hypothetical protein SSO1984	No	0	YES		1796525	1796974 +		Sulfolobus_solfataricus_P2_uid57721	NC_002754	chromosome										
	15898780	hypothetical protein SSO1985	No	1	YES		1797062	1797643 -		Sulfolobus_solfataricus_P2_uid57721	NC_002754	chromosome			arCOG10265	3	S		Uncharacterized conserved protein			
	15898781	hypothetical protein SSO1986	No	0	YES		1798337	1798930 +		Sulfolobus_solfataricus_P2_uid57721	NC_002754	chromosome			arCOG08552	1	S		Uncharacterized conserved protein			
	15898782	hypothetical protein SSO1987	No	0	YES		1798944	1799870 +		Sulfolobus_solfataricus_P2_uid57721	NC_002754	chromosome			arCOG02657	1 (-)	V	Cas7/Cmr	CRISPR system related protein, RAMP superfam	4E-34	COG1336	CRISPR system related protein, RAMP supe
===									5	Sulfolobus_tokodaii_7_uid57807	NC_003106	chromosome										
	15920903	hypothetical protein ST0681	No	0			662916	663749 +		Sulfolobus_tokodaii_7_uid57807	NC_003106	chromosome			arCOG07199	1	S		Uncharacterized conserved protein			
	15920904	hypothetical protein STS079	No	1			663966	664118 -		Sulfolobus_tokodaii_7_uid57807	NC_003106	chromosome										
	15920905	hypothetical protein ST0682	No	0			664084	664647 -		Sulfolobus_tokodaii_7_uid57807	NC_003106	chromosome			arCOG02860	1 (-)	P	CynT	Carbonic anhydrase			
	15920906	hypothetical protein STS080	No	0			664807	665085 -		Sulfolobus_tokodaii_7_uid57807	NC_003106	chromosome										
	15920907	hypothetical protein STS081	No	0			665195	665368 +		Sulfolobus_tokodaii_7_uid57807	NC_003106	chromosome										
===									16	Sulfolobus_tokodaii_7_uid57807	NC_003106	chromosome										
	15921435	hypothetical protein STS131	Yes	0			1176336	1176488 -		Sulfolobus_tokodaii_7_uid57807	NC_003106	chromosome										
	15921436	hypothetical protein ST1177	No	5			1176498	1176959 -		Sulfolobus_tokodaii_7_uid57807	NC_003106	chromosome										
	15921437	hypothetical protein ST1178	No	4			1176963	1178243 -		Sulfolobus_tokodaii_7_uid57807	NC_003106	chromosome										
	15921438	hypothetical protein ST1179	No	1			1179151	1179753 -		Sulfolobus_tokodaii_7_uid57807	NC_003106	chromosome			arCOG08366	1 (-)	S		Uncharacterized conserved protein			
	15921439	hypothetical protein ST1180	No	0			1180276	1181013 +		Sulfolobus_tokodaii_7_uid57807	NC_003106	chromosome										
	15921440	hypothetical protein ST1181	No	0			1181617	1181997 -		Sulfolobus_tokodaii_7_uid57807	NC_003106	chromosome										
	15921441	hypothetical protein STS132	No	0			1183586	1183774 -		Sulfolobus_tokodaii_7_uid57807	NC_003106	chromosome			arCOG05874	1	S		Uncharacterized conserved protein			
	15921442	hypothetical protein ST1182	No	0			1183655	1183969 +		Sulfolobus_tokodaii_7_uid57807	NC_003106	chromosome										
	15921443	hypothetical protein ST1183	No	0			1183765	1184118 +		Sulfolobus_tokodaii_7_uid57807	NC_003106	chromosome										
	15921444	hypothetical protein ST1184	No	0			1184734	1185066 -		Sulfolobus_tokodaii_7_uid57807	NC_003106	chromosome										
	15921445	hypothetical protein STS133	Yes	0			1185608	1185826 +		Sulfolobus_tokodaii_7_uid57807	NC_003106	chromosome										
	15921446	hypothetical protein STS134	No	0			1185931	1186116 +		Sulfolobus_tokodaii_7_uid57807	NC_003106	chromosome										
	15921447	hypothetical protein STS135	No	0			1186147	1186437 +		Sulfolobus_tokodaii_7_uid57807	NC_003106	chromosome										
	15921449	hypothetical protein ST1186	No	0			1187338	1187700 -		Sulfolobus_tokodaii_7_uid57807	NC_003106	chromosome										
	15921450	hypothetical protein ST1187	No	4			1188548	1189147 -		Sulfolobus_tokodaii_7_uid57807	NC_003106	chromosome			arCOG03580	2 (-)	O	STE14	Putative protein-S-isoprenylcysteine methyltransf	4E-20	COG2020	Putative protein-S-isoprenylcysteine met
	15921451	hypothetical protein ST1188	No	0			1189716	1190168 -		Sulfolobus_tokodaii_7_uid57807	NC_003106	chromosome										
===									8	Sulfolobus_tokodaii_7_uid57807	NC_003106	chromosome										
	15921601	hypothetical protein ST1321	No	0			1332209	1332676 +		Sulfolobus_tokodaii_7_uid57807	NC_003106	chromosome			arCOG08473	1	S		Uncharacterized conserved protein			
	15921602	hypothetical protein ST1322	No	1			1332712	1333380 +		Sulfolobus_tokodaii_7_uid57807	NC_003106	chromosome										
	15921603	hypothetical protein ST1323	No	0			1333510	1334703 -		Sulfolobus_tokodaii_7_uid57807	NC_003106	chromosome			arCOG03167	6 (-)	R		Predicted ATPase (AAA+ superfamily)			
	15921604	hypothetical protein STS158	No	0			1335059	1335340 -		Sulfolobus_tokodaii_7_uid57807	NC_003106	chromosome			arCOG07346	1	S		Uncharacterized conserved protein			
	15921605	hypothetical protein ST1324	No	0			1335555	1335893 -		Sulfolobus_tokodaii_7_uid57807	NC_003106	chromosome										
	15921606	hypothetical protein STS159	No	0			1336031	1336222 +		Sulfolobus_tokodaii_7_uid57807	NC_003106	chromosome										
	15921607	hypothetical protein ST1325	Yes	3			1336240	1336587 +		Sulfolobus_tokodaii_7_uid57807	NC_003106	chromosome										
	15921608	hypothetical protein ST1326	No	0			1337248	1340298 +		Sulfolobus_tokodaii_7_uid57807	NC_003106	chromosome			arCOG04816	4 (-)	U		TraG/TraD/VirD4 family enzyme, ATPase	0.000000002	COG0433	HerA helicase
===									7	Sulfolobus_tokodaii_7_uid57807	NC_003106	chromosome										
	15921610	hypothetical protein STS160	No	1			1340782	1340952 +		Sulfolobus_tokodaii_7_uid57807	NC_003106	chromosome										
	15921611	hypothetical protein ST1330	No	0			1341549	1342595 -		Sulfolobus_tokodaii_7_uid57807	NC_003106	chromosome			arCOG00881	1 (-)	K	SSL2	DNA or RNA helicase of superfamily II	0.0000002	pfam13091	PLD-like domain.
	15921612	hypothetical protein ST1331	No	0			1342958	1343332 -		Sulfolobus_tokodaii_7_uid57807	NC_003106	chromosome			arCOG09856	2	S		Uncharacterized conserved protein			
	15921613	hypothetical protein ST1332	No	0			1343295	1343747 -		Sulfolobus_tokodaii_7_uid57807	NC_003106	chromosome			arCOG09855	2	S		Uncharacterized conserved protein			
	15921614	hypothetical protein ST1333	No	0			1343835	1344197 -		Sulfolobus_tokodaii_7_uid57807	NC_003106	chromosome			arCOG10022	2	S		Uncharacterized conserved protein			
	15921615	hypothetical protein ST1334	No	1			1344672	1345367 +		Sulfolobus_tokodaii_7_uid57807	NC_003106	chromosome			arCOG09969	1	S		Uncharacterized conserved protein			
	15921616	hypothetical protein ST1335	No	0			1345504	1346523 +		Sulfolobus_tokodaii_7_uid57807	NC_003106	chromosome			arCOG08191	1 (-)	L		DNA methylase			
===									7	Sulfolobus_tokodaii_7_uid57807	NC_003106	chromosome										
	15921897	hypothetical protein ST1597	No	11	YES		1592977	1594107 -		Sulfolobus_tokodaii_7_uid57807	NC_003106	chromosome			arCOG07253	3	S		Uncharacterized conserved protein			
	15921898	hypothetical protein ST1598	No	0	YES		1596304	1596612 +		Sulfolobus_tokodaii_7_uid57807	NC_003106	chromosome			arCOG03902	8 (-)	L		Transposase			
	15921899	hypothetical protein ST1599	Yes	1	YES		1597034	1597837 +		Sulfolobus_tokodaii_7_uid57807	NC_003106	chromosome			arCOG07212	1	S		Uncharacterized conserved protein			
	15921900	hypothetical protein ST1600	No	1	YES		1597841	1598473 +		Sulfolobus_tokodaii_7_uid57807	NC_003106	chromosome			arCOG07211	2	S		Uncharacterized conserved protein			
	15921901	hypothetical protein ST1601	Yes	1	YES		1598555	1599160 +		Sulfolobus_tokodaii_7_uid57807	NC_003106	chromosome			arCOG07211	2	S		Uncharacterized conserved protein			
	15921902	hypothetical protein ST1602	No	0	YES		1599433	1600215 +		Sulfolobus_tokodaii_7_uid57807	NC_003106	chromosome			arCOG01422	2 (-)	R		RecB-like nuclease fused to coiled-coil domain	2E-99	COG5493	Uncharacterized conserved protein contai
	15921903	hypothetical protein ST1603	No	4	YES		1599519	1599896 -		Sulfolobus_tokodaii_7_uid57807	NC_003106	chromosome										
===									5	Sulfolobus_tokodaii_7_uid57807	NC_003106	chromosome										
	15922037	hypothetical protein ST1727	No	3			1730401	1730712 +		Sulfolobus_tokodaii_7_uid57807	NC_003106	chromosome			arCOG08572	1	Y		Sulfolobus virus associated uncharacterized membrane protein			
	15922038	hypothetical protein ST1728	No	0			1731468	1731800 +		Sulfolobus_tokodaii_7_uid57807	NC_003106	chromosome										
	15922039	hypothetical protein ST1729	No	2			1731808	1732647 +		Sulfolobus_tokodaii_7_uid57807	NC_003106	chromosome										
	15922040	ABC transporter ATP-binding protein	No	0			1732650	1733456 +		Sulfolobus_tokodaii_7_uid57807	NC_003106	chromosome			arCOG00208	5 (-)	V	CcmA	ABC-type multidrug transport system, ATPase co	7E-27	COG1131	ABC-type multidrug transport system, ATP
	15922041	hypothetical protein ST1731	No	6			1733443	1734204 +		Sulfolobus_tokodaii_7_uid57807	NC_003106	chromosome			arCOG11078	2	S		Uncharacterized conserved protein	0.000001	pfam12679	ABC-2 family transporter protein. This f
===									7	Sulfolobus_tokodaii_7_uid57807	NC_003106	chromosome										
	15922070	hypothetical protein ST1760	No	0	YES		1773863	1774861 +		Sulfolobus_tokodaii_7_uid57807	NC_003106	chromosome			arCOG08442	1	S		Uncharacterized conserved protein			
	15922071	hypothetical protein ST1761	No	0	YES		1775710	1776588 +		Sulfolobus_tokodaii_7_uid57807	NC_003106	chromosome			arCOG01422	2 (-)	R		RecB-like nuclease fused to coiled-coil domain	6E-73	COG5493	Uncharacterized conserved protein contai
	15922072	hypothetical protein ST1762	No	4	YES		1775799	1776257 -		Sulfolobus_tokodaii_7_uid57807	NC_003106	chromosome										
	15922073	hypothetical protein STS197	No	0	YES		1777914	1778084 -		Sulfolobus_tokodaii_7_uid57807	NC_003106	chromosome										
	15922074	hypothetical protein STS198	No	0	YES		1778200	1778406 +		Sulfolobus_tokodaii_7_uid57807	NC_003106	chromosome										
	15922075	hypothetical protein ST1763	No	0	YES		1778699	1779808 +		Sulfolobus_tokodaii_7_uid57807	NC_003106	chromosome			arCOG07301	1 (-)	S		Uncharacterized conserved protein			
	15922076	hypothetical protein ST1764	No	0	YES		1779957	1783070 -		Sulfolobus_tokodaii_7_uid57807	NC_003106	chromosome			arCOG05901	1	R		Predicted ATPase	0.00006	COG0419	ATPase involved in DNA repair
===									5	Sulfolobus_tokodaii_7_uid57807	NC_003106	chromosome										
	15922238	hypothetical protein ST1923	No	0			1932636	1933385 +		Sulfolobus_tokodaii_7_uid57807	NC_003106	chromosome			arCOG09705	1	S		Uncharacterized conserved protein			
	15922239	hypothetical protein ST1924	No	0			1934250	1934708 +		Sulfolobus_tokodaii_7_uid57807	NC_003106	chromosome			arCOG07308	1	S		Uncharacterized conserved protein			
	15922240	hypothetical protein ST1925	No	0			1934681	1935190 +		Sulfolobus_tokodaii_7_uid57807	NC_003106	chromosome			arCOG07851	1	S		Uncharacterized conserved protein			
	15922241	hypothetical protein ST1926	No	0			1935647	1936708 +		Sulfolobus_tokodaii_7_uid57807	NC_003106	chromosome			arCOG05431	1 (-)	M	RfaG	Glycosyltransferase	0.000000004	COG0438	Glycosyltransferase
	15922242	hypothetical protein ST1927	No	1	YES		1936399	1936749 -		Sulfolobus_tokodaii_7_uid57807	NC_003106	chromosome										
===									6	Sulfolobus_tokodaii_7_uid57807	NC_003106	chromosome										
	15922249	hypothetical protein ST1933	No	0	YES		1941105	1941524 -		Sulfolobus_tokodaii_7_uid57807	NC_003106	chromosome			arCOG07248	2 (-)	V		PIN domain			
	15922250	hypothetical protein ST1934	No	0	YES		1941524	1941994 -		Sulfolobus_tokodaii_7_uid57807	NC_003106	chromosome			arCOG08547	3	S		Uncharacterized conserved protein			
	15922251	hypothetical protein ST1935	No	0	YES		1942039	1942638 -		Sulfolobus_tokodaii_7_uid57807	NC_003106	chromosome			arCOG08548	3	S		Uncharacterized conserved protein			
	15922252	hypothetical protein ST1936	No	0	YES		1943281	1943670 -		Sulfolobus_tokodaii_7_uid57807	NC_003106	chromosome			arCOG07298	2	S		Uncharacterized conserved protein			
	15922253	hypothetical protein ST1937	No	0	YES		1943838	1944395 -		Sulfolobus_tokodaii_7_uid57807	NC_003106	chromosome	clust_187									
	15922254	hypothetical protein ST1938	No	0	YES		1944470	1945795 -		Sulfolobus_tokodaii_7_uid57807	NC_003106	chromosome			arCOG00679	4 (-)	L		Transposase			
===									7	Sulfolobus_tokodaii_7_uid57807	NC_003106	chromosome										
	15922829	hypothetical protein ST2498	No	0	YES		2504284	2505543 +		Sulfolobus_tokodaii_7_uid57807	NC_003106	chromosome			arCOG01247	4 (-)	L	XerD	Site-specific recombinase XerD	0.00000001	COG4974	Site-specific recombinase XerD
	15922830	hypothetical protein ST2499	No	1	YES		2505617	2506528 +		Sulfolobus_tokodaii_7_uid57807	NC_003106	chromosome			arCOG10017	2	S		Uncharacterized conserved protein			
	15922831	hypothetical protein ST2500	No	0	YES		2506958	2507323 +		Sulfolobus_tokodaii_7_uid57807	NC_003106	chromosome			arCOG10018	2	S		Uncharacterized conserved protein			
	15922832	transcription factor	No	0	YES		2507348	2507512 -		Sulfolobus_tokodaii_7_uid57807	NC_003106	chromosome			arCOG10019	2	S		Uncharacterized conserved protein			
	15922833	plasmid copy-number control protein	No	0	YES		2507854	2508021 +		Sulfolobus_tokodaii_7_uid57807	NC_003106	chromosome								0.00007	pfam01402	Ribbon-helix-helix protein, copG family.
	15922834	hypothetical protein ST2501	No	0	YES		2508081	2508776 +		Sulfolobus_tokodaii_7_uid57807	NC_003106	chromosome			arCOG00790	6 (-)	V	Cas4	CRISPR-associated protein Cas4, RecB family ex	0.00000004	COG1468	CRISPR-associated protein Cas4 (RecB fam
	15922835	hypothetical protein ST2502	No	0	YES		2508754	2509146 +		Sulfolobus_tokodaii_7_uid57807	NC_003106	chromosome			arCOG10020	2	S		Uncharacterized conserved protein			
===									9	Sulfolobus_tokodaii_7_uid57807	NC_003106	chromosome										
	15922918	cell division control protein	No	0			2601471	2603573 +		Sulfolobus_tokodaii_7_uid57807	NC_003106	chromosome			arCOG01308	10 (-)	O	Cdc48	ATPase of the AAA+ class , CDC48 family	8E-168	COG0464	ATPases of the AAA+ class
	15922919	hypothetical protein ST2585	No	2			2602880	2603209 -		Sulfolobus_tokodaii_7_uid57807	NC_003106	chromosome										
	15922920	hypothetical protein STS253	No	2			2603853	2604053 +		Sulfolobus_tokodaii_7_uid57807	NC_003106	chromosome										
	15922921	hypothetical protein ST2586	Yes	4			2604043	2604999 -		Sulfolobus_tokodaii_7_uid57807	NC_003106	chromosome										
	15922922	hypothetical protein ST2587	No	16			2604996	2606858 -		Sulfolobus_tokodaii_7_uid57807	NC_003106	chromosome			arCOG09693	1	S		Uncharacterized conserved membrane protein			
	15922923	hypothetical protein ST2589	No	6			2607095	2607706 -		Sulfolobus_tokodaii_7_uid57807	NC_003106	chromosome			arCOG06032	1 (-)	S		Uncharacterized conserved protein	6E-22	pfam07185	Protein of unknown function (DUF1404). T
	15922924	SepZ protein	No	2			2607697	2607894 -		Sulfolobus_tokodaii_7_uid57807	NC_003106	chromosome			arCOG11006	1	S		Uncharacterized conserved protein			
	15922925	hypothetical protein STS255	No	1			2607936	2608094 +		Sulfolobus_tokodaii_7_uid57807	NC_003106	chromosome										
	15922926	hypothetical protein ST2590	No	9			2607956	2608921 -		Sulfolobus_tokodaii_7_uid57807	NC_003106	chromosome			arCOG09694	1	S		Uncharacterized conserved protein			
===									5	Sulfolobus_tokodaii_7_uid57807	NC_003106	chromosome										
	15922931	hypothetical protein ST2596	No	0			2613771	2614079 +		Sulfolobus_tokodaii_7_uid57807	NC_003106	chromosome										
	15922932	hypothetical protein ST2597	No	1			2614446	2614910 +		Sulfolobus_tokodaii_7_uid57807	NC_003106	chromosome			arCOG09695	1	S		Uncharacterized conserved protein			
	15922933	hypothetical protein ST2598	No	7			2615309	2616490 +		Sulfolobus_tokodaii_7_uid57807	NC_003106	chromosome			arCOG02772	2 (-)	C	NapH	Polyferredoxin			
	15922934	hypothetical protein ST2599	No	3			2616487	2617137 +		Sulfolobus_tokodaii_7_uid57807	NC_003106	chromosome										
	15922935	hypothetical protein STS256	Yes	0			2617104	2617283 -		Sulfolobus_tokodaii_7_uid57807	NC_003106	chromosome										
===									5	Sulfolobus_tokodaii_7_uid57807	NC_003106	chromosome										
	15922968	hypothetical protein STS262	No	2	YES		2658256	2658516 +		Sulfolobus_tokodaii_7_uid57807	NC_003106	chromosome										
	15922969	hypothetical protein ST2629	No	0	YES		2659565	2659951 +		Sulfolobus_tokodaii_7_uid57807	NC_003106	chromosome										
	15922970	hypothetical protein ST2630	No	3	YES		2661035	2661361 +		Sulfolobus_tokodaii_7_uid57807	NC_003106	chromosome			arCOG08406	1 (-)	S		Uncharacterized conserved protein			
	15922971	hypothetical protein ST2631	Yes	2	YES		2663482	2663826 -		Sulfolobus_tokodaii_7_uid57807	NC_003106	chromosome			arCOG09866	2	S		Uncharacterized conserved protein			
	15922972	degradation enzyme regulation protein DegQ	No	0	YES		2666356	2666532 +		Sulfolobus_tokodaii_7_uid57807	NC_003106	chromosome										
===									5	Thermococcus_4557_uid70841	NC_015865	chromosome										
	341581470	hypothetical protein GQS_01915	No	3			393831	394673 +		Thermococcus_4557_uid70841	NC_015865	chromosome										
	341581471	hypothetical protein GQS_01920	No	1			394724	394867 +		Thermococcus_4557_uid70841	NC_015865	chromosome										
	341581472	hypothetical protein GQS_01925	No	0			395008	395292 +		Thermococcus_4557_uid70841	NC_015865	chromosome			arCOG05252	3 (-)	F		Predicted secreted endonuclease distantly relate	6E-28	COG4741	Predicted secreted endonuclease distantl
	341581473	hypothetical protein GQS_01930	No	1			395375	395659 +		Thermococcus_4557_uid70841	NC_015865	chromosome										
	341581474	hypothetical protein GQS_01935	No	0			395650	396042 +		Thermococcus_4557_uid70841	NC_015865	chromosome										
===									5	Thermococcus_4557_uid70841	NC_015865	chromosome										
	341582312	hypothetical protein GQS_06145	Yes	2			1164115	1165533 -		Thermococcus_4557_uid70841	NC_015865	chromosome										

	341582313	hypothetical protein GQS_06150	Yes	1			1165656	1166735	-		Thermococcus_4557_uid70841	NC_015865	chromosome									
	341582314	isoleucyl-tRNA synthetase	No	0			1167243	1170446	+		Thermococcus_4557_uid70841	NC_015865	chromosome		arCOG00807	4 (-)	J	IleS	Isoleucyl-tRNA synthetase		COG0060	Isoleucyl-tRNA synthetase
	341582315	Kef-type K+ transport system membrane comp	No	9			1170495	1171508	-		Thermococcus_4557_uid70841	NC_015865	chromosome									
	341582316	hypothetical protein GQS_06165	No	6			1171510	1172013	-		Thermococcus_4557_uid70841	NC_015865	chromosome									
===										12	Thermococcus_4557_uid70841	NC_015865	chromosome									
	341582409	hypothetical protein GQS_06630	No	0			1267199	1267327	+		Thermococcus_4557_uid70841	NC_015865	chromosome		arCOG10094	2	S		Uncharacterized conserved protein			
	341582410	hypothetical protein GQS_06635	Yes	1			1267521	1270748	+		Thermococcus_4557_uid70841	NC_015865	chromosome		arCOG07710	3 (-)	S		Cell suface protein			
	341582411	hypothetical protein GQS_06640	No	5			1270763	1271284	+		Thermococcus_4557_uid70841	NC_015865	chromosome									
	341582412	hypothetical protein GQS_06645	No	3			1271299	1271646	+		Thermococcus_4557_uid70841	NC_015865	chromosome									
	341582413	hypothetical protein GQS_06650	No	0			1271725	1272390	+		Thermococcus_4557_uid70841	NC_015865	chromosome									
	341582414	hypothetical protein GQS_06655	No	5			1272395	1272916	+		Thermococcus_4557_uid70841	NC_015865	chromosome	clust_202								
	341582415	hypothetical protein GQS_06660	No	0			1273351	1273602	+		Thermococcus_4557_uid70841	NC_015865	chromosome									
	341582416	hypothetical protein GQS_06665	No	7			1273679	1274410	+		Thermococcus_4557_uid70841	NC_015865	chromosome									
	341582417	hypothetical protein GQS_06670	No	6			1274407	1274922	+		Thermococcus_4557_uid70841	NC_015865	chromosome									
	341582418	hypothetical protein GQS_06675	No	2			1275270	1275509	+		Thermococcus_4557_uid70841	NC_015865	chromosome									
	341582419	hypothetical protein GQS_06680	No	3			1275590	1275988	+		Thermococcus_4557_uid70841	NC_015865	chromosome									
	341582420	histidyl-tRNA synthetase	No	0			1276011	1277318	-		Thermococcus_4557_uid70841	NC_015865	chromosome		arCOG00404	1 (-)	J	HisS	Histidyl-tRNA synthetase	1E-116	COG0124	Histidyl-tRNA synthetase
===										6	Thermococcus_4557_uid70841	NC_015865	chromosome									
	341582509	5'-methylthioadenosine phosphorylase II	No	0			1365727	1366500	-		Thermococcus_4557_uid70841	NC_015865	chromosome		arCOG01327	1 (-)	F	Pnp	Purine nucleoside phosphorylase	1E-71	COG0005	Purine nucleoside phosphorylase
	341582510	hypothetical protein GQS_07135	No	2			1366607	1366825	-		Thermococcus_4557_uid70841	NC_015865	chromosome									
	341582511	hypothetical protein GQS_07140	No	1			1366841	1366972	-		Thermococcus_4557_uid70841	NC_015865	chromosome									
	341582512	hypothetical protein GQS_07145	No	6			1366969	1367643	-		Thermococcus_4557_uid70841	NC_015865	chromosome									
	341582513	hypothetical protein GQS_07150	No	8			1367615	1368295	-		Thermococcus_4557_uid70841	NC_015865	chromosome									
	341582514	hypothetical protein GQS_07155	Yes	1			1368286	1369197	-		Thermococcus_4557_uid70841	NC_015865	chromosome		arCOG08787	3 (-)	S		Uncharacterized conserved protein			
===										8	Thermococcus_4557_uid70841	NC_015865	chromosome									
	341582740	btpA family protein (btpA)	No	0			1578102	1578887	+		Thermococcus_4557_uid70841	NC_015865	chromosome		arCOG01982	1 (-)	R	SgcQ	Predicted TIM-barrel enzyme	4E-102	pfam03437	BtpA family. The BtpA protein is tightly
	341582741	hypothetical protein GQS_08310	No	1			1579018	1579143	+		Thermococcus_4557_uid70841	NC_015865	chromosome									
	341582742	hypothetical protein GQS_08315	Yes	0			1579149	1579676	+		Thermococcus_4557_uid70841	NC_015865	chromosome									
	341582743	hypothetical protein GQS_08320	No	0			1581114	1581296	-		Thermococcus_4557_uid70841	NC_015865	chromosome									
	341582744	hypothetical protein GQS_08325	No	1			1581392	1581532	+		Thermococcus_4557_uid70841	NC_015865	chromosome									
	341582745	hypothetical protein GQS_08330	Yes	0			1581673	1582383	+		Thermococcus_4557_uid70841	NC_015865	chromosome		arCOG06146	3	S		Uncharacterized conserved protein			
	341582746	hypothetical protein GQS_08335	No	0			1582625	1583074	+		Thermococcus_4557_uid70841	NC_015865	chromosome		arCOG09434	2	S		Uncharacterized conserved protein			
	341582747	phosphoglucosamine mutase	No	0			1583134	1584483	+		Thermococcus_4557_uid70841	NC_015865	chromosome		arCOG00767	3 (-)	G	{ManB}	Phosphomannomutase	6E-115	COG1109	Phosphomannomutase
===										11	Thermococcus_AM4_uid54735	NC_016051	chromosome									
	350525816	hypothetical protein TAM4_1738	No	0			771123	773627	+		Thermococcus_AM4_uid54735	NC_016051	chromosome	arCOG03256,a	arCOG03256	2 (-)	S		Uncharacterized conserved secreted protein, contains OB-fold domain			
	350525817	hypothetical protein TAM4_1520	Yes	1			773624	774538	+		Thermococcus_AM4_uid54735	NC_016051	chromosome		arCOG10066	9	S		Uncharacterized conserved protein			
	223478328	hypothetical protein TAM4_1748	Yes	1			774546	775502	+		Thermococcus_AM4_uid54735	NC_016051	chromosome		arCOG10027	12	S		Uncharacterized conserved protein			
	223478134	hypothetical protein TAM4_1554	No	1			775532	776464	+		Thermococcus_AM4_uid54735	NC_016051	chromosome		arCOG10066	9	S		Uncharacterized conserved protein			
	223478169	hypothetical protein TAM4_1589	Yes	0			776497	777426	+		Thermococcus_AM4_uid54735	NC_016051	chromosome		arCOG10066	9	S		Uncharacterized conserved protein			
	223478307	hypothetical protein TAM4_1727	Yes	1			777456	778415	+		Thermococcus_AM4_uid54735	NC_016051	chromosome									
	223478198	hypothetical membrane protein	Yes	0			778461	779309	+		Thermococcus_AM4_uid54735	NC_016051	chromosome		arCOG10028	3	S		Uncharacterized conserved protein			
	223478367	hypothetical protein TAM4_1787	Yes	1			779360	780289	+		Thermococcus_AM4_uid54735	NC_016051	chromosome		arCOG10066	9	S		Uncharacterized conserved protein			
	223478221	hypothetical protein TAM4_1641	Yes	1			780347	781324	+		Thermococcus_AM4_uid54735	NC_016051	chromosome		arCOG10027	12	S		Uncharacterized conserved protein			
	223478346	hypothetical protein TAM4_1766	Yes	1			781436	782371	+		Thermococcus_AM4_uid54735	NC_016051	chromosome									
	223478161	arylmalonate decarboxylase	No	0			782487	783200	-		Thermococcus_AM4_uid54735	NC_016051	chromosome		arCOG02004	2 (-)	Q		Maleate cis-trans isomerase	5E-96	COG3473	Maleate cis-trans isomerase
===										8	Thermococcus_AM4_uid54735	NC_016051	chromosome									
	223477878	hypothetical protein TAM4_220	No	1			1135871	1137058	+		Thermococcus_AM4_uid54735	NC_016051	chromosome		arCOG10650	2 (-)	S		Uncharacterized conserved protein			
	350525866	Hypothetical protein TAM4_207	No	1			1137315	1138295	+		Thermococcus_AM4_uid54735	NC_016051	chromosome									
	350525867	hypothetical protein TAM4_2406	Yes	3			1138292	1139953	+		Thermococcus_AM4_uid54735	NC_016051	chromosome									
	350525868	hypothetical protein TAM4_2407	No	0			1140265	1140453	+		Thermococcus_AM4_uid54735	NC_016051	chromosome									
	223477944	hypothetical protein TAM4_286	Yes	1			1140535	1142025	+		Thermococcus_AM4_uid54735	NC_016051	chromosome									
	350525869	Hypothetical protein TAM4_253	No	6			1142116	1142871	+		Thermococcus_AM4_uid54735	NC_016051	chromosome									
	223477739	hypothetical protein TAM4_81	Yes	1			1142868	1143320	+		Thermococcus_AM4_uid54735	NC_016051	chromosome									
	223477988	ABC-type copper transport ATP-binding protei	No	0			1143304	1143996	+		Thermococcus_AM4_uid54735	NC_016051	chromosome		arCOG00194	14 (-)	V	CcmA	ABC-type multidrug transport system, ATPase co	3E-32	COG1131	ABC-type multidrug transport system, ATP
===										17	Thermococcus_AM4_uid54735	NC_016051	chromosome									
	350525903	hypothetical protein TAM4_212	No	0			1390742	1391320	+		Thermococcus_AM4_uid54735	NC_016051	chromosome		arCOG06115	2 (-)	S		Uncharacterized conserved protein			
	223477820	Hypothetical protein TAM4_162	No	4			1391389	1391949	-		Thermococcus_AM4_uid54735	NC_016051	chromosome		arCOG10881	5	S		Uncharacterized conserved membrane protein			
	223478031	Hypothetical protein TAM4_373	No	2			1391995	1392258	-		Thermococcus_AM4_uid54735	NC_016051	chromosome									
	223477704	Hypothetical protein TAM4_46	No	4			1392522	1392929	-		Thermococcus_AM4_uid54735	NC_016051	chromosome		arCOG11004	1	S		Uncharacterized conserved protein			
	223477755	Hypothetical protein TAM4_97	No	2			1393426	1393689	-		Thermococcus_AM4_uid54735	NC_016051	chromosome									
	223478029	Hypothetical protein TAM4_371	Yes	5			1393897	1394412	-		Thermococcus_AM4_uid54735	NC_016051	chromosome		arCOG10881	5	S		Uncharacterized conserved membrane protein			
	223477679	Hypothetical protein TAM4_21	No	1			1394432	1394626	-		Thermococcus_AM4_uid54735	NC_016051	chromosome									
	223477774	Hypothetical protein TAM4_116	No	5			1394764	1395381	-		Thermococcus_AM4_uid54735	NC_016051	chromosome	clust_165								
	223477896	Hypothetical protein TAM4_238	No	3			1395385	1395777	-		Thermococcus_AM4_uid54735	NC_016051	chromosome									
	223477874	Hypothetical protein TAM4_216	No	5			1396588	1397094	-		Thermococcus_AM4_uid54735	NC_016051	chromosome		arCOG10881	5	S		Uncharacterized conserved membrane protein			
	223478032	Hypothetical protein TAM4_374	No	3			1397096	1397362	-		Thermococcus_AM4_uid54735	NC_016051	chromosome									
	223477800	Hypothetical protein TAM4_142	No	5			1397614	1398201	-		Thermococcus_AM4_uid54735	NC_016051	chromosome		arCOG10881	5	S		Uncharacterized conserved membrane protein			
	223477783	Hypothetical protein TAM4_125	No	1			1398293	1398415	-		Thermococcus_AM4_uid54735	NC_016051	chromosome									
	223477726	Hypothetical protein TAM4_68	No	1			1399005	1399556	-		Thermococcus_AM4_uid54735	NC_016051	chromosome									
	223477725	Hypothetical protein TAM4_67	No	5			1399525	1400130	-		Thermococcus_AM4_uid54735	NC_016051	chromosome		arCOG10881	5	S		Uncharacterized conserved membrane protein			
	223477672	Hypothetical protein TAM4_14	No	1			1400136	1402082	-		Thermococcus_AM4_uid54735	NC_016051	chromosome		arCOG02081	3 (-)	S		Predicted membrane protein			
	223477736	Hypothetical protein TAM4_78	No	6			1402085	1402720	-		Thermococcus_AM4_uid54735	NC_016051	chromosome	clust_161								
===										9	Thermococcus_AM4_uid54735	NC_016051	chromosome									
	223477595	oxidoreductase	No	0			1684756	1686219	-		Thermococcus_AM4_uid54735	NC_016051	chromosome		arCOG02231	1 (-)	R		Uncharacterized FAD-dependent dehydrogenase	6E-155	COG2509	Uncharacterized FAD-dependent dehydrogen
	350525951	hypothetical protein TAM4_2447	Yes	0			1686346	1686543	-		Thermococcus_AM4_uid54735	NC_016051	chromosome									
	350525952	hypothetical protein TAM4_2448	No	0			1686731	1686853	+		Thermococcus_AM4_uid54735	NC_016051	chromosome									
	223477398	hypothetical protein TAM4_2449	No	1			1688621	1688761	-		Thermococcus_AM4_uid54735	NC_016051	chromosome									
	350525953	hypothetical protein TAM4_1117	No	2			1690908	1691135	-		Thermococcus_AM4_uid54735	NC_016051	chromosome		arCOG07151	3	S		Uncharacterized conserved protein			
	350525954	hypothetical protein TAM4_1340	No	2			1693152	1693442	-		Thermococcus_AM4_uid54735	NC_016051	chromosome									
	223477515	hypothetical protein TAM4_2450	No	8			1694889	1695596	-		Thermococcus_AM4_uid54735	NC_016051	chromosome									
	350525955	hypothetical protein TAM4_1150	No	1			1695601	1696518	-		Thermococcus_AM4_uid54735	NC_016051	chromosome									
	223477454	hypothetical protein TAM4_1258	No	0			1701207	1703942	-		Thermococcus_AM4_uid54735	NC_016051	chromosome	arCOG07560,a	arCOG07560	2 (-)	S		Cell suface protein			
===										7	Thermococcus_AM4_uid54735	NC_016051	chromosome									
	223478780	hypothetical protein TAM4_777	No	2			2002378	2003019	+		Thermococcus_AM4_uid54735	NC_016051	chromosome		arCOG05733	1 (-)	S		Uncharacterized conserved protein			
	350525992	hypothetical protein TAM4_789	No	2			2003100	2003318	-		Thermococcus_AM4_uid54735	NC_016051	chromosome									
	223478706	hypothetical protein TAM4_703	Yes	2			2003688	2004113	+		Thermococcus_AM4_uid54735	NC_016051	chromosome		arCOG09677	3	S		Uncharacterized conserved protein			
	223479004	hypothetical protein TAM4_1001	Yes	2			2004119	2004547	+		Thermococcus_AM4_uid54735	NC_016051	chromosome		arCOG09677	3	S		Uncharacterized conserved protein			
	223478871	hypothetical protein TAM4_868	Yes	2			2004544	2004990	+		Thermococcus_AM4_uid54735	NC_016051	chromosome		arCOG09677	3	S		Uncharacterized conserved protein			
	223478735	Isoleucyl-tRNA synthetase	No	0			2005178	2008378	+		Thermococcus_AM4_uid54735	NC_016051	chromosome		arCOG00807	4 (-)	J	IleS	Isoleucyl-tRNA synthetase		COG0060	Isoleucyl-tRNA synthetase
	350525993	hypothetical protein TAM4_988	No	0			2009048	2009326	-		Thermococcus_AM4_uid54735	NC_016051	chromosome									
===										5	Thermococcus_CL1_uid168259	NC_018015	chromosome									
	390960240	hypothetical protein CL1_0065	No	0			57813	57995	-		Thermococcus_CL1_uid168259	NC_018015	chromosome									
	390960241	hypothetical protein CL1_0066	No	0			58109	58327	-		Thermococcus_CL1_uid168259	NC_018015	chromosome		arCOG07509	1 (-)	S		Uncharacterized conserved protein			
	390960242	hypothetical protein CL1_0067	No	0			58489	59853	+		Thermococcus_CL1_uid168259	NC_018015	chromosome									
	390960243	hypothetical protein CL1_0068	No	0			59857	60159	+		Thermococcus_CL1_uid168259	NC_018015	chromosome									
	390960244	hypothetical protein CL1_0069	No	0			60428	61897	+		Thermococcus_CL1_uid168259	NC_018015	chromosome							0.00006	COG0419	ATPase involved in DNA repair
===										12	Thermococcus_CL1_uid168259	NC_018015	chromosome									
	390960484	hypothetical protein containing RNase III domai	No	0			280875	281261	+		Thermococcus_CL1_uid168259	NC_018015	chromosome		arCOG05836	1 (-)	R		RNAse III distant homolog	2E-46	pfam11469	Protein of unknown function (DUF3204). T
	390960485	hypothetical protein CL1_0314	No	3			281292	281732	-		Thermococcus_CL1_uid168259	NC_018015	chromosome									
	390960486	hypothetical protein CL1_0315	No	5			282314	282856	-		Thermococcus_CL1_uid168259	NC_018015	chromosome	clust_165								
	390960487	hypothetical protein CL1_0316	No	5			282880	283407	-		Thermococcus_CL1_uid168259	NC_018015	chromosome									
	390960488	hypothetical protein CL1_0317	No	0			283414	284001	-		Thermococcus_CL1_uid168259	NC_018015	chromosome									
	390960489	hypothetical protein CL1_0318	Yes	4			284040	284444	-		Thermococcus_CL1_uid168259	NC_018015	chromosome									
	390960490	hypothetical protein CL1_0319	No	0			284456	284905	-		Thermococcus_CL1_uid168259	NC_018015	chromosome									
	390960491	hypothetical protein CL1_0320	No	0			285754	286110	-		Thermococcus_CL1_uid168259	NC_018015	chromosome									
	390960492	hypothetical protein CL1_0321	No	4			286160	286585	-		Thermococcus_CL1_uid168259	NC_018015	chromosome									
	390960493	hypothetical protein CL1_0322	No	3			286763	287110	-		Thermococcus_CL1_uid168259	NC_018015	chromosome									
	390960494	hypothetical protein CL1_0323	No	4			287242	287691	-		Thermococcus_CL1_uid168259	NC_018015	chromosome									
	390960495	hypothetical protein CL1_0324	No	0			287698	290907	-		Thermococcus_CL1_uid168259	NC_018015	chromosome		arCOG05807	1 (-)	S		Uncharacterized conserved protein			
===										6	Thermococcus_CL1_uid168259	NC_018015	chromosome									
	390960681	hypothetical protein CL1_0513	No	0			479837	480754	-		Thermococcus_CL1_uid168259	NC_018015	chromosome									
	390960682	hypothetical protein CL1_0514	No	0			481011	481358	-		Thermococcus_CL1_uid168259	NC_018015	chromosome	clust_306								
	390960683	hypothetical protein CL1_0515	No	1			481355	481603	-		Thermococcus_CL1_uid168259	NC_018015	chromosome									
	390960684	hypothetical protein CL1_0516	No	0			481600	481872	-		Thermococcus_CL1_uid168259	NC_018015	chromosome		arCOG03881	5 (-)	S		Uncharacterized conserved protein			
	390960685	hypothetical protein CL1_0517	No	1			481869	482069	-		Thermococcus_CL1_uid168259	NC_018015	chromosome	clust_454								
	390960686	hypothetical protein CL1_0518	No	1			482177	483058	-		Thermococcus_CL1_uid168259	NC_018015	chromosome	clust_111								
===										5	Thermococcus_CL1_uid168259	NC_018015	chromosome									
	390961227	hypothetical protein CL1_1064	No	0			1032896	1033195	+		Thermococcus_CL1_uid168259	NC_018015	chromosome									
	390961228	hypothetical protein CL1_1065	No	0			1033167	1033559	+		Thermococcus_CL1_uid168259	NC_018015	chromosome									
	390961229	hypothetical protein CL1_1066	No	0			1033663	1034217	-		Thermococcus_CL1_uid168259	NC_018015	chromosome		arCOG06576	5 (-)	V		HEPN domain containing protein			
	390961230	hypothetical protein CL1_1067	No	0			1034231	1034656	-		Thermococcus_CL1_uid168259	NC_018015	chromosome									
	390961231	hypothetical protein CL1_1068	No	0			1034745	1035332	-		Thermococcus_CL1_uid168259	NC_018015	chromosome									
===										7	Thermococcus_CL1_uid168259	NC_018015	chromosome									
	390961270	transcription regulator, AsnC family 2	No	0			1077971	1078423	+		Thermococcus_CL1_uid168259	NC_018015	chromosome		arCOG01580	1 (-)	K	Lrp	Transcriptional regulator, IclR family	9E-23	COG1522	Transcriptional regulators
	390961271	hypothetical protein CL1_1108	Yes	1			1078522	1078728	-		Thermococcus_CL1_uid168259	NC_018015	chromosome									
	390961272	hypothetical protein CL1_1109	No	0			1078922	1079557	-		Thermococcus_CL1_uid168259	NC_018015	chromosome									
	390961273	hypothetical protein CL1_1110	No	1			1079662	1079901	-		Thermococcus_CL1_uid168259	NC_018015	chromosome									
	390961274	hypothetical protein CL1_1111	Yes	1			1079927	1080877	-		Thermococcus_CL1_uid168259	NC_018015	chromosome		arCOG10027	12	S		Uncharacterized conserved protein			
	390961275	hypothetical protein CL1_1112	No	0			1080902	1081654	-		Thermococcus_CL1_uid168259	NC_018015	chromosome		arCOG01310	1 (-)	S		Uncharacterized conserved protein			
	390961276	hypothetical protein CL1_1113	Yes	1			1082326	1083273	-		Thermococcus_CL1_uid168259	NC_018015	chromosome									
===										5	Thermococcus_barophilus_MP_uid54733	NC_014804	chromosome									
	315229823	hypothetical protein TERMP_00058	No	1			50169	51674	+		Thermococcus_barophilus_MP_uid54733	NC_014804	chromosome		arCOG10097	1	S		Uncharacterized conserved protein	0.000008	COG1196	Chromosome segregation ATPases
	315229824	hypothetical protein TERMP_00059	Yes	1			51688	52674	+		Thermococcus_barophilus_MP_uid54733	NC_014804	chromosome		arCOG02491	7 (-)	S		WD40 repeats containing protein	0.00002	pfam13360	PQQ-like domain. This domain contains se
	315229825	hypothetical protein TERMP_00060	No	0			52700	53164	+		Thermococcus_barophilus_MP_uid54733	NC_014804	chromosome									
	315229826	hypothetical protein TERMP_00061	Yes	0			53379	54581	+		Thermococcus_barophilus_MP_uid54733	NC_014804	chromosome									
	315229827	hypothetical protein TERMP_00062	No	1			54621	55532	+		Thermococcus_barophilus_MP_uid54733	NC_014804	chromosome		arCOG10107	1	S		Uncharacterized conserved protein			
===										5	Thermococcus_barophilus_MP_uid54733	NC_014804	chromosome									
	315231510	hypothetical protein TERMP_01748	Yes	6			1540272	1541465	-		Thermococcus_barophilus_MP_uid54733	NC_014804	chromosome		arCOG11354	2	S		Uncharacterized conserved protein			
	315231511	hypothetical protein TERMP_01749	No	0			1541452	1541613	-		Thermococcus_barophilus_MP_uid54733	NC_014804	chromosome		arCOG11354	2	S		Uncharacterized conserved protein			
	315231512	hypothetical protein TERMP_01750	Yes	6			1541808	1542845	-		Thermococcus_barophilus_MP_uid54733	NC_014804	chromosome									
	315231513	hypothetical protein TERMP_01751	No	0			1542904	1543365	-		Thermococcus_barophilus_MP_uid54733	NC_014804	chromosome		arCOG05087	1 (-)	S		Uncharacterized conserved protein with histone f	2E-30	pfam09123	Domain of unknown function (DUF1931). Me
	315231514	hypothetical protein TERMP_01752	No	4			1543434	1543757	-		Thermococcus_barophilus_MP_uid54733	NC_014804	chromosome									
===										5	Thermococcus_barophilus_MP_uid54733	NC_014804	chromosome									
	315231615	hypothetical protein TERMP_01853	No	0			1640473	1641075	-		Thermococcus_barophilus_MP_uid54733	NC_014804	chromosome									
	315231616	hypothetical protein TERMP_01854	No	0			1641130	1641495	-		Thermococcus_barophilus_MP_uid54733	NC_014804	chromosome									
	315231617	hypothetical protein TERMP_01855	No	0			1641550	1644723	-		Thermococcus_barophilus_MP_uid54733	NC_014804	chromosome		arCOG00889	6 (-)	L		Adenine-specific DNA methylase containing a Zn-ribbon		COG1743	Adenine-specific DNA methylase containin
	315231618	hypothetical protein TERMP_01856	No	5			1644800	1645423	+		Thermococcus_barophilus_MP_uid54733	NC_014804	chromosome									
	315231619	hypothetical protein TERMP_01857	Yes	2			1645486	1645791	+		Thermococcus_barophilus_MP_uid54733	NC_014804	chromosome									
===										6	Thermococcus_barophilus_MP_uid54733	NC_015471	plasmid pTBMP1									
	331746804	hypothetical protein TERMP_02226	No	0		plasmid	12275	12766	+		Thermococcus_barophilus_MP_uid54733	NC_015471	plasmid pTBMP1		arCOG11296	2	S		Uncharacterized conserved protein	0.000002	COG1196	Chromosome segregation ATPases
	331746805	hypothetical protein TERMP_02227	No	0		plasmid	13148	13735	+		Thermococcus_barophilus_MP_uid54733	NC_015471	plasmid pTBMP1									
	331746806	hypothetical protein TERMP_02228	No	0		plasmid	13846	14568	+		Thermococcus_barophilus_MP_uid54733	NC_015471	plasmid pTBMP1		arCOG03808	1 (-)	R		KaiC-like ATPase			
	331746807	hypothetical protein TERMP_02229	No	0		plasmid	14732	15103	-		Thermococcus_barophilus_MP_uid54733	NC_015471	plasmid pTBMP1		arCOG10970	3	S		Uncharacterized conserved protein			
	331746808	hypothetical protein TERMP_02230	No	0		plasmid	15451	16050	+		Thermococcus_barophilus_MP_uid54733	NC_015471	plasmid pTBMP1									
	331746809	hypothetical protein TERMP_02231	No	0		plasmid	16135	17061	-		Thermococcus_barophilus_MP_uid54733	NC_015471	plasmid pTBMP1									
===										6	Thermococcus_barophilus_MP_uid54733	NC_015471	plasmid pTBMP1									
	331746820	hypothetical protein TERMP_02242	No	0		plasmid	29579	30127	-		Thermococcus_barophilus_MP_uid54733	NC_015471	plasmid pTBMP1		arCOG02408	2 (-)	S		Uncharacterized conserved protein	0.0000002	pfam01936	NYN domain. These domains are found in t
	331746821	hypothetical protein TERMP_02243	No	0		plasmid	30707	31078	-		Thermococcus_barophilus_MP_uid54733	NC_015471	plasmid pTBMP1									
	331746822	hypothetical protein TERMP_02244	No	0		plasmid	31102	31494	-		Thermococcus_barophilus_MP_uid54733	NC_015471	plasmid pTBMP1									
	331746823	hypothetical protein TERMP_02245	No	0		plasmid	31643	33133	-		Thermococcus_barophilus_MP_uid54733	NC_015471	plasmid pTBMP1		arCOG10606	2	S		Uncharacterized conserved protein			
	331746824	hypothetical protein TERMP_02246	No	0		plasmid	33204	33473	+		Thermococcus_barophilus_MP_uid54733	NC_015471	plasmid pTBMP1	clust_372								
	331746825	Transcription initiation factor B	No	0		plasmid	33616	34521	+		Thermococcus_barophilus_MP_uid54733	NC_015471	plasmid pTBMP1		arCOG01981	8 (-)	K	SUA7	Transcription initiation factor TFIIB, Brf1 subunit/Tra	1E-109	COG1405	Transcription initiation factor TFIIIB,
===										7	Thermococcus_barophilus_MP_uid54733	NC_015471	plasmid pTBMP1									
	331746841	hypothetical protein TERMP_02263	No	0		plasmid	49536	49895	-		Thermococcus_barophilus_MP_uid54733	NC_015471	plasmid pTBMP1		arCOG01009	8 (-)	V		Ribbon-helix-helix protein, copG family	6E-10	COG3609	Predicted transcriptional regulators con
	331746842	hypothetical protein TERMP_02264	No	0		plasmid	50205	50654	+		Thermococcus_barophilus_MP_uid54733	NC_015471	plasmid pTBMP1									
	331746843	hypothetical protein TERMP_02265	No	0		plasmid	51074	51568	+		Thermococcus_barophilus_MP_uid54733	NC_015471	plasmid pTBMP1									
	331746844	hypothetical protein TERMP_02266	No	0		plasmid	51598	51828	+		Thermococcus_barophilus_MP_uid54733	NC_015471	plasmid pTBMP1									
	331746845	hypothetical protein TERMP_02267	No	0		plasmid	51825	52196	+		Thermococcus_barophilus_MP_uid54733	NC_015471	plasmid pTBMP1									
	331746846	hypothetical protein TERMP_02268	No	2		plasmid	52197	53201	-		Thermococcus_barophilus_MP_uid54733	NC_015471	plasmid pTBMP1		arCOG06149	1 (-)	E		Transglutaminase-like enzyme, putative cysteine protease			
	331746847	hypothetical protein TERMP_02269	No	2		plasmid	53185	53658	-		Thermococcus_barophilus_MP_uid54733	NC_015471	plasmid pTBMP1									
===										6	Thermococcus_gammatolerans_EJ3_uid59389	NC_012804	chromosome									
	240102352	hypothetical protein TGAM_0294	No	2			278590	278937	-		Thermococcus_gammatolerans_EJ3_uid59389	NC_012804	chromosome									
	240102353	hypothetical protein TGAM_0295	No	5			278924	279448	-		Thermococcus_gammatolerans_EJ3_uid59389	NC_012804	chromosome									
	240102354	hypothetical protein TGAM_0296	No	8			279662	280342	-		Thermococcus_gammatolerans_EJ3_uid59389	NC_012804	chromosome							0.00009	pfam01757	Acyltransferase family. This family incl
	240102355	hypothetical protein TGAM_0297	No	0			280349	281350	-		Thermococcus_gammatolerans_EJ3_uid59389	NC_012804	chromosome		arCOG07710	3 (-)	S		Cell suface protein			
	240102356	hypothetical protein TGAM_0298	No	7			281456	282022	-		Thermococcus_gammatolerans_EJ3_uid59389	NC_012804	chromosome		arCOG07151	3	S		Uncharacterized conserved protein			
	240102357	hypothetical protein TGAM_0299	No	5			282072	282620	-		Thermococcus_gammatolerans_EJ3_uid59389	NC_012804	chromosome									
===										12	Thermococcus_gammatolerans_EJ3_uid59389	NC_012804	chromosome									
	240102723	hypothetical protein TGAM_0666	No	2	YES	integrated_ele	635602	636390	-		Thermococcus_gammatolerans_EJ3_uid59389	NC_012804	chromosome		arCOG10043	3	S		Uncharacterized conserved protein			
	240102724	transcription regulator	No	0	YES	integrated_ele	636574	636954	+		Thermococcus_gammatolerans_EJ3_uid59389	NC_012804	chromosome		arCOG03936	6 (-)	K	AbrB	Transcriptional regulator AbrB			
	240102725	hypothetical protein TGAM_0668	No	0	YES	integrated_ele	637432	638376	+		Thermococcus_gammatolerans_EJ3_uid59389	NC_012804	chromosome		arCOG07161	3	S		Uncharacterized conserved protein			
	240102726	hypothetical protein TGAM_0669	No	0	YES	integrated_ele	638373	638618	+		Thermococcus_gammatolerans_EJ3_uid59389	NC_012804	chromosome		arCOG10044	4	S		Uncharacterized conserved protein			
	240102727	hypothetical protein TGAM_0670	No	0	YES	integrated_ele	638680	639066	+		Thermococcus_gammatolerans_EJ3_uid59389	NC_012804	chromosome									
	240102728	hypothetical protein TGAM_0671	No	0	YES	integrated_ele	639057	639455	+		Thermococcus_gammatolerans_EJ3_uid59389	NC_012804	chromosome									
	240102729	hypothetical protein TGAM_0672	No	0	YES	integrated_ele	639455	639979	+		Thermococcus_gammatolerans_EJ3_uid59389	NC_012804	chromosome		arCOG07102	9 (-)	L		PD-(D/E)XK superfamily nuclease			
	240102730	hypothetical protein TGAM_0673	No	0	YES	integrated_ele	639986	640324	+		Thermococcus_gammatolerans_EJ3_uid59389	NC_012804	chromosome		arCOG10045	2	S		Uncharacterized conserved protein			
	240102731	hypothetical protein TGAM_0674	No	0	YES	integrated_ele	640511	640999	+		Thermococcus_gammatolerans_EJ3_uid59389	NC_012804	chromosome									
	240102732	hypothetical protein TGAM_0675	No	0	YES	integrated_ele	641475	641939	+		Thermococcus_gammatolerans_EJ3_uid59389	NC_012804	chromosome									
	240102733	hypothetical protein TGAM_0676	No	0	YES	integrated_ele	641798	642154	+		Thermococcus_gammatolerans_EJ3_uid59389	NC_012804	chromosome		arCOG10046	1	S		Uncharacterized conserved protein			
	240102734	CRISPR-associated protein, Cas4 family	No	0	YES	integrated_ele	642576	643244	-		Thermococcus_gammatolerans_EJ3_uid59389	NC_012804	chromosome		arCOG00790	6 (-)	V	Cas4	CRISPR-associated protein Cas4, RecB family ex	2E-12	COG1468	CRISPR-associated protein Cas4 (RecB fam
===										9	Thermococcus_gammatolerans_EJ3_uid59389	NC_012804	chromosome									
	240102738	hypothetical protein TGAM_0681	No	2			646648	647214	-		Thermococcus_gammatolerans_EJ3_uid59389	NC_012804	chromosome									
	240102739	valyl-tRNA synthetase	No	0			647312	649978	-		Thermococcus_gammatolerans_EJ3_uid59389	NC_012804	chromosome	arCOG00405,a	arCOG00405	1 (-)	J	GRS1	Glycyl-tRNA synthetase (class II)		COG0525	Valyl-tRNA synthetase
	240102740	hypothetical protein TGAM_0683	No	4			650173	650670	-		Thermococcus_gammatolerans_EJ3_uid59389	NC_012804	chromosome									
	240102741	hypothetical protein TGAM_0684	No	0			650751	651521	-		Thermococcus_gammatolerans_EJ3_uid59389	NC_012804	chromosome		arCOG10047	4	S		Uncharacterized conserved protein			
	240102742	hypothetical protein TGAM_0685	No	0			651577	652482	-		Thermococcus_gammatolerans_EJ3_uid59389	NC_012804	chromosome		arCOG10047	4	S		Uncharacterized conserved protein			
	240102743	hypothetical protein TGAM_0686	No	0			652400	653188	-		Thermococcus_gammatolerans_EJ3_uid59389	NC_012804	chromosome		arCOG10047	4	S		Uncharacterized conserved protein			
	240102744	hypothetical protein TGAM_0687	No	2			653230	654288	-		Thermococcus_gammatolerans_EJ3_uid59389	NC_012804	chromosome		arCOG10048	5	S		Uncharacterized conserved protein			
	240102745	methyltransferase C-terminus	No	0			654303	654563	-		Thermococcus_gammatolerans_EJ3_uid59389	NC_012804	chromosome									
	240102746	methyltransferase N-terminus	No	0			654478	654768	-		Thermococcus_gammatolerans_EJ3_uid59389	NC_012804	chromosome		arCOG01791	2 (-)	Q		SAM-dependent methyltransferase	0.0000005	pfam12847	Methyltransferase domain. Protein in thi
===										15	Thermococcus_gammatolerans_EJ3_uid59389	NC_012804	chromosome									
	240103522	ADP-specific phosphofructokinase	No	0			1375601	1376983	-		Thermococcus_gammatolerans_EJ3_uid59389	NC_012804	chromosome		arCOG03370	1 (-)	G		Archaeal ADP-dependent phosphofructokinase/g	3E-168	pfam04587	ADP-specific Phosphofructokinase/Glucoki
	240103523	hypothetical protein TGAM_1466	No	0			1377193	1379715	+		Thermococcus_gammatolerans_EJ3_uid59389	NC_012804	chromosome		arCOG03256	2	S		Uncharacterized conserved secreted protein, contains OB-fold domain			
	240103524	hypothetical protein TGAM_1467	No	1			1379628	1380620	+		Thermococcus_gammatolerans_EJ3_uid59389	NC_012804	chromosome		arCOG10066	9	S		Uncharacterized conserved protein			
	240103525	hypothetical protein TGAM_1468	No	1			1380464	1381621	+		Thermococcus_gammatolerans_EJ3_uid59389	NC_012804	chromosome		arCOG10027	12	S		Uncharacterized conserved protein			
	240103526	hypothetical protein TGAM_1469	Yes	1			1381654	1382637	+		Thermococcus_gammatolerans_EJ3_uid59389	NC_012804	chromosome		arCOG10027	12	S		Uncharacterized conserved protein			
	240103527	hypothetical protein TGAM_1470	No	1			1382519	1383664	+		Thermococcus_gammatolerans_EJ3_uid59389	NC_012804	chromosome		arCOG10027	12	S		Uncharacterized conserved protein			
	240103528	hypothetical protein TGAM_1471	No	1			1383642	1384631	+		Thermococcus_gammatolerans_EJ3_uid59389	NC_012804	chromosome		arCOG10066	9	S		Uncharacterized conserved protein			
	240103529	hypothetical protein TGAM_1472	Yes	0			1384639	1385622	+		Thermococcus_gammatolerans_EJ3_uid59389	NC_012804	chromosome		arCOG10027	12	S		Uncharacterized conserved protein			
	240103530	hypothetical protein TGAM_1473	Yes	1			1385686	1386540	+		Thermococcus_gammatolerans_EJ3_uid59389	NC_012804	chromosome		arCOG10028	3	S		Uncharacterized conserved protein			
	240103531	hypothetical protein TGAM_1474	Yes	1			1386585	1387487	+		Thermococcus_gammatolerans_EJ3_uid59389	NC_012804	chromosome		arCOG10066	9	S		Uncharacterized conserved protein			
	240103532	hypothetical protein TGAM_1475	No	1			1387536	1388504	+		Thermococcus_gammatolerans_EJ3_uid59389	NC_012804	chromosome		arCOG10066	9	S		Uncharacterized conserved protein			
	240103533	hypothetical protein TGAM_1476	Yes	0			1388610	1389509	+		Thermococcus_gammatolerans_EJ3_uid59389	NC_012804	chromosome		arCOG10027	12	S		Uncharacterized conserved protein			
	240103534	hypothetical protein TGAM_1477	Yes	1			1389580	1390551	+		Thermococcus_gammatolerans_EJ3_uid59389	NC_012804	chromosome		arCOG10027	12	S		Uncharacterized conserved protein			
	240103535	hypothetical protein TGAM_1478	Yes	1			1390585	1391583	+		Thermococcus_gammatolerans_EJ3_uid59389	NC_012804	chromosome		arCOG10027	12	S		Uncharacterized conserved protein			
	240103536	Maleate cis-trans isomerase	No	0			1391814	1392527	-		Thermococcus_gammatolerans_EJ3_uid59389	NC_012804	chromosome		arCOG02004	2 (-)	Q		Maleate cis-trans isomerase	3E-96	COG3473	Maleate cis-trans isomerase
===										13	Thermococcus_kodakarensis_KOD1_uid58225	NC_006624	chromosome									
	57640010	hypothetical protein TK0075	Yes	1		plasmid	66046	67383	+		Thermococcus_kodakarensis_KOD1_uid58225	NC_006624	chromosome									
	57640011	subtilisin-like serine protease precursor	Yes	0		plasmid	67400	68974	+		Thermococcus_kodakarensis_KOD1_uid58225	NC_006624	chromosome		arCOG00702	6 (-)	O	AprE	Subtilisin-like serine protease	1E-39	COG1404	Subtilisin-like serine proteases
	57640012	hypothetical protein TK0077	No	1		plasmid	68971	69114	+		Thermococcus_kodakarensis_KOD1_uid58225	NC_006624	chromosome									
	57640013	hypothetical protein TK0078	No	0		plasmid	69118	69375	+		Thermococcus_kodakarensis_KOD1_uid58225	NC_006624	chromosome	clust_386								
	57640014	hypothetical protein TK0079	No	4		plasmid	69404	69796	+		Thermococcus_kodakarensis_KOD1_uid58225	NC_006624	chromosome									
	57640015	hypothetical protein TK0080	No	7		plasmid	69796	70512	+		Thermococcus_kodakarensis_KOD1_uid58225	NC_006624	chromosome									
	57640016	hypothetical protein TK0081	Yes	1		plasmid	70514	71113	+		Thermococcus_kodakarensis_KOD1_uid58225	NC_006624	chromosome									
	57640017	hypothetical protein TK0082	No	1		plasmid	71118	71246	+		Thermococcus_kodakarensis_KOD1_uid58225	NC_006624	chromosome									
	57640018	hypothetical protein TK0083	Yes	4		plasmid	71243	71563	+		Thermococcus_kodakarensis_KOD1_uid58225	NC_006624	chromosome									
	57640019	hypothetical protein TK0084	No	0		plasmid	71565	71705	+		Thermococcus_kodakarensis_KOD1_uid58225	NC_006624	chromosome									
	57640020	hypothetical protein TK0085	No	2		plasmid	71689	72594	+		Thermococcus_kodakarensis_KOD1_uid58225	NC_006624	chromosome									
	57640021	ArsR family transcriptional regulator	No	0		plasmid	72765	73340	+		Thermococcus_kodakarensis_KOD1_uid58225	NC_006624	chromosome									
	57640022	hypothetical protein TK0087	No	0	YES	plasmid	73337	74644	+		Thermococcus_kodakarensis_KOD1_uid58225	NC_006624	chromosome		arCOG00281	4 (-)	R		Predicted ATPase			
===										15	Thermococcus_kodakarensis_KOD1_uid58225	NC_006624	chromosome									
	57640024	hypothetical protein TK0089	Yes	1	YES	plasmid	75754	76026	+		Thermococcus_kodakarensis_KOD1_uid58225	NC_006624	chromosome									
	57640025	hypothetical protein TK0090	Yes	1	YES	plasmid	76023	77459	+		Thermococcus_kodakarensis_KOD1_uid58225	NC_006624	chromosome									
	57640026	hypothetical protein TK0091	Yes	1	YES	plasmid	77703	79046	+		Thermococcus_kodakarensis_KOD1_uid58225	NC_006624	chromosome									
	57640027	hypothetical protein TK0092	No	1	YES	plasmid	79043	79249	+		Thermococcus_kodakarensis_KOD1_uid58225	NC_006624	chromosome									
	57640028	hypothetical protein TK0093	No	2	YES	plasmid	79243	79497	+		Thermococcus_kodakarensis_KOD1_uid58225	NC_006624	chromosome									
	57640029	SpoVT/AbrB family transcription regulator	No	0	YES	plasmid	79753	80130	+		Thermococcus_kodakarensis_KOD1_uid58225	NC_006624	chromosome		arCOG03936	6 (-)	K	AbrB	Transcriptional regulator AbrB			
	57640030	hypothetical protein TK0095	No	0	YES	plasmid	80256	80429	+		Thermococcus_kodakarensis_KOD1_uid58225	NC_006624	chromosome									
	57640031	MCM2/3/5 family DNA replication licensing fact	No	0		plasmid	80433	83177	+		Thermococcus_kodakarensis_KOD1_uid58225	NC_006624	chromosome		arCOG00439	14 (-)	L	MCM2	Predicted ATPase involved in replication control,	3E-105	COG1241	Predicted ATPase involved in replication
	57640032	hypothetical protein TK0097	No	0		plasmid	83184	83435	+		Thermococcus_kodakarensis_KOD1_uid58225	NC_006624	chromosome		arCOG10044	4	S		Uncharacterized conserved protein			
	57640033	hypothetical protein TK0098	No	0		plasmid	83432	83629	+		Thermococcus_kodakarensis_KOD1_uid58225	NC_006624	chromosome									
	57640034	hypothetical protein TK0099	No	0		plasmid	83733	83912	+		Thermococcus_kodakarensis_KOD1_uid58225	NC_006624	chromosome									
	57640035	hypothetical protein TK0100	No	0		plasmid	83909	84268	+		Thermococcus_kodakarensis_KOD1_uid58225	NC_006624	chromosome									
	57640036	hypothetical protein TK0101	No	0		plasmid	84265	84441	+		Thermococcus_kodakarensis_KOD1_uid58225	NC_006624	chromosome									
	57640037	hypothetical protein TK0102	No	0		plasmid	84441	85019	+		Thermococcus_kodakarensis_KOD1_uid58225	NC_006624	chromosome		arCOG07102	9 (-)	L		PD-(D/E)XK superfamily nuclease			
	57640038	hypothetical protein TK0103	No	1		plasmid	85027	85341	+		Thermococcus_kodakarensis_KOD1_uid58225	NC_006624	chromosome									
===										5	Thermococcus_kodakarensis_KOD1_uid58225	NC_006624	chromosome									
	57640331	hypothetical protein TK0396	No	0	YES	plasmid	332458	332742	+		Thermococcus_kodakarensis_KOD1_uid58225	NC_006624	chromosome	clust_365								
	57640332	hypothetical protein TK0397	No	1	YES	plasmid	332751	333722	-		Thermococcus_kodakarensis_KOD1_uid58225	NC_006624	chromosome		arCOG08597	2	S		Uncharacterized conserved protein	0.000000002	COG1196	Chromosome segregation ATPases
	57640333	hypothetical protein TK0398	Yes	1	YES	plasmid	333809	334951	+		Thermococcus_kodakarensis_KOD1_uid58225	NC_006624	chromosome		arCOG09822	1 (-)	G	SKN1	Beta-glucanase/Beta-glucan synthetase			
	57640334	hypothetical protein TK0399	No	0	YES	plasmid	335074	335670	+		Thermococcus_kodakarensis_KOD1_uid58225	NC_006624	chromosome									
	57640335	hypothetical protein TK0400	No	0	YES	plasmid	335639	336118	+		Thermococcus_kodakarensis_KOD1_uid58225	NC_006624	chromosome									
===										16	Thermococcus_kodakarensis_KOD1_uid58225	NC_006624	chromosome									
	57640342	hypothetical protein TK0407	No	0	YES	plasmid	340152	340544	-		Thermococcus_kodakarensis_KOD1_uid58225	NC_006624	chromosome									
	57640343	hypothetical protein TK0408	No	0	YES	plasmid	340726	341310	-		Thermococcus_kodakarensis_KOD1_uid58225	NC_006624	chromosome									
	57640344	SpoVT/AbrB family transcriptional regulator	No	0	YES	plasmid	341564	342001	+		Thermococcus_kodakarensis_KOD1_uid58225	NC_006624	chromosome		arCOG03936	6 (-)	K	AbrB	Transcriptional regulator AbrB	0.0001	COG2002	Regulators of stationary/sporulation gen
	57640345	hypothetical protein TK0410	No	0	YES	plasmid	342439	343467	+		Thermococcus_kodakarensis_KOD1_uid58225	NC_006624	chromosome		arCOG07161	3	S		Uncharacterized conserved protein	0.000009	COG1196	Chromosome segregation ATPases
	57640346	hypothetical protein TK0411	No	0	YES	plasmid	343460	343897	+		Thermococcus_kodakarensis_KOD1_uid58225	NC_006624	chromosome		arCOG10044	4	S		Uncharacterized conserved protein			
	57640347	hypothetical protein TK0412	No	0	YES	plasmid	343894	344082	+		Thermococcus_kodakarensis_KOD1_uid58225	NC_006624	chromosome		arCOG07713	1	S		Uncharacterized conserved protein			
	57640348	hypothetical protein TK0413	No	0	YES	plasmid	344087	344407	+		Thermococcus_kodakarensis_KOD1_uid58225	NC_006624	chromosome									
	57640349	hypothetical protein TK0414	No	0	YES	plasmid	344404	344595	+		Thermococcus_kodakarensis_KOD1_uid58225	NC_006624	chromosome									
	57640350	hypothetical protein TK0415	No	0	YES	plasmid	344592	344903	+		Thermococcus_kodakarensis_KOD1_uid58225	NC_006624	chromosome	clust_337								
	57640351	hypothetical protein TK0416	No	0	YES	plasmid	344903	345478	+		Thermococcus_kodakarensis_KOD1_uid58225	NC_006624	chromosome		arCOG07102	9 (-)	L		PD-(D/E)XK superfamily nuclease			
	57640352	hypothetical protein TK0417	No	2	YES	plasmid	345486	345746	+		Thermococcus_kodakarensis_KOD1_uid58225	NC_006624	chromosome									
	57640353	hypothetical protein TK0418	No	0	YES	plasmid	345743	345997	+		Thermococcus_kodakarensis_KOD1_uid58225	NC_006624	chromosome									
	57640354	hypothetical protein TK0419	No	0	YES	plasmid	346012	346362	+		Thermococcus_kodakarensis_KOD1_uid58225	NC_006624	chromosome									
	57640355	hypothetical protein TK0420	No	0	YES	plasmid	346534	346887	+		Thermococcus_kodakarensis_KOD1_uid58225	NC_006624	chromosome		arCOG07101	4	S		Uncharacterized conserved protein			
	57640356	integrase, N-fragment	No	0	YES	plasmid	346893	347273	+		Thermococcus_kodakarensis_KOD1_uid58225	NC_006624	chromosome		arCOG07843	4	S		Uncharacterized conserved protein			
	57640357	tRNA m1G methyltransferase	No	0		plasmid	347357	348469	+		Thermococcus_kodakarensis_KOD1_uid58225	NC_006624	chromosome		arCOG00967	1 (-)	J		tRNA m(1)G methyltransferase	6E-87	COG2419	Uncharacterized conserved protein
===										6	Thermococcus_kodakarensis_KOD1_uid58225	NC_006624	chromosome									
	57640510	integrase, N-fragment	No	1		plasmid	498959	499552	-		Thermococcus_kodakarensis_KOD1_uid58225	NC_006624	chromosome		arCOG07843	4	S		Uncharacterized conserved protein			
	57640511	hypothetical protein TK0576	No	0		plasmid	499603	499956	-		Thermococcus_kodakarensis_KOD1_uid58225	NC_006624	chromosome		arCOG07101	4	S		Uncharacterized conserved protein			
	57640512	hypothetical protein TK0577	No	0		plasmid	500135	500362	-		Thermococcus_kodakarensis_KOD1_uid58225	NC_006624	chromosome									
	57640513	hypothetical protein TK0578	No	0		plasmid	500370	500945	-		Thermococcus_kodakarensis_KOD1_uid58225	NC_006624	chromosome		arCOG07102	9 (-)	L		PD-(D/E)XK superfamily nuclease			
	57640514	hypothetical protein TK0579	No	0		plasmid	500945	501259	-		Thermococcus_kodakarensis_KOD1_uid58225	NC_006624	chromosome	clust_337								
	57640515	hypothetical protein TK0580	No	0		plasmid	501256	501480	-		Thermococcus_kodakarensis_KOD1_uid58225	NC_006624	chromosome									
===										19	Thermococcus_kodakarensis_KOD1_uid58225	NC_006624	chromosome									
	57640517	DNA polymerase sliding clamp	No	0	YES	plasmid	501665	502426	-		Thermococcus_kodakarensis_KOD1_uid58225	NC_006624	chromosome		arCOG00488	4 (-)	L	DnaN	DNA polymerase sliding clamp subunit (PCNA ho	4E-14	COG0592	DNA polymerase sliding clamp subunit (PC
	57640518	hypothetical protein TK0583	No	0	YES	plasmid	502554	502799	-		Thermococcus_kodakarensis_KOD1_uid58225	NC_006624	chromosome		arCOG10044	4	S		Uncharacterized conserved protein			
	57640519	hypothetical protein TK0584	No	0	YES	plasmid	502792	503004	-		Thermococcus_kodakarensis_KOD1_uid58225	NC_006624	chromosome									
	57640520	hypothetical protein TK0585	No	0	YES	plasmid	503001	503246	-		Thermococcus_kodakarensis_KOD1_uid58225	NC_006624	chromosome									
	57640521	hypothetical protein TK0586	No	0	YES	plasmid	503239	503481	-		Thermococcus_kodakarensis_KOD1_uid58225	NC_006624	chromosome									
	57640522	hypothetical protein TK0587	No	0	YES	plasmid	503481	505568	-		Thermococcus_kodakarensis_KOD1_uid58225	NC_006624	chromosome		arCOG08578	4 (-)	Y		RepA plasmid replication protein			
	57640523	SpoVT/AbrB family transcriptional regulator	No	0	YES	plasmid	506249	506671	-		Thermococcus_kodakarensis_KOD1_uid58225	NC_006624	chromosome		arCOG03936	6 (-)	K	AbrB	Transcriptional regulator AbrB			
	57640524	hypothetical protein TK0589	No	0	YES	plasmid	506822	507331	-		Thermococcus_kodakarensis_KOD1_uid58225	NC_006624	chromosome									
	57640525	hypothetical protein TK0590	No	0	YES	plasmid	507342	507572	-		Thermococcus_kodakarensis_KOD1_uid58225	NC_006624	chromosome									
	57640526	N-acetyltransferase	No	0	YES	plasmid	507696	508703	+		Thermococcus_kodakarensis_KOD1_uid58225	NC_006624	chromosome									
	57640527	hypothetical protein TK0592	No	0	YES	plasmid	508810	510189	-		Thermococcus_kodakarensis_KOD1_uid58225	NC_006624	chromosome		arCOG06585	2	L		PD-(D/E)XK superfamily nuclease			
	57640528	hypothetical protein TK0593	No	0	YES	plasmid	510170	511333	-		Thermococcus_kodakarensis_KOD1_uid58225	NC_006624	chromosome		arCOG06586	2 (-)	S		Uncharacterized conserved protein			
	57640529	hypothetical protein TK0594	No	2	YES	plasmid	511603	511959	+		Thermococcus_kodakarensis_KOD1_uid58225	NC_006624	chromosome									
	57640530	hypothetical protein TK0595	No	3	YES	plasmid	511930	512484	-		Thermococcus_kodakarensis_KOD1_uid58225	NC_006624	chromosome									
	57640531	hypothetical protein TK0596	No	2	YES	plasmid	512652	513488	+		Thermococcus_kodakarensis_KOD1_uid58225	NC_006624	chromosome		arCOG10043	3	S		Uncharacterized conserved protein			
	57640532	hypothetical protein TK0597	No	0	YES	plasmid	513717	514268	-		Thermococcus_kodakarensis_KOD1_uid58225	NC_006624	chromosome									
	57640533	hypothetical protein TK0598	No	0	YES	plasmid	514265	514465	-		Thermococcus_kodakarensis_KOD1_uid58225	NC_006624	chromosome	clust_453								
	57640534	hypothetical protein TK0599	No	0	YES	plasmid	514543	514848	-		Thermococcus_kodakarensis_KOD1_uid58225	NC_006624	chromosome		arCOG10042	1	S		Uncharacterized conserved protein			
	57640535	hypothetical protein TK0600	No	0	YES	plasmid	514850	515539	-		Thermococcus_kodakarensis_KOD1_uid58225	NC_006624	chromosome		arCOG05103	4 (-)	S		Uncharacterized conserved protein			
===										5	Thermococcus_kodakarensis_KOD1_uid58225	NC_006624	chromosome									
	57641067	hypothetical protein TK1132	No	5			990158	990634	+		Thermococcus_kodakarensis_KOD1_uid58225	NC_006624	chromosome		arCOG10089	1	S		Uncharacterized conserved protein			
	57641068	hypothetical protein TK1133	Yes	0			990755	991456	+		Thermococcus_kodakarensis_KOD1_uid58225	NC_006624	chromosome									
	57641069	hypothetical protein TK1134	No	5			991466	991936	+		Thermococcus_kodakarensis_KOD1_uid58225	NC_006624	chromosome									
	57641070	hypothetical protein TK1135	No	0			991981	992955	-		Thermococcus_kodakarensis_KOD1_uid58225	NC_006624	chromosome		arCOG01360	1 (-)	R		MiaB family, Radical SAM enzyme	5E-108	COG1244	Predicted Fe-S oxidoreductase
	57641071	hypothetical protein TK1136	No	0			993003	994079	-		Thermococcus_kodakarensis_KOD1_uid58225	NC_006624	chromosome		arCOG07135	1	S		Uncharacterized conserved protein			
===										36	Thermococcus_kodakarensis_KOD1_uid58225	NC_006624	chromosome									
	57641278	nucleic acid-binding protein	No	0	YES	Virus_Turrivirid	1183812	1184369	-		Thermococcus_kodakarensis_KOD1_uid58225	NC_006624	chromosome		arCOG02120	1 (-)	V		PIN domain containing protein	0.00002	COG1569	Predicted nucleic acid-binding protein,
	57641279	hypothetical protein TK1344	No	0	YES	Virus_Turrivirid	1184373	1184714	-		Thermococcus_kodakarensis_KOD1_uid58225	NC_006624	chromosome									
	57641280	hypothetical protein TK1345	No	0	YES	Virus_Turrivirid	1184770	1185681	-		Thermococcus_kodakarensis_KOD1_uid58225	NC_006624	chromosome									
	57641281	hypothetical protein TK1346	No	0	YES	Virus_Turrivirid	1185668	1185853	-		Thermococcus_kodakarensis_KOD1_uid58225	NC_006624	chromosome									
	57641282	hypothetical protein TK1347	No	0	YES	Virus_Turrivirid	1185864	1186205	-		Thermococcus_kodakarensis_KOD1_uid58225	NC_006624	chromosome	clust_306								
	57641283	hypothetical protein TK1348	No	1	YES	Virus_Turrivirid	1186202	1186717	-		Thermococcus_kodakarensis_KOD1_uid58225	NC_006624	chromosome									
	57641284	hypothetical protein TK1349	No	0	YES	Virus_Turrivirid	1186724	1186996	-		Thermococcus_kodakarensis_KOD1_uid58225	NC_006624	chromosome		arCOG03881	5 (-)	S		Uncharacterized conserved protein			
	57641285	hypothetical protein TK1350	No	0	YES	Virus_Turrivirid	1186993	1187193	-		Thermococcus_kodakarensis_KOD1_uid58225	NC_006624	chromosome	clust_454								
	57641286	hypothetical protein TK1351	No	1	YES	Virus_Turrivirid	1187310	1188182	-		Thermococcus_kodakarensis_KOD1_uid58225	NC_006624	chromosome	clust_111								
	57641287	hypothetical protein TK1352	No	1	YES	Virus_Turrivirid	1188188	1188835	-		Thermococcus_kodakarensis_KOD1_uid58225	NC_006624	chromosome									
	57641288	hypothetical protein TK1353	No	0	YES	Virus_Turrivirid	1188900	1189844	-		Thermococcus_kodakarensis_KOD1_uid58225	NC_006624	chromosome		arCOG10335	5 (-)	Y		Major capsid protein, double jelly roll fold protein			
	57641289	hypothetical protein TK1354	No	0	YES	Virus_Turrivirid	1190102	1190266	-		Thermococcus_kodakarensis_KOD1_uid58225	NC_006624	chromosome									
	57641290	hypothetical protein TK1355	No	2	YES	Virus_Turrivirid	1190284	1190517	-		Thermococcus_kodakarensis_KOD1_uid58225	NC_006624	chromosome									
	57641291	ATPase	No	0	YES	Virus_Turrivirid	1190522	1191223	-		Thermococcus_kodakarensis_KOD1_uid58225	NC_006624	chromosome		arCOG00281	4 (-)	R		Predicted ATPase	0.0000001	COG0433	HerA helicase
	57641292	hypothetical protein TK1357	No	0	YES	Virus_Turrivirid	1191247	1191870	-		Thermococcus_kodakarensis_KOD1_uid58225	NC_006624	chromosome									
	57641293	hypothetical protein TK1358	No	0	YES	Virus_Turrivirid	1191863	1192063	-		Thermococcus_kodakarensis_KOD1_uid58225	NC_006624	chromosome									
	57641294	hypothetical protein TK1359	No	0	YES	Virus_Turrivirid	1192056	1192331	-		Thermococcus_kodakarensis_KOD1_uid58225	NC_006624	chromosome									
	57641295	hypothetical protein TK1360	No	1	YES	Virus_Turrivirid	1192334	1192999	-		Thermococcus_kodakarensis_KOD1_uid58225	NC_006624	chromosome									
	57641296	MCM2/3/5 family DNA replication licensing fact	No	0		Virus_Turrivirid	1193148	1195580	-		Thermococcus_kodakarensis_KOD1_uid58225	NC_006624	chromosome		arCOG00439	14 (-)	L	MCM2	Predicted ATPase involved in replication control,	6E-113	COG1241	Predicted ATPase involved in replication
	57641297	hypothetical protein TK1362	No	2	YES	Virus_Turrivirid	1195814	1196200	+		Thermococcus_kodakarensis_KOD1_uid58225	NC_006624	chromosome									
	57641298	hypothetical protein TK1363	No	0	YES	Virus_Turrivirid	1196202	1196354	-		Thermococcus_kodakarensis_KOD1_uid58225	NC_006624	chromosome									
	57641299	hypothetical protein TK1364	No	0	YES	Virus_Turrivirid	1196359	1196877	-		Thermococcus_kodakarensis_KOD1_uid58225	NC_006624	chromosome									
	57641300	hypothetical protein TK1365	No	0	YES	Virus_Turrivirid	1196874	1197062	-		Thermococcus_kodakarensis_KOD1_uid58225	NC_006624	chromosome									
	57641301	hypothetical protein TK1366	No	0	YES	Virus_Turrivirid	1197059	1197268	-		Thermococcus_kodakarensis_KOD1_uid58225	NC_006624	chromosome									
	57641302	hypothetical protein TK1367	No	0	YES	Virus_Turrivirid	1197265	1197615	-		Thermococcus_kodakarensis_KOD1_uid58225	NC_006624	chromosome									
	57641303	hypothetical protein TK1368	No	0	YES	Virus_Turrivirid	1197612	1197800	-		Thermococcus_kodakarensis_KOD1_uid58225	NC_006624	chromosome									
	57641304	hypothetical protein TK1369	No	0	YES	Virus_Turrivirid	1197797	1198054	-		Thermococcus_kodakarensis_KOD1_uid58225	NC_006624	chromosome		arCOG10045	2	S		Uncharacterized conserved protein			
	57641305	hypothetical protein TK1370	No	0	YES	Virus_Turrivirid	1198067	1198333	-		Thermococcus_kodakarensis_KOD1_uid58225	NC_006624	chromosome									
	57641306	hypothetical protein TK1371	No	0	YES	Virus_Turrivirid	1198330	1198563	-		Thermococcus_kodakarensis_KOD1_uid58225	NC_006624	chromosome									
	57641307	SpoVT/AbrB family transcriptional regulator	No	0	YES	Virus_Turrivirid	1198722	1198907	+		Thermococcus_kodakarensis_KOD1_uid58225	NC_006624	chromosome		arCOG00818	3 (-)	K	AbrB	Transcriptional regulator AbrB			
	57641308	hypothetical protein TK1373	No	1	YES	Virus_Turrivirid	1198946	1199155	-		Thermococcus_kodakarensis_KOD1_uid58225	NC_006624	chromosome		arCOG09561	2	S		Uncharacterized conserved protein			
	57641309	hypothetical protein TK1374	No	1	YES	Virus_Turrivirid	1199148	1199474	-		Thermococcus_kodakarensis_KOD1_uid58225	NC_006624	chromosome									

57641310	hypothetical protein TK1375	No	2	YES	Virus_Turrivirid	1199550	1200056	+		Thermococcus_kodakarensis_KOD1_uid58225	NC_006624	chromosome										
57641311	hypothetical protein TK1376	No	0	YES	Virus_Turrivirid	1200148	1200480	+		Thermococcus_kodakarensis_KOD1_uid58225	NC_006624	chromosome										
57641312	hypothetical protein TK1377	No	0	YES	Virus_Turrivirid	1200484	1201023	+		Thermococcus_kodakarensis_KOD1_uid58225	NC_006624	chromosome		arCOG06398	3	(-)	S		Uncharacterized conserved protein			
57641313	integrase, N-fragment	No	0	YES	Virus_Turrivirid	1201244	1201660	+		Thermococcus_kodakarensis_KOD1_uid58225	NC_006624	chromosome										
===									7	Thermococcus_kodakarensis_KOD1_uid58225	NC_006624	chromosome										
57641660	hypothetical protein TK1725	No	10			1517683	1521411	+		Thermococcus_kodakarensis_KOD1_uid58225	NC_006624	chromosome	arCOG07351,a	arCOG07351	1	(-)	S		Uncharacterized conserved membrane protein			
57641661	hypothetical protein TK1726	No	0			1521546	1522403	+		Thermococcus_kodakarensis_KOD1_uid58225	NC_006624	chromosome		arCOG04485	4		S		Uncharacterized conserved protein			
57641662	hypothetical protein TK1727	No	0			1522345	1522611	+		Thermococcus_kodakarensis_KOD1_uid58225	NC_006624	chromosome		arCOG04485	4		S		Uncharacterized conserved protein			
57641663	hypothetical protein TK1728	Yes	3			1523030	1524478	+		Thermococcus_kodakarensis_KOD1_uid58225	NC_006624	chromosome		arCOG10680	1		S		Uncharacterized conserved protein			
57641664	hypothetical protein TK1729	No	5			1524521	1525180	+		Thermococcus_kodakarensis_KOD1_uid58225	NC_006624	chromosome										
57641665	hypothetical protein TK1730	No	4			1525174	1525887	+		Thermococcus_kodakarensis_KOD1_uid58225	NC_006624	chromosome										
57641666	glycosyl transferase family protein	No	0			1525877	1526938	-		Thermococcus_kodakarensis_KOD1_uid58225	NC_006624	chromosome		arCOG01403	4	(-)	M	RfaG	Glycosyltransferase	5E-36	COG0438	Glycosyltransferase
===									6	Thermococcus_kodakarensis_KOD1_uid58225	NC_006624	chromosome										
57641881	translation initiation factor IF-2 subunit gamma	No	0			1756859	1758091	-		Thermococcus_kodakarensis_KOD1_uid58225	NC_006624	chromosome		arCOG01563	1	(-)	J	GCD11	Translation initiation factor 2, gamma subunit (eIF-2gamma; GTPa		COG5257	Translation initiation factor 2, gamma s
57641882	hypothetical protein TK1947	No	4			1758235	1758627	-		Thermococcus_kodakarensis_KOD1_uid58225	NC_006624	chromosome										
57641883	hypothetical protein TK1948	No	4			1758764	1759174	-		Thermococcus_kodakarensis_KOD1_uid58225	NC_006624	chromosome										
57641884	hypothetical protein TK1949	Yes	1			1759392	1759943	-		Thermococcus_kodakarensis_KOD1_uid58225	NC_006624	chromosome		arCOG10100	1		S		Uncharacterized conserved protein			
57641885	hypothetical protein TK1950	Yes	1			1760017	1761297	-		Thermococcus_kodakarensis_KOD1_uid58225	NC_006624	chromosome										
57641886	30S ribosomal protein S6e	No	0			1761531	1761908	-		Thermococcus_kodakarensis_KOD1_uid58225	NC_006624	chromosome		arCOG01946	4	(-)	J	RPS6A	Ribosomal protein S6E (S10)	2E-44	COG2125	Ribosomal protein S6E (S10)
===									6	Thermococcus_kodakarensis_KOD1_uid58225	NC_006624	chromosome										
57641919	pyruvate ferredoxin oxidoreductase subunit be	No	0			1788044	1789039	+		Thermococcus_kodakarensis_KOD1_uid58225	NC_006624	chromosome		arCOG01601	1	(-)	C	PorB	Pyruvate:ferredoxin oxidoreductase or related 2-	1E-58	COG1013	Pyruvate:ferredoxin oxidoreductase and r
57641920	hypothetical protein TK1985	Yes	0			1789114	1790061	+		Thermococcus_kodakarensis_KOD1_uid58225	NC_006624	chromosome		arCOG10048	5		S		Uncharacterized conserved protein			
57641921	hypothetical protein TK1986	No	0			1790069	1790932	+		Thermococcus_kodakarensis_KOD1_uid58225	NC_006624	chromosome		arCOG11323	5		S		Uncharacterized conserved protein			
57641922	hypothetical protein TK1987	No	0			1790937	1791323	+		Thermococcus_kodakarensis_KOD1_uid58225	NC_006624	chromosome										
57641923	hypothetical protein TK1988	No	0			1791348	1792133	+		Thermococcus_kodakarensis_KOD1_uid58225	NC_006624	chromosome		arCOG10047	4		S		Uncharacterized conserved protein			
57641924	hypothetical protein TK1989	No	1			1792156	1792692	+		Thermococcus_kodakarensis_KOD1_uid58225	NC_006624	chromosome		arCOG05855	1	(-)	S		Uncharacterized conserved protein			
===									6	Thermococcus_kodakarensis_KOD1_uid58225	NC_006624	chromosome										
57642108	hypothetical protein TK2173	No	0			1956762	1957343	-		Thermococcus_kodakarensis_KOD1_uid58225	NC_006624	chromosome		arCOG06115	2	(-)	S		Uncharacterized conserved protein			
57642109	hypothetical protein TK2174	Yes	1			1957819	1958415	-		Thermococcus_kodakarensis_KOD1_uid58225	NC_006624	chromosome		arCOG10027	12		S		Uncharacterized conserved protein			
57642110	hypothetical protein TK2175	Yes	1			1958749	1959345	-		Thermococcus_kodakarensis_KOD1_uid58225	NC_006624	chromosome		arCOG10066	9		S		Uncharacterized conserved protein			
57642111	hypothetical protein TK2176	No	1			1959362	1960336	-		Thermococcus_kodakarensis_KOD1_uid58225	NC_006624	chromosome		arCOG10027	12		S		Uncharacterized conserved protein			
57642112	hypothetical protein TK2177	Yes	1			1960404	1961279	-		Thermococcus_kodakarensis_KOD1_uid58225	NC_006624	chromosome		arCOG10028	3		S		Uncharacterized conserved protein			
57642113	hypothetical protein TK2178	Yes	1			1961276	1965046	-		Thermococcus_kodakarensis_KOD1_uid58225	NC_006624	chromosome	arCOG07561,a	arCOG07561	7	(-)	K		Predicted regulatory protein containing HTH domain			
===									5	Thermococcus_kodakarensis_KOD1_uid58225	NC_006624	chromosome										
57642115	hypothetical protein TK2180	Yes	1			1966264	1968768	+		Thermococcus_kodakarensis_KOD1_uid58225	NC_006624	chromosome		arCOG07530	3		S		Uncharacterized conserved protein			
57642116	calcium-binding protein	Yes	1			1968761	1970017	+		Thermococcus_kodakarensis_KOD1_uid58225	NC_006624	chromosome		arCOG07765	3		S		Uncharacterized conserved protein			
57642117	hypothetical protein TK2182	No	0			1970169	1970627	+		Thermococcus_kodakarensis_KOD1_uid58225	NC_006624	chromosome		arCOG09434	2		S		Uncharacterized conserved protein			
57642118	hypothetical protein TK2183	No	1			1970633	1971037	+		Thermococcus_kodakarensis_KOD1_uid58225	NC_006624	chromosome		arCOG06145	2	(-)	S		Uncharacterized conserved protein			
57642119	hypothetical protein TK2184	Yes	0			1971047	1971760	+		Thermococcus_kodakarensis_KOD1_uid58225	NC_006624	chromosome		arCOG06146	3		S		Uncharacterized conserved protein			
===									6	Thermococcus_onnurineus_NA1_uid59043	NC_011529	chromosome										
212223467	protein TON_0321	No	0	YES		296530	297198	-		Thermococcus_onnurineus_NA1_uid59043	NC_011529	chromosome		arCOG00790	6	(-)	V	Cas4	CRISPR-associated protein Cas4, RecB family exonuclease			
212223468	protein TON_0322	No	0	YES		297243	297965	-		Thermococcus_onnurineus_NA1_uid59043	NC_011529	chromosome										
212223469	protein TON_0323	No	0	YES		298165	299160	-		Thermococcus_onnurineus_NA1_uid59043	NC_011529	chromosome										
212223470	protein TON_0324	No	0	YES		299157	299519	-		Thermococcus_onnurineus_NA1_uid59043	NC_011529	chromosome										
212223471	protein TON_0325	No	0	YES		299497	301260	-		Thermococcus_onnurineus_NA1_uid59043	NC_011529	chromosome										
212223472	protein TON_0326	No	0	YES		301262	302053	-		Thermococcus_onnurineus_NA1_uid59043	NC_011529	chromosome		arCOG04342	2	(-)	V		CRISPR system related protein Cas6, RAMP sup	2E-56	COG1583	CRISPR system related protein, RAMP supe
===									6	Thermococcus_onnurineus_NA1_uid59043	NC_011529	chromosome										
212224420	NADH oxidase	No	0			1157017	1158336	+		Thermococcus_onnurineus_NA1_uid59043	NC_011529	chromosome		arCOG01069	1	(-)	P		CoA-dependent NAD(P)H Sulfur Oxidoreductase	8E-64	COG1251	NAD(P)H-nitrite reductase
212224421	membrane-bound metal-dependent hydrolase,	No	2			1158402	1158596	+		Thermococcus_onnurineus_NA1_uid59043	NC_011529	chromosome										
212224422	membrane-bound metal-dependent hydrolase	No	2			1158620	1158922	+		Thermococcus_onnurineus_NA1_uid59043	NC_011529	chromosome										
212224423	membrane-bound metal-dependent hydrolase	No	0			1159107	1159451	+		Thermococcus_onnurineus_NA1_uid59043	NC_011529	chromosome										
212224424	membrane-bound metal-dependent hydrolase,	No	0			1159482	1159652	+		Thermococcus_onnurineus_NA1_uid59043	NC_011529	chromosome										
212224425	Heat shock protein/ Zn-dependent protease wi	No	7			1159737	1160900	+		Thermococcus_onnurineus_NA1_uid59043	NC_011529	chromosome		arCOG01334	4	(-)	O	HtpX	Zn-dependent protease with chaperone function	2E-11	COG0501	Zn-dependent protease with chaperone fun
===									5	Thermococcus_onnurineus_NA1_uid59043	NC_011529	chromosome										
212224874	protein TON_1723	No	0			1591834	1591953	-		Thermococcus_onnurineus_NA1_uid59043	NC_011529	chromosome		arCOG10094	2		S		Uncharacterized conserved protein			
212224875	phospholipase D-related protein	Yes	1			1591993	1592757	-		Thermococcus_onnurineus_NA1_uid59043	NC_011529	chromosome		arCOG02041	1	(-)	I	Cls	Phosphatidylserine/phosphatidylglycerophospha	0.00000003	COG1502	Phosphatidylserine/phosphatidylglyceroph
212224876	protein TON_1725	No	2			1593026	1593280	+		Thermococcus_onnurineus_NA1_uid59043	NC_011529	chromosome										
212224877	protein TON_1726	Yes	1			1593266	1594894	-		Thermococcus_onnurineus_NA1_uid59043	NC_011529	chromosome										
212224878	protein TON_1727	No	0			1595089	1595256	+		Thermococcus_onnurineus_NA1_uid59043	NC_011529	chromosome										
===									7	Thermococcus_sibiricus_MM_739_uid59399	NC_012883	chromosome										
242398643	hypothetical protein TSIB_0653	No	4			609085	609408	+		Thermococcus_sibiricus_MM_739_uid59399	NC_012883	chromosome										
242398644	hypothetical protein TSIB_0654	Yes	0			609623	610345	+		Thermococcus_sibiricus_MM_739_uid59399	NC_012883	chromosome		arCOG07133	1	(-)	S		Uncharacterized conserved protein			
242398645	hypothetical protein TSIB_0655	No	4			610356	610700	+		Thermococcus_sibiricus_MM_739_uid59399	NC_012883	chromosome										
242398646	hypothetical protein TSIB_0656	No	1			610771	610911	-		Thermococcus_sibiricus_MM_739_uid59399	NC_012883	chromosome										
242398647	hypothetical protein TSIB_0657	No	0			611148	611288	+		Thermococcus_sibiricus_MM_739_uid59399	NC_012883	chromosome										
242398648	hypothetical protein TSIB_0658	No	1			611416	611568	+		Thermococcus_sibiricus_MM_739_uid59399	NC_012883	chromosome										
242398649	S-adenosyl-L-homocysteine hydrolase	No	0			611574	612839	-		Thermococcus_sibiricus_MM_739_uid59399	NC_012883	chromosome		arCOG04137	1	(-)	H	SAM1	S-adenosylhomocysteine hydrolase		COG0499	S-adenosylhomocysteine hydrolase
===									5	Thermococcus_sibiricus_MM_739_uid59399	NC_012883	chromosome										
242398723	hypothetical protein TSIB_0735	No	0	YES		677285	677980	-		Thermococcus_sibiricus_MM_739_uid59399	NC_012883	chromosome										
242398724	hypothetical protein TSIB_0736	No	0	YES		678004	678999	-		Thermococcus_sibiricus_MM_739_uid59399	NC_012883	chromosome										
242398725	hypothetical protein TSIB_0737	No	1	YES		679006	679341	-		Thermococcus_sibiricus_MM_739_uid59399	NC_012883	chromosome										
242398726	Putative nickel-responsive regulator	No	0	YES		679430	679846	+		Thermococcus_sibiricus_MM_739_uid59399	NC_012883	chromosome		arCOG01008	7	(-)	V	NikR	Transcriptional regulator, CopG/Arc/MetJ family (I	4E-43	COG0864	Predicted transcriptional regulators con
242398727	hypothetical protein TSIB_0740	No	0	YES		680454	680612	-		Thermococcus_sibiricus_MM_739_uid59399	NC_012883	chromosome										
===									8	Thermococcus_sibiricus_MM_739_uid59399	NC_012883	chromosome										
242399014	hypothetical protein TSIB_1033	No	0			942327	942743	+		Thermococcus_sibiricus_MM_739_uid59399	NC_012883	chromosome		arCOG04033	1	(-)	S		Uncharacterized conserved protein			
242399015	hypothetical protein TSIB_1034	No	1			942757	943359	+		Thermococcus_sibiricus_MM_739_uid59399	NC_012883	chromosome										
242399016	hypothetical protein TSIB_1035	No	4			943462	943842	+		Thermococcus_sibiricus_MM_739_uid59399	NC_012883	chromosome										
242399017	hypothetical protein TSIB_1036	No	2			943887	944588	+		Thermococcus_sibiricus_MM_739_uid59399	NC_012883	chromosome		arCOG08597	2		S		Uncharacterized conserved protein			
242399018	hypothetical protein TSIB_1037	No	1			944631	945098	+		Thermococcus_sibiricus_MM_739_uid59399	NC_012883	chromosome										
242399019	hypothetical protein TSIB_1038	No	0			945422	945931	+		Thermococcus_sibiricus_MM_739_uid59399	NC_012883	chromosome		arCOG03789	1	(-)	S		Uncharacterized conserved protein	3E-36	pfam03192	Pyrococcus protein of unknown function,
242399020	hypothetical protein TSIB_1039	No	2			946121	946300	-		Thermococcus_sibiricus_MM_739_uid59399	NC_012883	chromosome										
242399021	hypothetical protein TSIB_1040	No	2			946304	946489	-		Thermococcus_sibiricus_MM_739_uid59399	NC_012883	chromosome										
===									7	Thermococcus_sibiricus_MM_739_uid59399	NC_012883	chromosome										
242399134	Archaeosine biosynthesis protein queC	No	0			1055602	1056327	-		Thermococcus_sibiricus_MM_739_uid59399	NC_012883	chromosome		arCOG00039	1	(-)	R		Predicted PP-loop superfamily ATPase	2E-47	COG0603	Predicted PP-loop superfamily ATPase
242399135	hypothetical protein TSIB_1156	No	3			1056358	1056681	-		Thermococcus_sibiricus_MM_739_uid59399	NC_012883	chromosome										
242399136	hypothetical protein TSIB_1157	Yes	0			1057106	1057450	+		Thermococcus_sibiricus_MM_739_uid59399	NC_012883	chromosome										
242399137	hypothetical protein TSIB_1158	No	0			1057545	1057769	+		Thermococcus_sibiricus_MM_739_uid59399	NC_012883	chromosome										
242399138	hypothetical protein TSIB_1159	No	1			1057805	1058014	+		Thermococcus_sibiricus_MM_739_uid59399	NC_012883	chromosome										
242399139	hypothetical proteinn	No	0			1058220	1058339	-		Thermococcus_sibiricus_MM_739_uid59399	NC_012883	chromosome										
242399140	hypothetical protein TSIB_1161	No	0			1058489	1059076	-		Thermococcus_sibiricus_MM_739_uid59399	NC_012883	chromosome		arCOG00928	1	(-)	L		Endonuclease V homolog	5E-60	COG1628	Endonuclease V homolog
===									5	Thermococcus_sibiricus_MM_739_uid59399	NC_012883	chromosome										
242399422	hypothetical protein TSIB_1447	No	0			1291430	1291549	-		Thermococcus_sibiricus_MM_739_uid59399	NC_012883	chromosome										
242399423	kinase domain protein	Yes	2			1291848	1294772	+		Thermococcus_sibiricus_MM_739_uid59399	NC_012883	chromosome		arCOG02559	3	(-)	S		Cell suface protein			
242399424	hypothetical protein TSIB_1449	No	4			1294931	1295383	+		Thermococcus_sibiricus_MM_739_uid59399	NC_012883	chromosome										
242399425	hypothetical protein TSIB_1450	Yes	1			1295426	1297495	+		Thermococcus_sibiricus_MM_739_uid59399	NC_012883	chromosome		arCOG07530	3		S		Uncharacterized conserved protein			
242399426	Predicted calcium-binding protein	No	1			1297488	1298696	+		Thermococcus_sibiricus_MM_739_uid59399	NC_012883	chromosome		arCOG07765	3		S		Uncharacterized conserved protein			
===									9	Thermococcus_sibiricus_MM_739_uid59399	NC_012883	chromosome										
242399944	hypothetical protein TSIB_1971	No	2			1766225	1766473	-		Thermococcus_sibiricus_MM_739_uid59399	NC_012883	chromosome										
242399945	hypothetical protein TSIB_1972	No	3			1766520	1766861	-		Thermococcus_sibiricus_MM_739_uid59399	NC_012883	chromosome		arCOG09498	1		S		Uncharacterized conserved protein			
242399946	oxidoreductase molybdopterin binding	Yes	1			1766858	1767478	-		Thermococcus_sibiricus_MM_739_uid59399	NC_012883	chromosome		arCOG00266	1	(-)	R		Sulfite oxidase or related enzyme	7E-25	pfam00174	Oxidoreductase molybdopterin binding dom
242399947	hypothetical protein TSIB_1974	No	0			1767264	1767497	+		Thermococcus_sibiricus_MM_739_uid59399	NC_012883	chromosome										
242399948	hypothetical protein TSIB_1975	No	3			1767580	1767900	-		Thermococcus_sibiricus_MM_739_uid59399	NC_012883	chromosome										
242399949	hypothetical protein TSIB_1976	No	0			1767968	1768099	+		Thermococcus_sibiricus_MM_739_uid59399	NC_012883	chromosome										
242399950	hypothetical protein TSIB_1977	No	3			1768391	1768657	-		Thermococcus_sibiricus_MM_739_uid59399	NC_012883	chromosome										
242399951	hypothetical protein TSIB_1978	No	0			1768660	1768830	+		Thermococcus_sibiricus_MM_739_uid59399	NC_012883	chromosome										
242399952	hypothetical protein TSIB_1979	No	0			1768883	1769455	-		Thermococcus_sibiricus_MM_739_uid59399	NC_012883	chromosome		arCOG04225	1	(-)	R		Predicted phosphatase homologous to the C-terr	2E-31	COG2110	Predicted phosphatase homologous to the
===									52	Thermofilum_pendens_Hrk_5_uid58563	NC_008696	plasmid pTPEN01										
119709758	hypothetical protein Tpen_1840	No	7	YES	plasmid	47	1714	-		Thermofilum_pendens_Hrk_5_uid58563	NC_008696	plasmid pTPEN01										
119709759	hypothetical protein Tpen_1841	No	3	YES	plasmid	1778	2074	-		Thermofilum_pendens_Hrk_5_uid58563	NC_008696	plasmid pTPEN01								0.0001	pfam01032	FecCD transport family. This is a sub-fa ; Domain of unknown function (DUF3552). Th
119709760	abortive infection protein	No	8	YES	plasmid	2071	3015	-		Thermofilum_pendens_Hrk_5_uid58563	NC_008696	plasmid pTPEN01										
119709761	hypothetical protein Tpen_1843	No	4	YES	plasmid	3012	3572	-		Thermofilum_pendens_Hrk_5_uid58563	NC_008696	plasmid pTPEN01										
119709762	hypothetical protein Tpen_1844	No	2	YES	plasmid	3585	3803	-		Thermofilum_pendens_Hrk_5_uid58563	NC_008696	plasmid pTPEN01										
119709763	hypothetical protein Tpen_1845	No	0	YES	plasmid	3846	4214	-		Thermofilum_pendens_Hrk_5_uid58563	NC_008696	plasmid pTPEN01										
119709764	hypothetical protein Tpen_1846	No	0	YES	plasmid	4306	4677	-		Thermofilum_pendens_Hrk_5_uid58563	NC_008696	plasmid pTPEN01										
119709765	hypothetical protein Tpen_1847	No	0	YES	plasmid	4677	5063	-		Thermofilum_pendens_Hrk_5_uid58563	NC_008696	plasmid pTPEN01										
119709766	hypothetical protein Tpen_1848	No	0	YES	plasmid	5211	5609	-		Thermofilum_pendens_Hrk_5_uid58563	NC_008696	plasmid pTPEN01										
119709767	hypothetical protein Tpen_1849	No	0	YES	plasmid	5762	5995	-		Thermofilum_pendens_Hrk_5_uid58563	NC_008696	plasmid pTPEN01										
119709768	hypothetical protein Tpen_1850	No	0	YES	plasmid	6000	6356	-		Thermofilum_pendens_Hrk_5_uid58563	NC_008696	plasmid pTPEN01										
119709769	hypothetical protein Tpen_1851	No	0	YES	plasmid	6500	6697	-		Thermofilum_pendens_Hrk_5_uid58563	NC_008696	plasmid pTPEN01										
119709770	hypothetical protein Tpen_1852	No	0	YES	plasmid	6813	7586	-		Thermofilum_pendens_Hrk_5_uid58563	NC_008696	plasmid pTPEN01										
119709771	hypothetical protein Tpen_1853	No	0	YES	plasmid	8070	8768	-		Thermofilum_pendens_Hrk_5_uid58563	NC_008696	plasmid pTPEN01								0.000004	COG1196	Chromosome segregation ATPases
119709772	hypothetical protein Tpen_1854	No	0	YES	plasmid	8952	9350	+		Thermofilum_pendens_Hrk_5_uid58563	NC_008696	plasmid pTPEN01										
119709773	hypothetical protein Tpen_1855	No	0	YES	plasmid	9482	9862	+		Thermofilum_pendens_Hrk_5_uid58563	NC_008696	plasmid pTPEN01										
119709774	hypothetical protein Tpen_1856	No	0	YES	plasmid	9859	10311	+		Thermofilum_pendens_Hrk_5_uid58563	NC_008696	plasmid pTPEN01										
119709775	hypothetical protein Tpen_1857	No	1	YES	plasmid	10818	12032	+		Thermofilum_pendens_Hrk_5_uid58563	NC_008696	plasmid pTPEN01										
119709776	hypothetical protein Tpen_1858	Yes	1	YES	plasmid	12044	12730	+		Thermofilum_pendens_Hrk_5_uid58563	NC_008696	plasmid pTPEN01										
119709777	hypothetical protein Tpen_1859	Yes	4	YES	plasmid	12776	13279	+		Thermofilum_pendens_Hrk_5_uid58563	NC_008696	plasmid pTPEN01										
119709778	hypothetical protein Tpen_1860	No	0	YES	plasmid	13276	13575	+		Thermofilum_pendens_Hrk_5_uid58563	NC_008696	plasmid pTPEN01										
119709779	hypothetical protein Tpen_1861	No	0	YES	plasmid	13556	13774	+		Thermofilum_pendens_Hrk_5_uid58563	NC_008696	plasmid pTPEN01										
119709780	hypothetical protein Tpen_1862	No	0	YES	plasmid	13806	14255	+		Thermofilum_pendens_Hrk_5_uid58563	NC_008696	plasmid pTPEN01										
119709781	hypothetical protein Tpen_1863	No	0	YES	plasmid	14305	14574	+		Thermofilum_pendens_Hrk_5_uid58563	NC_008696	plasmid pTPEN01										
119709782	hypothetical protein Tpen_1864	No	0	YES	plasmid	14580	14876	+		Thermofilum_pendens_Hrk_5_uid58563	NC_008696	plasmid pTPEN01										
119709783	hypothetical protein Tpen_1865	No	2	YES	plasmid	14866	15402	+		Thermofilum_pendens_Hrk_5_uid58563	NC_008696	plasmid pTPEN01										
119709784	hypothetical protein Tpen_1866	Yes	2	YES	plasmid	15432	17306	+		Thermofilum_pendens_Hrk_5_uid58563	NC_008696	plasmid pTPEN01										
119709785	hypothetical protein Tpen_1867	No	0	YES	plasmid	17355	17555	+		Thermofilum_pendens_Hrk_5_uid58563	NC_008696	plasmid pTPEN01										
119709786	hypothetical protein Tpen_1868	No	2	YES	plasmid	17555	17845	+		Thermofilum_pendens_Hrk_5_uid58563	NC_008696	plasmid pTPEN01		arCOG04811	3	(-)	S		Uncharacterized conserved membrane protein, F	0.000000007	COG2383	Uncharacterized conserved protein
119709787	hypothetical protein Tpen_1869	Yes	1	YES	plasmid	17859	18299	+		Thermofilum_pendens_Hrk_5_uid58563	NC_008696	plasmid pTPEN01										
119709788	hypothetical protein Tpen_1870	No	0	YES	plasmid	18301	18462	+		Thermofilum_pendens_Hrk_5_uid58563	NC_008696	plasmid pTPEN01		arCOG06086	2		S		Uncharacterized conserved protein			
119709789	hypothetical protein Tpen_1871	No	0	YES	plasmid	18459	18707	-		Thermofilum_pendens_Hrk_5_uid58563	NC_008696	plasmid pTPEN01										
119709790	hypothetical protein Tpen_1872	No	0	YES	plasmid	18892	19323	-		Thermofilum_pendens_Hrk_5_uid58563	NC_008696	plasmid pTPEN01										
119709791	hypothetical protein Tpen_1873	No	0	YES	plasmid	19362	19532	-		Thermofilum_pendens_Hrk_5_uid58563	NC_008696	plasmid pTPEN01										
119709792	hypothetical protein Tpen_1874	No	0	YES	plasmid	19986	20183	-		Thermofilum_pendens_Hrk_5_uid58563	NC_008696	plasmid pTPEN01										
119709793	DEAD/DEAH box helicase domain-containing p	No	0	YES	plasmid	20180	22081	-		Thermofilum_pendens_Hrk_5_uid58563	NC_008696	plasmid pTPEN01		arCOG00874	2	(-)	K	xpb	ssDNA-dependent ATPase, helicase superfamily	4E-31	COG1061	DNA or RNA helicases of superfamily II [
119709794	hypothetical protein Tpen_1876	No	0	YES	plasmid	23125	23532	-		Thermofilum_pendens_Hrk_5_uid58563	NC_008696	plasmid pTPEN01										
119709795	hypothetical protein Tpen_1877	No	0	YES	plasmid	23724	24191	-		Thermofilum_pendens_Hrk_5_uid58563	NC_008696	plasmid pTPEN01										
119709796	hypothetical protein Tpen_1878	No	0	YES	plasmid	24413	24997	-		Thermofilum_pendens_Hrk_5_uid58563	NC_008696	plasmid pTPEN01										
119709797	hypothetical protein Tpen_1879	No	0	YES	plasmid	25066	25800	-		Thermofilum_pendens_Hrk_5_uid58563	NC_008696	plasmid pTPEN01										
119709798	hypothetical protein Tpen_1880	No	0	YES	plasmid	25827	26111	-		Thermofilum_pendens_Hrk_5_uid58563	NC_008696	plasmid pTPEN01										
119709799	hypothetical protein Tpen_1881	No	0	YES	plasmid	26218	26727	+		Thermofilum_pendens_Hrk_5_uid58563	NC_008696	plasmid pTPEN01										
119709800	hypothetical protein Tpen_1882	No	0	YES	plasmid	26746	26943	-		Thermofilum_pendens_Hrk_5_uid58563	NC_008696	plasmid pTPEN01										
119709801	hypothetical protein Tpen_1883	No	0	YES	plasmid	26940	27542	-		Thermofilum_pendens_Hrk_5_uid58563	NC_008696	plasmid pTPEN01										
119709802	hypothetical protein Tpen_1884	No	0	YES	plasmid	27627	27809	-		Thermofilum_pendens_Hrk_5_uid58563	NC_008696	plasmid pTPEN01										
119709803	hypothetical protein Tpen_1885	No	0	YES	plasmid	27844	28041	-		Thermofilum_pendens_Hrk_5_uid58563	NC_008696	plasmid pTPEN01										
119709804	hypothetical protein Tpen_1886	No	0	YES	plasmid	28094	28309	-		Thermofilum_pendens_Hrk_5_uid58563	NC_008696	plasmid pTPEN01										
119709805	hypothetical protein Tpen_1887	No	5	YES	plasmid	28378	28872	+		Thermofilum_pendens_Hrk_5_uid58563	NC_008696	plasmid pTPEN01										
119709806	hypothetical protein Tpen_1888	No	0	YES	plasmid	28869	29591	-		Thermofilum_pendens_Hrk_5_uid58563	NC_008696	plasmid pTPEN01										
119709807	hypothetical protein Tpen_1889	No	0	YES	plasmid	29696	30157	-		Thermofilum_pendens_Hrk_5_uid58563	NC_008696	plasmid pTPEN01										
119709808	hypothetical protein Tpen_1890	No	0	YES	plasmid	30135	30653	-		Thermofilum_pendens_Hrk_5_uid58563	NC_008696	plasmid pTPEN01										
119709809	phage integrase family protein	No	0	YES	plasmid	30662	31498	-		Thermofilum_pendens_Hrk_5_uid58563	NC_008696	plasmid pTPEN01		arCOG01241	31	(-)	L	XerC	Integrase	4E-30	COG4974	Site-specific recombinase XerD
===									7	Thermofilum_pendens_Hrk_5_uid58563	NC_008698	chromosome										
119718951	hypothetical protein Tpen_0033	No	0			24935	25309	+		Thermofilum_pendens_Hrk_5_uid58563	NC_008698	chromosome		arCOG00022	1	(-)	S		Uncharacterized conserved protein			
119718952	hypothetical protein Tpen_0034	No	0			25306	25722	-		Thermofilum_pendens_Hrk_5_uid58563	NC_008698	chromosome										
119718953	hypothetical protein Tpen_0035	No	0			25762	26838	-		Thermofilum_pendens_Hrk_5_uid58563	NC_008698	chromosome										
119718954	hypothetical protein Tpen_0036	No	0			26954	27238	+		Thermofilum_pendens_Hrk_5_uid58563	NC_008698	chromosome										
119718955	hypothetical protein Tpen_0037	No	0			27229	27654	+		Thermofilum_pendens_Hrk_5_uid58563	NC_008698	chromosome										
119718956	putative adenylyl cyclase CyaB	No	0			27657	28199	+		Thermofilum_pendens_Hrk_5_uid58563	NC_008698	chromosome		arCOG01723	2	(-)	F	CyaB	Adenylate cyclase, class 2 (thermophilic)	7E-42	COG1437	Adenylate cyclase, class 2 (thermophilic
119718957	hypothetical protein Tpen_0039	No	4			28180	28614	-		Thermofilum_pendens_Hrk_5_uid58563	NC_008698	chromosome										
===									6	Thermofilum_pendens_Hrk_5_uid58563	NC_008698	chromosome										
119718992	hypothetical protein Tpen_0074	Yes	1			57954	58676	-		Thermofilum_pendens_Hrk_5_uid58563	NC_008698	chromosome										
119718993	hypothetical protein Tpen_0075	No	0			58722	58985	-		Thermofilum_pendens_Hrk_5_uid58563	NC_008698	chromosome										
119718994	6-phosphogluconate dehydrogenase, NAD-bir	No	0			59056	59916	+		Thermofilum_pendens_Hrk_5_uid58563	NC_008698	chromosome		arCOG00247	1	(-)	I	MmsB	3-hydroxyisobutyrate dehydrogenase or related I	3E-42	COG2084	3-hydroxyisobutyrate dehydrogenase and r
119718995	hypothetical protein Tpen_0077	No	0			59913	60965	-		Thermofilum_pendens_Hrk_5_uid58563	NC_008698	chromosome										
119718996	hypothetical protein Tpen_0078	No	0			60972	61805	-		Thermofilum_pendens_Hrk_5_uid58563	NC_008698	chromosome										
119718997	hypothetical protein Tpen_0079	No	0			61805	62611	-		Thermofilum_pendens_Hrk_5_uid58563	NC_008698	chromosome										
===									11	Thermofilum_pendens_Hrk_5_uid58563	NC_008698	chromosome										
119719578	AsnC family transcriptional regulator	No	0			619138	619365	-		Thermofilum_pendens_Hrk_5_uid58563	NC_008698	chromosome		arCOG01117	5	(-)	K	Lrp	Lrp/AsnC family C-terminal domain			
119719579	hypothetical protein Tpen_0669	No	6			619709	620809	-		Thermofilum_pendens_Hrk_5_uid58563	NC_008698	chromosome										
119719580	hypothetical protein Tpen_0670	Yes	1			620827	621201	-		Thermofilum_pendens_Hrk_5_uid58563	NC_008698	chromosome										
119719581	hypothetical protein Tpen_0671	Yes	0			621323	621577	+		Thermofilum_pendens_Hrk_5_uid58563	NC_008698	chromosome										
119719582	hypothetical protein Tpen_0672	Yes	1			621585	624596	+		Thermofilum_pendens_Hrk_5_uid58563	NC_008698	chromosome										
119719583	hypothetical protein Tpen_0673	Yes	1			624578	625138	+		Thermofilum_pendens_Hrk_5_uid58563	NC_008698	chromosome										
119719584	hypothetical protein Tpen_0674	Yes	1			625125	625385	+		Thermofilum_pendens_Hrk_5_uid58563	NC_008698	chromosome										
119719585	hypothetical protein Tpen_0675	No	1			625385	625651	+		Thermofilum_pendens_Hrk_5_uid58563	NC_008698	chromosome										
119719586	hypothetical protein Tpen_0676	Yes	0			625648	626214	+		Thermofilum_pendens_Hrk_5_uid58563	NC_008698	chromosome										
119719587	hypothetical protein Tpen_0677	No	1			626215	626571	+		Thermofilum_pendens_Hrk_5_uid58563	NC_008698	chromosome										
119719588	TGS domain-containing protein	No	0			626568	627701	-		Thermofilum_pendens_Hrk_5_uid58563	NC_008698	chromosome		arCOG00358	1	(-)	R	DRG	Predicted GTPase	9E-65	COG1163	Predicted GTPase
===									7	Thermofilum_pendens_Hrk_5_uid58563	NC_008698	chromosome										
119719630	hypothetical protein Tpen_0720	No	0			669256	670311	-		Thermofilum_pendens_Hrk_5_uid58563	NC_008698	chromosome		arCOG00281	4	(-)	R		Predicted ATPase	9E-10	COG0433	HerA helicase
119719631	hypothetical protein Tpen_0721	Yes	0			670308	671372	-		Thermofilum_pendens_Hrk_5_uid58563	NC_008698	chromosome										
119719632	hypothetical protein Tpen_0722	No	5			671365	673962	-		Thermofilum_pendens_Hrk_5_uid58563	NC_008698	chromosome										
119719633	hypothetical protein Tpen_0723	No	5			673959	674732	-		Thermofilum_pendens_Hrk_5_uid58563	NC_008698	chromosome										
119719634	hypothetical protein Tpen_0724	No	2			674746	675270	-		Thermofilum_pendens_Hrk_5_uid58563	NC_008698	chromosome										
119719635	chromosome partitioning ATPase protein-like	No	0			675371	676084	+		Thermofilum_pendens_Hrk_5_uid58563	NC_008698	chromosome		arCOG00589	2	(-)	R		CobQ/CobB/MinD/ParA ATPase	1E-10	COG0455	Antiactivator of flagellar biosynthesis
119719636	hypothetical protein Tpen_0726	No	0			676081	676437	+		Thermofilum_pendens_Hrk_5_uid58563	NC_008698	chromosome										
===									7	Thermofilum_pendens_Hrk_5_uid58563	NC_008698	chromosome										
119719708	phage SPO1 DNA polymerase-related protein	No	0			752550	753203	+		Thermofilum_pendens_Hrk_5_uid58563	NC_008698	chromosome		arCOG00905	2	(-)	L		Uracil-DNA glycosylase	2E-44	COG1573	Uracil-DNA glycosylase
119719709	hypothetical protein Tpen_0799	No	2			753398	754012	+		Thermofilum_pendens_Hrk_5_uid58563	NC_008698	chromosome										
119719710	hypothetical protein Tpen_0800	No	0			754439	754831	+		Thermofilum_pendens_Hrk_5_uid58563	NC_008698	chromosome										
119719711	hypothetical protein Tpen_0801	No	0			754821	755564	-		Thermofilum_pendens_Hrk_5_uid58563	NC_008698	chromosome								2E-10	COG1196	Chromosome segregation ATPases
119719712	hypothetical protein Tpen_0802	No	0			755652	755825	-		Thermofilum_pendens_Hrk_5_uid58563	NC_008698	chromosome										
119719713	hypothetical protein Tpen_0804	No	0			757603	758406	+		Thermofilum_pendens_Hrk_5_uid58563	NC_008698	chromosome										
119719714	type II secretion system protein	No	5			758576	759463	+		Thermofilum_pendens_Hrk_5_uid58563	NC_008698	chromosome		arCOG01811	1	(-)	N	TadC	Flp pilus assembly protein TadC	6E-11	COG2064	Flp pilus assembly protein TadC
===									8	Thermofilum_pendens_Hrk_5_uid58563	NC_008698	chromosome										
119719826	hypothetical protein Tpen_0917	No	1			863700	866078	-		Thermofilum_pendens_Hrk_5_uid58563	NC_008698	chromosome										
119719827	hypothetical protein Tpen_0918	No	1			866085	866585	-		Thermofilum_pendens_Hrk_5_uid58563	NC_008698	chromosome		arCOG10502	4		S		Uncharacterized conserved protein			
119719828	hypothetical protein Tpen_0919	No	1			866596	867831	-		Thermofilum_pendens_Hrk_5_uid58563	NC_008698	chromosome		arCOG05475	4	(-)	S		Uncharacterized conserved protein			
119719829	hypothetical protein Tpen_0920	No	1			867896	868381	-		Thermofilum_pendens_Hrk_5_uid58563	NC_008698	chromosome										
119719830	hypothetical protein Tpen_0921	No	1			868412	869251	-		Thermofilum_pendens_Hrk_5_uid58563	NC_008698	chromosome										
119719831	hypothetical protein Tpen_0922	Yes	2			869270	870808	-		Thermofilum_pendens_Hrk_5_uid58563	NC_008698	chromosome										
119719832	PP-loop domain-containing protein	No	0			870998	871984	-		Thermofilum_pendens_Hrk_5_uid58563	NC_008698	chromosome		arCOG00042	4	(-)	J	MesJ	tRNA(Ile)-lysidine synthase MesJ	5E-34	COG0037	tRNA(Ile)-lysidine synthase MesJ
119719833	thiamineS protein	No	0			871998	872219	-		Thermofilum_pendens_Hrk_5_uid58563	NC_008698	chromosome								0.0000003	pfam02597	ThiS family. ThiS (thiaminS) is a 66 aa
===									6	Thermofilum_pendens_Hrk_5_uid58563	NC_008698	chromosome										
119719870	hypothetical protein Tpen_0962	No	0	YES		913240	913404	+		Thermofilum_pendens_Hrk_5_uid58563	NC_008698	chromosome		arCOG07831	2		S		Uncharacterized conserved protein			
119719871	hypothetical protein Tpen_0963	No	0	YES		913469	914344	+		Thermofilum_pendens_Hrk_5_uid58563	NC_008698	chromosome										
119719872	hypothetical protein Tpen_0964	No	0	YES		914482	916284	+		Thermofilum_pendens_Hrk_5_uid58563	NC_008698	chromosome		arCOG04708	2	(-)	V		ATP-dependent helicase fused to N-terminal NEF	3E-27	pfam09848	Uncharacterized conserved protein (DUF20
119719873	hypothetical protein Tpen_0965	No	0	YES		916356	916730	+		Thermofilum_pendens_Hrk_5_uid58563	NC_008698	chromosome										
119719874	hypothetical protein Tpen_0966	No	1	YES		916944	919310	+		Thermofilum_pendens_Hrk_5_uid58563	NC_008698	chromosome										
119719875	hypothetical protein Tpen_0967	No	0	YES		919463	919657	+		Thermofilum_pendens_Hrk_5_uid58563	NC_008698	chromosome										
===									9	Thermofilum_pendens_Hrk_5_uid58563	NC_008698	chromosome										
119719925	DNA repair and recombination protein RadA	No	0			962414	963490	-		Thermofilum_pendens_Hrk_5_uid58563	NC_008698	chromosome		arCOG00415	7	(-)	L	RecA	RecA/RadA recombinase	6E-79	pfam08423	Rad51. Rad51 is a DNA repair and recombi
119719926	hypothetical protein Tpen_1018	No	0			963502	964170	+		Thermofilum_pendens_Hrk_5_uid58563	NC_008698	chromosome										
119719927	hypothetical protein Tpen_1019	No	0			964237	965271	+		Thermofilum_pendens_Hrk_5_uid58563	NC_008698	chromosome										
119719928	hypothetical protein Tpen_1020	No	0			965268	965708	-		Thermofilum_pendens_Hrk_5_uid58563	NC_008698	chromosome										
119719929	hypothetical protein Tpen_1021	No	3			965828	966163	-		Thermofilum_pendens_Hrk_5_uid58563	NC_008698	chromosome		arCOG06137	1		S		Uncharacterized conserved protein	0.00002	pfam14018	Domain of unknown function (DUF4234). Th
119719930	hypothetical protein Tpen_1022	No	0			966203	966673	-		Thermofilum_pendens_Hrk_5_uid58563	NC_008698	chromosome										
119719931	Rad51-like	No	0			966677	967624	-		Thermofilum_pendens_Hrk_5_uid58563	NC_008698	chromosome		arCOG00415	7	(-)	L	RecA	RecA/RadA recombinase	3E-54	pfam08423	Rad51. Rad51 is a DNA repair and recombi
119719932	hypothetical protein Tpen_1024	No	0			967608	968177	-		Thermofilum_pendens_Hrk_5_uid58563	NC_008698	chromosome										
119719933	hypothetical protein Tpen_1025	No	0			968174	968575	-		Thermofilum_pendens_Hrk_5_uid58563	NC_008698	chromosome										
===									6	Thermofilum_pendens_Hrk_5_uid58563	NC_008698	chromosome										
119720003	oxidoreductase domain-containing protein	No	0			1034552	1035664	-		Thermofilum_pendens_Hrk_5_uid58563	NC_008698	chromosome		arCOG01622	3	(-)	R	MviM	Predicted dehydrogenase	4E-33	COG0673	Predicted dehydrogenases and related pro
119720004	PTS system sorbose subfamily IIB component	No	0			1035674	1036177	-		Thermofilum_pendens_Hrk_5_uid58563	NC_008698	chromosome								1E-25	COG3444	Phosphotransferase system, mannose/fruct
119720005	PTS system fructose subfamily IIA component	No	0			1036219	1036608	-		Thermofilum_pendens_Hrk_5_uid58563	NC_008698	chromosome								0.000000001	COG2893	Phosphotransferase system, mannose/fruct
119720006	hypothetical protein Tpen_1099	No	0			1036656	1037540	-		Thermofilum_pendens_Hrk_5_uid58563	NC_008698	chromosome										
119720007	PTS system mannose/fructose/sorbose family	No	9			1037629	1039233	+		Thermofilum_pendens_Hrk_5_uid58563	NC_008698	chromosome								7E-71	pfam03613	PTS system mannose/fructose/sorbose fami ; PTS system sorbose-specific iic componen
119720008	HAD family hydrolase	No	0			1039293	1039979	-		Thermofilum_pendens_Hrk_5_uid58563	NC_008698	chromosome		arCOG01213	2	(-)	R	Cof	HAD superfamily hydrolase	5E-18	COG0561	Predicted hydrolases of the HAD superfam
===									7	Thermofilum_pendens_Hrk_5_uid58563	NC_008698	chromosome										
119720184	hypothetical protein Tpen_1278	No	0	YES		1217145	1218128	-		Thermofilum_pendens_Hrk_5_uid58563	NC_008698	chromosome										
119720185	queuine tRNA-ribosyltransferase	No	0			1218264	1219022	+		Thermofilum_pendens_Hrk_5_uid58563	NC_008698	chromosome		arCOG00990	1	(-)	J		Queuine tRNA-ribosyltransferase, contain PUA dc	0.00000001	COG1549	Queuine tRNA-ribosyltransferases, contai
119720186	hypothetical protein Tpen_1280	No	2			1220106	1220594	-		Thermofilum_pendens_Hrk_5_uid58563	NC_008698	chromosome										
119720187	hypothetical protein Tpen_1281	No	0			1220622	1220717	-		Thermofilum_pendens_Hrk_5_uid58563	NC_008698	chromosome										
119720188	hypothetical protein Tpen_1282	No	0			1220854	1221183	-		Thermofilum_pendens_Hrk_5_uid58563	NC_008698	chromosome										
119720189	hypothetical protein Tpen_1283	Yes	0			1221180	1221527	-		Thermofilum_pendens_Hrk_5_uid58563	NC_008698	chromosome										
119720190	ankyrin	No	0			1222478	1225090	-		Thermofilum_pendens_Hrk_5_uid58563	NC_008698	chromosome	arCOG04004,a	arCOG04004	1	(-)	R	Arp	Ankyrin repeats containing protein	4E-26	COG0666	FOG: Ankyrin repeat
===									6	Thermofilum_pendens_Hrk_5_uid58563	NC_008698	chromosome										
119720231	hypothetical protein Tpen_1326	No	0	YES		1279404	1279880	-		Thermofilum_pendens_Hrk_5_uid58563	NC_008698	chromosome										
119720232	hypothetical protein Tpen_1327	No	0	YES		1279881	1281731	-		Thermofilum_pendens_Hrk_5_uid58563	NC_008698	chromosome										
119720233	ABC transporter related	No	0	YES		1282813	1283400	-		Thermofilum_pendens_Hrk_5_uid58563	NC_008698	chromosome		arCOG07131	7	(-)	V	CcmA	ABC-type multidrug transport system, ATPase co	1E-19	COG1121	ABC-type Mn/Zn transport systems, ATPase
119720234	hypothetical protein Tpen_1329	No	3	YES		1283402	1283830	-		Thermofilum_pendens_Hrk_5_uid58563	NC_008698	chromosome										
119720235	hypothetical protein Tpen_1330	No	10	YES		1283820	1284986	-		Thermofilum_pendens_Hrk_5_uid58563	NC_008698	chromosome										
119720236	hypothetical protein Tpen_1331	No	3	YES		1285032	1285493	-		Thermofilum_pendens_Hrk_5_uid58563	NC_008698	chromosome										
===									5	Thermofilum_pendens_Hrk_5_uid58563	NC_008698	chromosome										
119720634	Fis family transcriptional regulator	Yes	2			1675728	1676939	-		Thermofilum_pendens_Hrk_5_uid58563	NC_008698	chromosome										
119720635	hypothetical protein Tpen_1732	No	2			1677060	1677335	-		Thermofilum_pendens_Hrk_5_uid58563	NC_008698	chromosome		arCOG04189	2	(-)	S		Membrane protein	3E-10	COG5625	Predicted transcription regulator contai
119720636	hypothetical protein Tpen_1733	No	1	YES		1677407	1677700	-		Thermofilum_pendens_Hrk_5_uid58563	NC_008698	chromosome								0.00000004	pfam05557	Mitotic checkpoint protein. This family
119720637	hypothetical protein Tpen_1734	No	0	YES		1677754	1678038	-		Thermofilum_pendens_Hrk_5_uid58563	NC_008698	chromosome										
119720638	hypothetical protein Tpen_1735	No	0	YES		1678078	1678350	-		Thermofilum_pendens_Hrk_5_uid58563	NC_008698	chromosome										
===									5	Thermogladius_1633_uid167488	NC_017954	chromosome										
389860764	hypothetical protein TCELL_0441	No	3			402308	402559	-		Thermogladius_1633_uid167488	NC_017954	chromosome	clust_52									
389860765	hypothetical protein TCELL_0442	No	2			402561	403205	-		Thermogladius_1633_uid167488	NC_017954	chromosome										
389860766	hypothetical protein TCELL_0443	No	4			403217	403864	-		Thermogladius_1633_uid167488	NC_017954	chromosome										
389860767	methanol dehydrogenase regulatory protein (m	No	0			403875	404849	-		Thermogladius_1633_uid167488	NC_017954	chromosome		arCOG00434	3	(-)	R		MoxR-like ATPase	3E-68	COG0714	MoxR-like ATPases
389860768	hypothetical protein TCELL_0445	Yes	2			404882	405328	-		Thermogladius_1633_uid167488	NC_017954	chromosome										
===									5	Thermogladius_1633_uid167488	NC_017954	chromosome										
389861182	hypothetical protein TCELL_0860	No	0			793247	794101	-		Thermogladius_1633_uid167488	NC_017954	chromosome										
389861183	hypothetical protein TCELL_0861	No	0			794116	795765	-		Thermogladius_1633_uid167488	NC_017954	chromosome		arCOG06103	2	(-)	V		Predicted viral immunity protein, COG1517 superfamily, contains C-terminal PIN domain			
389861184	hypothetical protein TCELL_0862	No	0			795823	796086	-		Thermogladius_1633_uid167488	NC_017954	chromosome										
389861185	hypothetical protein TCELL_0863	No	0			796222	796518	+		Thermogladius_1633_uid167488	NC_017954	chromosome										
389861186	hypothetical protein TCELL_0864	No	1			796569	797285	+		Thermogladius_1633_uid167488	NC_017954	chromosome										
===									5	Thermogladius_1633_uid167488	NC_017954	chromosome										
389861352	hypothetical protein TCELL_1031	No	0			960335	960466	+		Thermogladius_1633_uid167488	NC_017954	chromosome										
389861353	hypothetical protein TCELL_1032	No	0			960594	961991	+		Thermogladius_1633_uid167488	NC_017954	chromosome										
389861354	hypothetical protein TCELL_1033	No	0			962020	962178	-		Thermogladius_1633_uid167488	NC_017954	chromosome										
389861355	aldo/keto reductase	No	0			962486	963484	-		Thermogladius_1633_uid167488	NC_017954	chromosome		arCOG01618	1	(-)	R	ARA1	Aldo/keto reductase, related to diketogulonate re	2E-50	COG0667	Predicted oxidoreductases (related to ar
389861356	hypothetical protein TCELL_1035	No	0			963525	963686	-		Thermogladius_1633_uid167488	NC_017954	chromosome										
===									7	Thermoplasma_acidophilum_DSM_1728_uid6157	NC_002578	chromosome										
16081495	hypothetical protein Ta0366	No	0			385601	386044	-		Thermoplasma_acidophilum_DSM_1728_uid6157	NC_002578	chromosome										
16081496	hypothetical protein Ta0367	No	1			386297	388153	+		Thermoplasma_acidophilum_DSM_1728_uid6157	NC_002578	chromosome		arCOG05334	2	(-)	L		Predicted DEAD/DEAH box helicase, fused to PD-(DE)xK superfamily endonuclease			
16081497	hypothetical protein Ta0368	No	2			388210	388854	+		Thermoplasma_acidophilum_DSM_1728_uid6157	NC_002578	chromosome										
16081498	hypothetical protein Ta0369	No	3			389058	389438	-		Thermoplasma_acidophilum_DSM_1728_uid6157	NC_002578	chromosome										
16081499	hypothetical protein Ta0370	No	1			389390	389791	-		Thermoplasma_acidophilum_DSM_1728_uid6157	NC_002578	chromosome										
16081500	hypothetical protein Ta0371	No	0			392567	393628	+		Thermoplasma_acidophilum_DSM_1728_uid6157	NC_002578	chromosome										
16081501	hypothetical protein Ta0372	No	0			393792	394751	+		Thermoplasma_acidophilum_DSM_1728_uid6157	NC_002578	chromosome		arCOG00288	8	(-)	C	NfnB	Nitroreductase			
===									5	Thermoplasma_acidophilum_DSM_1728_uid6157	NC_002578	chromosome										
16082059	hypothetical protein Ta1025	No	1			1097508	1098068	-		Thermoplasma_acidophilum_DSM_1728_uid6157	NC_002578	chromosome										
16082060	hypothetical protein Ta1026	No	6			1098124	1098852	-		Thermoplasma_acidophilum_DSM_1728_uid6157	NC_002578	chromosome										
16082061	ABC transporter ATP-binding protein	No	0			1098845	1099714	-		Thermoplasma_acidophilum_DSM_1728_uid6157	NC_002578	chromosome		arCOG00208	5	(-)	V	CcmA	ABC-type multidrug transport system, ATPase co	7E-30	COG1131	ABC-type multidrug transport system, ATP
16082062	hypothetical protein Ta1028	Yes	3			1099716	1100543	-		Thermoplasma_acidophilum_DSM_1728_uid6157	NC_002578	chromosome		arCOG11047	1		S		Uncharacterized conserved protein			
16082063	hypothetical protein Ta1029	No	1			1100715	1101392	-		Thermoplasma_acidophilum_DSM_1728_uid6157	NC_002578	chromosome										
===									6	Thermoplasma_acidophilum_DSM_1728_uid6157	NC_002578	chromosome										
16082362	hypothetical protein Ta1385	No	1			1429670	1431520	-		Thermoplasma_acidophilum_DSM_1728_uid6157	NC_002578	chromosome		arCOG05967	1	(-)	S		Uncharacterized conserved protein	3E-16	pfam12222	Peptide N-acetyl-beta-D-glucosaminyl asp
16082363	hypothetical protein Ta1386	No	0			1431956	1432756	+		Thermoplasma_acidophilum_DSM_1728_uid6157	NC_002578	chromosome										
16082364	hypothetical protein Ta1387	No	0			1433158	1434246	+		Thermoplasma_acidophilum_DSM_1728_uid6157	NC_002578	chromosome										
16082365	hypothetical protein Ta1388	No	0			1434396	1435001	-		Thermoplasma_acidophilum_DSM_1728_uid6157	NC_002578	chromosome										
16082366	hypothetical protein Ta1389	No	0			1435478	1436209	+		Thermoplasma_acidophilum_DSM_1728_uid6157	NC_002578	chromosome										
16082367	hypothetical protein Ta1390	No	0			1435789	1437066	-		Thermoplasma_acidophilum_DSM_1728_uid6157	NC_002578	chromosome		arCOG01355	1	(-)	C		Radical SAM superfamily enzyme			
===									5	Thermoplasma_volcanium_GSS1_uid57751	NC_002689	chromosome										
13540974	hypothetical protein TVN0143	No	4			149208	149603	+		Thermoplasma_volcanium_GSS1_uid57751	NC_002689	chromosome		arCOG06951	3		S		Uncharacterized conserved protein			
13540975	hypothetical protein TVN0144	No	3			149832	150464	+		Thermoplasma_volcanium_GSS1_uid57751	NC_002689	chromosome		arCOG05354	1		S		Uncharacterized conserved protein			
13540976	hypothetical protein TVN0145	No	0			151549	152070	+		Thermoplasma_volcanium_GSS1_uid57751	NC_002689	chromosome										
13540977	Na+/proline symporter permease	No	13			152244	153692	-		Thermoplasma_volcanium_GSS1_uid57751	NC_002689	chromosome		arCOG01316	1	(-)	E	PutP	Na+/proline symporter	1E-55	COG0591	Na+/proline symporter
13540978	hypothetical protein TVN0147	No	2			153694	153888	-		Thermoplasma_volcanium_GSS1_uid57751	NC_002689	chromosome		arCOG11300	1		S		Uncharacterized conserved protein	0.000000004	pfam11755	Protein of unknown function (DUF3311). T
===									6	Thermoplasma_volcanium_GSS1_uid57751	NC_002689	chromosome										
13541528	hypothetical protein TVN0697	No	0			702334	702900	-		Thermoplasma_volcanium_GSS1_uid57751	NC_002689	chromosome										
13541529	hypothetical protein TVN0698	No	0			703424	703840	-		Thermoplasma_volcanium_GSS1_uid57751	NC_002689	chromosome										
13541530	hypothetical protein TVN0699	No	0			703837	704973	-		Thermoplasma_volcanium_GSS1_uid57751	NC_002689	chromosome		arCOG06915	3	(-)	L		Primase-polymerase (primpol) domain			
13541531	hypothetical protein TVN0700	No	0			705358	705711	-		Thermoplasma_volcanium_GSS1_uid57751	NC_002689	chromosome										
13541532	hypothetical protein TVN0701	No	0			705804	706346	-		Thermoplasma_volcanium_GSS1_uid57751	NC_002689	chromosome										
13541533	hypothetical protein TVN0702	No	0			707052	708014	+		Thermoplasma_volcanium_GSS1_uid57751	NC_002689	chromosome										
===									9	Thermoplasma_volcanium_GSS1_uid57751	NC_002689	chromosome										
13542297	AAA family ATPase	No	0	YES		1545079	1545912	-		Thermoplasma_volcanium_GSS1_uid57751	NC_002689	chromosome		arCOG00467	13	(-)	L	CDC6	Cdc6-related protein, AAA superfamily ATPase	4E-16	COG1474	Cdc6-related protein, AAA superfamily AT
13542298	hypothetical protein TVN1467	No	2	YES		1547379	1547906	-		Thermoplasma_volcanium_GSS1_uid57751	NC_002689	chromosome	clust_37									
13542299	hypothetical protein TVN1468	No	0	YES		1548174	1548359	+		Thermoplasma_volcanium_GSS1_uid57751	NC_002689	chromosome								0.00002	COG3328	Transposase and inactivated derivatives
13542300	hypothetical protein TVN1469	No	1	YES		1548379	1548519	-		Thermoplasma_volcanium_GSS1_uid57751	NC_002689	chromosome										
13542301	hypothetical protein TVN1470	No	3	YES		1548715	1549047	-		Thermoplasma_volcanium_GSS1_uid57751	NC_002689	chromosome		arCOG06951	3		S		Uncharacterized conserved protein			
13542302	hypothetical protein TVN1471	No	0	YES		1549136	1550017	-		Thermoplasma_volcanium_GSS1_uid57751	NC_002689	chromosome		arCOG07686	2		S		Uncharacterized conserved protein			
13542303	hypothetical protein TVN1472	No	2	YES		1550441	1550650	-		Thermoplasma_volcanium_GSS1_uid57751	NC_002689	chromosome										
13542304	hypothetical protein TVN1473	No	0	YES		1550926	1551546	-		Thermoplasma_volcanium_GSS1_uid57751	NC_002689	chromosome		arCOG07527	5	(-)	S		Predicted metal-binding protein			
13542305	hypothetical protein TVN1474	No	0	YES		1552310	1552486	+		Thermoplasma_volcanium_GSS1_uid57751	NC_002689	chromosome		arCOG06123	1		S		Uncharacterized conserved protein			
===									8	Thermoplasma_volcanium_GSS1_uid57751	NC_002689	chromosome										
13542312	ATPase	No	0	YES		1561939	1563720	-		Thermoplasma_volcanium_GSS1_uid57751	NC_002689	chromosome		arCOG05334	2	(-)	L		Predicted DEAD/DEAH box helicase, fused to PD-(DE)xK superfamily endonuclease			
13542313	hypothetical protein TVN1482	No	3	YES		1563727	1564062	-		Thermoplasma_volcanium_GSS1_uid57751	NC_002689	chromosome										
13542314	hypothetical protein TVN1483	No	0	YES		1564065	1564430	-		Thermoplasma_volcanium_GSS1_uid57751	NC_002689	chromosome										
13542315	hypothetical protein TVN1484	No	0	YES		1564647	1565306	-		Thermoplasma_volcanium_GSS1_uid57751	NC_002689	chromosome										
13542316	integrase/recombinase	No	0	YES		1565306	1565548	-		Thermoplasma_volcanium_GSS1_uid57751	NC_002689	chromosome										
13542317	hypothetical protein TVN1486	No	2	YES		1565673	1565885	-		Thermoplasma_volcanium_GSS1_uid57751	NC_002689	chromosome										
13542318	hypothetical protein TVN1487	No	2	YES		1566346	1566873	-		Thermoplasma_volcanium_GSS1_uid57751	NC_002689	chromosome	clust_37									
13542319	hypothetical protein TVN1488	No	0	YES		1566919	1567560	-		Thermoplasma_volcanium_GSS1_uid57751	NC_002689	chromosome		arCOG07527	5	(-)	S		Predicted metal-binding protein			
===									7	Thermoproteus_tenax_Kra_1_uid74443	NC_016070	chromosome										
352681474	Rossmann fold nucleotide-binding protein	No	0			229144	229998	+		Thermoproteus_tenax_Kra_1_uid74443	NC_016070	chromosome		arCOG02431	4	(-)	R		Predicted Rossmann fold nucleotide-binding prot	4E-26	pfam02481	DNA recombination-mediator protein A. Th
352681475	amidophosphoribosyltransferase ComF	No	0			229982	230743	+		Thermoproteus_tenax_Kra_1_uid74443	NC_016070	chromosome								8E-15	COG1040	Predicted amidophosphoribosyltransferase
352681476	hypothetical protein TTX_0244	No	0			230859	231092	+		Thermoproteus_tenax_Kra_1_uid74443	NC_016070	chromosome										
352681477	proteasome-type protease	Yes	0			231089	231883	+		Thermoproteus_tenax_Kra_1_uid74443	NC_016070	chromosome		arCOG09992	2		S		Uncharacterized conserved protein			
352681478	hypothetical protein TTX_0246	No	0			231886	232188	+		Thermoproteus_tenax_Kra_1_uid74443	NC_016070	chromosome										
352681479	hypothetical protein TTX_0247	No	0			232185	233111	+		Thermoproteus_tenax_Kra_1_uid74443	NC_016070	chromosome										
352681480	hypothetical protein TTX_0248	No	0			233121	234449	+		Thermoproteus_tenax_Kra_1_uid74443	NC_016070	chromosome		arCOG04025	2	(-)	L		Competence protein CoiA homolog, contains C-terminal PD-DExK nuclease domain			

===							6	Thermoproteus_tenax_Kra_1_uid74443	NC_016070	chromosome									
352682445	putative ATPase	No	0	1092781	1093578	+		Thermoproteus_tenax_Kra_1_uid74443	NC_016070	chromosome		arCOG06984	1 (-)	P	ZnuC	ABC-type Mn/Zn transport system, ATPase component			
352682446	hypothetical protein TTX_1257	No	0	1093575	1094015	+		Thermoproteus_tenax_Kra_1_uid74443	NC_016070	chromosome		arCOG10104	1	S		Predicted metal-binding protein			
352682447	hypothetical protein TTX_1258	No	0	1094079	1094732	-		Thermoproteus_tenax_Kra_1_uid74443	NC_016070	chromosome									
352682448	hypothetical protein TTX_1259	No	0	1095081	1095236	-		Thermoproteus_tenax_Kra_1_uid74443	NC_016070	chromosome									
352682449	hypothetical protein TTX_1260	No	0	1095312	1095479	-		Thermoproteus_tenax_Kra_1_uid74443	NC_016070	chromosome									
352682450	cell suface protein containing PKD-like repeats	Yes	0	1097344	1098615	-		Thermoproteus_tenax_Kra_1_uid74443	NC_016070	chromosome		arCOG02499	1 (-)	S		NosD-like periplasmic protein	0.00000001	pfam05048	Periplasmic copper-binding protein (NosD
===							5	Thermoproteus_tenax_Kra_1_uid74443	NC_016070	chromosome									
352683279	hypothetical protein TTX_2113	No	0	1837178	1837300	-		Thermoproteus_tenax_Kra_1_uid74443	NC_016070	chromosome									
352683280	hypothetical protein TTX_2114	Yes	1	1837539	1837919	-		Thermoproteus_tenax_Kra_1_uid74443	NC_016070	chromosome									
352683281	hypothetical protein TTX_2115	No	4	1837947	1838489	-		Thermoproteus_tenax_Kra_1_uid74443	NC_016070	chromosome									
352683282	hypothetical protein TTX_2116	Yes	0	1838408	1839478	-		Thermoproteus_tenax_Kra_1_uid74443	NC_016070	chromosome		arCOG03962	1 (-)	S		Uncharacterized protein with SCP/PR1 domains	0.000005	COG2340	Uncharacterized protein with SCP/PR1 dom
352683283	hypothetical protein TTX_2117	No	0	1840167	1841048	-		Thermoproteus_tenax_Kra_1_uid74443	NC_016070	chromosome		arCOG07029	2	S		Uncharacterized conserved protein			
===							9	Thermoproteus_uzoniensis_768_20_uid65089	NC_015315	chromosome									
327310820	hypothetical protein TUZN_0922	No	0	803306	804835	+		Thermoproteus_uzoniensis_768_20_uid65089	NC_015315	chromosome	arCOG01694,a	arCOG01694	1 (-)	T		FHA domain and HTH domain	3E-10	COG2304	Uncharacterized protein containing a von ; FOG: FHA domain
327310821	hypothetical protein TUZN_0923	Yes	1	804891	808055	+		Thermoproteus_uzoniensis_768_20_uid65089	NC_015315	chromosome									
327310822	hypothetical protein TUZN_0924	No	1	808057	808668	-		Thermoproteus_uzoniensis_768_20_uid65089	NC_015315	chromosome									
327310823	hypothetical protein TUZN_0925	No	1	808665	809474	-		Thermoproteus_uzoniensis_768_20_uid65089	NC_015315	chromosome									
327310824	hypothetical protein TUZN_0926	No	1	809471	809866	-		Thermoproteus_uzoniensis_768_20_uid65089	NC_015315	chromosome									
327310825	hypothetical protein TUZN_0927	No	1	809863	810348	-		Thermoproteus_uzoniensis_768_20_uid65089	NC_015315	chromosome									
327310826	hypothetical protein TUZN_0928	No	1	810376	810903	-		Thermoproteus_uzoniensis_768_20_uid65089	NC_015315	chromosome									
327310827	hypothetical protein TUZN_0929	No	1	810905	811477	-		Thermoproteus_uzoniensis_768_20_uid65089	NC_015315	chromosome									
327310828	hypothetical protein TUZN_0930	No	1	811477	812019	-		Thermoproteus_uzoniensis_768_20_uid65089	NC_015315	chromosome		arCOG03871	6 (-)	N		Flagellar protein G			
===							8	Thermosphaera_aggregans_DSM_11486_uid489	NC_014160	chromosome									
296242002	Na+/solute symporter	No	2	232919	233116	-		Thermosphaera_aggregans_DSM_11486_uid489	NC_014160	chromosome									
296242003	hypothetical protein Tagg_0260	No	2	233122	233292	-		Thermosphaera_aggregans_DSM_11486_uid489	NC_014160	chromosome		arCOG11065	1	S		Uncharacterized conserved protein			
296242004	transcriptional regulator protein	No	0	233371	234381	+		Thermosphaera_aggregans_DSM_11486_uid489	NC_014160	chromosome		arCOG01804	1 (-)	K		Predicted transcriptional regulator	3E-31	COG3888	Predicted transcriptional regulator
296242005	hypothetical protein Tagg_0262	No	4	234664	235131	-		Thermosphaera_aggregans_DSM_11486_uid489	NC_014160	chromosome									
296242006	hypothetical protein Tagg_0263	No	1	235100	236170	-		Thermosphaera_aggregans_DSM_11486_uid489	NC_014160	chromosome		arCOG07686	2	S		Uncharacterized conserved protein			
296242007	hypothetical protein Tagg_0264	No	0	236184	236282	+		Thermosphaera_aggregans_DSM_11486_uid489	NC_014160	chromosome									
296242008	hypothetical protein Tagg_0265	No	0	236627	237361	+		Thermosphaera_aggregans_DSM_11486_uid489	NC_014160	chromosome		arCOG00367	3 (-)	L		NurA 5'-3' nuclease			
296242009	hypothetical protein Tagg_0266	No	0	237561	237926	+		Thermosphaera_aggregans_DSM_11486_uid489	NC_014160	chromosome									
===							6	Thermosphaera_aggregans_DSM_11486_uid489	NC_014160	chromosome									
296242320	4-oxalocrotonate tautomerase	No	0	555553	555756	-		Thermosphaera_aggregans_DSM_11486_uid489	NC_014160	chromosome		arCOG02240	1 (-)	R		4-oxalocrotonate tautomerase related protein	5E-11	pfam01361	Tautomerase enzyme. This family includes
296242321	hypothetical protein Tagg_0583	No	14	555793	557751	-		Thermosphaera_aggregans_DSM_11486_uid489	NC_014160	chromosome		arCOG07752	1	S		Uncharacterized conserved protein			
296242322	hypothetical protein Tagg_0584	No	1	558197	558469	-		Thermosphaera_aggregans_DSM_11486_uid489	NC_014160	chromosome									
296242323	hypothetical protein Tagg_0585	No	0	558668	558778	-		Thermosphaera_aggregans_DSM_11486_uid489	NC_014160	chromosome									
296242324	hypothetical protein Tagg_0586	No	0	558831	559031	-		Thermosphaera_aggregans_DSM_11486_uid489	NC_014160	chromosome									
296242325	family 2 glycosyltransferase	No	2	559098	560072	-		Thermosphaera_aggregans_DSM_11486_uid489	NC_014160	chromosome		arCOG00894	2 (-)	M	WcaA	Glycosyltransferase	4E-11	pfam00535	Glycosyl transferase family 2. Diverse f
===							11	Thermosphaera_aggregans_DSM_11486_uid489	NC_014160	chromosome									
296242474	hypothetical protein Tagg_0736	No	0	695270	696139	+		Thermosphaera_aggregans_DSM_11486_uid489	NC_014160	chromosome									
296242475	hypothetical protein Tagg_0737	No	0	696227	696514	+		Thermosphaera_aggregans_DSM_11486_uid489	NC_014160	chromosome		arCOG06071	1 (-)	S		Uncharacterized conserved protein			
296242476	hypothetical protein Tagg_0738	No	0	696614	697243	+		Thermosphaera_aggregans_DSM_11486_uid489	NC_014160	chromosome									
296242477	hypothetical protein Tagg_0739	Yes	2	697307	697756	+		Thermosphaera_aggregans_DSM_11486_uid489	NC_014160	chromosome									
296242478	exosortase EpsH-like protein	No	10	697840	699519	+		Thermosphaera_aggregans_DSM_11486_uid489	NC_014160	chromosome							0.00008	pfam09721	Transmembrane exosortase (Exosortase_Eps
296242479	ATPase	No	0	699509	700465	+		Thermosphaera_aggregans_DSM_11486_uid489	NC_014160	chromosome		arCOG00434	3 (-)	R		MoxR-like ATPase	4E-70	COG0714	MoxR-like ATPases
296242480	hypothetical protein Tagg_0742	No	5	700472	701122	+		Thermosphaera_aggregans_DSM_11486_uid489	NC_014160	chromosome									
296242481	hypothetical protein Tagg_0743	No	2	701119	701805	+		Thermosphaera_aggregans_DSM_11486_uid489	NC_014160	chromosome									
296242482	hypothetical protein Tagg_0744	No	3	701802	702056	+		Thermosphaera_aggregans_DSM_11486_uid489	NC_014160	chromosome	clust_52								
296242483	hypothetical protein Tagg_0745	Yes	2	702053	703831	+		Thermosphaera_aggregans_DSM_11486_uid489	NC_014160	chromosome									
296242484	hypothetical protein Tagg_0746	No	2	703818	704651	+		Thermosphaera_aggregans_DSM_11486_uid489	NC_014160	chromosome		arCOG01314	1 (-)	S		Uncharacterized conserved membrane protein			
===							8	Thermosphaera_aggregans_DSM_11486_uid489	NC_014160	chromosome									
296242780	50S ribosomal protein L40E	No	0	976216	976392	+		Thermosphaera_aggregans_DSM_11486_uid489	NC_014160	chromosome		arCOG04049	2 (-)	J	RPL40A	Ribosomal protein L40E	0.000000002	COG1552	Ribosomal protein L40E
296242781	hypothetical protein Tagg_1047	No	0	976757	977014	+		Thermosphaera_aggregans_DSM_11486_uid489	NC_014160	chromosome									
296242782	hypothetical protein Tagg_1048	No	2	977441	977653	-		Thermosphaera_aggregans_DSM_11486_uid489	NC_014160	chromosome		arCOG10278	1	S		Uncharacterized conserved protein			
296242783	hypothetical protein Tagg_1049	No	4	977693	978112	-		Thermosphaera_aggregans_DSM_11486_uid489	NC_014160	chromosome									
296242784	hypothetical protein Tagg_1050	Yes	0	978109	978438	-		Thermosphaera_aggregans_DSM_11486_uid489	NC_014160	chromosome									
296242785	hypothetical protein Tagg_1051	No	1	978447	978605	-		Thermosphaera_aggregans_DSM_11486_uid489	NC_014160	chromosome									
296242786	hypothetical protein Tagg_1052	No	1	978614	978727	-		Thermosphaera_aggregans_DSM_11486_uid489	NC_014160	chromosome									
296242787	translin	No	0	979187	979792	+		Thermosphaera_aggregans_DSM_11486_uid489	NC_014160	chromosome		arCOG04318	1 (-)	J		Predicted RNA-binding protein of the translin fami	2E-49	COG2178	Predicted RNA-binding protein of the tra
===							14	Vulcanisaeta_distributa_DSM_14429_uid52827	NC_014537	chromosome									
307594253	AAA ATPase	No	0	105200	105988	-		Vulcanisaeta_distributa_DSM_14429_uid52827	NC_014537	chromosome		arCOG05482	1 (-)	T	RAD55	RecA-superfamily ATPase implicated in signal tra	1E-15	COG0467	RecA-superfamily ATPases implicated in s
307594254	hypothetical protein Vdis_0107	No	7	106442	107146	+		Vulcanisaeta_distributa_DSM_14429_uid52827	NC_014537	chromosome									
307594255	hypothetical protein Vdis_0108	No	2	107275	108168	-		Vulcanisaeta_distributa_DSM_14429_uid52827	NC_014537	chromosome									
307594256	hypothetical protein Vdis_0109	No	2	108179	108475	-		Vulcanisaeta_distributa_DSM_14429_uid52827	NC_014537	chromosome									
307594257	hypothetical protein Vdis_0110	No	0	108669	109241	+		Vulcanisaeta_distributa_DSM_14429_uid52827	NC_014537	chromosome									
307594258	FO synthase subunit 1	No	0	109256	109549	+		Vulcanisaeta_distributa_DSM_14429_uid52827	NC_014537	chromosome									
307594259	hypothetical protein Vdis_0112	No	2	109783	110478	-		Vulcanisaeta_distributa_DSM_14429_uid52827	NC_014537	chromosome									
307594260	hypothetical protein Vdis_0113	No	0	110559	110939	-		Vulcanisaeta_distributa_DSM_14429_uid52827	NC_014537	chromosome									
307594261	hypothetical protein Vdis_0114	No	0	111377	111655	-		Vulcanisaeta_distributa_DSM_14429_uid52827	NC_014537	chromosome									
307594262	hypothetical protein Vdis_0115	No	0	111740	111928	+		Vulcanisaeta_distributa_DSM_14429_uid52827	NC_014537	chromosome									
307594263	hypothetical protein Vdis_0116	No	2	112280	113389	-		Vulcanisaeta_distributa_DSM_14429_uid52827	NC_014537	chromosome									
307594264	hypothetical protein Vdis_0117	No	4	113500	114225	-		Vulcanisaeta_distributa_DSM_14429_uid52827	NC_014537	chromosome									
307594265	hypothetical protein Vdis_0118	No	0	114417	114611	+		Vulcanisaeta_distributa_DSM_14429_uid52827	NC_014537	chromosome									
307594266	hypothetical protein Vdis_0119	No	0	114621	115370	-		Vulcanisaeta_distributa_DSM_14429_uid52827	NC_014537	chromosome		arCOG07730	1 (-)	L		DEDD family 3'-5' exonuclease			
===							13	Vulcanisaeta_distributa_DSM_14429_uid52827	NC_014537	chromosome									
307594268	hypothetical protein Vdis_0121	No	0	116630	116860	-		Vulcanisaeta_distributa_DSM_14429_uid52827	NC_014537	chromosome									
307594269	type 11 methyltransferase	No	0	117002	117697	+		Vulcanisaeta_distributa_DSM_14429_uid52827	NC_014537	chromosome		arCOG01773	2 (-)	Q		SAM-dependent methyltransferase	1E-14	pfam08241	Methyltransferase domain. Members of thi
307594270	hypothetical protein Vdis_0123	No	0	117776	117958	-		Vulcanisaeta_distributa_DSM_14429_uid52827	NC_014537	chromosome									
307594271	hypothetical protein Vdis_0124	No	0	118107	118361	-		Vulcanisaeta_distributa_DSM_14429_uid52827	NC_014537	chromosome									
307594272	hypothetical protein Vdis_0125	No	0	118772	118945	-		Vulcanisaeta_distributa_DSM_14429_uid52827	NC_014537	chromosome									
307594273	hypothetical protein Vdis_0126	No	11	119230	121749	-		Vulcanisaeta_distributa_DSM_14429_uid52827	NC_014537	chromosome	clust_66						0.00002	COG1824	Permease, similar to cation transporters
307594274	hypothetical protein Vdis_0127	No	3	121756	122151	-		Vulcanisaeta_distributa_DSM_14429_uid52827	NC_014537	chromosome									
307594275	hypothetical protein Vdis_0128	No	0	122244	123995	-		Vulcanisaeta_distributa_DSM_14429_uid52827	NC_014537	chromosome		arCOG10867	4	S		Uncharacterized conserved protein			
307594276	hypothetical protein Vdis_0129	No	0	124734	125375	-		Vulcanisaeta_distributa_DSM_14429_uid52827	NC_014537	chromosome	clust_159								
307594277	hypothetical protein Vdis_0130	No	0	125909	127285	-		Vulcanisaeta_distributa_DSM_14429_uid52827	NC_014537	chromosome		arCOG00770	3 (-)	K	DinG	Rad3-related DNA helicase			
307594278	hypothetical protein Vdis_0131	No	2	127290	127817	-		Vulcanisaeta_distributa_DSM_14429_uid52827	NC_014537	chromosome	clust_200						0.00004	COG0419	ATPase involved in DNA repair
307594279	hypothetical protein Vdis_0132	No	0	127807	128073	-		Vulcanisaeta_distributa_DSM_14429_uid52827	NC_014537	chromosome									
307594280	hypothetical protein Vdis_0133	No	0	128238	128747	-		Vulcanisaeta_distributa_DSM_14429_uid52827	NC_014537	chromosome	clust_209								
===							29	Vulcanisaeta_distributa_DSM_14429_uid52827	NC_014537	chromosome									
307594284	hypothetical protein Vdis_0137	No	2	136442	138532	-		Vulcanisaeta_distributa_DSM_14429_uid52827	NC_014537	chromosome		arCOG00280	21 (-)	L		HerA helicase	2E-13	pfam12846	AAA-like domain. This family of domains
307594285	hypothetical protein Vdis_0138	No	0	138568	139071	-		Vulcanisaeta_distributa_DSM_14429_uid52827	NC_014537	chromosome		arCOG10944	2	S		Uncharacterized conserved protein			
307594286	hypothetical protein Vdis_0139	No	0	139068	139424	-		Vulcanisaeta_distributa_DSM_14429_uid52827	NC_014537	chromosome	clust_293								
307594287	hypothetical protein Vdis_0140	No	1	139486	139632	-		Vulcanisaeta_distributa_DSM_14429_uid52827	NC_014537	chromosome									
307594288	hypothetical protein Vdis_0141	No	0	139698	139889	+		Vulcanisaeta_distributa_DSM_14429_uid52827	NC_014537	chromosome									
307594289	hypothetical protein Vdis_0142	No	1	139890	140567	-		Vulcanisaeta_distributa_DSM_14429_uid52827	NC_014537	chromosome									
307594290	hypothetical protein Vdis_0143	No	0	140624	141442	-		Vulcanisaeta_distributa_DSM_14429_uid52827	NC_014537	chromosome		arCOG00589	2 (-)	R		CobQ/CobB/MinD/ParA ATPase			
307594291	type II secretion system protein E	No	0	141442	143004	-		Vulcanisaeta_distributa_DSM_14429_uid52827	NC_014537	chromosome		arCOG01817	8 (-)	N	VirB11	Type IV secretory pathway, VirB11 component, o	3E-27	COG0630	Type IV secretory pathway, VirB11 compon
307594292	hypothetical protein Vdis_0145	No	7	143010	144083	-		Vulcanisaeta_distributa_DSM_14429_uid52827	NC_014537	chromosome		arCOG10359	3	S		Uncharacterized conserved protein			
307594293	hypothetical protein Vdis_0146	No	1	144164	144604	-		Vulcanisaeta_distributa_DSM_14429_uid52827	NC_014537	chromosome		arCOG10360	3	S		Uncharacterized conserved protein			
307594294	hypothetical protein Vdis_0147	No	2	144638	145411	-		Vulcanisaeta_distributa_DSM_14429_uid52827	NC_014537	chromosome		arCOG10434	1	S		Uncharacterized conserved protein			
307594295	hypothetical protein Vdis_0148	No	1	145447	145743	-		Vulcanisaeta_distributa_DSM_14429_uid52827	NC_014537	chromosome	clust_48								
307594296	hypothetical protein Vdis_0149	No	2	145791	146201	-		Vulcanisaeta_distributa_DSM_14429_uid52827	NC_014537	chromosome		arCOG10500	4	S		Uncharacterized conserved protein			
307594297	hypothetical protein Vdis_0150	No	5	146223	147092	-		Vulcanisaeta_distributa_DSM_14429_uid52827	NC_014537	chromosome		arCOG10497	2	S		Uncharacterized conserved protein			
307594298	hypothetical protein Vdis_0151	No	0	147125	148705	-		Vulcanisaeta_distributa_DSM_14429_uid52827	NC_014537	chromosome									
307594299	Rad51 domain-containing protein	No	0	148754	149737	+		Vulcanisaeta_distributa_DSM_14429_uid52827	NC_014537	chromosome		arCOG00415	7 (-)	L	RecA	RecA/RadA recombinase	6E-25	pfam08423	Rad51. Rad51 is a DNA repair and recombi
307594300	hypothetical protein Vdis_0153	No	0	149862	150635	+		Vulcanisaeta_distributa_DSM_14429_uid52827	NC_014537	chromosome									
307594301	hypothetical protein Vdis_0154	No	0	150632	150757	-		Vulcanisaeta_distributa_DSM_14429_uid52827	NC_014537	chromosome	clust_507								
307594302	hypothetical protein Vdis_0155	No	0	150754	151299	-		Vulcanisaeta_distributa_DSM_14429_uid52827	NC_014537	chromosome	clust_194								
307594303	hypothetical protein Vdis_0156	No	0	151627	151944	+		Vulcanisaeta_distributa_DSM_14429_uid52827	NC_014537	chromosome	clust_329								
307594304	zinc finger SWIM domain-containing protein	No	0	151919	152500	+		Vulcanisaeta_distributa_DSM_14429_uid52827	NC_014537	chromosome		arCOG05500	2 (-)	S		SWIM Zn-finger			
307594305	hypothetical protein Vdis_0158	No	0	152624	152725	-		Vulcanisaeta_distributa_DSM_14429_uid52827	NC_014537	chromosome									
307594306	hypothetical protein Vdis_0159	No	6	153169	153819	+		Vulcanisaeta_distributa_DSM_14429_uid52827	NC_014537	chromosome									
307594307	ATPase	No	0	153952	154971	-		Vulcanisaeta_distributa_DSM_14429_uid52827	NC_014537	chromosome		arCOG00441	3 (-)	R		MoxR-like ATPase			
307594308	hypothetical protein Vdis_0161	No	0	154981	155859	-		Vulcanisaeta_distributa_DSM_14429_uid52827	NC_014537	chromosome									
307594309	hypothetical protein Vdis_0162	No	0	155996	157186	+		Vulcanisaeta_distributa_DSM_14429_uid52827	NC_014537	chromosome									
307594310	hypothetical protein Vdis_0163	No	0	157176	158396	+		Vulcanisaeta_distributa_DSM_14429_uid52827	NC_014537	chromosome									
307594311	competence CoiA family protein	No	0	158393	160060	+		Vulcanisaeta_distributa_DSM_14429_uid52827	NC_014537	chromosome		arCOG04025	2 (-)	L		Competence protein CoiA homolog, contains C-terminal PD-DExK nuclease domain			
307594312	hypothetical protein Vdis_0165	No	0	160176	160304	-		Vulcanisaeta_distributa_DSM_14429_uid52827	NC_014537	chromosome									
===							6	Vulcanisaeta_distributa_DSM_14429_uid52827	NC_014537	chromosome									
307594619	haloacid dehalogenase, type II	No	0	466830	467459	-		Vulcanisaeta_distributa_DSM_14429_uid52827	NC_014537	chromosome		arCOG02291	4 (-)	R		HAD superfamily hydrolase	3E-16	pfam13419	Haloacid dehalogenase-like hydrolase.
307594620	hypothetical protein Vdis_0487	No	0	467685	467834	+		Vulcanisaeta_distributa_DSM_14429_uid52827	NC_014537	chromosome									
307594621	hypothetical protein Vdis_0488	No	0	467905	468060	+		Vulcanisaeta_distributa_DSM_14429_uid52827	NC_014537	chromosome									
307594622	hypothetical protein Vdis_0489	No	0	468474	469121	+		Vulcanisaeta_distributa_DSM_14429_uid52827	NC_014537	chromosome		arCOG10867	4	S		Uncharacterized conserved protein			
307594623	hypothetical protein Vdis_0490	No	0	469202	470026	+		Vulcanisaeta_distributa_DSM_14429_uid52827	NC_014537	chromosome		arCOG10867	4	S		Uncharacterized conserved protein			
307594624	quinolinate synthetase complex, A subunit	No	0	470379	471305	+		Vulcanisaeta_distributa_DSM_14429_uid52827	NC_014537	chromosome		arCOG04459	3 (-)	H	NadA	Quinolinate synthase	7E-93	pfam02445	Quinolinate synthetase A protein. Quinol
===							5	Vulcanisaeta_distributa_DSM_14429_uid52827	NC_014537	chromosome									
307594646	hypothetical protein Vdis_0513	No	1	493000	493980	-		Vulcanisaeta_distributa_DSM_14429_uid52827	NC_014537	chromosome									
307594647	hypothetical protein Vdis_0514	No	3	494407	494751	+		Vulcanisaeta_distributa_DSM_14429_uid52827	NC_014537	chromosome									
307594648	hypothetical protein Vdis_0515	No	0	494793	496067	+		Vulcanisaeta_distributa_DSM_14429_uid52827	NC_014537	chromosome	arCOG03239,a	arCOG03239	1 (-)	R		ATPase, predicted component of phage defense system			
307594649	hypothetical protein Vdis_0516	No	0	496024	496686	+		Vulcanisaeta_distributa_DSM_14429_uid52827	NC_014537	chromosome		arCOG11093	2	S		Uncharacterized conserved protein			
307594650	hypothetical protein Vdis_0517	No	0	496679	497077	+		Vulcanisaeta_distributa_DSM_14429_uid52827	NC_014537	chromosome		arCOG10288	2	S		Uncharacterized conserved protein			
===							10	Vulcanisaeta_distributa_DSM_14429_uid52827	NC_014537	chromosome									
307594693	hypothetical protein Vdis_0561	No	0	539409	539798	+		Vulcanisaeta_distributa_DSM_14429_uid52827	NC_014537	chromosome									
307594694	hypothetical protein Vdis_0562	No	1	539795	540154	-		Vulcanisaeta_distributa_DSM_14429_uid52827	NC_014537	chromosome									
307594695	peptidase A5, thermopsin	No	1	540197	541420	-		Vulcanisaeta_distributa_DSM_14429_uid52827	NC_014537	chromosome		arCOG03670	1 (-)	E		Thermopsin-like protease	3E-23	pfam05317	Thermopsin. This family consists of seve
307594696	hypothetical protein Vdis_0565	No	1	542515	543567	+		Vulcanisaeta_distributa_DSM_14429_uid52827	NC_014537	chromosome									
307594697	hypothetical protein Vdis_0566	No	3	543607	545685	-		Vulcanisaeta_distributa_DSM_14429_uid52827	NC_014537	chromosome		arCOG07029	2	S		Uncharacterized conserved protein			
307594698	hypothetical protein Vdis_0567	No	1	545811	546506	+		Vulcanisaeta_distributa_DSM_14429_uid52827	NC_014537	chromosome		arCOG10353	2	S		Uncharacterized conserved protein			
307594699	hypothetical protein Vdis_0568	No	0	546509	547123	-		Vulcanisaeta_distributa_DSM_14429_uid52827	NC_014537	chromosome		arCOG10265	3	S		Uncharacterized conserved protein			
307594700	hypothetical protein Vdis_0569	No	0	548231	549916	+		Vulcanisaeta_distributa_DSM_14429_uid52827	NC_014537	chromosome		arCOG09797	1 (-)	S		Uncharacterized conserved protein			
307594701	hypothetical protein Vdis_0570	No	2	550084	550353	+		Vulcanisaeta_distributa_DSM_14429_uid52827	NC_014537	chromosome									
307594702	hypothetical protein Vdis_0571	No	0	550307	550588	-		Vulcanisaeta_distributa_DSM_14429_uid52827	NC_014537	chromosome									
===							5	Vulcanisaeta_distributa_DSM_14429_uid52827	NC_014537	chromosome									
307594787	hypothetical protein Vdis_0658	Yes	5	641074	642195	-		Vulcanisaeta_distributa_DSM_14429_uid52827	NC_014537	chromosome									
307594788	hypothetical protein Vdis_0659	No	1	642245	642670	+		Vulcanisaeta_distributa_DSM_14429_uid52827	NC_014537	chromosome		arCOG10986	1	S		Uncharacterized conserved protein			
307594789	transcriptional coactivator/pterin dehydratase	No	0	642764	643060	-		Vulcanisaeta_distributa_DSM_14429_uid52827	NC_014537	chromosome		arCOG02939	1 (-)	H		Pterin-4a-carbinolamine dehydratase	6E-20	pfam01329	Pterin 4 alpha carbinolamine dehydratase
307594790	hypothetical protein Vdis_0661	No	0	643100	643609	-		Vulcanisaeta_distributa_DSM_14429_uid52827	NC_014537	chromosome									
307594791	hypothetical protein Vdis_0662	No	0	643581	643718	-		Vulcanisaeta_distributa_DSM_14429_uid52827	NC_014537	chromosome									
===							6	Vulcanisaeta_distributa_DSM_14429_uid52827	NC_014537	chromosome									
307594987	ATPase	No	0	832015	833019	-		Vulcanisaeta_distributa_DSM_14429_uid52827	NC_014537	chromosome		arCOG03408	3 (-)	R		Predicted ATPase (AAA+ superfamily)	3E-45	pfam01637	Archaeal ATPase. This family contain a c
307594988	hypothetical protein Vdis_0863	No	1	833222	834328	-		Vulcanisaeta_distributa_DSM_14429_uid52827	NC_014537	chromosome		arCOG09768	3	S		Uncharacterized conserved protein			
307594989	hypothetical protein Vdis_0864	No	0	834325	834891	-		Vulcanisaeta_distributa_DSM_14429_uid52827	NC_014537	chromosome									
307594990	hypothetical protein Vdis_0865	No	0	835063	835446	-		Vulcanisaeta_distributa_DSM_14429_uid52827	NC_014537	chromosome									
307594991	hypothetical protein Vdis_0866	No	1	835604	835786	+		Vulcanisaeta_distributa_DSM_14429_uid52827	NC_014537	chromosome									
307594992	PaREP1 family protein	No	0	835763	836314	-		Vulcanisaeta_distributa_DSM_14429_uid52827	NC_014537	chromosome		arCOG03712	4 (-)	V		HEPN domain containing protein	1E-14	pfam05942	Archaeal PaREP1/PaREP8 family. This fami
===							11	Vulcanisaeta_distributa_DSM_14429_uid52827	NC_014537	chromosome									
307595079	hypothetical protein Vdis_0955	Yes	1	913811	915151	-		Vulcanisaeta_distributa_DSM_14429_uid52827	NC_014537	chromosome		arCOG10495	2	S		Uncharacterized conserved protein			
307595080	type II secretion system protein E	No	0	915259	916794	+		Vulcanisaeta_distributa_DSM_14429_uid52827	NC_014537	chromosome		arCOG01817	8 (-)	N	VirB11	Type IV secretory pathway, VirB11 component, o	6E-88	COG0630	Type IV secretory pathway, VirB11 compon
307595081	hypothetical protein Vdis_0957	No	0	916769	917137	+		Vulcanisaeta_distributa_DSM_14429_uid52827	NC_014537	chromosome									
307595082	hypothetical protein Vdis_0958	No	3	917134	918003	+		Vulcanisaeta_distributa_DSM_14429_uid52827	NC_014537	chromosome	clust_112								
307595083	hypothetical protein Vdis_0959	No	0	918000	918407	-		Vulcanisaeta_distributa_DSM_14429_uid52827	NC_014537	chromosome	clust_258								
307595084	hypothetical protein Vdis_0960	No	0	918404	919051	-		Vulcanisaeta_distributa_DSM_14429_uid52827	NC_014537	chromosome	clust_158								
307595085	hypothetical protein Vdis_0961	No	0	919026	919415	-		Vulcanisaeta_distributa_DSM_14429_uid52827	NC_014537	chromosome	clust_274								
307595086	hypothetical protein Vdis_0962	No	0	919412	919807	-		Vulcanisaeta_distributa_DSM_14429_uid52827	NC_014537	chromosome	clust_270								
307595087	hypothetical protein Vdis_0963	No	0	919788	919883	-		Vulcanisaeta_distributa_DSM_14429_uid52827	NC_014537	chromosome									
307595088	H+ transporting ATPase	No	0	919883	920113	-		Vulcanisaeta_distributa_DSM_14429_uid52827	NC_014537	chromosome	clust_419								
307595089	hypothetical protein Vdis_0965	No	0	920159	922558	-		Vulcanisaeta_distributa_DSM_14429_uid52827	NC_014537	chromosome		arCOG01951	2 (-)	R		Predicted P-loop ATPase fused to an acetyltransferase		COG1444	Predicted P-loop ATPase fused to an acet
===							6	Vulcanisaeta_distributa_DSM_14429_uid52827	NC_014537	chromosome									
307595176	aldo/keto reductase	No	0	1008521	1009474	+		Vulcanisaeta_distributa_DSM_14429_uid52827	NC_014537	chromosome		arCOG01617	4 (-)	C	Tas	Predicted oxidoreductase (related to aryl-alcohol	5E-32	COG0667	Predicted oxidoreductases (related to ar
307595177	hypothetical protein Vdis_1054	Yes	1	1009883	1011520	+		Vulcanisaeta_distributa_DSM_14429_uid52827	NC_014537	chromosome		arCOG10875	3	S		Uncharacterized conserved protein			
307595178	hypothetical protein Vdis_1055	No	0	1011710	1011847	-		Vulcanisaeta_distributa_DSM_14429_uid52827	NC_014537	chromosome									
307595179	hypothetical protein Vdis_1056	No	0	1011844	1012137	-		Vulcanisaeta_distributa_DSM_14429_uid52827	NC_014537	chromosome									
307595180	hypothetical protein Vdis_1058	No	0	1012543	1013115	+		Vulcanisaeta_distributa_DSM_14429_uid52827	NC_014537	chromosome		arCOG10492	1	S		Uncharacterized conserved protein			
307595181	pyruvate ferredoxin/flavodoxin oxidoreductase	No	0	1013117	1014079	-		Vulcanisaeta_distributa_DSM_14429_uid52827	NC_014537	chromosome		arCOG01599	1 (-)	C	PorB	Pyruvate:ferredoxin oxidoreductase or related 2-	2E-62	COG1013	Pyruvate:ferredoxin oxidoreductase and r
===							5	Vulcanisaeta_distributa_DSM_14429_uid52827	NC_014537	chromosome									
307595201	hypothetical protein Vdis_1079	No	0	1027941	1028831	-		Vulcanisaeta_distributa_DSM_14429_uid52827	NC_014537	chromosome		arCOG07182	3	S		Uncharacterized conserved protein			
307595202	hypothetical protein Vdis_1080	Yes	1	1028831	1029379	-		Vulcanisaeta_distributa_DSM_14429_uid52827	NC_014537	chromosome		arCOG07182	3	S		Uncharacterized conserved protein			
307595203	hypothetical protein Vdis_1081	No	4	1029366	1030136	-		Vulcanisaeta_distributa_DSM_14429_uid52827	NC_014537	chromosome		arCOG10175	1	S		Uncharacterized conserved protein			
307595204	hypothetical protein Vdis_1082	No	0	1030133	1030819	-		Vulcanisaeta_distributa_DSM_14429_uid52827	NC_014537	chromosome		arCOG10174	1 (-)	S		Uncharacterized conserved protein			
307595205	hypothetical protein Vdis_1083	No	0	1030941	1031465	-		Vulcanisaeta_distributa_DSM_14429_uid52827	NC_014537	chromosome									
===							7	Vulcanisaeta_distributa_DSM_14429_uid52827	NC_014537	chromosome									
307595418	hypothetical protein Vdis_1298	No	1	1233870	1235375	+		Vulcanisaeta_distributa_DSM_14429_uid52827	NC_014537	chromosome		arCOG05475	4 (-)	S		Uncharacterized conserved protein			
307595419	hypothetical protein Vdis_1299	No	1	1235375	1237561	+		Vulcanisaeta_distributa_DSM_14429_uid52827	NC_014537	chromosome		arCOG10501	3	S		Uncharacterized conserved protein			
307595420	hypothetical protein Vdis_1300	No	1	1237540	1239333	+		Vulcanisaeta_distributa_DSM_14429_uid52827	NC_014537	chromosome		arCOG10502	4	S		Uncharacterized conserved protein			
307595421	hypothetical protein Vdis_1301	No	2	1239333	1240082	+		Vulcanisaeta_distributa_DSM_14429_uid52827	NC_014537	chromosome		arCOG10503	5	S		Uncharacterized conserved protein			
307595422	hypothetical protein Vdis_1302	No	3	1240087	1240929	+		Vulcanisaeta_distributa_DSM_14429_uid52827	NC_014537	chromosome		arCOG10503	5	S		Uncharacterized conserved protein			
307595423	hypothetical protein Vdis_1303	No	1	1240929	1241501	+		Vulcanisaeta_distributa_DSM_14429_uid52827	NC_014537	chromosome		arCOG10504	3	S		Uncharacterized conserved protein			
307595424	DNA polymerase subunit beta	No	0	1241513	1241929	+		Vulcanisaeta_distributa_DSM_14429_uid52827	NC_014537	chromosome		arCOG02110	2 (-)	V		Minimal nucleotidyltransferase			
===							5	Vulcanisaeta_distributa_DSM_14429_uid52827	NC_014537	chromosome									
307595987	hypothetical protein Vdis_1881	No	0	1770482	1770694	+		Vulcanisaeta_distributa_DSM_14429_uid52827	NC_014537	chromosome									
307595988	glutaredoxin	No	0	1770772	1770936	-		Vulcanisaeta_distributa_DSM_14429_uid52827	NC_014537	chromosome		arCOG04235	1 (-)	O		Predicted thioredoxin/glutaredoxin			
307595989	hypothetical protein Vdis_1883	No	0	1770921	1771232	-		Vulcanisaeta_distributa_DSM_14429_uid52827	NC_014537	chromosome									
307595990	hypothetical protein Vdis_1884	No	4	1771295	1771864	+		Vulcanisaeta_distributa_DSM_14429_uid52827	NC_014537	chromosome									
307595991	hypothetical protein Vdis_1885	No	0	1771815	1771985	-		Vulcanisaeta_distributa_DSM_14429_uid52827	NC_014537	chromosome									
===							5	Vulcanisaeta_distributa_DSM_14429_uid52827	NC_014537	chromosome									
307596171	hypothetical protein Vdis_2066	No	0	1943605	1943859	+		Vulcanisaeta_distributa_DSM_14429_uid52827	NC_014537	chromosome									
307596172	hypothetical protein Vdis_2067	No	0	1944164	1944640	+		Vulcanisaeta_distributa_DSM_14429_uid52827	NC_014537	chromosome									
307596173	hypothetical protein Vdis_2068	No	5	1944692	1945756	+		Vulcanisaeta_distributa_DSM_14429_uid52827	NC_014537	chromosome									
307596174	hypothetical protein Vdis_2070	No	0	1948015	1948383	-		Vulcanisaeta_distributa_DSM_14429_uid52827	NC_014537	chromosome		arCOG03723	2 (-)	V		HEPN domain containing protein			
307596175	hypothetical protein Vdis_2071	No	3	1948615	1949019	-		Vulcanisaeta_distributa_DSM_14429_uid52827	NC_014537	chromosome									
===							9	Vulcanisaeta_distributa_DSM_14429_uid52827	NC_014537	chromosome									
307596359	type II secretion system protein E	No	0	2107599	2109170	-		Vulcanisaeta_distributa_DSM_14429_uid52827	NC_014537	chromosome		arCOG01817	8 (-)	N	VirB11	Type IV secretory pathway, VirB11 component, o	6E-23	COG0630	Type IV secretory pathway, VirB11 compon
307596360	hypothetical protein Vdis_2258	No	8	2109170	2110243	-		Vulcanisaeta_distributa_DSM_14429_uid52827	NC_014537	chromosome		arCOG10359	3	S		Uncharacterized conserved protein			
307596361	hypothetical protein Vdis_2259	No	1	2110327	2110755	-		Vulcanisaeta_distributa_DSM_14429_uid52827	NC_014537	chromosome		arCOG10360	3	S		Uncharacterized conserved protein			
307596362	hypothetical protein Vdis_2260	No	5	2110913	2111896	-		Vulcanisaeta_distributa_DSM_14429_uid52827	NC_014537	chromosome									
307596363	hypothetical protein Vdis_2261	No	1	2111957	2112748	-		Vulcanisaeta_distributa_DSM_14429_uid52827	NC_014537	chromosome									
307596364	hypothetical protein Vdis_2262	No	1	2112781	2113065	-		Vulcanisaeta_distributa_DSM_14429_uid52827	NC_014537	chromosome	clust_48								
307596365	hypothetical protein Vdis_2263	No	0	2113181	2113882	+		Vulcanisaeta_distributa_DSM_14429_uid52827	NC_014537	chromosome									
307596366	hypothetical protein Vdis_2264	No	0	2114040	2115464	+		Vulcanisaeta_distributa_DSM_14429_uid52827	NC_014537	chromosome									
307596367	PP-loop domain-containing protein	No	0	2115943	2117124	+		Vulcanisaeta_distributa_DSM_14429_uid52827	NC_014537	chromosome		arCOG00042	4 (-)	J	MesJ	tRNA(Ile)-lysidine synthase MesJ	4E-39	COG0037	tRNA(Ile)-lysidine synthase MesJ
===							6	Vulcanisaeta_distributa_DSM_14429_uid52827	NC_014537	chromosome									
307596484	hypothetical protein Vdis_2383	No	1	2230067	2231110	-		Vulcanisaeta_distributa_DSM_14429_uid52827	NC_014537	chromosome		arCOG04164	1 (-)	S		Uncharacterized conserved protein	3E-73	pfam06626	Protein of unknown function (DUF1152). T
307596485	hypothetical protein Vdis_2384	No	1	2231154	2231960	-		Vulcanisaeta_distributa_DSM_14429_uid52827	NC_014537	chromosome	clust_122								
307596486	hypothetical protein Vdis_2385	No	1	2231967	2232275	-		Vulcanisaeta_distributa_DSM_14429_uid52827	NC_014537	chromosome	clust_345								
307596487	hypothetical protein Vdis_2386	No	1	2232244	2232405	-		Vulcanisaeta_distributa_DSM_14429_uid52827	NC_014537	chromosome	clust_493								
307596488	hypothetical protein Vdis_2387	No	0	2232493	2232615	+		Vulcanisaeta_distributa_DSM_14429_uid52827	NC_014537	chromosome									
307596489	helicase domain-containing protein	No	0	2232632	2235739	-		Vulcanisaeta_distributa_DSM_14429_uid52827	NC_014537	chromosome		arCOG00876	2 (-)	K	SSL2	DNA or RNA helicase of superfamily II	4E-29	COG1061	DNA or RNA helicases of superfamily II
===							6	Vulcanisaeta_moutnovskia_768_28_uid63631	NC_015151	chromosome									
325967679	hypothetical protein VMUT_0153	No	0	154743	155033	+		Vulcanisaeta_moutnovskia_768_28_uid63631	NC_015151	chromosome		arCOG10177	2 (-)	S		Uncharacterized conserved protein			
325967680	hypothetical protein VMUT_0154	No	0	155742	156002	+		Vulcanisaeta_moutnovskia_768_28_uid63631	NC_015151	chromosome									
325967681	hypothetical protein VMUT_0155	No	1	156617	158191	-		Vulcanisaeta_moutnovskia_768_28_uid63631	NC_015151	chromosome		arCOG10875	3	S		Uncharacterized conserved protein			
325967682	hypothetical protein VMUT_0156	No	4	158197	159054	-		Vulcanisaeta_moutnovskia_768_28_uid63631	NC_015151	chromosome									
325967683	hypothetical protein VMUT_0157	No	0	159129	159305	-		Vulcanisaeta_moutnovskia_768_28_uid63631	NC_015151	chromosome									
325967684	hypothetical protein VMUT_0158	No	0	159808	160104	-		Vulcanisaeta_moutnovskia_768_28_uid63631	NC_015151	chromosome		arCOG03698	1 (-)	K		Predicted transcriptional regulator, contains HTH d	2E-31	pfam10007	Uncharacterized protein conserved in arc
===							5	Vulcanisaeta_moutnovskia_768_28_uid63631	NC_015151	chromosome									
325968253	hypothetical protein VMUT_0732	No	1	703023	703823	-		Vulcanisaeta_moutnovskia_768_28_uid63631	NC_015151	chromosome	clust_122								
325968254	hypothetical protein VMUT_0733	No	1	703829	704104	-		Vulcanisaeta_moutnovskia_768_28_uid63631	NC_015151	chromosome	clust_345								
325968255	hypothetical protein VMUT_0734	No	1	704106	704267	-		Vulcanisaeta_moutnovskia_768_28_uid63631	NC_015151	chromosome	clust_493								
325968256	helicase	No	0	704484	707597	-		Vulcanisaeta_moutnovskia_768_28_uid63631	NC_015151	chromosome		arCOG00876	2 (-)	K	SSL2	DNA or RNA helicase of superfamily II	2E-28	COG1061	DNA or RNA helicases of superfamily II
325968257	hypothetical protein VMUT_0736	Yes	3	707993	709594	+		Vulcanisaeta_moutnovskia_768_28_uid63631	NC_015151	chromosome	arCOG07344,a	arCOG07344	1	S		Uncharacterized conserved protein			
===							9	Vulcanisaeta_moutnovskia_768_28_uid63631	NC_015151	chromosome									
325968505	hypothetical protein VMUT_0987	No	0	955650	955859	-		Vulcanisaeta_moutnovskia_768_28_uid63631	NC_015151	chromosome									
325968506	hypothetical protein VMUT_0988	No	8	955997	958537	-		Vulcanisaeta_moutnovskia_768_28_uid63631	NC_015151	chromosome	clust_66								
325968507	hypothetical protein VMUT_0989	No	1	958534	958779	-		Vulcanisaeta_moutnovskia_768_28_uid63631	NC_015151	chromosome									
325968508	hypothetical protein VMUT_0990	No	0	958921	960486	-		Vulcanisaeta_moutnovskia_768_28_uid63631	NC_015151	chromosome		arCOG02173	4 (-)	O	NrdG	Organic radical activating enzyme			
325968509	hypothetical protein VMUT_0991	No	0	961314	961952	-		Vulcanisaeta_moutnovskia_768_28_uid63631	NC_015151	chromosome	clust_159								
325968510	hypothetical protein VMUT_0992	No	0	962329	963708	-		Vulcanisaeta_moutnovskia_768_28_uid63631	NC_015151	chromosome		arCOG00770	3 (-)	K	DinG	Rad3-related DNA helicase			
325968511	hypothetical protein VMUT_0993	No	2	963709	964233	-		Vulcanisaeta_moutnovskia_768_28_uid63631	NC_015151	chromosome	clust_200								
325968512	hypothetical protein VMUT_0994	No	0	964223	964486	-		Vulcanisaeta_moutnovskia_768_28_uid63631	NC_015151	chromosome									
325968513	hypothetical protein VMUT_0995	No	0	964655	965155	-		Vulcanisaeta_moutnovskia_768_28_uid63631	NC_015151	chromosome	clust_209								
===							5	Vulcanisaeta_moutnovskia_768_28_uid63631	NC_015151	chromosome									
325968517	hypothetical protein VMUT_1000	No	3	976015	977103	-		Vulcanisaeta_moutnovskia_768_28_uid63631	NC_015151	chromosome									
325968518	hypothetical protein VMUT_1001	No	2	977336	979429	-		Vulcanisaeta_moutnovskia_768_28_uid63631	NC_015151	chromosome		arCOG00285	1 (-)	L		HerA helicase	2E-13	pfam12846	AAA-like domain. This family of domains
325968519	hypothetical protein VMUT_1002	No	0	979448	979948	-		Vulcanisaeta_moutnovskia_768_28_uid63631	NC_015151	chromosome		arCOG10944	2	S		Uncharacterized conserved protein			
325968520	hypothetical protein VMUT_1003	No	0	979945	980310	-		Vulcanisaeta_moutnovskia_768_28_uid63631	NC_015151	chromosome	clust_293								
325968521	hypothetical protein VMUT_1004	Yes	1	980432	982030	-		Vulcanisaeta_moutnovskia_768_28_uid63631	NC_015151	chromosome		arCOG10875	3	S		Uncharacterized conserved protein			
===							19	Vulcanisaeta_moutnovskia_768_28_uid63631	NC_015151	chromosome									
325968523	type II secretion system protein E	No	0	982953	984524	-		Vulcanisaeta_moutnovskia_768_28_uid63631	NC_015151	chromosome		arCOG01817	8 (-)	N	VirB11	Type IV secretory pathway, VirB11 component, o	5E-27	COG0630	Type IV secretory pathway, VirB11 compon
325968524	hypothetical protein VMUT_1007	No	7	984530	985597	-		Vulcanisaeta_moutnovskia_768_28_uid63631	NC_015151	chromosome		arCOG10359	3	S		Uncharacterized conserved protein			
325968525	hypothetical protein VMUT_1008	No	1	985777	986211	-		Vulcanisaeta_moutnovskia_768_28_uid63631	NC_015151	chromosome		arCOG10360	3	S		Uncharacterized conserved protein			
325968526	hypothetical protein VMUT_1009	No	2	986251	987039	-		Vulcanisaeta_moutnovskia_768_28_uid63631	NC_015151	chromosome									
325968527	hypothetical protein VMUT_1010	No	1	987071	987367	-		Vulcanisaeta_moutnovskia_768_28_uid63631	NC_015151	chromosome	clust_48								
325968528	hypothetical protein VMUT_1011	No	2	987406	987816	-		Vulcanisaeta_moutnovskia_768_28_uid63631	NC_015151	chromosome		arCOG10500	4	S		Uncharacterized conserved protein			
325968529	hypothetical protein VMUT_1012	No	5	987838	988719	-		Vulcanisaeta_moutnovskia_768_28_uid63631	NC_015151	chromosome		arCOG10497	2	S		Uncharacterized conserved protein			
325968530	hypothetical protein VMUT_1013	No	0	988728	990236	-		Vulcanisaeta_moutnovskia_768_28_uid63631	NC_015151	chromosome									
325968531	hypothetical protein VMUT_1014	No	0	990470	991243	-		Vulcanisaeta_moutnovskia_768_28_uid63631	NC_015151	chromosome									
325968532	Rad51 domain-containing protein	No	0	991323	992309	-		Vulcanisaeta_moutnovskia_768_28_uid63631	NC_015151	chromosome		arCOG00415	7 (-)	L	RecA	RecA/RadA recombinase	2E-23	pfam08423	Rad51. Rad51 is a DNA repair and recombi
325968533	hypothetical protein VMUT_1016	No	0	992333	992455	-		Vulcanisaeta_moutnovskia_768_28_uid63631	NC_015151	chromosome	clust_507								
325968534	hypothetical protein VMUT_1017	No	0	992456	992998	-		Vulcanisaeta_moutnovskia_768_28_uid63631	NC_015151	chromosome	clust_194								
325968535	hypothetical protein VMUT_1018	No	0	993340	993660	+		Vulcanisaeta_moutnovskia_768_28_uid63631	NC_015151	chromosome	clust_329								
325968536	zinc finger, SWIM domain protein	No	0	993713	994201	+		Vulcanisaeta_moutnovskia_768_28_uid63631	NC_015151	chromosome		arCOG05500	2 (-)	S		SWIM Zn-finger			
325968537	hypothetical protein VMUT_1020	No	0	994218	994958	-		Vulcanisaeta_moutnovskia_768_28_uid63631	NC_015151	chromosome									
325968538	hypothetical protein VMUT_1021	No	0	994955	996790	-		Vulcanisaeta_moutnovskia_768_28_uid63631	NC_015151	chromosome									
325968539	hypothetical protein VMUT_1022	No	0	997056	997373	-		Vulcanisaeta_moutnovskia_768_28_uid63631	NC_015151	chromosome									
325968540	hypothetical protein VMUT_1023	No	6	997730	998485	-		Vulcanisaeta_moutnovskia_768_28_uid63631	NC_015151	chromosome									
325968541	hypothetical protein VMUT_1024	No	1	998731	1000368	-		Vulcanisaeta_moutnovskia_768_28_uid63631	NC_015151	chromosome		arCOG07181	1 (-)	R		Predicted solute binding protein			
===							7	Vulcanisaeta_moutnovskia_768_28_uid63631	NC_015151	chromosome									
325968703	hypothetical protein VMUT_1188	No	0	1165444	1165767	-		Vulcanisaeta_moutnovskia_768_28_uid63631	NC_015151	chromosome		arCOG02105	3 (-)	V		Minimal nucleotidyltransferase			
325968704	hypothetical protein VMUT_1189	No	0	1165943	1166083	-		Vulcanisaeta_moutnovskia_768_28_uid63631	NC_015151	chromosome		arCOG04293	1	J		Ribosomal protein S30	0.00000002	COG4919	Ribosomal protein S30
325968705	hypothetical protein VMUT_1190	No	0	1166216	1167721	+		Vulcanisaeta_moutnovskia_768_28_uid63631	NC_015151	chromosome									
325968706	hypothetical protein VMUT_1191	No	2	1167736	1168194	-		Vulcanisaeta_moutnovskia_768_28_uid63631	NC_015151	chromosome									
325968707	hypothetical protein VMUT_1192	No	0	1168810	1169244	+		Vulcanisaeta_moutnovskia_768_28_uid63631	NC_015151	chromosome		arCOG11093	2	S		Uncharacterized conserved protein			
325968708	hypothetical protein VMUT_1193	No	0	1169290	1169688	+		Vulcanisaeta_moutnovskia_768_28_uid63631	NC_015151	chromosome		arCOG10288	2	S		Uncharacterized conserved protein			
325968709	hypothetical protein VMUT_1194	No	0	1169951	1170454	+		Vulcanisaeta_moutnovskia_768_28_uid63631	NC_015151	chromosome		arCOG00552	1 (-)	L		DNA replication initiation complex subunit, GINS23 family			
===							6	Vulcanisaeta_moutnovskia_768_28_uid63631	NC_015151	chromosome									
325969254	Diadenosine tetraphosphate (Ap4A) hydrolase	No	0	1724088	1724459	+		Vulcanisaeta_moutnovskia_768_28_uid63631	NC_015151	chromosome		arCOG00419	4 (-)	F	Hit	HIT family hydrolase	1E-10	COG0537	Diadenosine tetraphosphate (Ap4A) hydrol
325969255	hypothetical protein VMUT_1744	No	1	1724449	1724823	+		Vulcanisaeta_moutnovskia_768_28_uid63631	NC_015151	chromosome									
325969256	hypothetical protein VMUT_1745	No	0	1724835	1725395	-		Vulcanisaeta_moutnovskia_768_28_uid63631	NC_015151	chromosome		arCOG10867	4	S		Uncharacterized conserved protein			
325969257	hypothetical protein VMUT_1746	No	0	1725404	1725622	-		Vulcanisaeta_moutnovskia_768_28_uid63631	NC_015151	chromosome									
325969258	hypothetical protein VMUT_1747	No	4	1725632	1725982	-		Vulcanisaeta_moutnovskia_768_28_uid63631	NC_015151	chromosome									
325969259	50S ribosomal protein L13E	No	0	1726084	1726560	+		Vulcanisaeta_moutnovskia_768_28_uid63631	NC_015151	chromosome	arCOG01013,a	arCOG01013	1 (-)	J		Ribosomal protein L13E	0.000000004	COG4352	Ribosomal protein L13E
===							10	Vulcanisaeta_moutnovskia_768_28_uid63631	NC_015151	chromosome									
325969314	hypothetical protein VMUT_1803	No	1	1776721	1778067	-		Vulcanisaeta_moutnovskia_768_28_uid63631	NC_015151	chromosome		arCOG10495	2	S		Uncharacterized conserved protein			
325969315	type II secretion system protein E	No	0	1778168	1779685	+		Vulcanisaeta_moutnovskia_768_28_uid63631	NC_015151	chromosome		arCOG01817	8 (-)	N	VirB11	Type IV secretory pathway, VirB11 component, o	5E-88	COG0630	Type IV secretory pathway, VirB11 compon
325969316	hypothetical protein VMUT_1805	No	0	1779742	1779990	+		Vulcanisaeta_moutnovskia_768_28_uid63631	NC_015151	chromosome									
325969317	hypothetical protein VMUT_1806	No	3	1780007	1780876	+		Vulcanisaeta_moutnovskia_768_28_uid63631	NC_015151	chromosome	clust_112								
325969318	hypothetical protein VMUT_1807	No	0	1780873	1781277	-		Vulcanisaeta_moutnovskia_768_28_uid63631	NC_015151	chromosome	clust_258								
325969319	hypothetical protein VMUT_1808	No	0	1781274	1781915	-		Vulcanisaeta_moutnovskia_768_28_uid63631	NC_015151	chromosome	clust_158								
325969320	hypothetical protein VMUT_1809	No	0	1781896	1782285	-		Vulcanisaeta_moutnovskia_768_28_uid63631	NC_015151	chromosome	clust_274								
325969321	hypothetical protein VMUT_1810	No	0	1782282	1782677	-		Vulcanisaeta_moutnovskia_768_28_uid63631	NC_015151	chromosome	clust_270								
325969322	H+ transporting ATPase I	No	0	1782755	1782982	-		Vulcanisaeta_moutnovskia_768_28_uid63631	NC_015151	chromosome	clust_419								
325969323	P-loop ATPase	No	0	1783027	1785426	-		Vulcanisaeta_moutnovskia_768_28_uid63631	NC_015151	chromosome		arCOG01951	2 (-)	R		Predicted P-loop ATPase fused to an acetyltransferase		COG1444	Predicted P-loop ATPase fused to an acet
===							6	Vulcanisaeta_moutnovskia_768_28_uid63631	NC_015151	chromosome									
325969526	5-formyltetrahydrofolate cyclo-ligase	No	0	1976878	1977603	-		Vulcanisaeta_moutnovskia_768_28_uid63631	NC_015151	chromosome		arCOG00474	1 (-)	H		5-formyltetrahydrofolate cyclo-ligase	1E-11	pfam01812	5-formyltetrahydrofolate cyclo-ligase fa
325969527	hypothetical protein VMUT_2018	No	0	1977804	1978184	+		Vulcanisaeta_moutnovskia_768_28_uid63631	NC_015151	chromosome									
325969528	hypothetical protein VMUT_2020	No	5	1978921	1979553	+		Vulcanisaeta_moutnovskia_768_28_uid63631	NC_015151	chromosome							0.000004	pfam06157	Protein of unknown function (DUF973). Th
325969529	hypothetical protein VMUT_2021	No	3	1979558	1980202	+		Vulcanisaeta_moutnovskia_768_28_uid63631	NC_015151	chromosome									
325969530	hypothetical protein VMUT_2022	No	6	1980353	1981000	+		Vulcanisaeta_moutnovskia_768_28_uid63631	NC_015151	chromosome							3E-11	pfam06157	Protein of unknown function (DUF973). Th
325969531	major facilitator superfamily MFS_1	No	11	1981076	1982371	+		Vulcanisaeta_moutnovskia_768_28_uid63631	NC_015151	chromosome		arCOG00132	2 (-)	G	ProP	Permease of the major facilitator superfamily	5E-10	pfam07690	Major Facilitator Superfamily.
===							5	Vulcanisaeta_moutnovskia_768_28_uid63631	NC_015151	chromosome									
325969601	hypothetical protein VMUT_2094	Yes	0	2054204	2055613	-		Vulcanisaeta_moutnovskia_768_28_uid63631	NC_015151	chromosome									
325969602	hypothetical protein VMUT_2095	No	0	2055687	2056385	-		Vulcanisaeta_moutnovskia_768_28_uid63631	NC_015151	chromosome									
325969603	hypothetical protein VMUT_2096	No	0	2056473	2056655	+		Vulcanisaeta_moutnovskia_768_28_uid63631	NC_015151	chromosome									
325969604	ATP-NAD/AcoX kinase	No	0	2056642	2057628	-		Vulcanisaeta_moutnovskia_768_28_uid63631	NC_015151	chromosome	arCOG00394,a	arCOG00394	7 (-)	K	ArsR	Transcriptional regulator containing HTH domain,	1E-29	COG0061	NAD kinase
325969605	hypothetical protein VMUT_2098	No	0	2057924	2058505	-		Vulcanisaeta_moutnovskia_768_28_uid63631	NC_015151	chromosome									
===							7	Vulcanisaeta_moutnovskia_768_28_uid63631	NC_015151	chromosome									
325969632	hypothetical protein VMUT_2125	No	1	2082858	2084450	+		Vulcanisaeta_moutnovskia_768_28_uid63631	NC_015151	chromosome		arCOG05475	4 (-)	S		Uncharacterized conserved protein			
325969633	hypothetical protein VMUT_2126	No	2	2084457	2086682	+		Vulcanisaeta_moutnovskia_768_28_uid63631	NC_015151	chromosome		arCOG10501	3	S		Uncharacterized conserved protein			
325969634	hypothetical protein VMUT_2127	Yes	1	2086729	2088381	+		Vulcanisaeta_moutnovskia_768_28_uid63631	NC_015151	chromosome		arCOG10502	4	S		Uncharacterized conserved protein			
325969635	hypothetical protein VMUT_2128	Yes	3	2088381	2089130	+		Vulcanisaeta_moutnovskia_768_28_uid63631	NC_015151	chromosome		arCOG10503	5	S		Uncharacterized conserved protein			
325969636	hypothetical protein VMUT_2129	Yes	3	2089179	2089970	+		Vulcanisaeta_moutnovskia_768_28_uid63631	NC_015151	chromosome		arCOG10503	5	S		Uncharacterized conserved protein			
325969637	hypothetical protein VMUT_2130	No	1	2089975	2090535	+		Vulcanisaeta_moutnovskia_768_28_uid63631	NC_015151	chromosome		arCOG10504	3	S		Uncharacterized conserved protein			
325969638	hypothetical protein VMUT_2131	No	0	2090555	2090965	+		Vulcanisaeta_moutnovskia_768_28_uid63631	NC_015151	chromosome		arCOG02110	2 (-)	V		Minimal nucleotidyltransferase			
===							7	halophilic_archaeon_DL31_uid72619	NC_015954	chromosome									
345005551	transposase IS4 family protein	No	0	1603591	1604589	-		halophilic_archaeon_DL31_uid72619	NC_015954	chromosome		arCOG04442	3 (-)	L		Transposase			
345005552	hypothetical protein Halar_2367	No	0	1605131	1605688	+		halophilic_archaeon_DL31_uid72619	NC_015954	chromosome									
345005553	hypothetical protein Halar_2368	Yes	2	1605642	1605818	+		halophilic_archaeon_DL31_uid72619	NC_015954	chromosome									
345005554	hypothetical protein Halar_2369	No	0	1605889	1606020	-		halophilic_archaeon_DL31_uid72619	NC_015954	chromosome		arCOG11160	1	S		Uncharacterized conserved protein			
345005555	hypothetical protein Halar_2370	No	2	1606129	1606395	+		halophilic_archaeon_DL31_uid72619	NC_015954	chromosome									
345005556	aldehyde dehydrogenase	No	0	1607117	1607593	+		halophilic_archaeon_DL31_uid72619	NC_015954	chromosome		arCOG01252	3 (-)	C	PutA	Lactaldehyde dehydrogenase, Succinate semial	0.000000003	pfam00171	Aldehyde dehydrogenase family. This fami
345005557	hypothetical protein Halar_2372	No	0	1607735	1607911	-		halophilic_archaeon_DL31_uid72619	NC_015954	chromosome									
===							7	halophilic_archaeon_DL31_uid72619	NC_015954	chromosome									
345005824	hypothetical protein Halar_2657	No	0	1877688	1878242	+		halophilic_archaeon_DL31_uid72619	NC_015954	chromosome									
345005825	hypothetical protein Halar_2658	No	0	1878365	1879270	-		halophilic_archaeon_DL31_uid72619	NC_015954	chromosome		arCOG04572	1 (-)	S		Uncharacterized conserved protein			
345005826	hypothetical protein Halar_2659	Yes	2	1879432	1879599	+		halophilic_archaeon_DL31_uid72619	NC_015954	chromosome									
345005827	hypothetical protein Halar_2660	No	0	1879657	1879932	+		halophilic_archaeon_DL31_uid72619	NC_015954	chromosome									
345005828	hypothetical protein Halar_2661	No	0	1879916	1880194	+		halophilic_archaeon_DL31_uid72619	NC_015954	chromosome									
345005829	beta-lactamase domain-containing protein	No	0	1880258	1881145	-		halophilic_archaeon_DL31_uid72619	NC_015954	chromosome		arCOG00515	1 (-)	R		Zn-dependent hydrolase of the beta-lactamase fo	0.00000002	COG2220	Predicted Zn-dependent hydrolases of the
345005830	hypothetical protein Halar_2663	No	0	1881199	1881309	+		halophilic_archaeon_DL31_uid72619	NC_015954	chromosome									
===							6	halophilic_archaeon_DL31_uid72619	NC_015954	chromosome									
345005868	hypothetical protein Halar_2704	No	0	1926355	1926684	-		halophilic_archaeon_DL31_uid72619	NC_015954	chromosome		arCOG04394	1 (-)	S		Uncharacterized conserved protein	0.00000002	COG1416	Uncharacterized conserved protein
345005869	hypothetical protein Halar_2705	No	0	1926769	1926915	+		halophilic_archaeon_DL31_uid72619	NC_015954	chromosome									
345005870	hypothetical protein Halar_2706	No	4	1926928	1927428	-		halophilic_archaeon_DL31_uid72619	NC_015954	chromosome									
345005871	hypothetical protein Halar_2707	No	5	1927439	1927942	-		halophilic_archaeon_DL31_uid72619	NC_015954	chromosome									

345005872	hypothetical protein Halar_2708	No	3		1928222	1928503	-		halophilic_archaeon_DL31_uid72619	NC_015954	chromosome									
345005873	ribonuclease BN	No	5		1928778	1929827	+		halophilic_archaeon_DL31_uid72619	NC_015954	chromosome		arCOG04965	1 (-)	L	Rbn	Ribonuclease BN family enzyme	1E-23	pfam03631	Virulence factor BrkB. This family acts
===								5	halophilic_archaeon_DL31_uid72619	NC_015954	chromosome									
345006025	hypothetical protein Halar_2874	No	0		2085118	2085402	-		halophilic_archaeon_DL31_uid72619	NC_015954	chromosome									
345006026	transposase IS4 family protein	No	0		2085550	2085819	-		halophilic_archaeon_DL31_uid72619	NC_015954	chromosome									
345006027	hypothetical protein Halar_2876	No	2		2085847	2086113	-		halophilic_archaeon_DL31_uid72619	NC_015954	chromosome									
345006028	Tyrosyl-tRNA synthetase	No	0		2086219	2087217	+		halophilic_archaeon_DL31_uid72619	NC_015954	chromosome		arCOG01886	1 (-)	J	TyrS	Tyrosyl-tRNA synthetase	6E-22	pfam00579	tRNA synthetases class I (W and Y).
345006029	hypothetical protein Halar_2878	No	2		2087329	2087508	-		halophilic_archaeon_DL31_uid72619	NC_015954	chromosome									
===								5	halophilic_archaeon_DL31_uid72619	NC_015954	chromosome									
345006338	hypothetical protein Halar_3204	No	0		2412668	2413054	-		halophilic_archaeon_DL31_uid72619	NC_015954	chromosome	arCOG06890	arCOG06890	1	S		Uncharacterized conserved protein	0.0000006	COG1196	Chromosome segregation ATPases
345006339	transposase IS4 family protein	No	0		2413342	2414949	-		halophilic_archaeon_DL31_uid72619	NC_015954	chromosome	arCOG03900,a	arCOG03900	5 (-)	L		Transposase			
345006340	hypothetical protein Halar_3206	No	5		2415125	2415589	+		halophilic_archaeon_DL31_uid72619	NC_015954	chromosome									
345006341	hypothetical protein Halar_3207	No	0		2415607	2416317	-		halophilic_archaeon_DL31_uid72619	NC_015954	chromosome									
345006342	hypothetical protein Halar_3208	No	0		2416314	2416982	-		halophilic_archaeon_DL31_uid72619	NC_015954	chromosome									
===								7	halophilic_archaeon_DL31_uid72619	NC_015954	chromosome									
345006702	hypothetical protein Halar_3597	No	0		2787064	2787171	-		halophilic_archaeon_DL31_uid72619	NC_015954	chromosome									
345006703	HNH endonuclease	No	1		2787283	2787771	-		halophilic_archaeon_DL31_uid72619	NC_015954	chromosome		arCOG03898	7 (-)	V	McrA	Restriction endonuclease, McrA/HNH family	0.00000002	COG1403	Restriction endonuclease
345006704	hypothetical protein Halar_3599	No	0		2788361	2788495	-		halophilic_archaeon_DL31_uid72619	NC_015954	chromosome									
345006705	hypothetical protein Halar_3600	No	0		2788590	2788790	-		halophilic_archaeon_DL31_uid72619	NC_015954	chromosome									
345006706	hypothetical protein Halar_3601	No	0		2789129	2789296	-		halophilic_archaeon_DL31_uid72619	NC_015954	chromosome									
345006707	hypothetical protein Halar_3602	No	0		2789311	2789448	-		halophilic_archaeon_DL31_uid72619	NC_015954	chromosome									
345006708	hypothetical protein Halar_3603	No	0		2789445	2789873	-		halophilic_archaeon_DL31_uid72619	NC_015954	chromosome		arCOG03726	4 (-)	S		Uncharacterized conserved protein			
===								6	halophilic_archaeon_DL31_uid72619	NC_015955	plasmid phalar01									
345006833	hypothetical protein Halar_0037	No	0	plasmid	4744	5520	-		halophilic_archaeon_DL31_uid72619	NC_015955	plasmid phalar01		arCOG11350	3	S		Uncharacterized conserved protein			
345006834	hypothetical protein Halar_0038	No	2	plasmid	5760	5993	-		halophilic_archaeon_DL31_uid72619	NC_015955	plasmid phalar01									
345006835	hypothetical protein Halar_0039	No	0	plasmid	6125	7747	+		halophilic_archaeon_DL31_uid72619	NC_015955	plasmid phalar01									
345006836	AAA-4 family protein	No	0	plasmid	7976	8887	+		halophilic_archaeon_DL31_uid72619	NC_015955	plasmid phalar01		arCOG03296	2 (-)	K		Predicted transcriptional regulator containing an H	0.000001	pfam04326	Divergent AAA domain. This family is rel
345006837	hypothetical protein Halar_0041	No	4	plasmid	9121	10107	+		halophilic_archaeon_DL31_uid72619	NC_015955	plasmid phalar01									
345006838	hypothetical protein Halar_0042	No	0	plasmid	10377	10706	+		halophilic_archaeon_DL31_uid72619	NC_015955	plasmid phalar01									
===								5	halophilic_archaeon_DL31_uid72619	NC_015955	plasmid phalar01									
345006924	hypothetical protein Halar_0135	No	0	plasmid	116660	116812	+		halophilic_archaeon_DL31_uid72619	NC_015955	plasmid phalar01		arCOG10363	7	V		AbrB-like transcriptional regulator			
345006925	hypothetical protein Halar_0136	No	0	plasmid	116805	117203	+		halophilic_archaeon_DL31_uid72619	NC_015955	plasmid phalar01		arCOG08887	3	S		Uncharacterized conserved protein			
345006926	Resolvase domain-containing protein	No	0	plasmid	117196	117864	+		halophilic_archaeon_DL31_uid72619	NC_015955	plasmid phalar01		arCOG03162	12 (-)	L	PinR	Site-specific recombinase, DNA invertase Pin ho	0.0000003	pfam00239	Resolvase, N terminal domain. The N-term
345006927	hypothetical protein Halar_0138	No	0	plasmid	118001	118282	-		halophilic_archaeon_DL31_uid72619	NC_015955	plasmid phalar01	clust_367								
345006928	hypothetical protein Halar_0139	No	0	plasmid	118768	119499	-		halophilic_archaeon_DL31_uid72619	NC_015955	plasmid phalar01	clust_33								
===								5	halophilic_archaeon_DL31_uid72619	NC_015955	plasmid phalar01									
345006993	hypothetical protein Halar_0207	No	0	plasmid	196816	197547	+		halophilic_archaeon_DL31_uid72619	NC_015955	plasmid phalar01	clust_33								
345006994	hypothetical protein Halar_0208	No	0	plasmid	197654	198007	+		halophilic_archaeon_DL31_uid72619	NC_015955	plasmid phalar01									
345006995	hypothetical protein Halar_0209	No	0	plasmid	198124	198312	+		halophilic_archaeon_DL31_uid72619	NC_015955	plasmid phalar01		arCOG10631	2	S		Uncharacterized conserved protein			
345006996	PaaX domain-containing protein	No	0	plasmid	198753	199148	-		halophilic_archaeon_DL31_uid72619	NC_015955	plasmid phalar01		arCOG02749	3 (-)	K		Predicted transcriptional regulator			
345006997	hypothetical protein Halar_0211	No	0	plasmid	199162	199449	-		halophilic_archaeon_DL31_uid72619	NC_015955	plasmid phalar01									
===								6	halophilic_archaeon_DL31_uid72619	NC_015955	plasmid phalar01									
345007008	ATPase	No	0	plasmid	209797	211188	-		halophilic_archaeon_DL31_uid72619	NC_015955	plasmid phalar01		arCOG03166	9 (-)	R		AAA+ superfamily ATPase fused to HTH and Rec	1E-29	COG1672	Predicted ATPase (AAA+ superfamily)
345007009	hypothetical protein Halar_0225	No	0	plasmid	211356	212039	+		halophilic_archaeon_DL31_uid72619	NC_015955	plasmid phalar01	clust_149								
345007010	hypothetical protein Halar_0226	No	0	plasmid	212212	212535	+		halophilic_archaeon_DL31_uid72619	NC_015955	plasmid phalar01	clust_323								
345007011	hypothetical protein Halar_0227	No	0	plasmid	212601	212963	-		halophilic_archaeon_DL31_uid72619	NC_015955	plasmid phalar01	clust_45								
345007012	hypothetical protein Halar_0228	No	0	plasmid	213147	213995	-		halophilic_archaeon_DL31_uid72619	NC_015955	plasmid phalar01		arCOG11350	3	S		Uncharacterized conserved protein			
345007013	integrase family protein	No	0	plasmid	214132	214749	+		halophilic_archaeon_DL31_uid72619	NC_015955	plasmid phalar01		arCOG01241	31 (-)	L	XerC	Integrase	0.000001	COG4974	Site-specific recombinase XerD
===								9	halophilic_archaeon_DL31_uid72619	NC_015955	plasmid phalar01									
345007021	hypothetical protein Halar_0240	No	0	plasmid	231258	231935	+		halophilic_archaeon_DL31_uid72619	NC_015955	plasmid phalar01	arCOG09185,a	arCOG09185	3	S		Uncharacterized conserved protein			
345007022	hypothetical protein Halar_0241	No	0	plasmid	231982	232716	+		halophilic_archaeon_DL31_uid72619	NC_015955	plasmid phalar01									
345007023	hypothetical protein Halar_0242	No	0	plasmid	232898	233482	+		halophilic_archaeon_DL31_uid72619	NC_015955	plasmid phalar01									
345007024	transposase (ISH3)	No	0	plasmid	233993	235159	-		halophilic_archaeon_DL31_uid72619	NC_015955	plasmid phalar01		arCOG06160	11 (-)	L		Transposase			
345007025	hypothetical protein Halar_0244	No	0	plasmid	235888	236124	+		halophilic_archaeon_DL31_uid72619	NC_015955	plasmid phalar01		arCOG09232	3	S		Uncharacterized conserved protein			
345007026	hypothetical protein Halar_0245	No	0	plasmid	236372	236863	+		halophilic_archaeon_DL31_uid72619	NC_015955	plasmid phalar01	clust_218								
345007027	hypothetical protein Halar_0246	No	0	plasmid	236896	237168	+		halophilic_archaeon_DL31_uid72619	NC_015955	plasmid phalar01		arCOG01664	2 (-)	V	RelE	Cytotoxic translational repressor of toxin-antitoxin stability system			
345007028	hypothetical protein Halar_0247	No	0	plasmid	237481	237645	+		halophilic_archaeon_DL31_uid72619	NC_015955	plasmid phalar01		arCOG10363	7	V		AbrB-like transcriptional regulator			
345007029	hypothetical protein Halar_0248	No	0	plasmid	237638	237823	+		halophilic_archaeon_DL31_uid72619	NC_015955	plasmid phalar01	clust_470								
===								7	halophilic_archaeon_DL31_uid72619	NC_015955	plasmid phalar01									
345007064	hypothetical protein Halar_0283	No	0	plasmid	286526	286849	+		halophilic_archaeon_DL31_uid72619	NC_015955	plasmid phalar01		arCOG04516	1 (-)	S		Uncharacterized conserved protein			
345007065	hypothetical protein Halar_0284	No	2	plasmid	287308	287523	+		halophilic_archaeon_DL31_uid72619	NC_015955	plasmid phalar01									
345007066	hypothetical protein Halar_0285	No	2	plasmid	287535	287711	-		halophilic_archaeon_DL31_uid72619	NC_015955	plasmid phalar01		arCOG11352	1	S		Uncharacterized conserved protein			
345007067	hypothetical protein Halar_0286	No	3	plasmid	287708	288064	-		halophilic_archaeon_DL31_uid72619	NC_015955	plasmid phalar01	clust_300								
345007068	hypothetical protein Halar_0287	No	0	plasmid	288343	288519	+		halophilic_archaeon_DL31_uid72619	NC_015955	plasmid phalar01		arCOG09371	1	S		Uncharacterized conserved protein			
345007069	hypothetical protein Halar_0289	No	0	plasmid	290123	290923	-		halophilic_archaeon_DL31_uid72619	NC_015955	plasmid phalar01		arCOG02809	5 (-)	S		Uncharacterized conserved protein			
345007070	hypothetical protein Halar_0290	No	0	plasmid	291115	291378	+		halophilic_archaeon_DL31_uid72619	NC_015955	plasmid phalar01									
===								5	halophilic_archaeon_DL31_uid72619	NC_015955	plasmid phalar01									
345007146	hypothetical protein Halar_0373	No	0	plasmid	372033	372371	-		halophilic_archaeon_DL31_uid72619	NC_015955	plasmid phalar01									
345007147	hypothetical protein Halar_0374	No	0	plasmid	372405	372578	-		halophilic_archaeon_DL31_uid72619	NC_015955	plasmid phalar01									
345007148	hypothetical protein Halar_0375	No	0	plasmid	372817	373599	-		halophilic_archaeon_DL31_uid72619	NC_015955	plasmid phalar01		arCOG02809	5 (-)	S		Uncharacterized conserved protein			
345007149	hypothetical protein Halar_0376	No	0	plasmid	373795	374619	+		halophilic_archaeon_DL31_uid72619	NC_015955	plasmid phalar01									
345007150	hypothetical protein Halar_0377	No	0	plasmid	374720	374929	+		halophilic_archaeon_DL31_uid72619	NC_015955	plasmid phalar01		arCOG10965	1	S		Uncharacterized conserved protein			
===								8	halophilic_archaeon_DL31_uid72619	NC_015955	plasmid phalar01									
345007161	hypothetical protein Halar_0388	No	0	plasmid	388376	389017	-		halophilic_archaeon_DL31_uid72619	NC_015955	plasmid phalar01		arCOG11351	1	S		Uncharacterized conserved protein			
345007162	transposase (ISH3)	No	0	plasmid	391777	392943	+		halophilic_archaeon_DL31_uid72619	NC_015955	plasmid phalar01		arCOG06160	11 (-)	L		Transposase			
345007163	hypothetical protein Halar_0391	No	0	plasmid	393713	393940	-		halophilic_archaeon_DL31_uid72619	NC_015955	plasmid phalar01		arCOG08139	1	S		Uncharacterized conserved protein			
345007164	hypothetical protein Halar_0392	No	0	plasmid	394150	395058	+		halophilic_archaeon_DL31_uid72619	NC_015955	plasmid phalar01									
345007165	hypothetical protein Halar_0393	No	0	plasmid	395191	395646	+		halophilic_archaeon_DL31_uid72619	NC_015955	plasmid phalar01									
345007166	hypothetical protein Halar_0394	No	0	plasmid	395692	396051	+		halophilic_archaeon_DL31_uid72619	NC_015955	plasmid phalar01									
345007167	orc1/cdc6 family replication initiation protein	No	0	plasmid	396211	397239	+		halophilic_archaeon_DL31_uid72619	NC_015955	plasmid phalar01		arCOG00467	13 (-)	L	CDC6	Cdc6-related protein, AAA superfamily ATPase	8E-43	COG1474	Cdc6-related protein, AAA superfamily AT
345007168	hypothetical protein Halar_0396	No	0	plasmid	397329	398126	+		halophilic_archaeon_DL31_uid72619	NC_015955	plasmid phalar01									
===								7	halophilic_archaeon_DL31_uid72619	NC_015955	plasmid phalar01									
345007244	hypothetical protein Halar_0478	No	0	plasmid	470214	471146	-		halophilic_archaeon_DL31_uid72619	NC_015955	plasmid phalar01		arCOG02491	7 (-)	S		WD40 repeats containing protein			
345007245	hypothetical protein Halar_0479	No	0	plasmid	471217	473217	+		halophilic_archaeon_DL31_uid72619	NC_015955	plasmid phalar01									
345007246	hypothetical protein Halar_0480	No	0	plasmid	473378	474772	+		halophilic_archaeon_DL31_uid72619	NC_015955	plasmid phalar01									
345007247	hypothetical protein Halar_0481	No	0	plasmid	474882	475082	+		halophilic_archaeon_DL31_uid72619	NC_015955	plasmid phalar01									
345007248	hypothetical protein Halar_0482	No	0	plasmid	475079	475507	-		halophilic_archaeon_DL31_uid72619	NC_015955	plasmid phalar01									
345007249	hypothetical protein Halar_0483	No	0	plasmid	475797	476219	+		halophilic_archaeon_DL31_uid72619	NC_015955	plasmid phalar01									
345007250	HNH endonuclease	No	2	plasmid	476293	477192	+		halophilic_archaeon_DL31_uid72619	NC_015955	plasmid phalar01		arCOG03898	7 (-)	V	McrA	Restriction endonuclease, McrA/HNH family	0.0000007	COG1403	Restriction endonuclease
===								13	halophilic_archaeon_DL31_uid72619	NC_015955	plasmid phalar01									
345007439	Rad51 domain-containing protein	No	0	plasmid	688058	689047	-		halophilic_archaeon_DL31_uid72619	NC_015955	plasmid phalar01		arCOG00415	7 (-)	L	RecA	RecA/RadA recombinase	1E-37	pfam08423	Rad51. Rad51 is a DNA repair and recombi
345007440	hypothetical protein Halar_0702	No	0	plasmid	689201	690424	-		halophilic_archaeon_DL31_uid72619	NC_015955	plasmid phalar01									
345007441	hypothetical protein Halar_0703	No	0	plasmid	690590	690733	-		halophilic_archaeon_DL31_uid72619	NC_015955	plasmid phalar01									
345007442	hypothetical protein Halar_0704	No	2	plasmid	690730	691272	-		halophilic_archaeon_DL31_uid72619	NC_015955	plasmid phalar01									
345007443	hypothetical protein Halar_0705	No	0	plasmid	691329	691820	-		halophilic_archaeon_DL31_uid72619	NC_015955	plasmid phalar01									
345007444	transposase (ISH3)	No	0	plasmid	692085	693251	-		halophilic_archaeon_DL31_uid72619	NC_015955	plasmid phalar01		arCOG06160	11 (-)	L		Transposase			
345007445	hypothetical protein Halar_0707	No	0	plasmid	693312	693488	+		halophilic_archaeon_DL31_uid72619	NC_015955	plasmid phalar01									
345007446	hypothetical protein Halar_0708	No	0	plasmid	693735	694355	-		halophilic_archaeon_DL31_uid72619	NC_015955	plasmid phalar01									
345007447	hypothetical protein Halar_0709	No	0	plasmid	694689	695324	+		halophilic_archaeon_DL31_uid72619	NC_015955	plasmid phalar01									
345007448	hypothetical protein Halar_0710	No	0	plasmid	695770	697035	-		halophilic_archaeon_DL31_uid72619	NC_015955	plasmid phalar01									
345007449	integrase family protein	No	0	plasmid	697237	698415	-		halophilic_archaeon_DL31_uid72619	NC_015955	plasmid phalar01		arCOG01241	31 (-)	L	XerC	Integrase	0.000003	pfam00589	Phage integrase family. Members of this
345007450	hypothetical protein Halar_0712	No	0	plasmid	698405	699067	-		halophilic_archaeon_DL31_uid72619	NC_015955	plasmid phalar01									
345007451	hypothetical protein Halar_0713	No	0	plasmid	699301	699861	-		halophilic_archaeon_DL31_uid72619	NC_015955	plasmid phalar01									
===								6	halophilic_archaeon_DL31_uid72619	NC_015959	plasmid phalar02									
345133532	adenine-specific DNA methyltransferase	No	0	plasmid	121	1479	+		halophilic_archaeon_DL31_uid72619	NC_015959	plasmid phalar02		arCOG02636	3 (-)	L		Adenine-specific DNA methylase	4E-10	pfam13659	Methyltransferase domain. This family co
345133533	hypothetical protein Halar_0002	No	0	plasmid	2043	2723	+		halophilic_archaeon_DL31_uid72619	NC_015959	plasmid phalar02									
345133534	hypothetical protein Halar_0003	No	0	plasmid	2726	2926	+		halophilic_archaeon_DL31_uid72619	NC_015959	plasmid phalar02									
345133535	hypothetical protein Halar_0004	No	2	plasmid	2930	3106	+		halophilic_archaeon_DL31_uid72619	NC_015959	plasmid phalar02									
345133536	hypothetical protein Halar_0005	No	0	plasmid	3194	3373	+		halophilic_archaeon_DL31_uid72619	NC_015959	plasmid phalar02									
345133537	hypothetical protein Halar_0006	No	0	plasmid	3428	3925	+		halophilic_archaeon_DL31_uid72619	NC_015959	plasmid phalar02		arCOG00227	1 (-)	P	AfuA	ABC-type Fe3+ transport system, periplasmic component			
