# Supplementary material for: Dark matter in archaeal genomes: a rich source of novel mobile elements, defense systems and secretory complexes
Source: Extremophiles. 2014 Aug 12;18(5):877–93. doi: 10.1007/s00792-014-0672-7 (PMC4158269; doi:10.1007/s00792-014-0672-7)
Supplement: Supplementary file 3 — Supplementary material 3 (DOCX 19 kb) [file 792_2014_672_MOESM3_ESM.docx]

Table S3. Annotation of the putative provirus ArcVen-P3.

| \| **Gene name** \| **GI** \| **Function** \| **HHpred** \| **Blastp vs virus db** \| **Identity, E-value** \| **Blastp vs nr** \| **Identity, E-value** \| \| --- \| --- \| --- \| --- \| --- \| --- \| --- \| --- \| |
| --- | --- | --- | --- | --- | --- | --- | --- | --- |
| \| Int-N \| 1822857..1823131 \| Int-N gene fragment \|  \|  \|  \| \| \|  \|  \| \| --- \| --- \| --- \| --- \| --- \| --- \| --- \| --- \| --- \| --- \| \| Arcve_2053 \| 327401919 \| Transcriptional regulator, CopG \| RHH from P. Horikoshii (2bj7), P=99.5 \| SSV1 (DAA64513) \| 19/49(39%), 0.1 \| \| \| Methanocaldococcus infernus ME (YP_003616420) \| 26/63(41%), 6e-05 \| \| Arcve_2054 \| 327401920 \| Transcriptional regulator, CopG \| RHH from P. Horikoshii (2bj7), P=99.7 \| SSV1 (DAA64513) \| 19/42(45%), 8e-02 \| \| \| Candidatus Nanosalina sp. J07AB43 (EGQ44202) \| 16/41(39%), 9e-03 \| \| Arcve_2055 \| 327401921 \|  \|  \|  \|  \| \| \|  \|  \| \| Arcve_2056 \| 327401922 \|  \|  \|  \|  \| \| \|  \|  \| \| Arcve_2057 \| 327401923 \| Zn finger protein (C4) \| Zn finger protein from H.sapiens (1x6e), P=99.7 \|  \|  \| \| \|  \|  \| \| Arcve_2058 \| 327401924 \| Holliday junction resolvase \| HJR from S.solfataricus (1hh1), P=81 \|  \|  \| \| \| Archaeoglobus profundus DSM 5631 (YP_003401331) \| 28/75(37%), 3e-03 \| \| Arcve_2059 \| 327401925 \|  \|  \|  \|  \| \| \|  \|  \| \| Arcve_2060 \| 327401926 \| Zn finger protein (C4) \| Zn-binding protein from P.aeruginosa (2akl), P=93 \|  \|  \| \| \|  \|  \| \| Arcve_2061 \| 327401927 \| RHH-coiled-coil protein \| Nterm:RHH domain from phage P22 (1baz), P=94.6; C-term: coiled-coil domain \|  \|  \| \| \| \| Arcve_2062 \| 327401928 \|  \|  \|  \|  \| \| \|  \|  \| \| Arcve_2063 \| 327401929 \| Zn finger protein (2xC4) \| Zn finger protein from H.sapiens (3w5k), P=99.97 \|  \|  \| \| \| Dehalococcoides sp. CBDB1 (YP_307784) \| 23/65(35%), 1e-02 \| \| Arcve_2064 \| 327401930 \| Coiled-coil protein \|  \|  \|  \| \| \|  \|  \| \| Arcve_2065 \| 327401931 \|  \|  \|  \|  \| \| \|  \|  \| \| Arcve_2066 \| 327401932 \|  \|  \|  \|  \| \| \|  \|  \| \| Arcve_2067 \| 327401933 \| FtsK-like ATPase \| Numerous hits to ATPases, including packaging ATPase from STIV2 (4kfu), P=99.8 \| Bacillus thuringiensis phage GIL16c (YP_224111) \| 38/164(23%), 0.2 \| \| \| Dehalococcoides sp. CBDB1 (YP_307782) \| 66/163(40%), 3e-30 \| \| Arcve_2068 \| 327401934 \|  \|  \|  \|  \| \| \| Dehalococcoides sp. CBDB1 (YP_307781) \| 25/72(35%), 0.36 \| \| Arcve_2069 \| 327401935 \| VP16-like major capsid protein \| partial hit to VP16 (3zn4), P=27.5 \| against IN93: NP_777331 \| 35/139(25%) \| \| \| Dehalococcoides sp. CBDB1 (YP_307780) \| 45/113(40%), 7e-10 \| \| Arcve_2070 \| 327401936 \|  \|  \|  \|  \| \| \|  \| \| Arcve_2071 \| 327401937 \| Coiled-coil protein \|  \|  \|  \| \| \|  \| \| Arcve_2072 \| 327401938 \|  \|  \|  \|  \| \| \|  \| \| Arcve_2073 \| 327401939 \| VP2-like DNA-binding protein \|  \| Thermus phage IN93 (NP_777328); homologues in fuselloviruses (VP2) \| 29/68(43%), 3e-11 \| \| \|  \| \| Arcve_2074 \| 327401940 \|  \|  \|  \|  \| \| \|  \|  \| \| Arcve_2075 \| 327401941 \| Coiled-coil protein \|  \|  \|  \| \| \|  \|  \| \| Arcve_2076 \| 327401942 \|  \|  \|  \|  \| \| \| Archaeoglobus profundus DSM 5631 (YP_003401217) \| 30/67(45%), 4e-10 \| \| Arcve_2077 \| 327401943  (1831573..1832547) \| Integrase (C-term freagment) \| XerD from E.coli (1a0p), P=99.9 \| Thermococcus prieurii virus 1 (YP_005271232) \| \| 59/192(31%), 2e-24 \| Archaeoglobus profundus DSM 5631 (YP_003399856) \| \| 74/179(41%), 3e-33 \| |
