# Supplementary material for: Dark matter in archaeal genomes: a rich source of novel mobile elements, defense systems and secretory complexes
Source: Extremophiles. 2014 Aug 12;18(5):877–93. doi: 10.1007/s00792-014-0672-7 (PMC4158269; doi:10.1007/s00792-014-0672-7)
Supplement: Supplementary file 4 — Supplementary material 4 (DOCX 16 kb) [file 792_2014_672_MOESM4_ESM.docx]

**Table S4. Sequence similarity search results for selected proteins**

| Query Locus ID | Query Genome | HHpred hit ID and annotation | HHpred probablity | Selected PSI-Blast  hit GI/organism/  annotation | PSI-Blast  E-value | Predicted function and comment |
| --- | --- | --- | --- | --- | --- | --- |
| TON_0322 | Thermococcus onnurineus NA1 | PF03787 RAMP superfamily | 73 | 491525290/ Veillonella atypica/ CRISPR-associated RAMP protein, Csm4 family | 4.2 | G-rich loop, the signature of RAMP superfamily is present |
| TON_0323 | Thermococcus onnurineus NA1 | PF03787 RAMPs | 98.3 | 553768395/  Pseudomonas aeruginosa/ CRISPR type aferr-associated protein csf2 | 0.0008 | There hits with better E-value to hypothetical proteins, that belong to Type IV (aferr) CRISPR-Cas system (data not shown); G-rich loop, the signature of RAMP superfamily is present |
| TON_0325 | Thermococcus onnurineus NA1 | PF01966, HD domain | 68 | No hits |  | Catalytic motis preserved and have the same order as in Cas3 protein |
| Igag_0619 | Ignisphaera aggregans | COG1353, Predicted CRISPR-associated polymerase (Cas10) | 97 | No hits |  | The match covers onle C-terminal region of the protein, catalytic motifs of Cas10 family are preserved |
| TAM4_1738 | Thermococcus sp. AM4 | 1atl, Atrolysin C metalloendopeptidase | 87 | 488710485/Myxococcus sp/Cell envelope biogenesis protein OmpA | 0.0005 | Many hits to suface proteins from different bacteria |
|  |  |  |  |  |  |  |
|  |  |  |  |  |  |  |
